# Identification of neglected cestode *Taenia multiceps* microRNAs by illumina sequencing and bioinformatic analysis

**DOI:** 10.1186/1746-6148-9-162

**Published:** 2013-08-13

**Authors:** Xuhang Wu, Yan Fu, Deying Yang, Yue Xie, Runhui Zhang, Wanpeng Zheng, Huaming Nie, Ning Yan, Ning Wang, Jiahai Wang, Xiaobin Gu, Shuxian Wang, Xuerong Peng, Guangyou Yang

**Affiliations:** 1 Department of Parasitology, College of Veterinary Medicine, Sichuan Agricultural University, Ya’an 625014, China; 2 Department of Chemistry, College of Life and Basic Science, Sichuan Agricultural University, Ya’an 625014, China

**Keywords:** MicroRNA, *Taenia multiceps*, Unigene, Illumina sequencing, Candidate novel miRNA

## Abstract

**Background:**

Worldwide, but especially in developing countries, coenurosis of sheep and other livestock is caused by *Taenia multiceps* larvae, and zoonotic infections occur in humans. Infections frequently lead to host death, resulting in huge socioeconomic losses. MicroRNAs (miRNAs) have important roles in the post-transcriptional regulation of a large number of animal genes by imperfectly binding target mRNAs. To date, there have been no reports of miRNAs in *T. multiceps*.

**Results:**

In this study, we obtained 12.8 million high quality raw reads from adult *T. multiceps* small RNA library using Illumina sequencing technology. A total of 796 conserved miRNA families (containing 1,006 miRNAs) from 170,888 unique miRNAs were characterized using miRBase (Release 17.0). Here, we selected three conserved miRNA/miRNA* (antisense strand) duplexes at random and amplified their corresponding precursors using a PCR-based method. Furthermore, 20 candidate novel miRNA precursors were verified by genomic PCR. Among these, six corresponding *T. multiceps* miRNAs are considered specific for Taeniidae because no homologs were found in other species annotated in miRBase. In addition, 181,077 target sites within *T. multiceps* transcriptome were predicted for 20 candidate newly miRNAs.

**Conclusions:**

Our large-scale investigation of miRNAs in adult *T. multiceps* provides a substantial platform for improving our understanding of the molecular regulation of *T. multiceps* and other cestodes development.

## Background

Increasing evidence shows that control of gene expression is essential for normal development of organisms and for regulating fundamental biological processes [1-4]. Regulation of gene expression occurs at the transcriptional and post-transcriptional levels [4]. Small non-coding RNAs (sRNAs), including small interfering RNA (siRNA), microRNA (miRNA) and Piwi-interacting RNA (piRNA), interact with the RNA-induced silencing complex (RISC) to regulate target gene expression at the post-transcriptional level [4-6]. Notably, microRNAs (miRNAs), which comprise one of the most abundant classes of genes regulators [7], play important regulatory roles in metazoan animals and plants [8] by binding to complementary sequence in the 3 untranslated region (3-UTR) of target mRNAs, leading to cleavage or translational repression [2,9]. Mature miRNAs, derived from long primary stem-loop miRNA precursors approximately 70 bp in length [10], regulate many processes, including apoptosis, cell proliferation, fat metabolism, hematopoiesis and organogenesis [11]. Since lin-4 was originally discovered in *Caenorhabditis elegans*[12,13], many miRNAs have been identified in almost all metazoan genomes, including flies, worms, plants, and mammals [14]. Furthermore, miRNAs are associated with animal development, including germline, muscle, and neuronal development [15,16].

*Taenia multiceps* is a widespread cestode, which has two life cycle stages. The larval stage (coenurus) parasitizes the brain or spinal cord of domestic ruminants, such as buffalo, cattle, goats, horses, sheep, and yak, as well as wild species, causing lethal neurological symptoms [17]. Since human coenurosis was first reported by Brumpt in 1913 [18], further evidence has shown that this parasite causes zoonotic infections in humans [19-25]. Adult *T. multiceps* are found in the small intestine of dogs and other canids, and gravid proglottids in host feces are a source of infection following ingestion by an intermediate hosts [23].

Coenurus caused by *T. multiceps* occurs almost all over the world [26], especially in the developing countries of Africa and southeastern Asia, and causes huge economic losses from condemned meat and viscera [27]. Given the important regulatory functions of sRNAs in adapting to the environment and the lack of large-scale sRNA characterization in cestodes, it is important to investigate *T. multiceps* miRNAs and other sRNAs using high-throughput sequencing methods. In this study, we investigated *T. multiceps* miRNAs using an *Echinococcus multilocularis* reference genome and an adult *T. multiceps* transcriptome dataset. Our results increase the current understanding of the molecular mechanisms of cestode gene regulation [28], and will help to identify novel biomarkers by revealing the target genes [29,30] and develop new strategies to control parasitic zoonoses [31].

## Methods

### Parasite preparation

Larvae (coenuri) were collected from the brain of a naturally infected goat at an organic farm in Panzhihua, Sichuan, China. Infection was performed after morphological identification of the larvae. Two parasite-free beagles were orally infected with 20 larvae each. Forty-eight days after infection, adult *T. multiceps* were obtained from the small intestine of infected dogs after gravid proglottids were observed in their feces. *T. multiceps* were washed thoroughly in physiological saline solution (37°C) to avoid host cell contamination, transferred into liquid nitrogen, and stored at - 80°C.

All animals were handled in accordance with the Animal Protection Law of the People’s Republic of China (a draft of which was released on September 18, 2009). This study was approved by the National Institute of Animal Health Animal Care and Use Committee at Sichuan Agricultural University (approval number 2010–018).

### Small RNA library preparation and Illumina sequencing

Total RNA was extracted from adult *T. multiceps* (n = 6) using TRIZOL (Invitrogen, Carlsbad, CA), according to the manufacturer’s protocol. RNA integrity was checked by determining the RNA integrity number using an Agilent 2100 Bioanalyzer. RNA sequences ranging from 18–30 nt in length were isolated and purified from total RNA by Novex 15% TBE–Urea gel (Invitrogen) electrophoresis. Proprietary (Solexa) adaptors were then added to the 3 and 5 -termini of sRNAs, which were then used for cDNA synthesis. These ligation products were amplified by reverse transcription PCR (RT-PCR) using a RT-PCR kit (Invitrogen). PCR amplification products were purified for high-throughput sequencing by electrophoresis using a 6% TBE PAGE gel (Invitrogen). The produced libraries were sequenced using a Solexa sequencer at the Beijing Genomics Institute (BGI)-Shenzhen, Shenzhen, China, according to the manufacturer’s instruction.

### Bioinformatics analysis pipeline for searching conserved miRNAs

The workflow for obtaining ‘clean’ reads involved filtering low quality tags; removing raw reads with 5’ primer contaminants; trimming 3’ adaptors; removing reads without insert tags; discarding reads with polyA tails; and removing contaminants formed by adaptor–adaptor ligation. The length distribution of clean reads was summarized, all clean reads were mapped to the *E. multilocularis* genome, which was obtained from the Sanger Institute FTP site [32], using the Short Oligonucleotide Alignment Package (SOAP) [33], and the expression and distribution of sRNAs in the genome were analyzed. Subsequently, clean reads were annotated against the Rfam database (version 10.0) [34] and GenBank noncoding RNA database [35] to remove noncoding RNAs, such as rRNA, scRNA, snoRNA, snRNA and tRNA. Tag2repeat software (provided by BGI) was used to select repeat overlapping sequences as repeat-associated small RNAs and then eliminate them. The remaining sequences were used to identify conserved miRNAs and predict novel miRNAs.

To identify conserved miRNAs in *T. multiceps*, clean small RNA sequences were aligned with the miRNA precursor and mature sequences of all animals deposited in miRBase 17.0, using tag2miRNA software (provided by BGI). Sequences containing no more than two mismatches with known miRNAs were defined as conserved miRNAs, and the most abundantly expressed sequence was selected when multiple sequences were assigned as the same conserved miRNA. Sequences that could not be annotated from sequence alignments were defined as non-annotation unique reads.

### Prediction of novel miRNAs

The *E. multilocularis* genome [32] was also used to discover the potential miRNA precursors. Unannotated unique *T. multiceps* reads that perfectly matched the *E. multilocularis* genome were designated candidate miRNA precursors. Potential precursor sequences were selected from each end of the sequence matching the referenced genome, and MIREAP software [36] was used to determine whether these sequences form a characteristic miRNA hairpin-like structure, contain a Dicer cleavage site, and have a minimum free energy (MFE) lower than 18 kcal/mol. miRNAs with these characteristics were defined as qualified precursors for candidate miRNAs. Previous studies suggest that miRNA precursors with a minimum free energy index (MFEI) greater than 0.85 are likely to be miRNAs [37,38]. We used the formula,

$$\begin{array}{rl} \mathrm{MFEI}= & \left[\left(\mathrm{MFE}/\mathrm{length}\;\mathrm{of}\;\mathrm{the}\;\mathrm{RNA}\;\mathrm{sequence}\right)\;\times \;100\right]\\ & /\;\left(G+C\right)\%, \end{array}$$

to select novel miRNAs with MFEIs > 0.85.

### Reliability of conserved microRNAs and Validation of novel miRNA precursors

Conserved miRNAs and novel candidate miRNA precursors were verified using a PCR-based method. Genomic DNA was extracted from adult *T. multiceps* using a Gentra Puregene Tissue Kit (Qiagen, Valencia, CA, USA) following the manufacture’s protocol. Three conserved putative miRNA/miRNA* duplexes were selected randomly to check if their corresponding precursors form a characteristic pre-miRNA hairpin-like structure with low free energy and the putative miRNA in the stem. Using the candidate precursors which predicted based on the *E. multilocularis* genome, we designed primers for novel miRNA precursors and miRNA/miRNA* duplexes using Primer Premier 5.0. Primers successfully used for amplifying the three conserved and 20 novel miRNA precursors are shown in Additional file 1. PCR was carried out according to the PCR verification scheme for pinewood nematodes [28]. Amplification product length was examined by 3.5% agarose gels electrophoresis using a 50 bp DNA ladder. Fragments between 60 and 100 nt in length were subcloned into the pMD18-T vector (Takara, Dalian, Liaoning, China) for sequencing. RNAfold package [39] was used to predict the secondary structure of the amplified miRNA precursor.

### Prediction of miRNA target genes

*T. multiceps* transcriptome unigenes obtained by Illumina sequencing were used to predict novel miRNA targets (Unigene: JR916739–JR948020 in the Transcriptome Shotgun Assembly Sequence Database at NCBI from our previous study [40]). miRNA target genes for adult *T. multiceps* were predicted using RNAhybrid [41,42] parameters of -f 2,8 –v 3 –u 3 (helix constraint, 2–8 and maximal internal or bulge loop size per side, 3)*.*

To understand the main biological functions and identify the biochemical metabolic pathway/signal transduction pathways of novel miRNAs target gene candidates, all target gene candidates were annotated against the Gene Ontology (GO) database [43] and the Kyoto Encyclopedia of Genes and Genomes (KEGG) database [44], as described in our recent research [45].

## Results

### *Illumina sequencing of small RNAs from* T. multiceps

**Figure 1:**
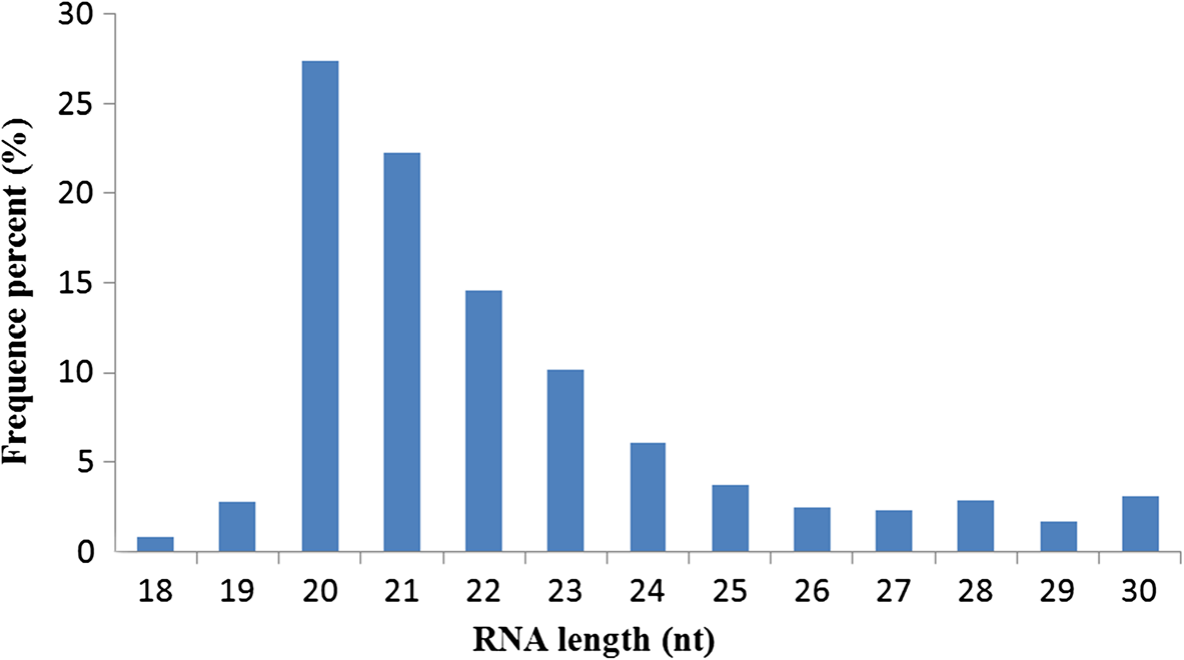
**Length distribution analysis of sequenced small RNAs in *Taenia multiceps*.** The frequencies of each unique read represent relative expression levels, and only small RNA sequences 18–30 nt in length were considered.

We employed Illumina sequencing technology on a library of small RNAs from adult *T. multiceps* to identify miRNAs, which yielded 13.5 million raw reads. The dataset was deposited in NCBI Gene Expression Omnibus [46] with the accession number [GEO: GSE35647]. We removed 312,411 low quality tags, 68,068 3 adapter null reads, 111,184 reads without insert tags, 30,727 reads with 5 adapter contaminants, and 197,872 reads smaller than 18 nt, and 462 reads with ployA tails to obtain 12.8 million clean reads (96.91% of the raw reads). The length distribution profile showed that small RNAs of 20–22 nt were the most abundant (Figure 1). After mapping all clean reads to the *E. multilocularis* genome (Additional file 2), 2,266,539 sRNAs (17.68% of 12.8 million) were perfectly matched sequences in the *E. multilocularis* genome, including 39,765 unique sRNAs (2.88% of total 1,379,196 unique sRNAs).

### Identification of microRNAs by computational algorithms

**Figure 2:**
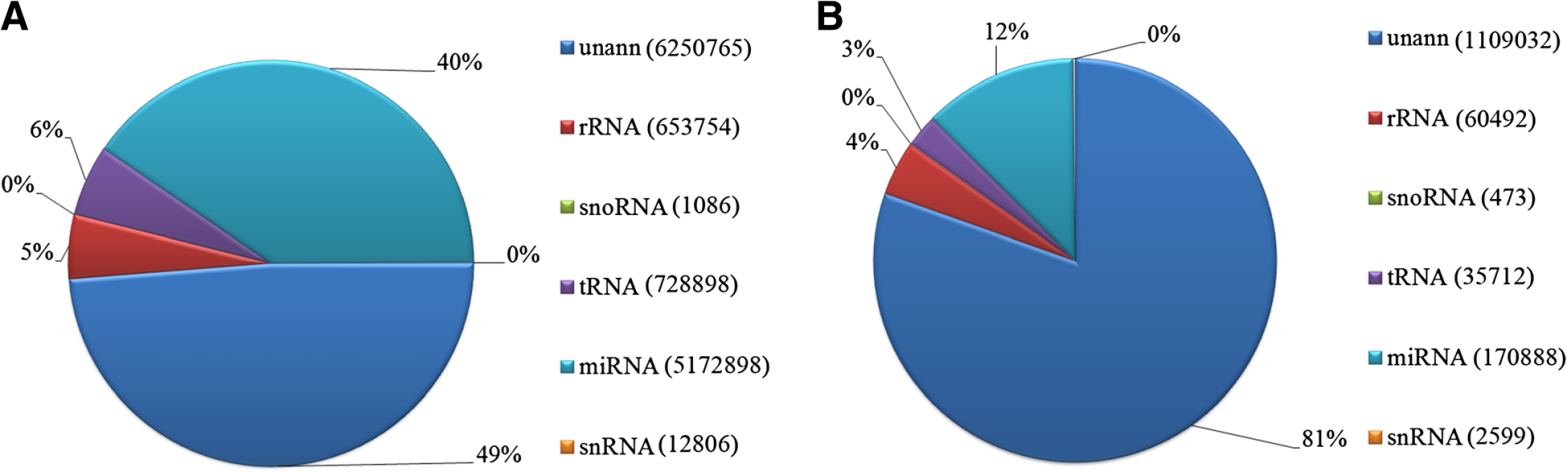
**Distribution of small RNAs in *Taenia multiceps *annotation following Illumina sequencing. A**. total reads. **B**. unique reads. “unann” stands for un-annotated reads/unique reads. One unique read means all the reads with the same sequence.

Non-coding small RNAs including 653,754 rRNAs, 1,086 snoRNAs, 12,806 snRNAs, and 728,898 tRNAs were removed after mapping all clean reads against the Rfam database (version 10.0). Subsequently, 392,874 total sRNAs (including 22,734 unique sRNAs) were identified as repeat associated sRNAs and then removed. A total of 11.2 million small RNA sequences were obtained. The distribution of total and unique sRNAs is shown in Figure 2. A total of 1,006 miRNAs (grouped into 796 miRNA families) were designated conserved miRNAs against miRBase 17.0. The expression levels and sequences of conserved miRNA families and miRNAs are shown in Additional file 3. Of these, 10 conserved miRNA families were identified in more than 100,000 counts: the five most abundant miRNA families were miR-71 (998,673 counts), miR-315 (774,438), miR-2840 (496,050), miR-1 (469,997), and miR-1422 (254,527) (Table 1). Among the conserved miRNAs, we also identified 95 miRNAs* (antisense strands of miRNAs) (Additional file 3). Of these, 38 miRNAs* had < 10 counts. The five most abundant miRNAs* were miR-1422f* (254,460), miR-959* (226,286), miR-133a* (97,989), bantam* (67,417), and miR-20a* (16,529). Interestingly, for more than half of these miRNAs*, no complementary sequences (such as miR-959* and miR-133a*) were detected. This is inconsistent with a previous report that miRNAs* are less stable than mature miRNAs in locusts [4].

**Table 1:** **Sequences and abundance of the top 10 miRNA families in *T. multiceps***

**miRNA family name**	**Sequence**^**a**^	**Counts**
miR-71	TGAAAGACGATGGTAGTGAGA	998673
miR-315	TTTTGTATTGTTGTTGGGATGTT	774438
miR-2840	TAGGACTGGAAGACGGGAGA	496050
miR-1	TGGAATGTTGTGAAGTATGT	469997
miR-1422	AATAAAATAGTAGGACTGAT	254527
bantam	TGAGATCGCGATTACAGCTGAT	227564
miR-959	TAAGGACTTGGGTTGTAAAA	226286
miR-10	CACCCTGTAGACCCGAGTTTGA	208265
miR-4491	AATTGTGGATTGATGGACCAAA	176753
miR-4175	GGGATGTAGCTCAGTGGTAG	150022

^a^ miRNA sequence with the highest count in a single miRNA family.

### Prediction of novel candidate miRNAs

In addition to conserved miRNAs, we identified 20 candidate miRNAs. A total of 44 precursor candidates (comprising 36 different precursors, as two precursors mapped to multiple locations) were predicted using MIREAP software; these contained a Dicer cleavage site, and exhibited an appropriate secondary structure and MFE. Two candidate miRNAs mapped to multiple locations in the *E. multilocularis* genome: *tmu*-miR-0004 corresponds to eight different locations on different chromosomes, while *tmu*-miR-0039 corresponds to two different locations (Additional file 4A). In addition, *tmu*-miR-0016 (designated *tmu*-novel-03) was the most abundant, with 9,463 reads, which suggests that it has an important role in adult *T. multiceps*. As a previous study on maize miRNAs suggested that miRNA precursors with MFEIs > 0.85 are most likely to be miRNAs [38], we determined MFEIs for all candidate miRNAs using the same formula. Following this analysis, 11 of the 36 candidates (MFEI ≥ 0.85) were identified as confident novel miRNAs, including tmu-novel-01 (0.88), tmu-novel-03 (0.97), and tmu-novel-06 (0.98; others are shown in Additional file 4B).

### Experimental validation of miRNA precursors

**Figure 3:**
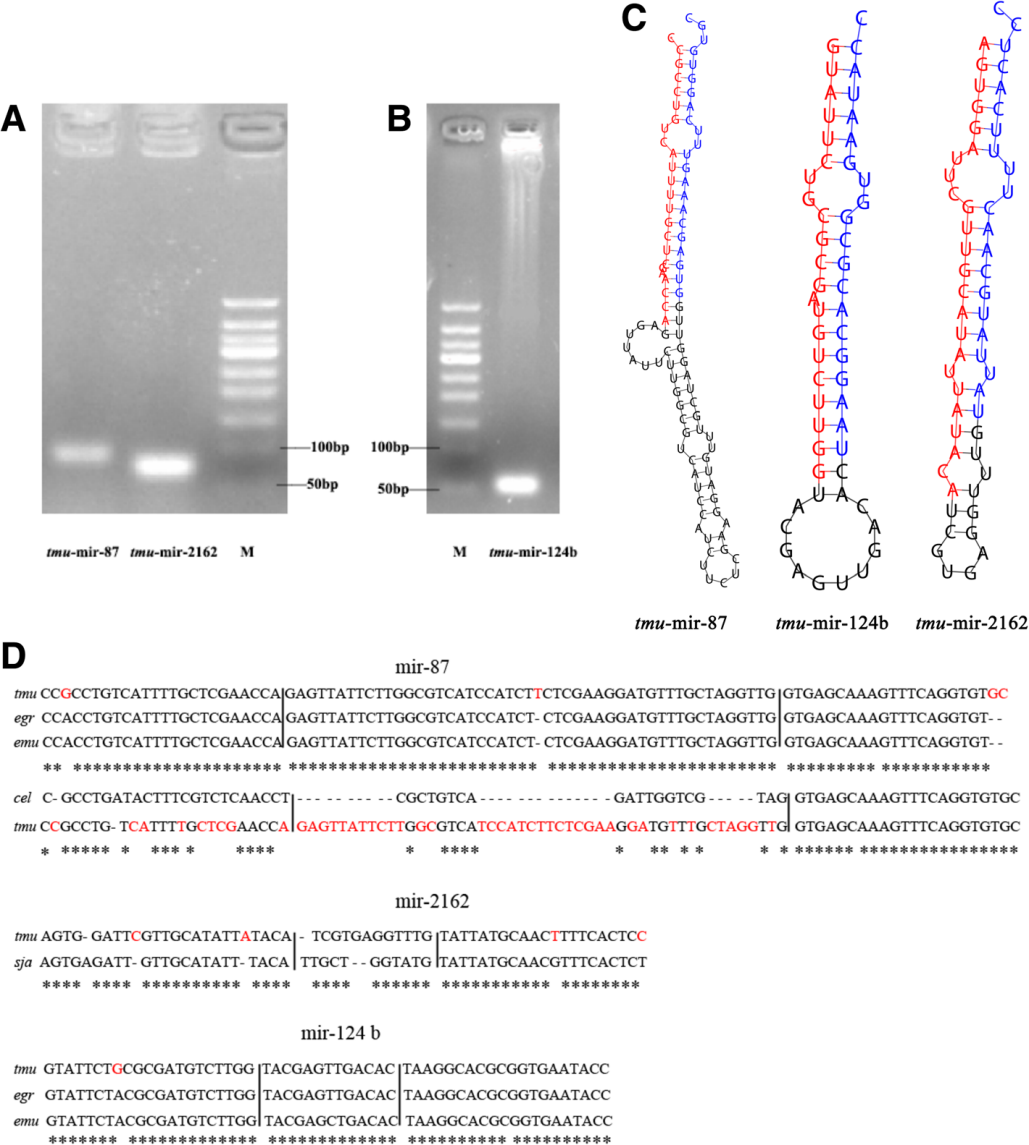
Conserved miRNAs (miRNA*/miRNA duplexes) verified in *Taenia multiceps*. (A, B) Electrophoretic analysis of genomic DNA corresponding to three conserved miRNA precursor PCR products from *T. multiceps*. **(C)** Secondary structures of *T. multiceps* miR-87, miR-124b and miR-2162 miRNA precursors. Nucleotide bases of mature miRNA-5p are marked with red color, while nucleotide bases of mature miRNA-3p are marked with blue color. **(D)** miRNA homologues with other parasites miRNAs. “*” represents the conserved site in miRNAs.

**Figure 4:**
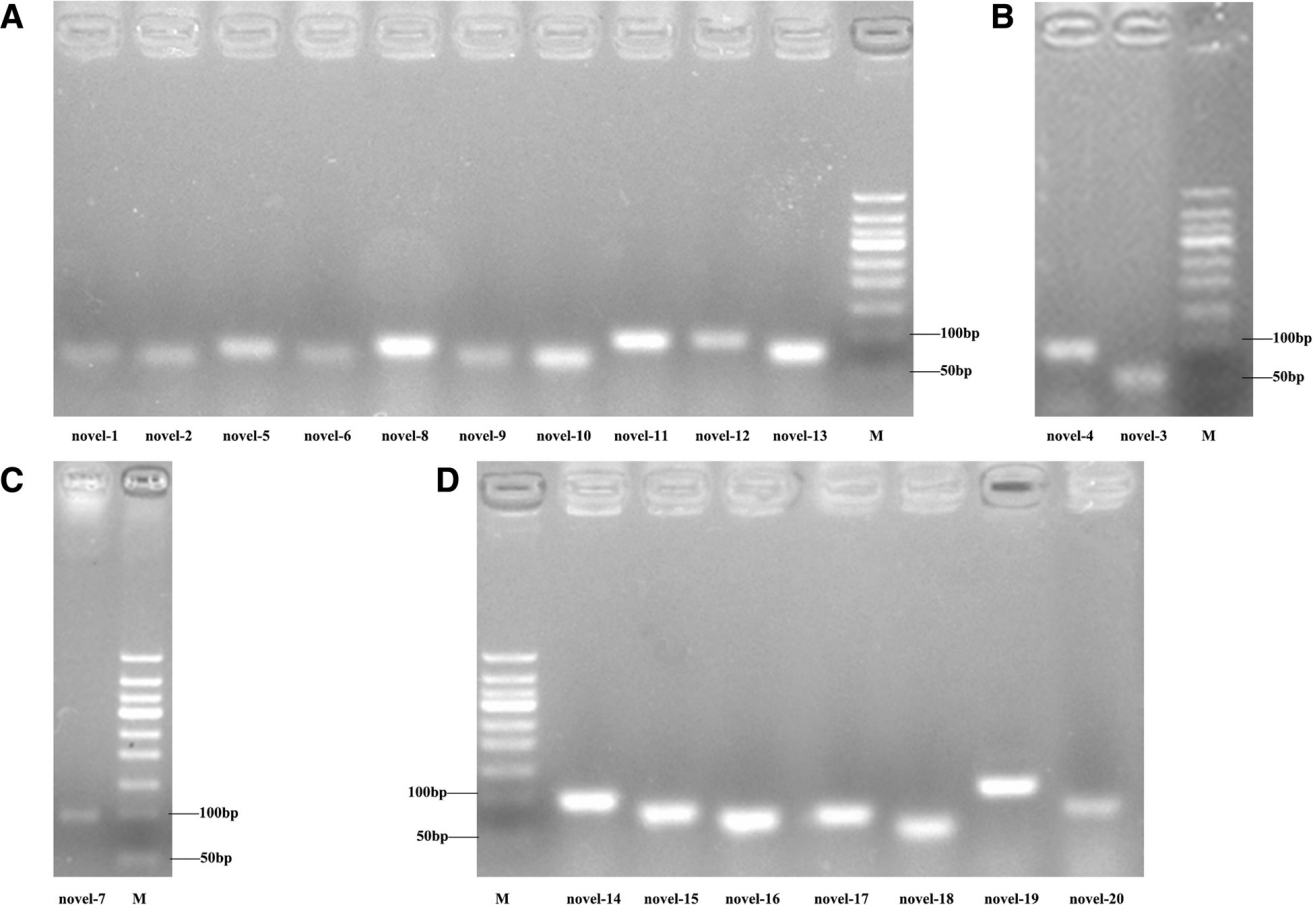
**Electrophoretic analysis of 20 novel miRNA precursor PCR products from Taenia multiceps. (A)** The actual size of precursors of novel candidate miRNA-1, 2, 5, 6, 8, 9, 10, 11, 12 and 13. **(B)** The actual size of precursors of novel candidate miRNA-3 and 4. **(C)** The actual size of precursor of novel candidate miRNA-7. **(D)** The actual size of precursors of novel candidate miRNA-14, 15, 16, 17, 18, 19, 20. “M” stands for Marker of 50bp DNA ladder (50–500 bp).

**Figure 5:**
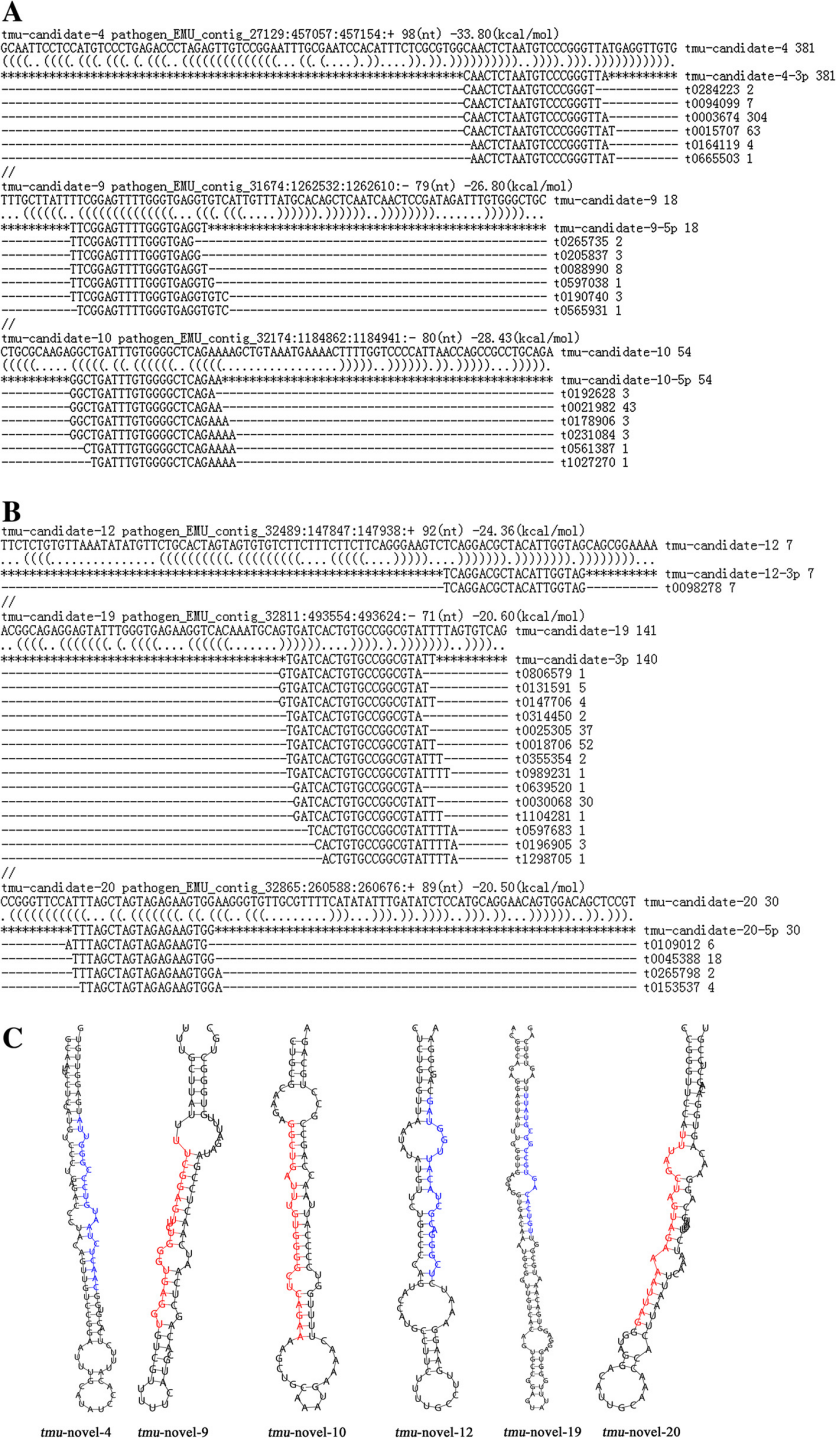
**Predicted and validated precursors of six specific miRNA. (A,B)** Location of putative miRNAs precursors in E. multilocularis genome. **(C)** Hairpin secondary structures of validated precursors of six species-specific miRNAs. Nucleotide bases of mature miRNA-5p (miRNA-3p) are marked with red (blue) color. Secondary structures were predicted by RNAfold package.

We used a PCR-based method to verify our predicted miRNA precursors, including three conserved miRNA/miRNA* duplexes (*tmu-*miR-87/miR-87*; *tmu-*miR-124b/miR-124b*; *tmu-*miR-2162/miR-2162*) and three novel miRNA/miRNA* duplexes (m0032, m0035, and m0041 were redesignated tmu-novel-14, novel-16, and novel-18, respectively). The electrophoretic analysis of three conserved miRNA precursor PCR products is shown in Figure 3A and 3B. The hairpin structures of conserved miRNA precursors are displayed in Figure 3C, and the miRNA homologues in other species *Echinococcus granulosus, E. multilocularis, Caenorhabditis elegans* and *Schistosoma japonicum* from miRBase are listed in Figure 3D. In all, 20 out of 36 novel miRNA precursor candidates could be verified using gel electrophoresis (Figure 4). 19 of 20 verified miRNAs can be folded into characteristic miRNA stem-loop secondary hairpin structures (see Additional file 5). Of these, 15 sequenced *T. multiceps* miRNA precursors have > 90% similarity with the corresponding predicted sequences from *E. multilocularis*. However, one novel miRNA precursor (*tmu*-novel-19) has low similarity (65.1%) and a different structure to the predicted *E. multilocularis* precursor, suggesting that this novel miRNA may have different functions in *T. multiceps* and *E. multilocularis.* Interestingly, six of the novel miRNAs did not share homology with sequences in the miRBase [47], and can therefore be considered specific miRNAs for Taeniidae (the sequences and structures of which are shown in Table 2 and Figure 5C), indicating they may play specific gene regulatory roles in Taeniidae cestodes. Additional file 4C showed the result of RNAfold software for predicting the secondary structure of the validated 23 (including 20 novel and 3 conserved) miRNA precursors.

**Table 2:** Six validated specific miRNAs

**Candidate**	**Sequence**	**Count**	**Validated precursor**
**name**			
***tmu*-novel-4**	CAACTCTAATGTCCCGGGTTA	381	GCAATTCCTCCATGTCCCTGAGACCCTACAGTTGTCCGGAATTTGCATATCCACATTTCTCACGTGGCAACTCTAATGTCCCGGGTTATGAGGTTGTG
***tmu*-novel-9**	TTCGGAGTTTTGGGTGAGGT	18	TTTGCTTATTTTCGGAGTTCTGGGTGAGGTGTCGTTTTTCATGCACAGCTCAATCAACTCCGATAGATTTGTGGGCTGC
***tmu*-novel-10**	GGCTGATTTGTGGGGCTCAGAA	54	CTGCGCAAGAGGCTGATTTGTGGGGCTCAGAAAAGCTGCAAATGAAAACTTTTGGTCCCCATTAACCAGCCGCCTGCAGA
***tmu*-novel-12**	TCAGGACGCTACATTGGTAG	7	TTCTCTGTGTTAAATATATGTTCTGCCCCAGTACCATGCCTTCTTTTGCCTTGAAGGAAATCTCGGGACGCTACATTGGTAGCAGCGGAAAA
***tmu*-novel-19**	TGATCACTGTGCCGGCGTATT	140	ACGGCAGAGGAGTATTTGGGTGAGGAGGTGACAAATGCGGTTGTCACAGTGCCGGAGTATTTGGGTGAGGAGGTGACAAATGCGGTTGTCACAGTGCCGGCGTATTTTAGTGTCAG
***tmu*-novel-20**	TTTAGCTAGTAGAGAAGTGG	30	CCGGGTTCCATTTAGCTAGTAGAAAATTAGGGTAGGACATTGCAAACCACTTAATTCAAATCTTTATGCAGGAACAGTGGACAGCTCCGT

“Count” means the amount of the candidate novel miRNA. “Validated precursor” shows the corresponding precursor sequence obtained from genomic PCR validation.

### Target gene prediction

To determine the functions of the 1,006 conserved miRNAs and 20 novel miRNAs in *T. multiceps*, we predicted their putative targets using a panel of 31,282 *T. multiceps* transcriptome unigenes; 10,121,914 and 181,077 target unigene sites (Additional file 6), respectively, were obtained. Unigene 1299, annotated as a fatty acid binding protein (FABP), was targeted by *tmu*-novel-15. In addition, unigene 18109, obtained by annotation of heat shock proteins (HSP), was targeted by tmu-novel-07. After mapping to the GO database, all putative target genes were classified into diverse GO functional groups. The GO functions of the predicted targets of novel miRNAs are shown in Additional file 7, Additional file 8, Additional file 9. In addition, KEGG pathway annotations revealed the biological functions of target unigenes of novel (Additional file 10) miRNAs.

## Discussion

Animal miRNAs are important regulators of gene expression that function through imprecise complementarity to their mRNA targets [48]. Hence, it is essential to understand the functions of miRNAs throughout the lifecycle of parasites and determine how these may regulate host infection [49]. We identified adult *T. multiceps* miRNAs by Illumina sequencing. Since the adult worms used for RNA extraction were gravid, the identified miRNAs could also be expressed by the oncospheres. This dataset might help the further study of miRNAs of *T. multiceps* oncospheres. Our results showed that a low percentage (17.68%) of reads had a perfect match to sequences and only 39,765 out of a total of 1,379,196 (19.46%) unique sRNAs matched sequences in the related *E. multilocularis* genome, as proposed in previous study of *Clonorchis sinensis* sRNAs when matching which into the related genome of *Schistosoma japonicum*[31]. This lack of homology may be due to using the *E. multilocularis* reference genome, as the *T. multiceps* whole-genome sequence is currently unavailable. *T. multiceps* and *E. multilocularis* are both *Taeniidae* cestodes, but significant differences exist between them [50].

Some kinds of miRNAs were expressed with high predominance. A previous data showed that known miRNAs of *Taenia saginata* were expression predominated by miR-71 (69.53% of the total reads) [51], which was also detected to be the most abundant conserved miRNA in adult *T. multiceps* (16.43%). Similar to *T. saginata*, members of the common miR-40 family were not detected in *T. multiceps* in this study. However, low expression of 12 members of another common miRNA family, let-7 (not found in *T. saginata*[51]), was shared among *T. multiceps* (17,125 counts), *E. granulosus* and *E. multilocularis*. Considering that *T. saginata* and *T. multiceps* belong to the same genus, *Taenia*, the absence of let-7 miRNAs in *T. saginata,* their presence in *T. multiceps* and their universal existence in some other species suggest that non-recovery of let-7 in *T. saginata* may be attributed to the specific stage of the parasite examined or experimental methods. In addition, we compared conserved miRNAs of *T. multiceps* with *E. granulosus* (*E. multilocularis*) which were obtained from miRBase. The miR-8, which was found in *E. granulosus* and *E. multilocularis,* was not identified in adult *T. multiceps.* While miR-96 was found both in *E. multilocularis* and *T. multiceps* but not in *E. granulosus.* Among these three species, two other miRNAs (miR-4990 and miR-4991) were only detected in *E. granulosus*. These findings could be useful for miRNAs’ function researches.

The miR-1, a highly conserved muscle-specific miRNA, is represented in the top four abundant *T. multiceps* miRNAs. Target searching identified many unigene targets of miR-1, including unigenes 754 and 757, both of which have been annotated as malate dehydrogenase. This finding is consistent with a recent report that malate dehydrogenase is regulated by miR-1 in *Trichomonas vaginalis*[52], illustrating that our method for miRNA target prediction was correcet. As malate dehydrogenase participates in the citric acid cycle [53] and *T. multiceps* are intestinal parasites that produce ATP mainly through anaerobic glycolysis (not the citric acid cycle) [54], it will be interesting to investigate how miR-1 and malate dehydrogenase interact in *T. multiceps*. However, we can infer that miR-1 may interact with other miRNAs to regulate glucose metabolism in *T. multiceps.*

**Figure 6:**
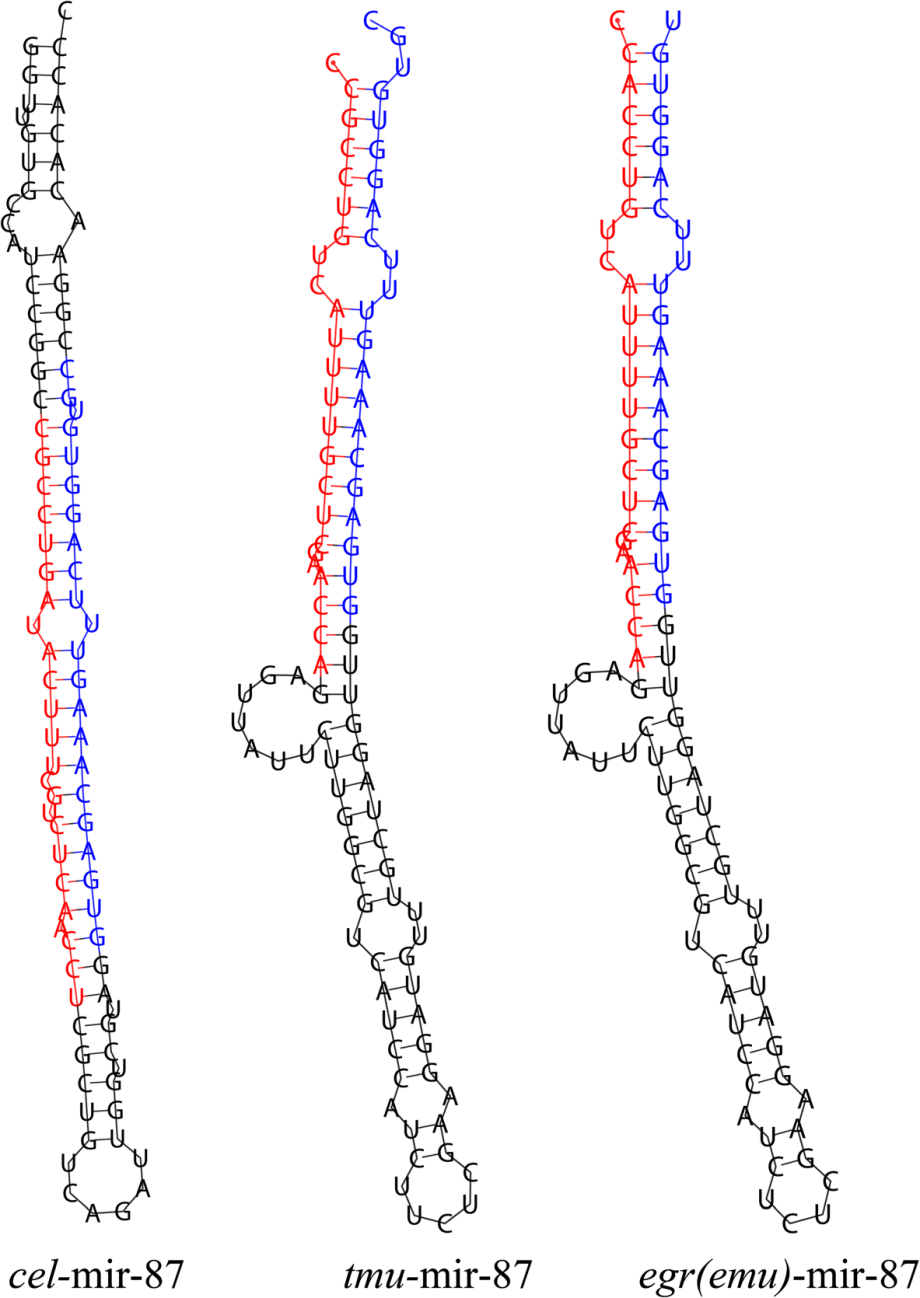
**Comparison the secondary structures of miR-87 precursor in *C. elegans, T. multiceps, E. granulosus and E. multilocularis*.** The *tmu*-mir-87 shares the similar secondary structure with *egr*-mir-87 and *emu*-mir-87, but differs from the *cel*-mir-87. Nucleotide bases of mature miRNA-5p are marked with red color, while nucleotide bases of mature miRNA-3p are marked with blue color.

To make sure the validity of the identified conserved miRNAs, we randomly selected three conserved miRNA/miRNA* duplexes for validation. However, the on-going precursor validation and detailed analyses of miRNAs’ structure and function are required in further studies. Compared with the *cel*-miR-87 from miRBase, the mature miRNA *tmu-*miR-87-5p seemed not so highly conserved (Figure 3D) and the secondary structure of *tmu*-miR-87 precursor seemed abnormal (Figure 6). However, compared with *egr-*miR-87 and *emu-*miR-87, the sequence of mature miRNA *tmu-*miR-87-5p was highly conserved with only one nucleotide variation (Figure 3D) and the hairpin structure was extremely similar (Figure 6).

As miRNAs* are less stable than mature miRNAs, being quickly degraded after mature miRNA enters the RISC complex [4,9], we did not expect these molecules to be sequenced at a high frequency. However, more than half of the 95 miRNAs* (including the top three abundant miRNAs*: miR-1422f*, miR-959*, and miR-133a*) were sequenced with high frequency in this study without their corresponding mature strands (miR-1422f, miR-959 and miR-133a) detected. Furthermore, many miRNAs* (e.g., miR-20a* and miR-216b*) were sequenced at similar frequencies to their corresponding mature miRNAs (miR-20a and miR-216b). This suggests that miRNAs* may play functional roles in *T. multiceps* and that small RNA precursors may form two different regulatory small RNAs, as has been proposed by previous studies in *E. granulosus* and *Drosophila melanogaster*[6,55]. The miR-1422 family is the second largest family in *C. sinensis*, consisting of 13 members [31]. Although miR-1422f* was the most abundant miRNA* sequenced in *T. multiceps*, only two miR-1422 family members (miR-1422f* and miR-1422j) were detected. The miR-1422 family reads therefore suggest a strong bias (99.97%) toward miR-1422* in adult *T. multiceps*. miR-959*, whose mature strand has only previously been reported in *D. melanogaster*[56], was the second most abundant miRNA* in *T. multiceps*.

We identified 20 candidate novel *T. multiceps* miRNAs using PCR-based methods, thus providing evidence that computational methods used for miRNA prediction were correctly executed. We also verified that miRNA precursor and flanking sequences could be folded into typical miRNA-like hairpin structures. The sequences and structures of these newly candidate miRNAs should help future studies of miRNA function in *T. multiceps*. All 20 novel miRNAs are regarded as candidate novel miRNAs here. Additional evidence is required to further validation their reliability. However, we failed to validate 16 other predicted novel miRNAs, including two candidate miRNAs with multiple loci in the *E. multilocularis* genome. We consider that this may be due to 1). the poor quality of primers and the difficulty of primer designing as we designed primers to amplify full-length predicted precursors. 2). the miRNA precursors were predicted by comparing to a related cestode species *E. multilocularis* instead of *T. multiceps*, which may highlight differences between the cestode species at the nucleotide level.

More importantly, we discovered six specific miRNAs with different structures in *T. multiceps* compared to *E. multilocularis* genome. As they both belong to Taeniidae, we speculate that these specific miRNAs might have specialized functions related to cellular processes in Taeniidae*,* and may therefore provide novel therapeutics for this disease [49]. Our identification of three candidate novel miRNA/miRNA* pairs in *T. multiceps* (Additional file 4B), m0032, m0035, and m0041 (predicted by computational methods), equivalent to the PCR-validated *tmu*-novel-14, *tmu*-novel-16 and *tmu*-novel-18, respectively, provides more supporting evidence that these are authentic miRNAs. Interestingly, the precursor of the 13 low-confidence candidate novel miRNA (MFEI < 0.85) determined using the MFEI formula could be validated by PCR-based method and meeting the secondary structure and the required MFE (Additional file 2). This is likely to be due to the differences that exist between animal and plant miRNAs [57]. Together with our recent study of *Dirofilaria immitis* miRNAs identification [58], these results indicate that the MFEI formula for plant miRNAs may not be suitable for selecting high-confidence animal miRNAs, or at least not *T. multiceps* and *D. immitis* miRNAs.

It was a challenge to predict miRNAs targets in *T. multiceps*, as the whole-genome sequence of *T. multiceps* is currently unavailable and *T. multiceps* expressed sequence tags (ESTs) are lacking. In previous miRNA studies of locust and pinewood nematode, researchers chose EST databases to predict miRNA targets [4,28]. We used *T. multiceps* transcriptome unigenes obtained by high-throughput sequencing as an alternative method to predict miRNA targets because animal miRNAs can bind to a broad spectrum of different target mRNAs through imprecise base pairing [48]; and unigene populations are derived from mRNAs expressed under specific conditions [59]. A large number of the 181,077 target sites in 31,282 adult *T. multiceps* unigenes were predicted by only 20 candidate novel miRNAs. This indicates that a single novel miRNA may have a large number of unigene target sites, thus suggesting that miRNAs have diverse functions. For example, we identified 8,665 target sites in multiple unigenes for the most abundant novel miRNA, *tmu*-novel-03. Most unigenes were predicted to contain more than one target site for different novel miRNAs. For example, unigene 17356, annotated as phosphoenolpyruvate carboxykinase (PEPCK), was targeted by both *tmu*-novel-01 and *tmu*-novel-11 (Additional file 6). As we discussed in our previous study of transcriptome that the lack of this important enzyme would interrupt *T. multiceps* glycometabolism [53], we presume that *tmu*-novel-1 and *tmu*-novel-11 play important roles in regulating PEPCK executive functions in *T. multiceps*. We showed all the target genes we got in Additional file 6. Nevertheless, further validation of putative miRNA targets is required.

## Conclusions

In this study, adult *T. multiceps* miRNAs were characterized and identified using Illumina sequencing to provide a platform for further research into the regulation of gene networks in this organism. Our discovery of novel miRNAs, including six specific miRNAs for Taeniidae, in adult *T. multiceps* may help to develop new therapeutic approaches for diseases caused by this parasite. We predicted target mRNAs for 20 candidate novel miRNAs and investigated the functions of target gene candidates in adult *T. multiceps*. Ongoing work is needed to verify the remaining 16 candidate novel miRNAs, validate their target genes, and elucidate the functions of newly identified *T. multiceps* miRNAs.

### Availability of supporting data

The dataset of small RNAs (13.5 million raw reads) from adult *T. multiceps* was deposited in NCBI Gene Expression Omnibus [46] with the accession number [GEO: GSE35647].

## Competing interests

The authors declare that they have no competing interests.

## Authors’ contributions

XHW and GYY conceived and designed the whole experiment. DYY, RHZ, WPZ, NW and JHW performed the experiments of verification. NY, HMN and YX analyzed the data. XBG, SXW and XRP contributed reagents/materials/analysis tools. XHW and YF drafted the manuscript. All authors read and approved the final manuscript.

## Supplementary Material

Additional file 1 **PCR primers for three conserved and 20 novel *Taenia multiceps* miRNA precursors.** A total of 23 pairs of primers were used successfully in validation experiments. Click here for file

Additional file 2 **Distribution of *Taenia multiceps *small RNAs across different chromosomes of *Echinococcus multilocularis.*** “sense” and “anti-sense” represent “+” and “-” strands of Chromosomes, respectively. Click here for file

Additional file 3 **Conserved *Taenia multiceps *miRNAs based on miRBase 17.0.** (A) Conserved miRNA families and miRNAs (B) List of *Taenia multiceps* antisense miRNAs (miRNAs*). Click here for file

Additional file 4 **Details of the novel miRNA precursors.** (A) Novel miRNA precursors predicted by MIREAP software using the *Echinococcus multilocularis* genome. (B) Confidence level of novel miRNAs selected using the minimal folding free energy index. (C) The result of RNAfold software for predicting the secondary structure of validated miRNA precusors. Click here for file

Additional file 5 **Hairpin secondary structures of 20 novel Taenia multiceps miRNA precursors.** Nucleotide bases of mature miRNA-5p (miRNA-3p) are marked with red (blue) color. The stem-loop structure of all validated novel miRNA precursors was predicted by RNAfold software. “Tmu-novel candidate-17” represents the non-typical secondary structure. Three novel miRNA/miRNA* pairs (tmu-novel-14, tmu-novel-16 amd tmu-novel-18) were validated. All 20 novel miRNAs noted in this Figure are the candidate names of novel miRNAs of T. multiceps. Click here for file

Additional file 6 **Novel miRNA target sites conserved in *Taenia multiceps *unigenes.** 181,077 target unigenes were predicted for 20 novel miRNAs based on 31,282 adult T. multiceps transcriptome, which were obtained by Illumina sequencing and Trinity assembling. Click here for file

Additional file 7 **Cellular component GO annotations for candidate target unigenes for novel *Taenia multiceps *miRNAs.** 5,696 target unigenes were assigned to 324 GO-terms from “Cellular component” ontology. “Gene Ontology term” means GO terms from Component Ontology with P-value as good as or better than 1. “Cluster frequency” stands for number and frequency of target unigenes related to this term. “Genome frequency of use” represents number and frequency of coding genes related to this term. Click here for file

Additional file 8 **Molecular function GO annotations for candidate target unigenes of novel *Taenia multiceps *miRNAs.** 7,095 target unigenes were assigned to 604 GO-terms from “Molecular function” ontology. Click here for file

Additional file 9 **Biological process GO annotations for candidate target unigenes of novel *Taenia multiceps *miRNAs.** 6,178 target unigenes were assigned to 2,262 GO-terms from “Biological process” ontology. Click here for file

Additional file 10 **KEGG pathways for predicted target genes of *Taenia multiceps *novel miRNAs.** 9,306 target unigenes were assigned into 240 KEGG pathways. . “Pvalue” and “Qvalue” represent P-value before correction and corrected P-value, respectively. Click here for file
