# Supplementary figures and images for: Identification of neglected cestode Taenia multiceps microRNAs by illumina sequencing and bioinformatic analysis
Source: BMC Vet Res. 2013 Aug 13;9:162. doi: 10.1186/1746-6148-9-162 (PMC3849562; doi:10.1186/1746-6148-9-162)

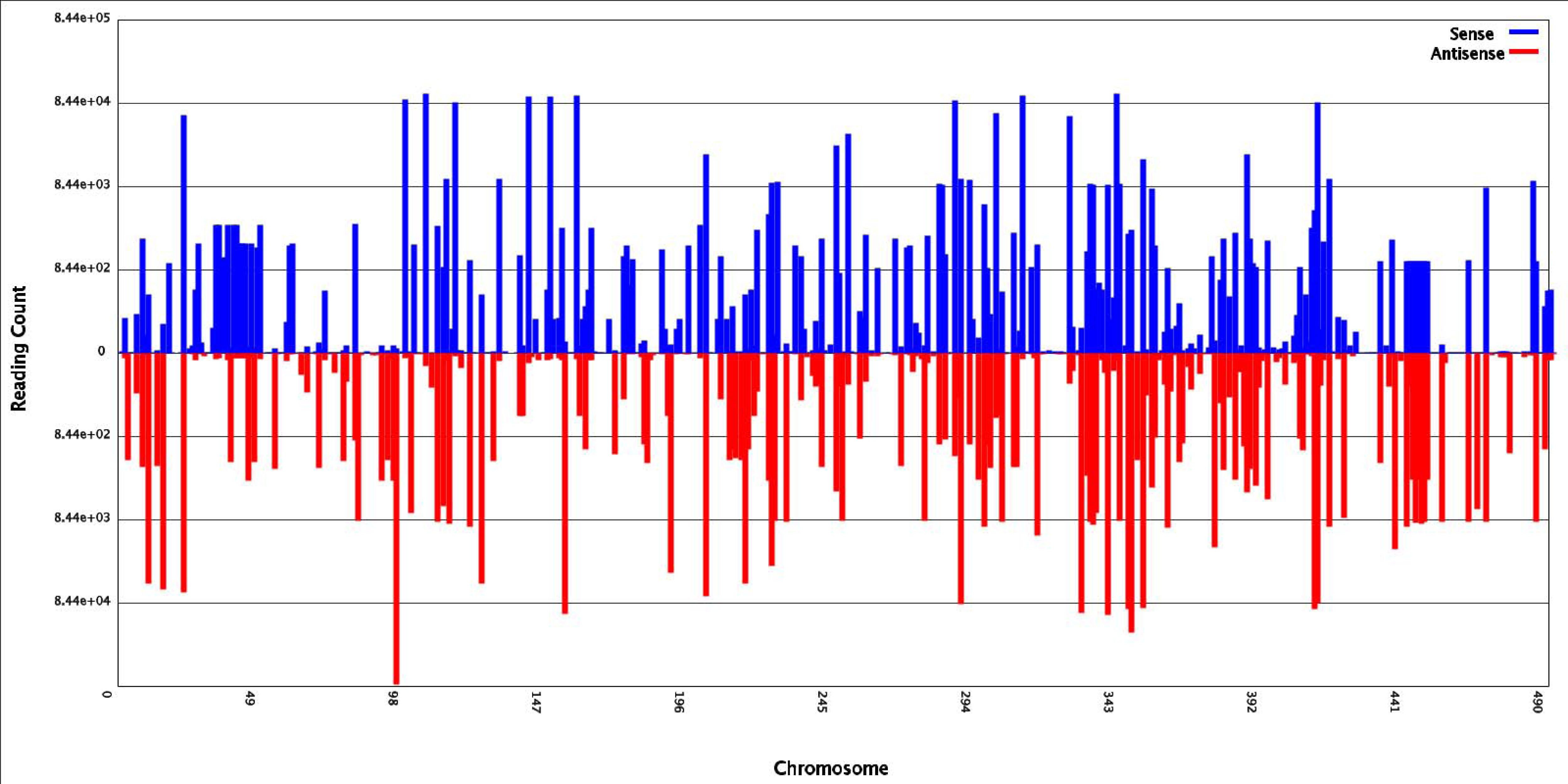

Supplement: Additional file 2 — Distribution of Taenia multiceps small RNAs across different chromosomes of Echinococcus multilocularis. “sense” and “anti-sense” represent “+” and “-” strands of Chromosomes, respectively. [file 1746-6148-9-162-S2.tiff]
