# Supplementary material for: Identification of neglected cestode Taenia multiceps microRNAs by illumina sequencing and bioinformatic analysis
Source: BMC Vet Res. 2013 Aug 13;9:162. doi: 10.1186/1746-6148-9-162 (PMC3849562; doi:10.1186/1746-6148-9-162)
Supplement: Additional file 7 — Cellular component GO annotations for candidate target unigenes for novel Taenia multiceps miRNAs. 5,696 target unigenes were assigned to 324 GO-terms from “Cellular component” ontology. “Gene Ontology term” means GO terms from Component Ontology with P-value as good as or better than 1. “Cluster frequency” stands for number and frequency of target unigenes related to this term. “Genome frequency of use” represents number and frequency of coding genes related to this term. [file 1746-6148-9-162-S7.html]

Terms for Taenia\_multiceps\_C


## Terms for Taenia\_multiceps\_C

---


### Result Table

|  |
| --- |
| **Terms from the Component Ontology with p-value as good or better than 1** |

| Gene Ontology term | Cluster frequency | Genome frequency of use | Corrected P-value |
| --- | --- | --- | --- |
| nuclear part | 626 out of 5696 genes, 11.0% | 627 out of 5777 genes, 10.9% | 0.30280 |
| nucleus | 728 out of 5696 genes, 12.8% | 730 out of 5777 genes, 12.6% | 0.43720 |
| membrane-enclosed lumen | 492 out of 5696 genes, 8.6% | 493 out of 5777 genes, 8.5% | 1 |
| organelle lumen | 490 out of 5696 genes, 8.6% | 491 out of 5777 genes, 8.5% | 1 |
| intracellular organelle lumen | 490 out of 5696 genes, 8.6% | 491 out of 5777 genes, 8.5% | 1 |
| nuclear lumen | 435 out of 5696 genes, 7.6% | 436 out of 5777 genes, 7.5% | 1 |
| organelle | 3326 out of 5696 genes, 58.4% | 3363 out of 5777 genes, 58.2% | 1 |
| intracellular membrane-bounded organelle | 2609 out of 5696 genes, 45.8% | 2636 out of 5777 genes, 45.6% | 1 |
| membrane-bounded organelle | 2664 out of 5696 genes, 46.8% | 2692 out of 5777 genes, 46.6% | 1 |
| intracellular organelle | 3281 out of 5696 genes, 57.6% | 3318 out of 5777 genes, 57.4% | 1 |
| endomembrane system | 243 out of 5696 genes, 4.3% | 243 out of 5777 genes, 4.2% | 1 |
| intracellular | 4233 out of 5696 genes, 74.3% | 4286 out of 5777 genes, 74.2% | 1 |
| organelle part | 1617 out of 5696 genes, 28.4% | 1633 out of 5777 genes, 28.3% | 1 |
| protein complex | 955 out of 5696 genes, 16.8% | 963 out of 5777 genes, 16.7% | 1 |
| intracellular part | 4151 out of 5696 genes, 72.9% | 4204 out of 5777 genes, 72.8% | 1 |
| nucleoplasm | 169 out of 5696 genes, 3.0% | 169 out of 5777 genes, 2.9% | 1 |
| nucleoplasm part | 166 out of 5696 genes, 2.9% | 166 out of 5777 genes, 2.9% | 1 |
| intracellular organelle part | 1475 out of 5696 genes, 25.9% | 1491 out of 5777 genes, 25.8% | 1 |
| cell projection | 317 out of 5696 genes, 5.6% | 319 out of 5777 genes, 5.5% | 1 |
| neuron projection | 116 out of 5696 genes, 2.0% | 116 out of 5777 genes, 2.0% | 1 |
| cytoskeleton | 627 out of 5696 genes, 11.0% | 633 out of 5777 genes, 11.0% | 1 |
| Golgi apparatus | 103 out of 5696 genes, 1.8% | 103 out of 5777 genes, 1.8% | 1 |
| microtubule cytoskeleton | 438 out of 5696 genes, 7.7% | 442 out of 5777 genes, 7.7% | 1 |
| Golgi apparatus part | 92 out of 5696 genes, 1.6% | 92 out of 5777 genes, 1.6% | 1 |
| cell junction | 89 out of 5696 genes, 1.6% | 89 out of 5777 genes, 1.5% | 1 |
| integral to membrane | 170 out of 5696 genes, 3.0% | 171 out of 5777 genes, 3.0% | 1 |
| cell body | 76 out of 5696 genes, 1.3% | 76 out of 5777 genes, 1.3% | 1 |
| macromolecular complex | 1343 out of 5696 genes, 23.6% | 1360 out of 5777 genes, 23.5% | 1 |
| cell projection part | 73 out of 5696 genes, 1.3% | 73 out of 5777 genes, 1.3% | 1 |
| cell periphery | 366 out of 5696 genes, 6.4% | 370 out of 5777 genes, 6.4% | 1 |
| plasma membrane part | 292 out of 5696 genes, 5.1% | 295 out of 5777 genes, 5.1% | 1 |
| clathrin-coated vesicle | 64 out of 5696 genes, 1.1% | 64 out of 5777 genes, 1.1% | 1 |
| vesicle | 216 out of 5696 genes, 3.8% | 218 out of 5777 genes, 3.8% | 1 |
| spliceosomal complex | 63 out of 5696 genes, 1.1% | 63 out of 5777 genes, 1.1% | 1 |
| vacuole | 62 out of 5696 genes, 1.1% | 62 out of 5777 genes, 1.1% | 1 |
| endosome | 56 out of 5696 genes, 1.0% | 56 out of 5777 genes, 1.0% | 1 |
| plasma membrane | 345 out of 5696 genes, 6.1% | 349 out of 5777 genes, 6.0% | 1 |
| cytoskeletal part | 341 out of 5696 genes, 6.0% | 345 out of 5777 genes, 6.0% | 1 |
| mitochondrial lumen | 53 out of 5696 genes, 0.9% | 53 out of 5777 genes, 0.9% | 1 |
| cell-cell junction | 51 out of 5696 genes, 0.9% | 51 out of 5777 genes, 0.9% | 1 |
| vesicle membrane | 51 out of 5696 genes, 0.9% | 51 out of 5777 genes, 0.9% | 1 |
| synapse | 51 out of 5696 genes, 0.9% | 51 out of 5777 genes, 0.9% | 1 |
| nuclear body | 50 out of 5696 genes, 0.9% | 50 out of 5777 genes, 0.9% | 1 |
| membrane coat | 50 out of 5696 genes, 0.9% | 50 out of 5777 genes, 0.9% | 1 |
| coated membrane | 50 out of 5696 genes, 0.9% | 50 out of 5777 genes, 0.9% | 1 |
| nuclear chromosome | 47 out of 5696 genes, 0.8% | 47 out of 5777 genes, 0.8% | 1 |
| cytoplasmic vesicle | 180 out of 5696 genes, 3.2% | 182 out of 5777 genes, 3.2% | 1 |
| lytic vacuole | 45 out of 5696 genes, 0.8% | 45 out of 5777 genes, 0.8% | 1 |
| nuclear membrane-endoplasmic reticulum network | 45 out of 5696 genes, 0.8% | 45 out of 5777 genes, 0.8% | 1 |
| membrane-bounded vesicle | 178 out of 5696 genes, 3.1% | 180 out of 5777 genes, 3.1% | 1 |
| endoplasmic reticulum membrane | 44 out of 5696 genes, 0.8% | 44 out of 5777 genes, 0.8% | 1 |
| non-membrane-bounded organelle | 997 out of 5696 genes, 17.5% | 1011 out of 5777 genes, 17.5% | 1 |
| intracellular non-membrane-bounded organelle | 997 out of 5696 genes, 17.5% | 1011 out of 5777 genes, 17.5% | 1 |
| nuclear envelope | 42 out of 5696 genes, 0.7% | 42 out of 5777 genes, 0.7% | 1 |
| chromosome, centromeric region | 40 out of 5696 genes, 0.7% | 40 out of 5777 genes, 0.7% | 1 |
| cytoplasmic vesicle membrane | 40 out of 5696 genes, 0.7% | 40 out of 5777 genes, 0.7% | 1 |
| cytoplasmic vesicle part | 40 out of 5696 genes, 0.7% | 40 out of 5777 genes, 0.7% | 1 |
| coated vesicle membrane | 39 out of 5696 genes, 0.7% | 39 out of 5777 genes, 0.7% | 1 |
| anchoring junction | 39 out of 5696 genes, 0.7% | 39 out of 5777 genes, 0.7% | 1 |
| chromosomal part | 166 out of 5696 genes, 2.9% | 168 out of 5777 genes, 2.9% | 1 |
| cytoplasmic membrane-bounded vesicle | 163 out of 5696 genes, 2.9% | 165 out of 5777 genes, 2.9% | 1 |
| organelle membrane | 361 out of 5696 genes, 6.3% | 366 out of 5777 genes, 6.3% | 1 |
| extracellular region | 97 out of 5696 genes, 1.7% | 98 out of 5777 genes, 1.7% | 1 |
| extracellular matrix | 36 out of 5696 genes, 0.6% | 36 out of 5777 genes, 0.6% | 1 |
| cell leading edge | 35 out of 5696 genes, 0.6% | 35 out of 5777 genes, 0.6% | 1 |
| small nuclear ribonucleoprotein complex | 34 out of 5696 genes, 0.6% | 34 out of 5777 genes, 0.6% | 1 |
| coated vesicle | 93 out of 5696 genes, 1.6% | 94 out of 5777 genes, 1.6% | 1 |
| transcription factor complex | 33 out of 5696 genes, 0.6% | 33 out of 5777 genes, 0.6% | 1 |
| spindle | 33 out of 5696 genes, 0.6% | 33 out of 5777 genes, 0.6% | 1 |
| nuclear chromosome part | 33 out of 5696 genes, 0.6% | 33 out of 5777 genes, 0.6% | 1 |
| endoplasmic reticulum | 90 out of 5696 genes, 1.6% | 91 out of 5777 genes, 1.6% | 1 |
| cell | 5546 out of 5696 genes, 97.4% | 5625 out of 5777 genes, 97.4% | 1 |
| cell part | 5546 out of 5696 genes, 97.4% | 5625 out of 5777 genes, 97.4% | 1 |
| Golgi membrane | 32 out of 5696 genes, 0.6% | 32 out of 5777 genes, 0.6% | 1 |
| axoneme | 32 out of 5696 genes, 0.6% | 32 out of 5777 genes, 0.6% | 1 |
| chromatin remodeling complex | 31 out of 5696 genes, 0.5% | 31 out of 5777 genes, 0.5% | 1 |
| cell cortex | 30 out of 5696 genes, 0.5% | 30 out of 5777 genes, 0.5% | 1 |
| membrane fraction | 86 out of 5696 genes, 1.5% | 87 out of 5777 genes, 1.5% | 1 |
| chromosome | 208 out of 5696 genes, 3.7% | 211 out of 5777 genes, 3.7% | 1 |
| cytoplasm | 1470 out of 5696 genes, 25.8% | 1492 out of 5777 genes, 25.8% | 1 |
| I band | 29 out of 5696 genes, 0.5% | 29 out of 5777 genes, 0.5% | 1 |
| methyltransferase complex | 29 out of 5696 genes, 0.5% | 29 out of 5777 genes, 0.5% | 1 |
| cell cortex part | 29 out of 5696 genes, 0.5% | 29 out of 5777 genes, 0.5% | 1 |
| microtubule organizing center | 84 out of 5696 genes, 1.5% | 85 out of 5777 genes, 1.5% | 1 |
| cilium | 28 out of 5696 genes, 0.5% | 28 out of 5777 genes, 0.5% | 1 |
| centrosome | 27 out of 5696 genes, 0.5% | 27 out of 5777 genes, 0.5% | 1 |
| protein serine/threonine phosphatase complex | 27 out of 5696 genes, 0.5% | 27 out of 5777 genes, 0.5% | 1 |
| axon | 27 out of 5696 genes, 0.5% | 27 out of 5777 genes, 0.5% | 1 |
| condensed chromosome | 25 out of 5696 genes, 0.4% | 25 out of 5777 genes, 0.4% | 1 |
| Golgi-associated vesicle | 25 out of 5696 genes, 0.4% | 25 out of 5777 genes, 0.4% | 1 |
| adherens junction | 25 out of 5696 genes, 0.4% | 25 out of 5777 genes, 0.4% | 1 |
| synapse part | 25 out of 5696 genes, 0.4% | 25 out of 5777 genes, 0.4% | 1 |
| extracellular region part | 76 out of 5696 genes, 1.3% | 77 out of 5777 genes, 1.3% | 1 |
| vesicle coat | 24 out of 5696 genes, 0.4% | 24 out of 5777 genes, 0.4% | 1 |
| histone methyltransferase complex | 24 out of 5696 genes, 0.4% | 24 out of 5777 genes, 0.4% | 1 |
| histone acetyltransferase complex | 23 out of 5696 genes, 0.4% | 23 out of 5777 genes, 0.4% | 1 |
| nuclear chromatin | 23 out of 5696 genes, 0.4% | 23 out of 5777 genes, 0.4% | 1 |
| proteasome accessory complex | 23 out of 5696 genes, 0.4% | 23 out of 5777 genes, 0.4% | 1 |
| microtubule associated complex | 128 out of 5696 genes, 2.2% | 130 out of 5777 genes, 2.3% | 1 |
| actin cytoskeleton | 72 out of 5696 genes, 1.3% | 73 out of 5777 genes, 1.3% | 1 |
| proteinaceous extracellular matrix | 22 out of 5696 genes, 0.4% | 22 out of 5777 genes, 0.4% | 1 |
| axon part | 22 out of 5696 genes, 0.4% | 22 out of 5777 genes, 0.4% | 1 |
| dendrite | 21 out of 5696 genes, 0.4% | 21 out of 5777 genes, 0.4% | 1 |
| extrinsic to membrane | 20 out of 5696 genes, 0.4% | 20 out of 5777 genes, 0.3% | 1 |
| intrinsic to organelle membrane | 20 out of 5696 genes, 0.4% | 20 out of 5777 genes, 0.3% | 1 |
| contractile fiber | 66 out of 5696 genes, 1.2% | 67 out of 5777 genes, 1.2% | 1 |
| cell fraction | 293 out of 5696 genes, 5.1% | 298 out of 5777 genes, 5.2% | 1 |
| transport vesicle membrane | 19 out of 5696 genes, 0.3% | 19 out of 5777 genes, 0.3% | 1 |
| pigment granule | 19 out of 5696 genes, 0.3% | 19 out of 5777 genes, 0.3% | 1 |
| contractile fiber part | 62 out of 5696 genes, 1.1% | 63 out of 5777 genes, 1.1% | 1 |
| cytoplasmic part | 1375 out of 5696 genes, 24.1% | 1397 out of 5777 genes, 24.2% | 1 |
| DNA-directed RNA polymerase II, holoenzyme | 17 out of 5696 genes, 0.3% | 17 out of 5777 genes, 0.3% | 1 |
| AP-type membrane coat adaptor complex | 17 out of 5696 genes, 0.3% | 17 out of 5777 genes, 0.3% | 1 |
| nuclear periphery | 17 out of 5696 genes, 0.3% | 17 out of 5777 genes, 0.3% | 1 |
| kinetochore | 16 out of 5696 genes, 0.3% | 16 out of 5777 genes, 0.3% | 1 |
| condensed nuclear chromosome | 16 out of 5696 genes, 0.3% | 16 out of 5777 genes, 0.3% | 1 |
| basolateral plasma membrane | 16 out of 5696 genes, 0.3% | 16 out of 5777 genes, 0.3% | 1 |
| extrinsic to plasma membrane | 16 out of 5696 genes, 0.3% | 16 out of 5777 genes, 0.3% | 1 |
| clathrin coated vesicle membrane | 16 out of 5696 genes, 0.3% | 16 out of 5777 genes, 0.3% | 1 |
| nonmotile primary cilium | 16 out of 5696 genes, 0.3% | 16 out of 5777 genes, 0.3% | 1 |
| A band | 16 out of 5696 genes, 0.3% | 16 out of 5777 genes, 0.3% | 1 |
| myofibril | 58 out of 5696 genes, 1.0% | 59 out of 5777 genes, 1.0% | 1 |
| endoplasmic reticulum part | 57 out of 5696 genes, 1.0% | 58 out of 5777 genes, 1.0% | 1 |
| mitochondrial matrix | 15 out of 5696 genes, 0.3% | 15 out of 5777 genes, 0.3% | 1 |
| cell-substrate adherens junction | 15 out of 5696 genes, 0.3% | 15 out of 5777 genes, 0.3% | 1 |
| cell-substrate junction | 15 out of 5696 genes, 0.3% | 15 out of 5777 genes, 0.3% | 1 |
| cortical cytoskeleton | 15 out of 5696 genes, 0.3% | 15 out of 5777 genes, 0.3% | 1 |
| cell projection membrane | 15 out of 5696 genes, 0.3% | 15 out of 5777 genes, 0.3% | 1 |
| ion channel complex | 15 out of 5696 genes, 0.3% | 15 out of 5777 genes, 0.3% | 1 |
| cation channel complex | 15 out of 5696 genes, 0.3% | 15 out of 5777 genes, 0.3% | 1 |
| respiratory chain | 15 out of 5696 genes, 0.3% | 15 out of 5777 genes, 0.3% | 1 |
| sarcomere | 56 out of 5696 genes, 1.0% | 57 out of 5777 genes, 1.0% | 1 |
| mitochondrial respiratory chain | 14 out of 5696 genes, 0.2% | 14 out of 5777 genes, 0.2% | 1 |
| proteasome core complex | 14 out of 5696 genes, 0.2% | 14 out of 5777 genes, 0.2% | 1 |
| synaptic vesicle | 14 out of 5696 genes, 0.2% | 14 out of 5777 genes, 0.2% | 1 |
| flagellum | 14 out of 5696 genes, 0.2% | 14 out of 5777 genes, 0.2% | 1 |
| intrinsic to endoplasmic reticulum membrane | 14 out of 5696 genes, 0.2% | 14 out of 5777 genes, 0.2% | 1 |
| chromatin | 53 out of 5696 genes, 0.9% | 54 out of 5777 genes, 0.9% | 1 |
| proteasome complex | 52 out of 5696 genes, 0.9% | 53 out of 5777 genes, 0.9% | 1 |
| lysosome | 13 out of 5696 genes, 0.2% | 13 out of 5777 genes, 0.2% | 1 |
| vacuolar membrane | 13 out of 5696 genes, 0.2% | 13 out of 5777 genes, 0.2% | 1 |
| proteasome regulatory particle | 13 out of 5696 genes, 0.2% | 13 out of 5777 genes, 0.2% | 1 |
| transcription elongation factor complex | 13 out of 5696 genes, 0.2% | 13 out of 5777 genes, 0.2% | 1 |
| trans-Golgi network transport vesicle membrane | 13 out of 5696 genes, 0.2% | 13 out of 5777 genes, 0.2% | 1 |
| endocytic vesicle | 13 out of 5696 genes, 0.2% | 13 out of 5777 genes, 0.2% | 1 |
| trans-Golgi network transport vesicle | 13 out of 5696 genes, 0.2% | 13 out of 5777 genes, 0.2% | 1 |
| Golgi-associated vesicle membrane | 13 out of 5696 genes, 0.2% | 13 out of 5777 genes, 0.2% | 1 |
| cortical actin cytoskeleton | 13 out of 5696 genes, 0.2% | 13 out of 5777 genes, 0.2% | 1 |
| proton-transporting V-type ATPase complex | 13 out of 5696 genes, 0.2% | 13 out of 5777 genes, 0.2% | 1 |
| H4/H2A histone acetyltransferase complex | 13 out of 5696 genes, 0.2% | 13 out of 5777 genes, 0.2% | 1 |
| vacuolar part | 13 out of 5696 genes, 0.2% | 13 out of 5777 genes, 0.2% | 1 |
| coated pit | 12 out of 5696 genes, 0.2% | 12 out of 5777 genes, 0.2% | 1 |
| apicolateral plasma membrane | 12 out of 5696 genes, 0.2% | 12 out of 5777 genes, 0.2% | 1 |
| intercellular bridge | 12 out of 5696 genes, 0.2% | 12 out of 5777 genes, 0.2% | 1 |
| chromosome, telomeric region | 11 out of 5696 genes, 0.2% | 11 out of 5777 genes, 0.2% | 1 |
| polytene chromosome | 11 out of 5696 genes, 0.2% | 11 out of 5777 genes, 0.2% | 1 |
| PcG protein complex | 11 out of 5696 genes, 0.2% | 11 out of 5777 genes, 0.2% | 1 |
| cell division site | 11 out of 5696 genes, 0.2% | 11 out of 5777 genes, 0.2% | 1 |
| cell division site part | 11 out of 5696 genes, 0.2% | 11 out of 5777 genes, 0.2% | 1 |
| calcium channel complex | 11 out of 5696 genes, 0.2% | 11 out of 5777 genes, 0.2% | 1 |
| germ cell nucleus | 11 out of 5696 genes, 0.2% | 11 out of 5777 genes, 0.2% | 1 |
| receptor complex | 11 out of 5696 genes, 0.2% | 11 out of 5777 genes, 0.2% | 1 |
| SWI/SNF-type complex | 11 out of 5696 genes, 0.2% | 11 out of 5777 genes, 0.2% | 1 |
| vesicular fraction | 46 out of 5696 genes, 0.8% | 47 out of 5777 genes, 0.8% | 1 |
| neuronal cell body | 10 out of 5696 genes, 0.2% | 10 out of 5777 genes, 0.2% | 1 |
| apical junction complex | 10 out of 5696 genes, 0.2% | 10 out of 5777 genes, 0.2% | 1 |
| extracellular matrix part | 10 out of 5696 genes, 0.2% | 10 out of 5777 genes, 0.2% | 1 |
| organellar ribosome | 9 out of 5696 genes, 0.2% | 9 out of 5777 genes, 0.2% | 1 |
| microtubule | 9 out of 5696 genes, 0.2% | 9 out of 5777 genes, 0.2% | 1 |
| clathrin coat | 9 out of 5696 genes, 0.2% | 9 out of 5777 genes, 0.2% | 1 |
| female germline ring canal | 9 out of 5696 genes, 0.2% | 9 out of 5777 genes, 0.2% | 1 |
| microbody | 9 out of 5696 genes, 0.2% | 9 out of 5777 genes, 0.2% | 1 |
| germline ring canal | 9 out of 5696 genes, 0.2% | 9 out of 5777 genes, 0.2% | 1 |
| ubiquitin ligase complex | 41 out of 5696 genes, 0.7% | 42 out of 5777 genes, 0.7% | 1 |
| integral to plasma membrane | 39 out of 5696 genes, 0.7% | 40 out of 5777 genes, 0.7% | 1 |
| intrinsic to plasma membrane | 39 out of 5696 genes, 0.7% | 40 out of 5777 genes, 0.7% | 1 |
| heterochromatin | 8 out of 5696 genes, 0.1% | 8 out of 5777 genes, 0.1% | 1 |
| sex chromosome | 8 out of 5696 genes, 0.1% | 8 out of 5777 genes, 0.1% | 1 |
| nucleolus | 8 out of 5696 genes, 0.1% | 8 out of 5777 genes, 0.1% | 1 |
| plastid | 8 out of 5696 genes, 0.1% | 8 out of 5777 genes, 0.1% | 1 |
| clathrin vesicle coat | 8 out of 5696 genes, 0.1% | 8 out of 5777 genes, 0.1% | 1 |
| nucleolar part | 8 out of 5696 genes, 0.1% | 8 out of 5777 genes, 0.1% | 1 |
| membrane | 1702 out of 5696 genes, 29.9% | 1731 out of 5777 genes, 30.0% | 1 |
| ribosome | 37 out of 5696 genes, 0.6% | 38 out of 5777 genes, 0.7% | 1 |
| organellar small ribosomal subunit | 7 out of 5696 genes, 0.1% | 7 out of 5777 genes, 0.1% | 1 |
| spindle pole | 7 out of 5696 genes, 0.1% | 7 out of 5777 genes, 0.1% | 1 |
| replication fork | 7 out of 5696 genes, 0.1% | 7 out of 5777 genes, 0.1% | 1 |
| late endosome | 7 out of 5696 genes, 0.1% | 7 out of 5777 genes, 0.1% | 1 |
| cell-cell adherens junction | 7 out of 5696 genes, 0.1% | 7 out of 5777 genes, 0.1% | 1 |
| cell surface | 7 out of 5696 genes, 0.1% | 7 out of 5777 genes, 0.1% | 1 |
| RNAi effector complex | 7 out of 5696 genes, 0.1% | 7 out of 5777 genes, 0.1% | 1 |
| actin filament bundle | 7 out of 5696 genes, 0.1% | 7 out of 5777 genes, 0.1% | 1 |
| proton-transporting two-sector ATPase complex, catalytic domain | 7 out of 5696 genes, 0.1% | 7 out of 5777 genes, 0.1% | 1 |
| signal recognition particle | 7 out of 5696 genes, 0.1% | 7 out of 5777 genes, 0.1% | 1 |
| SAGA-type complex | 7 out of 5696 genes, 0.1% | 7 out of 5777 genes, 0.1% | 1 |
| ribonucleoprotein complex | 265 out of 5696 genes, 4.7% | 271 out of 5777 genes, 4.7% | 1 |
| histone deacetylase complex | 6 out of 5696 genes, 0.1% | 6 out of 5777 genes, 0.1% | 1 |
| nuclear ubiquitin ligase complex | 6 out of 5696 genes, 0.1% | 6 out of 5777 genes, 0.1% | 1 |
| transcription export complex | 6 out of 5696 genes, 0.1% | 6 out of 5777 genes, 0.1% | 1 |
| condensed chromosome kinetochore | 6 out of 5696 genes, 0.1% | 6 out of 5777 genes, 0.1% | 1 |
| condensed chromosome, centromeric region | 6 out of 5696 genes, 0.1% | 6 out of 5777 genes, 0.1% | 1 |
| synaptonemal complex | 6 out of 5696 genes, 0.1% | 6 out of 5777 genes, 0.1% | 1 |
| sex chromatin | 6 out of 5696 genes, 0.1% | 6 out of 5777 genes, 0.1% | 1 |
| basement membrane | 6 out of 5696 genes, 0.1% | 6 out of 5777 genes, 0.1% | 1 |
| nuclear heterochromatin | 6 out of 5696 genes, 0.1% | 6 out of 5777 genes, 0.1% | 1 |
| thylakoid | 6 out of 5696 genes, 0.1% | 6 out of 5777 genes, 0.1% | 1 |
| ER to Golgi transport vesicle membrane | 6 out of 5696 genes, 0.1% | 6 out of 5777 genes, 0.1% | 1 |
| large ribosomal subunit | 6 out of 5696 genes, 0.1% | 6 out of 5777 genes, 0.1% | 1 |
| nuclear membrane | 6 out of 5696 genes, 0.1% | 6 out of 5777 genes, 0.1% | 1 |
| mismatch repair complex | 6 out of 5696 genes, 0.1% | 6 out of 5777 genes, 0.1% | 1 |
| exosome (RNase complex) | 5 out of 5696 genes, 0.1% | 5 out of 5777 genes, 0.1% | 1 |
| mitochondrial outer membrane | 5 out of 5696 genes, 0.1% | 5 out of 5777 genes, 0.1% | 1 |
| rough endoplasmic reticulum | 5 out of 5696 genes, 0.1% | 5 out of 5777 genes, 0.1% | 1 |
| actin filament | 5 out of 5696 genes, 0.1% | 5 out of 5777 genes, 0.1% | 1 |
| caveola | 5 out of 5696 genes, 0.1% | 5 out of 5777 genes, 0.1% | 1 |
| septate junction | 5 out of 5696 genes, 0.1% | 5 out of 5777 genes, 0.1% | 1 |
| ionotropic glutamate receptor complex | 5 out of 5696 genes, 0.1% | 5 out of 5777 genes, 0.1% | 1 |
| vacuolar proton-transporting V-type ATPase complex | 5 out of 5696 genes, 0.1% | 5 out of 5777 genes, 0.1% | 1 |
| outer membrane | 5 out of 5696 genes, 0.1% | 5 out of 5777 genes, 0.1% | 1 |
| clathrin coat of endocytic vesicle | 5 out of 5696 genes, 0.1% | 5 out of 5777 genes, 0.1% | 1 |
| endocytic vesicle membrane | 5 out of 5696 genes, 0.1% | 5 out of 5777 genes, 0.1% | 1 |
| clathrin-coated endocytic vesicle membrane | 5 out of 5696 genes, 0.1% | 5 out of 5777 genes, 0.1% | 1 |
| rough endoplasmic reticulum membrane | 5 out of 5696 genes, 0.1% | 5 out of 5777 genes, 0.1% | 1 |
| leading edge membrane | 5 out of 5696 genes, 0.1% | 5 out of 5777 genes, 0.1% | 1 |
| organelle outer membrane | 5 out of 5696 genes, 0.1% | 5 out of 5777 genes, 0.1% | 1 |
| organelle subcompartment | 5 out of 5696 genes, 0.1% | 5 out of 5777 genes, 0.1% | 1 |
| septin cytoskeleton | 5 out of 5696 genes, 0.1% | 5 out of 5777 genes, 0.1% | 1 |
| neuron projection membrane | 5 out of 5696 genes, 0.1% | 5 out of 5777 genes, 0.1% | 1 |
| protein-DNA complex | 5 out of 5696 genes, 0.1% | 5 out of 5777 genes, 0.1% | 1 |
| membrane raft | 5 out of 5696 genes, 0.1% | 5 out of 5777 genes, 0.1% | 1 |
| clathrin-coated endocytic vesicle | 5 out of 5696 genes, 0.1% | 5 out of 5777 genes, 0.1% | 1 |
| occluding junction | 5 out of 5696 genes, 0.1% | 5 out of 5777 genes, 0.1% | 1 |
| membrane part | 936 out of 5696 genes, 16.4% | 954 out of 5777 genes, 16.5% | 1 |
| mitochondrion | 151 out of 5696 genes, 2.7% | 155 out of 5777 genes, 2.7% | 1 |
| ribosomal subunit | 28 out of 5696 genes, 0.5% | 29 out of 5777 genes, 0.5% | 1 |
| peroxisome | 4 out of 5696 genes, 0.1% | 4 out of 5777 genes, 0.1% | 1 |
| aster | 4 out of 5696 genes, 0.1% | 4 out of 5777 genes, 0.1% | 1 |
| mRNA cleavage factor complex | 4 out of 5696 genes, 0.1% | 4 out of 5777 genes, 0.1% | 1 |
| kinesin complex | 4 out of 5696 genes, 0.1% | 4 out of 5777 genes, 0.1% | 1 |
| chloroplast | 4 out of 5696 genes, 0.1% | 4 out of 5777 genes, 0.1% | 1 |
| chloroplast thylakoid | 4 out of 5696 genes, 0.1% | 4 out of 5777 genes, 0.1% | 1 |
| endosome membrane | 4 out of 5696 genes, 0.1% | 4 out of 5777 genes, 0.1% | 1 |
| inclusion body | 4 out of 5696 genes, 0.1% | 4 out of 5777 genes, 0.1% | 1 |
| preribosome | 4 out of 5696 genes, 0.1% | 4 out of 5777 genes, 0.1% | 1 |
| plastid thylakoid | 4 out of 5696 genes, 0.1% | 4 out of 5777 genes, 0.1% | 1 |
| proton-transporting two-sector ATPase complex, proton-transporting domain | 4 out of 5696 genes, 0.1% | 4 out of 5777 genes, 0.1% | 1 |
| photosynthetic membrane | 4 out of 5696 genes, 0.1% | 4 out of 5777 genes, 0.1% | 1 |
| sarcolemma | 4 out of 5696 genes, 0.1% | 4 out of 5777 genes, 0.1% | 1 |
| chloroplast part | 4 out of 5696 genes, 0.1% | 4 out of 5777 genes, 0.1% | 1 |
| plastid part | 4 out of 5696 genes, 0.1% | 4 out of 5777 genes, 0.1% | 1 |
| thylakoid part | 4 out of 5696 genes, 0.1% | 4 out of 5777 genes, 0.1% | 1 |
| endosomal part | 4 out of 5696 genes, 0.1% | 4 out of 5777 genes, 0.1% | 1 |
| Sin3-type complex | 4 out of 5696 genes, 0.1% | 4 out of 5777 genes, 0.1% | 1 |
| cullin-RING ubiquitin ligase complex | 26 out of 5696 genes, 0.5% | 27 out of 5777 genes, 0.5% | 1 |
| dynein complex | 59 out of 5696 genes, 1.0% | 61 out of 5777 genes, 1.1% | 1 |
| insoluble fraction | 186 out of 5696 genes, 3.3% | 191 out of 5777 genes, 3.3% | 1 |
| transport vesicle | 24 out of 5696 genes, 0.4% | 25 out of 5777 genes, 0.4% | 1 |
| condensed nuclear chromosome kinetochore | 3 out of 5696 genes, 0.1% | 3 out of 5777 genes, 0.1% | 1 |
| condensed nuclear chromosome, centromeric region | 3 out of 5696 genes, 0.1% | 3 out of 5777 genes, 0.1% | 1 |
| nuclear pore | 3 out of 5696 genes, 0.1% | 3 out of 5777 genes, 0.1% | 1 |
| chromatin assembly complex | 3 out of 5696 genes, 0.1% | 3 out of 5777 genes, 0.1% | 1 |
| early endosome | 3 out of 5696 genes, 0.1% | 3 out of 5777 genes, 0.1% | 1 |
| Golgi stack | 3 out of 5696 genes, 0.1% | 3 out of 5777 genes, 0.1% | 1 |
| cis-Golgi network | 3 out of 5696 genes, 0.1% | 3 out of 5777 genes, 0.1% | 1 |
| intermediate filament | 3 out of 5696 genes, 0.1% | 3 out of 5777 genes, 0.1% | 1 |
| microvillus | 3 out of 5696 genes, 0.1% | 3 out of 5777 genes, 0.1% | 1 |
| photosystem | 3 out of 5696 genes, 0.1% | 3 out of 5777 genes, 0.1% | 1 |
| clathrin coat of trans-Golgi network vesicle | 3 out of 5696 genes, 0.1% | 3 out of 5777 genes, 0.1% | 1 |
| integral to endoplasmic reticulum membrane | 3 out of 5696 genes, 0.1% | 3 out of 5777 genes, 0.1% | 1 |
| ISWI complex | 3 out of 5696 genes, 0.1% | 3 out of 5777 genes, 0.1% | 1 |
| intrinsic to Golgi membrane | 3 out of 5696 genes, 0.1% | 3 out of 5777 genes, 0.1% | 1 |
| integral to organelle membrane | 3 out of 5696 genes, 0.1% | 3 out of 5777 genes, 0.1% | 1 |
| intrinsic to mitochondrial outer membrane | 3 out of 5696 genes, 0.1% | 3 out of 5777 genes, 0.1% | 1 |
| microbody part | 3 out of 5696 genes, 0.1% | 3 out of 5777 genes, 0.1% | 1 |
| peroxisomal part | 3 out of 5696 genes, 0.1% | 3 out of 5777 genes, 0.1% | 1 |
| intermediate filament cytoskeleton | 3 out of 5696 genes, 0.1% | 3 out of 5777 genes, 0.1% | 1 |
| pore complex | 3 out of 5696 genes, 0.1% | 3 out of 5777 genes, 0.1% | 1 |
| H3 histone acetyltransferase complex | 3 out of 5696 genes, 0.1% | 3 out of 5777 genes, 0.1% | 1 |
| CUL4 RING ubiquitin ligase complex | 3 out of 5696 genes, 0.1% | 3 out of 5777 genes, 0.1% | 1 |
| mitochondrial membrane | 53 out of 5696 genes, 0.9% | 55 out of 5777 genes, 1.0% | 1 |
| site of polarized growth | 20 out of 5696 genes, 0.4% | 21 out of 5777 genes, 0.4% | 1 |
| mitochondrial part | 119 out of 5696 genes, 2.1% | 123 out of 5777 genes, 2.1% | 1 |
| nucleotide-excision repair complex | 2 out of 5696 genes, 0.0% | 2 out of 5777 genes, 0.0% | 1 |
| cytoplasmic ubiquitin ligase complex | 2 out of 5696 genes, 0.0% | 2 out of 5777 genes, 0.0% | 1 |
| cyclin-dependent protein kinase holoenzyme complex | 2 out of 5696 genes, 0.0% | 2 out of 5777 genes, 0.0% | 1 |
| organellar large ribosomal subunit | 2 out of 5696 genes, 0.0% | 2 out of 5777 genes, 0.0% | 1 |
| collagen | 2 out of 5696 genes, 0.0% | 2 out of 5777 genes, 0.0% | 1 |
| basal lamina | 2 out of 5696 genes, 0.0% | 2 out of 5777 genes, 0.0% | 1 |
| mitochondrial intermembrane space | 2 out of 5696 genes, 0.0% | 2 out of 5777 genes, 0.0% | 1 |
| endoplasmic reticulum lumen | 2 out of 5696 genes, 0.0% | 2 out of 5777 genes, 0.0% | 1 |
| ER-Golgi intermediate compartment | 2 out of 5696 genes, 0.0% | 2 out of 5777 genes, 0.0% | 1 |
| internal side of plasma membrane | 2 out of 5696 genes, 0.0% | 2 out of 5777 genes, 0.0% | 1 |
| rhabdomere | 2 out of 5696 genes, 0.0% | 2 out of 5777 genes, 0.0% | 1 |
| unconventional myosin complex | 2 out of 5696 genes, 0.0% | 2 out of 5777 genes, 0.0% | 1 |
| sarcoplasm | 2 out of 5696 genes, 0.0% | 2 out of 5777 genes, 0.0% | 1 |
| sarcoplasmic reticulum | 2 out of 5696 genes, 0.0% | 2 out of 5777 genes, 0.0% | 1 |
| mitochondrial tricarboxylic acid cycle enzyme complex | 2 out of 5696 genes, 0.0% | 2 out of 5777 genes, 0.0% | 1 |
| COPI-coated vesicle | 2 out of 5696 genes, 0.0% | 2 out of 5777 genes, 0.0% | 1 |
| palmitoyltransferase complex | 2 out of 5696 genes, 0.0% | 2 out of 5777 genes, 0.0% | 1 |
| trailing edge | 2 out of 5696 genes, 0.0% | 2 out of 5777 genes, 0.0% | 1 |
| organelle envelope lumen | 2 out of 5696 genes, 0.0% | 2 out of 5777 genes, 0.0% | 1 |
| RNA cap binding complex | 2 out of 5696 genes, 0.0% | 2 out of 5777 genes, 0.0% | 1 |
| DNA polymerase complex | 2 out of 5696 genes, 0.0% | 2 out of 5777 genes, 0.0% | 1 |
| plasma membrane-derived chromatophore | 2 out of 5696 genes, 0.0% | 2 out of 5777 genes, 0.0% | 1 |
| presynaptic membrane | 2 out of 5696 genes, 0.0% | 2 out of 5777 genes, 0.0% | 1 |
| laminin complex | 2 out of 5696 genes, 0.0% | 2 out of 5777 genes, 0.0% | 1 |
| microtubule organizing center part | 2 out of 5696 genes, 0.0% | 2 out of 5777 genes, 0.0% | 1 |
| tricarboxylic acid cycle enzyme complex | 2 out of 5696 genes, 0.0% | 2 out of 5777 genes, 0.0% | 1 |
| translocon complex | 2 out of 5696 genes, 0.0% | 2 out of 5777 genes, 0.0% | 1 |
| stored secretory granule | 17 out of 5696 genes, 0.3% | 18 out of 5777 genes, 0.3% | 1 |
| small ribosomal subunit | 16 out of 5696 genes, 0.3% | 17 out of 5777 genes, 0.3% | 1 |
| mitochondrial inner membrane | 36 out of 5696 genes, 0.6% | 38 out of 5777 genes, 0.7% | 1 |
| proton-transporting two-sector ATPase complex | 36 out of 5696 genes, 0.6% | 38 out of 5777 genes, 0.7% | 1 |
| organelle inner membrane | 125 out of 5696 genes, 2.2% | 130 out of 5777 genes, 2.3% | 1 |
| organelle envelope | 198 out of 5696 genes, 3.5% | 205 out of 5777 genes, 3.5% | 1 |
| intrinsic to membrane | 520 out of 5696 genes, 9.1% | 534 out of 5777 genes, 9.2% | 1 |
| myosin complex | 8 out of 5696 genes, 0.1% | 9 out of 5777 genes, 0.2% | 1 |
| ER to Golgi transport vesicle | 7 out of 5696 genes, 0.1% | 8 out of 5777 genes, 0.1% | 1 |
| mitochondrial membrane part | 21 out of 5696 genes, 0.4% | 23 out of 5777 genes, 0.4% | 1 |
| mitochondrial envelope | 68 out of 5696 genes, 1.2% | 72 out of 5777 genes, 1.2% | 1 |
| cell envelope | 5 out of 5696 genes, 0.1% | 6 out of 5777 genes, 0.1% | 1 |
| envelope | 203 out of 5696 genes, 3.6% | 211 out of 5777 genes, 3.7% | 1 |
| mitochondrial proton-transporting ATP synthase complex | 4 out of 5696 genes, 0.1% | 6 out of 5777 genes, 0.1% | 1 |
| proton-transporting ATP synthase complex | 4 out of 5696 genes, 0.1% | 6 out of 5777 genes, 0.1% | 1 |

| Gene Ontology term | Genes annotated to the term |
| --- | --- |
| nuclear part | Unigene19912\_Sample\_011046841, Unigene7801\_Sample\_011046841, Unigene713\_Sample\_011046841, Unigene40921\_Sample\_011046841, Unigene34629\_Sample\_011046841, Unigene55221\_Sample\_011046841, Unigene57770\_Sample\_011046841, Unigene56530\_Sample\_011046841, Unigene4096\_Sample\_011046841, Unigene15270\_Sample\_011046841, Unigene52866\_Sample\_011046841, Unigene59633\_Sample\_011046841, Unigene57534\_Sample\_011046841, Unigene48415\_Sample\_011046841, Unigene9459\_Sample\_011046841, Unigene55254\_Sample\_011046841, Unigene2263\_Sample\_011046841, Unigene41700\_Sample\_011046841, Unigene33973\_Sample\_011046841, Unigene54454\_Sample\_011046841, Unigene9589\_Sample\_011046841, Unigene59972\_Sample\_011046841, Unigene46719\_Sample\_011046841, Unigene29658\_Sample\_011046841, Unigene48260\_Sample\_011046841, Unigene26047\_Sample\_011046841, Unigene28131\_Sample\_011046841, Unigene29839\_Sample\_011046841, Unigene60645\_Sample\_011046841, Unigene12172\_Sample\_011046841, Unigene60161\_Sample\_011046841, Unigene50631\_Sample\_011046841, Unigene8519\_Sample\_011046841, Unigene55156\_Sample\_011046841, Unigene57083\_Sample\_011046841, Unigene53650\_Sample\_011046841, Unigene60377\_Sample\_011046841, Unigene44447\_Sample\_011046841, Unigene31264\_Sample\_011046841, Unigene50051\_Sample\_011046841, Unigene4709\_Sample\_011046841, Unigene42827\_Sample\_011046841, Unigene59022\_Sample\_011046841, Unigene59195\_Sample\_011046841, Unigene60842\_Sample\_011046841, Unigene36851\_Sample\_011046841, Unigene9307\_Sample\_011046841, Unigene12309\_Sample\_011046841, Unigene39807\_Sample\_011046841, Unigene2913\_Sample\_011046841, Unigene46283\_Sample\_011046841, Unigene41574\_Sample\_011046841, Unigene27013\_Sample\_011046841, Unigene54061\_Sample\_011046841, Unigene31679\_Sample\_011046841, Unigene51504\_Sample\_011046841, Unigene59077\_Sample\_011046841, Unigene21535\_Sample\_011046841, Unigene45512\_Sample\_011046841, Unigene58715\_Sample\_011046841, Unigene13436\_Sample\_011046841, Unigene44587\_Sample\_011046841, Unigene23762\_Sample\_011046841, Unigene9912\_Sample\_011046841, Unigene29524\_Sample\_011046841, Unigene2398\_Sample\_011046841, Unigene23742\_Sample\_011046841, Unigene50506\_Sample\_011046841, Unigene12623\_Sample\_011046841, Unigene57828\_Sample\_011046841, Unigene55509\_Sample\_011046841, Unigene813\_Sample\_011046841, Unigene1129\_Sample\_011046841, Unigene55036\_Sample\_011046841, Unigene13748\_Sample\_011046841, Unigene10962\_Sample\_011046841, Unigene49168\_Sample\_011046841, Unigene13391\_Sample\_011046841, Unigene42378\_Sample\_011046841, Unigene54996\_Sample\_011046841, Unigene25267\_Sample\_011046841, Unigene55449\_Sample\_011046841, Unigene60561\_Sample\_011046841, Unigene24493\_Sample\_011046841, Unigene60944\_Sample\_011046841, Unigene47868\_Sample\_011046841, Unigene58386\_Sample\_011046841, Unigene44838\_Sample\_011046841, Unigene12390\_Sample\_011046841, Unigene49895\_Sample\_011046841, Unigene7366\_Sample\_011046841, Unigene19789\_Sample\_011046841, Unigene59985\_Sample\_011046841, Unigene41608\_Sample\_011046841, Unigene16763\_Sample\_011046841, Unigene18426\_Sample\_011046841, Unigene58496\_Sample\_011046841, Unigene57070\_Sample\_011046841, Unigene7778\_Sample\_011046841, Unigene57438\_Sample\_011046841, Unigene57453\_Sample\_011046841, Unigene44362\_Sample\_011046841, Unigene49099\_Sample\_011046841, Unigene9623\_Sample\_011046841, Unigene42229\_Sample\_011046841, Unigene32488\_Sample\_011046841, Unigene48049\_Sample\_011046841, Unigene32138\_Sample\_011046841, Unigene13325\_Sample\_011046841, Unigene55183\_Sample\_011046841, Unigene4847\_Sample\_011046841, Unigene57238\_Sample\_011046841, Unigene44749\_Sample\_011046841, Unigene45403\_Sample\_011046841, Unigene34659\_Sample\_011046841, Unigene58777\_Sample\_011046841, Unigene38021\_Sample\_011046841, Unigene8925\_Sample\_011046841, Unigene32309\_Sample\_011046841, Unigene37820\_Sample\_011046841, Unigene31420\_Sample\_011046841, Unigene21520\_Sample\_011046841, Unigene58502\_Sample\_011046841, Unigene16650\_Sample\_011046841, Unigene45620\_Sample\_011046841, Unigene54248\_Sample\_011046841, Unigene7092\_Sample\_011046841, Unigene59136\_Sample\_011046841, Unigene41959\_Sample\_011046841, Unigene50990\_Sample\_011046841, Unigene52322\_Sample\_011046841, Unigene57384\_Sample\_011046841, Unigene50223\_Sample\_011046841, Unigene56688\_Sample\_011046841, Unigene50164\_Sample\_011046841, Unigene50912\_Sample\_011046841, Unigene15865\_Sample\_011046841, Unigene54555\_Sample\_011046841, Unigene57062\_Sample\_011046841, Unigene46262\_Sample\_011046841, Unigene6052\_Sample\_011046841, Unigene18725\_Sample\_011046841, Unigene58480\_Sample\_011046841, Unigene50798\_Sample\_011046841, Unigene59512\_Sample\_011046841, Unigene11040\_Sample\_011046841, Unigene58403\_Sample\_011046841, Unigene53098\_Sample\_011046841, Unigene55512\_Sample\_011046841, Unigene54406\_Sample\_011046841, Unigene54852\_Sample\_011046841, Unigene58374\_Sample\_011046841, Unigene54790\_Sample\_011046841, Unigene38586\_Sample\_011046841, Unigene41393\_Sample\_011046841, Unigene22323\_Sample\_011046841, Unigene48764\_Sample\_011046841, Unigene29101\_Sample\_011046841, Unigene51803\_Sample\_011046841, Unigene44009\_Sample\_011046841, Unigene51264\_Sample\_011046841, Unigene46791\_Sample\_011046841, Unigene49268\_Sample\_011046841, Unigene8538\_Sample\_011046841, Unigene57100\_Sample\_011046841, Unigene48637\_Sample\_011046841, Unigene57158\_Sample\_011046841, Unigene56919\_Sample\_011046841, Unigene45539\_Sample\_011046841, Unigene28246\_Sample\_011046841, Unigene21907\_Sample\_011046841, Unigene60809\_Sample\_011046841, Unigene8165\_Sample\_011046841, Unigene11180\_Sample\_011046841, Unigene60701\_Sample\_011046841, Unigene60494\_Sample\_011046841, Unigene30162\_Sample\_011046841, Unigene35453\_Sample\_011046841, Unigene59679\_Sample\_011046841, Unigene33130\_Sample\_011046841, Unigene8392\_Sample\_011046841, Unigene50396\_Sample\_011046841, Unigene53015\_Sample\_011046841, Unigene58138\_Sample\_011046841, Unigene20591\_Sample\_011046841, Unigene36469\_Sample\_011046841, Unigene17370\_Sample\_011046841, Unigene30385\_Sample\_011046841, Unigene12748\_Sample\_011046841, Unigene4871\_Sample\_011046841, Unigene13249\_Sample\_011046841, Unigene43875\_Sample\_011046841, Unigene48708\_Sample\_011046841, Unigene60309\_Sample\_011046841, Unigene10640\_Sample\_011046841, Unigene53714\_Sample\_011046841, Unigene43848\_Sample\_011046841, Unigene21655\_Sample\_011046841, Unigene53757\_Sample\_011046841, Unigene13517\_Sample\_011046841, Unigene40807\_Sample\_011046841, Unigene13258\_Sample\_011046841, Unigene58839\_Sample\_011046841, Unigene50653\_Sample\_011046841, Unigene60381\_Sample\_011046841, Unigene60900\_Sample\_011046841, Unigene51569\_Sample\_011046841, Unigene12056\_Sample\_011046841, Unigene59535\_Sample\_011046841, Unigene58929\_Sample\_011046841, Unigene41822\_Sample\_011046841, Unigene49671\_Sample\_011046841, Unigene23667\_Sample\_011046841, Unigene53072\_Sample\_011046841, Unigene55175\_Sample\_011046841, Unigene59447\_Sample\_011046841, Unigene40147\_Sample\_011046841, Unigene50313\_Sample\_011046841, Unigene8243\_Sample\_011046841, Unigene1952\_Sample\_011046841, Unigene56358\_Sample\_011046841, Unigene13719\_Sample\_011046841, Unigene48020\_Sample\_011046841, Unigene40258\_Sample\_011046841, Unigene40558\_Sample\_011046841, Unigene9219\_Sample\_011046841, Unigene52688\_Sample\_011046841, Unigene41676\_Sample\_011046841, Unigene3486\_Sample\_011046841, Unigene9304\_Sample\_011046841, Unigene25573\_Sample\_011046841, Unigene21688\_Sample\_011046841, Unigene54402\_Sample\_011046841, Unigene56881\_Sample\_011046841, Unigene57132\_Sample\_011046841, Unigene60919\_Sample\_011046841, Unigene47368\_Sample\_011046841, Unigene14355\_Sample\_011046841, Unigene59891\_Sample\_011046841, Unigene53843\_Sample\_011046841, Unigene4523\_Sample\_011046841, Unigene22255\_Sample\_011046841, Unigene46321\_Sample\_011046841, Unigene10941\_Sample\_011046841, Unigene53037\_Sample\_011046841, Unigene10930\_Sample\_011046841, Unigene51110\_Sample\_011046841, Unigene38373\_Sample\_011046841, Unigene55196\_Sample\_011046841, Unigene44438\_Sample\_011046841, Unigene39200\_Sample\_011046841, Unigene57239\_Sample\_011046841, Unigene57610\_Sample\_011046841, Unigene48086\_Sample\_011046841, Unigene59492\_Sample\_011046841, Unigene47981\_Sample\_011046841, Unigene44734\_Sample\_011046841, Unigene60131\_Sample\_011046841, Unigene49633\_Sample\_011046841, Unigene45873\_Sample\_011046841, Unigene42899\_Sample\_011046841, Unigene2532\_Sample\_011046841, Unigene60714\_Sample\_011046841, Unigene22216\_Sample\_011046841, Unigene2325\_Sample\_011046841, Unigene57531\_Sample\_011046841, Unigene8480\_Sample\_011046841, Unigene30681\_Sample\_011046841, Unigene11939\_Sample\_011046841, Unigene9067\_Sample\_011046841, Unigene3946\_Sample\_011046841, Unigene53420\_Sample\_011046841, Unigene37278\_Sample\_011046841, Unigene12562\_Sample\_011046841, Unigene36721\_Sample\_011046841, Unigene58365\_Sample\_011046841, Unigene11053\_Sample\_011046841, Unigene57806\_Sample\_011046841, Unigene36824\_Sample\_011046841, Unigene58725\_Sample\_011046841, Unigene39209\_Sample\_011046841, Unigene12670\_Sample\_011046841, Unigene11846\_Sample\_011046841, Unigene12324\_Sample\_011046841, Unigene59653\_Sample\_011046841, Unigene59637\_Sample\_011046841, Unigene21163\_Sample\_011046841, Unigene2861\_Sample\_011046841, Unigene59587\_Sample\_011046841, Unigene31705\_Sample\_011046841, Unigene26904\_Sample\_011046841, Unigene28403\_Sample\_011046841, Unigene50902\_Sample\_011046841, Unigene55769\_Sample\_011046841, Unigene55247\_Sample\_011046841, Unigene33941\_Sample\_011046841, Unigene30285\_Sample\_011046841, Unigene24860\_Sample\_011046841, Unigene54870\_Sample\_011046841, Unigene50103\_Sample\_011046841, Unigene47555\_Sample\_011046841, Unigene58564\_Sample\_011046841, Unigene42735\_Sample\_011046841, Unigene46263\_Sample\_011046841, Unigene42749\_Sample\_011046841, Unigene46290\_Sample\_011046841, Unigene47871\_Sample\_011046841, Unigene30649\_Sample\_011046841, Unigene13390\_Sample\_011046841, Unigene53095\_Sample\_011046841, Unigene36452\_Sample\_011046841, Unigene20596\_Sample\_011046841, Unigene55077\_Sample\_011046841, Unigene60217\_Sample\_011046841, Unigene13694\_Sample\_011046841, Unigene5335\_Sample\_011046841, Unigene59560\_Sample\_011046841, Unigene641\_Sample\_011046841, Unigene11804\_Sample\_011046841, Unigene57614\_Sample\_011046841, Unigene39261\_Sample\_011046841, Unigene57972\_Sample\_011046841, Unigene29630\_Sample\_011046841, Unigene757\_Sample\_011046841, Unigene54668\_Sample\_011046841, Unigene38304\_Sample\_011046841, Unigene52375\_Sample\_011046841, Unigene34268\_Sample\_011046841, Unigene13245\_Sample\_011046841, Unigene39074\_Sample\_011046841, Unigene39621\_Sample\_011046841, Unigene58663\_Sample\_011046841, Unigene50213\_Sample\_011046841, Unigene27324\_Sample\_011046841, Unigene53179\_Sample\_011046841, Unigene13584\_Sample\_011046841, Unigene11431\_Sample\_011046841, Unigene58659\_Sample\_011046841, Unigene32707\_Sample\_011046841, Unigene58488\_Sample\_011046841, Unigene56395\_Sample\_011046841, Unigene4684\_Sample\_011046841, Unigene50764\_Sample\_011046841, Unigene12023\_Sample\_011046841, Unigene46030\_Sample\_011046841, Unigene58513\_Sample\_011046841, Unigene58135\_Sample\_011046841, Unigene51481\_Sample\_011046841, Unigene46220\_Sample\_011046841, Unigene51688\_Sample\_011046841, Unigene2068\_Sample\_011046841, Unigene59973\_Sample\_011046841, Unigene50734\_Sample\_011046841, Unigene55325\_Sample\_011046841, Unigene9011\_Sample\_011046841, Unigene12261\_Sample\_011046841, Unigene17921\_Sample\_011046841, Unigene58726\_Sample\_011046841, Unigene26327\_Sample\_011046841, Unigene25101\_Sample\_011046841, Unigene16692\_Sample\_011046841, Unigene14290\_Sample\_011046841, Unigene32247\_Sample\_011046841, Unigene2066\_Sample\_011046841, Unigene11201\_Sample\_011046841, Unigene44876\_Sample\_011046841, Unigene58907\_Sample\_011046841, Unigene38329\_Sample\_011046841, Unigene59628\_Sample\_011046841, Unigene45469\_Sample\_011046841, Unigene47924\_Sample\_011046841, Unigene27486\_Sample\_011046841, Unigene60269\_Sample\_011046841, Unigene2846\_Sample\_011046841, Unigene54628\_Sample\_011046841, Unigene35914\_Sample\_011046841, Unigene52467\_Sample\_011046841, Unigene39712\_Sample\_011046841, Unigene60232\_Sample\_011046841, Unigene55667\_Sample\_011046841, Unigene28662\_Sample\_011046841, Unigene11911\_Sample\_011046841, Unigene10982\_Sample\_011046841, Unigene52869\_Sample\_011046841, Unigene45777\_Sample\_011046841, Unigene25357\_Sample\_011046841, Unigene60189\_Sample\_011046841, Unigene47864\_Sample\_011046841, Unigene47083\_Sample\_011046841, Unigene33884\_Sample\_011046841, Unigene55311\_Sample\_011046841, Unigene19671\_Sample\_011046841, Unigene11351\_Sample\_011046841, Unigene10588\_Sample\_011046841, Unigene13819\_Sample\_011046841, Unigene12822\_Sample\_011046841, Unigene57776\_Sample\_011046841, Unigene46254\_Sample\_011046841, Unigene13722\_Sample\_011046841, Unigene48425\_Sample\_011046841, Unigene15720\_Sample\_011046841, Unigene47309\_Sample\_011046841, Unigene60006\_Sample\_011046841, Unigene15374\_Sample\_011046841, Unigene60642\_Sample\_011046841, Unigene30713\_Sample\_011046841, Unigene52396\_Sample\_011046841, Unigene37729\_Sample\_011046841, Unigene42399\_Sample\_011046841, Unigene23416\_Sample\_011046841, Unigene6254\_Sample\_011046841, Unigene31120\_Sample\_011046841, Unigene59727\_Sample\_011046841, Unigene11148\_Sample\_011046841, Unigene13697\_Sample\_011046841, Unigene35691\_Sample\_011046841, Unigene51977\_Sample\_011046841, Unigene44567\_Sample\_011046841, Unigene57570\_Sample\_011046841, Unigene5462\_Sample\_011046841, Unigene50243\_Sample\_011046841, Unigene7905\_Sample\_011046841, Unigene59743\_Sample\_011046841, Unigene19793\_Sample\_011046841, Unigene27612\_Sample\_011046841, Unigene56629\_Sample\_011046841, Unigene12206\_Sample\_011046841, Unigene22907\_Sample\_011046841, Unigene16199\_Sample\_011046841, Unigene60349\_Sample\_011046841, Unigene49055\_Sample\_011046841, Unigene25307\_Sample\_011046841, Unigene36791\_Sample\_011046841, Unigene23790\_Sample\_011046841, Unigene57773\_Sample\_011046841, Unigene50825\_Sample\_011046841, Unigene51468\_Sample\_011046841, Unigene59396\_Sample\_011046841, Unigene53415\_Sample\_011046841, Unigene3810\_Sample\_011046841, Unigene49192\_Sample\_011046841, Unigene48927\_Sample\_011046841, Unigene58697\_Sample\_011046841, Unigene49921\_Sample\_011046841, Unigene15204\_Sample\_011046841, Unigene4915\_Sample\_011046841, Unigene17752\_Sample\_011046841, Unigene48342\_Sample\_011046841, Unigene58584\_Sample\_011046841, Unigene42576\_Sample\_011046841, Unigene7095\_Sample\_011046841, Unigene56599\_Sample\_011046841, Unigene28537\_Sample\_011046841, Unigene51307\_Sample\_011046841, Unigene59935\_Sample\_011046841, Unigene56584\_Sample\_011046841, Unigene31249\_Sample\_011046841, Unigene13430\_Sample\_011046841, Unigene47837\_Sample\_011046841, Unigene49501\_Sample\_011046841, Unigene60375\_Sample\_011046841, Unigene12703\_Sample\_011046841, Unigene57039\_Sample\_011046841, Unigene40989\_Sample\_011046841, Unigene12331\_Sample\_011046841, Unigene52381\_Sample\_011046841, Unigene33607\_Sample\_011046841, Unigene35008\_Sample\_011046841, Unigene54549\_Sample\_011046841, Unigene50926\_Sample\_011046841, Unigene49337\_Sample\_011046841, Unigene9271\_Sample\_011046841, Unigene42633\_Sample\_011046841, Unigene8851\_Sample\_011046841, Unigene44479\_Sample\_011046841, Unigene46488\_Sample\_011046841, Unigene53302\_Sample\_011046841, Unigene31097\_Sample\_011046841, Unigene11998\_Sample\_011046841, Unigene45004\_Sample\_011046841, Unigene45662\_Sample\_011046841, Unigene60245\_Sample\_011046841, Unigene23201\_Sample\_011046841, Unigene49126\_Sample\_011046841, Unigene45183\_Sample\_011046841, Unigene47025\_Sample\_011046841, Unigene57713\_Sample\_011046841, Unigene54574\_Sample\_011046841, Unigene59114\_Sample\_011046841, Unigene60723\_Sample\_011046841, Unigene52788\_Sample\_011046841, Unigene60906\_Sample\_011046841, Unigene55856\_Sample\_011046841, Unigene13079\_Sample\_011046841, Unigene53888\_Sample\_011046841, Unigene22245\_Sample\_011046841, Unigene1989\_Sample\_011046841, Unigene775\_Sample\_011046841, Unigene44849\_Sample\_011046841, Unigene55021\_Sample\_011046841, Unigene34152\_Sample\_011046841, Unigene39967\_Sample\_011046841, Unigene30153\_Sample\_011046841, Unigene36891\_Sample\_011046841, Unigene38675\_Sample\_011046841, Unigene30530\_Sample\_011046841, Unigene59701\_Sample\_011046841, Unigene59496\_Sample\_011046841, Unigene59021\_Sample\_011046841, Unigene55702\_Sample\_011046841, Unigene8034\_Sample\_011046841, Unigene11726\_Sample\_011046841, Unigene58014\_Sample\_011046841, Unigene24615\_Sample\_011046841, Unigene11786\_Sample\_011046841, Unigene58808\_Sample\_011046841, Unigene12306\_Sample\_011046841, Unigene58285\_Sample\_011046841, Unigene38616\_Sample\_011046841, Unigene52808\_Sample\_011046841, Unigene48044\_Sample\_011046841, Unigene60036\_Sample\_011046841, Unigene40287\_Sample\_011046841, Unigene7011\_Sample\_011046841, Unigene17531\_Sample\_011046841, Unigene13902\_Sample\_011046841, Unigene55781\_Sample\_011046841, Unigene12212\_Sample\_011046841, Unigene60407\_Sample\_011046841, Unigene60478\_Sample\_011046841, Unigene39787\_Sample\_011046841, Unigene55302\_Sample\_011046841, Unigene27618\_Sample\_011046841, Unigene49724\_Sample\_011046841, Unigene47610\_Sample\_011046841, Unigene59005\_Sample\_011046841, Unigene60825\_Sample\_011046841, Unigene55890\_Sample\_011046841, Unigene58210\_Sample\_011046841, Unigene2221\_Sample\_011046841, Unigene26464\_Sample\_011046841, Unigene1887\_Sample\_011046841, Unigene59494\_Sample\_011046841, Unigene55792\_Sample\_011046841, Unigene48473\_Sample\_011046841, Unigene48999\_Sample\_011046841, Unigene44243\_Sample\_011046841, Unigene59514\_Sample\_011046841, Unigene16372\_Sample\_011046841, Unigene52249\_Sample\_011046841, Unigene60887\_Sample\_011046841, Unigene35733\_Sample\_011046841, Unigene12586\_Sample\_011046841, Unigene50207\_Sample\_011046841, Unigene41575\_Sample\_011046841, Unigene45451\_Sample\_011046841, Unigene47149\_Sample\_011046841, Unigene47125\_Sample\_011046841, Unigene2610\_Sample\_011046841, Unigene56391\_Sample\_011046841, Unigene6590\_Sample\_011046841, Unigene56588\_Sample\_011046841, Unigene56715\_Sample\_011046841, Unigene11099\_Sample\_011046841, Unigene3412\_Sample\_011046841, Unigene60847\_Sample\_011046841, Unigene56383\_Sample\_011046841, Unigene11124\_Sample\_011046841, Unigene52695\_Sample\_011046841, Unigene6946\_Sample\_011046841, Unigene10745\_Sample\_011046841, Unigene31905\_Sample\_011046841, Unigene44880\_Sample\_011046841, Unigene57929\_Sample\_011046841, Unigene5382\_Sample\_011046841, Unigene43097\_Sample\_011046841, Unigene46198\_Sample\_011046841, Unigene5054\_Sample\_011046841, Unigene56978\_Sample\_011046841, Unigene60916\_Sample\_011046841, Unigene58114\_Sample\_011046841, Unigene31129\_Sample\_011046841, Unigene48072\_Sample\_011046841, Unigene20702\_Sample\_011046841, Unigene47158\_Sample\_011046841, Unigene58649\_Sample\_011046841, Unigene45885\_Sample\_011046841, Unigene58518\_Sample\_011046841, Unigene39552\_Sample\_011046841, Unigene8229\_Sample\_011046841, Unigene55779\_Sample\_011046841, Unigene58343\_Sample\_011046841, Unigene24979\_Sample\_011046841, Unigene18715\_Sample\_011046841, Unigene57425\_Sample\_011046841, Unigene8641\_Sample\_011046841, Unigene43651\_Sample\_011046841, Unigene26928\_Sample\_011046841, Unigene56389\_Sample\_011046841, Unigene44939\_Sample\_011046841, Unigene42734\_Sample\_011046841, Unigene45951\_Sample\_011046841, Unigene59254\_Sample\_011046841, Unigene59167\_Sample\_011046841, Unigene54599\_Sample\_011046841, Unigene59946\_Sample\_011046841, Unigene48963\_Sample\_011046841, Unigene12957\_Sample\_011046841, Unigene41980\_Sample\_011046841, Unigene7072\_Sample\_011046841, Unigene41178\_Sample\_011046841, Unigene51429\_Sample\_011046841, Unigene54372\_Sample\_011046841, Unigene12412\_Sample\_011046841, Unigene28643\_Sample\_011046841, Unigene10421\_Sample\_011046841, Unigene58989\_Sample\_011046841, Unigene57573\_Sample\_011046841, Unigene2834\_Sample\_011046841, Unigene55499\_Sample\_011046841, Unigene48375\_Sample\_011046841, Unigene58205\_Sample\_011046841, Unigene11168\_Sample\_011046841, Unigene49320\_Sample\_011046841, Unigene251\_Sample\_011046841, Unigene56807\_Sample\_011046841 |
| nucleus | Unigene19912\_Sample\_011046841, Unigene7801\_Sample\_011046841, Unigene713\_Sample\_011046841, Unigene40921\_Sample\_011046841, Unigene34629\_Sample\_011046841, Unigene55221\_Sample\_011046841, Unigene57770\_Sample\_011046841, Unigene56530\_Sample\_011046841, Unigene4096\_Sample\_011046841, Unigene55543\_Sample\_011046841, Unigene15270\_Sample\_011046841, Unigene58571\_Sample\_011046841, Unigene52866\_Sample\_011046841, Unigene29723\_Sample\_011046841, Unigene59633\_Sample\_011046841, Unigene52937\_Sample\_011046841, Unigene57534\_Sample\_011046841, Unigene48415\_Sample\_011046841, Unigene9459\_Sample\_011046841, Unigene55254\_Sample\_011046841, Unigene2263\_Sample\_011046841, Unigene41700\_Sample\_011046841, Unigene33973\_Sample\_011046841, Unigene54454\_Sample\_011046841, Unigene9589\_Sample\_011046841, Unigene10187\_Sample\_011046841, Unigene59972\_Sample\_011046841, Unigene46719\_Sample\_011046841, Unigene29658\_Sample\_011046841, Unigene48260\_Sample\_011046841, Unigene26047\_Sample\_011046841, Unigene28131\_Sample\_011046841, Unigene29839\_Sample\_011046841, Unigene60645\_Sample\_011046841, Unigene12172\_Sample\_011046841, Unigene60161\_Sample\_011046841, Unigene50631\_Sample\_011046841, Unigene8519\_Sample\_011046841, Unigene47723\_Sample\_011046841, Unigene55156\_Sample\_011046841, Unigene57083\_Sample\_011046841, Unigene53650\_Sample\_011046841, Unigene60377\_Sample\_011046841, Unigene44447\_Sample\_011046841, Unigene60153\_Sample\_011046841, Unigene57702\_Sample\_011046841, Unigene31264\_Sample\_011046841, Unigene50051\_Sample\_011046841, Unigene4709\_Sample\_011046841, Unigene42827\_Sample\_011046841, Unigene59022\_Sample\_011046841, Unigene59195\_Sample\_011046841, Unigene8987\_Sample\_011046841, Unigene60842\_Sample\_011046841, Unigene36851\_Sample\_011046841, Unigene9307\_Sample\_011046841, Unigene12309\_Sample\_011046841, Unigene39807\_Sample\_011046841, Unigene2913\_Sample\_011046841, Unigene13256\_Sample\_011046841, Unigene46283\_Sample\_011046841, Unigene41574\_Sample\_011046841, Unigene27013\_Sample\_011046841, Unigene54061\_Sample\_011046841, Unigene31679\_Sample\_011046841, Unigene51504\_Sample\_011046841, Unigene57919\_Sample\_011046841, Unigene59077\_Sample\_011046841, Unigene21535\_Sample\_011046841, Unigene45512\_Sample\_011046841, Unigene55357\_Sample\_011046841, Unigene11114\_Sample\_011046841, Unigene58715\_Sample\_011046841, Unigene13859\_Sample\_011046841, Unigene13436\_Sample\_011046841, Unigene44587\_Sample\_011046841, Unigene23762\_Sample\_011046841, Unigene33440\_Sample\_011046841, Unigene9912\_Sample\_011046841, Unigene29524\_Sample\_011046841, Unigene2398\_Sample\_011046841, Unigene23742\_Sample\_011046841, Unigene50506\_Sample\_011046841, Unigene12623\_Sample\_011046841, Unigene57828\_Sample\_011046841, Unigene55509\_Sample\_011046841, Unigene813\_Sample\_011046841, Unigene1129\_Sample\_011046841, Unigene55036\_Sample\_011046841, Unigene13748\_Sample\_011046841, Unigene36888\_Sample\_011046841, Unigene10962\_Sample\_011046841, Unigene49168\_Sample\_011046841, Unigene13391\_Sample\_011046841, Unigene59547\_Sample\_011046841, Unigene42378\_Sample\_011046841, Unigene54996\_Sample\_011046841, Unigene25267\_Sample\_011046841, Unigene55449\_Sample\_011046841, Unigene60561\_Sample\_011046841, Unigene24493\_Sample\_011046841, Unigene60944\_Sample\_011046841, Unigene47868\_Sample\_011046841, Unigene58386\_Sample\_011046841, Unigene44838\_Sample\_011046841, Unigene12390\_Sample\_011046841, Unigene49895\_Sample\_011046841, Unigene7366\_Sample\_011046841, Unigene19789\_Sample\_011046841, Unigene59985\_Sample\_011046841, Unigene53798\_Sample\_011046841, Unigene41608\_Sample\_011046841, Unigene16763\_Sample\_011046841, Unigene18426\_Sample\_011046841, Unigene58496\_Sample\_011046841, Unigene57070\_Sample\_011046841, Unigene7778\_Sample\_011046841, Unigene59236\_Sample\_011046841, Unigene12701\_Sample\_011046841, Unigene57438\_Sample\_011046841, Unigene57453\_Sample\_011046841, Unigene44362\_Sample\_011046841, Unigene49099\_Sample\_011046841, Unigene50285\_Sample\_011046841, Unigene9623\_Sample\_011046841, Unigene42229\_Sample\_011046841, Unigene32488\_Sample\_011046841, Unigene48049\_Sample\_011046841, Unigene32138\_Sample\_011046841, Unigene13325\_Sample\_011046841, Unigene55183\_Sample\_011046841, Unigene4847\_Sample\_011046841, Unigene57238\_Sample\_011046841, Unigene44749\_Sample\_011046841, Unigene45403\_Sample\_011046841, Unigene34659\_Sample\_011046841, Unigene58777\_Sample\_011046841, Unigene38021\_Sample\_011046841, Unigene8925\_Sample\_011046841, Unigene10027\_Sample\_011046841, Unigene32309\_Sample\_011046841, Unigene32028\_Sample\_011046841, Unigene56883\_Sample\_011046841, Unigene37820\_Sample\_011046841, Unigene31420\_Sample\_011046841, Unigene57124\_Sample\_011046841, Unigene21520\_Sample\_011046841, Unigene58502\_Sample\_011046841, Unigene16650\_Sample\_011046841, Unigene45620\_Sample\_011046841, Unigene54248\_Sample\_011046841, Unigene7092\_Sample\_011046841, Unigene59136\_Sample\_011046841, Unigene41959\_Sample\_011046841, Unigene50990\_Sample\_011046841, Unigene52322\_Sample\_011046841, Unigene57384\_Sample\_011046841, Unigene50223\_Sample\_011046841, Unigene56688\_Sample\_011046841, Unigene50164\_Sample\_011046841, Unigene50912\_Sample\_011046841, Unigene15865\_Sample\_011046841, Unigene54555\_Sample\_011046841, Unigene57062\_Sample\_011046841, Unigene46262\_Sample\_011046841, Unigene6052\_Sample\_011046841, Unigene18725\_Sample\_011046841, Unigene58480\_Sample\_011046841, Unigene50798\_Sample\_011046841, Unigene59512\_Sample\_011046841, Unigene11040\_Sample\_011046841, Unigene58403\_Sample\_011046841, Unigene60049\_Sample\_011046841, Unigene53098\_Sample\_011046841, Unigene55512\_Sample\_011046841, Unigene54406\_Sample\_011046841, Unigene54852\_Sample\_011046841, Unigene58374\_Sample\_011046841, Unigene54790\_Sample\_011046841, Unigene38586\_Sample\_011046841, Unigene41393\_Sample\_011046841, Unigene22323\_Sample\_011046841, Unigene48764\_Sample\_011046841, Unigene29101\_Sample\_011046841, Unigene51803\_Sample\_011046841, Unigene44009\_Sample\_011046841, Unigene51264\_Sample\_011046841, Unigene46791\_Sample\_011046841, Unigene49268\_Sample\_011046841, Unigene8538\_Sample\_011046841, Unigene57100\_Sample\_011046841, Unigene48637\_Sample\_011046841, Unigene57158\_Sample\_011046841, Unigene56919\_Sample\_011046841, Unigene45539\_Sample\_011046841, Unigene28246\_Sample\_011046841, Unigene21907\_Sample\_011046841, Unigene60809\_Sample\_011046841, Unigene8165\_Sample\_011046841, Unigene11180\_Sample\_011046841, Unigene60701\_Sample\_011046841, Unigene60494\_Sample\_011046841, Unigene35453\_Sample\_011046841, Unigene30162\_Sample\_011046841, Unigene59679\_Sample\_011046841, Unigene33130\_Sample\_011046841, Unigene8392\_Sample\_011046841, Unigene50396\_Sample\_011046841, Unigene53015\_Sample\_011046841, Unigene58138\_Sample\_011046841, Unigene20591\_Sample\_011046841, Unigene36469\_Sample\_011046841, Unigene29669\_Sample\_011046841, Unigene17370\_Sample\_011046841, Unigene30385\_Sample\_011046841, Unigene12748\_Sample\_011046841, Unigene4871\_Sample\_011046841, Unigene13249\_Sample\_011046841, Unigene43875\_Sample\_011046841, Unigene48708\_Sample\_011046841, Unigene60309\_Sample\_011046841, Unigene10640\_Sample\_011046841, Unigene53714\_Sample\_011046841, Unigene43848\_Sample\_011046841, Unigene21655\_Sample\_011046841, Unigene53757\_Sample\_011046841, Unigene55306\_Sample\_011046841, Unigene13517\_Sample\_011046841, Unigene40807\_Sample\_011046841, Unigene13258\_Sample\_011046841, Unigene50653\_Sample\_011046841, Unigene58839\_Sample\_011046841, Unigene60381\_Sample\_011046841, Unigene60900\_Sample\_011046841, Unigene51282\_Sample\_011046841, Unigene51569\_Sample\_011046841, Unigene12056\_Sample\_011046841, Unigene59535\_Sample\_011046841, Unigene58929\_Sample\_011046841, Unigene41822\_Sample\_011046841, Unigene49671\_Sample\_011046841, Unigene13203\_Sample\_011046841, Unigene13431\_Sample\_011046841, Unigene23667\_Sample\_011046841, Unigene53072\_Sample\_011046841, Unigene55175\_Sample\_011046841, Unigene59447\_Sample\_011046841, Unigene40147\_Sample\_011046841, Unigene50313\_Sample\_011046841, Unigene8243\_Sample\_011046841, Unigene1952\_Sample\_011046841, Unigene56358\_Sample\_011046841, Unigene13719\_Sample\_011046841, Unigene5603\_Sample\_011046841, Unigene48020\_Sample\_011046841, Unigene40258\_Sample\_011046841, Unigene40558\_Sample\_011046841, Unigene49532\_Sample\_011046841, Unigene9219\_Sample\_011046841, Unigene52688\_Sample\_011046841, Unigene41676\_Sample\_011046841, Unigene3486\_Sample\_011046841, Unigene9304\_Sample\_011046841, Unigene25573\_Sample\_011046841, Unigene21688\_Sample\_011046841, Unigene54402\_Sample\_011046841, Unigene19047\_Sample\_011046841, Unigene56881\_Sample\_011046841, Unigene57132\_Sample\_011046841, Unigene60919\_Sample\_011046841, Unigene47368\_Sample\_011046841, Unigene14355\_Sample\_011046841, Unigene59891\_Sample\_011046841, Unigene44016\_Sample\_011046841, Unigene56920\_Sample\_011046841, Unigene53843\_Sample\_011046841, Unigene4523\_Sample\_011046841, Unigene22255\_Sample\_011046841, Unigene46321\_Sample\_011046841, Unigene10941\_Sample\_011046841, Unigene11187\_Sample\_011046841, Unigene53037\_Sample\_011046841, Unigene10930\_Sample\_011046841, Unigene51110\_Sample\_011046841, Unigene38373\_Sample\_011046841, Unigene55196\_Sample\_011046841, Unigene55432\_Sample\_011046841, Unigene57971\_Sample\_011046841, Unigene44438\_Sample\_011046841, Unigene39200\_Sample\_011046841, Unigene57239\_Sample\_011046841, Unigene50689\_Sample\_011046841, Unigene57610\_Sample\_011046841, Unigene48086\_Sample\_011046841, Unigene59492\_Sample\_011046841, Unigene47981\_Sample\_011046841, Unigene44734\_Sample\_011046841, Unigene60131\_Sample\_011046841, Unigene49633\_Sample\_011046841, Unigene45873\_Sample\_011046841, Unigene42899\_Sample\_011046841, Unigene2532\_Sample\_011046841, Unigene60714\_Sample\_011046841, Unigene22216\_Sample\_011046841, Unigene2325\_Sample\_011046841, Unigene57531\_Sample\_011046841, Unigene8480\_Sample\_011046841, Unigene30681\_Sample\_011046841, Unigene11939\_Sample\_011046841, Unigene9067\_Sample\_011046841, Unigene3946\_Sample\_011046841, Unigene53420\_Sample\_011046841, Unigene37278\_Sample\_011046841, Unigene12562\_Sample\_011046841, Unigene36721\_Sample\_011046841, Unigene58365\_Sample\_011046841, Unigene11053\_Sample\_011046841, Unigene51179\_Sample\_011046841, Unigene57806\_Sample\_011046841, Unigene36824\_Sample\_011046841, Unigene58725\_Sample\_011046841, Unigene39209\_Sample\_011046841, Unigene12670\_Sample\_011046841, Unigene11846\_Sample\_011046841, Unigene12324\_Sample\_011046841, Unigene59653\_Sample\_011046841, Unigene59637\_Sample\_011046841, Unigene21163\_Sample\_011046841, Unigene2861\_Sample\_011046841, Unigene59587\_Sample\_011046841, Unigene31705\_Sample\_011046841, Unigene26904\_Sample\_011046841, Unigene28403\_Sample\_011046841, Unigene50902\_Sample\_011046841, Unigene55769\_Sample\_011046841, Unigene33941\_Sample\_011046841, Unigene55247\_Sample\_011046841, Unigene30285\_Sample\_011046841, Unigene24860\_Sample\_011046841, Unigene55465\_Sample\_011046841, Unigene54870\_Sample\_011046841, Unigene52591\_Sample\_011046841, Unigene50103\_Sample\_011046841, Unigene47555\_Sample\_011046841, Unigene58564\_Sample\_011046841, Unigene56714\_Sample\_011046841, Unigene42735\_Sample\_011046841, Unigene46263\_Sample\_011046841, Unigene42749\_Sample\_011046841, Unigene32703\_Sample\_011046841, Unigene46290\_Sample\_011046841, Unigene47871\_Sample\_011046841, Unigene30649\_Sample\_011046841, Unigene8713\_Sample\_011046841, Unigene13390\_Sample\_011046841, Unigene53095\_Sample\_011046841, Unigene36452\_Sample\_011046841, Unigene20596\_Sample\_011046841, Unigene55077\_Sample\_011046841, Unigene5391\_Sample\_011046841, Unigene60217\_Sample\_011046841, Unigene13694\_Sample\_011046841, Unigene5335\_Sample\_011046841, Unigene59560\_Sample\_011046841, Unigene641\_Sample\_011046841, Unigene11804\_Sample\_011046841, Unigene57614\_Sample\_011046841, Unigene39261\_Sample\_011046841, Unigene57972\_Sample\_011046841, Unigene46207\_Sample\_011046841, Unigene42503\_Sample\_011046841, Unigene29630\_Sample\_011046841, Unigene60247\_Sample\_011046841, Unigene757\_Sample\_011046841, Unigene38304\_Sample\_011046841, Unigene54668\_Sample\_011046841, Unigene52375\_Sample\_011046841, Unigene34268\_Sample\_011046841, Unigene39074\_Sample\_011046841, Unigene13245\_Sample\_011046841, Unigene39621\_Sample\_011046841, Unigene58663\_Sample\_011046841, Unigene50213\_Sample\_011046841, Unigene54297\_Sample\_011046841, Unigene53179\_Sample\_011046841, Unigene27324\_Sample\_011046841, Unigene13584\_Sample\_011046841, Unigene11431\_Sample\_011046841, Unigene49021\_Sample\_011046841, Unigene58659\_Sample\_011046841, Unigene32707\_Sample\_011046841, Unigene33889\_Sample\_011046841, Unigene58488\_Sample\_011046841, Unigene56395\_Sample\_011046841, Unigene4684\_Sample\_011046841, Unigene54240\_Sample\_011046841, Unigene15846\_Sample\_011046841, Unigene40444\_Sample\_011046841, Unigene50764\_Sample\_011046841, Unigene12023\_Sample\_011046841, Unigene46030\_Sample\_011046841, Unigene58513\_Sample\_011046841, Unigene58135\_Sample\_011046841, Unigene51481\_Sample\_011046841, Unigene46220\_Sample\_011046841, Unigene51688\_Sample\_011046841, Unigene2068\_Sample\_011046841, Unigene59973\_Sample\_011046841, Unigene10683\_Sample\_011046841, Unigene50734\_Sample\_011046841, Unigene55325\_Sample\_011046841, Unigene9011\_Sample\_011046841, Unigene12261\_Sample\_011046841, Unigene17921\_Sample\_011046841, Unigene26327\_Sample\_011046841, Unigene58726\_Sample\_011046841, Unigene25101\_Sample\_011046841, Unigene16692\_Sample\_011046841, Unigene14290\_Sample\_011046841, Unigene32247\_Sample\_011046841, Unigene39075\_Sample\_011046841, Unigene51716\_Sample\_011046841, Unigene2066\_Sample\_011046841, Unigene11201\_Sample\_011046841, Unigene44876\_Sample\_011046841, Unigene58907\_Sample\_011046841, Unigene38329\_Sample\_011046841, Unigene45469\_Sample\_011046841, Unigene59628\_Sample\_011046841, Unigene47924\_Sample\_011046841, Unigene27486\_Sample\_011046841, Unigene60269\_Sample\_011046841, Unigene54628\_Sample\_011046841, Unigene2846\_Sample\_011046841, Unigene35914\_Sample\_011046841, Unigene52467\_Sample\_011046841, Unigene39712\_Sample\_011046841, Unigene60232\_Sample\_011046841, Unigene55667\_Sample\_011046841, Unigene28662\_Sample\_011046841, Unigene11911\_Sample\_011046841, Unigene47995\_Sample\_011046841, Unigene10982\_Sample\_011046841, Unigene52869\_Sample\_011046841, Unigene45777\_Sample\_011046841, Unigene25357\_Sample\_011046841, Unigene60189\_Sample\_011046841, Unigene11088\_Sample\_011046841, Unigene47864\_Sample\_011046841, Unigene13890\_Sample\_011046841, Unigene47083\_Sample\_011046841, Unigene33884\_Sample\_011046841, Unigene55311\_Sample\_011046841, Unigene19671\_Sample\_011046841, Unigene11351\_Sample\_011046841, Unigene10588\_Sample\_011046841, Unigene58058\_Sample\_011046841, Unigene13819\_Sample\_011046841, Unigene57776\_Sample\_011046841, Unigene12822\_Sample\_011046841, Unigene46254\_Sample\_011046841, Unigene13722\_Sample\_011046841, Unigene48425\_Sample\_011046841, Unigene15720\_Sample\_011046841, Unigene47309\_Sample\_011046841, Unigene60006\_Sample\_011046841, Unigene15374\_Sample\_011046841, Unigene60642\_Sample\_011046841, Unigene12971\_Sample\_011046841, Unigene30713\_Sample\_011046841, Unigene52396\_Sample\_011046841, Unigene37729\_Sample\_011046841, Unigene42399\_Sample\_011046841, Unigene23416\_Sample\_011046841, Unigene6254\_Sample\_011046841, Unigene31120\_Sample\_011046841, Unigene59727\_Sample\_011046841, Unigene56818\_Sample\_011046841, Unigene11148\_Sample\_011046841, Unigene28348\_Sample\_011046841, Unigene13697\_Sample\_011046841, Unigene35691\_Sample\_011046841, Unigene51977\_Sample\_011046841, Unigene44567\_Sample\_011046841, Unigene57570\_Sample\_011046841, Unigene5462\_Sample\_011046841, Unigene50243\_Sample\_011046841, Unigene3666\_Sample\_011046841, Unigene7905\_Sample\_011046841, Unigene59743\_Sample\_011046841, Unigene19793\_Sample\_011046841, Unigene27612\_Sample\_011046841, Unigene56629\_Sample\_011046841, Unigene12206\_Sample\_011046841, Unigene22907\_Sample\_011046841, Unigene16199\_Sample\_011046841, Unigene60349\_Sample\_011046841, Unigene49055\_Sample\_011046841, Unigene25307\_Sample\_011046841, Unigene7335\_Sample\_011046841, Unigene42958\_Sample\_011046841, Unigene36791\_Sample\_011046841, Unigene23790\_Sample\_011046841, Unigene57773\_Sample\_011046841, Unigene50825\_Sample\_011046841, Unigene11546\_Sample\_011046841, Unigene51468\_Sample\_011046841, Unigene59396\_Sample\_011046841, Unigene53415\_Sample\_011046841, Unigene3810\_Sample\_011046841, Unigene49192\_Sample\_011046841, Unigene48927\_Sample\_011046841, Unigene58697\_Sample\_011046841, Unigene49921\_Sample\_011046841, Unigene15204\_Sample\_011046841, Unigene4915\_Sample\_011046841, Unigene17752\_Sample\_011046841, Unigene48342\_Sample\_011046841, Unigene59170\_Sample\_011046841, Unigene58584\_Sample\_011046841, Unigene42576\_Sample\_011046841, Unigene7095\_Sample\_011046841, Unigene56599\_Sample\_011046841, Unigene58943\_Sample\_011046841, Unigene28537\_Sample\_011046841, Unigene51307\_Sample\_011046841, Unigene48197\_Sample\_011046841, Unigene60115\_Sample\_011046841, Unigene59935\_Sample\_011046841, Unigene56584\_Sample\_011046841, Unigene31249\_Sample\_011046841, Unigene13430\_Sample\_011046841, Unigene13213\_Sample\_011046841, Unigene9649\_Sample\_011046841, Unigene47837\_Sample\_011046841, Unigene49501\_Sample\_011046841, Unigene60375\_Sample\_011046841, Unigene12703\_Sample\_011046841, Unigene57039\_Sample\_011046841, Unigene40989\_Sample\_011046841, Unigene12331\_Sample\_011046841, Unigene52381\_Sample\_011046841, Unigene33607\_Sample\_011046841, Unigene35008\_Sample\_011046841, Unigene54549\_Sample\_011046841, Unigene50926\_Sample\_011046841, Unigene49337\_Sample\_011046841, Unigene9271\_Sample\_011046841, Unigene40440\_Sample\_011046841, Unigene42633\_Sample\_011046841, Unigene8851\_Sample\_011046841, Unigene44479\_Sample\_011046841, Unigene53302\_Sample\_011046841, Unigene46488\_Sample\_011046841, Unigene31097\_Sample\_011046841, Unigene11998\_Sample\_011046841, Unigene45004\_Sample\_011046841, Unigene45662\_Sample\_011046841, Unigene58224\_Sample\_011046841, Unigene60245\_Sample\_011046841, Unigene23201\_Sample\_011046841, Unigene49126\_Sample\_011046841, Unigene45183\_Sample\_011046841, Unigene47025\_Sample\_011046841, Unigene57713\_Sample\_011046841, Unigene54574\_Sample\_011046841, Unigene59114\_Sample\_011046841, Unigene60342\_Sample\_011046841, Unigene60723\_Sample\_011046841, Unigene52788\_Sample\_011046841, Unigene39966\_Sample\_011046841, Unigene60906\_Sample\_011046841, Unigene55856\_Sample\_011046841, Unigene13079\_Sample\_011046841, Unigene53888\_Sample\_011046841, Unigene22245\_Sample\_011046841, Unigene1989\_Sample\_011046841, Unigene775\_Sample\_011046841, Unigene44849\_Sample\_011046841, Unigene55021\_Sample\_011046841, Unigene34152\_Sample\_011046841, Unigene10669\_Sample\_011046841, Unigene10563\_Sample\_011046841, Unigene39967\_Sample\_011046841, Unigene30153\_Sample\_011046841, Unigene36891\_Sample\_011046841, Unigene38675\_Sample\_011046841, Unigene30530\_Sample\_011046841, Unigene59701\_Sample\_011046841, Unigene59496\_Sample\_011046841, Unigene59021\_Sample\_011046841, Unigene55702\_Sample\_011046841, Unigene60180\_Sample\_011046841, Unigene8034\_Sample\_011046841, Unigene11726\_Sample\_011046841, Unigene28739\_Sample\_011046841, Unigene58014\_Sample\_011046841, Unigene24615\_Sample\_011046841, Unigene11786\_Sample\_011046841, Unigene58808\_Sample\_011046841, Unigene12306\_Sample\_011046841, Unigene58285\_Sample\_011046841, Unigene38616\_Sample\_011046841, Unigene52808\_Sample\_011046841, Unigene48044\_Sample\_011046841, Unigene7206\_Sample\_011046841, Unigene60036\_Sample\_011046841, Unigene40287\_Sample\_011046841, Unigene7011\_Sample\_011046841, Unigene17531\_Sample\_011046841, Unigene13902\_Sample\_011046841, Unigene55781\_Sample\_011046841, Unigene12212\_Sample\_011046841, Unigene60407\_Sample\_011046841, Unigene60478\_Sample\_011046841, Unigene40344\_Sample\_011046841, Unigene39787\_Sample\_011046841, Unigene55302\_Sample\_011046841, Unigene27618\_Sample\_011046841, Unigene49724\_Sample\_011046841, Unigene47610\_Sample\_011046841, Unigene59005\_Sample\_011046841, Unigene60825\_Sample\_011046841, Unigene60294\_Sample\_011046841, Unigene55890\_Sample\_011046841, Unigene58210\_Sample\_011046841, Unigene2221\_Sample\_011046841, Unigene26464\_Sample\_011046841, Unigene1887\_Sample\_011046841, Unigene59494\_Sample\_011046841, Unigene8590\_Sample\_011046841, Unigene55792\_Sample\_011046841, Unigene48473\_Sample\_011046841, Unigene48999\_Sample\_011046841, Unigene44243\_Sample\_011046841, Unigene59514\_Sample\_011046841, Unigene16372\_Sample\_011046841, Unigene52249\_Sample\_011046841, Unigene60887\_Sample\_011046841, Unigene35733\_Sample\_011046841, Unigene12586\_Sample\_011046841, Unigene3252\_Sample\_011046841, Unigene50207\_Sample\_011046841, Unigene41575\_Sample\_011046841, Unigene45451\_Sample\_011046841, Unigene8502\_Sample\_011046841, Unigene47149\_Sample\_011046841, Unigene2610\_Sample\_011046841, Unigene47125\_Sample\_011046841, Unigene9517\_Sample\_011046841, Unigene56391\_Sample\_011046841, Unigene6590\_Sample\_011046841, Unigene56588\_Sample\_011046841, Unigene56715\_Sample\_011046841, Unigene11099\_Sample\_011046841, Unigene3412\_Sample\_011046841, Unigene60847\_Sample\_011046841, Unigene56383\_Sample\_011046841, Unigene11124\_Sample\_011046841, Unigene52695\_Sample\_011046841, Unigene6946\_Sample\_011046841, Unigene10745\_Sample\_011046841, Unigene31905\_Sample\_011046841, Unigene44880\_Sample\_011046841, Unigene29140\_Sample\_011046841, Unigene57929\_Sample\_011046841, Unigene5382\_Sample\_011046841, Unigene55995\_Sample\_011046841, Unigene43097\_Sample\_011046841, Unigene42559\_Sample\_011046841, Unigene46198\_Sample\_011046841, Unigene92\_Sample\_011046841, Unigene5054\_Sample\_011046841, Unigene56978\_Sample\_011046841, Unigene60916\_Sample\_011046841, Unigene58114\_Sample\_011046841, Unigene31129\_Sample\_011046841, Unigene3659\_Sample\_011046841, Unigene20702\_Sample\_011046841, Unigene48072\_Sample\_011046841, Unigene47158\_Sample\_011046841, Unigene58649\_Sample\_011046841, Unigene45885\_Sample\_011046841, Unigene58518\_Sample\_011046841, Unigene39552\_Sample\_011046841, Unigene8229\_Sample\_011046841, Unigene55779\_Sample\_011046841, Unigene58343\_Sample\_011046841, Unigene24979\_Sample\_011046841, Unigene18715\_Sample\_011046841, Unigene57425\_Sample\_011046841, Unigene8931\_Sample\_011046841, Unigene8641\_Sample\_011046841, Unigene43651\_Sample\_011046841, Unigene26928\_Sample\_011046841, Unigene56389\_Sample\_011046841, Unigene55068\_Sample\_011046841, Unigene44939\_Sample\_011046841, Unigene42734\_Sample\_011046841, Unigene56869\_Sample\_011046841, Unigene45951\_Sample\_011046841, Unigene59254\_Sample\_011046841, Unigene59167\_Sample\_011046841, Unigene54599\_Sample\_011046841, Unigene59946\_Sample\_011046841, Unigene48963\_Sample\_011046841, Unigene12957\_Sample\_011046841, Unigene7125\_Sample\_011046841, Unigene41980\_Sample\_011046841, Unigene7072\_Sample\_011046841, Unigene41178\_Sample\_011046841, Unigene51429\_Sample\_011046841, Unigene54372\_Sample\_011046841, Unigene59292\_Sample\_011046841, Unigene12412\_Sample\_011046841, Unigene28643\_Sample\_011046841, Unigene10421\_Sample\_011046841, Unigene58989\_Sample\_011046841, Unigene57573\_Sample\_011046841, Unigene2834\_Sample\_011046841, Unigene55499\_Sample\_011046841, Unigene48375\_Sample\_011046841, Unigene49320\_Sample\_011046841, Unigene58205\_Sample\_011046841, Unigene57490\_Sample\_011046841, Unigene11168\_Sample\_011046841, Unigene251\_Sample\_011046841, Unigene56807\_Sample\_011046841 |
| membrane-enclosed lumen | Unigene11804\_Sample\_011046841, Unigene19912\_Sample\_011046841, Unigene57614\_Sample\_011046841, Unigene39261\_Sample\_011046841, Unigene713\_Sample\_011046841, Unigene57972\_Sample\_011046841, Unigene40921\_Sample\_011046841, Unigene27178\_Sample\_011046841, Unigene34629\_Sample\_011046841, Unigene55221\_Sample\_011046841, Unigene57770\_Sample\_011046841, Unigene42259\_Sample\_011046841, Unigene29630\_Sample\_011046841, Unigene757\_Sample\_011046841, Unigene38304\_Sample\_011046841, Unigene54668\_Sample\_011046841, Unigene52375\_Sample\_011046841, Unigene15270\_Sample\_011046841, Unigene52866\_Sample\_011046841, Unigene34268\_Sample\_011046841, Unigene20433\_Sample\_011046841, Unigene59633\_Sample\_011046841, Unigene48415\_Sample\_011046841, Unigene39074\_Sample\_011046841, Unigene9459\_Sample\_011046841, Unigene39621\_Sample\_011046841, Unigene44985\_Sample\_011046841, Unigene55254\_Sample\_011046841, Unigene41700\_Sample\_011046841, Unigene54851\_Sample\_011046841, Unigene33973\_Sample\_011046841, Unigene54454\_Sample\_011046841, Unigene50213\_Sample\_011046841, Unigene9589\_Sample\_011046841, Unigene34039\_Sample\_011046841, Unigene40873\_Sample\_011046841, Unigene53179\_Sample\_011046841, Unigene27324\_Sample\_011046841, Unigene59972\_Sample\_011046841, Unigene11431\_Sample\_011046841, Unigene48260\_Sample\_011046841, Unigene58488\_Sample\_011046841, Unigene56395\_Sample\_011046841, Unigene12172\_Sample\_011046841, Unigene4684\_Sample\_011046841, Unigene50631\_Sample\_011046841, Unigene55156\_Sample\_011046841, Unigene57083\_Sample\_011046841, Unigene50764\_Sample\_011046841, Unigene58513\_Sample\_011046841, Unigene44447\_Sample\_011046841, Unigene58135\_Sample\_011046841, Unigene51481\_Sample\_011046841, Unigene26672\_Sample\_011046841, Unigene59973\_Sample\_011046841, Unigene2068\_Sample\_011046841, Unigene51688\_Sample\_011046841, Unigene50734\_Sample\_011046841, Unigene55325\_Sample\_011046841, Unigene9011\_Sample\_011046841, Unigene12261\_Sample\_011046841, Unigene31264\_Sample\_011046841, Unigene26327\_Sample\_011046841, Unigene58726\_Sample\_011046841, Unigene42827\_Sample\_011046841, Unigene59022\_Sample\_011046841, Unigene59195\_Sample\_011046841, Unigene16692\_Sample\_011046841, Unigene14290\_Sample\_011046841, Unigene46366\_Sample\_011046841, Unigene60842\_Sample\_011046841, Unigene57730\_Sample\_011046841, Unigene36851\_Sample\_011046841, Unigene2066\_Sample\_011046841, Unigene11201\_Sample\_011046841, Unigene52685\_Sample\_011046841, Unigene58907\_Sample\_011046841, Unigene44876\_Sample\_011046841, Unigene39807\_Sample\_011046841, Unigene55981\_Sample\_011046841, Unigene38329\_Sample\_011046841, Unigene46283\_Sample\_011046841, Unigene59628\_Sample\_011046841, Unigene41574\_Sample\_011046841, Unigene47924\_Sample\_011046841, Unigene54061\_Sample\_011046841, Unigene31679\_Sample\_011046841, Unigene51504\_Sample\_011046841, Unigene54628\_Sample\_011046841, Unigene35914\_Sample\_011046841, Unigene52467\_Sample\_011046841, Unigene21535\_Sample\_011046841, Unigene60232\_Sample\_011046841, Unigene39712\_Sample\_011046841, Unigene35438\_Sample\_011046841, Unigene18442\_Sample\_011046841, Unigene45512\_Sample\_011046841, Unigene55357\_Sample\_011046841, Unigene55667\_Sample\_011046841, Unigene13436\_Sample\_011046841, Unigene44587\_Sample\_011046841, Unigene11911\_Sample\_011046841, Unigene31853\_Sample\_011046841, Unigene9912\_Sample\_011046841, Unigene29524\_Sample\_011046841, Unigene2398\_Sample\_011046841, Unigene10982\_Sample\_011046841, Unigene52869\_Sample\_011046841, Unigene45777\_Sample\_011046841, Unigene12623\_Sample\_011046841, Unigene13951\_Sample\_011046841, Unigene55509\_Sample\_011046841, Unigene1129\_Sample\_011046841, Unigene55036\_Sample\_011046841, Unigene33884\_Sample\_011046841, Unigene58400\_Sample\_011046841, Unigene19671\_Sample\_011046841, Unigene10793\_Sample\_011046841, Unigene13748\_Sample\_011046841, Unigene10962\_Sample\_011046841, Unigene4226\_Sample\_011046841, Unigene49168\_Sample\_011046841, Unigene13391\_Sample\_011046841, Unigene42378\_Sample\_011046841, Unigene10588\_Sample\_011046841, Unigene54996\_Sample\_011046841, Unigene57776\_Sample\_011046841, Unigene12822\_Sample\_011046841, Unigene46254\_Sample\_011046841, Unigene47868\_Sample\_011046841, Unigene48425\_Sample\_011046841, Unigene13722\_Sample\_011046841, Unigene52077\_Sample\_011046841, Unigene58386\_Sample\_011046841, Unigene15720\_Sample\_011046841, Unigene48054\_Sample\_011046841, Unigene15374\_Sample\_011046841, Unigene12390\_Sample\_011046841, Unigene49895\_Sample\_011046841, Unigene52396\_Sample\_011046841, Unigene37729\_Sample\_011046841, Unigene23416\_Sample\_011046841, Unigene42399\_Sample\_011046841, Unigene19789\_Sample\_011046841, Unigene59985\_Sample\_011046841, Unigene59727\_Sample\_011046841, Unigene43662\_Sample\_011046841, Unigene16763\_Sample\_011046841, Unigene58496\_Sample\_011046841, Unigene57070\_Sample\_011046841, Unigene7778\_Sample\_011046841, Unigene13697\_Sample\_011046841, Unigene57438\_Sample\_011046841, Unigene57453\_Sample\_011046841, Unigene35691\_Sample\_011046841, Unigene51977\_Sample\_011046841, Unigene44567\_Sample\_011046841, Unigene50243\_Sample\_011046841, Unigene5462\_Sample\_011046841, Unigene57570\_Sample\_011046841, Unigene27612\_Sample\_011046841, Unigene59743\_Sample\_011046841, Unigene7905\_Sample\_011046841, Unigene44362\_Sample\_011046841, Unigene49099\_Sample\_011046841, Unigene12206\_Sample\_011046841, Unigene56629\_Sample\_011046841, Unigene22907\_Sample\_011046841, Unigene42229\_Sample\_011046841, Unigene48049\_Sample\_011046841, Unigene32138\_Sample\_011046841, Unigene13325\_Sample\_011046841, Unigene55183\_Sample\_011046841, Unigene16199\_Sample\_011046841, Unigene4847\_Sample\_011046841, Unigene57889\_Sample\_011046841, Unigene60349\_Sample\_011046841, Unigene44749\_Sample\_011046841, Unigene49055\_Sample\_011046841, Unigene45403\_Sample\_011046841, Unigene34659\_Sample\_011046841, Unigene25307\_Sample\_011046841, Unigene8925\_Sample\_011046841, Unigene57773\_Sample\_011046841, Unigene23790\_Sample\_011046841, Unigene60677\_Sample\_011046841, Unigene32309\_Sample\_011046841, Unigene37820\_Sample\_011046841, Unigene51468\_Sample\_011046841, Unigene58502\_Sample\_011046841, Unigene16650\_Sample\_011046841, Unigene45620\_Sample\_011046841, Unigene54248\_Sample\_011046841, Unigene7092\_Sample\_011046841, Unigene54891\_Sample\_011046841, Unigene59136\_Sample\_011046841, Unigene41959\_Sample\_011046841, Unigene50990\_Sample\_011046841, Unigene49192\_Sample\_011046841, Unigene3810\_Sample\_011046841, Unigene48927\_Sample\_011046841, Unigene52322\_Sample\_011046841, Unigene58697\_Sample\_011046841, Unigene13733\_Sample\_011046841, Unigene57384\_Sample\_011046841, Unigene50223\_Sample\_011046841, Unigene49921\_Sample\_011046841, Unigene15204\_Sample\_011046841, Unigene56688\_Sample\_011046841, Unigene50912\_Sample\_011046841, Unigene15865\_Sample\_011046841, Unigene54555\_Sample\_011046841, Unigene4915\_Sample\_011046841, Unigene57062\_Sample\_011046841, Unigene53170\_Sample\_011046841, Unigene48342\_Sample\_011046841, Unigene17752\_Sample\_011046841, Unigene46262\_Sample\_011046841, Unigene18725\_Sample\_011046841, Unigene58584\_Sample\_011046841, Unigene56599\_Sample\_011046841, Unigene58480\_Sample\_011046841, Unigene50798\_Sample\_011046841, Unigene28537\_Sample\_011046841, Unigene59512\_Sample\_011046841, Unigene51307\_Sample\_011046841, Unigene56584\_Sample\_011046841, Unigene13430\_Sample\_011046841, Unigene58403\_Sample\_011046841, Unigene47837\_Sample\_011046841, Unigene49501\_Sample\_011046841, Unigene12703\_Sample\_011046841, Unigene60375\_Sample\_011046841, Unigene57039\_Sample\_011046841, Unigene53098\_Sample\_011046841, Unigene12331\_Sample\_011046841, Unigene52381\_Sample\_011046841, Unigene33607\_Sample\_011046841, Unigene35008\_Sample\_011046841, Unigene50926\_Sample\_011046841, Unigene54549\_Sample\_011046841, Unigene58374\_Sample\_011046841, Unigene13817\_Sample\_011046841, Unigene9271\_Sample\_011046841, Unigene54790\_Sample\_011046841, Unigene49337\_Sample\_011046841, Unigene60872\_Sample\_011046841, Unigene42633\_Sample\_011046841, Unigene8851\_Sample\_011046841, Unigene38586\_Sample\_011046841, Unigene41393\_Sample\_011046841, Unigene44479\_Sample\_011046841, Unigene22323\_Sample\_011046841, Unigene29101\_Sample\_011046841, Unigene51803\_Sample\_011046841, Unigene44009\_Sample\_011046841, Unigene50996\_Sample\_011046841, Unigene41001\_Sample\_011046841, Unigene51264\_Sample\_011046841, Unigene45662\_Sample\_011046841, Unigene60245\_Sample\_011046841, Unigene49126\_Sample\_011046841, Unigene46791\_Sample\_011046841, Unigene45183\_Sample\_011046841, Unigene8538\_Sample\_011046841, Unigene57100\_Sample\_011046841, Unigene48637\_Sample\_011046841, Unigene57158\_Sample\_011046841, Unigene47025\_Sample\_011046841, Unigene56795\_Sample\_011046841, Unigene57713\_Sample\_011046841, Unigene45539\_Sample\_011046841, Unigene28246\_Sample\_011046841, Unigene54574\_Sample\_011046841, Unigene59114\_Sample\_011046841, Unigene60723\_Sample\_011046841, Unigene21907\_Sample\_011046841, Unigene3496\_Sample\_011046841, Unigene52788\_Sample\_011046841, Unigene60809\_Sample\_011046841, Unigene60906\_Sample\_011046841, Unigene13079\_Sample\_011046841, Unigene55856\_Sample\_011046841, Unigene36363\_Sample\_011046841, Unigene22245\_Sample\_011046841, Unigene8165\_Sample\_011046841, Unigene775\_Sample\_011046841, Unigene60701\_Sample\_011046841, Unigene60494\_Sample\_011046841, Unigene34152\_Sample\_011046841, Unigene33130\_Sample\_011046841, Unigene8392\_Sample\_011046841, Unigene50396\_Sample\_011046841, Unigene39967\_Sample\_011046841, Unigene58138\_Sample\_011046841, Unigene30153\_Sample\_011046841, Unigene36469\_Sample\_011046841, Unigene36891\_Sample\_011046841, Unigene49650\_Sample\_011046841, Unigene38675\_Sample\_011046841, Unigene30530\_Sample\_011046841, Unigene4871\_Sample\_011046841, Unigene51077\_Sample\_011046841, Unigene13249\_Sample\_011046841, Unigene59021\_Sample\_011046841, Unigene59496\_Sample\_011046841, Unigene43875\_Sample\_011046841, Unigene60309\_Sample\_011046841, Unigene10640\_Sample\_011046841, Unigene53714\_Sample\_011046841, Unigene55702\_Sample\_011046841, Unigene8034\_Sample\_011046841, Unigene42812\_Sample\_011046841, Unigene24335\_Sample\_011046841, Unigene45754\_Sample\_011046841, Unigene11726\_Sample\_011046841, Unigene24615\_Sample\_011046841, Unigene58014\_Sample\_011046841, Unigene13517\_Sample\_011046841, Unigene11786\_Sample\_011046841, Unigene58839\_Sample\_011046841, Unigene50653\_Sample\_011046841, Unigene60381\_Sample\_011046841, Unigene12306\_Sample\_011046841, Unigene58808\_Sample\_011046841, Unigene60900\_Sample\_011046841, Unigene58285\_Sample\_011046841, Unigene51569\_Sample\_011046841, Unigene17258\_Sample\_011046841, Unigene38616\_Sample\_011046841, Unigene12056\_Sample\_011046841, Unigene52808\_Sample\_011046841, Unigene40697\_Sample\_011046841, Unigene59535\_Sample\_011046841, Unigene58929\_Sample\_011046841, Unigene49671\_Sample\_011046841, Unigene58917\_Sample\_011046841, Unigene48334\_Sample\_011046841, Unigene40287\_Sample\_011046841, Unigene7011\_Sample\_011046841, Unigene17531\_Sample\_011046841, Unigene53072\_Sample\_011046841, Unigene13902\_Sample\_011046841, Unigene55781\_Sample\_011046841, Unigene12212\_Sample\_011046841, Unigene59447\_Sample\_011046841, Unigene40147\_Sample\_011046841, Unigene60478\_Sample\_011046841, Unigene8243\_Sample\_011046841, Unigene1952\_Sample\_011046841, Unigene55302\_Sample\_011046841, Unigene39787\_Sample\_011046841, Unigene13719\_Sample\_011046841, Unigene57000\_Sample\_011046841, Unigene27618\_Sample\_011046841, Unigene48020\_Sample\_011046841, Unigene49724\_Sample\_011046841, Unigene40258\_Sample\_011046841, Unigene47610\_Sample\_011046841, Unigene60825\_Sample\_011046841, Unigene52688\_Sample\_011046841, Unigene9219\_Sample\_011046841, Unigene58210\_Sample\_011046841, Unigene25573\_Sample\_011046841, Unigene21688\_Sample\_011046841, Unigene1887\_Sample\_011046841, Unigene59494\_Sample\_011046841, Unigene28781\_Sample\_011046841, Unigene50555\_Sample\_011046841, Unigene55792\_Sample\_011046841, Unigene48473\_Sample\_011046841, Unigene60919\_Sample\_011046841, Unigene47368\_Sample\_011046841, Unigene14355\_Sample\_011046841, Unigene59891\_Sample\_011046841, Unigene44243\_Sample\_011046841, Unigene59514\_Sample\_011046841, Unigene10327\_Sample\_011046841, Unigene16372\_Sample\_011046841, Unigene4523\_Sample\_011046841, Unigene52249\_Sample\_011046841, Unigene12586\_Sample\_011046841, Unigene53037\_Sample\_011046841, Unigene10930\_Sample\_011046841, Unigene51110\_Sample\_011046841, Unigene38373\_Sample\_011046841, Unigene2610\_Sample\_011046841, Unigene55196\_Sample\_011046841, Unigene47125\_Sample\_011046841, Unigene56391\_Sample\_011046841, Unigene44438\_Sample\_011046841, Unigene39200\_Sample\_011046841, Unigene57239\_Sample\_011046841, Unigene30939\_Sample\_011046841, Unigene11099\_Sample\_011046841, Unigene3412\_Sample\_011046841, Unigene55900\_Sample\_011046841, Unigene60847\_Sample\_011046841, Unigene57610\_Sample\_011046841, Unigene11478\_Sample\_011046841, Unigene59492\_Sample\_011046841, Unigene6946\_Sample\_011046841, Unigene52695\_Sample\_011046841, Unigene47981\_Sample\_011046841, Unigene31905\_Sample\_011046841, Unigene44734\_Sample\_011046841, Unigene44880\_Sample\_011046841, Unigene49633\_Sample\_011046841, Unigene57929\_Sample\_011046841, Unigene5382\_Sample\_011046841, Unigene45873\_Sample\_011046841, Unigene2532\_Sample\_011046841, Unigene60714\_Sample\_011046841, Unigene22216\_Sample\_011046841, Unigene2325\_Sample\_011046841, Unigene46198\_Sample\_011046841, Unigene8480\_Sample\_011046841, Unigene5054\_Sample\_011046841, Unigene30681\_Sample\_011046841, Unigene11939\_Sample\_011046841, Unigene9067\_Sample\_011046841, Unigene60916\_Sample\_011046841, Unigene3946\_Sample\_011046841, Unigene58114\_Sample\_011046841, Unigene31129\_Sample\_011046841, Unigene20702\_Sample\_011046841, Unigene48072\_Sample\_011046841, Unigene47158\_Sample\_011046841, Unigene37278\_Sample\_011046841, Unigene58649\_Sample\_011046841, Unigene45885\_Sample\_011046841, Unigene25961\_Sample\_011046841, Unigene58518\_Sample\_011046841, Unigene39552\_Sample\_011046841, Unigene58365\_Sample\_011046841, Unigene11053\_Sample\_011046841, Unigene57806\_Sample\_011046841, Unigene8229\_Sample\_011046841, Unigene36824\_Sample\_011046841, Unigene55779\_Sample\_011046841, Unigene39209\_Sample\_011046841, Unigene46688\_Sample\_011046841, Unigene24979\_Sample\_011046841, Unigene58343\_Sample\_011046841, Unigene11846\_Sample\_011046841, Unigene18715\_Sample\_011046841, Unigene12324\_Sample\_011046841, Unigene57425\_Sample\_011046841, Unigene59637\_Sample\_011046841, Unigene2861\_Sample\_011046841, Unigene59587\_Sample\_011046841, Unigene43651\_Sample\_011046841, Unigene8641\_Sample\_011046841, Unigene31705\_Sample\_011046841, Unigene26928\_Sample\_011046841, Unigene26904\_Sample\_011046841, Unigene56389\_Sample\_011046841, Unigene28403\_Sample\_011046841, Unigene31534\_Sample\_011046841, Unigene44939\_Sample\_011046841, Unigene50902\_Sample\_011046841, Unigene42734\_Sample\_011046841, Unigene55247\_Sample\_011046841, Unigene24860\_Sample\_011046841, Unigene12957\_Sample\_011046841, Unigene41980\_Sample\_011046841, Unigene7072\_Sample\_011046841, Unigene47555\_Sample\_011046841, Unigene58564\_Sample\_011046841, Unigene42735\_Sample\_011046841, Unigene46263\_Sample\_011046841, Unigene51429\_Sample\_011046841, Unigene12412\_Sample\_011046841, Unigene56559\_Sample\_011046841, Unigene42749\_Sample\_011046841, Unigene28643\_Sample\_011046841, Unigene47871\_Sample\_011046841, Unigene30649\_Sample\_011046841, Unigene58989\_Sample\_011046841, Unigene38930\_Sample\_011046841, Unigene36452\_Sample\_011046841, Unigene20596\_Sample\_011046841, Unigene55077\_Sample\_011046841, Unigene56726\_Sample\_011046841, Unigene55499\_Sample\_011046841, Unigene60217\_Sample\_011046841, Unigene59560\_Sample\_011046841, Unigene48375\_Sample\_011046841, Unigene11168\_Sample\_011046841, Unigene251\_Sample\_011046841, Unigene56807\_Sample\_011046841, Unigene641\_Sample\_011046841 |
| organelle lumen | Unigene11804\_Sample\_011046841, Unigene19912\_Sample\_011046841, Unigene57614\_Sample\_011046841, Unigene39261\_Sample\_011046841, Unigene713\_Sample\_011046841, Unigene57972\_Sample\_011046841, Unigene40921\_Sample\_011046841, Unigene27178\_Sample\_011046841, Unigene34629\_Sample\_011046841, Unigene55221\_Sample\_011046841, Unigene57770\_Sample\_011046841, Unigene42259\_Sample\_011046841, Unigene29630\_Sample\_011046841, Unigene757\_Sample\_011046841, Unigene38304\_Sample\_011046841, Unigene54668\_Sample\_011046841, Unigene52375\_Sample\_011046841, Unigene15270\_Sample\_011046841, Unigene52866\_Sample\_011046841, Unigene34268\_Sample\_011046841, Unigene20433\_Sample\_011046841, Unigene59633\_Sample\_011046841, Unigene48415\_Sample\_011046841, Unigene39074\_Sample\_011046841, Unigene9459\_Sample\_011046841, Unigene39621\_Sample\_011046841, Unigene44985\_Sample\_011046841, Unigene55254\_Sample\_011046841, Unigene41700\_Sample\_011046841, Unigene54851\_Sample\_011046841, Unigene33973\_Sample\_011046841, Unigene54454\_Sample\_011046841, Unigene50213\_Sample\_011046841, Unigene9589\_Sample\_011046841, Unigene34039\_Sample\_011046841, Unigene53179\_Sample\_011046841, Unigene27324\_Sample\_011046841, Unigene59972\_Sample\_011046841, Unigene11431\_Sample\_011046841, Unigene48260\_Sample\_011046841, Unigene58488\_Sample\_011046841, Unigene56395\_Sample\_011046841, Unigene12172\_Sample\_011046841, Unigene4684\_Sample\_011046841, Unigene50631\_Sample\_011046841, Unigene55156\_Sample\_011046841, Unigene57083\_Sample\_011046841, Unigene50764\_Sample\_011046841, Unigene58513\_Sample\_011046841, Unigene44447\_Sample\_011046841, Unigene58135\_Sample\_011046841, Unigene51481\_Sample\_011046841, Unigene26672\_Sample\_011046841, Unigene59973\_Sample\_011046841, Unigene2068\_Sample\_011046841, Unigene51688\_Sample\_011046841, Unigene50734\_Sample\_011046841, Unigene55325\_Sample\_011046841, Unigene9011\_Sample\_011046841, Unigene12261\_Sample\_011046841, Unigene31264\_Sample\_011046841, Unigene26327\_Sample\_011046841, Unigene58726\_Sample\_011046841, Unigene42827\_Sample\_011046841, Unigene59022\_Sample\_011046841, Unigene59195\_Sample\_011046841, Unigene16692\_Sample\_011046841, Unigene14290\_Sample\_011046841, Unigene46366\_Sample\_011046841, Unigene60842\_Sample\_011046841, Unigene57730\_Sample\_011046841, Unigene36851\_Sample\_011046841, Unigene2066\_Sample\_011046841, Unigene11201\_Sample\_011046841, Unigene52685\_Sample\_011046841, Unigene58907\_Sample\_011046841, Unigene44876\_Sample\_011046841, Unigene39807\_Sample\_011046841, Unigene55981\_Sample\_011046841, Unigene38329\_Sample\_011046841, Unigene46283\_Sample\_011046841, Unigene59628\_Sample\_011046841, Unigene41574\_Sample\_011046841, Unigene47924\_Sample\_011046841, Unigene54061\_Sample\_011046841, Unigene31679\_Sample\_011046841, Unigene51504\_Sample\_011046841, Unigene54628\_Sample\_011046841, Unigene35914\_Sample\_011046841, Unigene52467\_Sample\_011046841, Unigene21535\_Sample\_011046841, Unigene60232\_Sample\_011046841, Unigene39712\_Sample\_011046841, Unigene35438\_Sample\_011046841, Unigene18442\_Sample\_011046841, Unigene45512\_Sample\_011046841, Unigene55357\_Sample\_011046841, Unigene55667\_Sample\_011046841, Unigene13436\_Sample\_011046841, Unigene44587\_Sample\_011046841, Unigene11911\_Sample\_011046841, Unigene31853\_Sample\_011046841, Unigene9912\_Sample\_011046841, Unigene29524\_Sample\_011046841, Unigene2398\_Sample\_011046841, Unigene10982\_Sample\_011046841, Unigene52869\_Sample\_011046841, Unigene45777\_Sample\_011046841, Unigene12623\_Sample\_011046841, Unigene13951\_Sample\_011046841, Unigene55509\_Sample\_011046841, Unigene1129\_Sample\_011046841, Unigene55036\_Sample\_011046841, Unigene33884\_Sample\_011046841, Unigene58400\_Sample\_011046841, Unigene19671\_Sample\_011046841, Unigene10793\_Sample\_011046841, Unigene13748\_Sample\_011046841, Unigene10962\_Sample\_011046841, Unigene4226\_Sample\_011046841, Unigene49168\_Sample\_011046841, Unigene13391\_Sample\_011046841, Unigene42378\_Sample\_011046841, Unigene10588\_Sample\_011046841, Unigene54996\_Sample\_011046841, Unigene57776\_Sample\_011046841, Unigene12822\_Sample\_011046841, Unigene46254\_Sample\_011046841, Unigene47868\_Sample\_011046841, Unigene48425\_Sample\_011046841, Unigene13722\_Sample\_011046841, Unigene52077\_Sample\_011046841, Unigene58386\_Sample\_011046841, Unigene15720\_Sample\_011046841, Unigene48054\_Sample\_011046841, Unigene15374\_Sample\_011046841, Unigene12390\_Sample\_011046841, Unigene49895\_Sample\_011046841, Unigene52396\_Sample\_011046841, Unigene37729\_Sample\_011046841, Unigene23416\_Sample\_011046841, Unigene42399\_Sample\_011046841, Unigene19789\_Sample\_011046841, Unigene59985\_Sample\_011046841, Unigene59727\_Sample\_011046841, Unigene43662\_Sample\_011046841, Unigene16763\_Sample\_011046841, Unigene58496\_Sample\_011046841, Unigene57070\_Sample\_011046841, Unigene7778\_Sample\_011046841, Unigene13697\_Sample\_011046841, Unigene57438\_Sample\_011046841, Unigene57453\_Sample\_011046841, Unigene35691\_Sample\_011046841, Unigene51977\_Sample\_011046841, Unigene44567\_Sample\_011046841, Unigene50243\_Sample\_011046841, Unigene5462\_Sample\_011046841, Unigene57570\_Sample\_011046841, Unigene27612\_Sample\_011046841, Unigene59743\_Sample\_011046841, Unigene7905\_Sample\_011046841, Unigene44362\_Sample\_011046841, Unigene49099\_Sample\_011046841, Unigene12206\_Sample\_011046841, Unigene56629\_Sample\_011046841, Unigene22907\_Sample\_011046841, Unigene42229\_Sample\_011046841, Unigene48049\_Sample\_011046841, Unigene32138\_Sample\_011046841, Unigene13325\_Sample\_011046841, Unigene55183\_Sample\_011046841, Unigene16199\_Sample\_011046841, Unigene4847\_Sample\_011046841, Unigene60349\_Sample\_011046841, Unigene44749\_Sample\_011046841, Unigene49055\_Sample\_011046841, Unigene45403\_Sample\_011046841, Unigene34659\_Sample\_011046841, Unigene25307\_Sample\_011046841, Unigene8925\_Sample\_011046841, Unigene57773\_Sample\_011046841, Unigene23790\_Sample\_011046841, Unigene60677\_Sample\_011046841, Unigene32309\_Sample\_011046841, Unigene37820\_Sample\_011046841, Unigene51468\_Sample\_011046841, Unigene58502\_Sample\_011046841, Unigene16650\_Sample\_011046841, Unigene45620\_Sample\_011046841, Unigene54248\_Sample\_011046841, Unigene7092\_Sample\_011046841, Unigene54891\_Sample\_011046841, Unigene59136\_Sample\_011046841, Unigene41959\_Sample\_011046841, Unigene50990\_Sample\_011046841, Unigene49192\_Sample\_011046841, Unigene3810\_Sample\_011046841, Unigene48927\_Sample\_011046841, Unigene52322\_Sample\_011046841, Unigene58697\_Sample\_011046841, Unigene13733\_Sample\_011046841, Unigene57384\_Sample\_011046841, Unigene50223\_Sample\_011046841, Unigene49921\_Sample\_011046841, Unigene15204\_Sample\_011046841, Unigene56688\_Sample\_011046841, Unigene50912\_Sample\_011046841, Unigene15865\_Sample\_011046841, Unigene54555\_Sample\_011046841, Unigene4915\_Sample\_011046841, Unigene57062\_Sample\_011046841, Unigene53170\_Sample\_011046841, Unigene48342\_Sample\_011046841, Unigene17752\_Sample\_011046841, Unigene46262\_Sample\_011046841, Unigene18725\_Sample\_011046841, Unigene58584\_Sample\_011046841, Unigene56599\_Sample\_011046841, Unigene58480\_Sample\_011046841, Unigene50798\_Sample\_011046841, Unigene28537\_Sample\_011046841, Unigene59512\_Sample\_011046841, Unigene51307\_Sample\_011046841, Unigene56584\_Sample\_011046841, Unigene13430\_Sample\_011046841, Unigene58403\_Sample\_011046841, Unigene47837\_Sample\_011046841, Unigene49501\_Sample\_011046841, Unigene12703\_Sample\_011046841, Unigene60375\_Sample\_011046841, Unigene57039\_Sample\_011046841, Unigene53098\_Sample\_011046841, Unigene12331\_Sample\_011046841, Unigene52381\_Sample\_011046841, Unigene33607\_Sample\_011046841, Unigene35008\_Sample\_011046841, Unigene50926\_Sample\_011046841, Unigene54549\_Sample\_011046841, Unigene58374\_Sample\_011046841, Unigene13817\_Sample\_011046841, Unigene9271\_Sample\_011046841, Unigene54790\_Sample\_011046841, Unigene49337\_Sample\_011046841, Unigene60872\_Sample\_011046841, Unigene42633\_Sample\_011046841, Unigene8851\_Sample\_011046841, Unigene38586\_Sample\_011046841, Unigene41393\_Sample\_011046841, Unigene44479\_Sample\_011046841, Unigene22323\_Sample\_011046841, Unigene29101\_Sample\_011046841, Unigene51803\_Sample\_011046841, Unigene44009\_Sample\_011046841, Unigene50996\_Sample\_011046841, Unigene41001\_Sample\_011046841, Unigene51264\_Sample\_011046841, Unigene45662\_Sample\_011046841, Unigene60245\_Sample\_011046841, Unigene49126\_Sample\_011046841, Unigene46791\_Sample\_011046841, Unigene45183\_Sample\_011046841, Unigene8538\_Sample\_011046841, Unigene57100\_Sample\_011046841, Unigene48637\_Sample\_011046841, Unigene57158\_Sample\_011046841, Unigene47025\_Sample\_011046841, Unigene56795\_Sample\_011046841, Unigene57713\_Sample\_011046841, Unigene45539\_Sample\_011046841, Unigene28246\_Sample\_011046841, Unigene54574\_Sample\_011046841, Unigene59114\_Sample\_011046841, Unigene60723\_Sample\_011046841, Unigene21907\_Sample\_011046841, Unigene3496\_Sample\_011046841, Unigene52788\_Sample\_011046841, Unigene60809\_Sample\_011046841, Unigene60906\_Sample\_011046841, Unigene13079\_Sample\_011046841, Unigene55856\_Sample\_011046841, Unigene36363\_Sample\_011046841, Unigene22245\_Sample\_011046841, Unigene8165\_Sample\_011046841, Unigene775\_Sample\_011046841, Unigene60701\_Sample\_011046841, Unigene60494\_Sample\_011046841, Unigene34152\_Sample\_011046841, Unigene33130\_Sample\_011046841, Unigene8392\_Sample\_011046841, Unigene50396\_Sample\_011046841, Unigene39967\_Sample\_011046841, Unigene58138\_Sample\_011046841, Unigene30153\_Sample\_011046841, Unigene36469\_Sample\_011046841, Unigene36891\_Sample\_011046841, Unigene49650\_Sample\_011046841, Unigene38675\_Sample\_011046841, Unigene30530\_Sample\_011046841, Unigene4871\_Sample\_011046841, Unigene51077\_Sample\_011046841, Unigene13249\_Sample\_011046841, Unigene59021\_Sample\_011046841, Unigene59496\_Sample\_011046841, Unigene43875\_Sample\_011046841, Unigene60309\_Sample\_011046841, Unigene10640\_Sample\_011046841, Unigene53714\_Sample\_011046841, Unigene55702\_Sample\_011046841, Unigene8034\_Sample\_011046841, Unigene42812\_Sample\_011046841, Unigene24335\_Sample\_011046841, Unigene45754\_Sample\_011046841, Unigene11726\_Sample\_011046841, Unigene24615\_Sample\_011046841, Unigene58014\_Sample\_011046841, Unigene13517\_Sample\_011046841, Unigene11786\_Sample\_011046841, Unigene58839\_Sample\_011046841, Unigene50653\_Sample\_011046841, Unigene60381\_Sample\_011046841, Unigene12306\_Sample\_011046841, Unigene58808\_Sample\_011046841, Unigene60900\_Sample\_011046841, Unigene58285\_Sample\_011046841, Unigene51569\_Sample\_011046841, Unigene17258\_Sample\_011046841, Unigene38616\_Sample\_011046841, Unigene12056\_Sample\_011046841, Unigene52808\_Sample\_011046841, Unigene40697\_Sample\_011046841, Unigene59535\_Sample\_011046841, Unigene58929\_Sample\_011046841, Unigene49671\_Sample\_011046841, Unigene58917\_Sample\_011046841, Unigene48334\_Sample\_011046841, Unigene40287\_Sample\_011046841, Unigene7011\_Sample\_011046841, Unigene17531\_Sample\_011046841, Unigene53072\_Sample\_011046841, Unigene13902\_Sample\_011046841, Unigene55781\_Sample\_011046841, Unigene12212\_Sample\_011046841, Unigene59447\_Sample\_011046841, Unigene40147\_Sample\_011046841, Unigene60478\_Sample\_011046841, Unigene8243\_Sample\_011046841, Unigene1952\_Sample\_011046841, Unigene55302\_Sample\_011046841, Unigene39787\_Sample\_011046841, Unigene13719\_Sample\_011046841, Unigene57000\_Sample\_011046841, Unigene27618\_Sample\_011046841, Unigene48020\_Sample\_011046841, Unigene49724\_Sample\_011046841, Unigene40258\_Sample\_011046841, Unigene47610\_Sample\_011046841, Unigene60825\_Sample\_011046841, Unigene52688\_Sample\_011046841, Unigene9219\_Sample\_011046841, Unigene58210\_Sample\_011046841, Unigene25573\_Sample\_011046841, Unigene21688\_Sample\_011046841, Unigene1887\_Sample\_011046841, Unigene59494\_Sample\_011046841, Unigene28781\_Sample\_011046841, Unigene50555\_Sample\_011046841, Unigene55792\_Sample\_011046841, Unigene48473\_Sample\_011046841, Unigene60919\_Sample\_011046841, Unigene47368\_Sample\_011046841, Unigene14355\_Sample\_011046841, Unigene59891\_Sample\_011046841, Unigene44243\_Sample\_011046841, Unigene59514\_Sample\_011046841, Unigene10327\_Sample\_011046841, Unigene16372\_Sample\_011046841, Unigene4523\_Sample\_011046841, Unigene52249\_Sample\_011046841, Unigene12586\_Sample\_011046841, Unigene53037\_Sample\_011046841, Unigene10930\_Sample\_011046841, Unigene51110\_Sample\_011046841, Unigene38373\_Sample\_011046841, Unigene2610\_Sample\_011046841, Unigene55196\_Sample\_011046841, Unigene47125\_Sample\_011046841, Unigene56391\_Sample\_011046841, Unigene44438\_Sample\_011046841, Unigene39200\_Sample\_011046841, Unigene57239\_Sample\_011046841, Unigene30939\_Sample\_011046841, Unigene11099\_Sample\_011046841, Unigene3412\_Sample\_011046841, Unigene55900\_Sample\_011046841, Unigene60847\_Sample\_011046841, Unigene57610\_Sample\_011046841, Unigene11478\_Sample\_011046841, Unigene59492\_Sample\_011046841, Unigene6946\_Sample\_011046841, Unigene52695\_Sample\_011046841, Unigene47981\_Sample\_011046841, Unigene31905\_Sample\_011046841, Unigene44734\_Sample\_011046841, Unigene44880\_Sample\_011046841, Unigene49633\_Sample\_011046841, Unigene57929\_Sample\_011046841, Unigene5382\_Sample\_011046841, Unigene45873\_Sample\_011046841, Unigene2532\_Sample\_011046841, Unigene60714\_Sample\_011046841, Unigene22216\_Sample\_011046841, Unigene2325\_Sample\_011046841, Unigene46198\_Sample\_011046841, Unigene8480\_Sample\_011046841, Unigene5054\_Sample\_011046841, Unigene30681\_Sample\_011046841, Unigene11939\_Sample\_011046841, Unigene9067\_Sample\_011046841, Unigene60916\_Sample\_011046841, Unigene3946\_Sample\_011046841, Unigene58114\_Sample\_011046841, Unigene31129\_Sample\_011046841, Unigene20702\_Sample\_011046841, Unigene48072\_Sample\_011046841, Unigene47158\_Sample\_011046841, Unigene37278\_Sample\_011046841, Unigene58649\_Sample\_011046841, Unigene45885\_Sample\_011046841, Unigene25961\_Sample\_011046841, Unigene58518\_Sample\_011046841, Unigene39552\_Sample\_011046841, Unigene58365\_Sample\_011046841, Unigene11053\_Sample\_011046841, Unigene57806\_Sample\_011046841, Unigene8229\_Sample\_011046841, Unigene36824\_Sample\_011046841, Unigene55779\_Sample\_011046841, Unigene39209\_Sample\_011046841, Unigene46688\_Sample\_011046841, Unigene24979\_Sample\_011046841, Unigene58343\_Sample\_011046841, Unigene11846\_Sample\_011046841, Unigene18715\_Sample\_011046841, Unigene12324\_Sample\_011046841, Unigene57425\_Sample\_011046841, Unigene59637\_Sample\_011046841, Unigene2861\_Sample\_011046841, Unigene59587\_Sample\_011046841, Unigene43651\_Sample\_011046841, Unigene8641\_Sample\_011046841, Unigene31705\_Sample\_011046841, Unigene26928\_Sample\_011046841, Unigene26904\_Sample\_011046841, Unigene56389\_Sample\_011046841, Unigene28403\_Sample\_011046841, Unigene31534\_Sample\_011046841, Unigene44939\_Sample\_011046841, Unigene50902\_Sample\_011046841, Unigene42734\_Sample\_011046841, Unigene55247\_Sample\_011046841, Unigene24860\_Sample\_011046841, Unigene12957\_Sample\_011046841, Unigene41980\_Sample\_011046841, Unigene7072\_Sample\_011046841, Unigene47555\_Sample\_011046841, Unigene58564\_Sample\_011046841, Unigene42735\_Sample\_011046841, Unigene46263\_Sample\_011046841, Unigene51429\_Sample\_011046841, Unigene12412\_Sample\_011046841, Unigene56559\_Sample\_011046841, Unigene42749\_Sample\_011046841, Unigene28643\_Sample\_011046841, Unigene47871\_Sample\_011046841, Unigene30649\_Sample\_011046841, Unigene58989\_Sample\_011046841, Unigene38930\_Sample\_011046841, Unigene36452\_Sample\_011046841, Unigene20596\_Sample\_011046841, Unigene55077\_Sample\_011046841, Unigene56726\_Sample\_011046841, Unigene55499\_Sample\_011046841, Unigene60217\_Sample\_011046841, Unigene59560\_Sample\_011046841, Unigene48375\_Sample\_011046841, Unigene11168\_Sample\_011046841, Unigene251\_Sample\_011046841, Unigene56807\_Sample\_011046841, Unigene641\_Sample\_011046841 |
| intracellular organelle lumen | Unigene11804\_Sample\_011046841, Unigene19912\_Sample\_011046841, Unigene57614\_Sample\_011046841, Unigene39261\_Sample\_011046841, Unigene713\_Sample\_011046841, Unigene57972\_Sample\_011046841, Unigene40921\_Sample\_011046841, Unigene27178\_Sample\_011046841, Unigene34629\_Sample\_011046841, Unigene55221\_Sample\_011046841, Unigene57770\_Sample\_011046841, Unigene42259\_Sample\_011046841, Unigene29630\_Sample\_011046841, Unigene757\_Sample\_011046841, Unigene38304\_Sample\_011046841, Unigene54668\_Sample\_011046841, Unigene52375\_Sample\_011046841, Unigene15270\_Sample\_011046841, Unigene52866\_Sample\_011046841, Unigene34268\_Sample\_011046841, Unigene20433\_Sample\_011046841, Unigene59633\_Sample\_011046841, Unigene48415\_Sample\_011046841, Unigene39074\_Sample\_011046841, Unigene9459\_Sample\_011046841, Unigene39621\_Sample\_011046841, Unigene44985\_Sample\_011046841, Unigene55254\_Sample\_011046841, Unigene41700\_Sample\_011046841, Unigene54851\_Sample\_011046841, Unigene33973\_Sample\_011046841, Unigene54454\_Sample\_011046841, Unigene50213\_Sample\_011046841, Unigene9589\_Sample\_011046841, Unigene34039\_Sample\_011046841, Unigene53179\_Sample\_011046841, Unigene27324\_Sample\_011046841, Unigene59972\_Sample\_011046841, Unigene11431\_Sample\_011046841, Unigene48260\_Sample\_011046841, Unigene58488\_Sample\_011046841, Unigene56395\_Sample\_011046841, Unigene12172\_Sample\_011046841, Unigene4684\_Sample\_011046841, Unigene50631\_Sample\_011046841, Unigene55156\_Sample\_011046841, Unigene57083\_Sample\_011046841, Unigene50764\_Sample\_011046841, Unigene58513\_Sample\_011046841, Unigene44447\_Sample\_011046841, Unigene58135\_Sample\_011046841, Unigene51481\_Sample\_011046841, Unigene26672\_Sample\_011046841, Unigene59973\_Sample\_011046841, Unigene2068\_Sample\_011046841, Unigene51688\_Sample\_011046841, Unigene50734\_Sample\_011046841, Unigene55325\_Sample\_011046841, Unigene9011\_Sample\_011046841, Unigene12261\_Sample\_011046841, Unigene31264\_Sample\_011046841, Unigene26327\_Sample\_011046841, Unigene58726\_Sample\_011046841, Unigene42827\_Sample\_011046841, Unigene59022\_Sample\_011046841, Unigene59195\_Sample\_011046841, Unigene16692\_Sample\_011046841, Unigene14290\_Sample\_011046841, Unigene46366\_Sample\_011046841, Unigene60842\_Sample\_011046841, Unigene57730\_Sample\_011046841, Unigene36851\_Sample\_011046841, Unigene2066\_Sample\_011046841, Unigene11201\_Sample\_011046841, Unigene52685\_Sample\_011046841, Unigene58907\_Sample\_011046841, Unigene44876\_Sample\_011046841, Unigene39807\_Sample\_011046841, Unigene55981\_Sample\_011046841, Unigene38329\_Sample\_011046841, Unigene46283\_Sample\_011046841, Unigene59628\_Sample\_011046841, Unigene41574\_Sample\_011046841, Unigene47924\_Sample\_011046841, Unigene54061\_Sample\_011046841, Unigene31679\_Sample\_011046841, Unigene51504\_Sample\_011046841, Unigene54628\_Sample\_011046841, Unigene35914\_Sample\_011046841, Unigene52467\_Sample\_011046841, Unigene21535\_Sample\_011046841, Unigene60232\_Sample\_011046841, Unigene39712\_Sample\_011046841, Unigene35438\_Sample\_011046841, Unigene18442\_Sample\_011046841, Unigene45512\_Sample\_011046841, Unigene55357\_Sample\_011046841, Unigene55667\_Sample\_011046841, Unigene13436\_Sample\_011046841, Unigene44587\_Sample\_011046841, Unigene11911\_Sample\_011046841, Unigene31853\_Sample\_011046841, Unigene9912\_Sample\_011046841, Unigene29524\_Sample\_011046841, Unigene2398\_Sample\_011046841, Unigene10982\_Sample\_011046841, Unigene52869\_Sample\_011046841, Unigene45777\_Sample\_011046841, Unigene12623\_Sample\_011046841, Unigene13951\_Sample\_011046841, Unigene55509\_Sample\_011046841, Unigene1129\_Sample\_011046841, Unigene55036\_Sample\_011046841, Unigene33884\_Sample\_011046841, Unigene58400\_Sample\_011046841, Unigene19671\_Sample\_011046841, Unigene10793\_Sample\_011046841, Unigene13748\_Sample\_011046841, Unigene10962\_Sample\_011046841, Unigene4226\_Sample\_011046841, Unigene49168\_Sample\_011046841, Unigene13391\_Sample\_011046841, Unigene42378\_Sample\_011046841, Unigene10588\_Sample\_011046841, Unigene54996\_Sample\_011046841, Unigene57776\_Sample\_011046841, Unigene12822\_Sample\_011046841, Unigene46254\_Sample\_011046841, Unigene47868\_Sample\_011046841, Unigene48425\_Sample\_011046841, Unigene13722\_Sample\_011046841, Unigene52077\_Sample\_011046841, Unigene58386\_Sample\_011046841, Unigene15720\_Sample\_011046841, Unigene48054\_Sample\_011046841, Unigene15374\_Sample\_011046841, Unigene12390\_Sample\_011046841, Unigene49895\_Sample\_011046841, Unigene52396\_Sample\_011046841, Unigene37729\_Sample\_011046841, Unigene23416\_Sample\_011046841, Unigene42399\_Sample\_011046841, Unigene19789\_Sample\_011046841, Unigene59985\_Sample\_011046841, Unigene59727\_Sample\_011046841, Unigene43662\_Sample\_011046841, Unigene16763\_Sample\_011046841, Unigene58496\_Sample\_011046841, Unigene57070\_Sample\_011046841, Unigene7778\_Sample\_011046841, Unigene13697\_Sample\_011046841, Unigene57438\_Sample\_011046841, Unigene57453\_Sample\_011046841, Unigene35691\_Sample\_011046841, Unigene51977\_Sample\_011046841, Unigene44567\_Sample\_011046841, Unigene50243\_Sample\_011046841, Unigene5462\_Sample\_011046841, Unigene57570\_Sample\_011046841, Unigene27612\_Sample\_011046841, Unigene59743\_Sample\_011046841, Unigene7905\_Sample\_011046841, Unigene44362\_Sample\_011046841, Unigene49099\_Sample\_011046841, Unigene12206\_Sample\_011046841, Unigene56629\_Sample\_011046841, Unigene22907\_Sample\_011046841, Unigene42229\_Sample\_011046841, Unigene48049\_Sample\_011046841, Unigene32138\_Sample\_011046841, Unigene13325\_Sample\_011046841, Unigene55183\_Sample\_011046841, Unigene16199\_Sample\_011046841, Unigene4847\_Sample\_011046841, Unigene60349\_Sample\_011046841, Unigene44749\_Sample\_011046841, Unigene49055\_Sample\_011046841, Unigene45403\_Sample\_011046841, Unigene34659\_Sample\_011046841, Unigene25307\_Sample\_011046841, Unigene8925\_Sample\_011046841, Unigene57773\_Sample\_011046841, Unigene23790\_Sample\_011046841, Unigene60677\_Sample\_011046841, Unigene32309\_Sample\_011046841, Unigene37820\_Sample\_011046841, Unigene51468\_Sample\_011046841, Unigene58502\_Sample\_011046841, Unigene16650\_Sample\_011046841, Unigene45620\_Sample\_011046841, Unigene54248\_Sample\_011046841, Unigene7092\_Sample\_011046841, Unigene54891\_Sample\_011046841, Unigene59136\_Sample\_011046841, Unigene41959\_Sample\_011046841, Unigene50990\_Sample\_011046841, Unigene49192\_Sample\_011046841, Unigene3810\_Sample\_011046841, Unigene48927\_Sample\_011046841, Unigene52322\_Sample\_011046841, Unigene58697\_Sample\_011046841, Unigene13733\_Sample\_011046841, Unigene57384\_Sample\_011046841, Unigene50223\_Sample\_011046841, Unigene49921\_Sample\_011046841, Unigene15204\_Sample\_011046841, Unigene56688\_Sample\_011046841, Unigene50912\_Sample\_011046841, Unigene15865\_Sample\_011046841, Unigene54555\_Sample\_011046841, Unigene4915\_Sample\_011046841, Unigene57062\_Sample\_011046841, Unigene53170\_Sample\_011046841, Unigene48342\_Sample\_011046841, Unigene17752\_Sample\_011046841, Unigene46262\_Sample\_011046841, Unigene18725\_Sample\_011046841, Unigene58584\_Sample\_011046841, Unigene56599\_Sample\_011046841, Unigene58480\_Sample\_011046841, Unigene50798\_Sample\_011046841, Unigene28537\_Sample\_011046841, Unigene59512\_Sample\_011046841, Unigene51307\_Sample\_011046841, Unigene56584\_Sample\_011046841, Unigene13430\_Sample\_011046841, Unigene58403\_Sample\_011046841, Unigene47837\_Sample\_011046841, Unigene49501\_Sample\_011046841, Unigene12703\_Sample\_011046841, Unigene60375\_Sample\_011046841, Unigene57039\_Sample\_011046841, Unigene53098\_Sample\_011046841, Unigene12331\_Sample\_011046841, Unigene52381\_Sample\_011046841, Unigene33607\_Sample\_011046841, Unigene35008\_Sample\_011046841, Unigene50926\_Sample\_011046841, Unigene54549\_Sample\_011046841, Unigene58374\_Sample\_011046841, Unigene13817\_Sample\_011046841, Unigene9271\_Sample\_011046841, Unigene54790\_Sample\_011046841, Unigene49337\_Sample\_011046841, Unigene60872\_Sample\_011046841, Unigene42633\_Sample\_011046841, Unigene8851\_Sample\_011046841, Unigene38586\_Sample\_011046841, Unigene41393\_Sample\_011046841, Unigene44479\_Sample\_011046841, Unigene22323\_Sample\_011046841, Unigene29101\_Sample\_011046841, Unigene51803\_Sample\_011046841, Unigene44009\_Sample\_011046841, Unigene50996\_Sample\_011046841, Unigene41001\_Sample\_011046841, Unigene51264\_Sample\_011046841, Unigene45662\_Sample\_011046841, Unigene60245\_Sample\_011046841, Unigene49126\_Sample\_011046841, Unigene46791\_Sample\_011046841, Unigene45183\_Sample\_011046841, Unigene8538\_Sample\_011046841, Unigene57100\_Sample\_011046841, Unigene48637\_Sample\_011046841, Unigene57158\_Sample\_011046841, Unigene47025\_Sample\_011046841, Unigene56795\_Sample\_011046841, Unigene57713\_Sample\_011046841, Unigene45539\_Sample\_011046841, Unigene28246\_Sample\_011046841, Unigene54574\_Sample\_011046841, Unigene59114\_Sample\_011046841, Unigene60723\_Sample\_011046841, Unigene21907\_Sample\_011046841, Unigene3496\_Sample\_011046841, Unigene52788\_Sample\_011046841, Unigene60809\_Sample\_011046841, Unigene60906\_Sample\_011046841, Unigene13079\_Sample\_011046841, Unigene55856\_Sample\_011046841, Unigene36363\_Sample\_011046841, Unigene22245\_Sample\_011046841, Unigene8165\_Sample\_011046841, Unigene775\_Sample\_011046841, Unigene60701\_Sample\_011046841, Unigene60494\_Sample\_011046841, Unigene34152\_Sample\_011046841, Unigene33130\_Sample\_011046841, Unigene8392\_Sample\_011046841, Unigene50396\_Sample\_011046841, Unigene39967\_Sample\_011046841, Unigene58138\_Sample\_011046841, Unigene30153\_Sample\_011046841, Unigene36469\_Sample\_011046841, Unigene36891\_Sample\_011046841, Unigene49650\_Sample\_011046841, Unigene38675\_Sample\_011046841, Unigene30530\_Sample\_011046841, Unigene4871\_Sample\_011046841, Unigene51077\_Sample\_011046841, Unigene13249\_Sample\_011046841, Unigene59021\_Sample\_011046841, Unigene59496\_Sample\_011046841, Unigene43875\_Sample\_011046841, Unigene60309\_Sample\_011046841, Unigene10640\_Sample\_011046841, Unigene53714\_Sample\_011046841, Unigene55702\_Sample\_011046841, Unigene8034\_Sample\_011046841, Unigene42812\_Sample\_011046841, Unigene24335\_Sample\_011046841, Unigene45754\_Sample\_011046841, Unigene11726\_Sample\_011046841, Unigene24615\_Sample\_011046841, Unigene58014\_Sample\_011046841, Unigene13517\_Sample\_011046841, Unigene11786\_Sample\_011046841, Unigene58839\_Sample\_011046841, Unigene50653\_Sample\_011046841, Unigene60381\_Sample\_011046841, Unigene12306\_Sample\_011046841, Unigene58808\_Sample\_011046841, Unigene60900\_Sample\_011046841, Unigene58285\_Sample\_011046841, Unigene51569\_Sample\_011046841, Unigene17258\_Sample\_011046841, Unigene38616\_Sample\_011046841, Unigene12056\_Sample\_011046841, Unigene52808\_Sample\_011046841, Unigene40697\_Sample\_011046841, Unigene59535\_Sample\_011046841, Unigene58929\_Sample\_011046841, Unigene49671\_Sample\_011046841, Unigene58917\_Sample\_011046841, Unigene48334\_Sample\_011046841, Unigene40287\_Sample\_011046841, Unigene7011\_Sample\_011046841, Unigene17531\_Sample\_011046841, Unigene53072\_Sample\_011046841, Unigene13902\_Sample\_011046841, Unigene55781\_Sample\_011046841, Unigene12212\_Sample\_011046841, Unigene59447\_Sample\_011046841, Unigene40147\_Sample\_011046841, Unigene60478\_Sample\_011046841, Unigene8243\_Sample\_011046841, Unigene1952\_Sample\_011046841, Unigene55302\_Sample\_011046841, Unigene39787\_Sample\_011046841, Unigene13719\_Sample\_011046841, Unigene57000\_Sample\_011046841, Unigene27618\_Sample\_011046841, Unigene48020\_Sample\_011046841, Unigene49724\_Sample\_011046841, Unigene40258\_Sample\_011046841, Unigene47610\_Sample\_011046841, Unigene60825\_Sample\_011046841, Unigene52688\_Sample\_011046841, Unigene9219\_Sample\_011046841, Unigene58210\_Sample\_011046841, Unigene25573\_Sample\_011046841, Unigene21688\_Sample\_011046841, Unigene1887\_Sample\_011046841, Unigene59494\_Sample\_011046841, Unigene28781\_Sample\_011046841, Unigene50555\_Sample\_011046841, Unigene55792\_Sample\_011046841, Unigene48473\_Sample\_011046841, Unigene60919\_Sample\_011046841, Unigene47368\_Sample\_011046841, Unigene14355\_Sample\_011046841, Unigene59891\_Sample\_011046841, Unigene44243\_Sample\_011046841, Unigene59514\_Sample\_011046841, Unigene10327\_Sample\_011046841, Unigene16372\_Sample\_011046841, Unigene4523\_Sample\_011046841, Unigene52249\_Sample\_011046841, Unigene12586\_Sample\_011046841, Unigene53037\_Sample\_011046841, Unigene10930\_Sample\_011046841, Unigene51110\_Sample\_011046841, Unigene38373\_Sample\_011046841, Unigene2610\_Sample\_011046841, Unigene55196\_Sample\_011046841, Unigene47125\_Sample\_011046841, Unigene56391\_Sample\_011046841, Unigene44438\_Sample\_011046841, Unigene39200\_Sample\_011046841, Unigene57239\_Sample\_011046841, Unigene30939\_Sample\_011046841, Unigene11099\_Sample\_011046841, Unigene3412\_Sample\_011046841, Unigene55900\_Sample\_011046841, Unigene60847\_Sample\_011046841, Unigene57610\_Sample\_011046841, Unigene11478\_Sample\_011046841, Unigene59492\_Sample\_011046841, Unigene6946\_Sample\_011046841, Unigene52695\_Sample\_011046841, Unigene47981\_Sample\_011046841, Unigene31905\_Sample\_011046841, Unigene44734\_Sample\_011046841, Unigene44880\_Sample\_011046841, Unigene49633\_Sample\_011046841, Unigene57929\_Sample\_011046841, Unigene5382\_Sample\_011046841, Unigene45873\_Sample\_011046841, Unigene2532\_Sample\_011046841, Unigene60714\_Sample\_011046841, Unigene22216\_Sample\_011046841, Unigene2325\_Sample\_011046841, Unigene46198\_Sample\_011046841, Unigene8480\_Sample\_011046841, Unigene5054\_Sample\_011046841, Unigene30681\_Sample\_011046841, Unigene11939\_Sample\_011046841, Unigene9067\_Sample\_011046841, Unigene60916\_Sample\_011046841, Unigene3946\_Sample\_011046841, Unigene58114\_Sample\_011046841, Unigene31129\_Sample\_011046841, Unigene20702\_Sample\_011046841, Unigene48072\_Sample\_011046841, Unigene47158\_Sample\_011046841, Unigene37278\_Sample\_011046841, Unigene58649\_Sample\_011046841, Unigene45885\_Sample\_011046841, Unigene25961\_Sample\_011046841, Unigene58518\_Sample\_011046841, Unigene39552\_Sample\_011046841, Unigene58365\_Sample\_011046841, Unigene11053\_Sample\_011046841, Unigene57806\_Sample\_011046841, Unigene8229\_Sample\_011046841, Unigene36824\_Sample\_011046841, Unigene55779\_Sample\_011046841, Unigene39209\_Sample\_011046841, Unigene46688\_Sample\_011046841, Unigene24979\_Sample\_011046841, Unigene58343\_Sample\_011046841, Unigene11846\_Sample\_011046841, Unigene18715\_Sample\_011046841, Unigene12324\_Sample\_011046841, Unigene57425\_Sample\_011046841, Unigene59637\_Sample\_011046841, Unigene2861\_Sample\_011046841, Unigene59587\_Sample\_011046841, Unigene43651\_Sample\_011046841, Unigene8641\_Sample\_011046841, Unigene31705\_Sample\_011046841, Unigene26928\_Sample\_011046841, Unigene26904\_Sample\_011046841, Unigene56389\_Sample\_011046841, Unigene28403\_Sample\_011046841, Unigene31534\_Sample\_011046841, Unigene44939\_Sample\_011046841, Unigene50902\_Sample\_011046841, Unigene42734\_Sample\_011046841, Unigene55247\_Sample\_011046841, Unigene24860\_Sample\_011046841, Unigene12957\_Sample\_011046841, Unigene41980\_Sample\_011046841, Unigene7072\_Sample\_011046841, Unigene47555\_Sample\_011046841, Unigene58564\_Sample\_011046841, Unigene42735\_Sample\_011046841, Unigene46263\_Sample\_011046841, Unigene51429\_Sample\_011046841, Unigene12412\_Sample\_011046841, Unigene56559\_Sample\_011046841, Unigene42749\_Sample\_011046841, Unigene28643\_Sample\_011046841, Unigene47871\_Sample\_011046841, Unigene30649\_Sample\_011046841, Unigene58989\_Sample\_011046841, Unigene38930\_Sample\_011046841, Unigene36452\_Sample\_011046841, Unigene20596\_Sample\_011046841, Unigene55077\_Sample\_011046841, Unigene56726\_Sample\_011046841, Unigene55499\_Sample\_011046841, Unigene60217\_Sample\_011046841, Unigene59560\_Sample\_011046841, Unigene48375\_Sample\_011046841, Unigene11168\_Sample\_011046841, Unigene251\_Sample\_011046841, Unigene56807\_Sample\_011046841, Unigene641\_Sample\_011046841 |
| nuclear lumen | Unigene11804\_Sample\_011046841, Unigene19912\_Sample\_011046841, Unigene57614\_Sample\_011046841, Unigene39261\_Sample\_011046841, Unigene713\_Sample\_011046841, Unigene57972\_Sample\_011046841, Unigene40921\_Sample\_011046841, Unigene34629\_Sample\_011046841, Unigene55221\_Sample\_011046841, Unigene57770\_Sample\_011046841, Unigene29630\_Sample\_011046841, Unigene757\_Sample\_011046841, Unigene38304\_Sample\_011046841, Unigene54668\_Sample\_011046841, Unigene52375\_Sample\_011046841, Unigene15270\_Sample\_011046841, Unigene52866\_Sample\_011046841, Unigene34268\_Sample\_011046841, Unigene59633\_Sample\_011046841, Unigene48415\_Sample\_011046841, Unigene39074\_Sample\_011046841, Unigene9459\_Sample\_011046841, Unigene39621\_Sample\_011046841, Unigene55254\_Sample\_011046841, Unigene41700\_Sample\_011046841, Unigene33973\_Sample\_011046841, Unigene54454\_Sample\_011046841, Unigene50213\_Sample\_011046841, Unigene9589\_Sample\_011046841, Unigene53179\_Sample\_011046841, Unigene27324\_Sample\_011046841, Unigene59972\_Sample\_011046841, Unigene11431\_Sample\_011046841, Unigene48260\_Sample\_011046841, Unigene58488\_Sample\_011046841, Unigene56395\_Sample\_011046841, Unigene12172\_Sample\_011046841, Unigene4684\_Sample\_011046841, Unigene50631\_Sample\_011046841, Unigene55156\_Sample\_011046841, Unigene57083\_Sample\_011046841, Unigene50764\_Sample\_011046841, Unigene58513\_Sample\_011046841, Unigene44447\_Sample\_011046841, Unigene58135\_Sample\_011046841, Unigene51481\_Sample\_011046841, Unigene59973\_Sample\_011046841, Unigene51688\_Sample\_011046841, Unigene2068\_Sample\_011046841, Unigene50734\_Sample\_011046841, Unigene55325\_Sample\_011046841, Unigene9011\_Sample\_011046841, Unigene12261\_Sample\_011046841, Unigene31264\_Sample\_011046841, Unigene26327\_Sample\_011046841, Unigene58726\_Sample\_011046841, Unigene42827\_Sample\_011046841, Unigene59022\_Sample\_011046841, Unigene59195\_Sample\_011046841, Unigene16692\_Sample\_011046841, Unigene14290\_Sample\_011046841, Unigene60842\_Sample\_011046841, Unigene36851\_Sample\_011046841, Unigene2066\_Sample\_011046841, Unigene11201\_Sample\_011046841, Unigene58907\_Sample\_011046841, Unigene44876\_Sample\_011046841, Unigene39807\_Sample\_011046841, Unigene38329\_Sample\_011046841, Unigene46283\_Sample\_011046841, Unigene59628\_Sample\_011046841, Unigene41574\_Sample\_011046841, Unigene47924\_Sample\_011046841, Unigene54061\_Sample\_011046841, Unigene31679\_Sample\_011046841, Unigene51504\_Sample\_011046841, Unigene54628\_Sample\_011046841, Unigene35914\_Sample\_011046841, Unigene52467\_Sample\_011046841, Unigene21535\_Sample\_011046841, Unigene60232\_Sample\_011046841, Unigene39712\_Sample\_011046841, Unigene45512\_Sample\_011046841, Unigene55667\_Sample\_011046841, Unigene13436\_Sample\_011046841, Unigene44587\_Sample\_011046841, Unigene11911\_Sample\_011046841, Unigene9912\_Sample\_011046841, Unigene29524\_Sample\_011046841, Unigene2398\_Sample\_011046841, Unigene10982\_Sample\_011046841, Unigene52869\_Sample\_011046841, Unigene45777\_Sample\_011046841, Unigene12623\_Sample\_011046841, Unigene55509\_Sample\_011046841, Unigene1129\_Sample\_011046841, Unigene55036\_Sample\_011046841, Unigene33884\_Sample\_011046841, Unigene19671\_Sample\_011046841, Unigene13748\_Sample\_011046841, Unigene10962\_Sample\_011046841, Unigene49168\_Sample\_011046841, Unigene13391\_Sample\_011046841, Unigene42378\_Sample\_011046841, Unigene10588\_Sample\_011046841, Unigene54996\_Sample\_011046841, Unigene57776\_Sample\_011046841, Unigene12822\_Sample\_011046841, Unigene46254\_Sample\_011046841, Unigene47868\_Sample\_011046841, Unigene48425\_Sample\_011046841, Unigene13722\_Sample\_011046841, Unigene58386\_Sample\_011046841, Unigene15720\_Sample\_011046841, Unigene15374\_Sample\_011046841, Unigene12390\_Sample\_011046841, Unigene49895\_Sample\_011046841, Unigene52396\_Sample\_011046841, Unigene37729\_Sample\_011046841, Unigene23416\_Sample\_011046841, Unigene19789\_Sample\_011046841, Unigene42399\_Sample\_011046841, Unigene59985\_Sample\_011046841, Unigene59727\_Sample\_011046841, Unigene16763\_Sample\_011046841, Unigene58496\_Sample\_011046841, Unigene57070\_Sample\_011046841, Unigene7778\_Sample\_011046841, Unigene13697\_Sample\_011046841, Unigene57438\_Sample\_011046841, Unigene57453\_Sample\_011046841, Unigene35691\_Sample\_011046841, Unigene51977\_Sample\_011046841, Unigene44567\_Sample\_011046841, Unigene50243\_Sample\_011046841, Unigene5462\_Sample\_011046841, Unigene57570\_Sample\_011046841, Unigene27612\_Sample\_011046841, Unigene59743\_Sample\_011046841, Unigene7905\_Sample\_011046841, Unigene44362\_Sample\_011046841, Unigene49099\_Sample\_011046841, Unigene12206\_Sample\_011046841, Unigene56629\_Sample\_011046841, Unigene22907\_Sample\_011046841, Unigene42229\_Sample\_011046841, Unigene48049\_Sample\_011046841, Unigene32138\_Sample\_011046841, Unigene13325\_Sample\_011046841, Unigene55183\_Sample\_011046841, Unigene16199\_Sample\_011046841, Unigene4847\_Sample\_011046841, Unigene60349\_Sample\_011046841, Unigene44749\_Sample\_011046841, Unigene49055\_Sample\_011046841, Unigene45403\_Sample\_011046841, Unigene34659\_Sample\_011046841, Unigene25307\_Sample\_011046841, Unigene8925\_Sample\_011046841, Unigene57773\_Sample\_011046841, Unigene23790\_Sample\_011046841, Unigene32309\_Sample\_011046841, Unigene37820\_Sample\_011046841, Unigene51468\_Sample\_011046841, Unigene58502\_Sample\_011046841, Unigene16650\_Sample\_011046841, Unigene45620\_Sample\_011046841, Unigene54248\_Sample\_011046841, Unigene7092\_Sample\_011046841, Unigene59136\_Sample\_011046841, Unigene41959\_Sample\_011046841, Unigene50990\_Sample\_011046841, Unigene49192\_Sample\_011046841, Unigene3810\_Sample\_011046841, Unigene48927\_Sample\_011046841, Unigene52322\_Sample\_011046841, Unigene58697\_Sample\_011046841, Unigene57384\_Sample\_011046841, Unigene50223\_Sample\_011046841, Unigene49921\_Sample\_011046841, Unigene15204\_Sample\_011046841, Unigene56688\_Sample\_011046841, Unigene50912\_Sample\_011046841, Unigene15865\_Sample\_011046841, Unigene54555\_Sample\_011046841, Unigene4915\_Sample\_011046841, Unigene57062\_Sample\_011046841, Unigene48342\_Sample\_011046841, Unigene17752\_Sample\_011046841, Unigene46262\_Sample\_011046841, Unigene18725\_Sample\_011046841, Unigene58584\_Sample\_011046841, Unigene56599\_Sample\_011046841, Unigene58480\_Sample\_011046841, Unigene50798\_Sample\_011046841, Unigene28537\_Sample\_011046841, Unigene59512\_Sample\_011046841, Unigene51307\_Sample\_011046841, Unigene56584\_Sample\_011046841, Unigene13430\_Sample\_011046841, Unigene58403\_Sample\_011046841, Unigene47837\_Sample\_011046841, Unigene49501\_Sample\_011046841, Unigene12703\_Sample\_011046841, Unigene60375\_Sample\_011046841, Unigene57039\_Sample\_011046841, Unigene53098\_Sample\_011046841, Unigene12331\_Sample\_011046841, Unigene52381\_Sample\_011046841, Unigene33607\_Sample\_011046841, Unigene35008\_Sample\_011046841, Unigene50926\_Sample\_011046841, Unigene54549\_Sample\_011046841, Unigene58374\_Sample\_011046841, Unigene9271\_Sample\_011046841, Unigene54790\_Sample\_011046841, Unigene49337\_Sample\_011046841, Unigene42633\_Sample\_011046841, Unigene8851\_Sample\_011046841, Unigene38586\_Sample\_011046841, Unigene41393\_Sample\_011046841, Unigene44479\_Sample\_011046841, Unigene22323\_Sample\_011046841, Unigene29101\_Sample\_011046841, Unigene51803\_Sample\_011046841, Unigene44009\_Sample\_011046841, Unigene51264\_Sample\_011046841, Unigene45662\_Sample\_011046841, Unigene60245\_Sample\_011046841, Unigene49126\_Sample\_011046841, Unigene46791\_Sample\_011046841, Unigene45183\_Sample\_011046841, Unigene57100\_Sample\_011046841, Unigene8538\_Sample\_011046841, Unigene48637\_Sample\_011046841, Unigene57158\_Sample\_011046841, Unigene47025\_Sample\_011046841, Unigene57713\_Sample\_011046841, Unigene45539\_Sample\_011046841, Unigene28246\_Sample\_011046841, Unigene54574\_Sample\_011046841, Unigene59114\_Sample\_011046841, Unigene60723\_Sample\_011046841, Unigene21907\_Sample\_011046841, Unigene52788\_Sample\_011046841, Unigene60809\_Sample\_011046841, Unigene60906\_Sample\_011046841, Unigene13079\_Sample\_011046841, Unigene55856\_Sample\_011046841, Unigene22245\_Sample\_011046841, Unigene8165\_Sample\_011046841, Unigene775\_Sample\_011046841, Unigene60494\_Sample\_011046841, Unigene60701\_Sample\_011046841, Unigene34152\_Sample\_011046841, Unigene33130\_Sample\_011046841, Unigene8392\_Sample\_011046841, Unigene50396\_Sample\_011046841, Unigene39967\_Sample\_011046841, Unigene58138\_Sample\_011046841, Unigene30153\_Sample\_011046841, Unigene36469\_Sample\_011046841, Unigene36891\_Sample\_011046841, Unigene38675\_Sample\_011046841, Unigene30530\_Sample\_011046841, Unigene4871\_Sample\_011046841, Unigene13249\_Sample\_011046841, Unigene59021\_Sample\_011046841, Unigene59496\_Sample\_011046841, Unigene43875\_Sample\_011046841, Unigene60309\_Sample\_011046841, Unigene10640\_Sample\_011046841, Unigene53714\_Sample\_011046841, Unigene55702\_Sample\_011046841, Unigene8034\_Sample\_011046841, Unigene11726\_Sample\_011046841, Unigene24615\_Sample\_011046841, Unigene58014\_Sample\_011046841, Unigene13517\_Sample\_011046841, Unigene11786\_Sample\_011046841, Unigene58839\_Sample\_011046841, Unigene50653\_Sample\_011046841, Unigene60381\_Sample\_011046841, Unigene12306\_Sample\_011046841, Unigene58808\_Sample\_011046841, Unigene60900\_Sample\_011046841, Unigene58285\_Sample\_011046841, Unigene51569\_Sample\_011046841, Unigene38616\_Sample\_011046841, Unigene12056\_Sample\_011046841, Unigene52808\_Sample\_011046841, Unigene59535\_Sample\_011046841, Unigene58929\_Sample\_011046841, Unigene49671\_Sample\_011046841, Unigene40287\_Sample\_011046841, Unigene7011\_Sample\_011046841, Unigene17531\_Sample\_011046841, Unigene53072\_Sample\_011046841, Unigene13902\_Sample\_011046841, Unigene55781\_Sample\_011046841, Unigene12212\_Sample\_011046841, Unigene59447\_Sample\_011046841, Unigene40147\_Sample\_011046841, Unigene60478\_Sample\_011046841, Unigene8243\_Sample\_011046841, Unigene1952\_Sample\_011046841, Unigene39787\_Sample\_011046841, Unigene55302\_Sample\_011046841, Unigene13719\_Sample\_011046841, Unigene27618\_Sample\_011046841, Unigene48020\_Sample\_011046841, Unigene49724\_Sample\_011046841, Unigene40258\_Sample\_011046841, Unigene47610\_Sample\_011046841, Unigene60825\_Sample\_011046841, Unigene52688\_Sample\_011046841, Unigene9219\_Sample\_011046841, Unigene58210\_Sample\_011046841, Unigene25573\_Sample\_011046841, Unigene21688\_Sample\_011046841, Unigene1887\_Sample\_011046841, Unigene59494\_Sample\_011046841, Unigene55792\_Sample\_011046841, Unigene48473\_Sample\_011046841, Unigene60919\_Sample\_011046841, Unigene47368\_Sample\_011046841, Unigene14355\_Sample\_011046841, Unigene59891\_Sample\_011046841, Unigene44243\_Sample\_011046841, Unigene59514\_Sample\_011046841, Unigene16372\_Sample\_011046841, Unigene4523\_Sample\_011046841, Unigene52249\_Sample\_011046841, Unigene12586\_Sample\_011046841, Unigene53037\_Sample\_011046841, Unigene51110\_Sample\_011046841, Unigene10930\_Sample\_011046841, Unigene38373\_Sample\_011046841, Unigene2610\_Sample\_011046841, Unigene55196\_Sample\_011046841, Unigene47125\_Sample\_011046841, Unigene56391\_Sample\_011046841, Unigene44438\_Sample\_011046841, Unigene39200\_Sample\_011046841, Unigene57239\_Sample\_011046841, Unigene11099\_Sample\_011046841, Unigene3412\_Sample\_011046841, Unigene60847\_Sample\_011046841, Unigene57610\_Sample\_011046841, Unigene59492\_Sample\_011046841, Unigene6946\_Sample\_011046841, Unigene52695\_Sample\_011046841, Unigene47981\_Sample\_011046841, Unigene31905\_Sample\_011046841, Unigene44734\_Sample\_011046841, Unigene44880\_Sample\_011046841, Unigene49633\_Sample\_011046841, Unigene57929\_Sample\_011046841, Unigene5382\_Sample\_011046841, Unigene45873\_Sample\_011046841, Unigene2532\_Sample\_011046841, Unigene60714\_Sample\_011046841, Unigene22216\_Sample\_011046841, Unigene2325\_Sample\_011046841, Unigene46198\_Sample\_011046841, Unigene8480\_Sample\_011046841, Unigene5054\_Sample\_011046841, Unigene11939\_Sample\_011046841, Unigene30681\_Sample\_011046841, Unigene9067\_Sample\_011046841, Unigene60916\_Sample\_011046841, Unigene3946\_Sample\_011046841, Unigene58114\_Sample\_011046841, Unigene31129\_Sample\_011046841, Unigene20702\_Sample\_011046841, Unigene48072\_Sample\_011046841, Unigene47158\_Sample\_011046841, Unigene58649\_Sample\_011046841, Unigene37278\_Sample\_011046841, Unigene45885\_Sample\_011046841, Unigene58518\_Sample\_011046841, Unigene58365\_Sample\_011046841, Unigene39552\_Sample\_011046841, Unigene11053\_Sample\_011046841, Unigene57806\_Sample\_011046841, Unigene8229\_Sample\_011046841, Unigene36824\_Sample\_011046841, Unigene55779\_Sample\_011046841, Unigene39209\_Sample\_011046841, Unigene58343\_Sample\_011046841, Unigene11846\_Sample\_011046841, Unigene24979\_Sample\_011046841, Unigene18715\_Sample\_011046841, Unigene12324\_Sample\_011046841, Unigene57425\_Sample\_011046841, Unigene59637\_Sample\_011046841, Unigene2861\_Sample\_011046841, Unigene59587\_Sample\_011046841, Unigene43651\_Sample\_011046841, Unigene8641\_Sample\_011046841, Unigene31705\_Sample\_011046841, Unigene26928\_Sample\_011046841, Unigene26904\_Sample\_011046841, Unigene56389\_Sample\_011046841, Unigene28403\_Sample\_011046841, Unigene44939\_Sample\_011046841, Unigene50902\_Sample\_011046841, Unigene42734\_Sample\_011046841, Unigene55247\_Sample\_011046841, Unigene24860\_Sample\_011046841, Unigene12957\_Sample\_011046841, Unigene41980\_Sample\_011046841, Unigene47555\_Sample\_011046841, Unigene7072\_Sample\_011046841, Unigene58564\_Sample\_011046841, Unigene46263\_Sample\_011046841, Unigene42735\_Sample\_011046841, Unigene51429\_Sample\_011046841, Unigene12412\_Sample\_011046841, Unigene42749\_Sample\_011046841, Unigene28643\_Sample\_011046841, Unigene47871\_Sample\_011046841, Unigene30649\_Sample\_011046841, Unigene58989\_Sample\_011046841, Unigene36452\_Sample\_011046841, Unigene20596\_Sample\_011046841, Unigene55077\_Sample\_011046841, Unigene55499\_Sample\_011046841, Unigene60217\_Sample\_011046841, Unigene59560\_Sample\_011046841, Unigene48375\_Sample\_011046841, Unigene11168\_Sample\_011046841, Unigene251\_Sample\_011046841, Unigene641\_Sample\_011046841, Unigene56807\_Sample\_011046841 |
| organelle | Unigene60957\_Sample\_011046841, Unigene28655\_Sample\_011046841, Unigene7801\_Sample\_011046841, Unigene12969\_Sample\_011046841, Unigene43386\_Sample\_011046841, Unigene34629\_Sample\_011046841, Unigene40117\_Sample\_011046841, Unigene56478\_Sample\_011046841, Unigene44774\_Sample\_011046841, Unigene57691\_Sample\_011046841, Unigene52866\_Sample\_011046841, Unigene10419\_Sample\_011046841, Unigene52937\_Sample\_011046841, Unigene57534\_Sample\_011046841, Unigene2263\_Sample\_011046841, Unigene24428\_Sample\_011046841, Unigene54851\_Sample\_011046841, Unigene9589\_Sample\_011046841, Unigene58001\_Sample\_011046841, Unigene21625\_Sample\_011046841, Unigene49534\_Sample\_011046841, Unigene56578\_Sample\_011046841, Unigene648\_Sample\_011046841, Unigene53661\_Sample\_011046841, Unigene30076\_Sample\_011046841, Unigene40873\_Sample\_011046841, Unigene29658\_Sample\_011046841, Unigene51638\_Sample\_011046841, Unigene26047\_Sample\_011046841, Unigene8772\_Sample\_011046841, Unigene57913\_Sample\_011046841, Unigene60433\_Sample\_011046841, Unigene14639\_Sample\_011046841, Unigene48121\_Sample\_011046841, Unigene6313\_Sample\_011046841, Unigene42542\_Sample\_011046841, Unigene57835\_Sample\_011046841, Unigene37663\_Sample\_011046841, Unigene10955\_Sample\_011046841, Unigene60153\_Sample\_011046841, Unigene56979\_Sample\_011046841, Unigene3264\_Sample\_011046841, Unigene34920\_Sample\_011046841, Unigene47691\_Sample\_011046841, Unigene49657\_Sample\_011046841, Unigene56375\_Sample\_011046841, Unigene59568\_Sample\_011046841, Unigene21912\_Sample\_011046841, Unigene42523\_Sample\_011046841, Unigene22636\_Sample\_011046841, Unigene2482\_Sample\_011046841, Unigene3880\_Sample\_011046841, Unigene41413\_Sample\_011046841, Unigene51160\_Sample\_011046841, Unigene39222\_Sample\_011046841, Unigene46690\_Sample\_011046841, Unigene6521\_Sample\_011046841, Unigene55981\_Sample\_011046841, Unigene50110\_Sample\_011046841, Unigene46283\_Sample\_011046841, Unigene56425\_Sample\_011046841, Unigene27013\_Sample\_011046841, Unigene15188\_Sample\_011046841, Unigene12251\_Sample\_011046841, Unigene48164\_Sample\_011046841, Unigene59023\_Sample\_011046841, Unigene36311\_Sample\_011046841, Unigene57919\_Sample\_011046841, Unigene34525\_Sample\_011046841, Unigene59077\_Sample\_011046841, Unigene58644\_Sample\_011046841, Unigene34745\_Sample\_011046841, Unigene12808\_Sample\_011046841, Unigene54910\_Sample\_011046841, Unigene55357\_Sample\_011046841, Unigene44835\_Sample\_011046841, Unigene13859\_Sample\_011046841, Unigene60797\_Sample\_011046841, Unigene56024\_Sample\_011046841, Unigene57216\_Sample\_011046841, Unigene41085\_Sample\_011046841, Unigene32616\_Sample\_011046841, Unigene60411\_Sample\_011046841, Unigene40677\_Sample\_011046841, Unigene12458\_Sample\_011046841, Unigene39654\_Sample\_011046841, Unigene7899\_Sample\_011046841, Unigene59696\_Sample\_011046841, Unigene56000\_Sample\_011046841, Unigene58779\_Sample\_011046841, Unigene469\_Sample\_011046841, Unigene12623\_Sample\_011046841, Unigene55509\_Sample\_011046841, Unigene813\_Sample\_011046841, Unigene35619\_Sample\_011046841, Unigene13070\_Sample\_011046841, Unigene49579\_Sample\_011046841, Unigene33441\_Sample\_011046841, Unigene60321\_Sample\_011046841, Unigene18575\_Sample\_011046841, Unigene29968\_Sample\_011046841, Unigene37222\_Sample\_011046841, Unigene12998\_Sample\_011046841, Unigene39783\_Sample\_011046841, Unigene12887\_Sample\_011046841, Unigene58892\_Sample\_011046841, Unigene51837\_Sample\_011046841, Unigene43716\_Sample\_011046841, Unigene52998\_Sample\_011046841, Unigene27774\_Sample\_011046841, Unigene26531\_Sample\_011046841, Unigene28897\_Sample\_011046841, Unigene48054\_Sample\_011046841, Unigene44838\_Sample\_011046841, Unigene12490\_Sample\_011046841, Unigene12390\_Sample\_011046841, Unigene16060\_Sample\_011046841, Unigene49895\_Sample\_011046841, Unigene27615\_Sample\_011046841, Unigene19789\_Sample\_011046841, Unigene40280\_Sample\_011046841, Unigene57802\_Sample\_011046841, Unigene16763\_Sample\_011046841, Unigene59236\_Sample\_011046841, Unigene51182\_Sample\_011046841, Unigene57438\_Sample\_011046841, Unigene55707\_Sample\_011046841, Unigene25152\_Sample\_011046841, Unigene59084\_Sample\_011046841, Unigene34464\_Sample\_011046841, Unigene58048\_Sample\_011046841, Unigene51\_Sample\_011046841, Unigene47010\_Sample\_011046841, Unigene49099\_Sample\_011046841, Unigene58409\_Sample\_011046841, Unigene39289\_Sample\_011046841, Unigene60035\_Sample\_011046841, Unigene44749\_Sample\_011046841, Unigene36090\_Sample\_011046841, Unigene59178\_Sample\_011046841, Unigene59404\_Sample\_011046841, Unigene13397\_Sample\_011046841, Unigene57181\_Sample\_011046841, Unigene10027\_Sample\_011046841, Unigene37613\_Sample\_011046841, Unigene56883\_Sample\_011046841, Unigene23937\_Sample\_011046841, Unigene52611\_Sample\_011046841, Unigene1918\_Sample\_011046841, Unigene19179\_Sample\_011046841, Unigene29053\_Sample\_011046841, Unigene31420\_Sample\_011046841, Unigene58502\_Sample\_011046841, Unigene41959\_Sample\_011046841, Unigene21808\_Sample\_011046841, Unigene59380\_Sample\_011046841, Unigene13733\_Sample\_011046841, Unigene43223\_Sample\_011046841, Unigene13844\_Sample\_011046841, Unigene33186\_Sample\_011046841, Unigene44894\_Sample\_011046841, Unigene56688\_Sample\_011046841, Unigene13217\_Sample\_011046841, Unigene45340\_Sample\_011046841, Unigene50912\_Sample\_011046841, Unigene57062\_Sample\_011046841, Unigene60413\_Sample\_011046841, Unigene53170\_Sample\_011046841, Unigene17390\_Sample\_011046841, Unigene56209\_Sample\_011046841, Unigene46262\_Sample\_011046841, Unigene58466\_Sample\_011046841, Unigene50798\_Sample\_011046841, Unigene15548\_Sample\_011046841, Unigene58106\_Sample\_011046841, Unigene23425\_Sample\_011046841, Unigene41346\_Sample\_011046841, Unigene38048\_Sample\_011046841, Unigene11040\_Sample\_011046841, Unigene6573\_Sample\_011046841, Unigene52118\_Sample\_011046841, Unigene6343\_Sample\_011046841, Unigene28681\_Sample\_011046841, Unigene19264\_Sample\_011046841, Unigene57998\_Sample\_011046841, Unigene46558\_Sample\_011046841, Unigene53098\_Sample\_011046841, Unigene40116\_Sample\_011046841, Unigene23250\_Sample\_011046841, Unigene13321\_Sample\_011046841, Unigene43791\_Sample\_011046841, Unigene41753\_Sample\_011046841, Unigene44913\_Sample\_011046841, Unigene31438\_Sample\_011046841, Unigene54790\_Sample\_011046841, Unigene60872\_Sample\_011046841, Unigene60865\_Sample\_011046841, Unigene10202\_Sample\_011046841, Unigene22323\_Sample\_011046841, Unigene57892\_Sample\_011046841, Unigene21228\_Sample\_011046841, Unigene3070\_Sample\_011046841, Unigene47783\_Sample\_011046841, Unigene60019\_Sample\_011046841, Unigene59445\_Sample\_011046841, Unigene55518\_Sample\_011046841, Unigene2521\_Sample\_011046841, Unigene59709\_Sample\_011046841, Unigene59685\_Sample\_011046841, Unigene36481\_Sample\_011046841, Unigene11570\_Sample\_011046841, Unigene9792\_Sample\_011046841, Unigene52298\_Sample\_011046841, Unigene16813\_Sample\_011046841, Unigene47927\_Sample\_011046841, Unigene50945\_Sample\_011046841, Unigene58655\_Sample\_011046841, Unigene56919\_Sample\_011046841, Unigene52033\_Sample\_011046841, Unigene45539\_Sample\_011046841, Unigene28246\_Sample\_011046841, Unigene6917\_Sample\_011046841, Unigene34524\_Sample\_011046841, Unigene40737\_Sample\_011046841, Unigene60926\_Sample\_011046841, Unigene13826\_Sample\_011046841, Unigene26696\_Sample\_011046841, Unigene54323\_Sample\_011046841, Unigene24650\_Sample\_011046841, Unigene11932\_Sample\_011046841, Unigene60948\_Sample\_011046841, Unigene58828\_Sample\_011046841, Unigene31773\_Sample\_011046841, Unigene60494\_Sample\_011046841, Unigene47134\_Sample\_011046841, Unigene28322\_Sample\_011046841, Unigene30162\_Sample\_011046841, Unigene60392\_Sample\_011046841, Unigene12142\_Sample\_011046841, Unigene52432\_Sample\_011046841, Unigene12082\_Sample\_011046841, Unigene58913\_Sample\_011046841, Unigene54214\_Sample\_011046841, Unigene60506\_Sample\_011046841, Unigene29827\_Sample\_011046841, Unigene28200\_Sample\_011046841, Unigene27305\_Sample\_011046841, Unigene13887\_Sample\_011046841, Unigene12748\_Sample\_011046841, Unigene9545\_Sample\_011046841, Unigene13249\_Sample\_011046841, Unigene51253\_Sample\_011046841, Unigene48708\_Sample\_011046841, Unigene54695\_Sample\_011046841, Unigene25596\_Sample\_011046841, Unigene43848\_Sample\_011046841, Unigene48921\_Sample\_011046841, Unigene56941\_Sample\_011046841, Unigene13517\_Sample\_011046841, Unigene11362\_Sample\_011046841, Unigene59033\_Sample\_011046841, Unigene51682\_Sample\_011046841, Unigene28964\_Sample\_011046841, Unigene35387\_Sample\_011046841, Unigene53532\_Sample\_011046841, Unigene54256\_Sample\_011046841, Unigene44021\_Sample\_011046841, Unigene60900\_Sample\_011046841, Unigene3956\_Sample\_011046841, Unigene6164\_Sample\_011046841, Unigene13944\_Sample\_011046841, Unigene54168\_Sample\_011046841, Unigene60469\_Sample\_011046841, Unigene39753\_Sample\_011046841, Unigene49671\_Sample\_011046841, Unigene5898\_Sample\_011046841, Unigene55898\_Sample\_011046841, Unigene60178\_Sample\_011046841, Unigene14532\_Sample\_011046841, Unigene40752\_Sample\_011046841, Unigene14914\_Sample\_011046841, Unigene53072\_Sample\_011046841, Unigene25924\_Sample\_011046841, Unigene12712\_Sample\_011046841, Unigene45568\_Sample\_011046841, Unigene59447\_Sample\_011046841, Unigene60385\_Sample\_011046841, Unigene8429\_Sample\_011046841, Unigene54689\_Sample\_011046841, Unigene57943\_Sample\_011046841, Unigene49720\_Sample\_011046841, Unigene55026\_Sample\_011046841, Unigene8070\_Sample\_011046841, Unigene727\_Sample\_011046841, Unigene56887\_Sample\_011046841, Unigene46419\_Sample\_011046841, Unigene13719\_Sample\_011046841, Unigene15482\_Sample\_011046841, Unigene59158\_Sample\_011046841, Unigene49410\_Sample\_011046841, Unigene59528\_Sample\_011046841, Unigene54246\_Sample\_011046841, Unigene32652\_Sample\_011046841, Unigene5603\_Sample\_011046841, Unigene53167\_Sample\_011046841, Unigene56916\_Sample\_011046841, Unigene18126\_Sample\_011046841, Unigene52226\_Sample\_011046841, Unigene45803\_Sample\_011046841, Unigene40258\_Sample\_011046841, Unigene5271\_Sample\_011046841, Unigene2189\_Sample\_011046841, Unigene38963\_Sample\_011046841, Unigene2472\_Sample\_011046841, Unigene15487\_Sample\_011046841, Unigene7826\_Sample\_011046841, Unigene33507\_Sample\_011046841, Unigene52688\_Sample\_011046841, Unigene5602\_Sample\_011046841, Unigene57322\_Sample\_011046841, Unigene40006\_Sample\_011046841, Unigene3486\_Sample\_011046841, Unigene50302\_Sample\_011046841, Unigene33846\_Sample\_011046841, Unigene9572\_Sample\_011046841, Unigene43918\_Sample\_011046841, Unigene60919\_Sample\_011046841, Unigene35589\_Sample\_011046841, Unigene14355\_Sample\_011046841, Unigene59891\_Sample\_011046841, Unigene48337\_Sample\_011046841, Unigene55198\_Sample\_011046841, Unigene4523\_Sample\_011046841, Unigene24401\_Sample\_011046841, Unigene46321\_Sample\_011046841, Unigene10941\_Sample\_011046841, Unigene16741\_Sample\_011046841, Unigene12416\_Sample\_011046841, Unigene51110\_Sample\_011046841, Unigene47680\_Sample\_011046841, Unigene55432\_Sample\_011046841, Unigene26055\_Sample\_011046841, Unigene10309\_Sample\_011046841, Unigene3431\_Sample\_011046841, Unigene56517\_Sample\_011046841, Unigene57374\_Sample\_011046841, Unigene48105\_Sample\_011046841, Unigene44438\_Sample\_011046841, Unigene26111\_Sample\_011046841, Unigene27907\_Sample\_011046841, Unigene1222\_Sample\_011046841, Unigene57239\_Sample\_011046841, Unigene48735\_Sample\_011046841, Unigene40059\_Sample\_011046841, Unigene55335\_Sample\_011046841, Unigene28455\_Sample\_011046841, Unigene6871\_Sample\_011046841, Unigene36149\_Sample\_011046841, Unigene13983\_Sample\_011046841, Unigene37028\_Sample\_011046841, Unigene20559\_Sample\_011046841, Unigene42899\_Sample\_011046841, Unigene41472\_Sample\_011046841, Unigene45059\_Sample\_011046841, Unigene8480\_Sample\_011046841, Unigene60199\_Sample\_011046841, Unigene10961\_Sample\_011046841, Unigene4828\_Sample\_011046841, Unigene9067\_Sample\_011046841, Unigene32169\_Sample\_011046841, Unigene53609\_Sample\_011046841, Unigene58365\_Sample\_011046841, Unigene11053\_Sample\_011046841, Unigene51167\_Sample\_011046841, Unigene57765\_Sample\_011046841, Unigene58725\_Sample\_011046841, Unigene46688\_Sample\_011046841, Unigene12837\_Sample\_011046841, Unigene58904\_Sample\_011046841, Unigene16003\_Sample\_011046841, Unigene52769\_Sample\_011046841, Unigene44366\_Sample\_011046841, Unigene28578\_Sample\_011046841, Unigene9141\_Sample\_011046841, Unigene25827\_Sample\_011046841, Unigene25248\_Sample\_011046841, Unigene42509\_Sample\_011046841, Unigene41327\_Sample\_011046841, Unigene44670\_Sample\_011046841, Unigene20491\_Sample\_011046841, Unigene9674\_Sample\_011046841, Unigene44817\_Sample\_011046841, Unigene55247\_Sample\_011046841, Unigene40898\_Sample\_011046841, Unigene29952\_Sample\_011046841, Unigene4344\_Sample\_011046841, Unigene6467\_Sample\_011046841, Unigene58258\_Sample\_011046841, Unigene29081\_Sample\_011046841, Unigene40729\_Sample\_011046841, Unigene52883\_Sample\_011046841, Unigene26288\_Sample\_011046841, Unigene56886\_Sample\_011046841, Unigene48957\_Sample\_011046841, Unigene23044\_Sample\_011046841, Unigene37947\_Sample\_011046841, Unigene40727\_Sample\_011046841, Unigene21618\_Sample\_011046841, Unigene44507\_Sample\_011046841, Unigene57579\_Sample\_011046841, Unigene54823\_Sample\_011046841, Unigene53095\_Sample\_011046841, Unigene21814\_Sample\_011046841, Unigene20596\_Sample\_011046841, Unigene58668\_Sample\_011046841, Unigene5030\_Sample\_011046841, Unigene38803\_Sample\_011046841, Unigene13916\_Sample\_011046841, Unigene9082\_Sample\_011046841, Unigene29602\_Sample\_011046841, Unigene13694\_Sample\_011046841, Unigene5335\_Sample\_011046841, Unigene15933\_Sample\_011046841, Unigene47818\_Sample\_011046841, Unigene53139\_Sample\_011046841, Unigene21462\_Sample\_011046841, Unigene16524\_Sample\_011046841, Unigene49834\_Sample\_011046841, Unigene9960\_Sample\_011046841, Unigene46207\_Sample\_011046841, Unigene3829\_Sample\_011046841, Unigene42503\_Sample\_011046841, Unigene60075\_Sample\_011046841, Unigene24100\_Sample\_011046841, Unigene52674\_Sample\_011046841, Unigene54668\_Sample\_011046841, Unigene13416\_Sample\_011046841, Unigene4630\_Sample\_011046841, Unigene52375\_Sample\_011046841, Unigene50998\_Sample\_011046841, Unigene34268\_Sample\_011046841, Unigene8488\_Sample\_011046841, Unigene32195\_Sample\_011046841, Unigene52627\_Sample\_011046841, Unigene44863\_Sample\_011046841, Unigene57161\_Sample\_011046841, Unigene21108\_Sample\_011046841, Unigene50213\_Sample\_011046841, Unigene40263\_Sample\_011046841, Unigene52003\_Sample\_011046841, Unigene53449\_Sample\_011046841, Unigene58437\_Sample\_011046841, Unigene9190\_Sample\_011046841, Unigene13584\_Sample\_011046841, Unigene57699\_Sample\_011046841, Unigene33889\_Sample\_011046841, Unigene52085\_Sample\_011046841, Unigene56395\_Sample\_011046841, Unigene60358\_Sample\_011046841, Unigene58810\_Sample\_011046841, Unigene37790\_Sample\_011046841, Unigene51706\_Sample\_011046841, Unigene54733\_Sample\_011046841, Unigene15846\_Sample\_011046841, Unigene40444\_Sample\_011046841, Unigene38662\_Sample\_011046841, Unigene12023\_Sample\_011046841, Unigene17383\_Sample\_011046841, Unigene58558\_Sample\_011046841, Unigene11639\_Sample\_011046841, Unigene19285\_Sample\_011046841, Unigene3975\_Sample\_011046841, Unigene30697\_Sample\_011046841, Unigene43044\_Sample\_011046841, Unigene60216\_Sample\_011046841, Unigene51481\_Sample\_011046841, Unigene43377\_Sample\_011046841, Unigene2068\_Sample\_011046841, Unigene10683\_Sample\_011046841, Unigene48571\_Sample\_011046841, Unigene45493\_Sample\_011046841, Unigene55844\_Sample\_011046841, Unigene5336\_Sample\_011046841, Unigene60597\_Sample\_011046841, Unigene32247\_Sample\_011046841, Unigene14290\_Sample\_011046841, Unigene58907\_Sample\_011046841, Unigene15515\_Sample\_011046841, Unigene39978\_Sample\_011046841, Unigene5747\_Sample\_011046841, Unigene60138\_Sample\_011046841, Unigene47625\_Sample\_011046841, Unigene58134\_Sample\_011046841, Unigene34228\_Sample\_011046841, Unigene53056\_Sample\_011046841, Unigene60269\_Sample\_011046841, Unigene38271\_Sample\_011046841, Unigene55807\_Sample\_011046841, Unigene2846\_Sample\_011046841, Unigene57949\_Sample\_011046841, Unigene44697\_Sample\_011046841, Unigene60485\_Sample\_011046841, Unigene8647\_Sample\_011046841, Unigene43504\_Sample\_011046841, Unigene38339\_Sample\_011046841, Unigene54656\_Sample\_011046841, Unigene58128\_Sample\_011046841, Unigene39295\_Sample\_011046841, Unigene58529\_Sample\_011046841, Unigene31985\_Sample\_011046841, Unigene60964\_Sample\_011046841, Unigene18442\_Sample\_011046841, Unigene32174\_Sample\_011046841, Unigene51471\_Sample\_011046841, Unigene32935\_Sample\_011046841, Unigene55667\_Sample\_011046841, Unigene52610\_Sample\_011046841, Unigene59378\_Sample\_011046841, Unigene16612\_Sample\_011046841, Unigene5529\_Sample\_011046841, Unigene11911\_Sample\_011046841, Unigene26120\_Sample\_011046841, Unigene47995\_Sample\_011046841, Unigene52869\_Sample\_011046841, Unigene49125\_Sample\_011046841, Unigene13151\_Sample\_011046841, Unigene32470\_Sample\_011046841, Unigene60189\_Sample\_011046841, Unigene53184\_Sample\_011046841, Unigene47864\_Sample\_011046841, Unigene8128\_Sample\_011046841, Unigene5318\_Sample\_011046841, Unigene33884\_Sample\_011046841, Unigene47008\_Sample\_011046841, Unigene56524\_Sample\_011046841, Unigene35592\_Sample\_011046841, Unigene12950\_Sample\_011046841, Unigene59448\_Sample\_011046841, Unigene58587\_Sample\_011046841, Unigene19352\_Sample\_011046841, Unigene301\_Sample\_011046841, Unigene60051\_Sample\_011046841, Unigene47464\_Sample\_011046841, Unigene16974\_Sample\_011046841, Unigene52717\_Sample\_011046841, Unigene35141\_Sample\_011046841, Unigene13214\_Sample\_011046841, Unigene57677\_Sample\_011046841, Unigene49287\_Sample\_011046841, Unigene55251\_Sample\_011046841, Unigene59214\_Sample\_011046841, Unigene39341\_Sample\_011046841, Unigene12984\_Sample\_011046841, Unigene53979\_Sample\_011046841, Unigene4616\_Sample\_011046841, Unigene60298\_Sample\_011046841, Unigene42731\_Sample\_011046841, Unigene38547\_Sample\_011046841, Unigene59809\_Sample\_011046841, Unigene31593\_Sample\_011046841, Unigene53447\_Sample\_011046841, Unigene60006\_Sample\_011046841, Unigene26192\_Sample\_011046841, Unigene30713\_Sample\_011046841, Unigene52396\_Sample\_011046841, Unigene7142\_Sample\_011046841, Unigene53425\_Sample\_011046841, Unigene13697\_Sample\_011046841, Unigene47099\_Sample\_011046841, Unigene60354\_Sample\_011046841, Unigene5866\_Sample\_011046841, Unigene28640\_Sample\_011046841, Unigene27612\_Sample\_011046841, Unigene58173\_Sample\_011046841, Unigene51201\_Sample\_011046841, Unigene43698\_Sample\_011046841, Unigene16375\_Sample\_011046841, Unigene3393\_Sample\_011046841, Unigene30898\_Sample\_011046841, Unigene54868\_Sample\_011046841, Unigene45395\_Sample\_011046841, Unigene55904\_Sample\_011046841, Unigene57575\_Sample\_011046841, Unigene25307\_Sample\_011046841, Unigene2085\_Sample\_011046841, Unigene55588\_Sample\_011046841, Unigene42958\_Sample\_011046841, Unigene12940\_Sample\_011046841, Unigene58936\_Sample\_011046841, Unigene31954\_Sample\_011046841, Unigene22285\_Sample\_011046841, Unigene55076\_Sample\_011046841, Unigene25954\_Sample\_011046841, Unigene49192\_Sample\_011046841, Unigene56212\_Sample\_011046841, Unigene48927\_Sample\_011046841, Unigene58697\_Sample\_011046841, Unigene35464\_Sample\_011046841, Unigene36235\_Sample\_011046841, Unigene49528\_Sample\_011046841, Unigene4242\_Sample\_011046841, Unigene2371\_Sample\_011046841, Unigene12370\_Sample\_011046841, Unigene17752\_Sample\_011046841, Unigene45593\_Sample\_011046841, Unigene48342\_Sample\_011046841, Unigene6733\_Sample\_011046841, Unigene9845\_Sample\_011046841, Unigene10041\_Sample\_011046841, Unigene56599\_Sample\_011046841, Unigene19226\_Sample\_011046841, Unigene57375\_Sample\_011046841, Unigene58746\_Sample\_011046841, Unigene9578\_Sample\_011046841, Unigene51307\_Sample\_011046841, Unigene21909\_Sample\_011046841, Unigene40466\_Sample\_011046841, Unigene60450\_Sample\_011046841, Unigene17321\_Sample\_011046841, Unigene60115\_Sample\_011046841, Unigene30734\_Sample\_011046841, Unigene35480\_Sample\_011046841, Unigene10699\_Sample\_011046841, Unigene31249\_Sample\_011046841, Unigene49697\_Sample\_011046841, Unigene39833\_Sample\_011046841, Unigene13213\_Sample\_011046841, Unigene47837\_Sample\_011046841, Unigene20812\_Sample\_011046841, Unigene56779\_Sample\_011046841, Unigene35722\_Sample\_011046841, Unigene57039\_Sample\_011046841, Unigene45801\_Sample\_011046841, Unigene9271\_Sample\_011046841, Unigene42633\_Sample\_011046841, Unigene8851\_Sample\_011046841, Unigene44479\_Sample\_011046841, Unigene24927\_Sample\_011046841, Unigene48267\_Sample\_011046841, Unigene40571\_Sample\_011046841, Unigene49772\_Sample\_011046841, Unigene19044\_Sample\_011046841, Unigene51928\_Sample\_011046841, Unigene53016\_Sample\_011046841, Unigene58354\_Sample\_011046841, Unigene53302\_Sample\_011046841, Unigene46011\_Sample\_011046841, Unigene60493\_Sample\_011046841, Unigene11998\_Sample\_011046841, Unigene52326\_Sample\_011046841, Unigene41388\_Sample\_011046841, Unigene49083\_Sample\_011046841, Unigene58224\_Sample\_011046841, Unigene8047\_Sample\_011046841, Unigene55511\_Sample\_011046841, Unigene60318\_Sample\_011046841, Unigene17067\_Sample\_011046841, Unigene6709\_Sample\_011046841, Unigene18596\_Sample\_011046841, Unigene39966\_Sample\_011046841, Unigene8401\_Sample\_011046841, Unigene55856\_Sample\_011046841, Unigene38701\_Sample\_011046841, Unigene54697\_Sample\_011046841, Unigene14576\_Sample\_011046841, Unigene1989\_Sample\_011046841, Unigene44849\_Sample\_011046841, Unigene5018\_Sample\_011046841, Unigene38750\_Sample\_011046841, Unigene54985\_Sample\_011046841, Unigene34152\_Sample\_011046841, Unigene10496\_Sample\_011046841, Unigene46184\_Sample\_011046841, Unigene37565\_Sample\_011046841, Unigene22614\_Sample\_011046841, Unigene16356\_Sample\_011046841, Unigene8924\_Sample\_011046841, Unigene54528\_Sample\_011046841, Unigene30530\_Sample\_011046841, Unigene27440\_Sample\_011046841, Unigene9087\_Sample\_011046841, Unigene59021\_Sample\_011046841, Unigene55702\_Sample\_011046841, Unigene8034\_Sample\_011046841, Unigene51174\_Sample\_011046841, Unigene18163\_Sample\_011046841, Unigene11726\_Sample\_011046841, Unigene55520\_Sample\_011046841, Unigene56863\_Sample\_011046841, Unigene2518\_Sample\_011046841, Unigene59922\_Sample\_011046841, Unigene56191\_Sample\_011046841, Unigene34824\_Sample\_011046841, Unigene13660\_Sample\_011046841, Unigene6153\_Sample\_011046841, Unigene40276\_Sample\_011046841, Unigene40296\_Sample\_011046841, Unigene51626\_Sample\_011046841, Unigene58043\_Sample\_011046841, Unigene40423\_Sample\_011046841, Unigene7206\_Sample\_011046841, Unigene21213\_Sample\_011046841, Unigene6497\_Sample\_011046841, Unigene9672\_Sample\_011046841, Unigene48334\_Sample\_011046841, Unigene39553\_Sample\_011046841, Unigene11871\_Sample\_011046841, Unigene55781\_Sample\_011046841, Unigene13902\_Sample\_011046841, Unigene9238\_Sample\_011046841, Unigene8251\_Sample\_011046841, Unigene40344\_Sample\_011046841, Unigene39787\_Sample\_011046841, Unigene55466\_Sample\_011046841, Unigene46515\_Sample\_011046841, Unigene10236\_Sample\_011046841, Unigene11093\_Sample\_011046841, Unigene58821\_Sample\_011046841, Unigene41884\_Sample\_011046841, Unigene59914\_Sample\_011046841, Unigene58431\_Sample\_011046841, Unigene48836\_Sample\_011046841, Unigene8590\_Sample\_011046841, Unigene51125\_Sample\_011046841, Unigene10077\_Sample\_011046841, Unigene60095\_Sample\_011046841, Unigene51100\_Sample\_011046841, Unigene48473\_Sample\_011046841, Unigene52532\_Sample\_011046841, Unigene58528\_Sample\_011046841, Unigene43294\_Sample\_011046841, Unigene59747\_Sample\_011046841, Unigene13669\_Sample\_011046841, Unigene52249\_Sample\_011046841, Unigene50207\_Sample\_011046841, Unigene3252\_Sample\_011046841, Unigene55131\_Sample\_011046841, Unigene6719\_Sample\_011046841, Unigene993\_Sample\_011046841, Unigene12118\_Sample\_011046841, Unigene56391\_Sample\_011046841, Unigene6590\_Sample\_011046841, Unigene57871\_Sample\_011046841, Unigene50141\_Sample\_011046841, Unigene43426\_Sample\_011046841, Unigene30939\_Sample\_011046841, Unigene56715\_Sample\_011046841, Unigene12223\_Sample\_011046841, Unigene23431\_Sample\_011046841, Unigene55900\_Sample\_011046841, Unigene60847\_Sample\_011046841, Unigene12700\_Sample\_011046841, Unigene58737\_Sample\_011046841, Unigene26403\_Sample\_011046841, Unigene10289\_Sample\_011046841, Unigene47959\_Sample\_011046841, Unigene21756\_Sample\_011046841, Unigene55700\_Sample\_011046841, Unigene19526\_Sample\_011046841, Unigene48589\_Sample\_011046841, Unigene31905\_Sample\_011046841, Unigene44880\_Sample\_011046841, Unigene4364\_Sample\_011046841, Unigene50296\_Sample\_011046841, Unigene57929\_Sample\_011046841, Unigene55995\_Sample\_011046841, Unigene32630\_Sample\_011046841, Unigene53653\_Sample\_011046841, Unigene24152\_Sample\_011046841, Unigene42559\_Sample\_011046841, Unigene52802\_Sample\_011046841, Unigene46198\_Sample\_011046841, Unigene42123\_Sample\_011046841, Unigene29953\_Sample\_011046841, Unigene40609\_Sample\_011046841, Unigene60958\_Sample\_011046841, Unigene60319\_Sample\_011046841, Unigene5054\_Sample\_011046841, Unigene58487\_Sample\_011046841, Unigene56437\_Sample\_011046841, Unigene53846\_Sample\_011046841, Unigene56027\_Sample\_011046841, Unigene58649\_Sample\_011046841, Unigene60689\_Sample\_011046841, Unigene16098\_Sample\_011046841, Unigene13367\_Sample\_011046841, Unigene35586\_Sample\_011046841, Unigene29345\_Sample\_011046841, Unigene8229\_Sample\_011046841, Unigene22077\_Sample\_011046841, Unigene40953\_Sample\_011046841, Unigene42368\_Sample\_011046841, Unigene9107\_Sample\_011046841, Unigene50660\_Sample\_011046841, Unigene18715\_Sample\_011046841, Unigene8931\_Sample\_011046841, Unigene53614\_Sample\_011046841, Unigene58238\_Sample\_011046841, Unigene19792\_Sample\_011046841, Unigene32509\_Sample\_011046841, Unigene8641\_Sample\_011046841, Unigene26928\_Sample\_011046841, Unigene18856\_Sample\_011046841, Unigene26183\_Sample\_011046841, Unigene48047\_Sample\_011046841, Unigene4497\_Sample\_011046841, Unigene6435\_Sample\_011046841, Unigene55799\_Sample\_011046841, Unigene11923\_Sample\_011046841, Unigene59946\_Sample\_011046841, Unigene41236\_Sample\_011046841, Unigene6502\_Sample\_011046841, Unigene55384\_Sample\_011046841, Unigene38874\_Sample\_011046841, Unigene43906\_Sample\_011046841, Unigene5150\_Sample\_011046841, Unigene10013\_Sample\_011046841, Unigene1748\_Sample\_011046841, Unigene57255\_Sample\_011046841, Unigene42218\_Sample\_011046841, Unigene58565\_Sample\_011046841, Unigene54217\_Sample\_011046841, Unigene55342\_Sample\_011046841, Unigene58511\_Sample\_011046841, Unigene56559\_Sample\_011046841, Unigene55379\_Sample\_011046841, Unigene4883\_Sample\_011046841, Unigene12871\_Sample\_011046841, Unigene57573\_Sample\_011046841, Unigene4666\_Sample\_011046841, Unigene2834\_Sample\_011046841, Unigene12527\_Sample\_011046841, Unigene58951\_Sample\_011046841, Unigene55809\_Sample\_011046841, Unigene44703\_Sample\_011046841, Unigene48375\_Sample\_011046841, Unigene57490\_Sample\_011046841, Unigene43509\_Sample\_011046841, Unigene19912\_Sample\_011046841, Unigene37440\_Sample\_011046841, Unigene40921\_Sample\_011046841, Unigene16777\_Sample\_011046841, Unigene55221\_Sample\_011046841, Unigene59493\_Sample\_011046841, Unigene42259\_Sample\_011046841, Unigene42751\_Sample\_011046841, Unigene56530\_Sample\_011046841, Unigene55226\_Sample\_011046841, Unigene55543\_Sample\_011046841, Unigene58571\_Sample\_011046841, Unigene37696\_Sample\_011046841, Unigene49359\_Sample\_011046841, Unigene48095\_Sample\_011046841, Unigene11284\_Sample\_011046841, Unigene11521\_Sample\_011046841, Unigene58763\_Sample\_011046841, Unigene59633\_Sample\_011046841, Unigene38993\_Sample\_011046841, Unigene9459\_Sample\_011046841, Unigene59478\_Sample\_011046841, Unigene44985\_Sample\_011046841, Unigene55254\_Sample\_011046841, Unigene33973\_Sample\_011046841, Unigene10187\_Sample\_011046841, Unigene47674\_Sample\_011046841, Unigene7516\_Sample\_011046841, Unigene45808\_Sample\_011046841, Unigene43064\_Sample\_011046841, Unigene54795\_Sample\_011046841, Unigene13688\_Sample\_011046841, Unigene11715\_Sample\_011046841, Unigene44551\_Sample\_011046841, Unigene46719\_Sample\_011046841, Unigene58827\_Sample\_011046841, Unigene29839\_Sample\_011046841, Unigene3507\_Sample\_011046841, Unigene45303\_Sample\_011046841, Unigene51374\_Sample\_011046841, Unigene8519\_Sample\_011046841, Unigene38143\_Sample\_011046841, Unigene52288\_Sample\_011046841, Unigene13879\_Sample\_011046841, Unigene60377\_Sample\_011046841, Unigene25319\_Sample\_011046841, Unigene49752\_Sample\_011046841, Unigene31264\_Sample\_011046841, Unigene55675\_Sample\_011046841, Unigene49014\_Sample\_011046841, Unigene59195\_Sample\_011046841, Unigene58924\_Sample\_011046841, Unigene8987\_Sample\_011046841, Unigene46366\_Sample\_011046841, Unigene23984\_Sample\_011046841, Unigene36851\_Sample\_011046841, Unigene9307\_Sample\_011046841, Unigene31416\_Sample\_011046841, Unigene39807\_Sample\_011046841, Unigene57947\_Sample\_011046841, Unigene27139\_Sample\_011046841, Unigene15136\_Sample\_011046841, Unigene2913\_Sample\_011046841, Unigene13256\_Sample\_011046841, Unigene983\_Sample\_011046841, Unigene7929\_Sample\_011046841, Unigene44730\_Sample\_011046841, Unigene45371\_Sample\_011046841, Unigene31679\_Sample\_011046841, Unigene21818\_Sample\_011046841, Unigene26119\_Sample\_011046841, Unigene19717\_Sample\_011046841, Unigene5264\_Sample\_011046841, Unigene43721\_Sample\_011046841, Unigene60486\_Sample\_011046841, Unigene56587\_Sample\_011046841, Unigene12115\_Sample\_011046841, Unigene25063\_Sample\_011046841, Unigene39762\_Sample\_011046841, Unigene25597\_Sample\_011046841, Unigene54165\_Sample\_011046841, Unigene21535\_Sample\_011046841, Unigene59379\_Sample\_011046841, Unigene45635\_Sample\_011046841, Unigene43456\_Sample\_011046841, Unigene7296\_Sample\_011046841, Unigene34414\_Sample\_011046841, Unigene51178\_Sample\_011046841, Unigene57118\_Sample\_011046841, Unigene49793\_Sample\_011046841, Unigene59791\_Sample\_011046841, Unigene58715\_Sample\_011046841, Unigene41859\_Sample\_011046841, Unigene18444\_Sample\_011046841, Unigene12762\_Sample\_011046841, Unigene47562\_Sample\_011046841, Unigene3034\_Sample\_011046841, Unigene23762\_Sample\_011046841, Unigene8330\_Sample\_011046841, Unigene33118\_Sample\_011046841, Unigene6097\_Sample\_011046841, Unigene58519\_Sample\_011046841, Unigene2398\_Sample\_011046841, Unigene55036\_Sample\_011046841, Unigene3881\_Sample\_011046841, Unigene42893\_Sample\_011046841, Unigene54979\_Sample\_011046841, Unigene13776\_Sample\_011046841, Unigene10632\_Sample\_011046841, Unigene29330\_Sample\_011046841, Unigene46653\_Sample\_011046841, Unigene27530\_Sample\_011046841, Unigene10962\_Sample\_011046841, Unigene35626\_Sample\_011046841, Unigene12145\_Sample\_011046841, Unigene33542\_Sample\_011046841, Unigene33587\_Sample\_011046841, Unigene43793\_Sample\_011046841, Unigene49258\_Sample\_011046841, Unigene57659\_Sample\_011046841, Unigene24493\_Sample\_011046841, Unigene52997\_Sample\_011046841, Unigene36585\_Sample\_011046841, Unigene47868\_Sample\_011046841, Unigene52077\_Sample\_011046841, Unigene11794\_Sample\_011046841, Unigene42159\_Sample\_011046841, Unigene56914\_Sample\_011046841, Unigene22760\_Sample\_011046841, Unigene44244\_Sample\_011046841, Unigene18426\_Sample\_011046841, Unigene59018\_Sample\_011046841, Unigene57070\_Sample\_011046841, Unigene48524\_Sample\_011046841, Unigene19943\_Sample\_011046841, Unigene59437\_Sample\_011046841, Unigene12152\_Sample\_011046841, Unigene44362\_Sample\_011046841, Unigene41392\_Sample\_011046841, Unigene60238\_Sample\_011046841, Unigene50285\_Sample\_011046841, Unigene9623\_Sample\_011046841, Unigene11716\_Sample\_011046841, Unigene19043\_Sample\_011046841, Unigene13011\_Sample\_011046841, Unigene60097\_Sample\_011046841, Unigene59473\_Sample\_011046841, Unigene3761\_Sample\_011046841, Unigene13325\_Sample\_011046841, Unigene23745\_Sample\_011046841, Unigene51627\_Sample\_011046841, Unigene22650\_Sample\_011046841, Unigene53534\_Sample\_011046841, Unigene3247\_Sample\_011046841, Unigene7693\_Sample\_011046841, Unigene59095\_Sample\_011046841, Unigene32028\_Sample\_011046841, Unigene12869\_Sample\_011046841, Unigene54962\_Sample\_011046841, Unigene58221\_Sample\_011046841, Unigene21520\_Sample\_011046841, Unigene57124\_Sample\_011046841, Unigene16650\_Sample\_011046841, Unigene53431\_Sample\_011046841, Unigene7092\_Sample\_011046841, Unigene6178\_Sample\_011046841, Unigene55921\_Sample\_011046841, Unigene52322\_Sample\_011046841, Unigene54191\_Sample\_011046841, Unigene2963\_Sample\_011046841, Unigene39991\_Sample\_011046841, Unigene59118\_Sample\_011046841, Unigene38603\_Sample\_011046841, Unigene4064\_Sample\_011046841, Unigene6052\_Sample\_011046841, Unigene43713\_Sample\_011046841, Unigene58480\_Sample\_011046841, Unigene59512\_Sample\_011046841, Unigene56611\_Sample\_011046841, Unigene43704\_Sample\_011046841, Unigene41992\_Sample\_011046841, Unigene1441\_Sample\_011046841, Unigene60284\_Sample\_011046841, Unigene59971\_Sample\_011046841, Unigene54829\_Sample\_011046841, Unigene29966\_Sample\_011046841, Unigene58403\_Sample\_011046841, Unigene23671\_Sample\_011046841, Unigene30267\_Sample\_011046841, Unigene60753\_Sample\_011046841, Unigene11922\_Sample\_011046841, Unigene49622\_Sample\_011046841, Unigene60783\_Sample\_011046841, Unigene42323\_Sample\_011046841, Unigene53054\_Sample\_011046841, Unigene1893\_Sample\_011046841, Unigene41393\_Sample\_011046841, Unigene29101\_Sample\_011046841, Unigene23154\_Sample\_011046841, Unigene39808\_Sample\_011046841, Unigene27921\_Sample\_011046841, Unigene50996\_Sample\_011046841, Unigene2762\_Sample\_011046841, Unigene55496\_Sample\_011046841, Unigene29653\_Sample\_011046841, Unigene59323\_Sample\_011046841, Unigene38522\_Sample\_011046841, Unigene56835\_Sample\_011046841, Unigene52652\_Sample\_011046841, Unigene17449\_Sample\_011046841, Unigene33400\_Sample\_011046841, Unigene49268\_Sample\_011046841, Unigene20487\_Sample\_011046841, Unigene22564\_Sample\_011046841, Unigene57158\_Sample\_011046841, Unigene16834\_Sample\_011046841, Unigene59441\_Sample\_011046841, Unigene53915\_Sample\_011046841, Unigene58825\_Sample\_011046841, Unigene36298\_Sample\_011046841, Unigene50495\_Sample\_011046841, Unigene60271\_Sample\_011046841, Unigene60968\_Sample\_011046841, Unigene11180\_Sample\_011046841, Unigene53333\_Sample\_011046841, Unigene33017\_Sample\_011046841, Unigene52832\_Sample\_011046841, Unigene18301\_Sample\_011046841, Unigene56413\_Sample\_011046841, Unigene36252\_Sample\_011046841, Unigene36677\_Sample\_011046841, Unigene59672\_Sample\_011046841, Unigene54607\_Sample\_011046841, Unigene13893\_Sample\_011046841, Unigene20344\_Sample\_011046841, Unigene35603\_Sample\_011046841, Unigene60309\_Sample\_011046841, Unigene58166\_Sample\_011046841, Unigene53714\_Sample\_011046841, Unigene21655\_Sample\_011046841, Unigene24335\_Sample\_011046841, Unigene56645\_Sample\_011046841, Unigene53563\_Sample\_011046841, Unigene13607\_Sample\_011046841, Unigene58082\_Sample\_011046841, Unigene37611\_Sample\_011046841, Unigene6377\_Sample\_011046841, Unigene48701\_Sample\_011046841, Unigene37595\_Sample\_011046841, Unigene41401\_Sample\_011046841, Unigene50653\_Sample\_011046841, Unigene47036\_Sample\_011046841, Unigene60381\_Sample\_011046841, Unigene44861\_Sample\_011046841, Unigene20302\_Sample\_011046841, Unigene50329\_Sample\_011046841, Unigene59530\_Sample\_011046841, Unigene33843\_Sample\_011046841, Unigene34881\_Sample\_011046841, Unigene59003\_Sample\_011046841, Unigene24582\_Sample\_011046841, Unigene13407\_Sample\_011046841, Unigene32077\_Sample\_011046841, Unigene40567\_Sample\_011046841, Unigene59535\_Sample\_011046841, Unigene24726\_Sample\_011046841, Unigene29466\_Sample\_011046841, Unigene2493\_Sample\_011046841, Unigene39630\_Sample\_011046841, Unigene60526\_Sample\_011046841, Unigene13431\_Sample\_011046841, Unigene52427\_Sample\_011046841, Unigene57784\_Sample\_011046841, Unigene54293\_Sample\_011046841, Unigene41644\_Sample\_011046841, Unigene56278\_Sample\_011046841, Unigene38078\_Sample\_011046841, Unigene1952\_Sample\_011046841, Unigene10358\_Sample\_011046841, Unigene40186\_Sample\_011046841, Unigene48020\_Sample\_011046841, Unigene5340\_Sample\_011046841, Unigene5783\_Sample\_011046841, Unigene35850\_Sample\_011046841, Unigene43076\_Sample\_011046841, Unigene57928\_Sample\_011046841, Unigene13636\_Sample\_011046841, Unigene3139\_Sample\_011046841, Unigene41676\_Sample\_011046841, Unigene43380\_Sample\_011046841, Unigene5733\_Sample\_011046841, Unigene59859\_Sample\_011046841, Unigene9668\_Sample\_011046841, Unigene55420\_Sample\_011046841, Unigene25573\_Sample\_011046841, Unigene54402\_Sample\_011046841, Unigene22741\_Sample\_011046841, Unigene28781\_Sample\_011046841, Unigene50555\_Sample\_011046841, Unigene15838\_Sample\_011046841, Unigene59545\_Sample\_011046841, Unigene56163\_Sample\_011046841, Unigene48091\_Sample\_011046841, Unigene55314\_Sample\_011046841, Unigene56276\_Sample\_011046841, Unigene39096\_Sample\_011046841, Unigene52822\_Sample\_011046841, Unigene53910\_Sample\_011046841, Unigene58132\_Sample\_011046841, Unigene60102\_Sample\_011046841, Unigene21132\_Sample\_011046841, Unigene59139\_Sample\_011046841, Unigene55618\_Sample\_011046841, Unigene16257\_Sample\_011046841, Unigene13244\_Sample\_011046841, Unigene60693\_Sample\_011046841, Unigene55196\_Sample\_011046841, Unigene6629\_Sample\_011046841, Unigene17735\_Sample\_011046841, Unigene57690\_Sample\_011046841, Unigene29847\_Sample\_011046841, Unigene5154\_Sample\_011046841, Unigene48086\_Sample\_011046841, Unigene57706\_Sample\_011046841, Unigene59492\_Sample\_011046841, Unigene44734\_Sample\_011046841, Unigene20619\_Sample\_011046841, Unigene29551\_Sample\_011046841, Unigene2415\_Sample\_011046841, Unigene30349\_Sample\_011046841, Unigene30950\_Sample\_011046841, Unigene2532\_Sample\_011046841, Unigene39766\_Sample\_011046841, Unigene31335\_Sample\_011046841, Unigene11029\_Sample\_011046841, Unigene22216\_Sample\_011046841, Unigene12560\_Sample\_011046841, Unigene250\_Sample\_011046841, Unigene40544\_Sample\_011046841, Unigene5951\_Sample\_011046841, Unigene37987\_Sample\_011046841, Unigene41694\_Sample\_011046841, Unigene30681\_Sample\_011046841, Unigene20157\_Sample\_011046841, Unigene11939\_Sample\_011046841, Unigene53420\_Sample\_011046841, Unigene37456\_Sample\_011046841, Unigene26329\_Sample\_011046841, Unigene52796\_Sample\_011046841, Unigene10226\_Sample\_011046841, Unigene51179\_Sample\_011046841, Unigene57806\_Sample\_011046841, Unigene53994\_Sample\_011046841, Unigene57215\_Sample\_011046841, Unigene58593\_Sample\_011046841, Unigene36816\_Sample\_011046841, Unigene12670\_Sample\_011046841, Unigene11846\_Sample\_011046841, Unigene52824\_Sample\_011046841, Unigene37037\_Sample\_011046841, Unigene59653\_Sample\_011046841, Unigene14799\_Sample\_011046841, Unigene59637\_Sample\_011046841, Unigene51802\_Sample\_011046841, Unigene2861\_Sample\_011046841, Unigene54407\_Sample\_011046841, Unigene50902\_Sample\_011046841, Unigene46773\_Sample\_011046841, Unigene55465\_Sample\_011046841, Unigene8962\_Sample\_011046841, Unigene54692\_Sample\_011046841, Unigene38644\_Sample\_011046841, Unigene42188\_Sample\_011046841, Unigene26954\_Sample\_011046841, Unigene56144\_Sample\_011046841, Unigene54969\_Sample\_011046841, Unigene34417\_Sample\_011046841, Unigene59718\_Sample\_011046841, Unigene50103\_Sample\_011046841, Unigene54112\_Sample\_011046841, Unigene59849\_Sample\_011046841, Unigene58564\_Sample\_011046841, Unigene55564\_Sample\_011046841, Unigene59460\_Sample\_011046841, Unigene7821\_Sample\_011046841, Unigene59369\_Sample\_011046841, Unigene51702\_Sample\_011046841, Unigene33316\_Sample\_011046841, Unigene38182\_Sample\_011046841, Unigene8713\_Sample\_011046841, Unigene39125\_Sample\_011046841, Unigene55660\_Sample\_011046841, Unigene5391\_Sample\_011046841, Unigene56726\_Sample\_011046841, Unigene57173\_Sample\_011046841, Unigene60378\_Sample\_011046841, Unigene59560\_Sample\_011046841, Unigene55544\_Sample\_011046841, Unigene27178\_Sample\_011046841, Unigene13773\_Sample\_011046841, Unigene49211\_Sample\_011046841, Unigene59678\_Sample\_011046841, Unigene60247\_Sample\_011046841, Unigene54791\_Sample\_011046841, Unigene33791\_Sample\_011046841, Unigene60665\_Sample\_011046841, Unigene47421\_Sample\_011046841, Unigene38304\_Sample\_011046841, Unigene22525\_Sample\_011046841, Unigene35184\_Sample\_011046841, Unigene51599\_Sample\_011046841, Unigene59826\_Sample\_011046841, Unigene29462\_Sample\_011046841, Unigene58192\_Sample\_011046841, Unigene11699\_Sample\_011046841, Unigene324\_Sample\_011046841, Unigene20298\_Sample\_011046841, Unigene54297\_Sample\_011046841, Unigene35931\_Sample\_011046841, Unigene55533\_Sample\_011046841, Unigene13766\_Sample\_011046841, Unigene49021\_Sample\_011046841, Unigene58659\_Sample\_011046841, Unigene58488\_Sample\_011046841, Unigene44504\_Sample\_011046841, Unigene48690\_Sample\_011046841, Unigene23972\_Sample\_011046841, Unigene49608\_Sample\_011046841, Unigene54240\_Sample\_011046841, Unigene4131\_Sample\_011046841, Unigene16329\_Sample\_011046841, Unigene54683\_Sample\_011046841, Unigene46030\_Sample\_011046841, Unigene57540\_Sample\_011046841, Unigene11465\_Sample\_011046841, Unigene10973\_Sample\_011046841, Unigene58135\_Sample\_011046841, Unigene46220\_Sample\_011046841, Unigene51688\_Sample\_011046841, Unigene40435\_Sample\_011046841, Unigene36154\_Sample\_011046841, Unigene41668\_Sample\_011046841, Unigene55325\_Sample\_011046841, Unigene12261\_Sample\_011046841, Unigene56719\_Sample\_011046841, Unigene16692\_Sample\_011046841, Unigene50915\_Sample\_011046841, Unigene39075\_Sample\_011046841, Unigene51716\_Sample\_011046841, Unigene30322\_Sample\_011046841, Unigene54373\_Sample\_011046841, Unigene50563\_Sample\_011046841, Unigene53640\_Sample\_011046841, Unigene56466\_Sample\_011046841, Unigene55474\_Sample\_011046841, Unigene51070\_Sample\_011046841, Unigene10517\_Sample\_011046841, Unigene20789\_Sample\_011046841, Unigene6432\_Sample\_011046841, Unigene33523\_Sample\_011046841, Unigene40742\_Sample\_011046841, Unigene52839\_Sample\_011046841, Unigene42293\_Sample\_011046841, Unigene55975\_Sample\_011046841, Unigene27486\_Sample\_011046841, Unigene59688\_Sample\_011046841, Unigene54625\_Sample\_011046841, Unigene35438\_Sample\_011046841, Unigene39712\_Sample\_011046841, Unigene30708\_Sample\_011046841, Unigene56520\_Sample\_011046841, Unigene10425\_Sample\_011046841, Unigene59585\_Sample\_011046841, Unigene57404\_Sample\_011046841, Unigene59926\_Sample\_011046841, Unigene44834\_Sample\_011046841, Unigene25498\_Sample\_011046841, Unigene5329\_Sample\_011046841, Unigene57861\_Sample\_011046841, Unigene24028\_Sample\_011046841, Unigene50893\_Sample\_011046841, Unigene42231\_Sample\_011046841, Unigene57832\_Sample\_011046841, Unigene45374\_Sample\_011046841, Unigene26878\_Sample\_011046841, Unigene41847\_Sample\_011046841, Unigene32474\_Sample\_011046841, Unigene26650\_Sample\_011046841, Unigene46058\_Sample\_011046841, Unigene22433\_Sample\_011046841, Unigene55311\_Sample\_011046841, Unigene18925\_Sample\_011046841, Unigene42681\_Sample\_011046841, Unigene16136\_Sample\_011046841, Unigene10567\_Sample\_011046841, Unigene13332\_Sample\_011046841, Unigene1885\_Sample\_011046841, Unigene45020\_Sample\_011046841, Unigene60490\_Sample\_011046841, Unigene60057\_Sample\_011046841, Unigene21838\_Sample\_011046841, Unigene7431\_Sample\_011046841, Unigene28976\_Sample\_011046841, Unigene32869\_Sample\_011046841, Unigene9274\_Sample\_011046841, Unigene48943\_Sample\_011046841, Unigene37462\_Sample\_011046841, Unigene38398\_Sample\_011046841, Unigene10522\_Sample\_011046841, Unigene46254\_Sample\_011046841, Unigene48425\_Sample\_011046841, Unigene13722\_Sample\_011046841, Unigene47309\_Sample\_011046841, Unigene8113\_Sample\_011046841, Unigene48287\_Sample\_011046841, Unigene33405\_Sample\_011046841, Unigene30413\_Sample\_011046841, Unigene40851\_Sample\_011046841, Unigene24161\_Sample\_011046841, Unigene6254\_Sample\_011046841, Unigene23416\_Sample\_011046841, Unigene42399\_Sample\_011046841, Unigene51301\_Sample\_011046841, Unigene13099\_Sample\_011046841, Unigene58804\_Sample\_011046841, Unigene21986\_Sample\_011046841, Unigene8384\_Sample\_011046841, Unigene51852\_Sample\_011046841, Unigene5462\_Sample\_011046841, Unigene57570\_Sample\_011046841, Unigene12384\_Sample\_011046841, Unigene19793\_Sample\_011046841, Unigene7905\_Sample\_011046841, Unigene21362\_Sample\_011046841, Unigene48867\_Sample\_011046841, Unigene56266\_Sample\_011046841, Unigene12206\_Sample\_011046841, Unigene60629\_Sample\_011046841, Unigene50414\_Sample\_011046841, Unigene59176\_Sample\_011046841, Unigene58974\_Sample\_011046841, Unigene57889\_Sample\_011046841, Unigene6327\_Sample\_011046841, Unigene12149\_Sample\_011046841, Unigene5311\_Sample\_011046841, Unigene7335\_Sample\_011046841, Unigene36791\_Sample\_011046841, Unigene57773\_Sample\_011046841, Unigene54218\_Sample\_011046841, Unigene30495\_Sample\_011046841, Unigene51468\_Sample\_011046841, Unigene59396\_Sample\_011046841, Unigene3810\_Sample\_011046841, Unigene55863\_Sample\_011046841, Unigene21962\_Sample\_011046841, Unigene49921\_Sample\_011046841, Unigene12521\_Sample\_011046841, Unigene51411\_Sample\_011046841, Unigene59900\_Sample\_011046841, Unigene23477\_Sample\_011046841, Unigene48689\_Sample\_011046841, Unigene58494\_Sample\_011046841, Unigene60197\_Sample\_011046841, Unigene53073\_Sample\_011046841, Unigene11419\_Sample\_011046841, Unigene43623\_Sample\_011046841, Unigene42576\_Sample\_011046841, Unigene30743\_Sample\_011046841, Unigene48530\_Sample\_011046841, Unigene21128\_Sample\_011046841, Unigene25029\_Sample\_011046841, Unigene53241\_Sample\_011046841, Unigene40800\_Sample\_011046841, Unigene7966\_Sample\_011046841, Unigene37042\_Sample\_011046841, Unigene1194\_Sample\_011046841, Unigene44944\_Sample\_011046841, Unigene105\_Sample\_011046841, Unigene25755\_Sample\_011046841, Unigene57130\_Sample\_011046841, Unigene60375\_Sample\_011046841, Unigene57009\_Sample\_011046841, Unigene47793\_Sample\_011046841, Unigene52176\_Sample\_011046841, Unigene52381\_Sample\_011046841, Unigene54902\_Sample\_011046841, Unigene50926\_Sample\_011046841, Unigene35285\_Sample\_011046841, Unigene31194\_Sample\_011046841, Unigene10438\_Sample\_011046841, Unigene13433\_Sample\_011046841, Unigene45472\_Sample\_011046841, Unigene34403\_Sample\_011046841, Unigene55715\_Sample\_011046841, Unigene13817\_Sample\_011046841, Unigene12431\_Sample\_011046841, Unigene44237\_Sample\_011046841, Unigene55546\_Sample\_011046841, Unigene45740\_Sample\_011046841, Unigene60914\_Sample\_011046841, Unigene59357\_Sample\_011046841, Unigene6752\_Sample\_011046841, Unigene56904\_Sample\_011046841, Unigene5867\_Sample\_011046841, Unigene1793\_Sample\_011046841, Unigene45485\_Sample\_011046841, Unigene57183\_Sample\_011046841, Unigene54187\_Sample\_011046841, Unigene18699\_Sample\_011046841, Unigene33899\_Sample\_011046841, Unigene31097\_Sample\_011046841, Unigene11526\_Sample\_011046841, Unigene23201\_Sample\_011046841, Unigene47956\_Sample\_011046841, Unigene5237\_Sample\_011046841, Unigene45183\_Sample\_011046841, Unigene45310\_Sample\_011046841, Unigene1520\_Sample\_011046841, Unigene46800\_Sample\_011046841, Unigene16198\_Sample\_011046841, Unigene38130\_Sample\_011046841, Unigene27997\_Sample\_011046841, Unigene57713\_Sample\_011046841, Unigene53027\_Sample\_011046841, Unigene60342\_Sample\_011046841, Unigene59114\_Sample\_011046841, Unigene60780\_Sample\_011046841, Unigene52976\_Sample\_011046841, Unigene52788\_Sample\_011046841, Unigene24438\_Sample\_011046841, Unigene38898\_Sample\_011046841, Unigene36363\_Sample\_011046841, Unigene53773\_Sample\_011046841, Unigene51956\_Sample\_011046841, Unigene37526\_Sample\_011046841, Unigene55021\_Sample\_011046841, Unigene60515\_Sample\_011046841, Unigene15341\_Sample\_011046841, Unigene10669\_Sample\_011046841, Unigene13297\_Sample\_011046841, Unigene56653\_Sample\_011046841, Unigene39967\_Sample\_011046841, Unigene30153\_Sample\_011046841, Unigene12692\_Sample\_011046841, Unigene3342\_Sample\_011046841, Unigene38675\_Sample\_011046841, Unigene59917\_Sample\_011046841, Unigene50644\_Sample\_011046841, Unigene13852\_Sample\_011046841, Unigene23479\_Sample\_011046841, Unigene59496\_Sample\_011046841, Unigene8954\_Sample\_011046841, Unigene51295\_Sample\_011046841, Unigene30309\_Sample\_011046841, Unigene50483\_Sample\_011046841, Unigene30484\_Sample\_011046841, Unigene38437\_Sample\_011046841, Unigene42144\_Sample\_011046841, Unigene58014\_Sample\_011046841, Unigene53972\_Sample\_011046841, Unigene5118\_Sample\_011046841, Unigene56254\_Sample\_011046841, Unigene51699\_Sample\_011046841, Unigene49113\_Sample\_011046841, Unigene57572\_Sample\_011046841, Unigene46974\_Sample\_011046841, Unigene53689\_Sample\_011046841, Unigene58808\_Sample\_011046841, Unigene57272\_Sample\_011046841, Unigene11858\_Sample\_011046841, Unigene10024\_Sample\_011046841, Unigene34891\_Sample\_011046841, Unigene31782\_Sample\_011046841, Unigene30044\_Sample\_011046841, Unigene57293\_Sample\_011046841, Unigene38616\_Sample\_011046841, Unigene48044\_Sample\_011046841, Unigene14638\_Sample\_011046841, Unigene32987\_Sample\_011046841, Unigene55461\_Sample\_011046841, Unigene48737\_Sample\_011046841, Unigene54955\_Sample\_011046841, Unigene55948\_Sample\_011046841, Unigene47460\_Sample\_011046841, Unigene54866\_Sample\_011046841, Unigene16069\_Sample\_011046841, Unigene19103\_Sample\_011046841, Unigene60478\_Sample\_011046841, Unigene57000\_Sample\_011046841, Unigene54473\_Sample\_011046841, Unigene33512\_Sample\_011046841, Unigene59816\_Sample\_011046841, Unigene60718\_Sample\_011046841, Unigene3890\_Sample\_011046841, Unigene37646\_Sample\_011046841, Unigene4889\_Sample\_011046841, Unigene49724\_Sample\_011046841, Unigene29201\_Sample\_011046841, Unigene47610\_Sample\_011046841, Unigene60825\_Sample\_011046841, Unigene60294\_Sample\_011046841, Unigene60764\_Sample\_011046841, Unigene35668\_Sample\_011046841, Unigene26464\_Sample\_011046841, Unigene59746\_Sample\_011046841, Unigene58367\_Sample\_011046841, Unigene1887\_Sample\_011046841, Unigene49992\_Sample\_011046841, Unigene17159\_Sample\_011046841, Unigene52110\_Sample\_011046841, Unigene55315\_Sample\_011046841, Unigene48499\_Sample\_011046841, Unigene48999\_Sample\_011046841, Unigene41088\_Sample\_011046841, Unigene35788\_Sample\_011046841, Unigene59514\_Sample\_011046841, Unigene60431\_Sample\_011046841, Unigene57635\_Sample\_011046841, Unigene58815\_Sample\_011046841, Unigene12983\_Sample\_011046841, Unigene54468\_Sample\_011046841, Unigene5343\_Sample\_011046841, Unigene35733\_Sample\_011046841, Unigene9600\_Sample\_011046841, Unigene58952\_Sample\_011046841, Unigene11695\_Sample\_011046841, Unigene47149\_Sample\_011046841, Unigene53520\_Sample\_011046841, Unigene45818\_Sample\_011046841, Unigene40215\_Sample\_011046841, Unigene32993\_Sample\_011046841, Unigene986\_Sample\_011046841, Unigene45985\_Sample\_011046841, Unigene11099\_Sample\_011046841, Unigene3412\_Sample\_011046841, Unigene53929\_Sample\_011046841, Unigene53097\_Sample\_011046841, Unigene49145\_Sample\_011046841, Unigene12487\_Sample\_011046841, Unigene52695\_Sample\_011046841, Unigene10745\_Sample\_011046841, Unigene28763\_Sample\_011046841, Unigene2570\_Sample\_011046841, Unigene48546\_Sample\_011046841, Unigene864\_Sample\_011046841, Unigene59458\_Sample\_011046841, Unigene57306\_Sample\_011046841, Unigene10844\_Sample\_011046841, Unigene7647\_Sample\_011046841, Unigene28880\_Sample\_011046841, Unigene10037\_Sample\_011046841, Unigene58318\_Sample\_011046841, Unigene54648\_Sample\_011046841, Unigene21246\_Sample\_011046841, Unigene31129\_Sample\_011046841, Unigene58114\_Sample\_011046841, Unigene60916\_Sample\_011046841, Unigene10881\_Sample\_011046841, Unigene24046\_Sample\_011046841, Unigene47158\_Sample\_011046841, Unigene54198\_Sample\_011046841, Unigene25961\_Sample\_011046841, Unigene59711\_Sample\_011046841, Unigene58518\_Sample\_011046841, Unigene19441\_Sample\_011046841, Unigene42326\_Sample\_011046841, Unigene17084\_Sample\_011046841, Unigene9259\_Sample\_011046841, Unigene37448\_Sample\_011046841, Unigene9774\_Sample\_011046841, Unigene56982\_Sample\_011046841, Unigene24979\_Sample\_011046841, Unigene57667\_Sample\_011046841, Unigene53062\_Sample\_011046841, Unigene34027\_Sample\_011046841, Unigene58031\_Sample\_011046841, Unigene55486\_Sample\_011046841, Unigene8850\_Sample\_011046841, Unigene59673\_Sample\_011046841, Unigene55068\_Sample\_011046841, Unigene59814\_Sample\_011046841, Unigene58919\_Sample\_011046841, Unigene56869\_Sample\_011046841, Unigene12446\_Sample\_011046841, Unigene60218\_Sample\_011046841, Unigene25703\_Sample\_011046841, Unigene51736\_Sample\_011046841, Unigene45951\_Sample\_011046841, Unigene9064\_Sample\_011046841, Unigene41828\_Sample\_011046841, Unigene5939\_Sample\_011046841, Unigene37683\_Sample\_011046841, Unigene12338\_Sample\_011046841, Unigene38011\_Sample\_011046841, Unigene45389\_Sample\_011046841, Unigene58895\_Sample\_011046841, Unigene39189\_Sample\_011046841, Unigene60405\_Sample\_011046841, Unigene12195\_Sample\_011046841, Unigene54941\_Sample\_011046841, Unigene7766\_Sample\_011046841, Unigene5730\_Sample\_011046841, Unigene56913\_Sample\_011046841, Unigene24706\_Sample\_011046841, Unigene58049\_Sample\_011046841, Unigene50919\_Sample\_011046841, Unigene55555\_Sample\_011046841, Unigene2174\_Sample\_011046841, Unigene53149\_Sample\_011046841, Unigene36679\_Sample\_011046841, Unigene55348\_Sample\_011046841, Unigene41178\_Sample\_011046841, Unigene24065\_Sample\_011046841, Unigene17671\_Sample\_011046841, Unigene58818\_Sample\_011046841, Unigene26069\_Sample\_011046841, Unigene59588\_Sample\_011046841, Unigene37777\_Sample\_011046841, Unigene59292\_Sample\_011046841, Unigene50662\_Sample\_011046841, Unigene21921\_Sample\_011046841, Unigene36020\_Sample\_011046841, Unigene58390\_Sample\_011046841, Unigene57188\_Sample\_011046841, Unigene33513\_Sample\_011046841, Unigene19767\_Sample\_011046841, Unigene49399\_Sample\_011046841, Unigene53172\_Sample\_011046841, Unigene25630\_Sample\_011046841, Unigene10283\_Sample\_011046841, Unigene17090\_Sample\_011046841, Unigene50487\_Sample\_011046841, Unigene4863\_Sample\_011046841, Unigene56807\_Sample\_011046841, Unigene46224\_Sample\_011046841, Unigene21855\_Sample\_011046841, Unigene60028\_Sample\_011046841, Unigene6085\_Sample\_011046841, Unigene42565\_Sample\_011046841, Unigene713\_Sample\_011046841, Unigene36915\_Sample\_011046841, Unigene13774\_Sample\_011046841, Unigene4741\_Sample\_011046841, Unigene5889\_Sample\_011046841, Unigene32563\_Sample\_011046841, Unigene60924\_Sample\_011046841, Unigene12346\_Sample\_011046841, Unigene59910\_Sample\_011046841, Unigene52673\_Sample\_011046841, Unigene2442\_Sample\_011046841, Unigene29723\_Sample\_011046841, Unigene10260\_Sample\_011046841, Unigene11120\_Sample\_011046841, Unigene25340\_Sample\_011046841, Unigene54333\_Sample\_011046841, Unigene41700\_Sample\_011046841, Unigene56661\_Sample\_011046841, Unigene47326\_Sample\_011046841, Unigene50062\_Sample\_011046841, Unigene55044\_Sample\_011046841, Unigene60282\_Sample\_011046841, Unigene32116\_Sample\_011046841, Unigene24326\_Sample\_011046841, Unigene59972\_Sample\_011046841, Unigene59449\_Sample\_011046841, Unigene1605\_Sample\_011046841, Unigene28131\_Sample\_011046841, Unigene60645\_Sample\_011046841, Unigene4256\_Sample\_011046841, Unigene5964\_Sample\_011046841, Unigene37357\_Sample\_011046841, Unigene5058\_Sample\_011046841, Unigene47723\_Sample\_011046841, Unigene57083\_Sample\_011046841, Unigene44447\_Sample\_011046841, Unigene59381\_Sample\_011046841, Unigene24586\_Sample\_011046841, Unigene60566\_Sample\_011046841, Unigene56821\_Sample\_011046841, Unigene42064\_Sample\_011046841, Unigene57207\_Sample\_011046841, Unigene50963\_Sample\_011046841, Unigene37225\_Sample\_011046841, Unigene20439\_Sample\_011046841, Unigene58164\_Sample\_011046841, Unigene58942\_Sample\_011046841, Unigene16520\_Sample\_011046841, Unigene4709\_Sample\_011046841, Unigene3904\_Sample\_011046841, Unigene28571\_Sample\_011046841, Unigene39537\_Sample\_011046841, Unigene41427\_Sample\_011046841, Unigene57730\_Sample\_011046841, Unigene14236\_Sample\_011046841, Unigene52967\_Sample\_011046841, Unigene12769\_Sample\_011046841, Unigene52685\_Sample\_011046841, Unigene52030\_Sample\_011046841, Unigene51419\_Sample\_011046841, Unigene12309\_Sample\_011046841, Unigene20715\_Sample\_011046841, Unigene56373\_Sample\_011046841, Unigene42917\_Sample\_011046841, Unigene58733\_Sample\_011046841, Unigene45514\_Sample\_011046841, Unigene41574\_Sample\_011046841, Unigene54061\_Sample\_011046841, Unigene59250\_Sample\_011046841, Unigene58858\_Sample\_011046841, Unigene8182\_Sample\_011046841, Unigene51041\_Sample\_011046841, Unigene45512\_Sample\_011046841, Unigene28196\_Sample\_011046841, Unigene34589\_Sample\_011046841, Unigene47634\_Sample\_011046841, Unigene31853\_Sample\_011046841, Unigene9912\_Sample\_011046841, Unigene23155\_Sample\_011046841, Unigene23742\_Sample\_011046841, Unigene57140\_Sample\_011046841, Unigene11000\_Sample\_011046841, Unigene57828\_Sample\_011046841, Unigene49932\_Sample\_011046841, Unigene59629\_Sample\_011046841, Unigene16146\_Sample\_011046841, Unigene22671\_Sample\_011046841, Unigene55606\_Sample\_011046841, Unigene41778\_Sample\_011046841, Unigene20893\_Sample\_011046841, Unigene13748\_Sample\_011046841, Unigene36888\_Sample\_011046841, Unigene11925\_Sample\_011046841, Unigene5195\_Sample\_011046841, Unigene43826\_Sample\_011046841, Unigene49168\_Sample\_011046841, Unigene13391\_Sample\_011046841, Unigene43028\_Sample\_011046841, Unigene25267\_Sample\_011046841, Unigene14396\_Sample\_011046841, Unigene58160\_Sample\_011046841, Unigene55449\_Sample\_011046841, Unigene56075\_Sample\_011046841, Unigene36850\_Sample\_011046841, Unigene60944\_Sample\_011046841, Unigene5203\_Sample\_011046841, Unigene5223\_Sample\_011046841, Unigene51962\_Sample\_011046841, Unigene54134\_Sample\_011046841, Unigene41848\_Sample\_011046841, Unigene3481\_Sample\_011046841, Unigene7366\_Sample\_011046841, Unigene13881\_Sample\_011046841, Unigene59985\_Sample\_011046841, Unigene4851\_Sample\_011046841, Unigene57435\_Sample\_011046841, Unigene58298\_Sample\_011046841, Unigene7778\_Sample\_011046841, Unigene12701\_Sample\_011046841, Unigene55403\_Sample\_011046841, Unigene46703\_Sample\_011046841, Unigene25098\_Sample\_011046841, Unigene40509\_Sample\_011046841, Unigene50911\_Sample\_011046841, Unigene42229\_Sample\_011046841, Unigene48049\_Sample\_011046841, Unigene32138\_Sample\_011046841, Unigene38146\_Sample\_011046841, Unigene4847\_Sample\_011046841, Unigene55263\_Sample\_011046841, Unigene2588\_Sample\_011046841, Unigene55402\_Sample\_011046841, Unigene58777\_Sample\_011046841, Unigene59085\_Sample\_011046841, Unigene38021\_Sample\_011046841, Unigene47448\_Sample\_011046841, Unigene60677\_Sample\_011046841, Unigene45431\_Sample\_011046841, Unigene32309\_Sample\_011046841, Unigene144\_Sample\_011046841, Unigene24221\_Sample\_011046841, Unigene37820\_Sample\_011046841, Unigene27727\_Sample\_011046841, Unigene54248\_Sample\_011046841, Unigene13768\_Sample\_011046841, Unigene31588\_Sample\_011046841, Unigene57384\_Sample\_011046841, Unigene4117\_Sample\_011046841, Unigene46363\_Sample\_011046841, Unigene36927\_Sample\_011046841, Unigene44249\_Sample\_011046841, Unigene24961\_Sample\_011046841, Unigene5099\_Sample\_011046841, Unigene24807\_Sample\_011046841, Unigene60016\_Sample\_011046841, Unigene58000\_Sample\_011046841, Unigene36922\_Sample\_011046841, Unigene17785\_Sample\_011046841, Unigene46697\_Sample\_011046841, Unigene11028\_Sample\_011046841, Unigene42967\_Sample\_011046841, Unigene58345\_Sample\_011046841, Unigene2534\_Sample\_011046841, Unigene53100\_Sample\_011046841, Unigene59622\_Sample\_011046841, Unigene54425\_Sample\_011046841, Unigene26679\_Sample\_011046841, Unigene53067\_Sample\_011046841, Unigene53691\_Sample\_011046841, Unigene41336\_Sample\_011046841, Unigene48540\_Sample\_011046841, Unigene36271\_Sample\_011046841, Unigene60049\_Sample\_011046841, Unigene44069\_Sample\_011046841, Unigene53884\_Sample\_011046841, Unigene55512\_Sample\_011046841, Unigene43941\_Sample\_011046841, Unigene58374\_Sample\_011046841, Unigene54437\_Sample\_011046841, Unigene50910\_Sample\_011046841, Unigene38784\_Sample\_011046841, Unigene38586\_Sample\_011046841, Unigene48764\_Sample\_011046841, Unigene45973\_Sample\_011046841, Unigene10336\_Sample\_011046841, Unigene44009\_Sample\_011046841, Unigene60746\_Sample\_011046841, Unigene39941\_Sample\_011046841, Unigene47908\_Sample\_011046841, Unigene54276\_Sample\_011046841, Unigene3527\_Sample\_011046841, Unigene51264\_Sample\_011046841, Unigene21701\_Sample\_011046841, Unigene56876\_Sample\_011046841, Unigene46791\_Sample\_011046841, Unigene13571\_Sample\_011046841, Unigene42062\_Sample\_011046841, Unigene1958\_Sample\_011046841, Unigene57100\_Sample\_011046841, Unigene59671\_Sample\_011046841, Unigene53565\_Sample\_011046841, Unigene13651\_Sample\_011046841, Unigene48637\_Sample\_011046841, Unigene48242\_Sample\_011046841, Unigene18385\_Sample\_011046841, Unigene21907\_Sample\_011046841, Unigene49725\_Sample\_011046841, Unigene19495\_Sample\_011046841, Unigene55252\_Sample\_011046841, Unigene38694\_Sample\_011046841, Unigene58627\_Sample\_011046841, Unigene49227\_Sample\_011046841, Unigene7789\_Sample\_011046841, Unigene60809\_Sample\_011046841, Unigene34380\_Sample\_011046841, Unigene8612\_Sample\_011046841, Unigene8165\_Sample\_011046841, Unigene45838\_Sample\_011046841, Unigene6901\_Sample\_011046841, Unigene24508\_Sample\_011046841, Unigene60701\_Sample\_011046841, Unigene34404\_Sample\_011046841, Unigene53741\_Sample\_011046841, Unigene46495\_Sample\_011046841, Unigene33130\_Sample\_011046841, Unigene8392\_Sample\_011046841, Unigene17370\_Sample\_011046841, Unigene55838\_Sample\_011046841, Unigene21063\_Sample\_011046841, Unigene45342\_Sample\_011046841, Unigene30385\_Sample\_011046841, Unigene4871\_Sample\_011046841, Unigene7303\_Sample\_011046841, Unigene51077\_Sample\_011046841, Unigene43875\_Sample\_011046841, Unigene54751\_Sample\_011046841, Unigene1181\_Sample\_011046841, Unigene53757\_Sample\_011046841, Unigene51431\_Sample\_011046841, Unigene58077\_Sample\_011046841, Unigene40807\_Sample\_011046841, Unigene58839\_Sample\_011046841, Unigene53327\_Sample\_011046841, Unigene60801\_Sample\_011046841, Unigene3901\_Sample\_011046841, Unigene11522\_Sample\_011046841, Unigene51282\_Sample\_011046841, Unigene28658\_Sample\_011046841, Unigene31664\_Sample\_011046841, Unigene51696\_Sample\_011046841, Unigene3363\_Sample\_011046841, Unigene56665\_Sample\_011046841, Unigene50611\_Sample\_011046841, Unigene48786\_Sample\_011046841, Unigene12056\_Sample\_011046841, Unigene56019\_Sample\_011046841, Unigene13514\_Sample\_011046841, Unigene60704\_Sample\_011046841, Unigene57429\_Sample\_011046841, Unigene42214\_Sample\_011046841, Unigene11878\_Sample\_011046841, Unigene41352\_Sample\_011046841, Unigene58917\_Sample\_011046841, Unigene23180\_Sample\_011046841, Unigene33111\_Sample\_011046841, Unigene9610\_Sample\_011046841, Unigene21917\_Sample\_011046841, Unigene9773\_Sample\_011046841, Unigene49925\_Sample\_011046841, Unigene58720\_Sample\_011046841, Unigene7446\_Sample\_011046841, Unigene42430\_Sample\_011046841, Unigene49022\_Sample\_011046841, Unigene59322\_Sample\_011046841, Unigene50313\_Sample\_011046841, Unigene41140\_Sample\_011046841, Unigene56358\_Sample\_011046841, Unigene21284\_Sample\_011046841, Unigene10934\_Sample\_011046841, Unigene44453\_Sample\_011046841, Unigene42697\_Sample\_011046841, Unigene40558\_Sample\_011046841, Unigene32540\_Sample\_011046841, Unigene54525\_Sample\_011046841, Unigene49928\_Sample\_011046841, Unigene22762\_Sample\_011046841, Unigene12081\_Sample\_011046841, Unigene21688\_Sample\_011046841, Unigene49290\_Sample\_011046841, Unigene34034\_Sample\_011046841, Unigene53313\_Sample\_011046841, Unigene58577\_Sample\_011046841, Unigene52744\_Sample\_011046841, Unigene19047\_Sample\_011046841, Unigene56534\_Sample\_011046841, Unigene5904\_Sample\_011046841, Unigene53843\_Sample\_011046841, Unigene45012\_Sample\_011046841, Unigene22255\_Sample\_011046841, Unigene57475\_Sample\_011046841, Unigene17219\_Sample\_011046841, Unigene54189\_Sample\_011046841, Unigene51879\_Sample\_011046841, Unigene15918\_Sample\_011046841, Unigene44895\_Sample\_011046841, Unigene47129\_Sample\_011046841, Unigene57298\_Sample\_011046841, Unigene41466\_Sample\_011046841, Unigene52901\_Sample\_011046841, Unigene45107\_Sample\_011046841, Unigene59984\_Sample\_011046841, Unigene46466\_Sample\_011046841, Unigene47508\_Sample\_011046841, Unigene39200\_Sample\_011046841, Unigene57410\_Sample\_011046841, Unigene8126\_Sample\_011046841, Unigene57950\_Sample\_011046841, Unigene52428\_Sample\_011046841, Unigene48526\_Sample\_011046841, Unigene36948\_Sample\_011046841, Unigene11787\_Sample\_011046841, Unigene47981\_Sample\_011046841, Unigene60131\_Sample\_011046841, Unigene60124\_Sample\_011046841, Unigene19204\_Sample\_011046841, Unigene58640\_Sample\_011046841, Unigene49615\_Sample\_011046841, Unigene56344\_Sample\_011046841, Unigene53602\_Sample\_011046841, Unigene47257\_Sample\_011046841, Unigene46603\_Sample\_011046841, Unigene22655\_Sample\_011046841, Unigene57531\_Sample\_011046841, Unigene15292\_Sample\_011046841, Unigene56706\_Sample\_011046841, Unigene3946\_Sample\_011046841, Unigene37278\_Sample\_011046841, Unigene60353\_Sample\_011046841, Unigene12562\_Sample\_011046841, Unigene4977\_Sample\_011046841, Unigene1012\_Sample\_011046841, Unigene19594\_Sample\_011046841, Unigene38082\_Sample\_011046841, Unigene50194\_Sample\_011046841, Unigene36824\_Sample\_011046841, Unigene11629\_Sample\_011046841, Unigene28864\_Sample\_011046841, Unigene59189\_Sample\_011046841, Unigene2226\_Sample\_011046841, Unigene13675\_Sample\_011046841, Unigene39209\_Sample\_011046841, Unigene11706\_Sample\_011046841, Unigene12324\_Sample\_011046841, Unigene57939\_Sample\_011046841, Unigene762\_Sample\_011046841, Unigene56460\_Sample\_011046841, Unigene51677\_Sample\_011046841, Unigene55205\_Sample\_011046841, Unigene1594\_Sample\_011046841, Unigene15880\_Sample\_011046841, Unigene26904\_Sample\_011046841, Unigene28403\_Sample\_011046841, Unigene2731\_Sample\_011046841, Unigene48094\_Sample\_011046841, Unigene45546\_Sample\_011046841, Unigene58947\_Sample\_011046841, Unigene59408\_Sample\_011046841, Unigene52565\_Sample\_011046841, Unigene48977\_Sample\_011046841, Unigene55772\_Sample\_011046841, Unigene49013\_Sample\_011046841, Unigene26856\_Sample\_011046841, Unigene5953\_Sample\_011046841, Unigene47220\_Sample\_011046841, Unigene54870\_Sample\_011046841, Unigene7181\_Sample\_011046841, Unigene52591\_Sample\_011046841, Unigene55957\_Sample\_011046841, Unigene11520\_Sample\_011046841, Unigene33391\_Sample\_011046841, Unigene50758\_Sample\_011046841, Unigene58475\_Sample\_011046841, Unigene13122\_Sample\_011046841, Unigene47555\_Sample\_011046841, Unigene11725\_Sample\_011046841, Unigene48807\_Sample\_011046841, Unigene52025\_Sample\_011046841, Unigene56714\_Sample\_011046841, Unigene42735\_Sample\_011046841, Unigene44544\_Sample\_011046841, Unigene37289\_Sample\_011046841, Unigene39062\_Sample\_011046841, Unigene32703\_Sample\_011046841, Unigene46290\_Sample\_011046841, Unigene47871\_Sample\_011046841, Unigene34443\_Sample\_011046841, Unigene13390\_Sample\_011046841, Unigene36452\_Sample\_011046841, Unigene55077\_Sample\_011046841, Unigene8489\_Sample\_011046841, Unigene53706\_Sample\_011046841, Unigene53297\_Sample\_011046841, Unigene39483\_Sample\_011046841, Unigene28184\_Sample\_011046841, Unigene11804\_Sample\_011046841, Unigene39261\_Sample\_011046841, Unigene59045\_Sample\_011046841, Unigene60449\_Sample\_011046841, Unigene40575\_Sample\_011046841, Unigene53262\_Sample\_011046841, Unigene57719\_Sample\_011046841, Unigene59068\_Sample\_011046841, Unigene757\_Sample\_011046841, Unigene44282\_Sample\_011046841, Unigene13645\_Sample\_011046841, Unigene46764\_Sample\_011046841, Unigene16995\_Sample\_011046841, Unigene53367\_Sample\_011046841, Unigene41294\_Sample\_011046841, Unigene39074\_Sample\_011046841, Unigene13245\_Sample\_011046841, Unigene6720\_Sample\_011046841, Unigene58432\_Sample\_011046841, Unigene3202\_Sample\_011046841, Unigene58663\_Sample\_011046841, Unigene43870\_Sample\_011046841, Unigene34039\_Sample\_011046841, Unigene20001\_Sample\_011046841, Unigene58062\_Sample\_011046841, Unigene12157\_Sample\_011046841, Unigene43216\_Sample\_011046841, Unigene12211\_Sample\_011046841, Unigene42552\_Sample\_011046841, Unigene48575\_Sample\_011046841, Unigene20448\_Sample\_011046841, Unigene32707\_Sample\_011046841, Unigene24930\_Sample\_011046841, Unigene49242\_Sample\_011046841, Unigene7069\_Sample\_011046841, Unigene35806\_Sample\_011046841, Unigene9753\_Sample\_011046841, Unigene58463\_Sample\_011046841, Unigene60698\_Sample\_011046841, Unigene31713\_Sample\_011046841, Unigene58768\_Sample\_011046841, Unigene45440\_Sample\_011046841, Unigene58513\_Sample\_011046841, Unigene40230\_Sample\_011046841, Unigene47780\_Sample\_011046841, Unigene1598\_Sample\_011046841, Unigene59973\_Sample\_011046841, Unigene32418\_Sample\_011046841, Unigene55610\_Sample\_011046841, Unigene50734\_Sample\_011046841, Unigene4978\_Sample\_011046841, Unigene246\_Sample\_011046841, Unigene11729\_Sample\_011046841, Unigene31026\_Sample\_011046841, Unigene58422\_Sample\_011046841, Unigene17921\_Sample\_011046841, Unigene12445\_Sample\_011046841, Unigene26327\_Sample\_011046841, Unigene58726\_Sample\_011046841, Unigene50242\_Sample\_011046841, Unigene58462\_Sample\_011046841, Unigene37665\_Sample\_011046841, Unigene36984\_Sample\_011046841, Unigene25101\_Sample\_011046841, Unigene2066\_Sample\_011046841, Unigene11201\_Sample\_011046841, Unigene11865\_Sample\_011046841, Unigene44876\_Sample\_011046841, Unigene43620\_Sample\_011046841, Unigene51997\_Sample\_011046841, Unigene38329\_Sample\_011046841, Unigene1662\_Sample\_011046841, Unigene58481\_Sample\_011046841, Unigene42127\_Sample\_011046841, Unigene7339\_Sample\_011046841, Unigene24751\_Sample\_011046841, Unigene44086\_Sample\_011046841, Unigene56547\_Sample\_011046841, Unigene34853\_Sample\_011046841, Unigene60232\_Sample\_011046841, Unigene5122\_Sample\_011046841, Unigene25547\_Sample\_011046841, Unigene23548\_Sample\_011046841, Unigene16073\_Sample\_011046841, Unigene33988\_Sample\_011046841, Unigene56822\_Sample\_011046841, Unigene49661\_Sample\_011046841, Unigene54286\_Sample\_011046841, Unigene52146\_Sample\_011046841, Unigene28662\_Sample\_011046841, Unigene49803\_Sample\_011046841, Unigene10982\_Sample\_011046841, Unigene45777\_Sample\_011046841, Unigene49919\_Sample\_011046841, Unigene25357\_Sample\_011046841, Unigene52079\_Sample\_011046841, Unigene52621\_Sample\_011046841, Unigene13890\_Sample\_011046841, Unigene47083\_Sample\_011046841, Unigene47478\_Sample\_011046841, Unigene50769\_Sample\_011046841, Unigene52812\_Sample\_011046841, Unigene7359\_Sample\_011046841, Unigene43654\_Sample\_011046841, Unigene47887\_Sample\_011046841, Unigene5737\_Sample\_011046841, Unigene13819\_Sample\_011046841, Unigene50842\_Sample\_011046841, Unigene8526\_Sample\_011046841, Unigene52542\_Sample\_011046841, Unigene56119\_Sample\_011046841, Unigene25997\_Sample\_011046841, Unigene54933\_Sample\_011046841, Unigene15720\_Sample\_011046841, Unigene15374\_Sample\_011046841, Unigene55212\_Sample\_011046841, Unigene12971\_Sample\_011046841, Unigene37729\_Sample\_011046841, Unigene54368\_Sample\_011046841, Unigene43662\_Sample\_011046841, Unigene15565\_Sample\_011046841, Unigene33661\_Sample\_011046841, Unigene56818\_Sample\_011046841, Unigene58363\_Sample\_011046841, Unigene11148\_Sample\_011046841, Unigene41465\_Sample\_011046841, Unigene59540\_Sample\_011046841, Unigene50892\_Sample\_011046841, Unigene50243\_Sample\_011046841, Unigene46841\_Sample\_011046841, Unigene10489\_Sample\_011046841, Unigene45141\_Sample\_011046841, Unigene28376\_Sample\_011046841, Unigene19399\_Sample\_011046841, Unigene56629\_Sample\_011046841, Unigene13463\_Sample\_011046841, Unigene22907\_Sample\_011046841, Unigene43240\_Sample\_011046841, Unigene49909\_Sample\_011046841, Unigene27694\_Sample\_011046841, Unigene59712\_Sample\_011046841, Unigene23236\_Sample\_011046841, Unigene48755\_Sample\_011046841, Unigene60349\_Sample\_011046841, Unigene517\_Sample\_011046841, Unigene23790\_Sample\_011046841, Unigene59639\_Sample\_011046841, Unigene47334\_Sample\_011046841, Unigene13901\_Sample\_011046841, Unigene45316\_Sample\_011046841, Unigene39776\_Sample\_011046841, Unigene53719\_Sample\_011046841, Unigene56950\_Sample\_011046841, Unigene11546\_Sample\_011046841, Unigene29816\_Sample\_011046841, Unigene57184\_Sample\_011046841, Unigene37132\_Sample\_011046841, Unigene40532\_Sample\_011046841, Unigene33437\_Sample\_011046841, Unigene51426\_Sample\_011046841, Unigene4915\_Sample\_011046841, Unigene56503\_Sample\_011046841, Unigene13927\_Sample\_011046841, Unigene58943\_Sample\_011046841, Unigene27589\_Sample\_011046841, Unigene28537\_Sample\_011046841, Unigene59915\_Sample\_011046841, Unigene48197\_Sample\_011046841, Unigene41844\_Sample\_011046841, Unigene4599\_Sample\_011046841, Unigene41220\_Sample\_011046841, Unigene45262\_Sample\_011046841, Unigene42911\_Sample\_011046841, Unigene13430\_Sample\_011046841, Unigene35922\_Sample\_011046841, Unigene22154\_Sample\_011046841, Unigene39194\_Sample\_011046841, Unigene49501\_Sample\_011046841, Unigene12703\_Sample\_011046841, Unigene59987\_Sample\_011046841, Unigene39433\_Sample\_011046841, Unigene4236\_Sample\_011046841, Unigene50659\_Sample\_011046841, Unigene40989\_Sample\_011046841, Unigene3968\_Sample\_011046841, Unigene16224\_Sample\_011046841, Unigene12331\_Sample\_011046841, Unigene43481\_Sample\_011046841, Unigene35008\_Sample\_011046841, Unigene19743\_Sample\_011046841, Unigene54549\_Sample\_011046841, Unigene53926\_Sample\_011046841, Unigene52751\_Sample\_011046841, Unigene49426\_Sample\_011046841, Unigene53292\_Sample\_011046841, Unigene38234\_Sample\_011046841, Unigene46287\_Sample\_011046841, Unigene57997\_Sample\_011046841, Unigene56173\_Sample\_011046841, Unigene13656\_Sample\_011046841, Unigene55685\_Sample\_011046841, Unigene33194\_Sample\_011046841, Unigene54707\_Sample\_011046841, Unigene45004\_Sample\_011046841, Unigene22068\_Sample\_011046841, Unigene19437\_Sample\_011046841, Unigene12720\_Sample\_011046841, Unigene49126\_Sample\_011046841, Unigene9008\_Sample\_011046841, Unigene55877\_Sample\_011046841, Unigene29396\_Sample\_011046841, Unigene47025\_Sample\_011046841, Unigene56795\_Sample\_011046841, Unigene53326\_Sample\_011046841, Unigene59151\_Sample\_011046841, Unigene37422\_Sample\_011046841, Unigene59818\_Sample\_011046841, Unigene58960\_Sample\_011046841, Unigene56283\_Sample\_011046841, Unigene60906\_Sample\_011046841, Unigene54037\_Sample\_011046841, Unigene51763\_Sample\_011046841, Unigene48282\_Sample\_011046841, Unigene13920\_Sample\_011046841, Unigene60310\_Sample\_011046841, Unigene31656\_Sample\_011046841, Unigene53624\_Sample\_011046841, Unigene38787\_Sample\_011046841, Unigene60946\_Sample\_011046841, Unigene10563\_Sample\_011046841, Unigene9914\_Sample\_011046841, Unigene55879\_Sample\_011046841, Unigene1552\_Sample\_011046841, Unigene53462\_Sample\_011046841, Unigene40255\_Sample\_011046841, Unigene49650\_Sample\_011046841, Unigene46337\_Sample\_011046841, Unigene11539\_Sample\_011046841, Unigene56203\_Sample\_011046841, Unigene53018\_Sample\_011046841, Unigene13860\_Sample\_011046841, Unigene32973\_Sample\_011046841, Unigene54121\_Sample\_011046841, Unigene56579\_Sample\_011046841, Unigene32951\_Sample\_011046841, Unigene11563\_Sample\_011046841, Unigene33297\_Sample\_011046841, Unigene45754\_Sample\_011046841, Unigene53138\_Sample\_011046841, Unigene44764\_Sample\_011046841, Unigene24615\_Sample\_011046841, Unigene28739\_Sample\_011046841, Unigene30781\_Sample\_011046841, Unigene6757\_Sample\_011046841, Unigene13976\_Sample\_011046841, Unigene12306\_Sample\_011046841, Unigene58285\_Sample\_011046841, Unigene57843\_Sample\_011046841, Unigene59137\_Sample\_011046841, Unigene30989\_Sample\_011046841, Unigene48412\_Sample\_011046841, Unigene56081\_Sample\_011046841, Unigene60822\_Sample\_011046841, Unigene44725\_Sample\_011046841, Unigene36705\_Sample\_011046841, Unigene52551\_Sample\_011046841, Unigene60036\_Sample\_011046841, Unigene51163\_Sample\_011046841, Unigene49255\_Sample\_011046841, Unigene30684\_Sample\_011046841, Unigene59619\_Sample\_011046841, Unigene7507\_Sample\_011046841, Unigene11653\_Sample\_011046841, Unigene40287\_Sample\_011046841, Unigene7011\_Sample\_011046841, Unigene57426\_Sample\_011046841, Unigene59539\_Sample\_011046841, Unigene12212\_Sample\_011046841, Unigene60488\_Sample\_011046841, Unigene10700\_Sample\_011046841, Unigene21230\_Sample\_011046841, Unigene27108\_Sample\_011046841, Unigene30320\_Sample\_011046841, Unigene55302\_Sample\_011046841, Unigene46741\_Sample\_011046841, Unigene35902\_Sample\_011046841, Unigene47585\_Sample\_011046841, Unigene44049\_Sample\_011046841, Unigene55398\_Sample\_011046841, Unigene3263\_Sample\_011046841, Unigene55890\_Sample\_011046841, Unigene58210\_Sample\_011046841, Unigene6351\_Sample\_011046841, Unigene2986\_Sample\_011046841, Unigene52722\_Sample\_011046841, Unigene11139\_Sample\_011046841, Unigene25820\_Sample\_011046841, Unigene57559\_Sample\_011046841, Unigene59494\_Sample\_011046841, Unigene12170\_Sample\_011046841, Unigene12906\_Sample\_011046841, Unigene50528\_Sample\_011046841, Unigene10327\_Sample\_011046841, Unigene21023\_Sample\_011046841, Unigene59541\_Sample\_011046841, Unigene60887\_Sample\_011046841, Unigene39000\_Sample\_011046841, Unigene12586\_Sample\_011046841, Unigene8502\_Sample\_011046841, Unigene53546\_Sample\_011046841, Unigene23921\_Sample\_011046841, Unigene59687\_Sample\_011046841, Unigene2610\_Sample\_011046841, Unigene47125\_Sample\_011046841, Unigene9517\_Sample\_011046841, Unigene55187\_Sample\_011046841, Unigene56588\_Sample\_011046841, Unigene45483\_Sample\_011046841, Unigene59702\_Sample\_011046841, Unigene12523\_Sample\_011046841, Unigene30975\_Sample\_011046841, Unigene40874\_Sample\_011046841, Unigene23704\_Sample\_011046841, Unigene8301\_Sample\_011046841, Unigene11478\_Sample\_011046841, Unigene57792\_Sample\_011046841, Unigene56362\_Sample\_011046841, Unigene37760\_Sample\_011046841, Unigene2105\_Sample\_011046841, Unigene5382\_Sample\_011046841, Unigene60682\_Sample\_011046841, Unigene21647\_Sample\_011046841, Unigene42339\_Sample\_011046841, Unigene33766\_Sample\_011046841, Unigene15419\_Sample\_011046841, Unigene52334\_Sample\_011046841, Unigene92\_Sample\_011046841, Unigene34757\_Sample\_011046841, Unigene56978\_Sample\_011046841, Unigene11256\_Sample\_011046841, Unigene3148\_Sample\_011046841, Unigene6900\_Sample\_011046841, Unigene44425\_Sample\_011046841, Unigene45458\_Sample\_011046841, Unigene59695\_Sample\_011046841, Unigene19407\_Sample\_011046841, Unigene41781\_Sample\_011046841, Unigene5005\_Sample\_011046841, Unigene50606\_Sample\_011046841, Unigene53456\_Sample\_011046841, Unigene48293\_Sample\_011046841, Unigene47802\_Sample\_011046841, Unigene4749\_Sample\_011046841, Unigene22182\_Sample\_011046841, Unigene4218\_Sample\_011046841, Unigene56248\_Sample\_011046841, Unigene56389\_Sample\_011046841, Unigene4438\_Sample\_011046841, Unigene44939\_Sample\_011046841, Unigene31534\_Sample\_011046841, Unigene10122\_Sample\_011046841, Unigene42095\_Sample\_011046841, Unigene46754\_Sample\_011046841, Unigene38631\_Sample\_011046841, Unigene41153\_Sample\_011046841, Unigene59254\_Sample\_011046841, Unigene59167\_Sample\_011046841, Unigene48963\_Sample\_011046841, Unigene39422\_Sample\_011046841, Unigene7870\_Sample\_011046841, Unigene44683\_Sample\_011046841, Unigene55470\_Sample\_011046841, Unigene7125\_Sample\_011046841, Unigene32450\_Sample\_011046841, Unigene57556\_Sample\_011046841, Unigene56828\_Sample\_011046841, Unigene41980\_Sample\_011046841, Unigene31228\_Sample\_011046841, Unigene48316\_Sample\_011046841, Unigene56793\_Sample\_011046841, Unigene16291\_Sample\_011046841, Unigene10325\_Sample\_011046841, Unigene40347\_Sample\_011046841, Unigene22175\_Sample\_011046841, Unigene47018\_Sample\_011046841, Unigene28643\_Sample\_011046841, Unigene10421\_Sample\_011046841, Unigene30931\_Sample\_011046841, Unigene50088\_Sample\_011046841, Unigene36037\_Sample\_011046841, Unigene54416\_Sample\_011046841, Unigene50399\_Sample\_011046841, Unigene31717\_Sample\_011046841, Unigene53997\_Sample\_011046841, Unigene11168\_Sample\_011046841, Unigene58205\_Sample\_011046841, Unigene251\_Sample\_011046841, Unigene11842\_Sample\_011046841, Unigene8597\_Sample\_011046841, Unigene45898\_Sample\_011046841, Unigene41538\_Sample\_011046841, Unigene341\_Sample\_011046841, Unigene56288\_Sample\_011046841, Unigene60015\_Sample\_011046841, Unigene31772\_Sample\_011046841, Unigene57770\_Sample\_011046841, Unigene5896\_Sample\_011046841, Unigene60241\_Sample\_011046841, Unigene27250\_Sample\_011046841, Unigene4096\_Sample\_011046841, Unigene54581\_Sample\_011046841, Unigene15270\_Sample\_011046841, Unigene56901\_Sample\_011046841, Unigene56605\_Sample\_011046841, Unigene6061\_Sample\_011046841, Unigene48415\_Sample\_011046841, Unigene53259\_Sample\_011046841, Unigene44108\_Sample\_011046841, Unigene997\_Sample\_011046841, Unigene44635\_Sample\_011046841, Unigene55744\_Sample\_011046841, Unigene8613\_Sample\_011046841, Unigene45120\_Sample\_011046841, Unigene54454\_Sample\_011046841, Unigene475\_Sample\_011046841, Unigene46219\_Sample\_011046841, Unigene49114\_Sample\_011046841, Unigene12830\_Sample\_011046841, Unigene47947\_Sample\_011046841, Unigene18318\_Sample\_011046841, Unigene32348\_Sample\_011046841, Unigene10739\_Sample\_011046841, Unigene26651\_Sample\_011046841, Unigene34357\_Sample\_011046841, Unigene53617\_Sample\_011046841, Unigene8020\_Sample\_011046841, Unigene31768\_Sample\_011046841, Unigene48260\_Sample\_011046841, Unigene1345\_Sample\_011046841, Unigene52963\_Sample\_011046841, Unigene12172\_Sample\_011046841, Unigene50631\_Sample\_011046841, Unigene39987\_Sample\_011046841, Unigene15774\_Sample\_011046841, Unigene60161\_Sample\_011046841, Unigene55156\_Sample\_011046841, Unigene53650\_Sample\_011046841, Unigene19757\_Sample\_011046841, Unigene52734\_Sample\_011046841, Unigene57702\_Sample\_011046841, Unigene50051\_Sample\_011046841, Unigene48009\_Sample\_011046841, Unigene53736\_Sample\_011046841, Unigene57460\_Sample\_011046841, Unigene42827\_Sample\_011046841, Unigene56139\_Sample\_011046841, Unigene59022\_Sample\_011046841, Unigene41460\_Sample\_011046841, Unigene58133\_Sample\_011046841, Unigene55791\_Sample\_011046841, Unigene60842\_Sample\_011046841, Unigene15121\_Sample\_011046841, Unigene27784\_Sample\_011046841, Unigene59485\_Sample\_011046841, Unigene59398\_Sample\_011046841, Unigene30474\_Sample\_011046841, Unigene47936\_Sample\_011046841, Unigene51504\_Sample\_011046841, Unigene55650\_Sample\_011046841, Unigene49448\_Sample\_011046841, Unigene53160\_Sample\_011046841, Unigene13666\_Sample\_011046841, Unigene60020\_Sample\_011046841, Unigene11562\_Sample\_011046841, Unigene60937\_Sample\_011046841, Unigene51782\_Sample\_011046841, Unigene11114\_Sample\_011046841, Unigene58289\_Sample\_011046841, Unigene53278\_Sample\_011046841, Unigene57890\_Sample\_011046841, Unigene27010\_Sample\_011046841, Unigene13436\_Sample\_011046841, Unigene44587\_Sample\_011046841, Unigene57313\_Sample\_011046841, Unigene33440\_Sample\_011046841, Unigene29524\_Sample\_011046841, Unigene354\_Sample\_011046841, Unigene50506\_Sample\_011046841, Unigene1129\_Sample\_011046841, Unigene48398\_Sample\_011046841, Unigene4293\_Sample\_011046841, Unigene8290\_Sample\_011046841, Unigene55258\_Sample\_011046841, Unigene60388\_Sample\_011046841, Unigene58400\_Sample\_011046841, Unigene30825\_Sample\_011046841, Unigene56616\_Sample\_011046841, Unigene49776\_Sample\_011046841, Unigene59547\_Sample\_011046841, Unigene42378\_Sample\_011046841, Unigene54996\_Sample\_011046841, Unigene2138\_Sample\_011046841, Unigene42026\_Sample\_011046841, Unigene27770\_Sample\_011046841, Unigene60561\_Sample\_011046841, Unigene11448\_Sample\_011046841, Unigene12840\_Sample\_011046841, Unigene58386\_Sample\_011046841, Unigene60709\_Sample\_011046841, Unigene863\_Sample\_011046841, Unigene19327\_Sample\_011046841, Unigene27576\_Sample\_011046841, Unigene12076\_Sample\_011046841, Unigene1210\_Sample\_011046841, Unigene43570\_Sample\_011046841, Unigene55561\_Sample\_011046841, Unigene5615\_Sample\_011046841, Unigene13633\_Sample\_011046841, Unigene53798\_Sample\_011046841, Unigene45476\_Sample\_011046841, Unigene39678\_Sample\_011046841, Unigene41323\_Sample\_011046841, Unigene41608\_Sample\_011046841, Unigene59198\_Sample\_011046841, Unigene58496\_Sample\_011046841, Unigene43804\_Sample\_011046841, Unigene58845\_Sample\_011046841, Unigene57453\_Sample\_011046841, Unigene55575\_Sample\_011046841, Unigene26271\_Sample\_011046841, Unigene49487\_Sample\_011046841, Unigene36631\_Sample\_011046841, Unigene26737\_Sample\_011046841, Unigene9620\_Sample\_011046841, Unigene56991\_Sample\_011046841, Unigene54410\_Sample\_011046841, Unigene32488\_Sample\_011046841, Unigene55183\_Sample\_011046841, Unigene48170\_Sample\_011046841, Unigene40858\_Sample\_011046841, Unigene57238\_Sample\_011046841, Unigene46777\_Sample\_011046841, Unigene29126\_Sample\_011046841, Unigene57157\_Sample\_011046841, Unigene45403\_Sample\_011046841, Unigene34659\_Sample\_011046841, Unigene42148\_Sample\_011046841, Unigene8925\_Sample\_011046841, Unigene25272\_Sample\_011046841, Unigene14794\_Sample\_011046841, Unigene59567\_Sample\_011046841, Unigene10615\_Sample\_011046841, Unigene60123\_Sample\_011046841, Unigene53233\_Sample\_011046841, Unigene58169\_Sample\_011046841, Unigene26964\_Sample\_011046841, Unigene45620\_Sample\_011046841, Unigene54891\_Sample\_011046841, Unigene59136\_Sample\_011046841, Unigene50990\_Sample\_011046841, Unigene47487\_Sample\_011046841, Unigene55982\_Sample\_011046841, Unigene59621\_Sample\_011046841, Unigene50223\_Sample\_011046841, Unigene26723\_Sample\_011046841, Unigene50164\_Sample\_011046841, Unigene15865\_Sample\_011046841, Unigene55529\_Sample\_011046841, Unigene54555\_Sample\_011046841, Unigene38618\_Sample\_011046841, Unigene32930\_Sample\_011046841, Unigene55300\_Sample\_011046841, Unigene35963\_Sample\_011046841, Unigene46525\_Sample\_011046841, Unigene18725\_Sample\_011046841, Unigene12244\_Sample\_011046841, Unigene51260\_Sample\_011046841, Unigene5096\_Sample\_011046841, Unigene55754\_Sample\_011046841, Unigene57524\_Sample\_011046841, Unigene41168\_Sample\_011046841, Unigene44079\_Sample\_011046841, Unigene38180\_Sample\_011046841, Unigene57323\_Sample\_011046841, Unigene27581\_Sample\_011046841, Unigene60586\_Sample\_011046841, Unigene60793\_Sample\_011046841, Unigene23033\_Sample\_011046841, Unigene1306\_Sample\_011046841, Unigene19699\_Sample\_011046841, Unigene52106\_Sample\_011046841, Unigene54406\_Sample\_011046841, Unigene54852\_Sample\_011046841, Unigene59027\_Sample\_011046841, Unigene59457\_Sample\_011046841, Unigene22974\_Sample\_011046841, Unigene59563\_Sample\_011046841, Unigene204\_Sample\_011046841, Unigene18813\_Sample\_011046841, Unigene23568\_Sample\_011046841, Unigene51803\_Sample\_011046841, Unigene33068\_Sample\_011046841, Unigene58674\_Sample\_011046841, Unigene4166\_Sample\_011046841, Unigene39921\_Sample\_011046841, Unigene41696\_Sample\_011046841, Unigene21082\_Sample\_011046841, Unigene5102\_Sample\_011046841, Unigene47907\_Sample\_011046841, Unigene8538\_Sample\_011046841, Unigene60225\_Sample\_011046841, Unigene50775\_Sample\_011046841, Unigene31642\_Sample\_011046841, Unigene51737\_Sample\_011046841, Unigene45679\_Sample\_011046841, Unigene56457\_Sample\_011046841, Unigene8255\_Sample\_011046841, Unigene49371\_Sample\_011046841, Unigene43561\_Sample\_011046841, Unigene4944\_Sample\_011046841, Unigene3496\_Sample\_011046841, Unigene42012\_Sample\_011046841, Unigene38168\_Sample\_011046841, Unigene69\_Sample\_011046841, Unigene51555\_Sample\_011046841, Unigene46635\_Sample\_011046841, Unigene60249\_Sample\_011046841, Unigene6106\_Sample\_011046841, Unigene48882\_Sample\_011046841, Unigene35453\_Sample\_011046841, Unigene53930\_Sample\_011046841, Unigene59679\_Sample\_011046841, Unigene32103\_Sample\_011046841, Unigene46439\_Sample\_011046841, Unigene50396\_Sample\_011046841, Unigene53015\_Sample\_011046841, Unigene58138\_Sample\_011046841, Unigene20591\_Sample\_011046841, Unigene36469\_Sample\_011046841, Unigene47753\_Sample\_011046841, Unigene29669\_Sample\_011046841, Unigene53833\_Sample\_011046841, Unigene44429\_Sample\_011046841, Unigene60705\_Sample\_011046841, Unigene14125\_Sample\_011046841, Unigene54221\_Sample\_011046841, Unigene9329\_Sample\_011046841, Unigene47357\_Sample\_011046841, Unigene10640\_Sample\_011046841, Unigene6724\_Sample\_011046841, Unigene37898\_Sample\_011046841, Unigene42812\_Sample\_011046841, Unigene37098\_Sample\_011046841, Unigene56005\_Sample\_011046841, Unigene39223\_Sample\_011046841, Unigene55306\_Sample\_011046841, Unigene47206\_Sample\_011046841, Unigene43099\_Sample\_011046841, Unigene56127\_Sample\_011046841, Unigene57340\_Sample\_011046841, Unigene3756\_Sample\_011046841, Unigene13258\_Sample\_011046841, Unigene56364\_Sample\_011046841, Unigene28709\_Sample\_011046841, Unigene51569\_Sample\_011046841, Unigene10204\_Sample\_011046841, Unigene10963\_Sample\_011046841, Unigene15\_Sample\_011046841, Unigene60889\_Sample\_011046841, Unigene17258\_Sample\_011046841, Unigene51613\_Sample\_011046841, Unigene47150\_Sample\_011046841, Unigene7887\_Sample\_011046841, Unigene40697\_Sample\_011046841, Unigene49861\_Sample\_011046841, Unigene44518\_Sample\_011046841, Unigene58929\_Sample\_011046841, Unigene12620\_Sample\_011046841, Unigene49392\_Sample\_011046841, Unigene41822\_Sample\_011046841, Unigene13203\_Sample\_011046841, Unigene57106\_Sample\_011046841, Unigene42625\_Sample\_011046841, Unigene23667\_Sample\_011046841, Unigene50127\_Sample\_011046841, Unigene8959\_Sample\_011046841, Unigene33127\_Sample\_011046841, Unigene42747\_Sample\_011046841, Unigene51225\_Sample\_011046841, Unigene55175\_Sample\_011046841, Unigene58053\_Sample\_011046841, Unigene60317\_Sample\_011046841, Unigene19596\_Sample\_011046841, Unigene40147\_Sample\_011046841, Unigene16055\_Sample\_011046841, Unigene8243\_Sample\_011046841, Unigene45131\_Sample\_011046841, Unigene38974\_Sample\_011046841, Unigene54014\_Sample\_011046841, Unigene51363\_Sample\_011046841, Unigene60921\_Sample\_011046841, Unigene53797\_Sample\_011046841, Unigene39877\_Sample\_011046841, Unigene49532\_Sample\_011046841, Unigene9219\_Sample\_011046841, Unigene9304\_Sample\_011046841, Unigene52271\_Sample\_011046841, Unigene53986\_Sample\_011046841, Unigene36873\_Sample\_011046841, Unigene11300\_Sample\_011046841, Unigene27430\_Sample\_011046841, Unigene56881\_Sample\_011046841, Unigene57132\_Sample\_011046841, Unigene47368\_Sample\_011046841, Unigene60296\_Sample\_011046841, Unigene44016\_Sample\_011046841, Unigene56920\_Sample\_011046841, Unigene48876\_Sample\_011046841, Unigene8387\_Sample\_011046841, Unigene56058\_Sample\_011046841, Unigene12913\_Sample\_011046841, Unigene49698\_Sample\_011046841, Unigene11187\_Sample\_011046841, Unigene53037\_Sample\_011046841, Unigene10930\_Sample\_011046841, Unigene54326\_Sample\_011046841, Unigene38373\_Sample\_011046841, Unigene20608\_Sample\_011046841, Unigene57971\_Sample\_011046841, Unigene13853\_Sample\_011046841, Unigene46000\_Sample\_011046841, Unigene46774\_Sample\_011046841, Unigene31746\_Sample\_011046841, Unigene59224\_Sample\_011046841, Unigene50689\_Sample\_011046841, Unigene57610\_Sample\_011046841, Unigene50412\_Sample\_011046841, Unigene45626\_Sample\_011046841, Unigene11195\_Sample\_011046841, Unigene9110\_Sample\_011046841, Unigene49633\_Sample\_011046841, Unigene59919\_Sample\_011046841, Unigene53334\_Sample\_011046841, Unigene45873\_Sample\_011046841, Unigene42703\_Sample\_011046841, Unigene21480\_Sample\_011046841, Unigene60714\_Sample\_011046841, Unigene2325\_Sample\_011046841, Unigene6762\_Sample\_011046841, Unigene10098\_Sample\_011046841, Unigene56560\_Sample\_011046841, Unigene59819\_Sample\_011046841, Unigene36721\_Sample\_011046841, Unigene57060\_Sample\_011046841, Unigene42105\_Sample\_011046841, Unigene18305\_Sample\_011046841, Unigene58882\_Sample\_011046841, Unigene19753\_Sample\_011046841, Unigene40568\_Sample\_011046841, Unigene53410\_Sample\_011046841, Unigene60706\_Sample\_011046841, Unigene27866\_Sample\_011046841, Unigene59134\_Sample\_011046841, Unigene20324\_Sample\_011046841, Unigene47239\_Sample\_011046841, Unigene41573\_Sample\_011046841, Unigene21163\_Sample\_011046841, Unigene54842\_Sample\_011046841, Unigene59587\_Sample\_011046841, Unigene31705\_Sample\_011046841, Unigene54643\_Sample\_011046841, Unigene58326\_Sample\_011046841, Unigene58953\_Sample\_011046841, Unigene33436\_Sample\_011046841, Unigene56096\_Sample\_011046841, Unigene55769\_Sample\_011046841, Unigene2225\_Sample\_011046841, Unigene40143\_Sample\_011046841, Unigene33941\_Sample\_011046841, Unigene19813\_Sample\_011046841, Unigene7880\_Sample\_011046841, Unigene30285\_Sample\_011046841, Unigene54874\_Sample\_011046841, Unigene32364\_Sample\_011046841, Unigene42637\_Sample\_011046841, Unigene24860\_Sample\_011046841, Unigene29861\_Sample\_011046841, Unigene51384\_Sample\_011046841, Unigene37668\_Sample\_011046841, Unigene13569\_Sample\_011046841, Unigene46263\_Sample\_011046841, Unigene49204\_Sample\_011046841, Unigene29587\_Sample\_011046841, Unigene57696\_Sample\_011046841, Unigene42749\_Sample\_011046841, Unigene60127\_Sample\_011046841, Unigene30072\_Sample\_011046841, Unigene30649\_Sample\_011046841, Unigene11662\_Sample\_011046841, Unigene29183\_Sample\_011046841, Unigene24953\_Sample\_011046841, Unigene55491\_Sample\_011046841, Unigene59038\_Sample\_011046841, Unigene46978\_Sample\_011046841, Unigene38930\_Sample\_011046841, Unigene52253\_Sample\_011046841, Unigene60288\_Sample\_011046841, Unigene7850\_Sample\_011046841, Unigene54900\_Sample\_011046841, Unigene60217\_Sample\_011046841, Unigene56635\_Sample\_011046841, Unigene44384\_Sample\_011046841, Unigene641\_Sample\_011046841, Unigene55854\_Sample\_011046841, Unigene60596\_Sample\_011046841, Unigene58376\_Sample\_011046841, Unigene57614\_Sample\_011046841, Unigene57972\_Sample\_011046841, Unigene50665\_Sample\_011046841, Unigene16237\_Sample\_011046841, Unigene51483\_Sample\_011046841, Unigene29630\_Sample\_011046841, Unigene34269\_Sample\_011046841, Unigene57585\_Sample\_011046841, Unigene36328\_Sample\_011046841, Unigene49481\_Sample\_011046841, Unigene54370\_Sample\_011046841, Unigene46332\_Sample\_011046841, Unigene20433\_Sample\_011046841, Unigene39621\_Sample\_011046841, Unigene1934\_Sample\_011046841, Unigene42270\_Sample\_011046841, Unigene19030\_Sample\_011046841, Unigene51777\_Sample\_011046841, Unigene15267\_Sample\_011046841, Unigene25153\_Sample\_011046841, Unigene57259\_Sample\_011046841, Unigene53179\_Sample\_011046841, Unigene27324\_Sample\_011046841, Unigene55162\_Sample\_011046841, Unigene53053\_Sample\_011046841, Unigene43409\_Sample\_011046841, Unigene11431\_Sample\_011046841, Unigene36131\_Sample\_011046841, Unigene59179\_Sample\_011046841, Unigene48613\_Sample\_011046841, Unigene4684\_Sample\_011046841, Unigene50469\_Sample\_011046841, Unigene42151\_Sample\_011046841, Unigene53698\_Sample\_011046841, Unigene50681\_Sample\_011046841, Unigene50764\_Sample\_011046841, Unigene5863\_Sample\_011046841, Unigene60949\_Sample\_011046841, Unigene33792\_Sample\_011046841, Unigene43020\_Sample\_011046841, Unigene24568\_Sample\_011046841, Unigene60729\_Sample\_011046841, Unigene26672\_Sample\_011046841, Unigene10356\_Sample\_011046841, Unigene13791\_Sample\_011046841, Unigene51460\_Sample\_011046841, Unigene9011\_Sample\_011046841, Unigene10259\_Sample\_011046841, Unigene34203\_Sample\_011046841, Unigene22086\_Sample\_011046841, Unigene58190\_Sample\_011046841, Unigene41288\_Sample\_011046841, Unigene52342\_Sample\_011046841, Unigene25623\_Sample\_011046841, Unigene51527\_Sample\_011046841, Unigene35672\_Sample\_011046841, Unigene51340\_Sample\_011046841, Unigene1944\_Sample\_011046841, Unigene45469\_Sample\_011046841, Unigene59628\_Sample\_011046841, Unigene59442\_Sample\_011046841, Unigene58495\_Sample\_011046841, Unigene11211\_Sample\_011046841, Unigene24305\_Sample\_011046841, Unigene47924\_Sample\_011046841, Unigene60699\_Sample\_011046841, Unigene49863\_Sample\_011046841, Unigene54628\_Sample\_011046841, Unigene35914\_Sample\_011046841, Unigene55269\_Sample\_011046841, Unigene11514\_Sample\_011046841, Unigene52467\_Sample\_011046841, Unigene2385\_Sample\_011046841, Unigene49162\_Sample\_011046841, Unigene18124\_Sample\_011046841, Unigene54205\_Sample\_011046841, Unigene60785\_Sample\_011046841, Unigene21919\_Sample\_011046841, Unigene13261\_Sample\_011046841, Unigene53942\_Sample\_011046841, Unigene54448\_Sample\_011046841, Unigene36083\_Sample\_011046841, Unigene58611\_Sample\_011046841, Unigene60055\_Sample\_011046841, Unigene47846\_Sample\_011046841, Unigene58753\_Sample\_011046841, Unigene60966\_Sample\_011046841, Unigene13951\_Sample\_011046841, Unigene60106\_Sample\_011046841, Unigene55840\_Sample\_011046841, Unigene52776\_Sample\_011046841, Unigene11088\_Sample\_011046841, Unigene29206\_Sample\_011046841, Unigene56208\_Sample\_011046841, Unigene33721\_Sample\_011046841, Unigene60371\_Sample\_011046841, Unigene11730\_Sample\_011046841, Unigene10370\_Sample\_011046841, Unigene58223\_Sample\_011046841, Unigene10793\_Sample\_011046841, Unigene19671\_Sample\_011046841, Unigene11351\_Sample\_011046841, Unigene13007\_Sample\_011046841, Unigene27756\_Sample\_011046841, Unigene33218\_Sample\_011046841, Unigene4226\_Sample\_011046841, Unigene39581\_Sample\_011046841, Unigene47872\_Sample\_011046841, Unigene10588\_Sample\_011046841, Unigene58058\_Sample\_011046841, Unigene48598\_Sample\_011046841, Unigene57776\_Sample\_011046841, Unigene12822\_Sample\_011046841, Unigene47882\_Sample\_011046841, Unigene33755\_Sample\_011046841, Unigene31695\_Sample\_011046841, Unigene28860\_Sample\_011046841, Unigene23133\_Sample\_011046841, Unigene50443\_Sample\_011046841, Unigene42349\_Sample\_011046841, Unigene17987\_Sample\_011046841, Unigene54835\_Sample\_011046841, Unigene60642\_Sample\_011046841, Unigene10464\_Sample\_011046841, Unigene31515\_Sample\_011046841, Unigene31120\_Sample\_011046841, Unigene16810\_Sample\_011046841, Unigene52623\_Sample\_011046841, Unigene59727\_Sample\_011046841, Unigene7922\_Sample\_011046841, Unigene42686\_Sample\_011046841, Unigene12501\_Sample\_011046841, Unigene44142\_Sample\_011046841, Unigene33132\_Sample\_011046841, Unigene28348\_Sample\_011046841, Unigene51977\_Sample\_011046841, Unigene35691\_Sample\_011046841, Unigene55527\_Sample\_011046841, Unigene13115\_Sample\_011046841, Unigene44567\_Sample\_011046841, Unigene60418\_Sample\_011046841, Unigene13447\_Sample\_011046841, Unigene3666\_Sample\_011046841, Unigene59743\_Sample\_011046841, Unigene6187\_Sample\_011046841, Unigene53254\_Sample\_011046841, Unigene14570\_Sample\_011046841, Unigene14358\_Sample\_011046841, Unigene16199\_Sample\_011046841, Unigene55737\_Sample\_011046841, Unigene51942\_Sample\_011046841, Unigene49055\_Sample\_011046841, Unigene10152\_Sample\_011046841, Unigene40271\_Sample\_011046841, Unigene25397\_Sample\_011046841, Unigene55423\_Sample\_011046841, Unigene45275\_Sample\_011046841, Unigene53845\_Sample\_011046841, Unigene50825\_Sample\_011046841, Unigene59490\_Sample\_011046841, Unigene57219\_Sample\_011046841, Unigene60208\_Sample\_011046841, Unigene40648\_Sample\_011046841, Unigene53415\_Sample\_011046841, Unigene21239\_Sample\_011046841, Unigene49898\_Sample\_011046841, Unigene14538\_Sample\_011046841, Unigene41467\_Sample\_011046841, Unigene56385\_Sample\_011046841, Unigene28678\_Sample\_011046841, Unigene37420\_Sample\_011046841, Unigene12811\_Sample\_011046841, Unigene6240\_Sample\_011046841, Unigene15204\_Sample\_011046841, Unigene52238\_Sample\_011046841, Unigene27451\_Sample\_011046841, Unigene46897\_Sample\_011046841, Unigene59170\_Sample\_011046841, Unigene59001\_Sample\_011046841, Unigene7095\_Sample\_011046841, Unigene58584\_Sample\_011046841, Unigene58934\_Sample\_011046841, Unigene31545\_Sample\_011046841, Unigene59935\_Sample\_011046841, Unigene56584\_Sample\_011046841, Unigene4949\_Sample\_011046841, Unigene56360\_Sample\_011046841, Unigene20059\_Sample\_011046841, Unigene44216\_Sample\_011046841, Unigene9649\_Sample\_011046841, Unigene24715\_Sample\_011046841, Unigene13112\_Sample\_011046841, Unigene32360\_Sample\_011046841, Unigene46860\_Sample\_011046841, Unigene51270\_Sample\_011046841, Unigene53913\_Sample\_011046841, Unigene60666\_Sample\_011046841, Unigene33607\_Sample\_011046841, Unigene40565\_Sample\_011046841, Unigene60917\_Sample\_011046841, Unigene43690\_Sample\_011046841, Unigene39138\_Sample\_011046841, Unigene32964\_Sample\_011046841, Unigene23751\_Sample\_011046841, Unigene49337\_Sample\_011046841, Unigene40440\_Sample\_011046841, Unigene50303\_Sample\_011046841, Unigene55039\_Sample\_011046841, Unigene46598\_Sample\_011046841, Unigene45157\_Sample\_011046841, Unigene58610\_Sample\_011046841, Unigene55163\_Sample\_011046841, Unigene46488\_Sample\_011046841, Unigene33026\_Sample\_011046841, Unigene41903\_Sample\_011046841, Unigene41001\_Sample\_011046841, Unigene42862\_Sample\_011046841, Unigene45662\_Sample\_011046841, Unigene60245\_Sample\_011046841, Unigene54460\_Sample\_011046841, Unigene32550\_Sample\_011046841, Unigene52094\_Sample\_011046841, Unigene59861\_Sample\_011046841, Unigene31025\_Sample\_011046841, Unigene53128\_Sample\_011046841, Unigene31154\_Sample\_011046841, Unigene54574\_Sample\_011046841, Unigene55111\_Sample\_011046841, Unigene24973\_Sample\_011046841, Unigene60723\_Sample\_011046841, Unigene12846\_Sample\_011046841, Unigene8551\_Sample\_011046841, Unigene54003\_Sample\_011046841, Unigene59750\_Sample\_011046841, Unigene59877\_Sample\_011046841, Unigene53888\_Sample\_011046841, Unigene13079\_Sample\_011046841, Unigene22245\_Sample\_011046841, Unigene54338\_Sample\_011046841, Unigene775\_Sample\_011046841, Unigene1010\_Sample\_011046841, Unigene55582\_Sample\_011046841, Unigene48252\_Sample\_011046841, Unigene55301\_Sample\_011046841, Unigene50746\_Sample\_011046841, Unigene48257\_Sample\_011046841, Unigene56109\_Sample\_011046841, Unigene31514\_Sample\_011046841, Unigene59872\_Sample\_011046841, Unigene36891\_Sample\_011046841, Unigene46497\_Sample\_011046841, Unigene132\_Sample\_011046841, Unigene59701\_Sample\_011046841, Unigene21319\_Sample\_011046841, Unigene41135\_Sample\_011046841, Unigene19091\_Sample\_011046841, Unigene30295\_Sample\_011046841, Unigene53019\_Sample\_011046841, Unigene60180\_Sample\_011046841, Unigene45049\_Sample\_011046841, Unigene42742\_Sample\_011046841, Unigene31216\_Sample\_011046841, Unigene55112\_Sample\_011046841, Unigene51279\_Sample\_011046841, Unigene30035\_Sample\_011046841, Unigene57353\_Sample\_011046841, Unigene59546\_Sample\_011046841, Unigene55624\_Sample\_011046841, Unigene18756\_Sample\_011046841, Unigene21728\_Sample\_011046841, Unigene38349\_Sample\_011046841, Unigene11786\_Sample\_011046841, Unigene48926\_Sample\_011046841, Unigene56647\_Sample\_011046841, Unigene16264\_Sample\_011046841, Unigene55372\_Sample\_011046841, Unigene52808\_Sample\_011046841, Unigene54711\_Sample\_011046841, Unigene34657\_Sample\_011046841, Unigene58724\_Sample\_011046841, Unigene19355\_Sample\_011046841, Unigene59459\_Sample\_011046841, Unigene41206\_Sample\_011046841, Unigene17531\_Sample\_011046841, Unigene42107\_Sample\_011046841, Unigene53711\_Sample\_011046841, Unigene54273\_Sample\_011046841, Unigene60407\_Sample\_011046841, Unigene35030\_Sample\_011046841, Unigene39087\_Sample\_011046841, Unigene27618\_Sample\_011046841, Unigene57973\_Sample\_011046841, Unigene59177\_Sample\_011046841, Unigene32121\_Sample\_011046841, Unigene41604\_Sample\_011046841, Unigene60464\_Sample\_011046841, Unigene12189\_Sample\_011046841, Unigene26214\_Sample\_011046841, Unigene13116\_Sample\_011046841, Unigene59005\_Sample\_011046841, Unigene29365\_Sample\_011046841, Unigene44253\_Sample\_011046841, Unigene52177\_Sample\_011046841, Unigene2221\_Sample\_011046841, Unigene53424\_Sample\_011046841, Unigene60728\_Sample\_011046841, Unigene51126\_Sample\_011046841, Unigene59372\_Sample\_011046841, Unigene48026\_Sample\_011046841, Unigene36178\_Sample\_011046841, Unigene40161\_Sample\_011046841, Unigene58011\_Sample\_011046841, Unigene55792\_Sample\_011046841, Unigene23102\_Sample\_011046841, Unigene49667\_Sample\_011046841, Unigene7487\_Sample\_011046841, Unigene43515\_Sample\_011046841, Unigene30591\_Sample\_011046841, Unigene44243\_Sample\_011046841, Unigene16372\_Sample\_011046841, Unigene53154\_Sample\_011046841, Unigene58042\_Sample\_011046841, Unigene24797\_Sample\_011046841, Unigene60040\_Sample\_011046841, Unigene60220\_Sample\_011046841, Unigene41575\_Sample\_011046841, Unigene45451\_Sample\_011046841, Unigene57330\_Sample\_011046841, Unigene56877\_Sample\_011046841, Unigene52934\_Sample\_011046841, Unigene60100\_Sample\_011046841, Unigene51011\_Sample\_011046841, Unigene11358\_Sample\_011046841, Unigene38889\_Sample\_011046841, Unigene35814\_Sample\_011046841, Unigene9408\_Sample\_011046841, Unigene21679\_Sample\_011046841, Unigene9130\_Sample\_011046841, Unigene44178\_Sample\_011046841, Unigene18000\_Sample\_011046841, Unigene56383\_Sample\_011046841, Unigene12824\_Sample\_011046841, Unigene60733\_Sample\_011046841, Unigene11124\_Sample\_011046841, Unigene18356\_Sample\_011046841, Unigene6946\_Sample\_011046841, Unigene54386\_Sample\_011046841, Unigene57760\_Sample\_011046841, Unigene44025\_Sample\_011046841, Unigene55494\_Sample\_011046841, Unigene29140\_Sample\_011046841, Unigene43097\_Sample\_011046841, Unigene3893\_Sample\_011046841, Unigene21318\_Sample\_011046841, Unigene39698\_Sample\_011046841, Unigene31652\_Sample\_011046841, Unigene3659\_Sample\_011046841, Unigene40683\_Sample\_011046841, Unigene11203\_Sample\_011046841, Unigene13456\_Sample\_011046841, Unigene46158\_Sample\_011046841, Unigene20702\_Sample\_011046841, Unigene48072\_Sample\_011046841, Unigene45885\_Sample\_011046841, Unigene47635\_Sample\_011046841, Unigene39552\_Sample\_011046841, Unigene50927\_Sample\_011046841, Unigene55779\_Sample\_011046841, Unigene21594\_Sample\_011046841, Unigene45790\_Sample\_011046841, Unigene53787\_Sample\_011046841, Unigene13763\_Sample\_011046841, Unigene13947\_Sample\_011046841, Unigene58343\_Sample\_011046841, Unigene57194\_Sample\_011046841, Unigene57425\_Sample\_011046841, Unigene12725\_Sample\_011046841, Unigene53231\_Sample\_011046841, Unigene33738\_Sample\_011046841, Unigene52972\_Sample\_011046841, Unigene55896\_Sample\_011046841, Unigene36823\_Sample\_011046841, Unigene43651\_Sample\_011046841, Unigene1482\_Sample\_011046841, Unigene36199\_Sample\_011046841, Unigene21149\_Sample\_011046841, Unigene43986\_Sample\_011046841, Unigene42734\_Sample\_011046841, Unigene25064\_Sample\_011046841, Unigene51663\_Sample\_011046841, Unigene7016\_Sample\_011046841, Unigene53749\_Sample\_011046841, Unigene58963\_Sample\_011046841, Unigene39312\_Sample\_011046841, Unigene6279\_Sample\_011046841, Unigene54599\_Sample\_011046841, Unigene59532\_Sample\_011046841, Unigene12957\_Sample\_011046841, Unigene16092\_Sample\_011046841, Unigene54526\_Sample\_011046841, Unigene12559\_Sample\_011046841, Unigene11321\_Sample\_011046841, Unigene43897\_Sample\_011046841, Unigene7072\_Sample\_011046841, Unigene43528\_Sample\_011046841, Unigene33603\_Sample\_011046841, Unigene9211\_Sample\_011046841, Unigene51429\_Sample\_011046841, Unigene12412\_Sample\_011046841, Unigene54372\_Sample\_011046841, Unigene13839\_Sample\_011046841, Unigene51427\_Sample\_011046841, Unigene58989\_Sample\_011046841, Unigene53555\_Sample\_011046841, Unigene39557\_Sample\_011046841, Unigene44445\_Sample\_011046841, Unigene36986\_Sample\_011046841, Unigene10648\_Sample\_011046841, Unigene892\_Sample\_011046841, Unigene42117\_Sample\_011046841, Unigene60125\_Sample\_011046841, Unigene55499\_Sample\_011046841, Unigene45708\_Sample\_011046841, Unigene57678\_Sample\_011046841, Unigene13923\_Sample\_011046841, Unigene49320\_Sample\_011046841, Unigene57264\_Sample\_011046841, Unigene53451\_Sample\_011046841, Unigene53963\_Sample\_011046841 |
| intracellular membrane-bounded organelle | Unigene28655\_Sample\_011046841, Unigene7801\_Sample\_011046841, Unigene43386\_Sample\_011046841, Unigene34629\_Sample\_011046841, Unigene40117\_Sample\_011046841, Unigene56478\_Sample\_011046841, Unigene44774\_Sample\_011046841, Unigene57691\_Sample\_011046841, Unigene52866\_Sample\_011046841, Unigene10419\_Sample\_011046841, Unigene52937\_Sample\_011046841, Unigene57534\_Sample\_011046841, Unigene2263\_Sample\_011046841, Unigene24428\_Sample\_011046841, Unigene54851\_Sample\_011046841, Unigene9589\_Sample\_011046841, Unigene58001\_Sample\_011046841, Unigene21625\_Sample\_011046841, Unigene49534\_Sample\_011046841, Unigene56578\_Sample\_011046841, Unigene648\_Sample\_011046841, Unigene53661\_Sample\_011046841, Unigene40873\_Sample\_011046841, Unigene29658\_Sample\_011046841, Unigene51638\_Sample\_011046841, Unigene26047\_Sample\_011046841, Unigene57913\_Sample\_011046841, Unigene60433\_Sample\_011046841, Unigene14639\_Sample\_011046841, Unigene6313\_Sample\_011046841, Unigene42542\_Sample\_011046841, Unigene37663\_Sample\_011046841, Unigene10955\_Sample\_011046841, Unigene60153\_Sample\_011046841, Unigene56979\_Sample\_011046841, Unigene34920\_Sample\_011046841, Unigene47691\_Sample\_011046841, Unigene49657\_Sample\_011046841, Unigene56375\_Sample\_011046841, Unigene59568\_Sample\_011046841, Unigene21912\_Sample\_011046841, Unigene42523\_Sample\_011046841, Unigene22636\_Sample\_011046841, Unigene2482\_Sample\_011046841, Unigene51160\_Sample\_011046841, Unigene39222\_Sample\_011046841, Unigene46690\_Sample\_011046841, Unigene55981\_Sample\_011046841, Unigene50110\_Sample\_011046841, Unigene46283\_Sample\_011046841, Unigene56425\_Sample\_011046841, Unigene27013\_Sample\_011046841, Unigene15188\_Sample\_011046841, Unigene12251\_Sample\_011046841, Unigene48164\_Sample\_011046841, Unigene36311\_Sample\_011046841, Unigene57919\_Sample\_011046841, Unigene34525\_Sample\_011046841, Unigene59077\_Sample\_011046841, Unigene12808\_Sample\_011046841, Unigene55357\_Sample\_011046841, Unigene44835\_Sample\_011046841, Unigene13859\_Sample\_011046841, Unigene60797\_Sample\_011046841, Unigene57216\_Sample\_011046841, Unigene41085\_Sample\_011046841, Unigene32616\_Sample\_011046841, Unigene40677\_Sample\_011046841, Unigene12458\_Sample\_011046841, Unigene39654\_Sample\_011046841, Unigene58779\_Sample\_011046841, Unigene12623\_Sample\_011046841, Unigene55509\_Sample\_011046841, Unigene813\_Sample\_011046841, Unigene13070\_Sample\_011046841, Unigene49579\_Sample\_011046841, Unigene60321\_Sample\_011046841, Unigene18575\_Sample\_011046841, Unigene29968\_Sample\_011046841, Unigene39783\_Sample\_011046841, Unigene12887\_Sample\_011046841, Unigene58892\_Sample\_011046841, Unigene51837\_Sample\_011046841, Unigene43716\_Sample\_011046841, Unigene52998\_Sample\_011046841, Unigene27774\_Sample\_011046841, Unigene26531\_Sample\_011046841, Unigene48054\_Sample\_011046841, Unigene44838\_Sample\_011046841, Unigene12490\_Sample\_011046841, Unigene12390\_Sample\_011046841, Unigene16060\_Sample\_011046841, Unigene49895\_Sample\_011046841, Unigene27615\_Sample\_011046841, Unigene19789\_Sample\_011046841, Unigene40280\_Sample\_011046841, Unigene57802\_Sample\_011046841, Unigene16763\_Sample\_011046841, Unigene59236\_Sample\_011046841, Unigene51182\_Sample\_011046841, Unigene57438\_Sample\_011046841, Unigene55707\_Sample\_011046841, Unigene25152\_Sample\_011046841, Unigene34464\_Sample\_011046841, Unigene58048\_Sample\_011046841, Unigene51\_Sample\_011046841, Unigene47010\_Sample\_011046841, Unigene49099\_Sample\_011046841, Unigene58409\_Sample\_011046841, Unigene39289\_Sample\_011046841, Unigene44749\_Sample\_011046841, Unigene36090\_Sample\_011046841, Unigene59178\_Sample\_011046841, Unigene59404\_Sample\_011046841, Unigene13397\_Sample\_011046841, Unigene57181\_Sample\_011046841, Unigene10027\_Sample\_011046841, Unigene56883\_Sample\_011046841, Unigene23937\_Sample\_011046841, Unigene52611\_Sample\_011046841, Unigene1918\_Sample\_011046841, Unigene19179\_Sample\_011046841, Unigene29053\_Sample\_011046841, Unigene31420\_Sample\_011046841, Unigene58502\_Sample\_011046841, Unigene41959\_Sample\_011046841, Unigene21808\_Sample\_011046841, Unigene13733\_Sample\_011046841, Unigene13844\_Sample\_011046841, Unigene44894\_Sample\_011046841, Unigene56688\_Sample\_011046841, Unigene13217\_Sample\_011046841, Unigene50912\_Sample\_011046841, Unigene57062\_Sample\_011046841, Unigene53170\_Sample\_011046841, Unigene17390\_Sample\_011046841, Unigene56209\_Sample\_011046841, Unigene46262\_Sample\_011046841, Unigene58466\_Sample\_011046841, Unigene50798\_Sample\_011046841, Unigene58106\_Sample\_011046841, Unigene41346\_Sample\_011046841, Unigene38048\_Sample\_011046841, Unigene11040\_Sample\_011046841, Unigene6573\_Sample\_011046841, Unigene52118\_Sample\_011046841, Unigene28681\_Sample\_011046841, Unigene19264\_Sample\_011046841, Unigene46558\_Sample\_011046841, Unigene53098\_Sample\_011046841, Unigene40116\_Sample\_011046841, Unigene13321\_Sample\_011046841, Unigene43791\_Sample\_011046841, Unigene44913\_Sample\_011046841, Unigene31438\_Sample\_011046841, Unigene54790\_Sample\_011046841, Unigene60872\_Sample\_011046841, Unigene60865\_Sample\_011046841, Unigene10202\_Sample\_011046841, Unigene22323\_Sample\_011046841, Unigene57892\_Sample\_011046841, Unigene3070\_Sample\_011046841, Unigene60019\_Sample\_011046841, Unigene59445\_Sample\_011046841, Unigene55518\_Sample\_011046841, Unigene2521\_Sample\_011046841, Unigene59685\_Sample\_011046841, Unigene36481\_Sample\_011046841, Unigene52298\_Sample\_011046841, Unigene50945\_Sample\_011046841, Unigene58655\_Sample\_011046841, Unigene56919\_Sample\_011046841, Unigene52033\_Sample\_011046841, Unigene45539\_Sample\_011046841, Unigene28246\_Sample\_011046841, Unigene6917\_Sample\_011046841, Unigene34524\_Sample\_011046841, Unigene13826\_Sample\_011046841, Unigene54323\_Sample\_011046841, Unigene24650\_Sample\_011046841, Unigene31773\_Sample\_011046841, Unigene60494\_Sample\_011046841, Unigene47134\_Sample\_011046841, Unigene30162\_Sample\_011046841, Unigene12142\_Sample\_011046841, Unigene52432\_Sample\_011046841, Unigene12082\_Sample\_011046841, Unigene58913\_Sample\_011046841, Unigene29827\_Sample\_011046841, Unigene28200\_Sample\_011046841, Unigene13887\_Sample\_011046841, Unigene12748\_Sample\_011046841, Unigene9545\_Sample\_011046841, Unigene13249\_Sample\_011046841, Unigene51253\_Sample\_011046841, Unigene48708\_Sample\_011046841, Unigene54695\_Sample\_011046841, Unigene25596\_Sample\_011046841, Unigene43848\_Sample\_011046841, Unigene48921\_Sample\_011046841, Unigene56941\_Sample\_011046841, Unigene13517\_Sample\_011046841, Unigene11362\_Sample\_011046841, Unigene51682\_Sample\_011046841, Unigene28964\_Sample\_011046841, Unigene35387\_Sample\_011046841, Unigene54256\_Sample\_011046841, Unigene44021\_Sample\_011046841, Unigene60900\_Sample\_011046841, Unigene13944\_Sample\_011046841, Unigene54168\_Sample\_011046841, Unigene49671\_Sample\_011046841, Unigene60178\_Sample\_011046841, Unigene14914\_Sample\_011046841, Unigene53072\_Sample\_011046841, Unigene25924\_Sample\_011046841, Unigene45568\_Sample\_011046841, Unigene59447\_Sample\_011046841, Unigene8429\_Sample\_011046841, Unigene57943\_Sample\_011046841, Unigene49720\_Sample\_011046841, Unigene8070\_Sample\_011046841, Unigene727\_Sample\_011046841, Unigene56887\_Sample\_011046841, Unigene46419\_Sample\_011046841, Unigene13719\_Sample\_011046841, Unigene15482\_Sample\_011046841, Unigene59158\_Sample\_011046841, Unigene59528\_Sample\_011046841, Unigene54246\_Sample\_011046841, Unigene32652\_Sample\_011046841, Unigene5603\_Sample\_011046841, Unigene53167\_Sample\_011046841, Unigene56916\_Sample\_011046841, Unigene18126\_Sample\_011046841, Unigene52226\_Sample\_011046841, Unigene45803\_Sample\_011046841, Unigene40258\_Sample\_011046841, Unigene2189\_Sample\_011046841, Unigene38963\_Sample\_011046841, Unigene2472\_Sample\_011046841, Unigene7826\_Sample\_011046841, Unigene33507\_Sample\_011046841, Unigene52688\_Sample\_011046841, Unigene5602\_Sample\_011046841, Unigene40006\_Sample\_011046841, Unigene3486\_Sample\_011046841, Unigene33846\_Sample\_011046841, Unigene50302\_Sample\_011046841, Unigene9572\_Sample\_011046841, Unigene43918\_Sample\_011046841, Unigene60919\_Sample\_011046841, Unigene35589\_Sample\_011046841, Unigene14355\_Sample\_011046841, Unigene59891\_Sample\_011046841, Unigene48337\_Sample\_011046841, Unigene4523\_Sample\_011046841, Unigene24401\_Sample\_011046841, Unigene46321\_Sample\_011046841, Unigene10941\_Sample\_011046841, Unigene51110\_Sample\_011046841, Unigene47680\_Sample\_011046841, Unigene55432\_Sample\_011046841, Unigene10309\_Sample\_011046841, Unigene3431\_Sample\_011046841, Unigene56517\_Sample\_011046841, Unigene57374\_Sample\_011046841, Unigene48105\_Sample\_011046841, Unigene44438\_Sample\_011046841, Unigene27907\_Sample\_011046841, Unigene1222\_Sample\_011046841, Unigene57239\_Sample\_011046841, Unigene48735\_Sample\_011046841, Unigene40059\_Sample\_011046841, Unigene55335\_Sample\_011046841, Unigene28455\_Sample\_011046841, Unigene6871\_Sample\_011046841, Unigene36149\_Sample\_011046841, Unigene13983\_Sample\_011046841, Unigene37028\_Sample\_011046841, Unigene20559\_Sample\_011046841, Unigene42899\_Sample\_011046841, Unigene41472\_Sample\_011046841, Unigene8480\_Sample\_011046841, Unigene60199\_Sample\_011046841, Unigene10961\_Sample\_011046841, Unigene9067\_Sample\_011046841, Unigene32169\_Sample\_011046841, Unigene58365\_Sample\_011046841, Unigene11053\_Sample\_011046841, Unigene51167\_Sample\_011046841, Unigene58725\_Sample\_011046841, Unigene46688\_Sample\_011046841, Unigene58904\_Sample\_011046841, Unigene52769\_Sample\_011046841, Unigene44366\_Sample\_011046841, Unigene28578\_Sample\_011046841, Unigene9141\_Sample\_011046841, Unigene25827\_Sample\_011046841, Unigene25248\_Sample\_011046841, Unigene41327\_Sample\_011046841, Unigene44670\_Sample\_011046841, Unigene20491\_Sample\_011046841, Unigene9674\_Sample\_011046841, Unigene55247\_Sample\_011046841, Unigene40898\_Sample\_011046841, Unigene29952\_Sample\_011046841, Unigene29081\_Sample\_011046841, Unigene40729\_Sample\_011046841, Unigene52883\_Sample\_011046841, Unigene56886\_Sample\_011046841, Unigene48957\_Sample\_011046841, Unigene37947\_Sample\_011046841, Unigene40727\_Sample\_011046841, Unigene44507\_Sample\_011046841, Unigene54823\_Sample\_011046841, Unigene53095\_Sample\_011046841, Unigene21814\_Sample\_011046841, Unigene20596\_Sample\_011046841, Unigene5030\_Sample\_011046841, Unigene38803\_Sample\_011046841, Unigene13916\_Sample\_011046841, Unigene9082\_Sample\_011046841, Unigene29602\_Sample\_011046841, Unigene13694\_Sample\_011046841, Unigene5335\_Sample\_011046841, Unigene15933\_Sample\_011046841, Unigene47818\_Sample\_011046841, Unigene16524\_Sample\_011046841, Unigene49834\_Sample\_011046841, Unigene46207\_Sample\_011046841, Unigene42503\_Sample\_011046841, Unigene24100\_Sample\_011046841, Unigene54668\_Sample\_011046841, Unigene13416\_Sample\_011046841, Unigene4630\_Sample\_011046841, Unigene52375\_Sample\_011046841, Unigene34268\_Sample\_011046841, Unigene8488\_Sample\_011046841, Unigene32195\_Sample\_011046841, Unigene52627\_Sample\_011046841, Unigene44863\_Sample\_011046841, Unigene21108\_Sample\_011046841, Unigene50213\_Sample\_011046841, Unigene40263\_Sample\_011046841, Unigene52003\_Sample\_011046841, Unigene9190\_Sample\_011046841, Unigene13584\_Sample\_011046841, Unigene57699\_Sample\_011046841, Unigene33889\_Sample\_011046841, Unigene56395\_Sample\_011046841, Unigene60358\_Sample\_011046841, Unigene58810\_Sample\_011046841, Unigene51706\_Sample\_011046841, Unigene54733\_Sample\_011046841, Unigene15846\_Sample\_011046841, Unigene40444\_Sample\_011046841, Unigene12023\_Sample\_011046841, Unigene17383\_Sample\_011046841, Unigene58558\_Sample\_011046841, Unigene11639\_Sample\_011046841, Unigene3975\_Sample\_011046841, Unigene60216\_Sample\_011046841, Unigene51481\_Sample\_011046841, Unigene43377\_Sample\_011046841, Unigene2068\_Sample\_011046841, Unigene10683\_Sample\_011046841, Unigene55844\_Sample\_011046841, Unigene5336\_Sample\_011046841, Unigene60597\_Sample\_011046841, Unigene32247\_Sample\_011046841, Unigene14290\_Sample\_011046841, Unigene58907\_Sample\_011046841, Unigene39978\_Sample\_011046841, Unigene5747\_Sample\_011046841, Unigene60138\_Sample\_011046841, Unigene47625\_Sample\_011046841, Unigene58134\_Sample\_011046841, Unigene34228\_Sample\_011046841, Unigene53056\_Sample\_011046841, Unigene60269\_Sample\_011046841, Unigene2846\_Sample\_011046841, Unigene44697\_Sample\_011046841, Unigene8647\_Sample\_011046841, Unigene43504\_Sample\_011046841, Unigene38339\_Sample\_011046841, Unigene54656\_Sample\_011046841, Unigene58128\_Sample\_011046841, Unigene39295\_Sample\_011046841, Unigene58529\_Sample\_011046841, Unigene18442\_Sample\_011046841, Unigene32174\_Sample\_011046841, Unigene51471\_Sample\_011046841, Unigene55667\_Sample\_011046841, Unigene59378\_Sample\_011046841, Unigene16612\_Sample\_011046841, Unigene11911\_Sample\_011046841, Unigene26120\_Sample\_011046841, Unigene47995\_Sample\_011046841, Unigene52869\_Sample\_011046841, Unigene13151\_Sample\_011046841, Unigene60189\_Sample\_011046841, Unigene53184\_Sample\_011046841, Unigene47864\_Sample\_011046841, Unigene8128\_Sample\_011046841, Unigene5318\_Sample\_011046841, Unigene33884\_Sample\_011046841, Unigene47008\_Sample\_011046841, Unigene56524\_Sample\_011046841, Unigene35592\_Sample\_011046841, Unigene12950\_Sample\_011046841, Unigene59448\_Sample\_011046841, Unigene58587\_Sample\_011046841, Unigene19352\_Sample\_011046841, Unigene301\_Sample\_011046841, Unigene60051\_Sample\_011046841, Unigene47464\_Sample\_011046841, Unigene16974\_Sample\_011046841, Unigene52717\_Sample\_011046841, Unigene35141\_Sample\_011046841, Unigene13214\_Sample\_011046841, Unigene49287\_Sample\_011046841, Unigene53979\_Sample\_011046841, Unigene4616\_Sample\_011046841, Unigene60298\_Sample\_011046841, Unigene42731\_Sample\_011046841, Unigene38547\_Sample\_011046841, Unigene31593\_Sample\_011046841, Unigene53447\_Sample\_011046841, Unigene60006\_Sample\_011046841, Unigene26192\_Sample\_011046841, Unigene30713\_Sample\_011046841, Unigene52396\_Sample\_011046841, Unigene53425\_Sample\_011046841, Unigene13697\_Sample\_011046841, Unigene5866\_Sample\_011046841, Unigene28640\_Sample\_011046841, Unigene27612\_Sample\_011046841, Unigene58173\_Sample\_011046841, Unigene43698\_Sample\_011046841, Unigene16375\_Sample\_011046841, Unigene30898\_Sample\_011046841, Unigene54868\_Sample\_011046841, Unigene57575\_Sample\_011046841, Unigene25307\_Sample\_011046841, Unigene2085\_Sample\_011046841, Unigene42958\_Sample\_011046841, Unigene12940\_Sample\_011046841, Unigene58936\_Sample\_011046841, Unigene31954\_Sample\_011046841, Unigene22285\_Sample\_011046841, Unigene55076\_Sample\_011046841, Unigene49192\_Sample\_011046841, Unigene56212\_Sample\_011046841, Unigene48927\_Sample\_011046841, Unigene58697\_Sample\_011046841, Unigene35464\_Sample\_011046841, Unigene36235\_Sample\_011046841, Unigene49528\_Sample\_011046841, Unigene4242\_Sample\_011046841, Unigene12370\_Sample\_011046841, Unigene17752\_Sample\_011046841, Unigene48342\_Sample\_011046841, Unigene6733\_Sample\_011046841, Unigene56599\_Sample\_011046841, Unigene57375\_Sample\_011046841, Unigene58746\_Sample\_011046841, Unigene9578\_Sample\_011046841, Unigene51307\_Sample\_011046841, Unigene21909\_Sample\_011046841, Unigene40466\_Sample\_011046841, Unigene60450\_Sample\_011046841, Unigene17321\_Sample\_011046841, Unigene60115\_Sample\_011046841, Unigene30734\_Sample\_011046841, Unigene35480\_Sample\_011046841, Unigene10699\_Sample\_011046841, Unigene31249\_Sample\_011046841, Unigene49697\_Sample\_011046841, Unigene39833\_Sample\_011046841, Unigene13213\_Sample\_011046841, Unigene47837\_Sample\_011046841, Unigene56779\_Sample\_011046841, Unigene35722\_Sample\_011046841, Unigene57039\_Sample\_011046841, Unigene45801\_Sample\_011046841, Unigene9271\_Sample\_011046841, Unigene42633\_Sample\_011046841, Unigene8851\_Sample\_011046841, Unigene44479\_Sample\_011046841, Unigene24927\_Sample\_011046841, Unigene48267\_Sample\_011046841, Unigene40571\_Sample\_011046841, Unigene19044\_Sample\_011046841, Unigene51928\_Sample\_011046841, Unigene53016\_Sample\_011046841, Unigene58354\_Sample\_011046841, Unigene53302\_Sample\_011046841, Unigene46011\_Sample\_011046841, Unigene60493\_Sample\_011046841, Unigene11998\_Sample\_011046841, Unigene52326\_Sample\_011046841, Unigene41388\_Sample\_011046841, Unigene49083\_Sample\_011046841, Unigene58224\_Sample\_011046841, Unigene8047\_Sample\_011046841, Unigene55511\_Sample\_011046841, Unigene60318\_Sample\_011046841, Unigene17067\_Sample\_011046841, Unigene6709\_Sample\_011046841, Unigene39966\_Sample\_011046841, Unigene8401\_Sample\_011046841, Unigene55856\_Sample\_011046841, Unigene38701\_Sample\_011046841, Unigene54697\_Sample\_011046841, Unigene1989\_Sample\_011046841, Unigene44849\_Sample\_011046841, Unigene5018\_Sample\_011046841, Unigene38750\_Sample\_011046841, Unigene54985\_Sample\_011046841, Unigene34152\_Sample\_011046841, Unigene10496\_Sample\_011046841, Unigene46184\_Sample\_011046841, Unigene37565\_Sample\_011046841, Unigene22614\_Sample\_011046841, Unigene16356\_Sample\_011046841, Unigene54528\_Sample\_011046841, Unigene30530\_Sample\_011046841, Unigene27440\_Sample\_011046841, Unigene59021\_Sample\_011046841, Unigene55702\_Sample\_011046841, Unigene8034\_Sample\_011046841, Unigene51174\_Sample\_011046841, Unigene11726\_Sample\_011046841, Unigene56863\_Sample\_011046841, Unigene2518\_Sample\_011046841, Unigene56191\_Sample\_011046841, Unigene6153\_Sample\_011046841, Unigene40296\_Sample\_011046841, Unigene51626\_Sample\_011046841, Unigene58043\_Sample\_011046841, Unigene40423\_Sample\_011046841, Unigene7206\_Sample\_011046841, Unigene21213\_Sample\_011046841, Unigene6497\_Sample\_011046841, Unigene9672\_Sample\_011046841, Unigene48334\_Sample\_011046841, Unigene39553\_Sample\_011046841, Unigene55781\_Sample\_011046841, Unigene13902\_Sample\_011046841, Unigene8251\_Sample\_011046841, Unigene40344\_Sample\_011046841, Unigene39787\_Sample\_011046841, Unigene55466\_Sample\_011046841, Unigene11093\_Sample\_011046841, Unigene41884\_Sample\_011046841, Unigene59914\_Sample\_011046841, Unigene58431\_Sample\_011046841, Unigene8590\_Sample\_011046841, Unigene51125\_Sample\_011046841, Unigene10077\_Sample\_011046841, Unigene60095\_Sample\_011046841, Unigene51100\_Sample\_011046841, Unigene48473\_Sample\_011046841, Unigene58528\_Sample\_011046841, Unigene43294\_Sample\_011046841, Unigene59747\_Sample\_011046841, Unigene13669\_Sample\_011046841, Unigene52249\_Sample\_011046841, Unigene50207\_Sample\_011046841, Unigene3252\_Sample\_011046841, Unigene55131\_Sample\_011046841, Unigene6719\_Sample\_011046841, Unigene993\_Sample\_011046841, Unigene56391\_Sample\_011046841, Unigene6590\_Sample\_011046841, Unigene57871\_Sample\_011046841, Unigene50141\_Sample\_011046841, Unigene43426\_Sample\_011046841, Unigene30939\_Sample\_011046841, Unigene56715\_Sample\_011046841, Unigene12223\_Sample\_011046841, Unigene23431\_Sample\_011046841, Unigene55900\_Sample\_011046841, Unigene60847\_Sample\_011046841, Unigene12700\_Sample\_011046841, Unigene26403\_Sample\_011046841, Unigene10289\_Sample\_011046841, Unigene47959\_Sample\_011046841, Unigene21756\_Sample\_011046841, Unigene55700\_Sample\_011046841, Unigene19526\_Sample\_011046841, Unigene31905\_Sample\_011046841, Unigene44880\_Sample\_011046841, Unigene4364\_Sample\_011046841, Unigene50296\_Sample\_011046841, Unigene57929\_Sample\_011046841, Unigene55995\_Sample\_011046841, Unigene24152\_Sample\_011046841, Unigene42559\_Sample\_011046841, Unigene52802\_Sample\_011046841, Unigene46198\_Sample\_011046841, Unigene42123\_Sample\_011046841, Unigene40609\_Sample\_011046841, Unigene60319\_Sample\_011046841, Unigene5054\_Sample\_011046841, Unigene58487\_Sample\_011046841, Unigene56437\_Sample\_011046841, Unigene53846\_Sample\_011046841, Unigene56027\_Sample\_011046841, Unigene58649\_Sample\_011046841, Unigene60689\_Sample\_011046841, Unigene16098\_Sample\_011046841, Unigene13367\_Sample\_011046841, Unigene29345\_Sample\_011046841, Unigene8229\_Sample\_011046841, Unigene40953\_Sample\_011046841, Unigene9107\_Sample\_011046841, Unigene50660\_Sample\_011046841, Unigene18715\_Sample\_011046841, Unigene8931\_Sample\_011046841, Unigene53614\_Sample\_011046841, Unigene58238\_Sample\_011046841, Unigene19792\_Sample\_011046841, Unigene8641\_Sample\_011046841, Unigene26928\_Sample\_011046841, Unigene26183\_Sample\_011046841, Unigene4497\_Sample\_011046841, Unigene55799\_Sample\_011046841, Unigene11923\_Sample\_011046841, Unigene59946\_Sample\_011046841, Unigene41236\_Sample\_011046841, Unigene6502\_Sample\_011046841, Unigene55384\_Sample\_011046841, Unigene38874\_Sample\_011046841, Unigene43906\_Sample\_011046841, Unigene5150\_Sample\_011046841, Unigene10013\_Sample\_011046841, Unigene57255\_Sample\_011046841, Unigene42218\_Sample\_011046841, Unigene54217\_Sample\_011046841, Unigene55342\_Sample\_011046841, Unigene58511\_Sample\_011046841, Unigene56559\_Sample\_011046841, Unigene55379\_Sample\_011046841, Unigene4883\_Sample\_011046841, Unigene12871\_Sample\_011046841, Unigene57573\_Sample\_011046841, Unigene2834\_Sample\_011046841, Unigene55809\_Sample\_011046841, Unigene44703\_Sample\_011046841, Unigene48375\_Sample\_011046841, Unigene57490\_Sample\_011046841, Unigene43509\_Sample\_011046841, Unigene19912\_Sample\_011046841, Unigene37440\_Sample\_011046841, Unigene40921\_Sample\_011046841, Unigene16777\_Sample\_011046841, Unigene55221\_Sample\_011046841, Unigene59493\_Sample\_011046841, Unigene42259\_Sample\_011046841, Unigene56530\_Sample\_011046841, Unigene55226\_Sample\_011046841, Unigene55543\_Sample\_011046841, Unigene58571\_Sample\_011046841, Unigene37696\_Sample\_011046841, Unigene49359\_Sample\_011046841, Unigene48095\_Sample\_011046841, Unigene11284\_Sample\_011046841, Unigene59633\_Sample\_011046841, Unigene38993\_Sample\_011046841, Unigene9459\_Sample\_011046841, Unigene59478\_Sample\_011046841, Unigene44985\_Sample\_011046841, Unigene55254\_Sample\_011046841, Unigene33973\_Sample\_011046841, Unigene10187\_Sample\_011046841, Unigene47674\_Sample\_011046841, Unigene7516\_Sample\_011046841, Unigene45808\_Sample\_011046841, Unigene43064\_Sample\_011046841, Unigene54795\_Sample\_011046841, Unigene11715\_Sample\_011046841, Unigene44551\_Sample\_011046841, Unigene46719\_Sample\_011046841, Unigene58827\_Sample\_011046841, Unigene29839\_Sample\_011046841, Unigene45303\_Sample\_011046841, Unigene51374\_Sample\_011046841, Unigene8519\_Sample\_011046841, Unigene38143\_Sample\_011046841, Unigene52288\_Sample\_011046841, Unigene13879\_Sample\_011046841, Unigene60377\_Sample\_011046841, Unigene25319\_Sample\_011046841, Unigene49752\_Sample\_011046841, Unigene31264\_Sample\_011046841, Unigene49014\_Sample\_011046841, Unigene59195\_Sample\_011046841, Unigene8987\_Sample\_011046841, Unigene46366\_Sample\_011046841, Unigene23984\_Sample\_011046841, Unigene36851\_Sample\_011046841, Unigene9307\_Sample\_011046841, Unigene31416\_Sample\_011046841, Unigene39807\_Sample\_011046841, Unigene57947\_Sample\_011046841, Unigene27139\_Sample\_011046841, Unigene15136\_Sample\_011046841, Unigene2913\_Sample\_011046841, Unigene13256\_Sample\_011046841, Unigene983\_Sample\_011046841, Unigene44730\_Sample\_011046841, Unigene45371\_Sample\_011046841, Unigene31679\_Sample\_011046841, Unigene21818\_Sample\_011046841, Unigene26119\_Sample\_011046841, Unigene5264\_Sample\_011046841, Unigene60486\_Sample\_011046841, Unigene56587\_Sample\_011046841, Unigene12115\_Sample\_011046841, Unigene39762\_Sample\_011046841, Unigene25597\_Sample\_011046841, Unigene54165\_Sample\_011046841, Unigene21535\_Sample\_011046841, Unigene59379\_Sample\_011046841, Unigene45635\_Sample\_011046841, Unigene7296\_Sample\_011046841, Unigene43456\_Sample\_011046841, Unigene34414\_Sample\_011046841, Unigene51178\_Sample\_011046841, Unigene57118\_Sample\_011046841, Unigene49793\_Sample\_011046841, Unigene59791\_Sample\_011046841, Unigene58715\_Sample\_011046841, Unigene41859\_Sample\_011046841, Unigene18444\_Sample\_011046841, Unigene12762\_Sample\_011046841, Unigene47562\_Sample\_011046841, Unigene3034\_Sample\_011046841, Unigene23762\_Sample\_011046841, Unigene33118\_Sample\_011046841, Unigene6097\_Sample\_011046841, Unigene58519\_Sample\_011046841, Unigene2398\_Sample\_011046841, Unigene55036\_Sample\_011046841, Unigene3881\_Sample\_011046841, Unigene42893\_Sample\_011046841, Unigene13776\_Sample\_011046841, Unigene29330\_Sample\_011046841, Unigene46653\_Sample\_011046841, Unigene27530\_Sample\_011046841, Unigene10962\_Sample\_011046841, Unigene12145\_Sample\_011046841, Unigene33542\_Sample\_011046841, Unigene43793\_Sample\_011046841, Unigene49258\_Sample\_011046841, Unigene24493\_Sample\_011046841, Unigene52997\_Sample\_011046841, Unigene36585\_Sample\_011046841, Unigene47868\_Sample\_011046841, Unigene52077\_Sample\_011046841, Unigene11794\_Sample\_011046841, Unigene42159\_Sample\_011046841, Unigene56914\_Sample\_011046841, Unigene44244\_Sample\_011046841, Unigene18426\_Sample\_011046841, Unigene57070\_Sample\_011046841, Unigene48524\_Sample\_011046841, Unigene19943\_Sample\_011046841, Unigene59437\_Sample\_011046841, Unigene44362\_Sample\_011046841, Unigene41392\_Sample\_011046841, Unigene50285\_Sample\_011046841, Unigene11716\_Sample\_011046841, Unigene9623\_Sample\_011046841, Unigene19043\_Sample\_011046841, Unigene13011\_Sample\_011046841, Unigene60097\_Sample\_011046841, Unigene13325\_Sample\_011046841, Unigene23745\_Sample\_011046841, Unigene51627\_Sample\_011046841, Unigene22650\_Sample\_011046841, Unigene3247\_Sample\_011046841, Unigene7693\_Sample\_011046841, Unigene32028\_Sample\_011046841, Unigene12869\_Sample\_011046841, Unigene54962\_Sample\_011046841, Unigene21520\_Sample\_011046841, Unigene57124\_Sample\_011046841, Unigene16650\_Sample\_011046841, Unigene7092\_Sample\_011046841, Unigene6178\_Sample\_011046841, Unigene55921\_Sample\_011046841, Unigene52322\_Sample\_011046841, Unigene2963\_Sample\_011046841, Unigene39991\_Sample\_011046841, Unigene4064\_Sample\_011046841, Unigene6052\_Sample\_011046841, Unigene43713\_Sample\_011046841, Unigene58480\_Sample\_011046841, Unigene59512\_Sample\_011046841, Unigene56611\_Sample\_011046841, Unigene1441\_Sample\_011046841, Unigene60284\_Sample\_011046841, Unigene59971\_Sample\_011046841, Unigene58403\_Sample\_011046841, Unigene23671\_Sample\_011046841, Unigene60753\_Sample\_011046841, Unigene49622\_Sample\_011046841, Unigene60783\_Sample\_011046841, Unigene42323\_Sample\_011046841, Unigene53054\_Sample\_011046841, Unigene41393\_Sample\_011046841, Unigene29101\_Sample\_011046841, Unigene23154\_Sample\_011046841, Unigene27921\_Sample\_011046841, Unigene50996\_Sample\_011046841, Unigene2762\_Sample\_011046841, Unigene29653\_Sample\_011046841, Unigene38522\_Sample\_011046841, Unigene56835\_Sample\_011046841, Unigene52652\_Sample\_011046841, Unigene17449\_Sample\_011046841, Unigene33400\_Sample\_011046841, Unigene49268\_Sample\_011046841, Unigene22564\_Sample\_011046841, Unigene57158\_Sample\_011046841, Unigene16834\_Sample\_011046841, Unigene53915\_Sample\_011046841, Unigene36298\_Sample\_011046841, Unigene50495\_Sample\_011046841, Unigene11180\_Sample\_011046841, Unigene53333\_Sample\_011046841, Unigene33017\_Sample\_011046841, Unigene52832\_Sample\_011046841, Unigene18301\_Sample\_011046841, Unigene56413\_Sample\_011046841, Unigene36252\_Sample\_011046841, Unigene36677\_Sample\_011046841, Unigene54607\_Sample\_011046841, Unigene35603\_Sample\_011046841, Unigene20344\_Sample\_011046841, Unigene60309\_Sample\_011046841, Unigene58166\_Sample\_011046841, Unigene53714\_Sample\_011046841, Unigene21655\_Sample\_011046841, Unigene24335\_Sample\_011046841, Unigene56645\_Sample\_011046841, Unigene53563\_Sample\_011046841, Unigene13607\_Sample\_011046841, Unigene58082\_Sample\_011046841, Unigene6377\_Sample\_011046841, Unigene48701\_Sample\_011046841, Unigene41401\_Sample\_011046841, Unigene50653\_Sample\_011046841, Unigene47036\_Sample\_011046841, Unigene60381\_Sample\_011046841, Unigene20302\_Sample\_011046841, Unigene50329\_Sample\_011046841, Unigene33843\_Sample\_011046841, Unigene34881\_Sample\_011046841, Unigene13407\_Sample\_011046841, Unigene32077\_Sample\_011046841, Unigene24582\_Sample\_011046841, Unigene59535\_Sample\_011046841, Unigene24726\_Sample\_011046841, Unigene29466\_Sample\_011046841, Unigene2493\_Sample\_011046841, Unigene60526\_Sample\_011046841, Unigene13431\_Sample\_011046841, Unigene52427\_Sample\_011046841, Unigene57784\_Sample\_011046841, Unigene54293\_Sample\_011046841, Unigene41644\_Sample\_011046841, Unigene1952\_Sample\_011046841, Unigene48020\_Sample\_011046841, Unigene5340\_Sample\_011046841, Unigene35850\_Sample\_011046841, Unigene13636\_Sample\_011046841, Unigene41676\_Sample\_011046841, Unigene43380\_Sample\_011046841, Unigene59859\_Sample\_011046841, Unigene9668\_Sample\_011046841, Unigene55420\_Sample\_011046841, Unigene25573\_Sample\_011046841, Unigene54402\_Sample\_011046841, Unigene22741\_Sample\_011046841, Unigene28781\_Sample\_011046841, Unigene50555\_Sample\_011046841, Unigene15838\_Sample\_011046841, Unigene59545\_Sample\_011046841, Unigene56163\_Sample\_011046841, Unigene48091\_Sample\_011046841, Unigene56276\_Sample\_011046841, Unigene55314\_Sample\_011046841, Unigene39096\_Sample\_011046841, Unigene52822\_Sample\_011046841, Unigene53910\_Sample\_011046841, Unigene58132\_Sample\_011046841, Unigene60102\_Sample\_011046841, Unigene21132\_Sample\_011046841, Unigene59139\_Sample\_011046841, Unigene55618\_Sample\_011046841, Unigene16257\_Sample\_011046841, Unigene13244\_Sample\_011046841, Unigene60693\_Sample\_011046841, Unigene55196\_Sample\_011046841, Unigene6629\_Sample\_011046841, Unigene17735\_Sample\_011046841, Unigene57690\_Sample\_011046841, Unigene29847\_Sample\_011046841, Unigene5154\_Sample\_011046841, Unigene48086\_Sample\_011046841, Unigene57706\_Sample\_011046841, Unigene59492\_Sample\_011046841, Unigene44734\_Sample\_011046841, Unigene20619\_Sample\_011046841, Unigene29551\_Sample\_011046841, Unigene2415\_Sample\_011046841, Unigene30349\_Sample\_011046841, Unigene30950\_Sample\_011046841, Unigene2532\_Sample\_011046841, Unigene39766\_Sample\_011046841, Unigene31335\_Sample\_011046841, Unigene11029\_Sample\_011046841, Unigene22216\_Sample\_011046841, Unigene250\_Sample\_011046841, Unigene40544\_Sample\_011046841, Unigene5951\_Sample\_011046841, Unigene41694\_Sample\_011046841, Unigene30681\_Sample\_011046841, Unigene20157\_Sample\_011046841, Unigene11939\_Sample\_011046841, Unigene53420\_Sample\_011046841, Unigene37456\_Sample\_011046841, Unigene26329\_Sample\_011046841, Unigene52796\_Sample\_011046841, Unigene51179\_Sample\_011046841, Unigene57806\_Sample\_011046841, Unigene57215\_Sample\_011046841, Unigene58593\_Sample\_011046841, Unigene36816\_Sample\_011046841, Unigene12670\_Sample\_011046841, Unigene11846\_Sample\_011046841, Unigene52824\_Sample\_011046841, Unigene37037\_Sample\_011046841, Unigene59653\_Sample\_011046841, Unigene14799\_Sample\_011046841, Unigene59637\_Sample\_011046841, Unigene51802\_Sample\_011046841, Unigene2861\_Sample\_011046841, Unigene54407\_Sample\_011046841, Unigene50902\_Sample\_011046841, Unigene46773\_Sample\_011046841, Unigene55465\_Sample\_011046841, Unigene8962\_Sample\_011046841, Unigene54692\_Sample\_011046841, Unigene26954\_Sample\_011046841, Unigene56144\_Sample\_011046841, Unigene34417\_Sample\_011046841, Unigene59718\_Sample\_011046841, Unigene50103\_Sample\_011046841, Unigene54112\_Sample\_011046841, Unigene59849\_Sample\_011046841, Unigene58564\_Sample\_011046841, Unigene55564\_Sample\_011046841, Unigene59460\_Sample\_011046841, Unigene51702\_Sample\_011046841, Unigene33316\_Sample\_011046841, Unigene8713\_Sample\_011046841, Unigene39125\_Sample\_011046841, Unigene5391\_Sample\_011046841, Unigene56726\_Sample\_011046841, Unigene60378\_Sample\_011046841, Unigene59560\_Sample\_011046841, Unigene27178\_Sample\_011046841, Unigene49211\_Sample\_011046841, Unigene60247\_Sample\_011046841, Unigene59678\_Sample\_011046841, Unigene54791\_Sample\_011046841, Unigene33791\_Sample\_011046841, Unigene60665\_Sample\_011046841, Unigene47421\_Sample\_011046841, Unigene38304\_Sample\_011046841, Unigene22525\_Sample\_011046841, Unigene51599\_Sample\_011046841, Unigene29462\_Sample\_011046841, Unigene58192\_Sample\_011046841, Unigene11699\_Sample\_011046841, Unigene324\_Sample\_011046841, Unigene54297\_Sample\_011046841, Unigene55533\_Sample\_011046841, Unigene49021\_Sample\_011046841, Unigene58659\_Sample\_011046841, Unigene58488\_Sample\_011046841, Unigene44504\_Sample\_011046841, Unigene48690\_Sample\_011046841, Unigene49608\_Sample\_011046841, Unigene54240\_Sample\_011046841, Unigene16329\_Sample\_011046841, Unigene54683\_Sample\_011046841, Unigene46030\_Sample\_011046841, Unigene57540\_Sample\_011046841, Unigene11465\_Sample\_011046841, Unigene10973\_Sample\_011046841, Unigene58135\_Sample\_011046841, Unigene46220\_Sample\_011046841, Unigene51688\_Sample\_011046841, Unigene40435\_Sample\_011046841, Unigene36154\_Sample\_011046841, Unigene55325\_Sample\_011046841, Unigene12261\_Sample\_011046841, Unigene56719\_Sample\_011046841, Unigene16692\_Sample\_011046841, Unigene50915\_Sample\_011046841, Unigene39075\_Sample\_011046841, Unigene51716\_Sample\_011046841, Unigene50563\_Sample\_011046841, Unigene53640\_Sample\_011046841, Unigene56466\_Sample\_011046841, Unigene55474\_Sample\_011046841, Unigene10517\_Sample\_011046841, Unigene20789\_Sample\_011046841, Unigene6432\_Sample\_011046841, Unigene40742\_Sample\_011046841, Unigene42293\_Sample\_011046841, Unigene27486\_Sample\_011046841, Unigene55975\_Sample\_011046841, Unigene59688\_Sample\_011046841, Unigene54625\_Sample\_011046841, Unigene35438\_Sample\_011046841, Unigene39712\_Sample\_011046841, Unigene10425\_Sample\_011046841, Unigene59585\_Sample\_011046841, Unigene59926\_Sample\_011046841, Unigene44834\_Sample\_011046841, Unigene5329\_Sample\_011046841, Unigene25498\_Sample\_011046841, Unigene57861\_Sample\_011046841, Unigene24028\_Sample\_011046841, Unigene50893\_Sample\_011046841, Unigene42231\_Sample\_011046841, Unigene57832\_Sample\_011046841, Unigene45374\_Sample\_011046841, Unigene41847\_Sample\_011046841, Unigene32474\_Sample\_011046841, Unigene46058\_Sample\_011046841, Unigene22433\_Sample\_011046841, Unigene55311\_Sample\_011046841, Unigene18925\_Sample\_011046841, Unigene42681\_Sample\_011046841, Unigene1885\_Sample\_011046841, Unigene60490\_Sample\_011046841, Unigene60057\_Sample\_011046841, Unigene21838\_Sample\_011046841, Unigene28976\_Sample\_011046841, Unigene32869\_Sample\_011046841, Unigene9274\_Sample\_011046841, Unigene48943\_Sample\_011046841, Unigene37462\_Sample\_011046841, Unigene10522\_Sample\_011046841, Unigene46254\_Sample\_011046841, Unigene48425\_Sample\_011046841, Unigene13722\_Sample\_011046841, Unigene47309\_Sample\_011046841, Unigene8113\_Sample\_011046841, Unigene33405\_Sample\_011046841, Unigene30413\_Sample\_011046841, Unigene40851\_Sample\_011046841, Unigene24161\_Sample\_011046841, Unigene6254\_Sample\_011046841, Unigene23416\_Sample\_011046841, Unigene42399\_Sample\_011046841, Unigene58804\_Sample\_011046841, Unigene51852\_Sample\_011046841, Unigene5462\_Sample\_011046841, Unigene57570\_Sample\_011046841, Unigene12384\_Sample\_011046841, Unigene19793\_Sample\_011046841, Unigene7905\_Sample\_011046841, Unigene21362\_Sample\_011046841, Unigene48867\_Sample\_011046841, Unigene56266\_Sample\_011046841, Unigene12206\_Sample\_011046841, Unigene59176\_Sample\_011046841, Unigene57889\_Sample\_011046841, Unigene6327\_Sample\_011046841, Unigene12149\_Sample\_011046841, Unigene7335\_Sample\_011046841, Unigene36791\_Sample\_011046841, Unigene57773\_Sample\_011046841, Unigene54218\_Sample\_011046841, Unigene51468\_Sample\_011046841, Unigene59396\_Sample\_011046841, Unigene55863\_Sample\_011046841, Unigene3810\_Sample\_011046841, Unigene49921\_Sample\_011046841, Unigene21962\_Sample\_011046841, Unigene12521\_Sample\_011046841, Unigene23477\_Sample\_011046841, Unigene48689\_Sample\_011046841, Unigene53073\_Sample\_011046841, Unigene11419\_Sample\_011046841, Unigene43623\_Sample\_011046841, Unigene42576\_Sample\_011046841, Unigene30743\_Sample\_011046841, Unigene21128\_Sample\_011046841, Unigene53241\_Sample\_011046841, Unigene40800\_Sample\_011046841, Unigene37042\_Sample\_011046841, Unigene7966\_Sample\_011046841, Unigene1194\_Sample\_011046841, Unigene44944\_Sample\_011046841, Unigene25755\_Sample\_011046841, Unigene57130\_Sample\_011046841, Unigene60375\_Sample\_011046841, Unigene57009\_Sample\_011046841, Unigene52176\_Sample\_011046841, Unigene52381\_Sample\_011046841, Unigene50926\_Sample\_011046841, Unigene31194\_Sample\_011046841, Unigene10438\_Sample\_011046841, Unigene35285\_Sample\_011046841, Unigene13433\_Sample\_011046841, Unigene34403\_Sample\_011046841, Unigene45472\_Sample\_011046841, Unigene13817\_Sample\_011046841, Unigene44237\_Sample\_011046841, Unigene55546\_Sample\_011046841, Unigene45740\_Sample\_011046841, Unigene60914\_Sample\_011046841, Unigene59357\_Sample\_011046841, Unigene56904\_Sample\_011046841, Unigene5867\_Sample\_011046841, Unigene1793\_Sample\_011046841, Unigene45485\_Sample\_011046841, Unigene33899\_Sample\_011046841, Unigene31097\_Sample\_011046841, Unigene11526\_Sample\_011046841, Unigene23201\_Sample\_011046841, Unigene45183\_Sample\_011046841, Unigene45310\_Sample\_011046841, Unigene1520\_Sample\_011046841, Unigene46800\_Sample\_011046841, Unigene16198\_Sample\_011046841, Unigene38130\_Sample\_011046841, Unigene57713\_Sample\_011046841, Unigene60342\_Sample\_011046841, Unigene59114\_Sample\_011046841, Unigene60780\_Sample\_011046841, Unigene52788\_Sample\_011046841, Unigene38898\_Sample\_011046841, Unigene36363\_Sample\_011046841, Unigene53773\_Sample\_011046841, Unigene51956\_Sample\_011046841, Unigene37526\_Sample\_011046841, Unigene55021\_Sample\_011046841, Unigene60515\_Sample\_011046841, Unigene10669\_Sample\_011046841, Unigene39967\_Sample\_011046841, Unigene30153\_Sample\_011046841, Unigene3342\_Sample\_011046841, Unigene12692\_Sample\_011046841, Unigene38675\_Sample\_011046841, Unigene59917\_Sample\_011046841, Unigene13852\_Sample\_011046841, Unigene23479\_Sample\_011046841, Unigene59496\_Sample\_011046841, Unigene51295\_Sample\_011046841, Unigene30309\_Sample\_011046841, Unigene30484\_Sample\_011046841, Unigene38437\_Sample\_011046841, Unigene53972\_Sample\_011046841, Unigene58014\_Sample\_011046841, Unigene5118\_Sample\_011046841, Unigene56254\_Sample\_011046841, Unigene49113\_Sample\_011046841, Unigene57572\_Sample\_011046841, Unigene46974\_Sample\_011046841, Unigene53689\_Sample\_011046841, Unigene58808\_Sample\_011046841, Unigene57272\_Sample\_011046841, Unigene10024\_Sample\_011046841, Unigene34891\_Sample\_011046841, Unigene31782\_Sample\_011046841, Unigene30044\_Sample\_011046841, Unigene57293\_Sample\_011046841, Unigene38616\_Sample\_011046841, Unigene48044\_Sample\_011046841, Unigene32987\_Sample\_011046841, Unigene48737\_Sample\_011046841, Unigene54955\_Sample\_011046841, Unigene55948\_Sample\_011046841, Unigene47460\_Sample\_011046841, Unigene54866\_Sample\_011046841, Unigene16069\_Sample\_011046841, Unigene19103\_Sample\_011046841, Unigene60478\_Sample\_011046841, Unigene54473\_Sample\_011046841, Unigene33512\_Sample\_011046841, Unigene57000\_Sample\_011046841, Unigene59816\_Sample\_011046841, Unigene60718\_Sample\_011046841, Unigene4889\_Sample\_011046841, Unigene49724\_Sample\_011046841, Unigene29201\_Sample\_011046841, Unigene47610\_Sample\_011046841, Unigene60825\_Sample\_011046841, Unigene60294\_Sample\_011046841, Unigene60764\_Sample\_011046841, Unigene26464\_Sample\_011046841, Unigene59746\_Sample\_011046841, Unigene58367\_Sample\_011046841, Unigene1887\_Sample\_011046841, Unigene49992\_Sample\_011046841, Unigene17159\_Sample\_011046841, Unigene48999\_Sample\_011046841, Unigene41088\_Sample\_011046841, Unigene35788\_Sample\_011046841, Unigene59514\_Sample\_011046841, Unigene57635\_Sample\_011046841, Unigene12983\_Sample\_011046841, Unigene54468\_Sample\_011046841, Unigene35733\_Sample\_011046841, Unigene9600\_Sample\_011046841, Unigene58952\_Sample\_011046841, Unigene11695\_Sample\_011046841, Unigene47149\_Sample\_011046841, Unigene53520\_Sample\_011046841, Unigene45818\_Sample\_011046841, Unigene40215\_Sample\_011046841, Unigene986\_Sample\_011046841, Unigene45985\_Sample\_011046841, Unigene11099\_Sample\_011046841, Unigene3412\_Sample\_011046841, Unigene53097\_Sample\_011046841, Unigene49145\_Sample\_011046841, Unigene12487\_Sample\_011046841, Unigene52695\_Sample\_011046841, Unigene10745\_Sample\_011046841, Unigene28763\_Sample\_011046841, Unigene2570\_Sample\_011046841, Unigene48546\_Sample\_011046841, Unigene59458\_Sample\_011046841, Unigene7647\_Sample\_011046841, Unigene10037\_Sample\_011046841, Unigene54648\_Sample\_011046841, Unigene21246\_Sample\_011046841, Unigene31129\_Sample\_011046841, Unigene58114\_Sample\_011046841, Unigene60916\_Sample\_011046841, Unigene10881\_Sample\_011046841, Unigene24046\_Sample\_011046841, Unigene47158\_Sample\_011046841, Unigene54198\_Sample\_011046841, Unigene25961\_Sample\_011046841, Unigene58518\_Sample\_011046841, Unigene19441\_Sample\_011046841, Unigene42326\_Sample\_011046841, Unigene17084\_Sample\_011046841, Unigene9259\_Sample\_011046841, Unigene37448\_Sample\_011046841, Unigene9774\_Sample\_011046841, Unigene56982\_Sample\_011046841, Unigene24979\_Sample\_011046841, Unigene57667\_Sample\_011046841, Unigene53062\_Sample\_011046841, Unigene34027\_Sample\_011046841, Unigene58031\_Sample\_011046841, Unigene55486\_Sample\_011046841, Unigene8850\_Sample\_011046841, Unigene59673\_Sample\_011046841, Unigene55068\_Sample\_011046841, Unigene59814\_Sample\_011046841, Unigene56869\_Sample\_011046841, Unigene12446\_Sample\_011046841, Unigene60218\_Sample\_011046841, Unigene51736\_Sample\_011046841, Unigene45951\_Sample\_011046841, Unigene9064\_Sample\_011046841, Unigene41828\_Sample\_011046841, Unigene37683\_Sample\_011046841, Unigene12338\_Sample\_011046841, Unigene38011\_Sample\_011046841, Unigene58895\_Sample\_011046841, Unigene39189\_Sample\_011046841, Unigene12195\_Sample\_011046841, Unigene54941\_Sample\_011046841, Unigene7766\_Sample\_011046841, Unigene56913\_Sample\_011046841, Unigene24706\_Sample\_011046841, Unigene58049\_Sample\_011046841, Unigene50919\_Sample\_011046841, Unigene55555\_Sample\_011046841, Unigene2174\_Sample\_011046841, Unigene53149\_Sample\_011046841, Unigene55348\_Sample\_011046841, Unigene41178\_Sample\_011046841, Unigene24065\_Sample\_011046841, Unigene17671\_Sample\_011046841, Unigene26069\_Sample\_011046841, Unigene59588\_Sample\_011046841, Unigene37777\_Sample\_011046841, Unigene59292\_Sample\_011046841, Unigene50662\_Sample\_011046841, Unigene21921\_Sample\_011046841, Unigene57188\_Sample\_011046841, Unigene33513\_Sample\_011046841, Unigene19767\_Sample\_011046841, Unigene49399\_Sample\_011046841, Unigene53172\_Sample\_011046841, Unigene25630\_Sample\_011046841, Unigene10283\_Sample\_011046841, Unigene17090\_Sample\_011046841, Unigene50487\_Sample\_011046841, Unigene4863\_Sample\_011046841, Unigene56807\_Sample\_011046841, Unigene46224\_Sample\_011046841, Unigene21855\_Sample\_011046841, Unigene60028\_Sample\_011046841, Unigene6085\_Sample\_011046841, Unigene42565\_Sample\_011046841, Unigene36915\_Sample\_011046841, Unigene713\_Sample\_011046841, Unigene4741\_Sample\_011046841, Unigene13774\_Sample\_011046841, Unigene32563\_Sample\_011046841, Unigene60924\_Sample\_011046841, Unigene12346\_Sample\_011046841, Unigene59910\_Sample\_011046841, Unigene52673\_Sample\_011046841, Unigene29723\_Sample\_011046841, Unigene10260\_Sample\_011046841, Unigene11120\_Sample\_011046841, Unigene41700\_Sample\_011046841, Unigene56661\_Sample\_011046841, Unigene55044\_Sample\_011046841, Unigene32116\_Sample\_011046841, Unigene59972\_Sample\_011046841, Unigene24326\_Sample\_011046841, Unigene28131\_Sample\_011046841, Unigene60645\_Sample\_011046841, Unigene4256\_Sample\_011046841, Unigene5964\_Sample\_011046841, Unigene37357\_Sample\_011046841, Unigene5058\_Sample\_011046841, Unigene47723\_Sample\_011046841, Unigene57083\_Sample\_011046841, Unigene24586\_Sample\_011046841, Unigene59381\_Sample\_011046841, Unigene44447\_Sample\_011046841, Unigene56821\_Sample\_011046841, Unigene57207\_Sample\_011046841, Unigene50963\_Sample\_011046841, Unigene37225\_Sample\_011046841, Unigene20439\_Sample\_011046841, Unigene58164\_Sample\_011046841, Unigene58942\_Sample\_011046841, Unigene16520\_Sample\_011046841, Unigene4709\_Sample\_011046841, Unigene28571\_Sample\_011046841, Unigene39537\_Sample\_011046841, Unigene41427\_Sample\_011046841, Unigene57730\_Sample\_011046841, Unigene14236\_Sample\_011046841, Unigene12769\_Sample\_011046841, Unigene52685\_Sample\_011046841, Unigene51419\_Sample\_011046841, Unigene12309\_Sample\_011046841, Unigene20715\_Sample\_011046841, Unigene56373\_Sample\_011046841, Unigene58733\_Sample\_011046841, Unigene45514\_Sample\_011046841, Unigene41574\_Sample\_011046841, Unigene54061\_Sample\_011046841, Unigene59250\_Sample\_011046841, Unigene58858\_Sample\_011046841, Unigene8182\_Sample\_011046841, Unigene51041\_Sample\_011046841, Unigene28196\_Sample\_011046841, Unigene45512\_Sample\_011046841, Unigene34589\_Sample\_011046841, Unigene47634\_Sample\_011046841, Unigene9912\_Sample\_011046841, Unigene31853\_Sample\_011046841, Unigene23155\_Sample\_011046841, Unigene23742\_Sample\_011046841, Unigene57140\_Sample\_011046841, Unigene57828\_Sample\_011046841, Unigene49932\_Sample\_011046841, Unigene59629\_Sample\_011046841, Unigene16146\_Sample\_011046841, Unigene22671\_Sample\_011046841, Unigene13748\_Sample\_011046841, Unigene36888\_Sample\_011046841, Unigene5195\_Sample\_011046841, Unigene43826\_Sample\_011046841, Unigene49168\_Sample\_011046841, Unigene13391\_Sample\_011046841, Unigene43028\_Sample\_011046841, Unigene25267\_Sample\_011046841, Unigene14396\_Sample\_011046841, Unigene55449\_Sample\_011046841, Unigene36850\_Sample\_011046841, Unigene60944\_Sample\_011046841, Unigene5223\_Sample\_011046841, Unigene51962\_Sample\_011046841, Unigene54134\_Sample\_011046841, Unigene41848\_Sample\_011046841, Unigene3481\_Sample\_011046841, Unigene7366\_Sample\_011046841, Unigene59985\_Sample\_011046841, Unigene4851\_Sample\_011046841, Unigene57435\_Sample\_011046841, Unigene58298\_Sample\_011046841, Unigene7778\_Sample\_011046841, Unigene12701\_Sample\_011046841, Unigene25098\_Sample\_011046841, Unigene42229\_Sample\_011046841, Unigene32138\_Sample\_011046841, Unigene48049\_Sample\_011046841, Unigene38146\_Sample\_011046841, Unigene4847\_Sample\_011046841, Unigene55402\_Sample\_011046841, Unigene58777\_Sample\_011046841, Unigene38021\_Sample\_011046841, Unigene47448\_Sample\_011046841, Unigene60677\_Sample\_011046841, Unigene32309\_Sample\_011046841, Unigene144\_Sample\_011046841, Unigene24221\_Sample\_011046841, Unigene37820\_Sample\_011046841, Unigene27727\_Sample\_011046841, Unigene31588\_Sample\_011046841, Unigene13768\_Sample\_011046841, Unigene54248\_Sample\_011046841, Unigene57384\_Sample\_011046841, Unigene4117\_Sample\_011046841, Unigene46363\_Sample\_011046841, Unigene44249\_Sample\_011046841, Unigene5099\_Sample\_011046841, Unigene24807\_Sample\_011046841, Unigene17785\_Sample\_011046841, Unigene46697\_Sample\_011046841, Unigene11028\_Sample\_011046841, Unigene42967\_Sample\_011046841, Unigene53100\_Sample\_011046841, Unigene2534\_Sample\_011046841, Unigene54425\_Sample\_011046841, Unigene59622\_Sample\_011046841, Unigene26679\_Sample\_011046841, Unigene53067\_Sample\_011046841, Unigene53691\_Sample\_011046841, Unigene41336\_Sample\_011046841, Unigene48540\_Sample\_011046841, Unigene60049\_Sample\_011046841, Unigene44069\_Sample\_011046841, Unigene55512\_Sample\_011046841, Unigene58374\_Sample\_011046841, Unigene43941\_Sample\_011046841, Unigene50910\_Sample\_011046841, Unigene38586\_Sample\_011046841, Unigene48764\_Sample\_011046841, Unigene10336\_Sample\_011046841, Unigene44009\_Sample\_011046841, Unigene60746\_Sample\_011046841, Unigene47908\_Sample\_011046841, Unigene54276\_Sample\_011046841, Unigene3527\_Sample\_011046841, Unigene51264\_Sample\_011046841, Unigene21701\_Sample\_011046841, Unigene46791\_Sample\_011046841, Unigene42062\_Sample\_011046841, Unigene57100\_Sample\_011046841, Unigene53565\_Sample\_011046841, Unigene13651\_Sample\_011046841, Unigene48637\_Sample\_011046841, Unigene18385\_Sample\_011046841, Unigene21907\_Sample\_011046841, Unigene49725\_Sample\_011046841, Unigene19495\_Sample\_011046841, Unigene55252\_Sample\_011046841, Unigene38694\_Sample\_011046841, Unigene7789\_Sample\_011046841, Unigene60809\_Sample\_011046841, Unigene34380\_Sample\_011046841, Unigene8612\_Sample\_011046841, Unigene8165\_Sample\_011046841, Unigene45838\_Sample\_011046841, Unigene6901\_Sample\_011046841, Unigene60701\_Sample\_011046841, Unigene34404\_Sample\_011046841, Unigene53741\_Sample\_011046841, Unigene33130\_Sample\_011046841, Unigene8392\_Sample\_011046841, Unigene21063\_Sample\_011046841, Unigene17370\_Sample\_011046841, Unigene45342\_Sample\_011046841, Unigene30385\_Sample\_011046841, Unigene4871\_Sample\_011046841, Unigene7303\_Sample\_011046841, Unigene51077\_Sample\_011046841, Unigene43875\_Sample\_011046841, Unigene54751\_Sample\_011046841, Unigene1181\_Sample\_011046841, Unigene53757\_Sample\_011046841, Unigene58077\_Sample\_011046841, Unigene40807\_Sample\_011046841, Unigene58839\_Sample\_011046841, Unigene53327\_Sample\_011046841, Unigene60801\_Sample\_011046841, Unigene3901\_Sample\_011046841, Unigene11522\_Sample\_011046841, Unigene51282\_Sample\_011046841, Unigene28658\_Sample\_011046841, Unigene31664\_Sample\_011046841, Unigene3363\_Sample\_011046841, Unigene51696\_Sample\_011046841, Unigene48786\_Sample\_011046841, Unigene12056\_Sample\_011046841, Unigene13514\_Sample\_011046841, Unigene60704\_Sample\_011046841, Unigene57429\_Sample\_011046841, Unigene58917\_Sample\_011046841, Unigene23180\_Sample\_011046841, Unigene33111\_Sample\_011046841, Unigene9610\_Sample\_011046841, Unigene9773\_Sample\_011046841, Unigene58720\_Sample\_011046841, Unigene42430\_Sample\_011046841, Unigene59322\_Sample\_011046841, Unigene50313\_Sample\_011046841, Unigene41140\_Sample\_011046841, Unigene56358\_Sample\_011046841, Unigene21284\_Sample\_011046841, Unigene40558\_Sample\_011046841, Unigene42697\_Sample\_011046841, Unigene32540\_Sample\_011046841, Unigene54525\_Sample\_011046841, Unigene22762\_Sample\_011046841, Unigene12081\_Sample\_011046841, Unigene21688\_Sample\_011046841, Unigene34034\_Sample\_011046841, Unigene53313\_Sample\_011046841, Unigene58577\_Sample\_011046841, Unigene52744\_Sample\_011046841, Unigene19047\_Sample\_011046841, Unigene56534\_Sample\_011046841, Unigene5904\_Sample\_011046841, Unigene53843\_Sample\_011046841, Unigene45012\_Sample\_011046841, Unigene22255\_Sample\_011046841, Unigene57475\_Sample\_011046841, Unigene17219\_Sample\_011046841, Unigene51879\_Sample\_011046841, Unigene15918\_Sample\_011046841, Unigene44895\_Sample\_011046841, Unigene47129\_Sample\_011046841, Unigene57298\_Sample\_011046841, Unigene52901\_Sample\_011046841, Unigene41466\_Sample\_011046841, Unigene45107\_Sample\_011046841, Unigene59984\_Sample\_011046841, Unigene46466\_Sample\_011046841, Unigene47508\_Sample\_011046841, Unigene57410\_Sample\_011046841, Unigene8126\_Sample\_011046841, Unigene39200\_Sample\_011046841, Unigene48526\_Sample\_011046841, Unigene36948\_Sample\_011046841, Unigene47981\_Sample\_011046841, Unigene60131\_Sample\_011046841, Unigene58640\_Sample\_011046841, Unigene49615\_Sample\_011046841, Unigene56344\_Sample\_011046841, Unigene47257\_Sample\_011046841, Unigene57531\_Sample\_011046841, Unigene56706\_Sample\_011046841, Unigene3946\_Sample\_011046841, Unigene37278\_Sample\_011046841, Unigene60353\_Sample\_011046841, Unigene4977\_Sample\_011046841, Unigene1012\_Sample\_011046841, Unigene12562\_Sample\_011046841, Unigene19594\_Sample\_011046841, Unigene36824\_Sample\_011046841, Unigene59189\_Sample\_011046841, Unigene28864\_Sample\_011046841, Unigene2226\_Sample\_011046841, Unigene13675\_Sample\_011046841, Unigene39209\_Sample\_011046841, Unigene11706\_Sample\_011046841, Unigene12324\_Sample\_011046841, Unigene57939\_Sample\_011046841, Unigene56460\_Sample\_011046841, Unigene51677\_Sample\_011046841, Unigene762\_Sample\_011046841, Unigene55205\_Sample\_011046841, Unigene15880\_Sample\_011046841, Unigene26904\_Sample\_011046841, Unigene28403\_Sample\_011046841, Unigene58947\_Sample\_011046841, Unigene45546\_Sample\_011046841, Unigene52565\_Sample\_011046841, Unigene55772\_Sample\_011046841, Unigene49013\_Sample\_011046841, Unigene54870\_Sample\_011046841, Unigene52591\_Sample\_011046841, Unigene7181\_Sample\_011046841, Unigene11520\_Sample\_011046841, Unigene33391\_Sample\_011046841, Unigene58475\_Sample\_011046841, Unigene47555\_Sample\_011046841, Unigene48807\_Sample\_011046841, Unigene52025\_Sample\_011046841, Unigene56714\_Sample\_011046841, Unigene42735\_Sample\_011046841, Unigene44544\_Sample\_011046841, Unigene39062\_Sample\_011046841, Unigene32703\_Sample\_011046841, Unigene47871\_Sample\_011046841, Unigene46290\_Sample\_011046841, Unigene34443\_Sample\_011046841, Unigene13390\_Sample\_011046841, Unigene36452\_Sample\_011046841, Unigene55077\_Sample\_011046841, Unigene8489\_Sample\_011046841, Unigene53706\_Sample\_011046841, Unigene53297\_Sample\_011046841, Unigene39483\_Sample\_011046841, Unigene28184\_Sample\_011046841, Unigene11804\_Sample\_011046841, Unigene39261\_Sample\_011046841, Unigene40575\_Sample\_011046841, Unigene59068\_Sample\_011046841, Unigene757\_Sample\_011046841, Unigene44282\_Sample\_011046841, Unigene13645\_Sample\_011046841, Unigene46764\_Sample\_011046841, Unigene16995\_Sample\_011046841, Unigene53367\_Sample\_011046841, Unigene41294\_Sample\_011046841, Unigene6720\_Sample\_011046841, Unigene13245\_Sample\_011046841, Unigene39074\_Sample\_011046841, Unigene3202\_Sample\_011046841, Unigene58663\_Sample\_011046841, Unigene43870\_Sample\_011046841, Unigene34039\_Sample\_011046841, Unigene20001\_Sample\_011046841, Unigene58062\_Sample\_011046841, Unigene12211\_Sample\_011046841, Unigene43216\_Sample\_011046841, Unigene12157\_Sample\_011046841, Unigene48575\_Sample\_011046841, Unigene20448\_Sample\_011046841, Unigene32707\_Sample\_011046841, Unigene24930\_Sample\_011046841, Unigene7069\_Sample\_011046841, Unigene35806\_Sample\_011046841, Unigene9753\_Sample\_011046841, Unigene58463\_Sample\_011046841, Unigene60698\_Sample\_011046841, Unigene31713\_Sample\_011046841, Unigene45440\_Sample\_011046841, Unigene40230\_Sample\_011046841, Unigene58513\_Sample\_011046841, Unigene1598\_Sample\_011046841, Unigene59973\_Sample\_011046841, Unigene32418\_Sample\_011046841, Unigene55610\_Sample\_011046841, Unigene4978\_Sample\_011046841, Unigene246\_Sample\_011046841, Unigene50734\_Sample\_011046841, Unigene11729\_Sample\_011046841, Unigene31026\_Sample\_011046841, Unigene58422\_Sample\_011046841, Unigene17921\_Sample\_011046841, Unigene12445\_Sample\_011046841, Unigene26327\_Sample\_011046841, Unigene58726\_Sample\_011046841, Unigene50242\_Sample\_011046841, Unigene25101\_Sample\_011046841, Unigene2066\_Sample\_011046841, Unigene11201\_Sample\_011046841, Unigene44876\_Sample\_011046841, Unigene38329\_Sample\_011046841, Unigene1662\_Sample\_011046841, Unigene42127\_Sample\_011046841, Unigene44086\_Sample\_011046841, Unigene34853\_Sample\_011046841, Unigene60232\_Sample\_011046841, Unigene5122\_Sample\_011046841, Unigene25547\_Sample\_011046841, Unigene23548\_Sample\_011046841, Unigene16073\_Sample\_011046841, Unigene33988\_Sample\_011046841, Unigene49661\_Sample\_011046841, Unigene56822\_Sample\_011046841, Unigene54286\_Sample\_011046841, Unigene28662\_Sample\_011046841, Unigene49803\_Sample\_011046841, Unigene10982\_Sample\_011046841, Unigene45777\_Sample\_011046841, Unigene49919\_Sample\_011046841, Unigene25357\_Sample\_011046841, Unigene52079\_Sample\_011046841, Unigene52621\_Sample\_011046841, Unigene13890\_Sample\_011046841, Unigene47083\_Sample\_011046841, Unigene47478\_Sample\_011046841, Unigene50769\_Sample\_011046841, Unigene7359\_Sample\_011046841, Unigene43654\_Sample\_011046841, Unigene47887\_Sample\_011046841, Unigene13819\_Sample\_011046841, Unigene8526\_Sample\_011046841, Unigene52542\_Sample\_011046841, Unigene56119\_Sample\_011046841, Unigene25997\_Sample\_011046841, Unigene15720\_Sample\_011046841, Unigene15374\_Sample\_011046841, Unigene55212\_Sample\_011046841, Unigene12971\_Sample\_011046841, Unigene37729\_Sample\_011046841, Unigene54368\_Sample\_011046841, Unigene43662\_Sample\_011046841, Unigene56818\_Sample\_011046841, Unigene58363\_Sample\_011046841, Unigene11148\_Sample\_011046841, Unigene41465\_Sample\_011046841, Unigene50892\_Sample\_011046841, Unigene50243\_Sample\_011046841, Unigene46841\_Sample\_011046841, Unigene10489\_Sample\_011046841, Unigene56629\_Sample\_011046841, Unigene22907\_Sample\_011046841, Unigene43240\_Sample\_011046841, Unigene49909\_Sample\_011046841, Unigene59712\_Sample\_011046841, Unigene48755\_Sample\_011046841, Unigene60349\_Sample\_011046841, Unigene517\_Sample\_011046841, Unigene23790\_Sample\_011046841, Unigene47334\_Sample\_011046841, Unigene39776\_Sample\_011046841, Unigene53719\_Sample\_011046841, Unigene56950\_Sample\_011046841, Unigene11546\_Sample\_011046841, Unigene29816\_Sample\_011046841, Unigene57184\_Sample\_011046841, Unigene33437\_Sample\_011046841, Unigene51426\_Sample\_011046841, Unigene4915\_Sample\_011046841, Unigene56503\_Sample\_011046841, Unigene13927\_Sample\_011046841, Unigene27589\_Sample\_011046841, Unigene58943\_Sample\_011046841, Unigene28537\_Sample\_011046841, Unigene48197\_Sample\_011046841, Unigene41844\_Sample\_011046841, Unigene4599\_Sample\_011046841, Unigene41220\_Sample\_011046841, Unigene45262\_Sample\_011046841, Unigene42911\_Sample\_011046841, Unigene13430\_Sample\_011046841, Unigene22154\_Sample\_011046841, Unigene39194\_Sample\_011046841, Unigene49501\_Sample\_011046841, Unigene12703\_Sample\_011046841, Unigene59987\_Sample\_011046841, Unigene39433\_Sample\_011046841, Unigene4236\_Sample\_011046841, Unigene50659\_Sample\_011046841, Unigene40989\_Sample\_011046841, Unigene3968\_Sample\_011046841, Unigene16224\_Sample\_011046841, Unigene12331\_Sample\_011046841, Unigene43481\_Sample\_011046841, Unigene35008\_Sample\_011046841, Unigene54549\_Sample\_011046841, Unigene52751\_Sample\_011046841, Unigene53926\_Sample\_011046841, Unigene49426\_Sample\_011046841, Unigene53292\_Sample\_011046841, Unigene38234\_Sample\_011046841, Unigene13656\_Sample\_011046841, Unigene55685\_Sample\_011046841, Unigene33194\_Sample\_011046841, Unigene54707\_Sample\_011046841, Unigene45004\_Sample\_011046841, Unigene49126\_Sample\_011046841, Unigene9008\_Sample\_011046841, Unigene55877\_Sample\_011046841, Unigene29396\_Sample\_011046841, Unigene47025\_Sample\_011046841, Unigene56795\_Sample\_011046841, Unigene53326\_Sample\_011046841, Unigene59151\_Sample\_011046841, Unigene37422\_Sample\_011046841, Unigene59818\_Sample\_011046841, Unigene60906\_Sample\_011046841, Unigene54037\_Sample\_011046841, Unigene60310\_Sample\_011046841, Unigene10563\_Sample\_011046841, Unigene9914\_Sample\_011046841, Unigene55879\_Sample\_011046841, Unigene1552\_Sample\_011046841, Unigene53462\_Sample\_011046841, Unigene40255\_Sample\_011046841, Unigene49650\_Sample\_011046841, Unigene46337\_Sample\_011046841, Unigene11539\_Sample\_011046841, Unigene56203\_Sample\_011046841, Unigene53018\_Sample\_011046841, Unigene13860\_Sample\_011046841, Unigene32973\_Sample\_011046841, Unigene54121\_Sample\_011046841, Unigene56579\_Sample\_011046841, Unigene11563\_Sample\_011046841, Unigene33297\_Sample\_011046841, Unigene45754\_Sample\_011046841, Unigene28739\_Sample\_011046841, Unigene24615\_Sample\_011046841, Unigene44764\_Sample\_011046841, Unigene30781\_Sample\_011046841, Unigene6757\_Sample\_011046841, Unigene13976\_Sample\_011046841, Unigene12306\_Sample\_011046841, Unigene58285\_Sample\_011046841, Unigene59137\_Sample\_011046841, Unigene30989\_Sample\_011046841, Unigene48412\_Sample\_011046841, Unigene56081\_Sample\_011046841, Unigene60822\_Sample\_011046841, Unigene44725\_Sample\_011046841, Unigene36705\_Sample\_011046841, Unigene52551\_Sample\_011046841, Unigene60036\_Sample\_011046841, Unigene51163\_Sample\_011046841, Unigene30684\_Sample\_011046841, Unigene59619\_Sample\_011046841, Unigene7507\_Sample\_011046841, Unigene40287\_Sample\_011046841, Unigene11653\_Sample\_011046841, Unigene7011\_Sample\_011046841, Unigene59539\_Sample\_011046841, Unigene60488\_Sample\_011046841, Unigene12212\_Sample\_011046841, Unigene21230\_Sample\_011046841, Unigene27108\_Sample\_011046841, Unigene55302\_Sample\_011046841, Unigene46741\_Sample\_011046841, Unigene47585\_Sample\_011046841, Unigene35902\_Sample\_011046841, Unigene44049\_Sample\_011046841, Unigene3263\_Sample\_011046841, Unigene55890\_Sample\_011046841, Unigene58210\_Sample\_011046841, Unigene6351\_Sample\_011046841, Unigene2986\_Sample\_011046841, Unigene52722\_Sample\_011046841, Unigene25820\_Sample\_011046841, Unigene57559\_Sample\_011046841, Unigene59494\_Sample\_011046841, Unigene12170\_Sample\_011046841, Unigene12906\_Sample\_011046841, Unigene50528\_Sample\_011046841, Unigene10327\_Sample\_011046841, Unigene21023\_Sample\_011046841, Unigene59541\_Sample\_011046841, Unigene60887\_Sample\_011046841, Unigene39000\_Sample\_011046841, Unigene12586\_Sample\_011046841, Unigene8502\_Sample\_011046841, Unigene53546\_Sample\_011046841, Unigene2610\_Sample\_011046841, Unigene47125\_Sample\_011046841, Unigene9517\_Sample\_011046841, Unigene55187\_Sample\_011046841, Unigene56588\_Sample\_011046841, Unigene45483\_Sample\_011046841, Unigene59702\_Sample\_011046841, Unigene30975\_Sample\_011046841, Unigene23704\_Sample\_011046841, Unigene8301\_Sample\_011046841, Unigene11478\_Sample\_011046841, Unigene56362\_Sample\_011046841, Unigene37760\_Sample\_011046841, Unigene5382\_Sample\_011046841, Unigene2105\_Sample\_011046841, Unigene60682\_Sample\_011046841, Unigene52334\_Sample\_011046841, Unigene15419\_Sample\_011046841, Unigene33766\_Sample\_011046841, Unigene92\_Sample\_011046841, Unigene34757\_Sample\_011046841, Unigene56978\_Sample\_011046841, Unigene11256\_Sample\_011046841, Unigene3148\_Sample\_011046841, Unigene6900\_Sample\_011046841, Unigene44425\_Sample\_011046841, Unigene45458\_Sample\_011046841, Unigene19407\_Sample\_011046841, Unigene41781\_Sample\_011046841, Unigene5005\_Sample\_011046841, Unigene50606\_Sample\_011046841, Unigene53456\_Sample\_011046841, Unigene48293\_Sample\_011046841, Unigene47802\_Sample\_011046841, Unigene4749\_Sample\_011046841, Unigene22182\_Sample\_011046841, Unigene4218\_Sample\_011046841, Unigene56389\_Sample\_011046841, Unigene4438\_Sample\_011046841, Unigene44939\_Sample\_011046841, Unigene31534\_Sample\_011046841, Unigene10122\_Sample\_011046841, Unigene42095\_Sample\_011046841, Unigene46754\_Sample\_011046841, Unigene59254\_Sample\_011046841, Unigene59167\_Sample\_011046841, Unigene38631\_Sample\_011046841, Unigene48963\_Sample\_011046841, Unigene7870\_Sample\_011046841, Unigene44683\_Sample\_011046841, Unigene7125\_Sample\_011046841, Unigene32450\_Sample\_011046841, Unigene57556\_Sample\_011046841, Unigene56828\_Sample\_011046841, Unigene41980\_Sample\_011046841, Unigene31228\_Sample\_011046841, Unigene48316\_Sample\_011046841, Unigene56793\_Sample\_011046841, Unigene16291\_Sample\_011046841, Unigene10325\_Sample\_011046841, Unigene40347\_Sample\_011046841, Unigene47018\_Sample\_011046841, Unigene22175\_Sample\_011046841, Unigene28643\_Sample\_011046841, Unigene10421\_Sample\_011046841, Unigene30931\_Sample\_011046841, Unigene50088\_Sample\_011046841, Unigene36037\_Sample\_011046841, Unigene31717\_Sample\_011046841, Unigene53997\_Sample\_011046841, Unigene58205\_Sample\_011046841, Unigene11168\_Sample\_011046841, Unigene251\_Sample\_011046841, Unigene11842\_Sample\_011046841, Unigene8597\_Sample\_011046841, Unigene45898\_Sample\_011046841, Unigene341\_Sample\_011046841, Unigene57770\_Sample\_011046841, Unigene5896\_Sample\_011046841, Unigene60241\_Sample\_011046841, Unigene60015\_Sample\_011046841, Unigene27250\_Sample\_011046841, Unigene4096\_Sample\_011046841, Unigene15270\_Sample\_011046841, Unigene54581\_Sample\_011046841, Unigene56605\_Sample\_011046841, Unigene6061\_Sample\_011046841, Unigene48415\_Sample\_011046841, Unigene997\_Sample\_011046841, Unigene44108\_Sample\_011046841, Unigene44635\_Sample\_011046841, Unigene54454\_Sample\_011046841, Unigene475\_Sample\_011046841, Unigene49114\_Sample\_011046841, Unigene12830\_Sample\_011046841, Unigene47947\_Sample\_011046841, Unigene18318\_Sample\_011046841, Unigene32348\_Sample\_011046841, Unigene10739\_Sample\_011046841, Unigene26651\_Sample\_011046841, Unigene34357\_Sample\_011046841, Unigene8020\_Sample\_011046841, Unigene31768\_Sample\_011046841, Unigene53617\_Sample\_011046841, Unigene48260\_Sample\_011046841, Unigene52963\_Sample\_011046841, Unigene12172\_Sample\_011046841, Unigene50631\_Sample\_011046841, Unigene39987\_Sample\_011046841, Unigene15774\_Sample\_011046841, Unigene60161\_Sample\_011046841, Unigene55156\_Sample\_011046841, Unigene53650\_Sample\_011046841, Unigene57702\_Sample\_011046841, Unigene50051\_Sample\_011046841, Unigene48009\_Sample\_011046841, Unigene53736\_Sample\_011046841, Unigene57460\_Sample\_011046841, Unigene42827\_Sample\_011046841, Unigene56139\_Sample\_011046841, Unigene59022\_Sample\_011046841, Unigene60842\_Sample\_011046841, Unigene55791\_Sample\_011046841, Unigene15121\_Sample\_011046841, Unigene30474\_Sample\_011046841, Unigene47936\_Sample\_011046841, Unigene51504\_Sample\_011046841, Unigene55650\_Sample\_011046841, Unigene49448\_Sample\_011046841, Unigene53160\_Sample\_011046841, Unigene13666\_Sample\_011046841, Unigene11114\_Sample\_011046841, Unigene57890\_Sample\_011046841, Unigene44587\_Sample\_011046841, Unigene13436\_Sample\_011046841, Unigene57313\_Sample\_011046841, Unigene33440\_Sample\_011046841, Unigene29524\_Sample\_011046841, Unigene50506\_Sample\_011046841, Unigene1129\_Sample\_011046841, Unigene48398\_Sample\_011046841, Unigene55258\_Sample\_011046841, Unigene60388\_Sample\_011046841, Unigene58400\_Sample\_011046841, Unigene30825\_Sample\_011046841, Unigene56616\_Sample\_011046841, Unigene42378\_Sample\_011046841, Unigene59547\_Sample\_011046841, Unigene54996\_Sample\_011046841, Unigene42026\_Sample\_011046841, Unigene60561\_Sample\_011046841, Unigene11448\_Sample\_011046841, Unigene12840\_Sample\_011046841, Unigene58386\_Sample\_011046841, Unigene60709\_Sample\_011046841, Unigene27576\_Sample\_011046841, Unigene12076\_Sample\_011046841, Unigene43570\_Sample\_011046841, Unigene55561\_Sample\_011046841, Unigene13633\_Sample\_011046841, Unigene53798\_Sample\_011046841, Unigene45476\_Sample\_011046841, Unigene39678\_Sample\_011046841, Unigene41323\_Sample\_011046841, Unigene41608\_Sample\_011046841, Unigene59198\_Sample\_011046841, Unigene58496\_Sample\_011046841, Unigene58845\_Sample\_011046841, Unigene57453\_Sample\_011046841, Unigene26271\_Sample\_011046841, Unigene49487\_Sample\_011046841, Unigene26737\_Sample\_011046841, Unigene9620\_Sample\_011046841, Unigene56991\_Sample\_011046841, Unigene32488\_Sample\_011046841, Unigene55183\_Sample\_011046841, Unigene40858\_Sample\_011046841, Unigene57238\_Sample\_011046841, Unigene46777\_Sample\_011046841, Unigene29126\_Sample\_011046841, Unigene34659\_Sample\_011046841, Unigene45403\_Sample\_011046841, Unigene42148\_Sample\_011046841, Unigene8925\_Sample\_011046841, Unigene25272\_Sample\_011046841, Unigene14794\_Sample\_011046841, Unigene59567\_Sample\_011046841, Unigene10615\_Sample\_011046841, Unigene53233\_Sample\_011046841, Unigene45620\_Sample\_011046841, Unigene59136\_Sample\_011046841, Unigene54891\_Sample\_011046841, Unigene50990\_Sample\_011046841, Unigene55982\_Sample\_011046841, Unigene47487\_Sample\_011046841, Unigene59621\_Sample\_011046841, Unigene50223\_Sample\_011046841, Unigene26723\_Sample\_011046841, Unigene50164\_Sample\_011046841, Unigene15865\_Sample\_011046841, Unigene55529\_Sample\_011046841, Unigene54555\_Sample\_011046841, Unigene32930\_Sample\_011046841, Unigene55300\_Sample\_011046841, Unigene35963\_Sample\_011046841, Unigene18725\_Sample\_011046841, Unigene46525\_Sample\_011046841, Unigene55754\_Sample\_011046841, Unigene41168\_Sample\_011046841, Unigene44079\_Sample\_011046841, Unigene38180\_Sample\_011046841, Unigene27581\_Sample\_011046841, Unigene60586\_Sample\_011046841, Unigene60793\_Sample\_011046841, Unigene23033\_Sample\_011046841, Unigene19699\_Sample\_011046841, Unigene54406\_Sample\_011046841, Unigene52106\_Sample\_011046841, Unigene54852\_Sample\_011046841, Unigene59027\_Sample\_011046841, Unigene59457\_Sample\_011046841, Unigene59563\_Sample\_011046841, Unigene18813\_Sample\_011046841, Unigene51803\_Sample\_011046841, Unigene33068\_Sample\_011046841, Unigene58674\_Sample\_011046841, Unigene4166\_Sample\_011046841, Unigene39921\_Sample\_011046841, Unigene21082\_Sample\_011046841, Unigene8538\_Sample\_011046841, Unigene60225\_Sample\_011046841, Unigene50775\_Sample\_011046841, Unigene45679\_Sample\_011046841, Unigene56457\_Sample\_011046841, Unigene8255\_Sample\_011046841, Unigene49371\_Sample\_011046841, Unigene43561\_Sample\_011046841, Unigene42012\_Sample\_011046841, Unigene3496\_Sample\_011046841, Unigene38168\_Sample\_011046841, Unigene69\_Sample\_011046841, Unigene51555\_Sample\_011046841, Unigene46635\_Sample\_011046841, Unigene60249\_Sample\_011046841, Unigene35453\_Sample\_011046841, Unigene59679\_Sample\_011046841, Unigene46439\_Sample\_011046841, Unigene32103\_Sample\_011046841, Unigene50396\_Sample\_011046841, Unigene58138\_Sample\_011046841, Unigene53015\_Sample\_011046841, Unigene36469\_Sample\_011046841, Unigene20591\_Sample\_011046841, Unigene47753\_Sample\_011046841, Unigene29669\_Sample\_011046841, Unigene53833\_Sample\_011046841, Unigene14125\_Sample\_011046841, Unigene54221\_Sample\_011046841, Unigene9329\_Sample\_011046841, Unigene47357\_Sample\_011046841, Unigene10640\_Sample\_011046841, Unigene37898\_Sample\_011046841, Unigene42812\_Sample\_011046841, Unigene37098\_Sample\_011046841, Unigene56005\_Sample\_011046841, Unigene39223\_Sample\_011046841, Unigene55306\_Sample\_011046841, Unigene43099\_Sample\_011046841, Unigene56127\_Sample\_011046841, Unigene57340\_Sample\_011046841, Unigene3756\_Sample\_011046841, Unigene13258\_Sample\_011046841, Unigene56364\_Sample\_011046841, Unigene28709\_Sample\_011046841, Unigene51569\_Sample\_011046841, Unigene10204\_Sample\_011046841, Unigene10963\_Sample\_011046841, Unigene15\_Sample\_011046841, Unigene60889\_Sample\_011046841, Unigene17258\_Sample\_011046841, Unigene51613\_Sample\_011046841, Unigene47150\_Sample\_011046841, Unigene7887\_Sample\_011046841, Unigene40697\_Sample\_011046841, Unigene49861\_Sample\_011046841, Unigene44518\_Sample\_011046841, Unigene58929\_Sample\_011046841, Unigene12620\_Sample\_011046841, Unigene49392\_Sample\_011046841, Unigene41822\_Sample\_011046841, Unigene13203\_Sample\_011046841, Unigene57106\_Sample\_011046841, Unigene42625\_Sample\_011046841, Unigene23667\_Sample\_011046841, Unigene50127\_Sample\_011046841, Unigene8959\_Sample\_011046841, Unigene33127\_Sample\_011046841, Unigene42747\_Sample\_011046841, Unigene51225\_Sample\_011046841, Unigene55175\_Sample\_011046841, Unigene58053\_Sample\_011046841, Unigene60317\_Sample\_011046841, Unigene19596\_Sample\_011046841, Unigene40147\_Sample\_011046841, Unigene8243\_Sample\_011046841, Unigene38974\_Sample\_011046841, Unigene54014\_Sample\_011046841, Unigene51363\_Sample\_011046841, Unigene60921\_Sample\_011046841, Unigene53797\_Sample\_011046841, Unigene39877\_Sample\_011046841, Unigene49532\_Sample\_011046841, Unigene9219\_Sample\_011046841, Unigene9304\_Sample\_011046841, Unigene36873\_Sample\_011046841, Unigene11300\_Sample\_011046841, Unigene27430\_Sample\_011046841, Unigene56881\_Sample\_011046841, Unigene57132\_Sample\_011046841, Unigene47368\_Sample\_011046841, Unigene60296\_Sample\_011046841, Unigene44016\_Sample\_011046841, Unigene56920\_Sample\_011046841, Unigene48876\_Sample\_011046841, Unigene56058\_Sample\_011046841, Unigene12913\_Sample\_011046841, Unigene49698\_Sample\_011046841, Unigene11187\_Sample\_011046841, Unigene53037\_Sample\_011046841, Unigene10930\_Sample\_011046841, Unigene54326\_Sample\_011046841, Unigene38373\_Sample\_011046841, Unigene20608\_Sample\_011046841, Unigene57971\_Sample\_011046841, Unigene46000\_Sample\_011046841, Unigene46774\_Sample\_011046841, Unigene59224\_Sample\_011046841, Unigene50689\_Sample\_011046841, Unigene57610\_Sample\_011046841, Unigene50412\_Sample\_011046841, Unigene45626\_Sample\_011046841, Unigene11195\_Sample\_011046841, Unigene9110\_Sample\_011046841, Unigene49633\_Sample\_011046841, Unigene59919\_Sample\_011046841, Unigene53334\_Sample\_011046841, Unigene45873\_Sample\_011046841, Unigene42703\_Sample\_011046841, Unigene60714\_Sample\_011046841, Unigene21480\_Sample\_011046841, Unigene2325\_Sample\_011046841, Unigene6762\_Sample\_011046841, Unigene56560\_Sample\_011046841, Unigene36721\_Sample\_011046841, Unigene57060\_Sample\_011046841, Unigene42105\_Sample\_011046841, Unigene19753\_Sample\_011046841, Unigene40568\_Sample\_011046841, Unigene53410\_Sample\_011046841, Unigene27866\_Sample\_011046841, Unigene59134\_Sample\_011046841, Unigene41573\_Sample\_011046841, Unigene21163\_Sample\_011046841, Unigene54842\_Sample\_011046841, Unigene59587\_Sample\_011046841, Unigene31705\_Sample\_011046841, Unigene54643\_Sample\_011046841, Unigene58326\_Sample\_011046841, Unigene33436\_Sample\_011046841, Unigene56096\_Sample\_011046841, Unigene55769\_Sample\_011046841, Unigene33941\_Sample\_011046841, Unigene40143\_Sample\_011046841, Unigene2225\_Sample\_011046841, Unigene19813\_Sample\_011046841, Unigene30285\_Sample\_011046841, Unigene42637\_Sample\_011046841, Unigene32364\_Sample\_011046841, Unigene54874\_Sample\_011046841, Unigene24860\_Sample\_011046841, Unigene29861\_Sample\_011046841, Unigene37668\_Sample\_011046841, Unigene13569\_Sample\_011046841, Unigene46263\_Sample\_011046841, Unigene49204\_Sample\_011046841, Unigene57696\_Sample\_011046841, Unigene42749\_Sample\_011046841, Unigene30072\_Sample\_011046841, Unigene11662\_Sample\_011046841, Unigene30649\_Sample\_011046841, Unigene24953\_Sample\_011046841, Unigene55491\_Sample\_011046841, Unigene59038\_Sample\_011046841, Unigene46978\_Sample\_011046841, Unigene38930\_Sample\_011046841, Unigene52253\_Sample\_011046841, Unigene7850\_Sample\_011046841, Unigene54900\_Sample\_011046841, Unigene60217\_Sample\_011046841, Unigene56635\_Sample\_011046841, Unigene641\_Sample\_011046841, Unigene55854\_Sample\_011046841, Unigene58376\_Sample\_011046841, Unigene57614\_Sample\_011046841, Unigene57972\_Sample\_011046841, Unigene50665\_Sample\_011046841, Unigene51483\_Sample\_011046841, Unigene29630\_Sample\_011046841, Unigene34269\_Sample\_011046841, Unigene57585\_Sample\_011046841, Unigene36328\_Sample\_011046841, Unigene49481\_Sample\_011046841, Unigene46332\_Sample\_011046841, Unigene20433\_Sample\_011046841, Unigene39621\_Sample\_011046841, Unigene1934\_Sample\_011046841, Unigene19030\_Sample\_011046841, Unigene51777\_Sample\_011046841, Unigene15267\_Sample\_011046841, Unigene25153\_Sample\_011046841, Unigene53179\_Sample\_011046841, Unigene27324\_Sample\_011046841, Unigene43409\_Sample\_011046841, Unigene11431\_Sample\_011046841, Unigene36131\_Sample\_011046841, Unigene4684\_Sample\_011046841, Unigene50469\_Sample\_011046841, Unigene42151\_Sample\_011046841, Unigene50681\_Sample\_011046841, Unigene53698\_Sample\_011046841, Unigene50764\_Sample\_011046841, Unigene60949\_Sample\_011046841, Unigene43020\_Sample\_011046841, Unigene33792\_Sample\_011046841, Unigene24568\_Sample\_011046841, Unigene60729\_Sample\_011046841, Unigene26672\_Sample\_011046841, Unigene10356\_Sample\_011046841, Unigene9011\_Sample\_011046841, Unigene34203\_Sample\_011046841, Unigene58190\_Sample\_011046841, Unigene41288\_Sample\_011046841, Unigene51527\_Sample\_011046841, Unigene51340\_Sample\_011046841, Unigene35672\_Sample\_011046841, Unigene1944\_Sample\_011046841, Unigene58495\_Sample\_011046841, Unigene59628\_Sample\_011046841, Unigene59442\_Sample\_011046841, Unigene45469\_Sample\_011046841, Unigene11211\_Sample\_011046841, Unigene47924\_Sample\_011046841, Unigene60699\_Sample\_011046841, Unigene49863\_Sample\_011046841, Unigene54628\_Sample\_011046841, Unigene35914\_Sample\_011046841, Unigene55269\_Sample\_011046841, Unigene52467\_Sample\_011046841, Unigene2385\_Sample\_011046841, Unigene54205\_Sample\_011046841, Unigene60785\_Sample\_011046841, Unigene53942\_Sample\_011046841, Unigene54448\_Sample\_011046841, Unigene58611\_Sample\_011046841, Unigene47846\_Sample\_011046841, Unigene58753\_Sample\_011046841, Unigene60966\_Sample\_011046841, Unigene60106\_Sample\_011046841, Unigene13951\_Sample\_011046841, Unigene52776\_Sample\_011046841, Unigene11088\_Sample\_011046841, Unigene56208\_Sample\_011046841, Unigene33721\_Sample\_011046841, Unigene11730\_Sample\_011046841, Unigene60371\_Sample\_011046841, Unigene58223\_Sample\_011046841, Unigene10793\_Sample\_011046841, Unigene19671\_Sample\_011046841, Unigene13007\_Sample\_011046841, Unigene11351\_Sample\_011046841, Unigene27756\_Sample\_011046841, Unigene33218\_Sample\_011046841, Unigene4226\_Sample\_011046841, Unigene47872\_Sample\_011046841, Unigene10588\_Sample\_011046841, Unigene58058\_Sample\_011046841, Unigene12822\_Sample\_011046841, Unigene57776\_Sample\_011046841, Unigene47882\_Sample\_011046841, Unigene33755\_Sample\_011046841, Unigene31695\_Sample\_011046841, Unigene50443\_Sample\_011046841, Unigene42349\_Sample\_011046841, Unigene17987\_Sample\_011046841, Unigene54835\_Sample\_011046841, Unigene60642\_Sample\_011046841, Unigene31515\_Sample\_011046841, Unigene31120\_Sample\_011046841, Unigene52623\_Sample\_011046841, Unigene59727\_Sample\_011046841, Unigene7922\_Sample\_011046841, Unigene42686\_Sample\_011046841, Unigene33132\_Sample\_011046841, Unigene44142\_Sample\_011046841, Unigene28348\_Sample\_011046841, Unigene12501\_Sample\_011046841, Unigene55527\_Sample\_011046841, Unigene51977\_Sample\_011046841, Unigene35691\_Sample\_011046841, Unigene13115\_Sample\_011046841, Unigene44567\_Sample\_011046841, Unigene13447\_Sample\_011046841, Unigene3666\_Sample\_011046841, Unigene59743\_Sample\_011046841, Unigene14570\_Sample\_011046841, Unigene14358\_Sample\_011046841, Unigene16199\_Sample\_011046841, Unigene55737\_Sample\_011046841, Unigene51942\_Sample\_011046841, Unigene49055\_Sample\_011046841, Unigene10152\_Sample\_011046841, Unigene40271\_Sample\_011046841, Unigene25397\_Sample\_011046841, Unigene55423\_Sample\_011046841, Unigene50825\_Sample\_011046841, Unigene45275\_Sample\_011046841, Unigene59490\_Sample\_011046841, Unigene57219\_Sample\_011046841, Unigene40648\_Sample\_011046841, Unigene53415\_Sample\_011046841, Unigene49898\_Sample\_011046841, Unigene41467\_Sample\_011046841, Unigene56385\_Sample\_011046841, Unigene12811\_Sample\_011046841, Unigene28678\_Sample\_011046841, Unigene6240\_Sample\_011046841, Unigene15204\_Sample\_011046841, Unigene52238\_Sample\_011046841, Unigene46897\_Sample\_011046841, Unigene59170\_Sample\_011046841, Unigene58584\_Sample\_011046841, Unigene7095\_Sample\_011046841, Unigene58934\_Sample\_011046841, Unigene59935\_Sample\_011046841, Unigene56584\_Sample\_011046841, Unigene56360\_Sample\_011046841, Unigene44216\_Sample\_011046841, Unigene9649\_Sample\_011046841, Unigene24715\_Sample\_011046841, Unigene46860\_Sample\_011046841, Unigene51270\_Sample\_011046841, Unigene60666\_Sample\_011046841, Unigene33607\_Sample\_011046841, Unigene40565\_Sample\_011046841, Unigene39138\_Sample\_011046841, Unigene32964\_Sample\_011046841, Unigene23751\_Sample\_011046841, Unigene49337\_Sample\_011046841, Unigene40440\_Sample\_011046841, Unigene46598\_Sample\_011046841, Unigene58610\_Sample\_011046841, Unigene55163\_Sample\_011046841, Unigene46488\_Sample\_011046841, Unigene33026\_Sample\_011046841, Unigene41001\_Sample\_011046841, Unigene45662\_Sample\_011046841, Unigene42862\_Sample\_011046841, Unigene60245\_Sample\_011046841, Unigene54460\_Sample\_011046841, Unigene32550\_Sample\_011046841, Unigene31025\_Sample\_011046841, Unigene53128\_Sample\_011046841, Unigene31154\_Sample\_011046841, Unigene54574\_Sample\_011046841, Unigene60723\_Sample\_011046841, Unigene24973\_Sample\_011046841, Unigene12846\_Sample\_011046841, Unigene8551\_Sample\_011046841, Unigene54003\_Sample\_011046841, Unigene59750\_Sample\_011046841, Unigene53888\_Sample\_011046841, Unigene13079\_Sample\_011046841, Unigene22245\_Sample\_011046841, Unigene59877\_Sample\_011046841, Unigene54338\_Sample\_011046841, Unigene775\_Sample\_011046841, Unigene1010\_Sample\_011046841, Unigene55582\_Sample\_011046841, Unigene48252\_Sample\_011046841, Unigene50746\_Sample\_011046841, Unigene48257\_Sample\_011046841, Unigene56109\_Sample\_011046841, Unigene31514\_Sample\_011046841, Unigene59872\_Sample\_011046841, Unigene36891\_Sample\_011046841, Unigene132\_Sample\_011046841, Unigene59701\_Sample\_011046841, Unigene21319\_Sample\_011046841, Unigene41135\_Sample\_011046841, Unigene19091\_Sample\_011046841, Unigene30295\_Sample\_011046841, Unigene60180\_Sample\_011046841, Unigene45049\_Sample\_011046841, Unigene42742\_Sample\_011046841, Unigene31216\_Sample\_011046841, Unigene55112\_Sample\_011046841, Unigene30035\_Sample\_011046841, Unigene59546\_Sample\_011046841, Unigene55624\_Sample\_011046841, Unigene18756\_Sample\_011046841, Unigene21728\_Sample\_011046841, Unigene11786\_Sample\_011046841, Unigene48926\_Sample\_011046841, Unigene52808\_Sample\_011046841, Unigene54711\_Sample\_011046841, Unigene34657\_Sample\_011046841, Unigene19355\_Sample\_011046841, Unigene41206\_Sample\_011046841, Unigene17531\_Sample\_011046841, Unigene42107\_Sample\_011046841, Unigene53711\_Sample\_011046841, Unigene54273\_Sample\_011046841, Unigene60407\_Sample\_011046841, Unigene35030\_Sample\_011046841, Unigene39087\_Sample\_011046841, Unigene57973\_Sample\_011046841, Unigene27618\_Sample\_011046841, Unigene32121\_Sample\_011046841, Unigene41604\_Sample\_011046841, Unigene12189\_Sample\_011046841, Unigene59005\_Sample\_011046841, Unigene29365\_Sample\_011046841, Unigene44253\_Sample\_011046841, Unigene52177\_Sample\_011046841, Unigene2221\_Sample\_011046841, Unigene53424\_Sample\_011046841, Unigene51126\_Sample\_011046841, Unigene60728\_Sample\_011046841, Unigene59372\_Sample\_011046841, Unigene48026\_Sample\_011046841, Unigene36178\_Sample\_011046841, Unigene40161\_Sample\_011046841, Unigene58011\_Sample\_011046841, Unigene55792\_Sample\_011046841, Unigene7487\_Sample\_011046841, Unigene49667\_Sample\_011046841, Unigene23102\_Sample\_011046841, Unigene43515\_Sample\_011046841, Unigene30591\_Sample\_011046841, Unigene44243\_Sample\_011046841, Unigene16372\_Sample\_011046841, Unigene53154\_Sample\_011046841, Unigene58042\_Sample\_011046841, Unigene24797\_Sample\_011046841, Unigene60220\_Sample\_011046841, Unigene41575\_Sample\_011046841, Unigene45451\_Sample\_011046841, Unigene56877\_Sample\_011046841, Unigene52934\_Sample\_011046841, Unigene51011\_Sample\_011046841, Unigene60100\_Sample\_011046841, Unigene35814\_Sample\_011046841, Unigene9408\_Sample\_011046841, Unigene21679\_Sample\_011046841, Unigene9130\_Sample\_011046841, Unigene18000\_Sample\_011046841, Unigene56383\_Sample\_011046841, Unigene60733\_Sample\_011046841, Unigene11124\_Sample\_011046841, Unigene18356\_Sample\_011046841, Unigene6946\_Sample\_011046841, Unigene54386\_Sample\_011046841, Unigene57760\_Sample\_011046841, Unigene44025\_Sample\_011046841, Unigene55494\_Sample\_011046841, Unigene29140\_Sample\_011046841, Unigene3893\_Sample\_011046841, Unigene43097\_Sample\_011046841, Unigene21318\_Sample\_011046841, Unigene39698\_Sample\_011046841, Unigene40683\_Sample\_011046841, Unigene13456\_Sample\_011046841, Unigene3659\_Sample\_011046841, Unigene48072\_Sample\_011046841, Unigene20702\_Sample\_011046841, Unigene45885\_Sample\_011046841, Unigene47635\_Sample\_011046841, Unigene39552\_Sample\_011046841, Unigene50927\_Sample\_011046841, Unigene55779\_Sample\_011046841, Unigene45790\_Sample\_011046841, Unigene21594\_Sample\_011046841, Unigene58343\_Sample\_011046841, Unigene13947\_Sample\_011046841, Unigene57425\_Sample\_011046841, Unigene53231\_Sample\_011046841, Unigene33738\_Sample\_011046841, Unigene52972\_Sample\_011046841, Unigene55896\_Sample\_011046841, Unigene43651\_Sample\_011046841, Unigene1482\_Sample\_011046841, Unigene36199\_Sample\_011046841, Unigene21149\_Sample\_011046841, Unigene43986\_Sample\_011046841, Unigene42734\_Sample\_011046841, Unigene25064\_Sample\_011046841, Unigene51663\_Sample\_011046841, Unigene53749\_Sample\_011046841, Unigene58963\_Sample\_011046841, Unigene39312\_Sample\_011046841, Unigene6279\_Sample\_011046841, Unigene54599\_Sample\_011046841, Unigene59532\_Sample\_011046841, Unigene12957\_Sample\_011046841, Unigene16092\_Sample\_011046841, Unigene54526\_Sample\_011046841, Unigene7072\_Sample\_011046841, Unigene33603\_Sample\_011046841, Unigene43528\_Sample\_011046841, Unigene51429\_Sample\_011046841, Unigene54372\_Sample\_011046841, Unigene12412\_Sample\_011046841, Unigene58989\_Sample\_011046841, Unigene53555\_Sample\_011046841, Unigene44445\_Sample\_011046841, Unigene36986\_Sample\_011046841, Unigene10648\_Sample\_011046841, Unigene42117\_Sample\_011046841, Unigene55499\_Sample\_011046841, Unigene45708\_Sample\_011046841, Unigene49320\_Sample\_011046841, Unigene53451\_Sample\_011046841 |
[truncated: 1,677,322 more chars]
