# Supplementary material for: Identification of neglected cestode Taenia multiceps microRNAs by illumina sequencing and bioinformatic analysis
Source: BMC Vet Res. 2013 Aug 13;9:162. doi: 10.1186/1746-6148-9-162 (PMC3849562; doi:10.1186/1746-6148-9-162)
Supplement: Additional file 8 — Molecular function GO annotations for candidate target unigenes of novel Taenia multiceps miRNAs. 7,095 target unigenes were assigned to 604 GO-terms from “Molecular function” ontology. [file 1746-6148-9-162-S8.html]

Terms for Taenia\_multiceps\_F


## Terms for Taenia\_multiceps\_F

---


### Result Table

|  |
| --- |
| **Terms from the Function Ontology with p-value as good or better than 1** |

| Gene Ontology term | Cluster frequency | Genome frequency of use | Corrected P-value |
| --- | --- | --- | --- |
| binding | 5279 out of 7095 genes, 74.4% | 5340 out of 7190 genes, 74.3% | 1 |
| phosphotransferase activity, alcohol group as acceptor | 493 out of 7095 genes, 6.9% | 495 out of 7190 genes, 6.9% | 1 |
| hydrolase activity | 1684 out of 7095 genes, 23.7% | 1699 out of 7190 genes, 23.6% | 1 |
| transcription regulator activity | 229 out of 7095 genes, 3.2% | 229 out of 7190 genes, 3.2% | 1 |
| protein kinase activity | 457 out of 7095 genes, 6.4% | 459 out of 7190 genes, 6.4% | 1 |
| hydrolase activity, acting on ester bonds | 338 out of 7095 genes, 4.8% | 339 out of 7190 genes, 4.7% | 1 |
| protein serine/threonine kinase activity | 164 out of 7095 genes, 2.3% | 164 out of 7190 genes, 2.3% | 1 |
| kinase activity | 658 out of 7095 genes, 9.3% | 663 out of 7190 genes, 9.2% | 1 |
| transferase activity, transferring phosphorus-containing groups | 834 out of 7095 genes, 11.8% | 841 out of 7190 genes, 11.7% | 1 |
| protein binding | 1256 out of 7095 genes, 17.7% | 1268 out of 7190 genes, 17.6% | 1 |
| phosphoprotein phosphatase activity | 105 out of 7095 genes, 1.5% | 105 out of 7190 genes, 1.5% | 1 |
| phosphoric ester hydrolase activity | 199 out of 7095 genes, 2.8% | 200 out of 7190 genes, 2.8% | 1 |
| transcription factor binding | 101 out of 7095 genes, 1.4% | 101 out of 7190 genes, 1.4% | 1 |
| acid-amino acid ligase activity | 99 out of 7095 genes, 1.4% | 99 out of 7190 genes, 1.4% | 1 |
| metal ion binding | 702 out of 7095 genes, 9.9% | 709 out of 7190 genes, 9.9% | 1 |
| helicase activity | 90 out of 7095 genes, 1.3% | 90 out of 7190 genes, 1.3% | 1 |
| phosphatase activity | 168 out of 7095 genes, 2.4% | 169 out of 7190 genes, 2.4% | 1 |
| transcription cofactor activity | 74 out of 7095 genes, 1.0% | 74 out of 7190 genes, 1.0% | 1 |
| nuclease activity | 74 out of 7095 genes, 1.0% | 74 out of 7190 genes, 1.0% | 1 |
| ligase activity, forming carbon-oxygen bonds | 70 out of 7095 genes, 1.0% | 70 out of 7190 genes, 1.0% | 1 |
| ligase activity, forming aminoacyl-tRNA and related compounds | 70 out of 7095 genes, 1.0% | 70 out of 7190 genes, 1.0% | 1 |
| small conjugating protein ligase activity | 70 out of 7095 genes, 1.0% | 70 out of 7190 genes, 1.0% | 1 |
| DNA binding | 228 out of 7095 genes, 3.2% | 230 out of 7190 genes, 3.2% | 1 |
| lipid binding | 65 out of 7095 genes, 0.9% | 65 out of 7190 genes, 0.9% | 1 |
| aminoacyl-tRNA ligase activity | 64 out of 7095 genes, 0.9% | 64 out of 7190 genes, 0.9% | 1 |
| nucleic acid binding transcription factor activity | 143 out of 7095 genes, 2.0% | 144 out of 7190 genes, 2.0% | 1 |
| structure-specific DNA binding | 59 out of 7095 genes, 0.8% | 59 out of 7190 genes, 0.8% | 1 |
| metal cluster binding | 59 out of 7095 genes, 0.8% | 59 out of 7190 genes, 0.8% | 1 |
| nucleoside-triphosphatase activity | 742 out of 7095 genes, 10.5% | 751 out of 7190 genes, 10.4% | 1 |
| guanyl nucleotide binding | 209 out of 7095 genes, 2.9% | 211 out of 7190 genes, 2.9% | 1 |
| ligase activity, forming carbon-nitrogen bonds | 132 out of 7095 genes, 1.9% | 133 out of 7190 genes, 1.8% | 1 |
| guanyl ribonucleotide binding | 207 out of 7095 genes, 2.9% | 209 out of 7190 genes, 2.9% | 1 |
| nucleotide binding | 1546 out of 7095 genes, 21.8% | 1566 out of 7190 genes, 21.8% | 1 |
| cation binding | 866 out of 7095 genes, 12.2% | 877 out of 7190 genes, 12.2% | 1 |
| transition metal ion binding | 343 out of 7095 genes, 4.8% | 347 out of 7190 genes, 4.8% | 1 |
| carbohydrate binding | 48 out of 7095 genes, 0.7% | 48 out of 7190 genes, 0.7% | 1 |
| catalytic activity | 3968 out of 7095 genes, 55.9% | 4021 out of 7190 genes, 55.9% | 1 |
| nucleotidyltransferase activity | 116 out of 7095 genes, 1.6% | 117 out of 7190 genes, 1.6% | 1 |
| potassium channel activity | 46 out of 7095 genes, 0.6% | 46 out of 7190 genes, 0.6% | 1 |
| transferase activity | 1432 out of 7095 genes, 20.2% | 1451 out of 7190 genes, 20.2% | 1 |
| calcium channel activity | 45 out of 7095 genes, 0.6% | 45 out of 7190 genes, 0.6% | 1 |
| UDP-glycosyltransferase activity | 44 out of 7095 genes, 0.6% | 44 out of 7190 genes, 0.6% | 1 |
| peptidase activity | 253 out of 7095 genes, 3.6% | 256 out of 7190 genes, 3.6% | 1 |
| hydrolase activity, acting on carbon-nitrogen (but not peptide) bonds | 42 out of 7095 genes, 0.6% | 42 out of 7190 genes, 0.6% | 1 |
| protein domain specific binding | 42 out of 7095 genes, 0.6% | 42 out of 7190 genes, 0.6% | 1 |
| ATPase activity | 248 out of 7095 genes, 3.5% | 251 out of 7190 genes, 3.5% | 1 |
| enzyme regulator activity | 177 out of 7095 genes, 2.5% | 179 out of 7190 genes, 2.5% | 1 |
| hydrolase activity, hydrolyzing O-glycosyl compounds | 41 out of 7095 genes, 0.6% | 41 out of 7190 genes, 0.6% | 1 |
| metal ion transmembrane transporter activity | 41 out of 7095 genes, 0.6% | 41 out of 7190 genes, 0.6% | 1 |
| protein tyrosine kinase activity | 40 out of 7095 genes, 0.6% | 40 out of 7190 genes, 0.6% | 1 |
| RNA polymerase activity | 40 out of 7095 genes, 0.6% | 40 out of 7190 genes, 0.6% | 1 |
| iron-sulfur cluster binding | 40 out of 7095 genes, 0.6% | 40 out of 7190 genes, 0.6% | 1 |
| phospholipid binding | 38 out of 7095 genes, 0.5% | 38 out of 7190 genes, 0.5% | 1 |
| sequence-specific DNA binding | 38 out of 7095 genes, 0.5% | 38 out of 7190 genes, 0.5% | 1 |
| ion binding | 876 out of 7095 genes, 12.3% | 888 out of 7190 genes, 12.4% | 1 |
| purine nucleotide binding | 1089 out of 7095 genes, 15.3% | 1104 out of 7190 genes, 15.4% | 1 |
| endonuclease activity | 36 out of 7095 genes, 0.5% | 36 out of 7190 genes, 0.5% | 1 |
| enzyme activator activity | 36 out of 7095 genes, 0.5% | 36 out of 7190 genes, 0.5% | 1 |
| hydrolase activity, acting on acid anhydrides | 796 out of 7095 genes, 11.2% | 807 out of 7190 genes, 11.2% | 1 |
| transporter activity | 580 out of 7095 genes, 8.2% | 588 out of 7190 genes, 8.2% | 1 |
| cysteine-type peptidase activity | 34 out of 7095 genes, 0.5% | 34 out of 7190 genes, 0.5% | 1 |
| exopeptidase activity | 34 out of 7095 genes, 0.5% | 34 out of 7190 genes, 0.5% | 1 |
| hydrolase activity, acting on acid anhydrides, in phosphorus-containing anhydrides | 788 out of 7095 genes, 11.1% | 799 out of 7190 genes, 11.1% | 1 |
| pyrophosphatase activity | 783 out of 7095 genes, 11.0% | 794 out of 7190 genes, 11.0% | 1 |
| amine transmembrane transporter activity | 33 out of 7095 genes, 0.5% | 33 out of 7190 genes, 0.5% | 1 |
| ribonucleotide binding | 1068 out of 7095 genes, 15.1% | 1083 out of 7190 genes, 15.1% | 1 |
| purine ribonucleotide binding | 1068 out of 7095 genes, 15.1% | 1083 out of 7190 genes, 15.1% | 1 |
| nucleoside binding | 923 out of 7095 genes, 13.0% | 936 out of 7190 genes, 13.0% | 1 |
| ligase activity | 289 out of 7095 genes, 4.1% | 293 out of 7190 genes, 4.1% | 1 |
| RNA polymerase II transcription factor activity | 31 out of 7095 genes, 0.4% | 31 out of 7190 genes, 0.4% | 1 |
| ATPase activity, coupled | 219 out of 7095 genes, 3.1% | 222 out of 7190 genes, 3.1% | 1 |
| peptidase activity, acting on L-amino acid peptides | 218 out of 7095 genes, 3.1% | 221 out of 7190 genes, 3.1% | 1 |
| purine nucleoside binding | 910 out of 7095 genes, 12.8% | 923 out of 7190 genes, 12.8% | 1 |
| substrate-specific transporter activity | 488 out of 7095 genes, 6.9% | 495 out of 7190 genes, 6.9% | 1 |
| RNA helicase activity | 30 out of 7095 genes, 0.4% | 30 out of 7190 genes, 0.4% | 1 |
| receptor signaling protein activity | 30 out of 7095 genes, 0.4% | 30 out of 7190 genes, 0.4% | 1 |
| iron ion binding | 29 out of 7095 genes, 0.4% | 29 out of 7190 genes, 0.4% | 1 |
| protein methyltransferase activity | 29 out of 7095 genes, 0.4% | 29 out of 7190 genes, 0.4% | 1 |
| organic acid transmembrane transporter activity | 28 out of 7095 genes, 0.4% | 28 out of 7190 genes, 0.4% | 1 |
| N-methyltransferase activity | 28 out of 7095 genes, 0.4% | 28 out of 7190 genes, 0.4% | 1 |
| hydrolase activity, acting on carbon-nitrogen (but not peptide) bonds, in linear amides | 28 out of 7095 genes, 0.4% | 28 out of 7190 genes, 0.4% | 1 |
| adenyl nucleotide binding | 895 out of 7095 genes, 12.6% | 908 out of 7190 genes, 12.6% | 1 |
| carboxylic acid transmembrane transporter activity | 27 out of 7095 genes, 0.4% | 27 out of 7190 genes, 0.4% | 1 |
| lyase activity | 81 out of 7095 genes, 1.1% | 82 out of 7190 genes, 1.1% | 1 |
| nucleic acid binding | 814 out of 7095 genes, 11.5% | 826 out of 7190 genes, 11.5% | 1 |
| receptor signaling protein serine/threonine kinase activity | 26 out of 7095 genes, 0.4% | 26 out of 7190 genes, 0.4% | 1 |
| enzyme inhibitor activity | 26 out of 7095 genes, 0.4% | 26 out of 7190 genes, 0.4% | 1 |
| lipase activity | 26 out of 7095 genes, 0.4% | 26 out of 7190 genes, 0.4% | 1 |
| kinase regulator activity | 26 out of 7095 genes, 0.4% | 26 out of 7190 genes, 0.4% | 1 |
| adenyl ribonucleotide binding | 877 out of 7095 genes, 12.4% | 890 out of 7190 genes, 12.4% | 1 |
| exonuclease activity | 25 out of 7095 genes, 0.4% | 25 out of 7190 genes, 0.3% | 1 |
| phospholipase activity | 25 out of 7095 genes, 0.4% | 25 out of 7190 genes, 0.3% | 1 |
| protein kinase regulator activity | 25 out of 7095 genes, 0.4% | 25 out of 7190 genes, 0.3% | 1 |
| peptide binding | 25 out of 7095 genes, 0.4% | 25 out of 7190 genes, 0.3% | 1 |
| thiolester hydrolase activity | 24 out of 7095 genes, 0.3% | 24 out of 7190 genes, 0.3% | 1 |
| carbon-carbon lyase activity | 24 out of 7095 genes, 0.3% | 24 out of 7190 genes, 0.3% | 1 |
| hydrogen ion transmembrane transporter activity | 23 out of 7095 genes, 0.3% | 23 out of 7190 genes, 0.3% | 1 |
| carbon-oxygen lyase activity | 23 out of 7095 genes, 0.3% | 23 out of 7190 genes, 0.3% | 1 |
| phosphatase regulator activity | 23 out of 7095 genes, 0.3% | 23 out of 7190 genes, 0.3% | 1 |
| methyltransferase activity | 73 out of 7095 genes, 1.0% | 74 out of 7190 genes, 1.0% | 1 |
| inositol or phosphatidylinositol kinase activity | 22 out of 7095 genes, 0.3% | 22 out of 7190 genes, 0.3% | 1 |
| phosphoric diester hydrolase activity | 22 out of 7095 genes, 0.3% | 22 out of 7190 genes, 0.3% | 1 |
| transcription repressor activity | 22 out of 7095 genes, 0.3% | 22 out of 7190 genes, 0.3% | 1 |
| oxidoreductase activity, acting on a sulfur group of donors | 22 out of 7095 genes, 0.3% | 22 out of 7190 genes, 0.3% | 1 |
| GTPase regulator activity | 72 out of 7095 genes, 1.0% | 73 out of 7190 genes, 1.0% | 1 |
| nucleoside-triphosphatase regulator activity | 72 out of 7095 genes, 1.0% | 73 out of 7190 genes, 1.0% | 1 |
| motor activity | 128 out of 7095 genes, 1.8% | 130 out of 7190 genes, 1.8% | 1 |
| lipid kinase activity | 21 out of 7095 genes, 0.3% | 21 out of 7190 genes, 0.3% | 1 |
| DNA helicase activity | 21 out of 7095 genes, 0.3% | 21 out of 7190 genes, 0.3% | 1 |
| cyclase activity | 21 out of 7095 genes, 0.3% | 21 out of 7190 genes, 0.3% | 1 |
| protein phosphatase regulator activity | 21 out of 7095 genes, 0.3% | 21 out of 7190 genes, 0.3% | 1 |
| protein kinase binding | 21 out of 7095 genes, 0.3% | 21 out of 7190 genes, 0.3% | 1 |
| DNA polymerase activity | 21 out of 7095 genes, 0.3% | 21 out of 7190 genes, 0.3% | 1 |
| hormone receptor binding | 21 out of 7095 genes, 0.3% | 21 out of 7190 genes, 0.3% | 1 |
| RNA binding | 185 out of 7095 genes, 2.6% | 188 out of 7190 genes, 2.6% | 1 |
| hydro-lyase activity | 20 out of 7095 genes, 0.3% | 20 out of 7190 genes, 0.3% | 1 |
| protein complex binding | 20 out of 7095 genes, 0.3% | 20 out of 7190 genes, 0.3% | 1 |
| transmembrane transporter activity | 503 out of 7095 genes, 7.1% | 511 out of 7190 genes, 7.1% | 1 |
| transmembrane receptor activity | 67 out of 7095 genes, 0.9% | 68 out of 7190 genes, 0.9% | 1 |
| isomerase activity | 67 out of 7095 genes, 0.9% | 68 out of 7190 genes, 0.9% | 1 |
| protein dimerization activity | 67 out of 7095 genes, 0.9% | 68 out of 7190 genes, 0.9% | 1 |
| carboxylesterase activity | 19 out of 7095 genes, 0.3% | 19 out of 7190 genes, 0.3% | 1 |
| voltage-gated ion channel activity | 19 out of 7095 genes, 0.3% | 19 out of 7190 genes, 0.3% | 1 |
| lipid transporter activity | 19 out of 7095 genes, 0.3% | 19 out of 7190 genes, 0.3% | 1 |
| voltage-gated channel activity | 19 out of 7095 genes, 0.3% | 19 out of 7190 genes, 0.3% | 1 |
| peptidase regulator activity | 19 out of 7095 genes, 0.3% | 19 out of 7190 genes, 0.3% | 1 |
| hydrolase activity, acting on glycosyl bonds | 66 out of 7095 genes, 0.9% | 67 out of 7190 genes, 0.9% | 1 |
| signal transducer activity | 240 out of 7095 genes, 3.4% | 244 out of 7190 genes, 3.4% | 1 |
| substrate-specific transmembrane transporter activity | 430 out of 7095 genes, 6.1% | 437 out of 7190 genes, 6.1% | 1 |
| mRNA binding | 18 out of 7095 genes, 0.3% | 18 out of 7190 genes, 0.3% | 1 |
| intracellular ligand-gated ion channel activity | 18 out of 7095 genes, 0.3% | 18 out of 7190 genes, 0.3% | 1 |
| sugar binding | 18 out of 7095 genes, 0.3% | 18 out of 7190 genes, 0.3% | 1 |
| amino acid transmembrane transporter activity | 18 out of 7095 genes, 0.3% | 18 out of 7190 genes, 0.3% | 1 |
| antioxidant activity | 18 out of 7095 genes, 0.3% | 18 out of 7190 genes, 0.3% | 1 |
| receptor activity | 117 out of 7095 genes, 1.6% | 119 out of 7190 genes, 1.7% | 1 |
| SNARE binding | 17 out of 7095 genes, 0.2% | 17 out of 7190 genes, 0.2% | 1 |
| cysteine-type endopeptidase activity | 17 out of 7095 genes, 0.2% | 17 out of 7190 genes, 0.2% | 1 |
| endoribonuclease activity | 17 out of 7095 genes, 0.2% | 17 out of 7190 genes, 0.2% | 1 |
| ribonuclease activity | 17 out of 7095 genes, 0.2% | 17 out of 7190 genes, 0.2% | 1 |
| intracellular ligand-gated calcium channel activity | 17 out of 7095 genes, 0.2% | 17 out of 7190 genes, 0.2% | 1 |
| di-, tri-valent inorganic cation transmembrane transporter activity | 17 out of 7095 genes, 0.2% | 17 out of 7190 genes, 0.2% | 1 |
| calcium-release channel activity | 17 out of 7095 genes, 0.2% | 17 out of 7190 genes, 0.2% | 1 |
| cis-trans isomerase activity | 17 out of 7095 genes, 0.2% | 17 out of 7190 genes, 0.2% | 1 |
| endonuclease activity, active with either ribo- or deoxyribonucleic acids and producing 5'-phosphomonoesters | 17 out of 7095 genes, 0.2% | 17 out of 7190 genes, 0.2% | 1 |
| glucosyltransferase activity | 17 out of 7095 genes, 0.2% | 17 out of 7190 genes, 0.2% | 1 |
| kinase binding | 62 out of 7095 genes, 0.9% | 63 out of 7190 genes, 0.9% | 1 |
| secondary active transmembrane transporter activity | 60 out of 7095 genes, 0.8% | 61 out of 7190 genes, 0.8% | 1 |
| cyclic nucleotide-dependent protein kinase activity | 16 out of 7095 genes, 0.2% | 16 out of 7190 genes, 0.2% | 1 |
| ligand-dependent nuclear receptor activity | 16 out of 7095 genes, 0.2% | 16 out of 7190 genes, 0.2% | 1 |
| phosphotransferase activity, phosphate group as acceptor | 16 out of 7095 genes, 0.2% | 16 out of 7190 genes, 0.2% | 1 |
| intramolecular transferase activity | 16 out of 7095 genes, 0.2% | 16 out of 7190 genes, 0.2% | 1 |
| endoribonuclease activity, producing 5'-phosphomonoesters | 16 out of 7095 genes, 0.2% | 16 out of 7190 genes, 0.2% | 1 |
| carboxylic acid binding | 16 out of 7095 genes, 0.2% | 16 out of 7190 genes, 0.2% | 1 |
| phosphoinositide binding | 16 out of 7095 genes, 0.2% | 16 out of 7190 genes, 0.2% | 1 |
| UDP-glucosyltransferase activity | 16 out of 7095 genes, 0.2% | 16 out of 7190 genes, 0.2% | 1 |
| histone methyltransferase activity | 16 out of 7095 genes, 0.2% | 16 out of 7190 genes, 0.2% | 1 |
| enzyme binding | 166 out of 7095 genes, 2.3% | 169 out of 7190 genes, 2.4% | 1 |
| sequence-specific DNA binding transcription factor activity | 15 out of 7095 genes, 0.2% | 15 out of 7190 genes, 0.2% | 1 |
| deoxyribonuclease activity | 15 out of 7095 genes, 0.2% | 15 out of 7190 genes, 0.2% | 1 |
| phospholipid transporter activity | 15 out of 7095 genes, 0.2% | 15 out of 7190 genes, 0.2% | 1 |
| sodium ion transmembrane transporter activity | 15 out of 7095 genes, 0.2% | 15 out of 7190 genes, 0.2% | 1 |
| lysine N-methyltransferase activity | 15 out of 7095 genes, 0.2% | 15 out of 7190 genes, 0.2% | 1 |
| protein-lysine N-methyltransferase activity | 15 out of 7095 genes, 0.2% | 15 out of 7190 genes, 0.2% | 1 |
| transcription activator activity | 15 out of 7095 genes, 0.2% | 15 out of 7190 genes, 0.2% | 1 |
| cation channel activity | 109 out of 7095 genes, 1.5% | 111 out of 7190 genes, 1.5% | 1 |
| transferase activity, transferring glycosyl groups | 164 out of 7095 genes, 2.3% | 167 out of 7190 genes, 2.3% | 1 |
| transcription coactivator activity | 14 out of 7095 genes, 0.2% | 14 out of 7190 genes, 0.2% | 1 |
| GTPase activator activity | 14 out of 7095 genes, 0.2% | 14 out of 7190 genes, 0.2% | 1 |
| metallopeptidase activity | 14 out of 7095 genes, 0.2% | 14 out of 7190 genes, 0.2% | 1 |
| carbohydrate transmembrane transporter activity | 14 out of 7095 genes, 0.2% | 14 out of 7190 genes, 0.2% | 1 |
| histone-lysine N-methyltransferase activity | 14 out of 7095 genes, 0.2% | 14 out of 7190 genes, 0.2% | 1 |
| nuclear hormone receptor binding | 14 out of 7095 genes, 0.2% | 14 out of 7190 genes, 0.2% | 1 |
| transferase activity, transferring acyl groups other than amino-acyl groups | 103 out of 7095 genes, 1.5% | 105 out of 7190 genes, 1.5% | 1 |
| G-protein coupled receptor activity | 13 out of 7095 genes, 0.2% | 13 out of 7190 genes, 0.2% | 1 |
| acetylglucosaminyltransferase activity | 13 out of 7095 genes, 0.2% | 13 out of 7190 genes, 0.2% | 1 |
| oxidoreductase activity, acting on the CH-NH2 group of donors | 13 out of 7095 genes, 0.2% | 13 out of 7190 genes, 0.2% | 1 |
| transmembrane receptor protein kinase activity | 13 out of 7095 genes, 0.2% | 13 out of 7190 genes, 0.2% | 1 |
| voltage-gated cation channel activity | 13 out of 7095 genes, 0.2% | 13 out of 7190 genes, 0.2% | 1 |
| monosaccharide binding | 13 out of 7095 genes, 0.2% | 13 out of 7190 genes, 0.2% | 1 |
| endopeptidase regulator activity | 13 out of 7095 genes, 0.2% | 13 out of 7190 genes, 0.2% | 1 |
| sequence-specific DNA binding RNA polymerase II transcription factor activity | 12 out of 7095 genes, 0.2% | 12 out of 7190 genes, 0.2% | 1 |
| double-stranded DNA binding | 12 out of 7095 genes, 0.2% | 12 out of 7190 genes, 0.2% | 1 |
| voltage-gated potassium channel activity | 12 out of 7095 genes, 0.2% | 12 out of 7190 genes, 0.2% | 1 |
| protein transporter activity | 12 out of 7095 genes, 0.2% | 12 out of 7190 genes, 0.2% | 1 |
| channel regulator activity | 12 out of 7095 genes, 0.2% | 12 out of 7190 genes, 0.2% | 1 |
| transferase activity, transferring amino-acyl groups | 12 out of 7095 genes, 0.2% | 12 out of 7190 genes, 0.2% | 1 |
| transferase activity, transferring nitrogenous groups | 12 out of 7095 genes, 0.2% | 12 out of 7190 genes, 0.2% | 1 |
| carboxy-lyase activity | 12 out of 7095 genes, 0.2% | 12 out of 7190 genes, 0.2% | 1 |
| steroid hormone receptor binding | 12 out of 7095 genes, 0.2% | 12 out of 7190 genes, 0.2% | 1 |
| transferase activity, transferring hexosyl groups | 97 out of 7095 genes, 1.4% | 99 out of 7190 genes, 1.4% | 1 |
| cyclic-nucleotide phosphodiesterase activity | 11 out of 7095 genes, 0.2% | 11 out of 7190 genes, 0.2% | 1 |
| anion channel activity | 11 out of 7095 genes, 0.2% | 11 out of 7190 genes, 0.2% | 1 |
| transaminase activity | 11 out of 7095 genes, 0.2% | 11 out of 7190 genes, 0.2% | 1 |
| solute:sodium symporter activity | 11 out of 7095 genes, 0.2% | 11 out of 7190 genes, 0.2% | 1 |
| mannosidase activity | 11 out of 7095 genes, 0.2% | 11 out of 7190 genes, 0.2% | 1 |
| glucosidase activity | 11 out of 7095 genes, 0.2% | 11 out of 7190 genes, 0.2% | 1 |
| peptidase inhibitor activity | 11 out of 7095 genes, 0.2% | 11 out of 7190 genes, 0.2% | 1 |
| ribonucleoprotein binding | 11 out of 7095 genes, 0.2% | 11 out of 7190 genes, 0.2% | 1 |
| hydrolase activity, acting on acid anhydrides, catalyzing transmembrane movement of substances | 94 out of 7095 genes, 1.3% | 96 out of 7190 genes, 1.3% | 1 |
| receptor binding | 92 out of 7095 genes, 1.3% | 94 out of 7190 genes, 1.3% | 1 |
| acyltransferase activity | 92 out of 7095 genes, 1.3% | 94 out of 7190 genes, 1.3% | 1 |
| peptide receptor activity | 10 out of 7095 genes, 0.1% | 10 out of 7190 genes, 0.1% | 1 |
| endodeoxyribonuclease activity | 10 out of 7095 genes, 0.1% | 10 out of 7190 genes, 0.1% | 1 |
| signal sequence binding | 10 out of 7095 genes, 0.1% | 10 out of 7190 genes, 0.1% | 1 |
| Ras GTPase activator activity | 10 out of 7095 genes, 0.1% | 10 out of 7190 genes, 0.1% | 1 |
| nucleobase, nucleoside, nucleotide and nucleic acid transmembrane transporter activity | 10 out of 7095 genes, 0.1% | 10 out of 7190 genes, 0.1% | 1 |
| arginine N-methyltransferase activity | 10 out of 7095 genes, 0.1% | 10 out of 7190 genes, 0.1% | 1 |
| protein-arginine N-methyltransferase activity | 10 out of 7095 genes, 0.1% | 10 out of 7190 genes, 0.1% | 1 |
| oxidoreductase activity, acting on paired donors, with incorporation or reduction of molecular oxygen | 10 out of 7095 genes, 0.1% | 10 out of 7190 genes, 0.1% | 1 |
| small conjugating protein-specific protease activity | 10 out of 7095 genes, 0.1% | 10 out of 7190 genes, 0.1% | 1 |
| syntaxin binding | 10 out of 7095 genes, 0.1% | 10 out of 7190 genes, 0.1% | 1 |
| small conjugating protein binding | 10 out of 7095 genes, 0.1% | 10 out of 7190 genes, 0.1% | 1 |
| amine binding | 10 out of 7095 genes, 0.1% | 10 out of 7190 genes, 0.1% | 1 |
| ion transmembrane transporter activity | 366 out of 7095 genes, 5.2% | 373 out of 7190 genes, 5.2% | 1 |
| tubulin binding | 44 out of 7095 genes, 0.6% | 45 out of 7190 genes, 0.6% | 1 |
| transferase activity, transferring one-carbon groups | 89 out of 7095 genes, 1.3% | 91 out of 7190 genes, 1.3% | 1 |
| ion channel activity | 191 out of 7095 genes, 2.7% | 195 out of 7190 genes, 2.7% | 1 |
| channel activity | 191 out of 7095 genes, 2.7% | 195 out of 7190 genes, 2.7% | 1 |
| passive transmembrane transporter activity | 191 out of 7095 genes, 2.7% | 195 out of 7190 genes, 2.7% | 1 |
| substrate-specific channel activity | 191 out of 7095 genes, 2.7% | 195 out of 7190 genes, 2.7% | 1 |
| G-protein-coupled receptor binding | 9 out of 7095 genes, 0.1% | 9 out of 7190 genes, 0.1% | 1 |
| pattern binding | 9 out of 7095 genes, 0.1% | 9 out of 7190 genes, 0.1% | 1 |
| nucleobase binding | 9 out of 7095 genes, 0.1% | 9 out of 7190 genes, 0.1% | 1 |
| inositol or phosphatidylinositol phosphatase activity | 9 out of 7095 genes, 0.1% | 9 out of 7190 genes, 0.1% | 1 |
| transmembrane receptor protein serine/threonine kinase activity | 9 out of 7095 genes, 0.1% | 9 out of 7190 genes, 0.1% | 1 |
| calmodulin-dependent protein kinase activity | 9 out of 7095 genes, 0.1% | 9 out of 7190 genes, 0.1% | 1 |
| protein serine/threonine phosphatase activity | 9 out of 7095 genes, 0.1% | 9 out of 7190 genes, 0.1% | 1 |
| O-acyltransferase activity | 9 out of 7095 genes, 0.1% | 9 out of 7190 genes, 0.1% | 1 |
| malate dehydrogenase activity | 9 out of 7095 genes, 0.1% | 9 out of 7190 genes, 0.1% | 1 |
| intramolecular transferase activity, phosphotransferases | 9 out of 7095 genes, 0.1% | 9 out of 7190 genes, 0.1% | 1 |
| myosin binding | 9 out of 7095 genes, 0.1% | 9 out of 7190 genes, 0.1% | 1 |
| growth factor binding | 9 out of 7095 genes, 0.1% | 9 out of 7190 genes, 0.1% | 1 |
| neurotransmitter binding | 9 out of 7095 genes, 0.1% | 9 out of 7190 genes, 0.1% | 1 |
| oxidoreductase activity, acting on the CH-OH group of donors, NAD or NADP as acceptor | 42 out of 7095 genes, 0.6% | 43 out of 7190 genes, 0.6% | 1 |
| active transmembrane transporter activity | 188 out of 7095 genes, 2.6% | 192 out of 7190 genes, 2.7% | 1 |
| translation factor activity, nucleic acid binding | 85 out of 7095 genes, 1.2% | 87 out of 7190 genes, 1.2% | 1 |
| ATPase activity, coupled to movement of substances | 85 out of 7095 genes, 1.2% | 87 out of 7190 genes, 1.2% | 1 |
| acetyltransferase activity | 41 out of 7095 genes, 0.6% | 42 out of 7190 genes, 0.6% | 1 |
| molecular transducer activity | 295 out of 7095 genes, 4.2% | 301 out of 7190 genes, 4.2% | 1 |
| ATPase activity, coupled to transmembrane movement of substances | 83 out of 7095 genes, 1.2% | 85 out of 7190 genes, 1.2% | 1 |
| oxidoreductase activity, acting on NADH or NADPH | 40 out of 7095 genes, 0.6% | 41 out of 7190 genes, 0.6% | 1 |
| mannosyltransferase activity | 8 out of 7095 genes, 0.1% | 8 out of 7190 genes, 0.1% | 1 |
| 3',5'-cyclic-nucleotide phosphodiesterase activity | 8 out of 7095 genes, 0.1% | 8 out of 7190 genes, 0.1% | 1 |
| histone deacetylase activity | 8 out of 7095 genes, 0.1% | 8 out of 7190 genes, 0.1% | 1 |
| isocitrate dehydrogenase activity | 8 out of 7095 genes, 0.1% | 8 out of 7190 genes, 0.1% | 1 |
| ubiquitin-protein ligase activity | 8 out of 7095 genes, 0.1% | 8 out of 7190 genes, 0.1% | 1 |
| calcium activated cation channel activity | 8 out of 7095 genes, 0.1% | 8 out of 7190 genes, 0.1% | 1 |
| neurotransmitter transporter activity | 8 out of 7095 genes, 0.1% | 8 out of 7190 genes, 0.1% | 1 |
| nucleoside transmembrane transporter activity | 8 out of 7095 genes, 0.1% | 8 out of 7190 genes, 0.1% | 1 |
| organic acid:sodium symporter activity | 8 out of 7095 genes, 0.1% | 8 out of 7190 genes, 0.1% | 1 |
| potassium-transporting ATPase activity | 8 out of 7095 genes, 0.1% | 8 out of 7190 genes, 0.1% | 1 |
| potassium ion transmembrane transporter activity | 8 out of 7095 genes, 0.1% | 8 out of 7190 genes, 0.1% | 1 |
| fatty acid ligase activity | 8 out of 7095 genes, 0.1% | 8 out of 7190 genes, 0.1% | 1 |
| general RNA polymerase II transcription factor activity | 8 out of 7095 genes, 0.1% | 8 out of 7190 genes, 0.1% | 1 |
| phosphatidylinositol phosphate kinase activity | 8 out of 7095 genes, 0.1% | 8 out of 7190 genes, 0.1% | 1 |
| oxidoreductase activity, acting on a sulfur group of donors, disulfide as acceptor | 8 out of 7095 genes, 0.1% | 8 out of 7190 genes, 0.1% | 1 |
| carbohydrate kinase activity | 8 out of 7095 genes, 0.1% | 8 out of 7190 genes, 0.1% | 1 |
| nucleobase, nucleoside, nucleotide kinase activity | 8 out of 7095 genes, 0.1% | 8 out of 7190 genes, 0.1% | 1 |
| deacetylase activity | 8 out of 7095 genes, 0.1% | 8 out of 7190 genes, 0.1% | 1 |
| vitamin binding | 8 out of 7095 genes, 0.1% | 8 out of 7190 genes, 0.1% | 1 |
| mismatched DNA binding | 8 out of 7095 genes, 0.1% | 8 out of 7190 genes, 0.1% | 1 |
| heat shock protein binding | 8 out of 7095 genes, 0.1% | 8 out of 7190 genes, 0.1% | 1 |
| protein deacetylase activity | 8 out of 7095 genes, 0.1% | 8 out of 7190 genes, 0.1% | 1 |
| coenzyme binding | 8 out of 7095 genes, 0.1% | 8 out of 7190 genes, 0.1% | 1 |
| cation transmembrane transporter activity | 233 out of 7095 genes, 3.3% | 238 out of 7190 genes, 3.3% | 1 |
| transcription elongation regulator activity | 7 out of 7095 genes, 0.1% | 7 out of 7190 genes, 0.1% | 1 |
| DNA-directed RNA polymerase activity | 7 out of 7095 genes, 0.1% | 7 out of 7190 genes, 0.1% | 1 |
| oligosaccharyl transferase activity | 7 out of 7095 genes, 0.1% | 7 out of 7190 genes, 0.1% | 1 |
| protein serine/threonine/tyrosine kinase activity | 7 out of 7095 genes, 0.1% | 7 out of 7190 genes, 0.1% | 1 |
| protein tyrosine phosphatase activity | 7 out of 7095 genes, 0.1% | 7 out of 7190 genes, 0.1% | 1 |
| ionotropic glutamate receptor activity | 7 out of 7095 genes, 0.1% | 7 out of 7190 genes, 0.1% | 1 |
| protein transmembrane transporter activity | 7 out of 7095 genes, 0.1% | 7 out of 7190 genes, 0.1% | 1 |
| phosphofructokinase activity | 7 out of 7095 genes, 0.1% | 7 out of 7190 genes, 0.1% | 1 |
| L-amino acid transmembrane transporter activity | 7 out of 7095 genes, 0.1% | 7 out of 7190 genes, 0.1% | 1 |
| solute:cation antiporter activity | 7 out of 7095 genes, 0.1% | 7 out of 7190 genes, 0.1% | 1 |
| CoA carboxylase activity | 7 out of 7095 genes, 0.1% | 7 out of 7190 genes, 0.1% | 1 |
| oxidoreductase activity, acting on the CH-NH2 group of donors, NAD or NADP as acceptor | 7 out of 7095 genes, 0.1% | 7 out of 7190 genes, 0.1% | 1 |
| aldehyde-lyase activity | 7 out of 7095 genes, 0.1% | 7 out of 7190 genes, 0.1% | 1 |
| intramolecular oxidoreductase activity | 7 out of 7095 genes, 0.1% | 7 out of 7190 genes, 0.1% | 1 |
| ligase activity, forming carbon-carbon bonds | 7 out of 7095 genes, 0.1% | 7 out of 7190 genes, 0.1% | 1 |
| oxidoreductase activity, acting on the CH-OH group of donors, quinone or similar compound as acceptor | 7 out of 7095 genes, 0.1% | 7 out of 7190 genes, 0.1% | 1 |
| macromolecule transmembrane transporter activity | 7 out of 7095 genes, 0.1% | 7 out of 7190 genes, 0.1% | 1 |
| polysaccharide binding | 7 out of 7095 genes, 0.1% | 7 out of 7190 genes, 0.1% | 1 |
| histone binding | 7 out of 7095 genes, 0.1% | 7 out of 7190 genes, 0.1% | 1 |
| protein kinase A binding | 7 out of 7095 genes, 0.1% | 7 out of 7190 genes, 0.1% | 1 |
| NAD or NADH binding | 7 out of 7095 genes, 0.1% | 7 out of 7190 genes, 0.1% | 1 |
| inorganic cation transmembrane transporter activity | 77 out of 7095 genes, 1.1% | 79 out of 7190 genes, 1.1% | 1 |
| small GTPase regulator activity | 36 out of 7095 genes, 0.5% | 37 out of 7190 genes, 0.5% | 1 |
| S-adenosylmethionine-dependent methyltransferase activity | 36 out of 7095 genes, 0.5% | 37 out of 7190 genes, 0.5% | 1 |
| cytoskeletal protein binding | 173 out of 7095 genes, 2.4% | 177 out of 7190 genes, 2.5% | 1 |
| transferase activity, transferring acyl groups | 121 out of 7095 genes, 1.7% | 124 out of 7190 genes, 1.7% | 1 |
| glycogen debranching enzyme activity | 6 out of 7095 genes, 0.1% | 6 out of 7190 genes, 0.1% | 1 |
| dihydroorotate dehydrogenase activity | 6 out of 7095 genes, 0.1% | 6 out of 7190 genes, 0.1% | 1 |
| malic enzyme activity | 6 out of 7095 genes, 0.1% | 6 out of 7190 genes, 0.1% | 1 |
| exodeoxyribonuclease activity | 6 out of 7095 genes, 0.1% | 6 out of 7190 genes, 0.1% | 1 |
| phosphoglycerate mutase activity | 6 out of 7095 genes, 0.1% | 6 out of 7190 genes, 0.1% | 1 |
| phospholipase C activity | 6 out of 7095 genes, 0.1% | 6 out of 7190 genes, 0.1% | 1 |
| prenyltransferase activity | 6 out of 7095 genes, 0.1% | 6 out of 7190 genes, 0.1% | 1 |
| transforming growth factor beta receptor activity | 6 out of 7095 genes, 0.1% | 6 out of 7190 genes, 0.1% | 1 |
| cytokine receptor binding | 6 out of 7095 genes, 0.1% | 6 out of 7190 genes, 0.1% | 1 |
| nucleotide-sugar transmembrane transporter activity | 6 out of 7095 genes, 0.1% | 6 out of 7190 genes, 0.1% | 1 |
| translation termination factor activity | 6 out of 7095 genes, 0.1% | 6 out of 7190 genes, 0.1% | 1 |
| sulfotransferase activity | 6 out of 7095 genes, 0.1% | 6 out of 7190 genes, 0.1% | 1 |
| galactosyltransferase activity | 6 out of 7095 genes, 0.1% | 6 out of 7190 genes, 0.1% | 1 |
| disulfide oxidoreductase activity | 6 out of 7095 genes, 0.1% | 6 out of 7190 genes, 0.1% | 1 |
| monosaccharide transmembrane transporter activity | 6 out of 7095 genes, 0.1% | 6 out of 7190 genes, 0.1% | 1 |
| hexose transmembrane transporter activity | 6 out of 7095 genes, 0.1% | 6 out of 7190 genes, 0.1% | 1 |
| pyrimidine nucleotide sugar transmembrane transporter activity | 6 out of 7095 genes, 0.1% | 6 out of 7190 genes, 0.1% | 1 |
| acidic amino acid transmembrane transporter activity | 6 out of 7095 genes, 0.1% | 6 out of 7190 genes, 0.1% | 1 |
| outward rectifier potassium channel activity | 6 out of 7095 genes, 0.1% | 6 out of 7190 genes, 0.1% | 1 |
| solute:hydrogen antiporter activity | 6 out of 7095 genes, 0.1% | 6 out of 7190 genes, 0.1% | 1 |
| mannosyl-oligosaccharide mannosidase activity | 6 out of 7095 genes, 0.1% | 6 out of 7190 genes, 0.1% | 1 |
| AMP binding | 6 out of 7095 genes, 0.1% | 6 out of 7190 genes, 0.1% | 1 |
| amino acid binding | 6 out of 7095 genes, 0.1% | 6 out of 7190 genes, 0.1% | 1 |
| oxidoreductase activity, acting on the CH-CH group of donors, quinone or related compound as acceptor | 6 out of 7095 genes, 0.1% | 6 out of 7190 genes, 0.1% | 1 |
| oxidoreductase activity, acting on single donors with incorporation of molecular oxygen | 6 out of 7095 genes, 0.1% | 6 out of 7190 genes, 0.1% | 1 |
| diphosphotransferase activity | 6 out of 7095 genes, 0.1% | 6 out of 7190 genes, 0.1% | 1 |
| exonuclease activity, active with either ribo- or deoxyribonucleic acids and producing 5'-phosphomonoesters | 6 out of 7095 genes, 0.1% | 6 out of 7190 genes, 0.1% | 1 |
| hydrolase activity, hydrolyzing N-glycosyl compounds | 6 out of 7095 genes, 0.1% | 6 out of 7190 genes, 0.1% | 1 |
| exodeoxyribonuclease activity, producing 5'-phosphomonoesters | 6 out of 7095 genes, 0.1% | 6 out of 7190 genes, 0.1% | 1 |
| DNA N-glycosylase activity | 6 out of 7095 genes, 0.1% | 6 out of 7190 genes, 0.1% | 1 |
| phosphatase binding | 6 out of 7095 genes, 0.1% | 6 out of 7190 genes, 0.1% | 1 |
| neurotransmitter receptor activity | 6 out of 7095 genes, 0.1% | 6 out of 7190 genes, 0.1% | 1 |
| mismatch repair complex binding | 6 out of 7095 genes, 0.1% | 6 out of 7190 genes, 0.1% | 1 |
| MHC protein binding | 6 out of 7095 genes, 0.1% | 6 out of 7190 genes, 0.1% | 1 |
| SMAD binding | 6 out of 7095 genes, 0.1% | 6 out of 7190 genes, 0.1% | 1 |
| sugar transmembrane transporter activity | 6 out of 7095 genes, 0.1% | 6 out of 7190 genes, 0.1% | 1 |
| cofactor transporter activity | 6 out of 7095 genes, 0.1% | 6 out of 7190 genes, 0.1% | 1 |
| uridylyltransferase activity | 6 out of 7095 genes, 0.1% | 6 out of 7190 genes, 0.1% | 1 |
| identical protein binding | 116 out of 7095 genes, 1.6% | 119 out of 7190 genes, 1.7% | 1 |
| ATPase activity, coupled to transmembrane movement of ions | 71 out of 7095 genes, 1.0% | 73 out of 7190 genes, 1.0% | 1 |
| cation-transporting ATPase activity | 32 out of 7095 genes, 0.5% | 33 out of 7190 genes, 0.5% | 1 |
| N-acetyltransferase activity | 31 out of 7095 genes, 0.4% | 32 out of 7190 genes, 0.4% | 1 |
| transferase activity, transferring pentosyl groups | 31 out of 7095 genes, 0.4% | 32 out of 7190 genes, 0.4% | 1 |
| sulfur amino acid transmembrane transporter activity | 5 out of 7095 genes, 0.1% | 5 out of 7190 genes, 0.1% | 1 |
| chromatin binding | 5 out of 7095 genes, 0.1% | 5 out of 7190 genes, 0.1% | 1 |
| translation release factor activity | 5 out of 7095 genes, 0.1% | 5 out of 7190 genes, 0.1% | 1 |
| protein histidine kinase activity | 5 out of 7095 genes, 0.1% | 5 out of 7190 genes, 0.1% | 1 |
| endopeptidase inhibitor activity | 5 out of 7095 genes, 0.1% | 5 out of 7190 genes, 0.1% | 1 |
| chloride channel activity | 5 out of 7095 genes, 0.1% | 5 out of 7190 genes, 0.1% | 1 |
| glycosaminoglycan binding | 5 out of 7095 genes, 0.1% | 5 out of 7190 genes, 0.1% | 1 |
| ATP-dependent helicase activity | 5 out of 7095 genes, 0.1% | 5 out of 7190 genes, 0.1% | 1 |
| O-methyltransferase activity | 5 out of 7095 genes, 0.1% | 5 out of 7190 genes, 0.1% | 1 |
| G-protein coupled amine receptor activity | 5 out of 7095 genes, 0.1% | 5 out of 7190 genes, 0.1% | 1 |
| metalloexopeptidase activity | 5 out of 7095 genes, 0.1% | 5 out of 7190 genes, 0.1% | 1 |
| nucleotidase activity | 5 out of 7095 genes, 0.1% | 5 out of 7190 genes, 0.1% | 1 |
| anion:cation symporter activity | 5 out of 7095 genes, 0.1% | 5 out of 7190 genes, 0.1% | 1 |
| ammonia ligase activity | 5 out of 7095 genes, 0.1% | 5 out of 7190 genes, 0.1% | 1 |
| palmitoyltransferase activity | 5 out of 7095 genes, 0.1% | 5 out of 7190 genes, 0.1% | 1 |
| oxidoreductase activity, acting on CH or CH2 groups | 5 out of 7095 genes, 0.1% | 5 out of 7190 genes, 0.1% | 1 |
| oxidoreductase activity, acting on CH or CH2 groups, disulfide as acceptor | 5 out of 7095 genes, 0.1% | 5 out of 7190 genes, 0.1% | 1 |
| phosphotransferase activity, nitrogenous group as acceptor | 5 out of 7095 genes, 0.1% | 5 out of 7190 genes, 0.1% | 1 |
| acid-ammonia (or amide) ligase activity | 5 out of 7095 genes, 0.1% | 5 out of 7190 genes, 0.1% | 1 |
| carbon-nitrogen ligase activity, with glutamine as amido-N-donor | 5 out of 7095 genes, 0.1% | 5 out of 7190 genes, 0.1% | 1 |
| snRNA binding | 5 out of 7095 genes, 0.1% | 5 out of 7190 genes, 0.1% | 1 |
| receptor regulator activity | 5 out of 7095 genes, 0.1% | 5 out of 7190 genes, 0.1% | 1 |
| alkali metal ion binding | 5 out of 7095 genes, 0.1% | 5 out of 7190 genes, 0.1% | 1 |
| DNA insertion or deletion binding | 5 out of 7095 genes, 0.1% | 5 out of 7190 genes, 0.1% | 1 |
| phosphoinositide 3-kinase activity | 5 out of 7095 genes, 0.1% | 5 out of 7190 genes, 0.1% | 1 |
| glutamate receptor binding | 5 out of 7095 genes, 0.1% | 5 out of 7190 genes, 0.1% | 1 |
| cell surface binding | 5 out of 7095 genes, 0.1% | 5 out of 7190 genes, 0.1% | 1 |
| purine NTP-dependent helicase activity | 5 out of 7095 genes, 0.1% | 5 out of 7190 genes, 0.1% | 1 |
| cytidylyltransferase activity | 5 out of 7095 genes, 0.1% | 5 out of 7190 genes, 0.1% | 1 |
| guanylyltransferase activity | 5 out of 7095 genes, 0.1% | 5 out of 7190 genes, 0.1% | 1 |
| endopeptidase activity | 109 out of 7095 genes, 1.5% | 112 out of 7190 genes, 1.6% | 1 |
| anion transmembrane transporter activity | 29 out of 7095 genes, 0.4% | 30 out of 7190 genes, 0.4% | 1 |
| oxidoreductase activity, acting on CH-OH group of donors | 64 out of 7095 genes, 0.9% | 66 out of 7190 genes, 0.9% | 1 |
| primary active transmembrane transporter activity | 104 out of 7095 genes, 1.5% | 107 out of 7190 genes, 1.5% | 1 |
| P-P-bond-hydrolysis-driven transmembrane transporter activity | 104 out of 7095 genes, 1.5% | 107 out of 7190 genes, 1.5% | 1 |
| pyrimidine binding | 4 out of 7095 genes, 0.1% | 4 out of 7190 genes, 0.1% | 1 |
| single-stranded RNA binding | 4 out of 7095 genes, 0.1% | 4 out of 7190 genes, 0.1% | 1 |
| fatty acid synthase activity | 4 out of 7095 genes, 0.1% | 4 out of 7190 genes, 0.1% | 1 |
| monooxygenase activity | 4 out of 7095 genes, 0.1% | 4 out of 7190 genes, 0.1% | 1 |
| transmembrane receptor protein tyrosine kinase activity | 4 out of 7095 genes, 0.1% | 4 out of 7190 genes, 0.1% | 1 |
| pyruvate dehydrogenase activity | 4 out of 7095 genes, 0.1% | 4 out of 7190 genes, 0.1% | 1 |
| sulfate adenylyltransferase activity | 4 out of 7095 genes, 0.1% | 4 out of 7190 genes, 0.1% | 1 |
| transmembrane receptor protein serine/threonine kinase signaling protein activity | 4 out of 7095 genes, 0.1% | 4 out of 7190 genes, 0.1% | 1 |
| transforming growth factor beta receptor, cytoplasmic mediator activity | 4 out of 7095 genes, 0.1% | 4 out of 7190 genes, 0.1% | 1 |
| microtubule binding | 4 out of 7095 genes, 0.1% | 4 out of 7190 genes, 0.1% | 1 |
| ion channel inhibitor activity | 4 out of 7095 genes, 0.1% | 4 out of 7190 genes, 0.1% | 1 |
| acetylgalactosaminyltransferase activity | 4 out of 7095 genes, 0.1% | 4 out of 7190 genes, 0.1% | 1 |
| fucosyltransferase activity | 4 out of 7095 genes, 0.1% | 4 out of 7190 genes, 0.1% | 1 |
| monoamine transmembrane transporter activity | 4 out of 7095 genes, 0.1% | 4 out of 7190 genes, 0.1% | 1 |
| peptide receptor activity, G-protein coupled | 4 out of 7095 genes, 0.1% | 4 out of 7190 genes, 0.1% | 1 |
| NAD(P)+ transhydrogenase activity | 4 out of 7095 genes, 0.1% | 4 out of 7190 genes, 0.1% | 1 |
| glyceraldehyde-3-phosphate dehydrogenase activity | 4 out of 7095 genes, 0.1% | 4 out of 7190 genes, 0.1% | 1 |
| galactosidase activity | 4 out of 7095 genes, 0.1% | 4 out of 7190 genes, 0.1% | 1 |
| hexosaminidase activity | 4 out of 7095 genes, 0.1% | 4 out of 7190 genes, 0.1% | 1 |
| glutamate synthase activity | 4 out of 7095 genes, 0.1% | 4 out of 7190 genes, 0.1% | 1 |
| steroid dehydrogenase activity | 4 out of 7095 genes, 0.1% | 4 out of 7190 genes, 0.1% | 1 |
| channel inhibitor activity | 4 out of 7095 genes, 0.1% | 4 out of 7190 genes, 0.1% | 1 |
| oxidoreductase activity, acting on the aldehyde or oxo group of donors, disulfide as acceptor | 4 out of 7095 genes, 0.1% | 4 out of 7190 genes, 0.1% | 1 |
| oxidoreductase activity, acting on NADH or NADPH, NAD or NADP as acceptor | 4 out of 7095 genes, 0.1% | 4 out of 7190 genes, 0.1% | 1 |
| oxidoreductase activity, acting on paired donors, with incorporation or reduction of molecular oxygen, 2-oxoglutarate as one donor, and incorporation of one atom each of oxygen into both donors | 4 out of 7095 genes, 0.1% | 4 out of 7190 genes, 0.1% | 1 |
| hydrolase activity, acting on ether bonds | 4 out of 7095 genes, 0.1% | 4 out of 7190 genes, 0.1% | 1 |
| trialkylsulfonium hydrolase activity | 4 out of 7095 genes, 0.1% | 4 out of 7190 genes, 0.1% | 1 |
| hydrolase activity, acting on carbon-nitrogen (but not peptide) bonds, in cyclic amidines | 4 out of 7095 genes, 0.1% | 4 out of 7190 genes, 0.1% | 1 |
| racemase and epimerase activity | 4 out of 7095 genes, 0.1% | 4 out of 7190 genes, 0.1% | 1 |
| racemase and epimerase activity, acting on carbohydrates and derivatives | 4 out of 7095 genes, 0.1% | 4 out of 7190 genes, 0.1% | 1 |
| intramolecular oxidoreductase activity, interconverting aldoses and ketoses | 4 out of 7095 genes, 0.1% | 4 out of 7190 genes, 0.1% | 1 |
| GMP binding | 4 out of 7095 genes, 0.1% | 4 out of 7190 genes, 0.1% | 1 |
| carbohydrate phosphatase activity | 4 out of 7095 genes, 0.1% | 4 out of 7190 genes, 0.1% | 1 |
| receptor activator activity | 4 out of 7095 genes, 0.1% | 4 out of 7190 genes, 0.1% | 1 |
| protein binding, bridging | 4 out of 7095 genes, 0.1% | 4 out of 7190 genes, 0.1% | 1 |
| demethylase activity | 4 out of 7095 genes, 0.1% | 4 out of 7190 genes, 0.1% | 1 |
| histone demethylase activity | 4 out of 7095 genes, 0.1% | 4 out of 7190 genes, 0.1% | 1 |
| peptide N-acetyltransferase activity | 4 out of 7095 genes, 0.1% | 4 out of 7190 genes, 0.1% | 1 |
| histone kinase activity | 4 out of 7095 genes, 0.1% | 4 out of 7190 genes, 0.1% | 1 |
| hormone binding | 4 out of 7095 genes, 0.1% | 4 out of 7190 genes, 0.1% | 1 |
| ubiquitin binding | 4 out of 7095 genes, 0.1% | 4 out of 7190 genes, 0.1% | 1 |
| glutamate synthase activity, NADH or NADPH as acceptor | 4 out of 7095 genes, 0.1% | 4 out of 7190 genes, 0.1% | 1 |
| transition metal ion transmembrane transporter activity | 4 out of 7095 genes, 0.1% | 4 out of 7190 genes, 0.1% | 1 |
| beta-1,3-galactosyltransferase activity | 4 out of 7095 genes, 0.1% | 4 out of 7190 genes, 0.1% | 1 |
| protein-glycine ligase activity | 4 out of 7095 genes, 0.1% | 4 out of 7190 genes, 0.1% | 1 |
| monovalent inorganic cation transmembrane transporter activity | 61 out of 7095 genes, 0.9% | 63 out of 7190 genes, 0.9% | 1 |
| actin binding | 26 out of 7095 genes, 0.4% | 27 out of 7190 genes, 0.4% | 1 |
| cofactor binding | 57 out of 7095 genes, 0.8% | 59 out of 7190 genes, 0.8% | 1 |
| ATPase activity, coupled to transmembrane movement of ions, phosphorylative mechanism | 24 out of 7095 genes, 0.3% | 25 out of 7190 genes, 0.3% | 1 |
| lysine N-acetyltransferase activity | 23 out of 7095 genes, 0.3% | 24 out of 7190 genes, 0.3% | 1 |
| gated channel activity | 55 out of 7095 genes, 0.8% | 57 out of 7190 genes, 0.8% | 1 |
| microfilament motor activity | 3 out of 7095 genes, 0.0% | 3 out of 7190 genes, 0.0% | 1 |
| inositol pyrophosphate synthase activity | 3 out of 7095 genes, 0.0% | 3 out of 7190 genes, 0.0% | 1 |
| inositol hexakisphosphate kinase activity | 3 out of 7095 genes, 0.0% | 3 out of 7190 genes, 0.0% | 1 |
| regulatory region DNA binding | 3 out of 7095 genes, 0.0% | 3 out of 7190 genes, 0.0% | 1 |
| regulatory region nucleic acid binding | 3 out of 7095 genes, 0.0% | 3 out of 7190 genes, 0.0% | 1 |
| microtubule motor activity | 3 out of 7095 genes, 0.0% | 3 out of 7190 genes, 0.0% | 1 |
| peroxidase activity | 3 out of 7095 genes, 0.0% | 3 out of 7190 genes, 0.0% | 1 |
| phospholipase A2 activity | 3 out of 7095 genes, 0.0% | 3 out of 7190 genes, 0.0% | 1 |
| MAP kinase kinase kinase activity | 3 out of 7095 genes, 0.0% | 3 out of 7190 genes, 0.0% | 1 |
| protein kinase inhibitor activity | 3 out of 7095 genes, 0.0% | 3 out of 7190 genes, 0.0% | 1 |
| cysteine-type endopeptidase inhibitor activity | 3 out of 7095 genes, 0.0% | 3 out of 7190 genes, 0.0% | 1 |
| transforming growth factor beta receptor activity, type I | 3 out of 7095 genes, 0.0% | 3 out of 7190 genes, 0.0% | 1 |
| cytokine activity | 3 out of 7095 genes, 0.0% | 3 out of 7190 genes, 0.0% | 1 |
| transforming growth factor beta receptor binding | 3 out of 7095 genes, 0.0% | 3 out of 7190 genes, 0.0% | 1 |
| sodium:amino acid symporter activity | 3 out of 7095 genes, 0.0% | 3 out of 7190 genes, 0.0% | 1 |
| L-glutamate transmembrane transporter activity | 3 out of 7095 genes, 0.0% | 3 out of 7190 genes, 0.0% | 1 |
| purine transmembrane transporter activity | 3 out of 7095 genes, 0.0% | 3 out of 7190 genes, 0.0% | 1 |
| glucose transmembrane transporter activity | 3 out of 7095 genes, 0.0% | 3 out of 7190 genes, 0.0% | 1 |
| cation:amino acid symporter activity | 3 out of 7095 genes, 0.0% | 3 out of 7190 genes, 0.0% | 1 |
| steroid binding | 3 out of 7095 genes, 0.0% | 3 out of 7190 genes, 0.0% | 1 |
| DNA-dependent ATPase activity | 3 out of 7095 genes, 0.0% | 3 out of 7190 genes, 0.0% | 1 |
| drug binding | 3 out of 7095 genes, 0.0% | 3 out of 7190 genes, 0.0% | 1 |
| neuropeptide receptor activity | 3 out of 7095 genes, 0.0% | 3 out of 7190 genes, 0.0% | 1 |
| RNA guanylyltransferase activity | 3 out of 7095 genes, 0.0% | 3 out of 7190 genes, 0.0% | 1 |
| 5'-3' exonuclease activity | 3 out of 7095 genes, 0.0% | 3 out of 7190 genes, 0.0% | 1 |
| electron carrier activity | 3 out of 7095 genes, 0.0% | 3 out of 7190 genes, 0.0% | 1 |
| carboxyl-O-methyltransferase activity | 3 out of 7095 genes, 0.0% | 3 out of 7190 genes, 0.0% | 1 |
| promoter binding | 3 out of 7095 genes, 0.0% | 3 out of 7190 genes, 0.0% | 1 |
| neutral amino acid transmembrane transporter activity | 3 out of 7095 genes, 0.0% | 3 out of 7190 genes, 0.0% | 1 |
| nucleobase transmembrane transporter activity | 3 out of 7095 genes, 0.0% | 3 out of 7190 genes, 0.0% | 1 |
| cation:chloride symporter activity | 3 out of 7095 genes, 0.0% | 3 out of 7190 genes, 0.0% | 1 |
| acetylcholine receptor activity | 3 out of 7095 genes, 0.0% | 3 out of 7190 genes, 0.0% | 1 |
| cation:cation antiporter activity | 3 out of 7095 genes, 0.0% | 3 out of 7190 genes, 0.0% | 1 |
| CoA hydrolase activity | 3 out of 7095 genes, 0.0% | 3 out of 7190 genes, 0.0% | 1 |
| C-acyltransferase activity | 3 out of 7095 genes, 0.0% | 3 out of 7190 genes, 0.0% | 1 |
| acylglycerol O-acyltransferase activity | 3 out of 7095 genes, 0.0% | 3 out of 7190 genes, 0.0% | 1 |
| tRNA (guanine) methyltransferase activity | 3 out of 7095 genes, 0.0% | 3 out of 7190 genes, 0.0% | 1 |
| oxidoreductase activity, acting on peroxide as acceptor | 3 out of 7095 genes, 0.0% | 3 out of 7190 genes, 0.0% | 1 |
| oxidoreductase activity, acting on paired donors, with incorporation or reduction of molecular oxygen, reduced pteridine as one donor, and incorporation of one atom of oxygen | 3 out of 7095 genes, 0.0% | 3 out of 7190 genes, 0.0% | 1 |
| oxidoreductase activity, oxidizing metal ions | 3 out of 7095 genes, 0.0% | 3 out of 7190 genes, 0.0% | 1 |
| oxidoreductase activity, oxidizing metal ions, oxygen as acceptor | 3 out of 7095 genes, 0.0% | 3 out of 7190 genes, 0.0% | 1 |
| hydroxymethyl-, formyl- and related transferase activity | 3 out of 7095 genes, 0.0% | 3 out of 7190 genes, 0.0% | 1 |
| transferase activity, transferring aldehyde or ketonic groups | 3 out of 7095 genes, 0.0% | 3 out of 7190 genes, 0.0% | 1 |
| intramolecular oxidoreductase activity, interconverting keto- and enol-groups | 3 out of 7095 genes, 0.0% | 3 out of 7190 genes, 0.0% | 1 |
| G-protein coupled acetylcholine receptor activity | 3 out of 7095 genes, 0.0% | 3 out of 7190 genes, 0.0% | 1 |
| peptide hormone binding | 3 out of 7095 genes, 0.0% | 3 out of 7190 genes, 0.0% | 1 |
| NAD-dependent histone deacetylase activity | 3 out of 7095 genes, 0.0% | 3 out of 7190 genes, 0.0% | 1 |
| nucleoside kinase activity | 3 out of 7095 genes, 0.0% | 3 out of 7190 genes, 0.0% | 1 |
| kinase inhibitor activity | 3 out of 7095 genes, 0.0% | 3 out of 7190 genes, 0.0% | 1 |
| phosphatase inhibitor activity | 3 out of 7095 genes, 0.0% | 3 out of 7190 genes, 0.0% | 1 |
| protein serine/threonine kinase inhibitor activity | 3 out of 7095 genes, 0.0% | 3 out of 7190 genes, 0.0% | 1 |
| NAD-dependent protein deacetylase activity | 3 out of 7095 genes, 0.0% | 3 out of 7190 genes, 0.0% | 1 |
| histone threonine kinase activity | 3 out of 7095 genes, 0.0% | 3 out of 7190 genes, 0.0% | 1 |
| 5,10-methylenetetrahydrofolate-dependent methyltransferase activity | 3 out of 7095 genes, 0.0% | 3 out of 7190 genes, 0.0% | 1 |
| acetylcholine binding | 3 out of 7095 genes, 0.0% | 3 out of 7190 genes, 0.0% | 1 |
| neuropeptide binding | 3 out of 7095 genes, 0.0% | 3 out of 7190 genes, 0.0% | 1 |
| transcription regulatory region DNA binding | 3 out of 7095 genes, 0.0% | 3 out of 7190 genes, 0.0% | 1 |
| dynein binding | 3 out of 7095 genes, 0.0% | 3 out of 7190 genes, 0.0% | 1 |
| 3',5'-cyclic-GMP phosphodiesterase activity | 3 out of 7095 genes, 0.0% | 3 out of 7190 genes, 0.0% | 1 |
| alanine-oxo-acid transaminase activity | 3 out of 7095 genes, 0.0% | 3 out of 7190 genes, 0.0% | 1 |
| protein carboxyl O-methyltransferase activity | 3 out of 7095 genes, 0.0% | 3 out of 7190 genes, 0.0% | 1 |
| NADH dehydrogenase activity | 22 out of 7095 genes, 0.3% | 23 out of 7190 genes, 0.3% | 1 |
| symporter activity | 22 out of 7095 genes, 0.3% | 23 out of 7190 genes, 0.3% | 1 |
| oxidoreductase activity, acting on NADH or NADPH, quinone or similar compound as acceptor | 22 out of 7095 genes, 0.3% | 23 out of 7190 genes, 0.3% | 1 |
| oxidoreductase activity, acting on the aldehyde or oxo group of donors | 22 out of 7095 genes, 0.3% | 23 out of 7190 genes, 0.3% | 1 |
| NADH dehydrogenase (quinone) activity | 22 out of 7095 genes, 0.3% | 23 out of 7190 genes, 0.3% | 1 |
| transferase activity, transferring alkyl or aryl (other than methyl) groups | 21 out of 7095 genes, 0.3% | 22 out of 7190 genes, 0.3% | 1 |
| solute:cation symporter activity | 19 out of 7095 genes, 0.3% | 20 out of 7190 genes, 0.3% | 1 |
| DNA secondary structure binding | 2 out of 7095 genes, 0.0% | 2 out of 7190 genes, 0.0% | 1 |
| mismatch base pair DNA N-glycosylase activity | 2 out of 7095 genes, 0.0% | 2 out of 7190 genes, 0.0% | 1 |
| damaged DNA binding | 2 out of 7095 genes, 0.0% | 2 out of 7190 genes, 0.0% | 1 |
| protein C-terminal carboxyl O-methyltransferase activity | 2 out of 7095 genes, 0.0% | 2 out of 7190 genes, 0.0% | 1 |
| DNA photolyase activity | 2 out of 7095 genes, 0.0% | 2 out of 7190 genes, 0.0% | 1 |
| ATP-dependent DNA helicase activity | 2 out of 7095 genes, 0.0% | 2 out of 7190 genes, 0.0% | 1 |
| aldehyde dehydrogenase [NAD(P)+] activity | 2 out of 7095 genes, 0.0% | 2 out of 7190 genes, 0.0% | 1 |
| carboxypeptidase activity | 2 out of 7095 genes, 0.0% | 2 out of 7190 genes, 0.0% | 1 |
| glycerol-3-phosphate dehydrogenase activity | 2 out of 7095 genes, 0.0% | 2 out of 7190 genes, 0.0% | 1 |
| lactate dehydrogenase activity | 2 out of 7095 genes, 0.0% | 2 out of 7190 genes, 0.0% | 1 |
| nucleotide diphosphatase activity | 2 out of 7095 genes, 0.0% | 2 out of 7190 genes, 0.0% | 1 |
| beta-N-acetylhexosaminidase activity | 2 out of 7095 genes, 0.0% | 2 out of 7190 genes, 0.0% | 1 |
| protein kinase C activity | 2 out of 7095 genes, 0.0% | 2 out of 7190 genes, 0.0% | 1 |
| MAP kinase activity | 2 out of 7095 genes, 0.0% | 2 out of 7190 genes, 0.0% | 1 |
| MAP kinase kinase activity | 2 out of 7095 genes, 0.0% | 2 out of 7190 genes, 0.0% | 1 |
| Rho guanyl-nucleotide exchange factor activity | 2 out of 7095 genes, 0.0% | 2 out of 7190 genes, 0.0% | 1 |
| GDP-dissociation inhibitor activity | 2 out of 7095 genes, 0.0% | 2 out of 7190 genes, 0.0% | 1 |
| Rho GTPase activator activity | 2 out of 7095 genes, 0.0% | 2 out of 7190 genes, 0.0% | 1 |
| dicarboxylic acid transmembrane transporter activity | 2 out of 7095 genes, 0.0% | 2 out of 7190 genes, 0.0% | 1 |
| neurotransmitter:sodium symporter activity | 2 out of 7095 genes, 0.0% | 2 out of 7190 genes, 0.0% | 1 |
| copper ion transmembrane transporter activity | 2 out of 7095 genes, 0.0% | 2 out of 7190 genes, 0.0% | 1 |
| iron ion transmembrane transporter activity | 2 out of 7095 genes, 0.0% | 2 out of 7190 genes, 0.0% | 1 |
| monovalent cation:hydrogen antiporter activity | 2 out of 7095 genes, 0.0% | 2 out of 7190 genes, 0.0% | 1 |
| copper ion binding | 2 out of 7095 genes, 0.0% | 2 out of 7190 genes, 0.0% | 1 |
| monocarboxylic acid transmembrane transporter activity | 2 out of 7095 genes, 0.0% | 2 out of 7190 genes, 0.0% | 1 |
| MAP kinase kinase kinase kinase activity | 2 out of 7095 genes, 0.0% | 2 out of 7190 genes, 0.0% | 1 |
| 3'-5' exonuclease activity | 2 out of 7095 genes, 0.0% | 2 out of 7190 genes, 0.0% | 1 |
| histone-arginine N-methyltransferase activity | 2 out of 7095 genes, 0.0% | 2 out of 7190 genes, 0.0% | 1 |
| tetracycline transporter activity | 2 out of 7095 genes, 0.0% | 2 out of 7190 genes, 0.0% | 1 |
| rRNA methyltransferase activity | 2 out of 7095 genes, 0.0% | 2 out of 7190 genes, 0.0% | 1 |
| mannose-phosphate guanylyltransferase activity | 2 out of 7095 genes, 0.0% | 2 out of 7190 genes, 0.0% | 1 |
| glucuronosyltransferase activity | 2 out of 7095 genes, 0.0% | 2 out of 7190 genes, 0.0% | 1 |
| phosphate transmembrane transporter activity | 2 out of 7095 genes, 0.0% | 2 out of 7190 genes, 0.0% | 1 |
| bile acid transmembrane transporter activity | 2 out of 7095 genes, 0.0% | 2 out of 7190 genes, 0.0% | 1 |
| purine nucleoside transmembrane transporter activity | 2 out of 7095 genes, 0.0% | 2 out of 7190 genes, 0.0% | 1 |
| drug transmembrane transporter activity | 2 out of 7095 genes, 0.0% | 2 out of 7190 genes, 0.0% | 1 |
| calcium-activated potassium channel activity | 2 out of 7095 genes, 0.0% | 2 out of 7190 genes, 0.0% | 1 |
| C-palmitoyltransferase activity | 2 out of 7095 genes, 0.0% | 2 out of 7190 genes, 0.0% | 1 |
| cyclin-dependent protein kinase regulator activity | 2 out of 7095 genes, 0.0% | 2 out of 7190 genes, 0.0% | 1 |
| oxidoreductase activity, acting on the CH-NH group of donors | 2 out of 7095 genes, 0.0% | 2 out of 7190 genes, 0.0% | 1 |
| oxidoreductase activity, acting on the CH-NH group of donors, NAD or NADP as acceptor | 2 out of 7095 genes, 0.0% | 2 out of 7190 genes, 0.0% | 1 |
| oxidoreductase activity, acting on a sulfur group of donors, NAD or NADP as acceptor | 2 out of 7095 genes, 0.0% | 2 out of 7190 genes, 0.0% | 1 |
| oxidoreductase activity, acting on single donors with incorporation of molecular oxygen, incorporation of two atoms of oxygen | 2 out of 7095 genes, 0.0% | 2 out of 7190 genes, 0.0% | 1 |
| hydrolase activity, acting on carbon-nitrogen (but not peptide) bonds, in cyclic amides | 2 out of 7095 genes, 0.0% | 2 out of 7190 genes, 0.0% | 1 |
| endodeoxyribonuclease activity, producing 5'-phosphomonoesters | 2 out of 7095 genes, 0.0% | 2 out of 7190 genes, 0.0% | 1 |
| SAP kinase activity | 2 out of 7095 genes, 0.0% | 2 out of 7190 genes, 0.0% | 1 |
| nucleoside-diphosphatase activity | 2 out of 7095 genes, 0.0% | 2 out of 7190 genes, 0.0% | 1 |
| deoxynucleoside kinase activity | 2 out of 7095 genes, 0.0% | 2 out of 7190 genes, 0.0% | 1 |
| nucleotide kinase activity | 2 out of 7095 genes, 0.0% | 2 out of 7190 genes, 0.0% | 1 |
| protein phosphatase binding | 2 out of 7095 genes, 0.0% | 2 out of 7190 genes, 0.0% | 1 |
| cytokine binding | 2 out of 7095 genes, 0.0% | 2 out of 7190 genes, 0.0% | 1 |
| ion gated channel activity | 2 out of 7095 genes, 0.0% | 2 out of 7190 genes, 0.0% | 1 |
| single base insertion or deletion binding | 2 out of 7095 genes, 0.0% | 2 out of 7190 genes, 0.0% | 1 |
| dinucleotide insertion or deletion binding | 2 out of 7095 genes, 0.0% | 2 out of 7190 genes, 0.0% | 1 |
| histone demethylase activity (H3-K4 specific) | 2 out of 7095 genes, 0.0% | 2 out of 7190 genes, 0.0% | 1 |
| sterol binding | 2 out of 7095 genes, 0.0% | 2 out of 7190 genes, 0.0% | 1 |
| receptor serine/threonine kinase binding | 2 out of 7095 genes, 0.0% | 2 out of 7190 genes, 0.0% | 1 |
| steroid dehydrogenase activity, acting on the CH-OH group of donors, NAD or NADP as acceptor | 2 out of 7095 genes, 0.0% | 2 out of 7190 genes, 0.0% | 1 |
| steroid dehydrogenase activity, acting on the CH-CH group of donors | 2 out of 7095 genes, 0.0% | 2 out of 7190 genes, 0.0% | 1 |
| heparan sulfate sulfotransferase activity | 2 out of 7095 genes, 0.0% | 2 out of 7190 genes, 0.0% | 1 |
| antibiotic transporter activity | 2 out of 7095 genes, 0.0% | 2 out of 7190 genes, 0.0% | 1 |
| anion binding | 2 out of 7095 genes, 0.0% | 2 out of 7190 genes, 0.0% | 1 |
| copper-transporting ATPase activity | 2 out of 7095 genes, 0.0% | 2 out of 7190 genes, 0.0% | 1 |
| translation regulator activity | 2 out of 7095 genes, 0.0% | 2 out of 7190 genes, 0.0% | 1 |
| nucleoside-triphosphate diphosphatase activity | 2 out of 7095 genes, 0.0% | 2 out of 7190 genes, 0.0% | 1 |
| cell adhesion molecule binding | 2 out of 7095 genes, 0.0% | 2 out of 7190 genes, 0.0% | 1 |
| dioxygenase activity | 2 out of 7095 genes, 0.0% | 2 out of 7190 genes, 0.0% | 1 |
| UTP-monosaccharide-1-phosphate uridylyltransferase activity | 2 out of 7095 genes, 0.0% | 2 out of 7190 genes, 0.0% | 1 |
| inositol trisphosphate kinase activity | 2 out of 7095 genes, 0.0% | 2 out of 7190 genes, 0.0% | 1 |
| sn-glycerol-3-phosphate:ubiquinone oxidoreductase activity | 2 out of 7095 genes, 0.0% | 2 out of 7190 genes, 0.0% | 1 |
| lipase activator activity | 2 out of 7095 genes, 0.0% | 2 out of 7190 genes, 0.0% | 1 |
| fructose binding | 2 out of 7095 genes, 0.0% | 2 out of 7190 genes, 0.0% | 1 |
| tyrosyl-DNA phosphodiesterase activity | 2 out of 7095 genes, 0.0% | 2 out of 7190 genes, 0.0% | 1 |
| transmembrane receptor protein serine/threonine kinase binding | 2 out of 7095 genes, 0.0% | 2 out of 7190 genes, 0.0% | 1 |
| lysophospholipid acyltransferase activity | 2 out of 7095 genes, 0.0% | 2 out of 7190 genes, 0.0% | 1 |
| heme-copper terminal oxidase activity | 17 out of 7095 genes, 0.2% | 18 out of 7190 genes, 0.3% | 1 |
| small GTPase binding | 17 out of 7095 genes, 0.2% | 18 out of 7190 genes, 0.3% | 1 |
| GTPase binding | 17 out of 7095 genes, 0.2% | 18 out of 7190 genes, 0.3% | 1 |
| antiporter activity | 16 out of 7095 genes, 0.2% | 17 out of 7190 genes, 0.2% | 1 |
| Ras GTPase binding | 15 out of 7095 genes, 0.2% | 16 out of 7190 genes, 0.2% | 1 |
| oxidoreductase activity | 294 out of 7095 genes, 4.1% | 302 out of 7190 genes, 4.2% | 1 |
| glutamate receptor activity | 14 out of 7095 genes, 0.2% | 15 out of 7190 genes, 0.2% | 1 |
| ligase activity, forming carbon-sulfur bonds | 14 out of 7095 genes, 0.2% | 15 out of 7190 genes, 0.2% | 1 |
| adenylyltransferase activity | 14 out of 7095 genes, 0.2% | 15 out of 7190 genes, 0.2% | 1 |
| guanyl-nucleotide exchange factor activity | 13 out of 7095 genes, 0.2% | 14 out of 7190 genes, 0.2% | 1 |
| transferase activity, transferring sulfur-containing groups | 13 out of 7095 genes, 0.2% | 14 out of 7190 genes, 0.2% | 1 |
| N-acyltransferase activity | 35 out of 7095 genes, 0.5% | 37 out of 7190 genes, 0.5% | 1 |
| RNA methyltransferase activity | 12 out of 7095 genes, 0.2% | 13 out of 7190 genes, 0.2% | 1 |
| oxidoreductase activity, acting on the aldehyde or oxo group of donors, NAD or NADP as acceptor | 12 out of 7095 genes, 0.2% | 13 out of 7190 genes, 0.2% | 1 |
| ligand-gated ion channel activity | 34 out of 7095 genes, 0.5% | 36 out of 7190 genes, 0.5% | 1 |
| ligand-gated channel activity | 34 out of 7095 genes, 0.5% | 36 out of 7190 genes, 0.5% | 1 |
| excitatory extracellular ligand-gated ion channel activity | 11 out of 7095 genes, 0.2% | 12 out of 7190 genes, 0.2% | 1 |
| small protein activating enzyme activity | 11 out of 7095 genes, 0.2% | 12 out of 7190 genes, 0.2% | 1 |
| Rho GTPase binding | 11 out of 7095 genes, 0.2% | 12 out of 7190 genes, 0.2% | 1 |
| DNA topoisomerase activity | 10 out of 7095 genes, 0.1% | 11 out of 7190 genes, 0.2% | 1 |
| phosphorylase activity | 10 out of 7095 genes, 0.1% | 11 out of 7190 genes, 0.2% | 1 |
| solute:solute antiporter activity | 10 out of 7095 genes, 0.1% | 11 out of 7190 genes, 0.2% | 1 |
| oxidoreductase activity, acting on the CH-CH group of donors | 27 out of 7095 genes, 0.4% | 29 out of 7190 genes, 0.4% | 1 |
| Ras guanyl-nucleotide exchange factor activity | 8 out of 7095 genes, 0.1% | 9 out of 7190 genes, 0.1% | 1 |
| sulfurtransferase activity | 7 out of 7095 genes, 0.1% | 8 out of 7190 genes, 0.1% | 1 |
| tRNA methyltransferase activity | 6 out of 7095 genes, 0.1% | 7 out of 7190 genes, 0.1% | 1 |
| CoA-ligase activity | 6 out of 7095 genes, 0.1% | 7 out of 7190 genes, 0.1% | 1 |
| ligase activity, forming phosphoric ester bonds | 6 out of 7095 genes, 0.1% | 7 out of 7190 genes, 0.1% | 1 |
| oxidoreductase activity, acting on the CH-CH group of donors, NAD or NADP as acceptor | 21 out of 7095 genes, 0.3% | 23 out of 7190 genes, 0.3% | 1 |
| structural molecule activity | 104 out of 7095 genes, 1.5% | 109 out of 7190 genes, 1.5% | 1 |
| DNA ligase activity | 5 out of 7095 genes, 0.1% | 6 out of 7190 genes, 0.1% | 1 |
| aldo-keto reductase activity | 5 out of 7095 genes, 0.1% | 6 out of 7190 genes, 0.1% | 1 |
| inorganic anion transmembrane transporter activity | 5 out of 7095 genes, 0.1% | 6 out of 7190 genes, 0.1% | 1 |
| phosphotransferase activity, for other substituted phosphate groups | 5 out of 7095 genes, 0.1% | 6 out of 7190 genes, 0.1% | 1 |
| extracellular ligand-gated ion channel activity | 14 out of 7095 genes, 0.2% | 16 out of 7190 genes, 0.2% | 1 |
| carbon-sulfur lyase activity | 3 out of 7095 genes, 0.0% | 4 out of 7190 genes, 0.1% | 1 |
| transferase activity, transferring acyl groups, acyl groups converted into alkyl on transfer | 3 out of 7095 genes, 0.0% | 4 out of 7190 genes, 0.1% | 1 |
| cation:sugar symporter activity | 2 out of 7095 genes, 0.0% | 3 out of 7190 genes, 0.0% | 1 |
| anion:anion antiporter activity | 2 out of 7095 genes, 0.0% | 3 out of 7190 genes, 0.0% | 1 |
| Ral GTPase binding | 2 out of 7095 genes, 0.0% | 3 out of 7190 genes, 0.0% | 1 |
| immunoglobulin receptor binding | 2 out of 7095 genes, 0.0% | 3 out of 7190 genes, 0.0% | 1 |

| Gene Ontology term | Genes annotated to the term |
| --- | --- |
| binding | Unigene60957\_Sample\_011046841, Unigene28655\_Sample\_011046841, Unigene12969\_Sample\_011046841, Unigene40117\_Sample\_011046841, Unigene56478\_Sample\_011046841, Unigene44774\_Sample\_011046841, Unigene59064\_Sample\_011046841, Unigene56602\_Sample\_011046841, Unigene2784\_Sample\_011046841, Unigene57534\_Sample\_011046841, Unigene2263\_Sample\_011046841, Unigene24428\_Sample\_011046841, Unigene59887\_Sample\_011046841, Unigene60481\_Sample\_011046841, Unigene49534\_Sample\_011046841, Unigene12631\_Sample\_011046841, Unigene29658\_Sample\_011046841, Unigene51638\_Sample\_011046841, Unigene26047\_Sample\_011046841, Unigene49739\_Sample\_011046841, Unigene18749\_Sample\_011046841, Unigene14639\_Sample\_011046841, Unigene48121\_Sample\_011046841, Unigene60498\_Sample\_011046841, Unigene57589\_Sample\_011046841, Unigene35938\_Sample\_011046841, Unigene58925\_Sample\_011046841, Unigene21363\_Sample\_011046841, Unigene51845\_Sample\_011046841, Unigene39129\_Sample\_011046841, Unigene7462\_Sample\_011046841, Unigene13402\_Sample\_011046841, Unigene60235\_Sample\_011046841, Unigene3264\_Sample\_011046841, Unigene49657\_Sample\_011046841, Unigene46063\_Sample\_011046841, Unigene35621\_Sample\_011046841, Unigene47440\_Sample\_011046841, Unigene3880\_Sample\_011046841, Unigene46690\_Sample\_011046841, Unigene4253\_Sample\_011046841, Unigene54025\_Sample\_011046841, Unigene56425\_Sample\_011046841, Unigene12251\_Sample\_011046841, Unigene58290\_Sample\_011046841, Unigene17957\_Sample\_011046841, Unigene57357\_Sample\_011046841, Unigene35930\_Sample\_011046841, Unigene48164\_Sample\_011046841, Unigene58460\_Sample\_011046841, Unigene36311\_Sample\_011046841, Unigene57919\_Sample\_011046841, Unigene11014\_Sample\_011046841, Unigene58644\_Sample\_011046841, Unigene33743\_Sample\_011046841, Unigene41534\_Sample\_011046841, Unigene54614\_Sample\_011046841, Unigene53582\_Sample\_011046841, Unigene55357\_Sample\_011046841, Unigene44835\_Sample\_011046841, Unigene13859\_Sample\_011046841, Unigene5894\_Sample\_011046841, Unigene56024\_Sample\_011046841, Unigene57216\_Sample\_011046841, Unigene41085\_Sample\_011046841, Unigene32616\_Sample\_011046841, Unigene54440\_Sample\_011046841, Unigene39654\_Sample\_011046841, Unigene7899\_Sample\_011046841, Unigene59696\_Sample\_011046841, Unigene42733\_Sample\_011046841, Unigene58707\_Sample\_011046841, Unigene49989\_Sample\_011046841, Unigene55509\_Sample\_011046841, Unigene59299\_Sample\_011046841, Unigene33441\_Sample\_011046841, Unigene56232\_Sample\_011046841, Unigene12998\_Sample\_011046841, Unigene53029\_Sample\_011046841, Unigene4545\_Sample\_011046841, Unigene34856\_Sample\_011046841, Unigene51837\_Sample\_011046841, Unigene47633\_Sample\_011046841, Unigene52998\_Sample\_011046841, Unigene9862\_Sample\_011046841, Unigene38650\_Sample\_011046841, Unigene26531\_Sample\_011046841, Unigene28897\_Sample\_011046841, Unigene48054\_Sample\_011046841, Unigene27066\_Sample\_011046841, Unigene42781\_Sample\_011046841, Unigene56479\_Sample\_011046841, Unigene52614\_Sample\_011046841, Unigene12390\_Sample\_011046841, Unigene16060\_Sample\_011046841, Unigene27615\_Sample\_011046841, Unigene19789\_Sample\_011046841, Unigene57802\_Sample\_011046841, Unigene59199\_Sample\_011046841, Unigene60344\_Sample\_011046841, Unigene57438\_Sample\_011046841, Unigene55707\_Sample\_011046841, Unigene25152\_Sample\_011046841, Unigene30665\_Sample\_011046841, Unigene10448\_Sample\_011046841, Unigene58048\_Sample\_011046841, Unigene51\_Sample\_011046841, Unigene51144\_Sample\_011046841, Unigene58409\_Sample\_011046841, Unigene59748\_Sample\_011046841, Unigene13909\_Sample\_011046841, Unigene51298\_Sample\_011046841, Unigene60035\_Sample\_011046841, Unigene59178\_Sample\_011046841, Unigene20863\_Sample\_011046841, Unigene37613\_Sample\_011046841, Unigene6223\_Sample\_011046841, Unigene13815\_Sample\_011046841, Unigene23937\_Sample\_011046841, Unigene52611\_Sample\_011046841, Unigene5795\_Sample\_011046841, Unigene18836\_Sample\_011046841, Unigene21808\_Sample\_011046841, Unigene13844\_Sample\_011046841, Unigene33186\_Sample\_011046841, Unigene59986\_Sample\_011046841, Unigene44894\_Sample\_011046841, Unigene13217\_Sample\_011046841, Unigene60413\_Sample\_011046841, Unigene53170\_Sample\_011046841, Unigene17390\_Sample\_011046841, Unigene38030\_Sample\_011046841, Unigene56209\_Sample\_011046841, Unigene7020\_Sample\_011046841, Unigene16513\_Sample\_011046841, Unigene41346\_Sample\_011046841, Unigene31586\_Sample\_011046841, Unigene11040\_Sample\_011046841, Unigene3531\_Sample\_011046841, Unigene6573\_Sample\_011046841, Unigene2768\_Sample\_011046841, Unigene13838\_Sample\_011046841, Unigene19914\_Sample\_011046841, Unigene23333\_Sample\_011046841, Unigene60166\_Sample\_011046841, Unigene40116\_Sample\_011046841, Unigene23250\_Sample\_011046841, Unigene13321\_Sample\_011046841, Unigene31438\_Sample\_011046841, Unigene51857\_Sample\_011046841, Unigene60872\_Sample\_011046841, Unigene57004\_Sample\_011046841, Unigene12920\_Sample\_011046841, Unigene10765\_Sample\_011046841, Unigene35082\_Sample\_011046841, Unigene57892\_Sample\_011046841, Unigene21988\_Sample\_011046841, Unigene44330\_Sample\_011046841, Unigene5623\_Sample\_011046841, Unigene60019\_Sample\_011046841, Unigene2521\_Sample\_011046841, Unigene59709\_Sample\_011046841, Unigene55041\_Sample\_011046841, Unigene8288\_Sample\_011046841, Unigene36481\_Sample\_011046841, Unigene59016\_Sample\_011046841, Unigene41362\_Sample\_011046841, Unigene16813\_Sample\_011046841, Unigene59840\_Sample\_011046841, Unigene47927\_Sample\_011046841, Unigene53794\_Sample\_011046841, Unigene50945\_Sample\_011046841, Unigene45539\_Sample\_011046841, Unigene40737\_Sample\_011046841, Unigene13826\_Sample\_011046841, Unigene26696\_Sample\_011046841, Unigene24650\_Sample\_011046841, Unigene60948\_Sample\_011046841, Unigene54833\_Sample\_011046841, Unigene58673\_Sample\_011046841, Unigene13471\_Sample\_011046841, Unigene57418\_Sample\_011046841, Unigene60494\_Sample\_011046841, Unigene28322\_Sample\_011046841, Unigene12142\_Sample\_011046841, Unigene33197\_Sample\_011046841, Unigene52432\_Sample\_011046841, Unigene5402\_Sample\_011046841, Unigene59430\_Sample\_011046841, Unigene48406\_Sample\_011046841, Unigene13096\_Sample\_011046841, Unigene58913\_Sample\_011046841, Unigene56295\_Sample\_011046841, Unigene54214\_Sample\_011046841, Unigene60506\_Sample\_011046841, Unigene29827\_Sample\_011046841, Unigene28200\_Sample\_011046841, Unigene56865\_Sample\_011046841, Unigene12748\_Sample\_011046841, Unigene9545\_Sample\_011046841, Unigene51253\_Sample\_011046841, Unigene43848\_Sample\_011046841, Unigene48422\_Sample\_011046841, Unigene51559\_Sample\_011046841, Unigene39242\_Sample\_011046841, Unigene58227\_Sample\_011046841, Unigene8899\_Sample\_011046841, Unigene59033\_Sample\_011046841, Unigene51682\_Sample\_011046841, Unigene50477\_Sample\_011046841, Unigene45138\_Sample\_011046841, Unigene44021\_Sample\_011046841, Unigene47177\_Sample\_011046841, Unigene60900\_Sample\_011046841, Unigene59482\_Sample\_011046841, Unigene18672\_Sample\_011046841, Unigene57864\_Sample\_011046841, Unigene6164\_Sample\_011046841, Unigene55261\_Sample\_011046841, Unigene33091\_Sample\_011046841, Unigene33643\_Sample\_011046841, Unigene30466\_Sample\_011046841, Unigene58870\_Sample\_011046841, Unigene47261\_Sample\_011046841, Unigene5544\_Sample\_011046841, Unigene49912\_Sample\_011046841, Unigene59447\_Sample\_011046841, Unigene55026\_Sample\_011046841, Unigene8070\_Sample\_011046841, Unigene727\_Sample\_011046841, Unigene50269\_Sample\_011046841, Unigene13719\_Sample\_011046841, Unigene49410\_Sample\_011046841, Unigene13662\_Sample\_011046841, Unigene11366\_Sample\_011046841, Unigene52226\_Sample\_011046841, Unigene58817\_Sample\_011046841, Unigene57537\_Sample\_011046841, Unigene60610\_Sample\_011046841, Unigene40258\_Sample\_011046841, Unigene35261\_Sample\_011046841, Unigene2189\_Sample\_011046841, Unigene38963\_Sample\_011046841, Unigene49303\_Sample\_011046841, Unigene15487\_Sample\_011046841, Unigene7826\_Sample\_011046841, Unigene33507\_Sample\_011046841, Unigene15415\_Sample\_011046841, Unigene52688\_Sample\_011046841, Unigene55861\_Sample\_011046841, Unigene40006\_Sample\_011046841, Unigene3486\_Sample\_011046841, Unigene50302\_Sample\_011046841, Unigene33846\_Sample\_011046841, Unigene58175\_Sample\_011046841, Unigene9572\_Sample\_011046841, Unigene43918\_Sample\_011046841, Unigene35589\_Sample\_011046841, Unigene4308\_Sample\_011046841, Unigene42281\_Sample\_011046841, Unigene4523\_Sample\_011046841, Unigene19556\_Sample\_011046841, Unigene42820\_Sample\_011046841, Unigene25891\_Sample\_011046841, Unigene10941\_Sample\_011046841, Unigene16741\_Sample\_011046841, Unigene47680\_Sample\_011046841, Unigene55432\_Sample\_011046841, Unigene26055\_Sample\_011046841, Unigene48105\_Sample\_011046841, Unigene44438\_Sample\_011046841, Unigene57239\_Sample\_011046841, Unigene40034\_Sample\_011046841, Unigene55335\_Sample\_011046841, Unigene55909\_Sample\_011046841, Unigene28455\_Sample\_011046841, Unigene36149\_Sample\_011046841, Unigene57033\_Sample\_011046841, Unigene58721\_Sample\_011046841, Unigene13983\_Sample\_011046841, Unigene5533\_Sample\_011046841, Unigene41472\_Sample\_011046841, Unigene57862\_Sample\_011046841, Unigene8480\_Sample\_011046841, Unigene10961\_Sample\_011046841, Unigene9067\_Sample\_011046841, Unigene59344\_Sample\_011046841, Unigene33958\_Sample\_011046841, Unigene1477\_Sample\_011046841, Unigene58365\_Sample\_011046841, Unigene11053\_Sample\_011046841, Unigene51717\_Sample\_011046841, Unigene57765\_Sample\_011046841, Unigene46688\_Sample\_011046841, Unigene12837\_Sample\_011046841, Unigene54077\_Sample\_011046841, Unigene58904\_Sample\_011046841, Unigene50559\_Sample\_011046841, Unigene59156\_Sample\_011046841, Unigene44366\_Sample\_011046841, Unigene9141\_Sample\_011046841, Unigene25248\_Sample\_011046841, Unigene21527\_Sample\_011046841, Unigene60219\_Sample\_011046841, Unigene55247\_Sample\_011046841, Unigene49653\_Sample\_011046841, Unigene29081\_Sample\_011046841, Unigene51904\_Sample\_011046841, Unigene52883\_Sample\_011046841, Unigene33620\_Sample\_011046841, Unigene56886\_Sample\_011046841, Unigene26728\_Sample\_011046841, Unigene23044\_Sample\_011046841, Unigene30814\_Sample\_011046841, Unigene25210\_Sample\_011046841, Unigene42508\_Sample\_011046841, Unigene47860\_Sample\_011046841, Unigene46989\_Sample\_011046841, Unigene35537\_Sample\_011046841, Unigene54823\_Sample\_011046841, Unigene53095\_Sample\_011046841, Unigene9684\_Sample\_011046841, Unigene38803\_Sample\_011046841, Unigene29602\_Sample\_011046841, Unigene47198\_Sample\_011046841, Unigene50293\_Sample\_011046841, Unigene59660\_Sample\_011046841, Unigene29124\_Sample\_011046841, Unigene57613\_Sample\_011046841, Unigene42503\_Sample\_011046841, Unigene60075\_Sample\_011046841, Unigene44005\_Sample\_011046841, Unigene4630\_Sample\_011046841, Unigene44183\_Sample\_011046841, Unigene34268\_Sample\_011046841, Unigene58291\_Sample\_011046841, Unigene50983\_Sample\_011046841, Unigene8488\_Sample\_011046841, Unigene13929\_Sample\_011046841, Unigene55892\_Sample\_011046841, Unigene52779\_Sample\_011046841, Unigene13584\_Sample\_011046841, Unigene11219\_Sample\_011046841, Unigene56418\_Sample\_011046841, Unigene57699\_Sample\_011046841, Unigene52085\_Sample\_011046841, Unigene28388\_Sample\_011046841, Unigene56395\_Sample\_011046841, Unigene59421\_Sample\_011046841, Unigene37790\_Sample\_011046841, Unigene7261\_Sample\_011046841, Unigene60404\_Sample\_011046841, Unigene15846\_Sample\_011046841, Unigene12023\_Sample\_011046841, Unigene3975\_Sample\_011046841, Unigene30697\_Sample\_011046841, Unigene43044\_Sample\_011046841, Unigene10863\_Sample\_011046841, Unigene4209\_Sample\_011046841, Unigene43474\_Sample\_011046841, Unigene10683\_Sample\_011046841, Unigene48571\_Sample\_011046841, Unigene5909\_Sample\_011046841, Unigene9537\_Sample\_011046841, Unigene22548\_Sample\_011046841, Unigene32173\_Sample\_011046841, Unigene32247\_Sample\_011046841, Unigene60190\_Sample\_011046841, Unigene57641\_Sample\_011046841, Unigene50448\_Sample\_011046841, Unigene57643\_Sample\_011046841, Unigene39978\_Sample\_011046841, Unigene54721\_Sample\_011046841, Unigene13102\_Sample\_011046841, Unigene39454\_Sample\_011046841, Unigene2038\_Sample\_011046841, Unigene38271\_Sample\_011046841, Unigene2846\_Sample\_011046841, Unigene54656\_Sample\_011046841, Unigene51219\_Sample\_011046841, Unigene39295\_Sample\_011046841, Unigene58529\_Sample\_011046841, Unigene25353\_Sample\_011046841, Unigene36772\_Sample\_011046841, Unigene47299\_Sample\_011046841, Unigene32174\_Sample\_011046841, Unigene51471\_Sample\_011046841, Unigene55667\_Sample\_011046841, Unigene52610\_Sample\_011046841, Unigene31466\_Sample\_011046841, Unigene26685\_Sample\_011046841, Unigene37087\_Sample\_011046841, Unigene57825\_Sample\_011046841, Unigene7044\_Sample\_011046841, Unigene48672\_Sample\_011046841, Unigene11911\_Sample\_011046841, Unigene26120\_Sample\_011046841, Unigene52869\_Sample\_011046841, Unigene49125\_Sample\_011046841, Unigene21216\_Sample\_011046841, Unigene31490\_Sample\_011046841, Unigene6535\_Sample\_011046841, Unigene9879\_Sample\_011046841, Unigene22843\_Sample\_011046841, Unigene5318\_Sample\_011046841, Unigene17367\_Sample\_011046841, Unigene50720\_Sample\_011046841, Unigene56016\_Sample\_011046841, Unigene53226\_Sample\_011046841, Unigene59448\_Sample\_011046841, Unigene58587\_Sample\_011046841, Unigene19352\_Sample\_011046841, Unigene51604\_Sample\_011046841, Unigene13187\_Sample\_011046841, Unigene13214\_Sample\_011046841, Unigene8037\_Sample\_011046841, Unigene5937\_Sample\_011046841, Unigene55251\_Sample\_011046841, Unigene59214\_Sample\_011046841, Unigene39341\_Sample\_011046841, Unigene53979\_Sample\_011046841, Unigene4616\_Sample\_011046841, Unigene21528\_Sample\_011046841, Unigene26192\_Sample\_011046841, Unigene679\_Sample\_011046841, Unigene10720\_Sample\_011046841, Unigene18027\_Sample\_011046841, Unigene7142\_Sample\_011046841, Unigene53425\_Sample\_011046841, Unigene47684\_Sample\_011046841, Unigene5866\_Sample\_011046841, Unigene14181\_Sample\_011046841, Unigene27612\_Sample\_011046841, Unigene38019\_Sample\_011046841, Unigene58173\_Sample\_011046841, Unigene59112\_Sample\_011046841, Unigene51201\_Sample\_011046841, Unigene23117\_Sample\_011046841, Unigene60009\_Sample\_011046841, Unigene33907\_Sample\_011046841, Unigene9711\_Sample\_011046841, Unigene28718\_Sample\_011046841, Unigene47428\_Sample\_011046841, Unigene25307\_Sample\_011046841, Unigene12940\_Sample\_011046841, Unigene27565\_Sample\_011046841, Unigene19439\_Sample\_011046841, Unigene58936\_Sample\_011046841, Unigene41863\_Sample\_011046841, Unigene53257\_Sample\_011046841, Unigene52071\_Sample\_011046841, Unigene60116\_Sample\_011046841, Unigene48927\_Sample\_011046841, Unigene2371\_Sample\_011046841, Unigene57085\_Sample\_011046841, Unigene56711\_Sample\_011046841, Unigene12370\_Sample\_011046841, Unigene17752\_Sample\_011046841, Unigene9776\_Sample\_011046841, Unigene39805\_Sample\_011046841, Unigene53768\_Sample\_011046841, Unigene56599\_Sample\_011046841, Unigene21909\_Sample\_011046841, Unigene60115\_Sample\_011046841, Unigene37491\_Sample\_011046841, Unigene39689\_Sample\_011046841, Unigene51148\_Sample\_011046841, Unigene42437\_Sample\_011046841, Unigene10918\_Sample\_011046841, Unigene33337\_Sample\_011046841, Unigene49570\_Sample\_011046841, Unigene47837\_Sample\_011046841, Unigene7404\_Sample\_011046841, Unigene18638\_Sample\_011046841, Unigene25759\_Sample\_011046841, Unigene45801\_Sample\_011046841, Unigene26015\_Sample\_011046841, Unigene13964\_Sample\_011046841, Unigene28275\_Sample\_011046841, Unigene30723\_Sample\_011046841, Unigene40571\_Sample\_011046841, Unigene49772\_Sample\_011046841, Unigene51928\_Sample\_011046841, Unigene53302\_Sample\_011046841, Unigene46011\_Sample\_011046841, Unigene6450\_Sample\_011046841, Unigene58523\_Sample\_011046841, Unigene12061\_Sample\_011046841, Unigene29318\_Sample\_011046841, Unigene50014\_Sample\_011046841, Unigene41388\_Sample\_011046841, Unigene32914\_Sample\_011046841, Unigene8047\_Sample\_011046841, Unigene30077\_Sample\_011046841, Unigene11921\_Sample\_011046841, Unigene8943\_Sample\_011046841, Unigene60318\_Sample\_011046841, Unigene17067\_Sample\_011046841, Unigene37943\_Sample\_011046841, Unigene12447\_Sample\_011046841, Unigene36941\_Sample\_011046841, Unigene53368\_Sample\_011046841, Unigene6709\_Sample\_011046841, Unigene19040\_Sample\_011046841, Unigene18596\_Sample\_011046841, Unigene26922\_Sample\_011046841, Unigene14854\_Sample\_011046841, Unigene28546\_Sample\_011046841, Unigene38701\_Sample\_011046841, Unigene14576\_Sample\_011046841, Unigene1989\_Sample\_011046841, Unigene59880\_Sample\_011046841, Unigene52354\_Sample\_011046841, Unigene38750\_Sample\_011046841, Unigene54528\_Sample\_011046841, Unigene30530\_Sample\_011046841, Unigene3320\_Sample\_011046841, Unigene8034\_Sample\_011046841, Unigene51174\_Sample\_011046841, Unigene18963\_Sample\_011046841, Unigene56956\_Sample\_011046841, Unigene42885\_Sample\_011046841, Unigene56863\_Sample\_011046841, Unigene2518\_Sample\_011046841, Unigene59922\_Sample\_011046841, Unigene56191\_Sample\_011046841, Unigene20191\_Sample\_011046841, Unigene48297\_Sample\_011046841, Unigene58043\_Sample\_011046841, Unigene6828\_Sample\_011046841, Unigene40423\_Sample\_011046841, Unigene7206\_Sample\_011046841, Unigene21213\_Sample\_011046841, Unigene42225\_Sample\_011046841, Unigene6497\_Sample\_011046841, Unigene9238\_Sample\_011046841, Unigene28983\_Sample\_011046841, Unigene32619\_Sample\_011046841, Unigene39787\_Sample\_011046841, Unigene8312\_Sample\_011046841, Unigene43173\_Sample\_011046841, Unigene51382\_Sample\_011046841, Unigene52698\_Sample\_011046841, Unigene42712\_Sample\_011046841, Unigene26858\_Sample\_011046841, Unigene55378\_Sample\_011046841, Unigene57566\_Sample\_011046841, Unigene58431\_Sample\_011046841, Unigene12591\_Sample\_011046841, Unigene28361\_Sample\_011046841, Unigene48836\_Sample\_011046841, Unigene29977\_Sample\_011046841, Unigene51125\_Sample\_011046841, Unigene60095\_Sample\_011046841, Unigene10077\_Sample\_011046841, Unigene48473\_Sample\_011046841, Unigene10722\_Sample\_011046841, Unigene12515\_Sample\_011046841, Unigene60757\_Sample\_011046841, Unigene34400\_Sample\_011046841, Unigene34247\_Sample\_011046841, Unigene43294\_Sample\_011046841, Unigene57517\_Sample\_011046841, Unigene16836\_Sample\_011046841, Unigene50207\_Sample\_011046841, Unigene6719\_Sample\_011046841, Unigene7219\_Sample\_011046841, Unigene20785\_Sample\_011046841, Unigene50141\_Sample\_011046841, Unigene30939\_Sample\_011046841, Unigene23431\_Sample\_011046841, Unigene18609\_Sample\_011046841, Unigene55900\_Sample\_011046841, Unigene47959\_Sample\_011046841, Unigene55700\_Sample\_011046841, Unigene43733\_Sample\_011046841, Unigene39953\_Sample\_011046841, Unigene58150\_Sample\_011046841, Unigene31905\_Sample\_011046841, Unigene20614\_Sample\_011046841, Unigene31592\_Sample\_011046841, Unigene50708\_Sample\_011046841, Unigene59375\_Sample\_011046841, Unigene5967\_Sample\_011046841, Unigene59684\_Sample\_011046841, Unigene46198\_Sample\_011046841, Unigene40609\_Sample\_011046841, Unigene5054\_Sample\_011046841, Unigene58911\_Sample\_011046841, Unigene29813\_Sample\_011046841, Unigene53846\_Sample\_011046841, Unigene44852\_Sample\_011046841, Unigene47811\_Sample\_011046841, Unigene35586\_Sample\_011046841, Unigene40633\_Sample\_011046841, Unigene8229\_Sample\_011046841, Unigene40953\_Sample\_011046841, Unigene41811\_Sample\_011046841, Unigene18715\_Sample\_011046841, Unigene51237\_Sample\_011046841, Unigene8931\_Sample\_011046841, Unigene58180\_Sample\_011046841, Unigene21504\_Sample\_011046841, Unigene18404\_Sample\_011046841, Unigene41284\_Sample\_011046841, Unigene26183\_Sample\_011046841, Unigene51437\_Sample\_011046841, Unigene6435\_Sample\_011046841, Unigene55799\_Sample\_011046841, Unigene11923\_Sample\_011046841, Unigene59946\_Sample\_011046841, Unigene49914\_Sample\_011046841, Unigene48742\_Sample\_011046841, Unigene6502\_Sample\_011046841, Unigene54466\_Sample\_011046841, Unigene42218\_Sample\_011046841, Unigene54217\_Sample\_011046841, Unigene55342\_Sample\_011046841, Unigene58511\_Sample\_011046841, Unigene51091\_Sample\_011046841, Unigene56559\_Sample\_011046841, Unigene55379\_Sample\_011046841, Unigene27378\_Sample\_011046841, Unigene43091\_Sample\_011046841, Unigene2834\_Sample\_011046841, Unigene18780\_Sample\_011046841, Unigene9480\_Sample\_011046841, Unigene24811\_Sample\_011046841, Unigene2110\_Sample\_011046841, Unigene22682\_Sample\_011046841, Unigene19912\_Sample\_011046841, Unigene55833\_Sample\_011046841, Unigene40289\_Sample\_011046841, Unigene40346\_Sample\_011046841, Unigene53011\_Sample\_011046841, Unigene13634\_Sample\_011046841, Unigene19375\_Sample\_011046841, Unigene25239\_Sample\_011046841, Unigene55221\_Sample\_011046841, Unigene42259\_Sample\_011046841, Unigene42751\_Sample\_011046841, Unigene35134\_Sample\_011046841, Unigene58571\_Sample\_011046841, Unigene49359\_Sample\_011046841, Unigene55954\_Sample\_011046841, Unigene58763\_Sample\_011046841, Unigene47471\_Sample\_011046841, Unigene59633\_Sample\_011046841, Unigene38993\_Sample\_011046841, Unigene31696\_Sample\_011046841, Unigene26425\_Sample\_011046841, Unigene55254\_Sample\_011046841, Unigene12637\_Sample\_011046841, Unigene45808\_Sample\_011046841, Unigene59734\_Sample\_011046841, Unigene13688\_Sample\_011046841, Unigene11715\_Sample\_011046841, Unigene58118\_Sample\_011046841, Unigene47162\_Sample\_011046841, Unigene58827\_Sample\_011046841, Unigene54396\_Sample\_011046841, Unigene59690\_Sample\_011046841, Unigene1076\_Sample\_011046841, Unigene38913\_Sample\_011046841, Unigene52525\_Sample\_011046841, Unigene13879\_Sample\_011046841, Unigene28944\_Sample\_011046841, Unigene51006\_Sample\_011046841, Unigene53186\_Sample\_011046841, Unigene26892\_Sample\_011046841, Unigene52882\_Sample\_011046841, Unigene48380\_Sample\_011046841, Unigene55675\_Sample\_011046841, Unigene36373\_Sample\_011046841, Unigene8987\_Sample\_011046841, Unigene40130\_Sample\_011046841, Unigene36851\_Sample\_011046841, Unigene40692\_Sample\_011046841, Unigene51334\_Sample\_011046841, Unigene31416\_Sample\_011046841, Unigene39807\_Sample\_011046841, Unigene32413\_Sample\_011046841, Unigene13256\_Sample\_011046841, Unigene983\_Sample\_011046841, Unigene58113\_Sample\_011046841, Unigene13419\_Sample\_011046841, Unigene55889\_Sample\_011046841, Unigene31679\_Sample\_011046841, Unigene21818\_Sample\_011046841, Unigene49494\_Sample\_011046841, Unigene30184\_Sample\_011046841, Unigene32883\_Sample\_011046841, Unigene56587\_Sample\_011046841, Unigene25063\_Sample\_011046841, Unigene50797\_Sample\_011046841, Unigene54165\_Sample\_011046841, Unigene21535\_Sample\_011046841, Unigene45635\_Sample\_011046841, Unigene50712\_Sample\_011046841, Unigene51178\_Sample\_011046841, Unigene49927\_Sample\_011046841, Unigene57118\_Sample\_011046841, Unigene41859\_Sample\_011046841, Unigene47562\_Sample\_011046841, Unigene55865\_Sample\_011046841, Unigene58519\_Sample\_011046841, Unigene56468\_Sample\_011046841, Unigene2398\_Sample\_011046841, Unigene59828\_Sample\_011046841, Unigene5366\_Sample\_011046841, Unigene47082\_Sample\_011046841, Unigene43660\_Sample\_011046841, Unigene50009\_Sample\_011046841, Unigene51930\_Sample\_011046841, Unigene52932\_Sample\_011046841, Unigene59080\_Sample\_011046841, Unigene30073\_Sample\_011046841, Unigene10632\_Sample\_011046841, Unigene29330\_Sample\_011046841, Unigene12145\_Sample\_011046841, Unigene45996\_Sample\_011046841, Unigene33542\_Sample\_011046841, Unigene33587\_Sample\_011046841, Unigene49258\_Sample\_011046841, Unigene52488\_Sample\_011046841, Unigene36585\_Sample\_011046841, Unigene47868\_Sample\_011046841, Unigene59333\_Sample\_011046841, Unigene52077\_Sample\_011046841, Unigene56914\_Sample\_011046841, Unigene19943\_Sample\_011046841, Unigene57904\_Sample\_011046841, Unigene53668\_Sample\_011046841, Unigene9390\_Sample\_011046841, Unigene19043\_Sample\_011046841, Unigene13011\_Sample\_011046841, Unigene48930\_Sample\_011046841, Unigene50713\_Sample\_011046841, Unigene51627\_Sample\_011046841, Unigene11020\_Sample\_011046841, Unigene54937\_Sample\_011046841, Unigene22650\_Sample\_011046841, Unigene53534\_Sample\_011046841, Unigene3247\_Sample\_011046841, Unigene47540\_Sample\_011046841, Unigene7693\_Sample\_011046841, Unigene1417\_Sample\_011046841, Unigene32028\_Sample\_011046841, Unigene12869\_Sample\_011046841, Unigene60520\_Sample\_011046841, Unigene54962\_Sample\_011046841, Unigene16650\_Sample\_011046841, Unigene53431\_Sample\_011046841, Unigene38335\_Sample\_011046841, Unigene42605\_Sample\_011046841, Unigene6178\_Sample\_011046841, Unigene29309\_Sample\_011046841, Unigene24692\_Sample\_011046841, Unigene53121\_Sample\_011046841, Unigene52322\_Sample\_011046841, Unigene13080\_Sample\_011046841, Unigene33509\_Sample\_011046841, Unigene11728\_Sample\_011046841, Unigene20937\_Sample\_011046841, Unigene44373\_Sample\_011046841, Unigene57809\_Sample\_011046841, Unigene54191\_Sample\_011046841, Unigene59863\_Sample\_011046841, Unigene40390\_Sample\_011046841, Unigene59118\_Sample\_011046841, Unigene54640\_Sample\_011046841, Unigene43713\_Sample\_011046841, Unigene59512\_Sample\_011046841, Unigene60253\_Sample\_011046841, Unigene53916\_Sample\_011046841, Unigene41992\_Sample\_011046841, Unigene1441\_Sample\_011046841, Unigene29966\_Sample\_011046841, Unigene58403\_Sample\_011046841, Unigene23671\_Sample\_011046841, Unigene60753\_Sample\_011046841, Unigene11922\_Sample\_011046841, Unigene56177\_Sample\_011046841, Unigene5031\_Sample\_011046841, Unigene55312\_Sample\_011046841, Unigene34292\_Sample\_011046841, Unigene60783\_Sample\_011046841, Unigene15593\_Sample\_011046841, Unigene59074\_Sample\_011046841, Unigene8990\_Sample\_011046841, Unigene48062\_Sample\_011046841, Unigene56198\_Sample\_011046841, Unigene50163\_Sample\_011046841, Unigene42323\_Sample\_011046841, Unigene56105\_Sample\_011046841, Unigene29942\_Sample\_011046841, Unigene41393\_Sample\_011046841, Unigene23154\_Sample\_011046841, Unigene41250\_Sample\_011046841, Unigene50996\_Sample\_011046841, Unigene55496\_Sample\_011046841, Unigene59323\_Sample\_011046841, Unigene23611\_Sample\_011046841, Unigene56835\_Sample\_011046841, Unigene38505\_Sample\_011046841, Unigene60659\_Sample\_011046841, Unigene20487\_Sample\_011046841, Unigene53915\_Sample\_011046841, Unigene11180\_Sample\_011046841, Unigene36078\_Sample\_011046841, Unigene651\_Sample\_011046841, Unigene57086\_Sample\_011046841, Unigene23924\_Sample\_011046841, Unigene36252\_Sample\_011046841, Unigene59572\_Sample\_011046841, Unigene13142\_Sample\_011046841, Unigene54607\_Sample\_011046841, Unigene18741\_Sample\_011046841, Unigene59519\_Sample\_011046841, Unigene13893\_Sample\_011046841, Unigene60309\_Sample\_011046841, Unigene42912\_Sample\_011046841, Unigene58166\_Sample\_011046841, Unigene59779\_Sample\_011046841, Unigene21655\_Sample\_011046841, Unigene53173\_Sample\_011046841, Unigene56645\_Sample\_011046841, Unigene38212\_Sample\_011046841, Unigene52165\_Sample\_011046841, Unigene10917\_Sample\_011046841, Unigene37595\_Sample\_011046841, Unigene60381\_Sample\_011046841, Unigene44861\_Sample\_011046841, Unigene10284\_Sample\_011046841, Unigene29271\_Sample\_011046841, Unigene54949\_Sample\_011046841, Unigene59530\_Sample\_011046841, Unigene43972\_Sample\_011046841, Unigene6300\_Sample\_011046841, Unigene13407\_Sample\_011046841, Unigene32077\_Sample\_011046841, Unigene59535\_Sample\_011046841, Unigene24726\_Sample\_011046841, Unigene39630\_Sample\_011046841, Unigene13431\_Sample\_011046841, Unigene57944\_Sample\_011046841, Unigene20486\_Sample\_011046841, Unigene54293\_Sample\_011046841, Unigene30672\_Sample\_011046841, Unigene57933\_Sample\_011046841, Unigene47210\_Sample\_011046841, Unigene2441\_Sample\_011046841, Unigene55393\_Sample\_011046841, Unigene35850\_Sample\_011046841, Unigene57928\_Sample\_011046841, Unigene12213\_Sample\_011046841, Unigene57838\_Sample\_011046841, Unigene3139\_Sample\_011046841, Unigene41676\_Sample\_011046841, Unigene59859\_Sample\_011046841, Unigene9668\_Sample\_011046841, Unigene55420\_Sample\_011046841, Unigene25573\_Sample\_011046841, Unigene13695\_Sample\_011046841, Unigene54402\_Sample\_011046841, Unigene46813\_Sample\_011046841, Unigene22741\_Sample\_011046841, Unigene42450\_Sample\_011046841, Unigene28781\_Sample\_011046841, Unigene49407\_Sample\_011046841, Unigene12441\_Sample\_011046841, Unigene39361\_Sample\_011046841, Unigene48091\_Sample\_011046841, Unigene39096\_Sample\_011046841, Unigene60743\_Sample\_011046841, Unigene2821\_Sample\_011046841, Unigene11990\_Sample\_011046841, Unigene60102\_Sample\_011046841, Unigene47329\_Sample\_011046841, Unigene55618\_Sample\_011046841, Unigene16257\_Sample\_011046841, Unigene47839\_Sample\_011046841, Unigene55196\_Sample\_011046841, Unigene44220\_Sample\_011046841, Unigene59913\_Sample\_011046841, Unigene29847\_Sample\_011046841, Unigene15802\_Sample\_011046841, Unigene59936\_Sample\_011046841, Unigene31866\_Sample\_011046841, Unigene48086\_Sample\_011046841, Unigene33618\_Sample\_011046841, Unigene44734\_Sample\_011046841, Unigene20619\_Sample\_011046841, Unigene29551\_Sample\_011046841, Unigene30349\_Sample\_011046841, Unigene10215\_Sample\_011046841, Unigene22216\_Sample\_011046841, Unigene45641\_Sample\_011046841, Unigene250\_Sample\_011046841, Unigene40544\_Sample\_011046841, Unigene59962\_Sample\_011046841, Unigene41694\_Sample\_011046841, Unigene20157\_Sample\_011046841, Unigene11939\_Sample\_011046841, Unigene46170\_Sample\_011046841, Unigene37456\_Sample\_011046841, Unigene33624\_Sample\_011046841, Unigene38948\_Sample\_011046841, Unigene10226\_Sample\_011046841, Unigene57806\_Sample\_011046841, Unigene53994\_Sample\_011046841, Unigene50372\_Sample\_011046841, Unigene58593\_Sample\_011046841, Unigene37492\_Sample\_011046841, Unigene14263\_Sample\_011046841, Unigene11846\_Sample\_011046841, Unigene37037\_Sample\_011046841, Unigene59653\_Sample\_011046841, Unigene19677\_Sample\_011046841, Unigene50902\_Sample\_011046841, Unigene52526\_Sample\_011046841, Unigene32699\_Sample\_011046841, Unigene14511\_Sample\_011046841, Unigene52384\_Sample\_011046841, Unigene39772\_Sample\_011046841, Unigene30600\_Sample\_011046841, Unigene29900\_Sample\_011046841, Unigene53856\_Sample\_011046841, Unigene55465\_Sample\_011046841, Unigene8962\_Sample\_011046841, Unigene54692\_Sample\_011046841, Unigene56008\_Sample\_011046841, Unigene34417\_Sample\_011046841, Unigene37642\_Sample\_011046841, Unigene58564\_Sample\_011046841, Unigene51702\_Sample\_011046841, Unigene59978\_Sample\_011046841, Unigene19215\_Sample\_011046841, Unigene38182\_Sample\_011046841, Unigene39125\_Sample\_011046841, Unigene45306\_Sample\_011046841, Unigene55660\_Sample\_011046841, Unigene54535\_Sample\_011046841, Unigene53303\_Sample\_011046841, Unigene30120\_Sample\_011046841, Unigene60378\_Sample\_011046841, Unigene48756\_Sample\_011046841, Unigene55544\_Sample\_011046841, Unigene1176\_Sample\_011046841, Unigene6605\_Sample\_011046841, Unigene54791\_Sample\_011046841, Unigene60665\_Sample\_011046841, Unigene22917\_Sample\_011046841, Unigene60711\_Sample\_011046841, Unigene43139\_Sample\_011046841, Unigene57962\_Sample\_011046841, Unigene50729\_Sample\_011046841, Unigene60848\_Sample\_011046841, Unigene58192\_Sample\_011046841, Unigene16565\_Sample\_011046841, Unigene33573\_Sample\_011046841, Unigene55533\_Sample\_011046841, Unigene8746\_Sample\_011046841, Unigene44315\_Sample\_011046841, Unigene49021\_Sample\_011046841, Unigene51545\_Sample\_011046841, Unigene58488\_Sample\_011046841, Unigene23971\_Sample\_011046841, Unigene23972\_Sample\_011046841, Unigene33302\_Sample\_011046841, Unigene54240\_Sample\_011046841, Unigene4131\_Sample\_011046841, Unigene8451\_Sample\_011046841, Unigene8747\_Sample\_011046841, Unigene32425\_Sample\_011046841, Unigene46584\_Sample\_011046841, Unigene2942\_Sample\_011046841, Unigene58107\_Sample\_011046841, Unigene59303\_Sample\_011046841, Unigene46220\_Sample\_011046841, Unigene51688\_Sample\_011046841, Unigene40435\_Sample\_011046841, Unigene51950\_Sample\_011046841, Unigene43874\_Sample\_011046841, Unigene55325\_Sample\_011046841, Unigene29733\_Sample\_011046841, Unigene14996\_Sample\_011046841, Unigene41541\_Sample\_011046841, Unigene16692\_Sample\_011046841, Unigene50915\_Sample\_011046841, Unigene39075\_Sample\_011046841, Unigene54373\_Sample\_011046841, Unigene56466\_Sample\_011046841, Unigene55474\_Sample\_011046841, Unigene27309\_Sample\_011046841, Unigene38907\_Sample\_011046841, Unigene8798\_Sample\_011046841, Unigene6432\_Sample\_011046841, Unigene59476\_Sample\_011046841, Unigene40742\_Sample\_011046841, Unigene42293\_Sample\_011046841, Unigene19453\_Sample\_011046841, Unigene54670\_Sample\_011046841, Unigene45203\_Sample\_011046841, Unigene53061\_Sample\_011046841, Unigene56709\_Sample\_011046841, Unigene46843\_Sample\_011046841, Unigene35438\_Sample\_011046841, Unigene51937\_Sample\_011046841, Unigene13945\_Sample\_011046841, Unigene47292\_Sample\_011046841, Unigene51067\_Sample\_011046841, Unigene59926\_Sample\_011046841, Unigene44834\_Sample\_011046841, Unigene57861\_Sample\_011046841, Unigene9872\_Sample\_011046841, Unigene60093\_Sample\_011046841, Unigene45374\_Sample\_011046841, Unigene26878\_Sample\_011046841, Unigene2937\_Sample\_011046841, Unigene22433\_Sample\_011046841, Unigene60844\_Sample\_011046841, Unigene18925\_Sample\_011046841, Unigene42681\_Sample\_011046841, Unigene16136\_Sample\_011046841, Unigene41032\_Sample\_011046841, Unigene57235\_Sample\_011046841, Unigene13332\_Sample\_011046841, Unigene32401\_Sample\_011046841, Unigene45020\_Sample\_011046841, Unigene29319\_Sample\_011046841, Unigene38398\_Sample\_011046841, Unigene9579\_Sample\_011046841, Unigene46254\_Sample\_011046841, Unigene48425\_Sample\_011046841, Unigene47309\_Sample\_011046841, Unigene33405\_Sample\_011046841, Unigene40851\_Sample\_011046841, Unigene28626\_Sample\_011046841, Unigene23416\_Sample\_011046841, Unigene42399\_Sample\_011046841, Unigene58804\_Sample\_011046841, Unigene58516\_Sample\_011046841, Unigene28245\_Sample\_011046841, Unigene11735\_Sample\_011046841, Unigene2785\_Sample\_011046841, Unigene5462\_Sample\_011046841, Unigene57570\_Sample\_011046841, Unigene19793\_Sample\_011046841, Unigene7905\_Sample\_011046841, Unigene21362\_Sample\_011046841, Unigene48867\_Sample\_011046841, Unigene12206\_Sample\_011046841, Unigene11329\_Sample\_011046841, Unigene57752\_Sample\_011046841, Unigene17290\_Sample\_011046841, Unigene57669\_Sample\_011046841, Unigene58974\_Sample\_011046841, Unigene6327\_Sample\_011046841, Unigene59354\_Sample\_011046841, Unigene42357\_Sample\_011046841, Unigene12149\_Sample\_011046841, Unigene7335\_Sample\_011046841, Unigene14170\_Sample\_011046841, Unigene54218\_Sample\_011046841, Unigene30495\_Sample\_011046841, Unigene47097\_Sample\_011046841, Unigene59396\_Sample\_011046841, Unigene24600\_Sample\_011046841, Unigene21962\_Sample\_011046841, Unigene49921\_Sample\_011046841, Unigene59579\_Sample\_011046841, Unigene15108\_Sample\_011046841, Unigene23477\_Sample\_011046841, Unigene50012\_Sample\_011046841, Unigene48689\_Sample\_011046841, Unigene13667\_Sample\_011046841, Unigene22611\_Sample\_011046841, Unigene53073\_Sample\_011046841, Unigene42576\_Sample\_011046841, Unigene141\_Sample\_011046841, Unigene10011\_Sample\_011046841, Unigene56010\_Sample\_011046841, Unigene948\_Sample\_011046841, Unigene54742\_Sample\_011046841, Unigene48530\_Sample\_011046841, Unigene35636\_Sample\_011046841, Unigene17568\_Sample\_011046841, Unigene53241\_Sample\_011046841, Unigene44944\_Sample\_011046841, Unigene38302\_Sample\_011046841, Unigene25755\_Sample\_011046841, Unigene57130\_Sample\_011046841, Unigene60375\_Sample\_011046841, Unigene17289\_Sample\_011046841, Unigene10438\_Sample\_011046841, Unigene13433\_Sample\_011046841, Unigene44237\_Sample\_011046841, Unigene38689\_Sample\_011046841, Unigene23519\_Sample\_011046841, Unigene60914\_Sample\_011046841, Unigene1284\_Sample\_011046841, Unigene57183\_Sample\_011046841, Unigene54187\_Sample\_011046841, Unigene11526\_Sample\_011046841, Unigene47101\_Sample\_011046841, Unigene36472\_Sample\_011046841, Unigene59929\_Sample\_011046841, Unigene55122\_Sample\_011046841, Unigene23201\_Sample\_011046841, Unigene12280\_Sample\_011046841, Unigene17825\_Sample\_011046841, Unigene45310\_Sample\_011046841, Unigene9466\_Sample\_011046841, Unigene9772\_Sample\_011046841, Unigene16198\_Sample\_011046841, Unigene50108\_Sample\_011046841, Unigene38130\_Sample\_011046841, Unigene57713\_Sample\_011046841, Unigene59114\_Sample\_011046841, Unigene60342\_Sample\_011046841, Unigene59226\_Sample\_011046841, Unigene16301\_Sample\_011046841, Unigene30884\_Sample\_011046841, Unigene10426\_Sample\_011046841, Unigene50099\_Sample\_011046841, Unigene45033\_Sample\_011046841, Unigene3716\_Sample\_011046841, Unigene15341\_Sample\_011046841, Unigene8146\_Sample\_011046841, Unigene37889\_Sample\_011046841, Unigene59238\_Sample\_011046841, Unigene30153\_Sample\_011046841, Unigene18042\_Sample\_011046841, Unigene39867\_Sample\_011046841, Unigene51585\_Sample\_011046841, Unigene53034\_Sample\_011046841, Unigene5657\_Sample\_011046841, Unigene51697\_Sample\_011046841, Unigene9726\_Sample\_011046841, Unigene8954\_Sample\_011046841, Unigene30484\_Sample\_011046841, Unigene47187\_Sample\_011046841, Unigene5118\_Sample\_011046841, Unigene53972\_Sample\_011046841, Unigene56254\_Sample\_011046841, Unigene43147\_Sample\_011046841, Unigene39939\_Sample\_011046841, Unigene34858\_Sample\_011046841, Unigene49113\_Sample\_011046841, Unigene46974\_Sample\_011046841, Unigene53689\_Sample\_011046841, Unigene51171\_Sample\_011046841, Unigene6531\_Sample\_011046841, Unigene46026\_Sample\_011046841, Unigene11858\_Sample\_011046841, Unigene19343\_Sample\_011046841, Unigene10024\_Sample\_011046841, Unigene58899\_Sample\_011046841, Unigene34891\_Sample\_011046841, Unigene57735\_Sample\_011046841, Unigene18832\_Sample\_011046841, Unigene31782\_Sample\_011046841, Unigene57293\_Sample\_011046841, Unigene30044\_Sample\_011046841, Unigene14638\_Sample\_011046841, Unigene10184\_Sample\_011046841, Unigene56037\_Sample\_011046841, Unigene31304\_Sample\_011046841, Unigene29758\_Sample\_011046841, Unigene17057\_Sample\_011046841, Unigene9001\_Sample\_011046841, Unigene54955\_Sample\_011046841, Unigene13270\_Sample\_011046841, Unigene55948\_Sample\_011046841, Unigene54866\_Sample\_011046841, Unigene60478\_Sample\_011046841, Unigene2363\_Sample\_011046841, Unigene33512\_Sample\_011046841, Unigene54473\_Sample\_011046841, Unigene32854\_Sample\_011046841, Unigene49724\_Sample\_011046841, Unigene23393\_Sample\_011046841, Unigene29201\_Sample\_011046841, Unigene60825\_Sample\_011046841, Unigene60294\_Sample\_011046841, Unigene43411\_Sample\_011046841, Unigene41682\_Sample\_011046841, Unigene58367\_Sample\_011046841, Unigene28384\_Sample\_011046841, Unigene50185\_Sample\_011046841, Unigene35898\_Sample\_011046841, Unigene55315\_Sample\_011046841, Unigene12218\_Sample\_011046841, Unigene20459\_Sample\_011046841, Unigene48999\_Sample\_011046841, Unigene1886\_Sample\_011046841, Unigene12983\_Sample\_011046841, Unigene26087\_Sample\_011046841, Unigene55029\_Sample\_011046841, Unigene9600\_Sample\_011046841, Unigene11695\_Sample\_011046841, Unigene53520\_Sample\_011046841, Unigene45818\_Sample\_011046841, Unigene32993\_Sample\_011046841, Unigene51664\_Sample\_011046841, Unigene11099\_Sample\_011046841, Unigene10980\_Sample\_011046841, Unigene42643\_Sample\_011046841, Unigene8297\_Sample\_011046841, Unigene4210\_Sample\_011046841, Unigene53929\_Sample\_011046841, Unigene19079\_Sample\_011046841, Unigene31241\_Sample\_011046841, Unigene57433\_Sample\_011046841, Unigene48528\_Sample\_011046841, Unigene6977\_Sample\_011046841, Unigene18613\_Sample\_011046841, Unigene38769\_Sample\_011046841, Unigene10037\_Sample\_011046841, Unigene21246\_Sample\_011046841, Unigene54648\_Sample\_011046841, Unigene58114\_Sample\_011046841, Unigene6317\_Sample\_011046841, Unigene47158\_Sample\_011046841, Unigene59711\_Sample\_011046841, Unigene10623\_Sample\_011046841, Unigene10080\_Sample\_011046841, Unigene17084\_Sample\_011046841, Unigene37448\_Sample\_011046841, Unigene56982\_Sample\_011046841, Unigene58767\_Sample\_011046841, Unigene18599\_Sample\_011046841, Unigene24979\_Sample\_011046841, Unigene51137\_Sample\_011046841, Unigene47755\_Sample\_011046841, Unigene12578\_Sample\_011046841, Unigene49353\_Sample\_011046841, Unigene58552\_Sample\_011046841, Unigene55486\_Sample\_011046841, Unigene28066\_Sample\_011046841, Unigene43935\_Sample\_011046841, Unigene59961\_Sample\_011046841, Unigene29320\_Sample\_011046841, Unigene6253\_Sample\_011046841, Unigene12446\_Sample\_011046841, Unigene60218\_Sample\_011046841, Unigene41828\_Sample\_011046841, Unigene5939\_Sample\_011046841, Unigene37683\_Sample\_011046841, Unigene12338\_Sample\_011046841, Unigene34283\_Sample\_011046841, Unigene39189\_Sample\_011046841, Unigene60405\_Sample\_011046841, Unigene12195\_Sample\_011046841, Unigene34458\_Sample\_011046841, Unigene13164\_Sample\_011046841, Unigene50919\_Sample\_011046841, Unigene2174\_Sample\_011046841, Unigene53149\_Sample\_011046841, Unigene13251\_Sample\_011046841, Unigene2549\_Sample\_011046841, Unigene17671\_Sample\_011046841, Unigene58818\_Sample\_011046841, Unigene2968\_Sample\_011046841, Unigene47474\_Sample\_011046841, Unigene14590\_Sample\_011046841, Unigene59292\_Sample\_011046841, Unigene40182\_Sample\_011046841, Unigene21921\_Sample\_011046841, Unigene36020\_Sample\_011046841, Unigene33513\_Sample\_011046841, Unigene19767\_Sample\_011046841, Unigene40350\_Sample\_011046841, Unigene49635\_Sample\_011046841, Unigene50487\_Sample\_011046841, Unigene54612\_Sample\_011046841, Unigene46224\_Sample\_011046841, Unigene51209\_Sample\_011046841, Unigene6085\_Sample\_011046841, Unigene713\_Sample\_011046841, Unigene36915\_Sample\_011046841, Unigene38660\_Sample\_011046841, Unigene32563\_Sample\_011046841, Unigene6065\_Sample\_011046841, Unigene12346\_Sample\_011046841, Unigene59910\_Sample\_011046841, Unigene52673\_Sample\_011046841, Unigene7518\_Sample\_011046841, Unigene29723\_Sample\_011046841, Unigene13348\_Sample\_011046841, Unigene11120\_Sample\_011046841, Unigene25340\_Sample\_011046841, Unigene9354\_Sample\_011046841, Unigene29684\_Sample\_011046841, Unigene41395\_Sample\_011046841, Unigene57607\_Sample\_011046841, Unigene24326\_Sample\_011046841, Unigene59972\_Sample\_011046841, Unigene59449\_Sample\_011046841, Unigene60645\_Sample\_011046841, Unigene60174\_Sample\_011046841, Unigene58499\_Sample\_011046841, Unigene37357\_Sample\_011046841, Unigene5058\_Sample\_011046841, Unigene57083\_Sample\_011046841, Unigene56004\_Sample\_011046841, Unigene9149\_Sample\_011046841, Unigene60727\_Sample\_011046841, Unigene51224\_Sample\_011046841, Unigene60313\_Sample\_011046841, Unigene57398\_Sample\_011046841, Unigene44447\_Sample\_011046841, Unigene59381\_Sample\_011046841, Unigene34169\_Sample\_011046841, Unigene12783\_Sample\_011046841, Unigene60566\_Sample\_011046841, Unigene56821\_Sample\_011046841, Unigene57207\_Sample\_011046841, Unigene37225\_Sample\_011046841, Unigene4709\_Sample\_011046841, Unigene3904\_Sample\_011046841, Unigene39537\_Sample\_011046841, Unigene41427\_Sample\_011046841, Unigene26636\_Sample\_011046841, Unigene35744\_Sample\_011046841, Unigene52122\_Sample\_011046841, Unigene57730\_Sample\_011046841, Unigene14236\_Sample\_011046841, Unigene40349\_Sample\_011046841, Unigene12769\_Sample\_011046841, Unigene60657\_Sample\_011046841, Unigene52685\_Sample\_011046841, Unigene51419\_Sample\_011046841, Unigene12309\_Sample\_011046841, Unigene32409\_Sample\_011046841, Unigene56373\_Sample\_011046841, Unigene42917\_Sample\_011046841, Unigene58733\_Sample\_011046841, Unigene16247\_Sample\_011046841, Unigene11008\_Sample\_011046841, Unigene59250\_Sample\_011046841, Unigene11457\_Sample\_011046841, Unigene53463\_Sample\_011046841, Unigene27098\_Sample\_011046841, Unigene47314\_Sample\_011046841, Unigene22649\_Sample\_011046841, Unigene7598\_Sample\_011046841, Unigene50630\_Sample\_011046841, Unigene1819\_Sample\_011046841, Unigene744\_Sample\_011046841, Unigene28196\_Sample\_011046841, Unigene11130\_Sample\_011046841, Unigene15490\_Sample\_011046841, Unigene24171\_Sample\_011046841, Unigene31853\_Sample\_011046841, Unigene53862\_Sample\_011046841, Unigene23155\_Sample\_011046841, Unigene21433\_Sample\_011046841, Unigene56348\_Sample\_011046841, Unigene59629\_Sample\_011046841, Unigene22671\_Sample\_011046841, Unigene53948\_Sample\_011046841, Unigene13855\_Sample\_011046841, Unigene58626\_Sample\_011046841, Unigene20893\_Sample\_011046841, Unigene13748\_Sample\_011046841, Unigene11925\_Sample\_011046841, Unigene36888\_Sample\_011046841, Unigene4891\_Sample\_011046841, Unigene56814\_Sample\_011046841, Unigene36676\_Sample\_011046841, Unigene30554\_Sample\_011046841, Unigene49168\_Sample\_011046841, Unigene13391\_Sample\_011046841, Unigene13220\_Sample\_011046841, Unigene53992\_Sample\_011046841, Unigene18314\_Sample\_011046841, Unigene56825\_Sample\_011046841, Unigene37764\_Sample\_011046841, Unigene5223\_Sample\_011046841, Unigene36212\_Sample\_011046841, Unigene51962\_Sample\_011046841, Unigene54925\_Sample\_011046841, Unigene3481\_Sample\_011046841, Unigene22561\_Sample\_011046841, Unigene32259\_Sample\_011046841, Unigene57435\_Sample\_011046841, Unigene58298\_Sample\_011046841, Unigene13965\_Sample\_011046841, Unigene26465\_Sample\_011046841, Unigene23752\_Sample\_011046841, Unigene14408\_Sample\_011046841, Unigene8794\_Sample\_011046841, Unigene40509\_Sample\_011046841, Unigene13642\_Sample\_011046841, Unigene16847\_Sample\_011046841, Unigene42229\_Sample\_011046841, Unigene48049\_Sample\_011046841, Unigene25506\_Sample\_011046841, Unigene38146\_Sample\_011046841, Unigene20670\_Sample\_011046841, Unigene2588\_Sample\_011046841, Unigene58110\_Sample\_011046841, Unigene38021\_Sample\_011046841, Unigene47448\_Sample\_011046841, Unigene53030\_Sample\_011046841, Unigene45431\_Sample\_011046841, Unigene32309\_Sample\_011046841, Unigene40696\_Sample\_011046841, Unigene44263\_Sample\_011046841, Unigene144\_Sample\_011046841, Unigene18945\_Sample\_011046841, Unigene34466\_Sample\_011046841, Unigene330\_Sample\_011046841, Unigene54248\_Sample\_011046841, Unigene13768\_Sample\_011046841, Unigene31588\_Sample\_011046841, Unigene57981\_Sample\_011046841, Unigene48502\_Sample\_011046841, Unigene59850\_Sample\_011046841, Unigene5112\_Sample\_011046841, Unigene57020\_Sample\_011046841, Unigene4117\_Sample\_011046841, Unigene46363\_Sample\_011046841, Unigene44173\_Sample\_011046841, Unigene22804\_Sample\_011046841, Unigene36927\_Sample\_011046841, Unigene57609\_Sample\_011046841, Unigene24961\_Sample\_011046841, Unigene60016\_Sample\_011046841, Unigene24807\_Sample\_011046841, Unigene58342\_Sample\_011046841, Unigene26116\_Sample\_011046841, Unigene55427\_Sample\_011046841, Unigene58000\_Sample\_011046841, Unigene46879\_Sample\_011046841, Unigene50398\_Sample\_011046841, Unigene7002\_Sample\_011046841, Unigene2534\_Sample\_011046841, Unigene59622\_Sample\_011046841, Unigene26679\_Sample\_011046841, Unigene10441\_Sample\_011046841, Unigene7042\_Sample\_011046841, Unigene53691\_Sample\_011046841, Unigene41336\_Sample\_011046841, Unigene60805\_Sample\_011046841, Unigene4993\_Sample\_011046841, Unigene32666\_Sample\_011046841, Unigene55512\_Sample\_011046841, Unigene42223\_Sample\_011046841, Unigene58374\_Sample\_011046841, Unigene54331\_Sample\_011046841, Unigene50910\_Sample\_011046841, Unigene12249\_Sample\_011046841, Unigene38586\_Sample\_011046841, Unigene48764\_Sample\_011046841, Unigene45737\_Sample\_011046841, Unigene44009\_Sample\_011046841, Unigene60746\_Sample\_011046841, Unigene55180\_Sample\_011046841, Unigene51264\_Sample\_011046841, Unigene56473\_Sample\_011046841, Unigene2578\_Sample\_011046841, Unigene23441\_Sample\_011046841, Unigene42062\_Sample\_011046841, Unigene1958\_Sample\_011046841, Unigene36251\_Sample\_011046841, Unigene57100\_Sample\_011046841, Unigene15616\_Sample\_011046841, Unigene53565\_Sample\_011046841, Unigene54982\_Sample\_011046841, Unigene52144\_Sample\_011046841, Unigene18385\_Sample\_011046841, Unigene22222\_Sample\_011046841, Unigene22791\_Sample\_011046841, Unigene60809\_Sample\_011046841, Unigene58627\_Sample\_011046841, Unigene8612\_Sample\_011046841, Unigene44387\_Sample\_011046841, Unigene12140\_Sample\_011046841, Unigene8165\_Sample\_011046841, Unigene45838\_Sample\_011046841, Unigene60701\_Sample\_011046841, Unigene53738\_Sample\_011046841, Unigene34404\_Sample\_011046841, Unigene47085\_Sample\_011046841, Unigene53741\_Sample\_011046841, Unigene33130\_Sample\_011046841, Unigene18417\_Sample\_011046841, Unigene43552\_Sample\_011046841, Unigene17370\_Sample\_011046841, Unigene7303\_Sample\_011046841, Unigene4871\_Sample\_011046841, Unigene51077\_Sample\_011046841, Unigene43875\_Sample\_011046841, Unigene38251\_Sample\_011046841, Unigene14264\_Sample\_011046841, Unigene45660\_Sample\_011046841, Unigene9658\_Sample\_011046841, Unigene43445\_Sample\_011046841, Unigene60606\_Sample\_011046841, Unigene29850\_Sample\_011046841, Unigene45158\_Sample\_011046841, Unigene44573\_Sample\_011046841, Unigene12835\_Sample\_011046841, Unigene16924\_Sample\_011046841, Unigene11522\_Sample\_011046841, Unigene28658\_Sample\_011046841, Unigene24505\_Sample\_011046841, Unigene56695\_Sample\_011046841, Unigene3363\_Sample\_011046841, Unigene50611\_Sample\_011046841, Unigene42940\_Sample\_011046841, Unigene56019\_Sample\_011046841, Unigene60704\_Sample\_011046841, Unigene57429\_Sample\_011046841, Unigene13481\_Sample\_011046841, Unigene47693\_Sample\_011046841, Unigene41352\_Sample\_011046841, Unigene23180\_Sample\_011046841, Unigene7048\_Sample\_011046841, Unigene21917\_Sample\_011046841, Unigene9610\_Sample\_011046841, Unigene31483\_Sample\_011046841, Unigene49925\_Sample\_011046841, Unigene13211\_Sample\_011046841, Unigene59322\_Sample\_011046841, Unigene19947\_Sample\_011046841, Unigene18276\_Sample\_011046841, Unigene52184\_Sample\_011046841, Unigene44453\_Sample\_011046841, Unigene8418\_Sample\_011046841, Unigene11505\_Sample\_011046841, Unigene52951\_Sample\_011046841, Unigene59831\_Sample\_011046841, Unigene42697\_Sample\_011046841, Unigene40558\_Sample\_011046841, Unigene32540\_Sample\_011046841, Unigene49928\_Sample\_011046841, Unigene12081\_Sample\_011046841, Unigene39677\_Sample\_011046841, Unigene60191\_Sample\_011046841, Unigene54380\_Sample\_011046841, Unigene51009\_Sample\_011046841, Unigene58921\_Sample\_011046841, Unigene34034\_Sample\_011046841, Unigene13280\_Sample\_011046841, Unigene53313\_Sample\_011046841, Unigene41417\_Sample\_011046841, Unigene12621\_Sample\_011046841, Unigene24299\_Sample\_011046841, Unigene35259\_Sample\_011046841, Unigene12359\_Sample\_011046841, Unigene4378\_Sample\_011046841, Unigene28288\_Sample\_011046841, Unigene17962\_Sample\_011046841, Unigene59415\_Sample\_011046841, Unigene30813\_Sample\_011046841, Unigene45012\_Sample\_011046841, Unigene60575\_Sample\_011046841, Unigene54189\_Sample\_011046841, Unigene51879\_Sample\_011046841, Unigene15918\_Sample\_011046841, Unigene56777\_Sample\_011046841, Unigene9301\_Sample\_011046841, Unigene57298\_Sample\_011046841, Unigene52901\_Sample\_011046841, Unigene59984\_Sample\_011046841, Unigene34207\_Sample\_011046841, Unigene26541\_Sample\_011046841, Unigene47508\_Sample\_011046841, Unigene35763\_Sample\_011046841, Unigene52428\_Sample\_011046841, Unigene43141\_Sample\_011046841, Unigene60852\_Sample\_011046841, Unigene23449\_Sample\_011046841, Unigene47981\_Sample\_011046841, Unigene51882\_Sample\_011046841, Unigene12240\_Sample\_011046841, Unigene60131\_Sample\_011046841, Unigene12558\_Sample\_011046841, Unigene28693\_Sample\_011046841, Unigene60124\_Sample\_011046841, Unigene12894\_Sample\_011046841, Unigene19204\_Sample\_011046841, Unigene24234\_Sample\_011046841, Unigene58640\_Sample\_011046841, Unigene50966\_Sample\_011046841, Unigene56344\_Sample\_011046841, Unigene53602\_Sample\_011046841, Unigene22655\_Sample\_011046841, Unigene49151\_Sample\_011046841, Unigene56706\_Sample\_011046841, Unigene31821\_Sample\_011046841, Unigene48774\_Sample\_011046841, Unigene51947\_Sample\_011046841, Unigene37278\_Sample\_011046841, Unigene12562\_Sample\_011046841, Unigene26357\_Sample\_011046841, Unigene36824\_Sample\_011046841, Unigene59189\_Sample\_011046841, Unigene57939\_Sample\_011046841, Unigene31723\_Sample\_011046841, Unigene11706\_Sample\_011046841, Unigene56460\_Sample\_011046841, Unigene55205\_Sample\_011046841, Unigene1594\_Sample\_011046841, Unigene11592\_Sample\_011046841, Unigene57387\_Sample\_011046841, Unigene53191\_Sample\_011046841, Unigene47096\_Sample\_011046841, Unigene48977\_Sample\_011046841, Unigene28615\_Sample\_011046841, Unigene55772\_Sample\_011046841, Unigene53492\_Sample\_011046841, Unigene47220\_Sample\_011046841, Unigene13131\_Sample\_011046841, Unigene7181\_Sample\_011046841, Unigene52591\_Sample\_011046841, Unigene44354\_Sample\_011046841, Unigene50538\_Sample\_011046841, Unigene13122\_Sample\_011046841, Unigene47555\_Sample\_011046841, Unigene44544\_Sample\_011046841, Unigene57064\_Sample\_011046841, Unigene23757\_Sample\_011046841, Unigene59235\_Sample\_011046841, Unigene47871\_Sample\_011046841, Unigene11966\_Sample\_011046841, Unigene31083\_Sample\_011046841, Unigene13390\_Sample\_011046841, Unigene32523\_Sample\_011046841, Unigene36452\_Sample\_011046841, Unigene55077\_Sample\_011046841, Unigene4148\_Sample\_011046841, Unigene48006\_Sample\_011046841, Unigene50470\_Sample\_011046841, Unigene11804\_Sample\_011046841, Unigene12104\_Sample\_011046841, Unigene39261\_Sample\_011046841, Unigene51013\_Sample\_011046841, Unigene23065\_Sample\_011046841, Unigene48720\_Sample\_011046841, Unigene56878\_Sample\_011046841, Unigene59045\_Sample\_011046841, Unigene40628\_Sample\_011046841, Unigene11722\_Sample\_011046841, Unigene57719\_Sample\_011046841, Unigene59068\_Sample\_011046841, Unigene47923\_Sample\_011046841, Unigene13645\_Sample\_011046841, Unigene4830\_Sample\_011046841, Unigene53367\_Sample\_011046841, Unigene3202\_Sample\_011046841, Unigene46292\_Sample\_011046841, Unigene42536\_Sample\_011046841, Unigene47841\_Sample\_011046841, Unigene32928\_Sample\_011046841, Unigene15082\_Sample\_011046841, Unigene20001\_Sample\_011046841, Unigene58062\_Sample\_011046841, Unigene43216\_Sample\_011046841, Unigene33976\_Sample\_011046841, Unigene58863\_Sample\_011046841, Unigene3446\_Sample\_011046841, Unigene58040\_Sample\_011046841, Unigene12538\_Sample\_011046841, Unigene58463\_Sample\_011046841, Unigene60707\_Sample\_011046841, Unigene60574\_Sample\_011046841, Unigene53417\_Sample\_011046841, Unigene54701\_Sample\_011046841, Unigene49887\_Sample\_011046841, Unigene55329\_Sample\_011046841, Unigene40230\_Sample\_011046841, Unigene35773\_Sample\_011046841, Unigene59973\_Sample\_011046841, Unigene55610\_Sample\_011046841, Unigene50734\_Sample\_011046841, Unigene4978\_Sample\_011046841, Unigene31026\_Sample\_011046841, Unigene50178\_Sample\_011046841, Unigene17921\_Sample\_011046841, Unigene26327\_Sample\_011046841, Unigene58726\_Sample\_011046841, Unigene58462\_Sample\_011046841, Unigene36984\_Sample\_011046841, Unigene40474\_Sample\_011046841, Unigene59593\_Sample\_011046841, Unigene58408\_Sample\_011046841, Unigene17012\_Sample\_011046841, Unigene11201\_Sample\_011046841, Unigene11865\_Sample\_011046841, Unigene58195\_Sample\_011046841, Unigene53560\_Sample\_011046841, Unigene24137\_Sample\_011046841, Unigene27253\_Sample\_011046841, Unigene58481\_Sample\_011046841, Unigene54315\_Sample\_011046841, Unigene44997\_Sample\_011046841, Unigene41379\_Sample\_011046841, Unigene9242\_Sample\_011046841, Unigene25547\_Sample\_011046841, Unigene58267\_Sample\_011046841, Unigene60576\_Sample\_011046841, Unigene34582\_Sample\_011046841, Unigene33253\_Sample\_011046841, Unigene45777\_Sample\_011046841, Unigene49919\_Sample\_011046841, Unigene7885\_Sample\_011046841, Unigene43782\_Sample\_011046841, Unigene46027\_Sample\_011046841, Unigene43654\_Sample\_011046841, Unigene58531\_Sample\_011046841, Unigene57544\_Sample\_011046841, Unigene8526\_Sample\_011046841, Unigene56125\_Sample\_011046841, Unigene34164\_Sample\_011046841, Unigene22389\_Sample\_011046841, Unigene18375\_Sample\_011046841, Unigene55176\_Sample\_011046841, Unigene15720\_Sample\_011046841, Unigene47903\_Sample\_011046841, Unigene12971\_Sample\_011046841, Unigene55212\_Sample\_011046841, Unigene60018\_Sample\_011046841, Unigene56818\_Sample\_011046841, Unigene41465\_Sample\_011046841, Unigene59540\_Sample\_011046841, Unigene8468\_Sample\_011046841, Unigene50892\_Sample\_011046841, Unigene11755\_Sample\_011046841, Unigene50243\_Sample\_011046841, Unigene46841\_Sample\_011046841, Unigene47518\_Sample\_011046841, Unigene48005\_Sample\_011046841, Unigene19399\_Sample\_011046841, Unigene13463\_Sample\_011046841, Unigene43240\_Sample\_011046841, Unigene49909\_Sample\_011046841, Unigene56415\_Sample\_011046841, Unigene23236\_Sample\_011046841, Unigene60349\_Sample\_011046841, Unigene54619\_Sample\_011046841, Unigene43814\_Sample\_011046841, Unigene517\_Sample\_011046841, Unigene60369\_Sample\_011046841, Unigene53561\_Sample\_011046841, Unigene56484\_Sample\_011046841, Unigene60691\_Sample\_011046841, Unigene21308\_Sample\_011046841, Unigene40532\_Sample\_011046841, Unigene13454\_Sample\_011046841, Unigene20175\_Sample\_011046841, Unigene22189\_Sample\_011046841, Unigene45986\_Sample\_011046841, Unigene51426\_Sample\_011046841, Unigene4915\_Sample\_011046841, Unigene45949\_Sample\_011046841, Unigene46928\_Sample\_011046841, Unigene37180\_Sample\_011046841, Unigene56503\_Sample\_011046841, Unigene48127\_Sample\_011046841, Unigene14915\_Sample\_011046841, Unigene42844\_Sample\_011046841, Unigene13927\_Sample\_011046841, Unigene33209\_Sample\_011046841, Unigene11989\_Sample\_011046841, Unigene27589\_Sample\_011046841, Unigene13386\_Sample\_011046841, Unigene11536\_Sample\_011046841, Unigene6340\_Sample\_011046841, Unigene28537\_Sample\_011046841, Unigene59915\_Sample\_011046841, Unigene48197\_Sample\_011046841, Unigene4599\_Sample\_011046841, Unigene41844\_Sample\_011046841, Unigene57113\_Sample\_011046841, Unigene35922\_Sample\_011046841, Unigene27453\_Sample\_011046841, Unigene56265\_Sample\_011046841, Unigene40079\_Sample\_011046841, Unigene48975\_Sample\_011046841, Unigene40989\_Sample\_011046841, Unigene3968\_Sample\_011046841, Unigene16224\_Sample\_011046841, Unigene45661\_Sample\_011046841, Unigene35008\_Sample\_011046841, Unigene19743\_Sample\_011046841, Unigene53926\_Sample\_011046841, Unigene49869\_Sample\_011046841, Unigene38234\_Sample\_011046841, Unigene18654\_Sample\_011046841, Unigene56173\_Sample\_011046841, Unigene13656\_Sample\_011046841, Unigene54707\_Sample\_011046841, Unigene33194\_Sample\_011046841, Unigene22068\_Sample\_011046841, Unigene22555\_Sample\_011046841, Unigene50532\_Sample\_011046841, Unigene1049\_Sample\_011046841, Unigene42233\_Sample\_011046841, Unigene9008\_Sample\_011046841, Unigene55877\_Sample\_011046841, Unigene47025\_Sample\_011046841, Unigene50057\_Sample\_011046841, Unigene29187\_Sample\_011046841, Unigene53326\_Sample\_011046841, Unigene37422\_Sample\_011046841, Unigene56283\_Sample\_011046841, Unigene53041\_Sample\_011046841, Unigene44077\_Sample\_011046841, Unigene60906\_Sample\_011046841, Unigene41124\_Sample\_011046841, Unigene54601\_Sample\_011046841, Unigene55579\_Sample\_011046841, Unigene60946\_Sample\_011046841, Unigene1552\_Sample\_011046841, Unigene53462\_Sample\_011046841, Unigene40255\_Sample\_011046841, Unigene46337\_Sample\_011046841, Unigene53264\_Sample\_011046841, Unigene11539\_Sample\_011046841, Unigene59758\_Sample\_011046841, Unigene10317\_Sample\_011046841, Unigene13787\_Sample\_011046841, Unigene32973\_Sample\_011046841, Unigene12804\_Sample\_011046841, Unigene11563\_Sample\_011046841, Unigene23272\_Sample\_011046841, Unigene45754\_Sample\_011046841, Unigene28789\_Sample\_011046841, Unigene24615\_Sample\_011046841, Unigene30781\_Sample\_011046841, Unigene55504\_Sample\_011046841, Unigene13976\_Sample\_011046841, Unigene5051\_Sample\_011046841, Unigene54624\_Sample\_011046841, Unigene7299\_Sample\_011046841, Unigene45753\_Sample\_011046841, Unigene60323\_Sample\_011046841, Unigene55613\_Sample\_011046841, Unigene52551\_Sample\_011046841, Unigene20201\_Sample\_011046841, Unigene30684\_Sample\_011046841, Unigene49462\_Sample\_011046841, Unigene40287\_Sample\_011046841, Unigene7011\_Sample\_011046841, Unigene33032\_Sample\_011046841, Unigene54493\_Sample\_011046841, Unigene53217\_Sample\_011046841, Unigene47219\_Sample\_011046841, Unigene11142\_Sample\_011046841, Unigene60910\_Sample\_011046841, Unigene30320\_Sample\_011046841, Unigene23376\_Sample\_011046841, Unigene35902\_Sample\_011046841, Unigene44049\_Sample\_011046841, Unigene20312\_Sample\_011046841, Unigene55890\_Sample\_011046841, Unigene49397\_Sample\_011046841, Unigene52722\_Sample\_011046841, Unigene57559\_Sample\_011046841, Unigene57548\_Sample\_011046841, Unigene60117\_Sample\_011046841, Unigene11083\_Sample\_011046841, Unigene17629\_Sample\_011046841, Unigene49452\_Sample\_011046841, Unigene54414\_Sample\_011046841, Unigene12586\_Sample\_011046841, Unigene31613\_Sample\_011046841, Unigene20309\_Sample\_011046841, Unigene23921\_Sample\_011046841, Unigene9517\_Sample\_011046841, Unigene59035\_Sample\_011046841, Unigene4041\_Sample\_011046841, Unigene52423\_Sample\_011046841, Unigene23704\_Sample\_011046841, Unigene11478\_Sample\_011046841, Unigene13525\_Sample\_011046841, Unigene56362\_Sample\_011046841, Unigene32111\_Sample\_011046841, Unigene12010\_Sample\_011046841, Unigene37760\_Sample\_011046841, Unigene56185\_Sample\_011046841, Unigene50449\_Sample\_011046841, Unigene34757\_Sample\_011046841, Unigene11256\_Sample\_011046841, Unigene49602\_Sample\_011046841, Unigene25155\_Sample\_011046841, Unigene19407\_Sample\_011046841, Unigene45458\_Sample\_011046841, Unigene21749\_Sample\_011046841, Unigene41781\_Sample\_011046841, Unigene5005\_Sample\_011046841, Unigene11134\_Sample\_011046841, Unigene4749\_Sample\_011046841, Unigene11339\_Sample\_011046841, Unigene4438\_Sample\_011046841, Unigene31534\_Sample\_011046841, Unigene40491\_Sample\_011046841, Unigene38631\_Sample\_011046841, Unigene41153\_Sample\_011046841, Unigene55470\_Sample\_011046841, Unigene54684\_Sample\_011046841, Unigene56828\_Sample\_011046841, Unigene41980\_Sample\_011046841, Unigene48316\_Sample\_011046841, Unigene22175\_Sample\_011046841, Unigene52367\_Sample\_011046841, Unigene47018\_Sample\_011046841, Unigene10421\_Sample\_011046841, Unigene50823\_Sample\_011046841, Unigene50088\_Sample\_011046841, Unigene52530\_Sample\_011046841, Unigene46829\_Sample\_011046841, Unigene53997\_Sample\_011046841, Unigene11168\_Sample\_011046841, Unigene54220\_Sample\_011046841, Unigene13683\_Sample\_011046841, Unigene11842\_Sample\_011046841, Unigene31415\_Sample\_011046841, Unigene50036\_Sample\_011046841, Unigene41842\_Sample\_011046841, Unigene16874\_Sample\_011046841, Unigene60357\_Sample\_011046841, Unigene53581\_Sample\_011046841, Unigene45898\_Sample\_011046841, Unigene341\_Sample\_011046841, Unigene10461\_Sample\_011046841, Unigene31772\_Sample\_011046841, Unigene21770\_Sample\_011046841, Unigene54581\_Sample\_011046841, Unigene56901\_Sample\_011046841, Unigene15270\_Sample\_011046841, Unigene9858\_Sample\_011046841, Unigene46543\_Sample\_011046841, Unigene48415\_Sample\_011046841, Unigene44108\_Sample\_011046841, Unigene54454\_Sample\_011046841, Unigene57724\_Sample\_011046841, Unigene17471\_Sample\_011046841, Unigene49046\_Sample\_011046841, Unigene31768\_Sample\_011046841, Unigene26961\_Sample\_011046841, Unigene48260\_Sample\_011046841, Unigene60032\_Sample\_011046841, Unigene50641\_Sample\_011046841, Unigene12172\_Sample\_011046841, Unigene9122\_Sample\_011046841, Unigene39987\_Sample\_011046841, Unigene48544\_Sample\_011046841, Unigene53650\_Sample\_011046841, Unigene58259\_Sample\_011046841, Unigene55841\_Sample\_011046841, Unigene55971\_Sample\_011046841, Unigene56638\_Sample\_011046841, Unigene57702\_Sample\_011046841, Unigene43340\_Sample\_011046841, Unigene56139\_Sample\_011046841, Unigene20899\_Sample\_011046841, Unigene34225\_Sample\_011046841, Unigene51076\_Sample\_011046841, Unigene41460\_Sample\_011046841, Unigene15121\_Sample\_011046841, Unigene27784\_Sample\_011046841, Unigene43397\_Sample\_011046841, Unigene60604\_Sample\_011046841, Unigene55052\_Sample\_011046841, Unigene55806\_Sample\_011046841, Unigene42860\_Sample\_011046841, Unigene49009\_Sample\_011046841, Unigene52864\_Sample\_011046841, Unigene24015\_Sample\_011046841, Unigene30474\_Sample\_011046841, Unigene49448\_Sample\_011046841, Unigene52502\_Sample\_011046841, Unigene41287\_Sample\_011046841, Unigene32216\_Sample\_011046841, Unigene44927\_Sample\_011046841, Unigene59061\_Sample\_011046841, Unigene11114\_Sample\_011046841, Unigene58355\_Sample\_011046841, Unigene57890\_Sample\_011046841, Unigene43596\_Sample\_011046841, Unigene51458\_Sample\_011046841, Unigene13436\_Sample\_011046841, Unigene44587\_Sample\_011046841, Unigene29524\_Sample\_011046841, Unigene26229\_Sample\_011046841, Unigene354\_Sample\_011046841, Unigene51519\_Sample\_011046841, Unigene50506\_Sample\_011046841, Unigene16207\_Sample\_011046841, Unigene32486\_Sample\_011046841, Unigene1129\_Sample\_011046841, Unigene8290\_Sample\_011046841, Unigene60175\_Sample\_011046841, Unigene56258\_Sample\_011046841, Unigene58033\_Sample\_011046841, Unigene60388\_Sample\_011046841, Unigene21961\_Sample\_011046841, Unigene20434\_Sample\_011046841, Unigene21456\_Sample\_011046841, Unigene3945\_Sample\_011046841, Unigene23925\_Sample\_011046841, Unigene59547\_Sample\_011046841, Unigene2138\_Sample\_011046841, Unigene12482\_Sample\_011046841, Unigene27770\_Sample\_011046841, Unigene53639\_Sample\_011046841, Unigene60561\_Sample\_011046841, Unigene11448\_Sample\_011046841, Unigene12840\_Sample\_011046841, Unigene58386\_Sample\_011046841, Unigene37232\_Sample\_011046841, Unigene60709\_Sample\_011046841, Unigene863\_Sample\_011046841, Unigene27576\_Sample\_011046841, Unigene3358\_Sample\_011046841, Unigene5615\_Sample\_011046841, Unigene13633\_Sample\_011046841, Unigene39678\_Sample\_011046841, Unigene38492\_Sample\_011046841, Unigene41323\_Sample\_011046841, Unigene39444\_Sample\_011046841, Unigene55575\_Sample\_011046841, Unigene58845\_Sample\_011046841, Unigene56532\_Sample\_011046841, Unigene49487\_Sample\_011046841, Unigene5570\_Sample\_011046841, Unigene43894\_Sample\_011046841, Unigene12594\_Sample\_011046841, Unigene54290\_Sample\_011046841, Unigene36924\_Sample\_011046841, Unigene35276\_Sample\_011046841, Unigene30101\_Sample\_011046841, Unigene17850\_Sample\_011046841, Unigene48170\_Sample\_011046841, Unigene40858\_Sample\_011046841, Unigene56949\_Sample\_011046841, Unigene42148\_Sample\_011046841, Unigene11635\_Sample\_011046841, Unigene59567\_Sample\_011046841, Unigene60123\_Sample\_011046841, Unigene39488\_Sample\_011046841, Unigene58169\_Sample\_011046841, Unigene45620\_Sample\_011046841, Unigene457\_Sample\_011046841, Unigene59136\_Sample\_011046841, Unigene59621\_Sample\_011046841, Unigene55529\_Sample\_011046841, Unigene54555\_Sample\_011046841, Unigene38618\_Sample\_011046841, Unigene47707\_Sample\_011046841, Unigene55300\_Sample\_011046841, Unigene26587\_Sample\_011046841, Unigene35963\_Sample\_011046841, Unigene55754\_Sample\_011046841, Unigene38441\_Sample\_011046841, Unigene10613\_Sample\_011046841, Unigene16357\_Sample\_011046841, Unigene57524\_Sample\_011046841, Unigene30691\_Sample\_011046841, Unigene40162\_Sample\_011046841, Unigene46201\_Sample\_011046841, Unigene54167\_Sample\_011046841, Unigene11648\_Sample\_011046841, Unigene16727\_Sample\_011046841, Unigene52106\_Sample\_011046841, Unigene42036\_Sample\_011046841, Unigene59457\_Sample\_011046841, Unigene45675\_Sample\_011046841, Unigene11867\_Sample\_011046841, Unigene34810\_Sample\_011046841, Unigene22974\_Sample\_011046841, Unigene23568\_Sample\_011046841, Unigene18447\_Sample\_011046841, Unigene39921\_Sample\_011046841, Unigene8266\_Sample\_011046841, Unigene41696\_Sample\_011046841, Unigene21082\_Sample\_011046841, Unigene5102\_Sample\_011046841, Unigene31642\_Sample\_011046841, Unigene31749\_Sample\_011046841, Unigene18294\_Sample\_011046841, Unigene51737\_Sample\_011046841, Unigene32629\_Sample\_011046841, Unigene17110\_Sample\_011046841, Unigene51622\_Sample\_011046841, Unigene49371\_Sample\_011046841, Unigene8255\_Sample\_011046841, Unigene43561\_Sample\_011046841, Unigene55158\_Sample\_011046841, Unigene25489\_Sample\_011046841, Unigene69\_Sample\_011046841, Unigene51555\_Sample\_011046841, Unigene48882\_Sample\_011046841, Unigene59679\_Sample\_011046841, Unigene32103\_Sample\_011046841, Unigene53015\_Sample\_011046841, Unigene25087\_Sample\_011046841, Unigene58138\_Sample\_011046841, Unigene20591\_Sample\_011046841, Unigene47753\_Sample\_011046841, Unigene10388\_Sample\_011046841, Unigene1829\_Sample\_011046841, Unigene37995\_Sample\_011046841, Unigene60969\_Sample\_011046841, Unigene48423\_Sample\_011046841, Unigene8039\_Sample\_011046841, Unigene34457\_Sample\_011046841, Unigene9329\_Sample\_011046841, Unigene47357\_Sample\_011046841, Unigene11116\_Sample\_011046841, Unigene8499\_Sample\_011046841, Unigene6724\_Sample\_011046841, Unigene37898\_Sample\_011046841, Unigene37098\_Sample\_011046841, Unigene42812\_Sample\_011046841, Unigene60741\_Sample\_011046841, Unigene56005\_Sample\_011046841, Unigene22457\_Sample\_011046841, Unigene52704\_Sample\_011046841, Unigene55306\_Sample\_011046841, Unigene49677\_Sample\_011046841, Unigene45779\_Sample\_011046841, Unigene60670\_Sample\_011046841, Unigene6676\_Sample\_011046841, Unigene42852\_Sample\_011046841, Unigene16822\_Sample\_011046841, Unigene9689\_Sample\_011046841, Unigene60889\_Sample\_011046841, Unigene42079\_Sample\_011046841, Unigene52294\_Sample\_011046841, Unigene46510\_Sample\_011046841, Unigene51613\_Sample\_011046841, Unigene58387\_Sample\_011046841, Unigene39094\_Sample\_011046841, Unigene58929\_Sample\_011046841, Unigene49392\_Sample\_011046841, Unigene13203\_Sample\_011046841, Unigene33127\_Sample\_011046841, Unigene34566\_Sample\_011046841, Unigene51331\_Sample\_011046841, Unigene50921\_Sample\_011046841, Unigene19596\_Sample\_011046841, Unigene8243\_Sample\_011046841, Unigene42042\_Sample\_011046841, Unigene45131\_Sample\_011046841, Unigene54201\_Sample\_011046841, Unigene12266\_Sample\_011046841, Unigene38974\_Sample\_011046841, Unigene58987\_Sample\_011046841, Unigene54014\_Sample\_011046841, Unigene41450\_Sample\_011046841, Unigene53797\_Sample\_011046841, Unigene58535\_Sample\_011046841, Unigene9219\_Sample\_011046841, Unigene53893\_Sample\_011046841, Unigene54329\_Sample\_011046841, Unigene56049\_Sample\_011046841, Unigene27430\_Sample\_011046841, Unigene36873\_Sample\_011046841, Unigene11300\_Sample\_011046841, Unigene54552\_Sample\_011046841, Unigene47368\_Sample\_011046841, Unigene60296\_Sample\_011046841, Unigene56920\_Sample\_011046841, Unigene56058\_Sample\_011046841, Unigene12913\_Sample\_011046841, Unigene49698\_Sample\_011046841, Unigene10930\_Sample\_011046841, Unigene38373\_Sample\_011046841, Unigene20267\_Sample\_011046841, Unigene49418\_Sample\_011046841, Unigene54071\_Sample\_011046841, Unigene55673\_Sample\_011046841, Unigene46000\_Sample\_011046841, Unigene50674\_Sample\_011046841, Unigene31746\_Sample\_011046841, Unigene49640\_Sample\_011046841, Unigene52955\_Sample\_011046841, Unigene58879\_Sample\_011046841, Unigene4457\_Sample\_011046841, Unigene11195\_Sample\_011046841, Unigene31632\_Sample\_011046841, Unigene49633\_Sample\_011046841, Unigene17132\_Sample\_011046841, Unigene53334\_Sample\_011046841, Unigene45873\_Sample\_011046841, Unigene60912\_Sample\_011046841, Unigene21480\_Sample\_011046841, Unigene17021\_Sample\_011046841, Unigene44821\_Sample\_011046841, Unigene10676\_Sample\_011046841, Unigene49525\_Sample\_011046841, Unigene59819\_Sample\_011046841, Unigene54663\_Sample\_011046841, Unigene13744\_Sample\_011046841, Unigene41123\_Sample\_011046841, Unigene58882\_Sample\_011046841, Unigene37545\_Sample\_011046841, Unigene18305\_Sample\_011046841, Unigene11758\_Sample\_011046841, Unigene55308\_Sample\_011046841, Unigene49465\_Sample\_011046841, Unigene19753\_Sample\_011046841, Unigene11874\_Sample\_011046841, Unigene55568\_Sample\_011046841, Unigene59134\_Sample\_011046841, Unigene60683\_Sample\_011046841, Unigene57834\_Sample\_011046841, Unigene1803\_Sample\_011046841, Unigene31705\_Sample\_011046841, Unigene56096\_Sample\_011046841, Unigene30285\_Sample\_011046841, Unigene54874\_Sample\_011046841, Unigene42637\_Sample\_011046841, Unigene58509\_Sample\_011046841, Unigene24860\_Sample\_011046841, Unigene48003\_Sample\_011046841, Unigene18899\_Sample\_011046841, Unigene46783\_Sample\_011046841, Unigene56734\_Sample\_011046841, Unigene60681\_Sample\_011046841, Unigene48869\_Sample\_011046841, Unigene26254\_Sample\_011046841, Unigene50862\_Sample\_011046841, Unigene46263\_Sample\_011046841, Unigene57696\_Sample\_011046841, Unigene42749\_Sample\_011046841, Unigene30072\_Sample\_011046841, Unigene11662\_Sample\_011046841, Unigene29183\_Sample\_011046841, Unigene59038\_Sample\_011046841, Unigene38930\_Sample\_011046841, Unigene11676\_Sample\_011046841, Unigene52253\_Sample\_011046841, Unigene6707\_Sample\_011046841, Unigene60288\_Sample\_011046841, Unigene7850\_Sample\_011046841, Unigene59431\_Sample\_011046841, Unigene54900\_Sample\_011046841, Unigene39020\_Sample\_011046841, Unigene52140\_Sample\_011046841, Unigene57687\_Sample\_011046841, Unigene42561\_Sample\_011046841, Unigene641\_Sample\_011046841, Unigene40093\_Sample\_011046841, Unigene50808\_Sample\_011046841, Unigene34280\_Sample\_011046841, Unigene16237\_Sample\_011046841, Unigene35828\_Sample\_011046841, Unigene49481\_Sample\_011046841, Unigene20433\_Sample\_011046841, Unigene19919\_Sample\_011046841, Unigene1934\_Sample\_011046841, Unigene1260\_Sample\_011046841, Unigene19030\_Sample\_011046841, Unigene20911\_Sample\_011046841, Unigene51777\_Sample\_011046841, Unigene50002\_Sample\_011046841, Unigene60088\_Sample\_011046841, Unigene53179\_Sample\_011046841, Unigene27324\_Sample\_011046841, Unigene55162\_Sample\_011046841, Unigene57052\_Sample\_011046841, Unigene48482\_Sample\_011046841, Unigene37880\_Sample\_011046841, Unigene59179\_Sample\_011046841, Unigene40131\_Sample\_011046841, Unigene50681\_Sample\_011046841, Unigene50602\_Sample\_011046841, Unigene51258\_Sample\_011046841, Unigene50764\_Sample\_011046841, Unigene35648\_Sample\_011046841, Unigene56697\_Sample\_011046841, Unigene45674\_Sample\_011046841, Unigene33792\_Sample\_011046841, Unigene43020\_Sample\_011046841, Unigene10356\_Sample\_011046841, Unigene26672\_Sample\_011046841, Unigene57058\_Sample\_011046841, Unigene13791\_Sample\_011046841, Unigene23818\_Sample\_011046841, Unigene52116\_Sample\_011046841, Unigene52105\_Sample\_011046841, Unigene58190\_Sample\_011046841, Unigene50236\_Sample\_011046841, Unigene52342\_Sample\_011046841, Unigene25623\_Sample\_011046841, Unigene35672\_Sample\_011046841, Unigene1995\_Sample\_011046841, Unigene51029\_Sample\_011046841, Unigene35911\_Sample\_011046841, Unigene59442\_Sample\_011046841, Unigene58495\_Sample\_011046841, Unigene47924\_Sample\_011046841, Unigene56378\_Sample\_011046841, Unigene35914\_Sample\_011046841, Unigene8459\_Sample\_011046841, Unigene52467\_Sample\_011046841, Unigene54174\_Sample\_011046841, Unigene58688\_Sample\_011046841, Unigene52543\_Sample\_011046841, Unigene32032\_Sample\_011046841, Unigene13261\_Sample\_011046841, Unigene54859\_Sample\_011046841, Unigene53942\_Sample\_011046841, Unigene36083\_Sample\_011046841, Unigene60055\_Sample\_011046841, Unigene49291\_Sample\_011046841, Unigene35289\_Sample\_011046841, Unigene19688\_Sample\_011046841, Unigene33317\_Sample\_011046841, Unigene48698\_Sample\_011046841, Unigene49182\_Sample\_011046841, Unigene25993\_Sample\_011046841, Unigene13951\_Sample\_011046841, Unigene55840\_Sample\_011046841, Unigene47273\_Sample\_011046841, Unigene8278\_Sample\_011046841, Unigene11088\_Sample\_011046841, Unigene43963\_Sample\_011046841, Unigene56208\_Sample\_011046841, Unigene42794\_Sample\_011046841, Unigene8581\_Sample\_011046841, Unigene10370\_Sample\_011046841, Unigene22331\_Sample\_011046841, Unigene58223\_Sample\_011046841, Unigene11351\_Sample\_011046841, Unigene13007\_Sample\_011046841, Unigene44793\_Sample\_011046841, Unigene47872\_Sample\_011046841, Unigene48598\_Sample\_011046841, Unigene12822\_Sample\_011046841, Unigene33755\_Sample\_011046841, Unigene8654\_Sample\_011046841, Unigene7403\_Sample\_011046841, Unigene4173\_Sample\_011046841, Unigene52910\_Sample\_011046841, Unigene1591\_Sample\_011046841, Unigene31695\_Sample\_011046841, Unigene23133\_Sample\_011046841, Unigene21061\_Sample\_011046841, Unigene57858\_Sample\_011046841, Unigene7639\_Sample\_011046841, Unigene58022\_Sample\_011046841, Unigene54835\_Sample\_011046841, Unigene31515\_Sample\_011046841, Unigene16810\_Sample\_011046841, Unigene59727\_Sample\_011046841, Unigene47195\_Sample\_011046841, Unigene34813\_Sample\_011046841, Unigene12501\_Sample\_011046841, Unigene28348\_Sample\_011046841, Unigene44142\_Sample\_011046841, Unigene33132\_Sample\_011046841, Unigene35691\_Sample\_011046841, Unigene55527\_Sample\_011046841, Unigene13753\_Sample\_011046841, Unigene11050\_Sample\_011046841, Unigene44567\_Sample\_011046841, Unigene52393\_Sample\_011046841, Unigene13447\_Sample\_011046841, Unigene20484\_Sample\_011046841, Unigene30190\_Sample\_011046841, Unigene60955\_Sample\_011046841, Unigene5177\_Sample\_011046841, Unigene23466\_Sample\_011046841, Unigene8894\_Sample\_011046841, Unigene53254\_Sample\_011046841, Unigene14570\_Sample\_011046841, Unigene14358\_Sample\_011046841, Unigene16199\_Sample\_011046841, Unigene47355\_Sample\_011046841, Unigene12510\_Sample\_011046841, Unigene49055\_Sample\_011046841, Unigene40271\_Sample\_011046841, Unigene60\_Sample\_011046841, Unigene49664\_Sample\_011046841, Unigene55423\_Sample\_011046841, Unigene34450\_Sample\_011046841, Unigene60507\_Sample\_011046841, Unigene50825\_Sample\_011046841, Unigene40648\_Sample\_011046841, Unigene53415\_Sample\_011046841, Unigene10598\_Sample\_011046841, Unigene14528\_Sample\_011046841, Unigene14538\_Sample\_011046841, Unigene41467\_Sample\_011046841, Unigene11790\_Sample\_011046841, Unigene55404\_Sample\_011046841, Unigene55983\_Sample\_011046841, Unigene28678\_Sample\_011046841, Unigene53352\_Sample\_011046841, Unigene59001\_Sample\_011046841, Unigene18791\_Sample\_011046841, Unigene58546\_Sample\_011046841, Unigene60897\_Sample\_011046841, Unigene7095\_Sample\_011046841, Unigene58934\_Sample\_011046841, Unigene26757\_Sample\_011046841, Unigene31545\_Sample\_011046841, Unigene53861\_Sample\_011046841, Unigene53838\_Sample\_011046841, Unigene59059\_Sample\_011046841, Unigene32360\_Sample\_011046841, Unigene45952\_Sample\_011046841, Unigene53913\_Sample\_011046841, Unigene40565\_Sample\_011046841, Unigene32964\_Sample\_011046841, Unigene39138\_Sample\_011046841, Unigene49337\_Sample\_011046841, Unigene40767\_Sample\_011046841, Unigene40440\_Sample\_011046841, Unigene44497\_Sample\_011046841, Unigene15792\_Sample\_011046841, Unigene43096\_Sample\_011046841, Unigene57304\_Sample\_011046841, Unigene55938\_Sample\_011046841, Unigene40747\_Sample\_011046841, Unigene54718\_Sample\_011046841, Unigene37080\_Sample\_011046841, Unigene40823\_Sample\_011046841, Unigene10548\_Sample\_011046841, Unigene41001\_Sample\_011046841, Unigene45662\_Sample\_011046841, Unigene37743\_Sample\_011046841, Unigene60245\_Sample\_011046841, Unigene54460\_Sample\_011046841, Unigene59893\_Sample\_011046841, Unigene32550\_Sample\_011046841, Unigene59036\_Sample\_011046841, Unigene53128\_Sample\_011046841, Unigene31154\_Sample\_011046841, Unigene55409\_Sample\_011046841, Unigene22962\_Sample\_011046841, Unigene54574\_Sample\_011046841, Unigene42346\_Sample\_011046841, Unigene60537\_Sample\_011046841, Unigene54003\_Sample\_011046841, Unigene59877\_Sample\_011046841, Unigene53888\_Sample\_011046841, Unigene1010\_Sample\_011046841, Unigene55301\_Sample\_011046841, Unigene50746\_Sample\_011046841, Unigene58301\_Sample\_011046841, Unigene9752\_Sample\_011046841, Unigene48238\_Sample\_011046841, Unigene13836\_Sample\_011046841, Unigene59872\_Sample\_011046841, Unigene36891\_Sample\_011046841, Unigene29578\_Sample\_011046841, Unigene41207\_Sample\_011046841, Unigene7964\_Sample\_011046841, Unigene19091\_Sample\_011046841, Unigene41135\_Sample\_011046841, Unigene49953\_Sample\_011046841, Unigene50114\_Sample\_011046841, Unigene45049\_Sample\_011046841, Unigene42742\_Sample\_011046841, Unigene35406\_Sample\_011046841, Unigene53343\_Sample\_011046841, Unigene57353\_Sample\_011046841, Unigene10135\_Sample\_011046841, Unigene55624\_Sample\_011046841, Unigene38349\_Sample\_011046841, Unigene48926\_Sample\_011046841, Unigene36908\_Sample\_011046841, Unigene48090\_Sample\_011046841, Unigene386\_Sample\_011046841, Unigene55720\_Sample\_011046841, Unigene16264\_Sample\_011046841, Unigene55545\_Sample\_011046841, Unigene3542\_Sample\_011046841, Unigene37896\_Sample\_011046841, Unigene24754\_Sample\_011046841, Unigene54711\_Sample\_011046841, Unigene34657\_Sample\_011046841, Unigene19355\_Sample\_011046841, Unigene59459\_Sample\_011046841, Unigene48067\_Sample\_011046841, Unigene22531\_Sample\_011046841, Unigene16987\_Sample\_011046841, Unigene7295\_Sample\_011046841, Unigene17531\_Sample\_011046841, Unigene41231\_Sample\_011046841, Unigene46275\_Sample\_011046841, Unigene60407\_Sample\_011046841, Unigene39087\_Sample\_011046841, Unigene59177\_Sample\_011046841, Unigene45001\_Sample\_011046841, Unigene57973\_Sample\_011046841, Unigene32121\_Sample\_011046841, Unigene41604\_Sample\_011046841, Unigene7116\_Sample\_011046841, Unigene12189\_Sample\_011046841, Unigene19608\_Sample\_011046841, Unigene40355\_Sample\_011046841, Unigene50938\_Sample\_011046841, Unigene2221\_Sample\_011046841, Unigene53424\_Sample\_011046841, Unigene34802\_Sample\_011046841, Unigene11984\_Sample\_011046841, Unigene36178\_Sample\_011046841, Unigene58011\_Sample\_011046841, Unigene34405\_Sample\_011046841, Unigene29181\_Sample\_011046841, Unigene56746\_Sample\_011046841, Unigene58692\_Sample\_011046841, Unigene44243\_Sample\_011046841, Unigene24797\_Sample\_011046841, Unigene14116\_Sample\_011046841, Unigene27026\_Sample\_011046841, Unigene56877\_Sample\_011046841, Unigene60100\_Sample\_011046841, Unigene10179\_Sample\_011046841, Unigene55489\_Sample\_011046841, Unigene8272\_Sample\_011046841, Unigene51581\_Sample\_011046841, Unigene38889\_Sample\_011046841, Unigene35814\_Sample\_011046841, Unigene58681\_Sample\_011046841, Unigene9408\_Sample\_011046841, Unigene21679\_Sample\_011046841, Unigene56383\_Sample\_011046841, Unigene58152\_Sample\_011046841, Unigene18356\_Sample\_011046841, Unigene57887\_Sample\_011046841, Unigene54386\_Sample\_011046841, Unigene57760\_Sample\_011046841, Unigene44025\_Sample\_011046841, Unigene56885\_Sample\_011046841, Unigene55494\_Sample\_011046841, Unigene32534\_Sample\_011046841, Unigene5954\_Sample\_011046841, Unigene60014\_Sample\_011046841, Unigene21318\_Sample\_011046841, Unigene60258\_Sample\_011046841, Unigene17505\_Sample\_011046841, Unigene3659\_Sample\_011046841, Unigene13456\_Sample\_011046841, Unigene46158\_Sample\_011046841, Unigene48072\_Sample\_011046841, Unigene50143\_Sample\_011046841, Unigene46280\_Sample\_011046841, Unigene47635\_Sample\_011046841, Unigene53377\_Sample\_011046841, Unigene34160\_Sample\_011046841, Unigene39627\_Sample\_011046841, Unigene21404\_Sample\_011046841, Unigene55779\_Sample\_011046841, Unigene53787\_Sample\_011046841, Unigene57194\_Sample\_011046841, Unigene46950\_Sample\_011046841, Unigene13947\_Sample\_011046841, Unigene58343\_Sample\_011046841, Unigene12326\_Sample\_011046841, Unigene53231\_Sample\_011046841, Unigene33738\_Sample\_011046841, Unigene55896\_Sample\_011046841, Unigene3856\_Sample\_011046841, Unigene36872\_Sample\_011046841, Unigene24449\_Sample\_011046841, Unigene12633\_Sample\_011046841, Unigene56843\_Sample\_011046841, Unigene53676\_Sample\_011046841, Unigene43986\_Sample\_011046841, Unigene40610\_Sample\_011046841, Unigene48863\_Sample\_011046841, Unigene3670\_Sample\_011046841, Unigene59422\_Sample\_011046841, Unigene48590\_Sample\_011046841, Unigene43792\_Sample\_011046841, Unigene16092\_Sample\_011046841, Unigene12957\_Sample\_011046841, Unigene12559\_Sample\_011046841, Unigene11321\_Sample\_011046841, Unigene8056\_Sample\_011046841, Unigene43528\_Sample\_011046841, Unigene9211\_Sample\_011046841, Unigene8299\_Sample\_011046841, Unigene12412\_Sample\_011046841, Unigene54372\_Sample\_011046841, Unigene38527\_Sample\_011046841, Unigene31794\_Sample\_011046841, Unigene58989\_Sample\_011046841, Unigene39557\_Sample\_011046841, Unigene10648\_Sample\_011046841, Unigene25083\_Sample\_011046841, Unigene60125\_Sample\_011046841, Unigene60424\_Sample\_011046841, Unigene45708\_Sample\_011046841, Unigene57678\_Sample\_011046841, Unigene53451\_Sample\_011046841, Unigene53963\_Sample\_011046841, Unigene32947\_Sample\_011046841, Unigene42196\_Sample\_011046841, Unigene7801\_Sample\_011046841, Unigene34629\_Sample\_011046841, Unigene49851\_Sample\_011046841, Unigene60365\_Sample\_011046841, Unigene6794\_Sample\_011046841, Unigene57691\_Sample\_011046841, Unigene24614\_Sample\_011046841, Unigene10250\_Sample\_011046841, Unigene52866\_Sample\_011046841, Unigene10419\_Sample\_011046841, Unigene54851\_Sample\_011046841, Unigene24848\_Sample\_011046841, Unigene55419\_Sample\_011046841, Unigene9589\_Sample\_011046841, Unigene54231\_Sample\_011046841, Unigene56578\_Sample\_011046841, Unigene47763\_Sample\_011046841, Unigene60552\_Sample\_011046841, Unigene16118\_Sample\_011046841, Unigene60461\_Sample\_011046841, Unigene40873\_Sample\_011046841, Unigene30076\_Sample\_011046841, Unigene53661\_Sample\_011046841, Unigene52100\_Sample\_011046841, Unigene41607\_Sample\_011046841, Unigene25192\_Sample\_011046841, Unigene57913\_Sample\_011046841, Unigene8772\_Sample\_011046841, Unigene60433\_Sample\_011046841, Unigene57549\_Sample\_011046841, Unigene45561\_Sample\_011046841, Unigene3047\_Sample\_011046841, Unigene6313\_Sample\_011046841, Unigene57835\_Sample\_011046841, Unigene25028\_Sample\_011046841, Unigene37663\_Sample\_011046841, Unigene11703\_Sample\_011046841, Unigene40122\_Sample\_011046841, Unigene54571\_Sample\_011046841, Unigene28815\_Sample\_011046841, Unigene12673\_Sample\_011046841, Unigene56979\_Sample\_011046841, Unigene25764\_Sample\_011046841, Unigene25059\_Sample\_011046841, Unigene42523\_Sample\_011046841, Unigene19363\_Sample\_011046841, Unigene22636\_Sample\_011046841, Unigene2482\_Sample\_011046841, Unigene39222\_Sample\_011046841, Unigene38167\_Sample\_011046841, Unigene36867\_Sample\_011046841, Unigene55981\_Sample\_011046841, Unigene35857\_Sample\_011046841, Unigene50110\_Sample\_011046841, Unigene46283\_Sample\_011046841, Unigene41724\_Sample\_011046841, Unigene27013\_Sample\_011046841, Unigene15188\_Sample\_011046841, Unigene5554\_Sample\_011046841, Unigene13366\_Sample\_011046841, Unigene45114\_Sample\_011046841, Unigene49240\_Sample\_011046841, Unigene34525\_Sample\_011046841, Unigene59077\_Sample\_011046841, Unigene13034\_Sample\_011046841, Unigene59434\_Sample\_011046841, Unigene12808\_Sample\_011046841, Unigene51147\_Sample\_011046841, Unigene21562\_Sample\_011046841, Unigene54910\_Sample\_011046841, Unigene59469\_Sample\_011046841, Unigene60797\_Sample\_011046841, Unigene42676\_Sample\_011046841, Unigene19051\_Sample\_011046841, Unigene40677\_Sample\_011046841, Unigene11231\_Sample\_011046841, Unigene12458\_Sample\_011046841, Unigene57291\_Sample\_011046841, Unigene56000\_Sample\_011046841, Unigene34543\_Sample\_011046841, Unigene469\_Sample\_011046841, Unigene27245\_Sample\_011046841, Unigene13070\_Sample\_011046841, Unigene10726\_Sample\_011046841, Unigene36243\_Sample\_011046841, Unigene60321\_Sample\_011046841, Unigene8166\_Sample\_011046841, Unigene18575\_Sample\_011046841, Unigene29968\_Sample\_011046841, Unigene37222\_Sample\_011046841, Unigene57271\_Sample\_011046841, Unigene59513\_Sample\_011046841, Unigene12057\_Sample\_011046841, Unigene9348\_Sample\_011046841, Unigene39783\_Sample\_011046841, Unigene43716\_Sample\_011046841, Unigene39868\_Sample\_011046841, Unigene55065\_Sample\_011046841, Unigene48345\_Sample\_011046841, Unigene27682\_Sample\_011046841, Unigene49895\_Sample\_011046841, Unigene48317\_Sample\_011046841, Unigene45592\_Sample\_011046841, Unigene16763\_Sample\_011046841, Unigene18563\_Sample\_011046841, Unigene13959\_Sample\_011046841, Unigene47010\_Sample\_011046841, Unigene55282\_Sample\_011046841, Unigene39289\_Sample\_011046841, Unigene8557\_Sample\_011046841, Unigene58121\_Sample\_011046841, Unigene34060\_Sample\_011046841, Unigene59404\_Sample\_011046841, Unigene55671\_Sample\_011046841, Unigene57181\_Sample\_011046841, Unigene10027\_Sample\_011046841, Unigene12939\_Sample\_011046841, Unigene56883\_Sample\_011046841, Unigene57459\_Sample\_011046841, Unigene1918\_Sample\_011046841, Unigene30281\_Sample\_011046841, Unigene19179\_Sample\_011046841, Unigene31420\_Sample\_011046841, Unigene54521\_Sample\_011046841, Unigene58502\_Sample\_011046841, Unigene56023\_Sample\_011046841, Unigene41959\_Sample\_011046841, Unigene11373\_Sample\_011046841, Unigene54104\_Sample\_011046841, Unigene13733\_Sample\_011046841, Unigene28953\_Sample\_011046841, Unigene19951\_Sample\_011046841, Unigene12629\_Sample\_011046841, Unigene49498\_Sample\_011046841, Unigene19989\_Sample\_011046841, Unigene15862\_Sample\_011046841, Unigene50912\_Sample\_011046841, Unigene35762\_Sample\_011046841, Unigene57062\_Sample\_011046841, Unigene60834\_Sample\_011046841, Unigene55433\_Sample\_011046841, Unigene58466\_Sample\_011046841, Unigene1228\_Sample\_011046841, Unigene15548\_Sample\_011046841, Unigene50798\_Sample\_011046841, Unigene58106\_Sample\_011046841, Unigene23425\_Sample\_011046841, Unigene51218\_Sample\_011046841, Unigene43705\_Sample\_011046841, Unigene52118\_Sample\_011046841, Unigene28681\_Sample\_011046841, Unigene6343\_Sample\_011046841, Unigene22324\_Sample\_011046841, Unigene56444\_Sample\_011046841, Unigene28357\_Sample\_011046841, Unigene53098\_Sample\_011046841, Unigene43791\_Sample\_011046841, Unigene52573\_Sample\_011046841, Unigene33040\_Sample\_011046841, Unigene47056\_Sample\_011046841, Unigene10202\_Sample\_011046841, Unigene39016\_Sample\_011046841, Unigene49737\_Sample\_011046841, Unigene12853\_Sample\_011046841, Unigene22323\_Sample\_011046841, Unigene21228\_Sample\_011046841, Unigene54096\_Sample\_011046841, Unigene58999\_Sample\_011046841, Unigene47783\_Sample\_011046841, Unigene3070\_Sample\_011046841, Unigene56515\_Sample\_011046841, Unigene60630\_Sample\_011046841, Unigene42872\_Sample\_011046841, Unigene22286\_Sample\_011046841, Unigene50500\_Sample\_011046841, Unigene58955\_Sample\_011046841, Unigene59445\_Sample\_011046841, Unigene60539\_Sample\_011046841, Unigene20854\_Sample\_011046841, Unigene20907\_Sample\_011046841, Unigene9792\_Sample\_011046841, Unigene3316\_Sample\_011046841, Unigene12358\_Sample\_011046841, Unigene56919\_Sample\_011046841, Unigene38510\_Sample\_011046841, Unigene28246\_Sample\_011046841, Unigene23897\_Sample\_011046841, Unigene53605\_Sample\_011046841, Unigene51448\_Sample\_011046841, Unigene54323\_Sample\_011046841, Unigene11932\_Sample\_011046841, Unigene31773\_Sample\_011046841, Unigene58828\_Sample\_011046841, Unigene30162\_Sample\_011046841, Unigene56102\_Sample\_011046841, Unigene26316\_Sample\_011046841, Unigene59907\_Sample\_011046841, Unigene3621\_Sample\_011046841, Unigene27305\_Sample\_011046841, Unigene13887\_Sample\_011046841, Unigene4003\_Sample\_011046841, Unigene37401\_Sample\_011046841, Unigene51958\_Sample\_011046841, Unigene48708\_Sample\_011046841, Unigene54695\_Sample\_011046841, Unigene58416\_Sample\_011046841, Unigene13517\_Sample\_011046841, Unigene11362\_Sample\_011046841, Unigene28964\_Sample\_011046841, Unigene47794\_Sample\_011046841, Unigene17476\_Sample\_011046841, Unigene56944\_Sample\_011046841, Unigene3956\_Sample\_011046841, Unigene54253\_Sample\_011046841, Unigene60892\_Sample\_011046841, Unigene13944\_Sample\_011046841, Unigene19473\_Sample\_011046841, Unigene39753\_Sample\_011046841, Unigene60316\_Sample\_011046841, Unigene49671\_Sample\_011046841, Unigene5898\_Sample\_011046841, Unigene54397\_Sample\_011046841, Unigene55898\_Sample\_011046841, Unigene60178\_Sample\_011046841, Unigene14532\_Sample\_011046841, Unigene23164\_Sample\_011046841, Unigene14914\_Sample\_011046841, Unigene9734\_Sample\_011046841, Unigene25924\_Sample\_011046841, Unigene59904\_Sample\_011046841, Unigene12712\_Sample\_011046841, Unigene45568\_Sample\_011046841, Unigene8429\_Sample\_011046841, Unigene57943\_Sample\_011046841, Unigene60368\_Sample\_011046841, Unigene6000\_Sample\_011046841, Unigene56887\_Sample\_011046841, Unigene54514\_Sample\_011046841, Unigene15482\_Sample\_011046841, Unigene59528\_Sample\_011046841, Unigene59158\_Sample\_011046841, Unigene28737\_Sample\_011046841, Unigene45627\_Sample\_011046841, Unigene5603\_Sample\_011046841, Unigene18126\_Sample\_011046841, Unigene16760\_Sample\_011046841, Unigene45400\_Sample\_011046841, Unigene50951\_Sample\_011046841, Unigene2472\_Sample\_011046841, Unigene50811\_Sample\_011046841, Unigene57322\_Sample\_011046841, Unigene39745\_Sample\_011046841, Unigene42100\_Sample\_011046841, Unigene3808\_Sample\_011046841, Unigene7584\_Sample\_011046841, Unigene24176\_Sample\_011046841, Unigene60919\_Sample\_011046841, Unigene14355\_Sample\_011046841, Unigene59891\_Sample\_011046841, Unigene51090\_Sample\_011046841, Unigene60430\_Sample\_011046841, Unigene10035\_Sample\_011046841, Unigene48337\_Sample\_011046841, Unigene7901\_Sample\_011046841, Unigene40840\_Sample\_011046841, Unigene28390\_Sample\_011046841, Unigene57037\_Sample\_011046841, Unigene19465\_Sample\_011046841, Unigene50584\_Sample\_011046841, Unigene57783\_Sample\_011046841, Unigene56517\_Sample\_011046841, Unigene3431\_Sample\_011046841, Unigene57374\_Sample\_011046841, Unigene27907\_Sample\_011046841, Unigene40059\_Sample\_011046841, Unigene48076\_Sample\_011046841, Unigene54811\_Sample\_011046841, Unigene57417\_Sample\_011046841, Unigene19816\_Sample\_011046841, Unigene54378\_Sample\_011046841, Unigene57992\_Sample\_011046841, Unigene54763\_Sample\_011046841, Unigene37028\_Sample\_011046841, Unigene20559\_Sample\_011046841, Unigene48840\_Sample\_011046841, Unigene59788\_Sample\_011046841, Unigene59284\_Sample\_011046841, Unigene45059\_Sample\_011046841, Unigene60053\_Sample\_011046841, Unigene32169\_Sample\_011046841, Unigene51167\_Sample\_011046841, Unigene58725\_Sample\_011046841, Unigene39083\_Sample\_011046841, Unigene13790\_Sample\_011046841, Unigene45339\_Sample\_011046841, Unigene52769\_Sample\_011046841, Unigene23380\_Sample\_011046841, Unigene28578\_Sample\_011046841, Unigene25827\_Sample\_011046841, Unigene42509\_Sample\_011046841, Unigene24381\_Sample\_011046841, Unigene59010\_Sample\_011046841, Unigene31368\_Sample\_011046841, Unigene44817\_Sample\_011046841, Unigene36278\_Sample\_011046841, Unigene40898\_Sample\_011046841, Unigene29952\_Sample\_011046841, Unigene57560\_Sample\_011046841, Unigene22715\_Sample\_011046841, Unigene13435\_Sample\_011046841, Unigene40729\_Sample\_011046841, Unigene44912\_Sample\_011046841, Unigene21539\_Sample\_011046841, Unigene6469\_Sample\_011046841, Unigene13518\_Sample\_011046841, Unigene48957\_Sample\_011046841, Unigene37947\_Sample\_011046841, Unigene38104\_Sample\_011046841, Unigene52757\_Sample\_011046841, Unigene49669\_Sample\_011046841, Unigene56923\_Sample\_011046841, Unigene55866\_Sample\_011046841, Unigene40727\_Sample\_011046841, Unigene8411\_Sample\_011046841, Unigene54467\_Sample\_011046841, Unigene51952\_Sample\_011046841, Unigene21618\_Sample\_011046841, Unigene44507\_Sample\_011046841, Unigene25027\_Sample\_011046841, Unigene57579\_Sample\_011046841, Unigene31991\_Sample\_011046841, Unigene20596\_Sample\_011046841, Unigene36211\_Sample\_011046841, Unigene9082\_Sample\_011046841, Unigene13916\_Sample\_011046841, Unigene16999\_Sample\_011046841, Unigene5335\_Sample\_011046841, Unigene13694\_Sample\_011046841, Unigene15933\_Sample\_011046841, Unigene36468\_Sample\_011046841, Unigene49082\_Sample\_011046841, Unigene52270\_Sample\_011046841, Unigene16524\_Sample\_011046841, Unigene46207\_Sample\_011046841, Unigene9784\_Sample\_011046841, Unigene58864\_Sample\_011046841, Unigene12209\_Sample\_011046841, Unigene24899\_Sample\_011046841, Unigene54668\_Sample\_011046841, Unigene52375\_Sample\_011046841, Unigene50998\_Sample\_011046841, Unigene33928\_Sample\_011046841, Unigene40559\_Sample\_011046841, Unigene49017\_Sample\_011046841, Unigene52627\_Sample\_011046841, Unigene32195\_Sample\_011046841, Unigene44863\_Sample\_011046841, Unigene31411\_Sample\_011046841, Unigene40263\_Sample\_011046841, Unigene52041\_Sample\_011046841, Unigene37899\_Sample\_011046841, Unigene53449\_Sample\_011046841, Unigene58437\_Sample\_011046841, Unigene33889\_Sample\_011046841, Unigene53995\_Sample\_011046841, Unigene58810\_Sample\_011046841, Unigene24492\_Sample\_011046841, Unigene51706\_Sample\_011046841, Unigene3261\_Sample\_011046841, Unigene56622\_Sample\_011046841, Unigene31181\_Sample\_011046841, Unigene58558\_Sample\_011046841, Unigene17383\_Sample\_011046841, Unigene50089\_Sample\_011046841, Unigene11639\_Sample\_011046841, Unigene19285\_Sample\_011046841, Unigene40754\_Sample\_011046841, Unigene60216\_Sample\_011046841, Unigene46546\_Sample\_011046841, Unigene51481\_Sample\_011046841, Unigene2068\_Sample\_011046841, Unigene43377\_Sample\_011046841, Unigene13068\_Sample\_011046841, Unigene5336\_Sample\_011046841, Unigene52096\_Sample\_011046841, Unigene60597\_Sample\_011046841, Unigene22350\_Sample\_011046841, Unigene14290\_Sample\_011046841, Unigene58907\_Sample\_011046841, Unigene5747\_Sample\_011046841, Unigene60879\_Sample\_011046841, Unigene58199\_Sample\_011046841, Unigene60138\_Sample\_011046841, Unigene18090\_Sample\_011046841, Unigene24424\_Sample\_011046841, Unigene58134\_Sample\_011046841, Unigene53056\_Sample\_011046841, Unigene36180\_Sample\_011046841, Unigene60269\_Sample\_011046841, Unigene53069\_Sample\_011046841, Unigene57949\_Sample\_011046841, Unigene44697\_Sample\_011046841, Unigene60485\_Sample\_011046841, Unigene8647\_Sample\_011046841, Unigene43504\_Sample\_011046841, Unigene38339\_Sample\_011046841, Unigene59949\_Sample\_011046841, Unigene58128\_Sample\_011046841, Unigene60964\_Sample\_011046841, Unigene54638\_Sample\_011046841, Unigene18442\_Sample\_011046841, Unigene59378\_Sample\_011046841, Unigene49955\_Sample\_011046841, Unigene58099\_Sample\_011046841, Unigene4858\_Sample\_011046841, Unigene59614\_Sample\_011046841, Unigene27718\_Sample\_011046841, Unigene30454\_Sample\_011046841, Unigene13151\_Sample\_011046841, Unigene32470\_Sample\_011046841, Unigene58209\_Sample\_011046841, Unigene60189\_Sample\_011046841, Unigene47864\_Sample\_011046841, Unigene33884\_Sample\_011046841, Unigene18245\_Sample\_011046841, Unigene56524\_Sample\_011046841, Unigene12950\_Sample\_011046841, Unigene16974\_Sample\_011046841, Unigene35141\_Sample\_011046841, Unigene57677\_Sample\_011046841, Unigene22016\_Sample\_011046841, Unigene34884\_Sample\_011046841, Unigene58389\_Sample\_011046841, Unigene49906\_Sample\_011046841, Unigene59809\_Sample\_011046841, Unigene38547\_Sample\_011046841, Unigene42731\_Sample\_011046841, Unigene53447\_Sample\_011046841, Unigene18541\_Sample\_011046841, Unigene52396\_Sample\_011046841, Unigene32497\_Sample\_011046841, Unigene30373\_Sample\_011046841, Unigene13697\_Sample\_011046841, Unigene47099\_Sample\_011046841, Unigene60354\_Sample\_011046841, Unigene28640\_Sample\_011046841, Unigene54846\_Sample\_011046841, Unigene9128\_Sample\_011046841, Unigene42200\_Sample\_011046841, Unigene43698\_Sample\_011046841, Unigene30898\_Sample\_011046841, Unigene45395\_Sample\_011046841, Unigene55904\_Sample\_011046841, Unigene16977\_Sample\_011046841, Unigene1273\_Sample\_011046841, Unigene2085\_Sample\_011046841, Unigene51916\_Sample\_011046841, Unigene55588\_Sample\_011046841, Unigene47307\_Sample\_011046841, Unigene56495\_Sample\_011046841, Unigene60902\_Sample\_011046841, Unigene31954\_Sample\_011046841, Unigene29441\_Sample\_011046841, Unigene55076\_Sample\_011046841, Unigene22285\_Sample\_011046841, Unigene19564\_Sample\_011046841, Unigene49192\_Sample\_011046841, Unigene918\_Sample\_011046841, Unigene58697\_Sample\_011046841, Unigene36235\_Sample\_011046841, Unigene49528\_Sample\_011046841, Unigene43895\_Sample\_011046841, Unigene51891\_Sample\_011046841, Unigene4242\_Sample\_011046841, Unigene48342\_Sample\_011046841, Unigene37057\_Sample\_011046841, Unigene34087\_Sample\_011046841, Unigene51909\_Sample\_011046841, Unigene57375\_Sample\_011046841, Unigene10041\_Sample\_011046841, Unigene58890\_Sample\_011046841, Unigene54135\_Sample\_011046841, Unigene9578\_Sample\_011046841, Unigene51307\_Sample\_011046841, Unigene40466\_Sample\_011046841, Unigene60450\_Sample\_011046841, Unigene56870\_Sample\_011046841, Unigene54358\_Sample\_011046841, Unigene41805\_Sample\_011046841, Unigene35480\_Sample\_011046841, Unigene31249\_Sample\_011046841, Unigene44103\_Sample\_011046841, Unigene18110\_Sample\_011046841, Unigene33407\_Sample\_011046841, Unigene35722\_Sample\_011046841, Unigene56779\_Sample\_011046841, Unigene57039\_Sample\_011046841, Unigene13603\_Sample\_011046841, Unigene56168\_Sample\_011046841, Unigene40534\_Sample\_011046841, Unigene9269\_Sample\_011046841, Unigene9271\_Sample\_011046841, Unigene42633\_Sample\_011046841, Unigene8851\_Sample\_011046841, Unigene59486\_Sample\_011046841, Unigene44479\_Sample\_011046841, Unigene58548\_Sample\_011046841, Unigene51115\_Sample\_011046841, Unigene48267\_Sample\_011046841, Unigene13394\_Sample\_011046841, Unigene19044\_Sample\_011046841, Unigene58776\_Sample\_011046841, Unigene53016\_Sample\_011046841, Unigene58354\_Sample\_011046841, Unigene11998\_Sample\_011046841, Unigene35027\_Sample\_011046841, Unigene60540\_Sample\_011046841, Unigene27288\_Sample\_011046841, Unigene24545\_Sample\_011046841, Unigene58224\_Sample\_011046841, Unigene49083\_Sample\_011046841, Unigene2918\_Sample\_011046841, Unigene55511\_Sample\_011046841, Unigene12284\_Sample\_011046841, Unigene1352\_Sample\_011046841, Unigene55856\_Sample\_011046841, Unigene8401\_Sample\_011046841, Unigene54697\_Sample\_011046841, Unigene57757\_Sample\_011046841, Unigene44849\_Sample\_011046841, Unigene5018\_Sample\_011046841, Unigene39667\_Sample\_011046841, Unigene55341\_Sample\_011046841, Unigene54985\_Sample\_011046841, Unigene34152\_Sample\_011046841, Unigene37565\_Sample\_011046841, Unigene13597\_Sample\_011046841, Unigene6203\_Sample\_011046841, Unigene8924\_Sample\_011046841, Unigene43369\_Sample\_011046841, Unigene59021\_Sample\_011046841, Unigene17017\_Sample\_011046841, Unigene55825\_Sample\_011046841, Unigene18163\_Sample\_011046841, Unigene11726\_Sample\_011046841, Unigene49416\_Sample\_011046841, Unigene12947\_Sample\_011046841, Unigene12856\_Sample\_011046841, Unigene59736\_Sample\_011046841, Unigene23331\_Sample\_011046841, Unigene51626\_Sample\_011046841, Unigene43936\_Sample\_011046841, Unigene1526\_Sample\_011046841, Unigene48334\_Sample\_011046841, Unigene11871\_Sample\_011046841, Unigene60725\_Sample\_011046841, Unigene35504\_Sample\_011046841, Unigene13902\_Sample\_011046841, Unigene55781\_Sample\_011046841, Unigene47174\_Sample\_011046841, Unigene54507\_Sample\_011046841, Unigene13329\_Sample\_011046841, Unigene24831\_Sample\_011046841, Unigene55466\_Sample\_011046841, Unigene12549\_Sample\_011046841, Unigene46515\_Sample\_011046841, Unigene10236\_Sample\_011046841, Unigene58821\_Sample\_011046841, Unigene59109\_Sample\_011046841, Unigene33848\_Sample\_011046841, Unigene41813\_Sample\_011046841, Unigene52385\_Sample\_011046841, Unigene55630\_Sample\_011046841, Unigene59914\_Sample\_011046841, Unigene42315\_Sample\_011046841, Unigene56625\_Sample\_011046841, Unigene25753\_Sample\_011046841, Unigene58201\_Sample\_011046841, Unigene36429\_Sample\_011046841, Unigene52532\_Sample\_011046841, Unigene42343\_Sample\_011046841, Unigene59747\_Sample\_011046841, Unigene19113\_Sample\_011046841, Unigene33694\_Sample\_011046841, Unigene2743\_Sample\_011046841, Unigene13669\_Sample\_011046841, Unigene57602\_Sample\_011046841, Unigene3252\_Sample\_011046841, Unigene55131\_Sample\_011046841, Unigene993\_Sample\_011046841, Unigene56391\_Sample\_011046841, Unigene6590\_Sample\_011046841, Unigene12700\_Sample\_011046841, Unigene60847\_Sample\_011046841, Unigene26403\_Sample\_011046841, Unigene10289\_Sample\_011046841, Unigene29803\_Sample\_011046841, Unigene50152\_Sample\_011046841, Unigene57563\_Sample\_011046841, Unigene55930\_Sample\_011046841, Unigene48589\_Sample\_011046841, Unigene44880\_Sample\_011046841, Unigene4364\_Sample\_011046841, Unigene57929\_Sample\_011046841, Unigene55995\_Sample\_011046841, Unigene32630\_Sample\_011046841, Unigene24152\_Sample\_011046841, Unigene57282\_Sample\_011046841, Unigene42559\_Sample\_011046841, Unigene52802\_Sample\_011046841, Unigene39412\_Sample\_011046841, Unigene60958\_Sample\_011046841, Unigene13586\_Sample\_011046841, Unigene56437\_Sample\_011046841, Unigene56027\_Sample\_011046841, Unigene58649\_Sample\_011046841, Unigene60689\_Sample\_011046841, Unigene13367\_Sample\_011046841, Unigene29345\_Sample\_011046841, Unigene22077\_Sample\_011046841, Unigene44769\_Sample\_011046841, Unigene42368\_Sample\_011046841, Unigene9107\_Sample\_011046841, Unigene50660\_Sample\_011046841, Unigene46294\_Sample\_011046841, Unigene27725\_Sample\_011046841, Unigene58238\_Sample\_011046841, Unigene32509\_Sample\_011046841, Unigene12369\_Sample\_011046841, Unigene38310\_Sample\_011046841, Unigene8641\_Sample\_011046841, Unigene26928\_Sample\_011046841, Unigene13775\_Sample\_011046841, Unigene60412\_Sample\_011046841, Unigene48047\_Sample\_011046841, Unigene36036\_Sample\_011046841, Unigene12354\_Sample\_011046841, Unigene4497\_Sample\_011046841, Unigene41236\_Sample\_011046841, Unigene38874\_Sample\_011046841, Unigene60126\_Sample\_011046841, Unigene43906\_Sample\_011046841, Unigene58799\_Sample\_011046841, Unigene30337\_Sample\_011046841, Unigene1748\_Sample\_011046841, Unigene50263\_Sample\_011046841, Unigene45824\_Sample\_011046841, Unigene5317\_Sample\_011046841, Unigene51294\_Sample\_011046841, Unigene60091\_Sample\_011046841, Unigene57255\_Sample\_011046841, Unigene42510\_Sample\_011046841, Unigene53284\_Sample\_011046841, Unigene12072\_Sample\_011046841, Unigene4883\_Sample\_011046841, Unigene4666\_Sample\_011046841, Unigene22290\_Sample\_011046841, Unigene10330\_Sample\_011046841, Unigene54415\_Sample\_011046841, Unigene44703\_Sample\_011046841, Unigene27016\_Sample\_011046841, Unigene48375\_Sample\_011046841, Unigene37440\_Sample\_011046841, Unigene43476\_Sample\_011046841, Unigene38773\_Sample\_011046841, Unigene40921\_Sample\_011046841, Unigene16112\_Sample\_011046841, Unigene55226\_Sample\_011046841, Unigene57716\_Sample\_011046841, Unigene55543\_Sample\_011046841, Unigene34313\_Sample\_011046841, Unigene37696\_Sample\_011046841, Unigene48095\_Sample\_011046841, Unigene44985\_Sample\_011046841, Unigene59478\_Sample\_011046841, Unigene41021\_Sample\_011046841, Unigene56357\_Sample\_011046841, Unigene48727\_Sample\_011046841, Unigene43064\_Sample\_011046841, Unigene54795\_Sample\_011046841, Unigene35210\_Sample\_011046841, Unigene45746\_Sample\_011046841, Unigene45303\_Sample\_011046841, Unigene8670\_Sample\_011046841, Unigene10970\_Sample\_011046841, Unigene21177\_Sample\_011046841, Unigene38143\_Sample\_011046841, Unigene8519\_Sample\_011046841, Unigene59294\_Sample\_011046841, Unigene38688\_Sample\_011046841, Unigene60377\_Sample\_011046841, Unigene40261\_Sample\_011046841, Unigene12507\_Sample\_011046841, Unigene54262\_Sample\_011046841, Unigene16398\_Sample\_011046841, Unigene31264\_Sample\_011046841, Unigene59195\_Sample\_011046841, Unigene34877\_Sample\_011046841, Unigene58924\_Sample\_011046841, Unigene58317\_Sample\_011046841, Unigene56529\_Sample\_011046841, Unigene52276\_Sample\_011046841, Unigene9307\_Sample\_011046841, Unigene29641\_Sample\_011046841, Unigene15136\_Sample\_011046841, Unigene2913\_Sample\_011046841, Unigene27139\_Sample\_011046841, Unigene60700\_Sample\_011046841, Unigene55895\_Sample\_011046841, Unigene7929\_Sample\_011046841, Unigene39238\_Sample\_011046841, Unigene53360\_Sample\_011046841, Unigene18578\_Sample\_011046841, Unigene19717\_Sample\_011046841, Unigene43536\_Sample\_011046841, Unigene43721\_Sample\_011046841, Unigene60486\_Sample\_011046841, Unigene39762\_Sample\_011046841, Unigene57394\_Sample\_011046841, Unigene25597\_Sample\_011046841, Unigene14069\_Sample\_011046841, Unigene11714\_Sample\_011046841, Unigene58715\_Sample\_011046841, Unigene55565\_Sample\_011046841, Unigene12762\_Sample\_011046841, Unigene13287\_Sample\_011046841, Unigene3034\_Sample\_011046841, Unigene47563\_Sample\_011046841, Unigene54860\_Sample\_011046841, Unigene23762\_Sample\_011046841, Unigene59714\_Sample\_011046841, Unigene33118\_Sample\_011046841, Unigene54116\_Sample\_011046841, Unigene6097\_Sample\_011046841, Unigene39539\_Sample\_011046841, Unigene59051\_Sample\_011046841, Unigene55036\_Sample\_011046841, Unigene26777\_Sample\_011046841, Unigene54979\_Sample\_011046841, Unigene29438\_Sample\_011046841, Unigene42893\_Sample\_011046841, Unigene23512\_Sample\_011046841, Unigene35626\_Sample\_011046841, Unigene53496\_Sample\_011046841, Unigene46750\_Sample\_011046841, Unigene57659\_Sample\_011046841, Unigene52997\_Sample\_011046841, Unigene13368\_Sample\_011046841, Unigene57441\_Sample\_011046841, Unigene11794\_Sample\_011046841, Unigene13796\_Sample\_011046841, Unigene22760\_Sample\_011046841, Unigene14248\_Sample\_011046841, Unigene18426\_Sample\_011046841, Unigene57692\_Sample\_011046841, Unigene48524\_Sample\_011046841, Unigene46795\_Sample\_011046841, Unigene12152\_Sample\_011046841, Unigene6207\_Sample\_011046841, Unigene41392\_Sample\_011046841, Unigene60238\_Sample\_011046841, Unigene9615\_Sample\_011046841, Unigene49600\_Sample\_011046841, Unigene60097\_Sample\_011046841, Unigene23590\_Sample\_011046841, Unigene13325\_Sample\_011046841, Unigene3761\_Sample\_011046841, Unigene47759\_Sample\_011046841, Unigene50841\_Sample\_011046841, Unigene59095\_Sample\_011046841, Unigene21520\_Sample\_011046841, Unigene35737\_Sample\_011046841, Unigene47042\_Sample\_011046841, Unigene51984\_Sample\_011046841, Unigene11073\_Sample\_011046841, Unigene7092\_Sample\_011046841, Unigene6235\_Sample\_011046841, Unigene58026\_Sample\_011046841, Unigene40682\_Sample\_011046841, Unigene39991\_Sample\_011046841, Unigene23312\_Sample\_011046841, Unigene38603\_Sample\_011046841, Unigene57266\_Sample\_011046841, Unigene11652\_Sample\_011046841, Unigene58480\_Sample\_011046841, Unigene52877\_Sample\_011046841, Unigene34482\_Sample\_011046841, Unigene56611\_Sample\_011046841, Unigene7143\_Sample\_011046841, Unigene21018\_Sample\_011046841, Unigene60284\_Sample\_011046841, Unigene54829\_Sample\_011046841, Unigene249\_Sample\_011046841, Unigene30267\_Sample\_011046841, Unigene54876\_Sample\_011046841, Unigene57151\_Sample\_011046841, Unigene9618\_Sample\_011046841, Unigene11492\_Sample\_011046841, Unigene49854\_Sample\_011046841, Unigene49622\_Sample\_011046841, Unigene33665\_Sample\_011046841, Unigene56009\_Sample\_011046841, Unigene54443\_Sample\_011046841, Unigene29101\_Sample\_011046841, Unigene16365\_Sample\_011046841, Unigene49010\_Sample\_011046841, Unigene39808\_Sample\_011046841, Unigene8203\_Sample\_011046841, Unigene19418\_Sample\_011046841, Unigene17449\_Sample\_011046841, Unigene36463\_Sample\_011046841, Unigene46446\_Sample\_011046841, Unigene11223\_Sample\_011046841, Unigene57041\_Sample\_011046841, Unigene57158\_Sample\_011046841, Unigene852\_Sample\_011046841, Unigene50677\_Sample\_011046841, Unigene23225\_Sample\_011046841, Unigene9139\_Sample\_011046841, Unigene59441\_Sample\_011046841, Unigene11407\_Sample\_011046841, Unigene36298\_Sample\_011046841, Unigene50495\_Sample\_011046841, Unigene16600\_Sample\_011046841, Unigene33017\_Sample\_011046841, Unigene18301\_Sample\_011046841, Unigene5159\_Sample\_011046841, Unigene59995\_Sample\_011046841, Unigene52832\_Sample\_011046841, Unigene53096\_Sample\_011046841, Unigene56413\_Sample\_011046841, Unigene58397\_Sample\_011046841, Unigene52561\_Sample\_011046841, Unigene34151\_Sample\_011046841, Unigene59672\_Sample\_011046841, Unigene50224\_Sample\_011046841, Unigene54898\_Sample\_011046841, Unigene44148\_Sample\_011046841, Unigene53714\_Sample\_011046841, Unigene56403\_Sample\_011046841, Unigene24335\_Sample\_011046841, Unigene56744\_Sample\_011046841, Unigene55275\_Sample\_011046841, Unigene47370\_Sample\_011046841, Unigene48850\_Sample\_011046841, Unigene5630\_Sample\_011046841, Unigene58082\_Sample\_011046841, Unigene58905\_Sample\_011046841, Unigene6377\_Sample\_011046841, Unigene48701\_Sample\_011046841, Unigene37611\_Sample\_011046841, Unigene41401\_Sample\_011046841, Unigene47036\_Sample\_011046841, Unigene52904\_Sample\_011046841, Unigene51117\_Sample\_011046841, Unigene20302\_Sample\_011046841, Unigene59062\_Sample\_011046841, Unigene50329\_Sample\_011046841, Unigene13794\_Sample\_011046841, Unigene60821\_Sample\_011046841, Unigene24582\_Sample\_011046841, Unigene59003\_Sample\_011046841, Unigene40567\_Sample\_011046841, Unigene2493\_Sample\_011046841, Unigene56615\_Sample\_011046841, Unigene52427\_Sample\_011046841, Unigene57784\_Sample\_011046841, Unigene41644\_Sample\_011046841, Unigene56278\_Sample\_011046841, Unigene22964\_Sample\_011046841, Unigene38078\_Sample\_011046841, Unigene1952\_Sample\_011046841, Unigene5431\_Sample\_011046841, Unigene50025\_Sample\_011046841, Unigene18946\_Sample\_011046841, Unigene60303\_Sample\_011046841, Unigene8175\_Sample\_011046841, Unigene40186\_Sample\_011046841, Unigene48020\_Sample\_011046841, Unigene5783\_Sample\_011046841, Unigene43076\_Sample\_011046841, Unigene6383\_Sample\_011046841, Unigene13636\_Sample\_011046841, Unigene46699\_Sample\_011046841, Unigene43380\_Sample\_011046841, Unigene40209\_Sample\_011046841, Unigene5733\_Sample\_011046841, Unigene49604\_Sample\_011046841, Unigene18785\_Sample\_011046841, Unigene50555\_Sample\_011046841, Unigene23203\_Sample\_011046841, Unigene53817\_Sample\_011046841, Unigene52275\_Sample\_011046841, Unigene15838\_Sample\_011046841, Unigene58018\_Sample\_011046841, Unigene58508\_Sample\_011046841, Unigene31169\_Sample\_011046841, Unigene56163\_Sample\_011046841, Unigene2095\_Sample\_011046841, Unigene56276\_Sample\_011046841, Unigene55314\_Sample\_011046841, Unigene13884\_Sample\_011046841, Unigene58132\_Sample\_011046841, Unigene60712\_Sample\_011046841, Unigene40771\_Sample\_011046841, Unigene13478\_Sample\_011046841, Unigene59362\_Sample\_011046841, Unigene60693\_Sample\_011046841, Unigene50835\_Sample\_011046841, Unigene15479\_Sample\_011046841, Unigene57569\_Sample\_011046841, Unigene60188\_Sample\_011046841, Unigene12530\_Sample\_011046841, Unigene55947\_Sample\_011046841, Unigene11415\_Sample\_011046841, Unigene5154\_Sample\_011046841, Unigene59492\_Sample\_011046841, Unigene54567\_Sample\_011046841, Unigene48996\_Sample\_011046841, Unigene13812\_Sample\_011046841, Unigene11003\_Sample\_011046841, Unigene43840\_Sample\_011046841, Unigene12300\_Sample\_011046841, Unigene2415\_Sample\_011046841, Unigene30570\_Sample\_011046841, Unigene2532\_Sample\_011046841, Unigene39766\_Sample\_011046841, Unigene11029\_Sample\_011046841, Unigene12560\_Sample\_011046841, Unigene52999\_Sample\_011046841, Unigene56100\_Sample\_011046841, Unigene37987\_Sample\_011046841, Unigene30681\_Sample\_011046841, Unigene53420\_Sample\_011046841, Unigene26329\_Sample\_011046841, Unigene10341\_Sample\_011046841, Unigene51179\_Sample\_011046841, Unigene36816\_Sample\_011046841, Unigene58321\_Sample\_011046841, Unigene12670\_Sample\_011046841, Unigene14799\_Sample\_011046841, Unigene59637\_Sample\_011046841, Unigene51802\_Sample\_011046841, Unigene2861\_Sample\_011046841, Unigene6729\_Sample\_011046841, Unigene54407\_Sample\_011046841, Unigene52667\_Sample\_011046841, Unigene56817\_Sample\_011046841, Unigene36788\_Sample\_011046841, Unigene46773\_Sample\_011046841, Unigene44187\_Sample\_011046841, Unigene23723\_Sample\_011046841, Unigene38644\_Sample\_011046841, Unigene42188\_Sample\_011046841, Unigene47569\_Sample\_011046841, Unigene59718\_Sample\_011046841, Unigene47654\_Sample\_011046841, Unigene55564\_Sample\_011046841, Unigene54312\_Sample\_011046841, Unigene7821\_Sample\_011046841, Unigene11667\_Sample\_011046841, Unigene33316\_Sample\_011046841, Unigene56726\_Sample\_011046841, Unigene29219\_Sample\_011046841, Unigene52501\_Sample\_011046841, Unigene57173\_Sample\_011046841, Unigene59560\_Sample\_011046841, Unigene28950\_Sample\_011046841, Unigene32095\_Sample\_011046841, Unigene59575\_Sample\_011046841, Unigene54\_Sample\_011046841, Unigene27178\_Sample\_011046841, Unigene13773\_Sample\_011046841, Unigene49211\_Sample\_011046841, Unigene32596\_Sample\_011046841, Unigene60247\_Sample\_011046841, Unigene59678\_Sample\_011046841, Unigene47421\_Sample\_011046841, Unigene35184\_Sample\_011046841, Unigene21677\_Sample\_011046841, Unigene58137\_Sample\_011046841, Unigene60366\_Sample\_011046841, Unigene15607\_Sample\_011046841, Unigene59826\_Sample\_011046841, Unigene46589\_Sample\_011046841, Unigene56851\_Sample\_011046841, Unigene29462\_Sample\_011046841, Unigene56459\_Sample\_011046841, Unigene59786\_Sample\_011046841, Unigene60207\_Sample\_011046841, Unigene324\_Sample\_011046841, Unigene20298\_Sample\_011046841, Unigene54297\_Sample\_011046841, Unigene35931\_Sample\_011046841, Unigene24814\_Sample\_011046841, Unigene4269\_Sample\_011046841, Unigene13766\_Sample\_011046841, Unigene44504\_Sample\_011046841, Unigene48690\_Sample\_011046841, Unigene36794\_Sample\_011046841, Unigene56464\_Sample\_011046841, Unigene39429\_Sample\_011046841, Unigene54683\_Sample\_011046841, Unigene34209\_Sample\_011046841, Unigene28074\_Sample\_011046841, Unigene57451\_Sample\_011046841, Unigene46030\_Sample\_011046841, Unigene57540\_Sample\_011046841, Unigene41318\_Sample\_011046841, Unigene11465\_Sample\_011046841, Unigene57201\_Sample\_011046841, Unigene58135\_Sample\_011046841, Unigene54884\_Sample\_011046841, Unigene49581\_Sample\_011046841, Unigene30301\_Sample\_011046841, Unigene60044\_Sample\_011046841, Unigene13672\_Sample\_011046841, Unigene55310\_Sample\_011046841, Unigene56531\_Sample\_011046841, Unigene5763\_Sample\_011046841, Unigene7879\_Sample\_011046841, Unigene12261\_Sample\_011046841, Unigene37789\_Sample\_011046841, Unigene60695\_Sample\_011046841, Unigene27839\_Sample\_011046841, Unigene56719\_Sample\_011046841, Unigene49403\_Sample\_011046841, Unigene51716\_Sample\_011046841, Unigene51070\_Sample\_011046841, Unigene17668\_Sample\_011046841, Unigene10517\_Sample\_011046841, Unigene4361\_Sample\_011046841, Unigene20789\_Sample\_011046841, Unigene41373\_Sample\_011046841, Unigene55975\_Sample\_011046841, Unigene59688\_Sample\_011046841, Unigene39712\_Sample\_011046841, Unigene56847\_Sample\_011046841, Unigene46306\_Sample\_011046841, Unigene3740\_Sample\_011046841, Unigene57404\_Sample\_011046841, Unigene52843\_Sample\_011046841, Unigene32802\_Sample\_011046841, Unigene5329\_Sample\_011046841, Unigene25498\_Sample\_011046841, Unigene44391\_Sample\_011046841, Unigene8472\_Sample\_011046841, Unigene43093\_Sample\_011046841, Unigene24028\_Sample\_011046841, Unigene14624\_Sample\_011046841, Unigene50893\_Sample\_011046841, Unigene57832\_Sample\_011046841, Unigene57995\_Sample\_011046841, Unigene56760\_Sample\_011046841, Unigene48881\_Sample\_011046841, Unigene58445\_Sample\_011046841, Unigene57182\_Sample\_011046841, Unigene1750\_Sample\_011046841, Unigene46058\_Sample\_011046841, Unigene40759\_Sample\_011046841, Unigene48357\_Sample\_011046841, Unigene55311\_Sample\_011046841, Unigene58447\_Sample\_011046841, Unigene10567\_Sample\_011046841, Unigene58551\_Sample\_011046841, Unigene33226\_Sample\_011046841, Unigene50325\_Sample\_011046841, Unigene60490\_Sample\_011046841, Unigene60057\_Sample\_011046841, Unigene21838\_Sample\_011046841, Unigene7431\_Sample\_011046841, Unigene18666\_Sample\_011046841, Unigene59220\_Sample\_011046841, Unigene48943\_Sample\_011046841, Unigene37462\_Sample\_011046841, Unigene10522\_Sample\_011046841, Unigene10217\_Sample\_011046841, Unigene27952\_Sample\_011046841, Unigene13722\_Sample\_011046841, Unigene31259\_Sample\_011046841, Unigene8113\_Sample\_011046841, Unigene59664\_Sample\_011046841, Unigene48470\_Sample\_011046841, Unigene39479\_Sample\_011046841, Unigene30413\_Sample\_011046841, Unigene38051\_Sample\_011046841, Unigene6254\_Sample\_011046841, Unigene51301\_Sample\_011046841, Unigene21986\_Sample\_011046841, Unigene51852\_Sample\_011046841, Unigene57966\_Sample\_011046841, Unigene4853\_Sample\_011046841, Unigene60629\_Sample\_011046841, Unigene50414\_Sample\_011046841, Unigene52014\_Sample\_011046841, Unigene33439\_Sample\_011046841, Unigene38549\_Sample\_011046841, Unigene57909\_Sample\_011046841, Unigene57889\_Sample\_011046841, Unigene60766\_Sample\_011046841, Unigene5311\_Sample\_011046841, Unigene28563\_Sample\_011046841, Unigene36791\_Sample\_011046841, Unigene24841\_Sample\_011046841, Unigene52522\_Sample\_011046841, Unigene51468\_Sample\_011046841, Unigene60770\_Sample\_011046841, Unigene3810\_Sample\_011046841, Unigene42880\_Sample\_011046841, Unigene52731\_Sample\_011046841, Unigene12521\_Sample\_011046841, Unigene40554\_Sample\_011046841, Unigene44600\_Sample\_011046841, Unigene16413\_Sample\_011046841, Unigene53267\_Sample\_011046841, Unigene58494\_Sample\_011046841, Unigene60197\_Sample\_011046841, Unigene11419\_Sample\_011046841, Unigene43623\_Sample\_011046841, Unigene30743\_Sample\_011046841, Unigene56518\_Sample\_011046841, Unigene57318\_Sample\_011046841, Unigene60455\_Sample\_011046841, Unigene59228\_Sample\_011046841, Unigene55647\_Sample\_011046841, Unigene25029\_Sample\_011046841, Unigene37042\_Sample\_011046841, Unigene59558\_Sample\_011046841, Unigene58021\_Sample\_011046841, Unigene1194\_Sample\_011046841, Unigene60810\_Sample\_011046841, Unigene50393\_Sample\_011046841, Unigene37467\_Sample\_011046841, Unigene50434\_Sample\_011046841, Unigene46176\_Sample\_011046841, Unigene57009\_Sample\_011046841, Unigene59999\_Sample\_011046841, Unigene32760\_Sample\_011046841, Unigene52381\_Sample\_011046841, Unigene54902\_Sample\_011046841, Unigene35285\_Sample\_011046841, Unigene50926\_Sample\_011046841, Unigene55715\_Sample\_011046841, Unigene13817\_Sample\_011046841, Unigene19290\_Sample\_011046841, Unigene9254\_Sample\_011046841, Unigene37367\_Sample\_011046841, Unigene45740\_Sample\_011046841, Unigene58229\_Sample\_011046841, Unigene56904\_Sample\_011046841, Unigene21863\_Sample\_011046841, Unigene1793\_Sample\_011046841, Unigene18699\_Sample\_011046841, Unigene29113\_Sample\_011046841, Unigene60454\_Sample\_011046841, Unigene24733\_Sample\_011046841, Unigene40306\_Sample\_011046841, Unigene27012\_Sample\_011046841, Unigene42480\_Sample\_011046841, Unigene47956\_Sample\_011046841, Unigene5237\_Sample\_011046841, Unigene60435\_Sample\_011046841, Unigene45183\_Sample\_011046841, Unigene5491\_Sample\_011046841, Unigene1520\_Sample\_011046841, Unigene19438\_Sample\_011046841, Unigene46800\_Sample\_011046841, Unigene21780\_Sample\_011046841, Unigene53131\_Sample\_011046841, Unigene59161\_Sample\_011046841, Unigene60780\_Sample\_011046841, Unigene52788\_Sample\_011046841, Unigene52976\_Sample\_011046841, Unigene24438\_Sample\_011046841, Unigene1671\_Sample\_011046841, Unigene36363\_Sample\_011046841, Unigene54081\_Sample\_011046841, Unigene53773\_Sample\_011046841, Unigene51956\_Sample\_011046841, Unigene51575\_Sample\_011046841, Unigene60515\_Sample\_011046841, Unigene10669\_Sample\_011046841, Unigene45188\_Sample\_011046841, Unigene3342\_Sample\_011046841, Unigene12692\_Sample\_011046841, Unigene38675\_Sample\_011046841, Unigene59917\_Sample\_011046841, Unigene466\_Sample\_011046841, Unigene50644\_Sample\_011046841, Unigene13852\_Sample\_011046841, Unigene23479\_Sample\_011046841, Unigene59496\_Sample\_011046841, Unigene50300\_Sample\_011046841, Unigene51295\_Sample\_011046841, Unigene30309\_Sample\_011046841, Unigene12611\_Sample\_011046841, Unigene50483\_Sample\_011046841, Unigene56930\_Sample\_011046841, Unigene56439\_Sample\_011046841, Unigene9769\_Sample\_011046841, Unigene58014\_Sample\_011046841, Unigene51699\_Sample\_011046841, Unigene22482\_Sample\_011046841, Unigene57572\_Sample\_011046841, Unigene60130\_Sample\_011046841, Unigene38616\_Sample\_011046841, Unigene48044\_Sample\_011046841, Unigene39338\_Sample\_011046841, Unigene9865\_Sample\_011046841, Unigene32987\_Sample\_011046841, Unigene47966\_Sample\_011046841, Unigene55461\_Sample\_011046841, Unigene24611\_Sample\_011046841, Unigene36330\_Sample\_011046841, Unigene39577\_Sample\_011046841, Unigene50073\_Sample\_011046841, Unigene48737\_Sample\_011046841, Unigene24060\_Sample\_011046841, Unigene51756\_Sample\_011046841, Unigene15159\_Sample\_011046841, Unigene59548\_Sample\_011046841, Unigene59366\_Sample\_011046841, Unigene19103\_Sample\_011046841, Unigene37707\_Sample\_011046841, Unigene59556\_Sample\_011046841, Unigene22782\_Sample\_011046841, Unigene51978\_Sample\_011046841, Unigene57000\_Sample\_011046841, Unigene60718\_Sample\_011046841, Unigene35470\_Sample\_011046841, Unigene3890\_Sample\_011046841, Unigene4889\_Sample\_011046841, Unigene47610\_Sample\_011046841, Unigene57656\_Sample\_011046841, Unigene60764\_Sample\_011046841, Unigene18509\_Sample\_011046841, Unigene26464\_Sample\_011046841, Unigene60328\_Sample\_011046841, Unigene1887\_Sample\_011046841, Unigene60652\_Sample\_011046841, Unigene48499\_Sample\_011046841, Unigene55850\_Sample\_011046841, Unigene19604\_Sample\_011046841, Unigene59514\_Sample\_011046841, Unigene40878\_Sample\_011046841, Unigene10948\_Sample\_011046841, Unigene35733\_Sample\_011046841, Unigene57051\_Sample\_011046841, Unigene23389\_Sample\_011046841, Unigene57712\_Sample\_011046841, Unigene47149\_Sample\_011046841, Unigene40215\_Sample\_011046841, Unigene986\_Sample\_011046841, Unigene44175\_Sample\_011046841, Unigene47910\_Sample\_011046841, Unigene35808\_Sample\_011046841, Unigene58831\_Sample\_011046841, Unigene53097\_Sample\_011046841, Unigene53816\_Sample\_011046841, Unigene49145\_Sample\_011046841, Unigene5231\_Sample\_011046841, Unigene12487\_Sample\_011046841, Unigene52695\_Sample\_011046841, Unigene59439\_Sample\_011046841, Unigene10745\_Sample\_011046841, Unigene5816\_Sample\_011046841, Unigene28763\_Sample\_011046841, Unigene24332\_Sample\_011046841, Unigene29109\_Sample\_011046841, Unigene57306\_Sample\_011046841, Unigene7647\_Sample\_011046841, Unigene28880\_Sample\_011046841, Unigene60916\_Sample\_011046841, Unigene31129\_Sample\_011046841, Unigene10881\_Sample\_011046841, Unigene24046\_Sample\_011046841, Unigene54198\_Sample\_011046841, Unigene30595\_Sample\_011046841, Unigene58518\_Sample\_011046841, Unigene9259\_Sample\_011046841, Unigene9774\_Sample\_011046841, Unigene54989\_Sample\_011046841, Unigene9076\_Sample\_011046841, Unigene34027\_Sample\_011046841, Unigene58031\_Sample\_011046841, Unigene27200\_Sample\_011046841, Unigene58444\_Sample\_011046841, Unigene27457\_Sample\_011046841, Unigene60839\_Sample\_011046841, Unigene59673\_Sample\_011046841, Unigene13223\_Sample\_011046841, Unigene59814\_Sample\_011046841, Unigene58919\_Sample\_011046841, Unigene51736\_Sample\_011046841, Unigene9064\_Sample\_011046841, Unigene49874\_Sample\_011046841, Unigene58895\_Sample\_011046841, Unigene39240\_Sample\_011046841, Unigene37063\_Sample\_011046841, Unigene15953\_Sample\_011046841, Unigene5730\_Sample\_011046841, Unigene56913\_Sample\_011046841, Unigene58049\_Sample\_011046841, Unigene57305\_Sample\_011046841, Unigene36679\_Sample\_011046841, Unigene41178\_Sample\_011046841, Unigene45416\_Sample\_011046841, Unigene26069\_Sample\_011046841, Unigene59588\_Sample\_011046841, Unigene16046\_Sample\_011046841, Unigene41057\_Sample\_011046841, Unigene50662\_Sample\_011046841, Unigene15991\_Sample\_011046841, Unigene44047\_Sample\_011046841, Unigene57188\_Sample\_011046841, Unigene49399\_Sample\_011046841, Unigene55425\_Sample\_011046841, Unigene59634\_Sample\_011046841, Unigene10283\_Sample\_011046841, Unigene17090\_Sample\_011046841, Unigene60859\_Sample\_011046841, Unigene43009\_Sample\_011046841, Unigene56807\_Sample\_011046841, Unigene21855\_Sample\_011046841, Unigene58451\_Sample\_011046841, Unigene42565\_Sample\_011046841, Unigene36528\_Sample\_011046841, Unigene4741\_Sample\_011046841, Unigene13774\_Sample\_011046841, Unigene51130\_Sample\_011046841, Unigene60924\_Sample\_011046841, Unigene8197\_Sample\_011046841, Unigene57095\_Sample\_011046841, Unigene7102\_Sample\_011046841, Unigene2442\_Sample\_011046841, Unigene10260\_Sample\_011046841, Unigene13541\_Sample\_011046841, Unigene11754\_Sample\_011046841, Unigene11034\_Sample\_011046841, Unigene54333\_Sample\_011046841, Unigene4303\_Sample\_011046841, Unigene19517\_Sample\_011046841, Unigene41700\_Sample\_011046841, Unigene56661\_Sample\_011046841, Unigene50062\_Sample\_011046841, Unigene55044\_Sample\_011046841, Unigene33803\_Sample\_011046841, Unigene46625\_Sample\_011046841, Unigene32116\_Sample\_011046841, Unigene44926\_Sample\_011046841, Unigene13640\_Sample\_011046841, Unigene28131\_Sample\_011046841, Unigene41591\_Sample\_011046841, Unigene21582\_Sample\_011046841, Unigene56545\_Sample\_011046841, Unigene21225\_Sample\_011046841, Unigene4256\_Sample\_011046841, Unigene47723\_Sample\_011046841, Unigene57921\_Sample\_011046841, Unigene55961\_Sample\_011046841, Unigene24586\_Sample\_011046841, Unigene51582\_Sample\_011046841, Unigene38646\_Sample\_011046841, Unigene56214\_Sample\_011046841, Unigene58293\_Sample\_011046841, Unigene50963\_Sample\_011046841, Unigene20439\_Sample\_011046841, Unigene58942\_Sample\_011046841, Unigene16520\_Sample\_011046841, Unigene50131\_Sample\_011046841, Unigene60129\_Sample\_011046841, Unigene55126\_Sample\_011046841, Unigene52274\_Sample\_011046841, Unigene35987\_Sample\_011046841, Unigene20715\_Sample\_011046841, Unigene10875\_Sample\_011046841, Unigene23831\_Sample\_011046841, Unigene41574\_Sample\_011046841, Unigene8428\_Sample\_011046841, Unigene54061\_Sample\_011046841, Unigene58858\_Sample\_011046841, Unigene47050\_Sample\_011046841, Unigene6238\_Sample\_011046841, Unigene4662\_Sample\_011046841, Unigene56251\_Sample\_011046841, Unigene45512\_Sample\_011046841, Unigene21268\_Sample\_011046841, Unigene16001\_Sample\_011046841, Unigene50220\_Sample\_011046841, Unigene34589\_Sample\_011046841, Unigene13224\_Sample\_011046841, Unigene57170\_Sample\_011046841, Unigene54803\_Sample\_011046841, Unigene42051\_Sample\_011046841, Unigene47634\_Sample\_011046841, Unigene23742\_Sample\_011046841, Unigene55696\_Sample\_011046841, Unigene11000\_Sample\_011046841, Unigene49932\_Sample\_011046841, Unigene59365\_Sample\_011046841, Unigene16146\_Sample\_011046841, Unigene59822\_Sample\_011046841, Unigene37381\_Sample\_011046841, Unigene15650\_Sample\_011046841, Unigene41778\_Sample\_011046841, Unigene24385\_Sample\_011046841, Unigene40758\_Sample\_011046841, Unigene57265\_Sample\_011046841, Unigene59738\_Sample\_011046841, Unigene5195\_Sample\_011046841, Unigene34961\_Sample\_011046841, Unigene58685\_Sample\_011046841, Unigene60680\_Sample\_011046841, Unigene43028\_Sample\_011046841, Unigene58727\_Sample\_011046841, Unigene25267\_Sample\_011046841, Unigene19413\_Sample\_011046841, Unigene14396\_Sample\_011046841, Unigene22357\_Sample\_011046841, Unigene3167\_Sample\_011046841, Unigene5203\_Sample\_011046841, Unigene11464\_Sample\_011046841, Unigene57096\_Sample\_011046841, Unigene41848\_Sample\_011046841, Unigene7366\_Sample\_011046841, Unigene47\_Sample\_011046841, Unigene13881\_Sample\_011046841, Unigene59985\_Sample\_011046841, Unigene4416\_Sample\_011046841, Unigene7559\_Sample\_011046841, Unigene7778\_Sample\_011046841, Unigene46703\_Sample\_011046841, Unigene60376\_Sample\_011046841, Unigene25098\_Sample\_011046841, Unigene59397\_Sample\_011046841, Unigene50911\_Sample\_011046841, Unigene56769\_Sample\_011046841, Unigene32138\_Sample\_011046841, Unigene4847\_Sample\_011046841, Unigene50197\_Sample\_011046841, Unigene5220\_Sample\_011046841, Unigene60139\_Sample\_011046841, Unigene47967\_Sample\_011046841, Unigene56234\_Sample\_011046841, Unigene44889\_Sample\_011046841, Unigene21150\_Sample\_011046841, Unigene52031\_Sample\_011046841, Unigene24221\_Sample\_011046841, Unigene27727\_Sample\_011046841, Unigene8426\_Sample\_011046841, Unigene48688\_Sample\_011046841, Unigene57384\_Sample\_011046841, Unigene60567\_Sample\_011046841, Unigene3269\_Sample\_011046841, Unigene55248\_Sample\_011046841, Unigene47067\_Sample\_011046841, Unigene37614\_Sample\_011046841, Unigene60444\_Sample\_011046841, Unigene46340\_Sample\_011046841, Unigene47804\_Sample\_011046841, Unigene44249\_Sample\_011046841, Unigene5099\_Sample\_011046841, Unigene7340\_Sample\_011046841, Unigene41062\_Sample\_011046841, Unigene5805\_Sample\_011046841, Unigene36922\_Sample\_011046841, Unigene46697\_Sample\_011046841, Unigene37679\_Sample\_011046841, Unigene11028\_Sample\_011046841, Unigene42967\_Sample\_011046841, Unigene53100\_Sample\_011046841, Unigene7908\_Sample\_011046841, Unigene46256\_Sample\_011046841, Unigene54425\_Sample\_011046841, Unigene19480\_Sample\_011046841, Unigene50585\_Sample\_011046841, Unigene48540\_Sample\_011046841, Unigene60049\_Sample\_011046841, Unigene3875\_Sample\_011046841, Unigene48383\_Sample\_011046841, Unigene53884\_Sample\_011046841, Unigene30787\_Sample\_011046841, Unigene244\_Sample\_011046841, Unigene13585\_Sample\_011046841, Unigene49264\_Sample\_011046841, Unigene47023\_Sample\_011046841, Unigene38784\_Sample\_011046841, Unigene60311\_Sample\_011046841, Unigene10336\_Sample\_011046841, Unigene49107\_Sample\_011046841, Unigene13969\_Sample\_011046841, Unigene54276\_Sample\_011046841, Unigene13571\_Sample\_011046841, Unigene59671\_Sample\_011046841, Unigene57189\_Sample\_011046841, Unigene13651\_Sample\_011046841, Unigene48637\_Sample\_011046841, Unigene12269\_Sample\_011046841, Unigene38947\_Sample\_011046841, Unigene57795\_Sample\_011046841, Unigene5104\_Sample\_011046841, Unigene21907\_Sample\_011046841, Unigene45510\_Sample\_011046841, Unigene19495\_Sample\_011046841, Unigene55252\_Sample\_011046841, Unigene34380\_Sample\_011046841, Unigene24508\_Sample\_011046841, Unigene60101\_Sample\_011046841, Unigene34472\_Sample\_011046841, Unigene46495\_Sample\_011046841, Unigene50134\_Sample\_011046841, Unigene8392\_Sample\_011046841, Unigene45342\_Sample\_011046841, Unigene30385\_Sample\_011046841, Unigene56803\_Sample\_011046841, Unigene59309\_Sample\_011046841, Unigene54751\_Sample\_011046841, Unigene60244\_Sample\_011046841, Unigene53757\_Sample\_011046841, Unigene11065\_Sample\_011046841, Unigene11233\_Sample\_011046841, Unigene27464\_Sample\_011046841, Unigene48081\_Sample\_011046841, Unigene58077\_Sample\_011046841, Unigene10102\_Sample\_011046841, Unigene57339\_Sample\_011046841, Unigene60618\_Sample\_011046841, Unigene45211\_Sample\_011046841, Unigene40807\_Sample\_011046841, Unigene58839\_Sample\_011046841, Unigene53327\_Sample\_011046841, Unigene3901\_Sample\_011046841, Unigene51282\_Sample\_011046841, Unigene47820\_Sample\_011046841, Unigene31664\_Sample\_011046841, Unigene31498\_Sample\_011046841, Unigene51696\_Sample\_011046841, Unigene56665\_Sample\_011046841, Unigene12056\_Sample\_011046841, Unigene44628\_Sample\_011046841, Unigene13514\_Sample\_011046841, Unigene15776\_Sample\_011046841, Unigene60434\_Sample\_011046841, Unigene58917\_Sample\_011046841, Unigene33111\_Sample\_011046841, Unigene12833\_Sample\_011046841, Unigene30632\_Sample\_011046841, Unigene56897\_Sample\_011046841, Unigene57312\_Sample\_011046841, Unigene42430\_Sample\_011046841, Unigene49022\_Sample\_011046841, Unigene56566\_Sample\_011046841, Unigene10934\_Sample\_011046841, Unigene50618\_Sample\_011046841, Unigene6489\_Sample\_011046841, Unigene50085\_Sample\_011046841, Unigene60671\_Sample\_011046841, Unigene44562\_Sample\_011046841, Unigene5972\_Sample\_011046841, Unigene22762\_Sample\_011046841, Unigene44929\_Sample\_011046841, Unigene36865\_Sample\_011046841, Unigene21688\_Sample\_011046841, Unigene29131\_Sample\_011046841, Unigene52744\_Sample\_011046841, Unigene19047\_Sample\_011046841, Unigene52689\_Sample\_011046841, Unigene34410\_Sample\_011046841, Unigene10201\_Sample\_011046841, Unigene47313\_Sample\_011046841, Unigene58436\_Sample\_011046841, Unigene5904\_Sample\_011046841, Unigene50475\_Sample\_011046841, Unigene46541\_Sample\_011046841, Unigene28804\_Sample\_011046841, Unigene22255\_Sample\_011046841, Unigene49733\_Sample\_011046841, Unigene57475\_Sample\_011046841, Unigene17219\_Sample\_011046841, Unigene59399\_Sample\_011046841, Unigene41466\_Sample\_011046841, Unigene57410\_Sample\_011046841, Unigene60625\_Sample\_011046841, Unigene51024\_Sample\_011046841, Unigene39200\_Sample\_011046841, Unigene11951\_Sample\_011046841, Unigene32476\_Sample\_011046841, Unigene46973\_Sample\_011046841, Unigene44347\_Sample\_011046841, Unigene48526\_Sample\_011046841, Unigene11787\_Sample\_011046841, Unigene47140\_Sample\_011046841, Unigene20770\_Sample\_011046841, Unigene34605\_Sample\_011046841, Unigene56339\_Sample\_011046841, Unigene23602\_Sample\_011046841, Unigene10285\_Sample\_011046841, Unigene49615\_Sample\_011046841, Unigene47257\_Sample\_011046841, Unigene46603\_Sample\_011046841, Unigene57531\_Sample\_011046841, Unigene10485\_Sample\_011046841, Unigene15292\_Sample\_011046841, Unigene28636\_Sample\_011046841, Unigene3946\_Sample\_011046841, Unigene1012\_Sample\_011046841, Unigene19594\_Sample\_011046841, Unigene48645\_Sample\_011046841, Unigene57847\_Sample\_011046841, Unigene28864\_Sample\_011046841, Unigene2226\_Sample\_011046841, Unigene13675\_Sample\_011046841, Unigene39209\_Sample\_011046841, Unigene12324\_Sample\_011046841, Unigene51677\_Sample\_011046841, Unigene3030\_Sample\_011046841, Unigene50440\_Sample\_011046841, Unigene22022\_Sample\_011046841, Unigene26904\_Sample\_011046841, Unigene28403\_Sample\_011046841, Unigene29038\_Sample\_011046841, Unigene48094\_Sample\_011046841, Unigene58947\_Sample\_011046841, Unigene30764\_Sample\_011046841, Unigene32242\_Sample\_011046841, Unigene60871\_Sample\_011046841, Unigene59408\_Sample\_011046841, Unigene49574\_Sample\_011046841, Unigene26856\_Sample\_011046841, Unigene5953\_Sample\_011046841, Unigene54870\_Sample\_011046841, Unigene13955\_Sample\_011046841, Unigene54375\_Sample\_011046841, Unigene9898\_Sample\_011046841, Unigene55957\_Sample\_011046841, Unigene11520\_Sample\_011046841, Unigene55144\_Sample\_011046841, Unigene58475\_Sample\_011046841, Unigene60441\_Sample\_011046841, Unigene56714\_Sample\_011046841, Unigene37289\_Sample\_011046841, Unigene32703\_Sample\_011046841, Unigene46290\_Sample\_011046841, Unigene13106\_Sample\_011046841, Unigene8489\_Sample\_011046841, Unigene57766\_Sample\_011046841, Unigene53706\_Sample\_011046841, Unigene13620\_Sample\_011046841, Unigene41999\_Sample\_011046841, Unigene53297\_Sample\_011046841, Unigene28184\_Sample\_011046841, Unigene53993\_Sample\_011046841, Unigene12134\_Sample\_011046841, Unigene40575\_Sample\_011046841, Unigene53262\_Sample\_011046841, Unigene8158\_Sample\_011046841, Unigene757\_Sample\_011046841, Unigene60285\_Sample\_011046841, Unigene60226\_Sample\_011046841, Unigene31969\_Sample\_011046841, Unigene46764\_Sample\_011046841, Unigene11741\_Sample\_011046841, Unigene6720\_Sample\_011046841, Unigene13245\_Sample\_011046841, Unigene39074\_Sample\_011046841, Unigene58432\_Sample\_011046841, Unigene7024\_Sample\_011046841, Unigene58663\_Sample\_011046841, Unigene59599\_Sample\_011046841, Unigene34039\_Sample\_011046841, Unigene6601\_Sample\_011046841, Unigene1281\_Sample\_011046841, Unigene247\_Sample\_011046841, Unigene28937\_Sample\_011046841, Unigene20448\_Sample\_011046841, Unigene42954\_Sample\_011046841, Unigene32707\_Sample\_011046841, Unigene24930\_Sample\_011046841, Unigene967\_Sample\_011046841, Unigene35806\_Sample\_011046841, Unigene52590\_Sample\_011046841, Unigene58550\_Sample\_011046841, Unigene6209\_Sample\_011046841, Unigene60698\_Sample\_011046841, Unigene57811\_Sample\_011046841, Unigene58606\_Sample\_011046841, Unigene31713\_Sample\_011046841, Unigene32119\_Sample\_011046841, Unigene21731\_Sample\_011046841, Unigene58513\_Sample\_011046841, Unigene55818\_Sample\_011046841, Unigene17678\_Sample\_011046841, Unigene47780\_Sample\_011046841, Unigene15868\_Sample\_011046841, Unigene32418\_Sample\_011046841, Unigene246\_Sample\_011046841, Unigene11729\_Sample\_011046841, Unigene58422\_Sample\_011046841, Unigene12445\_Sample\_011046841, Unigene40142\_Sample\_011046841, Unigene37665\_Sample\_011046841, Unigene59452\_Sample\_011046841, Unigene25101\_Sample\_011046841, Unigene34643\_Sample\_011046841, Unigene20390\_Sample\_011046841, Unigene43620\_Sample\_011046841, Unigene38329\_Sample\_011046841, Unigene1662\_Sample\_011046841, Unigene7752\_Sample\_011046841, Unigene13841\_Sample\_011046841, Unigene59259\_Sample\_011046841, Unigene42127\_Sample\_011046841, Unigene19937\_Sample\_011046841, Unigene7339\_Sample\_011046841, Unigene24751\_Sample\_011046841, Unigene44086\_Sample\_011046841, Unigene58104\_Sample\_011046841, Unigene56547\_Sample\_011046841, Unigene60557\_Sample\_011046841, Unigene7900\_Sample\_011046841, Unigene30720\_Sample\_011046841, Unigene5122\_Sample\_011046841, Unigene8906\_Sample\_011046841, Unigene56822\_Sample\_011046841, Unigene13044\_Sample\_011046841, Unigene52010\_Sample\_011046841, Unigene10982\_Sample\_011046841, Unigene50994\_Sample\_011046841, Unigene25357\_Sample\_011046841, Unigene52079\_Sample\_011046841, Unigene52621\_Sample\_011046841, Unigene13473\_Sample\_011046841, Unigene13890\_Sample\_011046841, Unigene52007\_Sample\_011046841, Unigene47083\_Sample\_011046841, Unigene51221\_Sample\_011046841, Unigene50664\_Sample\_011046841, Unigene52174\_Sample\_011046841, Unigene54965\_Sample\_011046841, Unigene34149\_Sample\_011046841, Unigene20440\_Sample\_011046841, Unigene54139\_Sample\_011046841, Unigene7359\_Sample\_011046841, Unigene47887\_Sample\_011046841, Unigene36181\_Sample\_011046841, Unigene51573\_Sample\_011046841, Unigene5737\_Sample\_011046841, Unigene60248\_Sample\_011046841, Unigene13819\_Sample\_011046841, Unigene6836\_Sample\_011046841, Unigene16539\_Sample\_011046841, Unigene56182\_Sample\_011046841, Unigene52542\_Sample\_011046841, Unigene50842\_Sample\_011046841, Unigene23857\_Sample\_011046841, Unigene56119\_Sample\_011046841, Unigene19018\_Sample\_011046841, Unigene54197\_Sample\_011046841, Unigene15374\_Sample\_011046841, Unigene44813\_Sample\_011046841, Unigene52306\_Sample\_011046841, Unigene34859\_Sample\_011046841, Unigene54368\_Sample\_011046841, Unigene33661\_Sample\_011046841, Unigene52838\_Sample\_011046841, Unigene54629\_Sample\_011046841, Unigene2204\_Sample\_011046841, Unigene11547\_Sample\_011046841, Unigene31394\_Sample\_011046841, Unigene37797\_Sample\_011046841, Unigene28376\_Sample\_011046841, Unigene56629\_Sample\_011046841, Unigene35836\_Sample\_011046841, Unigene22907\_Sample\_011046841, Unigene27694\_Sample\_011046841, Unigene48755\_Sample\_011046841, Unigene55391\_Sample\_011046841, Unigene3618\_Sample\_011046841, Unigene13901\_Sample\_011046841, Unigene45316\_Sample\_011046841, Unigene6091\_Sample\_011046841, Unigene11546\_Sample\_011046841, Unigene29816\_Sample\_011046841, Unigene60399\_Sample\_011046841, Unigene37132\_Sample\_011046841, Unigene37984\_Sample\_011046841, Unigene33286\_Sample\_011046841, Unigene33437\_Sample\_011046841, Unigene27092\_Sample\_011046841, Unigene58645\_Sample\_011046841, Unigene13197\_Sample\_011046841, Unigene60855\_Sample\_011046841, Unigene59527\_Sample\_011046841, Unigene18257\_Sample\_011046841, Unigene59606\_Sample\_011046841, Unigene41220\_Sample\_011046841, Unigene45262\_Sample\_011046841, Unigene42911\_Sample\_011046841, Unigene13430\_Sample\_011046841, Unigene19293\_Sample\_011046841, Unigene22154\_Sample\_011046841, Unigene12345\_Sample\_011046841, Unigene49501\_Sample\_011046841, Unigene57251\_Sample\_011046841, Unigene59987\_Sample\_011046841, Unigene49063\_Sample\_011046841, Unigene12331\_Sample\_011046841, Unigene54549\_Sample\_011046841, Unigene52751\_Sample\_011046841, Unigene49426\_Sample\_011046841, Unigene33456\_Sample\_011046841, Unigene45945\_Sample\_011046841, Unigene48232\_Sample\_011046841, Unigene46287\_Sample\_011046841, Unigene12975\_Sample\_011046841, Unigene57997\_Sample\_011046841, Unigene45004\_Sample\_011046841, Unigene50288\_Sample\_011046841, Unigene53571\_Sample\_011046841, Unigene10744\_Sample\_011046841, Unigene19437\_Sample\_011046841, Unigene60594\_Sample\_011046841, Unigene49126\_Sample\_011046841, Unigene60425\_Sample\_011046841, Unigene24944\_Sample\_011046841, Unigene39983\_Sample\_011046841, Unigene47725\_Sample\_011046841, Unigene29396\_Sample\_011046841, Unigene47199\_Sample\_011046841, Unigene41845\_Sample\_011046841, Unigene16307\_Sample\_011046841, Unigene56795\_Sample\_011046841, Unigene59151\_Sample\_011046841, Unigene59818\_Sample\_011046841, Unigene58960\_Sample\_011046841, Unigene59705\_Sample\_011046841, Unigene54037\_Sample\_011046841, Unigene58675\_Sample\_011046841, Unigene51763\_Sample\_011046841, Unigene57057\_Sample\_011046841, Unigene60779\_Sample\_011046841, Unigene13920\_Sample\_011046841, Unigene59656\_Sample\_011046841, Unigene31656\_Sample\_011046841, Unigene28148\_Sample\_011046841, Unigene47795\_Sample\_011046841, Unigene53587\_Sample\_011046841, Unigene26577\_Sample\_011046841, Unigene42580\_Sample\_011046841, Unigene13712\_Sample\_011046841, Unigene9971\_Sample\_011046841, Unigene5888\_Sample\_011046841, Unigene34330\_Sample\_011046841, Unigene49650\_Sample\_011046841, Unigene56203\_Sample\_011046841, Unigene43420\_Sample\_011046841, Unigene53018\_Sample\_011046841, Unigene13860\_Sample\_011046841, Unigene54121\_Sample\_011046841, Unigene37321\_Sample\_011046841, Unigene33297\_Sample\_011046841, Unigene58341\_Sample\_011046841, Unigene17436\_Sample\_011046841, Unigene57225\_Sample\_011046841, Unigene37228\_Sample\_011046841, Unigene12306\_Sample\_011046841, Unigene55517\_Sample\_011046841, Unigene46240\_Sample\_011046841, Unigene48922\_Sample\_011046841, Unigene43730\_Sample\_011046841, Unigene30989\_Sample\_011046841, Unigene59137\_Sample\_011046841, Unigene53718\_Sample\_011046841, Unigene60292\_Sample\_011046841, Unigene6265\_Sample\_011046841, Unigene48412\_Sample\_011046841, Unigene56081\_Sample\_011046841, Unigene36705\_Sample\_011046841, Unigene60036\_Sample\_011046841, Unigene51163\_Sample\_011046841, Unigene2933\_Sample\_011046841, Unigene59619\_Sample\_011046841, Unigene7507\_Sample\_011046841, Unigene56356\_Sample\_011046841, Unigene30654\_Sample\_011046841, Unigene59539\_Sample\_011046841, Unigene51416\_Sample\_011046841, Unigene60488\_Sample\_011046841, Unigene24566\_Sample\_011046841, Unigene21230\_Sample\_011046841, Unigene27108\_Sample\_011046841, Unigene55302\_Sample\_011046841, Unigene47585\_Sample\_011046841, Unigene21879\_Sample\_011046841, Unigene12972\_Sample\_011046841, Unigene44093\_Sample\_011046841, Unigene3263\_Sample\_011046841, Unigene34739\_Sample\_011046841, Unigene58210\_Sample\_011046841, Unigene17411\_Sample\_011046841, Unigene2986\_Sample\_011046841, Unigene6351\_Sample\_011046841, Unigene11139\_Sample\_011046841, Unigene45634\_Sample\_011046841, Unigene56253\_Sample\_011046841, Unigene9017\_Sample\_011046841, Unigene50528\_Sample\_011046841, Unigene38860\_Sample\_011046841, Unigene22474\_Sample\_011046841, Unigene11884\_Sample\_011046841, Unigene15848\_Sample\_011046841, Unigene10327\_Sample\_011046841, Unigene3588\_Sample\_011046841, Unigene59820\_Sample\_011046841, Unigene21023\_Sample\_011046841, Unigene32716\_Sample\_011046841, Unigene60887\_Sample\_011046841, Unigene39000\_Sample\_011046841, Unigene3075\_Sample\_011046841, Unigene8502\_Sample\_011046841, Unigene47125\_Sample\_011046841, Unigene59687\_Sample\_011046841, Unigene2610\_Sample\_011046841, Unigene46730\_Sample\_011046841, Unigene13638\_Sample\_011046841, Unigene56588\_Sample\_011046841, Unigene48097\_Sample\_011046841, Unigene45483\_Sample\_011046841, Unigene59702\_Sample\_011046841, Unigene41478\_Sample\_011046841, Unigene58747\_Sample\_011046841, Unigene40874\_Sample\_011046841, Unigene60448\_Sample\_011046841, Unigene8301\_Sample\_011046841, Unigene50690\_Sample\_011046841, Unigene1882\_Sample\_011046841, Unigene16353\_Sample\_011046841, Unigene16051\_Sample\_011046841, Unigene57665\_Sample\_011046841, Unigene8000\_Sample\_011046841, Unigene5382\_Sample\_011046841, Unigene42339\_Sample\_011046841, Unigene9421\_Sample\_011046841, Unigene52334\_Sample\_011046841, Unigene16171\_Sample\_011046841, Unigene33766\_Sample\_011046841, Unigene3199\_Sample\_011046841, Unigene92\_Sample\_011046841, Unigene56978\_Sample\_011046841, Unigene53720\_Sample\_011046841, Unigene11249\_Sample\_011046841, Unigene57853\_Sample\_011046841, Unigene56567\_Sample\_011046841, Unigene3148\_Sample\_011046841, Unigene53848\_Sample\_011046841, Unigene58415\_Sample\_011046841, Unigene6900\_Sample\_011046841, Unigene1936\_Sample\_011046841, Unigene46059\_Sample\_011046841, Unigene58686\_Sample\_011046841, Unigene50606\_Sample\_011046841, Unigene53456\_Sample\_011046841, Unigene47802\_Sample\_011046841, Unigene4218\_Sample\_011046841, Unigene15166\_Sample\_011046841, Unigene56389\_Sample\_011046841, Unigene60096\_Sample\_011046841, Unigene10122\_Sample\_011046841, Unigene42095\_Sample\_011046841, Unigene50829\_Sample\_011046841, Unigene47686\_Sample\_011046841, Unigene59254\_Sample\_011046841, Unigene59167\_Sample\_011046841, Unigene48963\_Sample\_011046841, Unigene39422\_Sample\_011046841, Unigene44683\_Sample\_011046841, Unigene12074\_Sample\_011046841, Unigene7125\_Sample\_011046841, Unigene20555\_Sample\_011046841, Unigene32450\_Sample\_011046841, Unigene57556\_Sample\_011046841, Unigene9210\_Sample\_011046841, Unigene31228\_Sample\_011046841, Unigene56793\_Sample\_011046841, Unigene10325\_Sample\_011046841, Unigene40347\_Sample\_011046841, Unigene28643\_Sample\_011046841, Unigene30931\_Sample\_011046841, Unigene59868\_Sample\_011046841, Unigene36037\_Sample\_011046841, Unigene50399\_Sample\_011046841, Unigene31717\_Sample\_011046841, Unigene8597\_Sample\_011046841, Unigene36121\_Sample\_011046841, Unigene12706\_Sample\_011046841, Unigene59557\_Sample\_011046841, Unigene41538\_Sample\_011046841, Unigene57562\_Sample\_011046841, Unigene60241\_Sample\_011046841, Unigene57119\_Sample\_011046841, Unigene60052\_Sample\_011046841, Unigene4096\_Sample\_011046841, Unigene56605\_Sample\_011046841, Unigene6061\_Sample\_011046841, Unigene53259\_Sample\_011046841, Unigene8613\_Sample\_011046841, Unigene28573\_Sample\_011046841, Unigene46219\_Sample\_011046841, Unigene12830\_Sample\_011046841, Unigene47947\_Sample\_011046841, Unigene16803\_Sample\_011046841, Unigene18318\_Sample\_011046841, Unigene10739\_Sample\_011046841, Unigene9044\_Sample\_011046841, Unigene34357\_Sample\_011046841, Unigene1345\_Sample\_011046841, Unigene28565\_Sample\_011046841, Unigene52963\_Sample\_011046841, Unigene22486\_Sample\_011046841, Unigene45758\_Sample\_011046841, Unigene53606\_Sample\_011046841, Unigene50631\_Sample\_011046841, Unigene15774\_Sample\_011046841, Unigene60161\_Sample\_011046841, Unigene57634\_Sample\_011046841, Unigene55156\_Sample\_011046841, Unigene51380\_Sample\_011046841, Unigene19273\_Sample\_011046841, Unigene60173\_Sample\_011046841, Unigene59416\_Sample\_011046841, Unigene52004\_Sample\_011046841, Unigene52734\_Sample\_011046841, Unigene45770\_Sample\_011046841, Unigene27767\_Sample\_011046841, Unigene55081\_Sample\_011046841, Unigene60452\_Sample\_011046841, Unigene46485\_Sample\_011046841, Unigene50051\_Sample\_011046841, Unigene48009\_Sample\_011046841, Unigene42838\_Sample\_011046841, Unigene53736\_Sample\_011046841, Unigene57845\_Sample\_011046841, Unigene57460\_Sample\_011046841, Unigene59022\_Sample\_011046841, Unigene35605\_Sample\_011046841, Unigene58133\_Sample\_011046841, Unigene60842\_Sample\_011046841, Unigene55791\_Sample\_011046841, Unigene43045\_Sample\_011046841, Unigene46245\_Sample\_011046841, Unigene60202\_Sample\_011046841, Unigene59485\_Sample\_011046841, Unigene37427\_Sample\_011046841, Unigene59398\_Sample\_011046841, Unigene58336\_Sample\_011046841, Unigene8939\_Sample\_011046841, Unigene49837\_Sample\_011046841, Unigene47936\_Sample\_011046841, Unigene52139\_Sample\_011046841, Unigene51504\_Sample\_011046841, Unigene8027\_Sample\_011046841, Unigene60020\_Sample\_011046841, Unigene46825\_Sample\_011046841, Unigene11562\_Sample\_011046841, Unigene60937\_Sample\_011046841, Unigene55709\_Sample\_011046841, Unigene1957\_Sample\_011046841, Unigene51782\_Sample\_011046841, Unigene10525\_Sample\_011046841, Unigene58100\_Sample\_011046841, Unigene27010\_Sample\_011046841, Unigene57313\_Sample\_011046841, Unigene33440\_Sample\_011046841, Unigene48772\_Sample\_011046841, Unigene6945\_Sample\_011046841, Unigene60523\_Sample\_011046841, Unigene4293\_Sample\_011046841, Unigene58243\_Sample\_011046841, Unigene55258\_Sample\_011046841, Unigene37761\_Sample\_011046841, Unigene58400\_Sample\_011046841, Unigene59171\_Sample\_011046841, Unigene56616\_Sample\_011046841, Unigene53419\_Sample\_011046841, Unigene46638\_Sample\_011046841, Unigene42378\_Sample\_011046841, Unigene19280\_Sample\_011046841, Unigene59834\_Sample\_011046841, Unigene7415\_Sample\_011046841, Unigene54996\_Sample\_011046841, Unigene42026\_Sample\_011046841, Unigene27802\_Sample\_011046841, Unigene57761\_Sample\_011046841, Unigene35926\_Sample\_011046841, Unigene1691\_Sample\_011046841, Unigene11259\_Sample\_011046841, Unigene13689\_Sample\_011046841, Unigene12802\_Sample\_011046841, Unigene1210\_Sample\_011046841, Unigene55561\_Sample\_011046841, Unigene1107\_Sample\_011046841, Unigene53798\_Sample\_011046841, Unigene59198\_Sample\_011046841, Unigene58496\_Sample\_011046841, Unigene43804\_Sample\_011046841, Unigene57453\_Sample\_011046841, Unigene9703\_Sample\_011046841, Unigene35368\_Sample\_011046841, Unigene36631\_Sample\_011046841, Unigene26737\_Sample\_011046841, Unigene9620\_Sample\_011046841, Unigene56991\_Sample\_011046841, Unigene54410\_Sample\_011046841, Unigene8160\_Sample\_011046841, Unigene55183\_Sample\_011046841, Unigene46777\_Sample\_011046841, Unigene29126\_Sample\_011046841, Unigene57157\_Sample\_011046841, Unigene54897\_Sample\_011046841, Unigene45403\_Sample\_011046841, Unigene12789\_Sample\_011046841, Unigene8925\_Sample\_011046841, Unigene23713\_Sample\_011046841, Unigene10615\_Sample\_011046841, Unigene29105\_Sample\_011046841, Unigene53233\_Sample\_011046841, Unigene59581\_Sample\_011046841, Unigene2459\_Sample\_011046841, Unigene54891\_Sample\_011046841, Unigene50990\_Sample\_011046841, Unigene55982\_Sample\_011046841, Unigene47487\_Sample\_011046841, Unigene26714\_Sample\_011046841, Unigene50223\_Sample\_011046841, Unigene26723\_Sample\_011046841, Unigene50164\_Sample\_011046841, Unigene15865\_Sample\_011046841, Unigene55136\_Sample\_011046841, Unigene12699\_Sample\_011046841, Unigene15751\_Sample\_011046841, Unigene39842\_Sample\_011046841, Unigene18725\_Sample\_011046841, Unigene39276\_Sample\_011046841, Unigene51260\_Sample\_011046841, Unigene59257\_Sample\_011046841, Unigene41168\_Sample\_011046841, Unigene12397\_Sample\_011046841, Unigene44079\_Sample\_011046841, Unigene38180\_Sample\_011046841, Unigene27641\_Sample\_011046841, Unigene43135\_Sample\_011046841, Unigene59311\_Sample\_011046841, Unigene40774\_Sample\_011046841, Unigene60586\_Sample\_011046841, Unigene32556\_Sample\_011046841, Unigene45912\_Sample\_011046841, Unigene1306\_Sample\_011046841, Unigene9607\_Sample\_011046841, Unigene19699\_Sample\_011046841, Unigene54406\_Sample\_011046841, Unigene50596\_Sample\_011046841, Unigene35715\_Sample\_011046841, Unigene53819\_Sample\_011046841, Unigene46064\_Sample\_011046841, Unigene36725\_Sample\_011046841, Unigene43838\_Sample\_011046841, Unigene59563\_Sample\_011046841, Unigene51820\_Sample\_011046841, Unigene41631\_Sample\_011046841, Unigene51369\_Sample\_011046841, Unigene18813\_Sample\_011046841, Unigene4166\_Sample\_011046841, Unigene31055\_Sample\_011046841, Unigene60192\_Sample\_011046841, Unigene47907\_Sample\_011046841, Unigene60225\_Sample\_011046841, Unigene50775\_Sample\_011046841, Unigene2140\_Sample\_011046841, Unigene45679\_Sample\_011046841, Unigene6597\_Sample\_011046841, Unigene5169\_Sample\_011046841, Unigene52097\_Sample\_011046841, Unigene16689\_Sample\_011046841, Unigene13476\_Sample\_011046841, Unigene42012\_Sample\_011046841, Unigene38168\_Sample\_011046841, Unigene46414\_Sample\_011046841, Unigene32054\_Sample\_011046841, Unigene6958\_Sample\_011046841, Unigene35453\_Sample\_011046841, Unigene8997\_Sample\_011046841, Unigene59201\_Sample\_011046841, Unigene54997\_Sample\_011046841, Unigene50396\_Sample\_011046841, Unigene53865\_Sample\_011046841, Unigene4113\_Sample\_011046841, Unigene36469\_Sample\_011046841, Unigene44429\_Sample\_011046841, Unigene60705\_Sample\_011046841, Unigene54221\_Sample\_011046841, Unigene60325\_Sample\_011046841, Unigene10640\_Sample\_011046841, Unigene59234\_Sample\_011046841, Unigene39223\_Sample\_011046841, Unigene43099\_Sample\_011046841, Unigene56127\_Sample\_011046841, Unigene57340\_Sample\_011046841, Unigene3756\_Sample\_011046841, Unigene13258\_Sample\_011046841, Unigene56364\_Sample\_011046841, Unigene59440\_Sample\_011046841, Unigene51569\_Sample\_011046841, Unigene10204\_Sample\_011046841, Unigene10963\_Sample\_011046841, Unigene15\_Sample\_011046841, Unigene23779\_Sample\_011046841, Unigene17258\_Sample\_011046841, Unigene7887\_Sample\_011046841, Unigene47549\_Sample\_011046841, Unigene40697\_Sample\_011046841, Unigene12620\_Sample\_011046841, Unigene9855\_Sample\_011046841, Unigene57106\_Sample\_011046841, Unigene44038\_Sample\_011046841, Unigene31920\_Sample\_011046841, Unigene8959\_Sample\_011046841, Unigene42747\_Sample\_011046841, Unigene51225\_Sample\_011046841, Unigene55175\_Sample\_011046841, Unigene58053\_Sample\_011046841, Unigene54341\_Sample\_011046841, Unigene47356\_Sample\_011046841, Unigene40147\_Sample\_011046841, Unigene16055\_Sample\_011046841, Unigene11184\_Sample\_011046841, Unigene51363\_Sample\_011046841, Unigene60921\_Sample\_011046841, Unigene39877\_Sample\_011046841, Unigene53957\_Sample\_011046841, Unigene44676\_Sample\_011046841, Unigene49532\_Sample\_011046841, Unigene60000\_Sample\_011046841, Unigene56881\_Sample\_011046841, Unigene57132\_Sample\_011046841, Unigene13805\_Sample\_011046841, Unigene58617\_Sample\_011046841, Unigene48876\_Sample\_011046841, Unigene9125\_Sample\_011046841, Unigene13511\_Sample\_011046841, Unigene27899\_Sample\_011046841, Unigene11187\_Sample\_011046841, Unigene53037\_Sample\_011046841, Unigene54326\_Sample\_011046841, Unigene59812\_Sample\_011046841, Unigene20608\_Sample\_011046841, Unigene47078\_Sample\_011046841, Unigene60149\_Sample\_011046841, Unigene46774\_Sample\_011046841, Unigene59224\_Sample\_011046841, Unigene50689\_Sample\_011046841, Unigene57610\_Sample\_011046841, Unigene50412\_Sample\_011046841, Unigene13740\_Sample\_011046841, Unigene49228\_Sample\_011046841, Unigene56093\_Sample\_011046841, Unigene51954\_Sample\_011046841, Unigene48339\_Sample\_011046841, Unigene4547\_Sample\_011046841, Unigene59919\_Sample\_011046841, Unigene42703\_Sample\_011046841, Unigene60714\_Sample\_011046841, Unigene14776\_Sample\_011046841, Unigene2325\_Sample\_011046841, Unigene31849\_Sample\_011046841, Unigene43574\_Sample\_011046841, Unigene6762\_Sample\_011046841, Unigene32785\_Sample\_011046841, Unigene52303\_Sample\_011046841, Unigene10098\_Sample\_011046841, Unigene56560\_Sample\_011046841, Unigene10694\_Sample\_011046841, Unigene57060\_Sample\_011046841, Unigene28319\_Sample\_011046841, Unigene42105\_Sample\_011046841, Unigene58404\_Sample\_011046841, Unigene40568\_Sample\_011046841, Unigene53410\_Sample\_011046841, Unigene38066\_Sample\_011046841, Unigene59749\_Sample\_011046841, Unigene51285\_Sample\_011046841, Unigene20324\_Sample\_011046841, Unigene3797\_Sample\_011046841, Unigene58669\_Sample\_011046841, Unigene27211\_Sample\_011046841, Unigene54643\_Sample\_011046841, Unigene58326\_Sample\_011046841, Unigene58953\_Sample\_011046841, Unigene55769\_Sample\_011046841, Unigene52984\_Sample\_011046841, Unigene19673\_Sample\_011046841, Unigene19813\_Sample\_011046841, Unigene55519\_Sample\_011046841, Unigene59371\_Sample\_011046841, Unigene36735\_Sample\_011046841, Unigene6520\_Sample\_011046841, Unigene21486\_Sample\_011046841, Unigene42996\_Sample\_011046841, Unigene29861\_Sample\_011046841, Unigene13569\_Sample\_011046841, Unigene25605\_Sample\_011046841, Unigene46082\_Sample\_011046841, Unigene22643\_Sample\_011046841, Unigene49204\_Sample\_011046841, Unigene49038\_Sample\_011046841, Unigene60127\_Sample\_011046841, Unigene60886\_Sample\_011046841, Unigene40649\_Sample\_011046841, Unigene55735\_Sample\_011046841, Unigene55491\_Sample\_011046841, Unigene56230\_Sample\_011046841, Unigene46978\_Sample\_011046841, Unigene30575\_Sample\_011046841, Unigene60217\_Sample\_011046841, Unigene40620\_Sample\_011046841, Unigene58612\_Sample\_011046841, Unigene55854\_Sample\_011046841, Unigene60596\_Sample\_011046841, Unigene58482\_Sample\_011046841, Unigene57614\_Sample\_011046841, Unigene57972\_Sample\_011046841, Unigene50665\_Sample\_011046841, Unigene5194\_Sample\_011046841, Unigene28318\_Sample\_011046841, Unigene24228\_Sample\_011046841, Unigene51483\_Sample\_011046841, Unigene29630\_Sample\_011046841, Unigene44651\_Sample\_011046841, Unigene20892\_Sample\_011046841, Unigene39713\_Sample\_011046841, Unigene16440\_Sample\_011046841, Unigene46332\_Sample\_011046841, Unigene33510\_Sample\_011046841, Unigene39621\_Sample\_011046841, Unigene42270\_Sample\_011046841, Unigene35476\_Sample\_011046841, Unigene2179\_Sample\_011046841, Unigene30971\_Sample\_011046841, Unigene10842\_Sample\_011046841, Unigene14320\_Sample\_011046841, Unigene4684\_Sample\_011046841, Unigene34653\_Sample\_011046841, Unigene9546\_Sample\_011046841, Unigene60802\_Sample\_011046841, Unigene60436\_Sample\_011046841, Unigene16808\_Sample\_011046841, Unigene60949\_Sample\_011046841, Unigene32258\_Sample\_011046841, Unigene45334\_Sample\_011046841, Unigene42516\_Sample\_011046841, Unigene12782\_Sample\_011046841, Unigene24568\_Sample\_011046841, Unigene42890\_Sample\_011046841, Unigene60729\_Sample\_011046841, Unigene52566\_Sample\_011046841, Unigene18133\_Sample\_011046841, Unigene224\_Sample\_011046841, Unigene1378\_Sample\_011046841, Unigene37162\_Sample\_011046841, Unigene9011\_Sample\_011046841, Unigene34641\_Sample\_011046841, Unigene22086\_Sample\_011046841, Unigene19137\_Sample\_011046841, Unigene41288\_Sample\_011046841, Unigene9024\_Sample\_011046841, Unigene608\_Sample\_011046841, Unigene20373\_Sample\_011046841, Unigene60699\_Sample\_011046841, Unigene13820\_Sample\_011046841, Unigene60923\_Sample\_011046841, Unigene54628\_Sample\_011046841, Unigene24776\_Sample\_011046841, Unigene49162\_Sample\_011046841, Unigene18124\_Sample\_011046841, Unigene60785\_Sample\_011046841, Unigene21919\_Sample\_011046841, Unigene51537\_Sample\_011046841, Unigene54448\_Sample\_011046841, Unigene7277\_Sample\_011046841, Unigene58611\_Sample\_011046841, Unigene140\_Sample\_011046841, Unigene5325\_Sample\_011046841, Unigene58753\_Sample\_011046841, Unigene13943\_Sample\_011046841, Unigene60966\_Sample\_011046841, Unigene39756\_Sample\_011046841, Unigene13937\_Sample\_011046841, Unigene60106\_Sample\_011046841, Unigene52776\_Sample\_011046841, Unigene75\_Sample\_011046841, Unigene60819\_Sample\_011046841, Unigene29206\_Sample\_011046841, Unigene45295\_Sample\_011046841, Unigene33721\_Sample\_011046841, Unigene26171\_Sample\_011046841, Unigene47769\_Sample\_011046841, Unigene60371\_Sample\_011046841, Unigene22728\_Sample\_011046841, Unigene10793\_Sample\_011046841, Unigene19671\_Sample\_011046841, Unigene27756\_Sample\_011046841, Unigene4226\_Sample\_011046841, Unigene10588\_Sample\_011046841, Unigene58058\_Sample\_011046841, Unigene25856\_Sample\_011046841, Unigene54379\_Sample\_011046841, Unigene40785\_Sample\_011046841, Unigene57776\_Sample\_011046841, Unigene47882\_Sample\_011046841, Unigene59938\_Sample\_011046841, Unigene51659\_Sample\_011046841, Unigene28860\_Sample\_011046841, Unigene47915\_Sample\_011046841, Unigene38959\_Sample\_011046841, Unigene50443\_Sample\_011046841, Unigene42349\_Sample\_011046841, Unigene17987\_Sample\_011046841, Unigene60931\_Sample\_011046841, Unigene10464\_Sample\_011046841, Unigene60642\_Sample\_011046841, Unigene51051\_Sample\_011046841, Unigene7922\_Sample\_011046841, Unigene54048\_Sample\_011046841, Unigene8835\_Sample\_011046841, Unigene42686\_Sample\_011046841, Unigene36841\_Sample\_011046841, Unigene51977\_Sample\_011046841, Unigene57289\_Sample\_011046841, Unigene13115\_Sample\_011046841, Unigene39430\_Sample\_011046841, Unigene10997\_Sample\_011046841, Unigene59743\_Sample\_011046841, Unigene28536\_Sample\_011046841, Unigene50445\_Sample\_011046841, Unigene29346\_Sample\_011046841, Unigene21502\_Sample\_011046841, Unigene51942\_Sample\_011046841, Unigene59844\_Sample\_011046841, Unigene9737\_Sample\_011046841, Unigene25397\_Sample\_011046841, Unigene60862\_Sample\_011046841, Unigene59467\_Sample\_011046841, Unigene53845\_Sample\_011046841, Unigene58706\_Sample\_011046841, Unigene60208\_Sample\_011046841, Unigene21239\_Sample\_011046841, Unigene49898\_Sample\_011046841, Unigene54626\_Sample\_011046841, Unigene12811\_Sample\_011046841, Unigene60159\_Sample\_011046841, Unigene41464\_Sample\_011046841, Unigene6398\_Sample\_011046841, Unigene59559\_Sample\_011046841, Unigene52238\_Sample\_011046841, Unigene25745\_Sample\_011046841, Unigene27451\_Sample\_011046841, Unigene20667\_Sample\_011046841, Unigene46897\_Sample\_011046841, Unigene19408\_Sample\_011046841, Unigene58584\_Sample\_011046841, Unigene25525\_Sample\_011046841, Unigene46142\_Sample\_011046841, Unigene9811\_Sample\_011046841, Unigene14121\_Sample\_011046841, Unigene12867\_Sample\_011046841, Unigene59935\_Sample\_011046841, Unigene56584\_Sample\_011046841, Unigene4949\_Sample\_011046841, Unigene56360\_Sample\_011046841, Unigene20059\_Sample\_011046841, Unigene59518\_Sample\_011046841, Unigene52701\_Sample\_011046841, Unigene28077\_Sample\_011046841, Unigene24715\_Sample\_011046841, Unigene13112\_Sample\_011046841, Unigene383\_Sample\_011046841, Unigene25928\_Sample\_011046841, Unigene60666\_Sample\_011046841, Unigene21201\_Sample\_011046841, Unigene47701\_Sample\_011046841, Unigene43556\_Sample\_011046841, Unigene56502\_Sample\_011046841, Unigene33607\_Sample\_011046841, Unigene60917\_Sample\_011046841, Unigene43690\_Sample\_011046841, Unigene26220\_Sample\_011046841, Unigene23751\_Sample\_011046841, Unigene38158\_Sample\_011046841, Unigene59268\_Sample\_011046841, Unigene50303\_Sample\_011046841, Unigene49377\_Sample\_011046841, Unigene50317\_Sample\_011046841, Unigene58610\_Sample\_011046841, Unigene52005\_Sample\_011046841, Unigene56952\_Sample\_011046841, Unigene46488\_Sample\_011046841, Unigene52842\_Sample\_011046841, Unigene33026\_Sample\_011046841, Unigene34637\_Sample\_011046841, Unigene30916\_Sample\_011046841, Unigene37830\_Sample\_011046841, Unigene41903\_Sample\_011046841, Unigene42862\_Sample\_011046841, Unigene50039\_Sample\_011046841, Unigene8516\_Sample\_011046841, Unigene13354\_Sample\_011046841, Unigene52094\_Sample\_011046841, Unigene56819\_Sample\_011046841, Unigene59861\_Sample\_011046841, Unigene31025\_Sample\_011046841, Unigene39219\_Sample\_011046841, Unigene60723\_Sample\_011046841, Unigene48474\_Sample\_011046841, Unigene24973\_Sample\_011046841, Unigene12846\_Sample\_011046841, Unigene48570\_Sample\_011046841, Unigene59750\_Sample\_011046841, Unigene13079\_Sample\_011046841, Unigene15705\_Sample\_011046841, Unigene55582\_Sample\_011046841, Unigene57487\_Sample\_011046841, Unigene12245\_Sample\_011046841, Unigene4966\_Sample\_011046841, Unigene56109\_Sample\_011046841, Unigene60667\_Sample\_011046841, Unigene55570\_Sample\_011046841, Unigene13371\_Sample\_011046841, Unigene52247\_Sample\_011046841, Unigene41698\_Sample\_011046841, Unigene58661\_Sample\_011046841, Unigene132\_Sample\_011046841, Unigene59701\_Sample\_011046841, Unigene30295\_Sample\_011046841, Unigene60180\_Sample\_011046841, Unigene31216\_Sample\_011046841, Unigene22796\_Sample\_011046841, Unigene55112\_Sample\_011046841, Unigene51279\_Sample\_011046841, Unigene40934\_Sample\_011046841, Unigene59546\_Sample\_011046841, Unigene58020\_Sample\_011046841, Unigene18756\_Sample\_011046841, Unigene40166\_Sample\_011046841, Unigene56647\_Sample\_011046841, Unigene51233\_Sample\_011046841, Unigene35664\_Sample\_011046841, Unigene20592\_Sample\_011046841, Unigene12652\_Sample\_011046841, Unigene58724\_Sample\_011046841, Unigene13516\_Sample\_011046841, Unigene58309\_Sample\_011046841, Unigene60543\_Sample\_011046841, Unigene41206\_Sample\_011046841, Unigene23218\_Sample\_011046841, Unigene53711\_Sample\_011046841, Unigene18170\_Sample\_011046841, Unigene28796\_Sample\_011046841, Unigene46756\_Sample\_011046841, Unigene35030\_Sample\_011046841, Unigene13123\_Sample\_011046841, Unigene18796\_Sample\_011046841, Unigene45119\_Sample\_011046841, Unigene59005\_Sample\_011046841, Unigene29365\_Sample\_011046841, Unigene54800\_Sample\_011046841, Unigene9660\_Sample\_011046841, Unigene60749\_Sample\_011046841, Unigene60272\_Sample\_011046841, Unigene59372\_Sample\_011046841, Unigene23619\_Sample\_011046841, Unigene55792\_Sample\_011046841, Unigene12903\_Sample\_011046841, Unigene7487\_Sample\_011046841, Unigene49667\_Sample\_011046841, Unigene23102\_Sample\_011046841, Unigene52196\_Sample\_011046841, Unigene57681\_Sample\_011046841, Unigene43515\_Sample\_011046841, Unigene16372\_Sample\_011046841, Unigene16432\_Sample\_011046841, Unigene33690\_Sample\_011046841, Unigene58340\_Sample\_011046841, Unigene57633\_Sample\_011046841, Unigene58042\_Sample\_011046841, Unigene60040\_Sample\_011046841, Unigene60390\_Sample\_011046841, Unigene41575\_Sample\_011046841, Unigene57330\_Sample\_011046841, Unigene55037\_Sample\_011046841, Unigene51011\_Sample\_011046841, Unigene11358\_Sample\_011046841, Unigene37176\_Sample\_011046841, Unigene9130\_Sample\_011046841, Unigene44178\_Sample\_011046841, Unigene40157\_Sample\_011046841, Unigene18000\_Sample\_011046841, Unigene47429\_Sample\_011046841, Unigene60734\_Sample\_011046841, Unigene12824\_Sample\_011046841, Unigene47387\_Sample\_011046841, Unigene60733\_Sample\_011046841, Unigene11124\_Sample\_011046841, Unigene44756\_Sample\_011046841, Unigene46613\_Sample\_011046841, Unigene47203\_Sample\_011046841, Unigene13616\_Sample\_011046841, Unigene36370\_Sample\_011046841, Unigene3893\_Sample\_011046841, Unigene8588\_Sample\_011046841, Unigene57710\_Sample\_011046841, Unigene39698\_Sample\_011046841, Unigene31652\_Sample\_011046841, Unigene23246\_Sample\_011046841, Unigene40683\_Sample\_011046841, Unigene26711\_Sample\_011046841, Unigene38155\_Sample\_011046841, Unigene20702\_Sample\_011046841, Unigene45885\_Sample\_011046841, Unigene40222\_Sample\_011046841, Unigene50927\_Sample\_011046841, Unigene1702\_Sample\_011046841, Unigene41564\_Sample\_011046841, Unigene55071\_Sample\_011046841, Unigene45790\_Sample\_011046841, Unigene41740\_Sample\_011046841, Unigene11504\_Sample\_011046841, Unigene13763\_Sample\_011046841, Unigene12725\_Sample\_011046841, Unigene57425\_Sample\_011046841, Unigene30456\_Sample\_011046841, Unigene10251\_Sample\_011046841, Unigene57876\_Sample\_011046841, Unigene33255\_Sample\_011046841, Unigene1482\_Sample\_011046841, Unigene36199\_Sample\_011046841, Unigene39709\_Sample\_011046841, Unigene25064\_Sample\_011046841, Unigene51663\_Sample\_011046841, Unigene6829\_Sample\_011046841, Unigene52181\_Sample\_011046841, Unigene58963\_Sample\_011046841, Unigene54599\_Sample\_011046841, Unigene59532\_Sample\_011046841, Unigene11186\_Sample\_011046841, Unigene5530\_Sample\_011046841, Unigene13727\_Sample\_011046841, Unigene54526\_Sample\_011046841, Unigene2484\_Sample\_011046841, Unigene6246\_Sample\_011046841, Unigene35468\_Sample\_011046841, Unigene37815\_Sample\_011046841, Unigene7072\_Sample\_011046841, Unigene43897\_Sample\_011046841, Unigene12396\_Sample\_011046841, Unigene51429\_Sample\_011046841, Unigene50071\_Sample\_011046841, Unigene13839\_Sample\_011046841, Unigene53450\_Sample\_011046841, Unigene46831\_Sample\_011046841, Unigene32313\_Sample\_011046841, Unigene53555\_Sample\_011046841, Unigene44445\_Sample\_011046841, Unigene36986\_Sample\_011046841, Unigene42117\_Sample\_011046841, Unigene5468\_Sample\_011046841, Unigene55499\_Sample\_011046841, Unigene49199\_Sample\_011046841, Unigene58372\_Sample\_011046841, Unigene52246\_Sample\_011046841 |
| phosphotransferase activity, alcohol group as acceptor | Unigene55833\_Sample\_011046841, Unigene57972\_Sample\_011046841, Unigene58864\_Sample\_011046841, Unigene55221\_Sample\_011046841, Unigene16112\_Sample\_011046841, Unigene51130\_Sample\_011046841, Unigene31969\_Sample\_011046841, Unigene2784\_Sample\_011046841, Unigene52866\_Sample\_011046841, Unigene10250\_Sample\_011046841, Unigene9858\_Sample\_011046841, Unigene11120\_Sample\_011046841, Unigene9354\_Sample\_011046841, Unigene29684\_Sample\_011046841, Unigene4303\_Sample\_011046841, Unigene51777\_Sample\_011046841, Unigene33573\_Sample\_011046841, Unigene60481\_Sample\_011046841, Unigene16803\_Sample\_011046841, Unigene49534\_Sample\_011046841, Unigene18318\_Sample\_011046841, Unigene55162\_Sample\_011046841, Unigene30971\_Sample\_011046841, Unigene48482\_Sample\_011046841, Unigene18749\_Sample\_011046841, Unigene53995\_Sample\_011046841, Unigene12538\_Sample\_011046841, Unigene59421\_Sample\_011046841, Unigene21225\_Sample\_011046841, Unigene10970\_Sample\_011046841, Unigene54240\_Sample\_011046841, Unigene56545\_Sample\_011046841, Unigene51706\_Sample\_011046841, Unigene57634\_Sample\_011046841, Unigene54683\_Sample\_011046841, Unigene9546\_Sample\_011046841, Unigene60498\_Sample\_011046841, Unigene57083\_Sample\_011046841, Unigene57589\_Sample\_011046841, Unigene51380\_Sample\_011046841, Unigene54701\_Sample\_011046841, Unigene41318\_Sample\_011046841, Unigene2942\_Sample\_011046841, Unigene32119\_Sample\_011046841, Unigene56697\_Sample\_011046841, Unigene60173\_Sample\_011046841, Unigene51845\_Sample\_011046841, Unigene59756\_Sample\_011046841, Unigene45770\_Sample\_011046841, Unigene51582\_Sample\_011046841, Unigene40435\_Sample\_011046841, Unigene4209\_Sample\_011046841, Unigene13672\_Sample\_011046841, Unigene10683\_Sample\_011046841, Unigene55610\_Sample\_011046841, Unigene13068\_Sample\_011046841, Unigene7879\_Sample\_011046841, Unigene46485\_Sample\_011046841, Unigene31264\_Sample\_011046841, Unigene26327\_Sample\_011046841, Unigene50051\_Sample\_011046841, Unigene48009\_Sample\_011046841, Unigene30954\_Sample\_011046841, Unigene59022\_Sample\_011046841, Unigene55126\_Sample\_011046841, Unigene36373\_Sample\_011046841, Unigene26636\_Sample\_011046841, Unigene60190\_Sample\_011046841, Unigene56529\_Sample\_011046841, Unigene40349\_Sample\_011046841, Unigene60657\_Sample\_011046841, Unigene37753\_Sample\_011046841, Unigene57641\_Sample\_011046841, Unigene12309\_Sample\_011046841, Unigene10198\_Sample\_011046841, Unigene32409\_Sample\_011046841, Unigene10875\_Sample\_011046841, Unigene4361\_Sample\_011046841, Unigene23831\_Sample\_011046841, Unigene43397\_Sample\_011046841, Unigene46283\_Sample\_011046841, Unigene24424\_Sample\_011046841, Unigene608\_Sample\_011046841, Unigene41574\_Sample\_011046841, Unigene50170\_Sample\_011046841, Unigene47924\_Sample\_011046841, Unigene58134\_Sample\_011046841, Unigene52918\_Sample\_011046841, Unigene13419\_Sample\_011046841, Unigene39238\_Sample\_011046841, Unigene49837\_Sample\_011046841, Unigene19453\_Sample\_011046841, Unigene30474\_Sample\_011046841, Unigene11457\_Sample\_011046841, Unigene47936\_Sample\_011046841, Unigene27253\_Sample\_011046841, Unigene35914\_Sample\_011046841, Unigene58460\_Sample\_011046841, Unigene60557\_Sample\_011046841, Unigene43504\_Sample\_011046841, Unigene50797\_Sample\_011046841, Unigene58128\_Sample\_011046841, Unigene4662\_Sample\_011046841, Unigene39675\_Sample\_011046841, Unigene36772\_Sample\_011046841, Unigene47292\_Sample\_011046841, Unigene54614\_Sample\_011046841, Unigene59061\_Sample\_011046841, Unigene11114\_Sample\_011046841, Unigene11130\_Sample\_011046841, Unigene51537\_Sample\_011046841, Unigene16001\_Sample\_011046841, Unigene52610\_Sample\_011046841, Unigene59469\_Sample\_011046841, Unigene58100\_Sample\_011046841, Unigene13224\_Sample\_011046841, Unigene13287\_Sample\_011046841, Unigene5297\_Sample\_011046841, Unigene15490\_Sample\_011046841, Unigene60576\_Sample\_011046841, Unigene11231\_Sample\_011046841, Unigene26229\_Sample\_011046841, Unigene57832\_Sample\_011046841, Unigene56054\_Sample\_011046841, Unigene10726\_Sample\_011046841, Unigene9879\_Sample\_011046841, Unigene26777\_Sample\_011046841, Unigene56258\_Sample\_011046841, Unigene42893\_Sample\_011046841, Unigene41032\_Sample\_011046841, Unigene59448\_Sample\_011046841, Unigene19352\_Sample\_011046841, Unigene301\_Sample\_011046841, Unigene13332\_Sample\_011046841, Unigene11925\_Sample\_011046841, Unigene57775\_Sample\_011046841, Unigene23925\_Sample\_011046841, Unigene9348\_Sample\_011046841, Unigene47887\_Sample\_011046841, Unigene47872\_Sample\_011046841, Unigene51573\_Sample\_011046841, Unigene58058\_Sample\_011046841, Unigene37549\_Sample\_011046841, Unigene60248\_Sample\_011046841, Unigene54379\_Sample\_011046841, Unigene56798\_Sample\_011046841, Unigene16539\_Sample\_011046841, Unigene10522\_Sample\_011046841, Unigene9862\_Sample\_011046841, Unigene13368\_Sample\_011046841, Unigene13722\_Sample\_011046841, Unigene23133\_Sample\_011046841, Unigene58386\_Sample\_011046841, Unigene56825\_Sample\_011046841, Unigene27682\_Sample\_011046841, Unigene48345\_Sample\_011046841, Unigene26192\_Sample\_011046841, Unigene12390\_Sample\_011046841, Unigene49895\_Sample\_011046841, Unigene51301\_Sample\_011046841, Unigene1107\_Sample\_011046841, Unigene13633\_Sample\_011046841, Unigene59727\_Sample\_011046841, Unigene45307\_Sample\_011046841, Unigene14248\_Sample\_011046841, Unigene42686\_Sample\_011046841, Unigene25326\_Sample\_011046841, Unigene60344\_Sample\_011046841, Unigene11547\_Sample\_011046841, Unigene49487\_Sample\_011046841, Unigene21362\_Sample\_011046841, Unigene30190\_Sample\_011046841, Unigene57904\_Sample\_011046841, Unigene40509\_Sample\_011046841, Unigene17850\_Sample\_011046841, Unigene50713\_Sample\_011046841, Unigene23590\_Sample\_011046841, Unigene35290\_Sample\_011046841, Unigene16977\_Sample\_011046841, Unigene5220\_Sample\_011046841, Unigene38549\_Sample\_011046841, Unigene47967\_Sample\_011046841, Unigene59844\_Sample\_011046841, Unigene60349\_Sample\_011046841, Unigene49055\_Sample\_011046841, Unigene51916\_Sample\_011046841, Unigene28563\_Sample\_011046841, Unigene7335\_Sample\_011046841, Unigene21150\_Sample\_011046841, Unigene55423\_Sample\_011046841, Unigene13901\_Sample\_011046841, Unigene60507\_Sample\_011046841, Unigene6223\_Sample\_011046841, Unigene55644\_Sample\_011046841, Unigene27562\_Sample\_011046841, Unigene11073\_Sample\_011046841, Unigene25810\_Sample\_011046841, Unigene59850\_Sample\_011046841, Unigene3810\_Sample\_011046841, Unigene54626\_Sample\_011046841, Unigene55404\_Sample\_011046841, Unigene50223\_Sample\_011046841, Unigene3269\_Sample\_011046841, Unigene41464\_Sample\_011046841, Unigene36927\_Sample\_011046841, Unigene59559\_Sample\_011046841, Unigene22189\_Sample\_011046841, Unigene48342\_Sample\_011046841, Unigene37057\_Sample\_011046841, Unigene13197\_Sample\_011046841, Unigene26587\_Sample\_011046841, Unigene18791\_Sample\_011046841, Unigene54453\_Sample\_011046841, Unigene11419\_Sample\_011046841, Unigene9776\_Sample\_011046841, Unigene59228\_Sample\_011046841, Unigene26757\_Sample\_011046841, Unigene48530\_Sample\_011046841, Unigene59257\_Sample\_011046841, Unigene51307\_Sample\_011046841, Unigene46142\_Sample\_011046841, Unigene34482\_Sample\_011046841, Unigene9811\_Sample\_011046841, Unigene53100\_Sample\_011046841, Unigene46256\_Sample\_011046841, Unigene58021\_Sample\_011046841, Unigene26679\_Sample\_011046841, Unigene44079\_Sample\_011046841, Unigene7042\_Sample\_011046841, Unigene11040\_Sample\_011046841, Unigene44103\_Sample\_011046841, Unigene59221\_Sample\_011046841, Unigene57130\_Sample\_011046841, Unigene46176\_Sample\_011046841, Unigene60753\_Sample\_011046841, Unigene59987\_Sample\_011046841, Unigene32760\_Sample\_011046841, Unigene60166\_Sample\_011046841, Unigene60721\_Sample\_011046841, Unigene53884\_Sample\_011046841, Unigene13321\_Sample\_011046841, Unigene60042\_Sample\_011046841, Unigene32964\_Sample\_011046841, Unigene54549\_Sample\_011046841, Unigene49264\_Sample\_011046841, Unigene52573\_Sample\_011046841, Unigene48062\_Sample\_011046841, Unigene35715\_Sample\_011046841, Unigene53819\_Sample\_011046841, Unigene59457\_Sample\_011046841, Unigene42633\_Sample\_011046841, Unigene57004\_Sample\_011046841, Unigene49737\_Sample\_011046841, Unigene11867\_Sample\_011046841, Unigene12975\_Sample\_011046841, Unigene35082\_Sample\_011046841, Unigene48267\_Sample\_011046841, Unigene1793\_Sample\_011046841, Unigene52842\_Sample\_011046841, Unigene54707\_Sample\_011046841, Unigene60746\_Sample\_011046841, Unigene5623\_Sample\_011046841, Unigene11998\_Sample\_011046841, Unigene58523\_Sample\_011046841, Unigene11526\_Sample\_011046841, Unigene60540\_Sample\_011046841, Unigene29318\_Sample\_011046841, Unigene45662\_Sample\_011046841, Unigene50039\_Sample\_011046841, Unigene32914\_Sample\_011046841, Unigene55041\_Sample\_011046841, Unigene8288\_Sample\_011046841, Unigene52094\_Sample\_011046841, Unigene49268\_Sample\_011046841, Unigene16813\_Sample\_011046841, Unigene59840\_Sample\_011046841, Unigene31749\_Sample\_011046841, Unigene18294\_Sample\_011046841, Unigene17843\_Sample\_011046841, Unigene47025\_Sample\_011046841, Unigene7481\_Sample\_011046841, Unigene39219\_Sample\_011046841, Unigene50057\_Sample\_011046841, Unigene6597\_Sample\_011046841, Unigene54574\_Sample\_011046841, Unigene54982\_Sample\_011046841, Unigene36941\_Sample\_011046841, Unigene52097\_Sample\_011046841, Unigene47591\_Sample\_011046841, Unigene35398\_Sample\_011046841, Unigene59705\_Sample\_011046841, Unigene32054\_Sample\_011046841, Unigene15705\_Sample\_011046841, Unigene11932\_Sample\_011046841, Unigene58675\_Sample\_011046841, Unigene59226\_Sample\_011046841, Unigene13920\_Sample\_011046841, Unigene58427\_Sample\_011046841, Unigene8997\_Sample\_011046841, Unigene47085\_Sample\_011046841, Unigene58913\_Sample\_011046841, Unigene34330\_Sample\_011046841, Unigene59758\_Sample\_011046841, Unigene7964\_Sample\_011046841, Unigene9329\_Sample\_011046841, Unigene51958\_Sample\_011046841, Unigene60244\_Sample\_011046841, Unigene42912\_Sample\_011046841, Unigene53714\_Sample\_011046841, Unigene3842\_Sample\_011046841, Unigene21655\_Sample\_011046841, Unigene56744\_Sample\_011046841, Unigene30484\_Sample\_011046841, Unigene28789\_Sample\_011046841, Unigene2076\_Sample\_011046841, Unigene27464\_Sample\_011046841, Unigene3750\_Sample\_011046841, Unigene6531\_Sample\_011046841, Unigene44021\_Sample\_011046841, Unigene58285\_Sample\_011046841, Unigene34824\_Sample\_011046841, Unigene28658\_Sample\_011046841, Unigene59440\_Sample\_011046841, Unigene15\_Sample\_011046841, Unigene3363\_Sample\_011046841, Unigene55720\_Sample\_011046841, Unigene55261\_Sample\_011046841, Unigene43936\_Sample\_011046841, Unigene60821\_Sample\_011046841, Unigene12652\_Sample\_011046841, Unigene55461\_Sample\_011046841, Unigene49671\_Sample\_011046841, Unigene60434\_Sample\_011046841, Unigene2493\_Sample\_011046841, Unigene4305\_Sample\_011046841, Unigene20201\_Sample\_011046841, Unigene13516\_Sample\_011046841, Unigene2933\_Sample\_011046841, Unigene59459\_Sample\_011046841, Unigene30684\_Sample\_011046841, Unigene53958\_Sample\_011046841, Unigene48334\_Sample\_011046841, Unigene52959\_Sample\_011046841, Unigene57278\_Sample\_011046841, Unigene51756\_Sample\_011046841, Unigene23218\_Sample\_011046841, Unigene47210\_Sample\_011046841, Unigene18170\_Sample\_011046841, Unigene22964\_Sample\_011046841, Unigene8429\_Sample\_011046841, Unigene37707\_Sample\_011046841, Unigene5431\_Sample\_011046841, Unigene56566\_Sample\_011046841, Unigene55302\_Sample\_011046841, Unigene15482\_Sample\_011046841, Unigene35470\_Sample\_011046841, Unigene12972\_Sample\_011046841, Unigene18796\_Sample\_011046841, Unigene40258\_Sample\_011046841, Unigene5972\_Sample\_011046841, Unigene58821\_Sample\_011046841, Unigene52385\_Sample\_011046841, Unigene9219\_Sample\_011046841, Unigene41676\_Sample\_011046841, Unigene41682\_Sample\_011046841, Unigene40209\_Sample\_011046841, Unigene25989\_Sample\_011046841, Unigene60191\_Sample\_011046841, Unigene46813\_Sample\_011046841, Unigene36178\_Sample\_011046841, Unigene58254\_Sample\_011046841, Unigene12359\_Sample\_011046841, Unigene43515\_Sample\_011046841, Unigene29314\_Sample\_011046841, Unigene9017\_Sample\_011046841, Unigene59891\_Sample\_011046841, Unigene13966\_Sample\_011046841, Unigene4308\_Sample\_011046841, Unigene42281\_Sample\_011046841, Unigene28288\_Sample\_011046841, Unigene13884\_Sample\_011046841, Unigene58340\_Sample\_011046841, Unigene19556\_Sample\_011046841, Unigene42820\_Sample\_011046841, Unigene35217\_Sample\_011046841, Unigene58042\_Sample\_011046841, Unigene60220\_Sample\_011046841, Unigene59362\_Sample\_011046841, Unigene20267\_Sample\_011046841, Unigene51011\_Sample\_011046841, Unigene40215\_Sample\_011046841, Unigene56391\_Sample\_011046841, Unigene47078\_Sample\_011046841, Unigene6590\_Sample\_011046841, Unigene34207\_Sample\_011046841, Unigene47910\_Sample\_011046841, Unigene10980\_Sample\_011046841, Unigene12824\_Sample\_011046841, Unigene47387\_Sample\_011046841, Unigene60733\_Sample\_011046841, Unigene46613\_Sample\_011046841, Unigene57887\_Sample\_011046841, Unigene16051\_Sample\_011046841, Unigene44734\_Sample\_011046841, Unigene24332\_Sample\_011046841, Unigene51882\_Sample\_011046841, Unigene56062\_Sample\_011046841, Unigene57665\_Sample\_011046841, Unigene30349\_Sample\_011046841, Unigene8000\_Sample\_011046841, Unigene48528\_Sample\_011046841, Unigene32534\_Sample\_011046841, Unigene55101\_Sample\_011046841, Unigene4432\_Sample\_011046841, Unigene14776\_Sample\_011046841, Unigene46198\_Sample\_011046841, Unigene11314\_Sample\_011046841, Unigene29813\_Sample\_011046841, Unigene41694\_Sample\_011046841, Unigene34757\_Sample\_011046841, Unigene3946\_Sample\_011046841, Unigene45885\_Sample\_011046841, Unigene49525\_Sample\_011046841, Unigene59711\_Sample\_011046841, Unigene13367\_Sample\_011046841, Unigene47811\_Sample\_011046841, Unigene19594\_Sample\_011046841, Unigene9774\_Sample\_011046841, Unigene58882\_Sample\_011046841, Unigene11758\_Sample\_011046841, Unigene58415\_Sample\_011046841, Unigene11504\_Sample\_011046841, Unigene58321\_Sample\_011046841, Unigene12837\_Sample\_011046841, Unigene57194\_Sample\_011046841, Unigene39083\_Sample\_011046841, Unigene11706\_Sample\_011046841, Unigene12324\_Sample\_011046841, Unigene30456\_Sample\_011046841, Unigene58180\_Sample\_011046841, Unigene3797\_Sample\_011046841, Unigene32509\_Sample\_011046841, Unigene58669\_Sample\_011046841, Unigene43935\_Sample\_011046841, Unigene50322\_Sample\_011046841, Unigene60181\_Sample\_011046841, Unigene18856\_Sample\_011046841, Unigene40491\_Sample\_011046841, Unigene56096\_Sample\_011046841, Unigene55247\_Sample\_011046841, Unigene52526\_Sample\_011046841, Unigene51736\_Sample\_011046841, Unigene47560\_Sample\_011046841, Unigene6435\_Sample\_011046841, Unigene59596\_Sample\_011046841, Unigene47686\_Sample\_011046841, Unigene54599\_Sample\_011046841, Unigene43792\_Sample\_011046841, Unigene44683\_Sample\_011046841, Unigene46783\_Sample\_011046841, Unigene11520\_Sample\_011046841, Unigene44354\_Sample\_011046841, Unigene21539\_Sample\_011046841, Unigene9210\_Sample\_011046841, Unigene57305\_Sample\_011046841, Unigene35468\_Sample\_011046841, Unigene50758\_Sample\_011046841, Unigene56828\_Sample\_011046841, Unigene6197\_Sample\_011046841, Unigene53723\_Sample\_011046841, Unigene45824\_Sample\_011046841, Unigene26728\_Sample\_011046841, Unigene47555\_Sample\_011046841, Unigene37642\_Sample\_011046841, Unigene17671\_Sample\_011046841, Unigene55564\_Sample\_011046841, Unigene51429\_Sample\_011046841, Unigene58511\_Sample\_011046841, Unigene29587\_Sample\_011046841, Unigene16046\_Sample\_011046841, Unigene23757\_Sample\_011046841, Unigene38527\_Sample\_011046841, Unigene19215\_Sample\_011046841, Unigene59868\_Sample\_011046841, Unigene48647\_Sample\_011046841, Unigene11676\_Sample\_011046841, Unigene39020\_Sample\_011046841, Unigene60217\_Sample\_011046841, Unigene9082\_Sample\_011046841, Unigene60859\_Sample\_011046841, Unigene2110\_Sample\_011046841, Unigene42561\_Sample\_011046841, Unigene50293\_Sample\_011046841 |
| hydrolase activity | Unigene51209\_Sample\_011046841, Unigene60028\_Sample\_011046841, Unigene7801\_Sample\_011046841, Unigene58451\_Sample\_011046841, Unigene57303\_Sample\_011046841, Unigene8723\_Sample\_011046841, Unigene36915\_Sample\_011046841, Unigene4700\_Sample\_011046841, Unigene5749\_Sample\_011046841, Unigene43386\_Sample\_011046841, Unigene12051\_Sample\_011046841, Unigene40117\_Sample\_011046841, Unigene6065\_Sample\_011046841, Unigene8975\_Sample\_011046841, Unigene59910\_Sample\_011046841, Unigene57691\_Sample\_011046841, Unigene13541\_Sample\_011046841, Unigene57381\_Sample\_011046841, Unigene11034\_Sample\_011046841, Unigene41395\_Sample\_011046841, Unigene24428\_Sample\_011046841, Unigene59887\_Sample\_011046841, Unigene50062\_Sample\_011046841, Unigene29849\_Sample\_011046841, Unigene16118\_Sample\_011046841, Unigene12631\_Sample\_011046841, Unigene51528\_Sample\_011046841, Unigene29658\_Sample\_011046841, Unigene55286\_Sample\_011046841, Unigene60645\_Sample\_011046841, Unigene60433\_Sample\_011046841, Unigene58969\_Sample\_011046841, Unigene47553\_Sample\_011046841, Unigene12801\_Sample\_011046841, Unigene57398\_Sample\_011046841, Unigene11319\_Sample\_011046841, Unigene40122\_Sample\_011046841, Unigene59381\_Sample\_011046841, Unigene42606\_Sample\_011046841, Unigene34169\_Sample\_011046841, Unigene28815\_Sample\_011046841, Unigene12673\_Sample\_011046841, Unigene8474\_Sample\_011046841, Unigene3264\_Sample\_011046841, Unigene31079\_Sample\_011046841, Unigene49657\_Sample\_011046841, Unigene58164\_Sample\_011046841, Unigene56375\_Sample\_011046841, Unigene21912\_Sample\_011046841, Unigene42523\_Sample\_011046841, Unigene22636\_Sample\_011046841, Unigene60129\_Sample\_011046841, Unigene54011\_Sample\_011046841, Unigene46690\_Sample\_011046841, Unigene11982\_Sample\_011046841, Unigene35744\_Sample\_011046841, Unigene6521\_Sample\_011046841, Unigene42917\_Sample\_011046841, Unigene10147\_Sample\_011046841, Unigene46283\_Sample\_011046841, Unigene51648\_Sample\_011046841, Unigene49646\_Sample\_011046841, Unigene54025\_Sample\_011046841, Unigene25226\_Sample\_011046841, Unigene60324\_Sample\_011046841, Unigene15188\_Sample\_011046841, Unigene58290\_Sample\_011046841, Unigene31101\_Sample\_011046841, Unigene43629\_Sample\_011046841, Unigene35930\_Sample\_011046841, Unigene34525\_Sample\_011046841, Unigene59077\_Sample\_011046841, Unigene33743\_Sample\_011046841, Unigene41534\_Sample\_011046841, Unigene46186\_Sample\_011046841, Unigene9770\_Sample\_011046841, Unigene6941\_Sample\_011046841, Unigene54910\_Sample\_011046841, Unigene44835\_Sample\_011046841, Unigene50220\_Sample\_011046841, Unigene54803\_Sample\_011046841, Unigene19051\_Sample\_011046841, Unigene47634\_Sample\_011046841, Unigene40677\_Sample\_011046841, Unigene23742\_Sample\_011046841, Unigene58080\_Sample\_011046841, Unigene469\_Sample\_011046841, Unigene58707\_Sample\_011046841, Unigene36243\_Sample\_011046841, Unigene33441\_Sample\_011046841, Unigene22671\_Sample\_011046841, Unigene54532\_Sample\_011046841, Unigene37222\_Sample\_011046841, Unigene20893\_Sample\_011046841, Unigene12998\_Sample\_011046841, Unigene57265\_Sample\_011046841, Unigene57271\_Sample\_011046841, Unigene57856\_Sample\_011046841, Unigene36888\_Sample\_011046841, Unigene43448\_Sample\_011046841, Unigene5195\_Sample\_011046841, Unigene39783\_Sample\_011046841, Unigene58892\_Sample\_011046841, Unigene25267\_Sample\_011046841, Unigene55132\_Sample\_011046841, Unigene43716\_Sample\_011046841, Unigene56075\_Sample\_011046841, Unigene54105\_Sample\_011046841, Unigene39868\_Sample\_011046841, Unigene31557\_Sample\_011046841, Unigene50497\_Sample\_011046841, Unigene18314\_Sample\_011046841, Unigene28897\_Sample\_011046841, Unigene5203\_Sample\_011046841, Unigene44838\_Sample\_011046841, Unigene54134\_Sample\_011046841, Unigene12390\_Sample\_011046841, Unigene19789\_Sample\_011046841, Unigene22561\_Sample\_011046841, Unigene59985\_Sample\_011046841, Unigene32259\_Sample\_011046841, Unigene40280\_Sample\_011046841, Unigene57435\_Sample\_011046841, Unigene18563\_Sample\_011046841, Unigene59199\_Sample\_011046841, Unigene25152\_Sample\_011046841, Unigene51\_Sample\_011046841, Unigene47010\_Sample\_011046841, Unigene50911\_Sample\_011046841, Unigene58409\_Sample\_011046841, Unigene35485\_Sample\_011046841, Unigene11769\_Sample\_011046841, Unigene51298\_Sample\_011046841, Unigene12379\_Sample\_011046841, Unigene59178\_Sample\_011046841, Unigene34060\_Sample\_011046841, Unigene57181\_Sample\_011046841, Unigene10027\_Sample\_011046841, Unigene56883\_Sample\_011046841, Unigene29053\_Sample\_011046841, Unigene54521\_Sample\_011046841, Unigene5795\_Sample\_011046841, Unigene56023\_Sample\_011046841, Unigene8426\_Sample\_011046841, Unigene57769\_Sample\_011046841, Unigene11373\_Sample\_011046841, Unigene21808\_Sample\_011046841, Unigene5112\_Sample\_011046841, Unigene13733\_Sample\_011046841, Unigene46363\_Sample\_011046841, Unigene4082\_Sample\_011046841, Unigene52053\_Sample\_011046841, Unigene44173\_Sample\_011046841, Unigene60444\_Sample\_011046841, Unigene33186\_Sample\_011046841, Unigene36927\_Sample\_011046841, Unigene59986\_Sample\_011046841, Unigene56688\_Sample\_011046841, Unigene15862\_Sample\_011046841, Unigene7282\_Sample\_011046841, Unigene43072\_Sample\_011046841, Unigene60016\_Sample\_011046841, Unigene7340\_Sample\_011046841, Unigene3454\_Sample\_011046841, Unigene36922\_Sample\_011046841, Unigene46697\_Sample\_011046841, Unigene55433\_Sample\_011046841, Unigene50798\_Sample\_011046841, Unigene15548\_Sample\_011046841, Unigene1228\_Sample\_011046841, Unigene11028\_Sample\_011046841, Unigene7908\_Sample\_011046841, Unigene32529\_Sample\_011046841, Unigene49552\_Sample\_011046841, Unigene59067\_Sample\_011046841, Unigene59622\_Sample\_011046841, Unigene13241\_Sample\_011046841, Unigene26679\_Sample\_011046841, Unigene31586\_Sample\_011046841, Unigene50585\_Sample\_011046841, Unigene36271\_Sample\_011046841, Unigene32034\_Sample\_011046841, Unigene60049\_Sample\_011046841, Unigene56444\_Sample\_011046841, Unigene55848\_Sample\_011046841, Unigene32666\_Sample\_011046841, Unigene46558\_Sample\_011046841, Unigene44069\_Sample\_011046841, Unigene53884\_Sample\_011046841, Unigene52462\_Sample\_011046841, Unigene52573\_Sample\_011046841, Unigene18506\_Sample\_011046841, Unigene31438\_Sample\_011046841, Unigene51857\_Sample\_011046841, Unigene47023\_Sample\_011046841, Unigene54331\_Sample\_011046841, Unigene50910\_Sample\_011046841, Unigene38784\_Sample\_011046841, Unigene48764\_Sample\_011046841, Unigene50609\_Sample\_011046841, Unigene45737\_Sample\_011046841, Unigene21988\_Sample\_011046841, Unigene47783\_Sample\_011046841, Unigene42255\_Sample\_011046841, Unigene51524\_Sample\_011046841, Unigene13969\_Sample\_011046841, Unigene27203\_Sample\_011046841, Unigene55180\_Sample\_011046841, Unigene59410\_Sample\_011046841, Unigene59445\_Sample\_011046841, Unigene3527\_Sample\_011046841, Unigene20854\_Sample\_011046841, Unigene59709\_Sample\_011046841, Unigene59685\_Sample\_011046841, Unigene57616\_Sample\_011046841, Unigene13571\_Sample\_011046841, Unigene42062\_Sample\_011046841, Unigene10446\_Sample\_011046841, Unigene48242\_Sample\_011046841, Unigene48637\_Sample\_011046841, Unigene38510\_Sample\_011046841, Unigene39572\_Sample\_011046841, Unigene28246\_Sample\_011046841, Unigene53605\_Sample\_011046841, Unigene18385\_Sample\_011046841, Unigene59219\_Sample\_011046841, Unigene34524\_Sample\_011046841, Unigene60926\_Sample\_011046841, Unigene19495\_Sample\_011046841, Unigene13826\_Sample\_011046841, Unigene7789\_Sample\_011046841, Unigene58627\_Sample\_011046841, Unigene26696\_Sample\_011046841, Unigene8612\_Sample\_011046841, Unigene44387\_Sample\_011046841, Unigene34380\_Sample\_011046841, Unigene8165\_Sample\_011046841, Unigene45838\_Sample\_011046841, Unigene31773\_Sample\_011046841, Unigene24508\_Sample\_011046841, Unigene44248\_Sample\_011046841, Unigene30162\_Sample\_011046841, Unigene12142\_Sample\_011046841, Unigene21699\_Sample\_011046841, Unigene54214\_Sample\_011046841, Unigene17370\_Sample\_011046841, Unigene21063\_Sample\_011046841, Unigene27305\_Sample\_011046841, Unigene43875\_Sample\_011046841, Unigene51253\_Sample\_011046841, Unigene38251\_Sample\_011046841, Unigene55553\_Sample\_011046841, Unigene20399\_Sample\_011046841, Unigene43848\_Sample\_011046841, Unigene48422\_Sample\_011046841, Unigene51431\_Sample\_011046841, Unigene51559\_Sample\_011046841, Unigene9658\_Sample\_011046841, Unigene58303\_Sample\_011046841, Unigene58077\_Sample\_011046841, Unigene57339\_Sample\_011046841, Unigene35387\_Sample\_011046841, Unigene53327\_Sample\_011046841, Unigene11522\_Sample\_011046841, Unigene6164\_Sample\_011046841, Unigene31498\_Sample\_011046841, Unigene60892\_Sample\_011046841, Unigene50611\_Sample\_011046841, Unigene48786\_Sample\_011046841, Unigene33091\_Sample\_011046841, Unigene60469\_Sample\_011046841, Unigene33643\_Sample\_011046841, Unigene14918\_Sample\_011046841, Unigene5865\_Sample\_011046841, Unigene12056\_Sample\_011046841, Unigene39753\_Sample\_011046841, Unigene15776\_Sample\_011046841, Unigene47693\_Sample\_011046841, Unigene46743\_Sample\_011046841, Unigene41352\_Sample\_011046841, Unigene23180\_Sample\_011046841, Unigene14532\_Sample\_011046841, Unigene17523\_Sample\_011046841, Unigene31483\_Sample\_011046841, Unigene9773\_Sample\_011046841, Unigene9734\_Sample\_011046841, Unigene58720\_Sample\_011046841, Unigene58870\_Sample\_011046841, Unigene47261\_Sample\_011046841, Unigene13043\_Sample\_011046841, Unigene7446\_Sample\_011046841, Unigene60385\_Sample\_011046841, Unigene57943\_Sample\_011046841, Unigene49720\_Sample\_011046841, Unigene47778\_Sample\_011046841, Unigene19947\_Sample\_011046841, Unigene56358\_Sample\_011046841, Unigene59218\_Sample\_011046841, Unigene10934\_Sample\_011046841, Unigene54246\_Sample\_011046841, Unigene44453\_Sample\_011046841, Unigene28737\_Sample\_011046841, Unigene8603\_Sample\_011046841, Unigene8418\_Sample\_011046841, Unigene18126\_Sample\_011046841, Unigene16760\_Sample\_011046841, Unigene59831\_Sample\_011046841, Unigene2189\_Sample\_011046841, Unigene47308\_Sample\_011046841, Unigene38963\_Sample\_011046841, Unigene40558\_Sample\_011046841, Unigene33507\_Sample\_011046841, Unigene49928\_Sample\_011046841, Unigene46509\_Sample\_011046841, Unigene50302\_Sample\_011046841, Unigene34034\_Sample\_011046841, Unigene41996\_Sample\_011046841, Unigene58900\_Sample\_011046841, Unigene52689\_Sample\_011046841, Unigene14355\_Sample\_011046841, Unigene10201\_Sample\_011046841, Unigene41447\_Sample\_011046841, Unigene40840\_Sample\_011046841, Unigene28390\_Sample\_011046841, Unigene11213\_Sample\_011046841, Unigene45012\_Sample\_011046841, Unigene46704\_Sample\_011046841, Unigene28804\_Sample\_011046841, Unigene48409\_Sample\_011046841, Unigene22255\_Sample\_011046841, Unigene59662\_Sample\_011046841, Unigene10941\_Sample\_011046841, Unigene51110\_Sample\_011046841, Unigene47129\_Sample\_011046841, Unigene56517\_Sample\_011046841, Unigene44438\_Sample\_011046841, Unigene26621\_Sample\_011046841, Unigene57298\_Sample\_011046841, Unigene26111\_Sample\_011046841, Unigene52901\_Sample\_011046841, Unigene45107\_Sample\_011046841, Unigene27907\_Sample\_011046841, Unigene57239\_Sample\_011046841, Unigene57950\_Sample\_011046841, Unigene51024\_Sample\_011046841, Unigene57410\_Sample\_011046841, Unigene8859\_Sample\_011046841, Unigene55335\_Sample\_011046841, Unigene59387\_Sample\_011046841, Unigene46973\_Sample\_011046841, Unigene5740\_Sample\_011046841, Unigene23566\_Sample\_011046841, Unigene54378\_Sample\_011046841, Unigene48227\_Sample\_011046841, Unigene54288\_Sample\_011046841, Unigene54763\_Sample\_011046841, Unigene12558\_Sample\_011046841, Unigene55719\_Sample\_011046841, Unigene12894\_Sample\_011046841, Unigene24234\_Sample\_011046841, Unigene49615\_Sample\_011046841, Unigene42768\_Sample\_011046841, Unigene56344\_Sample\_011046841, Unigene59788\_Sample\_011046841, Unigene45059\_Sample\_011046841, Unigene18137\_Sample\_011046841, Unigene46603\_Sample\_011046841, Unigene22655\_Sample\_011046841, Unigene49151\_Sample\_011046841, Unigene15292\_Sample\_011046841, Unigene9067\_Sample\_011046841, Unigene3946\_Sample\_011046841, Unigene33958\_Sample\_011046841, Unigene44181\_Sample\_011046841, Unigene53168\_Sample\_011046841, Unigene32169\_Sample\_011046841, Unigene53609\_Sample\_011046841, Unigene11971\_Sample\_011046841, Unigene19594\_Sample\_011046841, Unigene48645\_Sample\_011046841, Unigene28864\_Sample\_011046841, Unigene59189\_Sample\_011046841, Unigene22492\_Sample\_011046841, Unigene13675\_Sample\_011046841, Unigene46688\_Sample\_011046841, Unigene55205\_Sample\_011046841, Unigene1594\_Sample\_011046841, Unigene56630\_Sample\_011046841, Unigene36905\_Sample\_011046841, Unigene59156\_Sample\_011046841, Unigene22022\_Sample\_011046841, Unigene26904\_Sample\_011046841, Unigene11592\_Sample\_011046841, Unigene25827\_Sample\_011046841, Unigene3620\_Sample\_011046841, Unigene35064\_Sample\_011046841, Unigene25248\_Sample\_011046841, Unigene24381\_Sample\_011046841, Unigene60871\_Sample\_011046841, Unigene48977\_Sample\_011046841, Unigene51161\_Sample\_011046841, Unigene47220\_Sample\_011046841, Unigene49653\_Sample\_011046841, Unigene29081\_Sample\_011046841, Unigene29267\_Sample\_011046841, Unigene27881\_Sample\_011046841, Unigene55144\_Sample\_011046841, Unigene5518\_Sample\_011046841, Unigene58475\_Sample\_011046841, Unigene53063\_Sample\_011046841, Unigene11354\_Sample\_011046841, Unigene48807\_Sample\_011046841, Unigene52025\_Sample\_011046841, Unigene30814\_Sample\_011046841, Unigene42071\_Sample\_011046841, Unigene25210\_Sample\_011046841, Unigene36308\_Sample\_011046841, Unigene46290\_Sample\_011046841, Unigene13106\_Sample\_011046841, Unigene32213\_Sample\_011046841, Unigene35537\_Sample\_011046841, Unigene13390\_Sample\_011046841, Unigene57579\_Sample\_011046841, Unigene54823\_Sample\_011046841, Unigene53095\_Sample\_011046841, Unigene21814\_Sample\_011046841, Unigene36211\_Sample\_011046841, Unigene8489\_Sample\_011046841, Unigene4148\_Sample\_011046841, Unigene54593\_Sample\_011046841, Unigene39483\_Sample\_011046841, Unigene15933\_Sample\_011046841, Unigene48006\_Sample\_011046841, Unigene11042\_Sample\_011046841, Unigene53548\_Sample\_011046841, Unigene48720\_Sample\_011046841, Unigene44005\_Sample\_011046841, Unigene13229\_Sample\_011046841, Unigene12209\_Sample\_011046841, Unigene24100\_Sample\_011046841, Unigene11722\_Sample\_011046841, Unigene53262\_Sample\_011046841, Unigene57719\_Sample\_011046841, Unigene43358\_Sample\_011046841, Unigene54668\_Sample\_011046841, Unigene44183\_Sample\_011046841, Unigene44282\_Sample\_011046841, Unigene33928\_Sample\_011046841, Unigene16995\_Sample\_011046841, Unigene11593\_Sample\_011046841, Unigene12929\_Sample\_011046841, Unigene13245\_Sample\_011046841, Unigene47012\_Sample\_011046841, Unigene58432\_Sample\_011046841, Unigene34039\_Sample\_011046841, Unigene52003\_Sample\_011046841, Unigene12157\_Sample\_011046841, Unigene42552\_Sample\_011046841, Unigene247\_Sample\_011046841, Unigene47859\_Sample\_011046841, Unigene11219\_Sample\_011046841, Unigene42954\_Sample\_011046841, Unigene32707\_Sample\_011046841, Unigene10000\_Sample\_011046841, Unigene34550\_Sample\_011046841, Unigene60358\_Sample\_011046841, Unigene58810\_Sample\_011046841, Unigene37790\_Sample\_011046841, Unigene52821\_Sample\_011046841, Unigene35806\_Sample\_011046841, Unigene3261\_Sample\_011046841, Unigene58463\_Sample\_011046841, Unigene56622\_Sample\_011046841, Unigene2372\_Sample\_011046841, Unigene53417\_Sample\_011046841, Unigene54701\_Sample\_011046841, Unigene45440\_Sample\_011046841, Unigene57154\_Sample\_011046841, Unigene3975\_Sample\_011046841, Unigene21731\_Sample\_011046841, Unigene7726\_Sample\_011046841, Unigene43044\_Sample\_011046841, Unigene47780\_Sample\_011046841, Unigene46546\_Sample\_011046841, Unigene48687\_Sample\_011046841, Unigene59925\_Sample\_011046841, Unigene2068\_Sample\_011046841, Unigene43474\_Sample\_011046841, Unigene45493\_Sample\_011046841, Unigene5909\_Sample\_011046841, Unigene55844\_Sample\_011046841, Unigene50178\_Sample\_011046841, Unigene58422\_Sample\_011046841, Unigene26327\_Sample\_011046841, Unigene41737\_Sample\_011046841, Unigene58462\_Sample\_011046841, Unigene37665\_Sample\_011046841, Unigene47997\_Sample\_011046841, Unigene59452\_Sample\_011046841, Unigene22548\_Sample\_011046841, Unigene55151\_Sample\_011046841, Unigene36984\_Sample\_011046841, Unigene8035\_Sample\_011046841, Unigene25101\_Sample\_011046841, Unigene60571\_Sample\_011046841, Unigene17012\_Sample\_011046841, Unigene20390\_Sample\_011046841, Unigene10886\_Sample\_011046841, Unigene11865\_Sample\_011046841, Unigene58907\_Sample\_011046841, Unigene39978\_Sample\_011046841, Unigene60879\_Sample\_011046841, Unigene58199\_Sample\_011046841, Unigene60138\_Sample\_011046841, Unigene47625\_Sample\_011046841, Unigene58481\_Sample\_011046841, Unigene42127\_Sample\_011046841, Unigene54315\_Sample\_011046841, Unigene11799\_Sample\_011046841, Unigene24751\_Sample\_011046841, Unigene19937\_Sample\_011046841, Unigene44697\_Sample\_011046841, Unigene8647\_Sample\_011046841, Unigene7900\_Sample\_011046841, Unigene42564\_Sample\_011046841, Unigene57567\_Sample\_011046841, Unigene60964\_Sample\_011046841, Unigene54401\_Sample\_011046841, Unigene31466\_Sample\_011046841, Unigene33988\_Sample\_011046841, Unigene59378\_Sample\_011046841, Unigene6116\_Sample\_011046841, Unigene4702\_Sample\_011046841, Unigene49955\_Sample\_011046841, Unigene49661\_Sample\_011046841, Unigene47577\_Sample\_011046841, Unigene13044\_Sample\_011046841, Unigene59614\_Sample\_011046841, Unigene41239\_Sample\_011046841, Unigene27718\_Sample\_011046841, Unigene34582\_Sample\_011046841, Unigene33253\_Sample\_011046841, Unigene30454\_Sample\_011046841, Unigene49125\_Sample\_011046841, Unigene13151\_Sample\_011046841, Unigene32470\_Sample\_011046841, Unigene60189\_Sample\_011046841, Unigene8128\_Sample\_011046841, Unigene47083\_Sample\_011046841, Unigene37765\_Sample\_011046841, Unigene54098\_Sample\_011046841, Unigene42395\_Sample\_011046841, Unigene12950\_Sample\_011046841, Unigene58587\_Sample\_011046841, Unigene50245\_Sample\_011046841, Unigene12796\_Sample\_011046841, Unigene43654\_Sample\_011046841, Unigene32292\_Sample\_011046841, Unigene35141\_Sample\_011046841, Unigene58531\_Sample\_011046841, Unigene40974\_Sample\_011046841, Unigene13214\_Sample\_011046841, Unigene5937\_Sample\_011046841, Unigene55251\_Sample\_011046841, Unigene2569\_Sample\_011046841, Unigene59214\_Sample\_011046841, Unigene22016\_Sample\_011046841, Unigene56125\_Sample\_011046841, Unigene52104\_Sample\_011046841, Unigene22389\_Sample\_011046841, Unigene60136\_Sample\_011046841, Unigene38547\_Sample\_011046841, Unigene13643\_Sample\_011046841, Unigene11749\_Sample\_011046841, Unigene47903\_Sample\_011046841, Unigene26192\_Sample\_011046841, Unigene18541\_Sample\_011046841, Unigene23701\_Sample\_011046841, Unigene44813\_Sample\_011046841, Unigene18027\_Sample\_011046841, Unigene13686\_Sample\_011046841, Unigene29740\_Sample\_011046841, Unigene56818\_Sample\_011046841, Unigene33661\_Sample\_011046841, Unigene39306\_Sample\_011046841, Unigene30373\_Sample\_011046841, Unigene13697\_Sample\_011046841, Unigene59540\_Sample\_011046841, Unigene11755\_Sample\_011046841, Unigene47099\_Sample\_011046841, Unigene60354\_Sample\_011046841, Unigene14181\_Sample\_011046841, Unigene2359\_Sample\_011046841, Unigene47518\_Sample\_011046841, Unigene51201\_Sample\_011046841, Unigene45141\_Sample\_011046841, Unigene43235\_Sample\_011046841, Unigene43698\_Sample\_011046841, Unigene19399\_Sample\_011046841, Unigene35836\_Sample\_011046841, Unigene3393\_Sample\_011046841, Unigene9711\_Sample\_011046841, Unigene5263\_Sample\_011046841, Unigene47428\_Sample\_011046841, Unigene57575\_Sample\_011046841, Unigene55391\_Sample\_011046841, Unigene54619\_Sample\_011046841, Unigene517\_Sample\_011046841, Unigene58620\_Sample\_011046841, Unigene17873\_Sample\_011046841, Unigene58936\_Sample\_011046841, Unigene52071\_Sample\_011046841, Unigene22285\_Sample\_011046841, Unigene60399\_Sample\_011046841, Unigene57530\_Sample\_011046841, Unigene58697\_Sample\_011046841, Unigene35464\_Sample\_011046841, Unigene49087\_Sample\_011046841, Unigene51891\_Sample\_011046841, Unigene33437\_Sample\_011046841, Unigene43487\_Sample\_011046841, Unigene2371\_Sample\_011046841, Unigene27092\_Sample\_011046841, Unigene45986\_Sample\_011046841, Unigene51426\_Sample\_011046841, Unigene49205\_Sample\_011046841, Unigene12370\_Sample\_011046841, Unigene49894\_Sample\_011046841, Unigene14915\_Sample\_011046841, Unigene9845\_Sample\_011046841, Unigene39805\_Sample\_011046841, Unigene53768\_Sample\_011046841, Unigene10041\_Sample\_011046841, Unigene57375\_Sample\_011046841, Unigene27589\_Sample\_011046841, Unigene13386\_Sample\_011046841, Unigene16556\_Sample\_011046841, Unigene11536\_Sample\_011046841, Unigene6340\_Sample\_011046841, Unigene33662\_Sample\_011046841, Unigene59915\_Sample\_011046841, Unigene4599\_Sample\_011046841, Unigene60450\_Sample\_011046841, Unigene41220\_Sample\_011046841, Unigene57113\_Sample\_011046841, Unigene42437\_Sample\_011046841, Unigene49570\_Sample\_011046841, Unigene39833\_Sample\_011046841, Unigene13430\_Sample\_011046841, Unigene35922\_Sample\_011046841, Unigene27453\_Sample\_011046841, Unigene40008\_Sample\_011046841, Unigene24353\_Sample\_011046841, Unigene57629\_Sample\_011046841, Unigene7404\_Sample\_011046841, Unigene18638\_Sample\_011046841, Unigene57039\_Sample\_011046841, Unigene13603\_Sample\_011046841, Unigene49063\_Sample\_011046841, Unigene16224\_Sample\_011046841, Unigene40534\_Sample\_011046841, Unigene13505\_Sample\_011046841, Unigene25759\_Sample\_011046841, Unigene19743\_Sample\_011046841, Unigene45801\_Sample\_011046841, Unigene49426\_Sample\_011046841, Unigene9271\_Sample\_011046841, Unigene26015\_Sample\_011046841, Unigene54524\_Sample\_011046841, Unigene49869\_Sample\_011046841, Unigene59945\_Sample\_011046841, Unigene38234\_Sample\_011046841, Unigene45945\_Sample\_011046841, Unigene28275\_Sample\_011046841, Unigene44479\_Sample\_011046841, Unigene46287\_Sample\_011046841, Unigene57997\_Sample\_011046841, Unigene19044\_Sample\_011046841, Unigene58354\_Sample\_011046841, Unigene12061\_Sample\_011046841, Unigene60614\_Sample\_011046841, Unigene10744\_Sample\_011046841, Unigene32914\_Sample\_011046841, Unigene58224\_Sample\_011046841, Unigene19437\_Sample\_011046841, Unigene30077\_Sample\_011046841, Unigene42233\_Sample\_011046841, Unigene24944\_Sample\_011046841, Unigene8943\_Sample\_011046841, Unigene55877\_Sample\_011046841, Unigene56281\_Sample\_011046841, Unigene12284\_Sample\_011046841, Unigene47025\_Sample\_011046841, Unigene17067\_Sample\_011046841, Unigene12447\_Sample\_011046841, Unigene53326\_Sample\_011046841, Unigene37422\_Sample\_011046841, Unigene45191\_Sample\_011046841, Unigene11435\_Sample\_011046841, Unigene6709\_Sample\_011046841, Unigene18596\_Sample\_011046841, Unigene60906\_Sample\_011046841, Unigene55856\_Sample\_011046841, Unigene54697\_Sample\_011046841, Unigene57627\_Sample\_011046841, Unigene41124\_Sample\_011046841, Unigene53685\_Sample\_011046841, Unigene57757\_Sample\_011046841, Unigene59880\_Sample\_011046841, Unigene1989\_Sample\_011046841, Unigene44849\_Sample\_011046841, Unigene55579\_Sample\_011046841, Unigene31656\_Sample\_011046841, Unigene39667\_Sample\_011046841, Unigene47795\_Sample\_011046841, Unigene53587\_Sample\_011046841, Unigene60946\_Sample\_011046841, Unigene42728\_Sample\_011046841, Unigene51811\_Sample\_011046841, Unigene6203\_Sample\_011046841, Unigene46337\_Sample\_011046841, Unigene8924\_Sample\_011046841, Unigene48217\_Sample\_011046841, Unigene11539\_Sample\_011046841, Unigene10317\_Sample\_011046841, Unigene59021\_Sample\_011046841, Unigene13860\_Sample\_011046841, Unigene32973\_Sample\_011046841, Unigene58341\_Sample\_011046841, Unigene8034\_Sample\_011046841, Unigene45754\_Sample\_011046841, Unigene37228\_Sample\_011046841, Unigene18963\_Sample\_011046841, Unigene56293\_Sample\_011046841, Unigene6757\_Sample\_011046841, Unigene55520\_Sample\_011046841, Unigene13976\_Sample\_011046841, Unigene56191\_Sample\_011046841, Unigene54624\_Sample\_011046841, Unigene6153\_Sample\_011046841, Unigene53718\_Sample\_011046841, Unigene6828\_Sample\_011046841, Unigene55613\_Sample\_011046841, Unigene60036\_Sample\_011046841, Unigene13878\_Sample\_011046841, Unigene28106\_Sample\_011046841, Unigene49255\_Sample\_011046841, Unigene6497\_Sample\_011046841, Unigene59619\_Sample\_011046841, Unigene49462\_Sample\_011046841, Unigene56356\_Sample\_011046841, Unigene57480\_Sample\_011046841, Unigene40287\_Sample\_011046841, Unigene53498\_Sample\_011046841, Unigene33032\_Sample\_011046841, Unigene30654\_Sample\_011046841, Unigene55781\_Sample\_011046841, Unigene24566\_Sample\_011046841, Unigene60488\_Sample\_011046841, Unigene9238\_Sample\_011046841, Unigene18129\_Sample\_011046841, Unigene60910\_Sample\_011046841, Unigene54507\_Sample\_011046841, Unigene46515\_Sample\_011046841, Unigene10236\_Sample\_011046841, Unigene11093\_Sample\_011046841, Unigene49397\_Sample\_011046841, Unigene58653\_Sample\_011046841, Unigene25820\_Sample\_011046841, Unigene8590\_Sample\_011046841, Unigene29977\_Sample\_011046841, Unigene60095\_Sample\_011046841, Unigene48473\_Sample\_011046841, Unigene52532\_Sample\_011046841, Unigene50528\_Sample\_011046841, Unigene34247\_Sample\_011046841, Unigene38860\_Sample\_011046841, Unigene9679\_Sample\_011046841, Unigene22474\_Sample\_011046841, Unigene42343\_Sample\_011046841, Unigene10327\_Sample\_011046841, Unigene59747\_Sample\_011046841, Unigene19113\_Sample\_011046841, Unigene53531\_Sample\_011046841, Unigene2743\_Sample\_011046841, Unigene53956\_Sample\_011046841, Unigene54414\_Sample\_011046841, Unigene60887\_Sample\_011046841, Unigene3252\_Sample\_011046841, Unigene29575\_Sample\_011046841, Unigene59687\_Sample\_011046841, Unigene20785\_Sample\_011046841, Unigene42236\_Sample\_011046841, Unigene48097\_Sample\_011046841, Unigene26460\_Sample\_011046841, Unigene52423\_Sample\_011046841, Unigene45483\_Sample\_011046841, Unigene40874\_Sample\_011046841, Unigene23431\_Sample\_011046841, Unigene55936\_Sample\_011046841, Unigene30736\_Sample\_011046841, Unigene11478\_Sample\_011046841, Unigene10518\_Sample\_011046841, Unigene12010\_Sample\_011046841, Unigene19526\_Sample\_011046841, Unigene31905\_Sample\_011046841, Unigene55930\_Sample\_011046841, Unigene16353\_Sample\_011046841, Unigene50296\_Sample\_011046841, Unigene36839\_Sample\_011046841, Unigene57929\_Sample\_011046841, Unigene46799\_Sample\_011046841, Unigene50708\_Sample\_011046841, Unigene42339\_Sample\_011046841, Unigene24152\_Sample\_011046841, Unigene56185\_Sample\_011046841, Unigene42123\_Sample\_011046841, Unigene60958\_Sample\_011046841, Unigene92\_Sample\_011046841, Unigene56437\_Sample\_011046841, Unigene19570\_Sample\_011046841, Unigene56027\_Sample\_011046841, Unigene57853\_Sample\_011046841, Unigene58649\_Sample\_011046841, Unigene29345\_Sample\_011046841, Unigene8229\_Sample\_011046841, Unigene49840\_Sample\_011046841, Unigene6900\_Sample\_011046841, Unigene42368\_Sample\_011046841, Unigene50660\_Sample\_011046841, Unigene13912\_Sample\_011046841, Unigene18715\_Sample\_011046841, Unigene45876\_Sample\_011046841, Unigene41781\_Sample\_011046841, Unigene58180\_Sample\_011046841, Unigene19792\_Sample\_011046841, Unigene4749\_Sample\_011046841, Unigene56389\_Sample\_011046841, Unigene58095\_Sample\_011046841, Unigene44848\_Sample\_011046841, Unigene44301\_Sample\_011046841, Unigene51437\_Sample\_011046841, Unigene12492\_Sample\_011046841, Unigene17584\_Sample\_011046841, Unigene41153\_Sample\_011046841, Unigene11923\_Sample\_011046841, Unigene27258\_Sample\_011046841, Unigene55470\_Sample\_011046841, Unigene60231\_Sample\_011046841, Unigene58799\_Sample\_011046841, Unigene1748\_Sample\_011046841, Unigene45824\_Sample\_011046841, Unigene31228\_Sample\_011046841, Unigene43331\_Sample\_011046841, Unigene54217\_Sample\_011046841, Unigene42265\_Sample\_011046841, Unigene51091\_Sample\_011046841, Unigene52367\_Sample\_011046841, Unigene10421\_Sample\_011046841, Unigene55379\_Sample\_011046841, Unigene36037\_Sample\_011046841, Unigene33567\_Sample\_011046841, Unigene4883\_Sample\_011046841, Unigene51231\_Sample\_011046841, Unigene22290\_Sample\_011046841, Unigene46829\_Sample\_011046841, Unigene50399\_Sample\_011046841, Unigene53997\_Sample\_011046841, Unigene22682\_Sample\_011046841, Unigene57490\_Sample\_011046841, Unigene19912\_Sample\_011046841, Unigene50036\_Sample\_011046841, Unigene40921\_Sample\_011046841, Unigene45898\_Sample\_011046841, Unigene59557\_Sample\_011046841, Unigene36949\_Sample\_011046841, Unigene41538\_Sample\_011046841, Unigene32500\_Sample\_011046841, Unigene56288\_Sample\_011046841, Unigene53011\_Sample\_011046841, Unigene10461\_Sample\_011046841, Unigene19375\_Sample\_011046841, Unigene31772\_Sample\_011046841, Unigene57562\_Sample\_011046841, Unigene57119\_Sample\_011046841, Unigene6973\_Sample\_011046841, Unigene9991\_Sample\_011046841, Unigene55226\_Sample\_011046841, Unigene56301\_Sample\_011046841, Unigene54581\_Sample\_011046841, Unigene15270\_Sample\_011046841, Unigene37696\_Sample\_011046841, Unigene6061\_Sample\_011046841, Unigene48095\_Sample\_011046841, Unigene45858\_Sample\_011046841, Unigene59633\_Sample\_011046841, Unigene48415\_Sample\_011046841, Unigene53259\_Sample\_011046841, Unigene26425\_Sample\_011046841, Unigene19575\_Sample\_011046841, Unigene20921\_Sample\_011046841, Unigene475\_Sample\_011046841, Unigene46219\_Sample\_011046841, Unigene57724\_Sample\_011046841, Unigene12830\_Sample\_011046841, Unigene47947\_Sample\_011046841, Unigene45808\_Sample\_011046841, Unigene43064\_Sample\_011046841, Unigene34114\_Sample\_011046841, Unigene46561\_Sample\_011046841, Unigene49046\_Sample\_011046841, Unigene7796\_Sample\_011046841, Unigene8020\_Sample\_011046841, Unigene31768\_Sample\_011046841, Unigene52963\_Sample\_011046841, Unigene39821\_Sample\_011046841, Unigene3235\_Sample\_011046841, Unigene60032\_Sample\_011046841, Unigene9122\_Sample\_011046841, Unigene55156\_Sample\_011046841, Unigene43846\_Sample\_011046841, Unigene12507\_Sample\_011046841, Unigene60452\_Sample\_011046841, Unigene43340\_Sample\_011046841, Unigene49014\_Sample\_011046841, Unigene20899\_Sample\_011046841, Unigene51076\_Sample\_011046841, Unigene58924\_Sample\_011046841, Unigene31416\_Sample\_011046841, Unigene46245\_Sample\_011046841, Unigene27784\_Sample\_011046841, Unigene55052\_Sample\_011046841, Unigene37427\_Sample\_011046841, Unigene8939\_Sample\_011046841, Unigene49837\_Sample\_011046841, Unigene26119\_Sample\_011046841, Unigene21282\_Sample\_011046841, Unigene39762\_Sample\_011046841, Unigene43456\_Sample\_011046841, Unigene39875\_Sample\_011046841, Unigene34414\_Sample\_011046841, Unigene50712\_Sample\_011046841, Unigene32216\_Sample\_011046841, Unigene14069\_Sample\_011046841, Unigene60937\_Sample\_011046841, Unigene51782\_Sample\_011046841, Unigene10525\_Sample\_011046841, Unigene47562\_Sample\_011046841, Unigene13436\_Sample\_011046841, Unigene20745\_Sample\_011046841, Unigene13799\_Sample\_011046841, Unigene46746\_Sample\_011046841, Unigene29524\_Sample\_011046841, Unigene58519\_Sample\_011046841, Unigene354\_Sample\_011046841, Unigene50009\_Sample\_011046841, Unigene4293\_Sample\_011046841, Unigene55036\_Sample\_011046841, Unigene8290\_Sample\_011046841, Unigene49028\_Sample\_011046841, Unigene59080\_Sample\_011046841, Unigene60388\_Sample\_011046841, Unigene30073\_Sample\_011046841, Unigene20434\_Sample\_011046841, Unigene12344\_Sample\_011046841, Unigene35626\_Sample\_011046841, Unigene53419\_Sample\_011046841, Unigene46638\_Sample\_011046841, Unigene42378\_Sample\_011046841, Unigene42026\_Sample\_011046841, Unigene33587\_Sample\_011046841, Unigene60561\_Sample\_011046841, Unigene23207\_Sample\_011046841, Unigene24493\_Sample\_011046841, Unigene46243\_Sample\_011046841, Unigene47868\_Sample\_011046841, Unigene12840\_Sample\_011046841, Unigene59333\_Sample\_011046841, Unigene13689\_Sample\_011046841, Unigene55717\_Sample\_011046841, Unigene11794\_Sample\_011046841, Unigene3358\_Sample\_011046841, Unigene4434\_Sample\_011046841, Unigene5615\_Sample\_011046841, Unigene45476\_Sample\_011046841, Unigene22760\_Sample\_011046841, Unigene58596\_Sample\_011046841, Unigene44244\_Sample\_011046841, Unigene18426\_Sample\_011046841, Unigene43804\_Sample\_011046841, Unigene55575\_Sample\_011046841, Unigene48524\_Sample\_011046841, Unigene11283\_Sample\_011046841, Unigene35368\_Sample\_011046841, Unigene54290\_Sample\_011046841, Unigene36631\_Sample\_011046841, Unigene19043\_Sample\_011046841, Unigene54410\_Sample\_011046841, Unigene48170\_Sample\_011046841, Unigene40858\_Sample\_011046841, Unigene9059\_Sample\_011046841, Unigene54937\_Sample\_011046841, Unigene3247\_Sample\_011046841, Unigene47540\_Sample\_011046841, Unigene59567\_Sample\_011046841, Unigene10615\_Sample\_011046841, Unigene60123\_Sample\_011046841, Unigene12614\_Sample\_011046841, Unigene12869\_Sample\_011046841, Unigene59595\_Sample\_011046841, Unigene53431\_Sample\_011046841, Unigene16650\_Sample\_011046841, Unigene35737\_Sample\_011046841, Unigene45620\_Sample\_011046841, Unigene47042\_Sample\_011046841, Unigene2509\_Sample\_011046841, Unigene54891\_Sample\_011046841, Unigene6178\_Sample\_011046841, Unigene55921\_Sample\_011046841, Unigene47487\_Sample\_011046841, Unigene24692\_Sample\_011046841, Unigene59621\_Sample\_011046841, Unigene58026\_Sample\_011046841, Unigene50223\_Sample\_011046841, Unigene26723\_Sample\_011046841, Unigene19292\_Sample\_011046841, Unigene57809\_Sample\_011046841, Unigene23312\_Sample\_011046841, Unigene39991\_Sample\_011046841, Unigene38618\_Sample\_011046841, Unigene54627\_Sample\_011046841, Unigene59118\_Sample\_011046841, Unigene39842\_Sample\_011046841, Unigene7532\_Sample\_011046841, Unigene35963\_Sample\_011046841, Unigene18725\_Sample\_011046841, Unigene39276\_Sample\_011046841, Unigene58480\_Sample\_011046841, Unigene51260\_Sample\_011046841, Unigene59257\_Sample\_011046841, Unigene59512\_Sample\_011046841, Unigene56611\_Sample\_011046841, Unigene34850\_Sample\_011046841, Unigene43704\_Sample\_011046841, Unigene53916\_Sample\_011046841, Unigene41992\_Sample\_011046841, Unigene29966\_Sample\_011046841, Unigene54829\_Sample\_011046841, Unigene27641\_Sample\_011046841, Unigene57323\_Sample\_011046841, Unigene17723\_Sample\_011046841, Unigene60586\_Sample\_011046841, Unigene32556\_Sample\_011046841, Unigene30267\_Sample\_011046841, Unigene1306\_Sample\_011046841, Unigene56177\_Sample\_011046841, Unigene59074\_Sample\_011046841, Unigene50596\_Sample\_011046841, Unigene56198\_Sample\_011046841, Unigene56105\_Sample\_011046841, Unigene3977\_Sample\_011046841, Unigene204\_Sample\_011046841, Unigene41631\_Sample\_011046841, Unigene51369\_Sample\_011046841, Unigene23568\_Sample\_011046841, Unigene57236\_Sample\_011046841, Unigene39808\_Sample\_011046841, Unigene19418\_Sample\_011046841, Unigene39921\_Sample\_011046841, Unigene59323\_Sample\_011046841, Unigene17449\_Sample\_011046841, Unigene47907\_Sample\_011046841, Unigene33400\_Sample\_011046841, Unigene60225\_Sample\_011046841, Unigene20487\_Sample\_011046841, Unigene51737\_Sample\_011046841, Unigene35800\_Sample\_011046841, Unigene55828\_Sample\_011046841, Unigene43989\_Sample\_011046841, Unigene32629\_Sample\_011046841, Unigene17110\_Sample\_011046841, Unigene48877\_Sample\_011046841, Unigene5169\_Sample\_011046841, Unigene35060\_Sample\_011046841, Unigene16689\_Sample\_011046841, Unigene8255\_Sample\_011046841, Unigene59441\_Sample\_011046841, Unigene43561\_Sample\_011046841, Unigene53915\_Sample\_011046841, Unigene36298\_Sample\_011046841, Unigene46635\_Sample\_011046841, Unigene51555\_Sample\_011046841, Unigene60968\_Sample\_011046841, Unigene52508\_Sample\_011046841, Unigene53333\_Sample\_011046841, Unigene16600\_Sample\_011046841, Unigene48882\_Sample\_011046841, Unigene59201\_Sample\_011046841, Unigene53930\_Sample\_011046841, Unigene57086\_Sample\_011046841, Unigene59679\_Sample\_011046841, Unigene23924\_Sample\_011046841, Unigene36252\_Sample\_011046841, Unigene54997\_Sample\_011046841, Unigene60387\_Sample\_011046841, Unigene50396\_Sample\_011046841, Unigene58138\_Sample\_011046841, Unigene60639\_Sample\_011046841, Unigene44429\_Sample\_011046841, Unigene44148\_Sample\_011046841, Unigene60705\_Sample\_011046841, Unigene37995\_Sample\_011046841, Unigene13893\_Sample\_011046841, Unigene60309\_Sample\_011046841, Unigene60325\_Sample\_011046841, Unigene6724\_Sample\_011046841, Unigene59234\_Sample\_011046841, Unigene60741\_Sample\_011046841, Unigene56005\_Sample\_011046841, Unigene48567\_Sample\_011046841, Unigene22457\_Sample\_011046841, Unigene2492\_Sample\_011046841, Unigene29165\_Sample\_011046841, Unigene58082\_Sample\_011046841, Unigene48701\_Sample\_011046841, Unigene13258\_Sample\_011046841, Unigene41401\_Sample\_011046841, Unigene44328\_Sample\_011046841, Unigene50653\_Sample\_011046841, Unigene60381\_Sample\_011046841, Unigene44861\_Sample\_011046841, Unigene54949\_Sample\_011046841, Unigene50329\_Sample\_011046841, Unigene47549\_Sample\_011046841, Unigene46510\_Sample\_011046841, Unigene43972\_Sample\_011046841, Unigene4314\_Sample\_011046841, Unigene39094\_Sample\_011046841, Unigene49980\_Sample\_011046841, Unigene9855\_Sample\_011046841, Unigene56615\_Sample\_011046841, Unigene57106\_Sample\_011046841, Unigene52427\_Sample\_011046841, Unigene28625\_Sample\_011046841, Unigene56278\_Sample\_011046841, Unigene46431\_Sample\_011046841, Unigene58053\_Sample\_011046841, Unigene40147\_Sample\_011046841, Unigene38078\_Sample\_011046841, Unigene19596\_Sample\_011046841, Unigene45131\_Sample\_011046841, Unigene55393\_Sample\_011046841, Unigene60303\_Sample\_011046841, Unigene38974\_Sample\_011046841, Unigene60921\_Sample\_011046841, Unigene5783\_Sample\_011046841, Unigene43380\_Sample\_011046841, Unigene38523\_Sample\_011046841, Unigene5733\_Sample\_011046841, Unigene59859\_Sample\_011046841, Unigene55420\_Sample\_011046841, Unigene52271\_Sample\_011046841, Unigene54853\_Sample\_011046841, Unigene53817\_Sample\_011046841, Unigene57132\_Sample\_011046841, Unigene47368\_Sample\_011046841, Unigene60043\_Sample\_011046841, Unigene48091\_Sample\_011046841, Unigene39096\_Sample\_011046841, Unigene56058\_Sample\_011046841, Unigene49698\_Sample\_011046841, Unigene29107\_Sample\_011046841, Unigene58132\_Sample\_011046841, Unigene27899\_Sample\_011046841, Unigene21132\_Sample\_011046841, Unigene11187\_Sample\_011046841, Unigene55618\_Sample\_011046841, Unigene60712\_Sample\_011046841, Unigene43500\_Sample\_011046841, Unigene54326\_Sample\_011046841, Unigene60693\_Sample\_011046841, Unigene54235\_Sample\_011046841, Unigene51120\_Sample\_011046841, Unigene50835\_Sample\_011046841, Unigene60149\_Sample\_011046841, Unigene44220\_Sample\_011046841, Unigene46774\_Sample\_011046841, Unigene6629\_Sample\_011046841, Unigene31746\_Sample\_011046841, Unigene29847\_Sample\_011046841, Unigene15802\_Sample\_011046841, Unigene50412\_Sample\_011046841, Unigene57610\_Sample\_011046841, Unigene55947\_Sample\_011046841, Unigene5154\_Sample\_011046841, Unigene39302\_Sample\_011046841, Unigene4547\_Sample\_011046841, Unigene44734\_Sample\_011046841, Unigene20619\_Sample\_011046841, Unigene2415\_Sample\_011046841, Unigene59919\_Sample\_011046841, Unigene30570\_Sample\_011046841, Unigene42703\_Sample\_011046841, Unigene17479\_Sample\_011046841, Unigene10215\_Sample\_011046841, Unigene31849\_Sample\_011046841, Unigene52303\_Sample\_011046841, Unigene41694\_Sample\_011046841, Unigene30681\_Sample\_011046841, Unigene44106\_Sample\_011046841, Unigene59819\_Sample\_011046841, Unigene58515\_Sample\_011046841, Unigene13744\_Sample\_011046841, Unigene54663\_Sample\_011046841, Unigene26664\_Sample\_011046841, Unigene55308\_Sample\_011046841, Unigene57215\_Sample\_011046841, Unigene36816\_Sample\_011046841, Unigene59346\_Sample\_011046841, Unigene55568\_Sample\_011046841, Unigene20324\_Sample\_011046841, Unigene59637\_Sample\_011046841, Unigene57711\_Sample\_011046841, Unigene27211\_Sample\_011046841, Unigene7882\_Sample\_011046841, Unigene58326\_Sample\_011046841, Unigene58953\_Sample\_011046841, Unigene57247\_Sample\_011046841, Unigene19813\_Sample\_011046841, Unigene32699\_Sample\_011046841, Unigene39854\_Sample\_011046841, Unigene27679\_Sample\_011046841, Unigene39772\_Sample\_011046841, Unigene37890\_Sample\_011046841, Unigene56150\_Sample\_011046841, Unigene55905\_Sample\_011046841, Unigene21486\_Sample\_011046841, Unigene34417\_Sample\_011046841, Unigene60681\_Sample\_011046841, Unigene42996\_Sample\_011046841, Unigene59718\_Sample\_011046841, Unigene8180\_Sample\_011046841, Unigene37668\_Sample\_011046841, Unigene25605\_Sample\_011046841, Unigene46082\_Sample\_011046841, Unigene51702\_Sample\_011046841, Unigene40497\_Sample\_011046841, Unigene30072\_Sample\_011046841, Unigene19215\_Sample\_011046841, Unigene11662\_Sample\_011046841, Unigene33778\_Sample\_011046841, Unigene46978\_Sample\_011046841, Unigene13718\_Sample\_011046841, Unigene54053\_Sample\_011046841, Unigene55660\_Sample\_011046841, Unigene54900\_Sample\_011046841, Unigene57687\_Sample\_011046841, Unigene52501\_Sample\_011046841, Unigene57173\_Sample\_011046841, Unigene40620\_Sample\_011046841, Unigene60378\_Sample\_011046841, Unigene35362\_Sample\_011046841, Unigene32095\_Sample\_011046841, Unigene48756\_Sample\_011046841, Unigene57759\_Sample\_011046841, Unigene57614\_Sample\_011046841, Unigene1176\_Sample\_011046841, Unigene57972\_Sample\_011046841, Unigene27178\_Sample\_011046841, Unigene16237\_Sample\_011046841, Unigene28858\_Sample\_011046841, Unigene27900\_Sample\_011046841, Unigene32596\_Sample\_011046841, Unigene59678\_Sample\_011046841, Unigene35828\_Sample\_011046841, Unigene46498\_Sample\_011046841, Unigene60711\_Sample\_011046841, Unigene43139\_Sample\_011046841, Unigene57962\_Sample\_011046841, Unigene20433\_Sample\_011046841, Unigene19919\_Sample\_011046841, Unigene1934\_Sample\_011046841, Unigene42270\_Sample\_011046841, Unigene55154\_Sample\_011046841, Unigene35476\_Sample\_011046841, Unigene20911\_Sample\_011046841, Unigene50002\_Sample\_011046841, Unigene25153\_Sample\_011046841, Unigene57259\_Sample\_011046841, Unigene20298\_Sample\_011046841, Unigene55533\_Sample\_011046841, Unigene18461\_Sample\_011046841, Unigene44315\_Sample\_011046841, Unigene24814\_Sample\_011046841, Unigene30971\_Sample\_011046841, Unigene57052\_Sample\_011046841, Unigene53188\_Sample\_011046841, Unigene8391\_Sample\_011046841, Unigene33302\_Sample\_011046841, Unigene50523\_Sample\_011046841, Unigene39429\_Sample\_011046841, Unigene34653\_Sample\_011046841, Unigene34209\_Sample\_011046841, Unigene46760\_Sample\_011046841, Unigene60949\_Sample\_011046841, Unigene33498\_Sample\_011046841, Unigene45334\_Sample\_011046841, Unigene11465\_Sample\_011046841, Unigene57201\_Sample\_011046841, Unigene12782\_Sample\_011046841, Unigene52566\_Sample\_011046841, Unigene46220\_Sample\_011046841, Unigene54884\_Sample\_011046841, Unigene34055\_Sample\_011046841, Unigene30301\_Sample\_011046841, Unigene13791\_Sample\_011046841, Unigene51950\_Sample\_011046841, Unigene37162\_Sample\_011046841, Unigene34203\_Sample\_011046841, Unigene19868\_Sample\_011046841, Unigene22086\_Sample\_011046841, Unigene60963\_Sample\_011046841, Unigene14996\_Sample\_011046841, Unigene41288\_Sample\_011046841, Unigene16692\_Sample\_011046841, Unigene54373\_Sample\_011046841, Unigene51070\_Sample\_011046841, Unigene59965\_Sample\_011046841, Unigene59475\_Sample\_011046841, Unigene1995\_Sample\_011046841, Unigene51029\_Sample\_011046841, Unigene41373\_Sample\_011046841, Unigene46500\_Sample\_011046841, Unigene56378\_Sample\_011046841, Unigene54670\_Sample\_011046841, Unigene45203\_Sample\_011046841, Unigene59688\_Sample\_011046841, Unigene6994\_Sample\_011046841, Unigene54174\_Sample\_011046841, Unigene35438\_Sample\_011046841, Unigene13945\_Sample\_011046841, Unigene21919\_Sample\_011046841, Unigene54859\_Sample\_011046841, Unigene59926\_Sample\_011046841, Unigene60157\_Sample\_011046841, Unigene22776\_Sample\_011046841, Unigene54448\_Sample\_011046841, Unigene7277\_Sample\_011046841, Unigene42999\_Sample\_011046841, Unigene58611\_Sample\_011046841, Unigene9872\_Sample\_011046841, Unigene5325\_Sample\_011046841, Unigene140\_Sample\_011046841, Unigene60093\_Sample\_011046841, Unigene13180\_Sample\_011046841, Unigene45374\_Sample\_011046841, Unigene60966\_Sample\_011046841, Unigene13951\_Sample\_011046841, Unigene47273\_Sample\_011046841, Unigene8278\_Sample\_011046841, Unigene26878\_Sample\_011046841, Unigene41847\_Sample\_011046841, Unigene29206\_Sample\_011046841, Unigene829\_Sample\_011046841, Unigene12064\_Sample\_011046841, Unigene55311\_Sample\_011046841, Unigene26171\_Sample\_011046841, Unigene11730\_Sample\_011046841, Unigene22728\_Sample\_011046841, Unigene16136\_Sample\_011046841, Unigene10567\_Sample\_011046841, Unigene19671\_Sample\_011046841, Unigene33226\_Sample\_011046841, Unigene4226\_Sample\_011046841, Unigene11658\_Sample\_011046841, Unigene60490\_Sample\_011046841, Unigene60057\_Sample\_011046841, Unigene12822\_Sample\_011046841, Unigene47882\_Sample\_011046841, Unigene38398\_Sample\_011046841, Unigene7639\_Sample\_011046841, Unigene59664\_Sample\_011046841, Unigene10464\_Sample\_011046841, Unigene16810\_Sample\_011046841, Unigene54048\_Sample\_011046841, Unigene8384\_Sample\_011046841, Unigene28245\_Sample\_011046841, Unigene36841\_Sample\_011046841, Unigene55527\_Sample\_011046841, Unigene35691\_Sample\_011046841, Unigene44567\_Sample\_011046841, Unigene11050\_Sample\_011046841, Unigene3666\_Sample\_011046841, Unigene40723\_Sample\_011046841, Unigene12384\_Sample\_011046841, Unigene7905\_Sample\_011046841, Unigene30520\_Sample\_011046841, Unigene11329\_Sample\_011046841, Unigene14570\_Sample\_011046841, Unigene59692\_Sample\_011046841, Unigene50414\_Sample\_011046841, Unigene58145\_Sample\_011046841, Unigene16199\_Sample\_011046841, Unigene11350\_Sample\_011046841, Unigene51942\_Sample\_011046841, Unigene36791\_Sample\_011046841, Unigene41248\_Sample\_011046841, Unigene50825\_Sample\_011046841, Unigene58706\_Sample\_011046841, Unigene60208\_Sample\_011046841, Unigene40648\_Sample\_011046841, Unigene53415\_Sample\_011046841, Unigene10598\_Sample\_011046841, Unigene12811\_Sample\_011046841, Unigene35946\_Sample\_011046841, Unigene21962\_Sample\_011046841, Unigene15108\_Sample\_011046841, Unigene58494\_Sample\_011046841, Unigene57896\_Sample\_011046841, Unigene7095\_Sample\_011046841, Unigene58584\_Sample\_011046841, Unigene30743\_Sample\_011046841, Unigene58934\_Sample\_011046841, Unigene56518\_Sample\_011046841, Unigene10011\_Sample\_011046841, Unigene57318\_Sample\_011046841, Unigene60455\_Sample\_011046841, Unigene31545\_Sample\_011046841, Unigene55647\_Sample\_011046841, Unigene53838\_Sample\_011046841, Unigene8281\_Sample\_011046841, Unigene53241\_Sample\_011046841, Unigene4949\_Sample\_011046841, Unigene56584\_Sample\_011046841, Unigene59059\_Sample\_011046841, Unigene56360\_Sample\_011046841, Unigene38302\_Sample\_011046841, Unigene2488\_Sample\_011046841, Unigene59518\_Sample\_011046841, Unigene52701\_Sample\_011046841, Unigene28077\_Sample\_011046841, Unigene25755\_Sample\_011046841, Unigene45952\_Sample\_011046841, Unigene57009\_Sample\_011046841, Unigene46490\_Sample\_011046841, Unigene58255\_Sample\_011046841, Unigene54854\_Sample\_011046841, Unigene56502\_Sample\_011046841, Unigene43690\_Sample\_011046841, Unigene10438\_Sample\_011046841, Unigene13433\_Sample\_011046841, Unigene21757\_Sample\_011046841, Unigene55715\_Sample\_011046841, Unigene23751\_Sample\_011046841, Unigene59194\_Sample\_011046841, Unigene13817\_Sample\_011046841, Unigene38158\_Sample\_011046841, Unigene40767\_Sample\_011046841, Unigene44237\_Sample\_011046841, Unigene50303\_Sample\_011046841, Unigene45740\_Sample\_011046841, Unigene59357\_Sample\_011046841, Unigene58229\_Sample\_011046841, Unigene6752\_Sample\_011046841, Unigene15792\_Sample\_011046841, Unigene60950\_Sample\_011046841, Unigene49377\_Sample\_011046841, Unigene46598\_Sample\_011046841, Unigene4504\_Sample\_011046841, Unigene36902\_Sample\_011046841, Unigene58610\_Sample\_011046841, Unigene57183\_Sample\_011046841, Unigene52005\_Sample\_011046841, Unigene55938\_Sample\_011046841, Unigene10548\_Sample\_011046841, Unigene41903\_Sample\_011046841, Unigene8516\_Sample\_011046841, Unigene12280\_Sample\_011046841, Unigene32550\_Sample\_011046841, Unigene41499\_Sample\_011046841, Unigene46800\_Sample\_011046841, Unigene59861\_Sample\_011046841, Unigene53252\_Sample\_011046841, Unigene21780\_Sample\_011046841, Unigene27997\_Sample\_011046841, Unigene24973\_Sample\_011046841, Unigene52788\_Sample\_011046841, Unigene22806\_Sample\_011046841, Unigene59750\_Sample\_011046841, Unigene36363\_Sample\_011046841, Unigene53888\_Sample\_011046841, Unigene13079\_Sample\_011046841, Unigene56753\_Sample\_011046841, Unigene53773\_Sample\_011046841, Unigene4775\_Sample\_011046841, Unigene59413\_Sample\_011046841, Unigene1010\_Sample\_011046841, Unigene10426\_Sample\_011046841, Unigene12245\_Sample\_011046841, Unigene55301\_Sample\_011046841, Unigene50746\_Sample\_011046841, Unigene4966\_Sample\_011046841, Unigene37889\_Sample\_011046841, Unigene55570\_Sample\_011046841, Unigene59872\_Sample\_011046841, Unigene38675\_Sample\_011046841, Unigene54305\_Sample\_011046841, Unigene53034\_Sample\_011046841, Unigene466\_Sample\_011046841, Unigene50644\_Sample\_011046841, Unigene55794\_Sample\_011046841, Unigene7288\_Sample\_011046841, Unigene8954\_Sample\_011046841, Unigene19091\_Sample\_011046841, Unigene59092\_Sample\_011046841, Unigene60180\_Sample\_011046841, Unigene50114\_Sample\_011046841, Unigene38437\_Sample\_011046841, Unigene30035\_Sample\_011046841, Unigene57353\_Sample\_011046841, Unigene55528\_Sample\_011046841, Unigene40934\_Sample\_011046841, Unigene38349\_Sample\_011046841, Unigene48998\_Sample\_011046841, Unigene51699\_Sample\_011046841, Unigene11786\_Sample\_011046841, Unigene22482\_Sample\_011046841, Unigene49113\_Sample\_011046841, Unigene24376\_Sample\_011046841, Unigene36908\_Sample\_011046841, Unigene56647\_Sample\_011046841, Unigene60130\_Sample\_011046841, Unigene46026\_Sample\_011046841, Unigene35664\_Sample\_011046841, Unigene11858\_Sample\_011046841, Unigene16264\_Sample\_011046841, Unigene34891\_Sample\_011046841, Unigene38616\_Sample\_011046841, Unigene48873\_Sample\_011046841, Unigene60543\_Sample\_011046841, Unigene16987\_Sample\_011046841, Unigene24060\_Sample\_011046841, Unigene17531\_Sample\_011046841, Unigene15159\_Sample\_011046841, Unigene23218\_Sample\_011046841, Unigene55948\_Sample\_011046841, Unigene18170\_Sample\_011046841, Unigene28796\_Sample\_011046841, Unigene10049\_Sample\_011046841, Unigene19103\_Sample\_011046841, Unigene60478\_Sample\_011046841, Unigene46756\_Sample\_011046841, Unigene60718\_Sample\_011046841, Unigene50548\_Sample\_011046841, Unigene32854\_Sample\_011046841, Unigene19608\_Sample\_011046841, Unigene29365\_Sample\_011046841, Unigene54800\_Sample\_011046841, Unigene44253\_Sample\_011046841, Unigene43411\_Sample\_011046841, Unigene12295\_Sample\_011046841, Unigene35668\_Sample\_011046841, Unigene53424\_Sample\_011046841, Unigene58367\_Sample\_011046841, Unigene51126\_Sample\_011046841, Unigene60272\_Sample\_011046841, Unigene41\_Sample\_011046841, Unigene9034\_Sample\_011046841, Unigene40161\_Sample\_011046841, Unigene12903\_Sample\_011046841, Unigene49667\_Sample\_011046841, Unigene52110\_Sample\_011046841, Unigene29181\_Sample\_011046841, Unigene56746\_Sample\_011046841, Unigene48499\_Sample\_011046841, Unigene35788\_Sample\_011046841, Unigene12849\_Sample\_011046841, Unigene16432\_Sample\_011046841, Unigene57633\_Sample\_011046841, Unigene60040\_Sample\_011046841, Unigene60390\_Sample\_011046841, Unigene25799\_Sample\_011046841, Unigene11695\_Sample\_011046841, Unigene47149\_Sample\_011046841, Unigene53520\_Sample\_011046841, Unigene55037\_Sample\_011046841, Unigene32993\_Sample\_011046841, Unigene37176\_Sample\_011046841, Unigene51664\_Sample\_011046841, Unigene1004\_Sample\_011046841, Unigene58681\_Sample\_011046841, Unigene35814\_Sample\_011046841, Unigene44175\_Sample\_011046841, Unigene9130\_Sample\_011046841, Unigene44178\_Sample\_011046841, Unigene11099\_Sample\_011046841, Unigene18000\_Sample\_011046841, Unigene47429\_Sample\_011046841, Unigene53097\_Sample\_011046841, Unigene53816\_Sample\_011046841, Unigene12487\_Sample\_011046841, Unigene7220\_Sample\_011046841, Unigene54386\_Sample\_011046841, Unigene2570\_Sample\_011046841, Unigene29109\_Sample\_011046841, Unigene13616\_Sample\_011046841, Unigene36370\_Sample\_011046841, Unigene8588\_Sample\_011046841, Unigene3893\_Sample\_011046841, Unigene5954\_Sample\_011046841, Unigene38769\_Sample\_011046841, Unigene7979\_Sample\_011046841, Unigene60258\_Sample\_011046841, Unigene3659\_Sample\_011046841, Unigene40683\_Sample\_011046841, Unigene6317\_Sample\_011046841, Unigene24046\_Sample\_011046841, Unigene51441\_Sample\_011046841, Unigene59273\_Sample\_011046841, Unigene58330\_Sample\_011046841, Unigene55071\_Sample\_011046841, Unigene60773\_Sample\_011046841, Unigene54989\_Sample\_011046841, Unigene56094\_Sample\_011046841, Unigene13763\_Sample\_011046841, Unigene57667\_Sample\_011046841, Unigene12326\_Sample\_011046841, Unigene51137\_Sample\_011046841, Unigene47755\_Sample\_011046841, Unigene49353\_Sample\_011046841, Unigene12578\_Sample\_011046841, Unigene60866\_Sample\_011046841, Unigene9076\_Sample\_011046841, Unigene56969\_Sample\_011046841, Unigene33738\_Sample\_011046841, Unigene36823\_Sample\_011046841, Unigene58031\_Sample\_011046841, Unigene36872\_Sample\_011046841, Unigene28066\_Sample\_011046841, Unigene58444\_Sample\_011046841, Unigene43986\_Sample\_011046841, Unigene53676\_Sample\_011046841, Unigene25064\_Sample\_011046841, Unigene58919\_Sample\_011046841, Unigene51663\_Sample\_011046841, Unigene53749\_Sample\_011046841, Unigene6829\_Sample\_011046841, Unigene41828\_Sample\_011046841, Unigene59532\_Sample\_011046841, Unigene25779\_Sample\_011046841, Unigene38805\_Sample\_011046841, Unigene39240\_Sample\_011046841, Unigene15953\_Sample\_011046841, Unigene8056\_Sample\_011046841, Unigene36679\_Sample\_011046841, Unigene53305\_Sample\_011046841, Unigene45416\_Sample\_011046841, Unigene2968\_Sample\_011046841, Unigene26069\_Sample\_011046841, Unigene14590\_Sample\_011046841, Unigene9211\_Sample\_011046841, Unigene59588\_Sample\_011046841, Unigene59292\_Sample\_011046841, Unigene36020\_Sample\_011046841, Unigene21921\_Sample\_011046841, Unigene58390\_Sample\_011046841, Unigene32313\_Sample\_011046841, Unigene39557\_Sample\_011046841, Unigene44445\_Sample\_011046841, Unigene13051\_Sample\_011046841, Unigene892\_Sample\_011046841, Unigene25083\_Sample\_011046841, Unigene60125\_Sample\_011046841, Unigene50487\_Sample\_011046841, Unigene45708\_Sample\_011046841, Unigene57678\_Sample\_011046841, Unigene49320\_Sample\_011046841, Unigene50315\_Sample\_011046841, Unigene53451\_Sample\_011046841, Unigene59172\_Sample\_011046841, Unigene56311\_Sample\_011046841, Unigene53963\_Sample\_011046841, Unigene46224\_Sample\_011046841, Unigene53025\_Sample\_011046841 |
| transcription regulator activity | Unigene58776\_Sample\_011046841, Unigene6085\_Sample\_011046841, Unigene42565\_Sample\_011046841, Unigene39261\_Sample\_011046841, Unigene28318\_Sample\_011046841, Unigene56835\_Sample\_011046841, Unigene41388\_Sample\_011046841, Unigene55122\_Sample\_011046841, Unigene54460\_Sample\_011046841, Unigene49481\_Sample\_011046841, Unigene12346\_Sample\_011046841, Unigene59068\_Sample\_011046841, Unigene45310\_Sample\_011046841, Unigene54668\_Sample\_011046841, Unigene54581\_Sample\_011046841, Unigene13651\_Sample\_011046841, Unigene50945\_Sample\_011046841, Unigene47025\_Sample\_011046841, Unigene11120\_Sample\_011046841, Unigene20836\_Sample\_011046841, Unigene59633\_Sample\_011046841, Unigene11741\_Sample\_011046841, Unigene39074\_Sample\_011046841, Unigene55254\_Sample\_011046841, Unigene59151\_Sample\_011046841, Unigene58192\_Sample\_011046841, Unigene60780\_Sample\_011046841, Unigene69\_Sample\_011046841, Unigene1989\_Sample\_011046841, Unigene31773\_Sample\_011046841, Unigene53179\_Sample\_011046841, Unigene29658\_Sample\_011046841, Unigene48260\_Sample\_011046841, Unigene8392\_Sample\_011046841, Unigene57913\_Sample\_011046841, Unigene58810\_Sample\_011046841, Unigene30153\_Sample\_011046841, Unigene4113\_Sample\_011046841, Unigene3342\_Sample\_011046841, Unigene55156\_Sample\_011046841, Unigene30385\_Sample\_011046841, Unigene30530\_Sample\_011046841, Unigene7303\_Sample\_011046841, Unigene12748\_Sample\_011046841, Unigene6313\_Sample\_011046841, Unigene58558\_Sample\_011046841, Unigene9726\_Sample\_011046841, Unigene43020\_Sample\_011046841, Unigene58166\_Sample\_011046841, Unigene10640\_Sample\_011046841, Unigene8034\_Sample\_011046841, Unigene2068\_Sample\_011046841, Unigene19409\_Sample\_011046841, Unigene50734\_Sample\_011046841, Unigene246\_Sample\_011046841, Unigene58014\_Sample\_011046841, Unigene31026\_Sample\_011046841, Unigene56254\_Sample\_011046841, Unigene56127\_Sample\_011046841, Unigene26327\_Sample\_011046841, Unigene58190\_Sample\_011046841, Unigene51696\_Sample\_011046841, Unigene3363\_Sample\_011046841, Unigene10963\_Sample\_011046841, Unigene11201\_Sample\_011046841, Unigene12769\_Sample\_011046841, Unigene56466\_Sample\_011046841, Unigene58907\_Sample\_011046841, Unigene39807\_Sample\_011046841, Unigene5747\_Sample\_011046841, Unigene13256\_Sample\_011046841, Unigene58043\_Sample\_011046841, Unigene50110\_Sample\_011046841, Unigene12620\_Sample\_011046841, Unigene58113\_Sample\_011046841, Unigene12251\_Sample\_011046841, Unigene33111\_Sample\_011046841, Unigene54628\_Sample\_011046841, Unigene35914\_Sample\_011046841, Unigene8959\_Sample\_011046841, Unigene17531\_Sample\_011046841, Unigene9001\_Sample\_011046841, Unigene51225\_Sample\_011046841, Unigene59447\_Sample\_011046841, Unigene59556\_Sample\_011046841, Unigene60478\_Sample\_011046841, Unigene8243\_Sample\_011046841, Unigene27108\_Sample\_011046841, Unigene1952\_Sample\_011046841, Unigene51471\_Sample\_011046841, Unigene55302\_Sample\_011046841, Unigene58715\_Sample\_011046841, Unigene57216\_Sample\_011046841, Unigene13436\_Sample\_011046841, Unigene4889\_Sample\_011046841, Unigene49724\_Sample\_011046841, Unigene13847\_Sample\_011046841, Unigene33118\_Sample\_011046841, Unigene54116\_Sample\_011046841, Unigene58753\_Sample\_011046841, Unigene13636\_Sample\_011046841, Unigene51519\_Sample\_011046841, Unigene52688\_Sample\_011046841, Unigene9668\_Sample\_011046841, Unigene54380\_Sample\_011046841, Unigene17367\_Sample\_011046841, Unigene55258\_Sample\_011046841, Unigene56909\_Sample\_011046841, Unigene60371\_Sample\_011046841, Unigene51125\_Sample\_011046841, Unigene48473\_Sample\_011046841, Unigene54139\_Sample\_011046841, Unigene13748\_Sample\_011046841, Unigene10962\_Sample\_011046841, Unigene11351\_Sample\_011046841, Unigene5904\_Sample\_011046841, Unigene47872\_Sample\_011046841, Unigene4523\_Sample\_011046841, Unigene57776\_Sample\_011046841, Unigene24797\_Sample\_011046841, Unigene11187\_Sample\_011046841, Unigene12586\_Sample\_011046841, Unigene53979\_Sample\_011046841, Unigene57544\_Sample\_011046841, Unigene9579\_Sample\_011046841, Unigene52542\_Sample\_011046841, Unigene8526\_Sample\_011046841, Unigene52998\_Sample\_011046841, Unigene8654\_Sample\_011046841, Unigene47680\_Sample\_011046841, Unigene993\_Sample\_011046841, Unigene56391\_Sample\_011046841, Unigene9301\_Sample\_011046841, Unigene41466\_Sample\_011046841, Unigene59702\_Sample\_011046841, Unigene12390\_Sample\_011046841, Unigene9408\_Sample\_011046841, Unigene57096\_Sample\_011046841, Unigene48317\_Sample\_011046841, Unigene58747\_Sample\_011046841, Unigene53798\_Sample\_011046841, Unigene12700\_Sample\_011046841, Unigene57610\_Sample\_011046841, Unigene41323\_Sample\_011046841, Unigene10289\_Sample\_011046841, Unigene59198\_Sample\_011046841, Unigene57070\_Sample\_011046841, Unigene57438\_Sample\_011046841, Unigene35691\_Sample\_011046841, Unigene55707\_Sample\_011046841, Unigene44734\_Sample\_011046841, Unigene60376\_Sample\_011046841, Unigene50243\_Sample\_011046841, Unigene8000\_Sample\_011046841, Unigene59743\_Sample\_011046841, Unigene4364\_Sample\_011046841, Unigene49099\_Sample\_011046841, Unigene37028\_Sample\_011046841, Unigene37760\_Sample\_011046841, Unigene56629\_Sample\_011046841, Unigene2325\_Sample\_011046841, Unigene42229\_Sample\_011046841, Unigene55737\_Sample\_011046841, Unigene29126\_Sample\_011046841, Unigene11939\_Sample\_011046841, Unigene3946\_Sample\_011046841, Unigene11249\_Sample\_011046841, Unigene45403\_Sample\_011046841, Unigene25307\_Sample\_011046841, Unigene37278\_Sample\_011046841, Unigene60689\_Sample\_011046841, Unigene8925\_Sample\_011046841, Unigene12562\_Sample\_011046841, Unigene58518\_Sample\_011046841, Unigene12940\_Sample\_011046841, Unigene47635\_Sample\_011046841, Unigene23790\_Sample\_011046841, Unigene32028\_Sample\_011046841, Unigene9259\_Sample\_011046841, Unigene58365\_Sample\_011046841, Unigene26964\_Sample\_011046841, Unigene31420\_Sample\_011046841, Unigene9107\_Sample\_011046841, Unigene16650\_Sample\_011046841, Unigene57425\_Sample\_011046841, Unigene59136\_Sample\_011046841, Unigene762\_Sample\_011046841, Unigene41959\_Sample\_011046841, Unigene49192\_Sample\_011046841, Unigene47952\_Sample\_011046841, Unigene55863\_Sample\_011046841, Unigene41467\_Sample\_011046841, Unigene58697\_Sample\_011046841, Unigene2861\_Sample\_011046841, Unigene50223\_Sample\_011046841, Unigene13844\_Sample\_011046841, Unigene8641\_Sample\_011046841, Unigene4218\_Sample\_011046841, Unigene36199\_Sample\_011046841, Unigene50902\_Sample\_011046841, Unigene32930\_Sample\_011046841, Unigene58947\_Sample\_011046841, Unigene55247\_Sample\_011046841, Unigene12446\_Sample\_011046841, Unigene56503\_Sample\_011046841, Unigene38631\_Sample\_011046841, Unigene24860\_Sample\_011046841, Unigene55465\_Sample\_011046841, Unigene58106\_Sample\_011046841, Unigene52591\_Sample\_011046841, Unigene29861\_Sample\_011046841, Unigene57305\_Sample\_011046841, Unigene6469\_Sample\_011046841, Unigene13251\_Sample\_011046841, Unigene58564\_Sample\_011046841, Unigene47837\_Sample\_011046841, Unigene19264\_Sample\_011046841, Unigene59987\_Sample\_011046841, Unigene31706\_Sample\_011046841, Unigene52381\_Sample\_011046841, Unigene42036\_Sample\_011046841, Unigene8990\_Sample\_011046841, Unigene36452\_Sample\_011046841, Unigene49337\_Sample\_011046841, Unigene7850\_Sample\_011046841, Unigene53297\_Sample\_011046841, Unigene59560\_Sample\_011046841, Unigene251\_Sample\_011046841, Unigene56807\_Sample\_011046841 |
| protein kinase activity | Unigene55833\_Sample\_011046841, Unigene57972\_Sample\_011046841, Unigene58864\_Sample\_011046841, Unigene55221\_Sample\_011046841, Unigene16112\_Sample\_011046841, Unigene51130\_Sample\_011046841, Unigene31969\_Sample\_011046841, Unigene2784\_Sample\_011046841, Unigene52866\_Sample\_011046841, Unigene10250\_Sample\_011046841, Unigene9858\_Sample\_011046841, Unigene11120\_Sample\_011046841, Unigene9354\_Sample\_011046841, Unigene4303\_Sample\_011046841, Unigene51777\_Sample\_011046841, Unigene33573\_Sample\_011046841, Unigene60481\_Sample\_011046841, Unigene16803\_Sample\_011046841, Unigene49534\_Sample\_011046841, Unigene18318\_Sample\_011046841, Unigene55162\_Sample\_011046841, Unigene30971\_Sample\_011046841, Unigene48482\_Sample\_011046841, Unigene18749\_Sample\_011046841, Unigene53995\_Sample\_011046841, Unigene12538\_Sample\_011046841, Unigene59421\_Sample\_011046841, Unigene21225\_Sample\_011046841, Unigene10970\_Sample\_011046841, Unigene54240\_Sample\_011046841, Unigene56545\_Sample\_011046841, Unigene51706\_Sample\_011046841, Unigene57634\_Sample\_011046841, Unigene9546\_Sample\_011046841, Unigene57083\_Sample\_011046841, Unigene57589\_Sample\_011046841, Unigene51380\_Sample\_011046841, Unigene54701\_Sample\_011046841, Unigene2942\_Sample\_011046841, Unigene32119\_Sample\_011046841, Unigene56697\_Sample\_011046841, Unigene51845\_Sample\_011046841, Unigene59756\_Sample\_011046841, Unigene45770\_Sample\_011046841, Unigene51582\_Sample\_011046841, Unigene40435\_Sample\_011046841, Unigene13672\_Sample\_011046841, Unigene10683\_Sample\_011046841, Unigene55610\_Sample\_011046841, Unigene13068\_Sample\_011046841, Unigene7879\_Sample\_011046841, Unigene46485\_Sample\_011046841, Unigene31264\_Sample\_011046841, Unigene26327\_Sample\_011046841, Unigene50051\_Sample\_011046841, Unigene48009\_Sample\_011046841, Unigene59022\_Sample\_011046841, Unigene55126\_Sample\_011046841, Unigene36373\_Sample\_011046841, Unigene26636\_Sample\_011046841, Unigene60190\_Sample\_011046841, Unigene40349\_Sample\_011046841, Unigene60657\_Sample\_011046841, Unigene37753\_Sample\_011046841, Unigene57641\_Sample\_011046841, Unigene12309\_Sample\_011046841, Unigene10198\_Sample\_011046841, Unigene32409\_Sample\_011046841, Unigene10875\_Sample\_011046841, Unigene4361\_Sample\_011046841, Unigene23831\_Sample\_011046841, Unigene43397\_Sample\_011046841, Unigene46283\_Sample\_011046841, Unigene24424\_Sample\_011046841, Unigene608\_Sample\_011046841, Unigene41574\_Sample\_011046841, Unigene50170\_Sample\_011046841, Unigene47924\_Sample\_011046841, Unigene58134\_Sample\_011046841, Unigene52918\_Sample\_011046841, Unigene13419\_Sample\_011046841, Unigene39238\_Sample\_011046841, Unigene49837\_Sample\_011046841, Unigene19453\_Sample\_011046841, Unigene30474\_Sample\_011046841, Unigene11457\_Sample\_011046841, Unigene47936\_Sample\_011046841, Unigene27253\_Sample\_011046841, Unigene35914\_Sample\_011046841, Unigene58460\_Sample\_011046841, Unigene60557\_Sample\_011046841, Unigene43504\_Sample\_011046841, Unigene50797\_Sample\_011046841, Unigene58128\_Sample\_011046841, Unigene4662\_Sample\_011046841, Unigene39675\_Sample\_011046841, Unigene36772\_Sample\_011046841, Unigene47292\_Sample\_011046841, Unigene54614\_Sample\_011046841, Unigene59061\_Sample\_011046841, Unigene11130\_Sample\_011046841, Unigene51537\_Sample\_011046841, Unigene16001\_Sample\_011046841, Unigene52610\_Sample\_011046841, Unigene59469\_Sample\_011046841, Unigene58100\_Sample\_011046841, Unigene13224\_Sample\_011046841, Unigene13287\_Sample\_011046841, Unigene5297\_Sample\_011046841, Unigene15490\_Sample\_011046841, Unigene60576\_Sample\_011046841, Unigene11231\_Sample\_011046841, Unigene26229\_Sample\_011046841, Unigene57832\_Sample\_011046841, Unigene56054\_Sample\_011046841, Unigene10726\_Sample\_011046841, Unigene9879\_Sample\_011046841, Unigene26777\_Sample\_011046841, Unigene56258\_Sample\_011046841, Unigene42893\_Sample\_011046841, Unigene41032\_Sample\_011046841, Unigene59448\_Sample\_011046841, Unigene19352\_Sample\_011046841, Unigene13332\_Sample\_011046841, Unigene11925\_Sample\_011046841, Unigene57775\_Sample\_011046841, Unigene23925\_Sample\_011046841, Unigene9348\_Sample\_011046841, Unigene47887\_Sample\_011046841, Unigene47872\_Sample\_011046841, Unigene51573\_Sample\_011046841, Unigene58058\_Sample\_011046841, Unigene37549\_Sample\_011046841, Unigene60248\_Sample\_011046841, Unigene54379\_Sample\_011046841, Unigene56798\_Sample\_011046841, Unigene16539\_Sample\_011046841, Unigene10522\_Sample\_011046841, Unigene9862\_Sample\_011046841, Unigene13368\_Sample\_011046841, Unigene13722\_Sample\_011046841, Unigene23133\_Sample\_011046841, Unigene58386\_Sample\_011046841, Unigene48345\_Sample\_011046841, Unigene26192\_Sample\_011046841, Unigene12390\_Sample\_011046841, Unigene49895\_Sample\_011046841, Unigene51301\_Sample\_011046841, Unigene1107\_Sample\_011046841, Unigene13633\_Sample\_011046841, Unigene59727\_Sample\_011046841, Unigene45307\_Sample\_011046841, Unigene14248\_Sample\_011046841, Unigene42686\_Sample\_011046841, Unigene25326\_Sample\_011046841, Unigene60344\_Sample\_011046841, Unigene11547\_Sample\_011046841, Unigene49487\_Sample\_011046841, Unigene21362\_Sample\_011046841, Unigene30190\_Sample\_011046841, Unigene57904\_Sample\_011046841, Unigene40509\_Sample\_011046841, Unigene17850\_Sample\_011046841, Unigene50713\_Sample\_011046841, Unigene23590\_Sample\_011046841, Unigene16977\_Sample\_011046841, Unigene5220\_Sample\_011046841, Unigene38549\_Sample\_011046841, Unigene47967\_Sample\_011046841, Unigene59844\_Sample\_011046841, Unigene60349\_Sample\_011046841, Unigene49055\_Sample\_011046841, Unigene51916\_Sample\_011046841, Unigene28563\_Sample\_011046841, Unigene7335\_Sample\_011046841, Unigene21150\_Sample\_011046841, Unigene55423\_Sample\_011046841, Unigene13901\_Sample\_011046841, Unigene60507\_Sample\_011046841, Unigene6223\_Sample\_011046841, Unigene55644\_Sample\_011046841, Unigene11073\_Sample\_011046841, Unigene25810\_Sample\_011046841, Unigene59850\_Sample\_011046841, Unigene3810\_Sample\_011046841, Unigene54626\_Sample\_011046841, Unigene55404\_Sample\_011046841, Unigene50223\_Sample\_011046841, Unigene3269\_Sample\_011046841, Unigene41464\_Sample\_011046841, Unigene36927\_Sample\_011046841, Unigene59559\_Sample\_011046841, Unigene22189\_Sample\_011046841, Unigene48342\_Sample\_011046841, Unigene37057\_Sample\_011046841, Unigene13197\_Sample\_011046841, Unigene26587\_Sample\_011046841, Unigene18791\_Sample\_011046841, Unigene54453\_Sample\_011046841, Unigene11419\_Sample\_011046841, Unigene9776\_Sample\_011046841, Unigene59228\_Sample\_011046841, Unigene26757\_Sample\_011046841, Unigene48530\_Sample\_011046841, Unigene59257\_Sample\_011046841, Unigene51307\_Sample\_011046841, Unigene46142\_Sample\_011046841, Unigene9811\_Sample\_011046841, Unigene53100\_Sample\_011046841, Unigene46256\_Sample\_011046841, Unigene58021\_Sample\_011046841, Unigene26679\_Sample\_011046841, Unigene44079\_Sample\_011046841, Unigene7042\_Sample\_011046841, Unigene11040\_Sample\_011046841, Unigene44103\_Sample\_011046841, Unigene59221\_Sample\_011046841, Unigene57130\_Sample\_011046841, Unigene46176\_Sample\_011046841, Unigene60753\_Sample\_011046841, Unigene59987\_Sample\_011046841, Unigene32760\_Sample\_011046841, Unigene60166\_Sample\_011046841, Unigene60721\_Sample\_011046841, Unigene53884\_Sample\_011046841, Unigene13321\_Sample\_011046841, Unigene60042\_Sample\_011046841, Unigene32964\_Sample\_011046841, Unigene54549\_Sample\_011046841, Unigene49264\_Sample\_011046841, Unigene52573\_Sample\_011046841, Unigene48062\_Sample\_011046841, Unigene35715\_Sample\_011046841, Unigene53819\_Sample\_011046841, Unigene59457\_Sample\_011046841, Unigene42633\_Sample\_011046841, Unigene57004\_Sample\_011046841, Unigene49737\_Sample\_011046841, Unigene11867\_Sample\_011046841, Unigene12975\_Sample\_011046841, Unigene35082\_Sample\_011046841, Unigene48267\_Sample\_011046841, Unigene1793\_Sample\_011046841, Unigene52842\_Sample\_011046841, Unigene60746\_Sample\_011046841, Unigene5623\_Sample\_011046841, Unigene11998\_Sample\_011046841, Unigene58523\_Sample\_011046841, Unigene11526\_Sample\_011046841, Unigene60540\_Sample\_011046841, Unigene29318\_Sample\_011046841, Unigene45662\_Sample\_011046841, Unigene50039\_Sample\_011046841, Unigene32914\_Sample\_011046841, Unigene55041\_Sample\_011046841, Unigene8288\_Sample\_011046841, Unigene52094\_Sample\_011046841, Unigene49268\_Sample\_011046841, Unigene16813\_Sample\_011046841, Unigene59840\_Sample\_011046841, Unigene31749\_Sample\_011046841, Unigene18294\_Sample\_011046841, Unigene47025\_Sample\_011046841, Unigene7481\_Sample\_011046841, Unigene39219\_Sample\_011046841, Unigene50057\_Sample\_011046841, Unigene6597\_Sample\_011046841, Unigene54574\_Sample\_011046841, Unigene52097\_Sample\_011046841, Unigene54982\_Sample\_011046841, Unigene47591\_Sample\_011046841, Unigene59705\_Sample\_011046841, Unigene15705\_Sample\_011046841, Unigene11932\_Sample\_011046841, Unigene58675\_Sample\_011046841, Unigene13920\_Sample\_011046841, Unigene58427\_Sample\_011046841, Unigene8997\_Sample\_011046841, Unigene47085\_Sample\_011046841, Unigene58913\_Sample\_011046841, Unigene34330\_Sample\_011046841, Unigene59758\_Sample\_011046841, Unigene7964\_Sample\_011046841, Unigene9329\_Sample\_011046841, Unigene51958\_Sample\_011046841, Unigene60244\_Sample\_011046841, Unigene42912\_Sample\_011046841, Unigene53714\_Sample\_011046841, Unigene3842\_Sample\_011046841, Unigene21655\_Sample\_011046841, Unigene56744\_Sample\_011046841, Unigene30484\_Sample\_011046841, Unigene28789\_Sample\_011046841, Unigene2076\_Sample\_011046841, Unigene27464\_Sample\_011046841, Unigene3750\_Sample\_011046841, Unigene6531\_Sample\_011046841, Unigene44021\_Sample\_011046841, Unigene58285\_Sample\_011046841, Unigene34824\_Sample\_011046841, Unigene28658\_Sample\_011046841, Unigene59440\_Sample\_011046841, Unigene15\_Sample\_011046841, Unigene3363\_Sample\_011046841, Unigene55720\_Sample\_011046841, Unigene55261\_Sample\_011046841, Unigene43936\_Sample\_011046841, Unigene60821\_Sample\_011046841, Unigene12652\_Sample\_011046841, Unigene55461\_Sample\_011046841, Unigene49671\_Sample\_011046841, Unigene60434\_Sample\_011046841, Unigene2493\_Sample\_011046841, Unigene4305\_Sample\_011046841, Unigene20201\_Sample\_011046841, Unigene13516\_Sample\_011046841, Unigene2933\_Sample\_011046841, Unigene59459\_Sample\_011046841, Unigene30684\_Sample\_011046841, Unigene53958\_Sample\_011046841, Unigene48334\_Sample\_011046841, Unigene51756\_Sample\_011046841, Unigene23218\_Sample\_011046841, Unigene47210\_Sample\_011046841, Unigene18170\_Sample\_011046841, Unigene22964\_Sample\_011046841, Unigene8429\_Sample\_011046841, Unigene37707\_Sample\_011046841, Unigene5431\_Sample\_011046841, Unigene56566\_Sample\_011046841, Unigene55302\_Sample\_011046841, Unigene15482\_Sample\_011046841, Unigene35470\_Sample\_011046841, Unigene12972\_Sample\_011046841, Unigene18796\_Sample\_011046841, Unigene40258\_Sample\_011046841, Unigene5972\_Sample\_011046841, Unigene58821\_Sample\_011046841, Unigene52385\_Sample\_011046841, Unigene9219\_Sample\_011046841, Unigene41676\_Sample\_011046841, Unigene41682\_Sample\_011046841, Unigene40209\_Sample\_011046841, Unigene25989\_Sample\_011046841, Unigene60191\_Sample\_011046841, Unigene46813\_Sample\_011046841, Unigene36178\_Sample\_011046841, Unigene58254\_Sample\_011046841, Unigene12359\_Sample\_011046841, Unigene43515\_Sample\_011046841, Unigene29314\_Sample\_011046841, Unigene9017\_Sample\_011046841, Unigene59891\_Sample\_011046841, Unigene13966\_Sample\_011046841, Unigene4308\_Sample\_011046841, Unigene42281\_Sample\_011046841, Unigene28288\_Sample\_011046841, Unigene13884\_Sample\_011046841, Unigene58340\_Sample\_011046841, Unigene19556\_Sample\_011046841, Unigene42820\_Sample\_011046841, Unigene35217\_Sample\_011046841, Unigene60220\_Sample\_011046841, Unigene59362\_Sample\_011046841, Unigene20267\_Sample\_011046841, Unigene51011\_Sample\_011046841, Unigene40215\_Sample\_011046841, Unigene56391\_Sample\_011046841, Unigene47078\_Sample\_011046841, Unigene6590\_Sample\_011046841, Unigene34207\_Sample\_011046841, Unigene47910\_Sample\_011046841, Unigene12824\_Sample\_011046841, Unigene47387\_Sample\_011046841, Unigene60733\_Sample\_011046841, Unigene57887\_Sample\_011046841, Unigene16051\_Sample\_011046841, Unigene44734\_Sample\_011046841, Unigene24332\_Sample\_011046841, Unigene51882\_Sample\_011046841, Unigene56062\_Sample\_011046841, Unigene57665\_Sample\_011046841, Unigene30349\_Sample\_011046841, Unigene8000\_Sample\_011046841, Unigene48528\_Sample\_011046841, Unigene32534\_Sample\_011046841, Unigene55101\_Sample\_011046841, Unigene4432\_Sample\_011046841, Unigene14776\_Sample\_011046841, Unigene46198\_Sample\_011046841, Unigene11314\_Sample\_011046841, Unigene29813\_Sample\_011046841, Unigene41694\_Sample\_011046841, Unigene34757\_Sample\_011046841, Unigene3946\_Sample\_011046841, Unigene45885\_Sample\_011046841, Unigene49525\_Sample\_011046841, Unigene59711\_Sample\_011046841, Unigene13367\_Sample\_011046841, Unigene47811\_Sample\_011046841, Unigene19594\_Sample\_011046841, Unigene9774\_Sample\_011046841, Unigene58882\_Sample\_011046841, Unigene11758\_Sample\_011046841, Unigene11504\_Sample\_011046841, Unigene58321\_Sample\_011046841, Unigene12837\_Sample\_011046841, Unigene57194\_Sample\_011046841, Unigene11706\_Sample\_011046841, Unigene12324\_Sample\_011046841, Unigene30456\_Sample\_011046841, Unigene3797\_Sample\_011046841, Unigene32509\_Sample\_011046841, Unigene58669\_Sample\_011046841, Unigene43935\_Sample\_011046841, Unigene50322\_Sample\_011046841, Unigene18856\_Sample\_011046841, Unigene56096\_Sample\_011046841, Unigene40491\_Sample\_011046841, Unigene55247\_Sample\_011046841, Unigene51736\_Sample\_011046841, Unigene47560\_Sample\_011046841, Unigene6435\_Sample\_011046841, Unigene59596\_Sample\_011046841, Unigene47686\_Sample\_011046841, Unigene54599\_Sample\_011046841, Unigene43792\_Sample\_011046841, Unigene44683\_Sample\_011046841, Unigene46783\_Sample\_011046841, Unigene44354\_Sample\_011046841, Unigene21539\_Sample\_011046841, Unigene9210\_Sample\_011046841, Unigene57305\_Sample\_011046841, Unigene56828\_Sample\_011046841, Unigene6197\_Sample\_011046841, Unigene50758\_Sample\_011046841, Unigene53723\_Sample\_011046841, Unigene26728\_Sample\_011046841, Unigene47555\_Sample\_011046841, Unigene17671\_Sample\_011046841, Unigene55564\_Sample\_011046841, Unigene51429\_Sample\_011046841, Unigene58511\_Sample\_011046841, Unigene29587\_Sample\_011046841, Unigene16046\_Sample\_011046841, Unigene23757\_Sample\_011046841, Unigene38527\_Sample\_011046841, Unigene59868\_Sample\_011046841, Unigene48647\_Sample\_011046841, Unigene11676\_Sample\_011046841, Unigene39020\_Sample\_011046841, Unigene60217\_Sample\_011046841, Unigene9082\_Sample\_011046841, Unigene60859\_Sample\_011046841, Unigene2110\_Sample\_011046841, Unigene42561\_Sample\_011046841, Unigene50293\_Sample\_011046841 |
| hydrolase activity, acting on ester bonds | Unigene7801\_Sample\_011046841, Unigene53548\_Sample\_011046841, Unigene32500\_Sample\_011046841, Unigene5749\_Sample\_011046841, Unigene28858\_Sample\_011046841, Unigene48720\_Sample\_011046841, Unigene6065\_Sample\_011046841, Unigene57719\_Sample\_011046841, Unigene46498\_Sample\_011046841, Unigene44282\_Sample\_011046841, Unigene15270\_Sample\_011046841, Unigene57962\_Sample\_011046841, Unigene45858\_Sample\_011046841, Unigene20433\_Sample\_011046841, Unigene50002\_Sample\_011046841, Unigene25153\_Sample\_011046841, Unigene12830\_Sample\_011046841, Unigene47947\_Sample\_011046841, Unigene12157\_Sample\_011046841, Unigene20298\_Sample\_011046841, Unigene16118\_Sample\_011046841, Unigene34114\_Sample\_011046841, Unigene46561\_Sample\_011046841, Unigene32707\_Sample\_011046841, Unigene55286\_Sample\_011046841, Unigene34550\_Sample\_011046841, Unigene53188\_Sample\_011046841, Unigene3235\_Sample\_011046841, Unigene8391\_Sample\_011046841, Unigene60032\_Sample\_011046841, Unigene50523\_Sample\_011046841, Unigene52821\_Sample\_011046841, Unigene34653\_Sample\_011046841, Unigene54701\_Sample\_011046841, Unigene11465\_Sample\_011046841, Unigene21731\_Sample\_011046841, Unigene3975\_Sample\_011046841, Unigene43846\_Sample\_011046841, Unigene11319\_Sample\_011046841, Unigene54884\_Sample\_011046841, Unigene59925\_Sample\_011046841, Unigene12507\_Sample\_011046841, Unigene55844\_Sample\_011046841, Unigene59452\_Sample\_011046841, Unigene22636\_Sample\_011046841, Unigene55151\_Sample\_011046841, Unigene8035\_Sample\_011046841, Unigene20899\_Sample\_011046841, Unigene11982\_Sample\_011046841, Unigene51076\_Sample\_011046841, Unigene54373\_Sample\_011046841, Unigene59475\_Sample\_011046841, Unigene60879\_Sample\_011046841, Unigene55052\_Sample\_011046841, Unigene41373\_Sample\_011046841, Unigene49646\_Sample\_011046841, Unigene25226\_Sample\_011046841, Unigene56378\_Sample\_011046841, Unigene8939\_Sample\_011046841, Unigene49837\_Sample\_011046841, Unigene31101\_Sample\_011046841, Unigene43629\_Sample\_011046841, Unigene11799\_Sample\_011046841, Unigene19937\_Sample\_011046841, Unigene8647\_Sample\_011046841, Unigene39875\_Sample\_011046841, Unigene13945\_Sample\_011046841, Unigene50712\_Sample\_011046841, Unigene54859\_Sample\_011046841, Unigene54401\_Sample\_011046841, Unigene58611\_Sample\_011046841, Unigene20745\_Sample\_011046841, Unigene60093\_Sample\_011046841, Unigene34582\_Sample\_011046841, Unigene23742\_Sample\_011046841, Unigene33253\_Sample\_011046841, Unigene45374\_Sample\_011046841, Unigene58080\_Sample\_011046841, Unigene8128\_Sample\_011046841, Unigene50009\_Sample\_011046841, Unigene33441\_Sample\_011046841, Unigene47083\_Sample\_011046841, Unigene11730\_Sample\_011046841, Unigene54098\_Sample\_011046841, Unigene19671\_Sample\_011046841, Unigene57265\_Sample\_011046841, Unigene57856\_Sample\_011046841, Unigene43448\_Sample\_011046841, Unigene50245\_Sample\_011046841, Unigene4226\_Sample\_011046841, Unigene58531\_Sample\_011046841, Unigene40974\_Sample\_011046841, Unigene13214\_Sample\_011046841, Unigene55132\_Sample\_011046841, Unigene39868\_Sample\_011046841, Unigene24493\_Sample\_011046841, Unigene50497\_Sample\_011046841, Unigene59664\_Sample\_011046841, Unigene7639\_Sample\_011046841, Unigene44838\_Sample\_011046841, Unigene47903\_Sample\_011046841, Unigene18541\_Sample\_011046841, Unigene19789\_Sample\_011046841, Unigene29740\_Sample\_011046841, Unigene40280\_Sample\_011046841, Unigene32259\_Sample\_011046841, Unigene13697\_Sample\_011046841, Unigene48524\_Sample\_011046841, Unigene11050\_Sample\_011046841, Unigene47099\_Sample\_011046841, Unigene40723\_Sample\_011046841, Unigene14181\_Sample\_011046841, Unigene47010\_Sample\_011046841, Unigene35368\_Sample\_011046841, Unigene54290\_Sample\_011046841, Unigene35485\_Sample\_011046841, Unigene19399\_Sample\_011046841, Unigene11769\_Sample\_011046841, Unigene9711\_Sample\_011046841, Unigene59692\_Sample\_011046841, Unigene48170\_Sample\_011046841, Unigene5263\_Sample\_011046841, Unigene47428\_Sample\_011046841, Unigene11350\_Sample\_011046841, Unigene12379\_Sample\_011046841, Unigene47540\_Sample\_011046841, Unigene58620\_Sample\_011046841, Unigene59567\_Sample\_011046841, Unigene17873\_Sample\_011046841, Unigene10615\_Sample\_011046841, Unigene12614\_Sample\_011046841, Unigene12869\_Sample\_011046841, Unigene59595\_Sample\_011046841, Unigene54521\_Sample\_011046841, Unigene40648\_Sample\_011046841, Unigene16650\_Sample\_011046841, Unigene53415\_Sample\_011046841, Unigene10598\_Sample\_011046841, Unigene5112\_Sample\_011046841, Unigene58697\_Sample\_011046841, Unigene51891\_Sample\_011046841, Unigene21962\_Sample\_011046841, Unigene36927\_Sample\_011046841, Unigene45986\_Sample\_011046841, Unigene23312\_Sample\_011046841, Unigene51426\_Sample\_011046841, Unigene43072\_Sample\_011046841, Unigene39991\_Sample\_011046841, Unigene54627\_Sample\_011046841, Unigene39842\_Sample\_011046841, Unigene7340\_Sample\_011046841, Unigene7532\_Sample\_011046841, Unigene30743\_Sample\_011046841, Unigene57375\_Sample\_011046841, Unigene10011\_Sample\_011046841, Unigene58480\_Sample\_011046841, Unigene6340\_Sample\_011046841, Unigene1228\_Sample\_011046841, Unigene31545\_Sample\_011046841, Unigene55647\_Sample\_011046841, Unigene53838\_Sample\_011046841, Unigene60450\_Sample\_011046841, Unigene59067\_Sample\_011046841, Unigene59059\_Sample\_011046841, Unigene26679\_Sample\_011046841, Unigene31586\_Sample\_011046841, Unigene27641\_Sample\_011046841, Unigene57323\_Sample\_011046841, Unigene25755\_Sample\_011046841, Unigene17723\_Sample\_011046841, Unigene46490\_Sample\_011046841, Unigene32666\_Sample\_011046841, Unigene54854\_Sample\_011046841, Unigene56502\_Sample\_011046841, Unigene25759\_Sample\_011046841, Unigene52573\_Sample\_011046841, Unigene45801\_Sample\_011046841, Unigene50596\_Sample\_011046841, Unigene31438\_Sample\_011046841, Unigene23751\_Sample\_011046841, Unigene9271\_Sample\_011046841, Unigene56198\_Sample\_011046841, Unigene47023\_Sample\_011046841, Unigene54524\_Sample\_011046841, Unigene45740\_Sample\_011046841, Unigene59945\_Sample\_011046841, Unigene38234\_Sample\_011046841, Unigene15792\_Sample\_011046841, Unigene51369\_Sample\_011046841, Unigene36902\_Sample\_011046841, Unigene50609\_Sample\_011046841, Unigene42255\_Sample\_011046841, Unigene51524\_Sample\_011046841, Unigene19418\_Sample\_011046841, Unigene20854\_Sample\_011046841, Unigene10744\_Sample\_011046841, Unigene58224\_Sample\_011046841, Unigene12280\_Sample\_011046841, Unigene20487\_Sample\_011046841, Unigene56281\_Sample\_011046841, Unigene48637\_Sample\_011046841, Unigene32629\_Sample\_011046841, Unigene17110\_Sample\_011046841, Unigene12447\_Sample\_011046841, Unigene8255\_Sample\_011046841, Unigene11435\_Sample\_011046841, Unigene22806\_Sample\_011046841, Unigene60906\_Sample\_011046841, Unigene53888\_Sample\_011046841, Unigene56753\_Sample\_011046841, Unigene59880\_Sample\_011046841, Unigene1010\_Sample\_011046841, Unigene30162\_Sample\_011046841, Unigene50746\_Sample\_011046841, Unigene47795\_Sample\_011046841, Unigene23924\_Sample\_011046841, Unigene6203\_Sample\_011046841, Unigene21063\_Sample\_011046841, Unigene38675\_Sample\_011046841, Unigene10317\_Sample\_011046841, Unigene55794\_Sample\_011046841, Unigene32973\_Sample\_011046841, Unigene43875\_Sample\_011046841, Unigene45754\_Sample\_011046841, Unigene22457\_Sample\_011046841, Unigene40934\_Sample\_011046841, Unigene18963\_Sample\_011046841, Unigene55520\_Sample\_011046841, Unigene58082\_Sample\_011046841, Unigene11786\_Sample\_011046841, Unigene24376\_Sample\_011046841, Unigene11522\_Sample\_011046841, Unigene54624\_Sample\_011046841, Unigene6153\_Sample\_011046841, Unigene43972\_Sample\_011046841, Unigene48873\_Sample\_011046841, Unigene46743\_Sample\_011046841, Unigene28106\_Sample\_011046841, Unigene6497\_Sample\_011046841, Unigene59619\_Sample\_011046841, Unigene31483\_Sample\_011046841, Unigene17523\_Sample\_011046841, Unigene57480\_Sample\_011046841, Unigene33032\_Sample\_011046841, Unigene58720\_Sample\_011046841, Unigene23218\_Sample\_011046841, Unigene46431\_Sample\_011046841, Unigene18170\_Sample\_011046841, Unigene24566\_Sample\_011046841, Unigene28796\_Sample\_011046841, Unigene47778\_Sample\_011046841, Unigene29365\_Sample\_011046841, Unigene33507\_Sample\_011046841, Unigene58653\_Sample\_011046841, Unigene43380\_Sample\_011046841, Unigene52271\_Sample\_011046841, Unigene41996\_Sample\_011046841, Unigene58900\_Sample\_011046841, Unigene53817\_Sample\_011046841, Unigene48473\_Sample\_011046841, Unigene52110\_Sample\_011046841, Unigene10201\_Sample\_011046841, Unigene10327\_Sample\_011046841, Unigene16432\_Sample\_011046841, Unigene59747\_Sample\_011046841, Unigene19113\_Sample\_011046841, Unigene53531\_Sample\_011046841, Unigene49698\_Sample\_011046841, Unigene48409\_Sample\_011046841, Unigene59662\_Sample\_011046841, Unigene22255\_Sample\_011046841, Unigene53956\_Sample\_011046841, Unigene60712\_Sample\_011046841, Unigene60149\_Sample\_011046841, Unigene27907\_Sample\_011046841, Unigene52423\_Sample\_011046841, Unigene8859\_Sample\_011046841, Unigene55335\_Sample\_011046841, Unigene15802\_Sample\_011046841, Unigene59387\_Sample\_011046841, Unigene46973\_Sample\_011046841, Unigene47429\_Sample\_011046841, Unigene55936\_Sample\_011046841, Unigene55947\_Sample\_011046841, Unigene5740\_Sample\_011046841, Unigene19526\_Sample\_011046841, Unigene29109\_Sample\_011046841, Unigene50296\_Sample\_011046841, Unigene55719\_Sample\_011046841, Unigene49615\_Sample\_011046841, Unigene5954\_Sample\_011046841, Unigene41694\_Sample\_011046841, Unigene19570\_Sample\_011046841, Unigene40683\_Sample\_011046841, Unigene24046\_Sample\_011046841, Unigene58515\_Sample\_011046841, Unigene53168\_Sample\_011046841, Unigene54663\_Sample\_011046841, Unigene53609\_Sample\_011046841, Unigene19594\_Sample\_011046841, Unigene49840\_Sample\_011046841, Unigene6900\_Sample\_011046841, Unigene59189\_Sample\_011046841, Unigene13912\_Sample\_011046841, Unigene57667\_Sample\_011046841, Unigene51137\_Sample\_011046841, Unigene18715\_Sample\_011046841, Unigene59346\_Sample\_011046841, Unigene33738\_Sample\_011046841, Unigene58180\_Sample\_011046841, Unigene59637\_Sample\_011046841, Unigene36872\_Sample\_011046841, Unigene56389\_Sample\_011046841, Unigene3620\_Sample\_011046841, Unigene25248\_Sample\_011046841, Unigene59532\_Sample\_011046841, Unigene51161\_Sample\_011046841, Unigene37890\_Sample\_011046841, Unigene56150\_Sample\_011046841, Unigene38805\_Sample\_011046841, Unigene60681\_Sample\_011046841, Unigene34417\_Sample\_011046841, Unigene59718\_Sample\_011046841, Unigene37668\_Sample\_011046841, Unigene8056\_Sample\_011046841, Unigene53063\_Sample\_011046841, Unigene53305\_Sample\_011046841, Unigene25605\_Sample\_011046841, Unigene46082\_Sample\_011046841, Unigene43331\_Sample\_011046841, Unigene42071\_Sample\_011046841, Unigene19215\_Sample\_011046841, Unigene33567\_Sample\_011046841, Unigene892\_Sample\_011046841, Unigene54593\_Sample\_011046841, Unigene45708\_Sample\_011046841, Unigene35362\_Sample\_011046841, Unigene59172\_Sample\_011046841 |
| protein serine/threonine kinase activity | Unigene5623\_Sample\_011046841, Unigene11526\_Sample\_011046841, Unigene57972\_Sample\_011046841, Unigene58864\_Sample\_011046841, Unigene45662\_Sample\_011046841, Unigene50039\_Sample\_011046841, Unigene55221\_Sample\_011046841, Unigene32914\_Sample\_011046841, Unigene52094\_Sample\_011046841, Unigene49268\_Sample\_011046841, Unigene16813\_Sample\_011046841, Unigene59840\_Sample\_011046841, Unigene18294\_Sample\_011046841, Unigene10250\_Sample\_011046841, Unigene47025\_Sample\_011046841, Unigene11120\_Sample\_011046841, Unigene50057\_Sample\_011046841, Unigene9354\_Sample\_011046841, Unigene49534\_Sample\_011046841, Unigene18318\_Sample\_011046841, Unigene13920\_Sample\_011046841, Unigene55162\_Sample\_011046841, Unigene48482\_Sample\_011046841, Unigene18749\_Sample\_011046841, Unigene59421\_Sample\_011046841, Unigene54240\_Sample\_011046841, Unigene57589\_Sample\_011046841, Unigene9329\_Sample\_011046841, Unigene2942\_Sample\_011046841, Unigene51845\_Sample\_011046841, Unigene3842\_Sample\_011046841, Unigene59756\_Sample\_011046841, Unigene30484\_Sample\_011046841, Unigene10683\_Sample\_011046841, Unigene55610\_Sample\_011046841, Unigene27464\_Sample\_011046841, Unigene31264\_Sample\_011046841, Unigene26327\_Sample\_011046841, Unigene48009\_Sample\_011046841, Unigene44021\_Sample\_011046841, Unigene6531\_Sample\_011046841, Unigene55126\_Sample\_011046841, Unigene28658\_Sample\_011046841, Unigene59440\_Sample\_011046841, Unigene3363\_Sample\_011046841, Unigene55720\_Sample\_011046841, Unigene60657\_Sample\_011046841, Unigene4361\_Sample\_011046841, Unigene23831\_Sample\_011046841, Unigene60821\_Sample\_011046841, Unigene46283\_Sample\_011046841, Unigene608\_Sample\_011046841, Unigene41574\_Sample\_011046841, Unigene50170\_Sample\_011046841, Unigene58134\_Sample\_011046841, Unigene49671\_Sample\_011046841, Unigene30474\_Sample\_011046841, Unigene47936\_Sample\_011046841, Unigene2933\_Sample\_011046841, Unigene27253\_Sample\_011046841, Unigene35914\_Sample\_011046841, Unigene58460\_Sample\_011046841, Unigene58128\_Sample\_011046841, Unigene4662\_Sample\_011046841, Unigene37707\_Sample\_011046841, Unigene8429\_Sample\_011046841, Unigene36772\_Sample\_011046841, Unigene5431\_Sample\_011046841, Unigene55302\_Sample\_011046841, Unigene15482\_Sample\_011046841, Unigene40258\_Sample\_011046841, Unigene52385\_Sample\_011046841, Unigene56054\_Sample\_011046841, Unigene41682\_Sample\_011046841, Unigene25989\_Sample\_011046841, Unigene60191\_Sample\_011046841, Unigene10726\_Sample\_011046841, Unigene36178\_Sample\_011046841, Unigene42893\_Sample\_011046841, Unigene19352\_Sample\_011046841, Unigene43515\_Sample\_011046841, Unigene13332\_Sample\_011046841, Unigene59891\_Sample\_011046841, Unigene42281\_Sample\_011046841, Unigene4308\_Sample\_011046841, Unigene9348\_Sample\_011046841, Unigene47887\_Sample\_011046841, Unigene47872\_Sample\_011046841, Unigene13884\_Sample\_011046841, Unigene35217\_Sample\_011046841, Unigene16539\_Sample\_011046841, Unigene60220\_Sample\_011046841, Unigene9862\_Sample\_011046841, Unigene59362\_Sample\_011046841, Unigene13722\_Sample\_011046841, Unigene56391\_Sample\_011046841, Unigene40215\_Sample\_011046841, Unigene26192\_Sample\_011046841, Unigene48345\_Sample\_011046841, Unigene12390\_Sample\_011046841, Unigene47910\_Sample\_011046841, Unigene51301\_Sample\_011046841, Unigene42686\_Sample\_011046841, Unigene44734\_Sample\_011046841, Unigene57665\_Sample\_011046841, Unigene30349\_Sample\_011046841, Unigene57904\_Sample\_011046841, Unigene21362\_Sample\_011046841, Unigene32534\_Sample\_011046841, Unigene4432\_Sample\_011046841, Unigene17850\_Sample\_011046841, Unigene14776\_Sample\_011046841, Unigene59844\_Sample\_011046841, Unigene3946\_Sample\_011046841, Unigene7335\_Sample\_011046841, Unigene45885\_Sample\_011046841, Unigene59711\_Sample\_011046841, Unigene13367\_Sample\_011046841, Unigene19594\_Sample\_011046841, Unigene13901\_Sample\_011046841, Unigene6223\_Sample\_011046841, Unigene12837\_Sample\_011046841, Unigene12324\_Sample\_011046841, Unigene25810\_Sample\_011046841, Unigene3810\_Sample\_011046841, Unigene50223\_Sample\_011046841, Unigene50322\_Sample\_011046841, Unigene37057\_Sample\_011046841, Unigene48342\_Sample\_011046841, Unigene55247\_Sample\_011046841, Unigene51736\_Sample\_011046841, Unigene54453\_Sample\_011046841, Unigene9776\_Sample\_011046841, Unigene26757\_Sample\_011046841, Unigene59257\_Sample\_011046841, Unigene51307\_Sample\_011046841, Unigene44683\_Sample\_011046841, Unigene53100\_Sample\_011046841, Unigene9811\_Sample\_011046841, Unigene58021\_Sample\_011046841, Unigene44354\_Sample\_011046841, Unigene9210\_Sample\_011046841, Unigene21539\_Sample\_011046841, Unigene56828\_Sample\_011046841, Unigene6197\_Sample\_011046841, Unigene50758\_Sample\_011046841, Unigene44079\_Sample\_011046841, Unigene26728\_Sample\_011046841, Unigene47555\_Sample\_011046841, Unigene46176\_Sample\_011046841, Unigene51429\_Sample\_011046841, Unigene29587\_Sample\_011046841, Unigene58511\_Sample\_011046841, Unigene32760\_Sample\_011046841, Unigene13321\_Sample\_011046841, Unigene32964\_Sample\_011046841, Unigene39020\_Sample\_011046841, Unigene60217\_Sample\_011046841, Unigene59457\_Sample\_011046841, Unigene49737\_Sample\_011046841, Unigene42633\_Sample\_011046841, Unigene2110\_Sample\_011046841, Unigene12975\_Sample\_011046841, Unigene48267\_Sample\_011046841 |
| kinase activity | Unigene55833\_Sample\_011046841, Unigene55221\_Sample\_011046841, Unigene16112\_Sample\_011046841, Unigene49851\_Sample\_011046841, Unigene51130\_Sample\_011046841, Unigene2784\_Sample\_011046841, Unigene52866\_Sample\_011046841, Unigene10250\_Sample\_011046841, Unigene9858\_Sample\_011046841, Unigene11120\_Sample\_011046841, Unigene9354\_Sample\_011046841, Unigene2263\_Sample\_011046841, Unigene4303\_Sample\_011046841, Unigene41021\_Sample\_011046841, Unigene12637\_Sample\_011046841, Unigene60481\_Sample\_011046841, Unigene16803\_Sample\_011046841, Unigene49534\_Sample\_011046841, Unigene18318\_Sample\_011046841, Unigene60552\_Sample\_011046841, Unigene17236\_Sample\_011046841, Unigene18749\_Sample\_011046841, Unigene11055\_Sample\_011046841, Unigene21225\_Sample\_011046841, Unigene10970\_Sample\_011046841, Unigene56545\_Sample\_011046841, Unigene57634\_Sample\_011046841, Unigene60498\_Sample\_011046841, Unigene57083\_Sample\_011046841, Unigene57589\_Sample\_011046841, Unigene51380\_Sample\_011046841, Unigene60173\_Sample\_011046841, Unigene51845\_Sample\_011046841, Unigene59756\_Sample\_011046841, Unigene45770\_Sample\_011046841, Unigene51582\_Sample\_011046841, Unigene25764\_Sample\_011046841, Unigene49752\_Sample\_011046841, Unigene46485\_Sample\_011046841, Unigene31264\_Sample\_011046841, Unigene50051\_Sample\_011046841, Unigene48009\_Sample\_011046841, Unigene30954\_Sample\_011046841, Unigene59022\_Sample\_011046841, Unigene55126\_Sample\_011046841, Unigene36373\_Sample\_011046841, Unigene26636\_Sample\_011046841, Unigene56529\_Sample\_011046841, Unigene40349\_Sample\_011046841, Unigene60657\_Sample\_011046841, Unigene12309\_Sample\_011046841, Unigene32409\_Sample\_011046841, Unigene10875\_Sample\_011046841, Unigene48847\_Sample\_011046841, Unigene23831\_Sample\_011046841, Unigene43397\_Sample\_011046841, Unigene46283\_Sample\_011046841, Unigene41574\_Sample\_011046841, Unigene13419\_Sample\_011046841, Unigene39238\_Sample\_011046841, Unigene49837\_Sample\_011046841, Unigene30474\_Sample\_011046841, Unigene53360\_Sample\_011046841, Unigene55889\_Sample\_011046841, Unigene31679\_Sample\_011046841, Unigene11457\_Sample\_011046841, Unigene47936\_Sample\_011046841, Unigene49494\_Sample\_011046841, Unigene5554\_Sample\_011046841, Unigene58460\_Sample\_011046841, Unigene56587\_Sample\_011046841, Unigene43878\_Sample\_011046841, Unigene50797\_Sample\_011046841, Unigene4662\_Sample\_011046841, Unigene54614\_Sample\_011046841, Unigene59061\_Sample\_011046841, Unigene11114\_Sample\_011046841, Unigene11130\_Sample\_011046841, Unigene16001\_Sample\_011046841, Unigene59469\_Sample\_011046841, Unigene58100\_Sample\_011046841, Unigene13224\_Sample\_011046841, Unigene59993\_Sample\_011046841, Unigene13287\_Sample\_011046841, Unigene15490\_Sample\_011046841, Unigene11231\_Sample\_011046841, Unigene56468\_Sample\_011046841, Unigene26229\_Sample\_011046841, Unigene56054\_Sample\_011046841, Unigene6945\_Sample\_011046841, Unigene50506\_Sample\_011046841, Unigene10726\_Sample\_011046841, Unigene26777\_Sample\_011046841, Unigene56258\_Sample\_011046841, Unigene42893\_Sample\_011046841, Unigene11925\_Sample\_011046841, Unigene57775\_Sample\_011046841, Unigene4545\_Sample\_011046841, Unigene21456\_Sample\_011046841, Unigene49168\_Sample\_011046841, Unigene23925\_Sample\_011046841, Unigene9348\_Sample\_011046841, Unigene43028\_Sample\_011046841, Unigene36850\_Sample\_011046841, Unigene9862\_Sample\_011046841, Unigene13368\_Sample\_011046841, Unigene58386\_Sample\_011046841, Unigene27682\_Sample\_011046841, Unigene48345\_Sample\_011046841, Unigene12390\_Sample\_011046841, Unigene49895\_Sample\_011046841, Unigene1107\_Sample\_011046841, Unigene13633\_Sample\_011046841, Unigene45307\_Sample\_011046841, Unigene14248\_Sample\_011046841, Unigene25326\_Sample\_011046841, Unigene60344\_Sample\_011046841, Unigene16442\_Sample\_011046841, Unigene49487\_Sample\_011046841, Unigene57904\_Sample\_011046841, Unigene40509\_Sample\_011046841, Unigene53668\_Sample\_011046841, Unigene43718\_Sample\_011046841, Unigene17850\_Sample\_011046841, Unigene50713\_Sample\_011046841, Unigene59669\_Sample\_011046841, Unigene23590\_Sample\_011046841, Unigene35290\_Sample\_011046841, Unigene48170\_Sample\_011046841, Unigene5220\_Sample\_011046841, Unigene47967\_Sample\_011046841, Unigene21150\_Sample\_011046841, Unigene47448\_Sample\_011046841, Unigene39488\_Sample\_011046841, Unigene6223\_Sample\_011046841, Unigene57459\_Sample\_011046841, Unigene27562\_Sample\_011046841, Unigene11073\_Sample\_011046841, Unigene31588\_Sample\_011046841, Unigene42605\_Sample\_011046841, Unigene25810\_Sample\_011046841, Unigene59850\_Sample\_011046841, Unigene52322\_Sample\_011046841, Unigene38202\_Sample\_011046841, Unigene50223\_Sample\_011046841, Unigene3269\_Sample\_011046841, Unigene36927\_Sample\_011046841, Unigene38603\_Sample\_011046841, Unigene47707\_Sample\_011046841, Unigene26587\_Sample\_011046841, Unigene54453\_Sample\_011046841, Unigene59257\_Sample\_011046841, Unigene53100\_Sample\_011046841, Unigene46256\_Sample\_011046841, Unigene26679\_Sample\_011046841, Unigene44079\_Sample\_011046841, Unigene11040\_Sample\_011046841, Unigene7042\_Sample\_011046841, Unigene60753\_Sample\_011046841, Unigene60166\_Sample\_011046841, Unigene32666\_Sample\_011046841, Unigene53884\_Sample\_011046841, Unigene13321\_Sample\_011046841, Unigene50981\_Sample\_011046841, Unigene49264\_Sample\_011046841, Unigene52573\_Sample\_011046841, Unigene50596\_Sample\_011046841, Unigene48062\_Sample\_011046841, Unigene35715\_Sample\_011046841, Unigene50163\_Sample\_011046841, Unigene53819\_Sample\_011046841, Unigene59457\_Sample\_011046841, Unigene57004\_Sample\_011046841, Unigene49737\_Sample\_011046841, Unigene11867\_Sample\_011046841, Unigene45724\_Sample\_011046841, Unigene35082\_Sample\_011046841, Unigene5623\_Sample\_011046841, Unigene60746\_Sample\_011046841, Unigene55041\_Sample\_011046841, Unigene8288\_Sample\_011046841, Unigene33400\_Sample\_011046841, Unigene49268\_Sample\_011046841, Unigene16813\_Sample\_011046841, Unigene31749\_Sample\_011046841, Unigene59840\_Sample\_011046841, Unigene18294\_Sample\_011046841, Unigene54459\_Sample\_011046841, Unigene17843\_Sample\_011046841, Unigene12358\_Sample\_011046841, Unigene50677\_Sample\_011046841, Unigene6597\_Sample\_011046841, Unigene54982\_Sample\_011046841, Unigene52144\_Sample\_011046841, Unigene52097\_Sample\_011046841, Unigene47591\_Sample\_011046841, Unigene35398\_Sample\_011046841, Unigene60809\_Sample\_011046841, Unigene32054\_Sample\_011046841, Unigene11932\_Sample\_011046841, Unigene54833\_Sample\_011046841, Unigene58427\_Sample\_011046841, Unigene8997\_Sample\_011046841, Unigene53096\_Sample\_011046841, Unigene47085\_Sample\_011046841, Unigene42901\_Sample\_011046841, Unigene53865\_Sample\_011046841, Unigene58913\_Sample\_011046841, Unigene9329\_Sample\_011046841, Unigene51958\_Sample\_011046841, Unigene60244\_Sample\_011046841, Unigene42912\_Sample\_011046841, Unigene53714\_Sample\_011046841, Unigene21655\_Sample\_011046841, Unigene56744\_Sample\_011046841, Unigene2076\_Sample\_011046841, Unigene27464\_Sample\_011046841, Unigene48081\_Sample\_011046841, Unigene9052\_Sample\_011046841, Unigene12835\_Sample\_011046841, Unigene44021\_Sample\_011046841, Unigene60900\_Sample\_011046841, Unigene28658\_Sample\_011046841, Unigene59440\_Sample\_011046841, Unigene15\_Sample\_011046841, Unigene3363\_Sample\_011046841, Unigene55261\_Sample\_011046841, Unigene52294\_Sample\_011046841, Unigene13782\_Sample\_011046841, Unigene60821\_Sample\_011046841, Unigene32077\_Sample\_011046841, Unigene49671\_Sample\_011046841, Unigene60434\_Sample\_011046841, Unigene2493\_Sample\_011046841, Unigene4305\_Sample\_011046841, Unigene53958\_Sample\_011046841, Unigene57278\_Sample\_011046841, Unigene47210\_Sample\_011046841, Unigene22964\_Sample\_011046841, Unigene8429\_Sample\_011046841, Unigene5431\_Sample\_011046841, Unigene56566\_Sample\_011046841, Unigene15482\_Sample\_011046841, Unigene40258\_Sample\_011046841, Unigene5972\_Sample\_011046841, Unigene9219\_Sample\_011046841, Unigene41676\_Sample\_011046841, Unigene40209\_Sample\_011046841, Unigene60191\_Sample\_011046841, Unigene46813\_Sample\_011046841, Unigene49407\_Sample\_011046841, Unigene12359\_Sample\_011046841, Unigene49729\_Sample\_011046841, Unigene59891\_Sample\_011046841, Unigene13966\_Sample\_011046841, Unigene4308\_Sample\_011046841, Unigene42281\_Sample\_011046841, Unigene28288\_Sample\_011046841, Unigene17643\_Sample\_011046841, Unigene7809\_Sample\_011046841, Unigene13884\_Sample\_011046841, Unigene42820\_Sample\_011046841, Unigene19556\_Sample\_011046841, Unigene57475\_Sample\_011046841, Unigene11367\_Sample\_011046841, Unigene50584\_Sample\_011046841, Unigene59362\_Sample\_011046841, Unigene20267\_Sample\_011046841, Unigene47078\_Sample\_011046841, Unigene60149\_Sample\_011046841, Unigene34207\_Sample\_011046841, Unigene49640\_Sample\_011046841, Unigene55947\_Sample\_011046841, Unigene59492\_Sample\_011046841, Unigene44734\_Sample\_011046841, Unigene51882\_Sample\_011046841, Unigene20770\_Sample\_011046841, Unigene30349\_Sample\_011046841, Unigene55101\_Sample\_011046841, Unigene39766\_Sample\_011046841, Unigene7197\_Sample\_011046841, Unigene14776\_Sample\_011046841, Unigene17021\_Sample\_011046841, Unigene40544\_Sample\_011046841, Unigene11314\_Sample\_011046841, Unigene41694\_Sample\_011046841, Unigene31544\_Sample\_011046841, Unigene3946\_Sample\_011046841, Unigene49525\_Sample\_011046841, Unigene19594\_Sample\_011046841, Unigene42105\_Sample\_011046841, Unigene58882\_Sample\_011046841, Unigene11758\_Sample\_011046841, Unigene21297\_Sample\_011046841, Unigene37492\_Sample\_011046841, Unigene58321\_Sample\_011046841, Unigene12837\_Sample\_011046841, Unigene39083\_Sample\_011046841, Unigene11706\_Sample\_011046841, Unigene12324\_Sample\_011046841, Unigene3797\_Sample\_011046841, Unigene58669\_Sample\_011046841, Unigene1803\_Sample\_011046841, Unigene50322\_Sample\_011046841, Unigene56096\_Sample\_011046841, Unigene55247\_Sample\_011046841, Unigene30285\_Sample\_011046841, Unigene47560\_Sample\_011046841, Unigene48730\_Sample\_011046841, Unigene5953\_Sample\_011046841, Unigene54870\_Sample\_011046841, Unigene13131\_Sample\_011046841, Unigene46783\_Sample\_011046841, Unigene11520\_Sample\_011046841, Unigene44354\_Sample\_011046841, Unigene21539\_Sample\_011046841, Unigene50758\_Sample\_011046841, Unigene26728\_Sample\_011046841, Unigene5744\_Sample\_011046841, Unigene60458\_Sample\_011046841, Unigene47555\_Sample\_011046841, Unigene60441\_Sample\_011046841, Unigene27550\_Sample\_011046841, Unigene37642\_Sample\_011046841, Unigene55564\_Sample\_011046841, Unigene49204\_Sample\_011046841, Unigene29587\_Sample\_011046841, Unigene23757\_Sample\_011046841, Unigene11667\_Sample\_011046841, Unigene19215\_Sample\_011046841, Unigene11676\_Sample\_011046841, Unigene8886\_Sample\_011046841, Unigene39020\_Sample\_011046841, Unigene60217\_Sample\_011046841, Unigene57766\_Sample\_011046841, Unigene9082\_Sample\_011046841, Unigene50470\_Sample\_011046841, Unigene50293\_Sample\_011046841, Unigene42561\_Sample\_011046841, Unigene55621\_Sample\_011046841, Unigene57972\_Sample\_011046841, Unigene58864\_Sample\_011046841, Unigene47923\_Sample\_011046841, Unigene54668\_Sample\_011046841, Unigene31969\_Sample\_011046841, Unigene56851\_Sample\_011046841, Unigene33510\_Sample\_011046841, Unigene39621\_Sample\_011046841, Unigene51777\_Sample\_011046841, Unigene33573\_Sample\_011046841, Unigene43216\_Sample\_011046841, Unigene55162\_Sample\_011046841, Unigene30971\_Sample\_011046841, Unigene48482\_Sample\_011046841, Unigene57699\_Sample\_011046841, Unigene23971\_Sample\_011046841, Unigene53995\_Sample\_011046841, Unigene12538\_Sample\_011046841, Unigene59421\_Sample\_011046841, Unigene54240\_Sample\_011046841, Unigene51706\_Sample\_011046841, Unigene54683\_Sample\_011046841, Unigene9546\_Sample\_011046841, Unigene54701\_Sample\_011046841, Unigene2942\_Sample\_011046841, Unigene41318\_Sample\_011046841, Unigene32119\_Sample\_011046841, Unigene56697\_Sample\_011046841, Unigene40435\_Sample\_011046841, Unigene10683\_Sample\_011046841, Unigene36154\_Sample\_011046841, Unigene13672\_Sample\_011046841, Unigene4209\_Sample\_011046841, Unigene55610\_Sample\_011046841, Unigene13068\_Sample\_011046841, Unigene7879\_Sample\_011046841, Unigene26327\_Sample\_011046841, Unigene59593\_Sample\_011046841, Unigene60190\_Sample\_011046841, Unigene60525\_Sample\_011046841, Unigene37753\_Sample\_011046841, Unigene57641\_Sample\_011046841, Unigene10198\_Sample\_011046841, Unigene4361\_Sample\_011046841, Unigene8798\_Sample\_011046841, Unigene24424\_Sample\_011046841, Unigene608\_Sample\_011046841, Unigene58134\_Sample\_011046841, Unigene47924\_Sample\_011046841, Unigene50170\_Sample\_011046841, Unigene52918\_Sample\_011046841, Unigene19453\_Sample\_011046841, Unigene27253\_Sample\_011046841, Unigene35914\_Sample\_011046841, Unigene60557\_Sample\_011046841, Unigene43504\_Sample\_011046841, Unigene58128\_Sample\_011046841, Unigene39675\_Sample\_011046841, Unigene36772\_Sample\_011046841, Unigene47292\_Sample\_011046841, Unigene51537\_Sample\_011046841, Unigene52610\_Sample\_011046841, Unigene5297\_Sample\_011046841, Unigene60576\_Sample\_011046841, Unigene54841\_Sample\_011046841, Unigene57832\_Sample\_011046841, Unigene7885\_Sample\_011046841, Unigene9879\_Sample\_011046841, Unigene19352\_Sample\_011046841, Unigene59448\_Sample\_011046841, Unigene41032\_Sample\_011046841, Unigene13332\_Sample\_011046841, Unigene301\_Sample\_011046841, Unigene47887\_Sample\_011046841, Unigene47872\_Sample\_011046841, Unigene58058\_Sample\_011046841, Unigene51573\_Sample\_011046841, Unigene37549\_Sample\_011046841, Unigene60248\_Sample\_011046841, Unigene54379\_Sample\_011046841, Unigene56798\_Sample\_011046841, Unigene16539\_Sample\_011046841, Unigene56182\_Sample\_011046841, Unigene10522\_Sample\_011046841, Unigene13722\_Sample\_011046841, Unigene23133\_Sample\_011046841, Unigene59664\_Sample\_011046841, Unigene26192\_Sample\_011046841, Unigene42399\_Sample\_011046841, Unigene51301\_Sample\_011046841, Unigene59727\_Sample\_011046841, Unigene58516\_Sample\_011046841, Unigene42686\_Sample\_011046841, Unigene52838\_Sample\_011046841, Unigene11547\_Sample\_011046841, Unigene30190\_Sample\_011046841, Unigene21362\_Sample\_011046841, Unigene9803\_Sample\_011046841, Unigene16977\_Sample\_011046841, Unigene44090\_Sample\_011046841, Unigene38549\_Sample\_011046841, Unigene59844\_Sample\_011046841, Unigene60349\_Sample\_011046841, Unigene12510\_Sample\_011046841, Unigene49055\_Sample\_011046841, Unigene28563\_Sample\_011046841, Unigene51916\_Sample\_011046841, Unigene7335\_Sample\_011046841, Unigene60\_Sample\_011046841, Unigene55423\_Sample\_011046841, Unigene13901\_Sample\_011046841, Unigene60507\_Sample\_011046841, Unigene55644\_Sample\_011046841, Unigene52071\_Sample\_011046841, Unigene3810\_Sample\_011046841, Unigene54626\_Sample\_011046841, Unigene55404\_Sample\_011046841, Unigene41464\_Sample\_011046841, Unigene52731\_Sample\_011046841, Unigene22189\_Sample\_011046841, Unigene59559\_Sample\_011046841, Unigene44600\_Sample\_011046841, Unigene20667\_Sample\_011046841, Unigene37057\_Sample\_011046841, Unigene48342\_Sample\_011046841, Unigene45949\_Sample\_011046841, Unigene13197\_Sample\_011046841, Unigene18791\_Sample\_011046841, Unigene11419\_Sample\_011046841, Unigene9776\_Sample\_011046841, Unigene26757\_Sample\_011046841, Unigene59228\_Sample\_011046841, Unigene48530\_Sample\_011046841, Unigene51307\_Sample\_011046841, Unigene46142\_Sample\_011046841, Unigene9811\_Sample\_011046841, Unigene58021\_Sample\_011046841, Unigene44103\_Sample\_011046841, Unigene51739\_Sample\_011046841, Unigene59221\_Sample\_011046841, Unigene57130\_Sample\_011046841, Unigene46176\_Sample\_011046841, Unigene59987\_Sample\_011046841, Unigene32760\_Sample\_011046841, Unigene60721\_Sample\_011046841, Unigene60042\_Sample\_011046841, Unigene54549\_Sample\_011046841, Unigene32964\_Sample\_011046841, Unigene37367\_Sample\_011046841, Unigene42633\_Sample\_011046841, Unigene52413\_Sample\_011046841, Unigene12975\_Sample\_011046841, Unigene48267\_Sample\_011046841, Unigene1793\_Sample\_011046841, Unigene54187\_Sample\_011046841, Unigene41680\_Sample\_011046841, Unigene54707\_Sample\_011046841, Unigene52842\_Sample\_011046841, Unigene11998\_Sample\_011046841, Unigene58523\_Sample\_011046841, Unigene60540\_Sample\_011046841, Unigene11526\_Sample\_011046841, Unigene29318\_Sample\_011046841, Unigene45662\_Sample\_011046841, Unigene50039\_Sample\_011046841, Unigene32914\_Sample\_011046841, Unigene12720\_Sample\_011046841, Unigene52094\_Sample\_011046841, Unigene16067\_Sample\_011046841, Unigene47025\_Sample\_011046841, Unigene39219\_Sample\_011046841, Unigene7481\_Sample\_011046841, Unigene50057\_Sample\_011046841, Unigene54574\_Sample\_011046841, Unigene59705\_Sample\_011046841, Unigene15705\_Sample\_011046841, Unigene59226\_Sample\_011046841, Unigene58675\_Sample\_011046841, Unigene13920\_Sample\_011046841, Unigene34330\_Sample\_011046841, Unigene59758\_Sample\_011046841, Unigene7964\_Sample\_011046841, Unigene3842\_Sample\_011046841, Unigene30484\_Sample\_011046841, Unigene56439\_Sample\_011046841, Unigene28789\_Sample\_011046841, Unigene57225\_Sample\_011046841, Unigene24615\_Sample\_011046841, Unigene40166\_Sample\_011046841, Unigene3750\_Sample\_011046841, Unigene6531\_Sample\_011046841, Unigene55517\_Sample\_011046841, Unigene58285\_Sample\_011046841, Unigene5051\_Sample\_011046841, Unigene34824\_Sample\_011046841, Unigene10024\_Sample\_011046841, Unigene19343\_Sample\_011046841, Unigene56191\_Sample\_011046841, Unigene20191\_Sample\_011046841, Unigene55720\_Sample\_011046841, Unigene57735\_Sample\_011046841, Unigene43936\_Sample\_011046841, Unigene12652\_Sample\_011046841, Unigene55461\_Sample\_011046841, Unigene13516\_Sample\_011046841, Unigene20201\_Sample\_011046841, Unigene2933\_Sample\_011046841, Unigene30684\_Sample\_011046841, Unigene59459\_Sample\_011046841, Unigene2116\_Sample\_011046841, Unigene52959\_Sample\_011046841, Unigene48334\_Sample\_011046841, Unigene51756\_Sample\_011046841, Unigene23218\_Sample\_011046841, Unigene18170\_Sample\_011046841, Unigene37707\_Sample\_011046841, Unigene55302\_Sample\_011046841, Unigene21879\_Sample\_011046841, Unigene12972\_Sample\_011046841, Unigene35470\_Sample\_011046841, Unigene47379\_Sample\_011046841, Unigene18796\_Sample\_011046841, Unigene58821\_Sample\_011046841, Unigene59109\_Sample\_011046841, Unigene52385\_Sample\_011046841, Unigene41682\_Sample\_011046841, Unigene25989\_Sample\_011046841, Unigene38223\_Sample\_011046841, Unigene50185\_Sample\_011046841, Unigene36178\_Sample\_011046841, Unigene58254\_Sample\_011046841, Unigene43515\_Sample\_011046841, Unigene29314\_Sample\_011046841, Unigene9017\_Sample\_011046841, Unigene51286\_Sample\_011046841, Unigene58340\_Sample\_011046841, Unigene57602\_Sample\_011046841, Unigene35217\_Sample\_011046841, Unigene58042\_Sample\_011046841, Unigene9600\_Sample\_011046841, Unigene60220\_Sample\_011046841, Unigene51011\_Sample\_011046841, Unigene56391\_Sample\_011046841, Unigene40215\_Sample\_011046841, Unigene6590\_Sample\_011046841, Unigene47910\_Sample\_011046841, Unigene10980\_Sample\_011046841, Unigene60847\_Sample\_011046841, Unigene12824\_Sample\_011046841, Unigene47387\_Sample\_011046841, Unigene60733\_Sample\_011046841, Unigene29803\_Sample\_011046841, Unigene46613\_Sample\_011046841, Unigene57887\_Sample\_011046841, Unigene16051\_Sample\_011046841, Unigene24332\_Sample\_011046841, Unigene56062\_Sample\_011046841, Unigene57665\_Sample\_011046841, Unigene8000\_Sample\_011046841, Unigene48528\_Sample\_011046841, Unigene32534\_Sample\_011046841, Unigene4432\_Sample\_011046841, Unigene46198\_Sample\_011046841, Unigene39412\_Sample\_011046841, Unigene3199\_Sample\_011046841, Unigene29813\_Sample\_011046841, Unigene58911\_Sample\_011046841, Unigene34757\_Sample\_011046841, Unigene56567\_Sample\_011046841, Unigene45885\_Sample\_011046841, Unigene59711\_Sample\_011046841, Unigene47811\_Sample\_011046841, Unigene13367\_Sample\_011046841, Unigene35586\_Sample\_011046841, Unigene9774\_Sample\_011046841, Unigene11504\_Sample\_011046841, Unigene57194\_Sample\_011046841, Unigene15343\_Sample\_011046841, Unigene30456\_Sample\_011046841, Unigene58180\_Sample\_011046841, Unigene55486\_Sample\_011046841, Unigene32509\_Sample\_011046841, Unigene43935\_Sample\_011046841, Unigene39709\_Sample\_011046841, Unigene56843\_Sample\_011046841, Unigene60096\_Sample\_011046841, Unigene18856\_Sample\_011046841, Unigene60181\_Sample\_011046841, Unigene40491\_Sample\_011046841, Unigene51736\_Sample\_011046841, Unigene59596\_Sample\_011046841, Unigene47686\_Sample\_011046841, Unigene6435\_Sample\_011046841, Unigene54599\_Sample\_011046841, Unigene43792\_Sample\_011046841, Unigene43367\_Sample\_011046841, Unigene44683\_Sample\_011046841, Unigene60126\_Sample\_011046841, Unigene9210\_Sample\_011046841, Unigene6197\_Sample\_011046841, Unigene56828\_Sample\_011046841, Unigene35468\_Sample\_011046841, Unigene57305\_Sample\_011046841, Unigene53723\_Sample\_011046841, Unigene45824\_Sample\_011046841, Unigene57255\_Sample\_011046841, Unigene17671\_Sample\_011046841, Unigene51429\_Sample\_011046841, Unigene58511\_Sample\_011046841, Unigene16046\_Sample\_011046841, Unigene38527\_Sample\_011046841, Unigene48647\_Sample\_011046841, Unigene59868\_Sample\_011046841, Unigene40350\_Sample\_011046841, Unigene49635\_Sample\_011046841, Unigene60859\_Sample\_011046841, Unigene2110\_Sample\_011046841, Unigene46224\_Sample\_011046841 |
| transferase activity, transferring phosphorus-containing groups | Unigene55833\_Sample\_011046841, Unigene55221\_Sample\_011046841, Unigene57770\_Sample\_011046841, Unigene16112\_Sample\_011046841, Unigene49851\_Sample\_011046841, Unigene51130\_Sample\_011046841, Unigene49827\_Sample\_011046841, Unigene2784\_Sample\_011046841, Unigene52866\_Sample\_011046841, Unigene10250\_Sample\_011046841, Unigene9858\_Sample\_011046841, Unigene11120\_Sample\_011046841, Unigene9354\_Sample\_011046841, Unigene29684\_Sample\_011046841, Unigene2263\_Sample\_011046841, Unigene4303\_Sample\_011046841, Unigene41021\_Sample\_011046841, Unigene12637\_Sample\_011046841, Unigene60481\_Sample\_011046841, Unigene16803\_Sample\_011046841, Unigene49534\_Sample\_011046841, Unigene18318\_Sample\_011046841, Unigene60552\_Sample\_011046841, Unigene35210\_Sample\_011046841, Unigene17236\_Sample\_011046841, Unigene18749\_Sample\_011046841, Unigene32237\_Sample\_011046841, Unigene11055\_Sample\_011046841, Unigene21225\_Sample\_011046841, Unigene10970\_Sample\_011046841, Unigene56545\_Sample\_011046841, Unigene50631\_Sample\_011046841, Unigene57634\_Sample\_011046841, Unigene60498\_Sample\_011046841, Unigene57083\_Sample\_011046841, Unigene57589\_Sample\_011046841, Unigene51380\_Sample\_011046841, Unigene25028\_Sample\_011046841, Unigene60173\_Sample\_011046841, Unigene51845\_Sample\_011046841, Unigene59756\_Sample\_011046841, Unigene45770\_Sample\_011046841, Unigene51582\_Sample\_011046841, Unigene38646\_Sample\_011046841, Unigene25764\_Sample\_011046841, Unigene49752\_Sample\_011046841, Unigene46485\_Sample\_011046841, Unigene31264\_Sample\_011046841, Unigene50051\_Sample\_011046841, Unigene48009\_Sample\_011046841, Unigene30954\_Sample\_011046841, Unigene59022\_Sample\_011046841, Unigene55126\_Sample\_011046841, Unigene36373\_Sample\_011046841, Unigene26636\_Sample\_011046841, Unigene56529\_Sample\_011046841, Unigene40349\_Sample\_011046841, Unigene60657\_Sample\_011046841, Unigene12309\_Sample\_011046841, Unigene32409\_Sample\_011046841, Unigene32413\_Sample\_011046841, Unigene10875\_Sample\_011046841, Unigene48847\_Sample\_011046841, Unigene23831\_Sample\_011046841, Unigene43397\_Sample\_011046841, Unigene55981\_Sample\_011046841, Unigene46283\_Sample\_011046841, Unigene41574\_Sample\_011046841, Unigene13419\_Sample\_011046841, Unigene39238\_Sample\_011046841, Unigene49837\_Sample\_011046841, Unigene30474\_Sample\_011046841, Unigene53360\_Sample\_011046841, Unigene55889\_Sample\_011046841, Unigene31679\_Sample\_011046841, Unigene11457\_Sample\_011046841, Unigene47936\_Sample\_011046841, Unigene49494\_Sample\_011046841, Unigene43536\_Sample\_011046841, Unigene5554\_Sample\_011046841, Unigene58460\_Sample\_011046841, Unigene58858\_Sample\_011046841, Unigene56587\_Sample\_011046841, Unigene39762\_Sample\_011046841, Unigene43878\_Sample\_011046841, Unigene50797\_Sample\_011046841, Unigene4662\_Sample\_011046841, Unigene56251\_Sample\_011046841, Unigene54614\_Sample\_011046841, Unigene59061\_Sample\_011046841, Unigene11114\_Sample\_011046841, Unigene11130\_Sample\_011046841, Unigene16001\_Sample\_011046841, Unigene59469\_Sample\_011046841, Unigene58100\_Sample\_011046841, Unigene13224\_Sample\_011046841, Unigene59993\_Sample\_011046841, Unigene13287\_Sample\_011046841, Unigene15490\_Sample\_011046841, Unigene11231\_Sample\_011046841, Unigene56468\_Sample\_011046841, Unigene26229\_Sample\_011046841, Unigene56054\_Sample\_011046841, Unigene42733\_Sample\_011046841, Unigene6945\_Sample\_011046841, Unigene50506\_Sample\_011046841, Unigene10726\_Sample\_011046841, Unigene1129\_Sample\_011046841, Unigene26777\_Sample\_011046841, Unigene56258\_Sample\_011046841, Unigene42893\_Sample\_011046841, Unigene22992\_Sample\_011046841, Unigene11925\_Sample\_011046841, Unigene57775\_Sample\_011046841, Unigene4545\_Sample\_011046841, Unigene21456\_Sample\_011046841, Unigene49168\_Sample\_011046841, Unigene23925\_Sample\_011046841, Unigene9348\_Sample\_011046841, Unigene43028\_Sample\_011046841, Unigene49900\_Sample\_011046841, Unigene32062\_Sample\_011046841, Unigene51837\_Sample\_011046841, Unigene36850\_Sample\_011046841, Unigene9862\_Sample\_011046841, Unigene13368\_Sample\_011046841, Unigene58386\_Sample\_011046841, Unigene56825\_Sample\_011046841, Unigene27682\_Sample\_011046841, Unigene48345\_Sample\_011046841, Unigene52614\_Sample\_011046841, Unigene12390\_Sample\_011046841, Unigene49895\_Sample\_011046841, Unigene41848\_Sample\_011046841, Unigene1107\_Sample\_011046841, Unigene13633\_Sample\_011046841, Unigene34778\_Sample\_011046841, Unigene57802\_Sample\_011046841, Unigene45307\_Sample\_011046841, Unigene14248\_Sample\_011046841, Unigene25326\_Sample\_011046841, Unigene60344\_Sample\_011046841, Unigene46703\_Sample\_011046841, Unigene23752\_Sample\_011046841, Unigene16442\_Sample\_011046841, Unigene49487\_Sample\_011046841, Unigene57904\_Sample\_011046841, Unigene43894\_Sample\_011046841, Unigene40509\_Sample\_011046841, Unigene53668\_Sample\_011046841, Unigene43718\_Sample\_011046841, Unigene17850\_Sample\_011046841, Unigene50713\_Sample\_011046841, Unigene59669\_Sample\_011046841, Unigene23590\_Sample\_011046841, Unigene35290\_Sample\_011046841, Unigene48170\_Sample\_011046841, Unigene55183\_Sample\_011046841, Unigene5220\_Sample\_011046841, Unigene4847\_Sample\_011046841, Unigene47967\_Sample\_011046841, Unigene21150\_Sample\_011046841, Unigene59567\_Sample\_011046841, Unigene47448\_Sample\_011046841, Unigene39488\_Sample\_011046841, Unigene6223\_Sample\_011046841, Unigene57459\_Sample\_011046841, Unigene27562\_Sample\_011046841, Unigene11073\_Sample\_011046841, Unigene31588\_Sample\_011046841, Unigene42605\_Sample\_011046841, Unigene25810\_Sample\_011046841, Unigene59850\_Sample\_011046841, Unigene52322\_Sample\_011046841, Unigene38202\_Sample\_011046841, Unigene13080\_Sample\_011046841, Unigene50223\_Sample\_011046841, Unigene3269\_Sample\_011046841, Unigene36927\_Sample\_011046841, Unigene38603\_Sample\_011046841, Unigene47707\_Sample\_011046841, Unigene26587\_Sample\_011046841, Unigene54453\_Sample\_011046841, Unigene59257\_Sample\_011046841, Unigene34482\_Sample\_011046841, Unigene53100\_Sample\_011046841, Unigene46256\_Sample\_011046841, Unigene26679\_Sample\_011046841, Unigene44079\_Sample\_011046841, Unigene11040\_Sample\_011046841, Unigene7042\_Sample\_011046841, Unigene60805\_Sample\_011046841, Unigene60753\_Sample\_011046841, Unigene57151\_Sample\_011046841, Unigene54167\_Sample\_011046841, Unigene60166\_Sample\_011046841, Unigene32666\_Sample\_011046841, Unigene16727\_Sample\_011046841, Unigene30787\_Sample\_011046841, Unigene53884\_Sample\_011046841, Unigene13321\_Sample\_011046841, Unigene50981\_Sample\_011046841, Unigene49264\_Sample\_011046841, Unigene15593\_Sample\_011046841, Unigene52573\_Sample\_011046841, Unigene50596\_Sample\_011046841, Unigene48062\_Sample\_011046841, Unigene35715\_Sample\_011046841, Unigene54790\_Sample\_011046841, Unigene50163\_Sample\_011046841, Unigene53819\_Sample\_011046841, Unigene39016\_Sample\_011046841, Unigene59457\_Sample\_011046841, Unigene57004\_Sample\_011046841, Unigene49737\_Sample\_011046841, Unigene11867\_Sample\_011046841, Unigene45724\_Sample\_011046841, Unigene35082\_Sample\_011046841, Unigene5623\_Sample\_011046841, Unigene60746\_Sample\_011046841, Unigene58955\_Sample\_011046841, Unigene29653\_Sample\_011046841, Unigene55041\_Sample\_011046841, Unigene8288\_Sample\_011046841, Unigene33400\_Sample\_011046841, Unigene49268\_Sample\_011046841, Unigene57100\_Sample\_011046841, Unigene16813\_Sample\_011046841, Unigene31642\_Sample\_011046841, Unigene31749\_Sample\_011046841, Unigene59840\_Sample\_011046841, Unigene18294\_Sample\_011046841, Unigene54459\_Sample\_011046841, Unigene17843\_Sample\_011046841, Unigene12358\_Sample\_011046841, Unigene50677\_Sample\_011046841, Unigene6597\_Sample\_011046841, Unigene54982\_Sample\_011046841, Unigene52144\_Sample\_011046841, Unigene52097\_Sample\_011046841, Unigene47591\_Sample\_011046841, Unigene35398\_Sample\_011046841, Unigene60809\_Sample\_011046841, Unigene32054\_Sample\_011046841, Unigene51456\_Sample\_011046841, Unigene11932\_Sample\_011046841, Unigene54833\_Sample\_011046841, Unigene45838\_Sample\_011046841, Unigene58427\_Sample\_011046841, Unigene60494\_Sample\_011046841, Unigene34404\_Sample\_011046841, Unigene8997\_Sample\_011046841, Unigene53096\_Sample\_011046841, Unigene25475\_Sample\_011046841, Unigene47085\_Sample\_011046841, Unigene42901\_Sample\_011046841, Unigene53865\_Sample\_011046841, Unigene58913\_Sample\_011046841, Unigene37401\_Sample\_011046841, Unigene9329\_Sample\_011046841, Unigene51958\_Sample\_011046841, Unigene60244\_Sample\_011046841, Unigene42912\_Sample\_011046841, Unigene53714\_Sample\_011046841, Unigene21655\_Sample\_011046841, Unigene56744\_Sample\_011046841, Unigene55275\_Sample\_011046841, Unigene27464\_Sample\_011046841, Unigene2076\_Sample\_011046841, Unigene48081\_Sample\_011046841, Unigene9052\_Sample\_011046841, Unigene12835\_Sample\_011046841, Unigene44021\_Sample\_011046841, Unigene60900\_Sample\_011046841, Unigene16924\_Sample\_011046841, Unigene28658\_Sample\_011046841, Unigene59440\_Sample\_011046841, Unigene47820\_Sample\_011046841, Unigene15\_Sample\_011046841, Unigene3363\_Sample\_011046841, Unigene29271\_Sample\_011046841, Unigene55261\_Sample\_011046841, Unigene13794\_Sample\_011046841, Unigene52294\_Sample\_011046841, Unigene13782\_Sample\_011046841, Unigene60821\_Sample\_011046841, Unigene32077\_Sample\_011046841, Unigene2493\_Sample\_011046841, Unigene49671\_Sample\_011046841, Unigene60434\_Sample\_011046841, Unigene4305\_Sample\_011046841, Unigene53958\_Sample\_011046841, Unigene57278\_Sample\_011046841, Unigene47210\_Sample\_011046841, Unigene22964\_Sample\_011046841, Unigene8429\_Sample\_011046841, Unigene5431\_Sample\_011046841, Unigene56566\_Sample\_011046841, Unigene15482\_Sample\_011046841, Unigene40258\_Sample\_011046841, Unigene5972\_Sample\_011046841, Unigene9219\_Sample\_011046841, Unigene41676\_Sample\_011046841, Unigene40209\_Sample\_011046841, Unigene60191\_Sample\_011046841, Unigene37438\_Sample\_011046841, Unigene49290\_Sample\_011046841, Unigene46813\_Sample\_011046841, Unigene56049\_Sample\_011046841, Unigene49407\_Sample\_011046841, Unigene12359\_Sample\_011046841, Unigene60919\_Sample\_011046841, Unigene49729\_Sample\_011046841, Unigene59891\_Sample\_011046841, Unigene13966\_Sample\_011046841, Unigene4308\_Sample\_011046841, Unigene42281\_Sample\_011046841, Unigene39361\_Sample\_011046841, Unigene48876\_Sample\_011046841, Unigene28288\_Sample\_011046841, Unigene17643\_Sample\_011046841, Unigene7809\_Sample\_011046841, Unigene13884\_Sample\_011046841, Unigene30813\_Sample\_011046841, Unigene2821\_Sample\_011046841, Unigene42820\_Sample\_011046841, Unigene19556\_Sample\_011046841, Unigene24401\_Sample\_011046841, Unigene25908\_Sample\_011046841, Unigene57475\_Sample\_011046841, Unigene11367\_Sample\_011046841, Unigene50584\_Sample\_011046841, Unigene59362\_Sample\_011046841, Unigene20267\_Sample\_011046841, Unigene47078\_Sample\_011046841, Unigene60149\_Sample\_011046841, Unigene43909\_Sample\_011046841, Unigene34207\_Sample\_011046841, Unigene50674\_Sample\_011046841, Unigene49640\_Sample\_011046841, Unigene55947\_Sample\_011046841, Unigene59492\_Sample\_011046841, Unigene44734\_Sample\_011046841, Unigene51882\_Sample\_011046841, Unigene20770\_Sample\_011046841, Unigene12300\_Sample\_011046841, Unigene30349\_Sample\_011046841, Unigene55101\_Sample\_011046841, Unigene39766\_Sample\_011046841, Unigene7197\_Sample\_011046841, Unigene14776\_Sample\_011046841, Unigene17021\_Sample\_011046841, Unigene40544\_Sample\_011046841, Unigene11314\_Sample\_011046841, Unigene41694\_Sample\_011046841, Unigene31544\_Sample\_011046841, Unigene3946\_Sample\_011046841, Unigene49525\_Sample\_011046841, Unigene1012\_Sample\_011046841, Unigene19594\_Sample\_011046841, Unigene42105\_Sample\_011046841, Unigene58882\_Sample\_011046841, Unigene11758\_Sample\_011046841, Unigene21297\_Sample\_011046841, Unigene37492\_Sample\_011046841, Unigene58321\_Sample\_011046841, Unigene12837\_Sample\_011046841, Unigene39083\_Sample\_011046841, Unigene12324\_Sample\_011046841, Unigene31723\_Sample\_011046841, Unigene11706\_Sample\_011046841, Unigene3797\_Sample\_011046841, Unigene58669\_Sample\_011046841, Unigene1803\_Sample\_011046841, Unigene50322\_Sample\_011046841, Unigene19677\_Sample\_011046841, Unigene56096\_Sample\_011046841, Unigene55247\_Sample\_011046841, Unigene52526\_Sample\_011046841, Unigene14511\_Sample\_011046841, Unigene30285\_Sample\_011046841, Unigene47560\_Sample\_011046841, Unigene48730\_Sample\_011046841, Unigene52384\_Sample\_011046841, Unigene5953\_Sample\_011046841, Unigene54870\_Sample\_011046841, Unigene60251\_Sample\_011046841, Unigene13131\_Sample\_011046841, Unigene46783\_Sample\_011046841, Unigene11520\_Sample\_011046841, Unigene44354\_Sample\_011046841, Unigene21539\_Sample\_011046841, Unigene50758\_Sample\_011046841, Unigene26728\_Sample\_011046841, Unigene53063\_Sample\_011046841, Unigene5744\_Sample\_011046841, Unigene60458\_Sample\_011046841, Unigene60441\_Sample\_011046841, Unigene47555\_Sample\_011046841, Unigene27550\_Sample\_011046841, Unigene37642\_Sample\_011046841, Unigene55564\_Sample\_011046841, Unigene49204\_Sample\_011046841, Unigene29587\_Sample\_011046841, Unigene42749\_Sample\_011046841, Unigene23757\_Sample\_011046841, Unigene11667\_Sample\_011046841, Unigene55866\_Sample\_011046841, Unigene19215\_Sample\_011046841, Unigene11676\_Sample\_011046841, Unigene8886\_Sample\_011046841, Unigene39020\_Sample\_011046841, Unigene60217\_Sample\_011046841, Unigene29219\_Sample\_011046841, Unigene57766\_Sample\_011046841, Unigene9082\_Sample\_011046841, Unigene50470\_Sample\_011046841, Unigene50293\_Sample\_011046841, Unigene42561\_Sample\_011046841, Unigene55621\_Sample\_011046841, Unigene49252\_Sample\_011046841, Unigene29124\_Sample\_011046841, Unigene57972\_Sample\_011046841, Unigene58864\_Sample\_011046841, Unigene47923\_Sample\_011046841, Unigene54668\_Sample\_011046841, Unigene31969\_Sample\_011046841, Unigene56851\_Sample\_011046841, Unigene33510\_Sample\_011046841, Unigene6720\_Sample\_011046841, Unigene39621\_Sample\_011046841, Unigene52002\_Sample\_011046841, Unigene51777\_Sample\_011046841, Unigene33573\_Sample\_011046841, Unigene60497\_Sample\_011046841, Unigene43216\_Sample\_011046841, Unigene52041\_Sample\_011046841, Unigene55162\_Sample\_011046841, Unigene30971\_Sample\_011046841, Unigene48482\_Sample\_011046841, Unigene57699\_Sample\_011046841, Unigene23971\_Sample\_011046841, Unigene53995\_Sample\_011046841, Unigene12538\_Sample\_011046841, Unigene59421\_Sample\_011046841, Unigene54240\_Sample\_011046841, Unigene51706\_Sample\_011046841, Unigene12799\_Sample\_011046841, Unigene54683\_Sample\_011046841, Unigene31181\_Sample\_011046841, Unigene9546\_Sample\_011046841, Unigene54701\_Sample\_011046841, Unigene2942\_Sample\_011046841, Unigene41318\_Sample\_011046841, Unigene32119\_Sample\_011046841, Unigene56697\_Sample\_011046841, Unigene51481\_Sample\_011046841, Unigene43377\_Sample\_011046841, Unigene40435\_Sample\_011046841, Unigene10683\_Sample\_011046841, Unigene36154\_Sample\_011046841, Unigene13672\_Sample\_011046841, Unigene4209\_Sample\_011046841, Unigene55610\_Sample\_011046841, Unigene13068\_Sample\_011046841, Unigene55325\_Sample\_011046841, Unigene7879\_Sample\_011046841, Unigene5336\_Sample\_011046841, Unigene12261\_Sample\_011046841, Unigene26327\_Sample\_011046841, Unigene40474\_Sample\_011046841, Unigene59593\_Sample\_011046841, Unigene60190\_Sample\_011046841, Unigene60525\_Sample\_011046841, Unigene37753\_Sample\_011046841, Unigene57641\_Sample\_011046841, Unigene10198\_Sample\_011046841, Unigene4361\_Sample\_011046841, Unigene38329\_Sample\_011046841, Unigene8798\_Sample\_011046841, Unigene24424\_Sample\_011046841, Unigene608\_Sample\_011046841, Unigene58134\_Sample\_011046841, Unigene47924\_Sample\_011046841, Unigene50170\_Sample\_011046841, Unigene52918\_Sample\_011046841, Unigene19453\_Sample\_011046841, Unigene27253\_Sample\_011046841, Unigene45203\_Sample\_011046841, Unigene35914\_Sample\_011046841, Unigene60557\_Sample\_011046841, Unigene43504\_Sample\_011046841, Unigene54656\_Sample\_011046841, Unigene59949\_Sample\_011046841, Unigene58128\_Sample\_011046841, Unigene39675\_Sample\_011046841, Unigene53942\_Sample\_011046841, Unigene36772\_Sample\_011046841, Unigene25547\_Sample\_011046841, Unigene47292\_Sample\_011046841, Unigene5329\_Sample\_011046841, Unigene51537\_Sample\_011046841, Unigene52610\_Sample\_011046841, Unigene5297\_Sample\_011046841, Unigene60576\_Sample\_011046841, Unigene56291\_Sample\_011046841, Unigene54841\_Sample\_011046841, Unigene57832\_Sample\_011046841, Unigene45777\_Sample\_011046841, Unigene1158\_Sample\_011046841, Unigene48881\_Sample\_011046841, Unigene28584\_Sample\_011046841, Unigene7885\_Sample\_011046841, Unigene6535\_Sample\_011046841, Unigene9879\_Sample\_011046841, Unigene12224\_Sample\_011046841, Unigene33820\_Sample\_011046841, Unigene41032\_Sample\_011046841, Unigene59448\_Sample\_011046841, Unigene19352\_Sample\_011046841, Unigene19671\_Sample\_011046841, Unigene301\_Sample\_011046841, Unigene13332\_Sample\_011046841, Unigene43813\_Sample\_011046841, Unigene4226\_Sample\_011046841, Unigene47887\_Sample\_011046841, Unigene39581\_Sample\_011046841, Unigene47872\_Sample\_011046841, Unigene58058\_Sample\_011046841, Unigene51573\_Sample\_011046841, Unigene37549\_Sample\_011046841, Unigene60248\_Sample\_011046841, Unigene54379\_Sample\_011046841, Unigene56798\_Sample\_011046841, Unigene16539\_Sample\_011046841, Unigene56182\_Sample\_011046841, Unigene10522\_Sample\_011046841, Unigene13722\_Sample\_011046841, Unigene27952\_Sample\_011046841, Unigene23133\_Sample\_011046841, Unigene59664\_Sample\_011046841, Unigene26192\_Sample\_011046841, Unigene57456\_Sample\_011046841, Unigene18027\_Sample\_011046841, Unigene34859\_Sample\_011046841, Unigene54368\_Sample\_011046841, Unigene42399\_Sample\_011046841, Unigene51301\_Sample\_011046841, Unigene59727\_Sample\_011046841, Unigene7922\_Sample\_011046841, Unigene58516\_Sample\_011046841, Unigene52838\_Sample\_011046841, Unigene42686\_Sample\_011046841, Unigene11735\_Sample\_011046841, Unigene11547\_Sample\_011046841, Unigene31394\_Sample\_011046841, Unigene30190\_Sample\_011046841, Unigene21362\_Sample\_011046841, Unigene9128\_Sample\_011046841, Unigene9803\_Sample\_011046841, Unigene19399\_Sample\_011046841, Unigene49909\_Sample\_011046841, Unigene16977\_Sample\_011046841, Unigene44090\_Sample\_011046841, Unigene38549\_Sample\_011046841, Unigene59844\_Sample\_011046841, Unigene12510\_Sample\_011046841, Unigene60349\_Sample\_011046841, Unigene49055\_Sample\_011046841, Unigene28563\_Sample\_011046841, Unigene51916\_Sample\_011046841, Unigene7335\_Sample\_011046841, Unigene60\_Sample\_011046841, Unigene55423\_Sample\_011046841, Unigene13901\_Sample\_011046841, Unigene17574\_Sample\_011046841, Unigene60507\_Sample\_011046841, Unigene55644\_Sample\_011046841, Unigene53719\_Sample\_011046841, Unigene52071\_Sample\_011046841, Unigene3810\_Sample\_011046841, Unigene48927\_Sample\_011046841, Unigene54626\_Sample\_011046841, Unigene55404\_Sample\_011046841, Unigene12811\_Sample\_011046841, Unigene41464\_Sample\_011046841, Unigene52731\_Sample\_011046841, Unigene22189\_Sample\_011046841, Unigene59559\_Sample\_011046841, Unigene44600\_Sample\_011046841, Unigene20667\_Sample\_011046841, Unigene48342\_Sample\_011046841, Unigene37057\_Sample\_011046841, Unigene45949\_Sample\_011046841, Unigene13197\_Sample\_011046841, Unigene22611\_Sample\_011046841, Unigene18791\_Sample\_011046841, Unigene53073\_Sample\_011046841, Unigene11419\_Sample\_011046841, Unigene9776\_Sample\_011046841, Unigene26757\_Sample\_011046841, Unigene59228\_Sample\_011046841, Unigene48530\_Sample\_011046841, Unigene51307\_Sample\_011046841, Unigene46142\_Sample\_011046841, Unigene9811\_Sample\_011046841, Unigene58021\_Sample\_011046841, Unigene44103\_Sample\_011046841, Unigene58038\_Sample\_011046841, Unigene51739\_Sample\_011046841, Unigene59221\_Sample\_011046841, Unigene57130\_Sample\_011046841, Unigene46176\_Sample\_011046841, Unigene59987\_Sample\_011046841, Unigene18638\_Sample\_011046841, Unigene32760\_Sample\_011046841, Unigene60721\_Sample\_011046841, Unigene60042\_Sample\_011046841, Unigene54549\_Sample\_011046841, Unigene32964\_Sample\_011046841, Unigene37367\_Sample\_011046841, Unigene42633\_Sample\_011046841, Unigene52413\_Sample\_011046841, Unigene12975\_Sample\_011046841, Unigene48267\_Sample\_011046841, Unigene1793\_Sample\_011046841, Unigene54187\_Sample\_011046841, Unigene56952\_Sample\_011046841, Unigene41680\_Sample\_011046841, Unigene52842\_Sample\_011046841, Unigene54707\_Sample\_011046841, Unigene11998\_Sample\_011046841, Unigene58523\_Sample\_011046841, Unigene11526\_Sample\_011046841, Unigene60540\_Sample\_011046841, Unigene29318\_Sample\_011046841, Unigene45662\_Sample\_011046841, Unigene50039\_Sample\_011046841, Unigene32914\_Sample\_011046841, Unigene12720\_Sample\_011046841, Unigene52094\_Sample\_011046841, Unigene16067\_Sample\_011046841, Unigene38893\_Sample\_011046841, Unigene50108\_Sample\_011046841, Unigene10229\_Sample\_011046841, Unigene47025\_Sample\_011046841, Unigene39219\_Sample\_011046841, Unigene7481\_Sample\_011046841, Unigene50057\_Sample\_011046841, Unigene2032\_Sample\_011046841, Unigene54574\_Sample\_011046841, Unigene36941\_Sample\_011046841, Unigene59705\_Sample\_011046841, Unigene15705\_Sample\_011046841, Unigene59226\_Sample\_011046841, Unigene58675\_Sample\_011046841, Unigene13920\_Sample\_011046841, Unigene21670\_Sample\_011046841, Unigene48257\_Sample\_011046841, Unigene59609\_Sample\_011046841, Unigene34330\_Sample\_011046841, Unigene59758\_Sample\_011046841, Unigene5657\_Sample\_011046841, Unigene7964\_Sample\_011046841, Unigene41135\_Sample\_011046841, Unigene3842\_Sample\_011046841, Unigene30309\_Sample\_011046841, Unigene30484\_Sample\_011046841, Unigene56439\_Sample\_011046841, Unigene47187\_Sample\_011046841, Unigene28789\_Sample\_011046841, Unigene57225\_Sample\_011046841, Unigene11726\_Sample\_011046841, Unigene24615\_Sample\_011046841, Unigene55646\_Sample\_011046841, Unigene3750\_Sample\_011046841, Unigene40166\_Sample\_011046841, Unigene55517\_Sample\_011046841, Unigene6531\_Sample\_011046841, Unigene58796\_Sample\_011046841, Unigene58285\_Sample\_011046841, Unigene5051\_Sample\_011046841, Unigene56191\_Sample\_011046841, Unigene34824\_Sample\_011046841, Unigene10024\_Sample\_011046841, Unigene19343\_Sample\_011046841, Unigene20191\_Sample\_011046841, Unigene6153\_Sample\_011046841, Unigene55720\_Sample\_011046841, Unigene57735\_Sample\_011046841, Unigene7029\_Sample\_011046841, Unigene43936\_Sample\_011046841, Unigene12652\_Sample\_011046841, Unigene55461\_Sample\_011046841, Unigene28106\_Sample\_011046841, Unigene20201\_Sample\_011046841, Unigene13516\_Sample\_011046841, Unigene2933\_Sample\_011046841, Unigene58309\_Sample\_011046841, Unigene48067\_Sample\_011046841, Unigene30684\_Sample\_011046841, Unigene59459\_Sample\_011046841, Unigene2116\_Sample\_011046841, Unigene48334\_Sample\_011046841, Unigene52959\_Sample\_011046841, Unigene51756\_Sample\_011046841, Unigene23218\_Sample\_011046841, Unigene18170\_Sample\_011046841, Unigene37707\_Sample\_011046841, Unigene55302\_Sample\_011046841, Unigene21879\_Sample\_011046841, Unigene41604\_Sample\_011046841, Unigene35470\_Sample\_011046841, Unigene12972\_Sample\_011046841, Unigene18796\_Sample\_011046841, Unigene47379\_Sample\_011046841, Unigene58821\_Sample\_011046841, Unigene59109\_Sample\_011046841, Unigene52385\_Sample\_011046841, Unigene41682\_Sample\_011046841, Unigene25989\_Sample\_011046841, Unigene38223\_Sample\_011046841, Unigene50185\_Sample\_011046841, Unigene36178\_Sample\_011046841, Unigene32594\_Sample\_011046841, Unigene58254\_Sample\_011046841, Unigene43515\_Sample\_011046841, Unigene29314\_Sample\_011046841, Unigene9017\_Sample\_011046841, Unigene51286\_Sample\_011046841, Unigene16836\_Sample\_011046841, Unigene10327\_Sample\_011046841, Unigene58340\_Sample\_011046841, Unigene57602\_Sample\_011046841, Unigene35217\_Sample\_011046841, Unigene58042\_Sample\_011046841, Unigene9600\_Sample\_011046841, Unigene60220\_Sample\_011046841, Unigene51011\_Sample\_011046841, Unigene56391\_Sample\_011046841, Unigene40215\_Sample\_011046841, Unigene6590\_Sample\_011046841, Unigene47910\_Sample\_011046841, Unigene10980\_Sample\_011046841, Unigene60847\_Sample\_011046841, Unigene12824\_Sample\_011046841, Unigene47387\_Sample\_011046841, Unigene60733\_Sample\_011046841, Unigene58152\_Sample\_011046841, Unigene29803\_Sample\_011046841, Unigene46613\_Sample\_011046841, Unigene52695\_Sample\_011046841, Unigene57887\_Sample\_011046841, Unigene16051\_Sample\_011046841, Unigene24332\_Sample\_011046841, Unigene56062\_Sample\_011046841, Unigene57665\_Sample\_011046841, Unigene8000\_Sample\_011046841, Unigene48528\_Sample\_011046841, Unigene5382\_Sample\_011046841, Unigene32534\_Sample\_011046841, Unigene12951\_Sample\_011046841, Unigene21868\_Sample\_011046841, Unigene4432\_Sample\_011046841, Unigene5967\_Sample\_011046841, Unigene46198\_Sample\_011046841, Unigene39412\_Sample\_011046841, Unigene3199\_Sample\_011046841, Unigene60319\_Sample\_011046841, Unigene29813\_Sample\_011046841, Unigene58911\_Sample\_011046841, Unigene34757\_Sample\_011046841, Unigene31129\_Sample\_011046841, Unigene56567\_Sample\_011046841, Unigene45885\_Sample\_011046841, Unigene59711\_Sample\_011046841, Unigene47811\_Sample\_011046841, Unigene13367\_Sample\_011046841, Unigene35586\_Sample\_011046841, Unigene9774\_Sample\_011046841, Unigene55779\_Sample\_011046841, Unigene58415\_Sample\_011046841, Unigene11504\_Sample\_011046841, Unigene57194\_Sample\_011046841, Unigene18715\_Sample\_011046841, Unigene30456\_Sample\_011046841, Unigene15343\_Sample\_011046841, Unigene45721\_Sample\_011046841, Unigene58180\_Sample\_011046841, Unigene55486\_Sample\_011046841, Unigene32509\_Sample\_011046841, Unigene43935\_Sample\_011046841, Unigene26928\_Sample\_011046841, Unigene41284\_Sample\_011046841, Unigene56843\_Sample\_011046841, Unigene39709\_Sample\_011046841, Unigene18856\_Sample\_011046841, Unigene60096\_Sample\_011046841, Unigene60181\_Sample\_011046841, Unigene40491\_Sample\_011046841, Unigene51736\_Sample\_011046841, Unigene6435\_Sample\_011046841, Unigene59596\_Sample\_011046841, Unigene47686\_Sample\_011046841, Unigene54599\_Sample\_011046841, Unigene38011\_Sample\_011046841, Unigene43792\_Sample\_011046841, Unigene43367\_Sample\_011046841, Unigene44683\_Sample\_011046841, Unigene60126\_Sample\_011046841, Unigene9210\_Sample\_011046841, Unigene57305\_Sample\_011046841, Unigene35468\_Sample\_011046841, Unigene56828\_Sample\_011046841, Unigene20762\_Sample\_011046841, Unigene6197\_Sample\_011046841, Unigene53723\_Sample\_011046841, Unigene45824\_Sample\_011046841, Unigene57255\_Sample\_011046841, Unigene7072\_Sample\_011046841, Unigene17671\_Sample\_011046841, Unigene51429\_Sample\_011046841, Unigene58511\_Sample\_011046841, Unigene16046\_Sample\_011046841, Unigene38527\_Sample\_011046841, Unigene59868\_Sample\_011046841, Unigene48647\_Sample\_011046841, Unigene5468\_Sample\_011046841, Unigene59634\_Sample\_011046841, Unigene40350\_Sample\_011046841, Unigene49635\_Sample\_011046841, Unigene60859\_Sample\_011046841, Unigene2110\_Sample\_011046841, Unigene46224\_Sample\_011046841 |
[truncated: 1,294,146 more chars]
