# Supplementary material for: Identification of neglected cestode Taenia multiceps microRNAs by illumina sequencing and bioinformatic analysis
Source: BMC Vet Res. 2013 Aug 13;9:162. doi: 10.1186/1746-6148-9-162 (PMC3849562; doi:10.1186/1746-6148-9-162)
Supplement: Additional file 9 — Biological process GO annotations for candidate target unigenes of novel Taenia multiceps miRNAs. 6,178 target unigenes were assigned to 2,262 GO-terms from “Biological process” ontology. [file 1746-6148-9-162-S9.html]

Terms for Taenia\_multiceps\_P


## Terms for Taenia\_multiceps\_P

---


### Result Table

|  |
| --- |
| **Terms from the Process Ontology with p-value as good or better than 1** |

| Gene Ontology term | Cluster frequency | Genome frequency of use | Corrected P-value |
| --- | --- | --- | --- |
| anatomical structure development | 1164 out of 6178 genes, 18.8% | 1169 out of 6259 genes, 18.7% | 1 |
| cellular process | 4592 out of 6178 genes, 74.3% | 4640 out of 6259 genes, 74.1% | 1 |
| multicellular organismal process | 1603 out of 6178 genes, 25.9% | 1613 out of 6259 genes, 25.8% | 1 |
| system development | 860 out of 6178 genes, 13.9% | 863 out of 6259 genes, 13.8% | 1 |
| cellular developmental process | 590 out of 6178 genes, 9.6% | 591 out of 6259 genes, 9.4% | 1 |
| multicellular organismal development | 1266 out of 6178 genes, 20.5% | 1273 out of 6259 genes, 20.3% | 1 |
| developmental process | 1508 out of 6178 genes, 24.4% | 1518 out of 6259 genes, 24.3% | 1 |
| cell differentiation | 518 out of 6178 genes, 8.4% | 519 out of 6259 genes, 8.3% | 1 |
| cell development | 335 out of 6178 genes, 5.4% | 335 out of 6259 genes, 5.4% | 1 |
| anatomical structure morphogenesis | 638 out of 6178 genes, 10.3% | 641 out of 6259 genes, 10.2% | 1 |
| protein modification process | 718 out of 6178 genes, 11.6% | 722 out of 6259 genes, 11.5% | 1 |
| nervous system development | 386 out of 6178 genes, 6.2% | 387 out of 6259 genes, 6.2% | 1 |
| regulation of RNA metabolic process | 238 out of 6178 genes, 3.9% | 238 out of 6259 genes, 3.8% | 1 |
| organ development | 582 out of 6178 genes, 9.4% | 585 out of 6259 genes, 9.3% | 1 |
| tissue development | 229 out of 6178 genes, 3.7% | 229 out of 6259 genes, 3.7% | 1 |
| regulation of transcription, DNA-dependent | 228 out of 6178 genes, 3.7% | 228 out of 6259 genes, 3.6% | 1 |
| cellular component morphogenesis | 226 out of 6178 genes, 3.7% | 226 out of 6259 genes, 3.6% | 1 |
| cellular component organization or biogenesis at cellular level | 847 out of 6178 genes, 13.7% | 853 out of 6259 genes, 13.6% | 1 |
| regulation of macromolecule biosynthetic process | 325 out of 6178 genes, 5.3% | 326 out of 6259 genes, 5.2% | 1 |
| biological regulation | 1866 out of 6178 genes, 30.2% | 1884 out of 6259 genes, 30.1% | 1 |
| cellular protein metabolic process | 1091 out of 6178 genes, 17.7% | 1100 out of 6259 genes, 17.6% | 1 |
| cellular macromolecule metabolic process | 2010 out of 6178 genes, 32.5% | 2030 out of 6259 genes, 32.4% | 1 |
| system process | 308 out of 6178 genes, 5.0% | 309 out of 6259 genes, 4.9% | 1 |
| cellular component organization at cellular level | 795 out of 6178 genes, 12.9% | 801 out of 6259 genes, 12.8% | 1 |
| cell morphogenesis | 179 out of 6178 genes, 2.9% | 179 out of 6259 genes, 2.9% | 1 |
| neuron development | 179 out of 6178 genes, 2.9% | 179 out of 6259 genes, 2.9% | 1 |
| protein metabolic process | 1232 out of 6178 genes, 19.9% | 1243 out of 6259 genes, 19.9% | 1 |
| M phase | 176 out of 6178 genes, 2.8% | 176 out of 6259 genes, 2.8% | 1 |
| response to organic substance | 173 out of 6178 genes, 2.8% | 173 out of 6259 genes, 2.8% | 1 |
| regulation of cellular macromolecule biosynthetic process | 290 out of 6178 genes, 4.7% | 291 out of 6259 genes, 4.6% | 1 |
| regulation of cellular process | 1130 out of 6178 genes, 18.3% | 1140 out of 6259 genes, 18.2% | 1 |
| regulation of biological process | 1633 out of 6178 genes, 26.4% | 1649 out of 6259 genes, 26.3% | 1 |
| signaling | 863 out of 6178 genes, 14.0% | 870 out of 6259 genes, 13.9% | 1 |
| macromolecule modification | 771 out of 6178 genes, 12.5% | 777 out of 6259 genes, 12.4% | 1 |
| macromolecule localization | 385 out of 6178 genes, 6.2% | 387 out of 6259 genes, 6.2% | 1 |
| regulation of transcription | 270 out of 6178 genes, 4.4% | 271 out of 6259 genes, 4.3% | 1 |
| cellular component organization or biogenesis | 1259 out of 6178 genes, 20.4% | 1271 out of 6259 genes, 20.3% | 1 |
| signaling pathway | 558 out of 6178 genes, 9.0% | 562 out of 6259 genes, 9.0% | 1 |
| cellular catabolic process | 259 out of 6178 genes, 4.2% | 260 out of 6259 genes, 4.2% | 1 |
| cellular component biogenesis | 360 out of 6178 genes, 5.8% | 362 out of 6259 genes, 5.8% | 1 |
| reproduction | 355 out of 6178 genes, 5.7% | 357 out of 6259 genes, 5.7% | 1 |
| neurogenesis | 253 out of 6178 genes, 4.1% | 254 out of 6259 genes, 4.1% | 1 |
| catabolic process | 348 out of 6178 genes, 5.6% | 350 out of 6259 genes, 5.6% | 1 |
| negative regulation of cellular process | 246 out of 6178 genes, 4.0% | 247 out of 6259 genes, 3.9% | 1 |
| regulation of transcription from RNA polymerase II promoter | 136 out of 6178 genes, 2.2% | 136 out of 6259 genes, 2.2% | 1 |
| neurological system process | 244 out of 6178 genes, 3.9% | 245 out of 6259 genes, 3.9% | 1 |
| cell morphogenesis involved in differentiation | 135 out of 6178 genes, 2.2% | 135 out of 6259 genes, 2.2% | 1 |
| response to DNA damage stimulus | 135 out of 6178 genes, 2.2% | 135 out of 6259 genes, 2.2% | 1 |
| reproductive process | 342 out of 6178 genes, 5.5% | 344 out of 6259 genes, 5.5% | 1 |
| embryonic morphogenesis | 134 out of 6178 genes, 2.2% | 134 out of 6259 genes, 2.1% | 1 |
| growth | 133 out of 6178 genes, 2.2% | 133 out of 6259 genes, 2.1% | 1 |
| organelle organization | 612 out of 6178 genes, 9.9% | 617 out of 6259 genes, 9.9% | 1 |
| cellular component organization | 1208 out of 6178 genes, 19.6% | 1220 out of 6259 genes, 19.5% | 1 |
| generation of neurons | 237 out of 6178 genes, 3.8% | 238 out of 6259 genes, 3.8% | 1 |
| cell part morphogenesis | 128 out of 6178 genes, 2.1% | 128 out of 6259 genes, 2.0% | 1 |
| cell projection morphogenesis | 128 out of 6178 genes, 2.1% | 128 out of 6259 genes, 2.0% | 1 |
| cell cycle phase | 229 out of 6178 genes, 3.7% | 230 out of 6259 genes, 3.7% | 1 |
| small molecule catabolic process | 124 out of 6178 genes, 2.0% | 124 out of 6259 genes, 2.0% | 1 |
| cell cycle process | 322 out of 6178 genes, 5.2% | 324 out of 6259 genes, 5.2% | 1 |
| neuron projection development | 121 out of 6178 genes, 2.0% | 121 out of 6259 genes, 1.9% | 1 |
| regulation of cellular component organization | 121 out of 6178 genes, 2.0% | 121 out of 6259 genes, 1.9% | 1 |
| embryo development ending in birth or egg hatching | 119 out of 6178 genes, 1.9% | 119 out of 6259 genes, 1.9% | 1 |
| intracellular transport | 218 out of 6178 genes, 3.5% | 219 out of 6259 genes, 3.5% | 1 |
| response to abiotic stimulus | 115 out of 6178 genes, 1.9% | 115 out of 6259 genes, 1.8% | 1 |
| regulation of cell differentiation | 115 out of 6178 genes, 1.9% | 115 out of 6259 genes, 1.8% | 1 |
| tissue morphogenesis | 115 out of 6178 genes, 1.9% | 115 out of 6259 genes, 1.8% | 1 |
| cell cycle | 401 out of 6178 genes, 6.5% | 404 out of 6259 genes, 6.5% | 1 |
| cellular component assembly | 309 out of 6178 genes, 5.0% | 311 out of 6259 genes, 5.0% | 1 |
| mitotic cell cycle | 214 out of 6178 genes, 3.5% | 215 out of 6259 genes, 3.4% | 1 |
| central nervous system development | 112 out of 6178 genes, 1.8% | 112 out of 6259 genes, 1.8% | 1 |
| neuron projection morphogenesis | 111 out of 6178 genes, 1.8% | 111 out of 6259 genes, 1.8% | 1 |
| cell surface receptor linked signaling pathway | 305 out of 6178 genes, 4.9% | 307 out of 6259 genes, 4.9% | 1 |
| establishment of localization in cell | 304 out of 6178 genes, 4.9% | 306 out of 6259 genes, 4.9% | 1 |
| DNA repair | 107 out of 6178 genes, 1.7% | 107 out of 6259 genes, 1.7% | 1 |
| cell morphogenesis involved in neuron differentiation | 107 out of 6178 genes, 1.7% | 107 out of 6259 genes, 1.7% | 1 |
| neuron differentiation | 205 out of 6178 genes, 3.3% | 206 out of 6259 genes, 3.3% | 1 |
| M phase of mitotic cell cycle | 105 out of 6178 genes, 1.7% | 105 out of 6259 genes, 1.7% | 1 |
| cellular macromolecule localization | 105 out of 6178 genes, 1.7% | 105 out of 6259 genes, 1.7% | 1 |
| establishment of protein localization | 203 out of 6178 genes, 3.3% | 204 out of 6259 genes, 3.3% | 1 |
| nucleic acid metabolic process | 972 out of 6178 genes, 15.7% | 982 out of 6259 genes, 15.7% | 1 |
| ncRNA metabolic process | 104 out of 6178 genes, 1.7% | 104 out of 6259 genes, 1.7% | 1 |
| protein transport | 200 out of 6178 genes, 3.2% | 201 out of 6259 genes, 3.2% | 1 |
| regionalization | 102 out of 6178 genes, 1.7% | 102 out of 6259 genes, 1.6% | 1 |
| regulation of cell cycle | 102 out of 6178 genes, 1.7% | 102 out of 6259 genes, 1.6% | 1 |
| cellular protein localization | 100 out of 6178 genes, 1.6% | 100 out of 6259 genes, 1.6% | 1 |
| primary metabolic process | 2763 out of 6178 genes, 44.7% | 2796 out of 6259 genes, 44.7% | 1 |
| developmental process involved in reproduction | 194 out of 6178 genes, 3.1% | 195 out of 6259 genes, 3.1% | 1 |
| microtubule cytoskeleton organization | 96 out of 6178 genes, 1.6% | 96 out of 6259 genes, 1.5% | 1 |
| cellular ketone metabolic process | 189 out of 6178 genes, 3.1% | 190 out of 6259 genes, 3.0% | 1 |
| embryo development | 450 out of 6178 genes, 7.3% | 454 out of 6259 genes, 7.3% | 1 |
| chromatin modification | 94 out of 6178 genes, 1.5% | 94 out of 6259 genes, 1.5% | 1 |
| regulation of biological quality | 449 out of 6178 genes, 7.3% | 453 out of 6259 genes, 7.2% | 1 |
| response to endogenous stimulus | 92 out of 6178 genes, 1.5% | 92 out of 6259 genes, 1.5% | 1 |
| cellular metabolic process | 2811 out of 6178 genes, 45.5% | 2845 out of 6259 genes, 45.5% | 1 |
| response to stimulus | 1016 out of 6178 genes, 16.4% | 1027 out of 6259 genes, 16.4% | 1 |
| organic acid metabolic process | 184 out of 6178 genes, 3.0% | 185 out of 6259 genes, 3.0% | 1 |
| response to chemical stimulus | 442 out of 6178 genes, 7.2% | 446 out of 6259 genes, 7.1% | 1 |
| oxoacid metabolic process | 183 out of 6178 genes, 3.0% | 184 out of 6259 genes, 2.9% | 1 |
| transcription | 271 out of 6178 genes, 4.4% | 273 out of 6259 genes, 4.4% | 1 |
| sex differentiation | 89 out of 6178 genes, 1.4% | 89 out of 6259 genes, 1.4% | 1 |
| carboxylic acid metabolic process | 181 out of 6178 genes, 2.9% | 182 out of 6259 genes, 2.9% | 1 |
| macromolecule biosynthetic process | 602 out of 6178 genes, 9.7% | 608 out of 6259 genes, 9.7% | 1 |
| cellular localization | 351 out of 6178 genes, 5.7% | 354 out of 6259 genes, 5.7% | 1 |
| epithelium development | 86 out of 6178 genes, 1.4% | 86 out of 6259 genes, 1.4% | 1 |
| homeostatic process | 176 out of 6178 genes, 2.8% | 177 out of 6259 genes, 2.8% | 1 |
| multi-organism process | 85 out of 6178 genes, 1.4% | 85 out of 6259 genes, 1.4% | 1 |
| cellular macromolecule biosynthetic process | 594 out of 6178 genes, 9.6% | 600 out of 6259 genes, 9.6% | 1 |
| protein localization | 261 out of 6178 genes, 4.2% | 263 out of 6259 genes, 4.2% | 1 |
| tRNA metabolic process | 84 out of 6178 genes, 1.4% | 84 out of 6259 genes, 1.3% | 1 |
| multicellular organismal aging | 84 out of 6178 genes, 1.4% | 84 out of 6259 genes, 1.3% | 1 |
| regulation of biosynthetic process | 344 out of 6178 genes, 5.6% | 347 out of 6259 genes, 5.5% | 1 |
| nucleobase, nucleoside, nucleotide and nucleic acid metabolic process | 1228 out of 6178 genes, 19.9% | 1242 out of 6259 genes, 19.8% | 1 |
| cellular component movement | 172 out of 6178 genes, 2.8% | 173 out of 6259 genes, 2.8% | 1 |
| RNA metabolic process | 588 out of 6178 genes, 9.5% | 594 out of 6259 genes, 9.5% | 1 |
| positive regulation of macromolecule metabolic process | 82 out of 6178 genes, 1.3% | 82 out of 6259 genes, 1.3% | 1 |
| cell projection organization | 170 out of 6178 genes, 2.8% | 171 out of 6259 genes, 2.7% | 1 |
| regulation of anatomical structure size | 81 out of 6178 genes, 1.3% | 81 out of 6259 genes, 1.3% | 1 |
| intracellular protein transport | 80 out of 6178 genes, 1.3% | 80 out of 6259 genes, 1.3% | 1 |
| anatomical structure formation involved in morphogenesis | 167 out of 6178 genes, 2.7% | 168 out of 6259 genes, 2.7% | 1 |
| cell motility | 167 out of 6178 genes, 2.7% | 168 out of 6259 genes, 2.7% | 1 |
| localization of cell | 167 out of 6178 genes, 2.7% | 168 out of 6259 genes, 2.7% | 1 |
| cell-cell signaling | 165 out of 6178 genes, 2.7% | 166 out of 6259 genes, 2.7% | 1 |
| organ morphogenesis | 165 out of 6178 genes, 2.7% | 166 out of 6259 genes, 2.7% | 1 |
| response to radiation | 75 out of 6178 genes, 1.2% | 75 out of 6259 genes, 1.2% | 1 |
| axonogenesis | 73 out of 6178 genes, 1.2% | 73 out of 6259 genes, 1.2% | 1 |
| response to hormone stimulus | 72 out of 6178 genes, 1.2% | 72 out of 6259 genes, 1.2% | 1 |
| imaginal disc development | 71 out of 6178 genes, 1.1% | 71 out of 6259 genes, 1.1% | 1 |
| chordate embryonic development | 71 out of 6178 genes, 1.1% | 71 out of 6259 genes, 1.1% | 1 |
| cellular macromolecule catabolic process | 155 out of 6178 genes, 2.5% | 156 out of 6259 genes, 2.5% | 1 |
| signaling process | 558 out of 6178 genes, 9.0% | 564 out of 6259 genes, 9.0% | 1 |
| post-embryonic organ development | 70 out of 6178 genes, 1.1% | 70 out of 6259 genes, 1.1% | 1 |
| cation homeostasis | 70 out of 6178 genes, 1.1% | 70 out of 6259 genes, 1.1% | 1 |
| signal transmission | 555 out of 6178 genes, 9.0% | 561 out of 6259 genes, 9.0% | 1 |
| cytoskeleton organization | 235 out of 6178 genes, 3.8% | 237 out of 6259 genes, 3.8% | 1 |
| covalent chromatin modification | 69 out of 6178 genes, 1.1% | 69 out of 6259 genes, 1.1% | 1 |
| histone modification | 69 out of 6178 genes, 1.1% | 69 out of 6259 genes, 1.1% | 1 |
| microtubule-based process | 234 out of 6178 genes, 3.8% | 236 out of 6259 genes, 3.8% | 1 |
| regulation of cell development | 67 out of 6178 genes, 1.1% | 67 out of 6259 genes, 1.1% | 1 |
| cellular component biogenesis at cellular level | 67 out of 6178 genes, 1.1% | 67 out of 6259 genes, 1.1% | 1 |
| ion transport | 309 out of 6178 genes, 5.0% | 312 out of 6259 genes, 5.0% | 1 |
| morphogenesis of an epithelium | 66 out of 6178 genes, 1.1% | 66 out of 6259 genes, 1.1% | 1 |
| brain development | 66 out of 6178 genes, 1.1% | 66 out of 6259 genes, 1.1% | 1 |
| ribonucleoprotein complex biogenesis | 66 out of 6178 genes, 1.1% | 66 out of 6259 genes, 1.1% | 1 |
| regulation of cellular biosynthetic process | 308 out of 6178 genes, 5.0% | 311 out of 6259 genes, 5.0% | 1 |
| pattern specification process | 147 out of 6178 genes, 2.4% | 148 out of 6259 genes, 2.4% | 1 |
| regulation of developmental process | 147 out of 6178 genes, 2.4% | 148 out of 6259 genes, 2.4% | 1 |
| reproductive behavior | 65 out of 6178 genes, 1.1% | 65 out of 6259 genes, 1.0% | 1 |
| genitalia development | 65 out of 6178 genes, 1.1% | 65 out of 6259 genes, 1.0% | 1 |
| proteolysis | 146 out of 6178 genes, 2.4% | 147 out of 6259 genes, 2.3% | 1 |
| tube development | 64 out of 6178 genes, 1.0% | 64 out of 6259 genes, 1.0% | 1 |
| sensory perception | 63 out of 6178 genes, 1.0% | 63 out of 6259 genes, 1.0% | 1 |
| regulation of cellular component size | 63 out of 6178 genes, 1.0% | 63 out of 6259 genes, 1.0% | 1 |
| macromolecule metabolic process | 2304 out of 6178 genes, 37.3% | 2333 out of 6259 genes, 37.3% | 1 |
| cellular response to stress | 222 out of 6178 genes, 3.6% | 224 out of 6259 genes, 3.6% | 1 |
| appendage development | 62 out of 6178 genes, 1.0% | 62 out of 6259 genes, 1.0% | 1 |
| macromolecular complex assembly | 221 out of 6178 genes, 3.6% | 223 out of 6259 genes, 3.6% | 1 |
| RNA processing | 298 out of 6178 genes, 4.8% | 301 out of 6259 genes, 4.8% | 1 |
| negative regulation of biological process | 298 out of 6178 genes, 4.8% | 301 out of 6259 genes, 4.8% | 1 |
| cellular nitrogen compound catabolic process | 61 out of 6178 genes, 1.0% | 61 out of 6259 genes, 1.0% | 1 |
| instar larval or pupal development | 60 out of 6178 genes, 1.0% | 60 out of 6259 genes, 1.0% | 1 |
| post-embryonic morphogenesis | 60 out of 6178 genes, 1.0% | 60 out of 6259 genes, 1.0% | 1 |
| nucleobase, nucleoside, nucleotide and nucleic acid catabolic process | 60 out of 6178 genes, 1.0% | 60 out of 6259 genes, 1.0% | 1 |
| nucleobase, nucleoside and nucleotide catabolic process | 60 out of 6178 genes, 1.0% | 60 out of 6259 genes, 1.0% | 1 |
| appendage morphogenesis | 60 out of 6178 genes, 1.0% | 60 out of 6259 genes, 1.0% | 1 |
| negative regulation of programmed cell death | 60 out of 6178 genes, 1.0% | 60 out of 6259 genes, 1.0% | 1 |
| heterocycle catabolic process | 60 out of 6178 genes, 1.0% | 60 out of 6259 genes, 1.0% | 1 |
| Golgi vesicle transport | 60 out of 6178 genes, 1.0% | 60 out of 6259 genes, 1.0% | 1 |
| negative regulation of cell death | 60 out of 6178 genes, 1.0% | 60 out of 6259 genes, 1.0% | 1 |
| carbohydrate metabolic process | 217 out of 6178 genes, 3.5% | 219 out of 6259 genes, 3.5% | 1 |
| carbohydrate catabolic process | 59 out of 6178 genes, 1.0% | 59 out of 6259 genes, 0.9% | 1 |
| amino acid activation | 59 out of 6178 genes, 1.0% | 59 out of 6259 genes, 0.9% | 1 |
| tRNA aminoacylation | 59 out of 6178 genes, 1.0% | 59 out of 6259 genes, 0.9% | 1 |
| negative regulation of apoptosis | 59 out of 6178 genes, 1.0% | 59 out of 6259 genes, 0.9% | 1 |
| tRNA aminoacylation for protein translation | 58 out of 6178 genes, 0.9% | 58 out of 6259 genes, 0.9% | 1 |
| response to metal ion | 58 out of 6178 genes, 0.9% | 58 out of 6259 genes, 0.9% | 1 |
| lipid localization | 57 out of 6178 genes, 0.9% | 57 out of 6259 genes, 0.9% | 1 |
| cellular cation homeostasis | 57 out of 6178 genes, 0.9% | 57 out of 6259 genes, 0.9% | 1 |
| cell fate commitment | 57 out of 6178 genes, 0.9% | 57 out of 6259 genes, 0.9% | 1 |
| regulation of cell death | 134 out of 6178 genes, 2.2% | 135 out of 6259 genes, 2.2% | 1 |
| multicellular organism reproduction | 210 out of 6178 genes, 3.4% | 212 out of 6259 genes, 3.4% | 1 |
| multicellular organismal reproductive process | 210 out of 6178 genes, 3.4% | 212 out of 6259 genes, 3.4% | 1 |
| transmission of nerve impulse | 133 out of 6178 genes, 2.2% | 134 out of 6259 genes, 2.1% | 1 |
| regulation of kinase activity | 56 out of 6178 genes, 0.9% | 56 out of 6259 genes, 0.9% | 1 |
| glycerolipid metabolic process | 56 out of 6178 genes, 0.9% | 56 out of 6259 genes, 0.9% | 1 |
| regulation of transferase activity | 56 out of 6178 genes, 0.9% | 56 out of 6259 genes, 0.9% | 1 |
| regulation of programmed cell death | 132 out of 6178 genes, 2.1% | 133 out of 6259 genes, 2.1% | 1 |
| behavior | 208 out of 6178 genes, 3.4% | 210 out of 6259 genes, 3.4% | 1 |
| metamorphosis | 55 out of 6178 genes, 0.9% | 55 out of 6259 genes, 0.9% | 1 |
| nucleotide catabolic process | 55 out of 6178 genes, 0.9% | 55 out of 6259 genes, 0.9% | 1 |
| peptidyl-amino acid modification | 55 out of 6178 genes, 0.9% | 55 out of 6259 genes, 0.9% | 1 |
| instar larval or pupal morphogenesis | 55 out of 6178 genes, 0.9% | 55 out of 6259 genes, 0.9% | 1 |
| cardiovascular system development | 55 out of 6178 genes, 0.9% | 55 out of 6259 genes, 0.9% | 1 |
| circulatory system development | 55 out of 6178 genes, 0.9% | 55 out of 6259 genes, 0.9% | 1 |
| locomotion | 282 out of 6178 genes, 4.6% | 285 out of 6259 genes, 4.6% | 1 |
| monocarboxylic acid metabolic process | 54 out of 6178 genes, 0.9% | 54 out of 6259 genes, 0.9% | 1 |
| cellular carbohydrate catabolic process | 54 out of 6178 genes, 0.9% | 54 out of 6259 genes, 0.9% | 1 |
| small molecule metabolic process | 660 out of 6178 genes, 10.7% | 668 out of 6259 genes, 10.7% | 1 |
| protein complex subunit organization | 204 out of 6178 genes, 3.3% | 206 out of 6259 genes, 3.3% | 1 |
| chemical homeostasis | 128 out of 6178 genes, 2.1% | 129 out of 6259 genes, 2.1% | 1 |
| monosaccharide metabolic process | 127 out of 6178 genes, 2.1% | 128 out of 6259 genes, 2.0% | 1 |
| cellular component assembly at cellular level | 202 out of 6178 genes, 3.3% | 204 out of 6259 genes, 3.3% | 1 |
| purine nucleotide catabolic process | 52 out of 6178 genes, 0.8% | 52 out of 6259 genes, 0.8% | 1 |
| imaginal disc morphogenesis | 52 out of 6178 genes, 0.8% | 52 out of 6259 genes, 0.8% | 1 |
| post-embryonic organ morphogenesis | 52 out of 6178 genes, 0.8% | 52 out of 6259 genes, 0.8% | 1 |
| cellular process involved in reproduction | 52 out of 6178 genes, 0.8% | 52 out of 6259 genes, 0.8% | 1 |
| regulation of nervous system development | 52 out of 6178 genes, 0.8% | 52 out of 6259 genes, 0.8% | 1 |
| cellular amino acid and derivative metabolic process | 126 out of 6178 genes, 2.0% | 127 out of 6259 genes, 2.0% | 1 |
| cellular homeostasis | 126 out of 6178 genes, 2.0% | 127 out of 6259 genes, 2.0% | 1 |
| lipid metabolic process | 201 out of 6178 genes, 3.3% | 203 out of 6259 genes, 3.2% | 1 |
| cell communication | 200 out of 6178 genes, 3.2% | 202 out of 6259 genes, 3.2% | 1 |
| protein catabolic process | 125 out of 6178 genes, 2.0% | 126 out of 6259 genes, 2.0% | 1 |
| glycoprotein metabolic process | 51 out of 6178 genes, 0.8% | 51 out of 6259 genes, 0.8% | 1 |
| multicellular organismal reproductive behavior | 51 out of 6178 genes, 0.8% | 51 out of 6259 genes, 0.8% | 1 |
| muscle structure development | 51 out of 6178 genes, 0.8% | 51 out of 6259 genes, 0.8% | 1 |
| macromolecular complex subunit organization | 274 out of 6178 genes, 4.4% | 277 out of 6259 genes, 4.4% | 1 |
| protein targeting | 50 out of 6178 genes, 0.8% | 50 out of 6259 genes, 0.8% | 1 |
| positive regulation of macromolecule biosynthetic process | 50 out of 6178 genes, 0.8% | 50 out of 6259 genes, 0.8% | 1 |
| regulation of cell cycle process | 50 out of 6178 genes, 0.8% | 50 out of 6259 genes, 0.8% | 1 |
| oogenesis | 50 out of 6178 genes, 0.8% | 50 out of 6259 genes, 0.8% | 1 |
| phosphate metabolic process | 197 out of 6178 genes, 3.2% | 199 out of 6259 genes, 3.2% | 1 |
| macromolecule catabolic process | 196 out of 6178 genes, 3.2% | 198 out of 6259 genes, 3.2% | 1 |
| multicellular organism growth | 49 out of 6178 genes, 0.8% | 49 out of 6259 genes, 0.8% | 1 |
| male gamete generation | 49 out of 6178 genes, 0.8% | 49 out of 6259 genes, 0.8% | 1 |
| regulation of response to stimulus | 49 out of 6178 genes, 0.8% | 49 out of 6259 genes, 0.8% | 1 |
| cellular protein catabolic process | 121 out of 6178 genes, 2.0% | 122 out of 6259 genes, 1.9% | 1 |
| proteolysis involved in cellular protein catabolic process | 121 out of 6178 genes, 2.0% | 122 out of 6259 genes, 1.9% | 1 |
| regulation of nitrogen compound metabolic process | 343 out of 6178 genes, 5.6% | 347 out of 6259 genes, 5.5% | 1 |
| meiotic cell cycle | 48 out of 6178 genes, 0.8% | 48 out of 6259 genes, 0.8% | 1 |
| regulation of nucleobase, nucleoside, nucleotide and nucleic acid metabolic process | 340 out of 6178 genes, 5.5% | 344 out of 6259 genes, 5.5% | 1 |
| generation of a signal involved in cell-cell signaling | 47 out of 6178 genes, 0.8% | 47 out of 6259 genes, 0.8% | 1 |
| sulfur compound metabolic process | 47 out of 6178 genes, 0.8% | 47 out of 6259 genes, 0.8% | 1 |
| gastrulation | 47 out of 6178 genes, 0.8% | 47 out of 6259 genes, 0.8% | 1 |
| nucleoside triphosphate catabolic process | 47 out of 6178 genes, 0.8% | 47 out of 6259 genes, 0.8% | 1 |
| purine ribonucleotide catabolic process | 47 out of 6178 genes, 0.8% | 47 out of 6259 genes, 0.8% | 1 |
| ribonucleotide catabolic process | 47 out of 6178 genes, 0.8% | 47 out of 6259 genes, 0.8% | 1 |
| alcohol catabolic process | 47 out of 6178 genes, 0.8% | 47 out of 6259 genes, 0.8% | 1 |
| protein oligomerization | 47 out of 6178 genes, 0.8% | 47 out of 6259 genes, 0.8% | 1 |
| organelle localization | 47 out of 6178 genes, 0.8% | 47 out of 6259 genes, 0.8% | 1 |
| hexose metabolic process | 118 out of 6178 genes, 1.9% | 119 out of 6259 genes, 1.9% | 1 |
| intracellular signaling pathway | 264 out of 6178 genes, 4.3% | 267 out of 6259 genes, 4.3% | 1 |
| transcription, DNA-dependent | 190 out of 6178 genes, 3.1% | 192 out of 6259 genes, 3.1% | 1 |
| RNA biosynthetic process | 190 out of 6178 genes, 3.1% | 192 out of 6259 genes, 3.1% | 1 |
| glucose catabolic process | 46 out of 6178 genes, 0.7% | 46 out of 6259 genes, 0.7% | 1 |
| regulation of cell size | 46 out of 6178 genes, 0.7% | 46 out of 6259 genes, 0.7% | 1 |
| purine nucleoside triphosphate catabolic process | 46 out of 6178 genes, 0.7% | 46 out of 6259 genes, 0.7% | 1 |
| ribonucleoside triphosphate catabolic process | 46 out of 6178 genes, 0.7% | 46 out of 6259 genes, 0.7% | 1 |
| purine ribonucleoside triphosphate catabolic process | 46 out of 6178 genes, 0.7% | 46 out of 6259 genes, 0.7% | 1 |
| hexose catabolic process | 46 out of 6178 genes, 0.7% | 46 out of 6259 genes, 0.7% | 1 |
| signal release | 46 out of 6178 genes, 0.7% | 46 out of 6259 genes, 0.7% | 1 |
| monosaccharide catabolic process | 46 out of 6178 genes, 0.7% | 46 out of 6259 genes, 0.7% | 1 |
| modification-dependent protein catabolic process | 115 out of 6178 genes, 1.9% | 116 out of 6259 genes, 1.9% | 1 |
| modification-dependent macromolecule catabolic process | 115 out of 6178 genes, 1.9% | 116 out of 6259 genes, 1.9% | 1 |
| tube morphogenesis | 45 out of 6178 genes, 0.7% | 45 out of 6259 genes, 0.7% | 1 |
| synaptic transmission | 114 out of 6178 genes, 1.8% | 115 out of 6259 genes, 1.8% | 1 |
| cell migration | 114 out of 6178 genes, 1.8% | 115 out of 6259 genes, 1.8% | 1 |
| protein complex assembly | 185 out of 6178 genes, 3.0% | 187 out of 6259 genes, 3.0% | 1 |
| protein complex biogenesis | 185 out of 6178 genes, 3.0% | 187 out of 6259 genes, 3.0% | 1 |
| glycoprotein biosynthetic process | 44 out of 6178 genes, 0.7% | 44 out of 6259 genes, 0.7% | 1 |
| muscle cell differentiation | 44 out of 6178 genes, 0.7% | 44 out of 6259 genes, 0.7% | 1 |
| actin filament-based process | 113 out of 6178 genes, 1.8% | 114 out of 6259 genes, 1.8% | 1 |
| localization | 1594 out of 6178 genes, 25.8% | 1615 out of 6259 genes, 25.8% | 1 |
| wing disc morphogenesis | 43 out of 6178 genes, 0.7% | 43 out of 6259 genes, 0.7% | 1 |
| response to light stimulus | 43 out of 6178 genes, 0.7% | 43 out of 6259 genes, 0.7% | 1 |
| Wnt receptor signaling pathway | 43 out of 6178 genes, 0.7% | 43 out of 6259 genes, 0.7% | 1 |
| wing disc development | 43 out of 6178 genes, 0.7% | 43 out of 6259 genes, 0.7% | 1 |
| regulation of cellular component biogenesis | 43 out of 6178 genes, 0.7% | 43 out of 6259 genes, 0.7% | 1 |
| metal ion homeostasis | 43 out of 6178 genes, 0.7% | 43 out of 6259 genes, 0.7% | 1 |
| mRNA metabolic process | 111 out of 6178 genes, 1.8% | 112 out of 6259 genes, 1.8% | 1 |
| regulation of localization | 111 out of 6178 genes, 1.8% | 112 out of 6259 genes, 1.8% | 1 |
| regulation of apoptosis | 111 out of 6178 genes, 1.8% | 112 out of 6259 genes, 1.8% | 1 |
| cellular response to stimulus | 254 out of 6178 genes, 4.1% | 257 out of 6259 genes, 4.1% | 1 |
| ion homeostasis | 110 out of 6178 genes, 1.8% | 111 out of 6259 genes, 1.8% | 1 |
| transmembrane receptor protein serine/threonine kinase signaling pathway | 42 out of 6178 genes, 0.7% | 42 out of 6259 genes, 0.7% | 1 |
| regulation of membrane potential | 42 out of 6178 genes, 0.7% | 42 out of 6259 genes, 0.7% | 1 |
| response to stress | 397 out of 6178 genes, 6.4% | 402 out of 6259 genes, 6.4% | 1 |
| cellular amino acid metabolic process | 109 out of 6178 genes, 1.8% | 110 out of 6259 genes, 1.8% | 1 |
| regulation of immune system process | 41 out of 6178 genes, 0.7% | 41 out of 6259 genes, 0.7% | 1 |
| muscle system process | 41 out of 6178 genes, 0.7% | 41 out of 6259 genes, 0.7% | 1 |
| cellular metal ion homeostasis | 41 out of 6178 genes, 0.7% | 41 out of 6259 genes, 0.7% | 1 |
| axon guidance | 41 out of 6178 genes, 0.7% | 41 out of 6259 genes, 0.7% | 1 |
| oxidation reduction | 41 out of 6178 genes, 0.7% | 41 out of 6259 genes, 0.7% | 1 |
| regulation of metabolic process | 835 out of 6178 genes, 13.5% | 846 out of 6259 genes, 13.5% | 1 |
| cellular carbohydrate metabolic process | 177 out of 6178 genes, 2.9% | 179 out of 6259 genes, 2.9% | 1 |
| DNA metabolic process | 320 out of 6178 genes, 5.2% | 324 out of 6259 genes, 5.2% | 1 |
| meiosis | 40 out of 6178 genes, 0.6% | 40 out of 6259 genes, 0.6% | 1 |
| learning or memory | 40 out of 6178 genes, 0.6% | 40 out of 6259 genes, 0.6% | 1 |
| nucleoside monophosphate metabolic process | 40 out of 6178 genes, 0.6% | 40 out of 6259 genes, 0.6% | 1 |
| cell growth | 40 out of 6178 genes, 0.6% | 40 out of 6259 genes, 0.6% | 1 |
| negative regulation of cell differentiation | 40 out of 6178 genes, 0.6% | 40 out of 6259 genes, 0.6% | 1 |
| organelle fission | 40 out of 6178 genes, 0.6% | 40 out of 6259 genes, 0.6% | 1 |
| cognition | 40 out of 6178 genes, 0.6% | 40 out of 6259 genes, 0.6% | 1 |
| negative regulation of developmental process | 40 out of 6178 genes, 0.6% | 40 out of 6259 genes, 0.6% | 1 |
| M phase of meiotic cell cycle | 40 out of 6178 genes, 0.6% | 40 out of 6259 genes, 0.6% | 1 |
| embryonic development via the syncytial blastoderm | 39 out of 6178 genes, 0.6% | 39 out of 6259 genes, 0.6% | 1 |
| RNA localization | 39 out of 6178 genes, 0.6% | 39 out of 6259 genes, 0.6% | 1 |
| chromosome segregation | 39 out of 6178 genes, 0.6% | 39 out of 6259 genes, 0.6% | 1 |
| nucleobase, nucleoside, nucleotide and nucleic acid transport | 39 out of 6178 genes, 0.6% | 39 out of 6259 genes, 0.6% | 1 |
| electron transport chain | 39 out of 6178 genes, 0.6% | 39 out of 6259 genes, 0.6% | 1 |
| regulation of gene-specific transcription | 39 out of 6178 genes, 0.6% | 39 out of 6259 genes, 0.6% | 1 |
| ribosome biogenesis | 39 out of 6178 genes, 0.6% | 39 out of 6259 genes, 0.6% | 1 |
| striated muscle cell differentiation | 39 out of 6178 genes, 0.6% | 39 out of 6259 genes, 0.6% | 1 |
| regulation of macromolecule metabolic process | 678 out of 6178 genes, 11.0% | 687 out of 6259 genes, 11.0% | 1 |
| vesicle-mediated transport | 315 out of 6178 genes, 5.1% | 319 out of 6259 genes, 5.1% | 1 |
| regulation of multicellular organismal process | 173 out of 6178 genes, 2.8% | 175 out of 6259 genes, 2.8% | 1 |
| actin cytoskeleton organization | 104 out of 6178 genes, 1.7% | 105 out of 6259 genes, 1.7% | 1 |
| post-embryonic development | 243 out of 6178 genes, 3.9% | 246 out of 6259 genes, 3.9% | 1 |
| glycerophospholipid metabolic process | 38 out of 6178 genes, 0.6% | 38 out of 6259 genes, 0.6% | 1 |
| nucleoside monophosphate biosynthetic process | 38 out of 6178 genes, 0.6% | 38 out of 6259 genes, 0.6% | 1 |
| response to biotic stimulus | 38 out of 6178 genes, 0.6% | 38 out of 6259 genes, 0.6% | 1 |
| negative regulation of cell communication | 38 out of 6178 genes, 0.6% | 38 out of 6259 genes, 0.6% | 1 |
| taxis | 103 out of 6178 genes, 1.7% | 104 out of 6259 genes, 1.7% | 1 |
| enzyme linked receptor protein signaling pathway | 102 out of 6178 genes, 1.7% | 103 out of 6259 genes, 1.6% | 1 |
| sensory organ development | 102 out of 6178 genes, 1.7% | 103 out of 6259 genes, 1.6% | 1 |
| alcohol metabolic process | 170 out of 6178 genes, 2.8% | 172 out of 6259 genes, 2.7% | 1 |
| DNA recombination | 37 out of 6178 genes, 0.6% | 37 out of 6259 genes, 0.6% | 1 |
| transcription initiation | 37 out of 6178 genes, 0.6% | 37 out of 6259 genes, 0.6% | 1 |
| nuclear transport | 37 out of 6178 genes, 0.6% | 37 out of 6259 genes, 0.6% | 1 |
| muscle cell development | 37 out of 6178 genes, 0.6% | 37 out of 6259 genes, 0.6% | 1 |
| muscle tissue development | 37 out of 6178 genes, 0.6% | 37 out of 6259 genes, 0.6% | 1 |
| cellular chemical homeostasis | 101 out of 6178 genes, 1.6% | 102 out of 6259 genes, 1.6% | 1 |
| cellular ion homeostasis | 100 out of 6178 genes, 1.6% | 101 out of 6259 genes, 1.6% | 1 |
| apoptosis | 100 out of 6178 genes, 1.6% | 101 out of 6259 genes, 1.6% | 1 |
| chemotaxis | 100 out of 6178 genes, 1.6% | 101 out of 6259 genes, 1.6% | 1 |
| regulation of transport | 100 out of 6178 genes, 1.6% | 101 out of 6259 genes, 1.6% | 1 |
| MAPKKK cascade | 36 out of 6178 genes, 0.6% | 36 out of 6259 genes, 0.6% | 1 |
| nuclear division | 36 out of 6178 genes, 0.6% | 36 out of 6259 genes, 0.6% | 1 |
| mitosis | 36 out of 6178 genes, 0.6% | 36 out of 6259 genes, 0.6% | 1 |
| striated muscle tissue development | 36 out of 6178 genes, 0.6% | 36 out of 6259 genes, 0.6% | 1 |
| di-, tri-valent inorganic cation homeostasis | 36 out of 6178 genes, 0.6% | 36 out of 6259 genes, 0.6% | 1 |
| transmembrane transport | 36 out of 6178 genes, 0.6% | 36 out of 6259 genes, 0.6% | 1 |
| regulation of primary metabolic process | 449 out of 6178 genes, 7.3% | 455 out of 6259 genes, 7.3% | 1 |
| regulation of gene expression | 520 out of 6178 genes, 8.4% | 527 out of 6259 genes, 8.4% | 1 |
| glucose metabolic process | 98 out of 6178 genes, 1.6% | 99 out of 6259 genes, 1.6% | 1 |
| sexual reproduction | 165 out of 6178 genes, 2.7% | 167 out of 6259 genes, 2.7% | 1 |
| neurotransmitter transport | 35 out of 6178 genes, 0.6% | 35 out of 6259 genes, 0.6% | 1 |
| establishment or maintenance of cell polarity | 35 out of 6178 genes, 0.6% | 35 out of 6259 genes, 0.6% | 1 |
| heart development | 35 out of 6178 genes, 0.6% | 35 out of 6259 genes, 0.6% | 1 |
| steroid metabolic process | 35 out of 6178 genes, 0.6% | 35 out of 6259 genes, 0.6% | 1 |
| protein alkylation | 35 out of 6178 genes, 0.6% | 35 out of 6259 genes, 0.6% | 1 |
| cellular component assembly involved in morphogenesis | 35 out of 6178 genes, 0.6% | 35 out of 6259 genes, 0.6% | 1 |
| establishment of organelle localization | 35 out of 6178 genes, 0.6% | 35 out of 6259 genes, 0.6% | 1 |
| spindle organization | 34 out of 6178 genes, 0.6% | 34 out of 6259 genes, 0.5% | 1 |
| axis specification | 34 out of 6178 genes, 0.6% | 34 out of 6259 genes, 0.5% | 1 |
| cellular di-, tri-valent inorganic cation homeostasis | 34 out of 6178 genes, 0.6% | 34 out of 6259 genes, 0.5% | 1 |
| protein acylation | 34 out of 6178 genes, 0.6% | 34 out of 6259 genes, 0.5% | 1 |
| establishment of localization | 1389 out of 6178 genes, 22.5% | 1408 out of 6259 genes, 22.5% | 1 |
| protein acetylation | 33 out of 6178 genes, 0.5% | 33 out of 6259 genes, 0.5% | 1 |
| fatty acid metabolic process | 33 out of 6178 genes, 0.5% | 33 out of 6259 genes, 0.5% | 1 |
| immune response | 33 out of 6178 genes, 0.5% | 33 out of 6259 genes, 0.5% | 1 |
| germ cell development | 33 out of 6178 genes, 0.5% | 33 out of 6259 genes, 0.5% | 1 |
| vesicle organization | 33 out of 6178 genes, 0.5% | 33 out of 6259 genes, 0.5% | 1 |
| regulation of protein kinase activity | 33 out of 6178 genes, 0.5% | 33 out of 6259 genes, 0.5% | 1 |
| response to other organism | 33 out of 6178 genes, 0.5% | 33 out of 6259 genes, 0.5% | 1 |
| striated muscle cell development | 33 out of 6178 genes, 0.5% | 33 out of 6259 genes, 0.5% | 1 |
| cell death | 158 out of 6178 genes, 2.6% | 160 out of 6259 genes, 2.6% | 1 |
| death | 158 out of 6178 genes, 2.6% | 160 out of 6259 genes, 2.6% | 1 |
| cellular aromatic compound metabolic process | 32 out of 6178 genes, 0.5% | 32 out of 6259 genes, 0.5% | 1 |
| nucleocytoplasmic transport | 32 out of 6178 genes, 0.5% | 32 out of 6259 genes, 0.5% | 1 |
| lipid biosynthetic process | 32 out of 6178 genes, 0.5% | 32 out of 6259 genes, 0.5% | 1 |
| regulation of organelle organization | 32 out of 6178 genes, 0.5% | 32 out of 6259 genes, 0.5% | 1 |
| protein localization to organelle | 32 out of 6178 genes, 0.5% | 32 out of 6259 genes, 0.5% | 1 |
| ion transmembrane transport | 32 out of 6178 genes, 0.5% | 32 out of 6259 genes, 0.5% | 1 |
| ncRNA processing | 32 out of 6178 genes, 0.5% | 32 out of 6259 genes, 0.5% | 1 |
| response to steroid hormone stimulus | 32 out of 6178 genes, 0.5% | 32 out of 6259 genes, 0.5% | 1 |
| transport | 1302 out of 6178 genes, 21.1% | 1320 out of 6259 genes, 21.1% | 1 |
| aging | 92 out of 6178 genes, 1.5% | 93 out of 6259 genes, 1.5% | 1 |
| intracellular signal transduction | 224 out of 6178 genes, 3.6% | 227 out of 6259 genes, 3.6% | 1 |
| protein glycosylation | 31 out of 6178 genes, 0.5% | 31 out of 6259 genes, 0.5% | 1 |
| negative regulation of macromolecule biosynthetic process | 31 out of 6178 genes, 0.5% | 31 out of 6259 genes, 0.5% | 1 |
| peptidyl-lysine modification | 31 out of 6178 genes, 0.5% | 31 out of 6259 genes, 0.5% | 1 |
| negative regulation of signaling pathway | 31 out of 6178 genes, 0.5% | 31 out of 6259 genes, 0.5% | 1 |
| macromolecule glycosylation | 31 out of 6178 genes, 0.5% | 31 out of 6259 genes, 0.5% | 1 |
| nucleic acid transport | 31 out of 6178 genes, 0.5% | 31 out of 6259 genes, 0.5% | 1 |
| regulation of secretion | 31 out of 6178 genes, 0.5% | 31 out of 6259 genes, 0.5% | 1 |
| behavioral interaction between organisms | 31 out of 6178 genes, 0.5% | 31 out of 6259 genes, 0.5% | 1 |
| glycosylation | 31 out of 6178 genes, 0.5% | 31 out of 6259 genes, 0.5% | 1 |
| negative regulation of cellular macromolecule biosynthetic process | 31 out of 6178 genes, 0.5% | 31 out of 6259 genes, 0.5% | 1 |
| gamete generation | 155 out of 6178 genes, 2.5% | 157 out of 6259 genes, 2.5% | 1 |
| protein methylation | 30 out of 6178 genes, 0.5% | 30 out of 6259 genes, 0.5% | 1 |
| neurotransmitter secretion | 30 out of 6178 genes, 0.5% | 30 out of 6259 genes, 0.5% | 1 |
| specification of symmetry | 30 out of 6178 genes, 0.5% | 30 out of 6259 genes, 0.5% | 1 |
| determination of bilateral symmetry | 30 out of 6178 genes, 0.5% | 30 out of 6259 genes, 0.5% | 1 |
| extracellular structure organization | 30 out of 6178 genes, 0.5% | 30 out of 6259 genes, 0.5% | 1 |
| negative regulation of nucleobase, nucleoside, nucleotide and nucleic acid metabolic process | 30 out of 6178 genes, 0.5% | 30 out of 6259 genes, 0.5% | 1 |
| developmental growth | 30 out of 6178 genes, 0.5% | 30 out of 6259 genes, 0.5% | 1 |
| RNA transport | 30 out of 6178 genes, 0.5% | 30 out of 6259 genes, 0.5% | 1 |
| negative regulation of nitrogen compound metabolic process | 30 out of 6178 genes, 0.5% | 30 out of 6259 genes, 0.5% | 1 |
| establishment of RNA localization | 30 out of 6178 genes, 0.5% | 30 out of 6259 genes, 0.5% | 1 |
| regulation of cellular metabolic process | 495 out of 6178 genes, 8.0% | 502 out of 6259 genes, 8.0% | 1 |
| nitrogen compound metabolic process | 1361 out of 6178 genes, 22.0% | 1380 out of 6259 genes, 22.0% | 1 |
| programmed cell death | 152 out of 6178 genes, 2.5% | 154 out of 6259 genes, 2.5% | 1 |
| regulation of growth | 151 out of 6178 genes, 2.4% | 153 out of 6259 genes, 2.4% | 1 |
| secretion | 87 out of 6178 genes, 1.4% | 88 out of 6259 genes, 1.4% | 1 |
| regulation of multicellular organismal development | 87 out of 6178 genes, 1.4% | 88 out of 6259 genes, 1.4% | 1 |
| internal protein amino acid acetylation | 29 out of 6178 genes, 0.5% | 29 out of 6259 genes, 0.5% | 1 |
| mitochondrion organization | 29 out of 6178 genes, 0.5% | 29 out of 6259 genes, 0.5% | 1 |
| regulation of neurogenesis | 29 out of 6178 genes, 0.5% | 29 out of 6259 genes, 0.5% | 1 |
| positive regulation of developmental process | 29 out of 6178 genes, 0.5% | 29 out of 6259 genes, 0.5% | 1 |
| amine metabolic process | 150 out of 6178 genes, 2.4% | 152 out of 6259 genes, 2.4% | 1 |
| chromatin organization | 148 out of 6178 genes, 2.4% | 150 out of 6259 genes, 2.4% | 1 |
| mating | 28 out of 6178 genes, 0.5% | 28 out of 6259 genes, 0.4% | 1 |
| negative regulation of transcription | 28 out of 6178 genes, 0.5% | 28 out of 6259 genes, 0.4% | 1 |
| histone acetylation | 28 out of 6178 genes, 0.5% | 28 out of 6259 genes, 0.4% | 1 |
| internal peptidyl-lysine acetylation | 28 out of 6178 genes, 0.5% | 28 out of 6259 genes, 0.4% | 1 |
| peptidyl-lysine acetylation | 28 out of 6178 genes, 0.5% | 28 out of 6259 genes, 0.4% | 1 |
| limb morphogenesis | 28 out of 6178 genes, 0.5% | 28 out of 6259 genes, 0.4% | 1 |
| regulation of gene expression, epigenetic | 28 out of 6178 genes, 0.5% | 28 out of 6259 genes, 0.4% | 1 |
| limb development | 28 out of 6178 genes, 0.5% | 28 out of 6259 genes, 0.4% | 1 |
| ameboidal cell migration | 27 out of 6178 genes, 0.4% | 27 out of 6259 genes, 0.4% | 1 |
| vacuole organization | 27 out of 6178 genes, 0.4% | 27 out of 6259 genes, 0.4% | 1 |
| regulation of mitotic cell cycle | 27 out of 6178 genes, 0.4% | 27 out of 6259 genes, 0.4% | 1 |
| monovalent inorganic cation transport | 27 out of 6178 genes, 0.4% | 27 out of 6259 genes, 0.4% | 1 |
| positive regulation of protein modification process | 27 out of 6178 genes, 0.4% | 27 out of 6259 genes, 0.4% | 1 |
| positive regulation of cellular protein metabolic process | 27 out of 6178 genes, 0.4% | 27 out of 6259 genes, 0.4% | 1 |
| imaginal disc-derived appendage morphogenesis | 27 out of 6178 genes, 0.4% | 27 out of 6259 genes, 0.4% | 1 |
| imaginal disc-derived appendage development | 27 out of 6178 genes, 0.4% | 27 out of 6259 genes, 0.4% | 1 |
| positive regulation of protein metabolic process | 27 out of 6178 genes, 0.4% | 27 out of 6259 genes, 0.4% | 1 |
| negative regulation of RNA metabolic process | 27 out of 6178 genes, 0.4% | 27 out of 6259 genes, 0.4% | 1 |
| protein maturation | 27 out of 6178 genes, 0.4% | 27 out of 6259 genes, 0.4% | 1 |
| epithelial tube morphogenesis | 27 out of 6178 genes, 0.4% | 27 out of 6259 genes, 0.4% | 1 |
| cell division | 83 out of 6178 genes, 1.3% | 84 out of 6259 genes, 1.3% | 1 |
| mRNA processing | 82 out of 6178 genes, 1.3% | 83 out of 6259 genes, 1.3% | 1 |
| response to inorganic substance | 82 out of 6178 genes, 1.3% | 83 out of 6259 genes, 1.3% | 1 |
| cation transport | 208 out of 6178 genes, 3.4% | 211 out of 6259 genes, 3.4% | 1 |
| signal transduction | 273 out of 6178 genes, 4.4% | 277 out of 6259 genes, 4.4% | 1 |
| transition metal ion transport | 26 out of 6178 genes, 0.4% | 26 out of 6259 genes, 0.4% | 1 |
| protein N-linked glycosylation | 26 out of 6178 genes, 0.4% | 26 out of 6259 genes, 0.4% | 1 |
| mating behavior | 26 out of 6178 genes, 0.4% | 26 out of 6259 genes, 0.4% | 1 |
| feeding behavior | 26 out of 6178 genes, 0.4% | 26 out of 6259 genes, 0.4% | 1 |
| developmental maturation | 26 out of 6178 genes, 0.4% | 26 out of 6259 genes, 0.4% | 1 |
| respiratory electron transport chain | 26 out of 6178 genes, 0.4% | 26 out of 6259 genes, 0.4% | 1 |
| phosphoinositide metabolic process | 26 out of 6178 genes, 0.4% | 26 out of 6259 genes, 0.4% | 1 |
| forebrain development | 26 out of 6178 genes, 0.4% | 26 out of 6259 genes, 0.4% | 1 |
| cell junction organization | 26 out of 6178 genes, 0.4% | 26 out of 6259 genes, 0.4% | 1 |
| post-embryonic appendage morphogenesis | 26 out of 6178 genes, 0.4% | 26 out of 6259 genes, 0.4% | 1 |
| molting cycle | 26 out of 6178 genes, 0.4% | 26 out of 6259 genes, 0.4% | 1 |
| negative regulation of transcription, DNA-dependent | 26 out of 6178 genes, 0.4% | 26 out of 6259 genes, 0.4% | 1 |
| gland development | 26 out of 6178 genes, 0.4% | 26 out of 6259 genes, 0.4% | 1 |
| regulation of DNA metabolic process | 26 out of 6178 genes, 0.4% | 26 out of 6259 genes, 0.4% | 1 |
| respiratory system development | 26 out of 6178 genes, 0.4% | 26 out of 6259 genes, 0.4% | 1 |
| regulation of vesicle-mediated transport | 26 out of 6178 genes, 0.4% | 26 out of 6259 genes, 0.4% | 1 |
| protein phosphorylation | 81 out of 6178 genes, 1.3% | 82 out of 6259 genes, 1.3% | 1 |
| regulation of neurotransmitter levels | 80 out of 6178 genes, 1.3% | 81 out of 6259 genes, 1.3% | 1 |
| cell cycle checkpoint | 25 out of 6178 genes, 0.4% | 25 out of 6259 genes, 0.4% | 1 |
| vasculature development | 25 out of 6178 genes, 0.4% | 25 out of 6259 genes, 0.4% | 1 |
| RNA catabolic process | 25 out of 6178 genes, 0.4% | 25 out of 6259 genes, 0.4% | 1 |
| learning | 25 out of 6178 genes, 0.4% | 25 out of 6259 genes, 0.4% | 1 |
| morphogenesis of embryonic epithelium | 25 out of 6178 genes, 0.4% | 25 out of 6259 genes, 0.4% | 1 |
| regulation of cell projection organization | 25 out of 6178 genes, 0.4% | 25 out of 6259 genes, 0.4% | 1 |
| development of primary sexual characteristics | 25 out of 6178 genes, 0.4% | 25 out of 6259 genes, 0.4% | 1 |
| positive regulation of cell differentiation | 25 out of 6178 genes, 0.4% | 25 out of 6259 genes, 0.4% | 1 |
| detection of stimulus | 25 out of 6178 genes, 0.4% | 25 out of 6259 genes, 0.4% | 1 |
| regulation of cell cycle arrest | 25 out of 6178 genes, 0.4% | 25 out of 6259 genes, 0.4% | 1 |
| female gamete generation | 79 out of 6178 genes, 1.3% | 80 out of 6259 genes, 1.3% | 1 |
| cellular membrane organization | 203 out of 6178 genes, 3.3% | 206 out of 6259 genes, 3.3% | 1 |
| membrane organization | 203 out of 6178 genes, 3.3% | 206 out of 6259 genes, 3.3% | 1 |
| cellular nitrogen compound metabolic process | 1322 out of 6178 genes, 21.4% | 1341 out of 6259 genes, 21.4% | 1 |
| RNA elongation | 24 out of 6178 genes, 0.4% | 24 out of 6259 genes, 0.4% | 1 |
| lipid transport | 24 out of 6178 genes, 0.4% | 24 out of 6259 genes, 0.4% | 1 |
| imaginal disc-derived wing morphogenesis | 24 out of 6178 genes, 0.4% | 24 out of 6259 genes, 0.4% | 1 |
| regulation of gene-specific transcription from RNA polymerase II promoter | 24 out of 6178 genes, 0.4% | 24 out of 6259 genes, 0.4% | 1 |
| regulation of MAPKKK cascade | 24 out of 6178 genes, 0.4% | 24 out of 6259 genes, 0.4% | 1 |
| response to peptide hormone stimulus | 24 out of 6178 genes, 0.4% | 24 out of 6259 genes, 0.4% | 1 |
| sensory perception of mechanical stimulus | 24 out of 6178 genes, 0.4% | 24 out of 6259 genes, 0.4% | 1 |
| regulation of protein serine/threonine kinase activity | 24 out of 6178 genes, 0.4% | 24 out of 6259 genes, 0.4% | 1 |
| cellular lipid metabolic process | 137 out of 6178 genes, 2.2% | 139 out of 6259 genes, 2.2% | 1 |
| phosphorus metabolic process | 200 out of 6178 genes, 3.2% | 203 out of 6259 genes, 3.2% | 1 |
| DNA replication | 76 out of 6178 genes, 1.2% | 77 out of 6259 genes, 1.2% | 1 |
| formation of primary germ layer | 23 out of 6178 genes, 0.4% | 23 out of 6259 genes, 0.4% | 1 |
| germarium-derived egg chamber formation | 23 out of 6178 genes, 0.4% | 23 out of 6259 genes, 0.4% | 1 |
| muscle organ development | 23 out of 6178 genes, 0.4% | 23 out of 6259 genes, 0.4% | 1 |
| cellular component disassembly | 23 out of 6178 genes, 0.4% | 23 out of 6259 genes, 0.4% | 1 |
| regulation of developmental growth | 23 out of 6178 genes, 0.4% | 23 out of 6259 genes, 0.4% | 1 |
| muscle fiber development | 23 out of 6178 genes, 0.4% | 23 out of 6259 genes, 0.4% | 1 |
| eye development | 75 out of 6178 genes, 1.2% | 76 out of 6259 genes, 1.2% | 1 |
| RNA splicing | 134 out of 6178 genes, 2.2% | 136 out of 6259 genes, 2.2% | 1 |
| chromosome organization | 196 out of 6178 genes, 3.2% | 199 out of 6259 genes, 3.2% | 1 |
| regulation of system process | 74 out of 6178 genes, 1.2% | 75 out of 6259 genes, 1.2% | 1 |
| in utero embryonic development | 22 out of 6178 genes, 0.4% | 22 out of 6259 genes, 0.4% | 1 |
| nematode larval development | 22 out of 6178 genes, 0.4% | 22 out of 6259 genes, 0.4% | 1 |
| protein folding | 22 out of 6178 genes, 0.4% | 22 out of 6259 genes, 0.4% | 1 |
| adult behavior | 22 out of 6178 genes, 0.4% | 22 out of 6259 genes, 0.4% | 1 |
| cell maturation | 22 out of 6178 genes, 0.4% | 22 out of 6259 genes, 0.4% | 1 |
| nucleus localization | 22 out of 6178 genes, 0.4% | 22 out of 6259 genes, 0.4% | 1 |
| regulation of peptidase activity | 22 out of 6178 genes, 0.4% | 22 out of 6259 genes, 0.4% | 1 |
| ubiquitin-dependent protein catabolic process | 72 out of 6178 genes, 1.2% | 73 out of 6259 genes, 1.2% | 1 |
| nuclear-transcribed mRNA catabolic process | 21 out of 6178 genes, 0.3% | 21 out of 6259 genes, 0.3% | 1 |
| morphogenesis of a branching structure | 21 out of 6178 genes, 0.3% | 21 out of 6259 genes, 0.3% | 1 |
| positive regulation of protein phosphorylation | 21 out of 6178 genes, 0.3% | 21 out of 6259 genes, 0.3% | 1 |
| nucleotide-excision repair | 21 out of 6178 genes, 0.3% | 21 out of 6259 genes, 0.3% | 1 |
| mRNA catabolic process | 21 out of 6178 genes, 0.3% | 21 out of 6259 genes, 0.3% | 1 |
| protein lipidation | 21 out of 6178 genes, 0.3% | 21 out of 6259 genes, 0.3% | 1 |
| cellular calcium ion homeostasis | 21 out of 6178 genes, 0.3% | 21 out of 6259 genes, 0.3% | 1 |
| membrane budding | 21 out of 6178 genes, 0.3% | 21 out of 6259 genes, 0.3% | 1 |
| response to bacterium | 21 out of 6178 genes, 0.3% | 21 out of 6259 genes, 0.3% | 1 |
| anterior/posterior pattern formation | 21 out of 6178 genes, 0.3% | 21 out of 6259 genes, 0.3% | 1 |
| positive regulation of phosphorus metabolic process | 21 out of 6178 genes, 0.3% | 21 out of 6259 genes, 0.3% | 1 |
| regulation of striated muscle tissue development | 21 out of 6178 genes, 0.3% | 21 out of 6259 genes, 0.3% | 1 |
| macromolecular complex disassembly | 21 out of 6178 genes, 0.3% | 21 out of 6259 genes, 0.3% | 1 |
| gliogenesis | 21 out of 6178 genes, 0.3% | 21 out of 6259 genes, 0.3% | 1 |
| lipoprotein metabolic process | 21 out of 6178 genes, 0.3% | 21 out of 6259 genes, 0.3% | 1 |
| lipoprotein biosynthetic process | 21 out of 6178 genes, 0.3% | 21 out of 6259 genes, 0.3% | 1 |
| positive regulation of phosphorylation | 21 out of 6178 genes, 0.3% | 21 out of 6259 genes, 0.3% | 1 |
| eating behavior | 21 out of 6178 genes, 0.3% | 21 out of 6259 genes, 0.3% | 1 |
| positive regulation of phosphate metabolic process | 21 out of 6178 genes, 0.3% | 21 out of 6259 genes, 0.3% | 1 |
| regulation of muscle organ development | 21 out of 6178 genes, 0.3% | 21 out of 6259 genes, 0.3% | 1 |
| regulation of synapse structure and activity | 21 out of 6178 genes, 0.3% | 21 out of 6259 genes, 0.3% | 1 |
| regulation of synapse organization | 21 out of 6178 genes, 0.3% | 21 out of 6259 genes, 0.3% | 1 |
| regulation of muscle cell differentiation | 21 out of 6178 genes, 0.3% | 21 out of 6259 genes, 0.3% | 1 |
| calcium ion homeostasis | 21 out of 6178 genes, 0.3% | 21 out of 6259 genes, 0.3% | 1 |
| skeletal muscle organ development | 21 out of 6178 genes, 0.3% | 21 out of 6259 genes, 0.3% | 1 |
| cellular component disassembly at cellular level | 21 out of 6178 genes, 0.3% | 21 out of 6259 genes, 0.3% | 1 |
| regulation of response to stress | 21 out of 6178 genes, 0.3% | 21 out of 6259 genes, 0.3% | 1 |
| response to external stimulus | 190 out of 6178 genes, 3.1% | 193 out of 6259 genes, 3.1% | 1 |
| cellular amine metabolic process | 128 out of 6178 genes, 2.1% | 130 out of 6259 genes, 2.1% | 1 |
| mesoderm development | 20 out of 6178 genes, 0.3% | 20 out of 6259 genes, 0.3% | 1 |
| skeletal muscle tissue development | 20 out of 6178 genes, 0.3% | 20 out of 6259 genes, 0.3% | 1 |
| response to carbohydrate stimulus | 20 out of 6178 genes, 0.3% | 20 out of 6259 genes, 0.3% | 1 |
| carbohydrate biosynthetic process | 20 out of 6178 genes, 0.3% | 20 out of 6259 genes, 0.3% | 1 |
| posttranscriptional gene silencing | 20 out of 6178 genes, 0.3% | 20 out of 6259 genes, 0.3% | 1 |
| molting cycle, protein-based cuticle | 20 out of 6178 genes, 0.3% | 20 out of 6259 genes, 0.3% | 1 |
| RNA 3'-end processing | 20 out of 6178 genes, 0.3% | 20 out of 6259 genes, 0.3% | 1 |
| posttranscriptional gene silencing by RNA | 20 out of 6178 genes, 0.3% | 20 out of 6259 genes, 0.3% | 1 |
| embryonic organ morphogenesis | 20 out of 6178 genes, 0.3% | 20 out of 6259 genes, 0.3% | 1 |
| regulation of skeletal muscle tissue development | 20 out of 6178 genes, 0.3% | 20 out of 6259 genes, 0.3% | 1 |
| skeletal muscle fiber development | 20 out of 6178 genes, 0.3% | 20 out of 6259 genes, 0.3% | 1 |
| regulation of skeletal muscle fiber development | 20 out of 6178 genes, 0.3% | 20 out of 6259 genes, 0.3% | 1 |
| sensory perception of light stimulus | 20 out of 6178 genes, 0.3% | 20 out of 6259 genes, 0.3% | 1 |
| regulation of striated muscle cell differentiation | 20 out of 6178 genes, 0.3% | 20 out of 6259 genes, 0.3% | 1 |
| nucleobase, nucleoside and nucleotide metabolic process | 247 out of 6178 genes, 4.0% | 251 out of 6259 genes, 4.0% | 1 |
| negative regulation of macromolecule metabolic process | 67 out of 6178 genes, 1.1% | 68 out of 6259 genes, 1.1% | 1 |
| sister chromatid segregation | 19 out of 6178 genes, 0.3% | 19 out of 6259 genes, 0.3% | 1 |
| regulation of action potential | 19 out of 6178 genes, 0.3% | 19 out of 6259 genes, 0.3% | 1 |
| blastocyst development | 19 out of 6178 genes, 0.3% | 19 out of 6259 genes, 0.3% | 1 |
| immune effector process | 19 out of 6178 genes, 0.3% | 19 out of 6259 genes, 0.3% | 1 |
| pyrimidine nucleotide metabolic process | 19 out of 6178 genes, 0.3% | 19 out of 6259 genes, 0.3% | 1 |
| neutral lipid metabolic process | 19 out of 6178 genes, 0.3% | 19 out of 6259 genes, 0.3% | 1 |
| vesicle coating | 19 out of 6178 genes, 0.3% | 19 out of 6259 genes, 0.3% | 1 |
| tRNA processing | 19 out of 6178 genes, 0.3% | 19 out of 6259 genes, 0.3% | 1 |
| actin polymerization or depolymerization | 19 out of 6178 genes, 0.3% | 19 out of 6259 genes, 0.3% | 1 |
| detection of external stimulus | 19 out of 6178 genes, 0.3% | 19 out of 6259 genes, 0.3% | 1 |
| regulation of cell adhesion | 19 out of 6178 genes, 0.3% | 19 out of 6259 genes, 0.3% | 1 |
| DNA integrity checkpoint | 19 out of 6178 genes, 0.3% | 19 out of 6259 genes, 0.3% | 1 |
| positive regulation of kinase activity | 19 out of 6178 genes, 0.3% | 19 out of 6259 genes, 0.3% | 1 |
| cellular macromolecular complex disassembly | 19 out of 6178 genes, 0.3% | 19 out of 6259 genes, 0.3% | 1 |
| establishment of nucleus localization | 19 out of 6178 genes, 0.3% | 19 out of 6259 genes, 0.3% | 1 |
| protein complex disassembly | 19 out of 6178 genes, 0.3% | 19 out of 6259 genes, 0.3% | 1 |
| branching morphogenesis of a tube | 19 out of 6178 genes, 0.3% | 19 out of 6259 genes, 0.3% | 1 |
| stem cell differentiation | 19 out of 6178 genes, 0.3% | 19 out of 6259 genes, 0.3% | 1 |
| positive regulation of cellular component organization | 19 out of 6178 genes, 0.3% | 19 out of 6259 genes, 0.3% | 1 |
| interphase | 19 out of 6178 genes, 0.3% | 19 out of 6259 genes, 0.3% | 1 |
| positive regulation of transferase activity | 19 out of 6178 genes, 0.3% | 19 out of 6259 genes, 0.3% | 1 |
| regulation of cytoskeleton organization | 19 out of 6178 genes, 0.3% | 19 out of 6259 genes, 0.3% | 1 |
| generation of precursor metabolites and energy | 123 out of 6178 genes, 2.0% | 125 out of 6259 genes, 2.0% | 1 |
| embryonic axis specification | 18 out of 6178 genes, 0.3% | 18 out of 6259 genes, 0.3% | 1 |
| urogenital system development | 18 out of 6178 genes, 0.3% | 18 out of 6259 genes, 0.3% | 1 |
| leukocyte differentiation | 18 out of 6178 genes, 0.3% | 18 out of 6259 genes, 0.3% | 1 |
| positive regulation of immune system process | 18 out of 6178 genes, 0.3% | 18 out of 6259 genes, 0.3% | 1 |
| circulatory system process | 18 out of 6178 genes, 0.3% | 18 out of 6259 genes, 0.3% | 1 |
| aminoglycan metabolic process | 18 out of 6178 genes, 0.3% | 18 out of 6259 genes, 0.3% | 1 |
| acylglycerol metabolic process | 18 out of 6178 genes, 0.3% | 18 out of 6259 genes, 0.3% | 1 |
| glycerol ether metabolic process | 18 out of 6178 genes, 0.3% | 18 out of 6259 genes, 0.3% | 1 |
| ensheathment of neurons | 18 out of 6178 genes, 0.3% | 18 out of 6259 genes, 0.3% | 1 |
| regulation of synaptic growth at neuromuscular junction | 18 out of 6178 genes, 0.3% | 18 out of 6259 genes, 0.3% | 1 |
| detection of abiotic stimulus | 18 out of 6178 genes, 0.3% | 18 out of 6259 genes, 0.3% | 1 |
| response to hexose stimulus | 18 out of 6178 genes, 0.3% | 18 out of 6259 genes, 0.3% | 1 |
| glial cell differentiation | 18 out of 6178 genes, 0.3% | 18 out of 6259 genes, 0.3% | 1 |
| body morphogenesis | 18 out of 6178 genes, 0.3% | 18 out of 6259 genes, 0.3% | 1 |
| regulation of hormone levels | 18 out of 6178 genes, 0.3% | 18 out of 6259 genes, 0.3% | 1 |
| proton transport | 18 out of 6178 genes, 0.3% | 18 out of 6259 genes, 0.3% | 1 |
| organic ether metabolic process | 18 out of 6178 genes, 0.3% | 18 out of 6259 genes, 0.3% | 1 |
| regulation of action potential in neuron | 18 out of 6178 genes, 0.3% | 18 out of 6259 genes, 0.3% | 1 |
| respiratory tube development | 18 out of 6178 genes, 0.3% | 18 out of 6259 genes, 0.3% | 1 |
| epithelial cell differentiation | 18 out of 6178 genes, 0.3% | 18 out of 6259 genes, 0.3% | 1 |
| regulation of actin cytoskeleton organization | 18 out of 6178 genes, 0.3% | 18 out of 6259 genes, 0.3% | 1 |
| regulation of actin filament-based process | 18 out of 6178 genes, 0.3% | 18 out of 6259 genes, 0.3% | 1 |
| response to monosaccharide stimulus | 18 out of 6178 genes, 0.3% | 18 out of 6259 genes, 0.3% | 1 |
| regulation of tube size | 18 out of 6178 genes, 0.3% | 18 out of 6259 genes, 0.3% | 1 |
| mesoderm morphogenesis | 18 out of 6178 genes, 0.3% | 18 out of 6259 genes, 0.3% | 1 |
| regulation of synaptogenesis | 18 out of 6178 genes, 0.3% | 18 out of 6259 genes, 0.3% | 1 |
| larval development | 179 out of 6178 genes, 2.9% | 182 out of 6259 genes, 2.9% | 1 |
| negative regulation of transcription from RNA polymerase II promoter | 17 out of 6178 genes, 0.3% | 17 out of 6259 genes, 0.3% | 1 |
| polysaccharide biosynthetic process | 17 out of 6178 genes, 0.3% | 17 out of 6259 genes, 0.3% | 1 |
| cytokinesis | 17 out of 6178 genes, 0.3% | 17 out of 6259 genes, 0.3% | 1 |
| ossification | 17 out of 6178 genes, 0.3% | 17 out of 6259 genes, 0.3% | 1 |
| somatic diversification of immune receptors | 17 out of 6178 genes, 0.3% | 17 out of 6259 genes, 0.3% | 1 |
| regulation of leukocyte activation | 17 out of 6178 genes, 0.3% | 17 out of 6259 genes, 0.3% | 1 |
| RNA export from nucleus | 17 out of 6178 genes, 0.3% | 17 out of 6259 genes, 0.3% | 1 |
| cell recognition | 17 out of 6178 genes, 0.3% | 17 out of 6259 genes, 0.3% | 1 |
| neuron recognition | 17 out of 6178 genes, 0.3% | 17 out of 6259 genes, 0.3% | 1 |
| organic acid catabolic process | 17 out of 6178 genes, 0.3% | 17 out of 6259 genes, 0.3% | 1 |
| regulation of endocytosis | 17 out of 6178 genes, 0.3% | 17 out of 6259 genes, 0.3% | 1 |
| intracellular receptor mediated signaling pathway | 17 out of 6178 genes, 0.3% | 17 out of 6259 genes, 0.3% | 1 |
| regulation of actin filament length | 17 out of 6178 genes, 0.3% | 17 out of 6259 genes, 0.3% | 1 |
| cell junction assembly | 17 out of 6178 genes, 0.3% | 17 out of 6259 genes, 0.3% | 1 |
| tube formation | 17 out of 6178 genes, 0.3% | 17 out of 6259 genes, 0.3% | 1 |
| pharyngeal pumping | 17 out of 6178 genes, 0.3% | 17 out of 6259 genes, 0.3% | 1 |
| pigmentation | 17 out of 6178 genes, 0.3% | 17 out of 6259 genes, 0.3% | 1 |
| cellular protein complex disassembly | 17 out of 6178 genes, 0.3% | 17 out of 6259 genes, 0.3% | 1 |
| dicarboxylic acid metabolic process | 17 out of 6178 genes, 0.3% | 17 out of 6259 genes, 0.3% | 1 |
| carboxylic acid catabolic process | 17 out of 6178 genes, 0.3% | 17 out of 6259 genes, 0.3% | 1 |
| regulation of hormone secretion | 17 out of 6178 genes, 0.3% | 17 out of 6259 genes, 0.3% | 1 |
| synaptic vesicle transport | 17 out of 6178 genes, 0.3% | 17 out of 6259 genes, 0.3% | 1 |
| developmental cell growth | 17 out of 6178 genes, 0.3% | 17 out of 6259 genes, 0.3% | 1 |
| reproductive structure development | 17 out of 6178 genes, 0.3% | 17 out of 6259 genes, 0.3% | 1 |
| regulation of cell activation | 17 out of 6178 genes, 0.3% | 17 out of 6259 genes, 0.3% | 1 |
| nuclear export | 17 out of 6178 genes, 0.3% | 17 out of 6259 genes, 0.3% | 1 |
| protein modification by small protein removal | 17 out of 6178 genes, 0.3% | 17 out of 6259 genes, 0.3% | 1 |
| ribonucleoprotein complex subunit organization | 17 out of 6178 genes, 0.3% | 17 out of 6259 genes, 0.3% | 1 |
| purine nucleotide metabolic process | 175 out of 6178 genes, 2.8% | 178 out of 6259 genes, 2.8% | 1 |
| G-protein coupled receptor protein signaling pathway | 62 out of 6178 genes, 1.0% | 63 out of 6259 genes, 1.0% | 1 |
| cellular respiration | 62 out of 6178 genes, 1.0% | 63 out of 6259 genes, 1.0% | 1 |
| translation | 174 out of 6178 genes, 2.8% | 177 out of 6259 genes, 2.8% | 1 |
| heterocycle metabolic process | 234 out of 6178 genes, 3.8% | 238 out of 6259 genes, 3.8% | 1 |
| organophosphate metabolic process | 61 out of 6178 genes, 1.0% | 62 out of 6259 genes, 1.0% | 1 |
| secretion by cell | 61 out of 6178 genes, 1.0% | 62 out of 6259 genes, 1.0% | 1 |
| RNA splicing, via transesterification reactions | 114 out of 6178 genes, 1.8% | 116 out of 6259 genes, 1.9% | 1 |
| syncytium formation by plasma membrane fusion | 16 out of 6178 genes, 0.3% | 16 out of 6259 genes, 0.3% | 1 |
| heart morphogenesis | 16 out of 6178 genes, 0.3% | 16 out of 6259 genes, 0.3% | 1 |
| muscle contraction | 16 out of 6178 genes, 0.3% | 16 out of 6259 genes, 0.3% | 1 |
| syncytium formation | 16 out of 6178 genes, 0.3% | 16 out of 6259 genes, 0.3% | 1 |
| nucleus organization | 16 out of 6178 genes, 0.3% | 16 out of 6259 genes, 0.3% | 1 |
| female meiosis | 16 out of 6178 genes, 0.3% | 16 out of 6259 genes, 0.3% | 1 |
| transforming growth factor beta receptor signaling pathway | 16 out of 6178 genes, 0.3% | 16 out of 6259 genes, 0.3% | 1 |
| sensory perception of chemical stimulus | 16 out of 6178 genes, 0.3% | 16 out of 6259 genes, 0.3% | 1 |
| courtship behavior | 16 out of 6178 genes, 0.3% | 16 out of 6259 genes, 0.3% | 1 |
| associative learning | 16 out of 6178 genes, 0.3% | 16 out of 6259 genes, 0.3% | 1 |
| gonad development | 16 out of 6178 genes, 0.3% | 16 out of 6259 genes, 0.3% | 1 |
| nucleobase metabolic process | 16 out of 6178 genes, 0.3% | 16 out of 6259 genes, 0.3% | 1 |
| nucleoside metabolic process | 16 out of 6178 genes, 0.3% | 16 out of 6259 genes, 0.3% | 1 |
| detection of light stimulus | 16 out of 6178 genes, 0.3% | 16 out of 6259 genes, 0.3% | 1 |
| regulation of cell morphogenesis involved in differentiation | 16 out of 6178 genes, 0.3% | 16 out of 6259 genes, 0.3% | 1 |
| endosome transport | 16 out of 6178 genes, 0.3% | 16 out of 6259 genes, 0.3% | 1 |
| protein import | 16 out of 6178 genes, 0.3% | 16 out of 6259 genes, 0.3% | 1 |
| regulation of cell morphogenesis | 16 out of 6178 genes, 0.3% | 16 out of 6259 genes, 0.3% | 1 |
| ribonucleoprotein complex assembly | 16 out of 6178 genes, 0.3% | 16 out of 6259 genes, 0.3% | 1 |
| behavioral signaling | 16 out of 6178 genes, 0.3% | 16 out of 6259 genes, 0.3% | 1 |
| steroid hormone receptor signaling pathway | 16 out of 6178 genes, 0.3% | 16 out of 6259 genes, 0.3% | 1 |
| embryonic appendage morphogenesis | 16 out of 6178 genes, 0.3% | 16 out of 6259 genes, 0.3% | 1 |
| regulation of ion transport | 16 out of 6178 genes, 0.3% | 16 out of 6259 genes, 0.3% | 1 |
| cell-cell junction organization | 16 out of 6178 genes, 0.3% | 16 out of 6259 genes, 0.3% | 1 |
| negative regulation of cell cycle | 16 out of 6178 genes, 0.3% | 16 out of 6259 genes, 0.3% | 1 |
| regulation of behavior | 16 out of 6178 genes, 0.3% | 16 out of 6259 genes, 0.3% | 1 |
| negative regulation of transport | 16 out of 6178 genes, 0.3% | 16 out of 6259 genes, 0.3% | 1 |
| cellular response to organic substance | 16 out of 6178 genes, 0.3% | 16 out of 6259 genes, 0.3% | 1 |
| nucleoside phosphate metabolic process | 231 out of 6178 genes, 3.7% | 235 out of 6259 genes, 3.8% | 1 |
| nucleotide metabolic process | 231 out of 6178 genes, 3.7% | 235 out of 6259 genes, 3.8% | 1 |
| proteasomal protein catabolic process | 60 out of 6178 genes, 1.0% | 61 out of 6259 genes, 1.0% | 1 |
| cellular macromolecular complex assembly | 113 out of 6178 genes, 1.8% | 115 out of 6259 genes, 1.8% | 1 |
| positive regulation of biological process | 292 out of 6178 genes, 4.7% | 297 out of 6259 genes, 4.7% | 1 |
| positive regulation of catalytic activity | 59 out of 6178 genes, 1.0% | 60 out of 6259 genes, 1.0% | 1 |
| mitotic sister chromatid segregation | 15 out of 6178 genes, 0.2% | 15 out of 6259 genes, 0.2% | 1 |
| recombinational repair | 15 out of 6178 genes, 0.2% | 15 out of 6259 genes, 0.2% | 1 |
| cell fate specification | 15 out of 6178 genes, 0.2% | 15 out of 6259 genes, 0.2% | 1 |
| morphogenesis of a polarized epithelium | 15 out of 6178 genes, 0.2% | 15 out of 6259 genes, 0.2% | 1 |
| regulation of peptide secretion | 15 out of 6178 genes, 0.2% | 15 out of 6259 genes, 0.2% | 1 |
| nuclear migration | 15 out of 6178 genes, 0.2% | 15 out of 6259 genes, 0.2% | 1 |
| meiosis I | 15 out of 6178 genes, 0.2% | 15 out of 6259 genes, 0.2% | 1 |
| epidermis development | 15 out of 6178 genes, 0.2% | 15 out of 6259 genes, 0.2% | 1 |
| response to mechanical stimulus | 15 out of 6178 genes, 0.2% | 15 out of 6259 genes, 0.2% | 1 |
| dorsal/ventral pattern formation | 15 out of 6178 genes, 0.2% | 15 out of 6259 genes, 0.2% | 1 |
| glial cell development | 15 out of 6178 genes, 0.2% | 15 out of 6259 genes, 0.2% | 1 |
| membrane docking | 15 out of 6178 genes, 0.2% | 15 out of 6259 genes, 0.2% | 1 |
| lymphocyte differentiation | 15 out of 6178 genes, 0.2% | 15 out of 6259 genes, 0.2% | 1 |
| embryonic limb morphogenesis | 15 out of 6178 genes, 0.2% | 15 out of 6259 genes, 0.2% | 1 |
| ovarian follicle cell development | 15 out of 6178 genes, 0.2% | 15 out of 6259 genes, 0.2% | 1 |
| actomyosin structure organization | 15 out of 6178 genes, 0.2% | 15 out of 6259 genes, 0.2% | 1 |
| microtubule polymerization or depolymerization | 15 out of 6178 genes, 0.2% | 15 out of 6259 genes, 0.2% | 1 |
| response to corticosteroid stimulus | 15 out of 6178 genes, 0.2% | 15 out of 6259 genes, 0.2% | 1 |
| fin development | 15 out of 6178 genes, 0.2% | 15 out of 6259 genes, 0.2% | 1 |
| camera-type eye development | 15 out of 6178 genes, 0.2% | 15 out of 6259 genes, 0.2% | 1 |
| positive regulation of response to stimulus | 15 out of 6178 genes, 0.2% | 15 out of 6259 genes, 0.2% | 1 |
| regulation of insulin secretion | 15 out of 6178 genes, 0.2% | 15 out of 6259 genes, 0.2% | 1 |
| neuromuscular process | 15 out of 6178 genes, 0.2% | 15 out of 6259 genes, 0.2% | 1 |
| regulation of lymphocyte activation | 15 out of 6178 genes, 0.2% | 15 out of 6259 genes, 0.2% | 1 |
| vesicle localization | 15 out of 6178 genes, 0.2% | 15 out of 6259 genes, 0.2% | 1 |
| DNA conformation change | 15 out of 6178 genes, 0.2% | 15 out of 6259 genes, 0.2% | 1 |
| regulation of peptide transport | 15 out of 6178 genes, 0.2% | 15 out of 6259 genes, 0.2% | 1 |
| regulation of peptide hormone secretion | 15 out of 6178 genes, 0.2% | 15 out of 6259 genes, 0.2% | 1 |
| small GTPase mediated signal transduction | 58 out of 6178 genes, 0.9% | 59 out of 6259 genes, 0.9% | 1 |
| posttranscriptional regulation of gene expression | 58 out of 6178 genes, 0.9% | 59 out of 6259 genes, 0.9% | 1 |
| negative regulation of gene expression | 58 out of 6178 genes, 0.9% | 59 out of 6259 genes, 0.9% | 1 |
| gene expression | 883 out of 6178 genes, 14.3% | 897 out of 6259 genes, 14.3% | 1 |
| RNA splicing, via transesterification reactions with bulged adenosine as nucleophile | 109 out of 6178 genes, 1.8% | 111 out of 6259 genes, 1.8% | 1 |
| cellular macromolecular complex subunit organization | 165 out of 6178 genes, 2.7% | 168 out of 6259 genes, 2.7% | 1 |
| actin filament organization | 56 out of 6178 genes, 0.9% | 57 out of 6259 genes, 0.9% | 1 |
| proteasomal ubiquitin-dependent protein catabolic process | 56 out of 6178 genes, 0.9% | 57 out of 6259 genes, 0.9% | 1 |
| response to acid | 14 out of 6178 genes, 0.2% | 14 out of 6259 genes, 0.2% | 1 |
| kidney development | 14 out of 6178 genes, 0.2% | 14 out of 6259 genes, 0.2% | 1 |
| somatic diversification of immune receptors via germline recombination within a single locus | 14 out of 6178 genes, 0.2% | 14 out of 6259 genes, 0.2% | 1 |
| proteoglycan metabolic process | 14 out of 6178 genes, 0.2% | 14 out of 6259 genes, 0.2% | 1 |
| pyrimidine nucleotide biosynthetic process | 14 out of 6178 genes, 0.2% | 14 out of 6259 genes, 0.2% | 1 |
| DNA-dependent DNA replication initiation | 14 out of 6178 genes, 0.2% | 14 out of 6259 genes, 0.2% | 1 |
| ER-nucleus signaling pathway | 14 out of 6178 genes, 0.2% | 14 out of 6259 genes, 0.2% | 1 |
| memory | 14 out of 6178 genes, 0.2% | 14 out of 6259 genes, 0.2% | 1 |
| asymmetric protein localization | 14 out of 6178 genes, 0.2% | 14 out of 6259 genes, 0.2% | 1 |
| phospholipid biosynthetic process | 14 out of 6178 genes, 0.2% | 14 out of 6259 genes, 0.2% | 1 |
| glutamine family amino acid metabolic process | 14 out of 6178 genes, 0.2% | 14 out of 6259 genes, 0.2% | 1 |
| purine nucleoside monophosphate metabolic process | 14 out of 6178 genes, 0.2% | 14 out of 6259 genes, 0.2% | 1 |
| purine nucleoside monophosphate biosynthetic process | 14 out of 6178 genes, 0.2% | 14 out of 6259 genes, 0.2% | 1 |
| response to toxin | 14 out of 6178 genes, 0.2% | 14 out of 6259 genes, 0.2% | 1 |
| positive regulation of peptidase activity | 14 out of 6178 genes, 0.2% | 14 out of 6259 genes, 0.2% | 1 |
| somatic cell DNA recombination | 14 out of 6178 genes, 0.2% | 14 out of 6259 genes, 0.2% | 1 |
| protein processing | 14 out of 6178 genes, 0.2% | 14 out of 6259 genes, 0.2% | 1 |
| response to insulin stimulus | 14 out of 6178 genes, 0.2% | 14 out of 6259 genes, 0.2% | 1 |
| fin morphogenesis | 14 out of 6178 genes, 0.2% | 14 out of 6259 genes, 0.2% | 1 |
| embryonic heart tube development | 14 out of 6178 genes, 0.2% | 14 out of 6259 genes, 0.2% | 1 |
| signal transduction in response to DNA damage | 14 out of 6178 genes, 0.2% | 14 out of 6259 genes, 0.2% | 1 |
| regulation of MAP kinase activity | 14 out of 6178 genes, 0.2% | 14 out of 6259 genes, 0.2% | 1 |
| vesicle docking | 14 out of 6178 genes, 0.2% | 14 out of 6259 genes, 0.2% | 1 |
| negative regulation of cellular component organization | 14 out of 6178 genes, 0.2% | 14 out of 6259 genes, 0.2% | 1 |
| protein depolymerization | 14 out of 6178 genes, 0.2% | 14 out of 6259 genes, 0.2% | 1 |
| regulation of endopeptidase activity | 14 out of 6178 genes, 0.2% | 14 out of 6259 genes, 0.2% | 1 |
| renal system development | 14 out of 6178 genes, 0.2% | 14 out of 6259 genes, 0.2% | 1 |
| phospholipid metabolic process | 55 out of 6178 genes, 0.9% | 56 out of 6259 genes, 0.9% | 1 |
| positive regulation of cell death | 55 out of 6178 genes, 0.9% | 56 out of 6259 genes, 0.9% | 1 |
| positive regulation of programmed cell death | 55 out of 6178 genes, 0.9% | 56 out of 6259 genes, 0.9% | 1 |
| regulation of cellular localization | 55 out of 6178 genes, 0.9% | 56 out of 6259 genes, 0.9% | 1 |
| phosphorylation | 105 out of 6178 genes, 1.7% | 107 out of 6259 genes, 1.7% | 1 |
| protein modification by small protein conjugation or removal | 104 out of 6178 genes, 1.7% | 106 out of 6259 genes, 1.7% | 1 |
| sulfur amino acid metabolic process | 13 out of 6178 genes, 0.2% | 13 out of 6259 genes, 0.2% | 1 |
| skeletal system development | 13 out of 6178 genes, 0.2% | 13 out of 6259 genes, 0.2% | 1 |
| blood vessel development | 13 out of 6178 genes, 0.2% | 13 out of 6259 genes, 0.2% | 1 |
| embryonic heart tube morphogenesis | 13 out of 6178 genes, 0.2% | 13 out of 6259 genes, 0.2% | 1 |
| rRNA processing | 13 out of 6178 genes, 0.2% | 13 out of 6259 genes, 0.2% | 1 |
| protein deacetylation | 13 out of 6178 genes, 0.2% | 13 out of 6259 genes, 0.2% | 1 |
| peptide metabolic process | 13 out of 6178 genes, 0.2% | 13 out of 6259 genes, 0.2% | 1 |
| protein import into nucleus | 13 out of 6178 genes, 0.2% | 13 out of 6259 genes, 0.2% | 1 |
| microtubule depolymerization | 13 out of 6178 genes, 0.2% | 13 out of 6259 genes, 0.2% | 1 |
| cell-cell junction assembly | 13 out of 6178 genes, 0.2% | 13 out of 6259 genes, 0.2% | 1 |
| ovarian follicle cell migration | 13 out of 6178 genes, 0.2% | 13 out of 6259 genes, 0.2% | 1 |
| open tracheal system development | 13 out of 6178 genes, 0.2% | 13 out of 6259 genes, 0.2% | 1 |
| protein-based cuticle development | 13 out of 6178 genes, 0.2% | 13 out of 6259 genes, 0.2% | 1 |
| regulation of heart contraction | 13 out of 6178 genes, 0.2% | 13 out of 6259 genes, 0.2% | 1 |
| regulation of actin polymerization or depolymerization | 13 out of 6178 genes, 0.2% | 13 out of 6259 genes, 0.2% | 1 |
| negative regulation of cell proliferation | 13 out of 6178 genes, 0.2% | 13 out of 6259 genes, 0.2% | 1 |
| axon ensheathment | 13 out of 6178 genes, 0.2% | 13 out of 6259 genes, 0.2% | 1 |
| ribonucleoside monophosphate biosynthetic process | 13 out of 6178 genes, 0.2% | 13 out of 6259 genes, 0.2% | 1 |
| ribonucleoside monophosphate metabolic process | 13 out of 6178 genes, 0.2% | 13 out of 6259 genes, 0.2% | 1 |
| response to organic cyclic substance | 13 out of 6178 genes, 0.2% | 13 out of 6259 genes, 0.2% | 1 |
| rRNA metabolic process | 13 out of 6178 genes, 0.2% | 13 out of 6259 genes, 0.2% | 1 |
| histone methylation | 13 out of 6178 genes, 0.2% | 13 out of 6259 genes, 0.2% | 1 |
| regulation of lipid metabolic process | 13 out of 6178 genes, 0.2% | 13 out of 6259 genes, 0.2% | 1 |
| telencephalon development | 13 out of 6178 genes, 0.2% | 13 out of 6259 genes, 0.2% | 1 |
| hindbrain development | 13 out of 6178 genes, 0.2% | 13 out of 6259 genes, 0.2% | 1 |
| negative regulation of kinase activity | 13 out of 6178 genes, 0.2% | 13 out of 6259 genes, 0.2% | 1 |
| protein localization to nucleus | 13 out of 6178 genes, 0.2% | 13 out of 6259 genes, 0.2% | 1 |
| exocrine system development | 13 out of 6178 genes, 0.2% | 13 out of 6259 genes, 0.2% | 1 |
| protein deacylation | 13 out of 6178 genes, 0.2% | 13 out of 6259 genes, 0.2% | 1 |
| establishment of mitotic spindle localization | 13 out of 6178 genes, 0.2% | 13 out of 6259 genes, 0.2% | 1 |
| regulation of caspase activity | 13 out of 6178 genes, 0.2% | 13 out of 6259 genes, 0.2% | 1 |
| sulfur compound biosynthetic process | 13 out of 6178 genes, 0.2% | 13 out of 6259 genes, 0.2% | 1 |
| glycerolipid biosynthetic process | 13 out of 6178 genes, 0.2% | 13 out of 6259 genes, 0.2% | 1 |
| regulation of neuron differentiation | 13 out of 6178 genes, 0.2% | 13 out of 6259 genes, 0.2% | 1 |
| glycerophospholipid biosynthetic process | 13 out of 6178 genes, 0.2% | 13 out of 6259 genes, 0.2% | 1 |
| regulation of body fluid levels | 13 out of 6178 genes, 0.2% | 13 out of 6259 genes, 0.2% | 1 |
| nuclear import | 13 out of 6178 genes, 0.2% | 13 out of 6259 genes, 0.2% | 1 |
| establishment of spindle localization | 13 out of 6178 genes, 0.2% | 13 out of 6259 genes, 0.2% | 1 |
| negative regulation of transferase activity | 13 out of 6178 genes, 0.2% | 13 out of 6259 genes, 0.2% | 1 |
| spindle localization | 13 out of 6178 genes, 0.2% | 13 out of 6259 genes, 0.2% | 1 |
| anatomical structure homeostasis | 13 out of 6178 genes, 0.2% | 13 out of 6259 genes, 0.2% | 1 |
| establishment or maintenance of bipolar cell polarity | 13 out of 6178 genes, 0.2% | 13 out of 6259 genes, 0.2% | 1 |
| metal ion transport | 158 out of 6178 genes, 2.6% | 161 out of 6259 genes, 2.6% | 1 |
| protein ubiquitination | 53 out of 6178 genes, 0.9% | 54 out of 6259 genes, 0.9% | 1 |
| regulation of neurological system process | 53 out of 6178 genes, 0.9% | 54 out of 6259 genes, 0.9% | 1 |
| regulation of growth rate | 102 out of 6178 genes, 1.7% | 104 out of 6259 genes, 1.7% | 1 |
| positive regulation of biosynthetic process | 52 out of 6178 genes, 0.8% | 53 out of 6259 genes, 0.8% | 1 |
| regulation of transmission of nerve impulse | 52 out of 6178 genes, 0.8% | 53 out of 6259 genes, 0.8% | 1 |
| biosynthetic process | 858 out of 6178 genes, 13.9% | 872 out of 6259 genes, 13.9% | 1 |
| mesoderm formation | 12 out of 6178 genes, 0.2% | 12 out of 6259 genes, 0.2% | 1 |
| cell fate determination | 12 out of 6178 genes, 0.2% | 12 out of 6259 genes, 0.2% | 1 |
| embryonic epithelial tube formation | 12 out of 6178 genes, 0.2% | 12 out of 6259 genes, 0.2% | 1 |
| adaptive immune response | 12 out of 6178 genes, 0.2% | 12 out of 6259 genes, 0.2% | 1 |
| adaptive immune response based on somatic recombination of immune receptors built from immunoglobulin superfamily domains | 12 out of 6178 genes, 0.2% | 12 out of 6259 genes, 0.2% | 1 |
| aminoglycan biosynthetic process | 12 out of 6178 genes, 0.2% | 12 out of 6259 genes, 0.2% | 1 |
| regulation of translation | 12 out of 6178 genes, 0.2% | 12 out of 6259 genes, 0.2% | 1 |
| triglyceride metabolic process | 12 out of 6178 genes, 0.2% | 12 out of 6259 genes, 0.2% | 1 |
| cellular membrane fusion | 12 out of 6178 genes, 0.2% | 12 out of 6259 genes, 0.2% | 1 |
| humoral immune response | 12 out of 6178 genes, 0.2% | 12 out of 6259 genes, 0.2% | 1 |
| salivary gland development | 12 out of 6178 genes, 0.2% | 12 out of 6259 genes, 0.2% | 1 |
| blood circulation | 12 out of 6178 genes, 0.2% | 12 out of 6259 genes, 0.2% | 1 |
| oligosaccharide metabolic process | 12 out of 6178 genes, 0.2% | 12 out of 6259 genes, 0.2% | 1 |
| response to UV | 12 out of 6178 genes, 0.2% | 12 out of 6259 genes, 0.2% | 1 |
| detection of visible light | 12 out of 6178 genes, 0.2% | 12 out of 6259 genes, 0.2% | 1 |
| drug transport | 12 out of 6178 genes, 0.2% | 12 out of 6259 genes, 0.2% | 1 |
| viral reproduction | 12 out of 6178 genes, 0.2% | 12 out of 6259 genes, 0.2% | 1 |
| organic acid biosynthetic process | 12 out of 6178 genes, 0.2% | 12 out of 6259 genes, 0.2% | 1 |
| axon choice point recognition | 12 out of 6178 genes, 0.2% | 12 out of 6259 genes, 0.2% | 1 |
| stem cell maintenance | 12 out of 6178 genes, 0.2% | 12 out of 6259 genes, 0.2% | 1 |
| viral reproductive process | 12 out of 6178 genes, 0.2% | 12 out of 6259 genes, 0.2% | 1 |
| cell projection assembly | 12 out of 6178 genes, 0.2% | 12 out of 6259 genes, 0.2% | 1 |
| proteoglycan biosynthetic process | 12 out of 6178 genes, 0.2% | 12 out of 6259 genes, 0.2% | 1 |
| metallo-sulfur cluster assembly | 12 out of 6178 genes, 0.2% | 12 out of 6259 genes, 0.2% | 1 |
| regulation of defense response | 12 out of 6178 genes, 0.2% | 12 out of 6259 genes, 0.2% | 1 |
| cell cycle cytokinesis | 12 out of 6178 genes, 0.2% | 12 out of 6259 genes, 0.2% | 1 |
| response to cytokine stimulus | 12 out of 6178 genes, 0.2% | 12 out of 6259 genes, 0.2% | 1 |
| hemocyte differentiation | 12 out of 6178 genes, 0.2% | 12 out of 6259 genes, 0.2% | 1 |
| response to drug | 12 out of 6178 genes, 0.2% | 12 out of 6259 genes, 0.2% | 1 |
| positive regulation of caspase activity | 12 out of 6178 genes, 0.2% | 12 out of 6259 genes, 0.2% | 1 |
| ear development | 12 out of 6178 genes, 0.2% | 12 out of 6259 genes, 0.2% | 1 |
| positive regulation of protein kinase activity | 12 out of 6178 genes, 0.2% | 12 out of 6259 genes, 0.2% | 1 |
| carboxylic acid biosynthetic process | 12 out of 6178 genes, 0.2% | 12 out of 6259 genes, 0.2% | 1 |
| blood vessel morphogenesis | 12 out of 6178 genes, 0.2% | 12 out of 6259 genes, 0.2% | 1 |
| stem cell development | 12 out of 6178 genes, 0.2% | 12 out of 6259 genes, 0.2% | 1 |
| protein homooligomerization | 12 out of 6178 genes, 0.2% | 12 out of 6259 genes, 0.2% | 1 |
| membrane fusion | 12 out of 6178 genes, 0.2% | 12 out of 6259 genes, 0.2% | 1 |
| epithelial tube formation | 12 out of 6178 genes, 0.2% | 12 out of 6259 genes, 0.2% | 1 |
| transmembrane receptor protein tyrosine kinase signaling pathway | 50 out of 6178 genes, 0.8% | 51 out of 6259 genes, 0.8% | 1 |
| immune system process | 151 out of 6178 genes, 2.4% | 154 out of 6259 genes, 2.5% | 1 |
| nuclear mRNA splicing, via spliceosome | 49 out of 6178 genes, 0.8% | 50 out of 6259 genes, 0.8% | 1 |
| induction of programmed cell death | 49 out of 6178 genes, 0.8% | 50 out of 6259 genes, 0.8% | 1 |
| negative regulation of cellular metabolic process | 49 out of 6178 genes, 0.8% | 50 out of 6259 genes, 0.8% | 1 |
| eye morphogenesis | 49 out of 6178 genes, 0.8% | 50 out of 6259 genes, 0.8% | 1 |
| regulation of cell growth | 11 out of 6178 genes, 0.2% | 11 out of 6259 genes, 0.2% | 1 |
| gastrulation with mouth forming first | 11 out of 6178 genes, 0.2% | 11 out of 6259 genes, 0.2% | 1 |
| regulation of cytokine production | 11 out of 6178 genes, 0.2% | 11 out of 6259 genes, 0.2% | 1 |
| production of molecular mediator of immune response | 11 out of 6178 genes, 0.2% | 11 out of 6259 genes, 0.2% | 1 |
| leukocyte mediated immunity | 11 out of 6178 genes, 0.2% | 11 out of 6259 genes, 0.2% | 1 |
| lymphocyte mediated immunity | 11 out of 6178 genes, 0.2% | 11 out of 6259 genes, 0.2% | 1 |
| glycerol metabolic process | 11 out of 6178 genes, 0.2% | 11 out of 6259 genes, 0.2% | 1 |
| acetyl-CoA metabolic process | 11 out of 6178 genes, 0.2% | 11 out of 6259 genes, 0.2% | 1 |
| chromatin remodeling | 11 out of 6178 genes, 0.2% | 11 out of 6259 genes, 0.2% | 1 |
| cell cycle arrest | 11 out of 6178 genes, 0.2% | 11 out of 6259 genes, 0.2% | 1 |
| mitotic spindle organization | 11 out of 6178 genes, 0.2% | 11 out of 6259 genes, 0.2% | 1 |
| blastoderm segmentation | 11 out of 6178 genes, 0.2% | 11 out of 6259 genes, 0.2% | 1 |
| dorsal closure | 11 out of 6178 genes, 0.2% | 11 out of 6259 genes, 0.2% | 1 |
| excretion | 11 out of 6178 genes, 0.2% | 11 out of 6259 genes, 0.2% | 1 |
| adult locomotory behavior | 11 out of 6178 genes, 0.2% | 11 out of 6259 genes, 0.2% | 1 |
| response to temperature stimulus | 11 out of 6178 genes, 0.2% | 11 out of 6259 genes, 0.2% | 1 |
| amine catabolic process | 11 out of 6178 genes, 0.2% | 11 out of 6259 genes, 0.2% | 1 |
| negative regulation of signal transduction | 11 out of 6178 genes, 0.2% | 11 out of 6259 genes, 0.2% | 1 |
| synaptic vesicle exocytosis | 11 out of 6178 genes, 0.2% | 11 out of 6259 genes, 0.2% | 1 |
| sterol metabolic process | 11 out of 6178 genes, 0.2% | 11 out of 6259 genes, 0.2% | 1 |
| spinal cord development | 11 out of 6178 genes, 0.2% | 11 out of 6259 genes, 0.2% | 1 |
| nerve development | 11 out of 6178 genes, 0.2% | 11 out of 6259 genes, 0.2% | 1 |
| negative regulation of signaling process | 11 out of 6178 genes, 0.2% | 11 out of 6259 genes, 0.2% | 1 |
| actin filament polymerization | 11 out of 6178 genes, 0.2% | 11 out of 6259 genes, 0.2% | 1 |
| establishment or maintenance of apical/basal cell polarity | 11 out of 6178 genes, 0.2% | 11 out of 6259 genes, 0.2% | 1 |
| segmentation | 11 out of 6178 genes, 0.2% | 11 out of 6259 genes, 0.2% | 1 |
| photoreceptor cell development | 11 out of 6178 genes, 0.2% | 11 out of 6259 genes, 0.2% | 1 |
| defense response to bacterium | 11 out of 6178 genes, 0.2% | 11 out of 6259 genes, 0.2% | 1 |
| regulation of transcription factor import into nucleus | 11 out of 6178 genes, 0.2% | 11 out of 6259 genes, 0.2% | 1 |
| positive regulation of MAPKKK cascade | 11 out of 6178 genes, 0.2% | 11 out of 6259 genes, 0.2% | 1 |
| developmental pigmentation | 11 out of 6178 genes, 0.2% | 11 out of 6259 genes, 0.2% | 1 |
| inner ear development | 11 out of 6178 genes, 0.2% | 11 out of 6259 genes, 0.2% | 1 |
| mRNA transport | 11 out of 6178 genes, 0.2% | 11 out of 6259 genes, 0.2% | 1 |
| protein polymerization | 11 out of 6178 genes, 0.2% | 11 out of 6259 genes, 0.2% | 1 |
| monovalent inorganic cation homeostasis | 11 out of 6178 genes, 0.2% | 11 out of 6259 genes, 0.2% | 1 |
| positive regulation of metabolic process | 96 out of 6178 genes, 1.6% | 98 out of 6259 genes, 1.6% | 1 |
| polysaccharide metabolic process | 48 out of 6178 genes, 0.8% | 49 out of 6259 genes, 0.8% | 1 |
| one-carbon metabolic process | 48 out of 6178 genes, 0.8% | 49 out of 6259 genes, 0.8% | 1 |
| response to extracellular stimulus | 48 out of 6178 genes, 0.8% | 49 out of 6259 genes, 0.8% | 1 |
| negative regulation of molecular function | 47 out of 6178 genes, 0.8% | 48 out of 6259 genes, 0.8% | 1 |
| regulation of synaptic transmission | 47 out of 6178 genes, 0.8% | 48 out of 6259 genes, 0.8% | 1 |
| energy derivation by oxidation of organic compounds | 94 out of 6178 genes, 1.5% | 96 out of 6259 genes, 1.5% | 1 |
| cellular biosynthetic process | 835 out of 6178 genes, 13.5% | 849 out of 6259 genes, 13.6% | 1 |
| endocytosis | 145 out of 6178 genes, 2.3% | 148 out of 6259 genes, 2.4% | 1 |
| membrane invagination | 145 out of 6178 genes, 2.3% | 148 out of 6259 genes, 2.4% | 1 |
| cell activation | 46 out of 6178 genes, 0.7% | 47 out of 6259 genes, 0.8% | 1 |
| response to nutrient levels | 46 out of 6178 genes, 0.7% | 47 out of 6259 genes, 0.8% | 1 |
| regulation of DNA recombination | 10 out of 6178 genes, 0.2% | 10 out of 6259 genes, 0.2% | 1 |
| regulation of cyclin-dependent protein kinase activity | 10 out of 6178 genes, 0.2% | 10 out of 6259 genes, 0.2% | 1 |
| response to molecule of bacterial origin | 10 out of 6178 genes, 0.2% | 10 out of 6259 genes, 0.2% | 1 |
| pyrimidine base metabolic process | 10 out of 6178 genes, 0.2% | 10 out of 6259 genes, 0.2% | 1 |
| chromatin assembly or disassembly | 10 out of 6178 genes, 0.2% | 10 out of 6259 genes, 0.2% | 1 |
| translational initiation | 10 out of 6178 genes, 0.2% | 10 out of 6259 genes, 0.2% | 1 |
| imaginal disc pattern formation | 10 out of 6178 genes, 0.2% | 10 out of 6259 genes, 0.2% | 1 |
| regulation of Notch signaling pathway | 10 out of 6178 genes, 0.2% | 10 out of 6259 genes, 0.2% | 1 |
| induction of apoptosis by intracellular signals | 10 out of 6178 genes, 0.2% | 10 out of 6259 genes, 0.2% | 1 |
| ribonucleoside metabolic process | 10 out of 6178 genes, 0.2% | 10 out of 6259 genes, 0.2% | 1 |
| purine ribonucleoside monophosphate metabolic process | 10 out of 6178 genes, 0.2% | 10 out of 6259 genes, 0.2% | 1 |
| purine ribonucleoside monophosphate biosynthetic process | 10 out of 6178 genes, 0.2% | 10 out of 6259 genes, 0.2% | 1 |
| pyrimidine ribonucleotide metabolic process | 10 out of 6178 genes, 0.2% | 10 out of 6259 genes, 0.2% | 1 |
| response to ionizing radiation | 10 out of 6178 genes, 0.2% | 10 out of 6259 genes, 0.2% | 1 |
| regulation of neuron projection development | 10 out of 6178 genes, 0.2% | 10 out of 6259 genes, 0.2% | 1 |
| energy coupled proton transport, down electrochemical gradient | 10 out of 6178 genes, 0.2% | 10 out of 6259 genes, 0.2% | 1 |
| ATP synthesis coupled proton transport | 10 out of 6178 genes, 0.2% | 10 out of 6259 genes, 0.2% | 1 |
| B cell mediated immunity | 10 out of 6178 genes, 0.2% | 10 out of 6259 genes, 0.2% | 1 |
| pallium development | 10 out of 6178 genes, 0.2% | 10 out of 6259 genes, 0.2% | 1 |
| central nervous system neuron differentiation | 10 out of 6178 genes, 0.2% | 10 out of 6259 genes, 0.2% | 1 |
| regulation of proteolysis | 10 out of 6178 genes, 0.2% | 10 out of 6259 genes, 0.2% | 1 |
| negative regulation of Wnt receptor signaling pathway | 10 out of 6178 genes, 0.2% | 10 out of 6259 genes, 0.2% | 1 |
| T cell differentiation | 10 out of 6178 genes, 0.2% | 10 out of 6259 genes, 0.2% | 1 |
| pancreas development | 10 out of 6178 genes, 0.2% | 10 out of 6259 genes, 0.2% | 1 |
| microtubule organizing center organization | 10 out of 6178 genes, 0.2% | 10 out of 6259 genes, 0.2% | 1 |
| mRNA 3'-end processing | 10 out of 6178 genes, 0.2% | 10 out of 6259 genes, 0.2% | 1 |
| cellular response to extracellular stimulus | 10 out of 6178 genes, 0.2% | 10 out of 6259 genes, 0.2% | 1 |
| cellular response to nutrient levels | 10 out of 6178 genes, 0.2% | 10 out of 6259 genes, 0.2% | 1 |
| cytokinetic process | 10 out of 6178 genes, 0.2% | 10 out of 6259 genes, 0.2% | 1 |
| regulation of homeostatic process | 10 out of 6178 genes, 0.2% | 10 out of 6259 genes, 0.2% | 1 |
| purine nucleoside metabolic process | 10 out of 6178 genes, 0.2% | 10 out of 6259 genes, 0.2% | 1 |
| purine ribonucleoside metabolic process | 10 out of 6178 genes, 0.2% | 10 out of 6259 genes, 0.2% | 1 |
| regulation of immune response | 10 out of 6178 genes, 0.2% | 10 out of 6259 genes, 0.2% | 1 |
| detection of stimulus involved in sensory perception | 10 out of 6178 genes, 0.2% | 10 out of 6259 genes, 0.2% | 1 |
| establishment of vesicle localization | 10 out of 6178 genes, 0.2% | 10 out of 6259 genes, 0.2% | 1 |
| lipid homeostasis | 10 out of 6178 genes, 0.2% | 10 out of 6259 genes, 0.2% | 1 |
| mesenchyme development | 10 out of 6178 genes, 0.2% | 10 out of 6259 genes, 0.2% | 1 |
| cellular response to external stimulus | 10 out of 6178 genes, 0.2% | 10 out of 6259 genes, 0.2% | 1 |
| tissue migration | 10 out of 6178 genes, 0.2% | 10 out of 6259 genes, 0.2% | 1 |
| signal transmission via phosphorylation event | 92 out of 6178 genes, 1.5% | 94 out of 6259 genes, 1.5% | 1 |
| cellular protein complex assembly | 92 out of 6178 genes, 1.5% | 94 out of 6259 genes, 1.5% | 1 |
| organic substance transport | 92 out of 6178 genes, 1.5% | 94 out of 6259 genes, 1.5% | 1 |
| ribonucleotide metabolic process | 142 out of 6178 genes, 2.3% | 145 out of 6259 genes, 2.3% | 1 |
| leukocyte activation | 44 out of 6178 genes, 0.7% | 45 out of 6259 genes, 0.7% | 1 |
| DNA damage checkpoint | 9 out of 6178 genes, 0.1% | 9 out of 6259 genes, 0.1% | 1 |
| blastocyst growth | 9 out of 6178 genes, 0.1% | 9 out of 6259 genes, 0.1% | 1 |
| epithelial cell development | 9 out of 6178 genes, 0.1% | 9 out of 6259 genes, 0.1% | 1 |
| activation of immune response | 9 out of 6178 genes, 0.1% | 9 out of 6259 genes, 0.1% | 1 |
| immunoglobulin production | 9 out of 6178 genes, 0.1% | 9 out of 6259 genes, 0.1% | 1 |
| immune response-activating signal transduction | 9 out of 6178 genes, 0.1% | 9 out of 6259 genes, 0.1% | 1 |
| immune response-regulating signaling pathway | 9 out of 6178 genes, 0.1% | 9 out of 6259 genes, 0.1% | 1 |
| peptide secretion | 9 out of 6178 genes, 0.1% | 9 out of 6259 genes, 0.1% | 1 |
| vascular process in circulatory system | 9 out of 6178 genes, 0.1% | 9 out of 6259 genes, 0.1% | 1 |
| purine base metabolic process | 9 out of 6178 genes, 0.1% | 9 out of 6259 genes, 0.1% | 1 |
| mRNA export from nucleus | 9 out of 6178 genes, 0.1% | 9 out of 6259 genes, 0.1% | 1 |
| translational elongation | 9 out of 6178 genes, 0.1% | 9 out of 6259 genes, 0.1% | 1 |
| 'de novo' protein folding | 9 out of 6178 genes, 0.1% | 9 out of 6259 genes, 0.1% | 1 |
| cellular amino acid derivative metabolic process | 9 out of 6178 genes, 0.1% | 9 out of 6259 genes, 0.1% | 1 |
| protein targeting to membrane | 9 out of 6178 genes, 0.1% | 9 out of 6259 genes, 0.1% | 1 |
| glycolipid metabolic process | 9 out of 6178 genes, 0.1% | 9 out of 6259 genes, 0.1% | 1 |
| isoprenoid metabolic process | 9 out of 6178 genes, 0.1% | 9 out of 6259 genes, 0.1% | 1 |
| calcium ion transport | 9 out of 6178 genes, 0.1% | 9 out of 6259 genes, 0.1% | 1 |
| striated muscle contraction | 9 out of 6178 genes, 0.1% | 9 out of 6259 genes, 0.1% | 1 |
| vacuolar transport | 9 out of 6178 genes, 0.1% | 9 out of 6259 genes, 0.1% | 1 |
| male meiosis | 9 out of 6178 genes, 0.1% | 9 out of 6259 genes, 0.1% | 1 |
| Rho protein signal transduction | 9 out of 6178 genes, 0.1% | 9 out of 6259 genes, 0.1% | 1 |
| nerve-nerve synaptic transmission | 9 out of 6178 genes, 0.1% | 9 out of 6259 genes, 0.1% | 1 |
| ovarian nurse cell to oocyte transport | 9 out of 6178 genes, 0.1% | 9 out of 6259 genes, 0.1% | 1 |
| oocyte construction | 9 out of 6178 genes, 0.1% | 9 out of 6259 genes, 0.1% | 1 |
| oocyte axis specification | 9 out of 6178 genes, 0.1% | 9 out of 6259 genes, 0.1% | 1 |
| rhythmic behavior | 9 out of 6178 genes, 0.1% | 9 out of 6259 genes, 0.1% | 1 |
| cellular amino acid catabolic process | 9 out of 6178 genes, 0.1% | 9 out of 6259 genes, 0.1% | 1 |
| pyrimidine ribonucleotide biosynthetic process | 9 out of 6178 genes, 0.1% | 9 out of 6259 genes, 0.1% | 1 |
| deoxyribonucleotide metabolic process | 9 out of 6178 genes, 0.1% | 9 out of 6259 genes, 0.1% | 1 |
| 2'-deoxyribonucleotide metabolic process | 9 out of 6178 genes, 0.1% | 9 out of 6259 genes, 0.1% | 1 |
| hormone transport | 9 out of 6178 genes, 0.1% | 9 out of 6259 genes, 0.1% | 1 |
| oocyte differentiation | 9 out of 6178 genes, 0.1% | 9 out of 6259 genes, 0.1% | 1 |
| response to organic nitrogen | 9 out of 6178 genes, 0.1% | 9 out of 6259 genes, 0.1% | 1 |
| mesenchymal cell development | 9 out of 6178 genes, 0.1% | 9 out of 6259 genes, 0.1% | 1 |
| monocarboxylic acid transport | 9 out of 6178 genes, 0.1% | 9 out of 6259 genes, 0.1% | 1 |
| peptide transport | 9 out of 6178 genes, 0.1% | 9 out of 6259 genes, 0.1% | 1 |
| immunoglobulin mediated immune response | 9 out of 6178 genes, 0.1% | 9 out of 6259 genes, 0.1% | 1 |
| somatic diversification of immunoglobulins | 9 out of 6178 genes, 0.1% | 9 out of 6259 genes, 0.1% | 1 |
| somatic recombination of immunoglobulin gene segments | 9 out of 6178 genes, 0.1% | 9 out of 6259 genes, 0.1% | 1 |
| establishment of cell polarity | 9 out of 6178 genes, 0.1% | 9 out of 6259 genes, 0.1% | 1 |
| cytokinesis after meiosis | 9 out of 6178 genes, 0.1% | 9 out of 6259 genes, 0.1% | 1 |
| post-embryonic hemopoiesis | 9 out of 6178 genes, 0.1% | 9 out of 6259 genes, 0.1% | 1 |
| larval lymph gland hemopoiesis | 9 out of 6178 genes, 0.1% | 9 out of 6259 genes, 0.1% | 1 |
| larval lymph gland hemocyte differentiation | 9 out of 6178 genes, 0.1% | 9 out of 6259 genes, 0.1% | 1 |
| gene silencing by miRNA | 9 out of 6178 genes, 0.1% | 9 out of 6259 genes, 0.1% | 1 |
| non-canonical Wnt receptor signaling pathway | 9 out of 6178 genes, 0.1% | 9 out of 6259 genes, 0.1% | 1 |
| vulval development | 9 out of 6178 genes, 0.1% | 9 out of 6259 genes, 0.1% | 1 |
| fluid transport | 9 out of 6178 genes, 0.1% | 9 out of 6259 genes, 0.1% | 1 |
| ethanolamine and derivative metabolic process | 9 out of 6178 genes, 0.1% | 9 out of 6259 genes, 0.1% | 1 |
| response to estrogen stimulus | 9 out of 6178 genes, 0.1% | 9 out of 6259 genes, 0.1% | 1 |
| nucleobase biosynthetic process | 9 out of 6178 genes, 0.1% | 9 out of 6259 genes, 0.1% | 1 |
| hormone secretion | 9 out of 6178 genes, 0.1% | 9 out of 6259 genes, 0.1% | 1 |
| lymph gland development | 9 out of 6178 genes, 0.1% | 9 out of 6259 genes, 0.1% | 1 |
| oocyte development | 9 out of 6178 genes, 0.1% | 9 out of 6259 genes, 0.1% | 1 |
| mesenchymal cell differentiation | 9 out of 6178 genes, 0.1% | 9 out of 6259 genes, 0.1% | 1 |
| positive regulation of immune response | 9 out of 6178 genes, 0.1% | 9 out of 6259 genes, 0.1% | 1 |
| regulation of T cell activation | 9 out of 6178 genes, 0.1% | 9 out of 6259 genes, 0.1% | 1 |
| actin filament bundle assembly | 9 out of 6178 genes, 0.1% | 9 out of 6259 genes, 0.1% | 1 |
| cofactor transport | 9 out of 6178 genes, 0.1% | 9 out of 6259 genes, 0.1% | 1 |
| positive regulation of multicellular organismal process | 9 out of 6178 genes, 0.1% | 9 out of 6259 genes, 0.1% | 1 |
| S phase | 9 out of 6178 genes, 0.1% | 9 out of 6259 genes, 0.1% | 1 |
| retina development in camera-type eye | 9 out of 6178 genes, 0.1% | 9 out of 6259 genes, 0.1% | 1 |
| regulation of cellular response to stress | 9 out of 6178 genes, 0.1% | 9 out of 6259 genes, 0.1% | 1 |
| metabolic process | 3432 out of 6178 genes, 55.6% | 3482 out of 6259 genes, 55.6% | 1 |
| protein modification by small protein conjugation | 87 out of 6178 genes, 1.4% | 89 out of 6259 genes, 1.4% | 1 |
| amine transport | 42 out of 6178 genes, 0.7% | 43 out of 6259 genes, 0.7% | 1 |
| lymphocyte activation | 42 out of 6178 genes, 0.7% | 43 out of 6259 genes, 0.7% | 1 |
| DNA-dependent DNA replication | 41 out of 6178 genes, 0.7% | 42 out of 6259 genes, 0.7% | 1 |
| purine ribonucleotide metabolic process | 134 out of 6178 genes, 2.2% | 137 out of 6259 genes, 2.2% | 1 |
| meiotic spindle organization | 8 out of 6178 genes, 0.1% | 8 out of 6259 genes, 0.1% | 1 |
| spliceosomal snRNP assembly | 8 out of 6178 genes, 0.1% | 8 out of 6259 genes, 0.1% | 1 |
| telomere maintenance | 8 out of 6178 genes, 0.1% | 8 out of 6259 genes, 0.1% | 1 |
| angiogenesis | 8 out of 6178 genes, 0.1% | 8 out of 6259 genes, 0.1% | 1 |
| establishment of planar polarity | 8 out of 6178 genes, 0.1% | 8 out of 6259 genes, 0.1% | 1 |
| immune response-activating cell surface receptor signaling pathway | 8 out of 6178 genes, 0.1% | 8 out of 6259 genes, 0.1% | 1 |
| positive regulation of leukocyte activation | 8 out of 6178 genes, 0.1% | 8 out of 6259 genes, 0.1% | 1 |
| regulation of immune effector process | 8 out of 6178 genes, 0.1% | 8 out of 6259 genes, 0.1% | 1 |
| immune response-regulating cell surface receptor signaling pathway | 8 out of 6178 genes, 0.1% | 8 out of 6259 genes, 0.1% | 1 |
| neural retina development | 8 out of 6178 genes, 0.1% | 8 out of 6259 genes, 0.1% | 1 |
| amino sugar metabolic process | 8 out of 6178 genes, 0.1% | 8 out of 6259 genes, 0.1% | 1 |
| glucosamine metabolic process | 8 out of 6178 genes, 0.1% | 8 out of 6259 genes, 0.1% | 1 |
| chromatin silencing | 8 out of 6178 genes, 0.1% | 8 out of 6259 genes, 0.1% | 1 |
| GPI anchor metabolic process | 8 out of 6178 genes, 0.1% | 8 out of 6259 genes, 0.1% | 1 |
| GPI anchor biosynthetic process | 8 out of 6178 genes, 0.1% | 8 out of 6259 genes, 0.1% | 1 |
| steroid biosynthetic process | 8 out of 6178 genes, 0.1% | 8 out of 6259 genes, 0.1% | 1 |
| regulation of pH | 8 out of 6178 genes, 0.1% | 8 out of 6259 genes, 0.1% | 1 |
| receptor-mediated endocytosis | 8 out of 6178 genes, 0.1% | 8 out of 6259 genes, 0.1% | 1 |
| autophagy | 8 out of 6178 genes, 0.1% | 8 out of 6259 genes, 0.1% | 1 |
| establishment of tissue polarity | 8 out of 6178 genes, 0.1% | 8 out of 6259 genes, 0.1% | 1 |
| dorsal/ventral pattern formation, imaginal disc | 8 out of 6178 genes, 0.1% | 8 out of 6259 genes, 0.1% | 1 |
| female gonad development | 8 out of 6178 genes, 0.1% | 8 out of 6259 genes, 0.1% | 1 |
| regulation of smoothened signaling pathway | 8 out of 6178 genes, 0.1% | 8 out of 6259 genes, 0.1% | 1 |
| hexose transport | 8 out of 6178 genes, 0.1% | 8 out of 6259 genes, 0.1% | 1 |
| deoxyribonucleoside metabolic process | 8 out of 6178 genes, 0.1% | 8 out of 6259 genes, 0.1% | 1 |
| nucleoside diphosphate metabolic process | 8 out of 6178 genes, 0.1% | 8 out of 6259 genes, 0.1% | 1 |
| pyrimidine nucleoside triphosphate metabolic process | 8 out of 6178 genes, 0.1% | 8 out of 6259 genes, 0.1% | 1 |
| epidermal cell differentiation | 8 out of 6178 genes, 0.1% | 8 out of 6259 genes, 0.1% | 1 |
| regulation of metal ion transport | 8 out of 6178 genes, 0.1% | 8 out of 6259 genes, 0.1% | 1 |
| heparan sulfate proteoglycan biosynthetic process | 8 out of 6178 genes, 0.1% | 8 out of 6259 genes, 0.1% | 1 |
| cadmium ion transport | 8 out of 6178 genes, 0.1% | 8 out of 6259 genes, 0.1% | 1 |
| organic anion transport | 8 out of 6178 genes, 0.1% | 8 out of 6259 genes, 0.1% | 1 |
| monosaccharide transport | 8 out of 6178 genes, 0.1% | 8 out of 6259 genes, 0.1% | 1 |
| organic alcohol transport | 8 out of 6178 genes, 0.1% | 8 out of 6259 genes, 0.1% | 1 |
| nucleoside transport | 8 out of 6178 genes, 0.1% | 8 out of 6259 genes, 0.1% | 1 |
| protein deubiquitination | 8 out of 6178 genes, 0.1% | 8 out of 6259 genes, 0.1% | 1 |
| cell differentiation in hindbrain | 8 out of 6178 genes, 0.1% | 8 out of 6259 genes, 0.1% | 1 |
| cranial nerve development | 8 out of 6178 genes, 0.1% | 8 out of 6259 genes, 0.1% | 1 |
| molting cycle process | 8 out of 6178 genes, 0.1% | 8 out of 6259 genes, 0.1% | 1 |
| peptide hormone secretion | 8 out of 6178 genes, 0.1% | 8 out of 6259 genes, 0.1% | 1 |
| heparan sulfate proteoglycan metabolic process | 8 out of 6178 genes, 0.1% | 8 out of 6259 genes, 0.1% | 1 |
| activation of protein kinase activity | 8 out of 6178 genes, 0.1% | 8 out of 6259 genes, 0.1% | 1 |
| telomere organization | 8 out of 6178 genes, 0.1% | 8 out of 6259 genes, 0.1% | 1 |
| cellular carbohydrate biosynthetic process | 8 out of 6178 genes, 0.1% | 8 out of 6259 genes, 0.1% | 1 |
| histone lysine methylation | 8 out of 6178 genes, 0.1% | 8 out of 6259 genes, 0.1% | 1 |
| lamellocyte differentiation | 8 out of 6178 genes, 0.1% | 8 out of 6259 genes, 0.1% | 1 |
| hormone metabolic process | 8 out of 6178 genes, 0.1% | 8 out of 6259 genes, 0.1% | 1 |
| mechanoreceptor differentiation | 8 out of 6178 genes, 0.1% | 8 out of 6259 genes, 0.1% | 1 |
| positive regulation of transcription factor import into nucleus | 8 out of 6178 genes, 0.1% | 8 out of 6259 genes, 0.1% | 1 |
| intercellular bridge organization | 8 out of 6178 genes, 0.1% | 8 out of 6259 genes, 0.1% | 1 |
| regulation of protein complex assembly | 8 out of 6178 genes, 0.1% | 8 out of 6259 genes, 0.1% | 1 |
| regulation of lymphocyte differentiation | 8 out of 6178 genes, 0.1% | 8 out of 6259 genes, 0.1% | 1 |
| negative regulation of gene expression, epigenetic | 8 out of 6178 genes, 0.1% | 8 out of 6259 genes, 0.1% | 1 |
| phosphoinositide biosynthetic process | 8 out of 6178 genes, 0.1% | 8 out of 6259 genes, 0.1% | 1 |
| development of primary female sexual characteristics | 8 out of 6178 genes, 0.1% | 8 out of 6259 genes, 0.1% | 1 |
| female sex differentiation | 8 out of 6178 genes, 0.1% | 8 out of 6259 genes, 0.1% | 1 |
| regulation of neuronal synaptic plasticity | 8 out of 6178 genes, 0.1% | 8 out of 6259 genes, 0.1% | 1 |
| multicellular organismal homeostasis | 8 out of 6178 genes, 0.1% | 8 out of 6259 genes, 0.1% | 1 |
| antigen receptor-mediated signaling pathway | 8 out of 6178 genes, 0.1% | 8 out of 6259 genes, 0.1% | 1 |
| positive regulation of cell activation | 8 out of 6178 genes, 0.1% | 8 out of 6259 genes, 0.1% | 1 |
| regulation of blood vessel size | 8 out of 6178 genes, 0.1% | 8 out of 6259 genes, 0.1% | 1 |
| sterol homeostasis | 8 out of 6178 genes, 0.1% | 8 out of 6259 genes, 0.1% | 1 |
| male mating behavior | 8 out of 6178 genes, 0.1% | 8 out of 6259 genes, 0.1% | 1 |
| regulation of reproductive process | 8 out of 6178 genes, 0.1% | 8 out of 6259 genes, 0.1% | 1 |
| regulation of cell communication | 132 out of 6178 genes, 2.1% | 135 out of 6259 genes, 2.2% | 1 |
| exocytosis | 39 out of 6178 genes, 0.6% | 40 out of 6259 genes, 0.6% | 1 |
| locomotory behavior | 39 out of 6178 genes, 0.6% | 40 out of 6259 genes, 0.6% | 1 |
| nucleoside triphosphate metabolic process | 130 out of 6178 genes, 2.1% | 133 out of 6259 genes, 2.1% | 1 |
| gene silencing | 38 out of 6178 genes, 0.6% | 39 out of 6259 genes, 0.6% | 1 |
| regulation of protein ubiquitination | 38 out of 6178 genes, 0.6% | 39 out of 6259 genes, 0.6% | 1 |
| regulation of phosphate metabolic process | 80 out of 6178 genes, 1.3% | 82 out of 6259 genes, 1.3% | 1 |
| positive regulation of molecular function | 80 out of 6178 genes, 1.3% | 82 out of 6259 genes, 1.3% | 1 |
| regulation of phosphorus metabolic process | 80 out of 6178 genes, 1.3% | 82 out of 6259 genes, 1.3% | 1 |
| ribonucleoside triphosphate metabolic process | 127 out of 6178 genes, 2.1% | 130 out of 6259 genes, 2.1% | 1 |
| polysaccharide catabolic process | 7 out of 6178 genes, 0.1% | 7 out of 6259 genes, 0.1% | 1 |
| positive regulation of cytokine production | 7 out of 6178 genes, 0.1% | 7 out of 6259 genes, 0.1% | 1 |
| epithelial to mesenchymal transition | 7 out of 6178 genes, 0.1% | 7 out of 6259 genes, 0.1% | 1 |
| somatic recombination of immunoglobulin genes involved in immune response | 7 out of 6178 genes, 0.1% | 7 out of 6259 genes, 0.1% | 1 |
| somatic diversification of immunoglobulins involved in immune response | 7 out of 6178 genes, 0.1% | 7 out of 6259 genes, 0.1% | 1 |
| immunoglobulin production involved in immunoglobulin mediated immune response | 7 out of 6178 genes, 0.1% | 7 out of 6259 genes, 0.1% | 1 |
| heart process | 7 out of 6178 genes, 0.1% | 7 out of 6259 genes, 0.1% | 1 |
| RNA-dependent DNA replication | 7 out of 6178 genes, 0.1% | 7 out of 6259 genes, 0.1% | 1 |
| regulation of DNA repair | 7 out of 6178 genes, 0.1% | 7 out of 6259 genes, 0.1% | 1 |
| DNA catabolic process | 7 out of 6178 genes, 0.1% | 7 out of 6259 genes, 0.1% | 1 |
| DNA packaging | 7 out of 6178 genes, 0.1% | 7 out of 6259 genes, 0.1% | 1 |
| regulation of translational initiation | 7 out of 6178 genes, 0.1% | 7 out of 6259 genes, 0.1% | 1 |
| vesicle targeting | 7 out of 6178 genes, 0.1% | 7 out of 6259 genes, 0.1% | 1 |
| vesicle docking involved in exocytosis | 7 out of 6178 genes, 0.1% | 7 out of 6259 genes, 0.1% | 1 |
| regulation of muscle contraction | 7 out of 6178 genes, 0.1% | 7 out of 6259 genes, 0.1% | 1 |
| regulation of striated muscle contraction | 7 out of 6178 genes, 0.1% | 7 out of 6259 genes, 0.1% | 1 |
| smoothened signaling pathway | 7 out of 6178 genes, 0.1% | 7 out of 6259 genes, 0.1% | 1 |
| JAK-STAT cascade | 7 out of 6178 genes, 0.1% | 7 out of 6259 genes, 0.1% | 1 |
| mesoderm migration involved in gastrulation | 7 out of 6178 genes, 0.1% | 7 out of 6259 genes, 0.1% | 1 |
| body fluid secretion | 7 out of 6178 genes, 0.1% | 7 out of 6259 genes, 0.1% | 1 |
| phototransduction | 7 out of 6178 genes, 0.1% | 7 out of 6259 genes, 0.1% | 1 |
| chemosensory behavior | 7 out of 6178 genes, 0.1% | 7 out of 6259 genes, 0.1% | 1 |
| male courtship behavior | 7 out of 6178 genes, 0.1% | 7 out of 6259 genes, 0.1% | 1 |
| protein dealkylation | 7 out of 6178 genes, 0.1% | 7 out of 6259 genes, 0.1% | 1 |
| asymmetric cell division | 7 out of 6178 genes, 0.1% | 7 out of 6259 genes, 0.1% | 1 |
| fatty acid catabolic process | 7 out of 6178 genes, 0.1% | 7 out of 6259 genes, 0.1% | 1 |
| pyrimidine nucleoside monophosphate metabolic process | 7 out of 6178 genes, 0.1% | 7 out of 6259 genes, 0.1% | 1 |
| pyrimidine nucleoside monophosphate biosynthetic process | 7 out of 6178 genes, 0.1% | 7 out of 6259 genes, 0.1% | 1 |
| anterior/posterior axis specification | 7 out of 6178 genes, 0.1% | 7 out of 6259 genes, 0.1% | 1 |
| negative regulation of peptidase activity | 7 out of 6178 genes, 0.1% | 7 out of 6259 genes, 0.1% | 1 |
| regulation of cell-substrate adhesion | 7 out of 6178 genes, 0.1% | 7 out of 6259 genes, 0.1% | 1 |
| regulation of glucose transport | 7 out of 6178 genes, 0.1% | 7 out of 6259 genes, 0.1% | 1 |
| negative regulation of cell cycle process | 7 out of 6178 genes, 0.1% | 7 out of 6259 genes, 0.1% | 1 |
| monoamine transport | 7 out of 6178 genes, 0.1% | 7 out of 6259 genes, 0.1% | 1 |
| lipid catabolic process | 7 out of 6178 genes, 0.1% | 7 out of 6259 genes, 0.1% | 1 |
| regulation of exocytosis | 7 out of 6178 genes, 0.1% | 7 out of 6259 genes, 0.1% | 1 |
| NAD metabolic process | 7 out of 6178 genes, 0.1% | 7 out of 6259 genes, 0.1% | 1 |
| lipid modification | 7 out of 6178 genes, 0.1% | 7 out of 6259 genes, 0.1% | 1 |
| dsRNA fragmentation | 7 out of 6178 genes, 0.1% | 7 out of 6259 genes, 0.1% | 1 |
| positive regulation of cyclase activity | 7 out of 6178 genes, 0.1% | 7 out of 6259 genes, 0.1% | 1 |
| negative regulation of cell projection organization | 7 out of 6178 genes, 0.1% | 7 out of 6259 genes, 0.1% | 1 |
| DNA geometric change | 7 out of 6178 genes, 0.1% | 7 out of 6259 genes, 0.1% | 1 |
| regulation of transporter activity | 7 out of 6178 genes, 0.1% | 7 out of 6259 genes, 0.1% | 1 |
| tube fusion | 7 out of 6178 genes, 0.1% | 7 out of 6259 genes, 0.1% | 1 |
| regulation of tube size, open tracheal system | 7 out of 6178 genes, 0.1% | 7 out of 6259 genes, 0.1% | 1 |
| regulation of tube architecture, open tracheal system | 7 out of 6178 genes, 0.1% | 7 out of 6259 genes, 0.1% | 1 |
| production of miRNAs involved in gene silencing by miRNA | 7 out of 6178 genes, 0.1% | 7 out of 6259 genes, 0.1% | 1 |
| eye photoreceptor cell development | 7 out of 6178 genes, 0.1% | 7 out of 6259 genes, 0.1% | 1 |
| ear morphogenesis | 7 out of 6178 genes, 0.1% | 7 out of 6259 genes, 0.1% | 1 |
| neuron maturation | 7 out of 6178 genes, 0.1% | 7 out of 6259 genes, 0.1% | 1 |
| response to dsRNA | 7 out of 6178 genes, 0.1% | 7 out of 6259 genes, 0.1% | 1 |
| regulation of generation of precursor metabolites and energy | 7 out of 6178 genes, 0.1% | 7 out of 6259 genes, 0.1% | 1 |
| regulation of RNA stability | 7 out of 6178 genes, 0.1% | 7 out of 6259 genes, 0.1% | 1 |
| cellular lipid catabolic process | 7 out of 6178 genes, 0.1% | 7 out of 6259 genes, 0.1% | 1 |
| regulation of T cell differentiation | 7 out of 6178 genes, 0.1% | 7 out of 6259 genes, 0.1% | 1 |
| positive regulation of adenylate cyclase activity | 7 out of 6178 genes, 0.1% | 7 out of 6259 genes, 0.1% | 1 |
| positive regulation of endocytosis | 7 out of 6178 genes, 0.1% | 7 out of 6259 genes, 0.1% | 1 |
| sensory system development | 7 out of 6178 genes, 0.1% | 7 out of 6259 genes, 0.1% | 1 |
| lateral line system development | 7 out of 6178 genes, 0.1% | 7 out of 6259 genes, 0.1% | 1 |
| epithelial cell proliferation | 7 out of 6178 genes, 0.1% | 7 out of 6259 genes, 0.1% | 1 |
| multicellular organismal movement | 7 out of 6178 genes, 0.1% | 7 out of 6259 genes, 0.1% | 1 |
| musculoskeletal movement | 7 out of 6178 genes, 0.1% | 7 out of 6259 genes, 0.1% | 1 |
| 'de novo' posttranslational protein folding | 7 out of 6178 genes, 0.1% | 7 out of 6259 genes, 0.1% | 1 |
| positive regulation of lymphocyte activation | 7 out of 6178 genes, 0.1% | 7 out of 6259 genes, 0.1% | 1 |
| negative regulation of hydrolase activity | 7 out of 6178 genes, 0.1% | 7 out of 6259 genes, 0.1% | 1 |
| positive regulation of lyase activity | 7 out of 6178 genes, 0.1% | 7 out of 6259 genes, 0.1% | 1 |
| cytosolic calcium ion homeostasis | 7 out of 6178 genes, 0.1% | 7 out of 6259 genes, 0.1% | 1 |
| inner ear receptor cell differentiation | 7 out of 6178 genes, 0.1% | 7 out of 6259 genes, 0.1% | 1 |
| regulation of cell projection assembly | 7 out of 6178 genes, 0.1% | 7 out of 6259 genes, 0.1% | 1 |
| production of small RNA involved in gene silencing by RNA | 7 out of 6178 genes, 0.1% | 7 out of 6259 genes, 0.1% | 1 |
| protein localization in endoplasmic reticulum | 7 out of 6178 genes, 0.1% | 7 out of 6259 genes, 0.1% | 1 |
| cellular response to dsRNA | 7 out of 6178 genes, 0.1% | 7 out of 6259 genes, 0.1% | 1 |
| protein-DNA complex subunit organization | 7 out of 6178 genes, 0.1% | 7 out of 6259 genes, 0.1% | 1 |
| monocarboxylic acid catabolic process | 7 out of 6178 genes, 0.1% | 7 out of 6259 genes, 0.1% | 1 |
| regulation of muscle system process | 7 out of 6178 genes, 0.1% | 7 out of 6259 genes, 0.1% | 1 |
| nucleic acid phosphodiester bond hydrolysis | 7 out of 6178 genes, 0.1% | 7 out of 6259 genes, 0.1% | 1 |
| regulation of ion homeostasis | 7 out of 6178 genes, 0.1% | 7 out of 6259 genes, 0.1% | 1 |
| immune system development | 79 out of 6178 genes, 1.3% | 81 out of 6259 genes, 1.3% | 1 |
| negative regulation of biosynthetic process | 37 out of 6178 genes, 0.6% | 38 out of 6259 genes, 0.6% | 1 |
| negative regulation of cellular biosynthetic process | 37 out of 6178 genes, 0.6% | 38 out of 6259 genes, 0.6% | 1 |
| rhythmic process | 37 out of 6178 genes, 0.6% | 38 out of 6259 genes, 0.6% | 1 |
| regulation of ligase activity | 37 out of 6178 genes, 0.6% | 38 out of 6259 genes, 0.6% | 1 |
| regulation of ubiquitin-protein ligase activity | 37 out of 6178 genes, 0.6% | 38 out of 6259 genes, 0.6% | 1 |
| organic acid transport | 36 out of 6178 genes, 0.6% | 37 out of 6259 genes, 0.6% | 1 |
| regulation of cell proliferation | 36 out of 6178 genes, 0.6% | 37 out of 6259 genes, 0.6% | 1 |
| carboxylic acid transport | 36 out of 6178 genes, 0.6% | 37 out of 6259 genes, 0.6% | 1 |
| regulation of ubiquitin-protein ligase activity involved in mitotic cell cycle | 36 out of 6178 genes, 0.6% | 37 out of 6259 genes, 0.6% | 1 |
| regulation of phosphorylation | 77 out of 6178 genes, 1.2% | 79 out of 6259 genes, 1.3% | 1 |
| small molecule biosynthetic process | 174 out of 6178 genes, 2.8% | 178 out of 6259 genes, 2.8% | 1 |
| regulation of intracellular protein kinase cascade | 35 out of 6178 genes, 0.6% | 36 out of 6259 genes, 0.6% | 1 |
| second-messenger-mediated signaling | 35 out of 6178 genes, 0.6% | 36 out of 6259 genes, 0.6% | 1 |
| methylation | 35 out of 6178 genes, 0.6% | 36 out of 6259 genes, 0.6% | 1 |
| purine nucleoside triphosphate metabolic process | 122 out of 6178 genes, 2.0% | 125 out of 6259 genes, 2.0% | 1 |
| purine ribonucleoside triphosphate metabolic process | 122 out of 6178 genes, 2.0% | 125 out of 6259 genes, 2.0% | 1 |
| regulation of catalytic activity | 172 out of 6178 genes, 2.8% | 176 out of 6259 genes, 2.8% | 1 |
| non-recombinational repair | 6 out of 6178 genes, 0.1% | 6 out of 6259 genes, 0.1% | 1 |
| DNA catabolic process, endonucleolytic | 6 out of 6178 genes, 0.1% | 6 out of 6259 genes, 0.1% | 1 |
| somatic diversification of immune receptors via somatic mutation | 6 out of 6178 genes, 0.1% | 6 out of 6259 genes, 0.1% | 1 |
| skeletal muscle contraction | 6 out of 6178 genes, 0.1% | 6 out of 6259 genes, 0.1% | 1 |
| fructose metabolic process | 6 out of 6178 genes, 0.1% | 6 out of 6259 genes, 0.1% | 1 |
| DNA strand elongation involved in DNA replication | 6 out of 6178 genes, 0.1% | 6 out of 6259 genes, 0.1% | 1 |
| regulation of DNA replication | 6 out of 6178 genes, 0.1% | 6 out of 6259 genes, 0.1% | 1 |
| tRNA modification | 6 out of 6178 genes, 0.1% | 6 out of 6259 genes, 0.1% | 1 |
| protein demethylation | 6 out of 6178 genes, 0.1% | 6 out of 6259 genes, 0.1% | 1 |
| glycosphingolipid metabolic process | 6 out of 6178 genes, 0.1% | 6 out of 6259 genes, 0.1% | 1 |
| ubiquinone metabolic process | 6 out of 6178 genes, 0.1% | 6 out of 6259 genes, 0.1% | 1 |
| vesicle fusion | 6 out of 6178 genes, 0.1% | 6 out of 6259 genes, 0.1% | 1 |
| response to osmotic stress | 6 out of 6178 genes, 0.1% | 6 out of 6259 genes, 0.1% | 1 |
| cytoplasm organization | 6 out of 6178 genes, 0.1% | 6 out of 6259 genes, 0.1% | 1 |
| regulation of mitosis | 6 out of 6178 genes, 0.1% | 6 out of 6259 genes, 0.1% | 1 |
| glutamate signaling pathway | 6 out of 6178 genes, 0.1% | 6 out of 6259 genes, 0.1% | 1 |
| tripartite regional subdivision | 6 out of 6178 genes, 0.1% | 6 out of 6259 genes, 0.1% | 1 |
| visual perception | 6 out of 6178 genes, 0.1% | 6 out of 6259 genes, 0.1% | 1 |
| phototransduction, visible light | 6 out of 6178 genes, 0.1% | 6 out of 6259 genes, 0.1% | 1 |
| axon cargo transport | 6 out of 6178 genes, 0.1% | 6 out of 6259 genes, 0.1% | 1 |
| anterior/posterior axis specification, embryo | 6 out of 6178 genes, 0.1% | 6 out of 6259 genes, 0.1% | 1 |
| apoptotic mitochondrial changes | 6 out of 6178 genes, 0.1% | 6 out of 6259 genes, 0.1% | 1 |
| pyrimidine nucleoside triphosphate biosynthetic process | 6 out of 6178 genes, 0.1% | 6 out of 6259 genes, 0.1% | 1 |
| pyrimidine ribonucleoside triphosphate metabolic process | 6 out of 6178 genes, 0.1% | 6 out of 6259 genes, 0.1% | 1 |
| pyrimidine ribonucleoside triphosphate biosynthetic process | 6 out of 6178 genes, 0.1% | 6 out of 6259 genes, 0.1% | 1 |
| pyrimidine deoxyribonucleotide metabolic process | 6 out of 6178 genes, 0.1% | 6 out of 6259 genes, 0.1% | 1 |
| response to heat | 6 out of 6178 genes, 0.1% | 6 out of 6259 genes, 0.1% | 1 |
| NADH dehydrogenase complex assembly | 6 out of 6178 genes, 0.1% | 6 out of 6259 genes, 0.1% | 1 |
| regulation of cellular ketone metabolic process | 6 out of 6178 genes, 0.1% | 6 out of 6259 genes, 0.1% | 1 |
| positive regulation of organelle organization | 6 out of 6178 genes, 0.1% | 6 out of 6259 genes, 0.1% | 1 |
| negative regulation of organelle organization | 6 out of 6178 genes, 0.1% | 6 out of 6259 genes, 0.1% | 1 |
| negative regulation of intracellular protein kinase cascade | 6 out of 6178 genes, 0.1% | 6 out of 6259 genes, 0.1% | 1 |
| regulation of gliogenesis | 6 out of 6178 genes, 0.1% | 6 out of 6259 genes, 0.1% | 1 |
| neutral amino acid transport | 6 out of 6178 genes, 0.1% | 6 out of 6259 genes, 0.1% | 1 |
| sterol transport | 6 out of 6178 genes, 0.1% | 6 out of 6259 genes, 0.1% | 1 |
| histone ubiquitination | 6 out of 6178 genes, 0.1% | 6 out of 6259 genes, 0.1% | 1 |
| histone demethylation | 6 out of 6178 genes, 0.1% | 6 out of 6259 genes, 0.1% | 1 |
| regulation of fatty acid metabolic process | 6 out of 6178 genes, 0.1% | 6 out of 6259 genes, 0.1% | 1 |
| secondary metabolic process | 6 out of 6178 genes, 0.1% | 6 out of 6259 genes, 0.1% | 1 |
| cell differentiation in spinal cord | 6 out of 6178 genes, 0.1% | 6 out of 6259 genes, 0.1% | 1 |
| hair cycle process | 6 out of 6178 genes, 0.1% | 6 out of 6259 genes, 0.1% | 1 |
| DNA strand elongation | 6 out of 6178 genes, 0.1% | 6 out of 6259 genes, 0.1% | 1 |
| regulation of transmembrane transporter activity | 6 out of 6178 genes, 0.1% | 6 out of 6259 genes, 0.1% | 1 |
| myeloid cell differentiation | 6 out of 6178 genes, 0.1% | 6 out of 6259 genes, 0.1% | 1 |
| myofibril assembly | 6 out of 6178 genes, 0.1% | 6 out of 6259 genes, 0.1% | 1 |
| cholesterol transport | 6 out of 6178 genes, 0.1% | 6 out of 6259 genes, 0.1% | 1 |
| biomineral tissue development | 6 out of 6178 genes, 0.1% | 6 out of 6259 genes, 0.1% | 1 |
| negative regulation of cellular catabolic process | 6 out of 6178 genes, 0.1% | 6 out of 6259 genes, 0.1% | 1 |
| positive regulation of defense response | 6 out of 6178 genes, 0.1% | 6 out of 6259 genes, 0.1% | 1 |
| positive regulation of protein ubiquitination | 6 out of 6178 genes, 0.1% | 6 out of 6259 genes, 0.1% | 1 |
| cellular response to nutrient | 6 out of 6178 genes, 0.1% | 6 out of 6259 genes, 0.1% | 1 |
| regulation of ion transmembrane transporter activity | 6 out of 6178 genes, 0.1% | 6 out of 6259 genes, 0.1% | 1 |
| carbohydrate homeostasis | 6 out of 6178 genes, 0.1% | 6 out of 6259 genes, 0.1% | 1 |
| response to lipid | 6 out of 6178 genes, 0.1% | 6 out of 6259 genes, 0.1% | 1 |
| cellular hormone metabolic process | 6 out of 6178 genes, 0.1% | 6 out of 6259 genes, 0.1% | 1 |
| regulation of ion transmembrane transport | 6 out of 6178 genes, 0.1% | 6 out of 6259 genes, 0.1% | 1 |
| compound eye photoreceptor development | 6 out of 6178 genes, 0.1% | 6 out of 6259 genes, 0.1% | 1 |
| quinone cofactor metabolic process | 6 out of 6178 genes, 0.1% | 6 out of 6259 genes, 0.1% | 1 |
| myelination | 6 out of 6178 genes, 0.1% | 6 out of 6259 genes, 0.1% | 1 |
| hair cycle | 6 out of 6178 genes, 0.1% | 6 out of 6259 genes, 0.1% | 1 |
| negative regulation of MAPKKK cascade | 6 out of 6178 genes, 0.1% | 6 out of 6259 genes, 0.1% | 1 |
| regulation of mRNA stability | 6 out of 6178 genes, 0.1% | 6 out of 6259 genes, 0.1% | 1 |
| fat cell differentiation | 6 out of 6178 genes, 0.1% | 6 out of 6259 genes, 0.1% | 1 |
| positive regulation of T cell differentiation | 6 out of 6178 genes, 0.1% | 6 out of 6259 genes, 0.1% | 1 |
| positive regulation of lymphocyte differentiation | 6 out of 6178 genes, 0.1% | 6 out of 6259 genes, 0.1% | 1 |
| organelle fusion | 6 out of 6178 genes, 0.1% | 6 out of 6259 genes, 0.1% | 1 |
| positive regulation of viral reproduction | 6 out of 6178 genes, 0.1% | 6 out of 6259 genes, 0.1% | 1 |
| neuron fate commitment | 6 out of 6178 genes, 0.1% | 6 out of 6259 genes, 0.1% | 1 |
| embryonic skeletal system morphogenesis | 6 out of 6178 genes, 0.1% | 6 out of 6259 genes, 0.1% | 1 |
| skeletal system morphogenesis | 6 out of 6178 genes, 0.1% | 6 out of 6259 genes, 0.1% | 1 |
| embryonic skeletal system development | 6 out of 6178 genes, 0.1% | 6 out of 6259 genes, 0.1% | 1 |
| regulation of viral reproduction | 6 out of 6178 genes, 0.1% | 6 out of 6259 genes, 0.1% | 1 |
| regulation of B cell activation | 6 out of 6178 genes, 0.1% | 6 out of 6259 genes, 0.1% | 1 |
| positive regulation of T cell activation | 6 out of 6178 genes, 0.1% | 6 out of 6259 genes, 0.1% | 1 |
| pigment cell differentiation | 6 out of 6178 genes, 0.1% | 6 out of 6259 genes, 0.1% | 1 |
| interphase of mitotic cell cycle | 6 out of 6178 genes, 0.1% | 6 out of 6259 genes, 0.1% | 1 |
| regulation of nuclear division | 6 out of 6178 genes, 0.1% | 6 out of 6259 genes, 0.1% | 1 |
| response to protein stimulus | 6 out of 6178 genes, 0.1% | 6 out of 6259 genes, 0.1% | 1 |
| regulation of lipase activity | 6 out of 6178 genes, 0.1% | 6 out of 6259 genes, 0.1% | 1 |
| morphogenesis of a branching epithelium | 6 out of 6178 genes, 0.1% | 6 out of 6259 genes, 0.1% | 1 |
| protein-DNA complex assembly | 6 out of 6178 genes, 0.1% | 6 out of 6259 genes, 0.1% | 1 |
| response to growth factor stimulus | 6 out of 6178 genes, 0.1% | 6 out of 6259 genes, 0.1% | 1 |
| demethylation | 6 out of 6178 genes, 0.1% | 6 out of 6259 genes, 0.1% | 1 |
| DNA biosynthetic process | 6 out of 6178 genes, 0.1% | 6 out of 6259 genes, 0.1% | 1 |
| macromolecule methylation | 34 out of 6178 genes, 0.6% | 35 out of 6259 genes, 0.6% | 1 |
| positive regulation of transport | 34 out of 6178 genes, 0.6% | 35 out of 6259 genes, 0.6% | 1 |
| Ras protein signal transduction | 33 out of 6178 genes, 0.5% | 34 out of 6259 genes, 0.5% | 1 |
| positive regulation of apoptosis | 33 out of 6178 genes, 0.5% | 34 out of 6259 genes, 0.5% | 1 |
| purine nucleotide biosynthetic process | 115 out of 6178 genes, 1.9% | 118 out of 6259 genes, 1.9% | 1 |
| cell proliferation | 115 out of 6178 genes, 1.9% | 118 out of 6259 genes, 1.9% | 1 |
| anion transport | 31 out of 6178 genes, 0.5% | 32 out of 6259 genes, 0.5% | 1 |
| regulation of intracellular transport | 31 out of 6178 genes, 0.5% | 32 out of 6259 genes, 0.5% | 1 |
| negative regulation of catalytic activity | 31 out of 6178 genes, 0.5% | 32 out of 6259 genes, 0.5% | 1 |
| cellular response to chemical stimulus | 31 out of 6178 genes, 0.5% | 32 out of 6259 genes, 0.5% | 1 |
| protein import into nucleus, translocation | 5 out of 6178 genes, 0.1% | 5 out of 6259 genes, 0.1% | 1 |
| sulfur amino acid transport | 5 out of 6178 genes, 0.1% | 5 out of 6259 genes, 0.1% | 1 |
| spliceosome assembly | 5 out of 6178 genes, 0.1% | 5 out of 6259 genes, 0.1% | 1 |
| compound eye photoreceptor fate commitment | 5 out of 6178 genes, 0.1% | 5 out of 6259 genes, 0.1% | 1 |
| cytokine production | 5 out of 6178 genes, 0.1% | 5 out of 6259 genes, 0.1% | 1 |
| blastocyst formation | 5 out of 6178 genes, 0.1% | 5 out of 6259 genes, 0.1% | 1 |
| neural tube formation | 5 out of 6178 genes, 0.1% | 5 out of 6259 genes, 0.1% | 1 |
| tissue homeostasis | 5 out of 6178 genes, 0.1% | 5 out of 6259 genes, 0.1% | 1 |
| cell killing | 5 out of 6178 genes, 0.1% | 5 out of 6259 genes, 0.1% | 1 |
| endothelial cell proliferation | 5 out of 6178 genes, 0.1% | 5 out of 6259 genes, 0.1% | 1 |
| cell activation involved in immune response | 5 out of 6178 genes, 0.1% | 5 out of 6259 genes, 0.1% | 1 |
| leukocyte activation involved in immune response | 5 out of 6178 genes, 0.1% | 5 out of 6259 genes, 0.1% | 1 |
| negative regulation of immune system process | 5 out of 6178 genes, 0.1% | 5 out of 6259 genes, 0.1% | 1 |
| glycogen biosynthetic process | 5 out of 6178 genes, 0.1% | 5 out of 6259 genes, 0.1% | 1 |
| mannose metabolic process | 5 out of 6178 genes, 0.1% | 5 out of 6259 genes, 0.1% | 1 |
| glycosaminoglycan biosynthetic process | 5 out of 6178 genes, 0.1% | 5 out of 6259 genes, 0.1% | 1 |
| acetyl-CoA biosynthetic process | 5 out of 6178 genes, 0.1% | 5 out of 6259 genes, 0.1% | 1 |
| pentose-phosphate shunt | 5 out of 6178 genes, 0.1% | 5 out of 6259 genes, 0.1% | 1 |
| regulation of carbohydrate metabolic process | 5 out of 6178 genes, 0.1% | 5 out of 6259 genes, 0.1% | 1 |
| pyrimidine nucleoside metabolic process | 5 out of 6178 genes, 0.1% | 5 out of 6259 genes, 0.1% | 1 |
| DNA amplification | 5 out of 6178 genes, 0.1% | 5 out of 6259 genes, 0.1% | 1 |
| transcription from RNA polymerase II promoter | 5 out of 6178 genes, 0.1% | 5 out of 6259 genes, 0.1% | 1 |
| cotranslational protein targeting to membrane | 5 out of 6178 genes, 0.1% | 5 out of 6259 genes, 0.1% | 1 |
| fatty acid beta-oxidation | 5 out of 6178 genes, 0.1% | 5 out of 6259 genes, 0.1% | 1 |
| NADP metabolic process | 5 out of 6178 genes, 0.1% | 5 out of 6259 genes, 0.1% | 1 |
| NADPH regeneration | 5 out of 6178 genes, 0.1% | 5 out of 6259 genes, 0.1% | 1 |
| sodium ion transport | 5 out of 6178 genes, 0.1% | 5 out of 6259 genes, 0.1% | 1 |
| lysosomal transport | 5 out of 6178 genes, 0.1% | 5 out of 6259 genes, 0.1% | 1 |
| negative regulation of cell adhesion | 5 out of 6178 genes, 0.1% | 5 out of 6259 genes, 0.1% | 1 |
| activation of adenylate cyclase activity | 5 out of 6178 genes, 0.1% | 5 out of 6259 genes, 0.1% | 1 |
| tyrosine phosphorylation of STAT protein | 5 out of 6178 genes, 0.1% | 5 out of 6259 genes, 0.1% | 1 |
| spermatogenesis | 5 out of 6178 genes, 0.1% | 5 out of 6259 genes, 0.1% | 1 |
| oocyte anterior/posterior axis specification | 5 out of 6178 genes, 0.1% | 5 out of 6259 genes, 0.1% | 1 |
| pole plasm assembly | 5 out of 6178 genes, 0.1% | 5 out of 6259 genes, 0.1% | 1 |
| pole plasm RNA localization | 5 out of 6178 genes, 0.1% | 5 out of 6259 genes, 0.1% | 1 |
| periodic partitioning | 5 out of 6178 genes, 0.1% | 5 out of 6259 genes, 0.1% | 1 |
| initiation of dorsal closure | 5 out of 6178 genes, 0.1% | 5 out of 6259 genes, 0.1% | 1 |
| neuroblast proliferation | 5 out of 6178 genes, 0.1% | 5 out of 6259 genes, 0.1% | 1 |
| salivary gland morphogenesis | 5 out of 6178 genes, 0.1% | 5 out of 6259 genes, 0.1% | 1 |
| leg disc morphogenesis | 5 out of 6178 genes, 0.1% | 5 out of 6259 genes, 0.1% | 1 |
| histolysis | 5 out of 6178 genes, 0.1% | 5 out of 6259 genes, 0.1% | 1 |
| intracellular mRNA localization | 5 out of 6178 genes, 0.1% | 5 out of 6259 genes, 0.1% | 1 |
| maternal determination of anterior/posterior axis, embryo | 5 out of 6178 genes, 0.1% | 5 out of 6259 genes, 0.1% | 1 |
| aspartate family amino acid metabolic process | 5 out of 6178 genes, 0.1% | 5 out of 6259 genes, 0.1% | 1 |
| serine family amino acid metabolic process | 5 out of 6178 genes, 0.1% | 5 out of 6259 genes, 0.1% | 1 |
| purine base biosynthetic process | 5 out of 6178 genes, 0.1% | 5 out of 6259 genes, 0.1% | 1 |
| deoxyribonucleoside triphosphate metabolic process | 5 out of 6178 genes, 0.1% | 5 out of 6259 genes, 0.1% | 1 |
| pyrimidine deoxyribonucleotide biosynthetic process | 5 out of 6178 genes, 0.1% | 5 out of 6259 genes, 0.1% | 1 |
| glucan biosynthetic process | 5 out of 6178 genes, 0.1% | 5 out of 6259 genes, 0.1% | 1 |
| deoxyribonucleotide biosynthetic process | 5 out of 6178 genes, 0.1% | 5 out of 6259 genes, 0.1% | 1 |
| 2'-deoxyribonucleotide biosynthetic process | 5 out of 6178 genes, 0.1% | 5 out of 6259 genes, 0.1% | 1 |
| rhodopsin mediated phototransduction | 5 out of 6178 genes, 0.1% | 5 out of 6259 genes, 0.1% | 1 |
| detection of chemical stimulus | 5 out of 6178 genes, 0.1% | 5 out of 6259 genes, 0.1% | 1 |
| gastrulation involving germ band extension | 5 out of 6178 genes, 0.1% | 5 out of 6259 genes, 0.1% | 1 |
| regulation of autophagy | 5 out of 6178 genes, 0.1% | 5 out of 6259 genes, 0.1% | 1 |
| negative regulation of autophagy | 5 out of 6178 genes, 0.1% | 5 out of 6259 genes, 0.1% | 1 |
| regulation of phospholipase activity | 5 out of 6178 genes, 0.1% | 5 out of 6259 genes, 0.1% | 1 |
| regulation of cellular carbohydrate metabolic process | 5 out of 6178 genes, 0.1% | 5 out of 6259 genes, 0.1% | 1 |
| positive regulation of cell development | 5 out of 6178 genes, 0.1% | 5 out of 6259 genes, 0.1% | 1 |
| regulation of glucose metabolic process | 5 out of 6178 genes, 0.1% | 5 out of 6259 genes, 0.1% | 1 |
| primary neural tube formation | 5 out of 6178 genes, 0.1% | 5 out of 6259 genes, 0.1% | 1 |
| organic cation transport | 5 out of 6178 genes, 0.1% | 5 out of 6259 genes, 0.1% | 1 |
| energy coupled proton transport, against electrochemical gradient | 5 out of 6178 genes, 0.1% | 5 out of 6259 genes, 0.1% | 1 |
| snRNA metabolic process | 5 out of 6178 genes, 0.1% | 5 out of 6259 genes, 0.1% | 1 |
| tissue death | 5 out of 6178 genes, 0.1% | 5 out of 6259 genes, 0.1% | 1 |
| peptidyl-tyrosine phosphorylation | 5 out of 6178 genes, 0.1% | 5 out of 6259 genes, 0.1% | 1 |
| peptidyl-tyrosine modification | 5 out of 6178 genes, 0.1% | 5 out of 6259 genes, 0.1% | 1 |
| viral infectious cycle | 5 out of 6178 genes, 0.1% | 5 out of 6259 genes, 0.1% | 1 |
| pole plasm mRNA localization | 5 out of 6178 genes, 0.1% | 5 out of 6259 genes, 0.1% | 1 |
| fatty acid oxidation | 5 out of 6178 genes, 0.1% | 5 out of 6259 genes, 0.1% | 1 |
| propionate metabolic process | 5 out of 6178 genes, 0.1% | 5 out of 6259 genes, 0.1% | 1 |
| neural tube development | 5 out of 6178 genes, 0.1% | 5 out of 6259 genes, 0.1% | 1 |
| gland morphogenesis | 5 out of 6178 genes, 0.1% | 5 out of 6259 genes, 0.1% | 1 |
| glycosaminoglycan metabolic process | 5 out of 6178 genes, 0.1% | 5 out of 6259 genes, 0.1% | 1 |
| BMP signaling pathway | 5 out of 6178 genes, 0.1% | 5 out of 6259 genes, 0.1% | 1 |
| regeneration | 5 out of 6178 genes, 0.1% | 5 out of 6259 genes, 0.1% | 1 |
| positive regulation of protein complex assembly | 5 out of 6178 genes, 0.1% | 5 out of 6259 genes, 0.1% | 1 |
| N-terminal protein amino acid modification | 5 out of 6178 genes, 0.1% | 5 out of 6259 genes, 0.1% | 1 |
| regulation of ARF protein signal transduction | 5 out of 6178 genes, 0.1% | 5 out of 6259 genes, 0.1% | 1 |
| regulation of response to external stimulus | 5 out of 6178 genes, 0.1% | 5 out of 6259 genes, 0.1% | 1 |
| regulation of synaptic transmission, GABAergic | 5 out of 6178 genes, 0.1% | 5 out of 6259 genes, 0.1% | 1 |
| negative regulation of cellular protein metabolic process | 5 out of 6178 genes, 0.1% | 5 out of 6259 genes, 0.1% | 1 |
| regulation of monooxygenase activity | 5 out of 6178 genes, 0.1% | 5 out of 6259 genes, 0.1% | 1 |
| cellular response to hormone stimulus | 5 out of 6178 genes, 0.1% | 5 out of 6259 genes, 0.1% | 1 |
| cellular pigmentation | 5 out of 6178 genes, 0.1% | 5 out of 6259 genes, 0.1% | 1 |
| nucleotide-excision repair, DNA incision | 5 out of 6178 genes, 0.1% | 5 out of 6259 genes, 0.1% | 1 |
| cellular polysaccharide biosynthetic process | 5 out of 6178 genes, 0.1% | 5 out of 6259 genes, 0.1% | 1 |
| lipid oxidation | 5 out of 6178 genes, 0.1% | 5 out of 6259 genes, 0.1% | 1 |
| protein localization to chromosome | 5 out of 6178 genes, 0.1% | 5 out of 6259 genes, 0.1% | 1 |
| cellular response to oxidative stress | 5 out of 6178 genes, 0.1% | 5 out of 6259 genes, 0.1% | 1 |
| cellular response to reactive oxygen species | 5 out of 6178 genes, 0.1% | 5 out of 6259 genes, 0.1% | 1 |
| cardiac cell differentiation | 5 out of 6178 genes, 0.1% | 5 out of 6259 genes, 0.1% | 1 |
| salivary gland histolysis | 5 out of 6178 genes, 0.1% | 5 out of 6259 genes, 0.1% | 1 |
| salivary gland cell autophagic cell death | 5 out of 6178 genes, 0.1% | 5 out of 6259 genes, 0.1% | 1 |
| leg morphogenesis | 5 out of 6178 genes, 0.1% | 5 out of 6259 genes, 0.1% | 1 |
| leg disc development | 5 out of 6178 genes, 0.1% | 5 out of 6259 genes, 0.1% | 1 |
| cell migration involved in gastrulation | 5 out of 6178 genes, 0.1% | 5 out of 6259 genes, 0.1% | 1 |
| neurotransmitter metabolic process | 5 out of 6178 genes, 0.1% | 5 out of 6259 genes, 0.1% | 1 |
| odontogenesis | 5 out of 6178 genes, 0.1% | 5 out of 6259 genes, 0.1% | 1 |
| eye photoreceptor cell fate commitment | 5 out of 6178 genes, 0.1% | 5 out of 6259 genes, 0.1% | 1 |
| hydrogen peroxide metabolic process | 5 out of 6178 genes, 0.1% | 5 out of 6259 genes, 0.1% | 1 |
| positive regulation of MAP kinase activity | 5 out of 6178 genes, 0.1% | 5 out of 6259 genes, 0.1% | 1 |
| cell-cell signaling involved in cell fate commitment | 5 out of 6178 genes, 0.1% | 5 out of 6259 genes, 0.1% | 1 |
| inosine metabolic process | 5 out of 6178 genes, 0.1% | 5 out of 6259 genes, 0.1% | 1 |
| pyrimidine deoxyribonucleoside metabolic process | 5 out of 6178 genes, 0.1% | 5 out of 6259 genes, 0.1% | 1 |
| photoreceptor cell fate commitment | 5 out of 6178 genes, 0.1% | 5 out of 6259 genes, 0.1% | 1 |
| regulation of developmental pigmentation | 5 out of 6178 genes, 0.1% | 5 out of 6259 genes, 0.1% | 1 |
| homeostasis of number of cells | 5 out of 6178 genes, 0.1% | 5 out of 6259 genes, 0.1% | 1 |
| detection of light stimulus involved in visual perception | 5 out of 6178 genes, 0.1% | 5 out of 6259 genes, 0.1% | 1 |
| detection of light stimulus involved in sensory perception | 5 out of 6178 genes, 0.1% | 5 out of 6259 genes, 0.1% | 1 |
| negative regulation of multicellular organismal process | 5 out of 6178 genes, 0.1% | 5 out of 6259 genes, 0.1% | 1 |
| negative regulation of protein metabolic process | 5 out of 6178 genes, 0.1% | 5 out of 6259 genes, 0.1% | 1 |
| protein tetramerization | 5 out of 6178 genes, 0.1% | 5 out of 6259 genes, 0.1% | 1 |
| centrosome organization | 5 out of 6178 genes, 0.1% | 5 out of 6259 genes, 0.1% | 1 |
| regulation of oxidoreductase activity | 5 out of 6178 genes, 0.1% | 5 out of 6259 genes, 0.1% | 1 |
| protein maturation by peptide bond cleavage | 5 out of 6178 genes, 0.1% | 5 out of 6259 genes, 0.1% | 1 |
| pigment granule localization | 5 out of 6178 genes, 0.1% | 5 out of 6259 genes, 0.1% | 1 |
| membrane depolarization | 5 out of 6178 genes, 0.1% | 5 out of 6259 genes, 0.1% | 1 |
| developmental growth involved in morphogenesis | 5 out of 6178 genes, 0.1% | 5 out of 6259 genes, 0.1% | 1 |
| intracellular mRNA localization involved in pattern specification process | 5 out of 6178 genes, 0.1% | 5 out of 6259 genes, 0.1% | 1 |
| intracellular mRNA localization involved in anterior/posterior axis specification | 5 out of 6178 genes, 0.1% | 5 out of 6259 genes, 0.1% | 1 |
| neural precursor cell proliferation | 5 out of 6178 genes, 0.1% | 5 out of 6259 genes, 0.1% | 1 |
| organelle assembly | 5 out of 6178 genes, 0.1% | 5 out of 6259 genes, 0.1% | 1 |
| cellular response to endogenous stimulus | 5 out of 6178 genes, 0.1% | 5 out of 6259 genes, 0.1% | 1 |
| positive regulation of protein serine/threonine kinase activity | 5 out of 6178 genes, 0.1% | 5 out of 6259 genes, 0.1% | 1 |
| sulfur compound transport | 5 out of 6178 genes, 0.1% | 5 out of 6259 genes, 0.1% | 1 |
| positive regulation of cellular process | 213 out of 6178 genes, 3.4% | 218 out of 6259 genes, 3.5% | 1 |
| glycogen metabolic process | 30 out of 6178 genes, 0.5% | 31 out of 6259 genes, 0.5% | 1 |
| cellular glucan metabolic process | 30 out of 6178 genes, 0.5% | 31 out of 6259 genes, 0.5% | 1 |
| energy reserve metabolic process | 30 out of 6178 genes, 0.5% | 31 out of 6259 genes, 0.5% | 1 |
| membrane lipid metabolic process | 30 out of 6178 genes, 0.5% | 31 out of 6259 genes, 0.5% | 1 |
| response to oxidative stress | 30 out of 6178 genes, 0.5% | 31 out of 6259 genes, 0.5% | 1 |
| nucleosome organization | 30 out of 6178 genes, 0.5% | 31 out of 6259 genes, 0.5% | 1 |
| glucan metabolic process | 30 out of 6178 genes, 0.5% | 31 out of 6259 genes, 0.5% | 1 |
| cellular polysaccharide metabolic process | 30 out of 6178 genes, 0.5% | 31 out of 6259 genes, 0.5% | 1 |
| regulation of molecular function | 211 out of 6178 genes, 3.4% | 216 out of 6259 genes, 3.5% | 1 |
| phagocytosis | 67 out of 6178 genes, 1.1% | 69 out of 6259 genes, 1.1% | 1 |
| di-, tri-valent inorganic cation transport | 67 out of 6178 genes, 1.1% | 69 out of 6259 genes, 1.1% | 1 |
| sphingolipid metabolic process | 28 out of 6178 genes, 0.5% | 29 out of 6259 genes, 0.5% | 1 |
| induction of apoptosis | 28 out of 6178 genes, 0.5% | 29 out of 6259 genes, 0.5% | 1 |
| intracellular protein kinase cascade | 64 out of 6178 genes, 1.0% | 66 out of 6259 genes, 1.1% | 1 |
| nucleobase, nucleoside and nucleotide biosynthetic process | 154 out of 6178 genes, 2.5% | 158 out of 6259 genes, 2.5% | 1 |
| nucleobase, nucleoside, nucleotide and nucleic acid biosynthetic process | 154 out of 6178 genes, 2.5% | 158 out of 6259 genes, 2.5% | 1 |
| hemopoietic or lymphoid organ development | 63 out of 6178 genes, 1.0% | 65 out of 6259 genes, 1.0% | 1 |
| activation of MAPK activity | 4 out of 6178 genes, 0.1% | 4 out of 6259 genes, 0.1% | 1 |
| assembly of actomyosin apparatus involved in cell cycle cytokinesis | 4 out of 6178 genes, 0.1% | 4 out of 6259 genes, 0.1% | 1 |
| regulation of cell-matrix adhesion | 4 out of 6178 genes, 0.1% | 4 out of 6259 genes, 0.1% | 1 |
| tRNA wobble base modification | 4 out of 6178 genes, 0.1% | 4 out of 6259 genes, 0.1% | 1 |
| aggressive behavior | 4 out of 6178 genes, 0.1% | 4 out of 6259 genes, 0.1% | 1 |
| lymphocyte activation involved in immune response | 4 out of 6178 genes, 0.1% | 4 out of 6259 genes, 0.1% | 1 |
| negative regulation of leukocyte activation | 4 out of 6178 genes, 0.1% | 4 out of 6259 genes, 0.1% | 1 |
| positive regulation of immune effector process | 4 out of 6178 genes, 0.1% | 4 out of 6259 genes, 0.1% | 1 |
| voluntary skeletal muscle contraction | 4 out of 6178 genes, 0.1% | 4 out of 6259 genes, 0.1% | 1 |
| endothelium development | 4 out of 6178 genes, 0.1% | 4 out of 6259 genes, 0.1% | 1 |
| N-acetylglucosamine metabolic process | 4 out of 6178 genes, 0.1% | 4 out of 6259 genes, 0.1% | 1 |
| acetate metabolic process | 4 out of 6178 genes, 0.1% | 4 out of 6259 genes, 0.1% | 1 |
| acetyl-CoA biosynthetic process from pyruvate | 4 out of 6178 genes, 0.1% | 4 out of 6259 genes, 0.1% | 1 |
| pyruvate metabolic process | 4 out of 6178 genes, 0.1% | 4 out of 6259 genes, 0.1% | 1 |
| pyrimidine base catabolic process | 4 out of 6178 genes, 0.1% | 4 out of 6259 genes, 0.1% | 1 |
| protein monoubiquitination | 4 out of 6178 genes, 0.1% | 4 out of 6259 genes, 0.1% | 1 |
| glutamate metabolic process | 4 out of 6178 genes, 0.1% | 4 out of 6259 genes, 0.1% | 1 |
| indolalkylamine metabolic process | 4 out of 6178 genes, 0.1% | 4 out of 6259 genes, 0.1% | 1 |
| fatty acid biosynthetic process | 4 out of 6178 genes, 0.1% | 4 out of 6259 genes, 0.1% | 1 |
| potassium ion transport | 4 out of 6178 genes, 0.1% | 4 out of 6259 genes, 0.1% | 1 |
| iron ion transport | 4 out of 6178 genes, 0.1% | 4 out of 6259 genes, 0.1% | 1 |
| post-Golgi vesicle-mediated transport | 4 out of 6178 genes, 0.1% | 4 out of 6259 genes, 0.1% | 1 |
| mitochondrial membrane organization | 4 out of 6178 genes, 0.1% | 4 out of 6259 genes, 0.1% | 1 |
| plasma membrane organization | 4 out of 6178 genes, 0.1% | 4 out of 6259 genes, 0.1% | 1 |
| regulation of S phase of mitotic cell cycle | 4 out of 6178 genes, 0.1% | 4 out of 6259 genes, 0.1% | 1 |
| regulation of exit from mitosis | 4 out of 6178 genes, 0.1% | 4 out of 6259 genes, 0.1% | 1 |
| activation of adenylate cyclase activity by G-protein signaling pathway | 4 out of 6178 genes, 0.1% | 4 out of 6259 genes, 0.1% | 1 |
| peripheral nervous system development | 4 out of 6178 genes, 0.1% | 4 out of 6259 genes, 0.1% | 1 |
| visual behavior | 4 out of 6178 genes, 0.1% | 4 out of 6259 genes, 0.1% | 1 |
| cholesterol metabolic process | 4 out of 6178 genes, 0.1% | 4 out of 6259 genes, 0.1% | 1 |
| C21-steroid hormone metabolic process | 4 out of 6178 genes, 0.1% | 4 out of 6259 genes, 0.1% | 1 |
| regulation of G-protein coupled receptor protein signaling pathway | 4 out of 6178 genes, 0.1% | 4 out of 6259 genes, 0.1% | 1 |
| histone mRNA metabolic process | 4 out of 6178 genes, 0.1% | 4 out of 6259 genes, 0.1% | 1 |
| induction of apoptosis by extracellular signals | 4 out of 6178 genes, 0.1% | 4 out of 6259 genes, 0.1% | 1 |
| cellular amino acid biosynthetic process | 4 out of 6178 genes, 0.1% | 4 out of 6259 genes, 0.1% | 1 |
| aromatic amino acid family metabolic process | 4 out of 6178 genes, 0.1% | 4 out of 6259 genes, 0.1% | 1 |
| deoxyribonucleoside monophosphate biosynthetic process | 4 out of 6178 genes, 0.1% | 4 out of 6259 genes, 0.1% | 1 |
| deoxyribonucleoside monophosphate metabolic process | 4 out of 6178 genes, 0.1% | 4 out of 6259 genes, 0.1% | 1 |
| pyrimidine deoxyribonucleoside monophosphate metabolic process | 4 out of 6178 genes, 0.1% | 4 out of 6259 genes, 0.1% | 1 |
| pyrimidine deoxyribonucleoside monophosphate biosynthetic process | 4 out of 6178 genes, 0.1% | 4 out of 6259 genes, 0.1% | 1 |
| nucleotide-sugar metabolic process | 4 out of 6178 genes, 0.1% | 4 out of 6259 genes, 0.1% | 1 |
| response to lithium ion | 4 out of 6178 genes, 0.1% | 4 out of 6259 genes, 0.1% | 1 |
| regulation of receptor activity | 4 out of 6178 genes, 0.1% | 4 out of 6259 genes, 0.1% | 1 |
| positive regulation of phospholipase activity | 4 out of 6178 genes, 0.1% | 4 out of 6259 genes, 0.1% | 1 |
| regulation of adenylate cyclase activity involved in G-protein signaling pathway | 4 out of 6178 genes, 0.1% | 4 out of 6259 genes, 0.1% | 1 |
| positive regulation of adenylate cyclase activity by G-protein signaling pathway | 4 out of 6178 genes, 0.1% | 4 out of 6259 genes, 0.1% | 1 |
| positive regulation of gene expression | 4 out of 6178 genes, 0.1% | 4 out of 6259 genes, 0.1% | 1 |
| negative regulation of cell development | 4 out of 6178 genes, 0.1% | 4 out of 6259 genes, 0.1% | 1 |
| positive regulation of glucose transport | 4 out of 6178 genes, 0.1% | 4 out of 6259 genes, 0.1% | 1 |
| oligodendrocyte development | 4 out of 6178 genes, 0.1% | 4 out of 6259 genes, 0.1% | 1 |
| positive regulation of gliogenesis | 4 out of 6178 genes, 0.1% | 4 out of 6259 genes, 0.1% | 1 |
| response to amine stimulus | 4 out of 6178 genes, 0.1% | 4 out of 6259 genes, 0.1% | 1 |
| twitch skeletal muscle contraction | 4 out of 6178 genes, 0.1% | 4 out of 6259 genes, 0.1% | 1 |
| nucleotide-sugar transport | 4 out of 6178 genes, 0.1% | 4 out of 6259 genes, 0.1% | 1 |
| pyrimidine nucleotide-sugar transport | 4 out of 6178 genes, 0.1% | 4 out of 6259 genes, 0.1% | 1 |
| branched-chain aliphatic amino acid transport | 4 out of 6178 genes, 0.1% | 4 out of 6259 genes, 0.1% | 1 |
| iron-sulfur cluster assembly | 4 out of 6178 genes, 0.1% | 4 out of 6259 genes, 0.1% | 1 |
| dephosphorylation | 4 out of 6178 genes, 0.1% | 4 out of 6259 genes, 0.1% | 1 |
| histone phosphorylation | 4 out of 6178 genes, 0.1% | 4 out of 6259 genes, 0.1% | 1 |
| peptidyl-arginine modification | 4 out of 6178 genes, 0.1% | 4 out of 6259 genes, 0.1% | 1 |
| peptidyl-serine modification | 4 out of 6178 genes, 0.1% | 4 out of 6259 genes, 0.1% | 1 |
| peptidyl-arginine methylation | 4 out of 6178 genes, 0.1% | 4 out of 6259 genes, 0.1% | 1 |
| pentose metabolic process | 4 out of 6178 genes, 0.1% | 4 out of 6259 genes, 0.1% | 1 |
| pyrimidine base biosynthetic process | 4 out of 6178 genes, 0.1% | 4 out of 6259 genes, 0.1% | 1 |
| spinal cord patterning | 4 out of 6178 genes, 0.1% | 4 out of 6259 genes, 0.1% | 1 |
| spinal cord dorsal/ventral patterning | 4 out of 6178 genes, 0.1% | 4 out of 6259 genes, 0.1% | 1 |
| diencephalon development | 4 out of 6178 genes, 0.1% | 4 out of 6259 genes, 0.1% | 1 |
| cerebellum development | 4 out of 6178 genes, 0.1% | 4 out of 6259 genes, 0.1% | 1 |
| cranial nerve morphogenesis | 4 out of 6178 genes, 0.1% | 4 out of 6259 genes, 0.1% | 1 |
| cranial nerve formation | 4 out of 6178 genes, 0.1% | 4 out of 6259 genes, 0.1% | 1 |
| forebrain generation of neurons | 4 out of 6178 genes, 0.1% | 4 out of 6259 genes, 0.1% | 1 |
| negative regulation of smoothened signaling pathway involved in ventral spinal cord patterning | 4 out of 6178 genes, 0.1% | 4 out of 6259 genes, 0.1% | 1 |
| metencephalon development | 4 out of 6178 genes, 0.1% | 4 out of 6259 genes, 0.1% | 1 |
| maintenance of cell polarity | 4 out of 6178 genes, 0.1% | 4 out of 6259 genes, 0.1% | 1 |
| regulation of mitotic metaphase/anaphase transition | 4 out of 6178 genes, 0.1% | 4 out of 6259 genes, 0.1% | 1 |
| insulin secretion | 4 out of 6178 genes, 0.1% | 4 out of 6259 genes, 0.1% | 1 |
| extracellular matrix organization | 4 out of 6178 genes, 0.1% | 4 out of 6259 genes, 0.1% | 1 |
| chromosome condensation | 4 out of 6178 genes, 0.1% | 4 out of 6259 genes, 0.1% | 1 |
| larval behavior | 4 out of 6178 genes, 0.1% | 4 out of 6259 genes, 0.1% | 1 |
| regulation of type I interferon production | 4 out of 6178 genes, 0.1% | 4 out of 6259 genes, 0.1% | 1 |
| positive regulation of type I interferon production | 4 out of 6178 genes, 0.1% | 4 out of 6259 genes, 0.1% | 1 |
| regulation of microtubule-based process | 4 out of 6178 genes, 0.1% | 4 out of 6259 genes, 0.1% | 1 |
| regulation of mononuclear cell proliferation | 4 out of 6178 genes, 0.1% | 4 out of 6259 genes, 0.1% | 1 |
| regulation of S phase | 4 out of 6178 genes, 0.1% | 4 out of 6259 genes, 0.1% | 1 |
| cell cycle comprising mitosis without cytokinesis | 4 out of 6178 genes, 0.1% | 4 out of 6259 genes, 0.1% | 1 |
| response to disaccharide stimulus | 4 out of 6178 genes, 0.1% | 4 out of 6259 genes, 0.1% | 1 |
| endocrine system development | 4 out of 6178 genes, 0.1% | 4 out of 6259 genes, 0.1% | 1 |
| maintenance of protein localization in endoplasmic reticulum | 4 out of 6178 genes, 0.1% | 4 out of 6259 genes, 0.1% | 1 |
| regulation of meiosis | 4 out of 6178 genes, 0.1% | 4 out of 6259 genes, 0.1% | 1 |
| wound healing | 4 out of 6178 genes, 0.1% | 4 out of 6259 genes, 0.1% | 1 |
| germ-line stem cell division | 4 out of 6178 genes, 0.1% | 4 out of 6259 genes, 0.1% | 1 |
| B cell activation | 4 out of 6178 genes, 0.1% | 4 out of 6259 genes, 0.1% | 1 |
| cellular biogenic amine catabolic process | 4 out of 6178 genes, 0.1% | 4 out of 6259 genes, 0.1% | 1 |
| indole and derivative metabolic process | 4 out of 6178 genes, 0.1% | 4 out of 6259 genes, 0.1% | 1 |
| indole derivative metabolic process | 4 out of 6178 genes, 0.1% | 4 out of 6259 genes, 0.1% | 1 |
| odontogenesis of dentine-containing tooth | 4 out of 6178 genes, 0.1% | 4 out of 6259 genes, 0.1% | 1 |
| tyrosine phosphorylation of Stat3 protein | 4 out of 6178 genes, 0.1% | 4 out of 6259 genes, 0.1% | 1 |
| peptide biosynthetic process | 4 out of 6178 genes, 0.1% | 4 out of 6259 genes, 0.1% | 1 |
| regulation of protein binding | 4 out of 6178 genes, 0.1% | 4 out of 6259 genes, 0.1% | 1 |
| regulation of viral genome replication | 4 out of 6178 genes, 0.1% | 4 out of 6259 genes, 0.1% | 1 |
| positive regulation of viral genome replication | 4 out of 6178 genes, 0.1% | 4 out of 6259 genes, 0.1% | 1 |
| regulation of myeloid cell differentiation | 4 out of 6178 genes, 0.1% | 4 out of 6259 genes, 0.1% | 1 |
| pH reduction | 4 out of 6178 genes, 0.1% | 4 out of 6259 genes, 0.1% | 1 |
| negative regulation of smoothened signaling pathway | 4 out of 6178 genes, 0.1% | 4 out of 6259 genes, 0.1% | 1 |
| negative regulation of growth | 4 out of 6178 genes, 0.1% | 4 out of 6259 genes, 0.1% | 1 |
| positive regulation of growth | 4 out of 6178 genes, 0.1% | 4 out of 6259 genes, 0.1% | 1 |
| positive regulation of nucleobase, nucleoside, nucleotide and nucleic acid metabolic process | 4 out of 6178 genes, 0.1% | 4 out of 6259 genes, 0.1% | 1 |
| nucleobase catabolic process | 4 out of 6178 genes, 0.1% | 4 out of 6259 genes, 0.1% | 1 |
| alcohol biosynthetic process | 4 out of 6178 genes, 0.1% | 4 out of 6259 genes, 0.1% | 1 |
| phosphatidylcholine metabolic process | 4 out of 6178 genes, 0.1% | 4 out of 6259 genes, 0.1% | 1 |
| negative regulation of hormone secretion | 4 out of 6178 genes, 0.1% | 4 out of 6259 genes, 0.1% | 1 |
| regulation of lipid biosynthetic process | 4 out of 6178 genes, 0.1% | 4 out of 6259 genes, 0.1% | 1 |
| Golgi vesicle budding | 4 out of 6178 genes, 0.1% | 4 out of 6259 genes, 0.1% | 1 |
| vesicle targeting, to, from or within Golgi | 4 out of 6178 genes, 0.1% | 4 out of 6259 genes, 0.1% | 1 |
| Golgi transport vesicle coating | 4 out of 6178 genes, 0.1% | 4 out of 6259 genes, 0.1% | 1 |
| circadian behavior | 4 out of 6178 genes, 0.1% | 4 out of 6259 genes, 0.1% | 1 |
| axon extension | 4 out of 6178 genes, 0.1% | 4 out of 6259 genes, 0.1% | 1 |
| oligodendrocyte differentiation | 4 out of 6178 genes, 0.1% | 4 out of 6259 genes, 0.1% | 1 |
| cardiac muscle tissue development | 4 out of 6178 genes, 0.1% | 4 out of 6259 genes, 0.1% | 1 |
| lateral line nerve development | 4 out of 6178 genes, 0.1% | 4 out of 6259 genes, 0.1% | 1 |
| regulation of lymphocyte proliferation | 4 out of 6178 genes, 0.1% | 4 out of 6259 genes, 0.1% | 1 |
| positive regulation of neurogenesis | 4 out of 6178 genes, 0.1% | 4 out of 6259 genes, 0.1% | 1 |
| negative regulation of cell activation | 4 out of 6178 genes, 0.1% | 4 out of 6259 genes, 0.1% | 1 |
| negative regulation of secretion | 4 out of 6178 genes, 0.1% | 4 out of 6259 genes, 0.1% | 1 |
| positive regulation of nitrogen compound metabolic process | 4 out of 6178 genes, 0.1% | 4 out of 6259 genes, 0.1% | 1 |
| spindle assembly | 4 out of 6178 genes, 0.1% | 4 out of 6259 genes, 0.1% | 1 |
| regulation of sequestering of calcium ion | 4 out of 6178 genes, 0.1% | 4 out of 6259 genes, 0.1% | 1 |
| negative regulation of sequestering of calcium ion | 4 out of 6178 genes, 0.1% | 4 out of 6259 genes, 0.1% | 1 |
| G1 phase | 4 out of 6178 genes, 0.1% | 4 out of 6259 genes, 0.1% | 1 |
| response to glucocorticoid stimulus | 4 out of 6178 genes, 0.1% | 4 out of 6259 genes, 0.1% | 1 |
| regulation of meiotic cell cycle | 4 out of 6178 genes, 0.1% | 4 out of 6259 genes, 0.1% | 1 |
| canonical Wnt receptor signaling pathway | 4 out of 6178 genes, 0.1% | 4 out of 6259 genes, 0.1% | 1 |
| female mating behavior | 4 out of 6178 genes, 0.1% | 4 out of 6259 genes, 0.1% | 1 |
| positive regulation of lipase activity | 4 out of 6178 genes, 0.1% | 4 out of 6259 genes, 0.1% | 1 |
| branching involved in open tracheal system development | 4 out of 6178 genes, 0.1% | 4 out of 6259 genes, 0.1% | 1 |
| histone lysine demethylation | 4 out of 6178 genes, 0.1% | 4 out of 6259 genes, 0.1% | 1 |
| regulation of leukocyte proliferation | 4 out of 6178 genes, 0.1% | 4 out of 6259 genes, 0.1% | 1 |
| regulation of organ morphogenesis | 4 out of 6178 genes, 0.1% | 4 out of 6259 genes, 0.1% | 1 |
| positive regulation of reproductive process | 4 out of 6178 genes, 0.1% | 4 out of 6259 genes, 0.1% | 1 |
| amino acid transport | 27 out of 6178 genes, 0.4% | 28 out of 6259 genes, 0.4% | 1 |
| RNA modification | 27 out of 6178 genes, 0.4% | 28 out of 6259 genes, 0.4% | 1 |
| photoreceptor cell differentiation | 27 out of 6178 genes, 0.4% | 28 out of 6259 genes, 0.4% | 1 |
| regulation of signaling pathway | 149 out of 6178 genes, 2.4% | 153 out of 6259 genes, 2.4% | 1 |
| microtubule-based movement | 26 out of 6178 genes, 0.4% | 27 out of 6259 genes, 0.4% | 1 |
| aerobic respiration | 26 out of 6178 genes, 0.4% | 27 out of 6259 genes, 0.4% | 1 |
| positive regulation of hydrolase activity | 26 out of 6178 genes, 0.4% | 27 out of 6259 genes, 0.4% | 1 |
| regulation of signaling process | 102 out of 6178 genes, 1.7% | 105 out of 6259 genes, 1.7% | 1 |
| regulation of signal transduction | 101 out of 6178 genes, 1.6% | 104 out of 6259 genes, 1.7% | 1 |
| oxidoreduction coenzyme metabolic process | 25 out of 6178 genes, 0.4% | 26 out of 6259 genes, 0.4% | 1 |
| embryonic pattern specification | 25 out of 6178 genes, 0.4% | 26 out of 6259 genes, 0.4% | 1 |
| regulation of anatomical structure morphogenesis | 25 out of 6178 genes, 0.4% | 26 out of 6259 genes, 0.4% | 1 |
| regulation of hydrolase activity | 59 out of 6178 genes, 1.0% | 61 out of 6259 genes, 1.0% | 1 |
| divalent metal ion transport | 59 out of 6178 genes, 1.0% | 61 out of 6259 genes, 1.0% | 1 |
| nucleotide biosynthetic process | 145 out of 6178 genes, 2.3% | 149 out of 6259 genes, 2.4% | 1 |
| alternative nuclear mRNA splicing, via spliceosome | 24 out of 6178 genes, 0.4% | 25 out of 6259 genes, 0.4% | 1 |
| response to wounding | 24 out of 6178 genes, 0.4% | 25 out of 6259 genes, 0.4% | 1 |
| embryonic organ development | 24 out of 6178 genes, 0.4% | 25 out of 6259 genes, 0.4% | 1 |
| ribonucleotide biosynthetic process | 97 out of 6178 genes, 1.6% | 100 out of 6259 genes, 1.6% | 1 |
| sulfur amino acid biosynthetic process | 3 out of 6178 genes, 0.0% | 3 out of 6259 genes, 0.0% | 1 |
| sulfur amino acid catabolic process | 3 out of 6178 genes, 0.0% | 3 out of 6259 genes, 0.0% | 1 |
| mitochondrial fission | 3 out of 6178 genes, 0.0% | 3 out of 6259 genes, 0.0% | 1 |
| lipoic acid metabolic process | 3 out of 6178 genes, 0.0% | 3 out of 6259 genes, 0.0% | 1 |
| maturation of LSU-rRNA | 3 out of 6178 genes, 0.0% | 3 out of 6259 genes, 0.0% | 1 |
| neurotransmitter uptake | 3 out of 6178 genes, 0.0% | 3 out of 6259 genes, 0.0% | 1 |
| ganglioside metabolic process | 3 out of 6178 genes, 0.0% | 3 out of 6259 genes, 0.0% | 1 |
| osteoblast differentiation | 3 out of 6178 genes, 0.0% | 3 out of 6259 genes, 0.0% | 1 |
| leukocyte homeostasis | 3 out of 6178 genes, 0.0% | 3 out of 6259 genes, 0.0% | 1 |
| trophectodermal cell differentiation | 3 out of 6178 genes, 0.0% | 3 out of 6259 genes, 0.0% | 1 |
| endothelial cell development | 3 out of 6178 genes, 0.0% | 3 out of 6259 genes, 0.0% | 1 |
| regulation of leukocyte mediated cytotoxicity | 3 out of 6178 genes, 0.0% | 3 out of 6259 genes, 0.0% | 1 |
| positive regulation of leukocyte mediated cytotoxicity | 3 out of 6178 genes, 0.0% | 3 out of 6259 genes, 0.0% | 1 |
| regulation of cytokine-mediated signaling pathway | 3 out of 6178 genes, 0.0% | 3 out of 6259 genes, 0.0% | 1 |
| regulation of sodium ion transport | 3 out of 6178 genes, 0.0% | 3 out of 6259 genes, 0.0% | 1 |
| instar larval development | 3 out of 6178 genes, 0.0% | 3 out of 6259 genes, 0.0% | 1 |
| lymphocyte homeostasis | 3 out of 6178 genes, 0.0% | 3 out of 6259 genes, 0.0% | 1 |
| myeloid leukocyte activation | 3 out of 6178 genes, 0.0% | 3 out of 6259 genes, 0.0% | 1 |
| inflammatory response to antigenic stimulus | 3 out of 6178 genes, 0.0% | 3 out of 6259 genes, 0.0% | 1 |
| regulation of response to biotic stimulus | 3 out of 6178 genes, 0.0% | 3 out of 6259 genes, 0.0% | 1 |
| regulation of systemic arterial blood pressure mediated by a chemical signal | 3 out of 6178 genes, 0.0% | 3 out of 6259 genes, 0.0% | 1 |
| regulation of systemic arterial blood pressure | 3 out of 6178 genes, 0.0% | 3 out of 6259 genes, 0.0% | 1 |
| regulation of organ formation | 3 out of 6178 genes, 0.0% | 3 out of 6259 genes, 0.0% | 1 |
| cardiac chamber development | 3 out of 6178 genes, 0.0% | 3 out of 6259 genes, 0.0% | 1 |
| amino acid transmembrane transport | 3 out of 6178 genes, 0.0% | 3 out of 6259 genes, 0.0% | 1 |
| glycogen catabolic process | 3 out of 6178 genes, 0.0% | 3 out of 6259 genes, 0.0% | 1 |
| galactose metabolic process | 3 out of 6178 genes, 0.0% | 3 out of 6259 genes, 0.0% | 1 |
| aminoglycan catabolic process | 3 out of 6178 genes, 0.0% | 3 out of 6259 genes, 0.0% | 1 |
| glycerol-3-phosphate metabolic process | 3 out of 6178 genes, 0.0% | 3 out of 6259 genes, 0.0% | 1 |
| regulation of glycolysis | 3 out of 6178 genes, 0.0% | 3 out of 6259 genes, 0.0% | 1 |
| DNA ligation | 3 out of 6178 genes, 0.0% | 3 out of 6259 genes, 0.0% | 1 |
| mismatch repair | 3 out of 6178 genes, 0.0% | 3 out of 6259 genes, 0.0% | 1 |
| nucleosome assembly | 3 out of 6178 genes, 0.0% | 3 out of 6259 genes, 0.0% | 1 |
| translational termination | 3 out of 6178 genes, 0.0% | 3 out of 6259 genes, 0.0% | 1 |
| negative regulation of protein kinase activity | 3 out of 6178 genes, 0.0% | 3 out of 6259 genes, 0.0% | 1 |
| alanine metabolic process | 3 out of 6178 genes, 0.0% | 3 out of 6259 genes, 0.0% | 1 |
| glutamine metabolic process | 3 out of 6178 genes, 0.0% | 3 out of 6259 genes, 0.0% | 1 |
| glycine metabolic process | 3 out of 6178 genes, 0.0% | 3 out of 6259 genes, 0.0% | 1 |
| ethanolamine metabolic process | 3 out of 6178 genes, 0.0% | 3 out of 6259 genes, 0.0% | 1 |
| catecholamine metabolic process | 3 out of 6178 genes, 0.0% | 3 out of 6259 genes, 0.0% | 1 |
| SRP-dependent cotranslational protein targeting to membrane | 3 out of 6178 genes, 0.0% | 3 out of 6259 genes, 0.0% | 1 |
| protein targeting to mitochondrion | 3 out of 6178 genes, 0.0% | 3 out of 6259 genes, 0.0% | 1 |
| phosphatidylcholine biosynthetic process | 3 out of 6178 genes, 0.0% | 3 out of 6259 genes, 0.0% | 1 |
| glycosylceramide metabolic process | 3 out of 6178 genes, 0.0% | 3 out of 6259 genes, 0.0% | 1 |
| sphingomyelin metabolic process | 3 out of 6178 genes, 0.0% | 3 out of 6259 genes, 0.0% | 1 |
| icosanoid metabolic process | 3 out of 6178 genes, 0.0% | 3 out of 6259 genes, 0.0% | 1 |
| glutathione metabolic process | 3 out of 6178 genes, 0.0% | 3 out of 6259 genes, 0.0% | 1 |
| superoxide metabolic process | 3 out of 6178 genes, 0.0% | 3 out of 6259 genes, 0.0% | 1 |
| chloride transport | 3 out of 6178 genes, 0.0% | 3 out of 6259 genes, 0.0% | 1 |
| mitochondrial transport | 3 out of 6178 genes, 0.0% | 3 out of 6259 genes, 0.0% | 1 |
| smooth muscle contraction | 3 out of 6178 genes, 0.0% | 3 out of 6259 genes, 0.0% | 1 |
| mitotic cell cycle checkpoint | 3 out of 6178 genes, 0.0% | 3 out of 6259 genes, 0.0% | 1 |
| epidermal growth factor receptor signaling pathway | 3 out of 6178 genes, 0.0% | 3 out of 6259 genes, 0.0% | 1 |
| imaginal disc-derived leg morphogenesis | 3 out of 6178 genes, 0.0% | 3 out of 6259 genes, 0.0% | 1 |
| sex determination | 3 out of 6178 genes, 0.0% | 3 out of 6259 genes, 0.0% | 1 |
| hemostasis | 3 out of 6178 genes, 0.0% | 3 out of 6259 genes, 0.0% | 1 |
| copulation | 3 out of 6178 genes, 0.0% | 3 out of 6259 genes, 0.0% | 1 |
| glucocorticoid metabolic process | 3 out of 6178 genes, 0.0% | 3 out of 6259 genes, 0.0% | 1 |
| regulation of blood pressure | 3 out of 6178 genes, 0.0% | 3 out of 6259 genes, 0.0% | 1 |
| germ cell migration | 3 out of 6178 genes, 0.0% | 3 out of 6259 genes, 0.0% | 1 |
| regulation of cell shape | 3 out of 6178 genes, 0.0% | 3 out of 6259 genes, 0.0% | 1 |
| imaginal disc-derived wing margin morphogenesis | 3 out of 6178 genes, 0.0% | 3 out of 6259 genes, 0.0% | 1 |
| guanosine metabolic process | 3 out of 6178 genes, 0.0% | 3 out of 6259 genes, 0.0% | 1 |
| DNA damage response, signal transduction resulting in induction of apoptosis | 3 out of 6178 genes, 0.0% | 3 out of 6259 genes, 0.0% | 1 |
| pyruvate family amino acid metabolic process | 3 out of 6178 genes, 0.0% | 3 out of 6259 genes, 0.0% | 1 |
| nucleoside diphosphate biosynthetic process | 3 out of 6178 genes, 0.0% | 3 out of 6259 genes, 0.0% | 1 |
| purine nucleoside diphosphate metabolic process | 3 out of 6178 genes, 0.0% | 3 out of 6259 genes, 0.0% | 1 |
| purine deoxyribonucleotide metabolic process | 3 out of 6178 genes, 0.0% | 3 out of 6259 genes, 0.0% | 1 |
| nucleoside biosynthetic process | 3 out of 6178 genes, 0.0% | 3 out of 6259 genes, 0.0% | 1 |
| pyrimidine ribonucleoside monophosphate metabolic process | 3 out of 6178 genes, 0.0% | 3 out of 6259 genes, 0.0% | 1 |
| pyrimidine ribonucleoside monophosphate biosynthetic process | 3 out of 6178 genes, 0.0% | 3 out of 6259 genes, 0.0% | 1 |
| purine ribonucleoside diphosphate metabolic process | 3 out of 6178 genes, 0.0% | 3 out of 6259 genes, 0.0% | 1 |
| ribonucleoside diphosphate metabolic process | 3 out of 6178 genes, 0.0% | 3 out of 6259 genes, 0.0% | 1 |
| cyclic nucleotide metabolic process | 3 out of 6178 genes, 0.0% | 3 out of 6259 genes, 0.0% | 1 |
| purine deoxyribonucleoside triphosphate metabolic process | 3 out of 6178 genes, 0.0% | 3 out of 6259 genes, 0.0% | 1 |
| nucleotide-sugar biosynthetic process | 3 out of 6178 genes, 0.0% | 3 out of 6259 genes, 0.0% | 1 |
| glucan catabolic process | 3 out of 6178 genes, 0.0% | 3 out of 6259 genes, 0.0% | 1 |
| fertilization | 3 out of 6178 genes, 0.0% | 3 out of 6259 genes, 0.0% | 1 |
| catechol metabolic process | 3 out of 6178 genes, 0.0% | 3 out of 6259 genes, 0.0% | 1 |
| alkaloid metabolic process | 3 out of 6178 genes, 0.0% | 3 out of 6259 genes, 0.0% | 1 |
| regulation of cell fate commitment | 3 out of 6178 genes, 0.0% | 3 out of 6259 genes, 0.0% | 1 |
| epithelial cell migration | 3 out of 6178 genes, 0.0% | 3 out of 6259 genes, 0.0% | 1 |
| negative regulation of glucose transport | 3 out of 6178 genes, 0.0% | 3 out of 6259 genes, 0.0% | 1 |
| positive regulation of phospholipase C activity | 3 out of 6178 genes, 0.0% | 3 out of 6259 genes, 0.0% | 1 |
| regulation of phosphatase activity | 3 out of 6178 genes, 0.0% | 3 out of 6259 genes, 0.0% | 1 |
| neuroblast differentiation | 3 out of 6178 genes, 0.0% | 3 out of 6259 genes, 0.0% | 1 |
| Schwann cell differentiation | 3 out of 6178 genes, 0.0% | 3 out of 6259 genes, 0.0% | 1 |
| glucose transport | 3 out of 6178 genes, 0.0% | 3 out of 6259 genes, 0.0% | 1 |
| polyol transport | 3 out of 6178 genes, 0.0% | 3 out of 6259 genes, 0.0% | 1 |
| leucine transport | 3 out of 6178 genes, 0.0% | 3 out of 6259 genes, 0.0% | 1 |
| purine nucleoside transport | 3 out of 6178 genes, 0.0% | 3 out of 6259 genes, 0.0% | 1 |
| bis(5'-nucleosidyl) oligophosphate metabolic process | 3 out of 6178 genes, 0.0% | 3 out of 6259 genes, 0.0% | 1 |
| bis(5'-nucleosidyl) oligophosphate biosynthetic process | 3 out of 6178 genes, 0.0% | 3 out of 6259 genes, 0.0% | 1 |
| diadenosine polyphosphate metabolic process | 3 out of 6178 genes, 0.0% | 3 out of 6259 genes, 0.0% | 1 |
| diadenosine polyphosphate biosynthetic process | 3 out of 6178 genes, 0.0% | 3 out of 6259 genes, 0.0% | 1 |
| synaptic vesicle priming | 3 out of 6178 genes, 0.0% | 3 out of 6259 genes, 0.0% | 1 |
| glycoside metabolic process | 3 out of 6178 genes, 0.0% | 3 out of 6259 genes, 0.0% | 1 |
| glycoside catabolic process | 3 out of 6178 genes, 0.0% | 3 out of 6259 genes, 0.0% | 1 |
| RNA interference | 3 out of 6178 genes, 0.0% | 3 out of 6259 genes, 0.0% | 1 |
| response to insecticide | 3 out of 6178 genes, 0.0% | 3 out of 6259 genes, 0.0% | 1 |
| calcium ion-dependent exocytosis | 3 out of 6178 genes, 0.0% | 3 out of 6259 genes, 0.0% | 1 |
| peptidyl-serine phosphorylation | 3 out of 6178 genes, 0.0% | 3 out of 6259 genes, 0.0% | 1 |
| peptidyl-threonine phosphorylation | 3 out of 6178 genes, 0.0% | 3 out of 6259 genes, 0.0% | 1 |
| peptidyl-threonine modification | 3 out of 6178 genes, 0.0% | 3 out of 6259 genes, 0.0% | 1 |
| phenol metabolic process | 3 out of 6178 genes, 0.0% | 3 out of 6259 genes, 0.0% | 1 |
| germ-line sex determination | 3 out of 6178 genes, 0.0% | 3 out of 6259 genes, 0.0% | 1 |
| latent virus infection | 3 out of 6178 genes, 0.0% | 3 out of 6259 genes, 0.0% | 1 |
| antigen processing and presentation | 3 out of 6178 genes, 0.0% | 3 out of 6259 genes, 0.0% | 1 |
| antigen processing and presentation of exogenous antigen | 3 out of 6178 genes, 0.0% | 3 out of 6259 genes, 0.0% | 1 |
| lipid storage | 3 out of 6178 genes, 0.0% | 3 out of 6259 genes, 0.0% | 1 |
| cerebellar cortex development | 3 out of 6178 genes, 0.0% | 3 out of 6259 genes, 0.0% | 1 |
| central nervous system neuron development | 3 out of 6178 genes, 0.0% | 3 out of 6259 genes, 0.0% | 1 |
| central nervous system neuron axonogenesis | 3 out of 6178 genes, 0.0% | 3 out of 6259 genes, 0.0% | 1 |
| central nervous system myelination | 3 out of 6178 genes, 0.0% | 3 out of 6259 genes, 0.0% | 1 |
| regulation of cell-cell adhesion | 3 out of 6178 genes, 0.0% | 3 out of 6259 genes, 0.0% | 1 |
| microspike assembly | 3 out of 6178 genes, 0.0% | 3 out of 6259 genes, 0.0% | 1 |
| bone mineralization | 3 out of 6178 genes, 0.0% | 3 out of 6259 genes, 0.0% | 1 |
| eggshell formation | 3 out of 6178 genes, 0.0% | 3 out of 6259 genes, 0.0% | 1 |
| regulation of actin filament polymerization | 3 out of 6178 genes, 0.0% | 3 out of 6259 genes, 0.0% | 1 |
| regulation of actin filament depolymerization | 3 out of 6178 genes, 0.0% | 3 out of 6259 genes, 0.0% | 1 |
| negative regulation of actin filament depolymerization | 3 out of 6178 genes, 0.0% | 3 out of 6259 genes, 0.0% | 1 |
| regulation of epithelial cell differentiation | 3 out of 6178 genes, 0.0% | 3 out of 6259 genes, 0.0% | 1 |
| mammary gland development | 3 out of 6178 genes, 0.0% | 3 out of 6259 genes, 0.0% | 1 |
| myosin filament assembly or disassembly | 3 out of 6178 genes, 0.0% | 3 out of 6259 genes, 0.0% | 1 |
| regulation of vesicle fusion | 3 out of 6178 genes, 0.0% | 3 out of 6259 genes, 0.0% | 1 |
| positive regulation of vesicle fusion | 3 out of 6178 genes, 0.0% | 3 out of 6259 genes, 0.0% | 1 |
| regulation of cell killing | 3 out of 6178 genes, 0.0% | 3 out of 6259 genes, 0.0% | 1 |
| positive regulation of cell killing | 3 out of 6178 genes, 0.0% | 3 out of 6259 genes, 0.0% | 1 |
| positive regulation of cell projection organization | 3 out of 6178 genes, 0.0% | 3 out of 6259 genes, 0.0% | 1 |
| chromatin assembly | 3 out of 6178 genes, 0.0% | 3 out of 6259 genes, 0.0% | 1 |
| regulation of protein polymerization | 3 out of 6178 genes, 0.0% | 3 out of 6259 genes, 0.0% | 1 |
| axon ensheathment in central nervous system | 3 out of 6178 genes, 0.0% | 3 out of 6259 genes, 0.0% | 1 |
| regulation of lipid transport | 3 out of 6178 genes, 0.0% | 3 out of 6259 genes, 0.0% | 1 |
| negative regulation of intracellular transport | 3 out of 6178 genes, 0.0% | 3 out of 6259 genes, 0.0% | 1 |
| melanosome localization | 3 out of 6178 genes, 0.0% | 3 out of 6259 genes, 0.0% | 1 |
| establishment of melanosome localization | 3 out of 6178 genes, 0.0% | 3 out of 6259 genes, 0.0% | 1 |
| negative regulation of transporter activity | 3 out of 6178 genes, 0.0% | 3 out of 6259 genes, 0.0% | 1 |
| positive regulation of transporter activity | 3 out of 6178 genes, 0.0% | 3 out of 6259 genes, 0.0% | 1 |
| negative regulation of ion transmembrane transporter activity | 3 out of 6178 genes, 0.0% | 3 out of 6259 genes, 0.0% | 1 |
| positive regulation of ion transmembrane transporter activity | 3 out of 6178 genes, 0.0% | 3 out of 6259 genes, 0.0% | 1 |
| regulation of organic acid transport | 3 out of 6178 genes, 0.0% | 3 out of 6259 genes, 0.0% | 1 |
| activin receptor signaling pathway | 3 out of 6178 genes, 0.0% | 3 out of 6259 genes, 0.0% | 1 |
| negative regulation of mononuclear cell proliferation | 3 out of 6178 genes, 0.0% | 3 out of 6259 genes, 0.0% | 1 |
| T cell differentiation in thymus | 3 out of 6178 genes, 0.0% | 3 out of 6259 genes, 0.0% | 1 |
| regulation of cellular amine metabolic process | 3 out of 6178 genes, 0.0% | 3 out of 6259 genes, 0.0% | 1 |
| unsaturated fatty acid metabolic process | 3 out of 6178 genes, 0.0% | 3 out of 6259 genes, 0.0% | 1 |
| diol metabolic process | 3 out of 6178 genes, 0.0% | 3 out of 6259 genes, 0.0% | 1 |
| cell junction maintenance | 3 out of 6178 genes, 0.0% | 3 out of 6259 genes, 0.0% | 1 |
| imaginal disc-derived limb morphogenesis | 3 out of 6178 genes, 0.0% | 3 out of 6259 genes, 0.0% | 1 |
| embryonic forelimb morphogenesis | 3 out of 6178 genes, 0.0% | 3 out of 6259 genes, 0.0% | 1 |
| post-embryonic limb morphogenesis | 3 out of 6178 genes, 0.0% | 3 out of 6259 genes, 0.0% | 1 |
| forelimb morphogenesis | 3 out of 6178 genes, 0.0% | 3 out of 6259 genes, 0.0% | 1 |
| regulation of dephosphorylation | 3 out of 6178 genes, 0.0% | 3 out of 6259 genes, 0.0% | 1 |
| histone-threonine phosphorylation | 3 out of 6178 genes, 0.0% | 3 out of 6259 genes, 0.0% | 1 |
| regulation of chromatin binding | 3 out of 6178 genes, 0.0% | 3 out of 6259 genes, 0.0% | 1 |
| regulation of locomotion | 3 out of 6178 genes, 0.0% | 3 out of 6259 genes, 0.0% | 1 |
| regulation of catecholamine metabolic process | 3 out of 6178 genes, 0.0% | 3 out of 6259 genes, 0.0% | 1 |
| purine nucleoside biosynthetic process | 3 out of 6178 genes, 0.0% | 3 out of 6259 genes, 0.0% | 1 |
| deoxyguanosine metabolic process | 3 out of 6178 genes, 0.0% | 3 out of 6259 genes, 0.0% | 1 |
| ribonucleoside biosynthetic process | 3 out of 6178 genes, 0.0% | 3 out of 6259 genes, 0.0% | 1 |
| thiamin and derivative metabolic process | 3 out of 6178 genes, 0.0% | 3 out of 6259 genes, 0.0% | 1 |
| receptor metabolic process | 3 out of 6178 genes, 0.0% | 3 out of 6259 genes, 0.0% | 1 |
| peptide catabolic process | 3 out of 6178 genes, 0.0% | 3 out of 6259 genes, 0.0% | 1 |
| negative regulation of protein complex disassembly | 3 out of 6178 genes, 0.0% | 3 out of 6259 genes, 0.0% | 1 |
| regulation of protein complex disassembly | 3 out of 6178 genes, 0.0% | 3 out of 6259 genes, 0.0% | 1 |
| negative regulation of MAP kinase activity | 3 out of 6178 genes, 0.0% | 3 out of 6259 genes, 0.0% | 1 |
| negative regulation of transcription factor activity | 3 out of 6178 genes, 0.0% | 3 out of 6259 genes, 0.0% | 1 |
| regulation of carbohydrate catabolic process | 3 out of 6178 genes, 0.0% | 3 out of 6259 genes, 0.0% | 1 |
| regulation of cellular carbohydrate catabolic process | 3 out of 6178 genes, 0.0% | 3 out of 6259 genes, 0.0% | 1 |
| RNA stabilization | 3 out of 6178 genes, 0.0% | 3 out of 6259 genes, 0.0% | 1 |
| regulation of neuron apoptosis | 3 out of 6178 genes, 0.0% | 3 out of 6259 genes, 0.0% | 1 |
| regulation of multi-organism process | 3 out of 6178 genes, 0.0% | 3 out of 6259 genes, 0.0% | 1 |
| histone H3 acetylation | 3 out of 6178 genes, 0.0% | 3 out of 6259 genes, 0.0% | 1 |
| histone H4 acetylation | 3 out of 6178 genes, 0.0% | 3 out of 6259 genes, 0.0% | 1 |
| cellular polysaccharide catabolic process | 3 out of 6178 genes, 0.0% | 3 out of 6259 genes, 0.0% | 1 |
| sulfur compound catabolic process | 3 out of 6178 genes, 0.0% | 3 out of 6259 genes, 0.0% | 1 |
| interspecies interaction between organisms | 3 out of 6178 genes, 0.0% | 3 out of 6259 genes, 0.0% | 1 |
| protein targeting to ER | 3 out of 6178 genes, 0.0% | 3 out of 6259 genes, 0.0% | 1 |
| endothelial cell differentiation | 3 out of 6178 genes, 0.0% | 3 out of 6259 genes, 0.0% | 1 |
| regulation of B cell differentiation | 3 out of 6178 genes, 0.0% | 3 out of 6259 genes, 0.0% | 1 |
| regulation of glial cell differentiation | 3 out of 6178 genes, 0.0% | 3 out of 6259 genes, 0.0% | 1 |
| positive regulation of glial cell differentiation | 3 out of 6178 genes, 0.0% | 3 out of 6259 genes, 0.0% | 1 |
| negative regulation of G-protein coupled receptor protein signaling pathway | 3 out of 6178 genes, 0.0% | 3 out of 6259 genes, 0.0% | 1 |
| positive regulation of cell cycle | 3 out of 6178 genes, 0.0% | 3 out of 6259 genes, 0.0% | 1 |
| negative regulation of mitosis | 3 out of 6178 genes, 0.0% | 3 out of 6259 genes, 0.0% | 1 |
| negative regulation of mitotic metaphase/anaphase transition | 3 out of 6178 genes, 0.0% | 3 out of 6259 genes, 0.0% | 1 |
| positive regulation of transcription | 3 out of 6178 genes, 0.0% | 3 out of 6259 genes, 0.0% | 1 |
| deoxyinosine metabolic process | 3 out of 6178 genes, 0.0% | 3 out of 6259 genes, 0.0% | 1 |
| thymidine metabolic process | 3 out of 6178 genes, 0.0% | 3 out of 6259 genes, 0.0% | 1 |
| purine deoxyribonucleoside metabolic process | 3 out of 6178 genes, 0.0% | 3 out of 6259 genes, 0.0% | 1 |
| purine ribonucleoside biosynthetic process | 3 out of 6178 genes, 0.0% | 3 out of 6259 genes, 0.0% | 1 |
| ethanolamine biosynthetic process | 3 out of 6178 genes, 0.0% | 3 out of 6259 genes, 0.0% | 1 |
| monosaccharide biosynthetic process | 3 out of 6178 genes, 0.0% | 3 out of 6259 genes, 0.0% | 1 |
| phosphatidylinositol metabolic process | 3 out of 6178 genes, 0.0% | 3 out of 6259 genes, 0.0% | 1 |
| nicotinamide riboside metabolic process | 3 out of 6178 genes, 0.0% | 3 out of 6259 genes, 0.0% | 1 |
| negative regulation of nucleocytoplasmic transport | 3 out of 6178 genes, 0.0% | 3 out of 6259 genes, 0.0% | 1 |
| nonassociative learning | 3 out of 6178 genes, 0.0% | 3 out of 6259 genes, 0.0% | 1 |
| germ-line cyst formation | 3 out of 6178 genes, 0.0% | 3 out of 6259 genes, 0.0% | 1 |
| mRNA stabilization | 3 out of 6178 genes, 0.0% | 3 out of 6259 genes, 0.0% | 1 |
| mesodermal cell differentiation | 3 out of 6178 genes, 0.0% | 3 out of 6259 genes, 0.0% | 1 |
| negative regulation of response to stimulus | 3 out of 6178 genes, 0.0% | 3 out of 6259 genes, 0.0% | 1 |
| tissue remodeling | 3 out of 6178 genes, 0.0% | 3 out of 6259 genes, 0.0% | 1 |
| pronephros development | 3 out of 6178 genes, 0.0% | 3 out of 6259 genes, 0.0% | 1 |
| lateral line development | 3 out of 6178 genes, 0.0% | 3 out of 6259 genes, 0.0% | 1 |
| neuromast development | 3 out of 6178 genes, 0.0% | 3 out of 6259 genes, 0.0% | 1 |
| negative regulation of lymphocyte proliferation | 3 out of 6178 genes, 0.0% | 3 out of 6259 genes, 0.0% | 1 |
| regulation of defense response to virus | 3 out of 6178 genes, 0.0% | 3 out of 6259 genes, 0.0% | 1 |
| negative regulation of lymphocyte activation | 3 out of 6178 genes, 0.0% | 3 out of 6259 genes, 0.0% | 1 |
| negative regulation of cytoskeleton organization | 3 out of 6178 genes, 0.0% | 3 out of 6259 genes, 0.0% | 1 |
| positive regulation of cytoskeleton organization | 3 out of 6178 genes, 0.0% | 3 out of 6259 genes, 0.0% | 1 |
| negative regulation of nuclear division | 3 out of 6178 genes, 0.0% | 3 out of 6259 genes, 0.0% | 1 |
| establishment of pigment granule localization | 3 out of 6178 genes, 0.0% | 3 out of 6259 genes, 0.0% | 1 |
| regulation of calcium ion transport | 3 out of 6178 genes, 0.0% | 3 out of 6259 genes, 0.0% | 1 |
| catecholamine transport | 3 out of 6178 genes, 0.0% | 3 out of 6259 genes, 0.0% | 1 |
| regulation of postsynaptic membrane potential | 3 out of 6178 genes, 0.0% | 3 out of 6259 genes, 0.0% | 1 |
| regulation of growth hormone secretion | 3 out of 6178 genes, 0.0% | 3 out of 6259 genes, 0.0% | 1 |
| mammary gland epithelium development | 3 out of 6178 genes, 0.0% | 3 out of 6259 genes, 0.0% | 1 |
| protein localization in mitochondrion | 3 out of 6178 genes, 0.0% | 3 out of 6259 genes, 0.0% | 1 |
| pyridine nucleoside metabolic process | 3 out of 6178 genes, 0.0% | 3 out of 6259 genes, 0.0% | 1 |
| negative regulation of leukocyte proliferation | 3 out of 6178 genes, 0.0% | 3 out of 6259 genes, 0.0% | 1 |
| negative regulation of protein serine/threonine kinase activity | 3 out of 6178 genes, 0.0% | 3 out of 6259 genes, 0.0% | 1 |
| cellular component maintenance at cellular level | 3 out of 6178 genes, 0.0% | 3 out of 6259 genes, 0.0% | 1 |
| nephron development | 3 out of 6178 genes, 0.0% | 3 out of 6259 genes, 0.0% | 1 |
| negative regulation of transcription regulator activity | 3 out of 6178 genes, 0.0% | 3 out of 6259 genes, 0.0% | 1 |
| epithelium migration | 3 out of 6178 genes, 0.0% | 3 out of 6259 genes, 0.0% | 1 |
| regulation of triglyceride metabolic process | 3 out of 6178 genes, 0.0% | 3 out of 6259 genes, 0.0% | 1 |
| spindle assembly involved in meiosis | 3 out of 6178 genes, 0.0% | 3 out of 6259 genes, 0.0% | 1 |
| regulation of fatty acid transport | 3 out of 6178 genes, 0.0% | 3 out of 6259 genes, 0.0% | 1 |
| cellular biogenic amine metabolic process | 23 out of 6178 genes, 0.4% | 24 out of 6259 genes, 0.4% | 1 |
| microtubule-based transport | 23 out of 6178 genes, 0.4% | 24 out of 6259 genes, 0.4% | 1 |
| cytoskeleton-dependent intracellular transport | 23 out of 6178 genes, 0.4% | 24 out of 6259 genes, 0.4% | 1 |
| regulation of protein localization | 23 out of 6178 genes, 0.4% | 24 out of 6259 genes, 0.4% | 1 |
| regulation of protein transport | 23 out of 6178 genes, 0.4% | 24 out of 6259 genes, 0.4% | 1 |
| regulation of establishment of protein localization | 23 out of 6178 genes, 0.4% | 24 out of 6259 genes, 0.4% | 1 |
| regulation of protein metabolic process | 94 out of 6178 genes, 1.5% | 97 out of 6259 genes, 1.5% | 1 |
| polyol metabolic process | 22 out of 6178 genes, 0.4% | 23 out of 6259 genes, 0.4% | 1 |
| sphingoid metabolic process | 22 out of 6178 genes, 0.4% | 23 out of 6259 genes, 0.4% | 1 |
| regulation of nucleocytoplasmic transport | 22 out of 6178 genes, 0.4% | 23 out of 6259 genes, 0.4% | 1 |
| eye photoreceptor cell differentiation | 21 out of 6178 genes, 0.3% | 22 out of 6259 genes, 0.4% | 1 |
| gene silencing by RNA | 21 out of 6178 genes, 0.3% | 22 out of 6259 genes, 0.4% | 1 |
| regulation of intracellular protein transport | 21 out of 6178 genes, 0.3% | 22 out of 6259 genes, 0.4% | 1 |
| regulation of transmembrane transport | 21 out of 6178 genes, 0.3% | 22 out of 6259 genes, 0.4% | 1 |
| coenzyme metabolic process | 52 out of 6178 genes, 0.8% | 54 out of 6259 genes, 0.9% | 1 |
| purine ribonucleotide biosynthetic process | 89 out of 6178 genes, 1.4% | 92 out of 6259 genes, 1.5% | 1 |
| inorganic anion transport | 20 out of 6178 genes, 0.3% | 21 out of 6259 genes, 0.3% | 1 |
| heterocycle biosynthetic process | 20 out of 6178 genes, 0.3% | 21 out of 6259 genes, 0.3% | 1 |
| T cell activation | 20 out of 6178 genes, 0.3% | 21 out of 6259 genes, 0.3% | 1 |
| maintenance of location | 20 out of 6178 genes, 0.3% | 21 out of 6259 genes, 0.3% | 1 |
| pyridine nucleotide metabolic process | 19 out of 6178 genes, 0.3% | 20 out of 6259 genes, 0.3% | 1 |
| cell adhesion | 48 out of 6178 genes, 0.8% | 50 out of 6259 genes, 0.8% | 1 |
| biological adhesion | 48 out of 6178 genes, 0.8% | 50 out of 6259 genes, 0.8% | 1 |
| G1 phase of mitotic cell cycle | 2 out of 6178 genes, 0.0% | 2 out of 6259 genes, 0.0% | 1 |
| G2/M transition of mitotic cell cycle | 2 out of 6178 genes, 0.0% | 2 out of 6259 genes, 0.0% | 1 |
| rRNA modification | 2 out of 6178 genes, 0.0% | 2 out of 6259 genes, 0.0% | 1 |
| activation of MAPKK activity | 2 out of 6178 genes, 0.0% | 2 out of 6259 genes, 0.0% | 1 |
| nuclear-transcribed mRNA catabolic process, deadenylation-dependent decay | 2 out of 6178 genes, 0.0% | 2 out of 6259 genes, 0.0% | 1 |
| response to oxygen radical | 2 out of 6178 genes, 0.0% | 2 out of 6259 genes, 0.0% | 1 |
| regulation of transposition, DNA-mediated | 2 out of 6178 genes, 0.0% | 2 out of 6259 genes, 0.0% | 1 |
| RNA splicing, via endonucleolytic cleavage and ligation | 2 out of 6178 genes, 0.0% | 2 out of 6259 genes, 0.0% | 1 |
| maturation of LSU-rRNA from tricistronic rRNA transcript (SSU-rRNA, 5.8S rRNA, LSU-rRNA) | 2 out of 6178 genes, 0.0% | 2 out of 6259 genes, 0.0% | 1 |
| cytokinetic cell separation | 2 out of 6178 genes, 0.0% | 2 out of 6259 genes, 0.0% | 1 |
| pseudouridine synthesis | 2 out of 6178 genes, 0.0% | 2 out of 6259 genes, 0.0% | 1 |
| ciliary or flagellar motility | 2 out of 6178 genes, 0.0% | 2 out of 6259 genes, 0.0% | 1 |
| microtubule bundle formation | 2 out of 6178 genes, 0.0% | 2 out of 6259 genes, 0.0% | 1 |
| long-chain fatty acid metabolic process | 2 out of 6178 genes, 0.0% | 2 out of 6259 genes, 0.0% | 1 |
| organ induction | 2 out of 6178 genes, 0.0% | 2 out of 6259 genes, 0.0% | 1 |
| receptor recycling | 2 out of 6178 genes, 0.0% | 2 out of 6259 genes, 0.0% | 1 |
| placenta development | 2 out of 6178 genes, 0.0% | 2 out of 6259 genes, 0.0% | 1 |
| leukocyte mediated cytotoxicity | 2 out of 6178 genes, 0.0% | 2 out of 6259 genes, 0.0% | 1 |
| negative regulation of protein phosphorylation | 2 out of 6178 genes, 0.0% | 2 out of 6259 genes, 0.0% | 1 |
| regulation of systemic arterial blood pressure by hormone | 2 out of 6178 genes, 0.0% | 2 out of 6259 genes, 0.0% | 1 |
| desensitization of G-protein coupled receptor protein signaling pathway | 2 out of 6178 genes, 0.0% | 2 out of 6259 genes, 0.0% | 1 |
| lens development in camera-type eye | 2 out of 6178 genes, 0.0% | 2 out of 6259 genes, 0.0% | 1 |
| humoral immune response mediated by circulating immunoglobulin | 2 out of 6178 genes, 0.0% | 2 out of 6259 genes, 0.0% | 1 |
| T cell mediated immunity | 2 out of 6178 genes, 0.0% | 2 out of 6259 genes, 0.0% | 1 |
| germinal center formation | 2 out of 6178 genes, 0.0% | 2 out of 6259 genes, 0.0% | 1 |
| antigen processing and presentation of exogenous peptide antigen | 2 out of 6178 genes, 0.0% | 2 out of 6259 genes, 0.0% | 1 |
| production of molecular mediator involved in inflammatory response | 2 out of 6178 genes, 0.0% | 2 out of 6259 genes, 0.0% | 1 |
| regulation of myeloid leukocyte differentiation | 2 out of 6178 genes, 0.0% | 2 out of 6259 genes, 0.0% | 1 |
| antimicrobial peptide production | 2 out of 6178 genes, 0.0% | 2 out of 6259 genes, 0.0% | 1 |
| antimicrobial peptide biosynthetic process | 2 out of 6178 genes, 0.0% | 2 out of 6259 genes, 0.0% | 1 |
| antibacterial peptide production | 2 out of 6178 genes, 0.0% | 2 out of 6259 genes, 0.0% | 1 |
| antibacterial peptide biosynthetic process | 2 out of 6178 genes, 0.0% | 2 out of 6259 genes, 0.0% | 1 |
| involuntary skeletal muscle contraction | 2 out of 6178 genes, 0.0% | 2 out of 6259 genes, 0.0% | 1 |
| renal system process | 2 out of 6178 genes, 0.0% | 2 out of 6259 genes, 0.0% | 1 |
| regulation of systemic arterial blood pressure by renin-angiotensin | 2 out of 6178 genes, 0.0% | 2 out of 6259 genes, 0.0% | 1 |
| cardiac muscle hypertrophy | 2 out of 6178 genes, 0.0% | 2 out of 6259 genes, 0.0% | 1 |
| regulation of extracellular matrix constituent secretion | 2 out of 6178 genes, 0.0% | 2 out of 6259 genes, 0.0% | 1 |
| cilium movement | 2 out of 6178 genes, 0.0% | 2 out of 6259 genes, 0.0% | 1 |
| disaccharide metabolic process | 2 out of 6178 genes, 0.0% | 2 out of 6259 genes, 0.0% | 1 |
| D-ribose metabolic process | 2 out of 6178 genes, 0.0% | 2 out of 6259 genes, 0.0% | 1 |
| chitin metabolic process | 2 out of 6178 genes, 0.0% | 2 out of 6259 genes, 0.0% | 1 |
| uronic acid metabolic process | 2 out of 6178 genes, 0.0% | 2 out of 6259 genes, 0.0% | 1 |
| cellular aldehyde metabolic process | 2 out of 6178 genes, 0.0% | 2 out of 6259 genes, 0.0% | 1 |
| glycolysis | 2 out of 6178 genes, 0.0% | 2 out of 6259 genes, 0.0% | 1 |
| 2-oxoglutarate metabolic process | 2 out of 6178 genes, 0.0% | 2 out of 6259 genes, 0.0% | 1 |
| oxidative phosphorylation | 2 out of 6178 genes, 0.0% | 2 out of 6259 genes, 0.0% | 1 |
| pyrimidine nucleotide catabolic process | 2 out of 6178 genes, 0.0% | 2 out of 6259 genes, 0.0% | 1 |
| postreplication repair | 2 out of 6178 genes, 0.0% | 2 out of 6259 genes, 0.0% | 1 |
| mRNA splice site selection | 2 out of 6178 genes, 0.0% | 2 out of 6259 genes, 0.0% | 1 |
| mRNA cleavage | 2 out of 6178 genes, 0.0% | 2 out of 6259 genes, 0.0% | 1 |
| transcription from RNA polymerase III promoter | 2 out of 6178 genes, 0.0% | 2 out of 6259 genes, 0.0% | 1 |
| translational readthrough | 2 out of 6178 genes, 0.0% | 2 out of 6259 genes, 0.0% | 1 |
| protein dephosphorylation | 2 out of 6178 genes, 0.0% | 2 out of 6259 genes, 0.0% | 1 |
| protein ADP-ribosylation | 2 out of 6178 genes, 0.0% | 2 out of 6259 genes, 0.0% | 1 |
| N-terminal protein amino acid acetylation | 2 out of 6178 genes, 0.0% | 2 out of 6259 genes, 0.0% | 1 |
| N-terminal protein lipidation | 2 out of 6178 genes, 0.0% | 2 out of 6259 genes, 0.0% | 1 |
| glycoprotein catabolic process | 2 out of 6178 genes, 0.0% | 2 out of 6259 genes, 0.0% | 1 |
| aspartate metabolic process | 2 out of 6178 genes, 0.0% | 2 out of 6259 genes, 0.0% | 1 |
| glutamate catabolic process | 2 out of 6178 genes, 0.0% | 2 out of 6259 genes, 0.0% | 1 |
| tryptophan metabolic process | 2 out of 6178 genes, 0.0% | 2 out of 6259 genes, 0.0% | 1 |
| tryptophan catabolic process | 2 out of 6178 genes, 0.0% | 2 out of 6259 genes, 0.0% | 1 |
| polyamine catabolic process | 2 out of 6178 genes, 0.0% | 2 out of 6259 genes, 0.0% | 1 |
| sphinganine metabolic process | 2 out of 6178 genes, 0.0% | 2 out of 6259 genes, 0.0% | 1 |
| sphingosine metabolic process | 2 out of 6178 genes, 0.0% | 2 out of 6259 genes, 0.0% | 1 |
| prostanoid metabolic process | 2 out of 6178 genes, 0.0% | 2 out of 6259 genes, 0.0% | 1 |
| prostaglandin metabolic process | 2 out of 6178 genes, 0.0% | 2 out of 6259 genes, 0.0% | 1 |
| NADH metabolic process | 2 out of 6178 genes, 0.0% | 2 out of 6259 genes, 0.0% | 1 |
| thiamin metabolic process | 2 out of 6178 genes, 0.0% | 2 out of 6259 genes, 0.0% | 1 |
| xenobiotic metabolic process | 2 out of 6178 genes, 0.0% | 2 out of 6259 genes, 0.0% | 1 |
| copper ion transport | 2 out of 6178 genes, 0.0% | 2 out of 6259 genes, 0.0% | 1 |
| dicarboxylic acid transport | 2 out of 6178 genes, 0.0% | 2 out of 6259 genes, 0.0% | 1 |
| cell volume homeostasis | 2 out of 6178 genes, 0.0% | 2 out of 6259 genes, 0.0% | 1 |
| phagocytosis, engulfment | 2 out of 6178 genes, 0.0% | 2 out of 6259 genes, 0.0% | 1 |
| cellular component disassembly involved in apoptosis | 2 out of 6178 genes, 0.0% | 2 out of 6259 genes, 0.0% | 1 |
| response to unfolded protein | 2 out of 6178 genes, 0.0% | 2 out of 6259 genes, 0.0% | 1 |
| nuclear envelope organization | 2 out of 6178 genes, 0.0% | 2 out of 6259 genes, 0.0% | 1 |
| nucleolus organization | 2 out of 6178 genes, 0.0% | 2 out of 6259 genes, 0.0% | 1 |
| telomere maintenance via telomerase | 2 out of 6178 genes, 0.0% | 2 out of 6259 genes, 0.0% | 1 |
| inner mitochondrial membrane organization | 2 out of 6178 genes, 0.0% | 2 out of 6259 genes, 0.0% | 1 |
| lysosome organization | 2 out of 6178 genes, 0.0% | 2 out of 6259 genes, 0.0% | 1 |
| cell-substrate junction assembly | 2 out of 6178 genes, 0.0% | 2 out of 6259 genes, 0.0% | 1 |
| spindle assembly involved in female meiosis | 2 out of 6178 genes, 0.0% | 2 out of 6259 genes, 0.0% | 1 |
| sister chromatid cohesion | 2 out of 6178 genes, 0.0% | 2 out of 6259 genes, 0.0% | 1 |
| mitotic sister chromatid cohesion | 2 out of 6178 genes, 0.0% | 2 out of 6259 genes, 0.0% | 1 |
| mitotic cell cycle spindle assembly checkpoint | 2 out of 6178 genes, 0.0% | 2 out of 6259 genes, 0.0% | 1 |
| meiotic prophase I | 2 out of 6178 genes, 0.0% | 2 out of 6259 genes, 0.0% | 1 |
| reciprocal meiotic recombination | 2 out of 6178 genes, 0.0% | 2 out of 6259 genes, 0.0% | 1 |
| regulation of epidermal growth factor receptor activity | 2 out of 6178 genes, 0.0% | 2 out of 6259 genes, 0.0% | 1 |
| activation of phospholipase C activity by G-protein coupled receptor protein signaling pathway coupled to IP3 second messenger | 2 out of 6178 genes, 0.0% | 2 out of 6259 genes, 0.0% | 1 |
| activation of phospholipase C activity | 2 out of 6178 genes, 0.0% | 2 out of 6259 genes, 0.0% | 1 |
| elevation of cytosolic calcium ion concentration | 2 out of 6178 genes, 0.0% | 2 out of 6259 genes, 0.0% | 1 |
| cystoblast division | 2 out of 6178 genes, 0.0% | 2 out of 6259 genes, 0.0% | 1 |
| insemination | 2 out of 6178 genes, 0.0% | 2 out of 6259 genes, 0.0% | 1 |
| single fertilization | 2 out of 6178 genes, 0.0% | 2 out of 6259 genes, 0.0% | 1 |
| respiratory gaseous exchange | 2 out of 6178 genes, 0.0% | 2 out of 6259 genes, 0.0% | 1 |
| digestion | 2 out of 6178 genes, 0.0% | 2 out of 6259 genes, 0.0% | 1 |
| molting cycle, chitin-based cuticle | 2 out of 6178 genes, 0.0% | 2 out of 6259 genes, 0.0% | 1 |
| spermidine metabolic process | 2 out of 6178 genes, 0.0% | 2 out of 6259 genes, 0.0% | 1 |
| isoprenoid biosynthetic process | 2 out of 6178 genes, 0.0% | 2 out of 6259 genes, 0.0% | 1 |
| larval chitin-based cuticle development | 2 out of 6178 genes, 0.0% | 2 out of 6259 genes, 0.0% | 1 |
| glutamine family amino acid catabolic process | 2 out of 6178 genes, 0.0% | 2 out of 6259 genes, 0.0% | 1 |
| aromatic amino acid family catabolic process | 2 out of 6178 genes, 0.0% | 2 out of 6259 genes, 0.0% | 1 |
| lipoic acid biosynthetic process | 2 out of 6178 genes, 0.0% | 2 out of 6259 genes, 0.0% | 1 |
| purine nucleoside diphosphate biosynthetic process | 2 out of 6178 genes, 0.0% | 2 out of 6259 genes, 0.0% | 1 |
| pyrimidine nucleoside diphosphate metabolic process | 2 out of 6178 genes, 0.0% | 2 out of 6259 genes, 0.0% | 1 |
| purine ribonucleoside diphosphate biosynthetic process | 2 out of 6178 genes, 0.0% | 2 out of 6259 genes, 0.0% | 1 |
| ribonucleoside diphosphate biosynthetic process | 2 out of 6178 genes, 0.0% | 2 out of 6259 genes, 0.0% | 1 |
| cyclic nucleotide biosynthetic process | 2 out of 6178 genes, 0.0% | 2 out of 6259 genes, 0.0% | 1 |
| pyrimidine deoxyribonucleoside triphosphate metabolic process | 2 out of 6178 genes, 0.0% | 2 out of 6259 genes, 0.0% | 1 |
| cellular response to starvation | 2 out of 6178 genes, 0.0% | 2 out of 6259 genes, 0.0% | 1 |
| protein secretion | 2 out of 6178 genes, 0.0% | 2 out of 6259 genes, 0.0% | 1 |
| response to xenobiotic stimulus | 2 out of 6178 genes, 0.0% | 2 out of 6259 genes, 0.0% | 1 |
| NAD biosynthetic process | 2 out of 6178 genes, 0.0% | 2 out of 6259 genes, 0.0% | 1 |
| response to fungus | 2 out of 6178 genes, 0.0% | 2 out of 6259 genes, 0.0% | 1 |
| carbohydrate utilization | 2 out of 6178 genes, 0.0% | 2 out of 6259 genes, 0.0% | 1 |
| photosynthetic electron transport chain | 2 out of 6178 genes, 0.0% | 2 out of 6259 genes, 0.0% | 1 |
| negative regulation of cell fate specification | 2 out of 6178 genes, 0.0% | 2 out of 6259 genes, 0.0% | 1 |
| negative regulation of cell fate commitment | 2 out of 6178 genes, 0.0% | 2 out of 6259 genes, 0.0% | 1 |
| regulation of calcium ion transport into cytosol | 2 out of 6178 genes, 0.0% | 2 out of 6259 genes, 0.0% | 1 |
| regulation of transposition | 2 out of 6178 genes, 0.0% | 2 out of 6259 genes, 0.0% | 1 |
| negative regulation of phosphorus metabolic process | 2 out of 6178 genes, 0.0% | 2 out of 6259 genes, 0.0% | 1 |
| regulation of lamellipodium assembly | 2 out of 6178 genes, 0.0% | 2 out of 6259 genes, 0.0% | 1 |
| regulation of plasma membrane long-chain fatty acid transport | 2 out of 6178 genes, 0.0% | 2 out of 6259 genes, 0.0% | 1 |
| regulation of myotube differentiation | 2 out of 6178 genes, 0.0% | 2 out of 6259 genes, 0.0% | 1 |
| telomere maintenance via telomere lengthening | 2 out of 6178 genes, 0.0% | 2 out of 6259 genes, 0.0% | 1 |
| regulation of triglyceride biosynthetic process | 2 out of 6178 genes, 0.0% | 2 out of 6259 genes, 0.0% | 1 |
| regulation of ubiquitin homeostasis | 2 out of 6178 genes, 0.0% | 2 out of 6259 genes, 0.0% | 1 |
| response to isoquinoline alkaloid | 2 out of 6178 genes, 0.0% | 2 out of 6259 genes, 0.0% | 1 |
| response to tropane | 2 out of 6178 genes, 0.0% | 2 out of 6259 genes, 0.0% | 1 |
| response to purine | 2 out of 6178 genes, 0.0% | 2 out of 6259 genes, 0.0% | 1 |
| muscle cell migration | 2 out of 6178 genes, 0.0% | 2 out of 6259 genes, 0.0% | 1 |
| muscle hypertrophy | 2 out of 6178 genes, 0.0% | 2 out of 6259 genes, 0.0% | 1 |
| striated muscle hypertrophy | 2 out of 6178 genes, 0.0% | 2 out of 6259 genes, 0.0% | 1 |
| smooth muscle cell migration | 2 out of 6178 genes, 0.0% | 2 out of 6259 genes, 0.0% | 1 |
| ferrous iron transport | 2 out of 6178 genes, 0.0% | 2 out of 6259 genes, 0.0% | 1 |
| quaternary ammonium group transport | 2 out of 6178 genes, 0.0% | 2 out of 6259 genes, 0.0% | 1 |
| acidic amino acid transport | 2 out of 6178 genes, 0.0% | 2 out of 6259 genes, 0.0% | 1 |
| basic amino acid transport | 2 out of 6178 genes, 0.0% | 2 out of 6259 genes, 0.0% | 1 |
| L-amino acid transport | 2 out of 6178 genes, 0.0% | 2 out of 6259 genes, 0.0% | 1 |
| fatty acid transport | 2 out of 6178 genes, 0.0% | 2 out of 6259 genes, 0.0% | 1 |
| photosynthesis | 2 out of 6178 genes, 0.0% | 2 out of 6259 genes, 0.0% | 1 |
| macroautophagy | 2 out of 6178 genes, 0.0% | 2 out of 6259 genes, 0.0% | 1 |
| peptide hormone processing | 2 out of 6178 genes, 0.0% | 2 out of 6259 genes, 0.0% | 1 |
| histone deacetylation | 2 out of 6178 genes, 0.0% | 2 out of 6259 genes, 0.0% | 1 |
| protein sumoylation | 2 out of 6178 genes, 0.0% | 2 out of 6259 genes, 0.0% | 1 |
| cytochrome complex assembly | 2 out of 6178 genes, 0.0% | 2 out of 6259 genes, 0.0% | 1 |
| respiratory chain complex III assembly | 2 out of 6178 genes, 0.0% | 2 out of 6259 genes, 0.0% | 1 |
| negative regulation of translation | 2 out of 6178 genes, 0.0% | 2 out of 6259 genes, 0.0% | 1 |
| regulation of calcium ion-dependent exocytosis | 2 out of 6178 genes, 0.0% | 2 out of 6259 genes, 0.0% | 1 |
| peptidyl-lysine methylation | 2 out of 6178 genes, 0.0% | 2 out of 6259 genes, 0.0% | 1 |
| protein-cofactor linkage | 2 out of 6178 genes, 0.0% | 2 out of 6259 genes, 0.0% | 1 |
| peptidyl-cysteine modification | 2 out of 6178 genes, 0.0% | 2 out of 6259 genes, 0.0% | 1 |
| peptidyl-glutamic acid modification | 2 out of 6178 genes, 0.0% | 2 out of 6259 genes, 0.0% | 1 |
| viral genome replication | 2 out of 6178 genes, 0.0% | 2 out of 6259 genes, 0.0% | 1 |
| regulation of steroid metabolic process | 2 out of 6178 genes, 0.0% | 2 out of 6259 genes, 0.0% | 1 |
| aromatic compound catabolic process | 2 out of 6178 genes, 0.0% | 2 out of 6259 genes, 0.0% | 1 |
| aldonic acid metabolic process | 2 out of 6178 genes, 0.0% | 2 out of 6259 genes, 0.0% | 1 |
| glucuronate metabolic process | 2 out of 6178 genes, 0.0% | 2 out of 6259 genes, 0.0% | 1 |
| photosynthesis, light reaction | 2 out of 6178 genes, 0.0% | 2 out of 6259 genes, 0.0% | 1 |
| antimicrobial humoral response | 2 out of 6178 genes, 0.0% | 2 out of 6259 genes, 0.0% | 1 |
| antibacterial humoral response | 2 out of 6178 genes, 0.0% | 2 out of 6259 genes, 0.0% | 1 |
| L-ascorbic acid metabolic process | 2 out of 6178 genes, 0.0% | 2 out of 6259 genes, 0.0% | 1 |
| medulla oblongata development | 2 out of 6178 genes, 0.0% | 2 out of 6259 genes, 0.0% | 1 |
| pyramidal neuron differentiation | 2 out of 6178 genes, 0.0% | 2 out of 6259 genes, 0.0% | 1 |
| forebrain neuron differentiation | 2 out of 6178 genes, 0.0% | 2 out of 6259 genes, 0.0% | 1 |
| central nervous system projection neuron axonogenesis | 2 out of 6178 genes, 0.0% | 2 out of 6259 genes, 0.0% | 1 |
| negative adaptation of signaling pathway | 2 out of 6178 genes, 0.0% | 2 out of 6259 genes, 0.0% | 1 |
| positive regulation of cell-cell adhesion | 2 out of 6178 genes, 0.0% | 2 out of 6259 genes, 0.0% | 1 |
| adaptation of signaling pathway | 2 out of 6178 genes, 0.0% | 2 out of 6259 genes, 0.0% | 1 |
| cellular monovalent inorganic cation homeostasis | 2 out of 6178 genes, 0.0% | 2 out of 6259 genes, 0.0% | 1 |
| positive regulation of cell growth | 2 out of 6178 genes, 0.0% | 2 out of 6259 genes, 0.0% | 1 |
| negative regulation of cell growth | 2 out of 6178 genes, 0.0% | 2 out of 6259 genes, 0.0% | 1 |
| DNA damage response, signal transduction by p53 class mediator | 2 out of 6178 genes, 0.0% | 2 out of 6259 genes, 0.0% | 1 |
| estrogen receptor signaling pathway | 2 out of 6178 genes, 0.0% | 2 out of 6259 genes, 0.0% | 1 |
| regulation of cellular pH | 2 out of 6178 genes, 0.0% | 2 out of 6259 genes, 0.0% | 1 |
| negative regulation of actin filament polymerization | 2 out of 6178 genes, 0.0% | 2 out of 6259 genes, 0.0% | 1 |
| polarized epithelial cell differentiation | 2 out of 6178 genes, 0.0% | 2 out of 6259 genes, 0.0% | 1 |
| regulation of B cell proliferation | 2 out of 6178 genes, 0.0% | 2 out of 6259 genes, 0.0% | 1 |
| myosin filament assembly | 2 out of 6178 genes, 0.0% | 2 out of 6259 genes, 0.0% | 1 |
| neuron projection regeneration | 2 out of 6178 genes, 0.0% | 2 out of 6259 genes, 0.0% | 1 |
| axon regeneration | 2 out of 6178 genes, 0.0% | 2 out of 6259 genes, 0.0% | 1 |
| developmental induction | 2 out of 6178 genes, 0.0% | 2 out of 6259 genes, 0.0% | 1 |
| negative regulation of protein complex assembly | 2 out of 6178 genes, 0.0% | 2 out of 6259 genes, 0.0% | 1 |
| negative regulation of defense response | 2 out of 6178 genes, 0.0% | 2 out of 6259 genes, 0.0% | 1 |
| negative regulation of protein modification process | 2 out of 6178 genes, 0.0% | 2 out of 6259 genes, 0.0% | 1 |
| fast-twitch skeletal muscle fiber contraction | 2 out of 6178 genes, 0.0% | 2 out of 6259 genes, 0.0% | 1 |
| G2/M transition DNA damage checkpoint | 2 out of 6178 genes, 0.0% | 2 out of 6259 genes, 0.0% | 1 |
| G2/M transition checkpoint | 2 out of 6178 genes, 0.0% | 2 out of 6259 genes, 0.0% | 1 |
| spindle checkpoint | 2 out of 6178 genes, 0.0% | 2 out of 6259 genes, 0.0% | 1 |
| negative regulation of neurological system process | 2 out of 6178 genes, 0.0% | 2 out of 6259 genes, 0.0% | 1 |
| positive regulation of neurological system process | 2 out of 6178 genes, 0.0% | 2 out of 6259 genes, 0.0% | 1 |
| negative regulation of translation in response to stress | 2 out of 6178 genes, 0.0% | 2 out of 6259 genes, 0.0% | 1 |
| response to food | 2 out of 6178 genes, 0.0% | 2 out of 6259 genes, 0.0% | 1 |
| regulation of actin filament bundle assembly | 2 out of 6178 genes, 0.0% | 2 out of 6259 genes, 0.0% | 1 |
| positive regulation of actin filament bundle assembly | 2 out of 6178 genes, 0.0% | 2 out of 6259 genes, 0.0% | 1 |
| negative regulation of protein polymerization | 2 out of 6178 genes, 0.0% | 2 out of 6259 genes, 0.0% | 1 |
| endoplasmic reticulum calcium ion homeostasis | 2 out of 6178 genes, 0.0% | 2 out of 6259 genes, 0.0% | 1 |
| response to retinoic acid | 2 out of 6178 genes, 0.0% | 2 out of 6259 genes, 0.0% | 1 |
| regulation of chemokine production | 2 out of 6178 genes, 0.0% | 2 out of 6259 genes, 0.0% | 1 |
| positive regulation of chemokine production | 2 out of 6178 genes, 0.0% | 2 out of 6259 genes, 0.0% | 1 |
| negative regulation of monooxygenase activity | 2 out of 6178 genes, 0.0% | 2 out of 6259 genes, 0.0% | 1 |
| regulation of stress-activated MAPK cascade | 2 out of 6178 genes, 0.0% | 2 out of 6259 genes, 0.0% | 1 |
| negative regulation of viral transcription | 2 out of 6178 genes, 0.0% | 2 out of 6259 genes, 0.0% | 1 |
| directional locomotion | 2 out of 6178 genes, 0.0% | 2 out of 6259 genes, 0.0% | 1 |
| regulation of protein import into nucleus, translocation | 2 out of 6178 genes, 0.0% | 2 out of 6259 genes, 0.0% | 1 |
| response to ATP | 2 out of 6178 genes, 0.0% | 2 out of 6259 genes, 0.0% | 1 |
| mammary gland epithelial cell proliferation | 2 out of 6178 genes, 0.0% | 2 out of 6259 genes, 0.0% | 1 |
| membrane protein proteolysis | 2 out of 6178 genes, 0.0% | 2 out of 6259 genes, 0.0% | 1 |
| cellular response to heat | 2 out of 6178 genes, 0.0% | 2 out of 6259 genes, 0.0% | 1 |
| negative regulation of transmembrane transport | 2 out of 6178 genes, 0.0% | 2 out of 6259 genes, 0.0% | 1 |
| histone arginine methylation | 2 out of 6178 genes, 0.0% | 2 out of 6259 genes, 0.0% | 1 |
| leading edge cell differentiation | 2 out of 6178 genes, 0.0% | 2 out of 6259 genes, 0.0% | 1 |
| tail morphogenesis | 2 out of 6178 genes, 0.0% | 2 out of 6259 genes, 0.0% | 1 |
| haltere disc development | 2 out of 6178 genes, 0.0% | 2 out of 6259 genes, 0.0% | 1 |
| peptidyl-arginine N-methylation | 2 out of 6178 genes, 0.0% | 2 out of 6259 genes, 0.0% | 1 |
| peptidyl-arginine omega-N-methylation | 2 out of 6178 genes, 0.0% | 2 out of 6259 genes, 0.0% | 1 |
| pronephric nephron development | 2 out of 6178 genes, 0.0% | 2 out of 6259 genes, 0.0% | 1 |
| dauer larval development | 2 out of 6178 genes, 0.0% | 2 out of 6259 genes, 0.0% | 1 |
| protein refolding | 2 out of 6178 genes, 0.0% | 2 out of 6259 genes, 0.0% | 1 |
| regulation of cytokine biosynthetic process | 2 out of 6178 genes, 0.0% | 2 out of 6259 genes, 0.0% | 1 |
| regulation of dopamine metabolic process | 2 out of 6178 genes, 0.0% | 2 out of 6259 genes, 0.0% | 1 |
| regulation of epidermal growth factor receptor signaling pathway | 2 out of 6178 genes, 0.0% | 2 out of 6259 genes, 0.0% | 1 |
| glial cell growth | 2 out of 6178 genes, 0.0% | 2 out of 6259 genes, 0.0% | 1 |
| neurotransmitter biosynthetic process | 2 out of 6178 genes, 0.0% | 2 out of 6259 genes, 0.0% | 1 |
| tissue regeneration | 2 out of 6178 genes, 0.0% | 2 out of 6259 genes, 0.0% | 1 |
| negative regulation of protein import into nucleus | 2 out of 6178 genes, 0.0% | 2 out of 6259 genes, 0.0% | 1 |
| vasoconstriction | 2 out of 6178 genes, 0.0% | 2 out of 6259 genes, 0.0% | 1 |
| vasodilation | 2 out of 6178 genes, 0.0% | 2 out of 6259 genes, 0.0% | 1 |
| negative regulation of phosphorylation | 2 out of 6178 genes, 0.0% | 2 out of 6259 genes, 0.0% | 1 |
| cuticle development involved in protein-based cuticle molting cycle | 2 out of 6178 genes, 0.0% | 2 out of 6259 genes, 0.0% | 1 |
| cuticle development involved in chitin-based cuticle molting cycle | 2 out of 6178 genes, 0.0% | 2 out of 6259 genes, 0.0% | 1 |
| cilium assembly | 2 out of 6178 genes, 0.0% | 2 out of 6259 genes, 0.0% | 1 |
| indole derivative catabolic process | 2 out of 6178 genes, 0.0% | 2 out of 6259 genes, 0.0% | 1 |
| hormone biosynthetic process | 2 out of 6178 genes, 0.0% | 2 out of 6259 genes, 0.0% | 1 |
| serine phosphorylation of STAT protein | 2 out of 6178 genes, 0.0% | 2 out of 6259 genes, 0.0% | 1 |
| pteridine and derivative metabolic process | 2 out of 6178 genes, 0.0% | 2 out of 6259 genes, 0.0% | 1 |
| response to starvation | 2 out of 6178 genes, 0.0% | 2 out of 6259 genes, 0.0% | 1 |
| regulation of cell fate specification | 2 out of 6178 genes, 0.0% | 2 out of 6259 genes, 0.0% | 1 |
| tRNA 3'-end processing | 2 out of 6178 genes, 0.0% | 2 out of 6259 genes, 0.0% | 1 |
| negative regulation of transcription factor import into nucleus | 2 out of 6178 genes, 0.0% | 2 out of 6259 genes, 0.0% | 1 |
| cellular metabolic compound salvage | 2 out of 6178 genes, 0.0% | 2 out of 6259 genes, 0.0% | 1 |
| purine base salvage | 2 out of 6178 genes, 0.0% | 2 out of 6259 genes, 0.0% | 1 |
| purine salvage | 2 out of 6178 genes, 0.0% | 2 out of 6259 genes, 0.0% | 1 |
| response to alkaloid | 2 out of 6178 genes, 0.0% | 2 out of 6259 genes, 0.0% | 1 |
| positive T cell selection | 2 out of 6178 genes, 0.0% | 2 out of 6259 genes, 0.0% | 1 |
| regulation of RNA splicing | 2 out of 6178 genes, 0.0% | 2 out of 6259 genes, 0.0% | 1 |
| regulation of translation in response to stress | 2 out of 6178 genes, 0.0% | 2 out of 6259 genes, 0.0% | 1 |
| ncRNA 3'-end processing | 2 out of 6178 genes, 0.0% | 2 out of 6259 genes, 0.0% | 1 |
| regulation of DNA methylation | 2 out of 6178 genes, 0.0% | 2 out of 6259 genes, 0.0% | 1 |
| multicellular organismal metabolic process | 2 out of 6178 genes, 0.0% | 2 out of 6259 genes, 0.0% | 1 |
| multicellular organismal macromolecule metabolic process | 2 out of 6178 genes, 0.0% | 2 out of 6259 genes, 0.0% | 1 |
| symbiosis, encompassing mutualism through parasitism | 2 out of 6178 genes, 0.0% | 2 out of 6259 genes, 0.0% | 1 |
| T cell selection | 2 out of 6178 genes, 0.0% | 2 out of 6259 genes, 0.0% | 1 |
| meiotic chromosome segregation | 2 out of 6178 genes, 0.0% | 2 out of 6259 genes, 0.0% | 1 |
| otolith mineralization | 2 out of 6178 genes, 0.0% | 2 out of 6259 genes, 0.0% | 1 |
| positive regulation of myeloid cell differentiation | 2 out of 6178 genes, 0.0% | 2 out of 6259 genes, 0.0% | 1 |
| regulation of anti-apoptosis | 2 out of 6178 genes, 0.0% | 2 out of 6259 genes, 0.0% | 1 |
| positive regulation of cell adhesion | 2 out of 6178 genes, 0.0% | 2 out of 6259 genes, 0.0% | 1 |
| negative regulation of cell size | 2 out of 6178 genes, 0.0% | 2 out of 6259 genes, 0.0% | 1 |
| positive regulation of cell size | 2 out of 6178 genes, 0.0% | 2 out of 6259 genes, 0.0% | 1 |
| positive regulation of transcription, DNA-dependent | 2 out of 6178 genes, 0.0% | 2 out of 6259 genes, 0.0% | 1 |
| regulation of transcription during mitosis | 2 out of 6178 genes, 0.0% | 2 out of 6259 genes, 0.0% | 1 |
| positive regulation of transcription during mitosis | 2 out of 6178 genes, 0.0% | 2 out of 6259 genes, 0.0% | 1 |
| negative regulation of phosphate metabolic process | 2 out of 6178 genes, 0.0% | 2 out of 6259 genes, 0.0% | 1 |
| carbon catabolite regulation of transcription | 2 out of 6178 genes, 0.0% | 2 out of 6259 genes, 0.0% | 1 |
| spermidine catabolic process | 2 out of 6178 genes, 0.0% | 2 out of 6259 genes, 0.0% | 1 |
| indolalkylamine catabolic process | 2 out of 6178 genes, 0.0% | 2 out of 6259 genes, 0.0% | 1 |
| S-adenosylmethionine metabolic process | 2 out of 6178 genes, 0.0% | 2 out of 6259 genes, 0.0% | 1 |
| regulation of organ growth | 2 out of 6178 genes, 0.0% | 2 out of 6259 genes, 0.0% | 1 |
| regulation of oviposition | 2 out of 6178 genes, 0.0% | 2 out of 6259 genes, 0.0% | 1 |
| response to arsenic | 2 out of 6178 genes, 0.0% | 2 out of 6259 genes, 0.0% | 1 |
| regulation of viral transcription | 2 out of 6178 genes, 0.0% | 2 out of 6259 genes, 0.0% | 1 |
| antigen processing and presentation of peptide antigen | 2 out of 6178 genes, 0.0% | 2 out of 6259 genes, 0.0% | 1 |
| phosphoinositide-mediated signaling | 2 out of 6178 genes, 0.0% | 2 out of 6259 genes, 0.0% | 1 |
| regulation of cuticle pigmentation | 2 out of 6178 genes, 0.0% | 2 out of 6259 genes, 0.0% | 1 |
| male germ-line cyst formation | 2 out of 6178 genes, 0.0% | 2 out of 6259 genes, 0.0% | 1 |
| mitochondrion distribution | 2 out of 6178 genes, 0.0% | 2 out of 6259 genes, 0.0% | 1 |
| synaptic vesicle endocytosis | 2 out of 6178 genes, 0.0% | 2 out of 6259 genes, 0.0% | 1 |
| digestive tract development | 2 out of 6178 genes, 0.0% | 2 out of 6259 genes, 0.0% | 1 |
| muscle organ morphogenesis | 2 out of 6178 genes, 0.0% | 2 out of 6259 genes, 0.0% | 1 |
| response to axon injury | 2 out of 6178 genes, 0.0% | 2 out of 6259 genes, 0.0% | 1 |
| otolith development | 2 out of 6178 genes, 0.0% | 2 out of 6259 genes, 0.0% | 1 |
| lateral line nerve glial cell differentiation | 2 out of 6178 genes, 0.0% | 2 out of 6259 genes, 0.0% | 1 |
| myelination of lateral line nerve axons | 2 out of 6178 genes, 0.0% | 2 out of 6259 genes, 0.0% | 1 |
| lateral line nerve glial cell development | 2 out of 6178 genes, 0.0% | 2 out of 6259 genes, 0.0% | 1 |
| lateral line nerve glial cell morphogenesis involved in differentiation | 2 out of 6178 genes, 0.0% | 2 out of 6259 genes, 0.0% | 1 |
| cytokine secretion | 2 out of 6178 genes, 0.0% | 2 out of 6259 genes, 0.0% | 1 |
| negative regulation of synaptic transmission | 2 out of 6178 genes, 0.0% | 2 out of 6259 genes, 0.0% | 1 |
| positive regulation of synaptic transmission | 2 out of 6178 genes, 0.0% | 2 out of 6259 genes, 0.0% | 1 |
| endocrine process | 2 out of 6178 genes, 0.0% | 2 out of 6259 genes, 0.0% | 1 |
| sensory perception of taste | 2 out of 6178 genes, 0.0% | 2 out of 6259 genes, 0.0% | 1 |
| regulation of chemotaxis | 2 out of 6178 genes, 0.0% | 2 out of 6259 genes, 0.0% | 1 |
| regulation of pigment cell differentiation | 2 out of 6178 genes, 0.0% | 2 out of 6259 genes, 0.0% | 1 |
| detection of mechanical stimulus involved in sensory perception | 2 out of 6178 genes, 0.0% | 2 out of 6259 genes, 0.0% | 1 |
| detection of mechanical stimulus | 2 out of 6178 genes, 0.0% | 2 out of 6259 genes, 0.0% | 1 |
| positive regulation of secretion | 2 out of 6178 genes, 0.0% | 2 out of 6259 genes, 0.0% | 1 |
| regulation of helicase activity | 2 out of 6178 genes, 0.0% | 2 out of 6259 genes, 0.0% | 1 |
| negative regulation of protein transport | 2 out of 6178 genes, 0.0% | 2 out of 6259 genes, 0.0% | 1 |
| positive regulation of RNA metabolic process | 2 out of 6178 genes, 0.0% | 2 out of 6259 genes, 0.0% | 1 |
| prophase | 2 out of 6178 genes, 0.0% | 2 out of 6259 genes, 0.0% | 1 |
| positive regulation of ligase activity | 2 out of 6178 genes, 0.0% | 2 out of 6259 genes, 0.0% | 1 |
| negative regulation of oxidoreductase activity | 2 out of 6178 genes, 0.0% | 2 out of 6259 genes, 0.0% | 1 |
| response to cortisol stimulus | 2 out of 6178 genes, 0.0% | 2 out of 6259 genes, 0.0% | 1 |
| positive regulation of ubiquitin-protein ligase activity | 2 out of 6178 genes, 0.0% | 2 out of 6259 genes, 0.0% | 1 |
| mitochondrial calcium ion homeostasis | 2 out of 6178 genes, 0.0% | 2 out of 6259 genes, 0.0% | 1 |
| mitochondrion localization | 2 out of 6178 genes, 0.0% | 2 out of 6259 genes, 0.0% | 1 |
| actin filament capping | 2 out of 6178 genes, 0.0% | 2 out of 6259 genes, 0.0% | 1 |
| cell cycle switching, mitotic to meiotic cell cycle | 2 out of 6178 genes, 0.0% | 2 out of 6259 genes, 0.0% | 1 |
| negative regulation of transmission of nerve impulse | 2 out of 6178 genes, 0.0% | 2 out of 6259 genes, 0.0% | 1 |
| positive regulation of transmission of nerve impulse | 2 out of 6178 genes, 0.0% | 2 out of 6259 genes, 0.0% | 1 |
| cardiac cell development | 2 out of 6178 genes, 0.0% | 2 out of 6259 genes, 0.0% | 1 |
| cardiac muscle cell differentiation | 2 out of 6178 genes, 0.0% | 2 out of 6259 genes, 0.0% | 1 |
| cardiac muscle tissue morphogenesis | 2 out of 6178 genes, 0.0% | 2 out of 6259 genes, 0.0% | 1 |
| cardiac muscle cell development | 2 out of 6178 genes, 0.0% | 2 out of 6259 genes, 0.0% | 1 |
| regulation of cardiac muscle contraction | 2 out of 6178 genes, 0.0% | 2 out of 6259 genes, 0.0% | 1 |
| digestive system development | 2 out of 6178 genes, 0.0% | 2 out of 6259 genes, 0.0% | 1 |
| inner ear receptor cell development | 2 out of 6178 genes, 0.0% | 2 out of 6259 genes, 0.0% | 1 |
| inner ear receptor stereocilium organization | 2 out of 6178 genes, 0.0% | 2 out of 6259 genes, 0.0% | 1 |
| cell cycle switching | 2 out of 6178 genes, 0.0% | 2 out of 6259 genes, 0.0% | 1 |
| cilium morphogenesis | 2 out of 6178 genes, 0.0% | 2 out of 6259 genes, 0.0% | 1 |
| trabecula formation | 2 out of 6178 genes, 0.0% | 2 out of 6259 genes, 0.0% | 1 |
| muscle tissue morphogenesis | 2 out of 6178 genes, 0.0% | 2 out of 6259 genes, 0.0% | 1 |
| dichotomous subdivision of an epithelial terminal unit | 2 out of 6178 genes, 0.0% | 2 out of 6259 genes, 0.0% | 1 |
| regulation of protein tyrosine kinase activity | 2 out of 6178 genes, 0.0% | 2 out of 6259 genes, 0.0% | 1 |
| regulation of stress-activated protein kinase signaling cascade | 2 out of 6178 genes, 0.0% | 2 out of 6259 genes, 0.0% | 1 |
| actin polymerization-dependent cell motility | 2 out of 6178 genes, 0.0% | 2 out of 6259 genes, 0.0% | 1 |
| calcium ion transmembrane transport | 2 out of 6178 genes, 0.0% | 2 out of 6259 genes, 0.0% | 1 |
| inclusion body assembly | 2 out of 6178 genes, 0.0% | 2 out of 6259 genes, 0.0% | 1 |
| misfolded protein transport | 2 out of 6178 genes, 0.0% | 2 out of 6259 genes, 0.0% | 1 |
| response to epidermal growth factor stimulus | 2 out of 6178 genes, 0.0% | 2 out of 6259 genes, 0.0% | 1 |
| spindle assembly checkpoint | 2 out of 6178 genes, 0.0% | 2 out of 6259 genes, 0.0% | 1 |
| mitotic cell cycle spindle checkpoint | 2 out of 6178 genes, 0.0% | 2 out of 6259 genes, 0.0% | 1 |
| cellular response to xenobiotic stimulus | 2 out of 6178 genes, 0.0% | 2 out of 6259 genes, 0.0% | 1 |
| negative regulation of intracellular protein transport | 2 out of 6178 genes, 0.0% | 2 out of 6259 genes, 0.0% | 1 |
| negative regulation of reproductive process | 2 out of 6178 genes, 0.0% | 2 out of 6259 genes, 0.0% | 1 |
| tetrapyrrole metabolic process | 18 out of 6178 genes, 0.3% | 19 out of 6259 genes, 0.3% | 1 |
| nicotinamide nucleotide metabolic process | 18 out of 6178 genes, 0.3% | 19 out of 6259 genes, 0.3% | 1 |
| positive regulation of binding | 18 out of 6178 genes, 0.3% | 19 out of 6259 genes, 0.3% | 1 |
| nucleoside triphosphate biosynthetic process | 82 out of 6178 genes, 1.3% | 85 out of 6259 genes, 1.4% | 1 |
| ribonucleoside triphosphate biosynthetic process | 82 out of 6178 genes, 1.3% | 85 out of 6259 genes, 1.4% | 1 |
| regulation of cellular protein metabolic process | 82 out of 6178 genes, 1.3% | 85 out of 6259 genes, 1.4% | 1 |
| hemopoiesis | 46 out of 6178 genes, 0.7% | 48 out of 6259 genes, 0.8% | 1 |
| negative regulation of metabolic process | 81 out of 6178 genes, 1.3% | 84 out of 6259 genes, 1.3% | 1 |
| positive regulation of cellular metabolic process | 45 out of 6178 genes, 0.7% | 47 out of 6259 genes, 0.8% | 1 |
| porphyrin metabolic process | 17 out of 6178 genes, 0.3% | 18 out of 6259 genes, 0.3% | 1 |
| positive regulation of catabolic process | 17 out of 6178 genes, 0.3% | 18 out of 6259 genes, 0.3% | 1 |
| positive regulation of signal transduction | 17 out of 6178 genes, 0.3% | 18 out of 6259 genes, 0.3% | 1 |
| positive regulation of signaling process | 17 out of 6178 genes, 0.3% | 18 out of 6259 genes, 0.3% | 1 |
| regulation of protein import into nucleus | 17 out of 6178 genes, 0.3% | 18 out of 6259 genes, 0.3% | 1 |
| autophagic cell death | 17 out of 6178 genes, 0.3% | 18 out of 6259 genes, 0.3% | 1 |
| compound eye development | 17 out of 6178 genes, 0.3% | 18 out of 6259 genes, 0.3% | 1 |
| cofactor metabolic process | 78 out of 6178 genes, 1.3% | 81 out of 6259 genes, 1.3% | 1 |
| response to reactive oxygen species | 16 out of 6178 genes, 0.3% | 17 out of 6259 genes, 0.3% | 1 |
| compound eye morphogenesis | 16 out of 6178 genes, 0.3% | 17 out of 6259 genes, 0.3% | 1 |
| response to nutrient | 16 out of 6178 genes, 0.3% | 17 out of 6259 genes, 0.3% | 1 |
| positive regulation of cell proliferation | 16 out of 6178 genes, 0.3% | 17 out of 6259 genes, 0.3% | 1 |
| positive regulation of intracellular protein kinase cascade | 16 out of 6178 genes, 0.3% | 17 out of 6259 genes, 0.3% | 1 |
| alditol metabolic process | 16 out of 6178 genes, 0.3% | 17 out of 6259 genes, 0.3% | 1 |
| maintenance of location in cell | 16 out of 6178 genes, 0.3% | 17 out of 6259 genes, 0.3% | 1 |
| purine nucleoside triphosphate biosynthetic process | 77 out of 6178 genes, 1.2% | 80 out of 6259 genes, 1.3% | 1 |
| purine ribonucleoside triphosphate biosynthetic process | 77 out of 6178 genes, 1.2% | 80 out of 6259 genes, 1.3% | 1 |
| positive regulation of intracellular transport | 15 out of 6178 genes, 0.2% | 16 out of 6259 genes, 0.3% | 1 |
| maintenance of protein location | 15 out of 6178 genes, 0.2% | 16 out of 6259 genes, 0.3% | 1 |
| positive regulation of nucleocytoplasmic transport | 15 out of 6178 genes, 0.2% | 16 out of 6259 genes, 0.3% | 1 |
| vitamin metabolic process | 14 out of 6178 genes, 0.2% | 15 out of 6259 genes, 0.2% | 1 |
| water-soluble vitamin metabolic process | 14 out of 6178 genes, 0.2% | 15 out of 6259 genes, 0.2% | 1 |
| I-kappaB kinase/NF-kappaB cascade | 14 out of 6178 genes, 0.2% | 15 out of 6259 genes, 0.2% | 1 |
| carbohydrate transport | 14 out of 6178 genes, 0.2% | 15 out of 6259 genes, 0.2% | 1 |
| regulation of Wnt receptor signaling pathway | 14 out of 6178 genes, 0.2% | 15 out of 6259 genes, 0.2% | 1 |
| regulation of protein stability | 14 out of 6178 genes, 0.2% | 15 out of 6259 genes, 0.2% | 1 |
| cuticle development | 14 out of 6178 genes, 0.2% | 15 out of 6259 genes, 0.2% | 1 |
| regulation of protein modification process | 69 out of 6178 genes, 1.1% | 72 out of 6259 genes, 1.2% | 1 |
| G-protein signaling, coupled to cyclic nucleotide second messenger | 13 out of 6178 genes, 0.2% | 14 out of 6259 genes, 0.2% | 1 |
| cyclic-nucleotide-mediated signaling | 13 out of 6178 genes, 0.2% | 14 out of 6259 genes, 0.2% | 1 |
| maintenance of protein location in cell | 13 out of 6178 genes, 0.2% | 14 out of 6259 genes, 0.2% | 1 |
| cellular component maintenance | 13 out of 6178 genes, 0.2% | 14 out of 6259 genes, 0.2% | 1 |
| regulation of synaptic plasticity | 13 out of 6178 genes, 0.2% | 14 out of 6259 genes, 0.2% | 1 |
| regulation of binding | 36 out of 6178 genes, 0.6% | 38 out of 6259 genes, 0.6% | 1 |
| compound eye photoreceptor cell differentiation | 12 out of 6178 genes, 0.2% | 13 out of 6259 genes, 0.2% | 1 |
| porphyrin biosynthetic process | 12 out of 6178 genes, 0.2% | 13 out of 6259 genes, 0.2% | 1 |
| mononuclear cell proliferation | 12 out of 6178 genes, 0.2% | 13 out of 6259 genes, 0.2% | 1 |
| tetrapyrrole biosynthetic process | 12 out of 6178 genes, 0.2% | 13 out of 6259 genes, 0.2% | 1 |
| leukocyte proliferation | 12 out of 6178 genes, 0.2% | 13 out of 6259 genes, 0.2% | 1 |
| ATP metabolic process | 34 out of 6178 genes, 0.6% | 36 out of 6259 genes, 0.6% | 1 |
| regulation of small GTPase mediated signal transduction | 33 out of 6178 genes, 0.5% | 35 out of 6259 genes, 0.6% | 1 |
| ceramide metabolic process | 11 out of 6178 genes, 0.2% | 12 out of 6259 genes, 0.2% | 1 |
| circadian rhythm | 11 out of 6178 genes, 0.2% | 12 out of 6259 genes, 0.2% | 1 |
| amine biosynthetic process | 11 out of 6178 genes, 0.2% | 12 out of 6259 genes, 0.2% | 1 |
| positive regulation of cellular catabolic process | 11 out of 6178 genes, 0.2% | 12 out of 6259 genes, 0.2% | 1 |
| positive regulation of transmembrane transport | 11 out of 6178 genes, 0.2% | 12 out of 6259 genes, 0.2% | 1 |
| positive regulation of protein import into nucleus | 11 out of 6178 genes, 0.2% | 12 out of 6259 genes, 0.2% | 1 |
| positive regulation of DNA binding | 11 out of 6178 genes, 0.2% | 12 out of 6259 genes, 0.2% | 1 |
| negative regulation of binding | 11 out of 6178 genes, 0.2% | 12 out of 6259 genes, 0.2% | 1 |
| positive regulation of protein transport | 11 out of 6178 genes, 0.2% | 12 out of 6259 genes, 0.2% | 1 |
| positive regulation of intracellular protein transport | 11 out of 6178 genes, 0.2% | 12 out of 6259 genes, 0.2% | 1 |
| ATP biosynthetic process | 32 out of 6178 genes, 0.5% | 34 out of 6259 genes, 0.5% | 1 |
| hydrogen transport | 32 out of 6178 genes, 0.5% | 34 out of 6259 genes, 0.5% | 1 |
| regulation of protein phosphorylation | 31 out of 6178 genes, 0.5% | 33 out of 6259 genes, 0.5% | 1 |
| regulation of Ras protein signal transduction | 31 out of 6178 genes, 0.5% | 33 out of 6259 genes, 0.5% | 1 |
| regulation of cyclase activity | 10 out of 6178 genes, 0.2% | 11 out of 6259 genes, 0.2% | 1 |
| response to vitamin | 10 out of 6178 genes, 0.2% | 11 out of 6259 genes, 0.2% | 1 |
| regulation of lyase activity | 10 out of 6178 genes, 0.2% | 11 out of 6259 genes, 0.2% | 1 |
| defense response | 29 out of 6178 genes, 0.5% | 31 out of 6259 genes, 0.5% | 1 |
| positive regulation of cell communication | 29 out of 6178 genes, 0.5% | 31 out of 6259 genes, 0.5% | 1 |
| oxygen and reactive oxygen species metabolic process | 9 out of 6178 genes, 0.1% | 10 out of 6259 genes, 0.2% | 1 |
| G-protein signaling, coupled to cAMP nucleotide second messenger | 9 out of 6178 genes, 0.1% | 10 out of 6259 genes, 0.2% | 1 |
| cAMP-mediated signaling | 9 out of 6178 genes, 0.1% | 10 out of 6259 genes, 0.2% | 1 |
| regulation of cyclic nucleotide metabolic process | 9 out of 6178 genes, 0.1% | 10 out of 6259 genes, 0.2% | 1 |
| regulation of cyclic nucleotide biosynthetic process | 9 out of 6178 genes, 0.1% | 10 out of 6259 genes, 0.2% | 1 |
| regulation of nucleotide biosynthetic process | 9 out of 6178 genes, 0.1% | 10 out of 6259 genes, 0.2% | 1 |
| regulation of cAMP metabolic process | 9 out of 6178 genes, 0.1% | 10 out of 6259 genes, 0.2% | 1 |
| regulation of cAMP biosynthetic process | 9 out of 6178 genes, 0.1% | 10 out of 6259 genes, 0.2% | 1 |
| T cell proliferation | 9 out of 6178 genes, 0.1% | 10 out of 6259 genes, 0.2% | 1 |
| pigment metabolic process | 9 out of 6178 genes, 0.1% | 10 out of 6259 genes, 0.2% | 1 |
| regulation of adenylate cyclase activity | 9 out of 6178 genes, 0.1% | 10 out of 6259 genes, 0.2% | 1 |
| lymphocyte proliferation | 9 out of 6178 genes, 0.1% | 10 out of 6259 genes, 0.2% | 1 |
| regulation of nucleotide catabolic process | 28 out of 6178 genes, 0.5% | 30 out of 6259 genes, 0.5% | 1 |
| regulation of purine nucleotide catabolic process | 28 out of 6178 genes, 0.5% | 30 out of 6259 genes, 0.5% | 1 |
| regulation of GTP catabolic process | 27 out of 6178 genes, 0.4% | 29 out of 6259 genes, 0.5% | 1 |
| negative regulation of catabolic process | 8 out of 6178 genes, 0.1% | 9 out of 6259 genes, 0.1% | 1 |
| positive regulation of cellular biosynthetic process | 8 out of 6178 genes, 0.1% | 9 out of 6259 genes, 0.1% | 1 |
| cell-substrate adhesion | 8 out of 6178 genes, 0.1% | 9 out of 6259 genes, 0.1% | 1 |
| positive regulation of signaling pathway | 26 out of 6178 genes, 0.4% | 28 out of 6259 genes, 0.4% | 1 |
| inflammatory response | 7 out of 6178 genes, 0.1% | 8 out of 6259 genes, 0.1% | 1 |
| vitamin biosynthetic process | 7 out of 6178 genes, 0.1% | 8 out of 6259 genes, 0.1% | 1 |
| nicotinamide nucleotide biosynthetic process | 7 out of 6178 genes, 0.1% | 8 out of 6259 genes, 0.1% | 1 |
| pyridine nucleotide biosynthetic process | 7 out of 6178 genes, 0.1% | 8 out of 6259 genes, 0.1% | 1 |
| positive regulation of Ras GTPase activity | 7 out of 6178 genes, 0.1% | 8 out of 6259 genes, 0.1% | 1 |
| positive regulation of Rho GTPase activity | 7 out of 6178 genes, 0.1% | 8 out of 6259 genes, 0.1% | 1 |
| water-soluble vitamin biosynthetic process | 7 out of 6178 genes, 0.1% | 8 out of 6259 genes, 0.1% | 1 |
| cellular biogenic amine biosynthetic process | 7 out of 6178 genes, 0.1% | 8 out of 6259 genes, 0.1% | 1 |
| negative regulation of DNA binding | 7 out of 6178 genes, 0.1% | 8 out of 6259 genes, 0.1% | 1 |
| positive regulation of GTPase activity | 7 out of 6178 genes, 0.1% | 8 out of 6259 genes, 0.1% | 1 |
| pigment biosynthetic process | 7 out of 6178 genes, 0.1% | 8 out of 6259 genes, 0.1% | 1 |
| regulation of cellular catabolic process | 49 out of 6178 genes, 0.8% | 52 out of 6259 genes, 0.8% | 1 |
| regulation of GTPase activity | 22 out of 6178 genes, 0.4% | 24 out of 6259 genes, 0.4% | 1 |
| polyamine metabolic process | 6 out of 6178 genes, 0.1% | 7 out of 6259 genes, 0.1% | 1 |
| signal complex assembly | 6 out of 6178 genes, 0.1% | 7 out of 6259 genes, 0.1% | 1 |
| response to vitamin A | 6 out of 6178 genes, 0.1% | 7 out of 6259 genes, 0.1% | 1 |
| cellular nitrogen compound biosynthetic process | 185 out of 6178 genes, 3.0% | 192 out of 6259 genes, 3.1% | 1 |
| regulation of Ras GTPase activity | 20 out of 6178 genes, 0.3% | 22 out of 6259 genes, 0.4% | 1 |
| regulation of catabolic process | 69 out of 6178 genes, 1.1% | 73 out of 6259 genes, 1.2% | 1 |
| coenzyme biosynthetic process | 19 out of 6178 genes, 0.3% | 21 out of 6259 genes, 0.3% | 1 |
| cofactor biosynthetic process | 40 out of 6178 genes, 0.6% | 43 out of 6259 genes, 0.7% | 1 |
| regulation of DNA binding | 17 out of 6178 genes, 0.3% | 19 out of 6259 genes, 0.3% | 1 |
| regulation of nucleotide metabolic process | 37 out of 6178 genes, 0.6% | 40 out of 6259 genes, 0.6% | 1 |
| polyamine biosynthetic process | 4 out of 6178 genes, 0.1% | 5 out of 6259 genes, 0.1% | 1 |
| stem cell division | 4 out of 6178 genes, 0.1% | 5 out of 6259 genes, 0.1% | 1 |
| positive regulation of Wnt receptor signaling pathway | 4 out of 6178 genes, 0.1% | 5 out of 6259 genes, 0.1% | 1 |
| regulation of transcription factor activity | 4 out of 6178 genes, 0.1% | 5 out of 6259 genes, 0.1% | 1 |
| regulation of transcription regulator activity | 4 out of 6178 genes, 0.1% | 5 out of 6259 genes, 0.1% | 1 |
| cell-cell adhesion | 16 out of 6178 genes, 0.3% | 18 out of 6259 genes, 0.3% | 1 |
| hexitol metabolic process | 3 out of 6178 genes, 0.0% | 4 out of 6259 genes, 0.1% | 1 |
| Rac protein signal transduction | 3 out of 6178 genes, 0.0% | 4 out of 6259 genes, 0.1% | 1 |
| negative regulation of cyclase activity | 3 out of 6178 genes, 0.0% | 4 out of 6259 genes, 0.1% | 1 |
| positive regulation of Rac GTPase activity | 3 out of 6178 genes, 0.0% | 4 out of 6259 genes, 0.1% | 1 |
| homotypic cell-cell adhesion | 3 out of 6178 genes, 0.0% | 4 out of 6259 genes, 0.1% | 1 |
| chitin-based cuticle development | 3 out of 6178 genes, 0.0% | 4 out of 6259 genes, 0.1% | 1 |
| leukocyte migration | 3 out of 6178 genes, 0.0% | 4 out of 6259 genes, 0.1% | 1 |
| regulation of Rho GTPase activity | 11 out of 6178 genes, 0.2% | 13 out of 6259 genes, 0.2% | 1 |
| regulation of Rho protein signal transduction | 11 out of 6178 genes, 0.2% | 13 out of 6259 genes, 0.2% | 1 |
| RNA methylation | 2 out of 6178 genes, 0.0% | 3 out of 6259 genes, 0.0% | 1 |
| heme biosynthetic process | 2 out of 6178 genes, 0.0% | 3 out of 6259 genes, 0.0% | 1 |
| inhibition of adenylate cyclase activity by G-protein signaling pathway | 2 out of 6178 genes, 0.0% | 3 out of 6259 genes, 0.0% | 1 |
| negative regulation of adenylate cyclase activity | 2 out of 6178 genes, 0.0% | 3 out of 6259 genes, 0.0% | 1 |
| gamma-aminobutyric acid metabolic process | 2 out of 6178 genes, 0.0% | 3 out of 6259 genes, 0.0% | 1 |
| leukocyte chemotaxis | 2 out of 6178 genes, 0.0% | 3 out of 6259 genes, 0.0% | 1 |
| embryonic hemopoiesis | 2 out of 6178 genes, 0.0% | 3 out of 6259 genes, 0.0% | 1 |
| heme metabolic process | 2 out of 6178 genes, 0.0% | 3 out of 6259 genes, 0.0% | 1 |
| regulation of angiogenesis | 2 out of 6178 genes, 0.0% | 3 out of 6259 genes, 0.0% | 1 |
| negative regulation of lyase activity | 2 out of 6178 genes, 0.0% | 3 out of 6259 genes, 0.0% | 1 |
| cell chemotaxis | 2 out of 6178 genes, 0.0% | 3 out of 6259 genes, 0.0% | 1 |

| Gene Ontology term | Genes annotated to the term |
| --- | --- |
| anatomical structure development | Unigene42196\_Sample\_011046841, Unigene60028\_Sample\_011046841, Unigene49777\_Sample\_011046841, Unigene6085\_Sample\_011046841, Unigene3491\_Sample\_011046841, Unigene12969\_Sample\_011046841, Unigene34629\_Sample\_011046841, Unigene56478\_Sample\_011046841, Unigene6410\_Sample\_011046841, Unigene60365\_Sample\_011046841, Unigene52673\_Sample\_011046841, Unigene52866\_Sample\_011046841, Unigene29723\_Sample\_011046841, Unigene9354\_Sample\_011046841, Unigene57381\_Sample\_011046841, Unigene11034\_Sample\_011046841, Unigene53075\_Sample\_011046841, Unigene60481\_Sample\_011046841, Unigene11416\_Sample\_011046841, Unigene49534\_Sample\_011046841, Unigene46625\_Sample\_011046841, Unigene60552\_Sample\_011046841, Unigene29658\_Sample\_011046841, Unigene41607\_Sample\_011046841, Unigene55286\_Sample\_011046841, Unigene8772\_Sample\_011046841, Unigene18749\_Sample\_011046841, Unigene60433\_Sample\_011046841, Unigene11802\_Sample\_011046841, Unigene4256\_Sample\_011046841, Unigene5964\_Sample\_011046841, Unigene37357\_Sample\_011046841, Unigene5058\_Sample\_011046841, Unigene3047\_Sample\_011046841, Unigene9981\_Sample\_011046841, Unigene6313\_Sample\_011046841, Unigene60211\_Sample\_011046841, Unigene54712\_Sample\_011046841, Unigene12539\_Sample\_011046841, Unigene32514\_Sample\_011046841, Unigene10955\_Sample\_011046841, Unigene59756\_Sample\_011046841, Unigene12783\_Sample\_011046841, Unigene12673\_Sample\_011046841, Unigene50963\_Sample\_011046841, Unigene55447\_Sample\_011046841, Unigene29743\_Sample\_011046841, Unigene39222\_Sample\_011046841, Unigene4456\_Sample\_011046841, Unigene26636\_Sample\_011046841, Unigene12769\_Sample\_011046841, Unigene12309\_Sample\_011046841, Unigene51419\_Sample\_011046841, Unigene50110\_Sample\_011046841, Unigene46283\_Sample\_011046841, Unigene13903\_Sample\_011046841, Unigene41574\_Sample\_011046841, Unigene27013\_Sample\_011046841, Unigene15188\_Sample\_011046841, Unigene48164\_Sample\_011046841, Unigene49240\_Sample\_011046841, Unigene58460\_Sample\_011046841, Unigene36311\_Sample\_011046841, Unigene13346\_Sample\_011046841, Unigene59077\_Sample\_011046841, Unigene47050\_Sample\_011046841, Unigene44386\_Sample\_011046841, Unigene4662\_Sample\_011046841, Unigene9967\_Sample\_011046841, Unigene51041\_Sample\_011046841, Unigene12808\_Sample\_011046841, Unigene53582\_Sample\_011046841, Unigene13224\_Sample\_011046841, Unigene42676\_Sample\_011046841, Unigene33239\_Sample\_011046841, Unigene11231\_Sample\_011046841, Unigene31853\_Sample\_011046841, Unigene58779\_Sample\_011046841, Unigene22671\_Sample\_011046841, Unigene60776\_Sample\_011046841, Unigene8956\_Sample\_011046841, Unigene60751\_Sample\_011046841, Unigene58247\_Sample\_011046841, Unigene57775\_Sample\_011046841, Unigene43826\_Sample\_011046841, Unigene34856\_Sample\_011046841, Unigene9348\_Sample\_011046841, Unigene39783\_Sample\_011046841, Unigene51837\_Sample\_011046841, Unigene31557\_Sample\_011046841, Unigene1349\_Sample\_011046841, Unigene42922\_Sample\_011046841, Unigene5203\_Sample\_011046841, Unigene11464\_Sample\_011046841, Unigene55065\_Sample\_011046841, Unigene48345\_Sample\_011046841, Unigene51962\_Sample\_011046841, Unigene52614\_Sample\_011046841, Unigene54134\_Sample\_011046841, Unigene49895\_Sample\_011046841, Unigene3481\_Sample\_011046841, Unigene13881\_Sample\_011046841, Unigene59985\_Sample\_011046841, Unigene4416\_Sample\_011046841, Unigene56663\_Sample\_011046841, Unigene46703\_Sample\_011046841, Unigene59397\_Sample\_011046841, Unigene51144\_Sample\_011046841, Unigene58409\_Sample\_011046841, Unigene39289\_Sample\_011046841, Unigene32138\_Sample\_011046841, Unigene60035\_Sample\_011046841, Unigene60139\_Sample\_011046841, Unigene44749\_Sample\_011046841, Unigene13397\_Sample\_011046841, Unigene20863\_Sample\_011046841, Unigene21150\_Sample\_011046841, Unigene53030\_Sample\_011046841, Unigene24221\_Sample\_011046841, Unigene57488\_Sample\_011046841, Unigene5795\_Sample\_011046841, Unigene59850\_Sample\_011046841, Unigene47952\_Sample\_011046841, Unigene54104\_Sample\_011046841, Unigene13733\_Sample\_011046841, Unigene4117\_Sample\_011046841, Unigene37614\_Sample\_011046841, Unigene13844\_Sample\_011046841, Unigene10174\_Sample\_011046841, Unigene44894\_Sample\_011046841, Unigene6675\_Sample\_011046841, Unigene60772\_Sample\_011046841, Unigene7340\_Sample\_011046841, Unigene8002\_Sample\_011046841, Unigene46697\_Sample\_011046841, Unigene15548\_Sample\_011046841, Unigene46256\_Sample\_011046841, Unigene3531\_Sample\_011046841, Unigene52118\_Sample\_011046841, Unigene60805\_Sample\_011046841, Unigene50695\_Sample\_011046841, Unigene60166\_Sample\_011046841, Unigene53884\_Sample\_011046841, Unigene39523\_Sample\_011046841, Unigene13321\_Sample\_011046841, Unigene43791\_Sample\_011046841, Unigene244\_Sample\_011046841, Unigene52573\_Sample\_011046841, Unigene31438\_Sample\_011046841, Unigene54437\_Sample\_011046841, Unigene12853\_Sample\_011046841, Unigene48764\_Sample\_011046841, Unigene3070\_Sample\_011046841, Unigene47783\_Sample\_011046841, Unigene39941\_Sample\_011046841, Unigene58955\_Sample\_011046841, Unigene51264\_Sample\_011046841, Unigene2521\_Sample\_011046841, Unigene36481\_Sample\_011046841, Unigene56876\_Sample\_011046841, Unigene53709\_Sample\_011046841, Unigene13930\_Sample\_011046841, Unigene11570\_Sample\_011046841, Unigene36251\_Sample\_011046841, Unigene16813\_Sample\_011046841, Unigene59671\_Sample\_011046841, Unigene9057\_Sample\_011046841, Unigene8612\_Sample\_011046841, Unigene11932\_Sample\_011046841, Unigene28322\_Sample\_011046841, Unigene60392\_Sample\_011046841, Unigene33130\_Sample\_011046841, Unigene60506\_Sample\_011046841, Unigene45342\_Sample\_011046841, Unigene28200\_Sample\_011046841, Unigene4003\_Sample\_011046841, Unigene51253\_Sample\_011046841, Unigene54695\_Sample\_011046841, Unigene22688\_Sample\_011046841, Unigene54828\_Sample\_011046841, Unigene48422\_Sample\_011046841, Unigene51559\_Sample\_011046841, Unigene43445\_Sample\_011046841, Unigene4773\_Sample\_011046841, Unigene45158\_Sample\_011046841, Unigene55363\_Sample\_011046841, Unigene59100\_Sample\_011046841, Unigene11522\_Sample\_011046841, Unigene60801\_Sample\_011046841, Unigene28658\_Sample\_011046841, Unigene31664\_Sample\_011046841, Unigene51696\_Sample\_011046841, Unigene3363\_Sample\_011046841, Unigene56665\_Sample\_011046841, Unigene13514\_Sample\_011046841, Unigene42214\_Sample\_011046841, Unigene46743\_Sample\_011046841, Unigene49671\_Sample\_011046841, Unigene33111\_Sample\_011046841, Unigene60178\_Sample\_011046841, Unigene57312\_Sample\_011046841, Unigene8971\_Sample\_011046841, Unigene7446\_Sample\_011046841, Unigene12888\_Sample\_011046841, Unigene49912\_Sample\_011046841, Unigene44085\_Sample\_011046841, Unigene8429\_Sample\_011046841, Unigene19947\_Sample\_011046841, Unigene56887\_Sample\_011046841, Unigene21284\_Sample\_011046841, Unigene59528\_Sample\_011046841, Unigene54246\_Sample\_011046841, Unigene45627\_Sample\_011046841, Unigene18126\_Sample\_011046841, Unigene51853\_Sample\_011046841, Unigene5972\_Sample\_011046841, Unigene2189\_Sample\_011046841, Unigene53593\_Sample\_011046841, Unigene40558\_Sample\_011046841, Unigene33507\_Sample\_011046841, Unigene52688\_Sample\_011046841, Unigene54525\_Sample\_011046841, Unigene49928\_Sample\_011046841, Unigene3488\_Sample\_011046841, Unigene44929\_Sample\_011046841, Unigene36865\_Sample\_011046841, Unigene54380\_Sample\_011046841, Unigene41417\_Sample\_011046841, Unigene28288\_Sample\_011046841, Unigene5904\_Sample\_011046841, Unigene42820\_Sample\_011046841, Unigene28804\_Sample\_011046841, Unigene58044\_Sample\_011046841, Unigene25891\_Sample\_011046841, Unigene49733\_Sample\_011046841, Unigene16741\_Sample\_011046841, Unigene17219\_Sample\_011046841, Unigene57783\_Sample\_011046841, Unigene57298\_Sample\_011046841, Unigene45390\_Sample\_011046841, Unigene45107\_Sample\_011046841, Unigene39200\_Sample\_011046841, Unigene48735\_Sample\_011046841, Unigene13252\_Sample\_011046841, Unigene46973\_Sample\_011046841, Unigene11787\_Sample\_011046841, Unigene13983\_Sample\_011046841, Unigene51882\_Sample\_011046841, Unigene18184\_Sample\_011046841, Unigene37028\_Sample\_011046841, Unigene58640\_Sample\_011046841, Unigene41472\_Sample\_011046841, Unigene10285\_Sample\_011046841, Unigene6561\_Sample\_011046841, Unigene49615\_Sample\_011046841, Unigene50966\_Sample\_011046841, Unigene53602\_Sample\_011046841, Unigene161\_Sample\_011046841, Unigene47257\_Sample\_011046841, Unigene46603\_Sample\_011046841, Unigene3632\_Sample\_011046841, Unigene31821\_Sample\_011046841, Unigene28636\_Sample\_011046841, Unigene3946\_Sample\_011046841, Unigene37278\_Sample\_011046841, Unigene26357\_Sample\_011046841, Unigene4977\_Sample\_011046841, Unigene4112\_Sample\_011046841, Unigene53609\_Sample\_011046841, Unigene11053\_Sample\_011046841, Unigene57765\_Sample\_011046841, Unigene12837\_Sample\_011046841, Unigene46688\_Sample\_011046841, Unigene59200\_Sample\_011046841, Unigene12324\_Sample\_011046841, Unigene55205\_Sample\_011046841, Unigene1594\_Sample\_011046841, Unigene15880\_Sample\_011046841, Unigene44366\_Sample\_011046841, Unigene2731\_Sample\_011046841, Unigene48094\_Sample\_011046841, Unigene21527\_Sample\_011046841, Unigene30764\_Sample\_011046841, Unigene58947\_Sample\_011046841, Unigene9674\_Sample\_011046841, Unigene44817\_Sample\_011046841, Unigene29952\_Sample\_011046841, Unigene6467\_Sample\_011046841, Unigene58855\_Sample\_011046841, Unigene52591\_Sample\_011046841, Unigene52883\_Sample\_011046841, Unigene55957\_Sample\_011046841, Unigene44354\_Sample\_011046841, Unigene21539\_Sample\_011046841, Unigene58359\_Sample\_011046841, Unigene50758\_Sample\_011046841, Unigene6469\_Sample\_011046841, Unigene26728\_Sample\_011046841, Unigene47555\_Sample\_011046841, Unigene11659\_Sample\_011046841, Unigene9535\_Sample\_011046841, Unigene44544\_Sample\_011046841, Unigene39062\_Sample\_011046841, Unigene55866\_Sample\_011046841, Unigene58440\_Sample\_011046841, Unigene60843\_Sample\_011046841, Unigene47860\_Sample\_011046841, Unigene44507\_Sample\_011046841, Unigene1975\_Sample\_011046841, Unigene20596\_Sample\_011046841, Unigene58668\_Sample\_011046841, Unigene8489\_Sample\_011046841, Unigene13916\_Sample\_011046841, Unigene9082\_Sample\_011046841, Unigene5335\_Sample\_011046841, Unigene53139\_Sample\_011046841, Unigene421\_Sample\_011046841, Unigene2891\_Sample\_011046841, Unigene59045\_Sample\_011046841, Unigene24100\_Sample\_011046841, Unigene59068\_Sample\_011046841, Unigene55651\_Sample\_011046841, Unigene47923\_Sample\_011046841, Unigene31969\_Sample\_011046841, Unigene46764\_Sample\_011046841, Unigene8488\_Sample\_011046841, Unigene11741\_Sample\_011046841, Unigene44863\_Sample\_011046841, Unigene57161\_Sample\_011046841, Unigene12211\_Sample\_011046841, Unigene55049\_Sample\_011046841, Unigene41720\_Sample\_011046841, Unigene13801\_Sample\_011046841, Unigene52739\_Sample\_011046841, Unigene51706\_Sample\_011046841, Unigene52590\_Sample\_011046841, Unigene31181\_Sample\_011046841, Unigene59463\_Sample\_011046841, Unigene17383\_Sample\_011046841, Unigene49887\_Sample\_011046841, Unigene58768\_Sample\_011046841, Unigene57623\_Sample\_011046841, Unigene58513\_Sample\_011046841, Unigene30697\_Sample\_011046841, Unigene47780\_Sample\_011046841, Unigene1598\_Sample\_011046841, Unigene10683\_Sample\_011046841, Unigene246\_Sample\_011046841, Unigene13936\_Sample\_011046841, Unigene55290\_Sample\_011046841, Unigene26327\_Sample\_011046841, Unigene9537\_Sample\_011046841, Unigene11201\_Sample\_011046841, Unigene5747\_Sample\_011046841, Unigene58199\_Sample\_011046841, Unigene60138\_Sample\_011046841, Unigene2582\_Sample\_011046841, Unigene55178\_Sample\_011046841, Unigene42127\_Sample\_011046841, Unigene54315\_Sample\_011046841, Unigene60485\_Sample\_011046841, Unigene43504\_Sample\_011046841, Unigene58128\_Sample\_011046841, Unigene39675\_Sample\_011046841, Unigene54638\_Sample\_011046841, Unigene36772\_Sample\_011046841, Unigene25547\_Sample\_011046841, Unigene32174\_Sample\_011046841, Unigene49859\_Sample\_011046841, Unigene52610\_Sample\_011046841, Unigene60576\_Sample\_011046841, Unigene10982\_Sample\_011046841, Unigene30454\_Sample\_011046841, Unigene49125\_Sample\_011046841, Unigene32470\_Sample\_011046841, Unigene47864\_Sample\_011046841, Unigene52007\_Sample\_011046841, Unigene9879\_Sample\_011046841, Unigene2950\_Sample\_011046841, Unigene45179\_Sample\_011046841, Unigene20440\_Sample\_011046841, Unigene47887\_Sample\_011046841, Unigene35141\_Sample\_011046841, Unigene8037\_Sample\_011046841, Unigene57677\_Sample\_011046841, Unigene16539\_Sample\_011046841, Unigene53979\_Sample\_011046841, Unigene8526\_Sample\_011046841, Unigene34164\_Sample\_011046841, Unigene38547\_Sample\_011046841, Unigene5012\_Sample\_011046841, Unigene19018\_Sample\_011046841, Unigene30713\_Sample\_011046841, Unigene679\_Sample\_011046841, Unigene1439\_Sample\_011046841, Unigene54368\_Sample\_011046841, Unigene60018\_Sample\_011046841, Unigene58363\_Sample\_011046841, Unigene56818\_Sample\_011046841, Unigene53425\_Sample\_011046841, Unigene41465\_Sample\_011046841, Unigene47099\_Sample\_011046841, Unigene11547\_Sample\_011046841, Unigene28640\_Sample\_011046841, Unigene51523\_Sample\_011046841, Unigene31394\_Sample\_011046841, Unigene57252\_Sample\_011046841, Unigene48005\_Sample\_011046841, Unigene58173\_Sample\_011046841, Unigene9128\_Sample\_011046841, Unigene43698\_Sample\_011046841, Unigene58915\_Sample\_011046841, Unigene47428\_Sample\_011046841, Unigene12940\_Sample\_011046841, Unigene58936\_Sample\_011046841, Unigene41863\_Sample\_011046841, Unigene56051\_Sample\_011046841, Unigene60902\_Sample\_011046841, Unigene29816\_Sample\_011046841, Unigene51084\_Sample\_011046841, Unigene314\_Sample\_011046841, Unigene4242\_Sample\_011046841, Unigene20175\_Sample\_011046841, Unigene22189\_Sample\_011046841, Unigene45986\_Sample\_011046841, Unigene56711\_Sample\_011046841, Unigene51426\_Sample\_011046841, Unigene49205\_Sample\_011046841, Unigene17752\_Sample\_011046841, Unigene9776\_Sample\_011046841, Unigene13927\_Sample\_011046841, Unigene48197\_Sample\_011046841, Unigene4599\_Sample\_011046841, Unigene27454\_Sample\_011046841, Unigene30734\_Sample\_011046841, Unigene60607\_Sample\_011046841, Unigene25050\_Sample\_011046841, Unigene40989\_Sample\_011046841, Unigene54549\_Sample\_011046841, Unigene52751\_Sample\_011046841, Unigene9271\_Sample\_011046841, Unigene21162\_Sample\_011046841, Unigene58776\_Sample\_011046841, Unigene24597\_Sample\_011046841, Unigene50014\_Sample\_011046841, Unigene10744\_Sample\_011046841, Unigene8047\_Sample\_011046841, Unigene29396\_Sample\_011046841, Unigene55511\_Sample\_011046841, Unigene53326\_Sample\_011046841, Unigene59151\_Sample\_011046841, Unigene58960\_Sample\_011046841, Unigene18596\_Sample\_011046841, Unigene13920\_Sample\_011046841, Unigene28148\_Sample\_011046841, Unigene47795\_Sample\_011046841, Unigene37565\_Sample\_011046841, Unigene40255\_Sample\_011046841, Unigene34330\_Sample\_011046841, Unigene53264\_Sample\_011046841, Unigene30530\_Sample\_011046841, Unigene59758\_Sample\_011046841, Unigene59021\_Sample\_011046841, Unigene56736\_Sample\_011046841, Unigene11563\_Sample\_011046841, Unigene8034\_Sample\_011046841, Unigene22684\_Sample\_011046841, Unigene51174\_Sample\_011046841, Unigene57225\_Sample\_011046841, Unigene42885\_Sample\_011046841, Unigene55520\_Sample\_011046841, Unigene56264\_Sample\_011046841, Unigene58285\_Sample\_011046841, Unigene48922\_Sample\_011046841, Unigene34824\_Sample\_011046841, Unigene57843\_Sample\_011046841, Unigene22955\_Sample\_011046841, Unigene51626\_Sample\_011046841, Unigene58043\_Sample\_011046841, Unigene56081\_Sample\_011046841, Unigene36705\_Sample\_011046841, Unigene60822\_Sample\_011046841, Unigene7206\_Sample\_011046841, Unigene1526\_Sample\_011046841, Unigene6497\_Sample\_011046841, Unigene39553\_Sample\_011046841, Unigene45039\_Sample\_011046841, Unigene47219\_Sample\_011046841, Unigene21230\_Sample\_011046841, Unigene9238\_Sample\_011046841, Unigene27108\_Sample\_011046841, Unigene57974\_Sample\_011046841, Unigene13329\_Sample\_011046841, Unigene30320\_Sample\_011046841, Unigene35902\_Sample\_011046841, Unigene8312\_Sample\_011046841, Unigene46515\_Sample\_011046841, Unigene58821\_Sample\_011046841, Unigene2805\_Sample\_011046841, Unigene58653\_Sample\_011046841, Unigene55378\_Sample\_011046841, Unigene50987\_Sample\_011046841, Unigene12591\_Sample\_011046841, Unigene12170\_Sample\_011046841, Unigene51125\_Sample\_011046841, Unigene9017\_Sample\_011046841, Unigene52532\_Sample\_011046841, Unigene57517\_Sample\_011046841, Unigene11884\_Sample\_011046841, Unigene3588\_Sample\_011046841, Unigene59747\_Sample\_011046841, Unigene13669\_Sample\_011046841, Unigene50207\_Sample\_011046841, Unigene2673\_Sample\_011046841, Unigene993\_Sample\_011046841, Unigene23921\_Sample\_011046841, Unigene59687\_Sample\_011046841, Unigene7149\_Sample\_011046841, Unigene23704\_Sample\_011046841, Unigene55900\_Sample\_011046841, Unigene12700\_Sample\_011046841, Unigene30736\_Sample\_011046841, Unigene47959\_Sample\_011046841, Unigene19526\_Sample\_011046841, Unigene57032\_Sample\_011046841, Unigene57665\_Sample\_011046841, Unigene13302\_Sample\_011046841, Unigene4364\_Sample\_011046841, Unigene50296\_Sample\_011046841, Unigene10803\_Sample\_011046841, Unigene2105\_Sample\_011046841, Unigene53653\_Sample\_011046841, Unigene51196\_Sample\_011046841, Unigene33766\_Sample\_011046841, Unigene29813\_Sample\_011046841, Unigene29970\_Sample\_011046841, Unigene11249\_Sample\_011046841, Unigene58649\_Sample\_011046841, Unigene11256\_Sample\_011046841, Unigene35586\_Sample\_011046841, Unigene44769\_Sample\_011046841, Unigene6900\_Sample\_011046841, Unigene19407\_Sample\_011046841, Unigene59695\_Sample\_011046841, Unigene11134\_Sample\_011046841, Unigene53456\_Sample\_011046841, Unigene32509\_Sample\_011046841, Unigene48293\_Sample\_011046841, Unigene50854\_Sample\_011046841, Unigene8641\_Sample\_011046841, Unigene11339\_Sample\_011046841, Unigene56248\_Sample\_011046841, Unigene4218\_Sample\_011046841, Unigene31534\_Sample\_011046841, Unigene18856\_Sample\_011046841, Unigene48047\_Sample\_011046841, Unigene6435\_Sample\_011046841, Unigene48963\_Sample\_011046841, Unigene49914\_Sample\_011046841, Unigene41236\_Sample\_011046841, Unigene44683\_Sample\_011046841, Unigene56828\_Sample\_011046841, Unigene51294\_Sample\_011046841, Unigene58149\_Sample\_011046841, Unigene23599\_Sample\_011046841, Unigene42218\_Sample\_011046841, Unigene55342\_Sample\_011046841, Unigene10325\_Sample\_011046841, Unigene41559\_Sample\_011046841, Unigene56559\_Sample\_011046841, Unigene53284\_Sample\_011046841, Unigene57337\_Sample\_011046841, Unigene28643\_Sample\_011046841, Unigene43091\_Sample\_011046841, Unigene9480\_Sample\_011046841, Unigene27016\_Sample\_011046841, Unigene10813\_Sample\_011046841, Unigene59097\_Sample\_011046841, Unigene37440\_Sample\_011046841, Unigene8148\_Sample\_011046841, Unigene38773\_Sample\_011046841, Unigene60824\_Sample\_011046841, Unigene59493\_Sample\_011046841, Unigene60241\_Sample\_011046841, Unigene60015\_Sample\_011046841, Unigene42259\_Sample\_011046841, Unigene1543\_Sample\_011046841, Unigene4096\_Sample\_011046841, Unigene45120\_Sample\_011046841, Unigene28573\_Sample\_011046841, Unigene46219\_Sample\_011046841, Unigene12830\_Sample\_011046841, Unigene47947\_Sample\_011046841, Unigene54169\_Sample\_011046841, Unigene60536\_Sample\_011046841, Unigene1076\_Sample\_011046841, Unigene45303\_Sample\_011046841, Unigene21177\_Sample\_011046841, Unigene4219\_Sample\_011046841, Unigene38688\_Sample\_011046841, Unigene52525\_Sample\_011046841, Unigene19273\_Sample\_011046841, Unigene13879\_Sample\_011046841, Unigene53186\_Sample\_011046841, Unigene13231\_Sample\_011046841, Unigene20899\_Sample\_011046841, Unigene59022\_Sample\_011046841, Unigene36373\_Sample\_011046841, Unigene41460\_Sample\_011046841, Unigene58924\_Sample\_011046841, Unigene10701\_Sample\_011046841, Unigene31416\_Sample\_011046841, Unigene42860\_Sample\_011046841, Unigene983\_Sample\_011046841, Unigene59398\_Sample\_011046841, Unigene30474\_Sample\_011046841, Unigene49837\_Sample\_011046841, Unigene47936\_Sample\_011046841, Unigene43721\_Sample\_011046841, Unigene60483\_Sample\_011046841, Unigene38854\_Sample\_011046841, Unigene49448\_Sample\_011046841, Unigene21282\_Sample\_011046841, Unigene25597\_Sample\_011046841, Unigene45635\_Sample\_011046841, Unigene60020\_Sample\_011046841, Unigene47046\_Sample\_011046841, Unigene14069\_Sample\_011046841, Unigene11562\_Sample\_011046841, Unigene55709\_Sample\_011046841, Unigene57118\_Sample\_011046841, Unigene11714\_Sample\_011046841, Unigene11114\_Sample\_011046841, Unigene10525\_Sample\_011046841, Unigene59993\_Sample\_011046841, Unigene44587\_Sample\_011046841, Unigene23762\_Sample\_011046841, Unigene6097\_Sample\_011046841, Unigene53585\_Sample\_011046841, Unigene51519\_Sample\_011046841, Unigene59828\_Sample\_011046841, Unigene47082\_Sample\_011046841, Unigene48532\_Sample\_011046841, Unigene58243\_Sample\_011046841, Unigene45282\_Sample\_011046841, Unigene53496\_Sample\_011046841, Unigene23925\_Sample\_011046841, Unigene12145\_Sample\_011046841, Unigene54996\_Sample\_011046841, Unigene42026\_Sample\_011046841, Unigene60561\_Sample\_011046841, Unigene58386\_Sample\_011046841, Unigene37232\_Sample\_011046841, Unigene11794\_Sample\_011046841, Unigene56596\_Sample\_011046841, Unigene13796\_Sample\_011046841, Unigene41323\_Sample\_011046841, Unigene59198\_Sample\_011046841, Unigene25326\_Sample\_011046841, Unigene12152\_Sample\_011046841, Unigene44362\_Sample\_011046841, Unigene60238\_Sample\_011046841, Unigene60097\_Sample\_011046841, Unigene57955\_Sample\_011046841, Unigene51627\_Sample\_011046841, Unigene57238\_Sample\_011046841, Unigene46777\_Sample\_011046841, Unigene54897\_Sample\_011046841, Unigene8925\_Sample\_011046841, Unigene1417\_Sample\_011046841, Unigene59095\_Sample\_011046841, Unigene39488\_Sample\_011046841, Unigene12869\_Sample\_011046841, Unigene58169\_Sample\_011046841, Unigene56228\_Sample\_011046841, Unigene53431\_Sample\_011046841, Unigene45620\_Sample\_011046841, Unigene6178\_Sample\_011046841, Unigene55921\_Sample\_011046841, Unigene29309\_Sample\_011046841, Unigene47487\_Sample\_011046841, Unigene33509\_Sample\_011046841, Unigene50223\_Sample\_011046841, Unigene26723\_Sample\_011046841, Unigene15865\_Sample\_011046841, Unigene10725\_Sample\_011046841, Unigene54555\_Sample\_011046841, Unigene32930\_Sample\_011046841, Unigene43713\_Sample\_011046841, Unigene18725\_Sample\_011046841, Unigene59885\_Sample\_011046841, Unigene55754\_Sample\_011046841, Unigene41992\_Sample\_011046841, Unigene60284\_Sample\_011046841, Unigene44079\_Sample\_011046841, Unigene5178\_Sample\_011046841, Unigene23671\_Sample\_011046841, Unigene56891\_Sample\_011046841, Unigene58403\_Sample\_011046841, Unigene11922\_Sample\_011046841, Unigene60442\_Sample\_011046841, Unigene56177\_Sample\_011046841, Unigene52106\_Sample\_011046841, Unigene42036\_Sample\_011046841, Unigene56198\_Sample\_011046841, Unigene35715\_Sample\_011046841, Unigene59457\_Sample\_011046841, Unigene11867\_Sample\_011046841, Unigene43838\_Sample\_011046841, Unigene23154\_Sample\_011046841, Unigene13639\_Sample\_011046841, Unigene19418\_Sample\_011046841, Unigene60659\_Sample\_011046841, Unigene50775\_Sample\_011046841, Unigene20487\_Sample\_011046841, Unigene32629\_Sample\_011046841, Unigene49371\_Sample\_011046841, Unigene53915\_Sample\_011046841, Unigene25489\_Sample\_011046841, Unigene3496\_Sample\_011046841, Unigene50495\_Sample\_011046841, Unigene18301\_Sample\_011046841, Unigene52832\_Sample\_011046841, Unigene48882\_Sample\_011046841, Unigene36469\_Sample\_011046841, Unigene49764\_Sample\_011046841, Unigene60705\_Sample\_011046841, Unigene9329\_Sample\_011046841, Unigene20344\_Sample\_011046841, Unigene13880\_Sample\_011046841, Unigene58166\_Sample\_011046841, Unigene59779\_Sample\_011046841, Unigene10640\_Sample\_011046841, Unigene21655\_Sample\_011046841, Unigene60741\_Sample\_011046841, Unigene5630\_Sample\_011046841, Unigene4785\_Sample\_011046841, Unigene22457\_Sample\_011046841, Unigene2492\_Sample\_011046841, Unigene13607\_Sample\_011046841, Unigene56127\_Sample\_011046841, Unigene44328\_Sample\_011046841, Unigene60381\_Sample\_011046841, Unigene10963\_Sample\_011046841, Unigene15\_Sample\_011046841, Unigene7887\_Sample\_011046841, Unigene52294\_Sample\_011046841, Unigene47549\_Sample\_011046841, Unigene60821\_Sample\_011046841, Unigene59003\_Sample\_011046841, Unigene39094\_Sample\_011046841, Unigene58929\_Sample\_011046841, Unigene12620\_Sample\_011046841, Unigene2493\_Sample\_011046841, Unigene45556\_Sample\_011046841, Unigene37886\_Sample\_011046841, Unigene52427\_Sample\_011046841, Unigene28625\_Sample\_011046841, Unigene50127\_Sample\_011046841, Unigene54293\_Sample\_011046841, Unigene33127\_Sample\_011046841, Unigene8959\_Sample\_011046841, Unigene49970\_Sample\_011046841, Unigene42129\_Sample\_011046841, Unigene51225\_Sample\_011046841, Unigene2441\_Sample\_011046841, Unigene19596\_Sample\_011046841, Unigene8243\_Sample\_011046841, Unigene11383\_Sample\_011046841, Unigene5431\_Sample\_011046841, Unigene60831\_Sample\_011046841, Unigene55393\_Sample\_011046841, Unigene13613\_Sample\_011046841, Unigene38974\_Sample\_011046841, Unigene54014\_Sample\_011046841, Unigene13636\_Sample\_011046841, Unigene9219\_Sample\_011046841, Unigene53893\_Sample\_011046841, Unigene2256\_Sample\_011046841, Unigene25573\_Sample\_011046841, Unigene60546\_Sample\_011046841, Unigene56049\_Sample\_011046841, Unigene49730\_Sample\_011046841, Unigene56881\_Sample\_011046841, Unigene15838\_Sample\_011046841, Unigene52275\_Sample\_011046841, Unigene2095\_Sample\_011046841, Unigene56920\_Sample\_011046841, Unigene48091\_Sample\_011046841, Unigene55314\_Sample\_011046841, Unigene7809\_Sample\_011046841, Unigene49698\_Sample\_011046841, Unigene53115\_Sample\_011046841, Unigene59006\_Sample\_011046841, Unigene60102\_Sample\_011046841, Unigene13511\_Sample\_011046841, Unigene16257\_Sample\_011046841, Unigene53037\_Sample\_011046841, Unigene11367\_Sample\_011046841, Unigene60693\_Sample\_011046841, Unigene20608\_Sample\_011046841, Unigene59812\_Sample\_011046841, Unigene59913\_Sample\_011046841, Unigene29847\_Sample\_011046841, Unigene31866\_Sample\_011046841, Unigene49228\_Sample\_011046841, Unigene57706\_Sample\_011046841, Unigene48339\_Sample\_011046841, Unigene48996\_Sample\_011046841, Unigene3237\_Sample\_011046841, Unigene44734\_Sample\_011046841, Unigene29551\_Sample\_011046841, Unigene4916\_Sample\_011046841, Unigene12300\_Sample\_011046841, Unigene53334\_Sample\_011046841, Unigene2532\_Sample\_011046841, Unigene55101\_Sample\_011046841, Unigene45873\_Sample\_011046841, Unigene10215\_Sample\_011046841, Unigene12560\_Sample\_011046841, Unigene40544\_Sample\_011046841, Unigene31849\_Sample\_011046841, Unigene52303\_Sample\_011046841, Unigene11939\_Sample\_011046841, Unigene20157\_Sample\_011046841, Unigene41694\_Sample\_011046841, Unigene53420\_Sample\_011046841, Unigene58067\_Sample\_011046841, Unigene37456\_Sample\_011046841, Unigene56560\_Sample\_011046841, Unigene59819\_Sample\_011046841, Unigene54663\_Sample\_011046841, Unigene58882\_Sample\_011046841, Unigene36816\_Sample\_011046841, Unigene40568\_Sample\_011046841, Unigene58321\_Sample\_011046841, Unigene11846\_Sample\_011046841, Unigene54842\_Sample\_011046841, Unigene35043\_Sample\_011046841, Unigene58326\_Sample\_011046841, Unigene58953\_Sample\_011046841, Unigene33436\_Sample\_011046841, Unigene52984\_Sample\_011046841, Unigene28012\_Sample\_011046841, Unigene56734\_Sample\_011046841, Unigene46852\_Sample\_011046841, Unigene47569\_Sample\_011046841, Unigene58564\_Sample\_011046841, Unigene37642\_Sample\_011046841, Unigene46263\_Sample\_011046841, Unigene59460\_Sample\_011046841, Unigene7821\_Sample\_011046841, Unigene29587\_Sample\_011046841, Unigene19215\_Sample\_011046841, Unigene38930\_Sample\_011046841, Unigene52253\_Sample\_011046841, Unigene7850\_Sample\_011046841, Unigene60288\_Sample\_011046841, Unigene39020\_Sample\_011046841, Unigene54900\_Sample\_011046841, Unigene60217\_Sample\_011046841, Unigene60548\_Sample\_011046841, Unigene60378\_Sample\_011046841, Unigene60828\_Sample\_011046841, Unigene42561\_Sample\_011046841, Unigene11254\_Sample\_011046841, Unigene57972\_Sample\_011046841, Unigene6605\_Sample\_011046841, Unigene49211\_Sample\_011046841, Unigene60665\_Sample\_011046841, Unigene50729\_Sample\_011046841, Unigene29462\_Sample\_011046841, Unigene1934\_Sample\_011046841, Unigene9142\_Sample\_011046841, Unigene1260\_Sample\_011046841, Unigene51777\_Sample\_011046841, Unigene13711\_Sample\_011046841, Unigene20298\_Sample\_011046841, Unigene44315\_Sample\_011046841, Unigene24814\_Sample\_011046841, Unigene43409\_Sample\_011046841, Unigene30971\_Sample\_011046841, Unigene11431\_Sample\_011046841, Unigene34547\_Sample\_011046841, Unigene44504\_Sample\_011046841, Unigene14320\_Sample\_011046841, Unigene49608\_Sample\_011046841, Unigene56464\_Sample\_011046841, Unigene51098\_Sample\_011046841, Unigene45926\_Sample\_011046841, Unigene48613\_Sample\_011046841, Unigene34653\_Sample\_011046841, Unigene52931\_Sample\_011046841, Unigene32258\_Sample\_011046841, Unigene33792\_Sample\_011046841, Unigene43020\_Sample\_011046841, Unigene58107\_Sample\_011046841, Unigene58135\_Sample\_011046841, Unigene49581\_Sample\_011046841, Unigene60044\_Sample\_011046841, Unigene47024\_Sample\_011046841, Unigene40435\_Sample\_011046841, Unigene1378\_Sample\_011046841, Unigene60695\_Sample\_011046841, Unigene7523\_Sample\_011046841, Unigene50563\_Sample\_011046841, Unigene54373\_Sample\_011046841, Unigene51070\_Sample\_011046841, Unigene56466\_Sample\_011046841, Unigene35911\_Sample\_011046841, Unigene6432\_Sample\_011046841, Unigene47924\_Sample\_011046841, Unigene55975\_Sample\_011046841, Unigene42293\_Sample\_011046841, Unigene54628\_Sample\_011046841, Unigene11626\_Sample\_011046841, Unigene49162\_Sample\_011046841, Unigene52467\_Sample\_011046841, Unigene56709\_Sample\_011046841, Unigene51937\_Sample\_011046841, Unigene18124\_Sample\_011046841, Unigene13945\_Sample\_011046841, Unigene52543\_Sample\_011046841, Unigene54859\_Sample\_011046841, Unigene57120\_Sample\_011046841, Unigene47292\_Sample\_011046841, Unigene59573\_Sample\_011046841, Unigene54448\_Sample\_011046841, Unigene60522\_Sample\_011046841, Unigene58611\_Sample\_011046841, Unigene57861\_Sample\_011046841, Unigene9154\_Sample\_011046841, Unigene52776\_Sample\_011046841, Unigene26878\_Sample\_011046841, Unigene60819\_Sample\_011046841, Unigene29206\_Sample\_011046841, Unigene22433\_Sample\_011046841, Unigene58447\_Sample\_011046841, Unigene22728\_Sample\_011046841, Unigene11351\_Sample\_011046841, Unigene1885\_Sample\_011046841, Unigene27756\_Sample\_011046841, Unigene48598\_Sample\_011046841, Unigene7431\_Sample\_011046841, Unigene8654\_Sample\_011046841, Unigene9579\_Sample\_011046841, Unigene13722\_Sample\_011046841, Unigene23133\_Sample\_011046841, Unigene50443\_Sample\_011046841, Unigene59664\_Sample\_011046841, Unigene60642\_Sample\_011046841, Unigene315\_Sample\_011046841, Unigene23416\_Sample\_011046841, Unigene31515\_Sample\_011046841, Unigene31120\_Sample\_011046841, Unigene59727\_Sample\_011046841, Unigene58804\_Sample\_011046841, Unigene42686\_Sample\_011046841, Unigene51977\_Sample\_011046841, Unigene9432\_Sample\_011046841, Unigene13447\_Sample\_011046841, Unigene12384\_Sample\_011046841, Unigene19793\_Sample\_011046841, Unigene8894\_Sample\_011046841, Unigene60629\_Sample\_011046841, Unigene58974\_Sample\_011046841, Unigene29346\_Sample\_011046841, Unigene6327\_Sample\_011046841, Unigene59844\_Sample\_011046841, Unigene58894\_Sample\_011046841, Unigene37372\_Sample\_011046841, Unigene50825\_Sample\_011046841, Unigene3810\_Sample\_011046841, Unigene14538\_Sample\_011046841, Unigene41467\_Sample\_011046841, Unigene60883\_Sample\_011046841, Unigene49921\_Sample\_011046841, Unigene15204\_Sample\_011046841, Unigene52238\_Sample\_011046841, Unigene47982\_Sample\_011046841, Unigene48689\_Sample\_011046841, Unigene60197\_Sample\_011046841, Unigene58494\_Sample\_011046841, Unigene59001\_Sample\_011046841, Unigene53073\_Sample\_011046841, Unigene7095\_Sample\_011046841, Unigene59228\_Sample\_011046841, Unigene31607\_Sample\_011046841, Unigene60643\_Sample\_011046841, Unigene17568\_Sample\_011046841, Unigene56584\_Sample\_011046841, Unigene58021\_Sample\_011046841, Unigene44216\_Sample\_011046841, Unigene57130\_Sample\_011046841, Unigene46176\_Sample\_011046841, Unigene31706\_Sample\_011046841, Unigene33607\_Sample\_011046841, Unigene32964\_Sample\_011046841, Unigene29503\_Sample\_011046841, Unigene10438\_Sample\_011046841, Unigene23519\_Sample\_011046841, Unigene6752\_Sample\_011046841, Unigene44497\_Sample\_011046841, Unigene15792\_Sample\_011046841, Unigene56904\_Sample\_011046841, Unigene45147\_Sample\_011046841, Unigene1793\_Sample\_011046841, Unigene55163\_Sample\_011046841, Unigene60454\_Sample\_011046841, Unigene11526\_Sample\_011046841, Unigene41001\_Sample\_011046841, Unigene55122\_Sample\_011046841, Unigene50039\_Sample\_011046841, Unigene16198\_Sample\_011046841, Unigene59036\_Sample\_011046841, Unigene54574\_Sample\_011046841, Unigene55111\_Sample\_011046841, Unigene60780\_Sample\_011046841, Unigene58024\_Sample\_011046841, Unigene7898\_Sample\_011046841, Unigene54003\_Sample\_011046841, Unigene53888\_Sample\_011046841, Unigene36363\_Sample\_011046841, Unigene56312\_Sample\_011046841, Unigene59413\_Sample\_011046841, Unigene1010\_Sample\_011046841, Unigene48625\_Sample\_011046841, Unigene48252\_Sample\_011046841, Unigene19676\_Sample\_011046841, Unigene31514\_Sample\_011046841, Unigene30153\_Sample\_011046841, Unigene51585\_Sample\_011046841, Unigene19091\_Sample\_011046841, Unigene9726\_Sample\_011046841, Unigene30309\_Sample\_011046841, Unigene53019\_Sample\_011046841, Unigene57942\_Sample\_011046841, Unigene31216\_Sample\_011046841, Unigene60555\_Sample\_011046841, Unigene47187\_Sample\_011046841, Unigene53343\_Sample\_011046841, Unigene30035\_Sample\_011046841, Unigene9769\_Sample\_011046841, Unigene10135\_Sample\_011046841, Unigene58020\_Sample\_011046841, Unigene55624\_Sample\_011046841, Unigene46974\_Sample\_011046841, Unigene51171\_Sample\_011046841, Unigene51233\_Sample\_011046841, Unigene10024\_Sample\_011046841, Unigene20592\_Sample\_011046841, Unigene57293\_Sample\_011046841, Unigene48044\_Sample\_011046841, Unigene12652\_Sample\_011046841, Unigene8335\_Sample\_011046841, Unigene54531\_Sample\_011046841, Unigene60935\_Sample\_011046841, Unigene19355\_Sample\_011046841, Unigene60543\_Sample\_011046841, Unigene50485\_Sample\_011046841, Unigene17531\_Sample\_011046841, Unigene17371\_Sample\_011046841, Unigene41012\_Sample\_011046841, Unigene55948\_Sample\_011046841, Unigene53711\_Sample\_011046841, Unigene33512\_Sample\_011046841, Unigene59177\_Sample\_011046841, Unigene32121\_Sample\_011046841, Unigene12189\_Sample\_011046841, Unigene4889\_Sample\_011046841, Unigene26214\_Sample\_011046841, Unigene41682\_Sample\_011046841, Unigene43411\_Sample\_011046841, Unigene2221\_Sample\_011046841, Unigene34802\_Sample\_011046841, Unigene60925\_Sample\_011046841, Unigene52884\_Sample\_011046841, Unigene13579\_Sample\_011046841, Unigene58011\_Sample\_011046841, Unigene49667\_Sample\_011046841, Unigene52110\_Sample\_011046841, Unigene43515\_Sample\_011046841, Unigene44243\_Sample\_011046841, Unigene1886\_Sample\_011046841, Unigene41088\_Sample\_011046841, Unigene60431\_Sample\_011046841, Unigene10948\_Sample\_011046841, Unigene24797\_Sample\_011046841, Unigene9600\_Sample\_011046841, Unigene57712\_Sample\_011046841, Unigene38676\_Sample\_011046841, Unigene45172\_Sample\_011046841, Unigene45985\_Sample\_011046841, Unigene9408\_Sample\_011046841, Unigene9130\_Sample\_011046841, Unigene58177\_Sample\_011046841, Unigene47429\_Sample\_011046841, Unigene60734\_Sample\_011046841, Unigene42643\_Sample\_011046841, Unigene53097\_Sample\_011046841, Unigene12487\_Sample\_011046841, Unigene57887\_Sample\_011046841, Unigene47581\_Sample\_011046841, Unigene44025\_Sample\_011046841, Unigene32534\_Sample\_011046841, Unigene3893\_Sample\_011046841, Unigene12448\_Sample\_011046841, Unigene60014\_Sample\_011046841, Unigene5954\_Sample\_011046841, Unigene38769\_Sample\_011046841, Unigene21318\_Sample\_011046841, Unigene7647\_Sample\_011046841, Unigene28880\_Sample\_011046841, Unigene17505\_Sample\_011046841, Unigene48747\_Sample\_011046841, Unigene3659\_Sample\_011046841, Unigene6317\_Sample\_011046841, Unigene58083\_Sample\_011046841, Unigene54198\_Sample\_011046841, Unigene46280\_Sample\_011046841, Unigene45885\_Sample\_011046841, Unigene47635\_Sample\_011046841, Unigene9259\_Sample\_011046841, Unigene39627\_Sample\_011046841, Unigene50927\_Sample\_011046841, Unigene36626\_Sample\_011046841, Unigene9774\_Sample\_011046841, Unigene57300\_Sample\_011046841, Unigene57194\_Sample\_011046841, Unigene45721\_Sample\_011046841, Unigene55486\_Sample\_011046841, Unigene58444\_Sample\_011046841, Unigene43935\_Sample\_011046841, Unigene43986\_Sample\_011046841, Unigene12446\_Sample\_011046841, Unigene51663\_Sample\_011046841, Unigene51736\_Sample\_011046841, Unigene60218\_Sample\_011046841, Unigene48064\_Sample\_011046841, Unigene52181\_Sample\_011046841, Unigene48863\_Sample\_011046841, Unigene39189\_Sample\_011046841, Unigene47667\_Sample\_011046841, Unigene12195\_Sample\_011046841, Unigene7766\_Sample\_011046841, Unigene5730\_Sample\_011046841, Unigene13164\_Sample\_011046841, Unigene57305\_Sample\_011046841, Unigene12559\_Sample\_011046841, Unigene50919\_Sample\_011046841, Unigene41178\_Sample\_011046841, Unigene43897\_Sample\_011046841, Unigene13251\_Sample\_011046841, Unigene24065\_Sample\_011046841, Unigene51429\_Sample\_011046841, Unigene59292\_Sample\_011046841, Unigene8299\_Sample\_011046841, Unigene50071\_Sample\_011046841, Unigene7112\_Sample\_011046841, Unigene44445\_Sample\_011046841, Unigene36986\_Sample\_011046841, Unigene25630\_Sample\_011046841, Unigene19089\_Sample\_011046841, Unigene60424\_Sample\_011046841 |
[truncated: 4,304,632 more chars]
