# Supplementary material for: Identification of neglected cestode Taenia multiceps microRNAs by illumina sequencing and bioinformatic analysis
Source: BMC Vet Res. 2013 Aug 13;9:162. doi: 10.1186/1746-6148-9-162 (PMC3849562; doi:10.1186/1746-6148-9-162)
Supplement: Additional file 10 — KEGG pathways for predicted target genes of Taenia multiceps novel miRNAs. 9,306 target unigenes were assigned into 240 KEGG pathways. . “Pvalue” and “Qvalue” represent P-value before correction and corrected P-value, respectively. [file 1746-6148-9-162-S10.htm]

Taenia\_multiceps

Pathway annotation of Sample Taenia\_multiceps

| # | Pathway | Sample1 (9306) | Sample2 (9400) | Pvalue | Qvalue | Pathway ID |
| 1 | Chemokine signaling pathway | 193 | 193 | 0.1408722 | 0.9995065 | ko04062 |
| 2 | Progesterone-mediated oocyte maturation | 159 | 159 | 0.1995570 | 0.9995065 | ko04914 |
| 3 | Purine metabolism | 293 | 294 | 0.2020267 | 0.9995065 | ko00230 |
| 4 | Meiosis - yeast | 156 | 156 | 0.2057713 | 0.9995065 | ko04113 |
| 5 | ErbB signaling pathway | 141 | 141 | 0.2398334 | 0.9995065 | ko04012 |
| 6 | T cell receptor signaling pathway | 119 | 119 | 0.3001122 | 0.9995065 | ko04660 |
| 7 | Prostate cancer | 114 | 114 | 0.3157779 | 0.9995065 | ko05215 |
| 8 | Cell cycle | 231 | 232 | 0.3210129 | 0.9995065 | ko04110 |
| 9 | Epithelial cell signaling in Helicobacter pylori infection | 112 | 112 | 0.3222683 | 0.9995065 | ko05120 |
| 10 | Long-term depression | 111 | 111 | 0.3255629 | 0.9995065 | ko04730 |
| 11 | Nucleotide excision repair | 110 | 110 | 0.3288907 | 0.9995065 | ko03420 |
| 12 | Huntington's disease | 340 | 342 | 0.3295134 | 0.9995065 | ko05016 |
| 13 | Renal cell carcinoma | 105 | 105 | 0.3460415 | 0.9995065 | ko05211 |
| 14 | Pathways in cancer | 319 | 321 | 0.3721985 | 0.9995065 | ko05200 |
| 15 | Cell cycle - yeast | 202 | 203 | 0.3935835 | 0.9995065 | ko04111 |
| 16 | Adherens junction | 200 | 201 | 0.3990138 | 0.9995065 | ko04520 |
| 17 | Chronic myeloid leukemia | 89 | 89 | 0.4070926 | 0.9995065 | ko05220 |
| 18 | Chagas disease | 86 | 86 | 0.4196725 | 0.9995065 | ko05142 |
| 19 | Notch signaling pathway | 85 | 85 | 0.4239507 | 0.9995065 | ko04330 |
| 20 | DNA replication | 85 | 85 | 0.4239507 | 0.9995065 | ko03030 |
| 21 | VEGF signaling pathway | 81 | 81 | 0.4414993 | 0.9995065 | ko04370 |
| 22 | Dorso-ventral axis formation | 79 | 79 | 0.4505411 | 0.9995065 | ko04320 |
| 23 | mTOR signaling pathway | 79 | 79 | 0.4505411 | 0.9995065 | ko04150 |
| 24 | Pyrimidine metabolism | 181 | 182 | 0.4533492 | 0.9995065 | ko00240 |
| 25 | Tight junction | 283 | 285 | 0.453561 | 0.9995065 | ko04530 |
| 26 | Adipocytokine signaling pathway | 78 | 78 | 0.4551305 | 0.9995065 | ko04920 |
| 27 | Gap junction | 179 | 180 | 0.4593554 | 0.9995065 | ko04540 |
| 28 | Amino sugar and nucleotide sugar metabolism | 75 | 75 | 0.469178 | 0.9995065 | ko00520 |
| 29 | Vascular smooth muscle contraction | 175 | 176 | 0.4715288 | 0.9995065 | ko04270 |
| 30 | Amyotrophic lateral sclerosis (ALS) | 74 | 74 | 0.4739552 | 0.9995065 | ko05014 |
| 31 | Non-small cell lung cancer | 70 | 70 | 0.4935498 | 0.9995065 | ko05223 |
| 32 | Fc epsilon RI signaling pathway | 70 | 70 | 0.4935498 | 0.9995065 | ko04664 |
| 33 | Lysosome | 167 | 168 | 0.4965098 | 0.9995065 | ko04142 |
| 34 | Jak-STAT signaling pathway | 68 | 68 | 0.5036455 | 0.9995065 | ko04630 |
| 35 | B cell receptor signaling pathway | 66 | 66 | 0.5139456 | 0.9995065 | ko04662 |
| 36 | Pancreatic cancer | 66 | 66 | 0.5139456 | 0.9995065 | ko05212 |
| 37 | Neurotrophin signaling pathway | 159 | 160 | 0.5223119 | 0.9995065 | ko04722 |
| 38 | Type II diabetes mellitus | 64 | 64 | 0.5244539 | 0.9995065 | ko04930 |
| 39 | Axon guidance | 152 | 153 | 0.545532 | 0.9995065 | ko04360 |
| 40 | Acute myeloid leukemia | 60 | 60 | 0.5461125 | 0.9995065 | ko05221 |
| 41 | Fc gamma R-mediated phagocytosis | 151 | 152 | 0.5488962 | 0.9995065 | ko04666 |
| 42 | Biosynthesis of secondary metabolites | 340 | 343 | 0.5496378 | 0.9995065 | ko01110 |
| 43 | p53 signaling pathway | 58 | 58 | 0.5572713 | 0.9995065 | ko04115 |
| 44 | RNA polymerase | 56 | 56 | 0.5686556 | 0.9995065 | ko03020 |
| 45 | Dilated cardiomyopathy | 145 | 146 | 0.5693178 | 0.9995065 | ko05414 |
| 46 | Endocytosis | 424 | 428 | 0.5728294 | 0.9995065 | ko04144 |
| 47 | Oocyte meiosis | 235 | 237 | 0.5758701 | 0.9995065 | ko04114 |
| 48 | Long-term potentiation | 142 | 143 | 0.579675 | 0.9995065 | ko04720 |
| 49 | Wnt signaling pathway | 229 | 231 | 0.5919893 | 0.9995065 | ko04310 |
| 50 | Melanoma | 52 | 52 | 0.592119 | 0.9995065 | ko05218 |
| 51 | Toll-like receptor signaling pathway | 52 | 52 | 0.592119 | 0.9995065 | ko04620 |
| 52 | Mismatch repair | 52 | 52 | 0.592119 | 0.9995065 | ko03430 |
| 53 | Pentose phosphate pathway | 49 | 49 | 0.6103428 | 0.9995065 | ko00030 |
| 54 | Hypertrophic cardiomyopathy (HCM) | 132 | 133 | 0.614845 | 0.9995065 | ko05410 |
| 55 | NOD-like receptor signaling pathway | 48 | 48 | 0.6165398 | 0.9995065 | ko04621 |
| 56 | Lysine degradation | 47 | 47 | 0.6227991 | 0.9995065 | ko00310 |
| 57 | MAPK signaling pathway - yeast | 47 | 47 | 0.6227991 | 0.9995065 | ko04011 |
| 58 | MAPK signaling pathway - fly | 47 | 47 | 0.6227991 | 0.9995065 | ko04013 |
| 59 | Peroxisome | 46 | 46 | 0.6291213 | 0.9995065 | ko04146 |
| 60 | Carbon fixation in photosynthetic organisms | 46 | 46 | 0.6291213 | 0.9995065 | ko00710 |
| 61 | Glycosylphosphatidylinositol(GPI)-anchor biosynthesis | 45 | 45 | 0.6355069 | 0.9995065 | ko00563 |
| 62 | Regulation of actin cytoskeleton | 301 | 304 | 0.6381354 | 0.9995065 | ko04810 |
| 63 | Ether lipid metabolism | 44 | 44 | 0.6419567 | 0.9995065 | ko00565 |
| 64 | Vibrio cholerae infection | 122 | 123 | 0.6508772 | 0.9995065 | ko05110 |
| 65 | Melanogenesis | 121 | 122 | 0.6545201 | 0.9995065 | ko04916 |
| 66 | Metabolic pathways | 1312 | 1326 | 0.6551183 | 0.9995065 | ko01100 |
| 67 | Taste transduction | 41 | 41 | 0.661697 | 0.9995065 | ko04742 |
| 68 | GnRH signaling pathway | 119 | 120 | 0.6618248 | 0.9995065 | ko04912 |
| 69 | Spliceosome | 467 | 472 | 0.6665701 | 0.9995065 | ko03040 |
| 70 | Antigen processing and presentation | 200 | 202 | 0.6712188 | 0.9995065 | ko04612 |
| 71 | TGF-beta signaling pathway | 116 | 117 | 0.672825 | 0.9995065 | ko04350 |
| 72 | Bladder cancer | 39 | 39 | 0.6751897 | 0.9995065 | ko05219 |
| 73 | Complement and coagulation cascades | 39 | 39 | 0.6751897 | 0.9995065 | ko04610 |
| 74 | Pyruvate metabolism | 115 | 116 | 0.6765021 | 0.9995065 | ko00620 |
| 75 | Sphingolipid metabolism | 38 | 38 | 0.6820378 | 0.9995065 | ko00600 |
| 76 | Glycerophospholipid metabolism | 113 | 114 | 0.6838706 | 0.9995065 | ko00564 |
| 77 | Aminoacyl-tRNA biosynthesis | 112 | 113 | 0.6875613 | 0.9995065 | ko00970 |
| 78 | N-Glycan biosynthesis | 111 | 112 | 0.691256 | 0.9995065 | ko00510 |
| 79 | Neuroactive ligand-receptor interaction | 110 | 111 | 0.6949542 | 0.9995065 | ko04080 |
| 80 | Collecting duct acid secretion | 36 | 36 | 0.6959407 | 0.9995065 | ko04966 |
| 81 | Regulation of autophagy | 36 | 36 | 0.6959407 | 0.9995065 | ko04140 |
| 82 | Thyroid cancer | 36 | 36 | 0.6959407 | 0.9995065 | ko05216 |
| 83 | Glycolysis / Gluconeogenesis | 107 | 108 | 0.7060679 | 0.9995065 | ko00010 |
| 84 | Ubiquitin mediated proteolysis | 271 | 274 | 0.7064588 | 0.9995065 | ko04120 |
| 85 | Drug metabolism - other enzymes | 34 | 34 | 0.710124 | 0.9995065 | ko00983 |
| 86 | RIG-I-like receptor signaling pathway | 34 | 34 | 0.710124 | 0.9995065 | ko04622 |
| 87 | Cytokine-cytokine receptor interaction | 34 | 34 | 0.710124 | 0.9995065 | ko04060 |
| 88 | Tryptophan metabolism | 32 | 32 | 0.7245932 | 0.9995065 | ko00380 |
| 89 | O-Glycan biosynthesis | 32 | 32 | 0.7245932 | 0.9995065 | ko00512 |
| 90 | Natural killer cell mediated cytotoxicity | 102 | 103 | 0.7246343 | 0.9995065 | ko04650 |
| 91 | Valine, leucine and isoleucine degradation | 31 | 31 | 0.7319367 | 0.9995065 | ko00280 |
| 92 | Fatty acid metabolism | 31 | 31 | 0.7319367 | 0.9995065 | ko00071 |
| 93 | Arrhythmogenic right ventricular cardiomyopathy (ARVC) | 98 | 99 | 0.739502 | 0.9995065 | ko05412 |
| 94 | Leukocyte transendothelial migration | 175 | 177 | 0.7395753 | 0.9995065 | ko04670 |
| 95 | Tyrosine metabolism | 29 | 29 | 0.7468455 | 0.9995065 | ko00350 |
| 96 | Valine, leucine and isoleucine biosynthesis | 29 | 29 | 0.7468455 | 0.9995065 | ko00290 |
| 97 | Pathogenic Escherichia coli infection | 171 | 173 | 0.7503518 | 0.9995065 | ko05130 |
| 98 | Inositol phosphate metabolism | 94 | 95 | 0.7543569 | 0.9995065 | ko00562 |
| 99 | Histidine metabolism | 27 | 27 | 0.7620547 | 0.9995065 | ko00340 |
| 100 | Glycerolipid metabolism | 89 | 90 | 0.7728682 | 0.9995065 | ko00561 |
| 101 | Glioma | 89 | 90 | 0.7728682 | 0.9995065 | ko05214 |
| 102 | Arachidonic acid metabolism | 25 | 25 | 0.7775702 | 0.9995065 | ko00590 |
| 103 | Non-homologous end-joining | 25 | 25 | 0.7775702 | 0.9995065 | ko03450 |
| 104 | Terpenoid backbone biosynthesis | 24 | 24 | 0.7854448 | 0.9995065 | ko00900 |
| 105 | Fructose and mannose metabolism | 85 | 86 | 0.7875971 | 0.9995065 | ko00051 |
| 106 | ECM-receptor interaction | 85 | 86 | 0.7875971 | 0.9995065 | ko04512 |
| 107 | Calcium signaling pathway | 231 | 234 | 0.7936176 | 0.9995065 | ko04020 |
| 108 | Fatty acid biosynthesis | 22 | 22 | 0.8014313 | 0.9995065 | ko00061 |
| 109 | Glycosphingolipid biosynthesis - lacto and neolacto series | 22 | 22 | 0.8014313 | 0.9995065 | ko00601 |
| 110 | Colorectal cancer | 79 | 80 | 0.8094853 | 0.9995065 | ko05210 |
| 111 | Hematopoietic cell lineage | 21 | 21 | 0.8095449 | 0.9995065 | ko04640 |
| 112 | Other glycan degradation | 21 | 21 | 0.8095449 | 0.9995065 | ko00511 |
| 113 | Cytosolic DNA-sensing pathway | 20 | 20 | 0.8177397 | 0.9995065 | ko04623 |
| 114 | Pantothenate and CoA biosynthesis | 20 | 20 | 0.8177397 | 0.9995065 | ko00770 |
| 115 | Nitrogen metabolism | 20 | 20 | 0.8177397 | 0.9995065 | ko00910 |
| 116 | Riboflavin metabolism | 19 | 19 | 0.8260166 | 0.9995065 | ko00740 |
| 117 | Parkinson's disease | 139 | 141 | 0.8329123 | 0.9995065 | ko05012 |
| 118 | Glyoxylate and dicarboxylate metabolism | 18 | 18 | 0.8343764 | 0.9995065 | ko00630 |
| 119 | ABC transporters | 71 | 72 | 0.8381072 | 0.9995065 | ko02010 |
| 120 | Small cell lung cancer | 71 | 72 | 0.8381072 | 0.9995065 | ko05222 |
| 121 | Basal transcription factors | 70 | 71 | 0.8416267 | 0.9995065 | ko03022 |
| 122 | Cardiac muscle contraction | 70 | 71 | 0.8416267 | 0.9995065 | ko04260 |
| 123 | Glycine, serine and threonine metabolism | 17 | 17 | 0.8428199 | 0.9995065 | ko00260 |
| 124 | MAPK signaling pathway | 433 | 439 | 0.8507177 | 0.9995065 | ko04010 |
| 125 | Reductive carboxylate cycle (CO2 fixation) | 16 | 16 | 0.8513479 | 0.9995065 | ko00720 |
| 126 | Metabolism of xenobiotics by cytochrome P450 | 15 | 15 | 0.8599612 | 0.9995065 | ko00980 |
| 127 | Naphthalene and anthracene degradation | 15 | 15 | 0.8599612 | 0.9995065 | ko00626 |
| 128 | Drug metabolism - cytochrome P450 | 15 | 15 | 0.8599612 | 0.9995065 | ko00982 |
| 129 | Linoleic acid metabolism | 14 | 14 | 0.8686608 | 0.9995065 | ko00591 |
| 130 | Primary immunodeficiency | 14 | 14 | 0.8686608 | 0.9995065 | ko05340 |
| 131 | Maturity onset diabetes of the young | 14 | 14 | 0.8686608 | 0.9995065 | ko04950 |
| 132 | Prion diseases | 62 | 63 | 0.8691863 | 0.9995065 | ko05020 |
| 133 | Phosphatidylinositol signaling system | 123 | 125 | 0.8704622 | 0.9995065 | ko04070 |
| 134 | Glycosaminoglycan degradation | 13 | 13 | 0.8774474 | 0.9995065 | ko00531 |
| 135 | Phosphonate and phosphinate metabolism | 13 | 13 | 0.8774474 | 0.9995065 | ko00440 |
| 136 | Hedgehog signaling pathway | 59 | 60 | 0.8792016 | 0.9995065 | ko04340 |
| 137 | Aldosterone-regulated sodium reabsorption | 59 | 60 | 0.8792016 | 0.9995065 | ko04960 |
| 138 | Homologous recombination | 59 | 60 | 0.8792016 | 0.9995065 | ko03440 |
| 139 | PPAR signaling pathway | 58 | 59 | 0.8824954 | 0.9995065 | ko03320 |
| 140 | Apoptosis | 57 | 58 | 0.8857655 | 0.9995065 | ko04210 |
| 141 | Glycosphingolipid biosynthesis - globo series | 12 | 12 | 0.886322 | 0.9995065 | ko00603 |
| 142 | Thiamine metabolism | 12 | 12 | 0.886322 | 0.9995065 | ko00730 |
| 143 | Glutathione metabolism | 55 | 56 | 0.8922318 | 0.9995065 | ko00480 |
| 144 | Cell adhesion molecules (CAMs) | 55 | 56 | 0.8922318 | 0.9995065 | ko04514 |
| 145 | Focal adhesion | 394 | 400 | 0.894947 | 0.9995065 | ko04510 |
| 146 | Glycosphingolipid biosynthesis - ganglio series | 11 | 11 | 0.8952853 | 0.9995065 | ko00604 |
| 147 | Limonene and pinene degradation | 11 | 11 | 0.8952853 | 0.9995065 | ko00903 |
| 148 | Streptomycin biosynthesis | 11 | 11 | 0.8952853 | 0.9995065 | ko00521 |
| 149 | Steroid hormone biosynthesis | 11 | 11 | 0.8952853 | 0.9995065 | ko00140 |
| 150 | Ascorbate and aldarate metabolism | 11 | 11 | 0.8952853 | 0.9995065 | ko00053 |
| 151 | Phenylalanine metabolism | 11 | 11 | 0.8952853 | 0.9995065 | ko00360 |
| 152 | Renin-angiotensin system | 11 | 11 | 0.8952853 | 0.9995065 | ko04614 |
| 153 | Citrate cycle (TCA cycle) | 53 | 54 | 0.8985939 | 0.9995065 | ko00020 |
| 154 | Ubiquinone and other terpenoid-quinone biosynthesis | 10 | 10 | 0.9043383 | 0.9995065 | ko00130 |
| 155 | One carbon pool by folate | 10 | 10 | 0.9043383 | 0.9995065 | ko00670 |
| 156 | Basal cell carcinoma | 49 | 50 | 0.9109779 | 0.9995065 | ko05217 |
| 157 | High-mannose type N-glycan biosynthesis | 9 | 9 | 0.9134819 | 0.9995065 | ko00513 |
| 158 | Biosynthesis of unsaturated fatty acids | 9 | 9 | 0.9134819 | 0.9995065 | ko01040 |
| 159 | Circadian rhythm - fly | 9 | 9 | 0.9134819 | 0.9995065 | ko04711 |
| 160 | Bisphenol A degradation | 9 | 9 | 0.9134819 | 0.9995065 | ko00363 |
| 161 | Fatty acid elongation in mitochondria | 9 | 9 | 0.9134819 | 0.9995065 | ko00062 |
| 162 | Pentose and glucuronate interconversions | 48 | 49 | 0.9139979 | 0.9995065 | ko00040 |
| 163 | Phototransduction | 47 | 48 | 0.9169856 | 0.9995065 | ko04744 |
| 164 | Olfactory transduction | 47 | 48 | 0.9169856 | 0.9995065 | ko04740 |
| 165 | Lipoic acid metabolism | 8 | 8 | 0.922717 | 0.9995065 | ko00785 |
| 166 | Isoquinoline alkaloid biosynthesis | 8 | 8 | 0.922717 | 0.9995065 | ko00950 |
| 167 | Tetrachloroethene degradation | 8 | 8 | 0.922717 | 0.9995065 | ko00625 |
| 168 | Type I diabetes mellitus | 8 | 8 | 0.922717 | 0.9995065 | ko04940 |
| 169 | Cyanoamino acid metabolism | 8 | 8 | 0.922717 | 0.9995065 | ko00460 |
| 170 | Bacterial invasion of epithelial cells | 152 | 155 | 0.9304813 | 0.9995065 | ko05100 |
| 171 | Protein export | 42 | 43 | 0.9314073 | 0.9995065 | ko03060 |
| 172 | Methane metabolism | 7 | 7 | 0.9320443 | 0.9995065 | ko00680 |
| 173 | Retinol metabolism | 7 | 7 | 0.9320443 | 0.9995065 | ko00830 |
| 174 | Bacterial secretion system | 7 | 7 | 0.9320443 | 0.9995065 | ko03070 |
| 175 | alpha-Linolenic acid metabolism | 7 | 7 | 0.9320443 | 0.9995065 | ko00592 |
| 176 | Benzoate degradation via CoA ligation | 7 | 7 | 0.9320443 | 0.9995065 | ko00632 |
| 177 | Viral myocarditis | 148 | 151 | 0.9355794 | 0.9995065 | ko05416 |
| 178 | Propanoate metabolism | 40 | 41 | 0.9369162 | 0.9995065 | ko00640 |
| 179 | Proteasome | 88 | 90 | 0.9389314 | 0.9995065 | ko03050 |
| 180 | Taurine and hypotaurine metabolism | 6 | 6 | 0.941465 | 0.9995065 | ko00430 |
| 181 | Primary bile acid biosynthesis | 6 | 6 | 0.941465 | 0.9995065 | ko00120 |
| 182 | 1,2-Dichloroethane degradation | 6 | 6 | 0.941465 | 0.9995065 | ko00631 |
| 183 | 3-Chloroacrylic acid degradation | 6 | 6 | 0.941465 | 0.9995065 | ko00641 |
| 184 | Endometrial cancer | 85 | 87 | 0.94373 | 0.9995065 | ko05213 |
| 185 | Glycosaminoglycan biosynthesis - heparan sulfate | 36 | 37 | 0.9474426 | 0.9995065 | ko00534 |
| 186 | Alanine, aspartate and glutamate metabolism | 36 | 37 | 0.9474426 | 0.9995065 | ko00250 |
| 187 | Insulin signaling pathway | 264 | 269 | 0.9476467 | 0.9995065 | ko04910 |
| 188 | SNARE interactions in vesicular transport | 35 | 36 | 0.9499655 | 0.9995065 | ko04130 |
| 189 | Glycosaminoglycan biosynthesis - keratan sulfate | 5 | 5 | 0.9509798 | 0.9995065 | ko00533 |
| 190 | 1- and 2-Methylnaphthalene degradation | 5 | 5 | 0.9509798 | 0.9995065 | ko00624 |
| 191 | Geraniol degradation | 5 | 5 | 0.9509798 | 0.9995065 | ko00281 |
| 192 | Vitamin B6 metabolism | 5 | 5 | 0.9509798 | 0.9995065 | ko00750 |
| 193 | Butirosin and neomycin biosynthesis | 5 | 5 | 0.9509798 | 0.9995065 | ko00524 |
| 194 | Galactose metabolism | 79 | 81 | 0.9527548 | 0.9995065 | ko00052 |
| 195 | Selenoamino acid metabolism | 32 | 33 | 0.957255 | 0.9995065 | ko00450 |
| 196 | Nicotinate and nicotinamide metabolism | 32 | 33 | 0.957255 | 0.9995065 | ko00760 |
| 197 | Proximal tubule bicarbonate reclamation | 32 | 33 | 0.957255 | 0.9995065 | ko04964 |
| 198 | Alzheimer's disease | 186 | 190 | 0.9584998 | 0.9995065 | ko05010 |
| 199 | Plant-pathogen interaction | 31 | 32 | 0.959588 | 0.9995065 | ko04626 |
| 200 | Butanoate metabolism | 31 | 32 | 0.959588 | 0.9995065 | ko00650 |
| 201 | Steroid biosynthesis | 4 | 4 | 0.9605898 | 0.9995065 | ko00100 |
| 202 | Autoimmune thyroid disease | 4 | 4 | 0.9605898 | 0.9995065 | ko05320 |
| 203 | Tropane, piperidine and pyridine alkaloid biosynthesis | 4 | 4 | 0.9605898 | 0.9995065 | ko00960 |
| 204 | Betalain biosynthesis | 4 | 4 | 0.9605898 | 0.9995065 | ko00965 |
| 205 | Synthesis and degradation of ketone bodies | 4 | 4 | 0.9605898 | 0.9995065 | ko00072 |
| 206 | Oxidative phosphorylation | 121 | 124 | 0.964534 | 0.9995065 | ko00190 |
| 207 | Base excision repair | 68 | 70 | 0.9672409 | 0.9995065 | ko03410 |
| 208 | Porphyrin and chlorophyll metabolism | 27 | 28 | 0.9684022 | 0.9995065 | ko00860 |
| 209 | Zeatin biosynthesis | 3 | 3 | 0.9702959 | 0.9995065 | ko00908 |
| 210 | D-Glutamine and D-glutamate metabolism | 3 | 3 | 0.9702959 | 0.9995065 | ko00471 |
| 211 | Sulfur metabolism | 3 | 3 | 0.9702959 | 0.9995065 | ko00920 |
| 212 | Circadian rhythm - plant | 3 | 3 | 0.9702959 | 0.9995065 | ko04712 |
| 213 | Phenylalanine, tyrosine and tryptophan biosynthesis | 3 | 3 | 0.9702959 | 0.9995065 | ko00400 |
| 214 | Indole alkaloid biosynthesis | 3 | 3 | 0.9702959 | 0.9995065 | ko00901 |
| 215 | Novobiocin biosynthesis | 3 | 3 | 0.9702959 | 0.9995065 | ko00401 |
| 216 | Cysteine and methionine metabolism | 26 | 27 | 0.97047 | 0.9995065 | ko00270 |
| 217 | Leishmaniasis | 22 | 23 | 0.9781598 | 0.9995065 | ko05140 |
| 218 | beta-Alanine metabolism | 22 | 23 | 0.9781598 | 0.9995065 | ko00410 |
| 219 | Systemic lupus erythematosus | 57 | 59 | 0.9789655 | 0.9995065 | ko05322 |
| 220 | Cell cycle - Caulobacter | 2 | 2 | 0.980099 | 0.9995065 | ko04112 |
| 221 | Ethylbenzene degradation | 2 | 2 | 0.980099 | 0.9995065 | ko00642 |
| 222 | Asthma | 2 | 2 | 0.980099 | 0.9995065 | ko05310 |
| 223 | Intestinal immune network for IgA production | 2 | 2 | 0.980099 | 0.9995065 | ko04672 |
| 224 | Vasopressin-regulated water reabsorption | 140 | 144 | 0.9854635 | 0.9995065 | ko04962 |
| 225 | RNA degradation | 139 | 143 | 0.9858592 | 0.9995065 | ko03018 |
| 226 | gamma-Hexachlorocyclohexane degradation | 16 | 17 | 0.9877958 | 0.9995065 | ko00361 |
| 227 | Glycosaminoglycan biosynthesis - chondroitin sulfate | 15 | 16 | 0.9891616 | 0.9995065 | ko00532 |
| 228 | Fluorobenzoate degradation | 1 | 1 | 0.99 | 0.9995065 | ko00364 |
| 229 | 1,4-Dichlorobenzene degradation | 1 | 1 | 0.99 | 0.9995065 | ko00627 |
| 230 | Lysine biosynthesis | 1 | 1 | 0.99 | 0.9995065 | ko00300 |
| 231 | Atrazine degradation | 1 | 1 | 0.99 | 0.9995065 | ko00791 |
| 232 | Biosynthesis of ansamycins | 1 | 1 | 0.99 | 0.9995065 | ko01051 |
| 233 | Biotin metabolism | 1 | 1 | 0.99 | 0.9995065 | ko00780 |
| 234 | Folate biosynthesis | 13 | 14 | 0.9916735 | 0.9995065 | ko00790 |
| 235 | Caprolactam degradation | 11 | 12 | 0.993882 | 0.9995065 | ko00930 |
| 236 | Arginine and proline metabolism | 35 | 37 | 0.994116 | 0.9995065 | ko00330 |
| 237 | Ribosome | 61 | 64 | 0.9962308 | 0.9995065 | ko03010 |
| 238 | Circadian rhythm - mammal | 5 | 6 | 0.9985541 | 0.9995065 | ko04710 |
| 239 | Starch and sucrose metabolism | 63 | 67 | 0.999468 | 0.9995065 | ko00500 |
| 240 | Two-component system | 14 | 16 | 0.9995065 | 0.9995065 | ko02020 |

| # | Pathway | Target genes involved in the pathway |
| --- | --- | --- |
| 1 | Chemokine signaling pathway Back to summary table | Unigene679\_Sample\_011046841, Unigene865\_Sample\_011046841, Unigene1273\_Sample\_011046841, Unigene2138\_Sample\_011046841, Unigene4209\_Sample\_011046841, Unigene7143\_Sample\_011046841, Unigene7281\_Sample\_011046841, Unigene8486\_Sample\_011046841, Unigene8859\_Sample\_011046841, Unigene9858\_Sample\_011046841, Unigene9929\_Sample\_011046841, Unigene10459\_Sample\_011046841, Unigene10970\_Sample\_011046841, Unigene11055\_Sample\_011046841, Unigene13069\_Sample\_011046841, Unigene13481\_Sample\_011046841, Unigene13789\_Sample\_011046841, Unigene13901\_Sample\_011046841, Unigene39219\_Sample\_011046841, Unigene40272\_Sample\_011046841, Unigene43518\_Sample\_011046841, Unigene43936\_Sample\_011046841, Unigene52094\_Sample\_011046841, Unigene53424\_Sample\_011046841, Unigene54160\_Sample\_011046841, Unigene54982\_Sample\_011046841, Unigene55610\_Sample\_011046841, Unigene56327\_Sample\_011046841, Unigene57298\_Sample\_011046841, Unigene57887\_Sample\_011046841, Unigene58460\_Sample\_011046841, Unigene58987\_Sample\_011046841, Unigene59022\_Sample\_011046841, Unigene59189\_Sample\_011046841, Unigene59559\_Sample\_011046841, Unigene60173\_Sample\_011046841, Unigene60267\_Sample\_011046841, Unigene60498\_Sample\_011046841, Unigene60789\_Sample\_011046841, Unigene4361\_Sample\_011046841, Unigene4993\_Sample\_011046841, Unigene5623\_Sample\_011046841, Unigene5744\_Sample\_011046841, Unigene6233\_Sample\_011046841, Unigene6977\_Sample\_011046841, Unigene8569\_Sample\_011046841, Unigene9354\_Sample\_011046841, Unigene9408\_Sample\_011046841, Unigene10198\_Sample\_011046841, Unigene11464\_Sample\_011046841, Unigene13329\_Sample\_011046841, Unigene13332\_Sample\_011046841, Unigene13855\_Sample\_011046841, Unigene13884\_Sample\_011046841, Unigene13938\_Sample\_011046841, Unigene19792\_Sample\_011046841, Unigene21418\_Sample\_011046841, Unigene21901\_Sample\_011046841, Unigene21962\_Sample\_011046841, Unigene33821\_Sample\_011046841, Unigene39360\_Sample\_011046841, Unigene40209\_Sample\_011046841, Unigene41574\_Sample\_011046841, Unigene42561\_Sample\_011046841, Unigene42820\_Sample\_011046841, Unigene43409\_Sample\_011046841, Unigene44906\_Sample\_011046841, Unigene45592\_Sample\_011046841, Unigene46219\_Sample\_011046841, Unigene46410\_Sample\_011046841, Unigene47555\_Sample\_011046841, Unigene48772\_Sample\_011046841, Unigene50163\_Sample\_011046841, Unigene50677\_Sample\_011046841, Unigene52146\_Sample\_011046841, Unigene53096\_Sample\_011046841, Unigene53264\_Sample\_011046841, Unigene53334\_Sample\_011046841, Unigene54900\_Sample\_011046841, Unigene56596\_Sample\_011046841, Unigene56681\_Sample\_011046841, Unigene57037\_Sample\_011046841, Unigene57566\_Sample\_011046841, Unigene58100\_Sample\_011046841, Unigene58209\_Sample\_011046841, Unigene58551\_Sample\_011046841, Unigene59071\_Sample\_011046841, Unigene59742\_Sample\_011046841, Unigene59902\_Sample\_011046841, Unigene59905\_Sample\_011046841, Unigene60190\_Sample\_011046841, Unigene60271\_Sample\_011046841, Unigene60880\_Sample\_011046841, Unigene3070\_Sample\_011046841, Unigene5972\_Sample\_011046841, Unigene13822\_Sample\_011046841, Unigene19453\_Sample\_011046841, Unigene23590\_Sample\_011046841, Unigene28658\_Sample\_011046841, Unigene29578\_Sample\_011046841, Unigene32259\_Sample\_011046841, Unigene36373\_Sample\_011046841, Unigene40491\_Sample\_011046841, Unigene40758\_Sample\_011046841, Unigene46930\_Sample\_011046841, Unigene48530\_Sample\_011046841, Unigene49837\_Sample\_011046841, Unigene54574\_Sample\_011046841, Unigene56118\_Sample\_011046841, Unigene56456\_Sample\_011046841, Unigene60391\_Sample\_011046841, Unigene60506\_Sample\_011046841, Unigene60765\_Sample\_011046841, Unigene4034\_Sample\_011046841, Unigene6435\_Sample\_011046841, Unigene8139\_Sample\_011046841, Unigene19951\_Sample\_011046841, Unigene30184\_Sample\_011046841, Unigene32964\_Sample\_011046841, Unigene44532\_Sample\_011046841, Unigene46684\_Sample\_011046841, Unigene47549\_Sample\_011046841, Unigene49878\_Sample\_011046841, Unigene53642\_Sample\_011046841, Unigene56300\_Sample\_011046841, Unigene59711\_Sample\_011046841, Unigene60042\_Sample\_011046841, Unigene29346\_Sample\_011046841, Unigene30190\_Sample\_011046841, Unigene45708\_Sample\_011046841, Unigene49525\_Sample\_011046841, Unigene55486\_Sample\_011046841, Unigene3797\_Sample\_011046841, Unigene38674\_Sample\_011046841, Unigene43782\_Sample\_011046841, Unigene47967\_Sample\_011046841, Unigene51429\_Sample\_011046841, Unigene51301\_Sample\_011046841, Unigene7997\_Sample\_011046841, Unigene11333\_Sample\_011046841, Unigene11549\_Sample\_011046841, Unigene29318\_Sample\_011046841, Unigene47054\_Sample\_011046841, Unigene48742\_Sample\_011046841, Unigene51756\_Sample\_011046841, Unigene56390\_Sample\_011046841, Unigene59221\_Sample\_011046841, Unigene46589\_Sample\_011046841, Unigene2110\_Sample\_011046841, Unigene21201\_Sample\_011046841, Unigene41793\_Sample\_011046841, Unigene53352\_Sample\_011046841, Unigene53995\_Sample\_011046841, Unigene55041\_Sample\_011046841, Unigene56969\_Sample\_011046841, Unigene57797\_Sample\_011046841, Unigene15705\_Sample\_011046841, Unigene23831\_Sample\_011046841, Unigene28288\_Sample\_011046841, Unigene36841\_Sample\_011046841, Unigene37707\_Sample\_011046841, Unigene42580\_Sample\_011046841, Unigene47686\_Sample\_011046841, Unigene47910\_Sample\_011046841, Unigene50832\_Sample\_011046841, Unigene54171\_Sample\_011046841, Unigene50989\_Sample\_011046841, Unigene51777\_Sample\_011046841, Unigene57344\_Sample\_011046841, Unigene12324\_Sample\_011046841, Unigene34984\_Sample\_011046841, Unigene20770\_Sample\_011046841, Unigene44021\_Sample\_011046841, Unigene50713\_Sample\_011046841, Unigene41464\_Sample\_011046841, Unigene43367\_Sample\_011046841, Unigene49482\_Sample\_011046841, Unigene19937\_Sample\_011046841, Unigene32258\_Sample\_011046841, Unigene35082\_Sample\_011046841, Unigene4308\_Sample\_011046841, Unigene9811\_Sample\_011046841, Unigene14248\_Sample\_011046841, Unigene46813\_Sample\_011046841, Unigene50293\_Sample\_011046841, Unigene14181\_Sample\_011046841, Unigene16813\_Sample\_011046841, Unigene26184\_Sample\_011046841, Unigene30474\_Sample\_011046841, Unigene7048\_Sample\_011046841, Unigene34414\_Sample\_011046841, Unigene56075\_Sample\_011046841, Unigene19596\_Sample\_011046841 |
| 2 | Progesterone-mediated oocyte maturation Back to summary table | Unigene1437\_Sample\_011046841, Unigene4209\_Sample\_011046841, Unigene5622\_Sample\_011046841, Unigene7281\_Sample\_011046841, Unigene8288\_Sample\_011046841, Unigene8797\_Sample\_011046841, Unigene9830\_Sample\_011046841, Unigene9876\_Sample\_011046841, Unigene10459\_Sample\_011046841, Unigene10855\_Sample\_011046841, Unigene10970\_Sample\_011046841, Unigene11573\_Sample\_011046841, Unigene11641\_Sample\_011046841, Unigene12306\_Sample\_011046841, Unigene13069\_Sample\_011046841, Unigene13481\_Sample\_011046841, Unigene13789\_Sample\_011046841, Unigene15356\_Sample\_011046841, Unigene26714\_Sample\_011046841, Unigene29183\_Sample\_011046841, Unigene35926\_Sample\_011046841, Unigene43072\_Sample\_011046841, Unigene47118\_Sample\_011046841, Unigene47924\_Sample\_011046841, Unigene51307\_Sample\_011046841, Unigene55744\_Sample\_011046841, Unigene55963\_Sample\_011046841, Unigene58460\_Sample\_011046841, Unigene59006\_Sample\_011046841, Unigene59321\_Sample\_011046841, Unigene59705\_Sample\_011046841, Unigene60173\_Sample\_011046841, Unigene60372\_Sample\_011046841, Unigene60498\_Sample\_011046841, Unigene60789\_Sample\_011046841, Unigene3202\_Sample\_011046841, Unigene3353\_Sample\_011046841, Unigene4993\_Sample\_011046841, Unigene5688\_Sample\_011046841, Unigene5744\_Sample\_011046841, Unigene6531\_Sample\_011046841, Unigene8299\_Sample\_011046841, Unigene8569\_Sample\_011046841, Unigene10325\_Sample\_011046841, Unigene11148\_Sample\_011046841, Unigene11676\_Sample\_011046841, Unigene13332\_Sample\_011046841, Unigene13825\_Sample\_011046841, Unigene35470\_Sample\_011046841, Unigene37264\_Sample\_011046841, Unigene41373\_Sample\_011046841, Unigene41574\_Sample\_011046841, Unigene47510\_Sample\_011046841, Unigene47555\_Sample\_011046841, Unigene48020\_Sample\_011046841, Unigene48342\_Sample\_011046841, Unigene49494\_Sample\_011046841, Unigene49615\_Sample\_011046841, Unigene50170\_Sample\_011046841, Unigene51148\_Sample\_011046841, Unigene55825\_Sample\_011046841, Unigene56004\_Sample\_011046841, Unigene56232\_Sample\_011046841, Unigene57566\_Sample\_011046841, Unigene58100\_Sample\_011046841, Unigene58285\_Sample\_011046841, Unigene59109\_Sample\_011046841, Unigene59742\_Sample\_011046841, Unigene60427\_Sample\_011046841, Unigene60552\_Sample\_011046841, Unigene60647\_Sample\_011046841, Unigene60880\_Sample\_011046841, Unigene2213\_Sample\_011046841, Unigene12837\_Sample\_011046841, Unigene13367\_Sample\_011046841, Unigene23590\_Sample\_011046841, Unigene27440\_Sample\_011046841, Unigene28658\_Sample\_011046841, Unigene44683\_Sample\_011046841, Unigene45662\_Sample\_011046841, Unigene52294\_Sample\_011046841, Unigene53989\_Sample\_011046841, Unigene55504\_Sample\_011046841, Unigene56062\_Sample\_011046841, Unigene58321\_Sample\_011046841, Unigene58640\_Sample\_011046841, Unigene60347\_Sample\_011046841, Unigene8139\_Sample\_011046841, Unigene32964\_Sample\_011046841, Unigene35917\_Sample\_011046841, Unigene49671\_Sample\_011046841, Unigene54240\_Sample\_011046841, Unigene55606\_Sample\_011046841, Unigene56300\_Sample\_011046841, Unigene58058\_Sample\_011046841, Unigene30190\_Sample\_011046841, Unigene42686\_Sample\_011046841, Unigene49525\_Sample\_011046841, Unigene52866\_Sample\_011046841, Unigene59421\_Sample\_011046841, Unigene59482\_Sample\_011046841, Unigene12023\_Sample\_011046841, Unigene25087\_Sample\_011046841, Unigene41980\_Sample\_011046841, Unigene43782\_Sample\_011046841, Unigene47967\_Sample\_011046841, Unigene51429\_Sample\_011046841, Unigene59010\_Sample\_011046841, Unigene60217\_Sample\_011046841, Unigene1147\_Sample\_011046841, Unigene42292\_Sample\_011046841, Unigene56828\_Sample\_011046841, Unigene18170\_Sample\_011046841, Unigene20298\_Sample\_011046841, Unigene29318\_Sample\_011046841, Unigene43515\_Sample\_011046841, Unigene48742\_Sample\_011046841, Unigene51756\_Sample\_011046841, Unigene59656\_Sample\_011046841, Unigene60248\_Sample\_011046841, Unigene60948\_Sample\_011046841, Unigene804\_Sample\_011046841, Unigene2110\_Sample\_011046841, Unigene21201\_Sample\_011046841, Unigene24065\_Sample\_011046841, Unigene26229\_Sample\_011046841, Unigene48939\_Sample\_011046841, Unigene53352\_Sample\_011046841, Unigene53995\_Sample\_011046841, Unigene55041\_Sample\_011046841, Unigene3810\_Sample\_011046841, Unigene7879\_Sample\_011046841, Unigene15705\_Sample\_011046841, Unigene21362\_Sample\_011046841, Unigene23831\_Sample\_011046841, Unigene28288\_Sample\_011046841, Unigene47910\_Sample\_011046841, Unigene50832\_Sample\_011046841, Unigene54171\_Sample\_011046841, Unigene60132\_Sample\_011046841, Unigene51582\_Sample\_011046841, Unigene12324\_Sample\_011046841, Unigene60003\_Sample\_011046841, Unigene31264\_Sample\_011046841, Unigene52096\_Sample\_011046841, Unigene58675\_Sample\_011046841, Unigene30349\_Sample\_011046841, Unigene44021\_Sample\_011046841, Unigene16442\_Sample\_011046841, Unigene30484\_Sample\_011046841, Unigene35082\_Sample\_011046841, Unigene47099\_Sample\_011046841, Unigene14248\_Sample\_011046841, Unigene43091\_Sample\_011046841, Unigene46813\_Sample\_011046841, Unigene54373\_Sample\_011046841, Unigene17997\_Sample\_011046841, Unigene30474\_Sample\_011046841, Unigene7048\_Sample\_011046841 |
| 3 | Purine metabolism Back to summary table | Unigene204\_Sample\_011046841, Unigene1049\_Sample\_011046841, Unigene1129\_Sample\_011046841, Unigene5271\_Sample\_011046841, Unigene6203\_Sample\_011046841, Unigene6724\_Sample\_011046841, Unigene7029\_Sample\_011046841, Unigene7331\_Sample\_011046841, Unigene8384\_Sample\_011046841, Unigene9330\_Sample\_011046841, Unigene12300\_Sample\_011046841, Unigene12447\_Sample\_011046841, Unigene13481\_Sample\_011046841, Unigene13500\_Sample\_011046841, Unigene13789\_Sample\_011046841, Unigene25547\_Sample\_011046841, Unigene32814\_Sample\_011046841, Unigene34404\_Sample\_011046841, Unigene38329\_Sample\_011046841, Unigene40350\_Sample\_011046841, Unigene43614\_Sample\_011046841, Unigene43894\_Sample\_011046841, Unigene44949\_Sample\_011046841, Unigene45674\_Sample\_011046841, Unigene45746\_Sample\_011046841, Unigene50611\_Sample\_011046841, Unigene50631\_Sample\_011046841, Unigene50712\_Sample\_011046841, Unigene51029\_Sample\_011046841, Unigene52799\_Sample\_011046841, Unigene53417\_Sample\_011046841, Unigene53687\_Sample\_011046841, Unigene54097\_Sample\_011046841, Unigene54870\_Sample\_011046841, Unigene55779\_Sample\_011046841, Unigene55794\_Sample\_011046841, Unigene56049\_Sample\_011046841, Unigene56182\_Sample\_011046841, Unigene56375\_Sample\_011046841, Unigene57225\_Sample\_011046841, Unigene57770\_Sample\_011046841, Unigene59194\_Sample\_011046841, Unigene60235\_Sample\_011046841, Unigene60449\_Sample\_011046841, Unigene60568\_Sample\_011046841, Unigene60743\_Sample\_011046841, Unigene60789\_Sample\_011046841, Unigene60809\_Sample\_011046841, Unigene60926\_Sample\_011046841, Unigene1158\_Sample\_011046841, Unigene2263\_Sample\_011046841, Unigene2913\_Sample\_011046841, Unigene3500\_Sample\_011046841, Unigene4916\_Sample\_011046841, Unigene5150\_Sample\_011046841, Unigene5329\_Sample\_011046841, Unigene5336\_Sample\_011046841, Unigene5382\_Sample\_011046841, Unigene5967\_Sample\_011046841, Unigene6034\_Sample\_011046841, Unigene6521\_Sample\_011046841, Unigene8938\_Sample\_011046841, Unigene10236\_Sample\_011046841, Unigene10721\_Sample\_011046841, Unigene11030\_Sample\_011046841, Unigene11283\_Sample\_011046841, Unigene11726\_Sample\_011046841, Unigene12908\_Sample\_011046841, Unigene13694\_Sample\_011046841, Unigene22428\_Sample\_011046841, Unigene26220\_Sample\_011046841, Unigene27510\_Sample\_011046841, Unigene30699\_Sample\_011046841, Unigene34859\_Sample\_011046841, Unigene39621\_Sample\_011046841, Unigene41171\_Sample\_011046841, Unigene41413\_Sample\_011046841, Unigene42325\_Sample\_011046841, Unigene42399\_Sample\_011046841, Unigene42733\_Sample\_011046841, Unigene42885\_Sample\_011046841, Unigene44275\_Sample\_011046841, Unigene45531\_Sample\_011046841, Unigene45816\_Sample\_011046841, Unigene47198\_Sample\_011046841, Unigene47221\_Sample\_011046841, Unigene47506\_Sample\_011046841, Unigene48003\_Sample\_011046841, Unigene48081\_Sample\_011046841, Unigene48094\_Sample\_011046841, Unigene48609\_Sample\_011046841, Unigene48881\_Sample\_011046841, Unigene48927\_Sample\_011046841, Unigene49640\_Sample\_011046841, Unigene49713\_Sample\_011046841, Unigene50108\_Sample\_011046841, Unigene50584\_Sample\_011046841, Unigene51933\_Sample\_011046841, Unigene52322\_Sample\_011046841, Unigene52423\_Sample\_011046841, Unigene52614\_Sample\_011046841, Unigene52769\_Sample\_011046841, Unigene53305\_Sample\_011046841, Unigene54563\_Sample\_011046841, Unigene54844\_Sample\_011046841, Unigene55492\_Sample\_011046841, Unigene55866\_Sample\_011046841, Unigene56333\_Sample\_011046841, Unigene56891\_Sample\_011046841, Unigene56938\_Sample\_011046841, Unigene56952\_Sample\_011046841, Unigene57259\_Sample\_011046841, Unigene57455\_Sample\_011046841, Unigene57560\_Sample\_011046841, Unigene57569\_Sample\_011046841, Unigene58018\_Sample\_011046841, Unigene58091\_Sample\_011046841, Unigene58152\_Sample\_011046841, Unigene58692\_Sample\_011046841, Unigene58721\_Sample\_011046841, Unigene58765\_Sample\_011046841, Unigene59330\_Sample\_011046841, Unigene59543\_Sample\_011046841, Unigene59634\_Sample\_011046841, Unigene59714\_Sample\_011046841, Unigene59742\_Sample\_011046841, Unigene59900\_Sample\_011046841, Unigene60126\_Sample\_011046841, Unigene60310\_Sample\_011046841, Unigene60469\_Sample\_011046841, Unigene60682\_Sample\_011046841, Unigene60880\_Sample\_011046841, Unigene60900\_Sample\_011046841, Unigene60919\_Sample\_011046841, Unigene60\_Sample\_011046841, Unigene967\_Sample\_011046841, Unigene5051\_Sample\_011046841, Unigene5708\_Sample\_011046841, Unigene6597\_Sample\_011046841, Unigene9806\_Sample\_011046841, Unigene10623\_Sample\_011046841, Unigene11743\_Sample\_011046841, Unigene12984\_Sample\_011046841, Unigene18609\_Sample\_011046841, Unigene19399\_Sample\_011046841, Unigene20854\_Sample\_011046841, Unigene24615\_Sample\_011046841, Unigene26928\_Sample\_011046841, Unigene30285\_Sample\_011046841, Unigene31723\_Sample\_011046841, Unigene34810\_Sample\_011046841, Unigene35210\_Sample\_011046841, Unigene41246\_Sample\_011046841, Unigene42079\_Sample\_011046841, Unigene42860\_Sample\_011046841, Unigene44391\_Sample\_011046841, Unigene48720\_Sample\_011046841, Unigene48876\_Sample\_011046841, Unigene51117\_Sample\_011046841, Unigene51481\_Sample\_011046841, Unigene51837\_Sample\_011046841, Unigene52140\_Sample\_011046841, Unigene52658\_Sample\_011046841, Unigene53838\_Sample\_011046841, Unigene54368\_Sample\_011046841, Unigene55381\_Sample\_011046841, Unigene55651\_Sample\_011046841, Unigene56098\_Sample\_011046841, Unigene56468\_Sample\_011046841, Unigene59491\_Sample\_011046841, Unigene60494\_Sample\_011046841, Unigene60606\_Sample\_011046841, Unigene60847\_Sample\_011046841, Unigene2821\_Sample\_011046841, Unigene5875\_Sample\_011046841, Unigene7129\_Sample\_011046841, Unigene8139\_Sample\_011046841, Unigene8750\_Sample\_011046841, Unigene10446\_Sample\_011046841, Unigene13099\_Sample\_011046841, Unigene22992\_Sample\_011046841, Unigene27899\_Sample\_011046841, Unigene32497\_Sample\_011046841, Unigene35737\_Sample\_011046841, Unigene39016\_Sample\_011046841, Unigene39766\_Sample\_011046841, Unigene40503\_Sample\_011046841, Unigene42794\_Sample\_011046841, Unigene44282\_Sample\_011046841, Unigene46297\_Sample\_011046841, Unigene47129\_Sample\_011046841, Unigene50609\_Sample\_011046841, Unigene54965\_Sample\_011046841, Unigene56270\_Sample\_011046841, Unigene59492\_Sample\_011046841, Unigene1748\_Sample\_011046841, Unigene39617\_Sample\_011046841, Unigene47187\_Sample\_011046841, Unigene50947\_Sample\_011046841, Unigene59545\_Sample\_011046841, Unigene5191\_Sample\_011046841, Unigene11735\_Sample\_011046841, Unigene12261\_Sample\_011046841, Unigene18785\_Sample\_011046841, Unigene21456\_Sample\_011046841, Unigene29803\_Sample\_011046841, Unigene29849\_Sample\_011046841, Unigene31394\_Sample\_011046841, Unigene34352\_Sample\_011046841, Unigene42159\_Sample\_011046841, Unigene43782\_Sample\_011046841, Unigene49168\_Sample\_011046841, Unigene49446\_Sample\_011046841, Unigene60596\_Sample\_011046841, Unigene9211\_Sample\_011046841, Unigene11712\_Sample\_011046841, Unigene18805\_Sample\_011046841, Unigene37401\_Sample\_011046841, Unigene53491\_Sample\_011046841, Unigene6972\_Sample\_011046841, Unigene11232\_Sample\_011046841, Unigene18715\_Sample\_011046841, Unigene25764\_Sample\_011046841, Unigene45777\_Sample\_011046841, Unigene48730\_Sample\_011046841, Unigene48742\_Sample\_011046841, Unigene54790\_Sample\_011046841, Unigene59567\_Sample\_011046841, Unigene60458\_Sample\_011046841, Unigene8080\_Sample\_011046841, Unigene37427\_Sample\_011046841, Unigene57802\_Sample\_011046841, Unigene8266\_Sample\_011046841, Unigene21201\_Sample\_011046841, Unigene28225\_Sample\_011046841, Unigene37087\_Sample\_011046841, Unigene41153\_Sample\_011046841, Unigene45345\_Sample\_011046841, Unigene45721\_Sample\_011046841, Unigene47820\_Sample\_011046841, Unigene49106\_Sample\_011046841, Unigene50506\_Sample\_011046841, Unigene53073\_Sample\_011046841, Unigene60441\_Sample\_011046841, Unigene1594\_Sample\_011046841, Unigene4847\_Sample\_011046841, Unigene31129\_Sample\_011046841, Unigene33976\_Sample\_011046841, Unigene36984\_Sample\_011046841, Unigene40559\_Sample\_011046841, Unigene42184\_Sample\_011046841, Unigene42508\_Sample\_011046841, Unigene42749\_Sample\_011046841, Unigene43528\_Sample\_011046841, Unigene43813\_Sample\_011046841, Unigene46287\_Sample\_011046841, Unigene47540\_Sample\_011046841, Unigene49204\_Sample\_011046841, Unigene54638\_Sample\_011046841, Unigene27881\_Sample\_011046841, Unigene35836\_Sample\_011046841, Unigene44453\_Sample\_011046841, Unigene51997\_Sample\_011046841, Unigene33510\_Sample\_011046841, Unigene16924\_Sample\_011046841, Unigene30309\_Sample\_011046841, Unigene11166\_Sample\_011046841, Unigene27363\_Sample\_011046841, Unigene28077\_Sample\_011046841, Unigene30743\_Sample\_011046841, Unigene23897\_Sample\_011046841, Unigene27719\_Sample\_011046841, Unigene17439\_Sample\_011046841, Unigene20893\_Sample\_011046841, Unigene34971\_Sample\_011046841, Unigene35708\_Sample\_011046841, Unigene43380\_Sample\_011046841, Unigene31664\_Sample\_011046841, Unigene54187\_Sample\_011046841, Unigene34466\_Sample\_011046841, Unigene40544\_Sample\_011046841, Unigene20294\_Sample\_011046841, Unigene22482\_Sample\_011046841, Unigene47587\_Sample\_011046841, Unigene37430\_Sample\_011046841, Unigene31679\_Sample\_011046841, Unigene38646\_Sample\_011046841, Unigene52018\_Sample\_011046841, Unigene21775\_Sample\_011046841, Unigene7048\_Sample\_011046841, Unigene29124\_Sample\_011046841, Unigene38066\_Sample\_011046841, Unigene18899\_Sample\_011046841 |
| 4 | Meiosis - yeast Back to summary table | Unigene517\_Sample\_011046841, Unigene1437\_Sample\_011046841, Unigene2686\_Sample\_011046841, Unigene5271\_Sample\_011046841, Unigene5402\_Sample\_011046841, Unigene5622\_Sample\_011046841, Unigene7331\_Sample\_011046841, Unigene7905\_Sample\_011046841, Unigene9876\_Sample\_011046841, Unigene11708\_Sample\_011046841, Unigene12306\_Sample\_011046841, Unigene12670\_Sample\_011046841, Unigene13939\_Sample\_011046841, Unigene19684\_Sample\_011046841, Unigene28326\_Sample\_011046841, Unigene38234\_Sample\_011046841, Unigene39875\_Sample\_011046841, Unigene40567\_Sample\_011046841, Unigene43331\_Sample\_011046841, Unigene45022\_Sample\_011046841, Unigene45746\_Sample\_011046841, Unigene45790\_Sample\_011046841, Unigene51697\_Sample\_011046841, Unigene52432\_Sample\_011046841, Unigene53888\_Sample\_011046841, Unigene55432\_Sample\_011046841, Unigene55744\_Sample\_011046841, Unigene56258\_Sample\_011046841, Unigene56389\_Sample\_011046841, Unigene56719\_Sample\_011046841, Unigene57076\_Sample\_011046841, Unigene59512\_Sample\_011046841, Unigene59588\_Sample\_011046841, Unigene59730\_Sample\_011046841, Unigene60372\_Sample\_011046841, Unigene60449\_Sample\_011046841, Unigene60568\_Sample\_011046841, Unigene60712\_Sample\_011046841, Unigene60755\_Sample\_011046841, Unigene4131\_Sample\_011046841, Unigene5744\_Sample\_011046841, Unigene8931\_Sample\_011046841, Unigene10325\_Sample\_011046841, Unigene11148\_Sample\_011046841, Unigene12971\_Sample\_011046841, Unigene13494\_Sample\_011046841, Unigene13825\_Sample\_011046841, Unigene23312\_Sample\_011046841, Unigene25098\_Sample\_011046841, Unigene26055\_Sample\_011046841, Unigene27694\_Sample\_011046841, Unigene30076\_Sample\_011046841, Unigene30495\_Sample\_011046841, Unigene40648\_Sample\_011046841, Unigene42106\_Sample\_011046841, Unigene43448\_Sample\_011046841, Unigene46763\_Sample\_011046841, Unigene47368\_Sample\_011046841, Unigene48020\_Sample\_011046841, Unigene49320\_Sample\_011046841, Unigene52184\_Sample\_011046841, Unigene52869\_Sample\_011046841, Unigene54454\_Sample\_011046841, Unigene56010\_Sample\_011046841, Unigene56663\_Sample\_011046841, Unigene56883\_Sample\_011046841, Unigene57082\_Sample\_011046841, Unigene57835\_Sample\_011046841, Unigene57929\_Sample\_011046841, Unigene58091\_Sample\_011046841, Unigene58169\_Sample\_011046841, Unigene58496\_Sample\_011046841, Unigene59543\_Sample\_011046841, Unigene59696\_Sample\_011046841, Unigene60006\_Sample\_011046841, Unigene2213\_Sample\_011046841, Unigene3491\_Sample\_011046841, Unigene13203\_Sample\_011046841, Unigene13430\_Sample\_011046841, Unigene23751\_Sample\_011046841, Unigene27440\_Sample\_011046841, Unigene34582\_Sample\_011046841, Unigene41246\_Sample\_011046841, Unigene42721\_Sample\_011046841, Unigene48072\_Sample\_011046841, Unigene48647\_Sample\_011046841, Unigene53168\_Sample\_011046841, Unigene54593\_Sample\_011046841, Unigene57678\_Sample\_011046841, Unigene60347\_Sample\_011046841, Unigene27828\_Sample\_011046841, Unigene35757\_Sample\_011046841, Unigene35917\_Sample\_011046841, Unigene37549\_Sample\_011046841, Unigene53041\_Sample\_011046841, Unigene58502\_Sample\_011046841, Unigene11000\_Sample\_011046841, Unigene12412\_Sample\_011046841, Unigene18699\_Sample\_011046841, Unigene21917\_Sample\_011046841, Unigene28681\_Sample\_011046841, Unigene30190\_Sample\_011046841, Unigene55251\_Sample\_011046841, Unigene7092\_Sample\_011046841, Unigene12023\_Sample\_011046841, Unigene41980\_Sample\_011046841, Unigene51429\_Sample\_011046841, Unigene57239\_Sample\_011046841, Unigene57304\_Sample\_011046841, Unigene58515\_Sample\_011046841, Unigene59178\_Sample\_011046841, Unigene60375\_Sample\_011046841, Unigene1147\_Sample\_011046841, Unigene15792\_Sample\_011046841, Unigene42292\_Sample\_011046841, Unigene55286\_Sample\_011046841, Unigene55840\_Sample\_011046841, Unigene29365\_Sample\_011046841, Unigene47753\_Sample\_011046841, Unigene59290\_Sample\_011046841, Unigene60948\_Sample\_011046841, Unigene47947\_Sample\_011046841, Unigene2110\_Sample\_011046841, Unigene24065\_Sample\_011046841, Unigene32103\_Sample\_011046841, Unigene48731\_Sample\_011046841, Unigene48939\_Sample\_011046841, Unigene55041\_Sample\_011046841, Unigene11415\_Sample\_011046841, Unigene15705\_Sample\_011046841, Unigene23831\_Sample\_011046841, Unigene28288\_Sample\_011046841, Unigene28348\_Sample\_011046841, Unigene33437\_Sample\_011046841, Unigene37595\_Sample\_011046841, Unigene39991\_Sample\_011046841, Unigene43918\_Sample\_011046841, Unigene44838\_Sample\_011046841, Unigene50550\_Sample\_011046841, Unigene17004\_Sample\_011046841, Unigene47083\_Sample\_011046841, Unigene12324\_Sample\_011046841, Unigene17192\_Sample\_011046841, Unigene32523\_Sample\_011046841, Unigene40117\_Sample\_011046841, Unigene44021\_Sample\_011046841, Unigene34971\_Sample\_011046841, Unigene31664\_Sample\_011046841, Unigene35082\_Sample\_011046841, Unigene40934\_Sample\_011046841, Unigene43097\_Sample\_011046841, Unigene14248\_Sample\_011046841, Unigene19789\_Sample\_011046841, Unigene6639\_Sample\_011046841, Unigene32977\_Sample\_011046841, Unigene39302\_Sample\_011046841 |
| 5 | ErbB signaling pathway Back to summary table | Unigene865\_Sample\_011046841, Unigene3199\_Sample\_011046841, Unigene4209\_Sample\_011046841, Unigene6324\_Sample\_011046841, Unigene7143\_Sample\_011046841, Unigene7809\_Sample\_011046841, Unigene9082\_Sample\_011046841, Unigene10459\_Sample\_011046841, Unigene10970\_Sample\_011046841, Unigene11073\_Sample\_011046841, Unigene11367\_Sample\_011046841, Unigene11706\_Sample\_011046841, Unigene12652\_Sample\_011046841, Unigene13069\_Sample\_011046841, Unigene13901\_Sample\_011046841, Unigene39219\_Sample\_011046841, Unigene40230\_Sample\_011046841, Unigene43963\_Sample\_011046841, Unigene47292\_Sample\_011046841, Unigene52094\_Sample\_011046841, Unigene53568\_Sample\_011046841, Unigene58460\_Sample\_011046841, Unigene58987\_Sample\_011046841, Unigene59022\_Sample\_011046841, Unigene59559\_Sample\_011046841, Unigene59978\_Sample\_011046841, Unigene60139\_Sample\_011046841, Unigene60173\_Sample\_011046841, Unigene60498\_Sample\_011046841, Unigene1414\_Sample\_011046841, Unigene3363\_Sample\_011046841, Unigene4892\_Sample\_011046841, Unigene9867\_Sample\_011046841, Unigene12943\_Sample\_011046841, Unigene13332\_Sample\_011046841, Unigene13855\_Sample\_011046841, Unigene27973\_Sample\_011046841, Unigene32409\_Sample\_011046841, Unigene34259\_Sample\_011046841, Unigene39360\_Sample\_011046841, Unigene40209\_Sample\_011046841, Unigene41574\_Sample\_011046841, Unigene42561\_Sample\_011046841, Unigene42820\_Sample\_011046841, Unigene46142\_Sample\_011046841, Unigene47555\_Sample\_011046841, Unigene47936\_Sample\_011046841, Unigene49534\_Sample\_011046841, Unigene50039\_Sample\_011046841, Unigene50674\_Sample\_011046841, Unigene50677\_Sample\_011046841, Unigene54900\_Sample\_011046841, Unigene54932\_Sample\_011046841, Unigene56232\_Sample\_011046841, Unigene56271\_Sample\_011046841, Unigene56681\_Sample\_011046841, Unigene56798\_Sample\_011046841, Unigene57037\_Sample\_011046841, Unigene57566\_Sample\_011046841, Unigene58100\_Sample\_011046841, Unigene58209\_Sample\_011046841, Unigene58669\_Sample\_011046841, Unigene58821\_Sample\_011046841, Unigene60190\_Sample\_011046841, Unigene60787\_Sample\_011046841, Unigene60859\_Sample\_011046841, Unigene5972\_Sample\_011046841, Unigene11932\_Sample\_011046841, Unigene13367\_Sample\_011046841, Unigene23590\_Sample\_011046841, Unigene28658\_Sample\_011046841, Unigene31181\_Sample\_011046841, Unigene40758\_Sample\_011046841, Unigene46930\_Sample\_011046841, Unigene53049\_Sample\_011046841, Unigene54574\_Sample\_011046841, Unigene55312\_Sample\_011046841, Unigene57775\_Sample\_011046841, Unigene58021\_Sample\_011046841, Unigene59581\_Sample\_011046841, Unigene60765\_Sample\_011046841, Unigene32964\_Sample\_011046841, Unigene44532\_Sample\_011046841, Unigene53642\_Sample\_011046841, Unigene56300\_Sample\_011046841, Unigene58058\_Sample\_011046841, Unigene59711\_Sample\_011046841, Unigene60863\_Sample\_011046841, Unigene49525\_Sample\_011046841, Unigene55486\_Sample\_011046841, Unigene57665\_Sample\_011046841, Unigene1107\_Sample\_011046841, Unigene2942\_Sample\_011046841, Unigene9329\_Sample\_011046841, Unigene38674\_Sample\_011046841, Unigene41032\_Sample\_011046841, Unigene43878\_Sample\_011046841, Unigene47967\_Sample\_011046841, Unigene60217\_Sample\_011046841, Unigene7042\_Sample\_011046841, Unigene51301\_Sample\_011046841, Unigene29318\_Sample\_011046841, Unigene47054\_Sample\_011046841, Unigene51756\_Sample\_011046841, Unigene55344\_Sample\_011046841, Unigene56139\_Sample\_011046841, Unigene57006\_Sample\_011046841, Unigene57641\_Sample\_011046841, Unigene16098\_Sample\_011046841, Unigene46589\_Sample\_011046841, Unigene39189\_Sample\_011046841, Unigene53995\_Sample\_011046841, Unigene12521\_Sample\_011046841, Unigene39020\_Sample\_011046841, Unigene43099\_Sample\_011046841, Unigene47686\_Sample\_011046841, Unigene47872\_Sample\_011046841, Unigene47910\_Sample\_011046841, Unigene50832\_Sample\_011046841, Unigene60132\_Sample\_011046841, Unigene52183\_Sample\_011046841, Unigene16539\_Sample\_011046841, Unigene31544\_Sample\_011046841, Unigene41406\_Sample\_011046841, Unigene43718\_Sample\_011046841, Unigene17671\_Sample\_011046841, Unigene20770\_Sample\_011046841, Unigene50713\_Sample\_011046841, Unigene41464\_Sample\_011046841, Unigene44354\_Sample\_011046841, Unigene21539\_Sample\_011046841, Unigene32258\_Sample\_011046841, Unigene48345\_Sample\_011046841, Unigene4308\_Sample\_011046841, Unigene46813\_Sample\_011046841, Unigene16813\_Sample\_011046841, Unigene26184\_Sample\_011046841, Unigene39361\_Sample\_011046841, Unigene30474\_Sample\_011046841, Unigene18796\_Sample\_011046841, Unigene26777\_Sample\_011046841 |
| 6 | T cell receptor signaling pathway Back to summary table | Unigene679\_Sample\_011046841, Unigene865\_Sample\_011046841, Unigene4209\_Sample\_011046841, Unigene7143\_Sample\_011046841, Unigene7245\_Sample\_011046841, Unigene8429\_Sample\_011046841, Unigene9774\_Sample\_011046841, Unigene10459\_Sample\_011046841, Unigene13069\_Sample\_011046841, Unigene13901\_Sample\_011046841, Unigene39219\_Sample\_011046841, Unigene43963\_Sample\_011046841, Unigene48524\_Sample\_011046841, Unigene51426\_Sample\_011046841, Unigene52094\_Sample\_011046841, Unigene52144\_Sample\_011046841, Unigene56697\_Sample\_011046841, Unigene57114\_Sample\_011046841, Unigene57298\_Sample\_011046841, Unigene57609\_Sample\_011046841, Unigene57887\_Sample\_011046841, Unigene58460\_Sample\_011046841, Unigene58987\_Sample\_011046841, Unigene59022\_Sample\_011046841, Unigene59559\_Sample\_011046841, Unigene59978\_Sample\_011046841, Unigene60139\_Sample\_011046841, Unigene60173\_Sample\_011046841, Unigene60252\_Sample\_011046841, Unigene60396\_Sample\_011046841, Unigene60498\_Sample\_011046841, Unigene4673\_Sample\_011046841, Unigene7709\_Sample\_011046841, Unigene13332\_Sample\_011046841, Unigene13855\_Sample\_011046841, Unigene13945\_Sample\_011046841, Unigene21418\_Sample\_011046841, Unigene32409\_Sample\_011046841, Unigene41574\_Sample\_011046841, Unigene42561\_Sample\_011046841, Unigene42820\_Sample\_011046841, Unigene43409\_Sample\_011046841, Unigene45592\_Sample\_011046841, Unigene46219\_Sample\_011046841, Unigene47210\_Sample\_011046841, Unigene47555\_Sample\_011046841, Unigene50170\_Sample\_011046841, Unigene53096\_Sample\_011046841, Unigene53264\_Sample\_011046841, Unigene53447\_Sample\_011046841, Unigene54900\_Sample\_011046841, Unigene56271\_Sample\_011046841, Unigene56681\_Sample\_011046841, Unigene57566\_Sample\_011046841, Unigene58100\_Sample\_011046841, Unigene58611\_Sample\_011046841, Unigene58669\_Sample\_011046841, Unigene59756\_Sample\_011046841, Unigene60190\_Sample\_011046841, Unigene60427\_Sample\_011046841, Unigene5972\_Sample\_011046841, Unigene23218\_Sample\_011046841, Unigene23590\_Sample\_011046841, Unigene25784\_Sample\_011046841, Unigene28658\_Sample\_011046841, Unigene29578\_Sample\_011046841, Unigene40758\_Sample\_011046841, Unigene49837\_Sample\_011046841, Unigene54574\_Sample\_011046841, Unigene55312\_Sample\_011046841, Unigene59322\_Sample\_011046841, Unigene59581\_Sample\_011046841, Unigene60765\_Sample\_011046841, Unigene2116\_Sample\_011046841, Unigene11547\_Sample\_011046841, Unigene19951\_Sample\_011046841, Unigene32964\_Sample\_011046841, Unigene53642\_Sample\_011046841, Unigene56300\_Sample\_011046841, Unigene59711\_Sample\_011046841, Unigene15\_Sample\_011046841, Unigene29346\_Sample\_011046841, Unigene49525\_Sample\_011046841, Unigene55486\_Sample\_011046841, Unigene59421\_Sample\_011046841, Unigene3797\_Sample\_011046841, Unigene11231\_Sample\_011046841, Unigene43878\_Sample\_011046841, Unigene7042\_Sample\_011046841, Unigene51301\_Sample\_011046841, Unigene29318\_Sample\_011046841, Unigene47054\_Sample\_011046841, Unigene51756\_Sample\_011046841, Unigene52573\_Sample\_011046841, Unigene57006\_Sample\_011046841, Unigene57641\_Sample\_011046841, Unigene39189\_Sample\_011046841, Unigene21362\_Sample\_011046841, Unigene47429\_Sample\_011046841, Unigene47910\_Sample\_011046841, Unigene49087\_Sample\_011046841, Unigene50832\_Sample\_011046841, Unigene54663\_Sample\_011046841, Unigene31969\_Sample\_011046841, Unigene31264\_Sample\_011046841, Unigene17671\_Sample\_011046841, Unigene20770\_Sample\_011046841, Unigene50713\_Sample\_011046841, Unigene16442\_Sample\_011046841, Unigene30484\_Sample\_011046841, Unigene41464\_Sample\_011046841, Unigene19937\_Sample\_011046841, Unigene32258\_Sample\_011046841, Unigene46813\_Sample\_011046841, Unigene16813\_Sample\_011046841, Unigene47389\_Sample\_011046841, Unigene26184\_Sample\_011046841, Unigene30474\_Sample\_011046841, Unigene19596\_Sample\_011046841 |
| 7 | Prostate cancer Back to summary table | Unigene865\_Sample\_011046841, Unigene4209\_Sample\_011046841, Unigene4963\_Sample\_011046841, Unigene7143\_Sample\_011046841, Unigene7809\_Sample\_011046841, Unigene9082\_Sample\_011046841, Unigene9668\_Sample\_011046841, Unigene9858\_Sample\_011046841, Unigene10459\_Sample\_011046841, Unigene10640\_Sample\_011046841, Unigene10744\_Sample\_011046841, Unigene10970\_Sample\_011046841, Unigene11367\_Sample\_011046841, Unigene11706\_Sample\_011046841, Unigene13069\_Sample\_011046841, Unigene13872\_Sample\_011046841, Unigene23925\_Sample\_011046841, Unigene26714\_Sample\_011046841, Unigene35926\_Sample\_011046841, Unigene37440\_Sample\_011046841, Unigene45114\_Sample\_011046841, Unigene56886\_Sample\_011046841, Unigene58307\_Sample\_011046841, Unigene58460\_Sample\_011046841, Unigene58987\_Sample\_011046841, Unigene59022\_Sample\_011046841, Unigene59151\_Sample\_011046841, Unigene59705\_Sample\_011046841, Unigene60173\_Sample\_011046841, Unigene60498\_Sample\_011046841, Unigene1414\_Sample\_011046841, Unigene4892\_Sample\_011046841, Unigene5744\_Sample\_011046841, Unigene8299\_Sample\_011046841, Unigene8641\_Sample\_011046841, Unigene9408\_Sample\_011046841, Unigene11676\_Sample\_011046841, Unigene12943\_Sample\_011046841, Unigene13855\_Sample\_011046841, Unigene40989\_Sample\_011046841, Unigene41574\_Sample\_011046841, Unigene42561\_Sample\_011046841, Unigene42820\_Sample\_011046841, Unigene47555\_Sample\_011046841, Unigene48337\_Sample\_011046841, Unigene49494\_Sample\_011046841, Unigene50674\_Sample\_011046841, Unigene50963\_Sample\_011046841, Unigene51148\_Sample\_011046841, Unigene54859\_Sample\_011046841, Unigene54900\_Sample\_011046841, Unigene55825\_Sample\_011046841, Unigene56004\_Sample\_011046841, Unigene56681\_Sample\_011046841, Unigene56798\_Sample\_011046841, Unigene57566\_Sample\_011046841, Unigene58100\_Sample\_011046841, Unigene58466\_Sample\_011046841, Unigene58833\_Sample\_011046841, Unigene59109\_Sample\_011046841, Unigene59822\_Sample\_011046841, Unigene60190\_Sample\_011046841, Unigene60219\_Sample\_011046841, Unigene60552\_Sample\_011046841, Unigene9726\_Sample\_011046841, Unigene11932\_Sample\_011046841, Unigene12975\_Sample\_011046841, Unigene23590\_Sample\_011046841, Unigene28658\_Sample\_011046841, Unigene31181\_Sample\_011046841, Unigene40758\_Sample\_011046841, Unigene52294\_Sample\_011046841, Unigene53989\_Sample\_011046841, Unigene54574\_Sample\_011046841, Unigene56062\_Sample\_011046841, Unigene57775\_Sample\_011046841, Unigene58321\_Sample\_011046841, Unigene60765\_Sample\_011046841, Unigene486\_Sample\_011046841, Unigene32964\_Sample\_011046841, Unigene56300\_Sample\_011046841, Unigene59061\_Sample\_011046841, Unigene60102\_Sample\_011046841, Unigene60863\_Sample\_011046841, Unigene49525\_Sample\_011046841, Unigene59482\_Sample\_011046841, Unigene1107\_Sample\_011046841, Unigene47967\_Sample\_011046841, Unigene18170\_Sample\_011046841, Unigene29318\_Sample\_011046841, Unigene47054\_Sample\_011046841, Unigene51756\_Sample\_011046841, Unigene55344\_Sample\_011046841, Unigene57117\_Sample\_011046841, Unigene59656\_Sample\_011046841, Unigene60248\_Sample\_011046841, Unigene804\_Sample\_011046841, Unigene27016\_Sample\_011046841, Unigene53995\_Sample\_011046841, Unigene7879\_Sample\_011046841, Unigene47910\_Sample\_011046841, Unigene50832\_Sample\_011046841, Unigene59748\_Sample\_011046841, Unigene51582\_Sample\_011046841, Unigene23857\_Sample\_011046841, Unigene28384\_Sample\_011046841, Unigene43718\_Sample\_011046841, Unigene32258\_Sample\_011046841, Unigene4308\_Sample\_011046841, Unigene43091\_Sample\_011046841, Unigene46813\_Sample\_011046841, Unigene39361\_Sample\_011046841, Unigene30474\_Sample\_011046841, Unigene26777\_Sample\_011046841 |
| 8 | Cell cycle Back to summary table | Unigene517\_Sample\_011046841, Unigene1437\_Sample\_011046841, Unigene5122\_Sample\_011046841, Unigene5622\_Sample\_011046841, Unigene6324\_Sample\_011046841, Unigene7281\_Sample\_011046841, Unigene7905\_Sample\_011046841, Unigene8288\_Sample\_011046841, Unigene8429\_Sample\_011046841, Unigene8797\_Sample\_011046841, Unigene9668\_Sample\_011046841, Unigene9830\_Sample\_011046841, Unigene9876\_Sample\_011046841, Unigene10427\_Sample\_011046841, Unigene10640\_Sample\_011046841, Unigene10855\_Sample\_011046841, Unigene11073\_Sample\_011046841, Unigene11114\_Sample\_011046841, Unigene11573\_Sample\_011046841, Unigene11641\_Sample\_011046841, Unigene11708\_Sample\_011046841, Unigene12306\_Sample\_011046841, Unigene12510\_Sample\_011046841, Unigene12652\_Sample\_011046841, Unigene12670\_Sample\_011046841, Unigene13872\_Sample\_011046841, Unigene13939\_Sample\_011046841, Unigene17090\_Sample\_011046841, Unigene19684\_Sample\_011046841, Unigene27324\_Sample\_011046841, Unigene32059\_Sample\_011046841, Unigene40567\_Sample\_011046841, Unigene43072\_Sample\_011046841, Unigene45114\_Sample\_011046841, Unigene45790\_Sample\_011046841, Unigene47118\_Sample\_011046841, Unigene47292\_Sample\_011046841, Unigene48058\_Sample\_011046841, Unigene49649\_Sample\_011046841, Unigene51307\_Sample\_011046841, Unigene51617\_Sample\_011046841, Unigene52432\_Sample\_011046841, Unigene53021\_Sample\_011046841, Unigene55432\_Sample\_011046841, Unigene55744\_Sample\_011046841, Unigene55963\_Sample\_011046841, Unigene56258\_Sample\_011046841, Unigene56719\_Sample\_011046841, Unigene57076\_Sample\_011046841, Unigene57083\_Sample\_011046841, Unigene58164\_Sample\_011046841, Unigene59006\_Sample\_011046841, Unigene59022\_Sample\_011046841, Unigene59321\_Sample\_011046841, Unigene59512\_Sample\_011046841, Unigene59588\_Sample\_011046841, Unigene59633\_Sample\_011046841, Unigene59730\_Sample\_011046841, Unigene60372\_Sample\_011046841, Unigene60712\_Sample\_011046841, Unigene60755\_Sample\_011046841, Unigene60877\_Sample\_011046841, Unigene3353\_Sample\_011046841, Unigene4131\_Sample\_011046841, Unigene5688\_Sample\_011046841, Unigene5747\_Sample\_011046841, Unigene6531\_Sample\_011046841, Unigene6590\_Sample\_011046841, Unigene7766\_Sample\_011046841, Unigene8569\_Sample\_011046841, Unigene8641\_Sample\_011046841, Unigene9985\_Sample\_011046841, Unigene10325\_Sample\_011046841, Unigene10683\_Sample\_011046841, Unigene10878\_Sample\_011046841, Unigene11148\_Sample\_011046841, Unigene11998\_Sample\_011046841, Unigene12170\_Sample\_011046841, Unigene12309\_Sample\_011046841, Unigene12971\_Sample\_011046841, Unigene13825\_Sample\_011046841, Unigene19273\_Sample\_011046841, Unigene26055\_Sample\_011046841, Unigene27694\_Sample\_011046841, Unigene30076\_Sample\_011046841, Unigene30295\_Sample\_011046841, Unigene30495\_Sample\_011046841, Unigene35470\_Sample\_011046841, Unigene37264\_Sample\_011046841, Unigene37278\_Sample\_011046841, Unigene39877\_Sample\_011046841, Unigene41845\_Sample\_011046841, Unigene42561\_Sample\_011046841, Unigene42820\_Sample\_011046841, Unigene47368\_Sample\_011046841, Unigene47510\_Sample\_011046841, Unigene48020\_Sample\_011046841, Unigene48342\_Sample\_011046841, Unigene49399\_Sample\_011046841, Unigene49615\_Sample\_011046841, Unigene50674\_Sample\_011046841, Unigene52869\_Sample\_011046841, Unigene54454\_Sample\_011046841, Unigene54932\_Sample\_011046841, Unigene56400\_Sample\_011046841, Unigene56883\_Sample\_011046841, Unigene57082\_Sample\_011046841, Unigene57390\_Sample\_011046841, Unigene57835\_Sample\_011046841, Unigene57929\_Sample\_011046841, Unigene58169\_Sample\_011046841, Unigene58209\_Sample\_011046841, Unigene58496\_Sample\_011046841, Unigene58564\_Sample\_011046841, Unigene58697\_Sample\_011046841, Unigene59036\_Sample\_011046841, Unigene59593\_Sample\_011046841, Unigene60086\_Sample\_011046841, Unigene60190\_Sample\_011046841, Unigene60427\_Sample\_011046841, Unigene60647\_Sample\_011046841, Unigene60859\_Sample\_011046841, Unigene2066\_Sample\_011046841, Unigene2213\_Sample\_011046841, Unigene8828\_Sample\_011046841, Unigene12837\_Sample\_011046841, Unigene13203\_Sample\_011046841, Unigene13430\_Sample\_011046841, Unigene18850\_Sample\_011046841, Unigene20297\_Sample\_011046841, Unigene20484\_Sample\_011046841, Unigene23671\_Sample\_011046841, Unigene25812\_Sample\_011046841, Unigene27440\_Sample\_011046841, Unigene30120\_Sample\_011046841, Unigene32413\_Sample\_011046841, Unigene42721\_Sample\_011046841, Unigene44683\_Sample\_011046841, Unigene45662\_Sample\_011046841, Unigene48072\_Sample\_011046841, Unigene48647\_Sample\_011046841, Unigene50396\_Sample\_011046841, Unigene51775\_Sample\_011046841, Unigene51803\_Sample\_011046841, Unigene54574\_Sample\_011046841, Unigene55183\_Sample\_011046841, Unigene56062\_Sample\_011046841, Unigene57678\_Sample\_011046841, Unigene58289\_Sample\_011046841, Unigene58640\_Sample\_011046841, Unigene60347\_Sample\_011046841, Unigene35917\_Sample\_011046841, Unigene37549\_Sample\_011046841, Unigene48297\_Sample\_011046841, Unigene49211\_Sample\_011046841, Unigene50901\_Sample\_011046841, Unigene53041\_Sample\_011046841, Unigene54240\_Sample\_011046841, Unigene55606\_Sample\_011046841, Unigene55769\_Sample\_011046841, Unigene57604\_Sample\_011046841, Unigene58502\_Sample\_011046841, Unigene11000\_Sample\_011046841, Unigene12412\_Sample\_011046841, Unigene18699\_Sample\_011046841, Unigene21917\_Sample\_011046841, Unigene28681\_Sample\_011046841, Unigene36463\_Sample\_011046841, Unigene42686\_Sample\_011046841, Unigene55121\_Sample\_011046841, Unigene55251\_Sample\_011046841, Unigene7092\_Sample\_011046841, Unigene12023\_Sample\_011046841, Unigene25087\_Sample\_011046841, Unigene34657\_Sample\_011046841, Unigene41032\_Sample\_011046841, Unigene41980\_Sample\_011046841, Unigene52611\_Sample\_011046841, Unigene57239\_Sample\_011046841, Unigene59178\_Sample\_011046841, Unigene60375\_Sample\_011046841, Unigene1147\_Sample\_011046841, Unigene42292\_Sample\_011046841, Unigene55840\_Sample\_011046841, Unigene56828\_Sample\_011046841, Unigene60805\_Sample\_011046841, Unigene13688\_Sample\_011046841, Unigene20298\_Sample\_011046841, Unigene30153\_Sample\_011046841, Unigene41514\_Sample\_011046841, Unigene43515\_Sample\_011046841, Unigene47753\_Sample\_011046841, Unigene47915\_Sample\_011046841, Unigene57117\_Sample\_011046841, Unigene59290\_Sample\_011046841, Unigene60948\_Sample\_011046841, Unigene10605\_Sample\_011046841, Unigene20381\_Sample\_011046841, Unigene24065\_Sample\_011046841, Unigene48731\_Sample\_011046841, Unigene48939\_Sample\_011046841, Unigene3810\_Sample\_011046841, Unigene7879\_Sample\_011046841, Unigene11415\_Sample\_011046841, Unigene18975\_Sample\_011046841, Unigene28348\_Sample\_011046841, Unigene33437\_Sample\_011046841, Unigene37595\_Sample\_011046841, Unigene45724\_Sample\_011046841, Unigene47686\_Sample\_011046841, Unigene54104\_Sample\_011046841, Unigene54171\_Sample\_011046841, Unigene59748\_Sample\_011046841, Unigene12390\_Sample\_011046841, Unigene25028\_Sample\_011046841, Unigene26961\_Sample\_011046841, Unigene31969\_Sample\_011046841, Unigene14639\_Sample\_011046841, Unigene60003\_Sample\_011046841, Unigene17192\_Sample\_011046841, Unigene23954\_Sample\_011046841, Unigene28384\_Sample\_011046841, Unigene28643\_Sample\_011046841, Unigene47025\_Sample\_011046841, Unigene58675\_Sample\_011046841, Unigene30349\_Sample\_011046841, Unigene40117\_Sample\_011046841, Unigene42633\_Sample\_011046841, Unigene47099\_Sample\_011046841, Unigene46652\_Sample\_011046841, Unigene54373\_Sample\_011046841 |
| 9 | Epithelial cell signaling in Helicobacter pylori infection Back to summary table | Unigene7143\_Sample\_011046841, Unigene7158\_Sample\_011046841, Unigene7809\_Sample\_011046841, Unigene8035\_Sample\_011046841, Unigene9858\_Sample\_011046841, Unigene11055\_Sample\_011046841, Unigene13370\_Sample\_011046841, Unigene13901\_Sample\_011046841, Unigene39219\_Sample\_011046841, Unigene41694\_Sample\_011046841, Unigene43500\_Sample\_011046841, Unigene43936\_Sample\_011046841, Unigene46525\_Sample\_011046841, Unigene50548\_Sample\_011046841, Unigene52094\_Sample\_011046841, Unigene54246\_Sample\_011046841, Unigene55020\_Sample\_011046841, Unigene56437\_Sample\_011046841, Unigene56818\_Sample\_011046841, Unigene57236\_Sample\_011046841, Unigene57381\_Sample\_011046841, Unigene57947\_Sample\_011046841, Unigene57973\_Sample\_011046841, Unigene59374\_Sample\_011046841, Unigene59559\_Sample\_011046841, Unigene60269\_Sample\_011046841, Unigene1414\_Sample\_011046841, Unigene3620\_Sample\_011046841, Unigene4916\_Sample\_011046841, Unigene8020\_Sample\_011046841, Unigene9572\_Sample\_011046841, Unigene10198\_Sample\_011046841, Unigene12943\_Sample\_011046841, Unigene13332\_Sample\_011046841, Unigene13881\_Sample\_011046841, Unigene21418\_Sample\_011046841, Unigene35368\_Sample\_011046841, Unigene43409\_Sample\_011046841, Unigene44425\_Sample\_011046841, Unigene45592\_Sample\_011046841, Unigene45858\_Sample\_011046841, Unigene46219\_Sample\_011046841, Unigene47655\_Sample\_011046841, Unigene49697\_Sample\_011046841, Unigene50170\_Sample\_011046841, Unigene50585\_Sample\_011046841, Unigene53096\_Sample\_011046841, Unigene54278\_Sample\_011046841, Unigene56681\_Sample\_011046841, Unigene56798\_Sample\_011046841, Unigene58209\_Sample\_011046841, Unigene58821\_Sample\_011046841, Unigene60136\_Sample\_011046841, Unigene60225\_Sample\_011046841, Unigene4137\_Sample\_011046841, Unigene5972\_Sample\_011046841, Unigene8180\_Sample\_011046841, Unigene19453\_Sample\_011046841, Unigene23218\_Sample\_011046841, Unigene31438\_Sample\_011046841, Unigene36373\_Sample\_011046841, Unigene40491\_Sample\_011046841, Unigene40723\_Sample\_011046841, Unigene47562\_Sample\_011046841, Unigene57775\_Sample\_011046841, Unigene59581\_Sample\_011046841, Unigene53642\_Sample\_011046841, Unigene58610\_Sample\_011046841, Unigene59688\_Sample\_011046841, Unigene59711\_Sample\_011046841, Unigene60863\_Sample\_011046841, Unigene45952\_Sample\_011046841, Unigene54521\_Sample\_011046841, Unigene55486\_Sample\_011046841, Unigene59421\_Sample\_011046841, Unigene1107\_Sample\_011046841, Unigene58229\_Sample\_011046841, Unigene60217\_Sample\_011046841, Unigene475\_Sample\_011046841, Unigene51301\_Sample\_011046841, Unigene2492\_Sample\_011046841, Unigene5263\_Sample\_011046841, Unigene11549\_Sample\_011046841, Unigene41996\_Sample\_011046841, Unigene46399\_Sample\_011046841, Unigene57641\_Sample\_011046841, Unigene54134\_Sample\_011046841, Unigene54823\_Sample\_011046841, Unigene21362\_Sample\_011046841, Unigene42580\_Sample\_011046841, Unigene53018\_Sample\_011046841, Unigene31066\_Sample\_011046841, Unigene51777\_Sample\_011046841, Unigene49698\_Sample\_011046841, Unigene31264\_Sample\_011046841, Unigene31544\_Sample\_011046841, Unigene43718\_Sample\_011046841, Unigene20770\_Sample\_011046841, Unigene50713\_Sample\_011046841, Unigene16442\_Sample\_011046841, Unigene30484\_Sample\_011046841, Unigene41464\_Sample\_011046841, Unigene43367\_Sample\_011046841, Unigene19937\_Sample\_011046841, Unigene48345\_Sample\_011046841, Unigene42358\_Sample\_011046841, Unigene58257\_Sample\_011046841, Unigene17523\_Sample\_011046841, Unigene16813\_Sample\_011046841, Unigene26184\_Sample\_011046841, Unigene19596\_Sample\_011046841, Unigene26777\_Sample\_011046841 |
| 10 | Long-term depression Back to summary table | Unigene3199\_Sample\_011046841, Unigene4416\_Sample\_011046841, Unigene5402\_Sample\_011046841, Unigene6178\_Sample\_011046841, Unigene8859\_Sample\_011046841, Unigene10970\_Sample\_011046841, Unigene11876\_Sample\_011046841, Unigene11999\_Sample\_011046841, Unigene12527\_Sample\_011046841, Unigene28326\_Sample\_011046841, Unigene39094\_Sample\_011046841, Unigene40230\_Sample\_011046841, Unigene43936\_Sample\_011046841, Unigene46058\_Sample\_011046841, Unigene48482\_Sample\_011046841, Unigene52106\_Sample\_011046841, Unigene53981\_Sample\_011046841, Unigene58460\_Sample\_011046841, Unigene59189\_Sample\_011046841, Unigene59705\_Sample\_011046841, Unigene60462\_Sample\_011046841, Unigene250\_Sample\_011046841, Unigene2095\_Sample\_011046841, Unigene2509\_Sample\_011046841, Unigene3363\_Sample\_011046841, Unigene3890\_Sample\_011046841, Unigene4993\_Sample\_011046841, Unigene5740\_Sample\_011046841, Unigene9867\_Sample\_011046841, Unigene10198\_Sample\_011046841, Unigene10346\_Sample\_011046841, Unigene11526\_Sample\_011046841, Unigene11676\_Sample\_011046841, Unigene12336\_Sample\_011046841, Unigene13782\_Sample\_011046841, Unigene14467\_Sample\_011046841, Unigene18318\_Sample\_011046841, Unigene21962\_Sample\_011046841, Unigene25098\_Sample\_011046841, Unigene29480\_Sample\_011046841, Unigene41574\_Sample\_011046841, Unigene42106\_Sample\_011046841, Unigene43448\_Sample\_011046841, Unigene47555\_Sample\_011046841, Unigene49494\_Sample\_011046841, Unigene52184\_Sample\_011046841, Unigene53096\_Sample\_011046841, Unigene53457\_Sample\_011046841, Unigene54900\_Sample\_011046841, Unigene55948\_Sample\_011046841, Unigene56010\_Sample\_011046841, Unigene56177\_Sample\_011046841, Unigene59109\_Sample\_011046841, Unigene60552\_Sample\_011046841, Unigene8813\_Sample\_011046841, Unigene11266\_Sample\_011046841, Unigene19002\_Sample\_011046841, Unigene28658\_Sample\_011046841, Unigene32259\_Sample\_011046841, Unigene40491\_Sample\_011046841, Unigene52294\_Sample\_011046841, Unigene52303\_Sample\_011046841, Unigene58321\_Sample\_011046841, Unigene60517\_Sample\_011046841, Unigene27828\_Sample\_011046841, Unigene32964\_Sample\_011046841, Unigene35757\_Sample\_011046841, Unigene47860\_Sample\_011046841, Unigene45708\_Sample\_011046841, Unigene58199\_Sample\_011046841, Unigene2942\_Sample\_011046841, Unigene9329\_Sample\_011046841, Unigene38302\_Sample\_011046841, Unigene47967\_Sample\_011046841, Unigene51736\_Sample\_011046841, Unigene57304\_Sample\_011046841, Unigene23818\_Sample\_011046841, Unigene11549\_Sample\_011046841, Unigene18170\_Sample\_011046841, Unigene29318\_Sample\_011046841, Unigene29462\_Sample\_011046841, Unigene32914\_Sample\_011046841, Unigene56139\_Sample\_011046841, Unigene60248\_Sample\_011046841, Unigene5898\_Sample\_011046841, Unigene16098\_Sample\_011046841, Unigene24650\_Sample\_011046841, Unigene42069\_Sample\_011046841, Unigene47947\_Sample\_011046841, Unigene32103\_Sample\_011046841, Unigene53352\_Sample\_011046841, Unigene53995\_Sample\_011046841, Unigene132\_Sample\_011046841, Unigene12521\_Sample\_011046841, Unigene42293\_Sample\_011046841, Unigene43099\_Sample\_011046841, Unigene43918\_Sample\_011046841, Unigene47872\_Sample\_011046841, Unigene47910\_Sample\_011046841, Unigene10863\_Sample\_011046841, Unigene19947\_Sample\_011046841, Unigene51582\_Sample\_011046841, Unigene51777\_Sample\_011046841, Unigene3842\_Sample\_011046841, Unigene47487\_Sample\_011046841, Unigene14776\_Sample\_011046841, Unigene46813\_Sample\_011046841, Unigene55720\_Sample\_011046841, Unigene29441\_Sample\_011046841, Unigene14181\_Sample\_011046841, Unigene30474\_Sample\_011046841 |
| 11 | Nucleotide excision repair Back to summary table | Unigene3946\_Sample\_011046841, Unigene9271\_Sample\_011046841, Unigene11321\_Sample\_011046841, Unigene13500\_Sample\_011046841, Unigene13697\_Sample\_011046841, Unigene22016\_Sample\_011046841, Unigene28864\_Sample\_011046841, Unigene34410\_Sample\_011046841, Unigene43894\_Sample\_011046841, Unigene44567\_Sample\_011046841, Unigene45181\_Sample\_011046841, Unigene48006\_Sample\_011046841, Unigene52393\_Sample\_011046841, Unigene54555\_Sample\_011046841, Unigene55735\_Sample\_011046841, Unigene56979\_Sample\_011046841, Unigene57972\_Sample\_011046841, Unigene58164\_Sample\_011046841, Unigene58760\_Sample\_011046841, Unigene59396\_Sample\_011046841, Unigene60618\_Sample\_011046841, Unigene60738\_Sample\_011046841, Unigene5030\_Sample\_011046841, Unigene5967\_Sample\_011046841, Unigene6034\_Sample\_011046841, Unigene8165\_Sample\_011046841, Unigene8428\_Sample\_011046841, Unigene8472\_Sample\_011046841, Unigene8571\_Sample\_011046841, Unigene10027\_Sample\_011046841, Unigene11117\_Sample\_011046841, Unigene11726\_Sample\_011046841, Unigene13597\_Sample\_011046841, Unigene13774\_Sample\_011046841, Unigene15074\_Sample\_011046841, Unigene19273\_Sample\_011046841, Unigene30642\_Sample\_011046841, Unigene34649\_Sample\_011046841, Unigene39877\_Sample\_011046841, Unigene45539\_Sample\_011046841, Unigene46283\_Sample\_011046841, Unigene47769\_Sample\_011046841, Unigene48609\_Sample\_011046841, Unigene49241\_Sample\_011046841, Unigene53787\_Sample\_011046841, Unigene54563\_Sample\_011046841, Unigene55543\_Sample\_011046841, Unigene56952\_Sample\_011046841, Unigene57569\_Sample\_011046841, Unigene59583\_Sample\_011046841, Unigene60604\_Sample\_011046841, Unigene60919\_Sample\_011046841, Unigene967\_Sample\_011046841, Unigene7633\_Sample\_011046841, Unigene19399\_Sample\_011046841, Unigene19409\_Sample\_011046841, Unigene20927\_Sample\_011046841, Unigene36469\_Sample\_011046841, Unigene40977\_Sample\_011046841, Unigene42576\_Sample\_011046841, Unigene42967\_Sample\_011046841, Unigene45095\_Sample\_011046841, Unigene47329\_Sample\_011046841, Unigene50396\_Sample\_011046841, Unigene57384\_Sample\_011046841, Unigene14358\_Sample\_011046841, Unigene34819\_Sample\_011046841, Unigene53046\_Sample\_011046841, Unigene56911\_Sample\_011046841, Unigene16199\_Sample\_011046841, Unigene6900\_Sample\_011046841, Unigene58947\_Sample\_011046841, Unigene4253\_Sample\_011046841, Unigene12822\_Sample\_011046841, Unigene14915\_Sample\_011046841, Unigene22760\_Sample\_011046841, Unigene50223\_Sample\_011046841, Unigene6972\_Sample\_011046841, Unigene18715\_Sample\_011046841, Unigene32707\_Sample\_011046841, Unigene45777\_Sample\_011046841, Unigene46198\_Sample\_011046841, Unigene59567\_Sample\_011046841, Unigene11099\_Sample\_011046841, Unigene13180\_Sample\_011046841, Unigene17752\_Sample\_011046841, Unigene29775\_Sample\_011046841, Unigene44734\_Sample\_011046841, Unigene46716\_Sample\_011046841, Unigene46826\_Sample\_011046841, Unigene50798\_Sample\_011046841, Unigene4847\_Sample\_011046841, Unigene26327\_Sample\_011046841, Unigene42749\_Sample\_011046841, Unigene43336\_Sample\_011046841, Unigene48882\_Sample\_011046841, Unigene51331\_Sample\_011046841, Unigene2398\_Sample\_011046841, Unigene12390\_Sample\_011046841, Unigene19290\_Sample\_011046841, Unigene26961\_Sample\_011046841, Unigene11233\_Sample\_011046841, Unigene21521\_Sample\_011046841, Unigene47025\_Sample\_011046841, Unigene7219\_Sample\_011046841, Unigene17439\_Sample\_011046841, Unigene42633\_Sample\_011046841, Unigene23742\_Sample\_011046841, Unigene31249\_Sample\_011046841, Unigene22255\_Sample\_011046841 |
| 12 | Huntington's disease Back to summary table | Unigene4434\_Sample\_011046841, Unigene4773\_Sample\_011046841, Unigene6709\_Sample\_011046841, Unigene6724\_Sample\_011046841, Unigene7288\_Sample\_011046841, Unigene8181\_Sample\_011046841, Unigene8859\_Sample\_011046841, Unigene9259\_Sample\_011046841, Unigene9668\_Sample\_011046841, Unigene9845\_Sample\_011046841, Unigene10567\_Sample\_011046841, Unigene10640\_Sample\_011046841, Unigene10700\_Sample\_011046841, Unigene11876\_Sample\_011046841, Unigene11923\_Sample\_011046841, Unigene12104\_Sample\_011046841, Unigene13666\_Sample\_011046841, Unigene13872\_Sample\_011046841, Unigene20324\_Sample\_011046841, Unigene24706\_Sample\_011046841, Unigene28662\_Sample\_011046841, Unigene34881\_Sample\_011046841, Unigene36816\_Sample\_011046841, Unigene38078\_Sample\_011046841, Unigene42615\_Sample\_011046841, Unigene44954\_Sample\_011046841, Unigene45049\_Sample\_011046841, Unigene45114\_Sample\_011046841, Unigene45493\_Sample\_011046841, Unigene46170\_Sample\_011046841, Unigene48058\_Sample\_011046841, Unigene48688\_Sample\_011046841, Unigene49604\_Sample\_011046841, Unigene49992\_Sample\_011046841, Unigene50538\_Sample\_011046841, Unigene50611\_Sample\_011046841, Unigene50631\_Sample\_011046841, Unigene50829\_Sample\_011046841, Unigene54209\_Sample\_011046841, Unigene54246\_Sample\_011046841, Unigene55037\_Sample\_011046841, Unigene55263\_Sample\_011046841, Unigene55452\_Sample\_011046841, Unigene55779\_Sample\_011046841, Unigene55892\_Sample\_011046841, Unigene56228\_Sample\_011046841, Unigene56276\_Sample\_011046841, Unigene56500\_Sample\_011046841, Unigene56818\_Sample\_011046841, Unigene56887\_Sample\_011046841, Unigene57770\_Sample\_011046841, Unigene59189\_Sample\_011046841, Unigene59194\_Sample\_011046841, Unigene59600\_Sample\_011046841, Unigene59633\_Sample\_011046841, Unigene59681\_Sample\_011046841, Unigene59909\_Sample\_011046841, Unigene60125\_Sample\_011046841, Unigene60211\_Sample\_011046841, Unigene60385\_Sample\_011046841, Unigene60396\_Sample\_011046841, Unigene60529\_Sample\_011046841, Unigene60785\_Sample\_011046841, Unigene60809\_Sample\_011046841, Unigene60926\_Sample\_011046841, Unigene60937\_Sample\_011046841, Unigene60958\_Sample\_011046841, Unigene60968\_Sample\_011046841, Unigene469\_Sample\_011046841, Unigene2366\_Sample\_011046841, Unigene2570\_Sample\_011046841, Unigene3223\_Sample\_011046841, Unigene3500\_Sample\_011046841, Unigene5733\_Sample\_011046841, Unigene6176\_Sample\_011046841, Unigene6209\_Sample\_011046841, Unigene6521\_Sample\_011046841, Unigene7821\_Sample\_011046841, Unigene8290\_Sample\_011046841, Unigene8641\_Sample\_011046841, Unigene8924\_Sample\_011046841, Unigene10236\_Sample\_011046841, Unigene11539\_Sample\_011046841, Unigene11858\_Sample\_011046841, Unigene12081\_Sample\_011046841, Unigene13571\_Sample\_011046841, Unigene13686\_Sample\_011046841, Unigene13763\_Sample\_011046841, Unigene13893\_Sample\_011046841, Unigene21784\_Sample\_011046841, Unigene21962\_Sample\_011046841, Unigene24751\_Sample\_011046841, Unigene24894\_Sample\_011046841, Unigene26737\_Sample\_011046841, Unigene29626\_Sample\_011046841, Unigene32116\_Sample\_011046841, Unigene33132\_Sample\_011046841, Unigene35814\_Sample\_011046841, Unigene35922\_Sample\_011046841, Unigene36036\_Sample\_011046841, Unigene39223\_Sample\_011046841, Unigene39295\_Sample\_011046841, Unigene39621\_Sample\_011046841, Unigene39698\_Sample\_011046841, Unigene40874\_Sample\_011046841, Unigene41959\_Sample\_011046841, Unigene42270\_Sample\_011046841, Unigene42399\_Sample\_011046841, Unigene45561\_Sample\_011046841, Unigene47569\_Sample\_011046841, Unigene47581\_Sample\_011046841, Unigene47636\_Sample\_011046841, Unigene48337\_Sample\_011046841, Unigene48737\_Sample\_011046841, Unigene49013\_Sample\_011046841, Unigene50487\_Sample\_011046841, Unigene50975\_Sample\_011046841, Unigene51201\_Sample\_011046841, Unigene51279\_Sample\_011046841, Unigene51737\_Sample\_011046841, Unigene51852\_Sample\_011046841, Unigene51857\_Sample\_011046841, Unigene52003\_Sample\_011046841, Unigene52322\_Sample\_011046841, Unigene53011\_Sample\_011046841, Unigene53262\_Sample\_011046841, Unigene53602\_Sample\_011046841, Unigene53773\_Sample\_011046841, Unigene54410\_Sample\_011046841, Unigene55076\_Sample\_011046841, Unigene55301\_Sample\_011046841, Unigene56177\_Sample\_011046841, Unigene57215\_Sample\_011046841, Unigene57259\_Sample\_011046841, Unigene57313\_Sample\_011046841, Unigene57353\_Sample\_011046841, Unigene57841\_Sample\_011046841, Unigene58152\_Sample\_011046841, Unigene58258\_Sample\_011046841, Unigene58450\_Sample\_011046841, Unigene58481\_Sample\_011046841, Unigene58593\_Sample\_011046841, Unigene59149\_Sample\_011046841, Unigene59224\_Sample\_011046841, Unigene59410\_Sample\_011046841, Unigene59509\_Sample\_011046841, Unigene59629\_Sample\_011046841, Unigene59900\_Sample\_011046841, Unigene59915\_Sample\_011046841, Unigene60019\_Sample\_011046841, Unigene60051\_Sample\_011046841, Unigene60123\_Sample\_011046841, Unigene60219\_Sample\_011046841, Unigene60342\_Sample\_011046841, Unigene60469\_Sample\_011046841, Unigene60571\_Sample\_011046841, Unigene60682\_Sample\_011046841, Unigene60900\_Sample\_011046841, Unigene60921\_Sample\_011046841, Unigene60949\_Sample\_011046841, Unigene60950\_Sample\_011046841, Unigene60964\_Sample\_011046841, Unigene1194\_Sample\_011046841, Unigene8813\_Sample\_011046841, Unigene10041\_Sample\_011046841, Unigene11266\_Sample\_011046841, Unigene12113\_Sample\_011046841, Unigene12142\_Sample\_011046841, Unigene12984\_Sample\_011046841, Unigene13847\_Sample\_011046841, Unigene16189\_Sample\_011046841, Unigene24615\_Sample\_011046841, Unigene27178\_Sample\_011046841, Unigene27188\_Sample\_011046841, Unigene29602\_Sample\_011046841, Unigene31723\_Sample\_011046841, Unigene32259\_Sample\_011046841, Unigene33958\_Sample\_011046841, Unigene35626\_Sample\_011046841, Unigene41399\_Sample\_011046841, Unigene47319\_Sample\_011046841, Unigene48876\_Sample\_011046841, Unigene49931\_Sample\_011046841, Unigene50303\_Sample\_011046841, Unigene51837\_Sample\_011046841, Unigene52079\_Sample\_011046841, Unigene52812\_Sample\_011046841, Unigene53128\_Sample\_011046841, Unigene53605\_Sample\_011046841, Unigene54217\_Sample\_011046841, Unigene55212\_Sample\_011046841, Unigene55342\_Sample\_011046841, Unigene55575\_Sample\_011046841, Unigene55692\_Sample\_011046841, Unigene55900\_Sample\_011046841, Unigene56822\_Sample\_011046841, Unigene57843\_Sample\_011046841, Unigene57950\_Sample\_011046841, Unigene58557\_Sample\_011046841, Unigene58892\_Sample\_011046841, Unigene59540\_Sample\_011046841, Unigene59709\_Sample\_011046841, Unigene60847\_Sample\_011046841, Unigene60963\_Sample\_011046841, Unigene5863\_Sample\_011046841, Unigene6164\_Sample\_011046841, Unigene10669\_Sample\_011046841, Unigene13099\_Sample\_011046841, Unigene13122\_Sample\_011046841, Unigene33988\_Sample\_011046841, Unigene35737\_Sample\_011046841, Unigene35806\_Sample\_011046841, Unigene36363\_Sample\_011046841, Unigene36823\_Sample\_011046841, Unigene43713\_Sample\_011046841, Unigene44142\_Sample\_011046841, Unigene45246\_Sample\_011046841, Unigene46754\_Sample\_011046841, Unigene47036\_Sample\_011046841, Unigene47860\_Sample\_011046841, Unigene48158\_Sample\_011046841, Unigene55470\_Sample\_011046841, Unigene56081\_Sample\_011046841, Unigene58099\_Sample\_011046841, Unigene59492\_Sample\_011046841, Unigene59824\_Sample\_011046841, Unigene60966\_Sample\_011046841, Unigene3905\_Sample\_011046841, Unigene13011\_Sample\_011046841, Unigene45389\_Sample\_011046841, Unigene45708\_Sample\_011046841, Unigene54528\_Sample\_011046841, Unigene1441\_Sample\_011046841, Unigene6167\_Sample\_011046841, Unigene10464\_Sample\_011046841, Unigene22226\_Sample\_011046841, Unigene30591\_Sample\_011046841, Unigene30898\_Sample\_011046841, Unigene36849\_Sample\_011046841, Unigene37098\_Sample\_011046841, Unigene38302\_Sample\_011046841, Unigene39240\_Sample\_011046841, Unigene42977\_Sample\_011046841, Unigene44108\_Sample\_011046841, Unigene45107\_Sample\_011046841, Unigene49168\_Sample\_011046841, Unigene49709\_Sample\_011046841, Unigene50145\_Sample\_011046841, Unigene50644\_Sample\_011046841, Unigene312\_Sample\_011046841, Unigene9211\_Sample\_011046841, Unigene29861\_Sample\_011046841, Unigene52621\_Sample\_011046841, Unigene53241\_Sample\_011046841, Unigene55630\_Sample\_011046841, Unigene4293\_Sample\_011046841, Unigene12271\_Sample\_011046841, Unigene18385\_Sample\_011046841, Unigene19014\_Sample\_011046841, Unigene28830\_Sample\_011046841, Unigene30035\_Sample\_011046841, Unigene43793\_Sample\_011046841, Unigene43804\_Sample\_011046841, Unigene47956\_Sample\_011046841, Unigene57117\_Sample\_011046841, Unigene58462\_Sample\_011046841, Unigene5446\_Sample\_011046841, Unigene5898\_Sample\_011046841, Unigene22614\_Sample\_011046841, Unigene37267\_Sample\_011046841, Unigene42069\_Sample\_011046841, Unigene48164\_Sample\_011046841, Unigene59441\_Sample\_011046841, Unigene59473\_Sample\_011046841, Unigene6573\_Sample\_011046841, Unigene15774\_Sample\_011046841, Unigene23333\_Sample\_011046841, Unigene32476\_Sample\_011046841, Unigene32987\_Sample\_011046841, Unigene35030\_Sample\_011046841, Unigene40307\_Sample\_011046841, Unigene41153\_Sample\_011046841, Unigene45059\_Sample\_011046841, Unigene53343\_Sample\_011046841, Unigene53853\_Sample\_011046841, Unigene54134\_Sample\_011046841, Unigene54273\_Sample\_011046841, Unigene6279\_Sample\_011046841, Unigene8720\_Sample\_011046841, Unigene11865\_Sample\_011046841, Unigene14125\_Sample\_011046841, Unigene16014\_Sample\_011046841, Unigene17449\_Sample\_011046841, Unigene18975\_Sample\_011046841, Unigene27558\_Sample\_011046841, Unigene35762\_Sample\_011046841, Unigene36984\_Sample\_011046841, Unigene43813\_Sample\_011046841, Unigene44774\_Sample\_011046841, Unigene44861\_Sample\_011046841, Unigene46287\_Sample\_011046841, Unigene48497\_Sample\_011046841, Unigene52776\_Sample\_011046841, Unigene55420\_Sample\_011046841, Unigene59621\_Sample\_011046841, Unigene59748\_Sample\_011046841, Unigene16327\_Sample\_011046841, Unigene19947\_Sample\_011046841, Unigene38618\_Sample\_011046841, Unigene40390\_Sample\_011046841, Unigene44453\_Sample\_011046841, Unigene51997\_Sample\_011046841, Unigene54829\_Sample\_011046841, Unigene58803\_Sample\_011046841, Unigene14396\_Sample\_011046841, Unigene28941\_Sample\_011046841, Unigene18756\_Sample\_011046841, Unigene15838\_Sample\_011046841, Unigene18813\_Sample\_011046841, Unigene28384\_Sample\_011046841, Unigene23568\_Sample\_011046841, Unigene41725\_Sample\_011046841, Unigene42218\_Sample\_011046841, Unigene52428\_Sample\_011046841, Unigene30989\_Sample\_011046841, Unigene31713\_Sample\_011046841, Unigene17650\_Sample\_011046841, Unigene47097\_Sample\_011046841, Unigene14181\_Sample\_011046841, Unigene21035\_Sample\_011046841, Unigene24428\_Sample\_011046841, Unigene31679\_Sample\_011046841, Unigene39125\_Sample\_011046841, Unigene40532\_Sample\_011046841, Unigene47389\_Sample\_011046841, Unigene26621\_Sample\_011046841, Unigene32802\_Sample\_011046841, Unigene24848\_Sample\_011046841, Unigene20433\_Sample\_011046841, Unigene48499\_Sample\_011046841 |
| 13 | Renal cell carcinoma Back to summary table | Unigene865\_Sample\_011046841, Unigene4209\_Sample\_011046841, Unigene4889\_Sample\_011046841, Unigene5053\_Sample\_011046841, Unigene7143\_Sample\_011046841, Unigene8035\_Sample\_011046841, Unigene9668\_Sample\_011046841, Unigene10459\_Sample\_011046841, Unigene10640\_Sample\_011046841, Unigene10970\_Sample\_011046841, Unigene13069\_Sample\_011046841, Unigene13872\_Sample\_011046841, Unigene13901\_Sample\_011046841, Unigene39219\_Sample\_011046841, Unigene41694\_Sample\_011046841, Unigene45114\_Sample\_011046841, Unigene45346\_Sample\_011046841, Unigene52094\_Sample\_011046841, Unigene53424\_Sample\_011046841, Unigene58460\_Sample\_011046841, Unigene58987\_Sample\_011046841, Unigene59559\_Sample\_011046841, Unigene60173\_Sample\_011046841, Unigene60498\_Sample\_011046841, Unigene8641\_Sample\_011046841, Unigene13332\_Sample\_011046841, Unigene13855\_Sample\_011046841, Unigene13877\_Sample\_011046841, Unigene19273\_Sample\_011046841, Unigene21418\_Sample\_011046841, Unigene32409\_Sample\_011046841, Unigene35368\_Sample\_011046841, Unigene41574\_Sample\_011046841, Unigene43409\_Sample\_011046841, Unigene45592\_Sample\_011046841, Unigene45858\_Sample\_011046841, Unigene46219\_Sample\_011046841, Unigene47555\_Sample\_011046841, Unigene54900\_Sample\_011046841, Unigene54986\_Sample\_011046841, Unigene56348\_Sample\_011046841, Unigene56681\_Sample\_011046841, Unigene57566\_Sample\_011046841, Unigene58100\_Sample\_011046841, Unigene23218\_Sample\_011046841, Unigene23590\_Sample\_011046841, Unigene28658\_Sample\_011046841, Unigene31438\_Sample\_011046841, Unigene40723\_Sample\_011046841, Unigene40758\_Sample\_011046841, Unigene60765\_Sample\_011046841, Unigene32964\_Sample\_011046841, Unigene44532\_Sample\_011046841, Unigene45689\_Sample\_011046841, Unigene47549\_Sample\_011046841, Unigene53642\_Sample\_011046841, Unigene54375\_Sample\_011046841, Unigene56300\_Sample\_011046841, Unigene59711\_Sample\_011046841, Unigene60435\_Sample\_011046841, Unigene49525\_Sample\_011046841, Unigene54521\_Sample\_011046841, Unigene55486\_Sample\_011046841, Unigene26964\_Sample\_011046841, Unigene43878\_Sample\_011046841, Unigene47967\_Sample\_011046841, Unigene60152\_Sample\_011046841, Unigene51301\_Sample\_011046841, Unigene5263\_Sample\_011046841, Unigene29318\_Sample\_011046841, Unigene41996\_Sample\_011046841, Unigene47054\_Sample\_011046841, Unigene51756\_Sample\_011046841, Unigene56390\_Sample\_011046841, Unigene57117\_Sample\_011046841, Unigene53995\_Sample\_011046841, Unigene57169\_Sample\_011046841, Unigene36841\_Sample\_011046841, Unigene42580\_Sample\_011046841, Unigene47910\_Sample\_011046841, Unigene50832\_Sample\_011046841, Unigene59748\_Sample\_011046841, Unigene26961\_Sample\_011046841, Unigene44929\_Sample\_011046841, Unigene49698\_Sample\_011046841, Unigene28384\_Sample\_011046841, Unigene11577\_Sample\_011046841, Unigene17671\_Sample\_011046841, Unigene20350\_Sample\_011046841, Unigene20770\_Sample\_011046841, Unigene29944\_Sample\_011046841, Unigene50713\_Sample\_011046841, Unigene41464\_Sample\_011046841, Unigene19937\_Sample\_011046841, Unigene32258\_Sample\_011046841, Unigene42358\_Sample\_011046841, Unigene46813\_Sample\_011046841, Unigene17523\_Sample\_011046841, Unigene6551\_Sample\_011046841, Unigene16813\_Sample\_011046841, Unigene17471\_Sample\_011046841, Unigene26184\_Sample\_011046841, Unigene29437\_Sample\_011046841, Unigene30474\_Sample\_011046841, Unigene19596\_Sample\_011046841 |
| 14 | Pathways in cancer Back to summary table | Unigene865\_Sample\_011046841, Unigene1704\_Sample\_011046841, Unigene3199\_Sample\_011046841, Unigene3904\_Sample\_011046841, Unigene4209\_Sample\_011046841, Unigene4889\_Sample\_011046841, Unigene4963\_Sample\_011046841, Unigene5012\_Sample\_011046841, Unigene5122\_Sample\_011046841, Unigene5178\_Sample\_011046841, Unigene6324\_Sample\_011046841, Unigene6410\_Sample\_011046841, Unigene6605\_Sample\_011046841, Unigene6971\_Sample\_011046841, Unigene7143\_Sample\_011046841, Unigene7809\_Sample\_011046841, Unigene8429\_Sample\_011046841, Unigene8581\_Sample\_011046841, Unigene8881\_Sample\_011046841, Unigene9082\_Sample\_011046841, Unigene9668\_Sample\_011046841, Unigene9858\_Sample\_011046841, Unigene10459\_Sample\_011046841, Unigene10581\_Sample\_011046841, Unigene10640\_Sample\_011046841, Unigene10744\_Sample\_011046841, Unigene10970\_Sample\_011046841, Unigene11073\_Sample\_011046841, Unigene11124\_Sample\_011046841, Unigene11367\_Sample\_011046841, Unigene11562\_Sample\_011046841, Unigene11706\_Sample\_011046841, Unigene12213\_Sample\_011046841, Unigene12652\_Sample\_011046841, Unigene12835\_Sample\_011046841, Unigene13069\_Sample\_011046841, Unigene13346\_Sample\_011046841, Unigene13683\_Sample\_011046841, Unigene13872\_Sample\_011046841, Unigene17786\_Sample\_011046841, Unigene23925\_Sample\_011046841, Unigene24155\_Sample\_011046841, Unigene26714\_Sample\_011046841, Unigene30600\_Sample\_011046841, Unigene35406\_Sample\_011046841, Unigene35926\_Sample\_011046841, Unigene36772\_Sample\_011046841, Unigene37440\_Sample\_011046841, Unigene40230\_Sample\_011046841, Unigene40479\_Sample\_011046841, Unigene42704\_Sample\_011046841, Unigene45114\_Sample\_011046841, Unigene47292\_Sample\_011046841, Unigene48058\_Sample\_011046841, Unigene54213\_Sample\_011046841, Unigene54246\_Sample\_011046841, Unigene56121\_Sample\_011046841, Unigene56818\_Sample\_011046841, Unigene56886\_Sample\_011046841, Unigene57298\_Sample\_011046841, Unigene57934\_Sample\_011046841, Unigene58189\_Sample\_011046841, Unigene58307\_Sample\_011046841, Unigene58460\_Sample\_011046841, Unigene58506\_Sample\_011046841, Unigene58725\_Sample\_011046841, Unigene58987\_Sample\_011046841, Unigene59022\_Sample\_011046841, Unigene59151\_Sample\_011046841, Unigene59633\_Sample\_011046841, Unigene59705\_Sample\_011046841, Unigene59978\_Sample\_011046841, Unigene60173\_Sample\_011046841, Unigene60498\_Sample\_011046841, Unigene60579\_Sample\_011046841, Unigene60664\_Sample\_011046841, Unigene60685\_Sample\_011046841, Unigene60776\_Sample\_011046841, Unigene60826\_Sample\_011046841, Unigene1378\_Sample\_011046841, Unigene1414\_Sample\_011046841, Unigene1934\_Sample\_011046841, Unigene2591\_Sample\_011046841, Unigene2933\_Sample\_011046841, Unigene3363\_Sample\_011046841, Unigene3632\_Sample\_011046841, Unigene4858\_Sample\_011046841, Unigene4892\_Sample\_011046841, Unigene5237\_Sample\_011046841, Unigene5747\_Sample\_011046841, Unigene6101\_Sample\_011046841, Unigene6706\_Sample\_011046841, Unigene7206\_Sample\_011046841, Unigene8003\_Sample\_011046841, Unigene8299\_Sample\_011046841, Unigene8588\_Sample\_011046841, Unigene8641\_Sample\_011046841, Unigene9008\_Sample\_011046841, Unigene9408\_Sample\_011046841, Unigene9867\_Sample\_011046841, Unigene11134\_Sample\_011046841, Unigene11676\_Sample\_011046841, Unigene11715\_Sample\_011046841, Unigene11922\_Sample\_011046841, Unigene12413\_Sample\_011046841, Unigene12943\_Sample\_011046841, Unigene13332\_Sample\_011046841, Unigene13452\_Sample\_011046841, Unigene13575\_Sample\_011046841, Unigene13656\_Sample\_011046841, Unigene13667\_Sample\_011046841, Unigene13766\_Sample\_011046841, Unigene13855\_Sample\_011046841, Unigene13873\_Sample\_011046841, Unigene13930\_Sample\_011046841, Unigene19273\_Sample\_011046841, Unigene21418\_Sample\_011046841, Unigene33667\_Sample\_011046841, Unigene37278\_Sample\_011046841, Unigene40209\_Sample\_011046841, Unigene40989\_Sample\_011046841, Unigene41574\_Sample\_011046841, Unigene42561\_Sample\_011046841, Unigene42676\_Sample\_011046841, Unigene42820\_Sample\_011046841, Unigene43409\_Sample\_011046841, Unigene45592\_Sample\_011046841, Unigene46219\_Sample\_011046841, Unigene47555\_Sample\_011046841, Unigene49399\_Sample\_011046841, Unigene49494\_Sample\_011046841, Unigene50674\_Sample\_011046841, Unigene50677\_Sample\_011046841, Unigene50963\_Sample\_011046841, Unigene51148\_Sample\_011046841, Unigene53186\_Sample\_011046841, Unigene53983\_Sample\_011046841, Unigene54329\_Sample\_011046841, Unigene54525\_Sample\_011046841, Unigene54859\_Sample\_011046841, Unigene54900\_Sample\_011046841, Unigene54932\_Sample\_011046841, Unigene54986\_Sample\_011046841, Unigene55290\_Sample\_011046841, Unigene55465\_Sample\_011046841, Unigene55825\_Sample\_011046841, Unigene56004\_Sample\_011046841, Unigene56197\_Sample\_011046841, Unigene56348\_Sample\_011046841, Unigene56681\_Sample\_011046841, Unigene56798\_Sample\_011046841, Unigene57215\_Sample\_011046841, Unigene57430\_Sample\_011046841, Unigene57566\_Sample\_011046841, Unigene57614\_Sample\_011046841, Unigene57776\_Sample\_011046841, Unigene57862\_Sample\_011046841, Unigene58100\_Sample\_011046841, Unigene58149\_Sample\_011046841, Unigene58209\_Sample\_011046841, Unigene58466\_Sample\_011046841, Unigene58564\_Sample\_011046841, Unigene58833\_Sample\_011046841, Unigene59109\_Sample\_011046841, Unigene59265\_Sample\_011046841, Unigene59660\_Sample\_011046841, Unigene59671\_Sample\_011046841, Unigene59704\_Sample\_011046841, Unigene60041\_Sample\_011046841, Unigene60190\_Sample\_011046841, Unigene60299\_Sample\_011046841, Unigene60367\_Sample\_011046841, Unigene60427\_Sample\_011046841, Unigene60552\_Sample\_011046841, Unigene60624\_Sample\_011046841, Unigene60859\_Sample\_011046841, Unigene60878\_Sample\_011046841, Unigene60944\_Sample\_011046841, Unigene1526\_Sample\_011046841, Unigene2725\_Sample\_011046841, Unigene3582\_Sample\_011046841, Unigene9726\_Sample\_011046841, Unigene9831\_Sample\_011046841, Unigene10789\_Sample\_011046841, Unigene11932\_Sample\_011046841, Unigene13958\_Sample\_011046841, Unigene23590\_Sample\_011046841, Unigene28658\_Sample\_011046841, Unigene31181\_Sample\_011046841, Unigene37205\_Sample\_011046841, Unigene40758\_Sample\_011046841, Unigene45039\_Sample\_011046841, Unigene46930\_Sample\_011046841, Unigene51171\_Sample\_011046841, Unigene52294\_Sample\_011046841, Unigene53989\_Sample\_011046841, Unigene54574\_Sample\_011046841, Unigene54909\_Sample\_011046841, Unigene55164\_Sample\_011046841, Unigene56062\_Sample\_011046841, Unigene56160\_Sample\_011046841, Unigene57272\_Sample\_011046841, Unigene57649\_Sample\_011046841, Unigene57775\_Sample\_011046841, Unigene58321\_Sample\_011046841, Unigene58479\_Sample\_011046841, Unigene59585\_Sample\_011046841, Unigene60765\_Sample\_011046841, Unigene2805\_Sample\_011046841, Unigene19951\_Sample\_011046841, Unigene30184\_Sample\_011046841, Unigene32964\_Sample\_011046841, Unigene39807\_Sample\_011046841, Unigene44532\_Sample\_011046841, Unigene45689\_Sample\_011046841, Unigene50207\_Sample\_011046841, Unigene51950\_Sample\_011046841, Unigene53803\_Sample\_011046841, Unigene54375\_Sample\_011046841, Unigene56300\_Sample\_011046841, Unigene60102\_Sample\_011046841, Unigene60435\_Sample\_011046841, Unigene60863\_Sample\_011046841, Unigene1928\_Sample\_011046841, Unigene9480\_Sample\_011046841, Unigene13428\_Sample\_011046841, Unigene29346\_Sample\_011046841, Unigene44445\_Sample\_011046841, Unigene49525\_Sample\_011046841, Unigene59482\_Sample\_011046841, Unigene1107\_Sample\_011046841, Unigene2942\_Sample\_011046841, Unigene9329\_Sample\_011046841, Unigene26964\_Sample\_011046841, Unigene32596\_Sample\_011046841, Unigene34657\_Sample\_011046841, Unigene37429\_Sample\_011046841, Unigene38674\_Sample\_011046841, Unigene41032\_Sample\_011046841, Unigene47967\_Sample\_011046841, Unigene49162\_Sample\_011046841, Unigene52354\_Sample\_011046841, Unigene52611\_Sample\_011046841, Unigene54712\_Sample\_011046841, Unigene54809\_Sample\_011046841, Unigene60217\_Sample\_011046841, Unigene17370\_Sample\_011046841, Unigene18170\_Sample\_011046841, Unigene29318\_Sample\_011046841, Unigene30153\_Sample\_011046841, Unigene47054\_Sample\_011046841, Unigene51756\_Sample\_011046841, Unigene52590\_Sample\_011046841, Unigene55344\_Sample\_011046841, Unigene56139\_Sample\_011046841, Unigene57006\_Sample\_011046841, Unigene57117\_Sample\_011046841, Unigene57963\_Sample\_011046841, Unigene59656\_Sample\_011046841, Unigene60248\_Sample\_011046841, Unigene16098\_Sample\_011046841, Unigene56426\_Sample\_011046841, Unigene804\_Sample\_011046841, Unigene27016\_Sample\_011046841, Unigene28148\_Sample\_011046841, Unigene32121\_Sample\_011046841, Unigene36385\_Sample\_011046841, Unigene39189\_Sample\_011046841, Unigene53367\_Sample\_011046841, Unigene53995\_Sample\_011046841, Unigene54134\_Sample\_011046841, Unigene7879\_Sample\_011046841, Unigene12521\_Sample\_011046841, Unigene13126\_Sample\_011046841, Unigene18975\_Sample\_011046841, Unigene29733\_Sample\_011046841, Unigene31645\_Sample\_011046841, Unigene42580\_Sample\_011046841, Unigene43099\_Sample\_011046841, Unigene47686\_Sample\_011046841, Unigene47872\_Sample\_011046841, Unigene47910\_Sample\_011046841, Unigene50832\_Sample\_011046841, Unigene59748\_Sample\_011046841, Unigene12700\_Sample\_011046841, Unigene26961\_Sample\_011046841, Unigene31969\_Sample\_011046841, Unigene44929\_Sample\_011046841, Unigene51261\_Sample\_011046841, Unigene51582\_Sample\_011046841, Unigene53893\_Sample\_011046841, Unigene14639\_Sample\_011046841, Unigene55528\_Sample\_011046841, Unigene46176\_Sample\_011046841, Unigene23857\_Sample\_011046841, Unigene28384\_Sample\_011046841, Unigene43718\_Sample\_011046841, Unigene48052\_Sample\_011046841, Unigene48928\_Sample\_011046841, Unigene53768\_Sample\_011046841, Unigene20350\_Sample\_011046841, Unigene29944\_Sample\_011046841, Unigene35987\_Sample\_011046841, Unigene8243\_Sample\_011046841, Unigene32237\_Sample\_011046841, Unigene41007\_Sample\_011046841, Unigene19937\_Sample\_011046841, Unigene32258\_Sample\_011046841, Unigene43091\_Sample\_011046841, Unigene46813\_Sample\_011046841, Unigene6551\_Sample\_011046841, Unigene17471\_Sample\_011046841, Unigene29437\_Sample\_011046841, Unigene39361\_Sample\_011046841, Unigene50416\_Sample\_011046841, Unigene30474\_Sample\_011046841, Unigene20433\_Sample\_011046841, Unigene19596\_Sample\_011046841, Unigene26777\_Sample\_011046841 |
| 15 | Cell cycle - yeast Back to summary table | Unigene517\_Sample\_011046841, Unigene1437\_Sample\_011046841, Unigene5402\_Sample\_011046841, Unigene5622\_Sample\_011046841, Unigene7281\_Sample\_011046841, Unigene7905\_Sample\_011046841, Unigene8797\_Sample\_011046841, Unigene9830\_Sample\_011046841, Unigene9876\_Sample\_011046841, Unigene10179\_Sample\_011046841, Unigene10855\_Sample\_011046841, Unigene11573\_Sample\_011046841, Unigene11641\_Sample\_011046841, Unigene11708\_Sample\_011046841, Unigene11769\_Sample\_011046841, Unigene11871\_Sample\_011046841, Unigene12097\_Sample\_011046841, Unigene12306\_Sample\_011046841, Unigene12490\_Sample\_011046841, Unigene12510\_Sample\_011046841, Unigene12670\_Sample\_011046841, Unigene13808\_Sample\_011046841, Unigene13939\_Sample\_011046841, Unigene19684\_Sample\_011046841, Unigene27324\_Sample\_011046841, Unigene27948\_Sample\_011046841, Unigene28326\_Sample\_011046841, Unigene32059\_Sample\_011046841, Unigene36772\_Sample\_011046841, Unigene40567\_Sample\_011046841, Unigene45790\_Sample\_011046841, Unigene47118\_Sample\_011046841, Unigene49649\_Sample\_011046841, Unigene51617\_Sample\_011046841, Unigene52432\_Sample\_011046841, Unigene53021\_Sample\_011046841, Unigene53691\_Sample\_011046841, Unigene55432\_Sample\_011046841, Unigene55744\_Sample\_011046841, Unigene55963\_Sample\_011046841, Unigene56258\_Sample\_011046841, Unigene56719\_Sample\_011046841, Unigene57076\_Sample\_011046841, Unigene57083\_Sample\_011046841, Unigene58291\_Sample\_011046841, Unigene59062\_Sample\_011046841, Unigene59460\_Sample\_011046841, Unigene59512\_Sample\_011046841, Unigene59588\_Sample\_011046841, Unigene59730\_Sample\_011046841, Unigene60372\_Sample\_011046841, Unigene60712\_Sample\_011046841, Unigene60755\_Sample\_011046841, Unigene60941\_Sample\_011046841, Unigene1417\_Sample\_011046841, Unigene3353\_Sample\_011046841, Unigene4131\_Sample\_011046841, Unigene5688\_Sample\_011046841, Unigene7766\_Sample\_011046841, Unigene8569\_Sample\_011046841, Unigene8931\_Sample\_011046841, Unigene10325\_Sample\_011046841, Unigene11148\_Sample\_011046841, Unigene12138\_Sample\_011046841, Unigene12170\_Sample\_011046841, Unigene12378\_Sample\_011046841, Unigene12971\_Sample\_011046841, Unigene13825\_Sample\_011046841, Unigene19273\_Sample\_011046841, Unigene24438\_Sample\_011046841, Unigene25098\_Sample\_011046841, Unigene26055\_Sample\_011046841, Unigene27694\_Sample\_011046841, Unigene30076\_Sample\_011046841, Unigene30274\_Sample\_011046841, Unigene30495\_Sample\_011046841, Unigene37264\_Sample\_011046841, Unigene40648\_Sample\_011046841, Unigene42106\_Sample\_011046841, Unigene43377\_Sample\_011046841, Unigene43448\_Sample\_011046841, Unigene47368\_Sample\_011046841, Unigene47510\_Sample\_011046841, Unigene48020\_Sample\_011046841, Unigene49664\_Sample\_011046841, Unigene51017\_Sample\_011046841, Unigene52184\_Sample\_011046841, Unigene52869\_Sample\_011046841, Unigene54454\_Sample\_011046841, Unigene54711\_Sample\_011046841, Unigene56010\_Sample\_011046841, Unigene56400\_Sample\_011046841, Unigene56661\_Sample\_011046841, Unigene56883\_Sample\_011046841, Unigene57082\_Sample\_011046841, Unigene57390\_Sample\_011046841, Unigene57835\_Sample\_011046841, Unigene57929\_Sample\_011046841, Unigene58169\_Sample\_011046841, Unigene58273\_Sample\_011046841, Unigene58496\_Sample\_011046841, Unigene60647\_Sample\_011046841, Unigene60681\_Sample\_011046841, Unigene60846\_Sample\_011046841, Unigene2066\_Sample\_011046841, Unigene2213\_Sample\_011046841, Unigene6719\_Sample\_011046841, Unigene8828\_Sample\_011046841, Unigene12725\_Sample\_011046841, Unigene13203\_Sample\_011046841, Unigene13430\_Sample\_011046841, Unigene20297\_Sample\_011046841, Unigene20484\_Sample\_011046841, Unigene25812\_Sample\_011046841, Unigene27440\_Sample\_011046841, Unigene30120\_Sample\_011046841, Unigene32413\_Sample\_011046841, Unigene42721\_Sample\_011046841, Unigene45776\_Sample\_011046841, Unigene46000\_Sample\_011046841, Unigene48072\_Sample\_011046841, Unigene48647\_Sample\_011046841, Unigene51803\_Sample\_011046841, Unigene55183\_Sample\_011046841, Unigene55838\_Sample\_011046841, Unigene57678\_Sample\_011046841, Unigene57890\_Sample\_011046841, Unigene58289\_Sample\_011046841, Unigene58962\_Sample\_011046841, Unigene60347\_Sample\_011046841, Unigene13490\_Sample\_011046841, Unigene27828\_Sample\_011046841, Unigene35757\_Sample\_011046841, Unigene35917\_Sample\_011046841, Unigene37549\_Sample\_011046841, Unigene50901\_Sample\_011046841, Unigene53041\_Sample\_011046841, Unigene55769\_Sample\_011046841, Unigene56373\_Sample\_011046841, Unigene58502\_Sample\_011046841, Unigene59084\_Sample\_011046841, Unigene11000\_Sample\_011046841, Unigene12412\_Sample\_011046841, Unigene18699\_Sample\_011046841, Unigene21917\_Sample\_011046841, Unigene28681\_Sample\_011046841, Unigene55121\_Sample\_011046841, Unigene55251\_Sample\_011046841, Unigene7092\_Sample\_011046841, Unigene12023\_Sample\_011046841, Unigene41980\_Sample\_011046841, Unigene49441\_Sample\_011046841, Unigene57239\_Sample\_011046841, Unigene57304\_Sample\_011046841, Unigene59178\_Sample\_011046841, Unigene60375\_Sample\_011046841, Unigene1147\_Sample\_011046841, Unigene42292\_Sample\_011046841, Unigene55840\_Sample\_011046841, Unigene20298\_Sample\_011046841, Unigene29365\_Sample\_011046841, Unigene41514\_Sample\_011046841, Unigene47753\_Sample\_011046841, Unigene59290\_Sample\_011046841, Unigene60948\_Sample\_011046841, Unigene47947\_Sample\_011046841, Unigene57808\_Sample\_011046841, Unigene20381\_Sample\_011046841, Unigene24065\_Sample\_011046841, Unigene32103\_Sample\_011046841, Unigene38689\_Sample\_011046841, Unigene41249\_Sample\_011046841, Unigene48731\_Sample\_011046841, Unigene48939\_Sample\_011046841, Unigene11415\_Sample\_011046841, Unigene19526\_Sample\_011046841, Unigene28348\_Sample\_011046841, Unigene33317\_Sample\_011046841, Unigene33437\_Sample\_011046841, Unigene37595\_Sample\_011046841, Unigene39991\_Sample\_011046841, Unigene43918\_Sample\_011046841, Unigene50550\_Sample\_011046841, Unigene52384\_Sample\_011046841, Unigene54171\_Sample\_011046841, Unigene59578\_Sample\_011046841, Unigene11144\_Sample\_011046841, Unigene25028\_Sample\_011046841, Unigene26961\_Sample\_011046841, Unigene55544\_Sample\_011046841, Unigene60003\_Sample\_011046841, Unigene17192\_Sample\_011046841, Unigene46176\_Sample\_011046841, Unigene28643\_Sample\_011046841, Unigene40117\_Sample\_011046841, Unigene32237\_Sample\_011046841, Unigene47099\_Sample\_011046841, Unigene46652\_Sample\_011046841, Unigene54373\_Sample\_011046841, Unigene40474\_Sample\_011046841, Unigene15593\_Sample\_011046841, Unigene48502\_Sample\_011046841 |
| 16 | Adherens junction Back to summary table | Unigene290\_Sample\_011046841, Unigene863\_Sample\_011046841, Unigene1210\_Sample\_011046841, Unigene1273\_Sample\_011046841, Unigene3671\_Sample\_011046841, Unigene4208\_Sample\_011046841, Unigene4963\_Sample\_011046841, Unigene5122\_Sample\_011046841, Unigene7142\_Sample\_011046841, Unigene7809\_Sample\_011046841, Unigene8296\_Sample\_011046841, Unigene9237\_Sample\_011046841, Unigene9668\_Sample\_011046841, Unigene9774\_Sample\_011046841, Unigene9858\_Sample\_011046841, Unigene10518\_Sample\_011046841, Unigene10615\_Sample\_011046841, Unigene10640\_Sample\_011046841, Unigene11367\_Sample\_011046841, Unigene11938\_Sample\_011046841, Unigene12057\_Sample\_011046841, Unigene13370\_Sample\_011046841, Unigene13872\_Sample\_011046841, Unigene22077\_Sample\_011046841, Unigene27010\_Sample\_011046841, Unigene37440\_Sample\_011046841, Unigene45114\_Sample\_011046841, Unigene48622\_Sample\_011046841, Unigene48836\_Sample\_011046841, Unigene51559\_Sample\_011046841, Unigene52144\_Sample\_011046841, Unigene53157\_Sample\_011046841, Unigene53568\_Sample\_011046841, Unigene56886\_Sample\_011046841, Unigene57114\_Sample\_011046841, Unigene57298\_Sample\_011046841, Unigene57887\_Sample\_011046841, Unigene58189\_Sample\_011046841, Unigene58307\_Sample\_011046841, Unigene58906\_Sample\_011046841, Unigene59151\_Sample\_011046841, Unigene59705\_Sample\_011046841, Unigene60267\_Sample\_011046841, Unigene60460\_Sample\_011046841, Unigene60897\_Sample\_011046841, Unigene1414\_Sample\_011046841, Unigene2588\_Sample\_011046841, Unigene3761\_Sample\_011046841, Unigene4916\_Sample\_011046841, Unigene5568\_Sample\_011046841, Unigene5747\_Sample\_011046841, Unigene6260\_Sample\_011046841, Unigene6944\_Sample\_011046841, Unigene8641\_Sample\_011046841, Unigene9059\_Sample\_011046841, Unigene10844\_Sample\_011046841, Unigene11269\_Sample\_011046841, Unigene11464\_Sample\_011046841, Unigene11676\_Sample\_011046841, Unigene12379\_Sample\_011046841, Unigene12624\_Sample\_011046841, Unigene12943\_Sample\_011046841, Unigene13718\_Sample\_011046841, Unigene13722\_Sample\_011046841, Unigene13881\_Sample\_011046841, Unigene16650\_Sample\_011046841, Unigene21418\_Sample\_011046841, Unigene26587\_Sample\_011046841, Unigene26850\_Sample\_011046841, Unigene32938\_Sample\_011046841, Unigene34589\_Sample\_011046841, Unigene37278\_Sample\_011046841, Unigene38644\_Sample\_011046841, Unigene38889\_Sample\_011046841, Unigene40989\_Sample\_011046841, Unigene41574\_Sample\_011046841, Unigene43409\_Sample\_011046841, Unigene44906\_Sample\_011046841, Unigene45592\_Sample\_011046841, Unigene46219\_Sample\_011046841, Unigene47210\_Sample\_011046841, Unigene47555\_Sample\_011046841, Unigene49399\_Sample\_011046841, Unigene49494\_Sample\_011046841, Unigene50676\_Sample\_011046841, Unigene51782\_Sample\_011046841, Unigene52146\_Sample\_011046841, Unigene53714\_Sample\_011046841, Unigene54329\_Sample\_011046841, Unigene54979\_Sample\_011046841, Unigene55709\_Sample\_011046841, Unigene56798\_Sample\_011046841, Unigene57278\_Sample\_011046841, Unigene57597\_Sample\_011046841, Unigene58564\_Sample\_011046841, Unigene58821\_Sample\_011046841, Unigene59095\_Sample\_011046841, Unigene59109\_Sample\_011046841, Unigene59756\_Sample\_011046841, Unigene59822\_Sample\_011046841, Unigene60367\_Sample\_011046841, Unigene60552\_Sample\_011046841, Unigene3582\_Sample\_011046841, Unigene4821\_Sample\_011046841, Unigene7339\_Sample\_011046841, Unigene9726\_Sample\_011046841, Unigene11932\_Sample\_011046841, Unigene12727\_Sample\_011046841, Unigene13261\_Sample\_011046841, Unigene19285\_Sample\_011046841, Unigene23218\_Sample\_011046841, Unigene28658\_Sample\_011046841, Unigene31321\_Sample\_011046841, Unigene35184\_Sample\_011046841, Unigene39422\_Sample\_011046841, Unigene42071\_Sample\_011046841, Unigene43504\_Sample\_011046841, Unigene44178\_Sample\_011046841, Unigene47387\_Sample\_011046841, Unigene52294\_Sample\_011046841, Unigene54191\_Sample\_011046841, Unigene57158\_Sample\_011046841, Unigene57775\_Sample\_011046841, Unigene58035\_Sample\_011046841, Unigene58321\_Sample\_011046841, Unigene58818\_Sample\_011046841, Unigene59581\_Sample\_011046841, Unigene2116\_Sample\_011046841, Unigene13853\_Sample\_011046841, Unigene13968\_Sample\_011046841, Unigene19951\_Sample\_011046841, Unigene28860\_Sample\_011046841, Unigene30184\_Sample\_011046841, Unigene32964\_Sample\_011046841, Unigene42339\_Sample\_011046841, Unigene46684\_Sample\_011046841, Unigene48046\_Sample\_011046841, Unigene49022\_Sample\_011046841, Unigene49878\_Sample\_011046841, Unigene59998\_Sample\_011046841, Unigene60102\_Sample\_011046841, Unigene60863\_Sample\_011046841, Unigene15\_Sample\_011046841, Unigene29346\_Sample\_011046841, Unigene45679\_Sample\_011046841, Unigene54333\_Sample\_011046841, Unigene1107\_Sample\_011046841, Unigene11231\_Sample\_011046841, Unigene34657\_Sample\_011046841, Unigene40162\_Sample\_011046841, Unigene52611\_Sample\_011046841, Unigene54828\_Sample\_011046841, Unigene46498\_Sample\_011046841, Unigene51271\_Sample\_011046841, Unigene55389\_Sample\_011046841, Unigene11333\_Sample\_011046841, Unigene18170\_Sample\_011046841, Unigene30153\_Sample\_011046841, Unigene33632\_Sample\_011046841, Unigene57117\_Sample\_011046841, Unigene57641\_Sample\_011046841, Unigene58176\_Sample\_011046841, Unigene60248\_Sample\_011046841, Unigene41298\_Sample\_011046841, Unigene7431\_Sample\_011046841, Unigene16055\_Sample\_011046841, Unigene37613\_Sample\_011046841, Unigene38948\_Sample\_011046841, Unigene43721\_Sample\_011046841, Unigene24961\_Sample\_011046841, Unigene26878\_Sample\_011046841, Unigene32470\_Sample\_011046841, Unigene42580\_Sample\_011046841, Unigene47910\_Sample\_011046841, Unigene48977\_Sample\_011046841, Unigene59748\_Sample\_011046841, Unigene3374\_Sample\_011046841, Unigene46495\_Sample\_011046841, Unigene51582\_Sample\_011046841, Unigene9390\_Sample\_011046841, Unigene14639\_Sample\_011046841, Unigene31101\_Sample\_011046841, Unigene32973\_Sample\_011046841, Unigene31821\_Sample\_011046841, Unigene43437\_Sample\_011046841, Unigene19717\_Sample\_011046841, Unigene28384\_Sample\_011046841, Unigene43718\_Sample\_011046841, Unigene53845\_Sample\_011046841, Unigene15058\_Sample\_011046841, Unigene18305\_Sample\_011046841, Unigene41007\_Sample\_011046841, Unigene19937\_Sample\_011046841, Unigene36078\_Sample\_011046841, Unigene41097\_Sample\_011046841, Unigene48121\_Sample\_011046841, Unigene21228\_Sample\_011046841, Unigene30474\_Sample\_011046841, Unigene19596\_Sample\_011046841, Unigene26777\_Sample\_011046841 |
| 17 | Chronic myeloid leukemia Back to summary table | Unigene865\_Sample\_011046841, Unigene1704\_Sample\_011046841, Unigene4209\_Sample\_011046841, Unigene5122\_Sample\_011046841, Unigene6324\_Sample\_011046841, Unigene7143\_Sample\_011046841, Unigene8035\_Sample\_011046841, Unigene8429\_Sample\_011046841, Unigene10459\_Sample\_011046841, Unigene10970\_Sample\_011046841, Unigene11073\_Sample\_011046841, Unigene12652\_Sample\_011046841, Unigene13069\_Sample\_011046841, Unigene30600\_Sample\_011046841, Unigene41694\_Sample\_011046841, Unigene47292\_Sample\_011046841, Unigene48058\_Sample\_011046841, Unigene54213\_Sample\_011046841, Unigene58460\_Sample\_011046841, Unigene58987\_Sample\_011046841, Unigene59633\_Sample\_011046841, Unigene59978\_Sample\_011046841, Unigene60173\_Sample\_011046841, Unigene60498\_Sample\_011046841, Unigene60664\_Sample\_011046841, Unigene5237\_Sample\_011046841, Unigene12413\_Sample\_011046841, Unigene13667\_Sample\_011046841, Unigene13855\_Sample\_011046841, Unigene35368\_Sample\_011046841, Unigene39360\_Sample\_011046841, Unigene41574\_Sample\_011046841, Unigene45858\_Sample\_011046841, Unigene47555\_Sample\_011046841, Unigene50963\_Sample\_011046841, Unigene54900\_Sample\_011046841, Unigene54932\_Sample\_011046841, Unigene56681\_Sample\_011046841, Unigene57037\_Sample\_011046841, Unigene57566\_Sample\_011046841, Unigene58100\_Sample\_011046841, Unigene58209\_Sample\_011046841, Unigene60427\_Sample\_011046841, Unigene60624\_Sample\_011046841, Unigene60859\_Sample\_011046841, Unigene23218\_Sample\_011046841, Unigene23590\_Sample\_011046841, Unigene28658\_Sample\_011046841, Unigene31438\_Sample\_011046841, Unigene40723\_Sample\_011046841, Unigene40758\_Sample\_011046841, Unigene54909\_Sample\_011046841, Unigene60765\_Sample\_011046841, Unigene32964\_Sample\_011046841, Unigene39807\_Sample\_011046841, Unigene44532\_Sample\_011046841, Unigene56300\_Sample\_011046841, Unigene1928\_Sample\_011046841, Unigene49525\_Sample\_011046841, Unigene54521\_Sample\_011046841, Unigene34657\_Sample\_011046841, Unigene41032\_Sample\_011046841, Unigene47967\_Sample\_011046841, Unigene52354\_Sample\_011046841, Unigene52611\_Sample\_011046841, Unigene5263\_Sample\_011046841, Unigene29318\_Sample\_011046841, Unigene41996\_Sample\_011046841, Unigene47054\_Sample\_011046841, Unigene51756\_Sample\_011046841, Unigene57006\_Sample\_011046841, Unigene46589\_Sample\_011046841, Unigene56426\_Sample\_011046841, Unigene39189\_Sample\_011046841, Unigene53995\_Sample\_011046841, Unigene18975\_Sample\_011046841, Unigene47686\_Sample\_011046841, Unigene47910\_Sample\_011046841, Unigene50832\_Sample\_011046841, Unigene31969\_Sample\_011046841, Unigene49698\_Sample\_011046841, Unigene53768\_Sample\_011046841, Unigene8243\_Sample\_011046841, Unigene32258\_Sample\_011046841, Unigene42358\_Sample\_011046841, Unigene46813\_Sample\_011046841, Unigene17523\_Sample\_011046841, Unigene50416\_Sample\_011046841, Unigene30474\_Sample\_011046841 |
| 18 | Chagas disease (no map in kegg database) Back to summary table | Unigene4209\_Sample\_011046841, Unigene5402\_Sample\_011046841, Unigene6178\_Sample\_011046841, Unigene8859\_Sample\_011046841, Unigene10459\_Sample\_011046841, Unigene11020\_Sample\_011046841, Unigene11769\_Sample\_011046841, Unigene12490\_Sample\_011046841, Unigene13069\_Sample\_011046841, Unigene13481\_Sample\_011046841, Unigene13789\_Sample\_011046841, Unigene27948\_Sample\_011046841, Unigene28326\_Sample\_011046841, Unigene39094\_Sample\_011046841, Unigene52106\_Sample\_011046841, Unigene59189\_Sample\_011046841, Unigene59460\_Sample\_011046841, Unigene60173\_Sample\_011046841, Unigene60498\_Sample\_011046841, Unigene4993\_Sample\_011046841, Unigene5747\_Sample\_011046841, Unigene21962\_Sample\_011046841, Unigene25098\_Sample\_011046841, Unigene30274\_Sample\_011046841, Unigene32550\_Sample\_011046841, Unigene37278\_Sample\_011046841, Unigene41574\_Sample\_011046841, Unigene42106\_Sample\_011046841, Unigene43448\_Sample\_011046841, Unigene47555\_Sample\_011046841, Unigene49399\_Sample\_011046841, Unigene50170\_Sample\_011046841, Unigene52184\_Sample\_011046841, Unigene55948\_Sample\_011046841, Unigene56010\_Sample\_011046841, Unigene56177\_Sample\_011046841, Unigene57215\_Sample\_011046841, Unigene57566\_Sample\_011046841, Unigene58100\_Sample\_011046841, Unigene58564\_Sample\_011046841, Unigene60681\_Sample\_011046841, Unigene8813\_Sample\_011046841, Unigene23590\_Sample\_011046841, Unigene28658\_Sample\_011046841, Unigene32259\_Sample\_011046841, Unigene45776\_Sample\_011046841, Unigene47471\_Sample\_011046841, Unigene52303\_Sample\_011046841, Unigene27828\_Sample\_011046841, Unigene32964\_Sample\_011046841, Unigene35757\_Sample\_011046841, Unigene47860\_Sample\_011046841, Unigene56300\_Sample\_011046841, Unigene45708\_Sample\_011046841, Unigene49525\_Sample\_011046841, Unigene58199\_Sample\_011046841, Unigene59421\_Sample\_011046841, Unigene38302\_Sample\_011046841, Unigene57304\_Sample\_011046841, Unigene60217\_Sample\_011046841, Unigene23818\_Sample\_011046841, Unigene30153\_Sample\_011046841, Unigene51756\_Sample\_011046841, Unigene47947\_Sample\_011046841, Unigene32103\_Sample\_011046841, Unigene53352\_Sample\_011046841, Unigene19526\_Sample\_011046841, Unigene21362\_Sample\_011046841, Unigene42293\_Sample\_011046841, Unigene43918\_Sample\_011046841, Unigene47910\_Sample\_011046841, Unigene50832\_Sample\_011046841, Unigene10863\_Sample\_011046841, Unigene19947\_Sample\_011046841, Unigene14639\_Sample\_011046841, Unigene26316\_Sample\_011046841, Unigene31264\_Sample\_011046841, Unigene31544\_Sample\_011046841, Unigene47487\_Sample\_011046841, Unigene16442\_Sample\_011046841, Unigene30484\_Sample\_011046841, Unigene48345\_Sample\_011046841, Unigene49009\_Sample\_011046841, Unigene29441\_Sample\_011046841, Unigene14181\_Sample\_011046841, Unigene30474\_Sample\_011046841 |
| 19 | Notch signaling pathway Back to summary table | Unigene583\_Sample\_011046841, Unigene2871\_Sample\_011046841, Unigene4236\_Sample\_011046841, Unigene7158\_Sample\_011046841, Unigene9154\_Sample\_011046841, Unigene9668\_Sample\_011046841, Unigene10118\_Sample\_011046841, Unigene10640\_Sample\_011046841, Unigene12213\_Sample\_011046841, Unigene12709\_Sample\_011046841, Unigene13872\_Sample\_011046841, Unigene40554\_Sample\_011046841, Unigene44362\_Sample\_011046841, Unigene45114\_Sample\_011046841, Unigene48058\_Sample\_011046841, Unigene51937\_Sample\_011046841, Unigene53492\_Sample\_011046841, Unigene56742\_Sample\_011046841, Unigene58753\_Sample\_011046841, Unigene59633\_Sample\_011046841, Unigene59765\_Sample\_011046841, Unigene60797\_Sample\_011046841, Unigene993\_Sample\_011046841, Unigene1378\_Sample\_011046841, Unigene2325\_Sample\_011046841, Unigene4508\_Sample\_011046841, Unigene8641\_Sample\_011046841, Unigene9380\_Sample\_011046841, Unigene10879\_Sample\_011046841, Unigene11134\_Sample\_011046841, Unigene11802\_Sample\_011046841, Unigene11899\_Sample\_011046841, Unigene12160\_Sample\_011046841, Unigene12739\_Sample\_011046841, Unigene12888\_Sample\_011046841, Unigene13955\_Sample\_011046841, Unigene22684\_Sample\_011046841, Unigene37469\_Sample\_011046841, Unigene49581\_Sample\_011046841, Unigene53171\_Sample\_011046841, Unigene56283\_Sample\_011046841, Unigene57517\_Sample\_011046841, Unigene57783\_Sample\_011046841, Unigene58387\_Sample\_011046841, Unigene60136\_Sample\_011046841, Unigene60406\_Sample\_011046841, Unigene60728\_Sample\_011046841, Unigene779\_Sample\_011046841, Unigene8180\_Sample\_011046841, Unigene9725\_Sample\_011046841, Unigene47257\_Sample\_011046841, Unigene47460\_Sample\_011046841, Unigene54603\_Sample\_011046841, Unigene56203\_Sample\_011046841, Unigene56875\_Sample\_011046841, Unigene4669\_Sample\_011046841, Unigene39807\_Sample\_011046841, Unigene47759\_Sample\_011046841, Unigene53145\_Sample\_011046841, Unigene2404\_Sample\_011046841, Unigene8726\_Sample\_011046841, Unigene13447\_Sample\_011046841, Unigene13530\_Sample\_011046841, Unigene35872\_Sample\_011046841, Unigene43289\_Sample\_011046841, Unigene59077\_Sample\_011046841, Unigene60680\_Sample\_011046841, Unigene11339\_Sample\_011046841, Unigene52354\_Sample\_011046841, Unigene4785\_Sample\_011046841, Unigene9537\_Sample\_011046841, Unigene19507\_Sample\_011046841, Unigene50423\_Sample\_011046841, Unigene57117\_Sample\_011046841, Unigene8302\_Sample\_011046841, Unigene18975\_Sample\_011046841, Unigene45101\_Sample\_011046841, Unigene49017\_Sample\_011046841, Unigene59748\_Sample\_011046841, Unigene8312\_Sample\_011046841, Unigene17505\_Sample\_011046841, Unigene28384\_Sample\_011046841, Unigene53768\_Sample\_011046841, Unigene8953\_Sample\_011046841, Unigene22955\_Sample\_011046841 |
| 20 | DNA replication Back to summary table | Unigene517\_Sample\_011046841, Unigene1129\_Sample\_011046841, Unigene7905\_Sample\_011046841, Unigene13500\_Sample\_011046841, Unigene22715\_Sample\_011046841, Unigene28864\_Sample\_011046841, Unigene34410\_Sample\_011046841, Unigene38329\_Sample\_011046841, Unigene43894\_Sample\_011046841, Unigene44567\_Sample\_011046841, Unigene45790\_Sample\_011046841, Unigene54555\_Sample\_011046841, Unigene55432\_Sample\_011046841, Unigene55735\_Sample\_011046841, Unigene56979\_Sample\_011046841, Unigene59512\_Sample\_011046841, Unigene59588\_Sample\_011046841, Unigene1158\_Sample\_011046841, Unigene2913\_Sample\_011046841, Unigene5382\_Sample\_011046841, Unigene5967\_Sample\_011046841, Unigene6034\_Sample\_011046841, Unigene8165\_Sample\_011046841, Unigene10315\_Sample\_011046841, Unigene11726\_Sample\_011046841, Unigene13694\_Sample\_011046841, Unigene13774\_Sample\_011046841, Unigene39877\_Sample\_011046841, Unigene45539\_Sample\_011046841, Unigene47221\_Sample\_011046841, Unigene47368\_Sample\_011046841, Unigene47769\_Sample\_011046841, Unigene48609\_Sample\_011046841, Unigene54563\_Sample\_011046841, Unigene55543\_Sample\_011046841, Unigene56883\_Sample\_011046841, Unigene56952\_Sample\_011046841, Unigene57569\_Sample\_011046841, Unigene57929\_Sample\_011046841, Unigene58496\_Sample\_011046841, Unigene58627\_Sample\_011046841, Unigene59532\_Sample\_011046841, Unigene60919\_Sample\_011046841, Unigene967\_Sample\_011046841, Unigene8565\_Sample\_011046841, Unigene11050\_Sample\_011046841, Unigene13430\_Sample\_011046841, Unigene19399\_Sample\_011046841, Unigene20927\_Sample\_011046841, Unigene36469\_Sample\_011046841, Unigene42576\_Sample\_011046841, Unigene42967\_Sample\_011046841, Unigene50396\_Sample\_011046841, Unigene57678\_Sample\_011046841, Unigene22992\_Sample\_011046841, Unigene53041\_Sample\_011046841, Unigene58502\_Sample\_011046841, Unigene16199\_Sample\_011046841, Unigene47187\_Sample\_011046841, Unigene55251\_Sample\_011046841, Unigene11735\_Sample\_011046841, Unigene12261\_Sample\_011046841, Unigene57239\_Sample\_011046841, Unigene59178\_Sample\_011046841, Unigene60375\_Sample\_011046841, Unigene12822\_Sample\_011046841, Unigene6972\_Sample\_011046841, Unigene18715\_Sample\_011046841, Unigene45777\_Sample\_011046841, Unigene59567\_Sample\_011046841, Unigene17752\_Sample\_011046841, Unigene50798\_Sample\_011046841, Unigene1344\_Sample\_011046841, Unigene4847\_Sample\_011046841, Unigene25755\_Sample\_011046841, Unigene33437\_Sample\_011046841, Unigene42749\_Sample\_011046841, Unigene48882\_Sample\_011046841, Unigene51331\_Sample\_011046841, Unigene11233\_Sample\_011046841, Unigene17192\_Sample\_011046841, Unigene40117\_Sample\_011046841, Unigene17439\_Sample\_011046841, Unigene31249\_Sample\_011046841, Unigene33738\_Sample\_011046841 |
| 21 | VEGF signaling pathway Back to summary table | Unigene2138\_Sample\_011046841, Unigene3199\_Sample\_011046841, Unigene4209\_Sample\_011046841, Unigene7143\_Sample\_011046841, Unigene10459\_Sample\_011046841, Unigene11999\_Sample\_011046841, Unigene12527\_Sample\_011046841, Unigene13069\_Sample\_011046841, Unigene40230\_Sample\_011046841, Unigene48524\_Sample\_011046841, Unigene51426\_Sample\_011046841, Unigene55404\_Sample\_011046841, Unigene57609\_Sample\_011046841, Unigene58460\_Sample\_011046841, Unigene60173\_Sample\_011046841, Unigene60252\_Sample\_011046841, Unigene60462\_Sample\_011046841, Unigene60498\_Sample\_011046841, Unigene2509\_Sample\_011046841, Unigene3353\_Sample\_011046841, Unigene3363\_Sample\_011046841, Unigene5740\_Sample\_011046841, Unigene7709\_Sample\_011046841, Unigene9867\_Sample\_011046841, Unigene10346\_Sample\_011046841, Unigene13329\_Sample\_011046841, Unigene13945\_Sample\_011046841, Unigene21418\_Sample\_011046841, Unigene40209\_Sample\_011046841, Unigene41574\_Sample\_011046841, Unigene43409\_Sample\_011046841, Unigene45592\_Sample\_011046841, Unigene46219\_Sample\_011046841, Unigene47555\_Sample\_011046841, Unigene48772\_Sample\_011046841, Unigene50170\_Sample\_011046841, Unigene50677\_Sample\_011046841, Unigene53334\_Sample\_011046841, Unigene54900\_Sample\_011046841, Unigene57566\_Sample\_011046841, Unigene58100\_Sample\_011046841, Unigene58611\_Sample\_011046841, Unigene58821\_Sample\_011046841, Unigene23590\_Sample\_011046841, Unigene28658\_Sample\_011046841, Unigene46930\_Sample\_011046841, Unigene56567\_Sample\_011046841, Unigene59322\_Sample\_011046841, Unigene59581\_Sample\_011046841, Unigene30184\_Sample\_011046841, Unigene32964\_Sample\_011046841, Unigene42893\_Sample\_011046841, Unigene56300\_Sample\_011046841, Unigene49525\_Sample\_011046841, Unigene59421\_Sample\_011046841, Unigene2942\_Sample\_011046841, Unigene9329\_Sample\_011046841, Unigene38674\_Sample\_011046841, Unigene29318\_Sample\_011046841, Unigene51756\_Sample\_011046841, Unigene56139\_Sample\_011046841, Unigene57641\_Sample\_011046841, Unigene16098\_Sample\_011046841, Unigene12521\_Sample\_011046841, Unigene21362\_Sample\_011046841, Unigene42580\_Sample\_011046841, Unigene43099\_Sample\_011046841, Unigene47429\_Sample\_011046841, Unigene47686\_Sample\_011046841, Unigene47872\_Sample\_011046841, Unigene47910\_Sample\_011046841, Unigene49087\_Sample\_011046841, Unigene50832\_Sample\_011046841, Unigene54663\_Sample\_011046841, Unigene31264\_Sample\_011046841, Unigene16442\_Sample\_011046841, Unigene30484\_Sample\_011046841, Unigene19937\_Sample\_011046841, Unigene46813\_Sample\_011046841, Unigene30474\_Sample\_011046841, Unigene19596\_Sample\_011046841 |
| 22 | Dorso-ventral axis formation Back to summary table | Unigene583\_Sample\_011046841, Unigene865\_Sample\_011046841, Unigene2871\_Sample\_011046841, Unigene6127\_Sample\_011046841, Unigene7020\_Sample\_011046841, Unigene7143\_Sample\_011046841, Unigene7809\_Sample\_011046841, Unigene9154\_Sample\_011046841, Unigene10118\_Sample\_011046841, Unigene10970\_Sample\_011046841, Unigene11130\_Sample\_011046841, Unigene15356\_Sample\_011046841, Unigene29183\_Sample\_011046841, Unigene40554\_Sample\_011046841, Unigene51937\_Sample\_011046841, Unigene58460\_Sample\_011046841, Unigene58987\_Sample\_011046841, Unigene59765\_Sample\_011046841, Unigene1414\_Sample\_011046841, Unigene2197\_Sample\_011046841, Unigene2320\_Sample\_011046841, Unigene2349\_Sample\_011046841, Unigene3929\_Sample\_011046841, Unigene4256\_Sample\_011046841, Unigene4508\_Sample\_011046841, Unigene9380\_Sample\_011046841, Unigene9971\_Sample\_011046841, Unigene10879\_Sample\_011046841, Unigene11899\_Sample\_011046841, Unigene12160\_Sample\_011046841, Unigene12943\_Sample\_011046841, Unigene13855\_Sample\_011046841, Unigene13955\_Sample\_011046841, Unigene22684\_Sample\_011046841, Unigene41373\_Sample\_011046841, Unigene41574\_Sample\_011046841, Unigene47555\_Sample\_011046841, Unigene54045\_Sample\_011046841, Unigene55306\_Sample\_011046841, Unigene56681\_Sample\_011046841, Unigene56798\_Sample\_011046841, Unigene57783\_Sample\_011046841, Unigene60406\_Sample\_011046841, Unigene9093\_Sample\_011046841, Unigene9725\_Sample\_011046841, Unigene28658\_Sample\_011046841, Unigene40059\_Sample\_011046841, Unigene40758\_Sample\_011046841, Unigene47257\_Sample\_011046841, Unigene54603\_Sample\_011046841, Unigene55504\_Sample\_011046841, Unigene56203\_Sample\_011046841, Unigene56875\_Sample\_011046841, Unigene57775\_Sample\_011046841, Unigene60765\_Sample\_011046841, Unigene100\_Sample\_011046841, Unigene32964\_Sample\_011046841, Unigene60863\_Sample\_011046841, Unigene2404\_Sample\_011046841, Unigene13530\_Sample\_011046841, Unigene43289\_Sample\_011046841, Unigene1107\_Sample\_011046841, Unigene53075\_Sample\_011046841, Unigene59010\_Sample\_011046841, Unigene47054\_Sample\_011046841, Unigene50423\_Sample\_011046841, Unigene50110\_Sample\_011046841, Unigene53995\_Sample\_011046841, Unigene7035\_Sample\_011046841, Unigene45101\_Sample\_011046841, Unigene47910\_Sample\_011046841, Unigene8312\_Sample\_011046841, Unigene17505\_Sample\_011046841, Unigene43718\_Sample\_011046841, Unigene52096\_Sample\_011046841, Unigene32258\_Sample\_011046841, Unigene46813\_Sample\_011046841, Unigene30474\_Sample\_011046841, Unigene26777\_Sample\_011046841 |
| 23 | mTOR signaling pathway Back to summary table | Unigene1793\_Sample\_011046841, Unigene2493\_Sample\_011046841, Unigene4209\_Sample\_011046841, Unigene9082\_Sample\_011046841, Unigene10459\_Sample\_011046841, Unigene10970\_Sample\_011046841, Unigene11314\_Sample\_011046841, Unigene11706\_Sample\_011046841, Unigene13069\_Sample\_011046841, Unigene13551\_Sample\_011046841, Unigene47924\_Sample\_011046841, Unigene50434\_Sample\_011046841, Unigene54601\_Sample\_011046841, Unigene60173\_Sample\_011046841, Unigene60498\_Sample\_011046841, Unigene4892\_Sample\_011046841, Unigene5220\_Sample\_011046841, Unigene5744\_Sample\_011046841, Unigene6117\_Sample\_011046841, Unigene11639\_Sample\_011046841, Unigene13332\_Sample\_011046841, Unigene30456\_Sample\_011046841, Unigene41574\_Sample\_011046841, Unigene47555\_Sample\_011046841, Unigene47936\_Sample\_011046841, Unigene49534\_Sample\_011046841, Unigene50674\_Sample\_011046841, Unigene54626\_Sample\_011046841, Unigene56025\_Sample\_011046841, Unigene56232\_Sample\_011046841, Unigene57566\_Sample\_011046841, Unigene58100\_Sample\_011046841, Unigene58285\_Sample\_011046841, Unigene60787\_Sample\_011046841, Unigene12975\_Sample\_011046841, Unigene13367\_Sample\_011046841, Unigene23590\_Sample\_011046841, Unigene28658\_Sample\_011046841, Unigene31181\_Sample\_011046841, Unigene53956\_Sample\_011046841, Unigene58021\_Sample\_011046841, Unigene59479\_Sample\_011046841, Unigene486\_Sample\_011046841, Unigene32947\_Sample\_011046841, Unigene32964\_Sample\_011046841, Unigene45982\_Sample\_011046841, Unigene49671\_Sample\_011046841, Unigene56300\_Sample\_011046841, Unigene58058\_Sample\_011046841, Unigene59061\_Sample\_011046841, Unigene5570\_Sample\_011046841, Unigene49525\_Sample\_011046841, Unigene52866\_Sample\_011046841, Unigene12846\_Sample\_011046841, Unigene47967\_Sample\_011046841, Unigene49876\_Sample\_011046841, Unigene56028\_Sample\_011046841, Unigene51756\_Sample\_011046841, Unigene55344\_Sample\_011046841, Unigene48847\_Sample\_011046841, Unigene26229\_Sample\_011046841, Unigene53995\_Sample\_011046841, Unigene39020\_Sample\_011046841, Unigene47910\_Sample\_011046841, Unigene50445\_Sample\_011046841, Unigene50832\_Sample\_011046841, Unigene51916\_Sample\_011046841, Unigene60132\_Sample\_011046841, Unigene48528\_Sample\_011046841, Unigene60003\_Sample\_011046841, Unigene52183\_Sample\_011046841, Unigene27138\_Sample\_011046841, Unigene41406\_Sample\_011046841, Unigene58511\_Sample\_011046841, Unigene4308\_Sample\_011046841, Unigene39361\_Sample\_011046841, Unigene17997\_Sample\_011046841, Unigene30474\_Sample\_011046841, Unigene18796\_Sample\_011046841 |
| 24 | Pyrimidine metabolism Back to summary table | Unigene1129\_Sample\_011046841, Unigene5865\_Sample\_011046841, Unigene12300\_Sample\_011046841, Unigene13500\_Sample\_011046841, Unigene19673\_Sample\_011046841, Unigene25547\_Sample\_011046841, Unigene32814\_Sample\_011046841, Unigene34404\_Sample\_011046841, Unigene37180\_Sample\_011046841, Unigene38329\_Sample\_011046841, Unigene43894\_Sample\_011046841, Unigene44949\_Sample\_011046841, Unigene45012\_Sample\_011046841, Unigene49914\_Sample\_011046841, Unigene50631\_Sample\_011046841, Unigene51029\_Sample\_011046841, Unigene51739\_Sample\_011046841, Unigene52799\_Sample\_011046841, Unigene54025\_Sample\_011046841, Unigene54097\_Sample\_011046841, Unigene55376\_Sample\_011046841, Unigene55779\_Sample\_011046841, Unigene56049\_Sample\_011046841, Unigene56375\_Sample\_011046841, Unigene57086\_Sample\_011046841, Unigene57712\_Sample\_011046841, Unigene57770\_Sample\_011046841, Unigene59609\_Sample\_011046841, Unigene60235\_Sample\_011046841, Unigene60809\_Sample\_011046841, Unigene1158\_Sample\_011046841, Unigene2913\_Sample\_011046841, Unigene5150\_Sample\_011046841, Unigene5329\_Sample\_011046841, Unigene5336\_Sample\_011046841, Unigene5382\_Sample\_011046841, Unigene5967\_Sample\_011046841, Unigene6034\_Sample\_011046841, Unigene6828\_Sample\_011046841, Unigene9769\_Sample\_011046841, Unigene11726\_Sample\_011046841, Unigene13694\_Sample\_011046841, Unigene23629\_Sample\_011046841, Unigene26220\_Sample\_011046841, Unigene27510\_Sample\_011046841, Unigene34859\_Sample\_011046841, Unigene39621\_Sample\_011046841, Unigene41171\_Sample\_011046841, Unigene42325\_Sample\_011046841, Unigene42399\_Sample\_011046841, Unigene42733\_Sample\_011046841, Unigene42994\_Sample\_011046841, Unigene44275\_Sample\_011046841, Unigene47221\_Sample\_011046841, Unigene47506\_Sample\_011046841, Unigene48003\_Sample\_011046841, Unigene48609\_Sample\_011046841, Unigene48881\_Sample\_011046841, Unigene48927\_Sample\_011046841, Unigene49713\_Sample\_011046841, Unigene50108\_Sample\_011046841, Unigene50726\_Sample\_011046841, Unigene51933\_Sample\_011046841, Unigene52322\_Sample\_011046841, Unigene52423\_Sample\_011046841, Unigene52769\_Sample\_011046841, Unigene53305\_Sample\_011046841, Unigene53419\_Sample\_011046841, Unigene54563\_Sample\_011046841, Unigene55489\_Sample\_011046841, Unigene55954\_Sample\_011046841, Unigene56333\_Sample\_011046841, Unigene56952\_Sample\_011046841, Unigene57455\_Sample\_011046841, Unigene57569\_Sample\_011046841, Unigene58152\_Sample\_011046841, Unigene58692\_Sample\_011046841, Unigene58721\_Sample\_011046841, Unigene59330\_Sample\_011046841, Unigene59634\_Sample\_011046841, Unigene60522\_Sample\_011046841, Unigene60682\_Sample\_011046841, Unigene60900\_Sample\_011046841, Unigene60919\_Sample\_011046841, Unigene967\_Sample\_011046841, Unigene5708\_Sample\_011046841, Unigene9806\_Sample\_011046841, Unigene19399\_Sample\_011046841, Unigene20854\_Sample\_011046841, Unigene24351\_Sample\_011046841, Unigene24492\_Sample\_011046841, Unigene24615\_Sample\_011046841, Unigene26928\_Sample\_011046841, Unigene31723\_Sample\_011046841, Unigene41499\_Sample\_011046841, Unigene42860\_Sample\_011046841, Unigene45635\_Sample\_011046841, Unigene48876\_Sample\_011046841, Unigene49935\_Sample\_011046841, Unigene51481\_Sample\_011046841, Unigene51837\_Sample\_011046841, Unigene52658\_Sample\_011046841, Unigene53838\_Sample\_011046841, Unigene54368\_Sample\_011046841, Unigene55381\_Sample\_011046841, Unigene56098\_Sample\_011046841, Unigene60494\_Sample\_011046841, Unigene60847\_Sample\_011046841, Unigene2821\_Sample\_011046841, Unigene5875\_Sample\_011046841, Unigene10446\_Sample\_011046841, Unigene15607\_Sample\_011046841, Unigene22992\_Sample\_011046841, Unigene27899\_Sample\_011046841, Unigene39016\_Sample\_011046841, Unigene42794\_Sample\_011046841, Unigene44282\_Sample\_011046841, Unigene47129\_Sample\_011046841, Unigene52064\_Sample\_011046841, Unigene54965\_Sample\_011046841, Unigene57735\_Sample\_011046841, Unigene59492\_Sample\_011046841, Unigene47187\_Sample\_011046841, Unigene50947\_Sample\_011046841, Unigene59545\_Sample\_011046841, Unigene11735\_Sample\_011046841, Unigene12261\_Sample\_011046841, Unigene34352\_Sample\_011046841, Unigene42159\_Sample\_011046841, Unigene42685\_Sample\_011046841, Unigene49014\_Sample\_011046841, Unigene49168\_Sample\_011046841, Unigene53487\_Sample\_011046841, Unigene11712\_Sample\_011046841, Unigene18805\_Sample\_011046841, Unigene37401\_Sample\_011046841, Unigene6972\_Sample\_011046841, Unigene18715\_Sample\_011046841, Unigene36472\_Sample\_011046841, Unigene41364\_Sample\_011046841, Unigene45777\_Sample\_011046841, Unigene48730\_Sample\_011046841, Unigene49498\_Sample\_011046841, Unigene54790\_Sample\_011046841, Unigene59567\_Sample\_011046841, Unigene8080\_Sample\_011046841, Unigene37427\_Sample\_011046841, Unigene57802\_Sample\_011046841, Unigene21863\_Sample\_011046841, Unigene28225\_Sample\_011046841, Unigene37087\_Sample\_011046841, Unigene49106\_Sample\_011046841, Unigene53073\_Sample\_011046841, Unigene4847\_Sample\_011046841, Unigene31129\_Sample\_011046841, Unigene42749\_Sample\_011046841, Unigene43528\_Sample\_011046841, Unigene43813\_Sample\_011046841, Unigene54638\_Sample\_011046841, Unigene27881\_Sample\_011046841, Unigene29219\_Sample\_011046841, Unigene30309\_Sample\_011046841, Unigene11166\_Sample\_011046841, Unigene21757\_Sample\_011046841, Unigene27719\_Sample\_011046841, Unigene41680\_Sample\_011046841, Unigene17439\_Sample\_011046841, Unigene18613\_Sample\_011046841, Unigene35708\_Sample\_011046841, Unigene22492\_Sample\_011046841, Unigene9865\_Sample\_011046841, Unigene20294\_Sample\_011046841, Unigene22482\_Sample\_011046841, Unigene47587\_Sample\_011046841, Unigene37430\_Sample\_011046841, Unigene31679\_Sample\_011046841, Unigene38646\_Sample\_011046841, Unigene52018\_Sample\_011046841, Unigene21775\_Sample\_011046841, Unigene30813\_Sample\_011046841, Unigene29124\_Sample\_011046841 |
| 25 | Tight junction Back to summary table | Unigene223\_Sample\_011046841, Unigene290\_Sample\_011046841, Unigene863\_Sample\_011046841, Unigene1210\_Sample\_011046841, Unigene1273\_Sample\_011046841, Unigene3199\_Sample\_011046841, Unigene4440\_Sample\_011046841, Unigene4627\_Sample\_011046841, Unigene4963\_Sample\_011046841, Unigene5109\_Sample\_011046841, Unigene5402\_Sample\_011046841, Unigene5529\_Sample\_011046841, Unigene5953\_Sample\_011046841, Unigene7142\_Sample\_011046841, Unigene8429\_Sample\_011046841, Unigene8884\_Sample\_011046841, Unigene9237\_Sample\_011046841, Unigene10098\_Sample\_011046841, Unigene10250\_Sample\_011046841, Unigene10443\_Sample\_011046841, Unigene10744\_Sample\_011046841, Unigene11034\_Sample\_011046841, Unigene11769\_Sample\_011046841, Unigene11938\_Sample\_011046841, Unigene11951\_Sample\_011046841, Unigene12000\_Sample\_011046841, Unigene12278\_Sample\_011046841, Unigene12490\_Sample\_011046841, Unigene13188\_Sample\_011046841, Unigene13250\_Sample\_011046841, Unigene13370\_Sample\_011046841, Unigene13402\_Sample\_011046841, Unigene13808\_Sample\_011046841, Unigene13974\_Sample\_011046841, Unigene22077\_Sample\_011046841, Unigene27010\_Sample\_011046841, Unigene27948\_Sample\_011046841, Unigene28326\_Sample\_011046841, Unigene32048\_Sample\_011046841, Unigene40230\_Sample\_011046841, Unigene42901\_Sample\_011046841, Unigene48622\_Sample\_011046841, Unigene48836\_Sample\_011046841, Unigene50823\_Sample\_011046841, Unigene51251\_Sample\_011046841, Unigene54227\_Sample\_011046841, Unigene55452\_Sample\_011046841, Unigene56056\_Sample\_011046841, Unigene56248\_Sample\_011046841, Unigene56758\_Sample\_011046841, Unigene57088\_Sample\_011046841, Unigene57108\_Sample\_011046841, Unigene57160\_Sample\_011046841, Unigene57183\_Sample\_011046841, Unigene57298\_Sample\_011046841, Unigene57715\_Sample\_011046841, Unigene57887\_Sample\_011046841, Unigene58131\_Sample\_011046841, Unigene58189\_Sample\_011046841, Unigene58307\_Sample\_011046841, Unigene58763\_Sample\_011046841, Unigene58906\_Sample\_011046841, Unigene58924\_Sample\_011046841, Unigene59006\_Sample\_011046841, Unigene59151\_Sample\_011046841, Unigene59457\_Sample\_011046841, Unigene59460\_Sample\_011046841, Unigene59856\_Sample\_011046841, Unigene60061\_Sample\_011046841, Unigene60267\_Sample\_011046841, Unigene60471\_Sample\_011046841, Unigene60510\_Sample\_011046841, Unigene60657\_Sample\_011046841, Unigene60658\_Sample\_011046841, Unigene60695\_Sample\_011046841, Unigene60794\_Sample\_011046841, Unigene2588\_Sample\_011046841, Unigene2768\_Sample\_011046841, Unigene2868\_Sample\_011046841, Unigene3363\_Sample\_011046841, Unigene4916\_Sample\_011046841, Unigene4993\_Sample\_011046841, Unigene5568\_Sample\_011046841, Unigene6199\_Sample\_011046841, Unigene7238\_Sample\_011046841, Unigene9517\_Sample\_011046841, Unigene9606\_Sample\_011046841, Unigene9867\_Sample\_011046841, Unigene11464\_Sample\_011046841, Unigene11626\_Sample\_011046841, Unigene11629\_Sample\_011046841, Unigene11787\_Sample\_011046841, Unigene12138\_Sample\_011046841, Unigene12853\_Sample\_011046841, Unigene12856\_Sample\_011046841, Unigene12885\_Sample\_011046841, Unigene13280\_Sample\_011046841, Unigene13452\_Sample\_011046841, Unigene13564\_Sample\_011046841, Unigene13745\_Sample\_011046841, Unigene13881\_Sample\_011046841, Unigene16650\_Sample\_011046841, Unigene21418\_Sample\_011046841, Unigene25098\_Sample\_011046841, Unigene26850\_Sample\_011046841, Unigene30274\_Sample\_011046841, Unigene34750\_Sample\_011046841, Unigene36178\_Sample\_011046841, Unigene38644\_Sample\_011046841, Unigene41967\_Sample\_011046841, Unigene42106\_Sample\_011046841, Unigene43409\_Sample\_011046841, Unigene43448\_Sample\_011046841, Unigene44906\_Sample\_011046841, Unigene45592\_Sample\_011046841, Unigene46219\_Sample\_011046841, Unigene46485\_Sample\_011046841, Unigene50676\_Sample\_011046841, Unigene51070\_Sample\_011046841, Unigene51782\_Sample\_011046841, Unigene52184\_Sample\_011046841, Unigene53602\_Sample\_011046841, Unigene53714\_Sample\_011046841, Unigene53983\_Sample\_011046841, Unigene54329\_Sample\_011046841, Unigene54859\_Sample\_011046841, Unigene54900\_Sample\_011046841, Unigene54979\_Sample\_011046841, Unigene55755\_Sample\_011046841, Unigene55807\_Sample\_011046841, Unigene56010\_Sample\_011046841, Unigene56403\_Sample\_011046841, Unigene56891\_Sample\_011046841, Unigene56938\_Sample\_011046841, Unigene58100\_Sample\_011046841, Unigene58107\_Sample\_011046841, Unigene58448\_Sample\_011046841, Unigene58578\_Sample\_011046841, Unigene58619\_Sample\_011046841, Unigene58816\_Sample\_011046841, Unigene58821\_Sample\_011046841, Unigene58953\_Sample\_011046841, Unigene59018\_Sample\_011046841, Unigene59095\_Sample\_011046841, Unigene59676\_Sample\_011046841, Unigene59726\_Sample\_011046841, Unigene59809\_Sample\_011046841, Unigene59939\_Sample\_011046841, Unigene60030\_Sample\_011046841, Unigene60169\_Sample\_011046841, Unigene60271\_Sample\_011046841, Unigene60367\_Sample\_011046841, Unigene60427\_Sample\_011046841, Unigene60474\_Sample\_011046841, Unigene60508\_Sample\_011046841, Unigene60681\_Sample\_011046841, Unigene2725\_Sample\_011046841, Unigene4821\_Sample\_011046841, Unigene7339\_Sample\_011046841, Unigene9726\_Sample\_011046841, Unigene11014\_Sample\_011046841, Unigene11743\_Sample\_011046841, Unigene12294\_Sample\_011046841, Unigene13367\_Sample\_011046841, Unigene19285\_Sample\_011046841, Unigene23590\_Sample\_011046841, Unigene35184\_Sample\_011046841, Unigene37492\_Sample\_011046841, Unigene38773\_Sample\_011046841, Unigene39422\_Sample\_011046841, Unigene40557\_Sample\_011046841, Unigene43504\_Sample\_011046841, Unigene44178\_Sample\_011046841, Unigene45776\_Sample\_011046841, Unigene47387\_Sample\_011046841, Unigene51309\_Sample\_011046841, Unigene52984\_Sample\_011046841, Unigene54191\_Sample\_011046841, Unigene55818\_Sample\_011046841, Unigene57158\_Sample\_011046841, Unigene57365\_Sample\_011046841, Unigene57890\_Sample\_011046841, Unigene58035\_Sample\_011046841, Unigene58128\_Sample\_011046841, Unigene58134\_Sample\_011046841, Unigene58336\_Sample\_011046841, Unigene58818\_Sample\_011046841, Unigene59430\_Sample\_011046841, Unigene59581\_Sample\_011046841, Unigene59784\_Sample\_011046841, Unigene8400\_Sample\_011046841, Unigene13853\_Sample\_011046841, Unigene13968\_Sample\_011046841, Unigene19951\_Sample\_011046841, Unigene27828\_Sample\_011046841, Unigene28860\_Sample\_011046841, Unigene32497\_Sample\_011046841, Unigene35757\_Sample\_011046841, Unigene42339\_Sample\_011046841, Unigene45295\_Sample\_011046841, Unigene48046\_Sample\_011046841, Unigene54416\_Sample\_011046841, Unigene59819\_Sample\_011046841, Unigene59976\_Sample\_011046841, Unigene59998\_Sample\_011046841, Unigene60102\_Sample\_011046841, Unigene60857\_Sample\_011046841, Unigene60894\_Sample\_011046841, Unigene5420\_Sample\_011046841, Unigene10726\_Sample\_011046841, Unigene29346\_Sample\_011046841, Unigene49525\_Sample\_011046841, Unigene59577\_Sample\_011046841, Unigene60749\_Sample\_011046841, Unigene2942\_Sample\_011046841, Unigene9329\_Sample\_011046841, Unigene36872\_Sample\_011046841, Unigene40162\_Sample\_011046841, Unigene44103\_Sample\_011046841, Unigene49290\_Sample\_011046841, Unigene54220\_Sample\_011046841, Unigene54691\_Sample\_011046841, Unigene54828\_Sample\_011046841, Unigene57304\_Sample\_011046841, Unigene57418\_Sample\_011046841, Unigene21919\_Sample\_011046841, Unigene48749\_Sample\_011046841, Unigene58426\_Sample\_011046841, Unigene59517\_Sample\_011046841, Unigene11333\_Sample\_011046841, Unigene27378\_Sample\_011046841, Unigene29113\_Sample\_011046841, Unigene39557\_Sample\_011046841, Unigene51756\_Sample\_011046841, Unigene56073\_Sample\_011046841, Unigene56139\_Sample\_011046841, Unigene57641\_Sample\_011046841, Unigene58176\_Sample\_011046841, Unigene5468\_Sample\_011046841, Unigene16098\_Sample\_011046841, Unigene47947\_Sample\_011046841, Unigene60705\_Sample\_011046841, Unigene7431\_Sample\_011046841, Unigene16055\_Sample\_011046841, Unigene24508\_Sample\_011046841, Unigene32103\_Sample\_011046841, Unigene38948\_Sample\_011046841, Unigene43721\_Sample\_011046841, Unigene53352\_Sample\_011046841, Unigene56673\_Sample\_011046841, Unigene9715\_Sample\_011046841, Unigene12521\_Sample\_011046841, Unigene19526\_Sample\_011046841, Unigene24961\_Sample\_011046841, Unigene26878\_Sample\_011046841, Unigene32470\_Sample\_011046841, Unigene40166\_Sample\_011046841, Unigene43099\_Sample\_011046841, Unigene43918\_Sample\_011046841, Unigene44025\_Sample\_011046841, Unigene45282\_Sample\_011046841, Unigene47872\_Sample\_011046841, Unigene48977\_Sample\_011046841, Unigene59250\_Sample\_011046841, Unigene3374\_Sample\_011046841, Unigene31969\_Sample\_011046841, Unigene46495\_Sample\_011046841, Unigene52883\_Sample\_011046841, Unigene31821\_Sample\_011046841, Unigene10280\_Sample\_011046841, Unigene19717\_Sample\_011046841, Unigene53845\_Sample\_011046841, Unigene45675\_Sample\_011046841, Unigene15058\_Sample\_011046841, Unigene18305\_Sample\_011046841, Unigene33186\_Sample\_011046841, Unigene19937\_Sample\_011046841, Unigene36078\_Sample\_011046841, Unigene41097\_Sample\_011046841, Unigene48121\_Sample\_011046841, Unigene21228\_Sample\_011046841, Unigene47780\_Sample\_011046841, Unigene19596\_Sample\_011046841 |
| 26 | Adipocytokine signaling pathway Back to summary table | Unigene2493\_Sample\_011046841, Unigene8035\_Sample\_011046841, Unigene8581\_Sample\_011046841, Unigene9082\_Sample\_011046841, Unigene9858\_Sample\_011046841, Unigene11706\_Sample\_011046841, Unigene20686\_Sample\_011046841, Unigene25984\_Sample\_011046841, Unigene30323\_Sample\_011046841, Unigene36781\_Sample\_011046841, Unigene41694\_Sample\_011046841, Unigene45580\_Sample\_011046841, Unigene51354\_Sample\_011046841, Unigene55348\_Sample\_011046841, Unigene58676\_Sample\_011046841, Unigene58796\_Sample\_011046841, Unigene59402\_Sample\_011046841, Unigene59971\_Sample\_011046841, Unigene60298\_Sample\_011046841, Unigene60304\_Sample\_011046841, Unigene4892\_Sample\_011046841, Unigene5220\_Sample\_011046841, Unigene8251\_Sample\_011046841, Unigene21319\_Sample\_011046841, Unigene26631\_Sample\_011046841, Unigene28584\_Sample\_011046841, Unigene30456\_Sample\_011046841, Unigene35368\_Sample\_011046841, Unigene45858\_Sample\_011046841, Unigene50674\_Sample\_011046841, Unigene54468\_Sample\_011046841, Unigene54650\_Sample\_011046841, Unigene54986\_Sample\_011046841, Unigene56681\_Sample\_011046841, Unigene58100\_Sample\_011046841, Unigene59746\_Sample\_011046841, Unigene60532\_Sample\_011046841, Unigene60822\_Sample\_011046841, Unigene23218\_Sample\_011046841, Unigene23590\_Sample\_011046841, Unigene31181\_Sample\_011046841, Unigene31438\_Sample\_011046841, Unigene40723\_Sample\_011046841, Unigene56856\_Sample\_011046841, Unigene59541\_Sample\_011046841, Unigene14511\_Sample\_011046841, Unigene29226\_Sample\_011046841, Unigene31165\_Sample\_011046841, Unigene45689\_Sample\_011046841, Unigene27921\_Sample\_011046841, Unigene49525\_Sample\_011046841, Unigene54521\_Sample\_011046841, Unigene60217\_Sample\_011046841, Unigene5263\_Sample\_011046841, Unigene23984\_Sample\_011046841, Unigene41996\_Sample\_011046841, Unigene51756\_Sample\_011046841, Unigene52695\_Sample\_011046841, Unigene55344\_Sample\_011046841, Unigene56082\_Sample\_011046841, Unigene56772\_Sample\_011046841, Unigene25908\_Sample\_011046841, Unigene18294\_Sample\_011046841, Unigene19677\_Sample\_011046841, Unigene49579\_Sample\_011046841, Unigene51916\_Sample\_011046841, Unigene49698\_Sample\_011046841, Unigene40605\_Sample\_011046841, Unigene58511\_Sample\_011046841, Unigene20350\_Sample\_011046841, Unigene21220\_Sample\_011046841, Unigene29944\_Sample\_011046841, Unigene42358\_Sample\_011046841, Unigene17523\_Sample\_011046841, Unigene22791\_Sample\_011046841, Unigene41604\_Sample\_011046841, Unigene29437\_Sample\_011046841, Unigene39361\_Sample\_011046841 |
| 27 | Gap junction Back to summary table | Unigene865\_Sample\_011046841, Unigene3199\_Sample\_011046841, Unigene6178\_Sample\_011046841, Unigene7143\_Sample\_011046841, Unigene7809\_Sample\_011046841, Unigene8288\_Sample\_011046841, Unigene8859\_Sample\_011046841, Unigene9132\_Sample\_011046841, Unigene11876\_Sample\_011046841, Unigene12998\_Sample\_011046841, Unigene13370\_Sample\_011046841, Unigene13481\_Sample\_011046841, Unigene13789\_Sample\_011046841, Unigene19864\_Sample\_011046841, Unigene19905\_Sample\_011046841, Unigene37665\_Sample\_011046841, Unigene39808\_Sample\_011046841, Unigene40230\_Sample\_011046841, Unigene46058\_Sample\_011046841, Unigene48482\_Sample\_011046841, Unigene51260\_Sample\_011046841, Unigene54214\_Sample\_011046841, Unigene58432\_Sample\_011046841, Unigene58460\_Sample\_011046841, Unigene58987\_Sample\_011046841, Unigene59189\_Sample\_011046841, Unigene59214\_Sample\_011046841, Unigene60531\_Sample\_011046841, Unigene60789\_Sample\_011046841, Unigene1414\_Sample\_011046841, Unigene3363\_Sample\_011046841, Unigene4916\_Sample\_011046841, Unigene4993\_Sample\_011046841, Unigene5744\_Sample\_011046841, Unigene6531\_Sample\_011046841, Unigene9867\_Sample\_011046841, Unigene11526\_Sample\_011046841, Unigene12943\_Sample\_011046841, Unigene13782\_Sample\_011046841, Unigene13855\_Sample\_011046841, Unigene13881\_Sample\_011046841, Unigene18318\_Sample\_011046841, Unigene21962\_Sample\_011046841, Unigene31656\_Sample\_011046841, Unigene31772\_Sample\_011046841, Unigene34566\_Sample\_011046841, Unigene35470\_Sample\_011046841, Unigene38784\_Sample\_011046841, Unigene39753\_Sample\_011046841, Unigene41574\_Sample\_011046841, Unigene45593\_Sample\_011046841, Unigene47555\_Sample\_011046841, Unigene48589\_Sample\_011046841, Unigene53402\_Sample\_011046841, Unigene54900\_Sample\_011046841, Unigene56177\_Sample\_011046841, Unigene56278\_Sample\_011046841, Unigene56647\_Sample\_011046841, Unigene56681\_Sample\_011046841, Unigene56798\_Sample\_011046841, Unigene58821\_Sample\_011046841, Unigene59742\_Sample\_011046841, Unigene59830\_Sample\_011046841, Unigene60427\_Sample\_011046841, Unigene60880\_Sample\_011046841, Unigene2371\_Sample\_011046841, Unigene8813\_Sample\_011046841, Unigene11266\_Sample\_011046841, Unigene12837\_Sample\_011046841, Unigene16810\_Sample\_011046841, Unigene20768\_Sample\_011046841, Unigene28658\_Sample\_011046841, Unigene32259\_Sample\_011046841, Unigene36922\_Sample\_011046841, Unigene40758\_Sample\_011046841, Unigene41668\_Sample\_011046841, Unigene43690\_Sample\_011046841, Unigene44683\_Sample\_011046841, Unigene57775\_Sample\_011046841, Unigene59581\_Sample\_011046841, Unigene60765\_Sample\_011046841, Unigene8139\_Sample\_011046841, Unigene21225\_Sample\_011046841, Unigene26696\_Sample\_011046841, Unigene32964\_Sample\_011046841, Unigene47860\_Sample\_011046841, Unigene50399\_Sample\_011046841, Unigene51604\_Sample\_011046841, Unigene55398\_Sample\_011046841, Unigene55715\_Sample\_011046841, Unigene57670\_Sample\_011046841, Unigene57832\_Sample\_011046841, Unigene60863\_Sample\_011046841, Unigene20407\_Sample\_011046841, Unigene30190\_Sample\_011046841, Unigene41992\_Sample\_011046841, Unigene42686\_Sample\_011046841, Unigene45708\_Sample\_011046841, Unigene54514\_Sample\_011046841, Unigene58199\_Sample\_011046841, Unigene1107\_Sample\_011046841, Unigene2942\_Sample\_011046841, Unigene9329\_Sample\_011046841, Unigene22086\_Sample\_011046841, Unigene38302\_Sample\_011046841, Unigene38763\_Sample\_011046841, Unigene43782\_Sample\_011046841, Unigene51429\_Sample\_011046841, Unigene51736\_Sample\_011046841, Unigene58418\_Sample\_011046841, Unigene58919\_Sample\_011046841, Unigene56828\_Sample\_011046841, Unigene29318\_Sample\_011046841, Unigene32914\_Sample\_011046841, Unigene36020\_Sample\_011046841, Unigene43515\_Sample\_011046841, Unigene46515\_Sample\_011046841, Unigene47054\_Sample\_011046841, Unigene47783\_Sample\_011046841, Unigene48742\_Sample\_011046841, Unigene56139\_Sample\_011046841, Unigene57641\_Sample\_011046841, Unigene5898\_Sample\_011046841, Unigene16098\_Sample\_011046841, Unigene16237\_Sample\_011046841, Unigene42069\_Sample\_011046841, Unigene54376\_Sample\_011046841, Unigene2110\_Sample\_011046841, Unigene21201\_Sample\_011046841, Unigene53259\_Sample\_011046841, Unigene53352\_Sample\_011046841, Unigene55041\_Sample\_011046841, Unigene3810\_Sample\_011046841, Unigene7879\_Sample\_011046841, Unigene12521\_Sample\_011046841, Unigene15705\_Sample\_011046841, Unigene23831\_Sample\_011046841, Unigene28288\_Sample\_011046841, Unigene42940\_Sample\_011046841, Unigene43099\_Sample\_011046841, Unigene47872\_Sample\_011046841, Unigene47910\_Sample\_011046841, Unigene50911\_Sample\_011046841, Unigene59861\_Sample\_011046841, Unigene3264\_Sample\_011046841, Unigene3380\_Sample\_011046841, Unigene10863\_Sample\_011046841, Unigene19947\_Sample\_011046841, Unigene12324\_Sample\_011046841, Unigene19743\_Sample\_011046841, Unigene42133\_Sample\_011046841, Unigene43718\_Sample\_011046841, Unigene53709\_Sample\_011046841, Unigene58675\_Sample\_011046841, Unigene3842\_Sample\_011046841, Unigene18596\_Sample\_011046841, Unigene30349\_Sample\_011046841, Unigene43044\_Sample\_011046841, Unigene44021\_Sample\_011046841, Unigene47487\_Sample\_011046841, Unigene27561\_Sample\_011046841, Unigene14776\_Sample\_011046841, Unigene19352\_Sample\_011046841, Unigene32258\_Sample\_011046841, Unigene35082\_Sample\_011046841, Unigene42917\_Sample\_011046841, Unigene15548\_Sample\_011046841, Unigene52245\_Sample\_011046841, Unigene14248\_Sample\_011046841, Unigene37987\_Sample\_011046841, Unigene46813\_Sample\_011046841, Unigene55720\_Sample\_011046841, Unigene41903\_Sample\_011046841, Unigene29441\_Sample\_011046841, Unigene42368\_Sample\_011046841, Unigene14181\_Sample\_011046841, Unigene30474\_Sample\_011046841, Unigene7048\_Sample\_011046841, Unigene26777\_Sample\_011046841 |
| 28 | Amino sugar and nucleotide sugar metabolism Back to summary table | Unigene140\_Sample\_011046841, Unigene3261\_Sample\_011046841, Unigene22611\_Sample\_011046841, Unigene34739\_Sample\_011046841, Unigene56475\_Sample\_011046841, Unigene59828\_Sample\_011046841, Unigene60759\_Sample\_011046841, Unigene6707\_Sample\_011046841, Unigene7299\_Sample\_011046841, Unigene10217\_Sample\_011046841, Unigene16545\_Sample\_011046841, Unigene16727\_Sample\_011046841, Unigene21670\_Sample\_011046841, Unigene31490\_Sample\_011046841, Unigene35149\_Sample\_011046841, Unigene46957\_Sample\_011046841, Unigene47414\_Sample\_011046841, Unigene50014\_Sample\_011046841, Unigene52526\_Sample\_011046841, Unigene55255\_Sample\_011046841, Unigene57300\_Sample\_011046841, Unigene57710\_Sample\_011046841, Unigene58309\_Sample\_011046841, Unigene59236\_Sample\_011046841, Unigene2189\_Sample\_011046841, Unigene12951\_Sample\_011046841, Unigene23752\_Sample\_011046841, Unigene25475\_Sample\_011046841, Unigene29684\_Sample\_011046841, Unigene33337\_Sample\_011046841, Unigene34482\_Sample\_011046841, Unigene53097\_Sample\_011046841, Unigene58556\_Sample\_011046841, Unigene59675\_Sample\_011046841, Unigene60448\_Sample\_011046841, Unigene13080\_Sample\_011046841, Unigene20762\_Sample\_011046841, Unigene43909\_Sample\_011046841, Unigene49197\_Sample\_011046841, Unigene49912\_Sample\_011046841, Unigene51811\_Sample\_011046841, Unigene56825\_Sample\_011046841, Unigene60318\_Sample\_011046841, Unigene47976\_Sample\_011046841, Unigene7585\_Sample\_011046841, Unigene16008\_Sample\_011046841, Unigene38688\_Sample\_011046841, Unigene39312\_Sample\_011046841, Unigene54358\_Sample\_011046841, Unigene8335\_Sample\_011046841, Unigene6829\_Sample\_011046841, Unigene29271\_Sample\_011046841, Unigene54325\_Sample\_011046841, Unigene4296\_Sample\_011046841, Unigene7779\_Sample\_011046841, Unigene16836\_Sample\_011046841, Unigene17586\_Sample\_011046841, Unigene19914\_Sample\_011046841, Unigene34778\_Sample\_011046841, Unigene20440\_Sample\_011046841, Unigene38547\_Sample\_011046841, Unigene43536\_Sample\_011046841, Unigene39842\_Sample\_011046841, Unigene31042\_Sample\_011046841, Unigene40365\_Sample\_011046841, Unigene41284\_Sample\_011046841, Unigene49192\_Sample\_011046841, Unigene4523\_Sample\_011046841, Unigene43946\_Sample\_011046841, Unigene50987\_Sample\_011046841, Unigene19875\_Sample\_011046841, Unigene32054\_Sample\_011046841, Unigene44836\_Sample\_011046841, Unigene36941\_Sample\_011046841, Unigene33820\_Sample\_011046841 |
| 29 | Vascular smooth muscle contraction Back to summary table | Unigene1137\_Sample\_011046841, Unigene2441\_Sample\_011046841, Unigene3199\_Sample\_011046841, Unigene4416\_Sample\_011046841, Unigene5297\_Sample\_011046841, Unigene6178\_Sample\_011046841, Unigene8859\_Sample\_011046841, Unigene10174\_Sample\_011046841, Unigene10250\_Sample\_011046841, Unigene10970\_Sample\_011046841, Unigene11999\_Sample\_011046841, Unigene12527\_Sample\_011046841, Unigene13398\_Sample\_011046841, Unigene13481\_Sample\_011046841, Unigene13789\_Sample\_011046841, Unigene38234\_Sample\_011046841, Unigene39858\_Sample\_011046841, Unigene39875\_Sample\_011046841, Unigene40230\_Sample\_011046841, Unigene43331\_Sample\_011046841, Unigene43614\_Sample\_011046841, Unigene48170\_Sample\_011046841, Unigene49240\_Sample\_011046841, Unigene53888\_Sample\_011046841, Unigene54982\_Sample\_011046841, Unigene56389\_Sample\_011046841, Unigene57225\_Sample\_011046841, Unigene57298\_Sample\_011046841, Unigene58460\_Sample\_011046841, Unigene59189\_Sample\_011046841, Unigene59382\_Sample\_011046841, Unigene60462\_Sample\_011046841, Unigene60500\_Sample\_011046841, Unigene60657\_Sample\_011046841, Unigene60669\_Sample\_011046841, Unigene60743\_Sample\_011046841, Unigene60789\_Sample\_011046841, Unigene60930\_Sample\_011046841, Unigene1345\_Sample\_011046841, Unigene2095\_Sample\_011046841, Unigene2509\_Sample\_011046841, Unigene3363\_Sample\_011046841, Unigene4210\_Sample\_011046841, Unigene5740\_Sample\_011046841, Unigene5744\_Sample\_011046841, Unigene9867\_Sample\_011046841, Unigene10189\_Sample\_011046841, Unigene10346\_Sample\_011046841, Unigene12867\_Sample\_011046841, Unigene13091\_Sample\_011046841, Unigene13332\_Sample\_011046841, Unigene13494\_Sample\_011046841, Unigene21962\_Sample\_011046841, Unigene23312\_Sample\_011046841, Unigene41574\_Sample\_011046841, Unigene42885\_Sample\_011046841, Unigene47555\_Sample\_011046841, Unigene48081\_Sample\_011046841, Unigene48850\_Sample\_011046841, Unigene49320\_Sample\_011046841, Unigene50163\_Sample\_011046841, Unigene50584\_Sample\_011046841, Unigene54722\_Sample\_011046841, Unigene56177\_Sample\_011046841, Unigene59449\_Sample\_011046841, Unigene59742\_Sample\_011046841, Unigene59902\_Sample\_011046841, Unigene60006\_Sample\_011046841, Unigene60271\_Sample\_011046841, Unigene60655\_Sample\_011046841, Unigene60880\_Sample\_011046841, Unigene3070\_Sample\_011046841, Unigene6597\_Sample\_011046841, Unigene8083\_Sample\_011046841, Unigene8813\_Sample\_011046841, Unigene10623\_Sample\_011046841, Unigene13367\_Sample\_011046841, Unigene18609\_Sample\_011046841, Unigene23751\_Sample\_011046841, Unigene28658\_Sample\_011046841, Unigene32259\_Sample\_011046841, Unigene34582\_Sample\_011046841, Unigene44316\_Sample\_011046841, Unigene48530\_Sample\_011046841, Unigene51051\_Sample\_011046841, Unigene53168\_Sample\_011046841, Unigene54593\_Sample\_011046841, Unigene56456\_Sample\_011046841, Unigene57634\_Sample\_011046841, Unigene58134\_Sample\_011046841, Unigene6435\_Sample\_011046841, Unigene8139\_Sample\_011046841, Unigene8670\_Sample\_011046841, Unigene12791\_Sample\_011046841, Unigene19951\_Sample\_011046841, Unigene32964\_Sample\_011046841, Unigene47860\_Sample\_011046841, Unigene56496\_Sample\_011046841, Unigene57387\_Sample\_011046841, Unigene57675\_Sample\_011046841, Unigene10726\_Sample\_011046841, Unigene13695\_Sample\_011046841, Unigene29346\_Sample\_011046841, Unigene30190\_Sample\_011046841, Unigene45708\_Sample\_011046841, Unigene58199\_Sample\_011046841, Unigene2942\_Sample\_011046841, Unigene9329\_Sample\_011046841, Unigene38302\_Sample\_011046841, Unigene43782\_Sample\_011046841, Unigene47967\_Sample\_011046841, Unigene51429\_Sample\_011046841, Unigene58394\_Sample\_011046841, Unigene15792\_Sample\_011046841, Unigene55286\_Sample\_011046841, Unigene421\_Sample\_011046841, Unigene25764\_Sample\_011046841, Unigene29318\_Sample\_011046841, Unigene31920\_Sample\_011046841, Unigene45798\_Sample\_011046841, Unigene47560\_Sample\_011046841, Unigene48237\_Sample\_011046841, Unigene48742\_Sample\_011046841, Unigene52499\_Sample\_011046841, Unigene56073\_Sample\_011046841, Unigene56139\_Sample\_011046841, Unigene16098\_Sample\_011046841, Unigene2110\_Sample\_011046841, Unigene8266\_Sample\_011046841, Unigene16055\_Sample\_011046841, Unigene21201\_Sample\_011046841, Unigene24508\_Sample\_011046841, Unigene38787\_Sample\_011046841, Unigene38948\_Sample\_011046841, Unigene53995\_Sample\_011046841, Unigene55041\_Sample\_011046841, Unigene60441\_Sample\_011046841, Unigene3490\_Sample\_011046841, Unigene12521\_Sample\_011046841, Unigene15705\_Sample\_011046841, Unigene23831\_Sample\_011046841, Unigene28288\_Sample\_011046841, Unigene43099\_Sample\_011046841, Unigene44838\_Sample\_011046841, Unigene47872\_Sample\_011046841, Unigene47910\_Sample\_011046841, Unigene55126\_Sample\_011046841, Unigene10863\_Sample\_011046841, Unigene17004\_Sample\_011046841, Unigene19947\_Sample\_011046841, Unigene47083\_Sample\_011046841, Unigene50989\_Sample\_011046841, Unigene12324\_Sample\_011046841, Unigene33510\_Sample\_011046841, Unigene43437\_Sample\_011046841, Unigene27363\_Sample\_011046841, Unigene51380\_Sample\_011046841, Unigene59362\_Sample\_011046841, Unigene44021\_Sample\_011046841, Unigene47487\_Sample\_011046841, Unigene50758\_Sample\_011046841, Unigene35082\_Sample\_011046841, Unigene40934\_Sample\_011046841, Unigene53061\_Sample\_011046841, Unigene43097\_Sample\_011046841, Unigene14248\_Sample\_011046841, Unigene46813\_Sample\_011046841, Unigene19789\_Sample\_011046841, Unigene29441\_Sample\_011046841, Unigene59655\_Sample\_011046841, Unigene14181\_Sample\_011046841, Unigene30474\_Sample\_011046841, Unigene7048\_Sample\_011046841, Unigene32977\_Sample\_011046841, Unigene39302\_Sample\_011046841 |
| 30 | Amyotrophic lateral sclerosis (ALS) Back to summary table | Unigene40774\_Sample\_011046841, Unigene45147\_Sample\_011046841, Unigene48524\_Sample\_011046841, Unigene48957\_Sample\_011046841, Unigene50829\_Sample\_011046841, Unigene51426\_Sample\_011046841, Unigene54246\_Sample\_011046841, Unigene56500\_Sample\_011046841, Unigene56818\_Sample\_011046841, Unigene57503\_Sample\_011046841, Unigene57609\_Sample\_011046841, Unigene57654\_Sample\_011046841, Unigene59600\_Sample\_011046841, Unigene60252\_Sample\_011046841, Unigene60833\_Sample\_011046841, Unigene60915\_Sample\_011046841, Unigene3223\_Sample\_011046841, Unigene6176\_Sample\_011046841, Unigene13419\_Sample\_011046841, Unigene13945\_Sample\_011046841, Unigene14467\_Sample\_011046841, Unigene21418\_Sample\_011046841, Unigene46219\_Sample\_011046841, Unigene50170\_Sample\_011046841, Unigene50963\_Sample\_011046841, Unigene53457\_Sample\_011046841, Unigene53922\_Sample\_011046841, Unigene54868\_Sample\_011046841, Unigene58611\_Sample\_011046841, Unigene60600\_Sample\_011046841, Unigene31749\_Sample\_011046841, Unigene52805\_Sample\_011046841, Unigene57124\_Sample\_011046841, Unigene58411\_Sample\_011046841, Unigene58557\_Sample\_011046841, Unigene60517\_Sample\_011046841, Unigene60913\_Sample\_011046841, Unigene18444\_Sample\_011046841, Unigene58099\_Sample\_011046841, Unigene60810\_Sample\_011046841, Unigene3905\_Sample\_011046841, Unigene52790\_Sample\_011046841, Unigene59421\_Sample\_011046841, Unigene30898\_Sample\_011046841, Unigene37886\_Sample\_011046841, Unigene50145\_Sample\_011046841, Unigene312\_Sample\_011046841, Unigene22110\_Sample\_011046841, Unigene12271\_Sample\_011046841, Unigene24650\_Sample\_011046841, Unigene37267\_Sample\_011046841, Unigene37448\_Sample\_011046841, Unigene40307\_Sample\_011046841, Unigene49595\_Sample\_011046841, Unigene53853\_Sample\_011046841, Unigene54134\_Sample\_011046841, Unigene1482\_Sample\_011046841, Unigene16014\_Sample\_011046841, Unigene21362\_Sample\_011046841, Unigene42580\_Sample\_011046841, Unigene47429\_Sample\_011046841, Unigene47691\_Sample\_011046841, Unigene49087\_Sample\_011046841, Unigene52413\_Sample\_011046841, Unigene54663\_Sample\_011046841, Unigene57956\_Sample\_011046841, Unigene56851\_Sample\_011046841, Unigene31264\_Sample\_011046841, Unigene53521\_Sample\_011046841, Unigene16442\_Sample\_011046841, Unigene30484\_Sample\_011046841, Unigene42281\_Sample\_011046841, Unigene46813\_Sample\_011046841, Unigene20433\_Sample\_011046841 |
| 31 | Non-small cell lung cancer Back to summary table | Unigene865\_Sample\_011046841, Unigene3199\_Sample\_011046841, Unigene4209\_Sample\_011046841, Unigene7143\_Sample\_011046841, Unigene7809\_Sample\_011046841, Unigene8429\_Sample\_011046841, Unigene8581\_Sample\_011046841, Unigene10459\_Sample\_011046841, Unigene10970\_Sample\_011046841, Unigene11367\_Sample\_011046841, Unigene13069\_Sample\_011046841, Unigene13683\_Sample\_011046841, Unigene40230\_Sample\_011046841, Unigene58460\_Sample\_011046841, Unigene58987\_Sample\_011046841, Unigene60173\_Sample\_011046841, Unigene60498\_Sample\_011046841, Unigene1414\_Sample\_011046841, Unigene3363\_Sample\_011046841, Unigene5744\_Sample\_011046841, Unigene9408\_Sample\_011046841, Unigene9867\_Sample\_011046841, Unigene12943\_Sample\_011046841, Unigene13766\_Sample\_011046841, Unigene13855\_Sample\_011046841, Unigene41574\_Sample\_011046841, Unigene47555\_Sample\_011046841, Unigene54900\_Sample\_011046841, Unigene56681\_Sample\_011046841, Unigene56798\_Sample\_011046841, Unigene57430\_Sample\_011046841, Unigene57566\_Sample\_011046841, Unigene58100\_Sample\_011046841, Unigene60427\_Sample\_011046841, Unigene11932\_Sample\_011046841, Unigene12975\_Sample\_011046841, Unigene23590\_Sample\_011046841, Unigene28658\_Sample\_011046841, Unigene40758\_Sample\_011046841, Unigene57775\_Sample\_011046841, Unigene60765\_Sample\_011046841, Unigene486\_Sample\_011046841, Unigene32964\_Sample\_011046841, Unigene56300\_Sample\_011046841, Unigene59061\_Sample\_011046841, Unigene60863\_Sample\_011046841, Unigene49525\_Sample\_011046841, Unigene1107\_Sample\_011046841, Unigene2942\_Sample\_011046841, Unigene9329\_Sample\_011046841, Unigene47967\_Sample\_011046841, Unigene29318\_Sample\_011046841, Unigene47054\_Sample\_011046841, Unigene51756\_Sample\_011046841, Unigene56139\_Sample\_011046841, Unigene16098\_Sample\_011046841, Unigene53995\_Sample\_011046841, Unigene12521\_Sample\_011046841, Unigene43099\_Sample\_011046841, Unigene47872\_Sample\_011046841, Unigene47910\_Sample\_011046841, Unigene50832\_Sample\_011046841, Unigene31969\_Sample\_011046841, Unigene28077\_Sample\_011046841, Unigene43718\_Sample\_011046841, Unigene32258\_Sample\_011046841, Unigene4308\_Sample\_011046841, Unigene46813\_Sample\_011046841, Unigene30474\_Sample\_011046841, Unigene26777\_Sample\_011046841 |
| 32 | Fc epsilon RI signaling pathway Back to summary table | Unigene679\_Sample\_011046841, Unigene865\_Sample\_011046841, Unigene4209\_Sample\_011046841, Unigene7143\_Sample\_011046841, Unigene9774\_Sample\_011046841, Unigene10459\_Sample\_011046841, Unigene11999\_Sample\_011046841, Unigene12527\_Sample\_011046841, Unigene13069\_Sample\_011046841, Unigene40774\_Sample\_011046841, Unigene43936\_Sample\_011046841, Unigene52144\_Sample\_011046841, Unigene58460\_Sample\_011046841, Unigene58987\_Sample\_011046841, Unigene60173\_Sample\_011046841, Unigene60462\_Sample\_011046841, Unigene60498\_Sample\_011046841, Unigene2509\_Sample\_011046841, Unigene5740\_Sample\_011046841, Unigene10198\_Sample\_011046841, Unigene10346\_Sample\_011046841, Unigene13419\_Sample\_011046841, Unigene13855\_Sample\_011046841, Unigene21418\_Sample\_011046841, Unigene41574\_Sample\_011046841, Unigene46219\_Sample\_011046841, Unigene47555\_Sample\_011046841, Unigene50170\_Sample\_011046841, Unigene53096\_Sample\_011046841, Unigene53264\_Sample\_011046841, Unigene54900\_Sample\_011046841, Unigene56681\_Sample\_011046841, Unigene57566\_Sample\_011046841, Unigene58100\_Sample\_011046841, Unigene58669\_Sample\_011046841, Unigene23590\_Sample\_011046841, Unigene28658\_Sample\_011046841, Unigene29578\_Sample\_011046841, Unigene40491\_Sample\_011046841, Unigene40758\_Sample\_011046841, Unigene60765\_Sample\_011046841, Unigene11547\_Sample\_011046841, Unigene30184\_Sample\_011046841, Unigene32964\_Sample\_011046841, Unigene56300\_Sample\_011046841, Unigene15\_Sample\_011046841, Unigene49525\_Sample\_011046841, Unigene59421\_Sample\_011046841, Unigene11231\_Sample\_011046841, Unigene60217\_Sample\_011046841, Unigene7042\_Sample\_011046841, Unigene11549\_Sample\_011046841, Unigene29318\_Sample\_011046841, Unigene47054\_Sample\_011046841, Unigene51756\_Sample\_011046841, Unigene57641\_Sample\_011046841, Unigene21362\_Sample\_011046841, Unigene42580\_Sample\_011046841, Unigene47910\_Sample\_011046841, Unigene50832\_Sample\_011046841, Unigene51777\_Sample\_011046841, Unigene26679\_Sample\_011046841, Unigene31264\_Sample\_011046841, Unigene31544\_Sample\_011046841, Unigene16442\_Sample\_011046841, Unigene30484\_Sample\_011046841, Unigene32258\_Sample\_011046841, Unigene48345\_Sample\_011046841, Unigene46813\_Sample\_011046841, Unigene30474\_Sample\_011046841 |
| 33 | Lysosome Back to summary table | Unigene7102\_Sample\_011046841, Unigene10152\_Sample\_011046841, Unigene10438\_Sample\_011046841, Unigene10881\_Sample\_011046841, Unigene12852\_Sample\_011046841, Unigene21912\_Sample\_011046841, Unigene24123\_Sample\_011046841, Unigene36781\_Sample\_011046841, Unigene40271\_Sample\_011046841, Unigene43500\_Sample\_011046841, Unigene44464\_Sample\_011046841, Unigene46525\_Sample\_011046841, Unigene47334\_Sample\_011046841, Unigene50998\_Sample\_011046841, Unigene51627\_Sample\_011046841, Unigene53054\_Sample\_011046841, Unigene53333\_Sample\_011046841, Unigene54102\_Sample\_011046841, Unigene55020\_Sample\_011046841, Unigene56375\_Sample\_011046841, Unigene57236\_Sample\_011046841, Unigene57281\_Sample\_011046841, Unigene57410\_Sample\_011046841, Unigene57629\_Sample\_011046841, Unigene57947\_Sample\_011046841, Unigene57973\_Sample\_011046841, Unigene58676\_Sample\_011046841, Unigene59273\_Sample\_011046841, Unigene59595\_Sample\_011046841, Unigene59622\_Sample\_011046841, Unigene59681\_Sample\_011046841, Unigene60466\_Sample\_011046841, Unigene60614\_Sample\_011046841, Unigene60652\_Sample\_011046841, Unigene2359\_Sample\_011046841, Unigene2987\_Sample\_011046841, Unigene3148\_Sample\_011046841, Unigene5005\_Sample\_011046841, Unigene7518\_Sample\_011046841, Unigene8721\_Sample\_011046841, Unigene9572\_Sample\_011046841, Unigene9674\_Sample\_011046841, Unigene12796\_Sample\_011046841, Unigene12801\_Sample\_011046841, Unigene13645\_Sample\_011046841, Unigene19030\_Sample\_011046841, Unigene22548\_Sample\_011046841, Unigene26120\_Sample\_011046841, Unigene33391\_Sample\_011046841, Unigene33662\_Sample\_011046841, Unigene37040\_Sample\_011046841, Unigene39295\_Sample\_011046841, Unigene43654\_Sample\_011046841, Unigene44425\_Sample\_011046841, Unigene46155\_Sample\_011046841, Unigene46243\_Sample\_011046841, Unigene47655\_Sample\_011046841, Unigene48578\_Sample\_011046841, Unigene49697\_Sample\_011046841, Unigene49894\_Sample\_011046841, Unigene50014\_Sample\_011046841, Unigene50585\_Sample\_011046841, Unigene52518\_Sample\_011046841, Unigene54425\_Sample\_011046841, Unigene55255\_Sample\_011046841, Unigene55494\_Sample\_011046841, Unigene55809\_Sample\_011046841, Unigene56094\_Sample\_011046841, Unigene57271\_Sample\_011046841, Unigene57313\_Sample\_011046841, Unigene57455\_Sample\_011046841, Unigene58593\_Sample\_011046841, Unigene58982\_Sample\_011046841, Unigene59127\_Sample\_011046841, Unigene59458\_Sample\_011046841, Unigene59629\_Sample\_011046841, Unigene60272\_Sample\_011046841, Unigene60446\_Sample\_011046841, Unigene60709\_Sample\_011046841, Unigene60729\_Sample\_011046841, Unigene60923\_Sample\_011046841, Unigene173\_Sample\_011046841, Unigene829\_Sample\_011046841, Unigene2189\_Sample\_011046841, Unigene2762\_Sample\_011046841, Unigene4137\_Sample\_011046841, Unigene9411\_Sample\_011046841, Unigene15650\_Sample\_011046841, Unigene24161\_Sample\_011046841, Unigene24214\_Sample\_011046841, Unigene30072\_Sample\_011046841, Unigene32313\_Sample\_011046841, Unigene41288\_Sample\_011046841, Unigene44848\_Sample\_011046841, Unigene51648\_Sample\_011046841, Unigene51663\_Sample\_011046841, Unigene53097\_Sample\_011046841, Unigene55491\_Sample\_011046841, Unigene57429\_Sample\_011046841, Unigene58011\_Sample\_011046841, Unigene12983\_Sample\_011046841, Unigene13591\_Sample\_011046841, Unigene43284\_Sample\_011046841, Unigene43713\_Sample\_011046841, Unigene44282\_Sample\_011046841, Unigene47129\_Sample\_011046841, Unigene51811\_Sample\_011046841, Unigene56027\_Sample\_011046841, Unigene24586\_Sample\_011046841, Unigene55257\_Sample\_011046841, Unigene18126\_Sample\_011046841, Unigene43986\_Sample\_011046841, Unigene48690\_Sample\_011046841, Unigene50302\_Sample\_011046841, Unigene58229\_Sample\_011046841, Unigene59886\_Sample\_011046841, Unigene475\_Sample\_011046841, Unigene18279\_Sample\_011046841, Unigene19179\_Sample\_011046841, Unigene22776\_Sample\_011046841, Unigene25397\_Sample\_011046841, Unigene52621\_Sample\_011046841, Unigene26183\_Sample\_011046841, Unigene29952\_Sample\_011046841, Unigene36131\_Sample\_011046841, Unigene46399\_Sample\_011046841, Unigene53498\_Sample\_011046841, Unigene52326\_Sample\_011046841, Unigene21190\_Sample\_011046841, Unigene32522\_Sample\_011046841, Unigene35030\_Sample\_011046841, Unigene36328\_Sample\_011046841, Unigene38235\_Sample\_011046841, Unigene39537\_Sample\_011046841, Unigene43749\_Sample\_011046841, Unigene45303\_Sample\_011046841, Unigene1439\_Sample\_011046841, Unigene25152\_Sample\_011046841, Unigene38547\_Sample\_011046841, Unigene43386\_Sample\_011046841, Unigene46799\_Sample\_011046841, Unigene48302\_Sample\_011046841, Unigene50296\_Sample\_011046841, Unigene52776\_Sample\_011046841, Unigene52997\_Sample\_011046841, Unigene53018\_Sample\_011046841, Unigene324\_Sample\_011046841, Unigene6762\_Sample\_011046841, Unigene31066\_Sample\_011046841, Unigene32945\_Sample\_011046841, Unigene40390\_Sample\_011046841, Unigene58030\_Sample\_011046841, Unigene58298\_Sample\_011046841, Unigene19051\_Sample\_011046841, Unigene55528\_Sample\_011046841, Unigene29188\_Sample\_011046841, Unigene23566\_Sample\_011046841, Unigene27689\_Sample\_011046841, Unigene45968\_Sample\_011046841, Unigene48293\_Sample\_011046841, Unigene25153\_Sample\_011046841, Unigene30989\_Sample\_011046841, Unigene40280\_Sample\_011046841, Unigene58257\_Sample\_011046841, Unigene26205\_Sample\_011046841, Unigene16995\_Sample\_011046841, Unigene48819\_Sample\_011046841 |
| 34 | Jak-STAT signaling pathway Back to summary table | Unigene865\_Sample\_011046841, Unigene4209\_Sample\_011046841, Unigene7143\_Sample\_011046841, Unigene8035\_Sample\_011046841, Unigene9668\_Sample\_011046841, Unigene9858\_Sample\_011046841, Unigene10459\_Sample\_011046841, Unigene10640\_Sample\_011046841, Unigene13069\_Sample\_011046841, Unigene13872\_Sample\_011046841, Unigene41694\_Sample\_011046841, Unigene45114\_Sample\_011046841, Unigene56295\_Sample\_011046841, Unigene58506\_Sample\_011046841, Unigene58987\_Sample\_011046841, Unigene59978\_Sample\_011046841, Unigene60173\_Sample\_011046841, Unigene60417\_Sample\_011046841, Unigene60498\_Sample\_011046841, Unigene60652\_Sample\_011046841, Unigene60685\_Sample\_011046841, Unigene60759\_Sample\_011046841, Unigene60826\_Sample\_011046841, Unigene3698\_Sample\_011046841, Unigene8641\_Sample\_011046841, Unigene13855\_Sample\_011046841, Unigene33667\_Sample\_011046841, Unigene35368\_Sample\_011046841, Unigene45858\_Sample\_011046841, Unigene50963\_Sample\_011046841, Unigene55696\_Sample\_011046841, Unigene56681\_Sample\_011046841, Unigene57566\_Sample\_011046841, Unigene57862\_Sample\_011046841, Unigene58100\_Sample\_011046841, Unigene23218\_Sample\_011046841, Unigene23590\_Sample\_011046841, Unigene31438\_Sample\_011046841, Unigene40723\_Sample\_011046841, Unigene40758\_Sample\_011046841, Unigene60765\_Sample\_011046841, Unigene13197\_Sample\_011046841, Unigene56300\_Sample\_011046841, Unigene49525\_Sample\_011046841, Unigene54521\_Sample\_011046841, Unigene54535\_Sample\_011046841, Unigene44628\_Sample\_011046841, Unigene36415\_Sample\_011046841, Unigene5263\_Sample\_011046841, Unigene41996\_Sample\_011046841, Unigene47054\_Sample\_011046841, Unigene51756\_Sample\_011046841, Unigene57006\_Sample\_011046841, Unigene57117\_Sample\_011046841, Unigene16836\_Sample\_011046841, Unigene39189\_Sample\_011046841, Unigene50832\_Sample\_011046841, Unigene54104\_Sample\_011046841, Unigene54519\_Sample\_011046841, Unigene59748\_Sample\_011046841, Unigene49698\_Sample\_011046841, Unigene28384\_Sample\_011046841, Unigene42712\_Sample\_011046841, Unigene32258\_Sample\_011046841, Unigene42358\_Sample\_011046841, Unigene17523\_Sample\_011046841, Unigene27675\_Sample\_011046841, Unigene45339\_Sample\_011046841 |
| 35 | B cell receptor signaling pathway Back to summary table | Unigene679\_Sample\_011046841, Unigene865\_Sample\_011046841, Unigene4209\_Sample\_011046841, Unigene7143\_Sample\_011046841, Unigene10459\_Sample\_011046841, Unigene13069\_Sample\_011046841, Unigene43936\_Sample\_011046841, Unigene48524\_Sample\_011046841, Unigene51426\_Sample\_011046841, Unigene57609\_Sample\_011046841, Unigene58460\_Sample\_011046841, Unigene58987\_Sample\_011046841, Unigene59022\_Sample\_011046841, Unigene60173\_Sample\_011046841, Unigene60252\_Sample\_011046841, Unigene60498\_Sample\_011046841, Unigene2987\_Sample\_011046841, Unigene7709\_Sample\_011046841, Unigene10198\_Sample\_011046841, Unigene13855\_Sample\_011046841, Unigene13945\_Sample\_011046841, Unigene21418\_Sample\_011046841, Unigene41574\_Sample\_011046841, Unigene42561\_Sample\_011046841, Unigene42820\_Sample\_011046841, Unigene46219\_Sample\_011046841, Unigene47555\_Sample\_011046841, Unigene53096\_Sample\_011046841, Unigene53264\_Sample\_011046841, Unigene53447\_Sample\_011046841, Unigene54900\_Sample\_011046841, Unigene56681\_Sample\_011046841, Unigene57566\_Sample\_011046841, Unigene58100\_Sample\_011046841, Unigene58611\_Sample\_011046841, Unigene60190\_Sample\_011046841, Unigene23218\_Sample\_011046841, Unigene23590\_Sample\_011046841, Unigene28658\_Sample\_011046841, Unigene29578\_Sample\_011046841, Unigene40491\_Sample\_011046841, Unigene40758\_Sample\_011046841, Unigene54574\_Sample\_011046841, Unigene59322\_Sample\_011046841, Unigene60765\_Sample\_011046841, Unigene11547\_Sample\_011046841, Unigene30184\_Sample\_011046841, Unigene32964\_Sample\_011046841, Unigene56300\_Sample\_011046841, Unigene49525\_Sample\_011046841, Unigene11549\_Sample\_011046841, Unigene29318\_Sample\_011046841, Unigene47054\_Sample\_011046841, Unigene51756\_Sample\_011046841, Unigene38235\_Sample\_011046841, Unigene42580\_Sample\_011046841, Unigene47429\_Sample\_011046841, Unigene47910\_Sample\_011046841, Unigene49087\_Sample\_011046841, Unigene50832\_Sample\_011046841, Unigene54663\_Sample\_011046841, Unigene51777\_Sample\_011046841, Unigene26679\_Sample\_011046841, Unigene32258\_Sample\_011046841, Unigene46813\_Sample\_011046841, Unigene30474\_Sample\_011046841 |
| 36 | Pancreatic cancer Back to summary table | Unigene3904\_Sample\_011046841, Unigene4209\_Sample\_011046841, Unigene5122\_Sample\_011046841, Unigene7809\_Sample\_011046841, Unigene8429\_Sample\_011046841, Unigene10459\_Sample\_011046841, Unigene10970\_Sample\_011046841, Unigene11367\_Sample\_011046841, Unigene13069\_Sample\_011046841, Unigene58460\_Sample\_011046841, Unigene60173\_Sample\_011046841, Unigene60498\_Sample\_011046841, Unigene1414\_Sample\_011046841, Unigene5747\_Sample\_011046841, Unigene8588\_Sample\_011046841, Unigene11922\_Sample\_011046841, Unigene12943\_Sample\_011046841, Unigene13873\_Sample\_011046841, Unigene21418\_Sample\_011046841, Unigene37278\_Sample\_011046841, Unigene41574\_Sample\_011046841, Unigene43409\_Sample\_011046841, Unigene45592\_Sample\_011046841, Unigene46219\_Sample\_011046841, Unigene47555\_Sample\_011046841, Unigene49399\_Sample\_011046841, Unigene50963\_Sample\_011046841, Unigene56798\_Sample\_011046841, Unigene57566\_Sample\_011046841, Unigene57614\_Sample\_011046841, Unigene58100\_Sample\_011046841, Unigene58564\_Sample\_011046841, Unigene60427\_Sample\_011046841, Unigene11932\_Sample\_011046841, Unigene23590\_Sample\_011046841, Unigene28658\_Sample\_011046841, Unigene55164\_Sample\_011046841, Unigene57775\_Sample\_011046841, Unigene30184\_Sample\_011046841, Unigene32964\_Sample\_011046841, Unigene56300\_Sample\_011046841, Unigene60863\_Sample\_011046841, Unigene49525\_Sample\_011046841, Unigene1107\_Sample\_011046841, Unigene32596\_Sample\_011046841, Unigene34657\_Sample\_011046841, Unigene47967\_Sample\_011046841, Unigene52611\_Sample\_011046841, Unigene60217\_Sample\_011046841, Unigene17370\_Sample\_011046841, Unigene29318\_Sample\_011046841, Unigene30153\_Sample\_011046841, Unigene51756\_Sample\_011046841, Unigene53995\_Sample\_011046841, Unigene42580\_Sample\_011046841, Unigene47910\_Sample\_011046841, Unigene50832\_Sample\_011046841, Unigene31969\_Sample\_011046841, Unigene14639\_Sample\_011046841, Unigene43718\_Sample\_011046841, Unigene48052\_Sample\_011046841, Unigene19937\_Sample\_011046841, Unigene46813\_Sample\_011046841, Unigene30474\_Sample\_011046841, Unigene19596\_Sample\_011046841, Unigene26777\_Sample\_011046841 |
| 37 | Neurotrophin signaling pathway Back to summary table | Unigene365\_Sample\_011046841, Unigene865\_Sample\_011046841, Unigene4209\_Sample\_011046841, Unigene5053\_Sample\_011046841, Unigene6130\_Sample\_011046841, Unigene6235\_Sample\_011046841, Unigene6324\_Sample\_011046841, Unigene7143\_Sample\_011046841, Unigene8035\_Sample\_011046841, Unigene9698\_Sample\_011046841, Unigene9858\_Sample\_011046841, Unigene10459\_Sample\_011046841, Unigene10970\_Sample\_011046841, Unigene11055\_Sample\_011046841, Unigene11073\_Sample\_011046841, Unigene12652\_Sample\_011046841, Unigene13069\_Sample\_011046841, Unigene41694\_Sample\_011046841, Unigene45346\_Sample\_011046841, Unigene47292\_Sample\_011046841, Unigene47924\_Sample\_011046841, Unigene53424\_Sample\_011046841, Unigene55404\_Sample\_011046841, Unigene55863\_Sample\_011046841, Unigene57298\_Sample\_011046841, Unigene58460\_Sample\_011046841, Unigene58987\_Sample\_011046841, Unigene59022\_Sample\_011046841, Unigene60173\_Sample\_011046841, Unigene60498\_Sample\_011046841, Unigene1345\_Sample\_011046841, Unigene3148\_Sample\_011046841, Unigene7034\_Sample\_011046841, Unigene9408\_Sample\_011046841, Unigene13332\_Sample\_011046841, Unigene13855\_Sample\_011046841, Unigene13877\_Sample\_011046841, Unigene21418\_Sample\_011046841, Unigene35368\_Sample\_011046841, Unigene39360\_Sample\_011046841, Unigene41574\_Sample\_011046841, Unigene41845\_Sample\_011046841, Unigene42561\_Sample\_011046841, Unigene42820\_Sample\_011046841, Unigene43409\_Sample\_011046841, Unigene45592\_Sample\_011046841, Unigene45858\_Sample\_011046841, Unigene46219\_Sample\_011046841, Unigene47555\_Sample\_011046841, Unigene50039\_Sample\_011046841, Unigene50170\_Sample\_011046841, Unigene50963\_Sample\_011046841, Unigene51503\_Sample\_011046841, Unigene51720\_Sample\_011046841, Unigene54900\_Sample\_011046841, Unigene54932\_Sample\_011046841, Unigene55975\_Sample\_011046841, Unigene56232\_Sample\_011046841, Unigene56681\_Sample\_011046841, Unigene57037\_Sample\_011046841, Unigene57566\_Sample\_011046841, Unigene58100\_Sample\_011046841, Unigene58209\_Sample\_011046841, Unigene58285\_Sample\_011046841, Unigene58669\_Sample\_011046841, Unigene59036\_Sample\_011046841, Unigene59403\_Sample\_011046841, Unigene60190\_Sample\_011046841, Unigene60600\_Sample\_011046841, Unigene60859\_Sample\_011046841, Unigene5972\_Sample\_011046841, Unigene13367\_Sample\_011046841, Unigene13920\_Sample\_011046841, Unigene18850\_Sample\_011046841, Unigene19453\_Sample\_011046841, Unigene23218\_Sample\_011046841, Unigene23590\_Sample\_011046841, Unigene23671\_Sample\_011046841, Unigene28658\_Sample\_011046841, Unigene31438\_Sample\_011046841, Unigene31749\_Sample\_011046841, Unigene36373\_Sample\_011046841, Unigene37205\_Sample\_011046841, Unigene40723\_Sample\_011046841, Unigene40758\_Sample\_011046841, Unigene44316\_Sample\_011046841, Unigene51775\_Sample\_011046841, Unigene54574\_Sample\_011046841, Unigene60765\_Sample\_011046841, Unigene19951\_Sample\_011046841, Unigene32964\_Sample\_011046841, Unigene42893\_Sample\_011046841, Unigene44532\_Sample\_011046841, Unigene47549\_Sample\_011046841, Unigene49211\_Sample\_011046841, Unigene49671\_Sample\_011046841, Unigene56300\_Sample\_011046841, Unigene57387\_Sample\_011046841, Unigene57675\_Sample\_011046841, Unigene58058\_Sample\_011046841, Unigene29346\_Sample\_011046841, Unigene36463\_Sample\_011046841, Unigene49525\_Sample\_011046841, Unigene52866\_Sample\_011046841, Unigene54521\_Sample\_011046841, Unigene57665\_Sample\_011046841, Unigene59077\_Sample\_011046841, Unigene59421\_Sample\_011046841, Unigene41032\_Sample\_011046841, Unigene47967\_Sample\_011046841, Unigene60217\_Sample\_011046841, Unigene7042\_Sample\_011046841, Unigene5263\_Sample\_011046841, Unigene10035\_Sample\_011046841, Unigene29318\_Sample\_011046841, Unigene41996\_Sample\_011046841, Unigene47054\_Sample\_011046841, Unigene51756\_Sample\_011046841, Unigene52499\_Sample\_011046841, Unigene56390\_Sample\_011046841, Unigene46589\_Sample\_011046841, Unigene26229\_Sample\_011046841, Unigene53995\_Sample\_011046841, Unigene57169\_Sample\_011046841, Unigene21362\_Sample\_011046841, Unigene36841\_Sample\_011046841, Unigene40166\_Sample\_011046841, Unigene42580\_Sample\_011046841, Unigene47686\_Sample\_011046841, Unigene47910\_Sample\_011046841, Unigene50832\_Sample\_011046841, Unigene52413\_Sample\_011046841, Unigene55459\_Sample\_011046841, Unigene57956\_Sample\_011046841, Unigene60132\_Sample\_011046841, Unigene56851\_Sample\_011046841, Unigene49698\_Sample\_011046841, Unigene60003\_Sample\_011046841, Unigene16539\_Sample\_011046841, Unigene31264\_Sample\_011046841, Unigene47789\_Sample\_011046841, Unigene48293\_Sample\_011046841, Unigene16442\_Sample\_011046841, Unigene30484\_Sample\_011046841, Unigene43367\_Sample\_011046841, Unigene44354\_Sample\_011046841, Unigene49261\_Sample\_011046841, Unigene19352\_Sample\_011046841, Unigene19937\_Sample\_011046841, Unigene21539\_Sample\_011046841, Unigene32258\_Sample\_011046841, Unigene42281\_Sample\_011046841, Unigene42358\_Sample\_011046841, Unigene46813\_Sample\_011046841, Unigene17523\_Sample\_011046841, Unigene17997\_Sample\_011046841, Unigene30474\_Sample\_011046841, Unigene48819\_Sample\_011046841, Unigene19596\_Sample\_011046841 |
| 38 | Type II diabetes mellitus Back to summary table | Unigene4209\_Sample\_011046841, Unigene9082\_Sample\_011046841, Unigene10459\_Sample\_011046841, Unigene11706\_Sample\_011046841, Unigene13069\_Sample\_011046841, Unigene13780\_Sample\_011046841, Unigene40350\_Sample\_011046841, Unigene56182\_Sample\_011046841, Unigene60173\_Sample\_011046841, Unigene60498\_Sample\_011046841, Unigene4892\_Sample\_011046841, Unigene5139\_Sample\_011046841, Unigene10189\_Sample\_011046841, Unigene11676\_Sample\_011046841, Unigene12336\_Sample\_011046841, Unigene26587\_Sample\_011046841, Unigene29480\_Sample\_011046841, Unigene41574\_Sample\_011046841, Unigene47555\_Sample\_011046841, Unigene49494\_Sample\_011046841, Unigene49640\_Sample\_011046841, Unigene50674\_Sample\_011046841, Unigene52526\_Sample\_011046841, Unigene57560\_Sample\_011046841, Unigene57566\_Sample\_011046841, Unigene59109\_Sample\_011046841, Unigene59822\_Sample\_011046841, Unigene60126\_Sample\_011046841, Unigene60532\_Sample\_011046841, Unigene2459\_Sample\_011046841, Unigene8083\_Sample\_011046841, Unigene28658\_Sample\_011046841, Unigene31181\_Sample\_011046841, Unigene31321\_Sample\_011046841, Unigene35808\_Sample\_011046841, Unigene51117\_Sample\_011046841, Unigene52294\_Sample\_011046841, Unigene56468\_Sample\_011046841, Unigene58321\_Sample\_011046841, Unigene59675\_Sample\_011046841, Unigene60606\_Sample\_011046841, Unigene32964\_Sample\_011046841, Unigene39766\_Sample\_011046841, Unigene45689\_Sample\_011046841, Unigene56300\_Sample\_011046841, Unigene56825\_Sample\_011046841, Unigene47976\_Sample\_011046841, Unigene29803\_Sample\_011046841, Unigene60217\_Sample\_011046841, Unigene18170\_Sample\_011046841, Unigene29462\_Sample\_011046841, Unigene55344\_Sample\_011046841, Unigene60248\_Sample\_011046841, Unigene34778\_Sample\_011046841, Unigene47910\_Sample\_011046841, Unigene50832\_Sample\_011046841, Unigene51582\_Sample\_011046841, Unigene20350\_Sample\_011046841, Unigene29944\_Sample\_011046841, Unigene54187\_Sample\_011046841, Unigene59655\_Sample\_011046841, Unigene29437\_Sample\_011046841, Unigene39361\_Sample\_011046841, Unigene30474\_Sample\_011046841 |
| 39 | Axon guidance Back to summary table | Unigene3904\_Sample\_011046841, Unigene6324\_Sample\_011046841, Unigene9774\_Sample\_011046841, Unigene9892\_Sample\_011046841, Unigene10388\_Sample\_011046841, Unigene10907\_Sample\_011046841, Unigene11073\_Sample\_011046841, Unigene12118\_Sample\_011046841, Unigene12652\_Sample\_011046841, Unigene13613\_Sample\_011046841, Unigene13823\_Sample\_011046841, Unigene13901\_Sample\_011046841, Unigene29813\_Sample\_011046841, Unigene39219\_Sample\_011046841, Unigene47292\_Sample\_011046841, Unigene48524\_Sample\_011046841, Unigene49240\_Sample\_011046841, Unigene49409\_Sample\_011046841, Unigene51426\_Sample\_011046841, Unigene52094\_Sample\_011046841, Unigene52144\_Sample\_011046841, Unigene53492\_Sample\_011046841, Unigene54982\_Sample\_011046841, Unigene57298\_Sample\_011046841, Unigene57407\_Sample\_011046841, Unigene57609\_Sample\_011046841, Unigene57921\_Sample\_011046841, Unigene59022\_Sample\_011046841, Unigene59559\_Sample\_011046841, Unigene60252\_Sample\_011046841, Unigene60530\_Sample\_011046841, Unigene60536\_Sample\_011046841, Unigene60576\_Sample\_011046841, Unigene60664\_Sample\_011046841, Unigene60808\_Sample\_011046841, Unigene1957\_Sample\_011046841, Unigene4993\_Sample\_011046841, Unigene6908\_Sample\_011046841, Unigene7709\_Sample\_011046841, Unigene8037\_Sample\_011046841, Unigene11680\_Sample\_011046841, Unigene11826\_Sample\_011046841, Unigene12096\_Sample\_011046841, Unigene13252\_Sample\_011046841, Unigene13332\_Sample\_011046841, Unigene13575\_Sample\_011046841, Unigene13945\_Sample\_011046841, Unigene13955\_Sample\_011046841, Unigene21418\_Sample\_011046841, Unigene32409\_Sample\_011046841, Unigene40209\_Sample\_011046841, Unigene41574\_Sample\_011046841, Unigene42561\_Sample\_011046841, Unigene42820\_Sample\_011046841, Unigene43409\_Sample\_011046841, Unigene45592\_Sample\_011046841, Unigene46219\_Sample\_011046841, Unigene46361\_Sample\_011046841, Unigene46625\_Sample\_011046841, Unigene46928\_Sample\_011046841, Unigene47555\_Sample\_011046841, Unigene48996\_Sample\_011046841, Unigene49315\_Sample\_011046841, Unigene49945\_Sample\_011046841, Unigene50163\_Sample\_011046841, Unigene50677\_Sample\_011046841, Unigene53389\_Sample\_011046841, Unigene54900\_Sample\_011046841, Unigene54932\_Sample\_011046841, Unigene58209\_Sample\_011046841, Unigene58611\_Sample\_011046841, Unigene58645\_Sample\_011046841, Unigene58915\_Sample\_011046841, Unigene59902\_Sample\_011046841, Unigene60190\_Sample\_011046841, Unigene60271\_Sample\_011046841, Unigene60406\_Sample\_011046841, Unigene60483\_Sample\_011046841, Unigene60522\_Sample\_011046841, Unigene60702\_Sample\_011046841, Unigene60831\_Sample\_011046841, Unigene60859\_Sample\_011046841, Unigene60890\_Sample\_011046841, Unigene879\_Sample\_011046841, Unigene3070\_Sample\_011046841, Unigene13811\_Sample\_011046841, Unigene19453\_Sample\_011046841, Unigene28658\_Sample\_011046841, Unigene41499\_Sample\_011046841, Unigene45885\_Sample\_011046841, Unigene46930\_Sample\_011046841, Unigene47887\_Sample\_011046841, Unigene48530\_Sample\_011046841, Unigene51330\_Sample\_011046841, Unigene52523\_Sample\_011046841, Unigene54574\_Sample\_011046841, Unigene55124\_Sample\_011046841, Unigene55644\_Sample\_011046841, Unigene56456\_Sample\_011046841, Unigene58468\_Sample\_011046841, Unigene59322\_Sample\_011046841, Unigene60365\_Sample\_011046841, Unigene6435\_Sample\_011046841, Unigene9254\_Sample\_011046841, Unigene9471\_Sample\_011046841, Unigene12448\_Sample\_011046841, Unigene19951\_Sample\_011046841, Unigene30184\_Sample\_011046841, Unigene32964\_Sample\_011046841, Unigene53642\_Sample\_011046841, Unigene57892\_Sample\_011046841, Unigene59711\_Sample\_011046841, Unigene60311\_Sample\_011046841, Unigene15\_Sample\_011046841, Unigene7293\_Sample\_011046841, Unigene29346\_Sample\_011046841, Unigene55486\_Sample\_011046841, Unigene2729\_Sample\_011046841, Unigene11231\_Sample\_011046841, Unigene38674\_Sample\_011046841, Unigene41032\_Sample\_011046841, Unigene43878\_Sample\_011046841, Unigene51301\_Sample\_011046841, Unigene52987\_Sample\_011046841, Unigene57641\_Sample\_011046841, Unigene11565\_Sample\_011046841, Unigene36385\_Sample\_011046841, Unigene36549\_Sample\_011046841, Unigene53352\_Sample\_011046841, Unigene12576\_Sample\_011046841, Unigene42580\_Sample\_011046841, Unigene47429\_Sample\_011046841, Unigene47686\_Sample\_011046841, Unigene47910\_Sample\_011046841, Unigene49087\_Sample\_011046841, Unigene54663\_Sample\_011046841, Unigene50989\_Sample\_011046841, Unigene54849\_Sample\_011046841, Unigene10948\_Sample\_011046841, Unigene20596\_Sample\_011046841, Unigene59882\_Sample\_011046841, Unigene17671\_Sample\_011046841, Unigene20770\_Sample\_011046841, Unigene21757\_Sample\_011046841, Unigene50713\_Sample\_011046841, Unigene41464\_Sample\_011046841, Unigene19937\_Sample\_011046841, Unigene16813\_Sample\_011046841, Unigene26184\_Sample\_011046841, Unigene22189\_Sample\_011046841, Unigene30474\_Sample\_011046841, Unigene19596\_Sample\_011046841 |
| 40 | Acute myeloid leukemia Back to summary table | Unigene865\_Sample\_011046841, Unigene4209\_Sample\_011046841, Unigene4963\_Sample\_011046841, Unigene7143\_Sample\_011046841, Unigene9082\_Sample\_011046841, Unigene10459\_Sample\_011046841, Unigene10970\_Sample\_011046841, Unigene11706\_Sample\_011046841, Unigene13069\_Sample\_011046841, Unigene37440\_Sample\_011046841, Unigene56886\_Sample\_011046841, Unigene58460\_Sample\_011046841, Unigene58987\_Sample\_011046841, Unigene60173\_Sample\_011046841, Unigene60498\_Sample\_011046841, Unigene4892\_Sample\_011046841, Unigene13855\_Sample\_011046841, Unigene40989\_Sample\_011046841, Unigene41574\_Sample\_011046841, Unigene47555\_Sample\_011046841, Unigene47936\_Sample\_011046841, Unigene49534\_Sample\_011046841, Unigene50674\_Sample\_011046841, Unigene54900\_Sample\_011046841, Unigene56232\_Sample\_011046841, Unigene56681\_Sample\_011046841, Unigene57566\_Sample\_011046841, Unigene58100\_Sample\_011046841, Unigene60787\_Sample\_011046841, Unigene13367\_Sample\_011046841, Unigene23590\_Sample\_011046841, Unigene28658\_Sample\_011046841, Unigene31181\_Sample\_011046841, Unigene40758\_Sample\_011046841, Unigene57649\_Sample\_011046841, Unigene58021\_Sample\_011046841, Unigene60765\_Sample\_011046841, Unigene13197\_Sample\_011046841, Unigene32964\_Sample\_011046841, Unigene56300\_Sample\_011046841, Unigene58058\_Sample\_011046841, Unigene49525\_Sample\_011046841, Unigene47967\_Sample\_011046841, Unigene29318\_Sample\_011046841, Unigene47054\_Sample\_011046841, Unigene51756\_Sample\_011046841, Unigene55344\_Sample\_011046841, Unigene53995\_Sample\_011046841, Unigene39020\_Sample\_011046841, Unigene47910\_Sample\_011046841, Unigene50832\_Sample\_011046841, Unigene60132\_Sample\_011046841, Unigene52183\_Sample\_011046841, Unigene41406\_Sample\_011046841, Unigene32258\_Sample\_011046841, Unigene4308\_Sample\_011046841, Unigene46813\_Sample\_011046841, Unigene39361\_Sample\_011046841, Unigene30474\_Sample\_011046841, Unigene18796\_Sample\_011046841 |
| 41 | Fc gamma R-mediated phagocytosis Back to summary table | Unigene679\_Sample\_011046841, Unigene3199\_Sample\_011046841, Unigene4209\_Sample\_011046841, Unigene7143\_Sample\_011046841, Unigene10459\_Sample\_011046841, Unigene13069\_Sample\_011046841, Unigene13901\_Sample\_011046841, Unigene25745\_Sample\_011046841, Unigene39219\_Sample\_011046841, Unigene40230\_Sample\_011046841, Unigene43936\_Sample\_011046841, Unigene45936\_Sample\_011046841, Unigene52094\_Sample\_011046841, Unigene52585\_Sample\_011046841, Unigene53730\_Sample\_011046841, Unigene56360\_Sample\_011046841, Unigene56914\_Sample\_011046841, Unigene57887\_Sample\_011046841, Unigene58447\_Sample\_011046841, Unigene58460\_Sample\_011046841, Unigene58817\_Sample\_011046841, Unigene59082\_Sample\_011046841, Unigene59559\_Sample\_011046841, Unigene59913\_Sample\_011046841, Unigene60015\_Sample\_011046841, Unigene60173\_Sample\_011046841, Unigene60498\_Sample\_011046841, Unigene60576\_Sample\_011046841, Unigene2442\_Sample\_011046841, Unigene2968\_Sample\_011046841, Unigene3363\_Sample\_011046841, Unigene9867\_Sample\_011046841, Unigene10198\_Sample\_011046841, Unigene13332\_Sample\_011046841, Unigene20439\_Sample\_011046841, Unigene21418\_Sample\_011046841, Unigene40942\_Sample\_011046841, Unigene41574\_Sample\_011046841, Unigene43409\_Sample\_011046841, Unigene45592\_Sample\_011046841, Unigene46219\_Sample\_011046841, Unigene47555\_Sample\_011046841, Unigene47936\_Sample\_011046841, Unigene49534\_Sample\_011046841, Unigene52044\_Sample\_011046841, Unigene52110\_Sample\_011046841, Unigene52146\_Sample\_011046841, Unigene52976\_Sample\_011046841, Unigene53096\_Sample\_011046841, Unigene53264\_Sample\_011046841, Unigene54286\_Sample\_011046841, Unigene55391\_Sample\_011046841, Unigene56232\_Sample\_011046841, Unigene56596\_Sample\_011046841, Unigene57375\_Sample\_011046841, Unigene57566\_Sample\_011046841, Unigene58042\_Sample\_011046841, Unigene58100\_Sample\_011046841, Unigene58409\_Sample\_011046841, Unigene58649\_Sample\_011046841, Unigene60140\_Sample\_011046841, Unigene60181\_Sample\_011046841, Unigene60430\_Sample\_011046841, Unigene60450\_Sample\_011046841, Unigene60787\_Sample\_011046841, Unigene11522\_Sample\_011046841, Unigene13367\_Sample\_011046841, Unigene14590\_Sample\_011046841, Unigene16993\_Sample\_011046841, Unigene21239\_Sample\_011046841, Unigene23590\_Sample\_011046841, Unigene28658\_Sample\_011046841, Unigene29578\_Sample\_011046841, Unigene30223\_Sample\_011046841, Unigene35664\_Sample\_011046841, Unigene40491\_Sample\_011046841, Unigene49909\_Sample\_011046841, Unigene56567\_Sample\_011046841, Unigene57890\_Sample\_011046841, Unigene58021\_Sample\_011046841, Unigene59434\_Sample\_011046841, Unigene59910\_Sample\_011046841, Unigene11547\_Sample\_011046841, Unigene30184\_Sample\_011046841, Unigene32964\_Sample\_011046841, Unigene44532\_Sample\_011046841, Unigene45808\_Sample\_011046841, Unigene46684\_Sample\_011046841, Unigene49878\_Sample\_011046841, Unigene53534\_Sample\_011046841, Unigene53642\_Sample\_011046841, Unigene56300\_Sample\_011046841, Unigene57529\_Sample\_011046841, Unigene58058\_Sample\_011046841, Unigene59711\_Sample\_011046841, Unigene42144\_Sample\_011046841, Unigene49525\_Sample\_011046841, Unigene55486\_Sample\_011046841, Unigene2942\_Sample\_011046841, Unigene3797\_Sample\_011046841, Unigene9329\_Sample\_011046841, Unigene51301\_Sample\_011046841, Unigene55389\_Sample\_011046841, Unigene55660\_Sample\_011046841, Unigene60792\_Sample\_011046841, Unigene11549\_Sample\_011046841, Unigene29318\_Sample\_011046841, Unigene51756\_Sample\_011046841, Unigene53019\_Sample\_011046841, Unigene54910\_Sample\_011046841, Unigene56139\_Sample\_011046841, Unigene16098\_Sample\_011046841, Unigene27839\_Sample\_011046841, Unigene50966\_Sample\_011046841, Unigene55520\_Sample\_011046841, Unigene12521\_Sample\_011046841, Unigene39020\_Sample\_011046841, Unigene41085\_Sample\_011046841, Unigene42047\_Sample\_011046841, Unigene42580\_Sample\_011046841, Unigene43099\_Sample\_011046841, Unigene47872\_Sample\_011046841, Unigene47910\_Sample\_011046841, Unigene50832\_Sample\_011046841, Unigene60132\_Sample\_011046841, Unigene51777\_Sample\_011046841, Unigene53609\_Sample\_011046841, Unigene59872\_Sample\_011046841, Unigene52183\_Sample\_011046841, Unigene19418\_Sample\_011046841, Unigene20596\_Sample\_011046841, Unigene41406\_Sample\_011046841, Unigene20770\_Sample\_011046841, Unigene50713\_Sample\_011046841, Unigene41464\_Sample\_011046841, Unigene19937\_Sample\_011046841, Unigene42026\_Sample\_011046841, Unigene4308\_Sample\_011046841, Unigene22728\_Sample\_011046841, Unigene38645\_Sample\_011046841, Unigene46813\_Sample\_011046841, Unigene50293\_Sample\_011046841, Unigene16813\_Sample\_011046841, Unigene23701\_Sample\_011046841, Unigene27784\_Sample\_011046841, Unigene49426\_Sample\_011046841, Unigene26184\_Sample\_011046841, Unigene25314\_Sample\_011046841, Unigene30474\_Sample\_011046841, Unigene18796\_Sample\_011046841, Unigene19596\_Sample\_011046841 |
| 42 | Biosynthesis of secondary metabolites (no map in kegg database) Back to summary table | Unigene1004\_Sample\_011046841, Unigene1049\_Sample\_011046841, Unigene1552\_Sample\_011046841, Unigene5169\_Sample\_011046841, Unigene7029\_Sample\_011046841, Unigene8282\_Sample\_011046841, Unigene8336\_Sample\_011046841, Unigene8871\_Sample\_011046841, Unigene11911\_Sample\_011046841, Unigene12076\_Sample\_011046841, Unigene12082\_Sample\_011046841, Unigene12420\_Sample\_011046841, Unigene12504\_Sample\_011046841, Unigene18832\_Sample\_011046841, Unigene19673\_Sample\_011046841, Unigene20907\_Sample\_011046841, Unigene21438\_Sample\_011046841, Unigene22611\_Sample\_011046841, Unigene24105\_Sample\_011046841, Unigene24171\_Sample\_011046841, Unigene28781\_Sample\_011046841, Unigene33884\_Sample\_011046841, Unigene36435\_Sample\_011046841, Unigene38930\_Sample\_011046841, Unigene39884\_Sample\_011046841, Unigene40350\_Sample\_011046841, Unigene40878\_Sample\_011046841, Unigene41250\_Sample\_011046841, Unigene42838\_Sample\_011046841, Unigene43544\_Sample\_011046841, Unigene43552\_Sample\_011046841, Unigene44603\_Sample\_011046841, Unigene45674\_Sample\_011046841, Unigene46439\_Sample\_011046841, Unigene47226\_Sample\_011046841, Unigene48532\_Sample\_011046841, Unigene50553\_Sample\_011046841, Unigene51029\_Sample\_011046841, Unigene51592\_Sample\_011046841, Unigene52799\_Sample\_011046841, Unigene53069\_Sample\_011046841, Unigene53862\_Sample\_011046841, Unigene54492\_Sample\_011046841, Unigene54550\_Sample\_011046841, Unigene54985\_Sample\_011046841, Unigene56182\_Sample\_011046841, Unigene57020\_Sample\_011046841, Unigene57575\_Sample\_011046841, Unigene58408\_Sample\_011046841, Unigene59158\_Sample\_011046841, Unigene59190\_Sample\_011046841, Unigene59828\_Sample\_011046841, Unigene59941\_Sample\_011046841, Unigene59965\_Sample\_011046841, Unigene59984\_Sample\_011046841, Unigene60317\_Sample\_011046841, Unigene60361\_Sample\_011046841, Unigene60431\_Sample\_011046841, Unigene60564\_Sample\_011046841, Unigene60677\_Sample\_011046841, Unigene60764\_Sample\_011046841, Unigene2385\_Sample\_011046841, Unigene5150\_Sample\_011046841, Unigene5329\_Sample\_011046841, Unigene6600\_Sample\_011046841, Unigene7507\_Sample\_011046841, Unigene7693\_Sample\_011046841, Unigene9455\_Sample\_011046841, Unigene11030\_Sample\_011046841, Unigene11283\_Sample\_011046841, Unigene13416\_Sample\_011046841, Unigene14236\_Sample\_011046841, Unigene15547\_Sample\_011046841, Unigene16727\_Sample\_011046841, Unigene20606\_Sample\_011046841, Unigene21594\_Sample\_011046841, Unigene22323\_Sample\_011046841, Unigene24754\_Sample\_011046841, Unigene27682\_Sample\_011046841, Unigene31490\_Sample\_011046841, Unigene31641\_Sample\_011046841, Unigene34145\_Sample\_011046841, Unigene34858\_Sample\_011046841, Unigene35134\_Sample\_011046841, Unigene35149\_Sample\_011046841, Unigene37679\_Sample\_011046841, Unigene38716\_Sample\_011046841, Unigene41171\_Sample\_011046841, Unigene42733\_Sample\_011046841, Unigene45531\_Sample\_011046841, Unigene45547\_Sample\_011046841, Unigene45816\_Sample\_011046841, Unigene46957\_Sample\_011046841, Unigene47414\_Sample\_011046841, Unigene48094\_Sample\_011046841, Unigene49640\_Sample\_011046841, Unigene49713\_Sample\_011046841, Unigene49729\_Sample\_011046841, Unigene49854\_Sample\_011046841, Unigene50242\_Sample\_011046841, Unigene50562\_Sample\_011046841, Unigene51598\_Sample\_011046841, Unigene52423\_Sample\_011046841, Unigene52526\_Sample\_011046841, Unigene52614\_Sample\_011046841, Unigene52769\_Sample\_011046841, Unigene52972\_Sample\_011046841, Unigene53002\_Sample\_011046841, Unigene53210\_Sample\_011046841, Unigene53305\_Sample\_011046841, Unigene53738\_Sample\_011046841, Unigene54081\_Sample\_011046841, Unigene54707\_Sample\_011046841, Unigene55065\_Sample\_011046841, Unigene55329\_Sample\_011046841, Unigene55357\_Sample\_011046841, Unigene55866\_Sample\_011046841, Unigene56100\_Sample\_011046841, Unigene56333\_Sample\_011046841, Unigene56529\_Sample\_011046841, Unigene57074\_Sample\_011046841, Unigene57300\_Sample\_011046841, Unigene57560\_Sample\_011046841, Unigene57608\_Sample\_011046841, Unigene58018\_Sample\_011046841, Unigene58309\_Sample\_011046841, Unigene58721\_Sample\_011046841, Unigene58765\_Sample\_011046841, Unigene59236\_Sample\_011046841, Unigene60126\_Sample\_011046841, Unigene60310\_Sample\_011046841, Unigene60574\_Sample\_011046841, Unigene60912\_Sample\_011046841, Unigene60\_Sample\_011046841, Unigene1477\_Sample\_011046841, Unigene5051\_Sample\_011046841, Unigene6222\_Sample\_011046841, Unigene8146\_Sample\_011046841, Unigene8728\_Sample\_011046841, Unigene12383\_Sample\_011046841, Unigene16357\_Sample\_011046841, Unigene18945\_Sample\_011046841, Unigene19169\_Sample\_011046841, Unigene20854\_Sample\_011046841, Unigene21061\_Sample\_011046841, Unigene21219\_Sample\_011046841, Unigene23752\_Sample\_011046841, Unigene25475\_Sample\_011046841, Unigene27725\_Sample\_011046841, Unigene28157\_Sample\_011046841, Unigene32280\_Sample\_011046841, Unigene33513\_Sample\_011046841, Unigene35210\_Sample\_011046841, Unigene35485\_Sample\_011046841, Unigene36181\_Sample\_011046841, Unigene37646\_Sample\_011046841, Unigene37947\_Sample\_011046841, Unigene44391\_Sample\_011046841, Unigene44834\_Sample\_011046841, Unigene47959\_Sample\_011046841, Unigene48956\_Sample\_011046841, Unigene50257\_Sample\_011046841, Unigene50269\_Sample\_011046841, Unigene51117\_Sample\_011046841, Unigene52177\_Sample\_011046841, Unigene52248\_Sample\_011046841, Unigene52685\_Sample\_011046841, Unigene53098\_Sample\_011046841, Unigene53462\_Sample\_011046841, Unigene53838\_Sample\_011046841, Unigene55314\_Sample\_011046841, Unigene55561\_Sample\_011046841, Unigene56468\_Sample\_011046841, Unigene59491\_Sample\_011046841, Unigene59675\_Sample\_011046841, Unigene60159\_Sample\_011046841, Unigene60448\_Sample\_011046841, Unigene60606\_Sample\_011046841, Unigene1966\_Sample\_011046841, Unigene5875\_Sample\_011046841, Unigene13080\_Sample\_011046841, Unigene13435\_Sample\_011046841, Unigene14511\_Sample\_011046841, Unigene15607\_Sample\_011046841, Unigene25319\_Sample\_011046841, Unigene26075\_Sample\_011046841, Unigene26417\_Sample\_011046841, Unigene27899\_Sample\_011046841, Unigene29870\_Sample\_011046841, Unigene39766\_Sample\_011046841, Unigene40503\_Sample\_011046841, Unigene45812\_Sample\_011046841, Unigene46297\_Sample\_011046841, Unigene47779\_Sample\_011046841, Unigene48529\_Sample\_011046841, Unigene51771\_Sample\_011046841, Unigene51823\_Sample\_011046841, Unigene56825\_Sample\_011046841, Unigene60089\_Sample\_011046841, Unigene30320\_Sample\_011046841, Unigene39617\_Sample\_011046841, Unigene47976\_Sample\_011046841, Unigene48698\_Sample\_011046841, Unigene52346\_Sample\_011046841, Unigene54528\_Sample\_011046841, Unigene54851\_Sample\_011046841, Unigene56353\_Sample\_011046841, Unigene57093\_Sample\_011046841, Unigene59545\_Sample\_011046841, Unigene7585\_Sample\_011046841, Unigene7978\_Sample\_011046841, Unigene16008\_Sample\_011046841, Unigene21456\_Sample\_011046841, Unigene21483\_Sample\_011046841, Unigene21731\_Sample\_011046841, Unigene25630\_Sample\_011046841, Unigene26689\_Sample\_011046841, Unigene29803\_Sample\_011046841, Unigene31394\_Sample\_011046841, Unigene34352\_Sample\_011046841, Unigene38650\_Sample\_011046841, Unigene42159\_Sample\_011046841, Unigene46888\_Sample\_011046841, Unigene50099\_Sample\_011046841, Unigene51946\_Sample\_011046841, Unigene54358\_Sample\_011046841, Unigene54624\_Sample\_011046841, Unigene57398\_Sample\_011046841, Unigene60523\_Sample\_011046841, Unigene18276\_Sample\_011046841, Unigene18805\_Sample\_011046841, Unigene21216\_Sample\_011046841, Unigene23199\_Sample\_011046841, Unigene41811\_Sample\_011046841, Unigene47162\_Sample\_011046841, Unigene49608\_Sample\_011046841, Unigene5530\_Sample\_011046841, Unigene17236\_Sample\_011046841, Unigene18442\_Sample\_011046841, Unigene23087\_Sample\_011046841, Unigene29271\_Sample\_011046841, Unigene32643\_Sample\_011046841, Unigene36472\_Sample\_011046841, Unigene48317\_Sample\_011046841, Unigene48730\_Sample\_011046841, Unigene48873\_Sample\_011046841, Unigene53698\_Sample\_011046841, Unigene60436\_Sample\_011046841, Unigene60458\_Sample\_011046841, Unigene8080\_Sample\_011046841, Unigene33744\_Sample\_011046841, Unigene36498\_Sample\_011046841, Unigene37427\_Sample\_011046841, Unigene41318\_Sample\_011046841, Unigene46059\_Sample\_011046841, Unigene19914\_Sample\_011046841, Unigene21863\_Sample\_011046841, Unigene34087\_Sample\_011046841, Unigene34550\_Sample\_011046841, Unigene34778\_Sample\_011046841, Unigene41423\_Sample\_011046841, Unigene47820\_Sample\_011046841, Unigene49106\_Sample\_011046841, Unigene49650\_Sample\_011046841, Unigene51420\_Sample\_011046841, Unigene53865\_Sample\_011046841, Unigene54935\_Sample\_011046841, Unigene12187\_Sample\_011046841, Unigene12706\_Sample\_011046841, Unigene15479\_Sample\_011046841, Unigene19013\_Sample\_011046841, Unigene19735\_Sample\_011046841, Unigene20440\_Sample\_011046841, Unigene22736\_Sample\_011046841, Unigene24516\_Sample\_011046841, Unigene27017\_Sample\_011046841, Unigene29438\_Sample\_011046841, Unigene37080\_Sample\_011046841, Unigene39532\_Sample\_011046841, Unigene40559\_Sample\_011046841, Unigene41140\_Sample\_011046841, Unigene42184\_Sample\_011046841, Unigene43528\_Sample\_011046841, Unigene43536\_Sample\_011046841, Unigene45837\_Sample\_011046841, Unigene48268\_Sample\_011046841, Unigene49204\_Sample\_011046841, Unigene49635\_Sample\_011046841, Unigene49966\_Sample\_011046841, Unigene51282\_Sample\_011046841, Unigene51677\_Sample\_011046841, Unigene52955\_Sample\_011046841, Unigene53290\_Sample\_011046841, Unigene54638\_Sample\_011046841, Unigene56571\_Sample\_011046841, Unigene22917\_Sample\_011046841, Unigene28279\_Sample\_011046841, Unigene37789\_Sample\_011046841, Unigene39842\_Sample\_011046841, Unigene49583\_Sample\_011046841, Unigene50185\_Sample\_011046841, Unigene51822\_Sample\_011046841, Unigene30309\_Sample\_011046841, Unigene31042\_Sample\_011046841, Unigene35592\_Sample\_011046841, Unigene41758\_Sample\_011046841, Unigene11166\_Sample\_011046841, Unigene16292\_Sample\_011046841, Unigene18813\_Sample\_011046841, Unigene40365\_Sample\_011046841, Unigene41284\_Sample\_011046841, Unigene48763\_Sample\_011046841, Unigene17285\_Sample\_011046841, Unigene20491\_Sample\_011046841, Unigene35830\_Sample\_011046841, Unigene43662\_Sample\_011046841, Unigene15416\_Sample\_011046841, Unigene18375\_Sample\_011046841, Unigene18613\_Sample\_011046841, Unigene21855\_Sample\_011046841, Unigene25961\_Sample\_011046841, Unigene31713\_Sample\_011046841, Unigene45758\_Sample\_011046841, Unigene33603\_Sample\_011046841, Unigene50987\_Sample\_011046841, Unigene54187\_Sample\_011046841, Unigene37642\_Sample\_011046841, Unigene40544\_Sample\_011046841, Unigene9865\_Sample\_011046841, Unigene22482\_Sample\_011046841, Unigene44109\_Sample\_011046841, Unigene16896\_Sample\_011046841, Unigene38646\_Sample\_011046841, Unigene41474\_Sample\_011046841, Unigene52018\_Sample\_011046841, Unigene21775\_Sample\_011046841, Unigene46950\_Sample\_011046841, Unigene24848\_Sample\_011046841, Unigene23065\_Sample\_011046841, Unigene21317\_Sample\_011046841 |
| 43 | p53 signaling pathway Back to summary table | Unigene8288\_Sample\_011046841, Unigene8429\_Sample\_011046841, Unigene9107\_Sample\_011046841, Unigene10427\_Sample\_011046841, Unigene10744\_Sample\_011046841, Unigene13706\_Sample\_011046841, Unigene54246\_Sample\_011046841, Unigene56818\_Sample\_011046841, Unigene57083\_Sample\_011046841, Unigene58784\_Sample\_011046841, Unigene59321\_Sample\_011046841, Unigene59600\_Sample\_011046841, Unigene6176\_Sample\_011046841, Unigene6531\_Sample\_011046841, Unigene6590\_Sample\_011046841, Unigene7869\_Sample\_011046841, Unigene10683\_Sample\_011046841, Unigene10878\_Sample\_011046841, Unigene11998\_Sample\_011046841, Unigene26220\_Sample\_011046841, Unigene35470\_Sample\_011046841, Unigene37760\_Sample\_011046841, Unigene41465\_Sample\_011046841, Unigene50674\_Sample\_011046841, Unigene54859\_Sample\_011046841, Unigene56025\_Sample\_011046841, Unigene56555\_Sample\_011046841, Unigene57215\_Sample\_011046841, Unigene57540\_Sample\_011046841, Unigene58692\_Sample\_011046841, Unigene60179\_Sample\_011046841, Unigene60427\_Sample\_011046841, Unigene12837\_Sample\_011046841, Unigene42510\_Sample\_011046841, Unigene44683\_Sample\_011046841, Unigene56062\_Sample\_011046841, Unigene58640\_Sample\_011046841, Unigene44121\_Sample\_011046841, Unigene57604\_Sample\_011046841, Unigene58099\_Sample\_011046841, Unigene42686\_Sample\_011046841, Unigene25087\_Sample\_011046841, Unigene312\_Sample\_011046841, Unigene56828\_Sample\_011046841, Unigene12271\_Sample\_011046841, Unigene41466\_Sample\_011046841, Unigene43515\_Sample\_011046841, Unigene37267\_Sample\_011046841, Unigene10605\_Sample\_011046841, Unigene54134\_Sample\_011046841, Unigene3810\_Sample\_011046841, Unigene7879\_Sample\_011046841, Unigene54104\_Sample\_011046841, Unigene31969\_Sample\_011046841, Unigene60003\_Sample\_011046841, Unigene58675\_Sample\_011046841, Unigene30349\_Sample\_011046841, Unigene20433\_Sample\_011046841 |
| 44 | RNA polymerase Back to summary table | Unigene12300\_Sample\_011046841, Unigene25547\_Sample\_011046841, Unigene32814\_Sample\_011046841, Unigene34404\_Sample\_011046841, Unigene44949\_Sample\_011046841, Unigene50631\_Sample\_011046841, Unigene54097\_Sample\_011046841, Unigene55779\_Sample\_011046841, Unigene56049\_Sample\_011046841, Unigene57770\_Sample\_011046841, Unigene60809\_Sample\_011046841, Unigene5336\_Sample\_011046841, Unigene27510\_Sample\_011046841, Unigene34859\_Sample\_011046841, Unigene39621\_Sample\_011046841, Unigene42399\_Sample\_011046841, Unigene47506\_Sample\_011046841, Unigene48881\_Sample\_011046841, Unigene48927\_Sample\_011046841, Unigene50108\_Sample\_011046841, Unigene52322\_Sample\_011046841, Unigene58152\_Sample\_011046841, Unigene59330\_Sample\_011046841, Unigene59634\_Sample\_011046841, Unigene60682\_Sample\_011046841, Unigene60900\_Sample\_011046841, Unigene5708\_Sample\_011046841, Unigene24615\_Sample\_011046841, Unigene26928\_Sample\_011046841, Unigene31723\_Sample\_011046841, Unigene48876\_Sample\_011046841, Unigene51481\_Sample\_011046841, Unigene51837\_Sample\_011046841, Unigene54368\_Sample\_011046841, Unigene55381\_Sample\_011046841, Unigene56098\_Sample\_011046841, Unigene60494\_Sample\_011046841, Unigene60847\_Sample\_011046841, Unigene2821\_Sample\_011046841, Unigene39016\_Sample\_011046841, Unigene42794\_Sample\_011046841, Unigene59492\_Sample\_011046841, Unigene50947\_Sample\_011046841, Unigene49168\_Sample\_011046841, Unigene37401\_Sample\_011046841, Unigene54790\_Sample\_011046841, Unigene57802\_Sample\_011046841, Unigene37087\_Sample\_011046841, Unigene53073\_Sample\_011046841, Unigene31129\_Sample\_011046841, Unigene43813\_Sample\_011046841, Unigene27719\_Sample\_011046841, Unigene35708\_Sample\_011046841, Unigene20294\_Sample\_011046841, Unigene31679\_Sample\_011046841, Unigene29124\_Sample\_011046841 |
| 45 | Dilated cardiomyopathy Back to summary table | Unigene290\_Sample\_011046841, Unigene863\_Sample\_011046841, Unigene1210\_Sample\_011046841, Unigene4208\_Sample\_011046841, Unigene6178\_Sample\_011046841, Unigene7142\_Sample\_011046841, Unigene8772\_Sample\_011046841, Unigene9237\_Sample\_011046841, Unigene10581\_Sample\_011046841, Unigene11938\_Sample\_011046841, Unigene13158\_Sample\_011046841, Unigene13481\_Sample\_011046841, Unigene13755\_Sample\_011046841, Unigene13789\_Sample\_011046841, Unigene22077\_Sample\_011046841, Unigene27010\_Sample\_011046841, Unigene43731\_Sample\_011046841, Unigene47875\_Sample\_011046841, Unigene48170\_Sample\_011046841, Unigene48836\_Sample\_011046841, Unigene53568\_Sample\_011046841, Unigene53928\_Sample\_011046841, Unigene55850\_Sample\_011046841, Unigene58388\_Sample\_011046841, Unigene58960\_Sample\_011046841, Unigene59870\_Sample\_011046841, Unigene60175\_Sample\_011046841, Unigene60485\_Sample\_011046841, Unigene60525\_Sample\_011046841, Unigene60612\_Sample\_011046841, Unigene60766\_Sample\_011046841, Unigene60789\_Sample\_011046841, Unigene60967\_Sample\_011046841, Unigene2588\_Sample\_011046841, Unigene2591\_Sample\_011046841, Unigene3890\_Sample\_011046841, Unigene4003\_Sample\_011046841, Unigene5744\_Sample\_011046841, Unigene7002\_Sample\_011046841, Unigene10189\_Sample\_011046841, Unigene11856\_Sample\_011046841, Unigene11912\_Sample\_011046841, Unigene13771\_Sample\_011046841, Unigene26850\_Sample\_011046841, Unigene38644\_Sample\_011046841, Unigene47384\_Sample\_011046841, Unigene50676\_Sample\_011046841, Unigene51782\_Sample\_011046841, Unigene52610\_Sample\_011046841, Unigene53584\_Sample\_011046841, Unigene53983\_Sample\_011046841, Unigene54140\_Sample\_011046841, Unigene58503\_Sample\_011046841, Unigene59003\_Sample\_011046841, Unigene59742\_Sample\_011046841, Unigene59983\_Sample\_011046841, Unigene60020\_Sample\_011046841, Unigene60195\_Sample\_011046841, Unigene60288\_Sample\_011046841, Unigene60554\_Sample\_011046841, Unigene60628\_Sample\_011046841, Unigene60806\_Sample\_011046841, Unigene60880\_Sample\_011046841, Unigene60947\_Sample\_011046841, Unigene7339\_Sample\_011046841, Unigene8083\_Sample\_011046841, Unigene13618\_Sample\_011046841, Unigene13958\_Sample\_011046841, Unigene18856\_Sample\_011046841, Unigene19285\_Sample\_011046841, Unigene20059\_Sample\_011046841, Unigene22974\_Sample\_011046841, Unigene35184\_Sample\_011046841, Unigene42781\_Sample\_011046841, Unigene57194\_Sample\_011046841, Unigene58818\_Sample\_011046841, Unigene60443\_Sample\_011046841, Unigene60813\_Sample\_011046841, Unigene8139\_Sample\_011046841, Unigene10486\_Sample\_011046841, Unigene13853\_Sample\_011046841, Unigene28860\_Sample\_011046841, Unigene48046\_Sample\_011046841, Unigene49117\_Sample\_011046841, Unigene57243\_Sample\_011046841, Unigene57267\_Sample\_011046841, Unigene58006\_Sample\_011046841, Unigene58882\_Sample\_011046841, Unigene59045\_Sample\_011046841, Unigene59998\_Sample\_011046841, Unigene10648\_Sample\_011046841, Unigene30190\_Sample\_011046841, Unigene58199\_Sample\_011046841, Unigene43782\_Sample\_011046841, Unigene51429\_Sample\_011046841, Unigene54809\_Sample\_011046841, Unigene48357\_Sample\_011046841, Unigene55740\_Sample\_011046841, Unigene58426\_Sample\_011046841, Unigene47560\_Sample\_011046841, Unigene48742\_Sample\_011046841, Unigene58176\_Sample\_011046841, Unigene23133\_Sample\_011046841, Unigene2110\_Sample\_011046841, Unigene7431\_Sample\_011046841, Unigene15451\_Sample\_011046841, Unigene16055\_Sample\_011046841, Unigene21201\_Sample\_011046841, Unigene26385\_Sample\_011046841, Unigene36385\_Sample\_011046841, Unigene38948\_Sample\_011046841, Unigene42173\_Sample\_011046841, Unigene43721\_Sample\_011046841, Unigene50460\_Sample\_011046841, Unigene52605\_Sample\_011046841, Unigene55041\_Sample\_011046841, Unigene55113\_Sample\_011046841, Unigene55234\_Sample\_011046841, Unigene60518\_Sample\_011046841, Unigene15705\_Sample\_011046841, Unigene23831\_Sample\_011046841, Unigene24961\_Sample\_011046841, Unigene26878\_Sample\_011046841, Unigene28288\_Sample\_011046841, Unigene32470\_Sample\_011046841, Unigene34824\_Sample\_011046841, Unigene48929\_Sample\_011046841, Unigene48977\_Sample\_011046841, Unigene57578\_Sample\_011046841, Unigene10863\_Sample\_011046841, Unigene46495\_Sample\_011046841, Unigene12324\_Sample\_011046841, Unigene19717\_Sample\_011046841, Unigene12560\_Sample\_011046841, Unigene44021\_Sample\_011046841, Unigene47487\_Sample\_011046841, Unigene15058\_Sample\_011046841, Unigene35082\_Sample\_011046841, Unigene37784\_Sample\_011046841, Unigene14248\_Sample\_011046841, Unigene48121\_Sample\_011046841, Unigene29441\_Sample\_011046841, Unigene59655\_Sample\_011046841, Unigene35586\_Sample\_011046841, Unigene7048\_Sample\_011046841 |
| 46 | Endocytosis Back to summary table | Unigene1090\_Sample\_011046841, Unigene1273\_Sample\_011046841, Unigene1382\_Sample\_011046841, Unigene2191\_Sample\_011046841, Unigene2363\_Sample\_011046841, Unigene3875\_Sample\_011046841, Unigene3904\_Sample\_011046841, Unigene5058\_Sample\_011046841, Unigene5109\_Sample\_011046841, Unigene5225\_Sample\_011046841, Unigene6601\_Sample\_011046841, Unigene7018\_Sample\_011046841, Unigene7261\_Sample\_011046841, Unigene7281\_Sample\_011046841, Unigene7809\_Sample\_011046841, Unigene7926\_Sample\_011046841, Unigene8486\_Sample\_011046841, Unigene9017\_Sample\_011046841, Unigene9535\_Sample\_011046841, Unigene10441\_Sample\_011046841, Unigene11367\_Sample\_011046841, Unigene11990\_Sample\_011046841, Unigene12266\_Sample\_011046841, Unigene12813\_Sample\_011046841, Unigene13109\_Sample\_011046841, Unigene13289\_Sample\_011046841, Unigene13466\_Sample\_011046841, Unigene13808\_Sample\_011046841, Unigene15082\_Sample\_011046841, Unigene19194\_Sample\_011046841, Unigene20175\_Sample\_011046841, Unigene21938\_Sample\_011046841, Unigene23925\_Sample\_011046841, Unigene25745\_Sample\_011046841, Unigene27522\_Sample\_011046841, Unigene27574\_Sample\_011046841, Unigene31954\_Sample\_011046841, Unigene32540\_Sample\_011046841, Unigene33040\_Sample\_011046841, Unigene39202\_Sample\_011046841, Unigene39476\_Sample\_011046841, Unigene40222\_Sample\_011046841, Unigene42267\_Sample\_011046841, Unigene42844\_Sample\_011046841, Unigene46741\_Sample\_011046841, Unigene46756\_Sample\_011046841, Unigene47219\_Sample\_011046841, Unigene47701\_Sample\_011046841, Unigene47994\_Sample\_011046841, Unigene49129\_Sample\_011046841, Unigene51416\_Sample\_011046841, Unigene52270\_Sample\_011046841, Unigene53368\_Sample\_011046841, Unigene54160\_Sample\_011046841, Unigene54218\_Sample\_011046841, Unigene55319\_Sample\_011046841, Unigene55610\_Sample\_011046841, Unigene55700\_Sample\_011046841, Unigene56327\_Sample\_011046841, Unigene56360\_Sample\_011046841, Unigene56887\_Sample\_011046841, Unigene57009\_Sample\_011046841, Unigene57298\_Sample\_011046841, Unigene57715\_Sample\_011046841, Unigene58567\_Sample\_011046841, Unigene58817\_Sample\_011046841, Unigene58889\_Sample\_011046841, Unigene59238\_Sample\_011046841, Unigene59457\_Sample\_011046841, Unigene59681\_Sample\_011046841, Unigene59705\_Sample\_011046841, Unigene59935\_Sample\_011046841, Unigene59978\_Sample\_011046841, Unigene60118\_Sample\_011046841, Unigene60267\_Sample\_011046841, Unigene60416\_Sample\_011046841, Unigene60510\_Sample\_011046841, Unigene60529\_Sample\_011046841, Unigene60652\_Sample\_011046841, Unigene60785\_Sample\_011046841, Unigene2\_Sample\_011046841, Unigene330\_Sample\_011046841, Unigene1404\_Sample\_011046841, Unigene1414\_Sample\_011046841, Unigene2899\_Sample\_011046841, Unigene2968\_Sample\_011046841, Unigene3353\_Sample\_011046841, Unigene3740\_Sample\_011046841, Unigene4361\_Sample\_011046841, Unigene4630\_Sample\_011046841, Unigene4917\_Sample\_011046841, Unigene4949\_Sample\_011046841, Unigene5623\_Sample\_011046841, Unigene5744\_Sample\_011046841, Unigene5747\_Sample\_011046841, Unigene6233\_Sample\_011046841, Unigene6977\_Sample\_011046841, Unigene7238\_Sample\_011046841, Unigene7462\_Sample\_011046841, Unigene8569\_Sample\_011046841, Unigene8818\_Sample\_011046841, Unigene8987\_Sample\_011046841, Unigene9354\_Sample\_011046841, Unigene9867\_Sample\_011046841, Unigene10260\_Sample\_011046841, Unigene10544\_Sample\_011046841, Unigene11464\_Sample\_011046841, Unigene11676\_Sample\_011046841, Unigene11772\_Sample\_011046841, Unigene12782\_Sample\_011046841, Unigene12943\_Sample\_011046841, Unigene13113\_Sample\_011046841, Unigene13131\_Sample\_011046841, Unigene13667\_Sample\_011046841, Unigene13714\_Sample\_011046841, Unigene13745\_Sample\_011046841, Unigene13884\_Sample\_011046841, Unigene17290\_Sample\_011046841, Unigene17359\_Sample\_011046841, Unigene17658\_Sample\_011046841, Unigene19044\_Sample\_011046841, Unigene20312\_Sample\_011046841, Unigene20439\_Sample\_011046841, Unigene21418\_Sample\_011046841, Unigene21528\_Sample\_011046841, Unigene21677\_Sample\_011046841, Unigene26403\_Sample\_011046841, Unigene27678\_Sample\_011046841, Unigene27795\_Sample\_011046841, Unigene27973\_Sample\_011046841, Unigene29038\_Sample\_011046841, Unigene31910\_Sample\_011046841, Unigene34259\_Sample\_011046841, Unigene34292\_Sample\_011046841, Unigene36178\_Sample\_011046841, Unigene36311\_Sample\_011046841, Unigene37278\_Sample\_011046841, Unigene37596\_Sample\_011046841, Unigene37984\_Sample\_011046841, Unigene39295\_Sample\_011046841, Unigene39647\_Sample\_011046841, Unigene39667\_Sample\_011046841, Unigene40261\_Sample\_011046841, Unigene40942\_Sample\_011046841, Unigene43096\_Sample\_011046841, Unigene43409\_Sample\_011046841, Unigene44162\_Sample\_011046841, Unigene44906\_Sample\_011046841, Unigene45001\_Sample\_011046841, Unigene45568\_Sample\_011046841, Unigene45592\_Sample\_011046841, Unigene46219\_Sample\_011046841, Unigene46410\_Sample\_011046841, Unigene47213\_Sample\_011046841, Unigene49399\_Sample\_011046841, Unigene49494\_Sample\_011046841, Unigene50131\_Sample\_011046841, Unigene51100\_Sample\_011046841, Unigene52110\_Sample\_011046841, Unigene52562\_Sample\_011046841, Unigene52815\_Sample\_011046841, Unigene53571\_Sample\_011046841, Unigene54286\_Sample\_011046841, Unigene54397\_Sample\_011046841, Unigene54556\_Sample\_011046841, Unigene54900\_Sample\_011046841, Unigene54923\_Sample\_011046841, Unigene55205\_Sample\_011046841, Unigene55391\_Sample\_011046841, Unigene55755\_Sample\_011046841, Unigene56271\_Sample\_011046841, Unigene56798\_Sample\_011046841, Unigene56836\_Sample\_011046841, Unigene56930\_Sample\_011046841, Unigene57072\_Sample\_011046841, Unigene57313\_Sample\_011046841, Unigene57375\_Sample\_011046841, Unigene57595\_Sample\_011046841, Unigene58042\_Sample\_011046841, Unigene58409\_Sample\_011046841, Unigene58564\_Sample\_011046841, Unigene58593\_Sample\_011046841, Unigene58649\_Sample\_011046841, Unigene58651\_Sample\_011046841, Unigene58821\_Sample\_011046841, Unigene59109\_Sample\_011046841, Unigene59395\_Sample\_011046841, Unigene59629\_Sample\_011046841, Unigene59879\_Sample\_011046841, Unigene59905\_Sample\_011046841, Unigene60140\_Sample\_011046841, Unigene60181\_Sample\_011046841, Unigene60306\_Sample\_011046841, Unigene60430\_Sample\_011046841, Unigene60450\_Sample\_011046841, Unigene60480\_Sample\_011046841, Unigene60503\_Sample\_011046841, Unigene60552\_Sample\_011046841, Unigene60566\_Sample\_011046841, Unigene60642\_Sample\_011046841, Unigene60659\_Sample\_011046841, Unigene60872\_Sample\_011046841, Unigene60889\_Sample\_011046841, Unigene60931\_Sample\_011046841, Unigene249\_Sample\_011046841, Unigene6000\_Sample\_011046841, Unigene9295\_Sample\_011046841, Unigene9784\_Sample\_011046841, Unigene10135\_Sample\_011046841, Unigene11522\_Sample\_011046841, Unigene11932\_Sample\_011046841, Unigene12789\_Sample\_011046841, Unigene12976\_Sample\_011046841, Unigene13822\_Sample\_011046841, Unigene14590\_Sample\_011046841, Unigene17957\_Sample\_011046841, Unigene18447\_Sample\_011046841, Unigene19923\_Sample\_011046841, Unigene20269\_Sample\_011046841, Unigene20724\_Sample\_011046841, Unigene22843\_Sample\_011046841, Unigene23713\_Sample\_011046841, Unigene27366\_Sample\_011046841, Unigene33618\_Sample\_011046841, Unigene35664\_Sample\_011046841, Unigene42151\_Sample\_011046841, Unigene42207\_Sample\_011046841, Unigene42922\_Sample\_011046841, Unigene44059\_Sample\_011046841, Unigene44187\_Sample\_011046841, Unigene47516\_Sample\_011046841, Unigene52294\_Sample\_011046841, Unigene52984\_Sample\_011046841, Unigene53049\_Sample\_011046841, Unigene53128\_Sample\_011046841, Unigene56118\_Sample\_011046841, Unigene56588\_Sample\_011046841, Unigene57727\_Sample\_011046841, Unigene57775\_Sample\_011046841, Unigene58040\_Sample\_011046841, Unigene58128\_Sample\_011046841, Unigene58321\_Sample\_011046841, Unigene58363\_Sample\_011046841, Unigene58479\_Sample\_011046841, Unigene59434\_Sample\_011046841, Unigene59581\_Sample\_011046841, Unigene59784\_Sample\_011046841, Unigene59910\_Sample\_011046841, Unigene60321\_Sample\_011046841, Unigene60324\_Sample\_011046841, Unigene60391\_Sample\_011046841, Unigene60751\_Sample\_011046841, Unigene4359\_Sample\_011046841, Unigene19951\_Sample\_011046841, Unigene21122\_Sample\_011046841, Unigene29101\_Sample\_011046841, Unigene33587\_Sample\_011046841, Unigene39377\_Sample\_011046841, Unigene43713\_Sample\_011046841, Unigene45808\_Sample\_011046841, Unigene48461\_Sample\_011046841, Unigene49359\_Sample\_011046841, Unigene50477\_Sample\_011046841, Unigene51024\_Sample\_011046841, Unigene52947\_Sample\_011046841, Unigene54111\_Sample\_011046841, Unigene54628\_Sample\_011046841, Unigene56081\_Sample\_011046841, Unigene56344\_Sample\_011046841, Unigene57716\_Sample\_011046841, Unigene60042\_Sample\_011046841, Unigene60735\_Sample\_011046841, Unigene60863\_Sample\_011046841, Unigene4163\_Sample\_011046841, Unigene9703\_Sample\_011046841, Unigene25753\_Sample\_011046841, Unigene29346\_Sample\_011046841, Unigene39087\_Sample\_011046841, Unigene43369\_Sample\_011046841, Unigene48764\_Sample\_011046841, Unigene53377\_Sample\_011046841, Unigene78\_Sample\_011046841, Unigene1107\_Sample\_011046841, Unigene3075\_Sample\_011046841, Unigene3388\_Sample\_011046841, Unigene20459\_Sample\_011046841, Unigene26084\_Sample\_011046841, Unigene34160\_Sample\_011046841, Unigene34225\_Sample\_011046841, Unigene44566\_Sample\_011046841, Unigene45458\_Sample\_011046841, Unigene48625\_Sample\_011046841, Unigene56665\_Sample\_011046841, Unigene57398\_Sample\_011046841, Unigene5783\_Sample\_011046841, Unigene12833\_Sample\_011046841, Unigene16725\_Sample\_011046841, Unigene32883\_Sample\_011046841, Unigene36415\_Sample\_011046841, Unigene52621\_Sample\_011046841, Unigene55660\_Sample\_011046841, Unigene59292\_Sample\_011046841, Unigene1549\_Sample\_011046841, Unigene7598\_Sample\_011046841, Unigene7997\_Sample\_011046841, Unigene11333\_Sample\_011046841, Unigene17668\_Sample\_011046841, Unigene18170\_Sample\_011046841, Unigene30153\_Sample\_011046841, Unigene33050\_Sample\_011046841, Unigene46875\_Sample\_011046841, Unigene50263\_Sample\_011046841, Unigene54910\_Sample\_011046841, Unigene57006\_Sample\_011046841, Unigene57641\_Sample\_011046841, Unigene59221\_Sample\_011046841, Unigene60248\_Sample\_011046841, Unigene54\_Sample\_011046841, Unigene3488\_Sample\_011046841, Unigene19355\_Sample\_011046841, Unigene19413\_Sample\_011046841, Unigene38907\_Sample\_011046841, Unigene47370\_Sample\_011046841, Unigene50197\_Sample\_011046841, Unigene58517\_Sample\_011046841, Unigene4143\_Sample\_011046841, Unigene6398\_Sample\_011046841, Unigene13879\_Sample\_011046841, Unigene17340\_Sample\_011046841, Unigene18417\_Sample\_011046841, Unigene27839\_Sample\_011046841, Unigene29330\_Sample\_011046841, Unigene31216\_Sample\_011046841, Unigene35030\_Sample\_011046841, Unigene39189\_Sample\_011046841, Unigene44199\_Sample\_011046841, Unigene48005\_Sample\_011046841, Unigene52667\_Sample\_011046841, Unigene55520\_Sample\_011046841, Unigene56734\_Sample\_011046841, Unigene57797\_Sample\_011046841, Unigene60156\_Sample\_011046841, Unigene224\_Sample\_011046841, Unigene383\_Sample\_011046841, Unigene704\_Sample\_011046841, Unigene1750\_Sample\_011046841, Unigene2121\_Sample\_011046841, Unigene14570\_Sample\_011046841, Unigene16822\_Sample\_011046841, Unigene18509\_Sample\_011046841, Unigene26481\_Sample\_011046841, Unigene31319\_Sample\_011046841, Unigene37707\_Sample\_011046841, Unigene38007\_Sample\_011046841, Unigene38167\_Sample\_011046841, Unigene38635\_Sample\_011046841, Unigene39983\_Sample\_011046841, Unigene42092\_Sample\_011046841, Unigene44025\_Sample\_011046841, Unigene45634\_Sample\_011046841, Unigene52776\_Sample\_011046841, Unigene54171\_Sample\_011046841, Unigene11759\_Sample\_011046841, Unigene40390\_Sample\_011046841, Unigene49571\_Sample\_011046841, Unigene51582\_Sample\_011046841, Unigene52883\_Sample\_011046841, Unigene53609\_Sample\_011046841, Unigene57344\_Sample\_011046841, Unigene59872\_Sample\_011046841, Unigene14639\_Sample\_011046841, Unigene35054\_Sample\_011046841, Unigene8133\_Sample\_011046841, Unigene13220\_Sample\_011046841, Unigene17371\_Sample\_011046841, Unigene39434\_Sample\_011046841, Unigene19418\_Sample\_011046841, Unigene20434\_Sample\_011046841, Unigene28937\_Sample\_011046841, Unigene34543\_Sample\_011046841, Unigene43718\_Sample\_011046841, Unigene49182\_Sample\_011046841, Unigene55112\_Sample\_011046841, Unigene16440\_Sample\_011046841, Unigene24899\_Sample\_011046841, Unigene28615\_Sample\_011046841, Unigene42218\_Sample\_011046841, Unigene45675\_Sample\_011046841, Unigene52524\_Sample\_011046841, Unigene30989\_Sample\_011046841, Unigene42014\_Sample\_011046841, Unigene43596\_Sample\_011046841, Unigene9941\_Sample\_011046841, Unigene19937\_Sample\_011046841, Unigene25029\_Sample\_011046841, Unigene36252\_Sample\_011046841, Unigene42026\_Sample\_011046841, Unigene51422\_Sample\_011046841, Unigene4308\_Sample\_011046841, Unigene9811\_Sample\_011046841, Unigene22357\_Sample\_011046841, Unigene24776\_Sample\_011046841, Unigene34359\_Sample\_011046841, Unigene1780\_Sample\_011046841, Unigene22728\_Sample\_011046841, Unigene3945\_Sample\_011046841, Unigene21561\_Sample\_011046841, Unigene50475\_Sample\_011046841, Unigene17067\_Sample\_011046841, Unigene32716\_Sample\_011046841, Unigene58020\_Sample\_011046841, Unigene23393\_Sample\_011046841, Unigene27784\_Sample\_011046841, Unigene29131\_Sample\_011046841, Unigene49426\_Sample\_011046841, Unigene3856\_Sample\_011046841, Unigene25314\_Sample\_011046841, Unigene27675\_Sample\_011046841, Unigene45339\_Sample\_011046841, Unigene50816\_Sample\_011046841, Unigene6540\_Sample\_011046841, Unigene37808\_Sample\_011046841, Unigene19596\_Sample\_011046841, Unigene26777\_Sample\_011046841 |
| 47 | Oocyte meiosis Back to summary table | Unigene1437\_Sample\_011046841, Unigene2686\_Sample\_011046841, Unigene5402\_Sample\_011046841, Unigene5622\_Sample\_011046841, Unigene8288\_Sample\_011046841, Unigene9876\_Sample\_011046841, Unigene12306\_Sample\_011046841, Unigene12594\_Sample\_011046841, Unigene13481\_Sample\_011046841, Unigene13789\_Sample\_011046841, Unigene15356\_Sample\_011046841, Unigene19684\_Sample\_011046841, Unigene27324\_Sample\_011046841, Unigene28326\_Sample\_011046841, Unigene29183\_Sample\_011046841, Unigene38234\_Sample\_011046841, Unigene39875\_Sample\_011046841, Unigene40567\_Sample\_011046841, Unigene43331\_Sample\_011046841, Unigene45022\_Sample\_011046841, Unigene47924\_Sample\_011046841, Unigene48524\_Sample\_011046841, Unigene49649\_Sample\_011046841, Unigene51307\_Sample\_011046841, Unigene51426\_Sample\_011046841, Unigene51697\_Sample\_011046841, Unigene52432\_Sample\_011046841, Unigene53888\_Sample\_011046841, Unigene55744\_Sample\_011046841, Unigene56389\_Sample\_011046841, Unigene56719\_Sample\_011046841, Unigene57609\_Sample\_011046841, Unigene58300\_Sample\_011046841, Unigene58460\_Sample\_011046841, Unigene59705\_Sample\_011046841, Unigene60252\_Sample\_011046841, Unigene60372\_Sample\_011046841, Unigene60755\_Sample\_011046841, Unigene60789\_Sample\_011046841, Unigene1345\_Sample\_011046841, Unigene3202\_Sample\_011046841, Unigene5744\_Sample\_011046841, Unigene6531\_Sample\_011046841, Unigene10325\_Sample\_011046841, Unigene11148\_Sample\_011046841, Unigene11676\_Sample\_011046841, Unigene12824\_Sample\_011046841, Unigene13332\_Sample\_011046841, Unigene13494\_Sample\_011046841, Unigene13825\_Sample\_011046841, Unigene13945\_Sample\_011046841, Unigene19273\_Sample\_011046841, Unigene23312\_Sample\_011046841, Unigene25098\_Sample\_011046841, Unigene26055\_Sample\_011046841, Unigene27694\_Sample\_011046841, Unigene30076\_Sample\_011046841, Unigene30495\_Sample\_011046841, Unigene35470\_Sample\_011046841, Unigene41373\_Sample\_011046841, Unigene41574\_Sample\_011046841, Unigene41845\_Sample\_011046841, Unigene42106\_Sample\_011046841, Unigene43448\_Sample\_011046841, Unigene46763\_Sample\_011046841, Unigene47555\_Sample\_011046841, Unigene48020\_Sample\_011046841, Unigene48342\_Sample\_011046841, Unigene49320\_Sample\_011046841, Unigene49494\_Sample\_011046841, Unigene49615\_Sample\_011046841, Unigene50039\_Sample\_011046841, Unigene52184\_Sample\_011046841, Unigene52869\_Sample\_011046841, Unigene54454\_Sample\_011046841, Unigene56010\_Sample\_011046841, Unigene56232\_Sample\_011046841, Unigene56663\_Sample\_011046841, Unigene56732\_Sample\_011046841, Unigene57390\_Sample\_011046841, Unigene57835\_Sample\_011046841, Unigene58169\_Sample\_011046841, Unigene58285\_Sample\_011046841, Unigene58611\_Sample\_011046841, Unigene59036\_Sample\_011046841, Unigene59109\_Sample\_011046841, Unigene59696\_Sample\_011046841, Unigene59742\_Sample\_011046841, Unigene60006\_Sample\_011046841, Unigene60427\_Sample\_011046841, Unigene60552\_Sample\_011046841, Unigene60880\_Sample\_011046841, Unigene2066\_Sample\_011046841, Unigene2213\_Sample\_011046841, Unigene3491\_Sample\_011046841, Unigene8828\_Sample\_011046841, Unigene12837\_Sample\_011046841, Unigene13367\_Sample\_011046841, Unigene18850\_Sample\_011046841, Unigene19343\_Sample\_011046841, Unigene19797\_Sample\_011046841, Unigene20297\_Sample\_011046841, Unigene20484\_Sample\_011046841, Unigene21655\_Sample\_011046841, Unigene23671\_Sample\_011046841, Unigene23751\_Sample\_011046841, Unigene25812\_Sample\_011046841, Unigene27440\_Sample\_011046841, Unigene28658\_Sample\_011046841, Unigene30120\_Sample\_011046841, Unigene32413\_Sample\_011046841, Unigene34582\_Sample\_011046841, Unigene42721\_Sample\_011046841, Unigene44316\_Sample\_011046841, Unigene44683\_Sample\_011046841, Unigene45662\_Sample\_011046841, Unigene48072\_Sample\_011046841, Unigene51775\_Sample\_011046841, Unigene51803\_Sample\_011046841, Unigene52294\_Sample\_011046841, Unigene53168\_Sample\_011046841, Unigene54593\_Sample\_011046841, Unigene55183\_Sample\_011046841, Unigene55504\_Sample\_011046841, Unigene56062\_Sample\_011046841, Unigene57145\_Sample\_011046841, Unigene58321\_Sample\_011046841, Unigene60347\_Sample\_011046841, Unigene5335\_Sample\_011046841, Unigene8139\_Sample\_011046841, Unigene27828\_Sample\_011046841, Unigene32964\_Sample\_011046841, Unigene35757\_Sample\_011046841, Unigene35917\_Sample\_011046841, Unigene49211\_Sample\_011046841, Unigene49671\_Sample\_011046841, Unigene50901\_Sample\_011046841, Unigene54240\_Sample\_011046841, Unigene55461\_Sample\_011046841, Unigene57387\_Sample\_011046841, Unigene57675\_Sample\_011046841, Unigene58058\_Sample\_011046841, Unigene17554\_Sample\_011046841, Unigene18699\_Sample\_011046841, Unigene21917\_Sample\_011046841, Unigene28681\_Sample\_011046841, Unigene30190\_Sample\_011046841, Unigene36463\_Sample\_011046841, Unigene42686\_Sample\_011046841, Unigene47409\_Sample\_011046841, Unigene52866\_Sample\_011046841, Unigene57665\_Sample\_011046841, Unigene7092\_Sample\_011046841, Unigene12023\_Sample\_011046841, Unigene41980\_Sample\_011046841, Unigene43782\_Sample\_011046841, Unigene51429\_Sample\_011046841, Unigene57304\_Sample\_011046841, Unigene58515\_Sample\_011046841, Unigene59010\_Sample\_011046841, Unigene1147\_Sample\_011046841, Unigene15792\_Sample\_011046841, Unigene42292\_Sample\_011046841, Unigene55286\_Sample\_011046841, Unigene55840\_Sample\_011046841, Unigene56828\_Sample\_011046841, Unigene18170\_Sample\_011046841, Unigene30684\_Sample\_011046841, Unigene41514\_Sample\_011046841, Unigene43515\_Sample\_011046841, Unigene47753\_Sample\_011046841, Unigene48742\_Sample\_011046841, Unigene51167\_Sample\_011046841, Unigene52499\_Sample\_011046841, Unigene60248\_Sample\_011046841, Unigene60948\_Sample\_011046841, Unigene47947\_Sample\_011046841, Unigene2110\_Sample\_011046841, Unigene20381\_Sample\_011046841, Unigene21201\_Sample\_011046841, Unigene24065\_Sample\_011046841, Unigene26229\_Sample\_011046841, Unigene32103\_Sample\_011046841, Unigene33512\_Sample\_011046841, Unigene36199\_Sample\_011046841, Unigene48731\_Sample\_011046841, Unigene48939\_Sample\_011046841, Unigene55041\_Sample\_011046841, Unigene3810\_Sample\_011046841, Unigene7879\_Sample\_011046841, Unigene11415\_Sample\_011046841, Unigene15705\_Sample\_011046841, Unigene23831\_Sample\_011046841, Unigene28288\_Sample\_011046841, Unigene28348\_Sample\_011046841, Unigene37595\_Sample\_011046841, Unigene43918\_Sample\_011046841, Unigene44749\_Sample\_011046841, Unigene44838\_Sample\_011046841, Unigene47429\_Sample\_011046841, Unigene47910\_Sample\_011046841, Unigene49087\_Sample\_011046841, Unigene54663\_Sample\_011046841, Unigene60132\_Sample\_011046841, Unigene17004\_Sample\_011046841, Unigene25028\_Sample\_011046841, Unigene26961\_Sample\_011046841, Unigene47083\_Sample\_011046841, Unigene51582\_Sample\_011046841, Unigene12324\_Sample\_011046841, Unigene60003\_Sample\_011046841, Unigene16539\_Sample\_011046841, Unigene28643\_Sample\_011046841, Unigene52096\_Sample\_011046841, Unigene58675\_Sample\_011046841, Unigene26757\_Sample\_011046841, Unigene30349\_Sample\_011046841, Unigene44021\_Sample\_011046841, Unigene44354\_Sample\_011046841, Unigene21539\_Sample\_011046841, Unigene35082\_Sample\_011046841, Unigene38019\_Sample\_011046841, Unigene40934\_Sample\_011046841, Unigene43097\_Sample\_011046841, Unigene46652\_Sample\_011046841, Unigene14248\_Sample\_011046841, Unigene46813\_Sample\_011046841, Unigene19789\_Sample\_011046841, Unigene21318\_Sample\_011046841, Unigene6639\_Sample\_011046841, Unigene17997\_Sample\_011046841, Unigene30474\_Sample\_011046841, Unigene7048\_Sample\_011046841, Unigene32977\_Sample\_011046841, Unigene39302\_Sample\_011046841 |
| 48 | Long-term potentiation Back to summary table | Unigene3199\_Sample\_011046841, Unigene8859\_Sample\_011046841, Unigene9668\_Sample\_011046841, Unigene10174\_Sample\_011046841, Unigene10640\_Sample\_011046841, Unigene10970\_Sample\_011046841, Unigene11876\_Sample\_011046841, Unigene13481\_Sample\_011046841, Unigene13789\_Sample\_011046841, Unigene13872\_Sample\_011046841, Unigene38234\_Sample\_011046841, Unigene39875\_Sample\_011046841, Unigene40230\_Sample\_011046841, Unigene43331\_Sample\_011046841, Unigene45114\_Sample\_011046841, Unigene46058\_Sample\_011046841, Unigene47924\_Sample\_011046841, Unigene48524\_Sample\_011046841, Unigene51426\_Sample\_011046841, Unigene53424\_Sample\_011046841, Unigene53888\_Sample\_011046841, Unigene56389\_Sample\_011046841, Unigene56500\_Sample\_011046841, Unigene57609\_Sample\_011046841, Unigene58460\_Sample\_011046841, Unigene59189\_Sample\_011046841, Unigene60252\_Sample\_011046841, Unigene1345\_Sample\_011046841, Unigene3223\_Sample\_011046841, Unigene3363\_Sample\_011046841, Unigene4210\_Sample\_011046841, Unigene5744\_Sample\_011046841, Unigene8641\_Sample\_011046841, Unigene9867\_Sample\_011046841, Unigene10189\_Sample\_011046841, Unigene13091\_Sample\_011046841, Unigene13332\_Sample\_011046841, Unigene13494\_Sample\_011046841, Unigene13945\_Sample\_011046841, Unigene14467\_Sample\_011046841, Unigene21962\_Sample\_011046841, Unigene23312\_Sample\_011046841, Unigene41574\_Sample\_011046841, Unigene47555\_Sample\_011046841, Unigene47903\_Sample\_011046841, Unigene49320\_Sample\_011046841, Unigene50039\_Sample\_011046841, Unigene53457\_Sample\_011046841, Unigene54900\_Sample\_011046841, Unigene56177\_Sample\_011046841, Unigene56232\_Sample\_011046841, Unigene58285\_Sample\_011046841, Unigene58611\_Sample\_011046841, Unigene60006\_Sample\_011046841, Unigene8083\_Sample\_011046841, Unigene8813\_Sample\_011046841, Unigene11266\_Sample\_011046841, Unigene13367\_Sample\_011046841, Unigene23751\_Sample\_011046841, Unigene28658\_Sample\_011046841, Unigene32259\_Sample\_011046841, Unigene34582\_Sample\_011046841, Unigene44316\_Sample\_011046841, Unigene53168\_Sample\_011046841, Unigene54593\_Sample\_011046841, Unigene58557\_Sample\_011046841, Unigene60517\_Sample\_011046841, Unigene32964\_Sample\_011046841, Unigene47549\_Sample\_011046841, Unigene47860\_Sample\_011046841, Unigene49671\_Sample\_011046841, Unigene57387\_Sample\_011046841, Unigene57675\_Sample\_011046841, Unigene58058\_Sample\_011046841, Unigene3905\_Sample\_011046841, Unigene30190\_Sample\_011046841, Unigene45708\_Sample\_011046841, Unigene50746\_Sample\_011046841, Unigene52866\_Sample\_011046841, Unigene57665\_Sample\_011046841, Unigene2942\_Sample\_011046841, Unigene9329\_Sample\_011046841, Unigene38302\_Sample\_011046841, Unigene47967\_Sample\_011046841, Unigene50145\_Sample\_011046841, Unigene51429\_Sample\_011046841, Unigene15792\_Sample\_011046841, Unigene55286\_Sample\_011046841, Unigene421\_Sample\_011046841, Unigene29318\_Sample\_011046841, Unigene52499\_Sample\_011046841, Unigene56139\_Sample\_011046841, Unigene56390\_Sample\_011046841, Unigene57117\_Sample\_011046841, Unigene5898\_Sample\_011046841, Unigene16098\_Sample\_011046841, Unigene24650\_Sample\_011046841, Unigene42069\_Sample\_011046841, Unigene2110\_Sample\_011046841, Unigene26229\_Sample\_011046841, Unigene40307\_Sample\_011046841, Unigene53853\_Sample\_011046841, Unigene53995\_Sample\_011046841, Unigene55041\_Sample\_011046841, Unigene12521\_Sample\_011046841, Unigene15705\_Sample\_011046841, Unigene16014\_Sample\_011046841, Unigene23831\_Sample\_011046841, Unigene28288\_Sample\_011046841, Unigene36841\_Sample\_011046841, Unigene40166\_Sample\_011046841, Unigene43099\_Sample\_011046841, Unigene44838\_Sample\_011046841, Unigene47429\_Sample\_011046841, Unigene47872\_Sample\_011046841, Unigene47910\_Sample\_011046841, Unigene49087\_Sample\_011046841, Unigene54663\_Sample\_011046841, Unigene59748\_Sample\_011046841, Unigene60132\_Sample\_011046841, Unigene17004\_Sample\_011046841, Unigene19947\_Sample\_011046841, Unigene47083\_Sample\_011046841, Unigene12324\_Sample\_011046841, Unigene60003\_Sample\_011046841, Unigene16539\_Sample\_011046841, Unigene28384\_Sample\_011046841, Unigene44021\_Sample\_011046841, Unigene44354\_Sample\_011046841, Unigene21539\_Sample\_011046841, Unigene35082\_Sample\_011046841, Unigene40934\_Sample\_011046841, Unigene43097\_Sample\_011046841, Unigene14248\_Sample\_011046841, Unigene46813\_Sample\_011046841, Unigene19789\_Sample\_011046841, Unigene59655\_Sample\_011046841, Unigene14181\_Sample\_011046841, Unigene17997\_Sample\_011046841, Unigene30474\_Sample\_011046841, Unigene32977\_Sample\_011046841, Unigene39302\_Sample\_011046841 |
| 49 | Wnt signaling pathway Back to summary table | Unigene2686\_Sample\_011046841, Unigene3199\_Sample\_011046841, Unigene4236\_Sample\_011046841, Unigene4963\_Sample\_011046841, Unigene5012\_Sample\_011046841, Unigene5122\_Sample\_011046841, Unigene5178\_Sample\_011046841, Unigene5402\_Sample\_011046841, Unigene6605\_Sample\_011046841, Unigene6971\_Sample\_011046841, Unigene8148\_Sample\_011046841, Unigene8859\_Sample\_011046841, Unigene8881\_Sample\_011046841, Unigene9107\_Sample\_011046841, Unigene9668\_Sample\_011046841, Unigene10640\_Sample\_011046841, Unigene11187\_Sample\_011046841, Unigene12213\_Sample\_011046841, Unigene12709\_Sample\_011046841, Unigene13346\_Sample\_011046841, Unigene13872\_Sample\_011046841, Unigene13927\_Sample\_011046841, Unigene27324\_Sample\_011046841, Unigene28326\_Sample\_011046841, Unigene37440\_Sample\_011046841, Unigene40230\_Sample\_011046841, Unigene40566\_Sample\_011046841, Unigene42704\_Sample\_011046841, Unigene45022\_Sample\_011046841, Unigene45114\_Sample\_011046841, Unigene48524\_Sample\_011046841, Unigene49649\_Sample\_011046841, Unigene51219\_Sample\_011046841, Unigene51426\_Sample\_011046841, Unigene51697\_Sample\_011046841, Unigene53585\_Sample\_011046841, Unigene54982\_Sample\_011046841, Unigene56371\_Sample\_011046841, Unigene56886\_Sample\_011046841, Unigene57114\_Sample\_011046841, Unigene57298\_Sample\_011046841, Unigene57609\_Sample\_011046841, Unigene58300\_Sample\_011046841, Unigene58307\_Sample\_011046841, Unigene58386\_Sample\_011046841, Unigene58719\_Sample\_011046841, Unigene59022\_Sample\_011046841, Unigene59151\_Sample\_011046841, Unigene59189\_Sample\_011046841, Unigene59558\_Sample\_011046841, Unigene60252\_Sample\_011046841, Unigene60313\_Sample\_011046841, Unigene1378\_Sample\_011046841, Unigene3363\_Sample\_011046841, Unigene5744\_Sample\_011046841, Unigene5747\_Sample\_011046841, Unigene7091\_Sample\_011046841, Unigene7709\_Sample\_011046841, Unigene8003\_Sample\_011046841, Unigene8641\_Sample\_011046841, Unigene9579\_Sample\_011046841, Unigene9867\_Sample\_011046841, Unigene9971\_Sample\_011046841, Unigene11134\_Sample\_011046841, Unigene12739\_Sample\_011046841, Unigene13656\_Sample\_011046841, Unigene13722\_Sample\_011046841, Unigene13945\_Sample\_011046841, Unigene19273\_Sample\_011046841, Unigene21418\_Sample\_011046841, Unigene21962\_Sample\_011046841, Unigene25098\_Sample\_011046841, Unigene30681\_Sample\_011046841, Unigene37278\_Sample\_011046841, Unigene37760\_Sample\_011046841, Unigene40989\_Sample\_011046841, Unigene41465\_Sample\_011046841, Unigene42106\_Sample\_011046841, Unigene42561\_Sample\_011046841, Unigene42676\_Sample\_011046841, Unigene42820\_Sample\_011046841, Unigene43448\_Sample\_011046841, Unigene44479\_Sample\_011046841, Unigene46219\_Sample\_011046841, Unigene46763\_Sample\_011046841, Unigene47210\_Sample\_011046841, Unigene49399\_Sample\_011046841, Unigene50039\_Sample\_011046841, Unigene50163\_Sample\_011046841, Unigene52184\_Sample\_011046841, Unigene52882\_Sample\_011046841, Unigene53186\_Sample\_011046841, Unigene53714\_Sample\_011046841, Unigene54525\_Sample\_011046841, Unigene55673\_Sample\_011046841, Unigene56010\_Sample\_011046841, Unigene56555\_Sample\_011046841, Unigene56663\_Sample\_011046841, Unigene57337\_Sample\_011046841, Unigene57390\_Sample\_011046841, Unigene58149\_Sample\_011046841, Unigene58564\_Sample\_011046841, Unigene58611\_Sample\_011046841, Unigene59660\_Sample\_011046841, Unigene59671\_Sample\_011046841, Unigene59696\_Sample\_011046841, Unigene59756\_Sample\_011046841, Unigene59894\_Sample\_011046841, Unigene59902\_Sample\_011046841, Unigene60190\_Sample\_011046841, Unigene60271\_Sample\_011046841, Unigene864\_Sample\_011046841, Unigene1526\_Sample\_011046841, Unigene2066\_Sample\_011046841, Unigene3070\_Sample\_011046841, Unigene3491\_Sample\_011046841, Unigene8828\_Sample\_011046841, Unigene9726\_Sample\_011046841, Unigene9831\_Sample\_011046841, Unigene19797\_Sample\_011046841, Unigene20297\_Sample\_011046841, Unigene20484\_Sample\_011046841, Unigene21055\_Sample\_011046841, Unigene25812\_Sample\_011046841, Unigene30120\_Sample\_011046841, Unigene32259\_Sample\_011046841, Unigene32413\_Sample\_011046841, Unigene43504\_Sample\_011046841, Unigene47387\_Sample\_011046841, Unigene47460\_Sample\_011046841, Unigene48530\_Sample\_011046841, Unigene49895\_Sample\_011046841, Unigene51171\_Sample\_011046841, Unigene51803\_Sample\_011046841, Unigene53029\_Sample\_011046841, Unigene54549\_Sample\_011046841, Unigene54574\_Sample\_011046841, Unigene55183\_Sample\_011046841, Unigene56160\_Sample\_011046841, Unigene56456\_Sample\_011046841, Unigene59322\_Sample\_011046841, Unigene59727\_Sample\_011046841, Unigene2116\_Sample\_011046841, Unigene6435\_Sample\_011046841, Unigene19951\_Sample\_011046841, Unigene21225\_Sample\_011046841, Unigene27828\_Sample\_011046841, Unigene30184\_Sample\_011046841, Unigene35757\_Sample\_011046841, Unigene38616\_Sample\_011046841, Unigene39807\_Sample\_011046841, Unigene50901\_Sample\_011046841, Unigene57832\_Sample\_011046841, Unigene60102\_Sample\_011046841, Unigene3481\_Sample\_011046841, Unigene8726\_Sample\_011046841, Unigene29346\_Sample\_011046841, Unigene30190\_Sample\_011046841, Unigene32509\_Sample\_011046841, Unigene45708\_Sample\_011046841, Unigene57665\_Sample\_011046841, Unigene59077\_Sample\_011046841, Unigene2942\_Sample\_011046841, Unigene9329\_Sample\_011046841, Unigene34657\_Sample\_011046841, Unigene51429\_Sample\_011046841, Unigene52354\_Sample\_011046841, Unigene52611\_Sample\_011046841, Unigene57304\_Sample\_011046841, Unigene58515\_Sample\_011046841, Unigene59361\_Sample\_011046841, Unigene60217\_Sample\_011046841, Unigene31905\_Sample\_011046841, Unigene30153\_Sample\_011046841, Unigene41466\_Sample\_011046841, Unigene41514\_Sample\_011046841, Unigene52590\_Sample\_011046841, Unigene56139\_Sample\_011046841, Unigene57117\_Sample\_011046841, Unigene16098\_Sample\_011046841, Unigene47947\_Sample\_011046841, Unigene2110\_Sample\_011046841, Unigene12562\_Sample\_011046841, Unigene20381\_Sample\_011046841, Unigene32103\_Sample\_011046841, Unigene33512\_Sample\_011046841, Unigene35691\_Sample\_011046841, Unigene36199\_Sample\_011046841, Unigene53367\_Sample\_011046841, Unigene55041\_Sample\_011046841, Unigene12521\_Sample\_011046841, Unigene13126\_Sample\_011046841, Unigene15705\_Sample\_011046841, Unigene23831\_Sample\_011046841, Unigene26636\_Sample\_011046841, Unigene28288\_Sample\_011046841, Unigene42580\_Sample\_011046841, Unigene43099\_Sample\_011046841, Unigene43918\_Sample\_011046841, Unigene44749\_Sample\_011046841, Unigene47429\_Sample\_011046841, Unigene47872\_Sample\_011046841, Unigene49087\_Sample\_011046841, Unigene54104\_Sample\_011046841, Unigene54663\_Sample\_011046841, Unigene59748\_Sample\_011046841, Unigene12700\_Sample\_011046841, Unigene25028\_Sample\_011046841, Unigene26961\_Sample\_011046841, Unigene50989\_Sample\_011046841, Unigene53893\_Sample\_011046841, Unigene12324\_Sample\_011046841, Unigene14639\_Sample\_011046841, Unigene16539\_Sample\_011046841, Unigene28384\_Sample\_011046841, Unigene28643\_Sample\_011046841, Unigene53768\_Sample\_011046841, Unigene44021\_Sample\_011046841, Unigene44354\_Sample\_011046841, Unigene21539\_Sample\_011046841, Unigene35082\_Sample\_011046841, Unigene38019\_Sample\_011046841, Unigene46652\_Sample\_011046841, Unigene14248\_Sample\_011046841, Unigene34330\_Sample\_011046841, Unigene43556\_Sample\_011046841, Unigene14181\_Sample\_011046841, Unigene21318\_Sample\_011046841, Unigene6639\_Sample\_011046841 |
| 50 | Melanoma Back to summary table | Unigene4209\_Sample\_011046841, Unigene7809\_Sample\_011046841, Unigene8429\_Sample\_011046841, Unigene9858\_Sample\_011046841, Unigene10459\_Sample\_011046841, Unigene10744\_Sample\_011046841, Unigene10970\_Sample\_011046841, Unigene13069\_Sample\_011046841, Unigene58460\_Sample\_011046841, Unigene59705\_Sample\_011046841, Unigene60173\_Sample\_011046841, Unigene60498\_Sample\_011046841, Unigene1414\_Sample\_011046841, Unigene11676\_Sample\_011046841, Unigene12943\_Sample\_011046841, Unigene41574\_Sample\_011046841, Unigene47555\_Sample\_011046841, Unigene49494\_Sample\_011046841, Unigene54859\_Sample\_011046841, Unigene54900\_Sample\_011046841, Unigene56798\_Sample\_011046841, Unigene57566\_Sample\_011046841, Unigene58100\_Sample\_011046841, Unigene59109\_Sample\_011046841, Unigene60427\_Sample\_011046841, Unigene60552\_Sample\_011046841, Unigene3582\_Sample\_011046841, Unigene23590\_Sample\_011046841, Unigene28658\_Sample\_011046841, Unigene52294\_Sample\_011046841, Unigene57775\_Sample\_011046841, Unigene58321\_Sample\_011046841, Unigene32964\_Sample\_011046841, Unigene56300\_Sample\_011046841, Unigene60863\_Sample\_011046841, Unigene49525\_Sample\_011046841, Unigene1107\_Sample\_011046841, Unigene47967\_Sample\_011046841, Unigene18170\_Sample\_011046841, Unigene29318\_Sample\_011046841, Unigene51756\_Sample\_011046841, Unigene60248\_Sample\_011046841, Unigene53995\_Sample\_011046841, Unigene47910\_Sample\_011046841, Unigene50832\_Sample\_011046841, Unigene31969\_Sample\_011046841, Unigene51582\_Sample\_011046841, Unigene43718\_Sample\_011046841, Unigene41007\_Sample\_011046841, Unigene46813\_Sample\_011046841, Unigene30474\_Sample\_011046841, Unigene26777\_Sample\_011046841 |
| 51 | Toll-like receptor signaling pathway Back to summary table | Unigene4209\_Sample\_011046841, Unigene10459\_Sample\_011046841, Unigene13069\_Sample\_011046841, Unigene40774\_Sample\_011046841, Unigene57114\_Sample\_011046841, Unigene57629\_Sample\_011046841, Unigene58460\_Sample\_011046841, Unigene60173\_Sample\_011046841, Unigene60498\_Sample\_011046841, Unigene13419\_Sample\_011046841, Unigene21418\_Sample\_011046841, Unigene41574\_Sample\_011046841, Unigene46219\_Sample\_011046841, Unigene46243\_Sample\_011046841, Unigene47210\_Sample\_011046841, Unigene47555\_Sample\_011046841, Unigene49894\_Sample\_011046841, Unigene50170\_Sample\_011046841, Unigene52714\_Sample\_011046841, Unigene57215\_Sample\_011046841, Unigene57566\_Sample\_011046841, Unigene58100\_Sample\_011046841, Unigene58669\_Sample\_011046841, Unigene59037\_Sample\_011046841, Unigene59756\_Sample\_011046841, Unigene23590\_Sample\_011046841, Unigene24214\_Sample\_011046841, Unigene28658\_Sample\_011046841, Unigene58423\_Sample\_011046841, Unigene2116\_Sample\_011046841, Unigene32964\_Sample\_011046841, Unigene56300\_Sample\_011046841, Unigene49525\_Sample\_011046841, Unigene59421\_Sample\_011046841, Unigene60217\_Sample\_011046841, Unigene7042\_Sample\_011046841, Unigene43285\_Sample\_011046841, Unigene51756\_Sample\_011046841, Unigene21362\_Sample\_011046841, Unigene42580\_Sample\_011046841, Unigene46799\_Sample\_011046841, Unigene47910\_Sample\_011046841, Unigene50832\_Sample\_011046841, Unigene31264\_Sample\_011046841, Unigene31544\_Sample\_011046841, Unigene16442\_Sample\_011046841, Unigene30484\_Sample\_011046841, Unigene47863\_Sample\_011046841, Unigene48345\_Sample\_011046841, Unigene46813\_Sample\_011046841, Unigene30474\_Sample\_011046841, Unigene50127\_Sample\_011046841 |
| 52 | Mismatch repair Back to summary table | Unigene7590\_Sample\_011046841, Unigene8647\_Sample\_011046841, Unigene11562\_Sample\_011046841, Unigene12869\_Sample\_011046841, Unigene13500\_Sample\_011046841, Unigene28864\_Sample\_011046841, Unigene34410\_Sample\_011046841, Unigene43894\_Sample\_011046841, Unigene44567\_Sample\_011046841, Unigene54555\_Sample\_011046841, Unigene55735\_Sample\_011046841, Unigene56979\_Sample\_011046841, Unigene1934\_Sample\_011046841, Unigene5795\_Sample\_011046841, Unigene5967\_Sample\_011046841, Unigene6034\_Sample\_011046841, Unigene8165\_Sample\_011046841, Unigene9008\_Sample\_011046841, Unigene13774\_Sample\_011046841, Unigene39877\_Sample\_011046841, Unigene45539\_Sample\_011046841, Unigene47769\_Sample\_011046841, Unigene55393\_Sample\_011046841, Unigene55543\_Sample\_011046841, Unigene56952\_Sample\_011046841, Unigene10789\_Sample\_011046841, Unigene19399\_Sample\_011046841, Unigene20927\_Sample\_011046841, Unigene36469\_Sample\_011046841, Unigene42576\_Sample\_011046841, Unigene42967\_Sample\_011046841, Unigene46973\_Sample\_011046841, Unigene50396\_Sample\_011046841, Unigene50207\_Sample\_011046841, Unigene13428\_Sample\_011046841, Unigene16199\_Sample\_011046841, Unigene44445\_Sample\_011046841, Unigene49162\_Sample\_011046841, Unigene12822\_Sample\_011046841, Unigene18715\_Sample\_011046841, Unigene45777\_Sample\_011046841, Unigene59567\_Sample\_011046841, Unigene17752\_Sample\_011046841, Unigene32121\_Sample\_011046841, Unigene50798\_Sample\_011046841, Unigene42749\_Sample\_011046841, Unigene48882\_Sample\_011046841, Unigene51331\_Sample\_011046841, Unigene11233\_Sample\_011046841, Unigene38004\_Sample\_011046841, Unigene17439\_Sample\_011046841, Unigene31249\_Sample\_011046841 |
| 53 | Pentose phosphate pathway Back to summary table | Unigene7029\_Sample\_011046841, Unigene18832\_Sample\_011046841, Unigene24105\_Sample\_011046841, Unigene51910\_Sample\_011046841, Unigene54492\_Sample\_011046841, Unigene59941\_Sample\_011046841, Unigene20606\_Sample\_011046841, Unigene24754\_Sample\_011046841, Unigene27682\_Sample\_011046841, Unigene31490\_Sample\_011046841, Unigene31641\_Sample\_011046841, Unigene49729\_Sample\_011046841, Unigene51598\_Sample\_011046841, Unigene52614\_Sample\_011046841, Unigene53210\_Sample\_011046841, Unigene54707\_Sample\_011046841, Unigene55866\_Sample\_011046841, Unigene56529\_Sample\_011046841, Unigene21061\_Sample\_011046841, Unigene35210\_Sample\_011046841, Unigene35485\_Sample\_011046841, Unigene50269\_Sample\_011046841, Unigene55314\_Sample\_011046841, Unigene58163\_Sample\_011046841, Unigene51771\_Sample\_011046841, Unigene39617\_Sample\_011046841, Unigene7585\_Sample\_011046841, Unigene31394\_Sample\_011046841, Unigene54624\_Sample\_011046841, Unigene18276\_Sample\_011046841, Unigene39082\_Sample\_011046841, Unigene41811\_Sample\_011046841, Unigene47162\_Sample\_011046841, Unigene48873\_Sample\_011046841, Unigene41318\_Sample\_011046841, Unigene19914\_Sample\_011046841, Unigene47820\_Sample\_011046841, Unigene12706\_Sample\_011046841, Unigene19013\_Sample\_011046841, Unigene20440\_Sample\_011046841, Unigene27017\_Sample\_011046841, Unigene42184\_Sample\_011046841, Unigene49635\_Sample\_011046841, Unigene39842\_Sample\_011046841, Unigene40365\_Sample\_011046841, Unigene17285\_Sample\_011046841, Unigene22652\_Sample\_011046841, Unigene46950\_Sample\_011046841, Unigene23065\_Sample\_011046841 |
| 54 | Hypertrophic cardiomyopathy (HCM) Back to summary table | Unigene290\_Sample\_011046841, Unigene863\_Sample\_011046841, Unigene1210\_Sample\_011046841, Unigene4208\_Sample\_011046841, Unigene7142\_Sample\_011046841, Unigene8772\_Sample\_011046841, Unigene9237\_Sample\_011046841, Unigene10581\_Sample\_011046841, Unigene11938\_Sample\_011046841, Unigene13158\_Sample\_011046841, Unigene13755\_Sample\_011046841, Unigene22077\_Sample\_011046841, Unigene27010\_Sample\_011046841, Unigene43731\_Sample\_011046841, Unigene47875\_Sample\_011046841, Unigene48170\_Sample\_011046841, Unigene48836\_Sample\_011046841, Unigene53568\_Sample\_011046841, Unigene53928\_Sample\_011046841, Unigene55850\_Sample\_011046841, Unigene58388\_Sample\_011046841, Unigene58796\_Sample\_011046841, Unigene58960\_Sample\_011046841, Unigene59870\_Sample\_011046841, Unigene60175\_Sample\_011046841, Unigene60485\_Sample\_011046841, Unigene60525\_Sample\_011046841, Unigene60612\_Sample\_011046841, Unigene60766\_Sample\_011046841, Unigene60967\_Sample\_011046841, Unigene2588\_Sample\_011046841, Unigene2591\_Sample\_011046841, Unigene3890\_Sample\_011046841, Unigene4003\_Sample\_011046841, Unigene7002\_Sample\_011046841, Unigene10189\_Sample\_011046841, Unigene11856\_Sample\_011046841, Unigene11912\_Sample\_011046841, Unigene13771\_Sample\_011046841, Unigene26850\_Sample\_011046841, Unigene28584\_Sample\_011046841, Unigene30456\_Sample\_011046841, Unigene38644\_Sample\_011046841, Unigene47384\_Sample\_011046841, Unigene50676\_Sample\_011046841, Unigene51782\_Sample\_011046841, Unigene52610\_Sample\_011046841, Unigene53584\_Sample\_011046841, Unigene53983\_Sample\_011046841, Unigene54140\_Sample\_011046841, Unigene54650\_Sample\_011046841, Unigene58503\_Sample\_011046841, Unigene59003\_Sample\_011046841, Unigene59983\_Sample\_011046841, Unigene60020\_Sample\_011046841, Unigene60195\_Sample\_011046841, Unigene60288\_Sample\_011046841, Unigene60554\_Sample\_011046841, Unigene60628\_Sample\_011046841, Unigene60806\_Sample\_011046841, Unigene60947\_Sample\_011046841, Unigene7339\_Sample\_011046841, Unigene8083\_Sample\_011046841, Unigene13618\_Sample\_011046841, Unigene13958\_Sample\_011046841, Unigene18856\_Sample\_011046841, Unigene19285\_Sample\_011046841, Unigene20059\_Sample\_011046841, Unigene22974\_Sample\_011046841, Unigene35184\_Sample\_011046841, Unigene42781\_Sample\_011046841, Unigene57194\_Sample\_011046841, Unigene58818\_Sample\_011046841, Unigene60443\_Sample\_011046841, Unigene60813\_Sample\_011046841, Unigene10486\_Sample\_011046841, Unigene13853\_Sample\_011046841, Unigene28860\_Sample\_011046841, Unigene48046\_Sample\_011046841, Unigene49117\_Sample\_011046841, Unigene57243\_Sample\_011046841, Unigene57267\_Sample\_011046841, Unigene58006\_Sample\_011046841, Unigene58882\_Sample\_011046841, Unigene59045\_Sample\_011046841, Unigene59998\_Sample\_011046841, Unigene10648\_Sample\_011046841, Unigene54809\_Sample\_011046841, Unigene48357\_Sample\_011046841, Unigene55740\_Sample\_011046841, Unigene58426\_Sample\_011046841, Unigene47560\_Sample\_011046841, Unigene52695\_Sample\_011046841, Unigene56082\_Sample\_011046841, Unigene58176\_Sample\_011046841, Unigene23133\_Sample\_011046841, Unigene25908\_Sample\_011046841, Unigene7431\_Sample\_011046841, Unigene15451\_Sample\_011046841, Unigene16055\_Sample\_011046841, Unigene26385\_Sample\_011046841, Unigene36385\_Sample\_011046841, Unigene38948\_Sample\_011046841, Unigene42173\_Sample\_011046841, Unigene43721\_Sample\_011046841, Unigene50460\_Sample\_011046841, Unigene52605\_Sample\_011046841, Unigene55113\_Sample\_011046841, Unigene55234\_Sample\_011046841, Unigene60518\_Sample\_011046841, Unigene19677\_Sample\_011046841, Unigene24961\_Sample\_011046841, Unigene26878\_Sample\_011046841, Unigene32470\_Sample\_011046841, Unigene34824\_Sample\_011046841, Unigene48929\_Sample\_011046841, Unigene48977\_Sample\_011046841, Unigene51916\_Sample\_011046841, Unigene57578\_Sample\_011046841, Unigene46495\_Sample\_011046841, Unigene40605\_Sample\_011046841, Unigene19717\_Sample\_011046841, Unigene58511\_Sample\_011046841, Unigene12560\_Sample\_011046841, Unigene21220\_Sample\_011046841, Unigene15058\_Sample\_011046841, Unigene37784\_Sample\_011046841, Unigene48121\_Sample\_011046841, Unigene22791\_Sample\_011046841, Unigene59655\_Sample\_011046841, Unigene35586\_Sample\_011046841, Unigene41604\_Sample\_011046841 |
| 55 | NOD-like receptor signaling pathway Back to summary table | Unigene26714\_Sample\_011046841, Unigene35926\_Sample\_011046841, Unigene57114\_Sample\_011046841, Unigene59374\_Sample\_011046841, Unigene60239\_Sample\_011046841, Unigene60269\_Sample\_011046841, Unigene60568\_Sample\_011046841, Unigene244\_Sample\_011046841, Unigene7869\_Sample\_011046841, Unigene8299\_Sample\_011046841, Unigene13620\_Sample\_011046841, Unigene41574\_Sample\_011046841, Unigene47210\_Sample\_011046841, Unigene47555\_Sample\_011046841, Unigene50170\_Sample\_011046841, Unigene51148\_Sample\_011046841, Unigene54278\_Sample\_011046841, Unigene55825\_Sample\_011046841, Unigene56004\_Sample\_011046841, Unigene57215\_Sample\_011046841, Unigene58466\_Sample\_011046841, Unigene58833\_Sample\_011046841, Unigene59756\_Sample\_011046841, Unigene60454\_Sample\_011046841, Unigene60543\_Sample\_011046841, Unigene60756\_Sample\_011046841, Unigene28658\_Sample\_011046841, Unigene49942\_Sample\_011046841, Unigene53989\_Sample\_011046841, Unigene2116\_Sample\_011046841, Unigene32964\_Sample\_011046841, Unigene46938\_Sample\_011046841, Unigene59421\_Sample\_011046841, Unigene59482\_Sample\_011046841, Unigene60217\_Sample\_011046841, Unigene43285\_Sample\_011046841, Unigene59656\_Sample\_011046841, Unigene804\_Sample\_011046841, Unigene27016\_Sample\_011046841, Unigene21362\_Sample\_011046841, Unigene47910\_Sample\_011046841, Unigene23857\_Sample\_011046841, Unigene31264\_Sample\_011046841, Unigene16442\_Sample\_011046841, Unigene30484\_Sample\_011046841, Unigene41460\_Sample\_011046841, Unigene43091\_Sample\_011046841, Unigene30474\_Sample\_011046841 |
| 56 | Lysine degradation Back to summary table | Unigene6007\_Sample\_011046841, Unigene8835\_Sample\_011046841, Unigene10184\_Sample\_011046841, Unigene13344\_Sample\_011046841, Unigene48532\_Sample\_011046841, Unigene54550\_Sample\_011046841, Unigene57000\_Sample\_011046841, Unigene59158\_Sample\_011046841, Unigene59984\_Sample\_011046841, Unigene60317\_Sample\_011046841, Unigene60764\_Sample\_011046841, Unigene60828\_Sample\_011046841, Unigene3734\_Sample\_011046841, Unigene6106\_Sample\_011046841, Unigene8047\_Sample\_011046841, Unigene11984\_Sample\_011046841, Unigene13909\_Sample\_011046841, Unigene36330\_Sample\_011046841, Unigene53032\_Sample\_011046841, Unigene57713\_Sample\_011046841, Unigene60366\_Sample\_011046841, Unigene60386\_Sample\_011046841, Unigene60574\_Sample\_011046841, Unigene3047\_Sample\_011046841, Unigene6222\_Sample\_011046841, Unigene11804\_Sample\_011046841, Unigene32280\_Sample\_011046841, Unigene53170\_Sample\_011046841, Unigene55561\_Sample\_011046841, Unigene56009\_Sample\_011046841, Unigene58173\_Sample\_011046841, Unigene60848\_Sample\_011046841, Unigene13015\_Sample\_011046841, Unigene25319\_Sample\_011046841, Unigene5603\_Sample\_011046841, Unigene56764\_Sample\_011046841, Unigene57452\_Sample\_011046841, Unigene10982\_Sample\_011046841, Unigene25630\_Sample\_011046841, Unigene60222\_Sample\_011046841, Unigene31259\_Sample\_011046841, Unigene49650\_Sample\_011046841, Unigene9623\_Sample\_011046841, Unigene40771\_Sample\_011046841, Unigene51822\_Sample\_011046841, Unigene47326\_Sample\_011046841, Unigene58800\_Sample\_011046841 |
| 57 | MAPK signaling pathway - yeast Back to summary table | Unigene3199\_Sample\_011046841, Unigene13901\_Sample\_011046841, Unigene39219\_Sample\_011046841, Unigene40230\_Sample\_011046841, Unigene52094\_Sample\_011046841, Unigene58725\_Sample\_011046841, Unigene59559\_Sample\_011046841, Unigene3363\_Sample\_011046841, Unigene9867\_Sample\_011046841, Unigene13332\_Sample\_011046841, Unigene13452\_Sample\_011046841, Unigene21418\_Sample\_011046841, Unigene43409\_Sample\_011046841, Unigene45592\_Sample\_011046841, Unigene46219\_Sample\_011046841, Unigene50170\_Sample\_011046841, Unigene60041\_Sample\_011046841, Unigene60299\_Sample\_011046841, Unigene60944\_Sample\_011046841, Unigene2725\_Sample\_011046841, Unigene58479\_Sample\_011046841, Unigene53642\_Sample\_011046841, Unigene53803\_Sample\_011046841, Unigene59711\_Sample\_011046841, Unigene55486\_Sample\_011046841, Unigene59421\_Sample\_011046841, Unigene2942\_Sample\_011046841, Unigene9329\_Sample\_011046841, Unigene51301\_Sample\_011046841, Unigene56139\_Sample\_011046841, Unigene16098\_Sample\_011046841, Unigene56969\_Sample\_011046841, Unigene12521\_Sample\_011046841, Unigene21362\_Sample\_011046841, Unigene43099\_Sample\_011046841, Unigene47872\_Sample\_011046841, Unigene31264\_Sample\_011046841, Unigene20770\_Sample\_011046841, Unigene50713\_Sample\_011046841, Unigene16442\_Sample\_011046841, Unigene30484\_Sample\_011046841, Unigene41464\_Sample\_011046841, Unigene49482\_Sample\_011046841, Unigene19937\_Sample\_011046841, Unigene16813\_Sample\_011046841, Unigene26184\_Sample\_011046841, Unigene19596\_Sample\_011046841 |
| 58 | MAPK signaling pathway - fly Back to summary table | Unigene865\_Sample\_011046841, Unigene7143\_Sample\_011046841, Unigene7809\_Sample\_011046841, Unigene8035\_Sample\_011046841, Unigene9892\_Sample\_011046841, Unigene10970\_Sample\_011046841, Unigene11130\_Sample\_011046841, Unigene41694\_Sample\_011046841, Unigene58460\_Sample\_011046841, Unigene58987\_Sample\_011046841, Unigene1414\_Sample\_011046841, Unigene4256\_Sample\_011046841, Unigene12943\_Sample\_011046841, Unigene13855\_Sample\_011046841, Unigene20886\_Sample\_011046841, Unigene35368\_Sample\_011046841, Unigene41574\_Sample\_011046841, Unigene45858\_Sample\_011046841, Unigene47555\_Sample\_011046841, Unigene56681\_Sample\_011046841, Unigene56798\_Sample\_011046841, Unigene23218\_Sample\_011046841, Unigene28658\_Sample\_011046841, Unigene31438\_Sample\_011046841, Unigene40723\_Sample\_011046841, Unigene40758\_Sample\_011046841, Unigene57775\_Sample\_011046841, Unigene60765\_Sample\_011046841, Unigene7964\_Sample\_011046841, Unigene32964\_Sample\_011046841, Unigene60863\_Sample\_011046841, Unigene54521\_Sample\_011046841, Unigene1107\_Sample\_011046841, Unigene5263\_Sample\_011046841, Unigene41996\_Sample\_011046841, Unigene47054\_Sample\_011046841, Unigene50110\_Sample\_011046841, Unigene53995\_Sample\_011046841, Unigene47910\_Sample\_011046841, Unigene49698\_Sample\_011046841, Unigene43718\_Sample\_011046841, Unigene32258\_Sample\_011046841, Unigene42358\_Sample\_011046841, Unigene46813\_Sample\_011046841, Unigene17523\_Sample\_011046841, Unigene30474\_Sample\_011046841, Unigene26777\_Sample\_011046841 |
| 59 | Peroxisome Back to summary table | Unigene1004\_Sample\_011046841, Unigene4732\_Sample\_011046841, Unigene6373\_Sample\_011046841, Unigene7331\_Sample\_011046841, Unigene9129\_Sample\_011046841, Unigene9929\_Sample\_011046841, Unigene12906\_Sample\_011046841, Unigene20686\_Sample\_011046841, Unigene23925\_Sample\_011046841, Unigene25984\_Sample\_011046841, Unigene49604\_Sample\_011046841, Unigene50829\_Sample\_011046841, Unigene55348\_Sample\_011046841, Unigene59971\_Sample\_011046841, Unigene60298\_Sample\_011046841, Unigene7507\_Sample\_011046841, Unigene12695\_Sample\_011046841, Unigene13938\_Sample\_011046841, Unigene21319\_Sample\_011046841, Unigene53002\_Sample\_011046841, Unigene54468\_Sample\_011046841, Unigene57074\_Sample\_011046841, Unigene59055\_Sample\_011046841, Unigene59746\_Sample\_011046841, Unigene60486\_Sample\_011046841, Unigene60822\_Sample\_011046841, Unigene24648\_Sample\_011046841, Unigene43891\_Sample\_011046841, Unigene55900\_Sample\_011046841, Unigene57635\_Sample\_011046841, Unigene59541\_Sample\_011046841, Unigene59663\_Sample\_011046841, Unigene31165\_Sample\_011046841, Unigene27921\_Sample\_011046841, Unigene30898\_Sample\_011046841, Unigene54933\_Sample\_011046841, Unigene23984\_Sample\_011046841, Unigene30428\_Sample\_011046841, Unigene56772\_Sample\_011046841, Unigene55373\_Sample\_011046841, Unigene53865\_Sample\_011046841, Unigene1482\_Sample\_011046841, Unigene49579\_Sample\_011046841, Unigene50185\_Sample\_011046841, Unigene43173\_Sample\_011046841, Unigene25993\_Sample\_011046841 |
| 60 | Carbon fixation in photosynthetic organisms Back to summary table | Unigene21438\_Sample\_011046841, Unigene24105\_Sample\_011046841, Unigene40350\_Sample\_011046841, Unigene54492\_Sample\_011046841, Unigene56182\_Sample\_011046841, Unigene59941\_Sample\_011046841, Unigene60564\_Sample\_011046841, Unigene9455\_Sample\_011046841, Unigene13979\_Sample\_011046841, Unigene19438\_Sample\_011046841, Unigene24754\_Sample\_011046841, Unigene31025\_Sample\_011046841, Unigene31641\_Sample\_011046841, Unigene49640\_Sample\_011046841, Unigene56579\_Sample\_011046841, Unigene57560\_Sample\_011046841, Unigene60126\_Sample\_011046841, Unigene60199\_Sample\_011046841, Unigene60884\_Sample\_011046841, Unigene33513\_Sample\_011046841, Unigene35485\_Sample\_011046841, Unigene43814\_Sample\_011046841, Unigene51117\_Sample\_011046841, Unigene56468\_Sample\_011046841, Unigene60606\_Sample\_011046841, Unigene39766\_Sample\_011046841, Unigene50393\_Sample\_011046841, Unigene51771\_Sample\_011046841, Unigene48766\_Sample\_011046841, Unigene26689\_Sample\_011046841, Unigene29803\_Sample\_011046841, Unigene18276\_Sample\_011046841, Unigene18442\_Sample\_011046841, Unigene48873\_Sample\_011046841, Unigene33197\_Sample\_011046841, Unigene30216\_Sample\_011046841, Unigene41423\_Sample\_011046841, Unigene45410\_Sample\_011046841, Unigene54935\_Sample\_011046841, Unigene19137\_Sample\_011046841, Unigene22736\_Sample\_011046841, Unigene27017\_Sample\_011046841, Unigene17285\_Sample\_011046841, Unigene22804\_Sample\_011046841, Unigene54187\_Sample\_011046841, Unigene46950\_Sample\_011046841 |
| 61 | Glycosylphosphatidylinositol(GPI)-anchor biosynthesis Back to summary table | Unigene2370\_Sample\_011046841, Unigene10379\_Sample\_011046841, Unigene10690\_Sample\_011046841, Unigene12850\_Sample\_011046841, Unigene22965\_Sample\_011046841, Unigene34724\_Sample\_011046841, Unigene38010\_Sample\_011046841, Unigene39454\_Sample\_011046841, Unigene46621\_Sample\_011046841, Unigene51460\_Sample\_011046841, Unigene57575\_Sample\_011046841, Unigene60069\_Sample\_011046841, Unigene60830\_Sample\_011046841, Unigene8044\_Sample\_011046841, Unigene10517\_Sample\_011046841, Unigene48857\_Sample\_011046841, Unigene53327\_Sample\_011046841, Unigene55039\_Sample\_011046841, Unigene55661\_Sample\_011046841, Unigene57056\_Sample\_011046841, Unigene57241\_Sample\_011046841, Unigene18735\_Sample\_011046841, Unigene50469\_Sample\_011046841, Unigene50662\_Sample\_011046841, Unigene51789\_Sample\_011046841, Unigene58884\_Sample\_011046841, Unigene1999\_Sample\_011046841, Unigene11258\_Sample\_011046841, Unigene54067\_Sample\_011046841, Unigene29653\_Sample\_011046841, Unigene37765\_Sample\_011046841, Unigene54689\_Sample\_011046841, Unigene55139\_Sample\_011046841, Unigene625\_Sample\_011046841, Unigene35394\_Sample\_011046841, Unigene53290\_Sample\_011046841, Unigene54239\_Sample\_011046841, Unigene12157\_Sample\_011046841, Unigene41828\_Sample\_011046841, Unigene45879\_Sample\_011046841, Unigene6153\_Sample\_011046841, Unigene39581\_Sample\_011046841, Unigene46635\_Sample\_011046841, Unigene12865\_Sample\_011046841, Unigene49716\_Sample\_011046841 |
| 62 | Regulation of actin cytoskeleton Back to summary table | Unigene290\_Sample\_011046841, Unigene679\_Sample\_011046841, Unigene863\_Sample\_011046841, Unigene865\_Sample\_011046841, Unigene1137\_Sample\_011046841, Unigene1210\_Sample\_011046841, Unigene2138\_Sample\_011046841, Unigene4209\_Sample\_011046841, Unigene4416\_Sample\_011046841, Unigene5297\_Sample\_011046841, Unigene6945\_Sample\_011046841, Unigene7142\_Sample\_011046841, Unigene7809\_Sample\_011046841, Unigene9017\_Sample\_011046841, Unigene9237\_Sample\_011046841, Unigene9858\_Sample\_011046841, Unigene10174\_Sample\_011046841, Unigene10459\_Sample\_011046841, Unigene10581\_Sample\_011046841, Unigene10970\_Sample\_011046841, Unigene11055\_Sample\_011046841, Unigene11938\_Sample\_011046841, Unigene13069\_Sample\_011046841, Unigene13664\_Sample\_011046841, Unigene13901\_Sample\_011046841, Unigene14576\_Sample\_011046841, Unigene22077\_Sample\_011046841, Unigene23925\_Sample\_011046841, Unigene27010\_Sample\_011046841, Unigene38234\_Sample\_011046841, Unigene39219\_Sample\_011046841, Unigene39875\_Sample\_011046841, Unigene43331\_Sample\_011046841, Unigene48170\_Sample\_011046841, Unigene48836\_Sample\_011046841, Unigene49240\_Sample\_011046841, Unigene49386\_Sample\_011046841, Unigene52094\_Sample\_011046841, Unigene52951\_Sample\_011046841, Unigene53582\_Sample\_011046841, Unigene53888\_Sample\_011046841, Unigene54876\_Sample\_011046841, Unigene54982\_Sample\_011046841, Unigene55850\_Sample\_011046841, Unigene56389\_Sample\_011046841, Unigene56914\_Sample\_011046841, Unigene57298\_Sample\_011046841, Unigene57974\_Sample\_011046841, Unigene58243\_Sample\_011046841, Unigene58447\_Sample\_011046841, Unigene58460\_Sample\_011046841, Unigene58762\_Sample\_011046841, Unigene58906\_Sample\_011046841, Unigene58987\_Sample\_011046841, Unigene59082\_Sample\_011046841, Unigene59235\_Sample\_011046841, Unigene59382\_Sample\_011046841, Unigene59559\_Sample\_011046841, Unigene59705\_Sample\_011046841, Unigene59870\_Sample\_011046841, Unigene59913\_Sample\_011046841, Unigene60015\_Sample\_011046841, Unigene60173\_Sample\_011046841, Unigene60175\_Sample\_011046841, Unigene60498\_Sample\_011046841, Unigene60500\_Sample\_011046841, Unigene60576\_Sample\_011046841, Unigene60640\_Sample\_011046841, Unigene60772\_Sample\_011046841, Unigene60930\_Sample\_011046841, Unigene1414\_Sample\_011046841, Unigene2095\_Sample\_011046841, Unigene2442\_Sample\_011046841, Unigene2588\_Sample\_011046841, Unigene4210\_Sample\_011046841, Unigene7340\_Sample\_011046841, Unigene9971\_Sample\_011046841, Unigene10114\_Sample\_011046841, Unigene11269\_Sample\_011046841, Unigene11912\_Sample\_011046841, Unigene12943\_Sample\_011046841, Unigene13091\_Sample\_011046841, Unigene13329\_Sample\_011046841, Unigene13332\_Sample\_011046841, Unigene13494\_Sample\_011046841, Unigene13656\_Sample\_011046841, Unigene13841\_Sample\_011046841, Unigene13855\_Sample\_011046841, Unigene16650\_Sample\_011046841, Unigene21418\_Sample\_011046841, Unigene23312\_Sample\_011046841, Unigene26850\_Sample\_011046841, Unigene32409\_Sample\_011046841, Unigene32938\_Sample\_011046841, Unigene36251\_Sample\_011046841, Unigene38644\_Sample\_011046841, Unigene40209\_Sample\_011046841, Unigene41574\_Sample\_011046841, Unigene43409\_Sample\_011046841, Unigene45592\_Sample\_011046841, Unigene46219\_Sample\_011046841, Unigene47384\_Sample\_011046841, Unigene47555\_Sample\_011046841, Unigene48274\_Sample\_011046841, Unigene48772\_Sample\_011046841, Unigene48850\_Sample\_011046841, Unigene49320\_Sample\_011046841, Unigene49494\_Sample\_011046841, Unigene50163\_Sample\_011046841, Unigene50676\_Sample\_011046841, Unigene50677\_Sample\_011046841, Unigene50915\_Sample\_011046841, Unigene51782\_Sample\_011046841, Unigene52044\_Sample\_011046841, Unigene52146\_Sample\_011046841, Unigene52976\_Sample\_011046841, Unigene53264\_Sample\_011046841, Unigene53334\_Sample\_011046841, Unigene54683\_Sample\_011046841, Unigene54900\_Sample\_011046841, Unigene54979\_Sample\_011046841, Unigene55719\_Sample\_011046841, Unigene56596\_Sample\_011046841, Unigene56681\_Sample\_011046841, Unigene56798\_Sample\_011046841, Unigene57566\_Sample\_011046841, Unigene58042\_Sample\_011046841, Unigene58083\_Sample\_011046841, Unigene58209\_Sample\_011046841, Unigene58241\_Sample\_011046841, Unigene58551\_Sample\_011046841, Unigene59001\_Sample\_011046841, Unigene59095\_Sample\_011046841, Unigene59704\_Sample\_011046841, Unigene59822\_Sample\_011046841, Unigene59902\_Sample\_011046841, Unigene60006\_Sample\_011046841, Unigene60181\_Sample\_011046841, Unigene60271\_Sample\_011046841, Unigene60655\_Sample\_011046841, Unigene3070\_Sample\_011046841, Unigene5972\_Sample\_011046841, Unigene7339\_Sample\_011046841, Unigene8813\_Sample\_011046841, Unigene9967\_Sample\_011046841, Unigene13261\_Sample\_011046841, Unigene13958\_Sample\_011046841, Unigene16993\_Sample\_011046841, Unigene19285\_Sample\_011046841, Unigene19453\_Sample\_011046841, Unigene21239\_Sample\_011046841, Unigene23751\_Sample\_011046841, Unigene28658\_Sample\_011046841, Unigene29578\_Sample\_011046841, Unigene30223\_Sample\_011046841, Unigene30954\_Sample\_011046841, Unigene34582\_Sample\_011046841, Unigene35184\_Sample\_011046841, Unigene36373\_Sample\_011046841, Unigene38773\_Sample\_011046841, Unigene39422\_Sample\_011046841, Unigene44178\_Sample\_011046841, Unigene46930\_Sample\_011046841, Unigene47299\_Sample\_011046841, Unigene48530\_Sample\_011046841, Unigene49909\_Sample\_011046841, Unigene52294\_Sample\_011046841, Unigene53168\_Sample\_011046841, Unigene54191\_Sample\_011046841, Unigene54593\_Sample\_011046841, Unigene56456\_Sample\_011046841, Unigene57158\_Sample\_011046841, Unigene57634\_Sample\_011046841, Unigene57775\_Sample\_011046841, Unigene58035\_Sample\_011046841, Unigene58818\_Sample\_011046841, Unigene59377\_Sample\_011046841, Unigene60512\_Sample\_011046841, Unigene60765\_Sample\_011046841, Unigene4034\_Sample\_011046841, Unigene6435\_Sample\_011046841, Unigene8670\_Sample\_011046841, Unigene11520\_Sample\_011046841, Unigene13853\_Sample\_011046841, Unigene19951\_Sample\_011046841, Unigene28860\_Sample\_011046841, Unigene30184\_Sample\_011046841, Unigene32964\_Sample\_011046841, Unigene35468\_Sample\_011046841, Unigene42339\_Sample\_011046841, Unigene44532\_Sample\_011046841, Unigene46684\_Sample\_011046841, Unigene48046\_Sample\_011046841, Unigene49022\_Sample\_011046841, Unigene49878\_Sample\_011046841, Unigene52181\_Sample\_011046841, Unigene53534\_Sample\_011046841, Unigene53642\_Sample\_011046841, Unigene56300\_Sample\_011046841, Unigene56496\_Sample\_011046841, Unigene57529\_Sample\_011046841, Unigene59711\_Sample\_011046841, Unigene59998\_Sample\_011046841, Unigene60863\_Sample\_011046841, Unigene29346\_Sample\_011046841, Unigene42144\_Sample\_011046841, Unigene55486\_Sample\_011046841, Unigene58954\_Sample\_011046841, Unigene1107\_Sample\_011046841, Unigene36872\_Sample\_011046841, Unigene38674\_Sample\_011046841, Unigene40162\_Sample\_011046841, Unigene43878\_Sample\_011046841, Unigene47967\_Sample\_011046841, Unigene54809\_Sample\_011046841, Unigene56051\_Sample\_011046841, Unigene57530\_Sample\_011046841, Unigene15792\_Sample\_011046841, Unigene51301\_Sample\_011046841, Unigene53030\_Sample\_011046841, Unigene55286\_Sample\_011046841, Unigene55389\_Sample\_011046841, Unigene58446\_Sample\_011046841, Unigene59527\_Sample\_011046841, Unigene60792\_Sample\_011046841, Unigene421\_Sample\_011046841, Unigene17843\_Sample\_011046841, Unigene29318\_Sample\_011046841, Unigene45798\_Sample\_011046841, Unigene47054\_Sample\_011046841, Unigene47560\_Sample\_011046841, Unigene50664\_Sample\_011046841, Unigene53019\_Sample\_011046841, Unigene58176\_Sample\_011046841, Unigene58332\_Sample\_011046841, Unigene5594\_Sample\_011046841, Unigene7431\_Sample\_011046841, Unigene16055\_Sample\_011046841, Unigene19816\_Sample\_011046841, Unigene24508\_Sample\_011046841, Unigene36385\_Sample\_011046841, Unigene37613\_Sample\_011046841, Unigene38948\_Sample\_011046841, Unigene43721\_Sample\_011046841, Unigene50966\_Sample\_011046841, Unigene52667\_Sample\_011046841, Unigene53995\_Sample\_011046841, Unigene3490\_Sample\_011046841, Unigene24961\_Sample\_011046841, Unigene26878\_Sample\_011046841, Unigene32470\_Sample\_011046841, Unigene41085\_Sample\_011046841, Unigene42047\_Sample\_011046841, Unigene42580\_Sample\_011046841, Unigene44838\_Sample\_011046841, Unigene47686\_Sample\_011046841, Unigene47910\_Sample\_011046841, Unigene48977\_Sample\_011046841, Unigene50832\_Sample\_011046841, Unigene55126\_Sample\_011046841, Unigene17004\_Sample\_011046841, Unigene46495\_Sample\_011046841, Unigene47083\_Sample\_011046841, Unigene50989\_Sample\_011046841, Unigene51325\_Sample\_011046841, Unigene43437\_Sample\_011046841, Unigene19717\_Sample\_011046841, Unigene20596\_Sample\_011046841, Unigene43718\_Sample\_011046841, Unigene48928\_Sample\_011046841, Unigene51380\_Sample\_011046841, Unigene53845\_Sample\_011046841, Unigene59362\_Sample\_011046841, Unigene17671\_Sample\_011046841, Unigene20770\_Sample\_011046841, Unigene50713\_Sample\_011046841, Unigene54835\_Sample\_011046841, Unigene15058\_Sample\_011046841, Unigene18305\_Sample\_011046841, Unigene41464\_Sample\_011046841, Unigene43367\_Sample\_011046841, Unigene50758\_Sample\_011046841, Unigene19937\_Sample\_011046841, Unigene36078\_Sample\_011046841, Unigene40934\_Sample\_011046841, Unigene37510\_Sample\_011046841, Unigene43097\_Sample\_011046841, Unigene41097\_Sample\_011046841, Unigene46813\_Sample\_011046841, Unigene48121\_Sample\_011046841, Unigene19789\_Sample\_011046841, Unigene21228\_Sample\_011046841, Unigene16813\_Sample\_011046841, Unigene26184\_Sample\_011046841, Unigene10226\_Sample\_011046841, Unigene30474\_Sample\_011046841, Unigene32977\_Sample\_011046841, Unigene18163\_Sample\_011046841, Unigene19596\_Sample\_011046841, Unigene26777\_Sample\_011046841, Unigene39302\_Sample\_011046841 |
| 63 | Ether lipid metabolism Back to summary table | Unigene4732\_Sample\_011046841, Unigene7647\_Sample\_011046841, Unigene9929\_Sample\_011046841, Unigene11999\_Sample\_011046841, Unigene12244\_Sample\_011046841, Unigene12527\_Sample\_011046841, Unigene24991\_Sample\_011046841, Unigene32071\_Sample\_011046841, Unigene39862\_Sample\_011046841, Unigene52585\_Sample\_011046841, Unigene53068\_Sample\_011046841, Unigene59177\_Sample\_011046841, Unigene60462\_Sample\_011046841, Unigene2509\_Sample\_011046841, Unigene5740\_Sample\_011046841, Unigene7069\_Sample\_011046841, Unigene10346\_Sample\_011046841, Unigene12713\_Sample\_011046841, Unigene28804\_Sample\_011046841, Unigene49827\_Sample\_011046841, Unigene52110\_Sample\_011046841, Unigene53037\_Sample\_011046841, Unigene57375\_Sample\_011046841, Unigene60140\_Sample\_011046841, Unigene60450\_Sample\_011046841, Unigene10675\_Sample\_011046841, Unigene11522\_Sample\_011046841, Unigene19793\_Sample\_011046841, Unigene22806\_Sample\_011046841, Unigene5340\_Sample\_011046841, Unigene32652\_Sample\_011046841, Unigene44518\_Sample\_011046841, Unigene45656\_Sample\_011046841, Unigene29070\_Sample\_011046841, Unigene32867\_Sample\_011046841, Unigene54933\_Sample\_011046841, Unigene55520\_Sample\_011046841, Unigene20272\_Sample\_011046841, Unigene34528\_Sample\_011046841, Unigene53609\_Sample\_011046841, Unigene19418\_Sample\_011046841, Unigene38645\_Sample\_011046841, Unigene23701\_Sample\_011046841, Unigene32062\_Sample\_011046841 |
| 64 | Vibrio cholerae infection Back to summary table | Unigene290\_Sample\_011046841, Unigene863\_Sample\_011046841, Unigene1210\_Sample\_011046841, Unigene3199\_Sample\_011046841, Unigene6178\_Sample\_011046841, Unigene7142\_Sample\_011046841, Unigene7143\_Sample\_011046841, Unigene9237\_Sample\_011046841, Unigene10511\_Sample\_011046841, Unigene11938\_Sample\_011046841, Unigene13370\_Sample\_011046841, Unigene13789\_Sample\_011046841, Unigene22077\_Sample\_011046841, Unigene25745\_Sample\_011046841, Unigene27010\_Sample\_011046841, Unigene40230\_Sample\_011046841, Unigene41207\_Sample\_011046841, Unigene43500\_Sample\_011046841, Unigene46525\_Sample\_011046841, Unigene48836\_Sample\_011046841, Unigene50548\_Sample\_011046841, Unigene55020\_Sample\_011046841, Unigene56437\_Sample\_011046841, Unigene57236\_Sample\_011046841, Unigene57381\_Sample\_011046841, Unigene57947\_Sample\_011046841, Unigene57973\_Sample\_011046841, Unigene58508\_Sample\_011046841, Unigene60789\_Sample\_011046841, Unigene2578\_Sample\_011046841, Unigene2588\_Sample\_011046841, Unigene3363\_Sample\_011046841, Unigene4916\_Sample\_011046841, Unigene5744\_Sample\_011046841, Unigene8020\_Sample\_011046841, Unigene9572\_Sample\_011046841, Unigene9867\_Sample\_011046841, Unigene12176\_Sample\_011046841, Unigene13881\_Sample\_011046841, Unigene26850\_Sample\_011046841, Unigene33297\_Sample\_011046841, Unigene36873\_Sample\_011046841, Unigene38644\_Sample\_011046841, Unigene44425\_Sample\_011046841, Unigene47655\_Sample\_011046841, Unigene49697\_Sample\_011046841, Unigene50585\_Sample\_011046841, Unigene50676\_Sample\_011046841, Unigene51269\_Sample\_011046841, Unigene51782\_Sample\_011046841, Unigene58238\_Sample\_011046841, Unigene59742\_Sample\_011046841, Unigene60225\_Sample\_011046841, Unigene60880\_Sample\_011046841, Unigene4137\_Sample\_011046841, Unigene7339\_Sample\_011046841, Unigene19285\_Sample\_011046841, Unigene25498\_Sample\_011046841, Unigene35184\_Sample\_011046841, Unigene47562\_Sample\_011046841, Unigene58818\_Sample\_011046841, Unigene8139\_Sample\_011046841, Unigene28860\_Sample\_011046841, Unigene48046\_Sample\_011046841, Unigene58610\_Sample\_011046841, Unigene59688\_Sample\_011046841, Unigene59998\_Sample\_011046841, Unigene30190\_Sample\_011046841, Unigene45952\_Sample\_011046841, Unigene58199\_Sample\_011046841, Unigene2942\_Sample\_011046841, Unigene9329\_Sample\_011046841, Unigene43782\_Sample\_011046841, Unigene51429\_Sample\_011046841, Unigene58229\_Sample\_011046841, Unigene475\_Sample\_011046841, Unigene17825\_Sample\_011046841, Unigene46399\_Sample\_011046841, Unigene48742\_Sample\_011046841, Unigene54625\_Sample\_011046841, Unigene56139\_Sample\_011046841, Unigene58176\_Sample\_011046841, Unigene16098\_Sample\_011046841, Unigene2110\_Sample\_011046841, Unigene7431\_Sample\_011046841, Unigene16055\_Sample\_011046841, Unigene21201\_Sample\_011046841, Unigene38948\_Sample\_011046841, Unigene43721\_Sample\_011046841, Unigene54823\_Sample\_011046841, Unigene55041\_Sample\_011046841, Unigene12521\_Sample\_011046841, Unigene15705\_Sample\_011046841, Unigene23831\_Sample\_011046841, Unigene24961\_Sample\_011046841, Unigene26878\_Sample\_011046841, Unigene28288\_Sample\_011046841, Unigene32470\_Sample\_011046841, Unigene43099\_Sample\_011046841, Unigene47872\_Sample\_011046841, Unigene48977\_Sample\_011046841, Unigene52283\_Sample\_011046841, Unigene53018\_Sample\_011046841, Unigene10863\_Sample\_011046841, Unigene31066\_Sample\_011046841, Unigene46495\_Sample\_011046841, Unigene12324\_Sample\_011046841, Unigene19717\_Sample\_011046841, Unigene28763\_Sample\_011046841, Unigene44021\_Sample\_011046841, Unigene47487\_Sample\_011046841, Unigene15058\_Sample\_011046841, Unigene35082\_Sample\_011046841, Unigene58257\_Sample\_011046841, Unigene14248\_Sample\_011046841, Unigene48121\_Sample\_011046841, Unigene29441\_Sample\_011046841, Unigene38143\_Sample\_011046841, Unigene42241\_Sample\_011046841, Unigene36948\_Sample\_011046841, Unigene37372\_Sample\_011046841, Unigene7048\_Sample\_011046841 |
| 65 | Melanogenesis Back to summary table | Unigene3199\_Sample\_011046841, Unigene4963\_Sample\_011046841, Unigene5012\_Sample\_011046841, Unigene5178\_Sample\_011046841, Unigene6178\_Sample\_011046841, Unigene6605\_Sample\_011046841, Unigene8859\_Sample\_011046841, Unigene8881\_Sample\_011046841, Unigene9668\_Sample\_011046841, Unigene10640\_Sample\_011046841, Unigene12213\_Sample\_011046841, Unigene13346\_Sample\_011046841, Unigene13481\_Sample\_011046841, Unigene13789\_Sample\_011046841, Unigene13872\_Sample\_011046841, Unigene37440\_Sample\_011046841, Unigene39094\_Sample\_011046841, Unigene40230\_Sample\_011046841, Unigene42704\_Sample\_011046841, Unigene45114\_Sample\_011046841, Unigene52106\_Sample\_011046841, Unigene56886\_Sample\_011046841, Unigene58307\_Sample\_011046841, Unigene58460\_Sample\_011046841, Unigene59022\_Sample\_011046841, Unigene59151\_Sample\_011046841, Unigene59189\_Sample\_011046841, Unigene60789\_Sample\_011046841, Unigene1345\_Sample\_011046841, Unigene1378\_Sample\_011046841, Unigene3363\_Sample\_011046841, Unigene4993\_Sample\_011046841, Unigene5744\_Sample\_011046841, Unigene8641\_Sample\_011046841, Unigene9867\_Sample\_011046841, Unigene11134\_Sample\_011046841, Unigene21962\_Sample\_011046841, Unigene37679\_Sample\_011046841, Unigene40989\_Sample\_011046841, Unigene41574\_Sample\_011046841, Unigene42561\_Sample\_011046841, Unigene42676\_Sample\_011046841, Unigene42820\_Sample\_011046841, Unigene47555\_Sample\_011046841, Unigene48337\_Sample\_011046841, Unigene50039\_Sample\_011046841, Unigene53186\_Sample\_011046841, Unigene54525\_Sample\_011046841, Unigene54900\_Sample\_011046841, Unigene55948\_Sample\_011046841, Unigene56177\_Sample\_011046841, Unigene58149\_Sample\_011046841, Unigene59660\_Sample\_011046841, Unigene59742\_Sample\_011046841, Unigene60190\_Sample\_011046841, Unigene60219\_Sample\_011046841, Unigene60880\_Sample\_011046841, Unigene1526\_Sample\_011046841, Unigene8813\_Sample\_011046841, Unigene9726\_Sample\_011046841, Unigene9831\_Sample\_011046841, Unigene28658\_Sample\_011046841, Unigene32259\_Sample\_011046841, Unigene44316\_Sample\_011046841, Unigene51171\_Sample\_011046841, Unigene52303\_Sample\_011046841, Unigene54574\_Sample\_011046841, Unigene56160\_Sample\_011046841, Unigene8139\_Sample\_011046841, Unigene32964\_Sample\_011046841, Unigene47860\_Sample\_011046841, Unigene57387\_Sample\_011046841, Unigene57675\_Sample\_011046841, Unigene60102\_Sample\_011046841, Unigene30190\_Sample\_011046841, Unigene45708\_Sample\_011046841, Unigene57665\_Sample\_011046841, Unigene58199\_Sample\_011046841, Unigene2942\_Sample\_011046841, Unigene9329\_Sample\_011046841, Unigene38302\_Sample\_011046841, Unigene43782\_Sample\_011046841, Unigene51429\_Sample\_011046841, Unigene23818\_Sample\_011046841, Unigene29318\_Sample\_011046841, Unigene48742\_Sample\_011046841, Unigene52499\_Sample\_011046841, Unigene52590\_Sample\_011046841, Unigene56139\_Sample\_011046841, Unigene57117\_Sample\_011046841, Unigene16098\_Sample\_011046841, Unigene2110\_Sample\_011046841, Unigene21201\_Sample\_011046841, Unigene53352\_Sample\_011046841, Unigene55041\_Sample\_011046841, Unigene12521\_Sample\_011046841, Unigene15705\_Sample\_011046841, Unigene23831\_Sample\_011046841, Unigene28288\_Sample\_011046841, Unigene42293\_Sample\_011046841, Unigene43099\_Sample\_011046841, Unigene47872\_Sample\_011046841, Unigene47910\_Sample\_011046841, Unigene59748\_Sample\_011046841, Unigene10863\_Sample\_011046841, Unigene19947\_Sample\_011046841, Unigene53893\_Sample\_011046841, Unigene12324\_Sample\_011046841, Unigene16539\_Sample\_011046841, Unigene28384\_Sample\_011046841, Unigene44021\_Sample\_011046841, Unigene47487\_Sample\_011046841, Unigene44354\_Sample\_011046841, Unigene21539\_Sample\_011046841, Unigene35082\_Sample\_011046841, Unigene14248\_Sample\_011046841, Unigene46813\_Sample\_011046841, Unigene29441\_Sample\_011046841, Unigene14181\_Sample\_011046841, Unigene30474\_Sample\_011046841, Unigene7048\_Sample\_011046841 |
| 66 | Metabolic pathways (no map in kegg database) Back to summary table | Unigene385\_Sample\_011046841, Unigene1004\_Sample\_011046841, Unigene1010\_Sample\_011046841, Unigene1049\_Sample\_011046841, Unigene1129\_Sample\_011046841, Unigene1552\_Sample\_011046841, Unigene2370\_Sample\_011046841, Unigene2488\_Sample\_011046841, Unigene2963\_Sample\_011046841, Unigene3004\_Sample\_011046841, Unigene3621\_Sample\_011046841, Unigene4130\_Sample\_011046841, Unigene4732\_Sample\_011046841, Unigene4863\_Sample\_011046841, Unigene5169\_Sample\_011046841, Unigene5554\_Sample\_011046841, Unigene5865\_Sample\_011046841, Unigene6127\_Sample\_011046841, Unigene6557\_Sample\_011046841, Unigene7023\_Sample\_011046841, Unigene7029\_Sample\_011046841, Unigene7143\_Sample\_011046841, Unigene7331\_Sample\_011046841, Unigene7516\_Sample\_011046841, Unigene7559\_Sample\_011046841, Unigene7636\_Sample\_011046841, Unigene7647\_Sample\_011046841, Unigene7922\_Sample\_011046841, Unigene8282\_Sample\_011046841, Unigene8336\_Sample\_011046841, Unigene8411\_Sample\_011046841, Unigene8859\_Sample\_011046841, Unigene8871\_Sample\_011046841, Unigene9098\_Sample\_011046841, Unigene9141\_Sample\_011046841, Unigene9172\_Sample\_011046841, Unigene9929\_Sample\_011046841, Unigene10379\_Sample\_011046841, Unigene10690\_Sample\_011046841, Unigene10700\_Sample\_011046841, Unigene11290\_Sample\_011046841, Unigene11409\_Sample\_011046841, Unigene11580\_Sample\_011046841, Unigene11911\_Sample\_011046841, Unigene11999\_Sample\_011046841, Unigene12076\_Sample\_011046841, Unigene12082\_Sample\_011046841, Unigene12224\_Sample\_011046841, Unigene12244\_Sample\_011046841, Unigene12300\_Sample\_011046841, Unigene12384\_Sample\_011046841, Unigene12420\_Sample\_011046841, Unigene12504\_Sample\_011046841, Unigene12527\_Sample\_011046841, Unigene12543\_Sample\_011046841, Unigene12594\_Sample\_011046841, Unigene12850\_Sample\_011046841, Unigene12982\_Sample\_011046841, Unigene13211\_Sample\_011046841, Unigene13344\_Sample\_011046841, Unigene13500\_Sample\_011046841, Unigene13526\_Sample\_011046841, Unigene13666\_Sample\_011046841, Unigene13809\_Sample\_011046841, Unigene13838\_Sample\_011046841, Unigene18327\_Sample\_011046841, Unigene18832\_Sample\_011046841, Unigene19441\_Sample\_011046841, Unigene19673\_Sample\_011046841, Unigene20191\_Sample\_011046841, Unigene20686\_Sample\_011046841, Unigene20907\_Sample\_011046841, Unigene21108\_Sample\_011046841, Unigene22611\_Sample\_011046841, Unigene22965\_Sample\_011046841, Unigene23040\_Sample\_011046841, Unigene23914\_Sample\_011046841, Unigene24105\_Sample\_011046841, Unigene24123\_Sample\_011046841, Unigene24171\_Sample\_011046841, Unigene24706\_Sample\_011046841, Unigene24991\_Sample\_011046841, Unigene25547\_Sample\_011046841, Unigene25984\_Sample\_011046841, Unigene26460\_Sample\_011046841, Unigene26779\_Sample\_011046841, Unigene27550\_Sample\_011046841, Unigene27562\_Sample\_011046841, Unigene28357\_Sample\_011046841, Unigene28781\_Sample\_011046841, Unigene30736\_Sample\_011046841, Unigene32071\_Sample\_011046841, Unigene32814\_Sample\_011046841, Unigene33884\_Sample\_011046841, Unigene34404\_Sample\_011046841, Unigene34724\_Sample\_011046841, Unigene34739\_Sample\_011046841, Unigene34881\_Sample\_011046841, Unigene36435\_Sample\_011046841, Unigene36850\_Sample\_011046841, Unigene38010\_Sample\_011046841, Unigene38329\_Sample\_011046841, Unigene38608\_Sample\_011046841, Unigene38930\_Sample\_011046841, Unigene39159\_Sample\_011046841, Unigene39454\_Sample\_011046841, Unigene39755\_Sample\_011046841, Unigene39862\_Sample\_011046841, Unigene39884\_Sample\_011046841, Unigene40350\_Sample\_011046841, Unigene40878\_Sample\_011046841, Unigene41250\_Sample\_011046841, Unigene41379\_Sample\_011046841, Unigene41612\_Sample\_011046841, Unigene42392\_Sample\_011046841, Unigene42615\_Sample\_011046841, Unigene42838\_Sample\_011046841, Unigene43461\_Sample\_011046841, Unigene43500\_Sample\_011046841, Unigene43544\_Sample\_011046841, Unigene43552\_Sample\_011046841, Unigene43574\_Sample\_011046841, Unigene43894\_Sample\_011046841, Unigene44562\_Sample\_011046841, Unigene44603\_Sample\_011046841, Unigene44949\_Sample\_011046841, Unigene44985\_Sample\_011046841, Unigene45012\_Sample\_011046841, Unigene45674\_Sample\_011046841, Unigene45744\_Sample\_011046841, Unigene46170\_Sample\_011046841, Unigene46439\_Sample\_011046841, Unigene46525\_Sample\_011046841, Unigene46621\_Sample\_011046841, Unigene47008\_Sample\_011046841, Unigene47226\_Sample\_011046841, Unigene47273\_Sample\_011046841, Unigene47448\_Sample\_011046841, Unigene47763\_Sample\_011046841, Unigene47882\_Sample\_011046841, Unigene48252\_Sample\_011046841, Unigene48532\_Sample\_011046841, Unigene49101\_Sample\_011046841, Unigene49303\_Sample\_011046841, Unigene49360\_Sample\_011046841, Unigene49383\_Sample\_011046841, Unigene49752\_Sample\_011046841, Unigene49914\_Sample\_011046841, Unigene49992\_Sample\_011046841, Unigene50538\_Sample\_011046841, Unigene50548\_Sample\_011046841, Unigene50553\_Sample\_011046841, Unigene50631\_Sample\_011046841, Unigene50827\_Sample\_011046841, Unigene50938\_Sample\_011046841, Unigene51029\_Sample\_011046841, Unigene51460\_Sample\_011046841, Unigene51527\_Sample\_011046841, Unigene51592\_Sample\_011046841, Unigene51618\_Sample\_011046841, Unigene51739\_Sample\_011046841, Unigene52407\_Sample\_011046841, Unigene52585\_Sample\_011046841, Unigene52731\_Sample\_011046841, Unigene52799\_Sample\_011046841, Unigene53068\_Sample\_011046841, Unigene53069\_Sample\_011046841, Unigene53138\_Sample\_011046841, Unigene53353\_Sample\_011046841, Unigene53833\_Sample\_011046841, Unigene53862\_Sample\_011046841, Unigene53919\_Sample\_011046841, Unigene53930\_Sample\_011046841, Unigene54014\_Sample\_011046841, Unigene54025\_Sample\_011046841, Unigene54097\_Sample\_011046841, Unigene54492\_Sample\_011046841, Unigene54550\_Sample\_011046841, Unigene54779\_Sample\_011046841, Unigene54985\_Sample\_011046841, Unigene55020\_Sample\_011046841, Unigene55348\_Sample\_011046841, Unigene55376\_Sample\_011046841, Unigene55779\_Sample\_011046841, Unigene55892\_Sample\_011046841, Unigene55978\_Sample\_011046841, Unigene55981\_Sample\_011046841, Unigene56049\_Sample\_011046841, Unigene56182\_Sample\_011046841, Unigene56276\_Sample\_011046841, Unigene56295\_Sample\_011046841, Unigene56437\_Sample\_011046841, Unigene56475\_Sample\_011046841, Unigene56495\_Sample\_011046841, Unigene57000\_Sample\_011046841, Unigene57020\_Sample\_011046841, Unigene57236\_Sample\_011046841, Unigene57381\_Sample\_011046841, Unigene57382\_Sample\_011046841, Unigene57575\_Sample\_011046841, Unigene57712\_Sample\_011046841, Unigene57770\_Sample\_011046841, Unigene57947\_Sample\_011046841, Unigene57973\_Sample\_011046841, Unigene58015\_Sample\_011046841, Unigene58038\_Sample\_011046841, Unigene58183\_Sample\_011046841, Unigene58303\_Sample\_011046841, Unigene58408\_Sample\_011046841, Unigene58653\_Sample\_011046841, Unigene58685\_Sample\_011046841, Unigene58779\_Sample\_011046841, Unigene58911\_Sample\_011046841, Unigene58934\_Sample\_011046841, Unigene58992\_Sample\_011046841, Unigene59038\_Sample\_011046841, Unigene59093\_Sample\_011046841, Unigene59158\_Sample\_011046841, Unigene59177\_Sample\_011046841, Unigene59189\_Sample\_011046841, Unigene59190\_Sample\_011046841, Unigene59226\_Sample\_011046841, Unigene59442\_Sample\_011046841, Unigene59445\_Sample\_011046841, Unigene59568\_Sample\_011046841, Unigene59595\_Sample\_011046841, Unigene59609\_Sample\_011046841, Unigene59673\_Sample\_011046841, Unigene59763\_Sample\_011046841, Unigene59828\_Sample\_011046841, Unigene59842\_Sample\_011046841, Unigene59904\_Sample\_011046841, Unigene59941\_Sample\_011046841, Unigene59965\_Sample\_011046841, Unigene59971\_Sample\_011046841, Unigene59984\_Sample\_011046841, Unigene59993\_Sample\_011046841, Unigene60069\_Sample\_011046841, Unigene60147\_Sample\_011046841, Unigene60235\_Sample\_011046841, Unigene60274\_Sample\_011046841, Unigene60298\_Sample\_011046841, Unigene60304\_Sample\_011046841, Unigene60317\_Sample\_011046841, Unigene60361\_Sample\_011046841, Unigene60429\_Sample\_011046841, Unigene60431\_Sample\_011046841, Unigene60437\_Sample\_011046841, Unigene60462\_Sample\_011046841, Unigene60564\_Sample\_011046841, Unigene60677\_Sample\_011046841, Unigene60727\_Sample\_011046841, Unigene60759\_Sample\_011046841, Unigene60764\_Sample\_011046841, Unigene60809\_Sample\_011046841, Unigene60830\_Sample\_011046841, Unigene60879\_Sample\_011046841, Unigene1012\_Sample\_011046841, Unigene1158\_Sample\_011046841, Unigene1164\_Sample\_011046841, Unigene1754\_Sample\_011046841, Unigene2320\_Sample\_011046841, Unigene2349\_Sample\_011046841, Unigene2385\_Sample\_011046841, Unigene2509\_Sample\_011046841, Unigene2913\_Sample\_011046841, Unigene3929\_Sample\_011046841, Unigene3975\_Sample\_011046841, Unigene4497\_Sample\_011046841, Unigene4616\_Sample\_011046841, Unigene4741\_Sample\_011046841, Unigene4916\_Sample\_011046841, Unigene5074\_Sample\_011046841, Unigene5150\_Sample\_011046841, Unigene5329\_Sample\_011046841, Unigene5336\_Sample\_011046841, Unigene5382\_Sample\_011046841, Unigene5657\_Sample\_011046841, Unigene5740\_Sample\_011046841, Unigene5777\_Sample\_011046841, Unigene5967\_Sample\_011046841, Unigene6034\_Sample\_011046841, Unigene6065\_Sample\_011046841, Unigene6377\_Sample\_011046841, Unigene6500\_Sample\_011046841, Unigene6600\_Sample\_011046841, Unigene6707\_Sample\_011046841, Unigene6828\_Sample\_011046841, Unigene7065\_Sample\_011046841, Unigene7069\_Sample\_011046841, Unigene7457\_Sample\_011046841, Unigene7507\_Sample\_011046841, Unigene7693\_Sample\_011046841, Unigene8020\_Sample\_011046841, Unigene8272\_Sample\_011046841, Unigene8938\_Sample\_011046841, Unigene8971\_Sample\_011046841, Unigene9038\_Sample\_011046841, Unigene9052\_Sample\_011046841, Unigene9274\_Sample\_011046841, Unigene9455\_Sample\_011046841, Unigene9572\_Sample\_011046841, Unigene9752\_Sample\_011046841, Unigene9769\_Sample\_011046841, Unigene10037\_Sample\_011046841, Unigene10229\_Sample\_011046841, Unigene10327\_Sample\_011046841, Unigene10346\_Sample\_011046841, Unigene10448\_Sample\_011046841, Unigene10517\_Sample\_011046841, Unigene10701\_Sample\_011046841, Unigene10739\_Sample\_011046841, Unigene11004\_Sample\_011046841, Unigene11030\_Sample\_011046841, Unigene11188\_Sample\_011046841, Unigene11283\_Sample\_011046841, Unigene11436\_Sample\_011046841, Unigene11726\_Sample\_011046841, Unigene12081\_Sample\_011046841, Unigene12087\_Sample\_011046841, Unigene12345\_Sample\_011046841, Unigene12713\_Sample\_011046841, Unigene13096\_Sample\_011046841, Unigene13416\_Sample\_011046841, Unigene13474\_Sample\_011046841, Unigene13694\_Sample\_011046841, Unigene13915\_Sample\_011046841, Unigene13979\_Sample\_011046841, Unigene14236\_Sample\_011046841, Unigene14656\_Sample\_011046841, Unigene15547\_Sample\_011046841, Unigene16545\_Sample\_011046841, Unigene16727\_Sample\_011046841, Unigene17132\_Sample\_011046841, Unigene18937\_Sample\_011046841, Unigene19438\_Sample\_011046841, Unigene20606\_Sample\_011046841, Unigene21319\_Sample\_011046841, Unigene21594\_Sample\_011046841, Unigene21670\_Sample\_011046841, Unigene21784\_Sample\_011046841, Unigene21962\_Sample\_011046841, Unigene22323\_Sample\_011046841, Unigene22428\_Sample\_011046841, Unigene22548\_Sample\_011046841, Unigene22564\_Sample\_011046841, Unigene23629\_Sample\_011046841, Unigene24754\_Sample\_011046841, Unigene24894\_Sample\_011046841, Unigene24943\_Sample\_011046841, Unigene26220\_Sample\_011046841, Unigene26737\_Sample\_011046841, Unigene27510\_Sample\_011046841, Unigene27682\_Sample\_011046841, Unigene28804\_Sample\_011046841, Unigene30699\_Sample\_011046841, Unigene31025\_Sample\_011046841, Unigene31490\_Sample\_011046841, Unigene31641\_Sample\_011046841, Unigene32116\_Sample\_011046841, Unigene32364\_Sample\_011046841, Unigene33132\_Sample\_011046841, Unigene34114\_Sample\_011046841, Unigene34145\_Sample\_011046841, Unigene34403\_Sample\_011046841, Unigene34858\_Sample\_011046841, Unigene34859\_Sample\_011046841, Unigene34911\_Sample\_011046841, Unigene35134\_Sample\_011046841, Unigene35149\_Sample\_011046841, Unigene35290\_Sample\_011046841, Unigene35814\_Sample\_011046841, Unigene36036\_Sample\_011046841, Unigene37679\_Sample\_011046841, Unigene38716\_Sample\_011046841, Unigene39223\_Sample\_011046841, Unigene39621\_Sample\_011046841, Unigene39709\_Sample\_011046841, Unigene39854\_Sample\_011046841, Unigene41024\_Sample\_011046841, Unigene41171\_Sample\_011046841, Unigene41238\_Sample\_011046841, Unigene42399\_Sample\_011046841, Unigene42733\_Sample\_011046841, Unigene42738\_Sample\_011046841, Unigene42742\_Sample\_011046841, Unigene42994\_Sample\_011046841, Unigene43426\_Sample\_011046841, Unigene43704\_Sample\_011046841, Unigene43906\_Sample\_011046841, Unigene44425\_Sample\_011046841, Unigene44557\_Sample\_011046841, Unigene45045\_Sample\_011046841, Unigene45172\_Sample\_011046841, Unigene45531\_Sample\_011046841, Unigene45547\_Sample\_011046841, Unigene45556\_Sample\_011046841, Unigene45561\_Sample\_011046841, Unigene45695\_Sample\_011046841, Unigene45816\_Sample\_011046841, Unigene46957\_Sample\_011046841, Unigene47198\_Sample\_011046841, Unigene47221\_Sample\_011046841, Unigene47414\_Sample\_011046841, Unigene47506\_Sample\_011046841, Unigene47655\_Sample\_011046841, Unigene47859\_Sample\_011046841, Unigene48003\_Sample\_011046841, Unigene48094\_Sample\_011046841, Unigene48303\_Sample\_011046841, Unigene48423\_Sample\_011046841, Unigene48569\_Sample\_011046841, Unigene48578\_Sample\_011046841, Unigene48609\_Sample\_011046841, Unigene48747\_Sample\_011046841, Unigene48857\_Sample\_011046841, Unigene48881\_Sample\_011046841, Unigene48927\_Sample\_011046841, Unigene49486\_Sample\_011046841, Unigene49640\_Sample\_011046841, Unigene49697\_Sample\_011046841, Unigene49713\_Sample\_011046841, Unigene49729\_Sample\_011046841, Unigene49803\_Sample\_011046841, Unigene49827\_Sample\_011046841, Unigene49854\_Sample\_011046841, Unigene49859\_Sample\_011046841, Unigene50014\_Sample\_011046841, Unigene50108\_Sample\_011046841, Unigene50242\_Sample\_011046841, Unigene50433\_Sample\_011046841, Unigene50562\_Sample\_011046841, Unigene50585\_Sample\_011046841, Unigene50726\_Sample\_011046841, Unigene50981\_Sample\_011046841, Unigene51041\_Sample\_011046841, Unigene51069\_Sample\_011046841, Unigene51256\_Sample\_011046841, Unigene51453\_Sample\_011046841, Unigene51456\_Sample\_011046841, Unigene51598\_Sample\_011046841, Unigene51852\_Sample\_011046841, Unigene51857\_Sample\_011046841, Unigene51902\_Sample\_011046841, Unigene51952\_Sample\_011046841, Unigene52110\_Sample\_011046841, Unigene52322\_Sample\_011046841, Unigene52423\_Sample\_011046841, Unigene52526\_Sample\_011046841, Unigene52535\_Sample\_011046841, Unigene52600\_Sample\_011046841, Unigene52614\_Sample\_011046841, Unigene52769\_Sample\_011046841, Unigene52888\_Sample\_011046841, Unigene52903\_Sample\_011046841, Unigene52972\_Sample\_011046841, Unigene53002\_Sample\_011046841, Unigene53010\_Sample\_011046841, Unigene53037\_Sample\_011046841, Unigene53115\_Sample\_011046841, Unigene53210\_Sample\_011046841, Unigene53305\_Sample\_011046841, Unigene53327\_Sample\_011046841, Unigene53360\_Sample\_011046841, Unigene53419\_Sample\_011046841, Unigene53427\_Sample\_011046841, Unigene53524\_Sample\_011046841, Unigene53738\_Sample\_011046841, Unigene53773\_Sample\_011046841, Unigene54081\_Sample\_011046841, Unigene54468\_Sample\_011046841, Unigene54563\_Sample\_011046841, Unigene54643\_Sample\_011046841, Unigene54707\_Sample\_011046841, Unigene54860\_Sample\_011046841, Unigene54958\_Sample\_011046841, Unigene55039\_Sample\_011046841, Unigene55065\_Sample\_011046841, Unigene55076\_Sample\_011046841, Unigene55255\_Sample\_011046841, Unigene55269\_Sample\_011046841, Unigene55275\_Sample\_011046841, Unigene55329\_Sample\_011046841, Unigene55357\_Sample\_011046841, Unigene55489\_Sample\_011046841, Unigene55549\_Sample\_011046841, Unigene55621\_Sample\_011046841, Unigene55661\_Sample\_011046841, Unigene55866\_Sample\_011046841, Unigene55886\_Sample\_011046841, Unigene55954\_Sample\_011046841, Unigene55994\_Sample\_011046841, Unigene56100\_Sample\_011046841, Unigene56185\_Sample\_011046841, Unigene56266\_Sample\_011046841, Unigene56298\_Sample\_011046841, Unigene56333\_Sample\_011046841, Unigene56337\_Sample\_011046841, Unigene56427\_Sample\_011046841, Unigene56529\_Sample\_011046841, Unigene56579\_Sample\_011046841, Unigene56690\_Sample\_011046841, Unigene56753\_Sample\_011046841, Unigene56795\_Sample\_011046841, Unigene56799\_Sample\_011046841, Unigene56891\_Sample\_011046841, Unigene56933\_Sample\_011046841, Unigene56952\_Sample\_011046841, Unigene57056\_Sample\_011046841, Unigene57074\_Sample\_011046841, Unigene57122\_Sample\_011046841, Unigene57300\_Sample\_011046841, Unigene57359\_Sample\_011046841, Unigene57375\_Sample\_011046841, Unigene57397\_Sample\_011046841, Unigene57560\_Sample\_011046841, Unigene57569\_Sample\_011046841, Unigene57608\_Sample\_011046841, Unigene57699\_Sample\_011046841, Unigene57706\_Sample\_011046841, Unigene57710\_Sample\_011046841, Unigene57741\_Sample\_011046841, Unigene58018\_Sample\_011046841, Unigene58042\_Sample\_011046841, Unigene58152\_Sample\_011046841, Unigene58309\_Sample\_011046841, Unigene58451\_Sample\_011046841, Unigene58482\_Sample\_011046841, Unigene58486\_Sample\_011046841, Unigene58692\_Sample\_011046841, Unigene58721\_Sample\_011046841, Unigene58765\_Sample\_011046841, Unigene58843\_Sample\_011046841, Unigene59236\_Sample\_011046841, Unigene59330\_Sample\_011046841, Unigene59415\_Sample\_011046841, Unigene59502\_Sample\_011046841, Unigene59519\_Sample\_011046841, Unigene59521\_Sample\_011046841, Unigene59524\_Sample\_011046841, Unigene59634\_Sample\_011046841, Unigene59664\_Sample\_011046841, Unigene59714\_Sample\_011046841, Unigene59746\_Sample\_011046841, Unigene59788\_Sample\_011046841, Unigene60019\_Sample\_011046841, Unigene60051\_Sample\_011046841, Unigene60126\_Sample\_011046841, Unigene60140\_Sample\_011046841, Unigene60181\_Sample\_011046841, Unigene60192\_Sample\_011046841, Unigene60199\_Sample\_011046841, Unigene60225\_Sample\_011046841, Unigene60310\_Sample\_011046841, Unigene60319\_Sample\_011046841, Unigene60442\_Sample\_011046841, Unigene60444\_Sample\_011046841, Unigene60450\_Sample\_011046841, Unigene60488\_Sample\_011046841, Unigene60522\_Sample\_011046841, Unigene60574\_Sample\_011046841, Unigene60682\_Sample\_011046841, Unigene60744\_Sample\_011046841, Unigene60822\_Sample\_011046841, Unigene60900\_Sample\_011046841, Unigene60912\_Sample\_011046841, Unigene60919\_Sample\_011046841, Unigene60\_Sample\_011046841, Unigene210\_Sample\_011046841, Unigene967\_Sample\_011046841, Unigene1194\_Sample\_011046841, Unigene1477\_Sample\_011046841, Unigene2189\_Sample\_011046841, Unigene3434\_Sample\_011046841, Unigene4137\_Sample\_011046841, Unigene4987\_Sample\_011046841, Unigene5051\_Sample\_011046841, Unigene5416\_Sample\_011046841, Unigene5708\_Sample\_011046841, Unigene6116\_Sample\_011046841, Unigene6222\_Sample\_011046841, Unigene6227\_Sample\_011046841, Unigene8146\_Sample\_011046841, Unigene8446\_Sample\_011046841, Unigene8728\_Sample\_011046841, Unigene9093\_Sample\_011046841, Unigene9610\_Sample\_011046841, Unigene9806\_Sample\_011046841, Unigene9900\_Sample\_011046841, Unigene10675\_Sample\_011046841, Unigene10699\_Sample\_011046841, Unigene11522\_Sample\_011046841, Unigene11743\_Sample\_011046841, Unigene12951\_Sample\_011046841, Unigene13027\_Sample\_011046841, Unigene16189\_Sample\_011046841, Unigene16357\_Sample\_011046841, Unigene16482\_Sample\_011046841, Unigene16547\_Sample\_011046841, Unigene18000\_Sample\_011046841, Unigene18735\_Sample\_011046841, Unigene18945\_Sample\_011046841, Unigene19169\_Sample\_011046841, Unigene19399\_Sample\_011046841, Unigene19793\_Sample\_011046841, Unigene20854\_Sample\_011046841, Unigene21061\_Sample\_011046841, Unigene21093\_Sample\_011046841, Unigene21191\_Sample\_011046841, Unigene21219\_Sample\_011046841, Unigene22806\_Sample\_011046841, Unigene23548\_Sample\_011046841, Unigene23667\_Sample\_011046841, Unigene23752\_Sample\_011046841, Unigene24351\_Sample\_011046841, Unigene24615\_Sample\_011046841, Unigene25475\_Sample\_011046841, Unigene25596\_Sample\_011046841, Unigene26928\_Sample\_011046841, Unigene27178\_Sample\_011046841, Unigene27188\_Sample\_011046841, Unigene27725\_Sample\_011046841, Unigene27774\_Sample\_011046841, Unigene28097\_Sample\_011046841, Unigene29602\_Sample\_011046841, Unigene29684\_Sample\_011046841, Unigene30072\_Sample\_011046841, Unigene31642\_Sample\_011046841, Unigene31723\_Sample\_011046841, Unigene32177\_Sample\_011046841, Unigene32259\_Sample\_011046841, Unigene32280\_Sample\_011046841, Unigene33337\_Sample\_011046841, Unigene33513\_Sample\_011046841, Unigene33868\_Sample\_011046841, Unigene34269\_Sample\_011046841, Unigene34323\_Sample\_011046841, Unigene34482\_Sample\_011046841, Unigene34810\_Sample\_011046841, Unigene35210\_Sample\_011046841, Unigene35485\_Sample\_011046841, Unigene36181\_Sample\_011046841, Unigene36647\_Sample\_011046841, Unigene37438\_Sample\_011046841, Unigene37646\_Sample\_011046841, Unigene37947\_Sample\_011046841, Unigene38603\_Sample\_011046841, Unigene39126\_Sample\_011046841, Unigene39719\_Sample\_011046841, Unigene40140\_Sample\_011046841, Unigene40555\_Sample\_011046841, Unigene41288\_Sample\_011046841, Unigene41355\_Sample\_011046841, Unigene41499\_Sample\_011046841, Unigene41608\_Sample\_011046841, Unigene42079\_Sample\_011046841, Unigene42105\_Sample\_011046841, Unigene42316\_Sample\_011046841, Unigene42860\_Sample\_011046841, Unigene43178\_Sample\_011046841, Unigene43200\_Sample\_011046841, Unigene43814\_Sample\_011046841, Unigene43998\_Sample\_011046841, Unigene44386\_Sample\_011046841, Unigene44391\_Sample\_011046841, Unigene44834\_Sample\_011046841, Unigene45518\_Sample\_011046841, Unigene45956\_Sample\_011046841, Unigene46704\_Sample\_011046841, Unigene47177\_Sample\_011046841, Unigene47307\_Sample\_011046841, Unigene47319\_Sample\_011046841, Unigene47379\_Sample\_011046841, Unigene47562\_Sample\_011046841, Unigene47654\_Sample\_011046841, Unigene47707\_Sample\_011046841, Unigene47959\_Sample\_011046841, Unigene47997\_Sample\_011046841, Unigene48448\_Sample\_011046841, Unigene48708\_Sample\_011046841, Unigene48807\_Sample\_011046841, Unigene48876\_Sample\_011046841, Unigene48956\_Sample\_011046841, Unigene49504\_Sample\_011046841, Unigene49839\_Sample\_011046841, Unigene49931\_Sample\_011046841, Unigene50113\_Sample\_011046841, Unigene50257\_Sample\_011046841, Unigene50269\_Sample\_011046841, Unigene50412\_Sample\_011046841, Unigene50469\_Sample\_011046841, Unigene50662\_Sample\_011046841, Unigene50956\_Sample\_011046841, Unigene51015\_Sample\_011046841, Unigene51117\_Sample\_011046841, Unigene51141\_Sample\_011046841, Unigene51286\_Sample\_011046841, Unigene51442\_Sample\_011046841, Unigene51481\_Sample\_011046841, Unigene51663\_Sample\_011046841, Unigene51789\_Sample\_011046841, Unigene51837\_Sample\_011046841, Unigene52079\_Sample\_011046841, Unigene52140\_Sample\_011046841, Unigene52177\_Sample\_011046841, Unigene52248\_Sample\_011046841, Unigene52265\_Sample\_011046841, Unigene52323\_Sample\_011046841, Unigene52673\_Sample\_011046841, Unigene52685\_Sample\_011046841, Unigene52831\_Sample\_011046841, Unigene53097\_Sample\_011046841, Unigene53098\_Sample\_011046841, Unigene53170\_Sample\_011046841, Unigene53284\_Sample\_011046841, Unigene53342\_Sample\_011046841, Unigene53462\_Sample\_011046841, Unigene53838\_Sample\_011046841, Unigene54030\_Sample\_011046841, Unigene54368\_Sample\_011046841, Unigene54502\_Sample\_011046841, Unigene55314\_Sample\_011046841, Unigene55342\_Sample\_011046841, Unigene55381\_Sample\_011046841, Unigene55434\_Sample\_011046841, Unigene55517\_Sample\_011046841, Unigene55561\_Sample\_011046841, Unigene55651\_Sample\_011046841, Unigene55675\_Sample\_011046841, Unigene56098\_Sample\_011046841, Unigene56293\_Sample\_011046841, Unigene56468\_Sample\_011046841, Unigene56567\_Sample\_011046841, Unigene56587\_Sample\_011046841, Unigene56645\_Sample\_011046841, Unigene56822\_Sample\_011046841, Unigene56843\_Sample\_011046841, Unigene56917\_Sample\_011046841, Unigene56999\_Sample\_011046841, Unigene57456\_Sample\_011046841, Unigene57882\_Sample\_011046841, Unigene58672\_Sample\_011046841, Unigene58892\_Sample\_011046841, Unigene59476\_Sample\_011046841, Unigene59491\_Sample\_011046841, Unigene59541\_Sample\_011046841, Unigene59663\_Sample\_011046841, Unigene59675\_Sample\_011046841, Unigene60159\_Sample\_011046841, Unigene60341\_Sample\_011046841, Unigene60448\_Sample\_011046841, Unigene60494\_Sample\_011046841, Unigene60546\_Sample\_011046841, Unigene60606\_Sample\_011046841, Unigene60847\_Sample\_011046841, Unigene100\_Sample\_011046841, Unigene1966\_Sample\_011046841, Unigene1999\_Sample\_011046841, Unigene2821\_Sample\_011046841, Unigene5340\_Sample\_011046841, Unigene5875\_Sample\_011046841, Unigene8144\_Sample\_011046841, Unigene9742\_Sample\_011046841, Unigene11214\_Sample\_011046841, Unigene12358\_Sample\_011046841, Unigene13080\_Sample\_011046841, Unigene13407\_Sample\_011046841, Unigene13435\_Sample\_011046841, Unigene14511\_Sample\_011046841, Unigene15607\_Sample\_011046841, Unigene16067\_Sample\_011046841, Unigene20762\_Sample\_011046841, Unigene22992\_Sample\_011046841, Unigene23532\_Sample\_011046841, Unigene24060\_Sample\_011046841, Unigene25319\_Sample\_011046841, Unigene26075\_Sample\_011046841, Unigene26417\_Sample\_011046841, Unigene27899\_Sample\_011046841, Unigene29675\_Sample\_011046841, Unigene31165\_Sample\_011046841, Unigene31588\_Sample\_011046841, Unigene32174\_Sample\_011046841, Unigene32497\_Sample\_011046841, Unigene32525\_Sample\_011046841, Unigene32652\_Sample\_011046841, Unigene34784\_Sample\_011046841, Unigene35806\_Sample\_011046841, Unigene36363\_Sample\_011046841, Unigene39016\_Sample\_011046841, Unigene39322\_Sample\_011046841, Unigene39766\_Sample\_011046841, Unigene40503\_Sample\_011046841, Unigene42324\_Sample\_011046841, Unigene42357\_Sample\_011046841, Unigene42794\_Sample\_011046841, Unigene43909\_Sample\_011046841, Unigene44139\_Sample\_011046841, Unigene44518\_Sample\_011046841, Unigene45246\_Sample\_011046841, Unigene45656\_Sample\_011046841, Unigene45812\_Sample\_011046841, Unigene46218\_Sample\_011046841, Unigene46297\_Sample\_011046841, Unigene47036\_Sample\_011046841, Unigene47779\_Sample\_011046841, Unigene48064\_Sample\_011046841, Unigene48158\_Sample\_011046841, Unigene48529\_Sample\_011046841, Unigene48687\_Sample\_011046841, Unigene49114\_Sample\_011046841, Unigene49197\_Sample\_011046841, Unigene49381\_Sample\_011046841, Unigene49912\_Sample\_011046841, Unigene50393\_Sample\_011046841, Unigene51084\_Sample\_011046841, Unigene51270\_Sample\_011046841, Unigene51299\_Sample\_011046841, Unigene51466\_Sample\_011046841, Unigene51685\_Sample\_011046841, Unigene51771\_Sample\_011046841, Unigene51811\_Sample\_011046841, Unigene51823\_Sample\_011046841, Unigene52064\_Sample\_011046841, Unigene52176\_Sample\_011046841, Unigene52824\_Sample\_011046841, Unigene53091\_Sample\_011046841, Unigene53494\_Sample\_011046841, Unigene54158\_Sample\_011046841, Unigene54424\_Sample\_011046841, Unigene54733\_Sample\_011046841, Unigene54965\_Sample\_011046841, Unigene55163\_Sample\_011046841, Unigene55799\_Sample\_011046841, Unigene56825\_Sample\_011046841, Unigene56976\_Sample\_011046841, Unigene57735\_Sample\_011046841, Unigene57871\_Sample\_011046841, Unigene58560\_Sample\_011046841, Unigene58610\_Sample\_011046841, Unigene59042\_Sample\_011046841, Unigene59204\_Sample\_011046841, Unigene59311\_Sample\_011046841, Unigene59492\_Sample\_011046841, Unigene59688\_Sample\_011046841, Unigene59824\_Sample\_011046841, Unigene60318\_Sample\_011046841, Unigene4545\_Sample\_011046841, Unigene10731\_Sample\_011046841, Unigene11258\_Sample\_011046841, Unigene12431\_Sample\_011046841, Unigene13011\_Sample\_011046841, Unigene27921\_Sample\_011046841, Unigene30320\_Sample\_011046841, Unigene39321\_Sample\_011046841, Unigene39617\_Sample\_011046841, Unigene42413\_Sample\_011046841, Unigene43630\_Sample\_011046841, Unigene45389\_Sample\_011046841, Unigene45708\_Sample\_011046841, Unigene45952\_Sample\_011046841, Unigene47187\_Sample\_011046841, Unigene47976\_Sample\_011046841, Unigene48398\_Sample\_011046841, Unigene48698\_Sample\_011046841, Unigene48766\_Sample\_011046841, Unigene49863\_Sample\_011046841, Unigene50530\_Sample\_011046841, Unigene50947\_Sample\_011046841, Unigene51151\_Sample\_011046841, Unigene52346\_Sample\_011046841, Unigene52751\_Sample\_011046841, Unigene54067\_Sample\_011046841, Unigene54107\_Sample\_011046841, Unigene54528\_Sample\_011046841, Unigene54535\_Sample\_011046841, Unigene54851\_Sample\_011046841, Unigene55049\_Sample\_011046841, Unigene55582\_Sample\_011046841, Unigene57093\_Sample\_011046841, Unigene58499\_Sample\_011046841, Unigene58917\_Sample\_011046841, Unigene59545\_Sample\_011046841, Unigene1441\_Sample\_011046841, Unigene5191\_Sample\_011046841, Unigene6167\_Sample\_011046841, Unigene7585\_Sample\_011046841, Unigene7978\_Sample\_011046841, Unigene10200\_Sample\_011046841, Unigene11735\_Sample\_011046841, Unigene12261\_Sample\_011046841, Unigene12818\_Sample\_011046841, Unigene16008\_Sample\_011046841, Unigene18434\_Sample\_011046841, Unigene18785\_Sample\_011046841, Unigene21456\_Sample\_011046841, Unigene21483\_Sample\_011046841, Unigene21731\_Sample\_011046841, Unigene24733\_Sample\_011046841, Unigene25630\_Sample\_011046841, Unigene29070\_Sample\_011046841, Unigene29803\_Sample\_011046841, Unigene30591\_Sample\_011046841, Unigene31394\_Sample\_011046841, Unigene32867\_Sample\_011046841, Unigene34175\_Sample\_011046841, Unigene34352\_Sample\_011046841, Unigene35398\_Sample\_011046841, Unigene37098\_Sample\_011046841, Unigene37765\_Sample\_011046841, Unigene37823\_Sample\_011046841, Unigene38267\_Sample\_011046841, Unigene38650\_Sample\_011046841, Unigene38688\_Sample\_011046841, Unigene42159\_Sample\_011046841, Unigene42443\_Sample\_011046841, Unigene43846\_Sample\_011046841, Unigene43986\_Sample\_011046841, Unigene44108\_Sample\_011046841, Unigene46888\_Sample\_011046841, Unigene47812\_Sample\_011046841, Unigene49014\_Sample\_011046841, Unigene49168\_Sample\_011046841, Unigene49709\_Sample\_011046841, Unigene50099\_Sample\_011046841, Unigene51441\_Sample\_011046841, Unigene51946\_Sample\_011046841, Unigene52797\_Sample\_011046841, Unigene53487\_Sample\_011046841, Unigene54159\_Sample\_011046841, Unigene54358\_Sample\_011046841, Unigene54624\_Sample\_011046841, Unigene54689\_Sample\_011046841, Unigene54767\_Sample\_011046841, Unigene55829\_Sample\_011046841, Unigene57398\_Sample\_011046841, Unigene57656\_Sample\_011046841, Unigene58229\_Sample\_011046841, Unigene60523\_Sample\_011046841, Unigene475\_Sample\_011046841, Unigene3670\_Sample\_011046841, Unigene7080\_Sample\_011046841, Unigene10875\_Sample\_011046841, Unigene18276\_Sample\_011046841, Unigene18805\_Sample\_011046841, Unigene20785\_Sample\_011046841, Unigene21216\_Sample\_011046841, Unigene23199\_Sample\_011046841, Unigene26069\_Sample\_011046841, Unigene34464\_Sample\_011046841, Unigene36235\_Sample\_011046841, Unigene37401\_Sample\_011046841, Unigene41811\_Sample\_011046841, Unigene44716\_Sample\_011046841, Unigene47162\_Sample\_011046841, Unigene49574\_Sample\_011046841, Unigene49608\_Sample\_011046841, Unigene54933\_Sample\_011046841, Unigene55630\_Sample\_011046841, Unigene56412\_Sample\_011046841, Unigene186\_Sample\_011046841, Unigene5530\_Sample\_011046841, Unigene6972\_Sample\_011046841, Unigene7554\_Sample\_011046841, Unigene16224\_Sample\_011046841, Unigene17179\_Sample\_011046841, Unigene17236\_Sample\_011046841, Unigene18385\_Sample\_011046841, Unigene18442\_Sample\_011046841, Unigene18461\_Sample\_011046841, Unigene18715\_Sample\_011046841, Unigene23087\_Sample\_011046841, Unigene23984\_Sample\_011046841, Unigene27454\_Sample\_011046841, Unigene28830\_Sample\_011046841, Unigene29271\_Sample\_011046841, Unigene30245\_Sample\_011046841, Unigene31849\_Sample\_011046841, Unigene32643\_Sample\_011046841, Unigene36472\_Sample\_011046841, Unigene38011\_Sample\_011046841, Unigene39062\_Sample\_011046841, Unigene41364\_Sample\_011046841, Unigene42381\_Sample\_011046841, Unigene42569\_Sample\_011046841, Unigene43793\_Sample\_011046841, Unigene45777\_Sample\_011046841, Unigene45986\_Sample\_011046841, Unigene46399\_Sample\_011046841, Unigene46613\_Sample\_011046841, Unigene48317\_Sample\_011046841, Unigene48678\_Sample\_011046841, Unigene48730\_Sample\_011046841, Unigene48863\_Sample\_011046841, Unigene48873\_Sample\_011046841, Unigene50382\_Sample\_011046841, Unigene50878\_Sample\_011046841, Unigene53668\_Sample\_011046841, Unigene53698\_Sample\_011046841, Unigene54034\_Sample\_011046841, Unigene54325\_Sample\_011046841, Unigene54459\_Sample\_011046841, Unigene54790\_Sample\_011046841, Unigene56772\_Sample\_011046841, Unigene59567\_Sample\_011046841, Unigene60436\_Sample\_011046841, Unigene60458\_Sample\_011046841, Unigene2521\_Sample\_011046841, Unigene8080\_Sample\_011046841, Unigene12223\_Sample\_011046841, Unigene22614\_Sample\_011046841, Unigene33197\_Sample\_011046841, Unigene33665\_Sample\_011046841, Unigene33744\_Sample\_011046841, Unigene36498\_Sample\_011046841, Unigene37427\_Sample\_011046841, Unigene41318\_Sample\_011046841, Unigene43491\_Sample\_011046841, Unigene46059\_Sample\_011046841, Unigene48164\_Sample\_011046841, Unigene48167\_Sample\_011046841, Unigene55373\_Sample\_011046841, Unigene57266\_Sample\_011046841, Unigene57802\_Sample\_011046841, Unigene4226\_Sample\_011046841, Unigene4296\_Sample\_011046841, Unigene6174\_Sample\_011046841, Unigene6573\_Sample\_011046841, Unigene7779\_Sample\_011046841, Unigene13221\_Sample\_011046841, Unigene15774\_Sample\_011046841, Unigene16836\_Sample\_011046841, Unigene18474\_Sample\_011046841, Unigene19914\_Sample\_011046841, Unigene20667\_Sample\_011046841, Unigene21699\_Sample\_011046841, Unigene21863\_Sample\_011046841, Unigene21868\_Sample\_011046841, Unigene23333\_Sample\_011046841, Unigene24927\_Sample\_011046841, Unigene30216\_Sample\_011046841, Unigene32077\_Sample\_011046841, Unigene32476\_Sample\_011046841, Unigene32987\_Sample\_011046841, Unigene34087\_Sample\_011046841, Unigene34550\_Sample\_011046841, Unigene34778\_Sample\_011046841, Unigene36090\_Sample\_011046841, Unigene36149\_Sample\_011046841, Unigene37087\_Sample\_011046841, Unigene38056\_Sample\_011046841, Unigene40432\_Sample\_011046841, Unigene41423\_Sample\_011046841, Unigene42360\_Sample\_011046841, Unigene42564\_Sample\_011046841, Unigene43818\_Sample\_011046841, Unigene45410\_Sample\_011046841, Unigene45721\_Sample\_011046841, Unigene45949\_Sample\_011046841, Unigene47820\_Sample\_011046841, Unigene49106\_Sample\_011046841, Unigene49650\_Sample\_011046841, Unigene51420\_Sample\_011046841, Unigene53073\_Sample\_011046841, Unigene53865\_Sample\_011046841, Unigene54198\_Sample\_011046841, Unigene54273\_Sample\_011046841, Unigene54823\_Sample\_011046841, Unigene54935\_Sample\_011046841, Unigene55520\_Sample\_011046841, Unigene625\_Sample\_011046841, Unigene1482\_Sample\_011046841, Unigene1594\_Sample\_011046841, Unigene4847\_Sample\_011046841, Unigene7035\_Sample\_011046841, Unigene12187\_Sample\_011046841, Unigene12706\_Sample\_011046841, Unigene14125\_Sample\_011046841, Unigene15479\_Sample\_011046841, Unigene16520\_Sample\_011046841, Unigene19013\_Sample\_011046841, Unigene19137\_Sample\_011046841, Unigene19275\_Sample\_011046841, Unigene19735\_Sample\_011046841, Unigene20272\_Sample\_011046841, Unigene20440\_Sample\_011046841, Unigene20632\_Sample\_011046841, Unigene23760\_Sample\_011046841, Unigene23792\_Sample\_011046841, Unigene24352\_Sample\_011046841, Unigene24516\_Sample\_011046841, Unigene24783\_Sample\_011046841, Unigene25152\_Sample\_011046841, Unigene25779\_Sample\_011046841, Unigene26842\_Sample\_011046841, Unigene27017\_Sample\_011046841, Unigene27558\_Sample\_011046841, Unigene29438\_Sample\_011046841, Unigene30077\_Sample\_011046841, Unigene31129\_Sample\_011046841, Unigene33976\_Sample\_011046841, Unigene34357\_Sample\_011046841, Unigene34528\_Sample\_011046841, Unigene35394\_Sample\_011046841, Unigene36216\_Sample\_011046841, Unigene37080\_Sample\_011046841, Unigene38547\_Sample\_011046841, Unigene40186\_Sample\_011046841, Unigene40210\_Sample\_011046841, Unigene40306\_Sample\_011046841, Unigene40559\_Sample\_011046841, Unigene40771\_Sample\_011046841, Unigene41140\_Sample\_011046841, Unigene41884\_Sample\_011046841, Unigene42067\_Sample\_011046841, Unigene42184\_Sample\_011046841, Unigene42508\_Sample\_011046841, Unigene42749\_Sample\_011046841, Unigene43216\_Sample\_011046841, Unigene43528\_Sample\_011046841, Unigene43536\_Sample\_011046841, Unigene43813\_Sample\_011046841, Unigene44466\_Sample\_011046841, Unigene44774\_Sample\_011046841, Unigene45156\_Sample\_011046841, Unigene45837\_Sample\_011046841, Unigene46028\_Sample\_011046841, Unigene46240\_Sample\_011046841, Unigene46376\_Sample\_011046841, Unigene47794\_Sample\_011046841, Unigene48068\_Sample\_011046841, Unigene48268\_Sample\_011046841, Unigene48938\_Sample\_011046841, Unigene49204\_Sample\_011046841, Unigene49252\_Sample\_011046841, Unigene49579\_Sample\_011046841, Unigene49635\_Sample\_011046841, Unigene49966\_Sample\_011046841, Unigene50058\_Sample\_011046841, Unigene50296\_Sample\_011046841, Unigene50362\_Sample\_011046841, Unigene50924\_Sample\_011046841, Unigene51282\_Sample\_011046841, Unigene51677\_Sample\_011046841, Unigene52955\_Sample\_011046841, Unigene53018\_Sample\_011046841, Unigene53290\_Sample\_011046841, Unigene54239\_Sample\_011046841, Unigene54567\_Sample\_011046841, Unigene54638\_Sample\_011046841, Unigene55252\_Sample\_011046841, Unigene56011\_Sample\_011046841, Unigene56559\_Sample\_011046841, Unigene56571\_Sample\_011046841, Unigene4851\_Sample\_011046841, Unigene9545\_Sample\_011046841, Unigene12157\_Sample\_011046841, Unigene16507\_Sample\_011046841, Unigene17203\_Sample\_011046841, Unigene22917\_Sample\_011046841, Unigene27881\_Sample\_011046841, Unigene28279\_Sample\_011046841, Unigene31066\_Sample\_011046841, Unigene37789\_Sample\_011046841, Unigene38271\_Sample\_011046841, Unigene39842\_Sample\_011046841, Unigene41828\_Sample\_011046841, Unigene45139\_Sample\_011046841, Unigene45879\_Sample\_011046841, Unigene49583\_Sample\_011046841, Unigene50185\_Sample\_011046841, Unigene51822\_Sample\_011046841, Unigene53609\_Sample\_011046841, Unigene54256\_Sample\_011046841, Unigene55419\_Sample\_011046841, Unigene14396\_Sample\_011046841, Unigene28941\_Sample\_011046841, Unigene37693\_Sample\_011046841, Unigene12799\_Sample\_011046841, Unigene16924\_Sample\_011046841, Unigene18506\_Sample\_011046841, Unigene18756\_Sample\_011046841, Unigene29219\_Sample\_011046841, Unigene30309\_Sample\_011046841, Unigene31042\_Sample\_011046841, Unigene35592\_Sample\_011046841, Unigene38048\_Sample\_011046841, Unigene39083\_Sample\_011046841, Unigene41758\_Sample\_011046841, Unigene42123\_Sample\_011046841, Unigene6153\_Sample\_011046841, Unigene11166\_Sample\_011046841, Unigene15838\_Sample\_011046841, Unigene16292\_Sample\_011046841, Unigene18813\_Sample\_011046841, Unigene19418\_Sample\_011046841, Unigene21313\_Sample\_011046841, Unigene39186\_Sample\_011046841, Unigene40365\_Sample\_011046841, Unigene40973\_Sample\_011046841, Unigene41284\_Sample\_011046841, Unigene48563\_Sample\_011046841, Unigene48763\_Sample\_011046841, Unigene53001\_Sample\_011046841, Unigene54887\_Sample\_011046841, Unigene17285\_Sample\_011046841, Unigene17735\_Sample\_011046841, Unigene20491\_Sample\_011046841, Unigene21757\_Sample\_011046841, Unigene23897\_Sample\_011046841, Unigene27719\_Sample\_011046841, Unigene30520\_Sample\_011046841, Unigene32666\_Sample\_011046841, Unigene33784\_Sample\_011046841, Unigene35830\_Sample\_011046841, Unigene41680\_Sample\_011046841, Unigene43028\_Sample\_011046841, Unigene43662\_Sample\_011046841, Unigene43946\_Sample\_011046841, Unigene54962\_Sample\_011046841, Unigene15416\_Sample\_011046841, Unigene17439\_Sample\_011046841, Unigene18375\_Sample\_011046841, Unigene18613\_Sample\_011046841, Unigene21855\_Sample\_011046841, Unigene22804\_Sample\_011046841, Unigene25961\_Sample\_011046841, Unigene31713\_Sample\_011046841, Unigene32701\_Sample\_011046841, Unigene34228\_Sample\_011046841, Unigene35708\_Sample\_011046841, Unigene39090\_Sample\_011046841, Unigene42712\_Sample\_011046841, Unigene45758\_Sample\_011046841, Unigene54335\_Sample\_011046841, Unigene55178\_Sample\_011046841, Unigene3315\_Sample\_011046841, Unigene3325\_Sample\_011046841, Unigene15828\_Sample\_011046841, Unigene18989\_Sample\_011046841, Unigene19222\_Sample\_011046841, Unigene21162\_Sample\_011046841, Unigene22492\_Sample\_011046841, Unigene25049\_Sample\_011046841, Unigene30332\_Sample\_011046841, Unigene33603\_Sample\_011046841, Unigene39581\_Sample\_011046841, Unigene50987\_Sample\_011046841, Unigene54187\_Sample\_011046841, Unigene56268\_Sample\_011046841, Unigene28275\_Sample\_011046841, Unigene34466\_Sample\_011046841, Unigene37642\_Sample\_011046841, Unigene40544\_Sample\_011046841, Unigene40575\_Sample\_011046841, Unigene41088\_Sample\_011046841, Unigene46786\_Sample\_011046841, Unigene58257\_Sample\_011046841, Unigene3881\_Sample\_011046841, Unigene9865\_Sample\_011046841, Unigene20294\_Sample\_011046841, Unigene22482\_Sample\_011046841, Unigene38645\_Sample\_011046841, Unigene44109\_Sample\_011046841, Unigene47097\_Sample\_011046841, Unigene48242\_Sample\_011046841, Unigene48943\_Sample\_011046841, Unigene51419\_Sample\_011046841, Unigene43673\_Sample\_011046841, Unigene44216\_Sample\_011046841, Unigene32558\_Sample\_011046841, Unigene46635\_Sample\_011046841, Unigene57937\_Sample\_011046841, Unigene27530\_Sample\_011046841, Unigene29503\_Sample\_011046841, Unigene144\_Sample\_011046841, Unigene14181\_Sample\_011046841, Unigene16896\_Sample\_011046841, Unigene18419\_Sample\_011046841, Unigene23701\_Sample\_011046841, Unigene26988\_Sample\_011046841, Unigene31679\_Sample\_011046841, Unigene38646\_Sample\_011046841, Unigene41474\_Sample\_011046841, Unigene43845\_Sample\_011046841, Unigene46509\_Sample\_011046841, Unigene48925\_Sample\_011046841, Unigene52018\_Sample\_011046841, Unigene54241\_Sample\_011046841, Unigene8115\_Sample\_011046841, Unigene12865\_Sample\_011046841, Unigene20259\_Sample\_011046841, Unigene21775\_Sample\_011046841, Unigene32594\_Sample\_011046841, Unigene32802\_Sample\_011046841, Unigene49716\_Sample\_011046841, Unigene29669\_Sample\_011046841, Unigene22335\_Sample\_011046841, Unigene30813\_Sample\_011046841, Unigene36941\_Sample\_011046841, Unigene46950\_Sample\_011046841, Unigene24848\_Sample\_011046841, Unigene23065\_Sample\_011046841, Unigene33820\_Sample\_011046841, Unigene21317\_Sample\_011046841, Unigene29124\_Sample\_011046841, Unigene32062\_Sample\_011046841, Unigene42449\_Sample\_011046841, Unigene38066\_Sample\_011046841, Unigene17643\_Sample\_011046841, Unigene18899\_Sample\_011046841, Unigene50660\_Sample\_011046841 |
| 67 | Taste transduction Back to summary table | Unigene6178\_Sample\_011046841, Unigene12475\_Sample\_011046841, Unigene13481\_Sample\_011046841, Unigene13780\_Sample\_011046841, Unigene43518\_Sample\_011046841, Unigene58436\_Sample\_011046841, Unigene60762\_Sample\_011046841, Unigene5139\_Sample\_011046841, Unigene5744\_Sample\_011046841, Unigene12336\_Sample\_011046841, Unigene26922\_Sample\_011046841, Unigene29480\_Sample\_011046841, Unigene33821\_Sample\_011046841, Unigene54228\_Sample\_011046841, Unigene59071\_Sample\_011046841, Unigene2459\_Sample\_011046841, Unigene35808\_Sample\_011046841, Unigene55826\_Sample\_011046841, Unigene30190\_Sample\_011046841, Unigene50948\_Sample\_011046841, Unigene58199\_Sample\_011046841, Unigene5814\_Sample\_011046841, Unigene51429\_Sample\_011046841, Unigene29462\_Sample\_011046841, Unigene36071\_Sample\_011046841, Unigene2110\_Sample\_011046841, Unigene55041\_Sample\_011046841, Unigene56969\_Sample\_011046841, Unigene15705\_Sample\_011046841, Unigene23831\_Sample\_011046841, Unigene28288\_Sample\_011046841, Unigene10863\_Sample\_011046841, Unigene12324\_Sample\_011046841, Unigene44021\_Sample\_011046841, Unigene47487\_Sample\_011046841, Unigene49482\_Sample\_011046841, Unigene35082\_Sample\_011046841, Unigene14248\_Sample\_011046841, Unigene29441\_Sample\_011046841, Unigene44852\_Sample\_011046841, Unigene56075\_Sample\_011046841 |
| 68 | GnRH signaling pathway Back to summary table | Unigene865\_Sample\_011046841, Unigene6130\_Sample\_011046841, Unigene6178\_Sample\_011046841, Unigene7143\_Sample\_011046841, Unigene7809\_Sample\_011046841, Unigene8859\_Sample\_011046841, Unigene11999\_Sample\_011046841, Unigene12527\_Sample\_011046841, Unigene13481\_Sample\_011046841, Unigene13789\_Sample\_011046841, Unigene40774\_Sample\_011046841, Unigene58460\_Sample\_011046841, Unigene58987\_Sample\_011046841, Unigene59189\_Sample\_011046841, Unigene60462\_Sample\_011046841, Unigene60789\_Sample\_011046841, Unigene1345\_Sample\_011046841, Unigene1414\_Sample\_011046841, Unigene2509\_Sample\_011046841, Unigene5740\_Sample\_011046841, Unigene5744\_Sample\_011046841, Unigene10189\_Sample\_011046841, Unigene10346\_Sample\_011046841, Unigene12943\_Sample\_011046841, Unigene13419\_Sample\_011046841, Unigene13855\_Sample\_011046841, Unigene21418\_Sample\_011046841, Unigene21962\_Sample\_011046841, Unigene41574\_Sample\_011046841, Unigene43409\_Sample\_011046841, Unigene45592\_Sample\_011046841, Unigene46219\_Sample\_011046841, Unigene47555\_Sample\_011046841, Unigene50039\_Sample\_011046841, Unigene50170\_Sample\_011046841, Unigene52110\_Sample\_011046841, Unigene54900\_Sample\_011046841, Unigene56177\_Sample\_011046841, Unigene56681\_Sample\_011046841, Unigene56798\_Sample\_011046841, Unigene57375\_Sample\_011046841, Unigene58669\_Sample\_011046841, Unigene58821\_Sample\_011046841, Unigene59742\_Sample\_011046841, Unigene60140\_Sample\_011046841, Unigene60450\_Sample\_011046841, Unigene60880\_Sample\_011046841, Unigene8083\_Sample\_011046841, Unigene8813\_Sample\_011046841, Unigene11522\_Sample\_011046841, Unigene13920\_Sample\_011046841, Unigene28658\_Sample\_011046841, Unigene32259\_Sample\_011046841, Unigene40758\_Sample\_011046841, Unigene44316\_Sample\_011046841, Unigene57775\_Sample\_011046841, Unigene59581\_Sample\_011046841, Unigene60765\_Sample\_011046841, Unigene8139\_Sample\_011046841, Unigene32964\_Sample\_011046841, Unigene47860\_Sample\_011046841, Unigene51950\_Sample\_011046841, Unigene57387\_Sample\_011046841, Unigene57675\_Sample\_011046841, Unigene60863\_Sample\_011046841, Unigene30190\_Sample\_011046841, Unigene45708\_Sample\_011046841, Unigene57665\_Sample\_011046841, Unigene58199\_Sample\_011046841, Unigene59421\_Sample\_011046841, Unigene1107\_Sample\_011046841, Unigene38302\_Sample\_011046841, Unigene43782\_Sample\_011046841, Unigene51429\_Sample\_011046841, Unigene60217\_Sample\_011046841, Unigene7042\_Sample\_011046841, Unigene29318\_Sample\_011046841, Unigene47054\_Sample\_011046841, Unigene48742\_Sample\_011046841, Unigene52499\_Sample\_011046841, Unigene57641\_Sample\_011046841, Unigene2110\_Sample\_011046841, Unigene21201\_Sample\_011046841, Unigene55041\_Sample\_011046841, Unigene55520\_Sample\_011046841, Unigene15705\_Sample\_011046841, Unigene21362\_Sample\_011046841, Unigene23831\_Sample\_011046841, Unigene28288\_Sample\_011046841, Unigene47910\_Sample\_011046841, Unigene10863\_Sample\_011046841, Unigene19947\_Sample\_011046841, Unigene53609\_Sample\_011046841, Unigene12324\_Sample\_011046841, Unigene16539\_Sample\_011046841, Unigene19418\_Sample\_011046841, Unigene31264\_Sample\_011046841, Unigene31544\_Sample\_011046841, Unigene43718\_Sample\_011046841, Unigene47789\_Sample\_011046841, Unigene44021\_Sample\_011046841, Unigene47487\_Sample\_011046841, Unigene16442\_Sample\_011046841, Unigene30484\_Sample\_011046841, Unigene44354\_Sample\_011046841, Unigene19937\_Sample\_011046841, Unigene21539\_Sample\_011046841, Unigene32258\_Sample\_011046841, Unigene35082\_Sample\_011046841, Unigene48345\_Sample\_011046841, Unigene14248\_Sample\_011046841, Unigene46813\_Sample\_011046841, Unigene29441\_Sample\_011046841, Unigene59655\_Sample\_011046841, Unigene14181\_Sample\_011046841, Unigene30474\_Sample\_011046841, Unigene7048\_Sample\_011046841, Unigene19596\_Sample\_011046841, Unigene26777\_Sample\_011046841 |
| 69 | Spliceosome Back to summary table | Unigene918\_Sample\_011046841, Unigene1090\_Sample\_011046841, Unigene1382\_Sample\_011046841, Unigene3875\_Sample\_011046841, Unigene5058\_Sample\_011046841, Unigene5225\_Sample\_011046841, Unigene7020\_Sample\_011046841, Unigene7095\_Sample\_011046841, Unigene7261\_Sample\_011046841, Unigene7926\_Sample\_011046841, Unigene8480\_Sample\_011046841, Unigene9481\_Sample\_011046841, Unigene9929\_Sample\_011046841, Unigene10441\_Sample\_011046841, Unigene11524\_Sample\_011046841, Unigene11755\_Sample\_011046841, Unigene12266\_Sample\_011046841, Unigene12703\_Sample\_011046841, Unigene13431\_Sample\_011046841, Unigene13541\_Sample\_011046841, Unigene13572\_Sample\_011046841, Unigene15082\_Sample\_011046841, Unigene20175\_Sample\_011046841, Unigene20702\_Sample\_011046841, Unigene23201\_Sample\_011046841, Unigene26047\_Sample\_011046841, Unigene27522\_Sample\_011046841, Unigene27574\_Sample\_011046841, Unigene29839\_Sample\_011046841, Unigene29942\_Sample\_011046841, Unigene30301\_Sample\_011046841, Unigene31954\_Sample\_011046841, Unigene32540\_Sample\_011046841, Unigene33040\_Sample\_011046841, Unigene37228\_Sample\_011046841, Unigene39202\_Sample\_011046841, Unigene39222\_Sample\_011046841, Unigene39476\_Sample\_011046841, Unigene40189\_Sample\_011046841, Unigene41072\_Sample\_011046841, Unigene42548\_Sample\_011046841, Unigene42844\_Sample\_011046841, Unigene44243\_Sample\_011046841, Unigene44703\_Sample\_011046841, Unigene47056\_Sample\_011046841, Unigene47701\_Sample\_011046841, Unigene49150\_Sample\_011046841, Unigene49955\_Sample\_011046841, Unigene50073\_Sample\_011046841, Unigene50288\_Sample\_011046841, Unigene50329\_Sample\_011046841, Unigene51416\_Sample\_011046841, Unigene51688\_Sample\_011046841, Unigene53302\_Sample\_011046841, Unigene53368\_Sample\_011046841, Unigene54260\_Sample\_011046841, Unigene54629\_Sample\_011046841, Unigene55319\_Sample\_011046841, Unigene55499\_Sample\_011046841, Unigene55791\_Sample\_011046841, Unigene55841\_Sample\_011046841, Unigene56210\_Sample\_011046841, Unigene56350\_Sample\_011046841, Unigene56530\_Sample\_011046841, Unigene58300\_Sample\_011046841, Unigene58567\_Sample\_011046841, Unigene58663\_Sample\_011046841, Unigene58753\_Sample\_011046841, Unigene58889\_Sample\_011046841, Unigene59035\_Sample\_011046841, Unigene59074\_Sample\_011046841, Unigene59340\_Sample\_011046841, Unigene59360\_Sample\_011046841, Unigene59381\_Sample\_011046841, Unigene59487\_Sample\_011046841, Unigene59539\_Sample\_011046841, Unigene59710\_Sample\_011046841, Unigene59992\_Sample\_011046841, Unigene60036\_Sample\_011046841, Unigene60213\_Sample\_011046841, Unigene60296\_Sample\_011046841, Unigene60377\_Sample\_011046841, Unigene60407\_Sample\_011046841, Unigene60416\_Sample\_011046841, Unigene60445\_Sample\_011046841, Unigene60697\_Sample\_011046841, Unigene60714\_Sample\_011046841, Unigene60740\_Sample\_011046841, Unigene60798\_Sample\_011046841, Unigene60817\_Sample\_011046841, Unigene60838\_Sample\_011046841, Unigene2\_Sample\_011046841, Unigene330\_Sample\_011046841, Unigene1404\_Sample\_011046841, Unigene2899\_Sample\_011046841, Unigene3740\_Sample\_011046841, Unigene4917\_Sample\_011046841, Unigene4949\_Sample\_011046841, Unigene7462\_Sample\_011046841, Unigene8818\_Sample\_011046841, Unigene8987\_Sample\_011046841, Unigene9307\_Sample\_011046841, Unigene9855\_Sample\_011046841, Unigene11546\_Sample\_011046841, Unigene12713\_Sample\_011046841, Unigene12748\_Sample\_011046841, Unigene12957\_Sample\_011046841, Unigene13245\_Sample\_011046841, Unigene13325\_Sample\_011046841, Unigene13397\_Sample\_011046841, Unigene13826\_Sample\_011046841, Unigene13969\_Sample\_011046841, Unigene17359\_Sample\_011046841, Unigene19044\_Sample\_011046841, Unigene20312\_Sample\_011046841, Unigene21528\_Sample\_011046841, Unigene21838\_Sample\_011046841, Unigene23762\_Sample\_011046841, Unigene25911\_Sample\_011046841, Unigene27678\_Sample\_011046841, Unigene29038\_Sample\_011046841, Unigene30875\_Sample\_011046841, Unigene31420\_Sample\_011046841, Unigene31910\_Sample\_011046841, Unigene33752\_Sample\_011046841, Unigene34292\_Sample\_011046841, Unigene35247\_Sample\_011046841, Unigene37596\_Sample\_011046841, Unigene37984\_Sample\_011046841, Unigene39647\_Sample\_011046841, Unigene39654\_Sample\_011046841, Unigene40144\_Sample\_011046841, Unigene40261\_Sample\_011046841, Unigene41575\_Sample\_011046841, Unigene43011\_Sample\_011046841, Unigene43096\_Sample\_011046841, Unigene44009\_Sample\_011046841, Unigene44427\_Sample\_011046841, Unigene44438\_Sample\_011046841, Unigene44587\_Sample\_011046841, Unigene45275\_Sample\_011046841, Unigene45469\_Sample\_011046841, Unigene45568\_Sample\_011046841, Unigene45685\_Sample\_011046841, Unigene45990\_Sample\_011046841, Unigene46421\_Sample\_011046841, Unigene46989\_Sample\_011046841, Unigene47428\_Sample\_011046841, Unigene47467\_Sample\_011046841, Unigene47486\_Sample\_011046841, Unigene48388\_Sample\_011046841, Unigene49560\_Sample\_011046841, Unigene50121\_Sample\_011046841, Unigene51868\_Sample\_011046841, Unigene52100\_Sample\_011046841, Unigene52683\_Sample\_011046841, Unigene52822\_Sample\_011046841, Unigene53451\_Sample\_011046841, Unigene53571\_Sample\_011046841, Unigene53718\_Sample\_011046841, Unigene53861\_Sample\_011046841, Unigene54165\_Sample\_011046841, Unigene54372\_Sample\_011046841, Unigene54407\_Sample\_011046841, Unigene54556\_Sample\_011046841, Unigene55300\_Sample\_011046841, Unigene55363\_Sample\_011046841, Unigene55512\_Sample\_011046841, Unigene55529\_Sample\_011046841, Unigene56008\_Sample\_011046841, Unigene56074\_Sample\_011046841, Unigene56209\_Sample\_011046841, Unigene56455\_Sample\_011046841, Unigene56616\_Sample\_011046841, Unigene56678\_Sample\_011046841, Unigene56836\_Sample\_011046841, Unigene56930\_Sample\_011046841, Unigene57072\_Sample\_011046841, Unigene57182\_Sample\_011046841, Unigene57282\_Sample\_011046841, Unigene57374\_Sample\_011046841, Unigene57595\_Sample\_011046841, Unigene57717\_Sample\_011046841, Unigene57866\_Sample\_011046841, Unigene58033\_Sample\_011046841, Unigene58133\_Sample\_011046841, Unigene58205\_Sample\_011046841, Unigene58317\_Sample\_011046841, Unigene58659\_Sample\_011046841, Unigene58715\_Sample\_011046841, Unigene58727\_Sample\_011046841, Unigene58943\_Sample\_011046841, Unigene59653\_Sample\_011046841, Unigene59672\_Sample\_011046841, Unigene59679\_Sample\_011046841, Unigene59750\_Sample\_011046841, Unigene59772\_Sample\_011046841, Unigene59831\_Sample\_011046841, Unigene59917\_Sample\_011046841, Unigene59973\_Sample\_011046841, Unigene60093\_Sample\_011046841, Unigene60129\_Sample\_011046841, Unigene60215\_Sample\_011046841, Unigene60232\_Sample\_011046841, Unigene60526\_Sample\_011046841, Unigene60642\_Sample\_011046841, Unigene60701\_Sample\_011046841, Unigene60872\_Sample\_011046841, Unigene60887\_Sample\_011046841, Unigene2415\_Sample\_011046841, Unigene3486\_Sample\_011046841, Unigene6000\_Sample\_011046841, Unigene9295\_Sample\_011046841, Unigene9784\_Sample\_011046841, Unigene10135\_Sample\_011046841, Unigene10941\_Sample\_011046841, Unigene12735\_Sample\_011046841, Unigene12976\_Sample\_011046841, Unigene17957\_Sample\_011046841, Unigene18426\_Sample\_011046841, Unigene18447\_Sample\_011046841, Unigene18578\_Sample\_011046841, Unigene19343\_Sample\_011046841, Unigene19604\_Sample\_011046841, Unigene19923\_Sample\_011046841, Unigene20334\_Sample\_011046841, Unigene20724\_Sample\_011046841, Unigene22843\_Sample\_011046841, Unigene23713\_Sample\_011046841, Unigene27366\_Sample\_011046841, Unigene28546\_Sample\_011046841, Unigene28759\_Sample\_011046841, Unigene30570\_Sample\_011046841, Unigene33618\_Sample\_011046841, Unigene36452\_Sample\_011046841, Unigene39075\_Sample\_011046841, Unigene39900\_Sample\_011046841, Unigene40059\_Sample\_011046841, Unigene40558\_Sample\_011046841, Unigene40759\_Sample\_011046841, Unigene42107\_Sample\_011046841, Unigene42899\_Sample\_011046841, Unigene42922\_Sample\_011046841, Unigene43255\_Sample\_011046841, Unigene47516\_Sample\_011046841, Unigene47667\_Sample\_011046841, Unigene49377\_Sample\_011046841, Unigene52367\_Sample\_011046841, Unigene52992\_Sample\_011046841, Unigene53151\_Sample\_011046841, Unigene53843\_Sample\_011046841, Unigene54996\_Sample\_011046841, Unigene55474\_Sample\_011046841, Unigene55890\_Sample\_011046841, Unigene56599\_Sample\_011046841, Unigene56919\_Sample\_011046841, Unigene56923\_Sample\_011046841, Unigene57132\_Sample\_011046841, Unigene58132\_Sample\_011046841, Unigene58535\_Sample\_011046841, Unigene58617\_Sample\_011046841, Unigene59167\_Sample\_011046841, Unigene59344\_Sample\_011046841, Unigene59379\_Sample\_011046841, Unigene60047\_Sample\_011046841, Unigene60388\_Sample\_011046841, Unigene60707\_Sample\_011046841, Unigene3903\_Sample\_011046841, Unigene4359\_Sample\_011046841, Unigene13106\_Sample\_011046841, Unigene16692\_Sample\_011046841, Unigene21122\_Sample\_011046841, Unigene25357\_Sample\_011046841, Unigene32947\_Sample\_011046841, Unigene33130\_Sample\_011046841, Unigene33587\_Sample\_011046841, Unigene39191\_Sample\_011046841, Unigene40287\_Sample\_011046841, Unigene40807\_Sample\_011046841, Unigene45451\_Sample\_011046841, Unigene47759\_Sample\_011046841, Unigene48147\_Sample\_011046841, Unigene50477\_Sample\_011046841, Unigene51713\_Sample\_011046841, Unigene51820\_Sample\_011046841, Unigene53145\_Sample\_011046841, Unigene54323\_Sample\_011046841, Unigene55103\_Sample\_011046841, Unigene55856\_Sample\_011046841, Unigene55956\_Sample\_011046841, Unigene56019\_Sample\_011046841, Unigene57716\_Sample\_011046841, Unigene60598\_Sample\_011046841, Unigene8851\_Sample\_011046841, Unigene9703\_Sample\_011046841, Unigene16999\_Sample\_011046841, Unigene22216\_Sample\_011046841, Unigene25753\_Sample\_011046841, Unigene30337\_Sample\_011046841, Unigene35966\_Sample\_011046841, Unigene38155\_Sample\_011046841, Unigene39087\_Sample\_011046841, Unigene40079\_Sample\_011046841, Unigene46222\_Sample\_011046841, Unigene48727\_Sample\_011046841, Unigene48764\_Sample\_011046841, Unigene54852\_Sample\_011046841, Unigene55047\_Sample\_011046841, Unigene55667\_Sample\_011046841, Unigene57531\_Sample\_011046841, Unigene58185\_Sample\_011046841, Unigene78\_Sample\_011046841, Unigene3075\_Sample\_011046841, Unigene3388\_Sample\_011046841, Unigene8502\_Sample\_011046841, Unigene18672\_Sample\_011046841, Unigene20459\_Sample\_011046841, Unigene26084\_Sample\_011046841, Unigene34160\_Sample\_011046841, Unigene34225\_Sample\_011046841, Unigene46220\_Sample\_011046841, Unigene46290\_Sample\_011046841, Unigene48183\_Sample\_011046841, Unigene48625\_Sample\_011046841, Unigene50152\_Sample\_011046841, Unigene54051\_Sample\_011046841, Unigene58626\_Sample\_011046841, Unigene60575\_Sample\_011046841, Unigene47\_Sample\_011046841, Unigene312\_Sample\_011046841, Unigene5783\_Sample\_011046841, Unigene12833\_Sample\_011046841, Unigene16725\_Sample\_011046841, Unigene25267\_Sample\_011046841, Unigene31097\_Sample\_011046841, Unigene50443\_Sample\_011046841, Unigene52165\_Sample\_011046841, Unigene53606\_Sample\_011046841, Unigene54933\_Sample\_011046841, Unigene57196\_Sample\_011046841, Unigene58777\_Sample\_011046841, Unigene59134\_Sample\_011046841, Unigene1549\_Sample\_011046841, Unigene1562\_Sample\_011046841, Unigene7598\_Sample\_011046841, Unigene13198\_Sample\_011046841, Unigene14290\_Sample\_011046841, Unigene17668\_Sample\_011046841, Unigene48926\_Sample\_011046841, Unigene50808\_Sample\_011046841, Unigene54443\_Sample\_011046841, Unigene57238\_Sample\_011046841, Unigene57572\_Sample\_011046841, Unigene54\_Sample\_011046841, Unigene3488\_Sample\_011046841, Unigene19413\_Sample\_011046841, Unigene20591\_Sample\_011046841, Unigene35504\_Sample\_011046841, Unigene37267\_Sample\_011046841, Unigene38832\_Sample\_011046841, Unigene38907\_Sample\_011046841, Unigene47981\_Sample\_011046841, Unigene49287\_Sample\_011046841, Unigene58517\_Sample\_011046841, Unigene4143\_Sample\_011046841, Unigene6398\_Sample\_011046841, Unigene13879\_Sample\_011046841, Unigene17340\_Sample\_011046841, Unigene18417\_Sample\_011046841, Unigene28403\_Sample\_011046841, Unigene31216\_Sample\_011046841, Unigene33941\_Sample\_011046841, Unigene35426\_Sample\_011046841, Unigene37993\_Sample\_011046841, Unigene38522\_Sample\_011046841, Unigene44199\_Sample\_011046841, Unigene45153\_Sample\_011046841, Unigene47199\_Sample\_011046841, Unigene49465\_Sample\_011046841, Unigene50143\_Sample\_011046841, Unigene50178\_Sample\_011046841, Unigene54493\_Sample\_011046841, Unigene56734\_Sample\_011046841, Unigene58398\_Sample\_011046841, Unigene224\_Sample\_011046841, Unigene383\_Sample\_011046841, Unigene704\_Sample\_011046841, Unigene1750\_Sample\_011046841, Unigene2121\_Sample\_011046841, Unigene16668\_Sample\_011046841, Unigene16822\_Sample\_011046841, Unigene18509\_Sample\_011046841, Unigene19989\_Sample\_011046841, Unigene26481\_Sample\_011046841, Unigene27767\_Sample\_011046841, Unigene32247\_Sample\_011046841, Unigene36791\_Sample\_011046841, Unigene37232\_Sample\_011046841, Unigene38167\_Sample\_011046841, Unigene38635\_Sample\_011046841, Unigene38770\_Sample\_011046841, Unigene40184\_Sample\_011046841, Unigene44730\_Sample\_011046841, Unigene44749\_Sample\_011046841, Unigene45401\_Sample\_011046841, Unigene45634\_Sample\_011046841, Unigene48375\_Sample\_011046841, Unigene49770\_Sample\_011046841, Unigene55792\_Sample\_011046841, Unigene60369\_Sample\_011046841, Unigene4953\_Sample\_011046841, Unigene11759\_Sample\_011046841, Unigene26087\_Sample\_011046841, Unigene38021\_Sample\_011046841, Unigene49571\_Sample\_011046841, Unigene35054\_Sample\_011046841, Unigene53015\_Sample\_011046841, Unigene8133\_Sample\_011046841, Unigene17371\_Sample\_011046841, Unigene21770\_Sample\_011046841, Unigene39434\_Sample\_011046841, Unigene42768\_Sample\_011046841, Unigene28937\_Sample\_011046841, Unigene34515\_Sample\_011046841, Unigene34543\_Sample\_011046841, Unigene39967\_Sample\_011046841, Unigene41805\_Sample\_011046841, Unigene49268\_Sample\_011046841, Unigene52808\_Sample\_011046841, Unigene54484\_Sample\_011046841, Unigene16440\_Sample\_011046841, Unigene24899\_Sample\_011046841, Unigene26757\_Sample\_011046841, Unigene28615\_Sample\_011046841, Unigene42148\_Sample\_011046841, Unigene52524\_Sample\_011046841, Unigene23416\_Sample\_011046841, Unigene43596\_Sample\_011046841, Unigene44447\_Sample\_011046841, Unigene51115\_Sample\_011046841, Unigene40747\_Sample\_011046841, Unigene47149\_Sample\_011046841, Unigene54348\_Sample\_011046841, Unigene15204\_Sample\_011046841, Unigene22357\_Sample\_011046841, Unigene24776\_Sample\_011046841, Unigene25101\_Sample\_011046841, Unigene34359\_Sample\_011046841, Unigene1780\_Sample\_011046841, Unigene16207\_Sample\_011046841, Unigene35285\_Sample\_011046841, Unigene3945\_Sample\_011046841, Unigene21561\_Sample\_011046841, Unigene50475\_Sample\_011046841, Unigene17067\_Sample\_011046841, Unigene32716\_Sample\_011046841, Unigene23393\_Sample\_011046841, Unigene23701\_Sample\_011046841, Unigene27200\_Sample\_011046841, Unigene32488\_Sample\_011046841, Unigene42378\_Sample\_011046841, Unigene3856\_Sample\_011046841, Unigene51290\_Sample\_011046841, Unigene6540\_Sample\_011046841, Unigene37808\_Sample\_011046841, Unigene28536\_Sample\_011046841, Unigene35733\_Sample\_011046841 |
| 70 | Antigen processing and presentation Back to summary table | Unigene1090\_Sample\_011046841, Unigene1382\_Sample\_011046841, Unigene3875\_Sample\_011046841, Unigene5058\_Sample\_011046841, Unigene5225\_Sample\_011046841, Unigene7261\_Sample\_011046841, Unigene7926\_Sample\_011046841, Unigene10438\_Sample\_011046841, Unigene10441\_Sample\_011046841, Unigene11020\_Sample\_011046841, Unigene12266\_Sample\_011046841, Unigene13794\_Sample\_011046841, Unigene15082\_Sample\_011046841, Unigene15848\_Sample\_011046841, Unigene20175\_Sample\_011046841, Unigene26714\_Sample\_011046841, Unigene27522\_Sample\_011046841, Unigene27574\_Sample\_011046841, Unigene31954\_Sample\_011046841, Unigene32540\_Sample\_011046841, Unigene33040\_Sample\_011046841, Unigene35605\_Sample\_011046841, Unigene35926\_Sample\_011046841, Unigene39202\_Sample\_011046841, Unigene39476\_Sample\_011046841, Unigene42844\_Sample\_011046841, Unigene47701\_Sample\_011046841, Unigene50945\_Sample\_011046841, Unigene51416\_Sample\_011046841, Unigene53368\_Sample\_011046841, Unigene55319\_Sample\_011046841, Unigene57629\_Sample\_011046841, Unigene58360\_Sample\_011046841, Unigene58567\_Sample\_011046841, Unigene58889\_Sample\_011046841, Unigene60416\_Sample\_011046841, Unigene2\_Sample\_011046841, Unigene330\_Sample\_011046841, Unigene1404\_Sample\_011046841, Unigene2359\_Sample\_011046841, Unigene2899\_Sample\_011046841, Unigene3740\_Sample\_011046841, Unigene4917\_Sample\_011046841, Unigene4949\_Sample\_011046841, Unigene7462\_Sample\_011046841, Unigene8299\_Sample\_011046841, Unigene8818\_Sample\_011046841, Unigene8987\_Sample\_011046841, Unigene17359\_Sample\_011046841, Unigene19044\_Sample\_011046841, Unigene20312\_Sample\_011046841, Unigene21528\_Sample\_011046841, Unigene27678\_Sample\_011046841, Unigene29038\_Sample\_011046841, Unigene31910\_Sample\_011046841, Unigene32550\_Sample\_011046841, Unigene33662\_Sample\_011046841, Unigene34292\_Sample\_011046841, Unigene37596\_Sample\_011046841, Unigene37984\_Sample\_011046841, Unigene39647\_Sample\_011046841, Unigene40261\_Sample\_011046841, Unigene43096\_Sample\_011046841, Unigene44788\_Sample\_011046841, Unigene45568\_Sample\_011046841, Unigene46243\_Sample\_011046841, Unigene49894\_Sample\_011046841, Unigene51148\_Sample\_011046841, Unigene53571\_Sample\_011046841, Unigene54556\_Sample\_011046841, Unigene55825\_Sample\_011046841, Unigene56004\_Sample\_011046841, Unigene56836\_Sample\_011046841, Unigene56930\_Sample\_011046841, Unigene57072\_Sample\_011046841, Unigene57271\_Sample\_011046841, Unigene57595\_Sample\_011046841, Unigene57828\_Sample\_011046841, Unigene58521\_Sample\_011046841, Unigene60272\_Sample\_011046841, Unigene60642\_Sample\_011046841, Unigene60768\_Sample\_011046841, Unigene60872\_Sample\_011046841, Unigene6000\_Sample\_011046841, Unigene9295\_Sample\_011046841, Unigene9784\_Sample\_011046841, Unigene10135\_Sample\_011046841, Unigene12976\_Sample\_011046841, Unigene17957\_Sample\_011046841, Unigene18447\_Sample\_011046841, Unigene19923\_Sample\_011046841, Unigene20724\_Sample\_011046841, Unigene22843\_Sample\_011046841, Unigene23713\_Sample\_011046841, Unigene27366\_Sample\_011046841, Unigene33618\_Sample\_011046841, Unigene42922\_Sample\_011046841, Unigene44848\_Sample\_011046841, Unigene47471\_Sample\_011046841, Unigene47516\_Sample\_011046841, Unigene51364\_Sample\_011046841, Unigene51648\_Sample\_011046841, Unigene53989\_Sample\_011046841, Unigene4359\_Sample\_011046841, Unigene21122\_Sample\_011046841, Unigene33587\_Sample\_011046841, Unigene45120\_Sample\_011046841, Unigene50477\_Sample\_011046841, Unigene57716\_Sample\_011046841, Unigene9703\_Sample\_011046841, Unigene25753\_Sample\_011046841, Unigene39087\_Sample\_011046841, Unigene48764\_Sample\_011046841, Unigene59482\_Sample\_011046841, Unigene78\_Sample\_011046841, Unigene3075\_Sample\_011046841, Unigene3388\_Sample\_011046841, Unigene20459\_Sample\_011046841, Unigene26084\_Sample\_011046841, Unigene34160\_Sample\_011046841, Unigene34225\_Sample\_011046841, Unigene39261\_Sample\_011046841, Unigene42064\_Sample\_011046841, Unigene48625\_Sample\_011046841, Unigene5783\_Sample\_011046841, Unigene12833\_Sample\_011046841, Unigene16725\_Sample\_011046841, Unigene22776\_Sample\_011046841, Unigene55254\_Sample\_011046841, Unigene1549\_Sample\_011046841, Unigene7598\_Sample\_011046841, Unigene17668\_Sample\_011046841, Unigene59656\_Sample\_011046841, Unigene54\_Sample\_011046841, Unigene3488\_Sample\_011046841, Unigene19413\_Sample\_011046841, Unigene38907\_Sample\_011046841, Unigene58517\_Sample\_011046841, Unigene804\_Sample\_011046841, Unigene4143\_Sample\_011046841, Unigene6398\_Sample\_011046841, Unigene13879\_Sample\_011046841, Unigene17340\_Sample\_011046841, Unigene18417\_Sample\_011046841, Unigene31216\_Sample\_011046841, Unigene40898\_Sample\_011046841, Unigene44199\_Sample\_011046841, Unigene56734\_Sample\_011046841, Unigene224\_Sample\_011046841, Unigene383\_Sample\_011046841, Unigene704\_Sample\_011046841, Unigene1750\_Sample\_011046841, Unigene2121\_Sample\_011046841, Unigene16822\_Sample\_011046841, Unigene18509\_Sample\_011046841, Unigene20789\_Sample\_011046841, Unigene24624\_Sample\_011046841, Unigene26481\_Sample\_011046841, Unigene38167\_Sample\_011046841, Unigene38635\_Sample\_011046841, Unigene43386\_Sample\_011046841, Unigene45634\_Sample\_011046841, Unigene46799\_Sample\_011046841, Unigene11759\_Sample\_011046841, Unigene49571\_Sample\_011046841, Unigene35054\_Sample\_011046841, Unigene8133\_Sample\_011046841, Unigene17371\_Sample\_011046841, Unigene26316\_Sample\_011046841, Unigene33973\_Sample\_011046841, Unigene39434\_Sample\_011046841, Unigene23566\_Sample\_011046841, Unigene28937\_Sample\_011046841, Unigene34543\_Sample\_011046841, Unigene16440\_Sample\_011046841, Unigene24899\_Sample\_011046841, Unigene28615\_Sample\_011046841, Unigene30931\_Sample\_011046841, Unigene52524\_Sample\_011046841, Unigene43596\_Sample\_011046841, Unigene22175\_Sample\_011046841, Unigene22357\_Sample\_011046841, Unigene24776\_Sample\_011046841, Unigene34359\_Sample\_011046841, Unigene1780\_Sample\_011046841, Unigene43091\_Sample\_011046841, Unigene3945\_Sample\_011046841, Unigene21561\_Sample\_011046841, Unigene50475\_Sample\_011046841, Unigene17067\_Sample\_011046841, Unigene32716\_Sample\_011046841, Unigene42241\_Sample\_011046841, Unigene23393\_Sample\_011046841, Unigene34381\_Sample\_011046841, Unigene3856\_Sample\_011046841, Unigene16995\_Sample\_011046841, Unigene37372\_Sample\_011046841, Unigene13983\_Sample\_011046841, Unigene6540\_Sample\_011046841, Unigene37808\_Sample\_011046841 |
| 71 | TGF-beta signaling pathway Back to summary table | Unigene5122\_Sample\_011046841, Unigene5402\_Sample\_011046841, Unigene9668\_Sample\_011046841, Unigene10640\_Sample\_011046841, Unigene13524\_Sample\_011046841, Unigene13872\_Sample\_011046841, Unigene17090\_Sample\_011046841, Unigene27324\_Sample\_011046841, Unigene28326\_Sample\_011046841, Unigene45114\_Sample\_011046841, Unigene49649\_Sample\_011046841, Unigene50699\_Sample\_011046841, Unigene51977\_Sample\_011046841, Unigene54982\_Sample\_011046841, Unigene57017\_Sample\_011046841, Unigene57298\_Sample\_011046841, Unigene57761\_Sample\_011046841, Unigene60510\_Sample\_011046841, Unigene60877\_Sample\_011046841, Unigene5747\_Sample\_011046841, Unigene7299\_Sample\_011046841, Unigene8641\_Sample\_011046841, Unigene9985\_Sample\_011046841, Unigene19273\_Sample\_011046841, Unigene25098\_Sample\_011046841, Unigene30295\_Sample\_011046841, Unigene32119\_Sample\_011046841, Unigene33405\_Sample\_011046841, Unigene37278\_Sample\_011046841, Unigene41574\_Sample\_011046841, Unigene42106\_Sample\_011046841, Unigene43448\_Sample\_011046841, Unigene47555\_Sample\_011046841, Unigene47936\_Sample\_011046841, Unigene49399\_Sample\_011046841, Unigene49534\_Sample\_011046841, Unigene50163\_Sample\_011046841, Unigene50720\_Sample\_011046841, Unigene52184\_Sample\_011046841, Unigene56010\_Sample\_011046841, Unigene56232\_Sample\_011046841, Unigene56413\_Sample\_011046841, Unigene57390\_Sample\_011046841, Unigene58564\_Sample\_011046841, Unigene58697\_Sample\_011046841, Unigene59122\_Sample\_011046841, Unigene59902\_Sample\_011046841, Unigene60271\_Sample\_011046841, Unigene60787\_Sample\_011046841, Unigene60868\_Sample\_011046841, Unigene2066\_Sample\_011046841, Unigene3070\_Sample\_011046841, Unigene8828\_Sample\_011046841, Unigene13367\_Sample\_011046841, Unigene20297\_Sample\_011046841, Unigene20484\_Sample\_011046841, Unigene25812\_Sample\_011046841, Unigene28658\_Sample\_011046841, Unigene30120\_Sample\_011046841, Unigene32413\_Sample\_011046841, Unigene35027\_Sample\_011046841, Unigene48530\_Sample\_011046841, Unigene51803\_Sample\_011046841, Unigene55183\_Sample\_011046841, Unigene56456\_Sample\_011046841, Unigene58021\_Sample\_011046841, Unigene6435\_Sample\_011046841, Unigene7988\_Sample\_011046841, Unigene13607\_Sample\_011046841, Unigene19951\_Sample\_011046841, Unigene27828\_Sample\_011046841, Unigene32964\_Sample\_011046841, Unigene35757\_Sample\_011046841, Unigene48297\_Sample\_011046841, Unigene50901\_Sample\_011046841, Unigene58058\_Sample\_011046841, Unigene4662\_Sample\_011046841, Unigene9480\_Sample\_011046841, Unigene29346\_Sample\_011046841, Unigene34657\_Sample\_011046841, Unigene52611\_Sample\_011046841, Unigene57304\_Sample\_011046841, Unigene48383\_Sample\_011046841, Unigene11867\_Sample\_011046841, Unigene30153\_Sample\_011046841, Unigene41514\_Sample\_011046841, Unigene47915\_Sample\_011046841, Unigene57117\_Sample\_011046841, Unigene47947\_Sample\_011046841, Unigene12184\_Sample\_011046841, Unigene20381\_Sample\_011046841, Unigene32103\_Sample\_011046841, Unigene39020\_Sample\_011046841, Unigene43918\_Sample\_011046841, Unigene47910\_Sample\_011046841, Unigene51882\_Sample\_011046841, Unigene59748\_Sample\_011046841, Unigene60132\_Sample\_011046841, Unigene25028\_Sample\_011046841, Unigene26961\_Sample\_011046841, Unigene50989\_Sample\_011046841, Unigene14639\_Sample\_011046841, Unigene43935\_Sample\_011046841, Unigene52183\_Sample\_011046841, Unigene23954\_Sample\_011046841, Unigene28384\_Sample\_011046841, Unigene28643\_Sample\_011046841, Unigene39675\_Sample\_011046841, Unigene41406\_Sample\_011046841, Unigene4308\_Sample\_011046841, Unigene46652\_Sample\_011046841, Unigene54898\_Sample\_011046841, Unigene47908\_Sample\_011046841, Unigene30474\_Sample\_011046841, Unigene18796\_Sample\_011046841, Unigene21150\_Sample\_011046841 |
| 72 | Bladder cancer Back to summary table | Unigene7809\_Sample\_011046841, Unigene8429\_Sample\_011046841, Unigene10970\_Sample\_011046841, Unigene11367\_Sample\_011046841, Unigene13683\_Sample\_011046841, Unigene23925\_Sample\_011046841, Unigene57712\_Sample\_011046841, Unigene58460\_Sample\_011046841, Unigene1414\_Sample\_011046841, Unigene2933\_Sample\_011046841, Unigene4858\_Sample\_011046841, Unigene12943\_Sample\_011046841, Unigene13332\_Sample\_011046841, Unigene13766\_Sample\_011046841, Unigene41574\_Sample\_011046841, Unigene47555\_Sample\_011046841, Unigene54900\_Sample\_011046841, Unigene56798\_Sample\_011046841, Unigene57430\_Sample\_011046841, Unigene60427\_Sample\_011046841, Unigene3582\_Sample\_011046841, Unigene11932\_Sample\_011046841, Unigene28658\_Sample\_011046841, Unigene37205\_Sample\_011046841, Unigene57775\_Sample\_011046841, Unigene32964\_Sample\_011046841, Unigene51950\_Sample\_011046841, Unigene60863\_Sample\_011046841, Unigene1107\_Sample\_011046841, Unigene47967\_Sample\_011046841, Unigene29318\_Sample\_011046841, Unigene53995\_Sample\_011046841, Unigene47910\_Sample\_011046841, Unigene31969\_Sample\_011046841, Unigene43718\_Sample\_011046841, Unigene41007\_Sample\_011046841, Unigene46813\_Sample\_011046841, Unigene30474\_Sample\_011046841, Unigene26777\_Sample\_011046841 |
| 73 | Complement and coagulation cascades Back to summary table | Unigene3167\_Sample\_011046841, Unigene7331\_Sample\_011046841, Unigene9066\_Sample\_011046841, Unigene10883\_Sample\_011046841, Unigene11037\_Sample\_011046841, Unigene12804\_Sample\_011046841, Unigene22606\_Sample\_011046841, Unigene48782\_Sample\_011046841, Unigene53503\_Sample\_011046841, Unigene55446\_Sample\_011046841, Unigene56210\_Sample\_011046841, Unigene60720\_Sample\_011046841, Unigene60852\_Sample\_011046841, Unigene3446\_Sample\_011046841, Unigene5816\_Sample\_011046841, Unigene8794\_Sample\_011046841, Unigene12234\_Sample\_011046841, Unigene43933\_Sample\_011046841, Unigene50720\_Sample\_011046841, Unigene54853\_Sample\_011046841, Unigene55669\_Sample\_011046841, Unigene55828\_Sample\_011046841, Unigene58228\_Sample\_011046841, Unigene59923\_Sample\_011046841, Unigene43487\_Sample\_011046841, Unigene43093\_Sample\_011046841, Unigene50578\_Sample\_011046841, Unigene55654\_Sample\_011046841, Unigene10117\_Sample\_011046841, Unigene29267\_Sample\_011046841, Unigene38677\_Sample\_011046841, Unigene13394\_Sample\_011046841, Unigene31825\_Sample\_011046841, Unigene58881\_Sample\_011046841, Unigene48479\_Sample\_011046841, Unigene54129\_Sample\_011046841, Unigene47605\_Sample\_011046841, Unigene45464\_Sample\_011046841, Unigene36924\_Sample\_011046841 |
| 74 | Pyruvate metabolism Back to summary table | Unigene11911\_Sample\_011046841, Unigene21438\_Sample\_011046841, Unigene26779\_Sample\_011046841, Unigene28781\_Sample\_011046841, Unigene33884\_Sample\_011046841, Unigene36435\_Sample\_011046841, Unigene38930\_Sample\_011046841, Unigene40350\_Sample\_011046841, Unigene42838\_Sample\_011046841, Unigene48532\_Sample\_011046841, Unigene50553\_Sample\_011046841, Unigene56182\_Sample\_011046841, Unigene59158\_Sample\_011046841, Unigene59673\_Sample\_011046841, Unigene59904\_Sample\_011046841, Unigene60317\_Sample\_011046841, Unigene60564\_Sample\_011046841, Unigene60764\_Sample\_011046841, Unigene4741\_Sample\_011046841, Unigene9038\_Sample\_011046841, Unigene9752\_Sample\_011046841, Unigene11004\_Sample\_011046841, Unigene11188\_Sample\_011046841, Unigene15547\_Sample\_011046841, Unigene18937\_Sample\_011046841, Unigene19438\_Sample\_011046841, Unigene21594\_Sample\_011046841, Unigene22323\_Sample\_011046841, Unigene31025\_Sample\_011046841, Unigene34145\_Sample\_011046841, Unigene34858\_Sample\_011046841, Unigene34911\_Sample\_011046841, Unigene45547\_Sample\_011046841, Unigene48569\_Sample\_011046841, Unigene49640\_Sample\_011046841, Unigene51453\_Sample\_011046841, Unigene55549\_Sample\_011046841, Unigene57560\_Sample\_011046841, Unigene59519\_Sample\_011046841, Unigene60126\_Sample\_011046841, Unigene60192\_Sample\_011046841, Unigene4987\_Sample\_011046841, Unigene8728\_Sample\_011046841, Unigene9900\_Sample\_011046841, Unigene16547\_Sample\_011046841, Unigene19169\_Sample\_011046841, Unigene32177\_Sample\_011046841, Unigene32280\_Sample\_011046841, Unigene36181\_Sample\_011046841, Unigene37646\_Sample\_011046841, Unigene41355\_Sample\_011046841, Unigene43814\_Sample\_011046841, Unigene48448\_Sample\_011046841, Unigene51015\_Sample\_011046841, Unigene51117\_Sample\_011046841, Unigene52265\_Sample\_011046841, Unigene53098\_Sample\_011046841, Unigene56468\_Sample\_011046841, Unigene60606\_Sample\_011046841, Unigene14511\_Sample\_011046841, Unigene29675\_Sample\_011046841, Unigene39766\_Sample\_011046841, Unigene46218\_Sample\_011046841, Unigene50393\_Sample\_011046841, Unigene52346\_Sample\_011046841, Unigene58499\_Sample\_011046841, Unigene18434\_Sample\_011046841, Unigene24733\_Sample\_011046841, Unigene25630\_Sample\_011046841, Unigene26689\_Sample\_011046841, Unigene29803\_Sample\_011046841, Unigene38650\_Sample\_011046841, Unigene46888\_Sample\_011046841, Unigene57656\_Sample\_011046841, Unigene60523\_Sample\_011046841, Unigene23199\_Sample\_011046841, Unigene45754\_Sample\_011046841, Unigene23087\_Sample\_011046841, Unigene50382\_Sample\_011046841, Unigene33197\_Sample\_011046841, Unigene33665\_Sample\_011046841, Unigene33744\_Sample\_011046841, Unigene18474\_Sample\_011046841, Unigene30216\_Sample\_011046841, Unigene36631\_Sample\_011046841, Unigene41423\_Sample\_011046841, Unigene45410\_Sample\_011046841, Unigene51420\_Sample\_011046841, Unigene54935\_Sample\_011046841, Unigene19137\_Sample\_011046841, Unigene19275\_Sample\_011046841, Unigene19735\_Sample\_011046841, Unigene22736\_Sample\_011046841, Unigene24783\_Sample\_011046841, Unigene36216\_Sample\_011046841, Unigene48011\_Sample\_011046841, Unigene51282\_Sample\_011046841, Unigene22917\_Sample\_011046841, Unigene28279\_Sample\_011046841, Unigene49583\_Sample\_011046841, Unigene51822\_Sample\_011046841, Unigene37693\_Sample\_011046841, Unigene41758\_Sample\_011046841, Unigene35830\_Sample\_011046841, Unigene18375\_Sample\_011046841, Unigene22804\_Sample\_011046841, Unigene25961\_Sample\_011046841, Unigene15828\_Sample\_011046841, Unigene19222\_Sample\_011046841, Unigene33603\_Sample\_011046841, Unigene54187\_Sample\_011046841, Unigene18419\_Sample\_011046841, Unigene23924\_Sample\_011046841, Unigene43845\_Sample\_011046841, Unigene42449\_Sample\_011046841 |
| 75 | Sphingolipid metabolism Back to summary table | Unigene12543\_Sample\_011046841, Unigene39755\_Sample\_011046841, Unigene52585\_Sample\_011046841, Unigene53930\_Sample\_011046841, Unigene57475\_Sample\_011046841, Unigene59038\_Sample\_011046841, Unigene59442\_Sample\_011046841, Unigene59568\_Sample\_011046841, Unigene60614\_Sample\_011046841, Unigene10701\_Sample\_011046841, Unigene12801\_Sample\_011046841, Unigene22548\_Sample\_011046841, Unigene43704\_Sample\_011046841, Unigene49859\_Sample\_011046841, Unigene51041\_Sample\_011046841, Unigene55269\_Sample\_011046841, Unigene58486\_Sample\_011046841, Unigene829\_Sample\_011046841, Unigene21191\_Sample\_011046841, Unigene30072\_Sample\_011046841, Unigene41288\_Sample\_011046841, Unigene49504\_Sample\_011046841, Unigene56567\_Sample\_011046841, Unigene44139\_Sample\_011046841, Unigene45656\_Sample\_011046841, Unigene36154\_Sample\_011046841, Unigene44090\_Sample\_011046841, Unigene55049\_Sample\_011046841, Unigene48863\_Sample\_011046841, Unigene25152\_Sample\_011046841, Unigene55252\_Sample\_011046841, Unigene4851\_Sample\_011046841, Unigene45139\_Sample\_011046841, Unigene40973\_Sample\_011046841, Unigene38645\_Sample\_011046841, Unigene48242\_Sample\_011046841, Unigene43673\_Sample\_011046841, Unigene23701\_Sample\_011046841 |
| 76 | Glycerophospholipid metabolism Back to summary table | Unigene4863\_Sample\_011046841, Unigene5554\_Sample\_011046841, Unigene8922\_Sample\_011046841, Unigene9172\_Sample\_011046841, Unigene11999\_Sample\_011046841, Unigene12527\_Sample\_011046841, Unigene24991\_Sample\_011046841, Unigene27550\_Sample\_011046841, Unigene39862\_Sample\_011046841, Unigene40800\_Sample\_011046841, Unigene41720\_Sample\_011046841, Unigene47008\_Sample\_011046841, Unigene48252\_Sample\_011046841, Unigene49303\_Sample\_011046841, Unigene51342\_Sample\_011046841, Unigene52462\_Sample\_011046841, Unigene52585\_Sample\_011046841, Unigene53068\_Sample\_011046841, Unigene54102\_Sample\_011046841, Unigene55271\_Sample\_011046841, Unigene56495\_Sample\_011046841, Unigene57382\_Sample\_011046841, Unigene58183\_Sample\_011046841, Unigene59093\_Sample\_011046841, Unigene60462\_Sample\_011046841, Unigene2509\_Sample\_011046841, Unigene4977\_Sample\_011046841, Unigene5740\_Sample\_011046841, Unigene8391\_Sample\_011046841, Unigene10229\_Sample\_011046841, Unigene10346\_Sample\_011046841, Unigene12344\_Sample\_011046841, Unigene13096\_Sample\_011046841, Unigene33721\_Sample\_011046841, Unigene37040\_Sample\_011046841, Unigene39709\_Sample\_011046841, Unigene48867\_Sample\_011046841, Unigene49827\_Sample\_011046841, Unigene51256\_Sample\_011046841, Unigene51456\_Sample\_011046841, Unigene52110\_Sample\_011046841, Unigene52535\_Sample\_011046841, Unigene52652\_Sample\_011046841, Unigene53188\_Sample\_011046841, Unigene57375\_Sample\_011046841, Unigene58620\_Sample\_011046841, Unigene59664\_Sample\_011046841, Unigene60140\_Sample\_011046841, Unigene60319\_Sample\_011046841, Unigene60450\_Sample\_011046841, Unigene6227\_Sample\_011046841, Unigene10699\_Sample\_011046841, Unigene11522\_Sample\_011046841, Unigene18141\_Sample\_011046841, Unigene31642\_Sample\_011046841, Unigene32786\_Sample\_011046841, Unigene37438\_Sample\_011046841, Unigene38603\_Sample\_011046841, Unigene38993\_Sample\_011046841, Unigene43281\_Sample\_011046841, Unigene43891\_Sample\_011046841, Unigene47707\_Sample\_011046841, Unigene51984\_Sample\_011046841, Unigene54502\_Sample\_011046841, Unigene55517\_Sample\_011046841, Unigene56343\_Sample\_011046841, Unigene56843\_Sample\_011046841, Unigene57882\_Sample\_011046841, Unigene5340\_Sample\_011046841, Unigene9110\_Sample\_011046841, Unigene11214\_Sample\_011046841, Unigene12358\_Sample\_011046841, Unigene44518\_Sample\_011046841, Unigene51466\_Sample\_011046841, Unigene4545\_Sample\_011046841, Unigene10731\_Sample\_011046841, Unigene29070\_Sample\_011046841, Unigene49677\_Sample\_011046841, Unigene52797\_Sample\_011046841, Unigene54109\_Sample\_011046841, Unigene31811\_Sample\_011046841, Unigene53668\_Sample\_011046841, Unigene54459\_Sample\_011046841, Unigene42911\_Sample\_011046841, Unigene20667\_Sample\_011046841, Unigene21868\_Sample\_011046841, Unigene37777\_Sample\_011046841, Unigene44756\_Sample\_011046841, Unigene55520\_Sample\_011046841, Unigene20632\_Sample\_011046841, Unigene21909\_Sample\_011046841, Unigene34528\_Sample\_011046841, Unigene40210\_Sample\_011046841, Unigene56011\_Sample\_011046841, Unigene53609\_Sample\_011046841, Unigene54865\_Sample\_011046841, Unigene12799\_Sample\_011046841, Unigene19418\_Sample\_011046841, Unigene40888\_Sample\_011046841, Unigene25153\_Sample\_011046841, Unigene32666\_Sample\_011046841, Unigene33784\_Sample\_011046841, Unigene39090\_Sample\_011046841, Unigene40280\_Sample\_011046841, Unigene41088\_Sample\_011046841, Unigene27629\_Sample\_011046841, Unigene38645\_Sample\_011046841, Unigene44216\_Sample\_011046841, Unigene23701\_Sample\_011046841, Unigene26988\_Sample\_011046841, Unigene8115\_Sample\_011046841, Unigene20259\_Sample\_011046841, Unigene32062\_Sample\_011046841 |
| 77 | Aminoacyl-tRNA biosynthesis Back to summary table | Unigene2534\_Sample\_011046841, Unigene5357\_Sample\_011046841, Unigene10077\_Sample\_011046841, Unigene10793\_Sample\_011046841, Unigene13838\_Sample\_011046841, Unigene16953\_Sample\_011046841, Unigene19517\_Sample\_011046841, Unigene36308\_Sample\_011046841, Unigene45641\_Sample\_011046841, Unigene50983\_Sample\_011046841, Unigene52145\_Sample\_011046841, Unigene56847\_Sample\_011046841, Unigene57730\_Sample\_011046841, Unigene59294\_Sample\_011046841, Unigene59431\_Sample\_011046841, Unigene59943\_Sample\_011046841, Unigene60188\_Sample\_011046841, Unigene60424\_Sample\_011046841, Unigene5367\_Sample\_011046841, Unigene5908\_Sample\_011046841, Unigene11469\_Sample\_011046841, Unigene11502\_Sample\_011046841, Unigene11921\_Sample\_011046841, Unigene11949\_Sample\_011046841, Unigene12394\_Sample\_011046841, Unigene12771\_Sample\_011046841, Unigene18836\_Sample\_011046841, Unigene19564\_Sample\_011046841, Unigene19858\_Sample\_011046841, Unigene24299\_Sample\_011046841, Unigene32131\_Sample\_011046841, Unigene38847\_Sample\_011046841, Unigene39751\_Sample\_011046841, Unigene45119\_Sample\_011046841, Unigene47795\_Sample\_011046841, Unigene49971\_Sample\_011046841, Unigene50775\_Sample\_011046841, Unigene55029\_Sample\_011046841, Unigene57909\_Sample\_011046841, Unigene57944\_Sample\_011046841, Unigene58400\_Sample\_011046841, Unigene58895\_Sample\_011046841, Unigene58952\_Sample\_011046841, Unigene59119\_Sample\_011046841, Unigene59408\_Sample\_011046841, Unigene60315\_Sample\_011046841, Unigene60844\_Sample\_011046841, Unigene60912\_Sample\_011046841, Unigene1520\_Sample\_011046841, Unigene31652\_Sample\_011046841, Unigene34400\_Sample\_011046841, Unigene46366\_Sample\_011046841, Unigene46446\_Sample\_011046841, Unigene47616\_Sample\_011046841, Unigene54925\_Sample\_011046841, Unigene55675\_Sample\_011046841, Unigene56726\_Sample\_011046841, Unigene58104\_Sample\_011046841, Unigene58890\_Sample\_011046841, Unigene59759\_Sample\_011046841, Unigene60159\_Sample\_011046841, Unigene3756\_Sample\_011046841, Unigene6240\_Sample\_011046841, Unigene12887\_Sample\_011046841, Unigene13244\_Sample\_011046841, Unigene13435\_Sample\_011046841, Unigene41445\_Sample\_011046841, Unigene42357\_Sample\_011046841, Unigene45660\_Sample\_011046841, Unigene54158\_Sample\_011046841, Unigene54312\_Sample\_011046841, Unigene57752\_Sample\_011046841, Unigene60670\_Sample\_011046841, Unigene5866\_Sample\_011046841, Unigene37321\_Sample\_011046841, Unigene44912\_Sample\_011046841, Unigene58917\_Sample\_011046841, Unigene28964\_Sample\_011046841, Unigene60520\_Sample\_011046841, Unigene24335\_Sample\_011046841, Unigene53720\_Sample\_011046841, Unigene32643\_Sample\_011046841, Unigene34149\_Sample\_011046841, Unigene43445\_Sample\_011046841, Unigene47421\_Sample\_011046841, Unigene51077\_Sample\_011046841, Unigene58642\_Sample\_011046841, Unigene23466\_Sample\_011046841, Unigene50951\_Sample\_011046841, Unigene54071\_Sample\_011046841, Unigene48672\_Sample\_011046841, Unigene21527\_Sample\_011046841, Unigene24137\_Sample\_011046841, Unigene31083\_Sample\_011046841, Unigene42437\_Sample\_011046841, Unigene54467\_Sample\_011046841, Unigene22917\_Sample\_011046841, Unigene6536\_Sample\_011046841, Unigene21618\_Sample\_011046841, Unigene27802\_Sample\_011046841, Unigene31696\_Sample\_011046841, Unigene16874\_Sample\_011046841, Unigene16901\_Sample\_011046841, Unigene34443\_Sample\_011046841, Unigene39867\_Sample\_011046841, Unigene31613\_Sample\_011046841, Unigene45758\_Sample\_011046841, Unigene50025\_Sample\_011046841, Unigene28471\_Sample\_011046841, Unigene41261\_Sample\_011046841, Unigene36212\_Sample\_011046841, Unigene1958\_Sample\_011046841 |
| 78 | N-Glycan biosynthesis Back to summary table | Unigene2502\_Sample\_011046841, Unigene9098\_Sample\_011046841, Unigene9141\_Sample\_011046841, Unigene11290\_Sample\_011046841, Unigene12982\_Sample\_011046841, Unigene13809\_Sample\_011046841, Unigene18327\_Sample\_011046841, Unigene19441\_Sample\_011046841, Unigene23040\_Sample\_011046841, Unigene23914\_Sample\_011046841, Unigene26460\_Sample\_011046841, Unigene30736\_Sample\_011046841, Unigene42392\_Sample\_011046841, Unigene43461\_Sample\_011046841, Unigene45744\_Sample\_011046841, Unigene48026\_Sample\_011046841, Unigene51527\_Sample\_011046841, Unigene53138\_Sample\_011046841, Unigene53833\_Sample\_011046841, Unigene53919\_Sample\_011046841, Unigene54779\_Sample\_011046841, Unigene55978\_Sample\_011046841, Unigene58934\_Sample\_011046841, Unigene58992\_Sample\_011046841, Unigene59445\_Sample\_011046841, Unigene59763\_Sample\_011046841, Unigene181\_Sample\_011046841, Unigene4616\_Sample\_011046841, Unigene9672\_Sample\_011046841, Unigene13915\_Sample\_011046841, Unigene14656\_Sample\_011046841, Unigene22564\_Sample\_011046841, Unigene41238\_Sample\_011046841, Unigene43426\_Sample\_011046841, Unigene43906\_Sample\_011046841, Unigene49486\_Sample\_011046841, Unigene49859\_Sample\_011046841, Unigene52043\_Sample\_011046841, Unigene52903\_Sample\_011046841, Unigene53115\_Sample\_011046841, Unigene53427\_Sample\_011046841, Unigene56185\_Sample\_011046841, Unigene56266\_Sample\_011046841, Unigene56799\_Sample\_011046841, Unigene57122\_Sample\_011046841, Unigene57741\_Sample\_011046841, Unigene59524\_Sample\_011046841, Unigene59788\_Sample\_011046841, Unigene16482\_Sample\_011046841, Unigene18000\_Sample\_011046841, Unigene23548\_Sample\_011046841, Unigene28097\_Sample\_011046841, Unigene34269\_Sample\_011046841, Unigene36647\_Sample\_011046841, Unigene40140\_Sample\_011046841, Unigene43200\_Sample\_011046841, Unigene43998\_Sample\_011046841, Unigene46704\_Sample\_011046841, Unigene47997\_Sample\_011046841, Unigene48807\_Sample\_011046841, Unigene50113\_Sample\_011046841, Unigene50956\_Sample\_011046841, Unigene52831\_Sample\_011046841, Unigene56645\_Sample\_011046841, Unigene56917\_Sample\_011046841, Unigene60341\_Sample\_011046841, Unigene39322\_Sample\_011046841, Unigene42324\_Sample\_011046841, Unigene51270\_Sample\_011046841, Unigene51299\_Sample\_011046841, Unigene43630\_Sample\_011046841, Unigene48398\_Sample\_011046841, Unigene49863\_Sample\_011046841, Unigene50659\_Sample\_011046841, Unigene51151\_Sample\_011046841, Unigene54107\_Sample\_011046841, Unigene55582\_Sample\_011046841, Unigene34175\_Sample\_011046841, Unigene37823\_Sample\_011046841, Unigene26069\_Sample\_011046841, Unigene34464\_Sample\_011046841, Unigene29772\_Sample\_011046841, Unigene30245\_Sample\_011046841, Unigene31849\_Sample\_011046841, Unigene38011\_Sample\_011046841, Unigene42381\_Sample\_011046841, Unigene48678\_Sample\_011046841, Unigene57266\_Sample\_011046841, Unigene24927\_Sample\_011046841, Unigene38056\_Sample\_011046841, Unigene23760\_Sample\_011046841, Unigene24352\_Sample\_011046841, Unigene33017\_Sample\_011046841, Unigene34357\_Sample\_011046841, Unigene46376\_Sample\_011046841, Unigene50362\_Sample\_011046841, Unigene56705\_Sample\_011046841, Unigene17203\_Sample\_011046841, Unigene38048\_Sample\_011046841, Unigene42123\_Sample\_011046841, Unigene39186\_Sample\_011046841, Unigene54887\_Sample\_011046841, Unigene17735\_Sample\_011046841, Unigene54962\_Sample\_011046841, Unigene32701\_Sample\_011046841, Unigene34228\_Sample\_011046841, Unigene25049\_Sample\_011046841, Unigene27530\_Sample\_011046841, Unigene39859\_Sample\_011046841, Unigene46509\_Sample\_011046841, Unigene50660\_Sample\_011046841 |
[truncated: 248,134 more chars]
